# Breaking the ‘rule-of-five’ to access bridged bicyclic heteroaromatic bioisosteres

**DOI:** 10.1038/s44160-026-00990-0

**Published:** 2026-02-11

**Authors:** Ze-Xin Zhang, KaiChen Shu, Michael J. Tilby, Mark John P. Mandigma, Yiheng Guo, Jasper L. Tyler, Adam Noble, Varinder K. Aggarwal

**Affiliations:** School of Chemistry, University of Bristol, Bristol, UK

**Keywords:** Synthetic chemistry methodology, Photocatalysis

## Abstract

Bioisosteric replacement of aromatic and heteroaromatic rings with bridged bicyclic hydrocarbons is an important strategy in drug discovery. Intramolecular [2+2] cycloadditions of unconjugated dienes can provide a route to such motifs but are governed by the ‘rule-of-five’, which dictates that five-membered rings are preferentially formed, limiting access to alternative ring sizes. Here we introduce a visible-light-mediated intramolecular [2 + 2] cycloaddition of aza-1,6-dienes that leverages radical stabilization strategies to enable the selective formation of bridged bicycles over typically favoured fused bicycles. This approach generates previously elusive 6-azabicyclo[3.1.1]heptanes with facile substitution at every position around the ring. Exit vector analysis and comparison of the physicochemical and pharmacological properties of a 6-azabicyclo[3.1.1]heptane analogue of a piperazine-based drug demonstrate the potential application of this scaffold in medicinal chemistry. The methodology enables access to new chemical space, with implications for drug discovery and beyond.

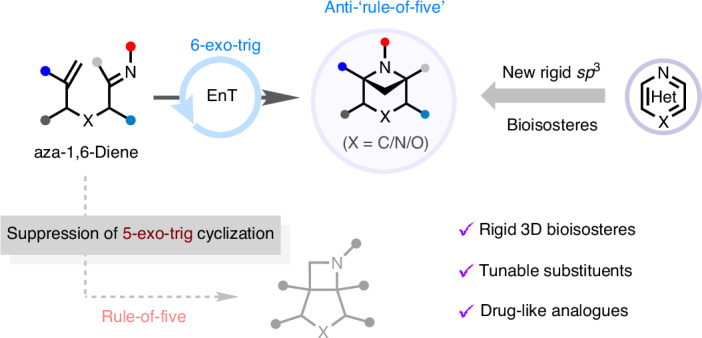

## Abstract (web summary)

Visible-light-mediated intramolecular [2+2] cycloaddition of aza-1,6-dienes gives bridged, not fused, heterocycles, in violation of the ‘rule-of-five’, which dictates that five-membered rings are preferentially formed. This method allows a variety of bridged bicyclic scaffolds to be accessed, enabling drug-relevant properties to be readily tuned.

## Main

**Fig. 1:**
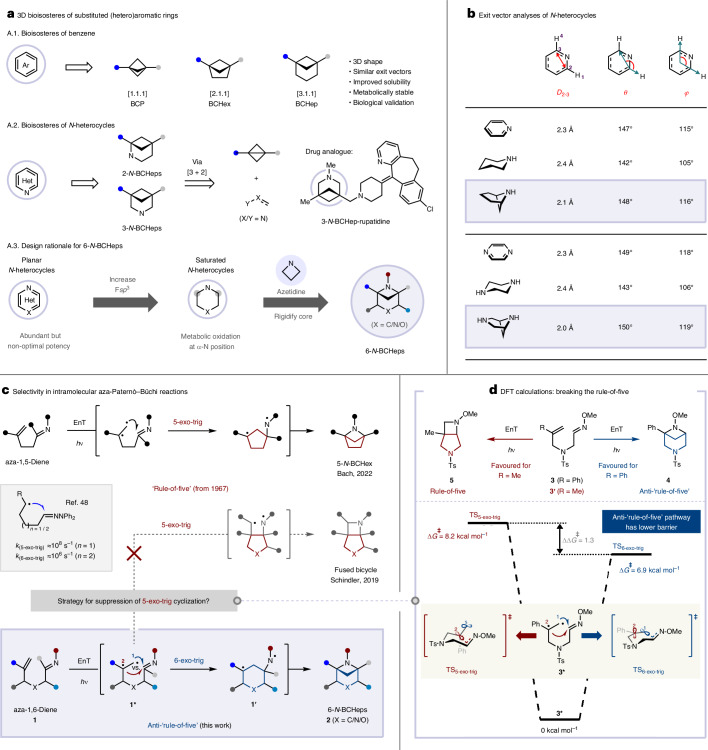
Three-dimensional scaffold mimetics of heteroaromatic rings, showing state of the art and reaction design using anti-rule-of-five intramolecular aza-Paternò–Büchi reaction. **a**, Three-dimensional (3D) bioisosteres of substituted (hetero)aromatic rings. **b**, Exit vector analyses of *N*-heterocycles. *D*_2-3_, distance between carbon atoms 2 and 3; *θ*, angle between C_2_–C_3_ atoms and H_1_–C_2_; *φ*, angle between H_4_–C_3_ and H_1_–C_2_. **c**, Selectivity in intramolecular aza-Paternò–Büchi reactions^39,47,48^. **d**, DFT calculations: breaking the rule-of-five. F*sp*^3^, fraction of *sp*^3^-hybridized carbon atoms; Ts, *p*-toluenesulfonyl; TS, transition state; *k*, rate constant.

Developing synthetic methodologies to construct complex, rigid, three-dimensional molecular architectures is a major focus in contemporary organic synthesis. This is because C(*sp*^3^)-rich polycyclic scaffolds have found widespread application in medicinal chemistry as replacements for planar aromatic rings, which can provide drug candidates with improved physicochemical and pharmacokinetic properties^1–3^. For example, structurally rigid bicyclo[*n*.1.1]alkanes have emerged as effective bioisosteres of benzene rings, with numerous reports of the use of bicyclo[1.1.1]pentane (BCP)^3–12^, bicyclo[2.1.1]hexane (BCHex)^3,8,9,12,13^ and bicyclo[3.1.1]heptane (BCHep)^9,12–14^ groups (Fig. 1a (A.1)). However, analogous isosteric mimetics of heteroaromatic compounds remain underexplored, despite the prevalence of nitrogen heterocycles in Food and Drug Administration-approved small-molecule drugs^15–17^. A notable study by Mykhailiuk and coworkers demonstrated that replacing the 3,5-disubstituted pyridine ring in the antihistamine rupatidine with a 3-azabicyclo[3.1.1]heptane (3-*N*-BCHep) improved solubility, lipophilicity and metabolic stability, underscoring the potential of this motif in medicinal chemistry^18^ (Fig. 1a (A.2)). Since then, substituted *N*-BCHep scaffolds have gained attention as bioisosteres of nitrogen heterocycles, with several studies focusing on 2-*N*-BCHep^19–24^ and 3-*N*-BCHep^18,20,25–31^ derivatives (Fig. 1a (A.2)). By contrast, there are limited examples of the use of substituted 6-*N*-BCHeps^32–34^, wherein the nitrogen atom is incorporated into the four-membered ring of the bicycle (Fig. 1a (A.3)). This modification reduces the basicity of the nitrogen and strengthens the adjacent C–H bonds^33,35,36^, which could offer distinct electronic properties and improved metabolic stability compared with other *N*-BCHep isomers. Furthermore, comparison of various geometric descriptors, including exit vectors, revealed good similarities between the 6-*N*-BCHeps and their aromatic counterparts (Fig. 1b). However, access to substituted 6-*N*-BCHeps is limited by a lack of general methodologies for their synthesis^32^, so there are scant data concerning their suitability as bioisosteres of nitrogen heterocycles^33,34,37^.

The most convergent route to substituted 2-*N*-BCHep and 3-*N*-BCHep derivatives is through intermolecular [3 + 2] cycloadditions of bridgehead-substituted bicyclo[1.1.0]butanes with nitrogen-containing 1,3-dipoles^19–28,31^ (Fig. 1a (A.2)). However, this method is unsuitable for the synthesis of 6-*N*-BCHeps because it requires replacement of the bicyclo[1.1.0]butanes with 2-azabicyclo[1.1.0]butanes, which have not previously been isolated due to their instability^38^. An alternative approach to azabicyclo[*n*.1.1]alkanes is intramolecular [2 + 2] photocycloadditions of acyclic unconjugated aza-dienes (aza-Paternò–Büchi) using energy transfer (EnT) photocatalysis. This method has been successfully applied to the synthesis of 5-azabicyclo[2.1.1]hexane (5-*N*-BCHex) rings from aza-1,5-dienes^39^ (Fig. 1c, top), but there are no reports of analogous reactions of aza-1,6-dienes providing 6-*N*-BCHeps. Intramolecular [2 + 2] photocycloadditions of unconjugated dienes are generally constrained by an empirical rule known as the ‘rule-of-five’, which dictates that the 1,2-biradical formed upon photoexcitation of the substrate undergoes kinetically favoured 5-exo-trig cyclization to form a five-membered ring^40–42^. This results in the generation of bridged BCHex products from 1,5-dienes, whereas 1,6-dienes selectively cyclize to give fused five-membered ring systems instead of bridged six-membered BCHeps. Although a limited number of reports in which geometric restrictions on the 1,6-diene substrates can bias the selectivity towards the BCHep scaffold^43–46^, no general methods for selectivity control or analogous syntheses of 6-*N*-BCHeps exist. Indeed, Schindler and coworkers observed that the intramolecular [2 + 2] photocycloadditions of aza-1,6-dienes (hex-5-enal oximes) afforded fused azetidine products, consistent with the rule-of-five^47^ (Fig. 1c, middle). Nonetheless, we recognized that using readily accessible aza-1,6-dienes as substrates in [2 + 2] cycloadditions could provide an efficient strategy for constructing multisubstituted 6-*N*-BCHeps and could also enable access to previously elusive substituted 3,6-diazabicyclo[3.1.1]heptane (3,6-*N*_2_-BCHep) and 3-oxa-6-azabicyclo[3.1.1]heptane (3-*O*-6-*N*-BCHep) products through simple substrate modification. Therefore, we sought to design an intramolecular photocycloaddition that defies the rule-of-five.

Achieving selective 6-*N*-BCHep formation in [2 + 2] photocycloadditions of aza-1,6-dienes **1** requires the alkene 1,2-biradical in 1* to react with the imine via 6-exo-trig cyclization at C1 instead of 5-exo-trig cyclization at C2 (Fig. 1c, bottom). This is challenging due to the disparity in rates of ring formation, with 5-exo-trig cyclization of non-stabilized alkyl radicals onto hydrazones reported to be more than 100 times faster than 6-exo-trig cyclization^48^ (Fig. 1c, middle). We hypothesized that a radical stabilization strategy could be used to overturn this inherent kinetic preference, wherein the introduction of a radical-stabilizing group at C2 of the alkene in **1** could reduce the rate of cyclization at C2 of the 1,2-biradical intermediate 1*, resulting in selective 6-exo-trig cyclization of the more reactive C1 position. In addition, the group at C2 would stabilize the resulting 1,4-biradical intermediate, thus providing a thermodynamic driving force for the formation of the 6-membered ring intermediate **1ʹ**, before radical recombination yields the bridged bicyclic product **2**. Support for this hypothesis was provided by a density functional theory (DFT) study of the transition state energies of two cyclization pathways of 1,2-biradical 3*, the precursor to 3,6-*N*_2_-BCHep **4** (Fig. 1d). Cyclization of C2-methylated substrate **3ʹ** led to the expected 5-exo-trig cyclization (see Supplementary Section 5 for DFT results), whereas introducing a phenyl ring as a radical-stabilizing group at C2 kinetically suppressed this undesired pathway, thereby enabling the anti-rule-of-five 6-exo-trig cyclization. Interestingly, this same selectivity reversal effect is not observed for the analogous 1,6-dienes^49–51^, highlighting the importance of the presence of the oxime.

Here, we report the successful implementation of this anti-rule-of-five intramolecular [2 + 2] photocycloaddition of aza-1,6-dienes using EnT photocatalysis with visible light, which provides a general strategy for the synthesis of substituted 6-*N*-BCHeps, 3,6-*N*_2_-BCHeps and 3-*O*-6-*N*-BCHeps. These structurally rigid motifs exhibit well-defined exit vectors and could therefore serve as bioisosteres for a range of nitrogen-containing heterocycles, including both aromatic and saturated systems. Moreover, the rich downstream chemistry offered by these compounds further underscores their synthetic utility in accessing structurally complex molecules. This allowed facile preparation of a 3,6-*N*_2_-BCHep analogue of a piperazine-based drug, whose physicochemical properties were tested to evaluate the potential of these scaffolds as bioisosteres in medicinal chemistry.

## Results and discussion

### Reaction design and optimization

To evaluate our hypothesis, aza-1,6-diene **3** containing an oxime and radical-stabilizing phenyl ring at C2 was chosen as the model cycloaddition precursor (Table 1). After evaluation of various parameters, we found that this reaction was indeed viable. Optimum conditions required the use of [Ir(dF[CF_3_]ppy)_2_(dtbbpy)]PF_6_ (1 mol%) as the photocatalyst under 427-nm light, in acetonitrile (0.1 M) at room temperature, which gave 3,6-*N*_2_-BCHep **4** in 68% isolated yield together with 13% of the fused product **4a** (entry 1). This showed that the intramolecular [2 + 2] photocycloaddition of aza-1,6-dienes could undergo the anti-rule-of-five pathway through tuning the reactivity of the 1,2-biradicals (1*; Fig. 1c). Alternative photocatalysts with different redox potentials and triplet energies were tested (entries 2–5), and a correlation was found between the yield and triplet energy of the photocatalyst, but not with redox potentials, indicating that the reaction probably occurs through triplet EnT rather than a photoredox process^52^. The reaction was found to be relatively insensitive to solvent polarity (Supplementary Table 1), which again supports an EnT mechanism, where a neutral encounter complex rather than charge separation is involved^53^. Although thioxanthone, which was discovered to be similarly as effective at promoting the desired reaction of substrate **3**, represents a cost-effective and practical alternative photosensitizer, it was deemed to be less general in its reactivity with a broad array of substrates. Finally, control experiments showed that both light and photocatalyst were necessary (entries 6 and 7).

**Table 1:** Optimization of the reaction conditions


Entry	Photocatalyst	*E*_T_ (kcal mol^−1^)	*E*_1/2_ [M^*^/M^•−^] (V)	*E*_1/2_ [M^•+^/M^*^] (V)	4 (% yield)	4a (% yield)
1	[Ir(dF[CF_3_]ppy)_2_(dtbbpy)]PF_6_	61.8	+1.21	–0.89	69 (68)	13 (13)
2	[Ir(ppy)_2_(dtbbpy)]PF_6_	49.2	+0.66	–0.96	0	0
3	*fac*-[Ir(ppy)_3_]	58.1	+0.31	–1.73	0	0
4	*fac*-[Ir(dFppy)_3_]	63.5	+0.34	–1.44	60	13
5^a^	Thioxanthone (10 mol%)	65.6	+1.18	–1.11	65	10
6	None	–	–	–	0	0
7^b^	[Ir(dF[CF_3_]ppy)_2_(dtbbpy)]PF_6_	61.8	+1.21	–0.89	0	0

The yields were determined by quantitative ^1^H NMR spectroscopy of the crude reaction mixture using CH_2_Br_2_ as the internal standard. The yields in parentheses are of isolated products. ^a^A 390-nm light-emitting diode (LED) was used. ^b^Reaction performed in the dark. *E*_1/2_, half-wave potential; *E*_T_, triplet excited-state energy; ppy, 2-phenylpyridine; dtbbpy, 4,4′-di-*tert*-butyl-2,2′-bipyridine.

### Substrate scope

**Fig. 2:**
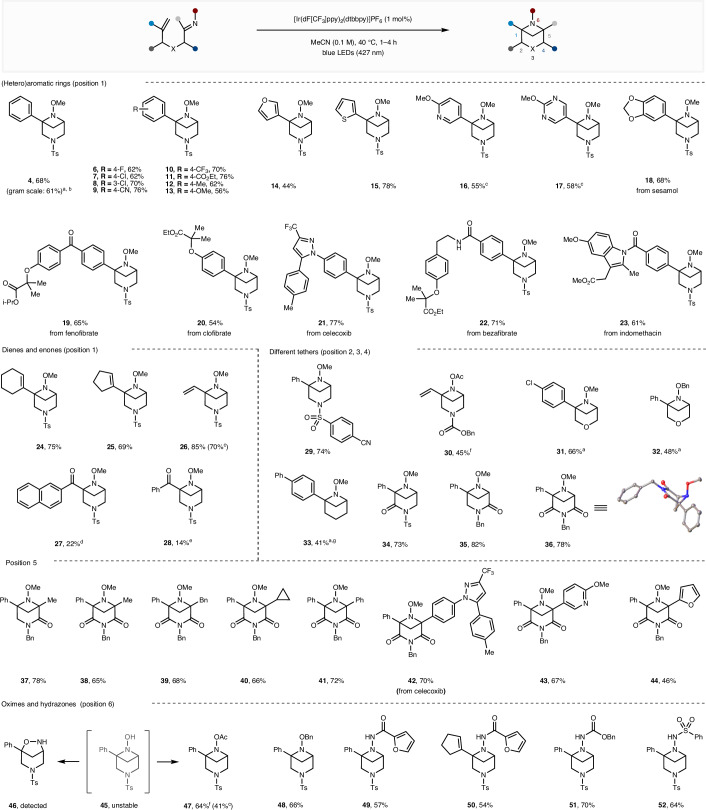
Substrate scope of the 6-*N*-BCHep synthesis. Reactions were performed with a mixture of *E*/*Z* oximes (or hydrazones) as the substrates, using 1 mol% [Ir(dF[CF_3_]ppy)_2_(dtbbpy)]PF_6_ in MeCN (0.1 M) under blue LED (427 nm) irradiation for 1–4 h. Yields are of isolated products. ^a^The reaction time is 20 h. ^b^0.5 mol% [Ir(dF[CF_3_]ppy)_2_(dtbbpy)]PF_6_ was used. ^c^Thioxanthone (10 mol%) was used as the photocatalyst under 390-nm LED irradiation. ^d^5 h reaction time, and 22% of the substrate was recovered. ^e^24 h reaction time, and 41% of the substrate was recovered. ^f^CH_2_Cl_2_ (0.1 M) as the solvent, then acylation using Ac_2_O. ^g^DMSO (0.1 M) as the solvent. i-Pr, iso-propyl; Bn, benzyl; Ac, acetyl; DMSO, dimethyl sulfoxide.

Having established the optimized conditions, we then explored the substrate scope (Fig. 2). Variation of the aromatic alkene substituent demonstrated that electron-deficient (**6**–**11**) and electron-rich phenyl rings (**12** and **13**) as well as heteroaromatics (**14**–**17**) could be used, giving the corresponding 3,6-*N*_2_-BCHeps in moderate-to-good yields. The reaction was successfully scaled up to gram scale with lower catalyst loading (0.5 mol%), giving the product **4** in similar yield (61% versus 68%). The reaction was further extended to incorporate aromatic fragments of six marketed pharmaceuticals (**18**–**23**), highlighting the broad functional group tolerance of the photocycloaddition. In all these cases, the fused bicycle side-products were either isolated in low yields or not observed at all (see Supplementary Section 1.11 for details). Importantly, the [2 + 2] photocycloaddition was not limited to aromatic alkenes and could be extended to dienes (**24**–**26**)^54^, which displayed complete regioselectivity for the formation of the bridged bicycle over the fused bicycle. Enones could also be used (**27** and **28**)^55^, although they were lower-yielding and returned substantial quantities of starting material (20–40%). Modifications to the tether between the styrene and oxime were also explored, including other *N*-protecting groups (**29** and **30**), oxygen (**31** and **32**) and all-carbon linkers (**33**). Furthermore, 2,3-amide (**34**), 3,4-amide (**35** and **37**) and 2,3,4-imide (**36** and **38**–**44**) tethers could be used, delivering the bridged bicycles in high yields. Using amide and imide tethers also enabled the bridgehead position (C5) to be substituted with various (hetero)alkyl or aryl groups (**37**–**44**), with examples including pharmaceutically relevant heterocycles (**42** and **43**). In addition to *O*-methyl oximes, it was found that a broader range of oximes (**47** and **48**) and hydrazones (**49**–**52**) underwent successful cycloaddition. While the parent unsubstituted (N–OH) oxime reacted efficiently, the N–OH product **45** was found to be unstable and rearranged to the [3.2.1] bicycle **46**. However, in situ O-acylation prevented this rearrangement and allowed isolation of the stable acetoxy derivative **47** in good yield.

### Synthetic transformations and applications

**Fig. 3:**
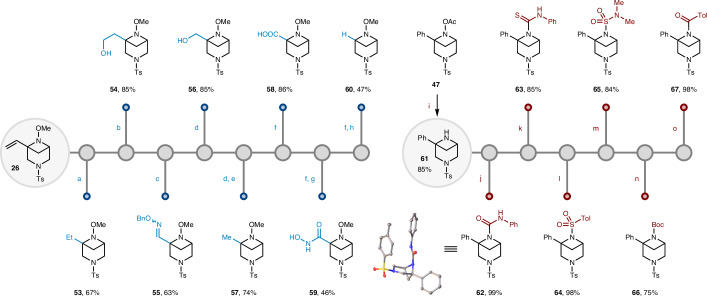
Product diversification. Diversification of products **26** and **61** using a wide variety of conditions. ^a^Pd/C, H_2_. ^b^9-Borabicyclo(3.3.1)nonane (9-BBN), then H_2_O_2_, NaOH. ^c^O_3_, Me_2_S, then NH_2_OBn·HCl, NaOAc. ^d^O_3_, then NaBH_4_. ^e^4-(Trifluoromethyl)benzenesulfonyl chloride, Et_3_N, 4-dimethylaminopyridine (DMAP), then NaBH_4_. ^f^O_3_, Me_2_S, then NaH_2_PO_4_, 2-methyl-2-butene, NaClO_2_. ^g^Oxalyl chloride, then NH_2_OH·HCl, K_2_CO_3_. ^h^*N*-hydroxyphthalimide, *N*,*N*′-diisopropylcarbodiimide, DMAP, then [Ir(ppy)_2_(dtbbpy)]PF_6_, Et_3_N, 1,4-cyclohexadiene, 456-nm blue LEDs. ^i^Iron powder, NH_4_Cl. ^j^Phenyl isocyanate. ^k^Phenyl isothiocyanate. ^l^*p*-Toluenesulfonyl chloride, Et_3_N, DMAP. ^m^*N*,*N*-dimethylsulfamoyl chloride, Et_3_N, DMAP. ^n^Di-*tert*-butyl dicarbonate, DMAP. ^o^*p*-Toluoyl chloride, Et_3_N, DMAP. Tol, 4-methylphenyl; Boc, *tert*-butoxycarbonyl.

Having shown the broad range of 6-*N*-BCHeps that could be accessed using this anti-rule-of-five photocycloaddition of aza-1,6-dienes, we were interested in extending the diversity of accessible products through further derivatisation. Vinyl 3,6-*N*_2_-BCHep **26** is a valuable substrate for diversification because of the synthetic utility of the vinyl group (Fig. 3, left). Importantly, the synthesis of **26** was scalable, with an 85% yield obtained when the cycloaddition was performed on a gram scale. We subsequently demonstrated that the vinyl group in **26** could be hydrogenated to give alkyl-substituted 3,6-*N*_2_-BCHep **53** and subjected to hydroboration/oxidation to give alcohol **54**. Ozonolysis followed by amination, reduction or oxidation provided oxime **55**, alcohol **56** and carboxylic acid **58**, respectively. In addition, methylated product **57** could be accessed by sulfonylation and reduction of alcohol **56**. Finally, derivatization of carboxylic acid **58** by amidation and reductive decarboxylation allowed the generation of hydroxamic acid **59** and the unsubstituted 3,6-*N*_2_-BCHep skeleton **60**.

Further product diversification was possible through reductive cleavage of the oxime-derived N–O bond and functionalization of the resulting secondary amine (Fig. 3, right). While reduction of the N–OMe bond in **4** was not possible due to the sensitivity of the aryl-azetidine group, the more polarized N–O bond in acetoxy derivative **47** was readily cleaved under mild reducing conditions (iron powder)^56^, providing amine **61** in 85% yield. This enabled high-yielding derivatization to generate urea **62**, thiourea **63**, sulfonamide **64**, sulfamide **65**, carbamate **66** and amide **67**, demonstrating the broad range of functional groups that could be incorporated onto the 3,6-*N*_2_-BCHeps.

**Fig. 4:**
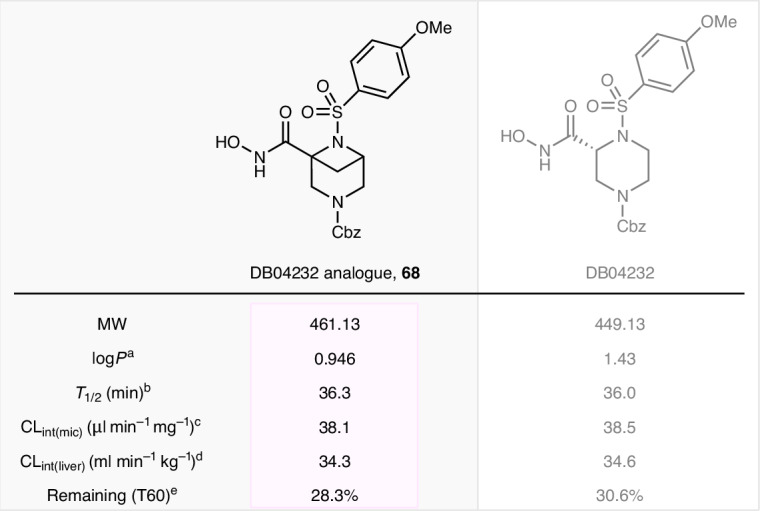
Metabolic profile of DB04232 3,6-*N*_2_-BCHep analogue **68**. ^a^Defined as log*P* at pH 7.4; determined by high-performance liquid chromatography (HPLC) analysis. ^b^Defined as metabolic half-life. ^c^Defined as microsome intrinsic clearance. ^d^Defined as hepatic intrinsic clearance. ^e^Determined at 60 min in the presence of human liver microsomes with NADPH (reduced nicotinamide adenine dinucleotide phosphate). Cbz, benzyloxycarbonyl.

To determine whether the 3,6-*N*_2_-BCHep scaffolds are suitable for use in bioactive molecules, **68** was prepared as an isosteric analogue of the matrix metalloproteinase inhibitor DB04232 and subjected to analysis^57^ (Fig. 4). The physicochemical and pharmacological properties of **68** were assessed in comparison with DB04232. Interestingly, although the isosteric analogue has a greater molecular weight (MW) than DB04232, it has reduced lipophilicity (log*P*). This counterintuitive reduction in lipophilicity has been observed previously and ascribed to an increase in solvent-exposed polar surface area resulting from conformational constraints^58^. Apart from this positive attribute, **68** exhibited similar intrinsic clearance (CL_int_) in human liver microsomes (in vitro human CL_int_, ml min^−1^ kg^−1^) and had a similar half-life (*T*_1/2_) compared with DB04232. These findings indicate that **68** is at least as metabolically stable as DB04232, demonstrating the potential for application of 3,6-*N*_2_-BCHep analogues as isosteres of frequently used heterocycles in drug candidates. However, further studies on these systems, including efficacy analysis and comparisons with unsaturated analogues, are required before the prospective utility of these systems can be fully evaluated.

## Conclusions

In this study, we have introduced a visible-light-mediated intramolecular [2 + 2] cycloaddition of aza-1,6-dienes, a synthetic approach that defies the long-standing rule-of-five in radical cyclization. By exploiting differential radical stabilization, we demonstrated that this strategy can be used to access a diverse range of substituted 6-azabicyclo[3.1.1]heptane (6-*N*-BCHep) scaffolds, a previously challenging to access class of bridged nitrogen heterocycles. The methodology demonstrates broad functional group tolerance and enables selective substitution at multiple positions, expanding the chemical space for medicinal chemistry applications. Moreover, preliminary studies on the newly synthesized 6-*N*-BCHeps show their potential as bioisosteres, offering similar or enhanced pharmacokinetic properties compared with their saturated counterparts. This work not only provides a synthetic tool for accessing complex heterocycles but also contributes to the ongoing exploration of three-dimensional chemical space, paving the way for future developments in medicinal chemistry and beyond.

## Methods

### General procedure for intramolecular [2 + 2] cyclization

An oven-dried round-bottom flask containing aza-1,6-dienes (1.0 equiv.) and [Ir(dF(CF_3_)ppy)_2_(dtbbpy)](PF_6_) (1 mol%) was sealed and subjected to three N_2_ evacuation–refill cycles before anhydrous solvent (0.1 M) was added. The flask was placed at a distance of approximately 1 cm from a 40 W KSPR160L-427 nm (or 390 nm) Kessil light (100% intensity), and the reaction was stirred under continuous irradiation under a nitrogen atmosphere for 1–4 h until complete (judged by thin-layer chromatography analysis). The solvent was removed in vacuo, and the crude product was purified by flash column chromatography.

## Supplementary information

Supplementary Information Experimental details, Supplementary Sections 1–7, Figs. 1–11 and Tables 1–7.

Supplementary Data 1 X-ray crystallographic data for compound **36**, CCDC 2451817.

Supplementary Data 2 X-ray crystallographic data for compound 62, CCDC 2451818.

Supplementary Data 3 DFT calculations, thermochemical data and XYZ coordinates.

## Data Availability

The X-ray crystallographic coordinates for structures **36** and **62** reported in this study have been deposited at the Cambridge Crystallographic Data Centre (CCDC), under deposition numbers 2451817 and 2451818, respectively. These data can be obtained free of charge via The Cambridge Crystallographic Data Centre at www.ccdc.cam.ac.uk/data_request/cif. All other data are available in the article or its Supplementary Information. A preprint version of this work was previously posted on ChemRxiv at 10.26434/chemrxiv-2025-285mh.
