# Supplementary material for: Breaking the ‘rule-of-five’ to access bridged bicyclic heteroaromatic bioisosteres
Source: Nat Synth. 2026 Feb 11;5(5):790–7. doi: 10.1038/s44160-026-00990-0 (PMC13171625; doi:10.1038/s44160-026-00990-0)
Supplement: Supplementary file 1 — Experimental details, Supplementary Sections 1–7, Figs. 1–11 and Tables 1–7. [file 44160_2026_990_MOESM1_ESM.pdf]

nature synthesis

Supplementary information

https://doi.org/10.1038/s44160-026-00990-0

Breaking the 'rule-of-five' to access bridged bicyclic heteroaromatic bioisosteres

In the format provided by the authors and unedited

Table of Contents

1. Synthetic Procedures and Characterisation Data 3

1.1. General Considerations 3

1.1.1 Glassware, Solvents and Reagents 3

1.1.2. Chromatography and Instrumentation 3

1.1.3. Naming of Compounds 4

1.2. Optimisation Table 5

1.3. Unsuccessful Substrates 7

1.4 Intramolecular [2+2] Cycloaddition of 1,6-dienes 8

1.5. Compound List and Numbering 9

1.5.1 Precursors 9

1.5.2. Substrate Scope 12

1.5.3. Product Diversification 14

1.5.4. Analogue of DB04232 14

1.6. General Procedure A for Cyclisation Precursor Synthesis 15

1.7. General Procedure B for Cyclization Precursor Synthesis 25

1.8. General Procedure C for Cyclization Precursor Synthesis 54

1.9. General Procedure D for Cyclization Precursor Synthesis 59

1.10 General Procedure E for Cyclization Precursor Synthesis 62

1.11. General Procedure F for Intramolecular [2+2] Cyclization 78

1.12 Procedures for Transformation of Product (Vinyl Group) 119

1.12.1 Hydrogenation of Alkene 119

1.12.2 Hydroboration-Oxidation of Alkene 119

1.12.3. Ozonolysis Followed by Amination of Alkene 120

1.12.4. Ozonation Followed by Reduction of Alkene 122

1.12.5 Deoxygenation of Alcohol 56 123

1.12.6 Ozonation Followed by Pinnick Oxidation of Alkene 124

1.12.7 Synthesis of Carboxylic Acid Derivatives 125

1.12.8 Decarboxylation of Carboxylic Acid 58 126

1.13 Procedures for *N*-Deprotection and *N*-Functionalization 129

1.14. Procedures for Analogue of DB04232 Synthesis 135

1.15. Electrochemical Measurements of Compound 3 141

2. NMR Spectra 142

3. Determination of LogP (pH 7.4) 336

4. Assessment of Metabolic Stability in Human Liver Microsomes 337

5. Computational Analysis 343

5.1. General Information 343
5.2. Parameter Calculation 344
5.3. Parameter Analysis 345
5.4. Transition State Analyses for Radical Cyclization: Breaking the Rule of 5 346
6. Crystallographic Data (X-Ray) 349
7. References 359

1. Synthetic Procedures and Characterisation Data

1.1. General Considerations

1.1.1 Glassware, Solvents and Reagents

All reactions were conducted under an inert atmosphere of nitrogen using standard Schlenk manifold techniques unless mentioned otherwise. All glassware was oven-dried at >100 °C and allowed to cool to room temperature under a positive pressure of nitrogen. Reactions were monitored by TLC until deemed complete using aluminium-backed silica plates. Plates were visualised under ultraviolet light (254 nm) and/or by staining with $KMnO_4$ solution. Cooling of reaction mixtures to 0 °C was achieved using an ice-water bath. Cooling of reaction mixtures to −78 °C was achieved using a dry ice-acetone bath. “Room temperature” refers to an ambient temperature of 23 ± 2 °C.

All anhydrous solvents (MeOH, CH_3CN, acetone, DMF, DMSO, 1,4-dioxane, Et_2O, THF, CH_2Cl_2 etc.) were commercially supplied (ACROS). All reagents were purchased from commercial sources [Sigma Aldrich (Merck), Across, Fischer, Fluorochem Ltd, TCI, etc.] and used as received unless otherwise stated. Irradiation of reaction mixtures was achieved using a 40W KSPR160L-390 nm (or 427 nm and 456 nm) Kessil light (100% intensity). Brine refers to a saturated aqueous solution of NaCl.

1.1.2. Chromatography and Instrumentation

Thin layer chromatography (TLC) was performed to monitor reactions when practical using Merck Kieselgel 60 F254 fluorescent treated silica, which was visualized under UV light, or by staining with aqueous basic potassium permanganate followed by heating, *p*-anisaldehyde solution followed by heating, Hanessian’s stain (CAM stain) followed by heating, or an ethanolic solution of phosphomolybdic acid followed by heating.

Flash column chromatography (FCC) was carried out using Sigma-Aldrich silica gel (60 Å, 230–400 mesh, 40–63 µm) or a Biotage IsoleraTM flash purification system.

NMR spectra were recorded at various field strengths, as indicated, using Bruker 400 MHz, Bruker 500 MHz or Bruker Cryo 600 MHz for ^{1}H, ^{13}C, ^{19}F acquisitions. All NMR spectra were recorded at ~25 °C in $CDCl_3$ unless otherwise stated. All reported ^{1}H and ^{13}C chemical shifts (δ_H, δ_C) are referenced to the residual signal of deuterated solvents ($CDCl_3$: δ_H = 7.26 ppm, δ_C = 77.16 ppm; $(CD_3)_2SO$: δ_H = 2.50 ppm, δ_C = 39.52 ppm; $(CD_3)_2CO$: δ_H = 2.05 ppm, δ_C = 206.26 ppm; CD_3CN: δ_H = 1.94 ppm, δ_C = 118.26 ppm; CD_2Cl_2: δ_H = 5.32 ppm, δ_C = 53.84 ppm). Chemical shifts (δ) are reported in parts per million (ppm) to the nearest 0.01 ppm for ^{1}H, ^{13}C and ^{19}F NMR. Coupling constants (*J*) are reported in Hertz (Hz). Multiplicities are reported as followings: s (singlet), d (doublet), t (triplet), q (quartet), pent. (quintet), hept. (septet), m (multiplet), br. (broad signal), app.

(apparent). The ^{1}H NMR spectra are reported as follows: chemical shift (multiplicity, coupling constants, number of protons, assignment). NMR assignments were made according to spin systems, using two-dimensional NMR spectroscopy (COSY, HSQC, HMBC) to assist the characterisation.

High resolution mass spectra (HRMS) were recorded on a Bruker Daltonics MicrOTOF II by Electrospray Ionisation (ESI); a Thermo Scientific QExactive by Electron Ionisation (EI); a Thermo Scientific Orbitrap Elite by ESI or Atmospheric Pressure Chemical Ionisation (APCI); or a Bruker UltrafleXtreme by Matrix-assisted Laser Desorption/Ionisation (MALDI).

IR spectra were recorded neat as a thin film on a Perkin Elmer Spectrum One FT-IR. Selected absorption maxima (ν_{max}) are reported in wavenumbers (cm^{-1})

1.1.3. Naming of Compounds

Compound names are those generated by ChemDraw Professional 20.0 software (PerkinElmer), following the IUPAC nomenclature.

1.2. Optimisation Table

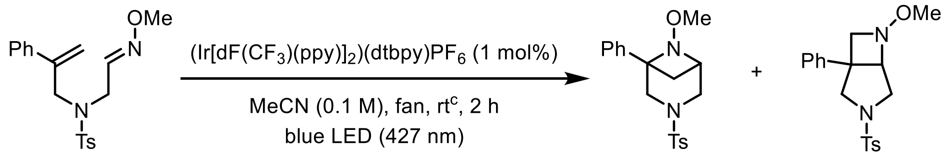

Entry	variation	$E_{1/2}(M^*/M^{\bullet-})$ (V)	$E_{1/2}(M^{\bullet-}/M^*)$ (V)	yield of **4** (%)	yield of **4a** (%)
**1**	no	+1.21	-0.89	69 (68)	13 (13)
	(E_T = 61.8 kcal/mol)				
**2**	$[Ru(bpy)_3](PF_6)_2$ as PC	+0.77	-0.81	0	0
	(E_T = 46.5 kcal/mol)				
**3**	$[Ir(ppy)_2(dtbbpy)]PF_6$ as PC	+0.66	-0.96	0	0
	(E_T = 49.2 kcal/mol)				
**4**	*fac*-$[Ir(ppy)_3]$ as PC	+0.31	-1.73	0	0
	(E_T= 58.1 kcal/mol)				
**5**	*fac*-$[Ir(dF(ppy))_3]$ as PC	+0.34	-1.44	60	13
	(E_T= 63.5 kcal/mol)				
**6**[a]	thioxanthone (10 mol%) as PC	+1.18	-1.11	65	10
	(E_T=65.6 kcal/mol)				
**7**	THF as solvent			65	16
**8**	DCM as solvent			65	13
**9**	DMSO as solvent			67	11
**10**	1,4-dioxane as solvent			65	14
**11**	MeOH as solvent			64	16
**12**	EtOAc as solvent			63	14
**13**	Acetone as solvent			60	11
**14**	MeCN (0.2 M)			65	10
**15**	MeCN (0.02 M)			60	8
**16**	without Ir-F			0	0
**17**	without light			0	0
**18**[b]	gram scale & 0.5 mol% PC			61	11

Yields determined by quantitative NMR spectroscopy of the crude reaction mixture using CH_2Br_2 as the internal standard. Isolated yields in the parentheses. a. 390 nm blue LED was used. b. 20 h. c. **Although the experiment was carried out at room temperature with a fan for cooling, the solvent temperature was often higher. For instance, when acetonitrile was used, the temperature measured with a mercury thermometer reached around 40 °C**.

Supplementary Table S1. Optimisation of the reaction conditions

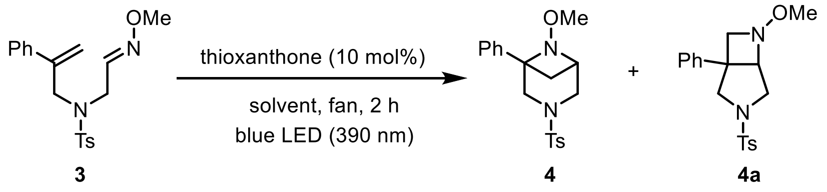

Entry	solvent	temp.	conc.	yield of **4** (%)	yield of **4a** (%)
**1**	DCM	rt[a]	0.1 M	63	14
**2**	THF	rt	0.1 M	62	16
**3**	DMF	rt	0.1 M	60	12
**4**	Acetone	rt	0.1 M	64	10
**5**	EtOAc	rt	0.1 M	63	15
**6**	DCE	rt	0.1 M	63	16
**7**	MeCN	rt	0.1 M	65	10
**8**	MeOH	rt	0.1 M	61	12
**9**	1,4-dioxane	rt	0.1 M	61	13
**10**	Et_2O	rt	0.1 M	60	16
**11**	DMSO	rt	0.1 M	66	7
**12**	DMSO	50 °C[b]	0.1 M	65	9
**13**[c]	Acetone	0 °C	0.05 M	65	7

Yields determined by quantitative NMR spectroscopy of the crude reaction mixture using CH_2Br_2 as the internal standard. a. **Although the experiment was carried out at room temperature with a fan for cooling, the solvent temperature was often higher. For instance, when acetonitrile was used, the temperature measured with a mercury thermometer reached around 40 °C.** b. The reaction was heated with a heating block. c. 3 h.

Supplementary Table S1 (continued). Optimisation of the reaction conditions

1.3. Unsuccessful Substrates

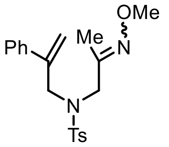

No product formation was detected, and the crude ^{1}H NMR spectrum exhibited significant complexity. This outcome is likely due to the increased steric hindrance of the ketimine moiety relative to its aldimine counterpart, which may inhibit effective reaction progress.

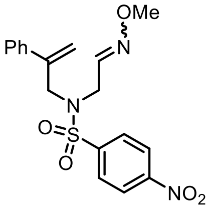

No product was detected, and the crude ^{1}H NMR spectrum after 24 hours indicated complete recovery of the starting material. This may be due to quenching of the photocatalyst's triplet excited state by the nitro group, thereby preventing the reaction from proceeding.

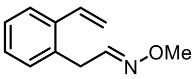

No product formation was detected, and the crude ^{1}H NMR spectrum exhibited significant complexity.

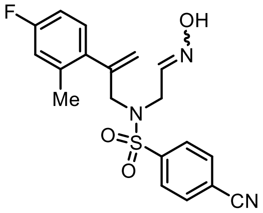

No product formation was observed, and the crude ^{1}H NMR spectrum after 24 hours showed complete recovery of the starting material. This lack of reactivity may stem from the steric hindrance introduced by the 2-methyl substituent on the styrene ring, which likely enforces a non-coplanar geometry between the aromatic ring and the vinyl group, thereby reducing π-conjugation. Disruption of conjugation raises the π–π* excitation energy of the alkene, thus increasing its triplet energy and making energy transfer less efficient.

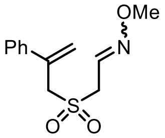

Crude ^{1}H NMR analysis after 2 hours showed a significant amount of unreacted starting material, whereas after 24 hours, the spectrum appeared complex.

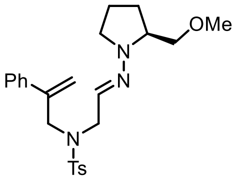

Crude ^{1}H NMR analysis after 2 hours showed a significant amount of unreacted starting material, whereas after 24 hours, the spectrum appeared complex.

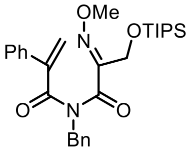

This cyclization precursor was found to be too challanging to obtain, even after exploring various synthetic routes.

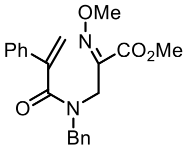

This cyclization precursor was found to be too challanging to obtain, even after exploring various synthetic routes.

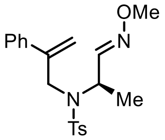

Crude ^{1}H NMR analysis after 2 hours showed a significant amount of unreacted starting material, whereas after 24 hours, the spectrum appeared complex.

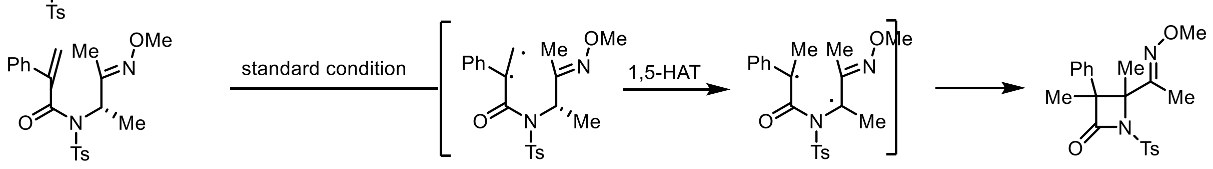

80 %, dr = 1.2:1

Supplementary Figure S1. Unsuccessful substrates

1.4 Intramolecular [2+2] Cycloaddition of 1,6-dienes

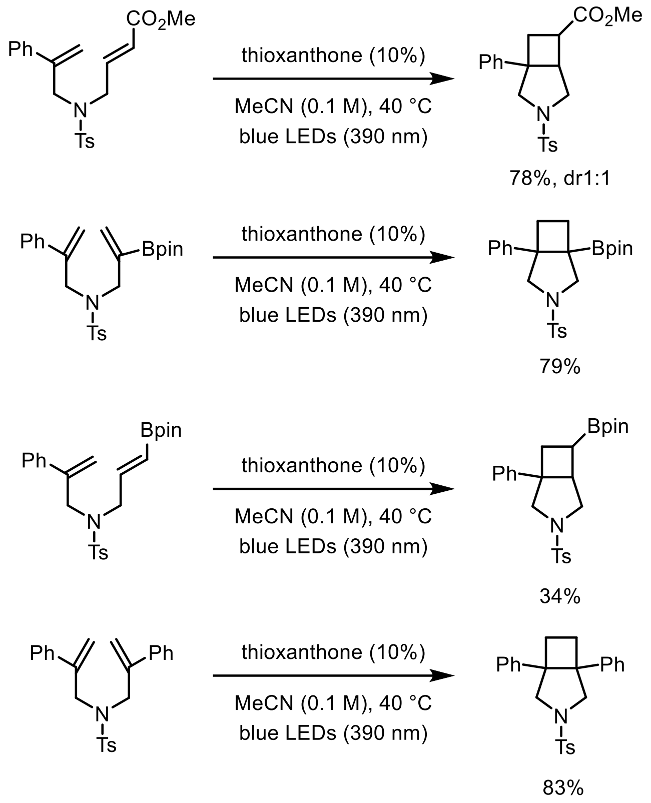

Supplementary Figure S2. Intramolecular [2+2] cycloaddition of 1,6-dienes

1.5. Compound List and Numbering

1.5.1 Precursors

Precursors of compounds **4**, **26**, **29**, **33**, **47**, **48**, **49**, **51**, **52** were prepared using **General Procedure A**

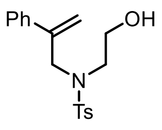
S4a

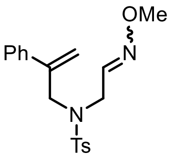
3

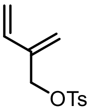
S26a'

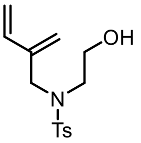
S26a

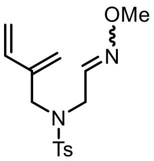
S26b

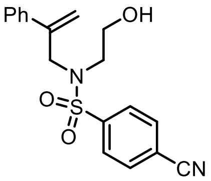
S29a

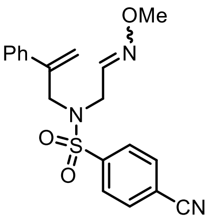
S29b

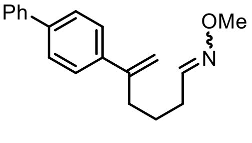
S33b

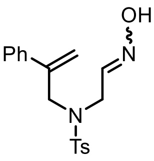
S47b

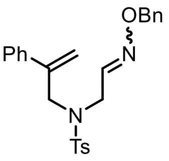
S48b

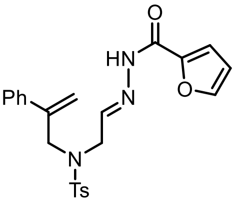
S49b

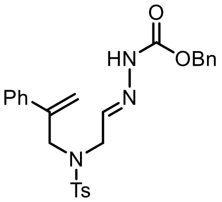
S51b

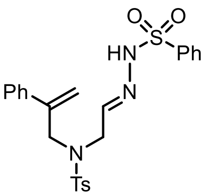
S52b

Precursors of compounds **6-25** and **50** were prepared using **General Procedure B**

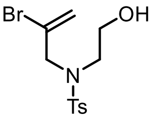
S6a'

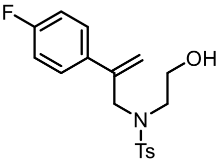
S6a

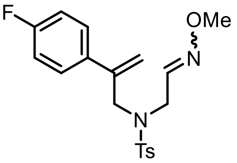
S6b

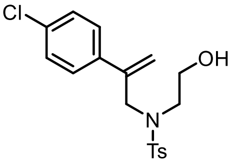
S7a

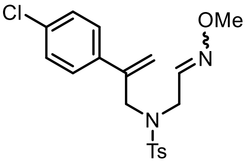
S7b

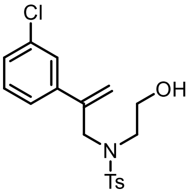
S8a

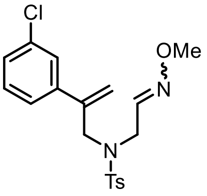
S8b

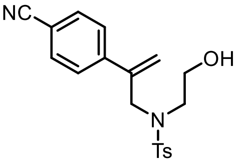
S9a

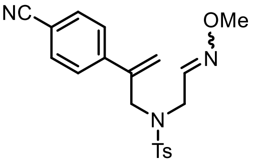
S9b

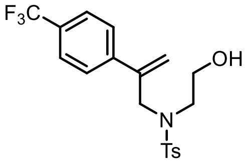
S10a

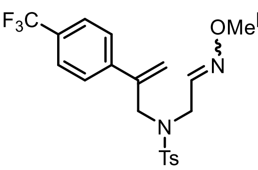
S10b

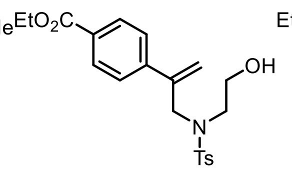
S11a

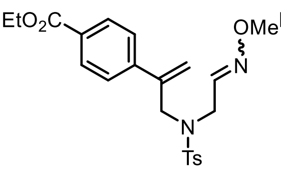
S11b

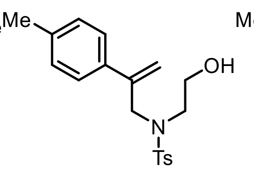
S12a

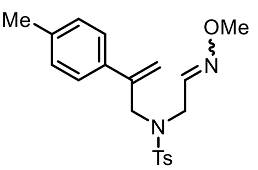
S12b

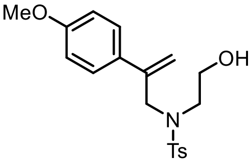
S13a

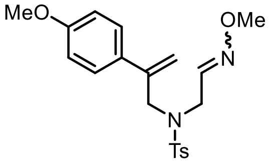
S13b

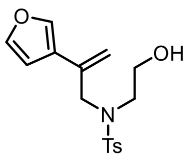
S14a

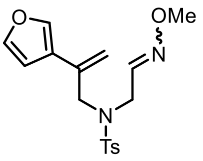
S14b

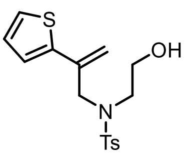
S15a

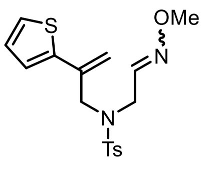

S15b

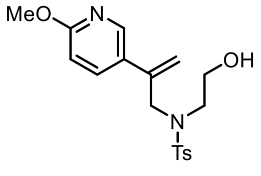

S16a

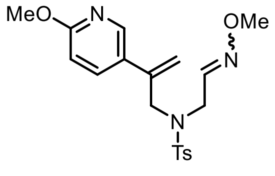

S16b

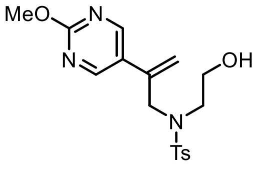

S17a

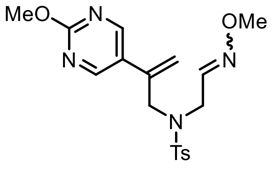

S17b

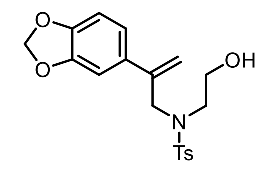

S18a

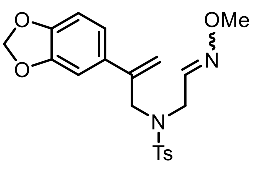

S18b

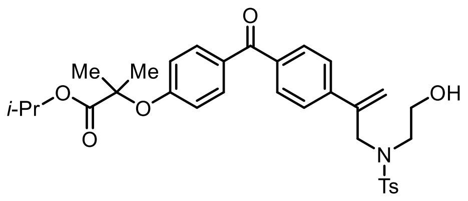

S19a

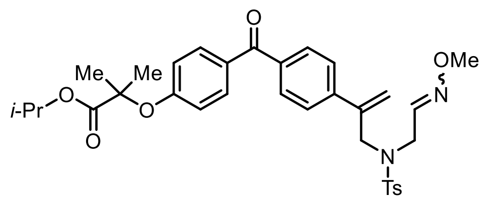

S19b

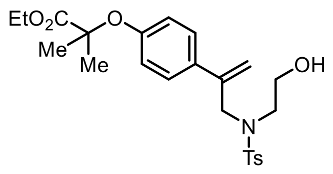

S20a

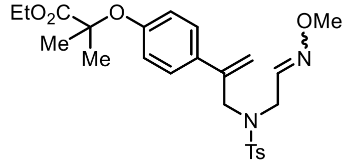

S20b

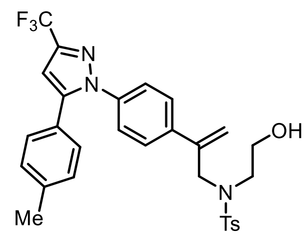

S21a

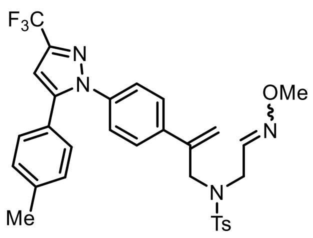

S21b

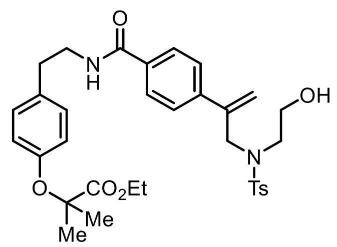

S22a

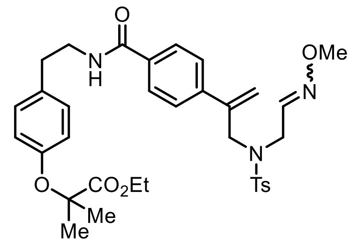

S22b

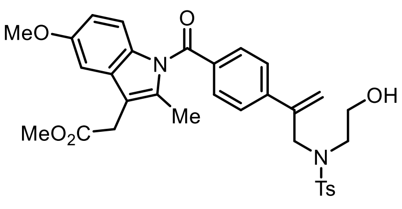

S23a

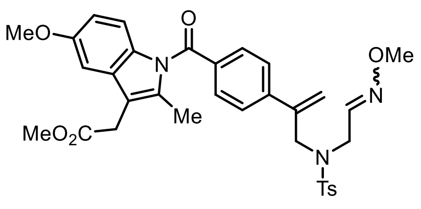

S23b

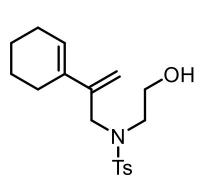

S24a

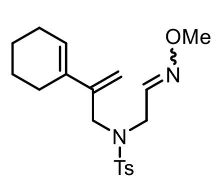

S24b

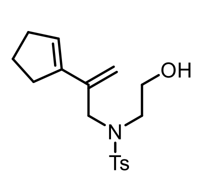

S25a

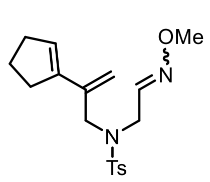

S25b

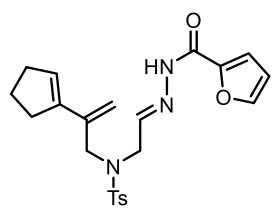

S50b

Precursors of compounds **27** and **28** were prepared using **General Procedure C**

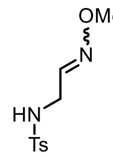
S27a'

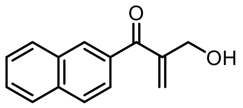
S27a''

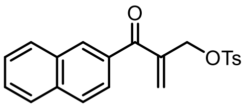
S27a

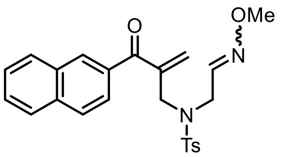
S27b

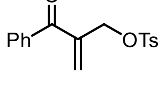
S28a

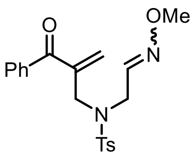
S28b

Precursors of compounds **31** and **32** were prepared using **General Procedure D**

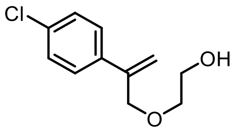
S31a

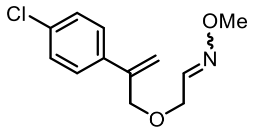
S31b

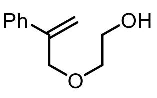
S32a

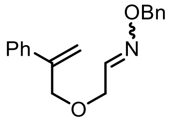
S32b

Precursors of compounds **34-44** were prepared using **General Procedure E**

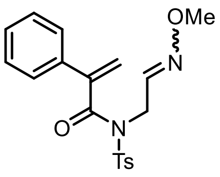
S34b

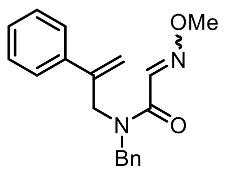
S35b

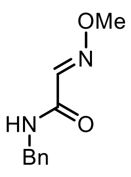
S36a

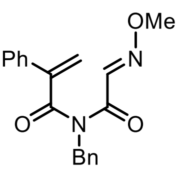
S36b

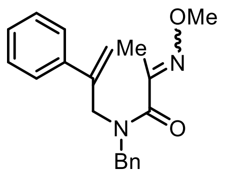
S37b

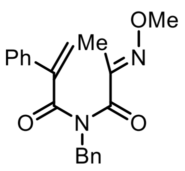
S38b

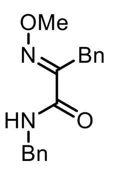
S39a

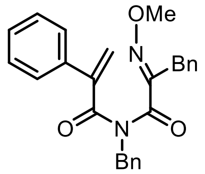
S39b

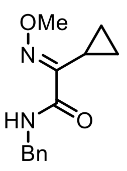
S40a

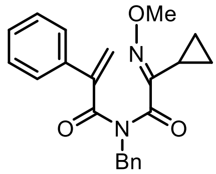
S40b

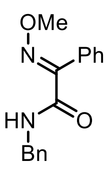
S41a

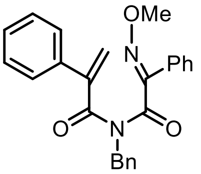
S41b

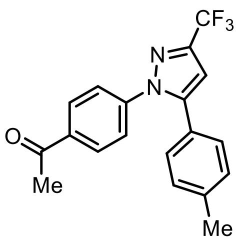
S42a'

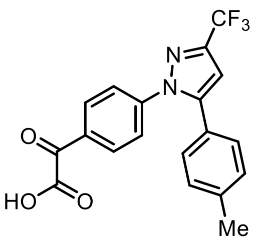
S42a''

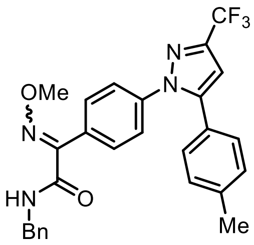
S42a

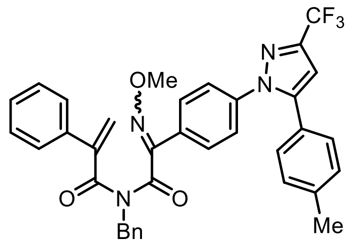
S42b

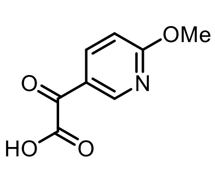
S43a'

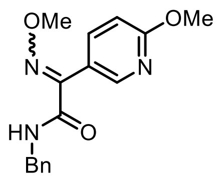
S43a

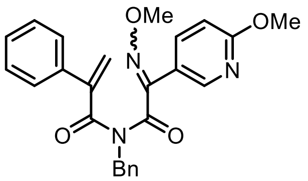
S43b

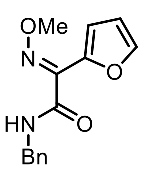
S44a

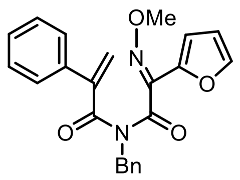
S44b

Note: The synthesis of the precursor of compound **30** is described in **Section 1.14** (“**The synthesis of the analogue of DB04232**”)

1.5.2. Substrate Scope

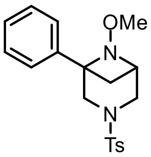
4

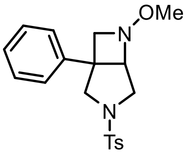
4a

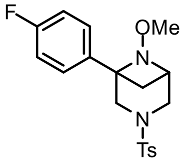
6

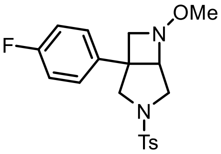
6a

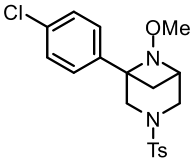
7

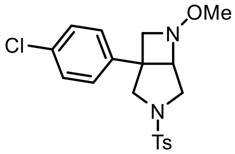
7a

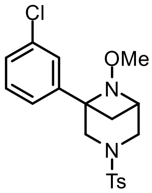
8

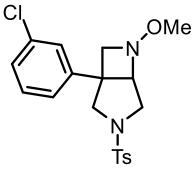
8a

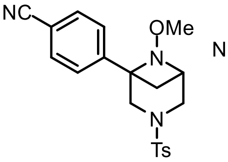
9

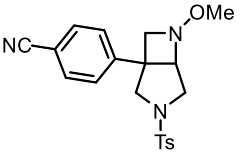
9a

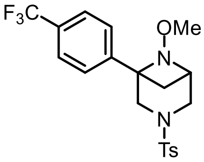
10

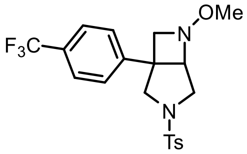
10a

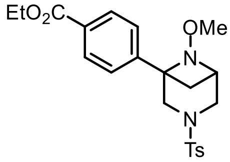
11

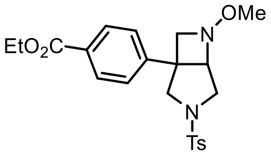
11a

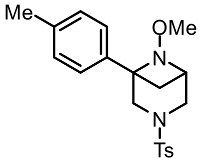
12

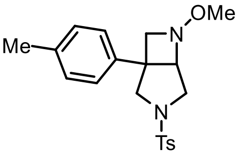
12a

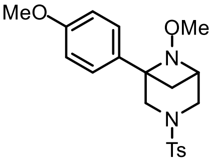
13

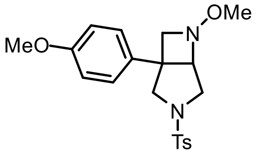
13a

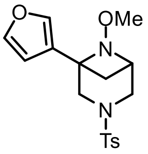
14

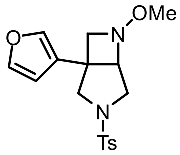
14a

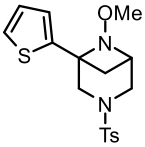
15

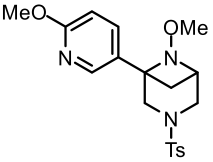
16

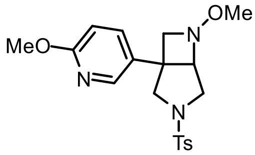
16a

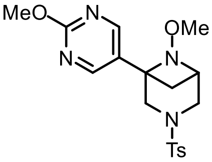
17

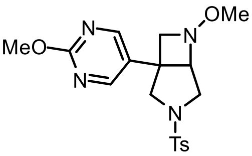
17a

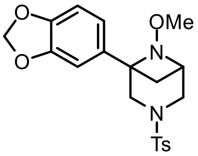
18

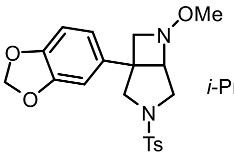
18a

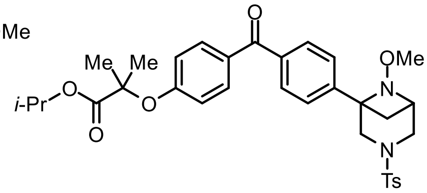
19

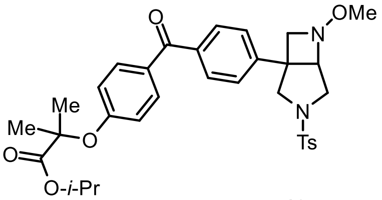
19a

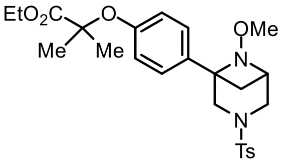
20

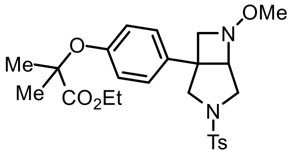
20a

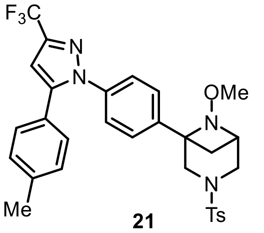
21

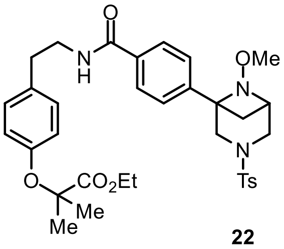
22

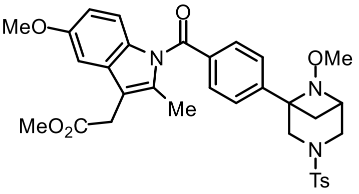
23

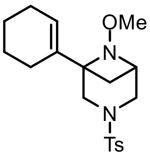
24

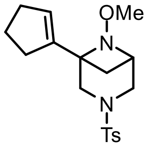
25

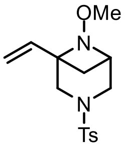
26

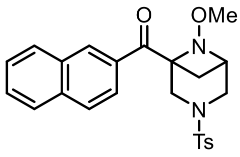
27

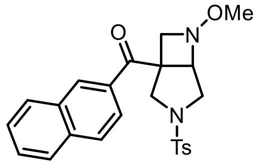
27a

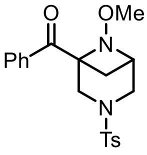
28

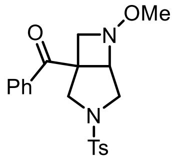
28a

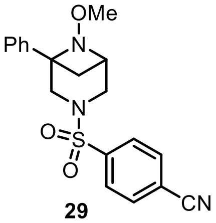
29

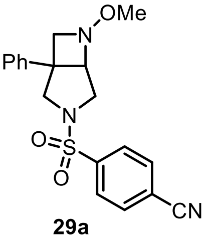
29a

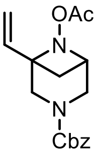
30

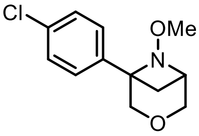
31

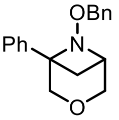
32

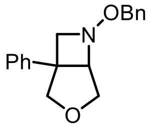
32a

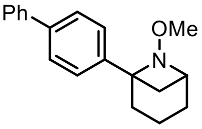
33

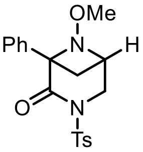
34

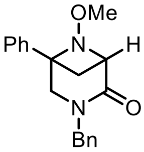
35

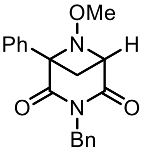
36

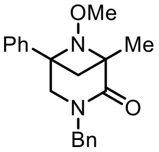
37

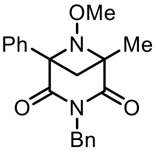
38

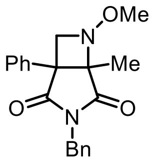
38a

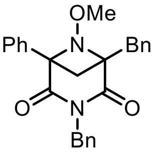
39

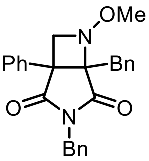
39a

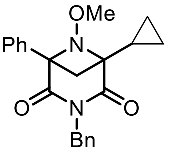
40

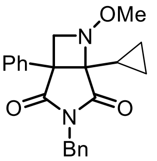
40a

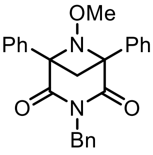
41

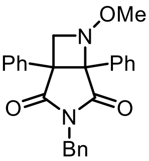
41a

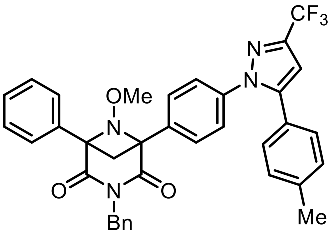
42

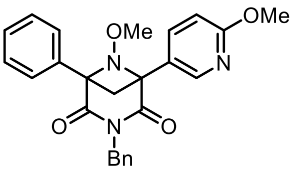
43

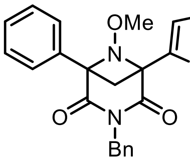
44

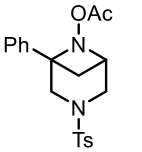
47

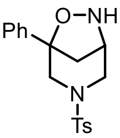
46

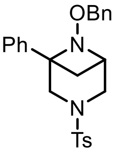
48

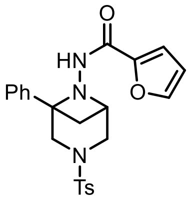
49

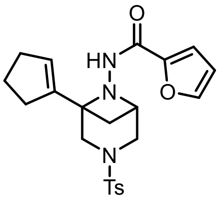
50

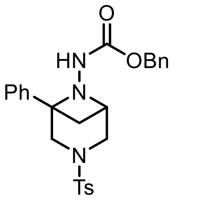
51

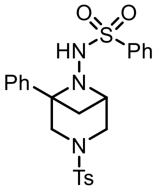
52

1.5.3. Product Diversification

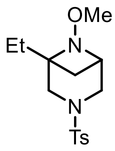
53

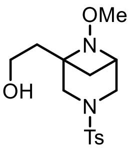
54

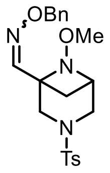
55

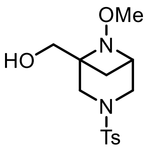
56

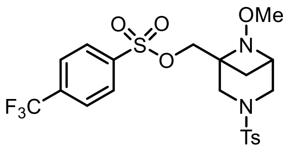
57'

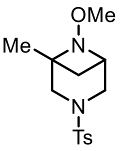
57

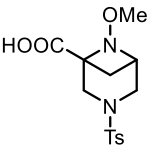
58

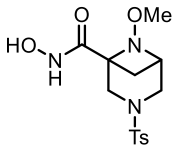
59

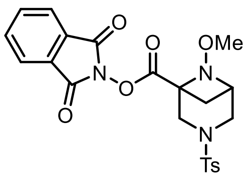
60'

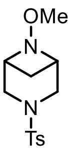
60

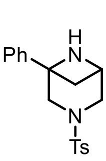
61

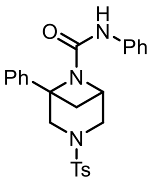
62

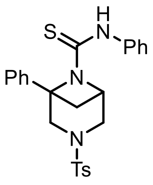
63

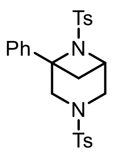
64

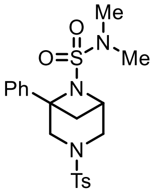
65

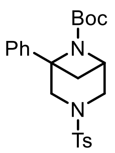
66

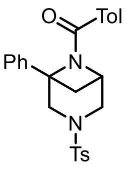
67

1.5.4. Analogue of DB04232

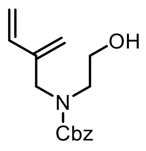
30a

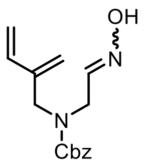
30b

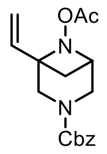
30

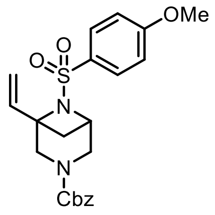
68a

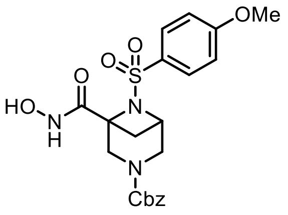
68

1.6. General Procedure A for Cyclisation Precursor Synthesis

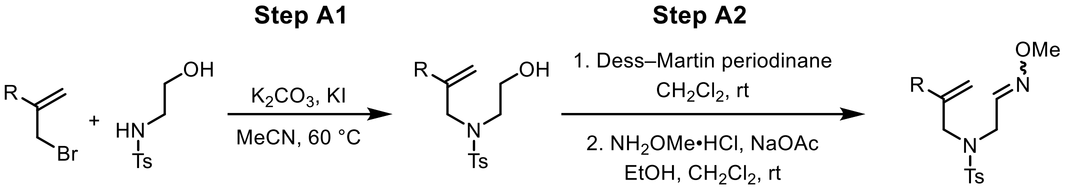

Step A1: An oven-dried round-bottom flask containing *N*-tosyl aminoethanol (1.0 equiv.) was sealed and subjected to three N_2 evacuation/refill cycles before anhydrous acetonitrile (0.5 M) was added. The bromide (1.0 equiv.) was added to the solution, followed by the addition of K_2CO_3 (2.0 equiv.) and KI (0.1 equiv.). The reaction mixture was then stirred at 60 °C for 5-12 h until completion (judged by TLC). The reaction was filtered through anhydrous $MgSO_4$ and washed by CH_2Cl_2. The solvent was evaporated under reduced pressure and the crude product was purified by flash column chromatography.

Step A2: The alcohol (1.0 equiv.) was dissolved in CH_2Cl_2 (0.5 M) in an oven-dried round-bottom flask. Dess-Martin periodinane (1.5 equiv.) was then added and the reaction mixture was stirred in an open flask at room temperature for 0.5-1 h until completion (judged by TLC). The reaction was quenched with sat. aq. $NaHCO_3$ solution and extracted with CH_2Cl_2. The combined organic layers were dried over anhydrous $MgSO_4$, filtered, and concentrated under reduced pressure. The crude aldehyde was then dissolved in ethanol (0.1 M) and CH_2Cl_2 (1.0 M) in an oven-dried round-bottom flask before sodium acetate (4.0 equiv.) and methoxyamine hydrochloride (2.0 equiv.) were added. The reaction was stirred at room temperature until completion (judged by TLC, typically within 1 h). The reaction was quenched with sat. aq. $NaHCO_3$ solution and extracted with CH_2Cl_2. The combined organic layers were dried over anhydrous $MgSO_4$, filtered, concentrated under reduced pressure and purified by flash column chromatography.

Notes: In the final step (amination), the purpose of adding CH_2Cl_2 (1.0 M) is to wash the aldehyde stuck to the wall of the round-bottom flask into the reaction solution.

***N*-(2-hydroxyethyl)-4-methyl-*N*-(2-phenylallyl)benzenesulfonamide (S4a)**

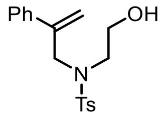

Prepared according to **General Procedure A (step A1)** using *N*-tosyl aminoethanol (6.86 g, 31.9 mmol, 1.00 equiv.), acetonitrile (64.0 mL), α-(bromomethyl)styrene (6.25 g, 31.9 mmol, 1.00 equiv.), K_2CO_3 (8.80 g, 63.77 mmol, 2.00 equiv.), KI (530 mg, 3.19 mmol, 0.1 equiv.). Purification by flash column chromatography (pentane/ethyl acetate, 5:1 to 3:1 to 1:1) afforded alcohol **S4a** as a white solid (9.32 g, 28.2 mmol, 88%).

M.P.: 71-73 °C

^{1}H NMR (400 MHz, $CDCl_3$): δ (ppm) = 7.66 (d, J = 8.3 Hz, 2H), 7.47-7.42 (m, 2H), 7.36-7.26 (m, 5H), 5.48 (d, J = 0.9 Hz, 1H), 5.22 (d, J = 1.1 Hz, 1H), 4.23 (s, 2H), 3.54 (app. q, J = 5.6 Hz, 2H), 3.14 (t, J = 5.5 Hz, 2H), 2.42 (s, 3H), 2.11 (t, J = 6.0 Hz, 1H).

^{13}C NMR (101 MHz, $CDCl_3$): δ (ppm) = 143.83, 143.07, 137.87, 135.27, 129.89, 128.68, 128.36, 127.60, 126.57, 116.89, 61.04, 53.78, 50.20, 21.63.

IR (ATR): $\tilde{\nu}$ (cm^{-1}) = 3497, 3058, 2979, 1598, 1334, 1158, 1088.

HRMS (ESI^+) calcd. for $C_{18}H_{22}NO_3S^+$ $[M+H]^+$: 332.1315, found: 332.1303.

***N*-(2-(methoxyimino)ethyl)-4-methyl-*N*-(2-phenylallyl)benzenesulfonamide (3)**

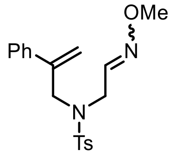

Prepared according to **General Procedure A (step A2)** using alcohol **S4a** (1.66 g, 5.0 mmol, 1.00 equiv.), CH_2Cl_2 (10.0 mL), Dess-Martin periodinane (3.18 g, 7.50 mmol, 1.50 equiv.), $NH_2OMe\cdot HCl$ (840 mg, 10.0 mmol, 2.00 equiv.), NaOAc (1.64 g, 20.0 mmol, 4.00 equiv.), EtOH (50.0 mL), CH_2Cl_2 (5.0 mL). Purification by flash column chromatography (pentane/ethyl acetate, 10:1 to 4:1) afforded oxime **3** as a white solid (1.35 g, 3.77 mmol, 75%, *E/Z* = 70:30).

M.P.: 64-66 °C

^{1}H NMR (500 MHz, $CDCl_3$) δ (ppm) = 7.68-7.62 (m, 2H), 7.48-7.39 (m, 2H), 7.37-7.27 (m, 5H), 7.04 (t, J = 5.9 Hz, 0.7H), 6.33 (t, J = 4.1 Hz, 0.3 H), 5.50 (d, J = 0.9 Hz, 0.3 H,), 5.49 (d, J = 0.9 Hz, 0.7

H), 5.26 (app. q, J = 1.2 Hz, 0.7 H), 5.22 (app. q, J = 1.1 Hz, 0.3 H), 4.24 (s, 1.4H), 4.22 (s, 0.6H), 3.91 (d, J = 4.1 Hz, 0.6H), 3.82-3.79 (m, 2.3 H), 3.75 (s, 2.1 H), 2.44 (s, 0.9 H), 2.43 (s, 2.1 H).

^{13}C NMR (126 MHz, $CDCl_3$) δ (ppm) = 148.00, 145.37, 143.93, 143.78, 142.40, 142.26, 138.29, 137.82, 136.15, 135.51, 129.99, 129.87, 128.65, 128.53, 128.35, 128.20, 127.54, 127.52, 126.69, 126.65, 117.41, 117.06, 62.12, 61.77, 53.28, 51.58, 45.71, 42.88, 21.65, 21.63.

IR (ATR): $\tilde{\nu}$ (cm^{-1}) = 2938, 1598, 1495, 1444, 1339, 1276, 1158, 1091, 1029.

HRMS (ESI^+) calcd. for $C_{19}H_{23}N_2O_3S^+$ $[M+H]^+$: 359.1424, found: 359.1408.

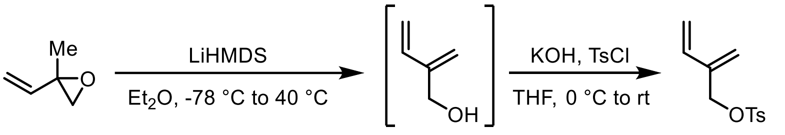

S26a'

Step 1: 2-methylenebut-3-en-1-ol was synthesized using a modified literature procedure.[1] To an oven-dried 250 mL round-bottom flask equipped with a magnetic stir bar and a 15 cm condenser, LiHMDS (13.9 g, 83.2 mmol, 1.20 equiv.) was added in an argon-filled glove box. The flask was sealed with a rubber septum and removed from the glove box. The flask was cooled to −78 °C before the addition of anhydrous Et_2O (90 mL) followed by vigorous stirring of the suspension for 10 min. The cooling bath was then removed, and the flask was allowed to warm to room temperature to give a white suspension. A solution of 2-methyl-2-vinyloxirane (5.82 g, 69.3 mmol, 1.00 equiv.) in anhydrous Et_2O (28 mL) was added dropwise to the reaction. The reaction was heated to reflux at 40 °C leading to the formation of a yellow solution. After 22 hours of stirring, the reaction was cooled to 0 °C and stirred for 10 min. Ice-cold 2 M aqueous HCl (70 mL) was added, and the reaction was stirred vigorously for 30 min. The reaction mixture was extracted three times with Et_2O (3 × 80 mL). The combined organic phases were dried over $MgSO_4$, filtered, and concentrated under reduced pressure in a 0 °C ice/water bath to a volume of 40-50 mL. The solution of volatile alcohol was purified by flash column chromatography (pentane/Et_2O, 10:1 to 5:1 to 1:1) and concentrated under reduced pressure in a 0 °C ice/water bath to a volume of 40-50 mL again. The clean, volatile alcohol solution (in Et_2O and pentane) was used for the next step without further purification.

Step 2: To an oven-dried 500 mL round-bottom flask alcohol solution (in Et_2O and pentane) and TsCl (19.8 g, 103.9 mmol, 1.50 equiv.) were dissolved in anhydrous THF (230 mL, 0.3 M) at 0 °C. KOH powder (15.5 g, 276.8 mmol, 4.00 equiv.) was added and the reaction was stirred at 0 °C for 1 h. The reaction mixture was then quenched with H_2O (500 mL) and extracted with ethyl acetate (3 × 80 mL). The combined organic layers were dried over anhydrous $MgSO_4$, filtered, and concentrated under

reduced pressure. The crude product was purified by flash column chromatography (pentane/ethyl acetate, 5:1 to 3:1) to afford **S26a'** as a colourless oil (5.61 g, 23.6 mmol, 34% for two steps).

Notes: (1) In step 1, the product alcohol is volatile so don't use high-boiling point solvents in the flash column chromatography. (2) In step 2, KOH powder will form a slurry in THF, so a larger magnetic stir bar is necessary to facilitate stirring. Using TLC to monitor the reaction shows incomplete conversion, however, a longer reaction time does not improve the yield, therefore the reaction is quenched after 1 h. (3) Product **S26a'** is unstable when stored at room temperature for more than 1 day, so it should be used immediately in the following step.

2-Methylenebut-3-en-1-yl 4-methylbenzenesulfonate (S26a')

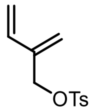

^{1}H NMR (400 MHz, $CDCl_3$) δ (ppm) = 7.80 (d, *J* = 8.4 Hz, 2H), 7.34 (d, *J* = 8.0 Hz, 2H), 6.27 (ddd, *J* = 17.9, 11.1, 0.6 Hz, 1H), 5.25 (s, 1H), 5.21 (s, 1H), 5.15 (d, *J* = 17.9 Hz, 1H), 5.10 (d, *J* = 11.3 Hz, 1H), 4.68 (d, *J* = 1.1 Hz, 2H), 2.44 (s, 3H).

^{13}C NMR (101 MHz, $CDCl_3$) δ (ppm) = 144.99, 138.75, 135.20, 133.16, 129.94, 128.06, 120.22, 115.45, 69.32, 21.74.

IR (ATR): $\tilde{\nu}$ (cm^{-1}) = 2988, 1598, 1358, 1189, 1175, 1097, 992, 936.

HRMS (ESI^+) calcd. for $C_{12}H_{14}O_3SNa^+$ $[M+Na]^+$: 261.0556, found: 261.0564.

***N*-(2-hydroxyethyl)-4-methyl-*N*-(2-methylenebut-3-en-1-yl)benzenesulfonamide (S26a)**

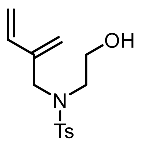

Prepared according to **General Procedure A (step A1)** using *N*-tosyl aminoethanol (1.33 g, 6.18 mmol, 1.00 equiv.), acetonitrile (12.4 mL), 2-methylenebut-3-en-1-yl 4-methylbenzenesulfonate **S26a'** (1.47 g, 6.18 mmol, 1.00 equiv.), K_2CO_3 (1.71 g, 12.39 mmol, 2.00 equiv.), KI (103 mg, 0.62 mmol, 0.10 equiv.). Purification by flash column chromatography (pentane/ethyl acetate, 5:1 to 3:1 to 1:1) afforded alcohol **S26a** as a colorless oil (1.37 g, 4.88 mmol, 79%).

^{1}H NMR (500 MHz, $CDCl_3$) δ (ppm) = 7.71 (d, *J* = 8.3 Hz, 2H), 7.32 (d, *J* = 8.0 Hz, 2H), 6.35 (dd, *J* = 17.8, 11.2 Hz, 1H), 5.56 (d, *J* = 17.8 Hz, 1H), 5.20 (s, 1H), 5.20 (d, *J* = 11.3 Hz, 1H), 5.10 (s, 1H),

3.93 (s, 2H), 3.64 (app. q, J = 5.7 Hz, 2H), 3.15 (t, J = 5.5 Hz, 2H), 2.43 (s, 3H), 2.33 (t, J = 6.1 Hz, 1H).

^{13}C NMR (126 MHz, $CDCl_3$) δ (ppm) = 143.86, 140.80, 136.08, 135.14, 129.93, 127.52, 119.65, 116.43, 61.36, 52.05, 50.64, 21.62.

IR (ATR): $\tilde{\nu}$ (cm^{-1}) = 3489, 2933, 1597, 1447, 1400, 1331, 1306, 1275, 1261, 1154, 1113, 1088, 1047, 1019, 1010.

HRMS (ESI^+) calcd. for $C_{14}H_{20}NO_3S^+$ $[M+H]^+$: 282.1158, found: 282.1153.

***N*-(2-(methoxyimino)ethyl)-4-methyl-*N*-(2-methylenebut-3-en-1-yl)benzenesulfonamide (S26b)**

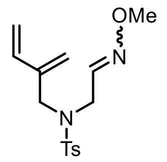

Prepared according to **General Procedure A (step A2)** using alcohol **S26a** (810 mg, 2.88 mmol, 1.00 equiv.), CH_2Cl_2 (5.80 mL), Dess-Martin periodinane (1.83 g, 4.32 mmol, 1.50 equiv.), $NH_2OMe\cdot HCl$ (487 mg, 5.80 mmol, 2.00 equiv.), NaOAc (951 mg, 11.6 mmol, 4.00 equiv.), EtOH (28.8 mL), CH_2Cl_2 (2.9 mL). Purification by flash column chromatography (pentane/ethyl acetate, 10:1 to 3:1) afforded oxime **S26b** as a colorless oil (650 mg, 2.11 mmol, 73%, *E/Z* = 70:30).

^{1}H NMR (500 MHz, $CDCl_3$) δ (ppm) = 7.72-7.67 (m, 2H), 7.34-7.28 (m, 2H), 7.08 (t, J = 5.8 Hz, 0.7 H), 6.53 (t, J = 4.1 Hz, 0.3 H), 6.37-6.27 (m, 1H), 5.57 (d, J = 17.8 Hz, 0.3 H), 5.45 (d, J = 17.8 Hz, 0.7 H), 5.23-5.07 (m, 3H), 3.96-3.80 (m, 4H), 3.80 (s, 0.9 H), 3.73 (s, 2.1 H), 2.42 (s, 3H).

^{13}C NMR (126 MHz, $CDCl_3$) δ (ppm) = 148.14, 145.30, 143.94, 143.80, 139.78, 139.77, 136.11, 136.02, 135.82, 135.34, 130.01, 129.89, 127.43, 127.39, 120.16, 119.44, 116.49, 115.76, 62.08, 61.72, 51.14, 49.32, 45.83, 43.21, 21.59.

IR (ATR): $\tilde{\nu}$ (cm^{-1}) = 2937, 1597, 1494, 1439, 1401, 1338, 1306, 1276, 1261, 1158, 1092, 1030.

HRMS (ESI^+) calcd. for $C_{15}H_{21}N_2O_3S^+$ $[M+H]^+$: 309.1267, found: 309.1262.

4-Cyano-*N*-(2-hydroxyethyl)-*N*-(2-phenylallyl)benzenesulfonamide (S29a)

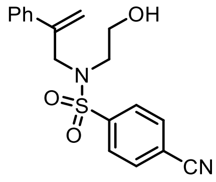

Prepared according to **General Procedure A (step A1)** using *N*-4-Cyanobenzenesulfonyl aminoethanol (730 mg, 3.23 mmol, 1.00 equiv.), acetonitrile (6.50 mL), α-(bromomethyl)styrene (759 mg, 3.87 mmol, 1.20 equiv.), K_2CO_3 (892 mg, 6.46 mmol, 2.00 equiv.), KI (53.1 mg, 0.32 mmol, 0.10 equiv.). Purification by flash column chromatography (pentane/ethyl acetate, 5:1 to 3:1 to 1:1) afforded alcohol **S29a** as a colorless oil (960 mg, 2.81 mmol, 87%).

^{1}H NMR (500 MHz, $CDCl_3$) δ (ppm) = 7.80 (d, *J* = 8.5 Hz, 2H), 7.69 (d, *J* = 8.5 Hz, 2H), 7.34-7.30 (dd, *J* = 6.7, 3.1 Hz, 2H), 7.29-7.24 (m, 3H), 5.42 (s, 1H), 5.20 (s, 1H), 4.31 (s, 2H), 3.58 (app. q, *J* = 5.6 Hz, 2H), 3.24 (t, *J* = 5.7 Hz, 2H), 2.40 (t, *J* = 5.6 Hz, 1H).

^{13}C NMR (126 MHz, $CDCl_3$) δ (ppm) = 143.20, 142.35, 137.84, 132.80, 128.51, 128.25, 127.78, 126.31, 117.30, 117.22, 116.03, 60.27, 52.66, 49.33.

IR (ATR): $\tilde{\nu}$ (cm^{-1}) = 3525, 3092, 2937, 2233, 1445, 1395, 1340, 1158, 1085, 1013.

HRMS (ESI^+) calcd. for $C_{18}H_{18}N_2O_3SNa^+$ $[M+Na]^+$: 365.0930, found: 365.0942.

4-Cyano-*N*-(2-(methoxyimino)ethyl)-*N*-(2-phenylallyl)benzenesulfonamide (S29b)

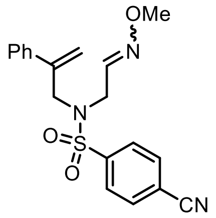

Prepared according to **General Procedure A (step A2)** using alcohol **S29a** (930 mg, 2.72 mmol, 1.00 equiv.), CH_2Cl_2 (5.50 mL), Dess-Martin periodinane (1.73 g, 4.08 mmol, 1.50 equiv.), $NH_2OMe{\cdot}HCl$ (457 mg, 5.44 mmol, 2.00 equiv.), NaOAc (892 mg, 10.9 mmol, 4.00 equiv.), EtOH (27.2 mL), CH_2Cl_2 (2.7 mL). Purification by flash column chromatography (pentane/ethyl acetate, 10:1 to 3:1) afforded oxime **S29b** as a white solid (820 mg, 2.22 mmol, 82%, *E/Z* = 70:30).

M.P.: 77-79 °C

^{1}H NMR (500 MHz, $CDCl_3$) δ (ppm) = 7.80 (d, *J* = 6.0 Hz, 0.6H), 7.79 (d, *J* = 6.1 Hz, 1.4H), 7.74 (d, *J* = 8.5 Hz, 0.6H), 7.71 (d, *J* = 8.5 Hz, 1.4H), 7.40-7.28 (m, 5H), 7.06 (t, *J* = 5.5 Hz, 0.7H), 6.36 (t, *J* = 4.1 Hz, 0.3H), 5.47 (s, 1H), 5.24 (s, 0.7H), 5.21 (s, 0.3H), 4.31 (s, 1.4H), 4.28 (s, 0.6H), 3.94 (d, *J* = 4.2 Hz, 0.6H), 3.85 (d, *J* = 5.6 Hz, 1.4H), 3.81 (s, 0.9H), 3.71 (s, 2.1H).

^{13}C NMR (126 MHz, $CDCl_3$) δ (ppm) = 146.88, 144.23, 143.59, 142.79, 141.82, 141.78, 137.93, 137.58, 132.98, 132.80, 128.61, 128.52, 128.40, 128.28, 127.90, 127.81, 126.50, 118.03, 117.73, 117.28, 117.21, 116.47, 116.22, 62.11, 61.76, 53.06, 51.32, 45.17, 42.50.

IR (ATR): $\tilde{\nu}$ (cm^{-1}) = 2967, 2933, 2235, 1630, 1572, 1463, 1393, 1344, 1301, 1165, 1102, 1029.

HRMS (ESI$^+$) calcd. for $C_{19}H_{20}N_3O_3S^+$ [M+H]$^+$: 370.1220, found: 370.1228.

5-([1,1'-Biphenyl]-4-yl)hex-5-enal *O*-methyl oxime (S33b)

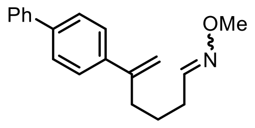

Prepared according to **General Procedure A (step A2)** using 5-([1,1'-biphenyl]-4-yl)hex-5-en-1-ol[2] (756 mg, 3.00 mmol, 1.00 equiv.), CH_2Cl_2 (6.0 mL), Dess-Martin periodinane (1.91 g, 4.50 mmol, 1.50 equiv.), $NH_2OMe{\cdot}HCl$ (504 mg, 6.00 mmol, 2.00 equiv.), NaOAc (984 mg, 12.00 mmol, 4.00 equiv.), EtOH (30.0 mL), CH_2Cl_2 (3.0 mL). Purification by flash column chromatography (pentane/ethyl acetate, 8:1 to 4:1) afforded oxime **S33b** as a colorless oil (590 mg, 2.11 mmol, 70%, *E/Z* = 60:40).

^{1}H NMR (500 MHz, $CDCl_3$) δ (ppm) = 7.65-7.57 (m, 4H), 7.53-7.43 (m, 4H), 7.41-7.35 (m, 1.6H), 6.66 (t, J = 5.5 Hz, 0.4H), 5.40 (s, 1H), 5.14 (s, 1H), 3.90 (s, 1.2H), 3.85 (s, 1.8H), 2.65-2.59 (m, 2H), 2.39 (td, J = 7.6, 5.4 Hz, 0.8H), 2.27 (td, J = 7.5, 6.1 Hz, 1.2H), 1.76-1.67 (m, 2H).

^{13}C NMR (126 MHz, $CDCl_3$) δ (ppm) = 151.48, 150.55, 147.23, 147.21, 140.81, 140.80, 140.37, 139.88, 139.84, 128.89, 127.40, 127.15, 127.08, 126.61, 113.01, 61.69, 61.33, 34.99, 34.71, 29.14, 25.39, 25.26, 24.93.

IR (ATR): $\tilde{\nu}$ (cm^{-1}) = 2936, 1624, 1600, 1487, 1461, 1447, 1403, 1276, 1261, 1078, 1006.

HRMS (ESI$^+$) calcd. for $C_{19}H_{22}NO^+$ [M+H]$^+$: 280.1696, found: 280.1689.

***N*-(2-(hydroxyimino)ethyl)-4-methyl-*N*-(2-phenylallyl)benzenesulfonamide (S47b)**

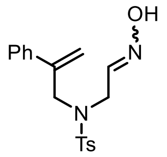

Prepared according to **General Procedure A (step A2)** using alcohol **S4a** (3.10 g, 9.36 mmol, 1.00 equiv.), CH_2Cl_2 (18.8 mL), Dess-Martin periodinane (5.96 g, 14.06 mmol, 1.50 equiv.), $NH_2OH{\cdot}HCl$ (1.30 g, 18.71 mmol, 2.00 equiv.), NaOAc (3.07 g, 37.44 mmol, 4.00 equiv.), EtOH (93.6 mL), CH_2Cl_2 (9.4 mL). Purification by flash column chromatography (pentane/ethyl acetate, 5:1 to 1:1 and CH_2Cl_2/ethyl acetate, 10:1 to 4:1) afforded oxime **S47b** as a white solid (2.51 g, 7.30 mmol, 78%, *E/Z* = 50:50).

M.P.: 81-83 °C

^{1}H NMR (500 MHz, $CDCl_3$) δ (ppm) = 9.04 (br. s, 0.5H), 8.65 (br. s, 0.5H), 7.67 (d, *J* = 8.3 Hz, 1H), 7.64 (d, *J* = 8.4 Hz, 1H), 7.49-7.45 (m, 1H), 7.40-7.26 (m, 6H), 7.10 (t, *J* = 5.9 Hz, 0.5H), 6.44 (t, *J* = 4.0 Hz, 0.5H), 5.49 (s, 0.5H), 5.46 (s, 0.5H), 5.22 (s, 1H), 4.22 (s, 2H), 3.97 (d, *J* = 4.1 Hz, 1H), 3.80 (d, *J* = 5.9 Hz, 1H), 2.43 (s, 1.5H), 2.42 (s, 1.5H).

^{13}C NMR (126 MHz, $CDCl_3$) δ (ppm) = 149.24, 147.00, 143.99, 143.86, 142.24, 142.08, 138.03, 137.66, 135.79, 135.02, 129.95, 129.86, 128.56, 128.46, 128.27, 128.14, 127.46, 127.43, 126.59, 126.56, 117.53, 117.14, 53.37, 51.71, 45.64, 42.54, 21.57, 21.55.

IR (ATR): $\tilde{\nu}$ (cm^{-1}) = 3436, 3057, 2921, 1597, 1494, 1444, 1335, 1276, 1157, 1091, 914.

HRMS (ESI^+) calcd. for $C_{18}H_{21}N_2O_3S^+$ $[M+H]^+$: 345.1267, found: 345.1251.

***N*-(2-((benzyloxy)imino)ethyl)-4-methyl-*N*-(2-phenylallyl)benzenesulfonamide (S48b)**

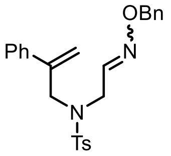

Prepared according to **General Procedure A (step A2)** using alcohol **S4a** (993 mg, 3.00 mmol, 1.00 equiv.), CH_2Cl_2 (6.0 mL), Dess-Martin periodinane (1.91 g, 4.50 mmol, 1.50 equiv.), $NH_2OBn{\cdot}HCl$ (960 mg, 6.0 mmol, 2.00 equiv.), NaOAc (984 mg, 12.0 mmol, 4.00 equiv.), EtOH (30.0 mL), CH_2Cl_2 (3.0 mL). Purification by flash column chromatography (pentane/ethyl acetate, 10:1 to 3:1) afforded oxime **S48b** as a colorless oil (990 mg, 2.28 mmol, 76%, *E/Z* = 55:45).

^{1}H NMR (500 MHz, $CDCl_3$) δ (ppm) = 7.64-7.60 (m, 2H), 7.44-7.41 (m, 1H), 7.38-7.24 (m, 11H), 7.12 (t, *J* = 5.8 Hz, 0.55H), 6.37 (t, *J* = 4.1 Hz, 0.45H), 5.44 (d, *J* = 0.8 Hz, 0.45H), 5.40 (d, *J* = 0.9 Hz, 0.55H), 5.17 (app. q, *J* = 1.1 Hz, 0.45H), 5.10 (app. q, *J* = 1.2 Hz, 0.55H), 5.04 (s, 0.9H), 4.97 (s, 1.1H), 4.21 (s, 0.9H), 4.18 (s, 1.1H), 3.96 (d, *J* = 4.1 Hz, 0.9H), 3.80 (d, *J* = 5.8 Hz, 1.1H), 2.41 (s, 3H).

^{13}C NMR (126 MHz, $CDCl_3$) δ (ppm) = 148.60, 145.87, 143.89, 143.69, 142.20, 142.18, 138.25, 137.75, 137.60, 137.53, 136.24, 135.48, 129.95, 129.82, 128.60, 128.49, 128.31, 128.17, 128.13, 128.06, 128.04, 127.52, 127.49, 126.64, 126.62, 117.43, 117.06, 76.34, 76.00, 53.21, 51.35, 45.61, 42.98, 21.61.

IR (ATR): $\tilde{\nu}$ (cm^{-1}) = 3006, 1598, 1496, 1454, 1341, 1261, 1276, 1159, 1091, 1012.

HRMS (ESI^+) calcd. for $C_{25}H_{27}N_2O_3S^+$ $[M+H]^+$: 435.1737, found: 435.1720.

(*E*)-*N*-(2-(2-(furan-2-carbonyl)hydrazineylidene)ethyl)-4-methyl-*N*-(2-phenylallyl)-benzenesulfonamide (S49b)

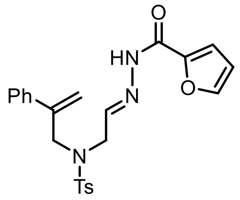

Prepared according to **General Procedure A (step A2)** using alcohol **S4a** (700 mg, 2.11 mmol, 1.00 equiv.), CH_2Cl_2 (4.2 mL), Dess-Martin periodinane (1.35 g, 3.18 mmol, 1.50 equiv.), 2-Furoic acid hydrazide (530 mg, 4.21 mmol, 2.00 equiv.), EtOH (21.0 mL), CH_2Cl_2 (2.0 mL). Purification by flash column chromatography (pentane/ethyl acetate, 5:1 to 1:1 to 1:4 and CH_2Cl_2/ethyl acetate, 5:1 to 2:1) afforded oxime **S49b** as a white solid (610 mg, 1.40 mmol, 66%, *E*-isomer).

M.P.: 148-150 °C

^{1}H NMR (500 MHz, $CDCl_3$) δ (ppm) = 9.13 (s, 1H), 7.63 (d, *J* = 8.3 Hz, 2H), 7.46-7.37 (m, 3H), 7.31-7.24 (m, 6H), 7.08 (br. s, 1H), 6.51 (dd, *J* = 3.6, 1.8 Hz, 1H), 5.44 (d, *J* = 0.8 Hz, 1H), 5.21 (d, *J* = 1.1 Hz, 1H), 4.20 (s, 2H), 3.85 (d, *J* = 5.1 Hz, 2H), 2.41 (s, 3H).

^{13}C NMR (126 MHz, $CDCl_3$) δ (ppm) = 147.71, 146.45, 144.68, 144.10, 142.36, 137.97, 130.02, 128.55, 128.24, 127.62, 126.86, 117.73, 116.44, 112.56, 53.08, 48.70, 21.66.

IR (ATR): $\tilde{\nu}$ (cm^{-1}) = 3006, 2989, 1663, 1590, 1540, 1472, 1340, 1276, 1261, 1160, 1090.

HRMS (ESI^+) calcd. for $C_{23}H_{24}N_3O_4S^+$ $[M+H]^+$: 438.1482, found: 438.1485.

Benzyl (*E*)-2-(2-((4-methyl-*N*-(2-phenylallyl)phenyl)sulfonamido)ethylidene)hydrazine-1-carboxylate (S51b)

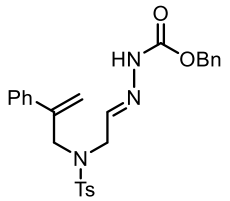

Prepared according to **General Procedure A (step A2)** using alcohol **S4a** (802 mg, 2.42 mmol, 1.00 equiv.), CH_2Cl_2 (4.8 mL), Dess-Martin periodinane (1.54 g, 3.63 mmol, 1.50 equiv.), benzyl carbazate (603 mg, 3.63 mmol, 1.50 equiv.), EtOH (24.2 mL), CH_2Cl_2 (2.4 mL). Purification by flash column chromatography (pentane/ethyl acetate, 5:1 to 3:1 to 1:1) afforded oxime **S51b** as a white solid (810 mg, 1.70 mmol, 70%, *E*-isomer).

M.P.: 156-158 °C

^{1}H NMR (500 MHz, DMSO-d$_6$) δ (ppm) = 10.93 (br. s, 1H), 7.72 (d, *J* = 8.4 Hz, 2H), 7.47-7.44 (m, 2H), 7.41-7.30 (m, 10H), 7.01 (t, *J* = 5.6 Hz, 1H), 5.50 (s, 1H), 5.28 (s, 1H), 5.12 (s, 2H), 4.17 (s, 2H), 3.75 (d, *J* = 5.4 Hz, 2H), 2.39 (s, 3H).

^{13}C NMR (126 MHz, DMSO-d$_6$) δ (ppm) = 153.15, 143.52, 142.75, 142.12, 137.96, 136.56, 135.22, 129.90, 128.43, 128.29, 128.06, 128.00, 127.96, 127.27, 126.34, 116.89, 65.80, 52.00, 48.68, 21.03.

IR (ATR): $\tilde{\nu}$ (cm^{-1}) = 3249, 3032, 1722, 1539, 1455, 1339, 1275, 1260, 1160, 1090, 1032.

HRMS (ESI$^+$) calcd. for $C_{26}H_{28}N_3O_4S^+$ [M+H]$^+$: 478.1795, found: 478.1803.

(*E*)-4-methyl-*N*-(2-phenylallyl)-*N*-(2-(2-(phenylsulfonyl)hydrazineylidene)ethyl)benzene-sulfonamide (S52b)

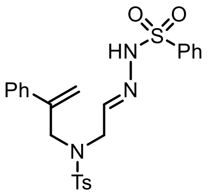

Prepared according to **General Procedure A (step A2)** using alcohol **S4a** (700 mg, 2.11 mmol, 1.00 equiv.), CH_2Cl_2 (4.2 mL), Dess-Martin periodinane (1.35 g, 3.18 mmol, 1.50 equiv.), Benzenesulfonyl hydrazide (430 mg, 2.52 mmol, 1.20 equiv.), EtOH (21.1 mL), CH_2Cl_2 (2.1 mL). Purification by flash column chromatography (pentane/ethyl acetate, 5:1 to 3:1 to 1:1 and CH_2Cl_2/ethyl acetate, 10:1 to 5:1) afforded oxime **S52b** as a white solid (650 mg, 1.35 mmol, 64%, *E*-isomer).

M.P.: 150-152 °C

^{1}H NMR (500 MHz, DMSO-d$_6$) δ (ppm) = 11.35 (s, 1H), 7.78 (d, *J* = 7.2 Hz, 2H), 7.65-7.55 (m, 5H), 7.37 (d, *J* = 8.2 Hz, 2H), 7.31 (s, 5H), 6.89 (t, *J* = 5.2 Hz, 1H), 5.29 (s, 1H), 4.98 (s, 1H), 4.02 (s, 2H), 3.65 (d, *J* = 5.2 Hz, 2H), 2.40 (s, 3H).

^{13}C NMR (126 MHz, DMSO-d$_6$) δ (ppm) = 145.75, 143.54, 141.72, 139.06, 137.80, 135.17, 132.99, 129.89, 129.20, 128.29, 127.94, 127.17, 127.07, 126.27, 116.91, 51.61, 48.30, 21.03.

IR (ATR): $\tilde{\nu}$ (cm^{-1}) = 3206, 3006, 1596, 1495, 1448, 1376, 1324, 1307, 1276, 1261, 1161, 1077.

HRMS (ESI$^+$) calcd. for $C_{24}H_{26}N_3O_4S_2^+$ [M+H]$^+$: 484.1359, found: 484.1373.

1.7. General Procedure B for Cyclization Precursor Synthesis

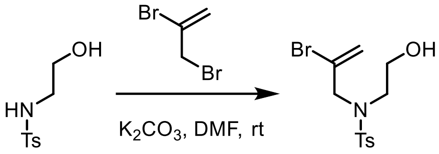

S6a'

An oven-dried round-bottom flask containing *N*-tosyl aminoethanol (3.22 g, 15.0 mmol, 1.00 equiv.) was sealed and subjected to three N_2 evacuation/refill cycles before anhydrous DMF (60.0 mL, 0.25 M) was added. The 2,3-dibromopropene (80% purity, 5.63 g, 22.5 mmol, 1.50 equiv.) was added to the solution, followed by the addition of K_2CO_3 (6.21 g, 45.0 mmol, 3.00 equiv.). The reaction mixture was then stirred at room temperature overnight until completion (judged by TLC). The reaction was quenched with H_2O (600 mL) and extracted with ethyl acetate (3 × 100 mL). The combined organic layers were washed with brine, dried over anhydrous $MgSO_4$, filtered and concentrated under reduced pressure. The crude product was purified by flash column chromatography (pentane/ethyl acetate, 5:1 to 1:1) to afford **S6a'** as a white solid (3.61 g, 10.8 mmol, 72%).

***N*-(2-bromoallyl)-*N*-(2-hydroxyethyl)-4-methylbenzenesulfonamide (S6a')**

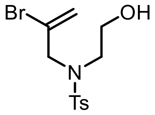

M.P.: 66-68 °C

^{1}H NMR (500 MHz, $CDCl_3$) δ (ppm) = 7.67 (d, *J* = 8.1 Hz, 2H), 7.27 (d, *J* = 8.0 Hz, 2H), 5.82 (d, *J* = 2.3 Hz, 1H), 5.55 (d, *J* = 2.3 Hz, 1H), 4.06 (s, 2H), 3.69 (app. q, *J* = 5.6 Hz, 2H), 3.26 (t, *J* = 5.6 Hz, 2H), 2.66 (t, *J* = 5.8 Hz, 1H), 2.38 (s, 3H).

^{13}C NMR (126 MHz, $CDCl_3$) δ (ppm) = 143.86, 136.00, 129.78, 128.23, 127.31, 119.87, 60.74, 56.74, 50.29, 21.51.

IR (ATR): $\tilde{\nu}$ (cm^{-1}) = 3487, 2926, 1629, 1597, 1444, 1331, 1276, 1154, 1088, 1020.

HRMS (ESI^+) calcd. for $C_{12}H_{17}BrNO_3S^+$ $[M+H]^+$: 334.0107, found: 334.0098.

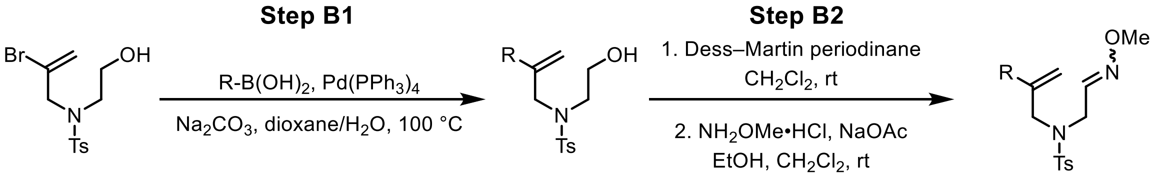

Step B1: S6a' (1.00 equiv.), aryl/alkene boric acid (1.20 equiv.), Na_2CO_3 (3.00 equiv.), and $Pd(PPh_3)_4$ (3 mol%) were dissolved in degassed 1,4-dioxane/H_2O (v = 9:1, 0.2 M) in an oven-dried round-bottom

flask and under nitrogen atmosphere. The solution was degassed by bubbling with a steam of nitrogen gas for 5 min and then the reaction was stirred at 100 °C for 24 h. The crude product was dried over anhydrous $MgSO_4$, filtered through anhydrous $MgSO_4$ (washed by CH_2Cl_2) and concentrated under reduced pressure. The crude product was purified by flash column chromatography.

Note: Owing to the presence of impurities, the crude alcohol from **Step B1** sometimes requires sequential flash column chromatography with two different solvent systems (e.g., pentane/ethyl acetate and CH_2Cl_2/ethyl acetate) for effective purification.

Step B2: The alcohol (1.00 equiv.) was dissolved in CH_2Cl_2 (0.5 M) in an oven-dried round-bottom flask. Dess-Martin periodinane (1.50 equiv.) was then added and the reaction mixture was stirred in an open flask at room temperature for 0.5-1 h until completion (judged by TLC). The reaction was quenched with sat. aq. $NaHCO_3$ solution and extracted with CH_2Cl_2. The combined organic layers were dried over anhydrous $MgSO_4$, filtered, and concentrated under reduced pressure. The crude aldehyde was then dissolved in ethanol (0.1 M) and CH_2Cl_2 (1.0 M) in an oven-dried round-bottom flask before sodium acetate (4.00 equiv.) and methoxyamine hydrochloride (2.00 equiv.) were added. The reaction was stirred at room temperature until completion (judged by TLC, typically within 1 h). The reaction was quenched with sat. aq. $NaHCO_3$ solution and extracted with CH_2Cl_2. The combined organic layers were dried over anhydrous $MgSO_4$, filtered, concentrated under reduced pressure and purified by flash column chromatography.

Notes: In the final step (amination), the purpose of adding CH_2Cl_2 (1.0 M) is to wash the aldehyde stuck to the wall of the round-bottom flask into the reaction solution.

***N*-(2-(4-fluorophenyl)allyl)-*N*-(2-hydroxyethyl)-4-methylbenzenesulfonamide (S6a)**

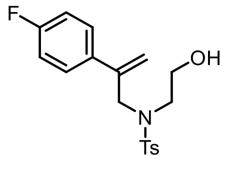

Prepared according to **General Procedure B (step B1)** using **S6a'** (2.66 g, 8.00 mmol, 1.00 equiv.), 4-fluorophenylboronic acid (1.34 g, 9.60 mmol, 1.20 equiv.), Na_2CO_3 (2.54 g, 24.0 mmol, 3.00 equiv.), $Pd(PPh_3)_4$ (277 mg, 0.24 mmol, 3 mol%), 1,4-dioxane/H_2O (36 mL/4 mL). Purification by flash column chromatography (pentane/ethyl acetate, 5:1 to 3:1 to 1:1 and CH_2Cl_2/ethyl acetate, 10:1 to 4:1) afforded alcohol **S6a** as a white solid (1.28 g, 3.67 mmol, 46%).

M.P.: 76-78 °C

^{1}**H NMR** (500 MHz, $CDCl_3$) δ (ppm) = 7.67 (d, J = 8.3 Hz, 2H), 7.47-7.42 (m, 2H), 7.31 (d, J = 7.8 Hz, 2H), 7.05-7.00 (m, 2H), 5.45 (s, 1H), 5.21 (s, 1H), 4.21 (s, 2H), 3.55 (app. q, J = 5.6 Hz, 2H), 3.15 (t, J = 5.4 Hz, 2H), 2.44 (s, 3H), 2.01 (t, J = 6.1 Hz, 1H).

^{13}**C NMR** (126 MHz, $CDCl_3$) δ (ppm) = 162.64 (d, J = 247.5 Hz), 143.84, 141.89, 135.17, 133.87 (d, J = 3.4 Hz), 129.83, 128.24 (d, J = 8.1 Hz), 127.41, 116.85, 115.40 (d, J = 21.4 Hz), 60.88, 53.79, 49.94, 21.50.

^{19}**F NMR** (471 MHz, $CDCl_3$) δ (ppm) = −113.54 (tt, J = 8.6, 5.3 Hz).

IR (ATR): $\tilde{\nu}$ (cm^{-1}) = 3496, 2988, 1601, 1510, 1451, 1332, 1276, 1261, 1229, 1157, 1088, 1012.

HRMS (ESI$^+$) calcd. for $C_{18}H_{21}FNO_3S^+$ [M+H]$^+$: 350.1221, found: 350.1208.

***N*-(2-(4-fluorophenyl)allyl)-*N*-(2-(methoxyimino)ethyl)-4-methylbenzenesulfonamide (S6b)**

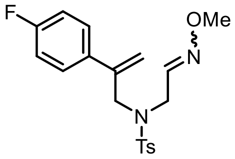

Prepared according to **General Procedure B (step B2)** using alcohol **S6a** (1.10 g, 3.15 mmol, 1.00 equiv.), CH_2Cl_2 (6.3 mL), Dess-Martin periodinane (2.01 g, 4.73 mmol, 1.50 equiv.), $NH_2OMe{\cdot}HCl$ (529 mg, 6.30 mmol, 2.00 equiv.), NaOAc (1.03 g, 12.6 mmol, 4.00 equiv.), EtOH (31.5 mL), CH_2Cl_2 (3.2 mL). Purification by flash column chromatography (pentane/ethyl acetate, 8:1 to 3:1) afforded oxime **S6b** as a colorless oil (977 mg, 2.60 mmol, 82%, E/Z = 70:30).

^{1}**H NMR** (500 MHz, $CDCl_3$) δ (ppm) = 7.68-7.62 (m, 2H), 7.47-7.43 (m, 0.6H), 7.41-7.37 (m, 1.4H), 7.31 (d, J = 7.8 Hz, 0.6H), 7.29 (d, J = 7.9 Hz, 1.4H), 7.04-6.98 (m, 2.7H), 6.30 (t, J = 4.1 Hz, 0.3H), 5.44 (s, 0.3H), 5.43 (s, 0.7H), 5.23 (s, 0.7H), 5.19 (s, 0.3H), 4.19 (s, 1.4H), 4.17 (s, 0.6H), 3.88 (d, J = 4.1 Hz, 0.6H), 3.80 (s, 0.9H), 3.78 (d, J = 5.9 Hz, 1.4H), 3.73 (s, 2.1H), 2.43 (s, 0.9H), 2.42 (s, 2.1H).

^{13}**C NMR** (126 MHz, $CDCl_3$) δ (ppm) =162.79 (d, J = 247.6 Hz), 162.72 (d, J = 247.1 Hz), 147.79, 145.18, 144.03, 143.88, 141.38, 141.20, 136.02, 135.33, 134.19 (d, J = 3.2 Hz), 133.73 (d, J = 3.2 Hz), 130.01, 129.89, 128.42 (d, J = 8.3 Hz), 128.40 (d, J = 8.3 Hz), 127.45, 127.44, 117.46, 117.19, 115.50 (d, J = 21.3 Hz), 115.35 (d, J = 21.3 Hz), 62.10, 61.74, 53.43, 51.76, 45.65, 42.80, 21.60, 21.58.

^{19}**F NMR** (471 MHz, $CDCl_3$) δ (ppm) = −113.62 (tt, J = 8.8, 5.3 Hz), −113.91 (tt, J = 8.6, 5.3 Hz).

IR (ATR): $\tilde{\nu}$ (cm^{-1}) = 2938, 2902, 1631, 1601, 1510, 1445, 1338, 1230, 1158, 1091, 1030.

HRMS (ESI$^+$) calcd. for $C_{19}H_{22}FN_2O_3S^+$ [M+H]$^+$: 377.1330, found: 377.1314.

***N*-(2-(4-chlorophenyl)allyl)-*N*-(2-hydroxyethyl)-4-methylbenzenesulfonamide (S7a)**

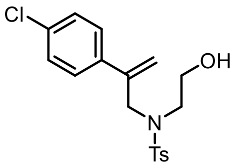

Prepared according to **General Procedure B (step B1)** using **S6a'** (2.66 g, 8.00 mmol, 1.00 equiv.), 4-chlorophenylboronic acid (1.50 g, 9.60 mmol, 1.20 equiv.), Na_2CO_3 (2.54 g, 24.0 mmol, 3.00 equiv.), $Pd(PPh_3)_4$ (277 mg, 0.24 mmol, 3 mol%), 1,4-dioxane/H_2O (36 mL/4 mL). Purification by flash column chromatography (pentane/ethyl acetate, 5:1 to 3:1 to 1:1 and CH_2Cl_2/ethyl acetate, 10:1 to 4:1) afforded alcohol **S7a** as a white solid (1.19 g, 3.26 mmol, 41%).

M.P.: 96-98 °C

^{1}H NMR (500 MHz, $CDCl_3$) δ (ppm) = 7.67 (d, *J* = 8.3 Hz, 2H), 7.40 (d, *J* = 8.6 Hz, 2H), 7.33-7.28 (m, 4H), 5.49 (s, 1H), 5.24 (s, 1H), 4.21 (s, 2H), 3.56 (app. q, *J* = 5.6 Hz, 2H), 3.15 (t, *J* = 5.4 Hz, 2H), 2.45 (s, 3H), 1.97 (t, *J* = 6.1 Hz, 1H).

^{13}C NMR (126 MHz, $CDCl_3$) δ (ppm) = 143.94, 142.01, 136.29, 135.22, 134.18, 129.91, 128.78, 127.91, 127.50, 117.53, 61.03, 53.77, 50.06, 21.62.

IR (ATR): $\tilde{\nu}$ (cm^{-1}) = 3500, 3006, 1597, 1494, 1452, 1334, 1276, 1261, 1158, 1089, 1012.

HRMS (ESI^+) calcd. for $C_{18}H_{21}ClNO_3S^+$ $[M+H]^+$: 366.0925, found: 366.0931.

***N*-(2-(4-chlorophenyl)allyl)-*N*-(2-(methoxyimino)ethyl)-4-methylbenzenesulfonamide (S7b)**

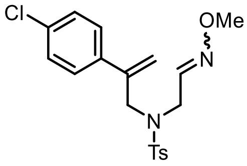

Prepared according to **General Procedure B (step B2)** using alcohol **S7a** (970 mg, 2.66 mmol, 1.00 equiv.), CH_2Cl_2 (5.3 mL), Dess-Martin periodinane (1.69 g, 3.99 mmol, 1.50 equiv.), $NH_2OMe \cdot HCl$ (447 mg, 5.32 mmol, 2.00 equiv.), NaOAc (872 mg, 10.64 mmol, 4.00 equiv.), EtOH (26.6 mL), CH_2Cl_2 (2.7 mL). Purification by flash column chromatography (pentane/ethyl acetate, 8:1 to 3:1) afforded oxime **S7b** as a colorless oil (880 mg, 2.24 mmol, 84%, *E/Z* = 70:30).

^{1}H NMR (500 MHz, $CDCl_3$) δ (ppm) = 7.69-7.63 (m, 2H), 7.42 (d, *J* = 8.6 Hz, 0.6H), 7.36 (d, *J* = 8.6 Hz, 1.4H), 7.33-7.27 (m, 4H), 7.02 (t, *J* = 5.9 Hz, 0.7H), 6.32 (t, *J* = 4.1 Hz, 0.3H), 5.49 (s, 0.3H), 5.48 (s, 0.7H), 5.28 (s, 0.7H), 5.24 (s, 0.3H), 4.21 (s, 1.4H), 4.19 (s, 0.6H), 3.90 (d, *J* = 4.1 Hz, 0.6H), 3.82 (s, 0.9H), 3.80 (d, *J* = 5.9 Hz, 1.4H), 3.75 (s, 2.1H), 2.44 (s, 3H).

^{13}**C NMR** (126 MHz, $CDCl_3$) δ (ppm) = 147.68, 145.07, 144.02, 143.87, 141.29, 141.11, 136.55, 136.09, 135.95, 135.25, 134.13, 133.96, 129.97, 129.85, 128.70, 128.57, 127.99, 127.94, 127.37, 127.36, 118.03, 117.76, 62.06, 61.70, 53.24, 51.56, 45.61, 42.76, 21.57, 21.55.

IR (ATR): $\tilde{\nu}$ (cm^{-1}) = 3004, 2988, 2937, 2901, 1630, 1597, 1493, 1449, 1338, 1276, 1261, 1158, 1090, 1030.

HRMS (ESI^+) calcd. for $C_{19}H_{22}ClN_2O_3S^+$ $[M+H]^+$: 393.1034, found: 393.1021.

***N*-(2-(3-chlorophenyl)allyl)-*N*-(2-hydroxyethyl)-4-methylbenzenesulfonamide (S8a)**

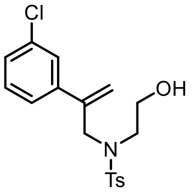

Prepared according to **General Procedure B (step B1)** using **S6a'** (2.66 g, 8.00 mmol, 1.00 equiv.), 3-chlorophenylboronic acid (1.50 g, 9.60 mmol, 1.20 equiv.), Na_2CO_3 (2.54 g, 24.0 mmol, 3.00 equiv.), $Pd(PPh_3)_4$ (277 mg, 0.24 mmol, 3 mol%), 1,4-dioxane/H_2O (36 mL/4 mL). Purification by flash column chromatography (pentane/ethyl acetate, 5:1 to 3:1 to 1:1 and CH_2Cl_2/ethyl acetate, 10:1 to 4:1) afforded alcohol **S8a** as a pale-yellow oil (1.29 g, 3.53 mmol, 44%).

^{1}**H NMR** (500 MHz, $CDCl_3$) δ (ppm) = 7.68 (d, *J* = 8.3 Hz, 2H), 7.40-7.27 (m, 6H), 5.51 (s, 1H), 5.30 (s, 1H), 4.23 (s, 2H), 3.60 (app. q, *J* = 5.6 Hz, 2H), 3.19 (t, *J* = 5.5 Hz, 2H), 2.45 (s, 3H), 2.13 (t, *J* = 5.9 Hz, 1H).

^{13}**C NMR** (126 MHz, $CDCl_3$) δ (ppm) = 143.96, 142.06, 139.94, 135.41, 134.59, 129.94, 128.38, 127.57, 126.71, 124.80, 118.03, 61.07, 53.49, 50.17, 21.67.

IR (ATR): $\tilde{\nu}$ (cm^{-1}) = 3006, 2989, 1595, 1460, 1335, 1276, 1261, 1158.

HRMS (ESI^+) calcd. for $C_{18}H_{21}ClNO_3S^+$ $[M+H]^+$: 366.0925, found: 366.0932.

***N*-(2-(3-chlorophenyl)allyl)-*N*-(2-(methoxyimino)ethyl)-4-methylbenzenesulfonamide (S8b)**

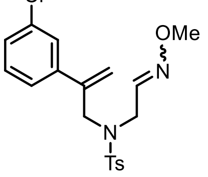

Prepared according to **General Procedure B (step B2)** using alcohol **S8a** (1.10 g, 3.00 mmol, 1.00 equiv.), CH_2Cl_2 (6.0 mL), Dess-Martin periodinane (1.91 g, 4.50 mmol, 1.50 equiv.),

$NH_2OMe{\cdot}HCl$ (504 mg, 6.00 mmol, 2.00 equiv.), NaOAc (984 mg, 12.00 mmol, 4.00 equiv.), EtOH (30.0 mL), CH_2Cl_2 (3.0 mL). Purification by flash column chromatography (pentane/ethyl acetate, 8:1 to 3:1) afforded oxime **S8b** as a colorless oil (980 mg, 2.50 mmol, 83%, *E/Z* = 70:30).

^{1}H NMR (500 MHz, $CDCl_3$) δ (ppm) = 7.60-7.64 (m, 2H), 7.42-7.25 (m, 6H), 7.06 (t, *J* = 5.9 Hz, 0.7H), 6.38 (t, *J* = 4.2 Hz, 0.3H), 5.51 (s, 0.3H), 5.50 (s, 0.7H), 5.32 (s, 0.7H), 5.28 (s, 0.3H), 4.21 (s, 1.4H), 4.19 (s, 0.6H), 3.93 (d, *J* = 4.2 Hz, 0.6H), 3.84-3.81 (m, 2.3H), 3.77 (s, 2.1H), 2.43 (s, 3H).

^{13}C NMR (126 MHz, $CDCl_3$) δ (ppm) = 147.54, 145.04, 144.01, 143.87, 141.32, 141.09, 140.17, 139.69, 135.98, 135.36, 134.44, 134.31, 129.97, 129.87, 129.84, 129.71, 128.27, 128.10, 127.38, 126.80, 126.65, 124.85, 124.80, 118.51, 118.20, 62.08, 61.74, 52.94, 51.31, 45.70, 42.76, 21.57, 21.56.

IR (ATR): $\tilde{\nu}$ (cm^{-1}) = 3005, 2989, 1595, 1563, 1444, 1338, 1276, 1261, 1158, 1090, 1029.

HRMS (ESI$^+$) calcd. for $C_{19}H_{22}ClN_2O_3S^+$ [M+H]$^+$: 393.1034, found: 393.1021.

***N*-(2-(4-cyanophenyl)allyl)-*N*-(2-hydroxyethyl)-4-methylbenzenesulfonamide (S9a)**

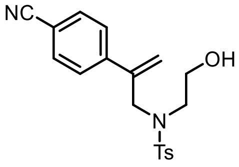

Prepared according to **General Procedure B (step B1)** using **S6a'** (2.66 g, 8.00 mmol, 1.00 equiv.), 4-cyanophenylboronic acid (1.41 g, 9.60 mmol, 1.20 equiv.), Na_2CO_3 (2.54 g, 24.0 mmol, 3.00 equiv.), $Pd(PPh_3)_4$ (277 mg, 0.24 mmol, 3 mol%), 1,4-dioxane/H_2O (36 mL/4 mL). Purification by flash column chromatography (pentane/ethyl acetate, 3:1 to 1:1 to 1:3 and CH_2Cl_2/ethyl acetate, 7:1 to 2:1) afforded alcohol **S9a** as a pale-yellow solid (1.52 g, 4.27 mmol, 53%).

M.P.: 70-72°C

^{1}H NMR (500 MHz, $CDCl_3$) δ (ppm) = 7.67 (d, *J* = 8.3 Hz, 2H), 7.62 (d, *J* = 8.7 Hz, 2H), 7.58 (d, *J* = 8.5 Hz, 2H), 7.32 (d, *J* = 8.2 Hz, 2H), 5.60 (s, 1H), 5.39 (s, 1H), 4.24 (s, 2H), 3.54 (app. q, *J* = 5.7 Hz, 2H), 3.15 (t, *J* = 5.6 Hz, 2H), 2.44 (s, 3H), 2.05 (t, *J* = 6.0 Hz, 1H).

^{13}C NMR (126 MHz, $CDCl_3$) δ (ppm) = 144.14, 142.43, 141.86, 135.13, 132.44, 130.01, 127.52, 127.30, 119.83, 118.76, 111.86, 61.09, 53.66, 50.15, 21.66.

IR (ATR): $\tilde{\nu}$ (cm^{-1}) = 3515, 3006, 2228, 1724, 1604, 1452, 1404, 1335, 1276, 1261, 1158, 1089, 1017.

HRMS (ESI$^+$) calcd. for $C_{19}H_{21}N_2O_3S^+$ [M+H]$^+$: 357.1267, found: 357.1255.

***N*-(2-(4-cyanophenyl)allyl)-*N*-(2-(methoxyimino)ethyl)-4-methylbenzenesulfonamide (S9b)**

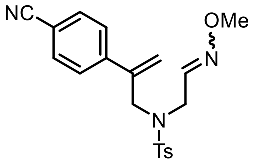

Prepared according to **General Procedure B (step B2)** using alcohol **S9a** (1.30 g, 3.65 mmol, 1.00 equiv.), CH_2Cl_2 (7.3 mL), Dess-Martin periodinane (2.32 g, 5.48 mmol, 1.50 equiv.), $NH_2OMe \cdot HCl$ (613 mg, 7.30 mmol, 2.00 equiv.), NaOAc (1.20 g, 14.63 mmol, 4.00 equiv.), EtOH (36.5 mL), CH_2Cl_2 (3.7 mL). Purification by flash column chromatography (pentane/ethyl acetate, 5:1 to 1:1) afforded oxime **S9b** as a colorless oil (1.13 g, 2.95 mmol, 81%, *E/Z* = 70:30).

^{1}H NMR (500 MHz, $CDCl_3$) δ (ppm) = 7.65-7.56 (m, 4.7H), 7.53-7.50 (m, 1.3H), 7.31 (d, *J* = 7.8 Hz, 0.6H), 7.29 (d, *J* = 7.8 Hz, 1.4H), 6.93 (t, *J* = 5.9 Hz, 0.7H), 6.24 (t, *J* = 4.2 Hz, 0.3H), 5.59 (s, 0.3H), 5.57 (s, 0.7H), 5.38 (s, 0.7H), 5.35 (s, 0.3H), 4.20 (s, 1.4H), 4.18 (s, 0.6H), 3.86 (d, *J* = 4.2 Hz, 0.6H), 3.77 (s, 0.9H), 3.75 (d, *J* = 5.8 Hz, 1.4H), 3.70 (s, 2.1H), 2.41 (s, 3H).

^{13}C NMR (126 MHz, $CDCl_3$) δ (ppm) = 147.22, 144.75, 144.20, 144.05, 142.57, 142.12, 141.06, 140.83, 135.71, 135.02, 132.30, 132.19, 130.03, 129.92, 127.35, 127.32, 127.27, 120.37, 120.07, 118.72, 118.66, 111.74, 111.59, 62.08, 61.71, 52.99, 51.36, 45.71, 42.75, 21.55, 21.54.

IR (ATR): $\tilde{\nu}$ (cm^{-1}) = 3005, 2989, 2227, 1606, 1598, 1449, 1405, 1338, 1276, 1261, 1159, 1091.

HRMS (ESI^+) calcd. for $C_{20}H_{22}N_3O_3S^+$ $[M+H]^+$: 384.1376, found: 384.1359.

***N*-(2-hydroxyethyl)-4-methyl-*N*-(2-(4-(trifluoromethyl)phenyl)allyl)benzenesulfonamide (S10a)**

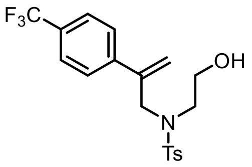

Prepared according to **General Procedure B (step B1)** using **S6a'** (2.66 g, 8.00 mmol, 1.00 equiv.), 4-(trifluoromethyl)phenylboronic acid (1.82 g, 9.60 mmol, 1.20 equiv.), Na_2CO_3 (2.54 g, 24.0 mmol, 3.00 equiv.), $Pd(PPh_3)_4$ (277 mg, 0.24 mmol, 3 mol%), 1,4-dioxane/H_2O (36 mL/4 mL). Purification by flash column chromatography (pentane/ethyl acetate, 5:1 to 3:1 to 1:1 and CH_2Cl_2/ethyl acetate, 10:1 to 4:1) afforded alcohol **S10a** as a white solid (1.46 g, 3.66 mmol, 46%).

M.P.: 94-96 °C

^{1}H NMR (500 MHz, $CDCl_3$) δ (ppm) = 7.68 (d, *J* = 8.3 Hz, 2H), 7.62 (d, *J* = 8.5 Hz, 2H), 7.59 (d, *J* = 8.5 Hz, 2H), 7.33 (d, *J* = 8.0 Hz, 2H), 5.59 (s, 1H), 5.38 (s, 1H), 4.29 (s, 2H), 3.61 (app. q, *J* = 5.6 Hz, 2H), 3.20 (t, *J* = 5.4 Hz, 2H), 2.47 (s, 3H), 1.97 (t, *J* = 6.0 Hz, 1H).

^{13}C NMR (126 MHz, $CDCl_3$) δ (ppm) = 144.02, 142.15, 141.56, 135.28, 130.15 (q, *J* = 32.3 Hz), 129.93, 127.49, 126.95, 125.55 (q, *J* = 3.7 Hz), 124.15 (q, *J* = 272.1 Hz), 119.01, 61.01, 53.61, 50.03, 21.57.

^{19}F NMR (471 MHz, $CDCl_3$) δ (ppm) = −62.62 (s).

IR (ATR): $\tilde{\nu}$ (cm^{-1}) = 3512, 3006, 2989, 1617, 1598, 1454, 1407, 1326, 1275, 1261, 1160, 1120, 1067.

HRMS (ESI$^+$) calcd. for $C_{19}H_{21}F_3NO_3S^+$ [M+H]$^+$: 400.1189, found: 400.1182.

***N*-(2-(methoxyimino)ethyl)-4-methyl-*N*-(2-(4-(trifluoromethyl)phenyl)allyl) benzene-sulfonamide (S10b)**

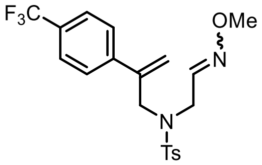

Prepared according to **General Procedure B (step B2)** using alcohol **S10a** (1.10 g, 2.76 mmol, 1.00 equiv.), CH_2Cl_2 (5.5 mL), Dess-Martin periodinane (1.76 g, 4.14 mmol, 1.50 equiv.), $NH_2OMe\cdot HCl$ (464 mg, 5.52 mmol, 2.00 equiv.), NaOAc (905 mg, 11.04 mmol, 4.00 equiv.), EtOH (27.6 mL), CH_2Cl_2 (2.8 mL). Purification by flash column chromatography (pentane/ethyl acetate, 8:1 to 3:1) afforded oxime **S10b** as a colorless oil (1.04 g, 2.44 mmol, 88%, *E/Z* = 70:30).

^{1}H NMR (500 MHz, $CDCl_3$) δ (ppm) = 7.65-7.60 (m, 2H), 7.58-7.50 (m, 4H), 7.28 (d, *J* = 7.9 Hz, 0.6H), 7.26 (d, *J* = 7.8 Hz, 1.4H), 7.00 (t, *J* = 5.9 Hz, 0.7H), 6.30 (t, *J* = 4.2 Hz, 0.3H), 5.55 (s, 0.3H), 5.54 (s, 0.7H), 5.36 (s, 0.7H), 5.32 (s, 0.3H), 4.24 (s, 1.4H), 4.21 (s, 0.6H), 3.90 (d, *J* = 4.2 Hz, 0.6H), 3.80-3.77 (m, 2.3H), 3.72 (s, 2.1H), 2.39 (s, 0.9H), 2.39 (s, 2.1H).

^{13}C NMR (126 MHz, $CDCl_3$) δ (ppm) = 147.45, 144.87, 144.08, 143.92, 141.77, 141.38, 141.30, 141.17, 135.89, 135.18, 130.24 (q, *J* = 32.8 Hz), 129.95, 129.86 (q, *J* = 32.3 Hz), 129.83, 127.31, 127.01, 126.94, 125.41 (q, *J* = 3.9 Hz), 125.27 (q, *J* = 3.7 Hz), 124.16 (q, *J* = 271.7 Hz), 124.15 (q, *J* = 272.2 Hz), 119.51, 119.21, 61.98, 61.62, 53.12, 51.46, 45.62, 42.73, 21.40, 21.38.

^{19}F NMR (471 MHz, $CDCl_3$) δ (ppm) = -62.51 (s), −62.53 (s).

IR (ATR): $\tilde{\nu}$ (cm^{-1}) = 2937, 1617, 1598, 1407, 1327, 1162, 1120, 1067, 1033.

HRMS (ESI$^+$) calcd. for $C_{20}H_{22}F_3N_2O_3S^+$ [M+H]$^+$: 427.1298, found: 427.1279.

Ethyl 4-(3-((*N*-(2-hydroxyethyl)-4-methylphenyl)sulfonamido)prop-1-en-2-yl)benzoate (S11a)

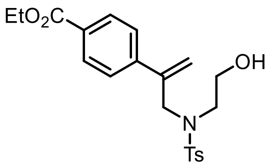

Prepared according to **General Procedure B (step B1)** using **S6a'** (2.66 g, 8.00 mmol, 1.00 equiv.), 4-Ethoxycarbonylphenylboronic acid (1.86 g, 9.60 mmol, 1.20 equiv.), Na_2CO_3 (2.54 g, 24.0 mmol, 3.00 equiv.), $Pd(PPh_3)_4$ (277 mg, 0.24 mmol, 3 mol%), 1,4-dioxane/H_2O (36 mL/4 mL). Purification by flash column chromatography (pentane/ethyl acetate, 5:1 to 3:1 to 1:1 and CH_2Cl_2/ethyl acetate, 10:1 to 4:1) afforded alcohol **S11a** as a pale-yellow oil (1.71 g, 4.24 mmol, 53%).

^{1}H NMR (500 MHz, $CDCl_3$) δ (ppm) = 7.98 (d, *J* = 8.4 Hz, 2H), 7.65 (d, *J* = 8.3 Hz, 2H), 7.49 (d, *J* = 8.4 Hz, 2H), 7.28 (d, *J* = 8.2 Hz, 2H), 5.56 (s, 1H), 5.32 (s, 1H), 4.36 (q, *J* = 7.1 Hz, 2H), 4.25 (s, 2H), 3.54 (app. q, *J* = 5.6 Hz, 2H), 3.15 (t, *J* = 5.6 Hz, 2H), 2.42 (s, 3H), 2.18 (t, *J* = 5.9 Hz, 1H), 1.38 (t, *J* = 7.1 Hz, 3H).

^{13}C NMR (126 MHz, $CDCl_3$) δ (ppm) = 166.34, 143.91, 142.43, 142.31, 135.31, 130.12, 129.90, 129.86, 127.51, 126.50, 118.67, 61.10, 61.03, 53.61, 50.09, 21.60, 14.41.

IR (ATR): $\tilde{\nu}$ (cm^{-1}) = 3004, 2986, 1712, 1608, 1449, 1405, 1335, 1276, 1158, 1109, 1019.

HRMS (ESI$^+$) calcd. for $C_{21}H_{26}NO_5S^+$ [M+H]$^+$: 404.1526, found: 404.1511.

Ethyl 4-(3-((*N*-(2-(methoxyimino)ethyl)-4-methylphenyl)sulfonamido)prop-1-en-2-yl)benzoate (S11b)

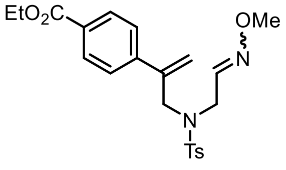

Prepared according to **General Procedure B (step B2)** using alcohol **S11a** (1.40 g, 3.47 mmol, 1.00 equiv.), CH_2Cl_2 (7.0 mL), Dess-Martin periodinane (2.21 g, 5.21 mmol, 1.50 equiv.), $NH_2OMe·HCl$ (583 mg, 6.94 mmol, 2.00 equiv.), NaOAc (1.14 g, 13.88 mmol, 4.00 equiv.), EtOH (34.7 mL), CH_2Cl_2 (3.5 mL). Purification by flash column chromatography (pentane/ethyl acetate, 8:1 to 3:1) afforded oxime **S11b** as a colorless oil (1.12 g, 2.60 mmol, 75%, *E*/*Z* = 70:30).

^{1}H NMR (500 MHz, $CDCl_3$) δ (ppm) = 7.99 (d, *J* = 8.5 Hz, 0.6H), 7.97 (d, *J* = 8.4 Hz, 1.4H), 7.65-7.60 (m, 2H), 7.50 (d, *J* = 8.4 Hz, 0.6H), 7.44 (d, *J* = 8.4 Hz, 1.4H), 7.28 (d, *J* = 8.1 Hz, 0.6H), 7.26

(d, J = 8.1 Hz, 1.4H), 6.99 (t, J = 5.9 Hz, 0.7H), 6.28 (t, J = 4.1 Hz, 0.3H), 5.56 (s, 0.3H), 5.55 (s, 0.7H), 5.34 (s, 0.7H), 5.30 (s, 0.3H), 4.41-4.32 (m, 2H), 4.22 (s, 1.4H), 4.20 (s, 0.6H), 3.88 (d, J = 4.1 Hz, 0.6H), 3.79-3.76 (m, 2.3H), 3.72 (s, 2.1H), 2.40 (s, 0.9H), 2.40 (s, 2.1H), 1.38 (t, J = 7.2 Hz, 3H).

^{13}C NMR (126 MHz, $CDCl_3$) δ (ppm) = 166.28, 166.24, 147.60, 145.04, 144.01, 143.86, 142.57, 142.07, 141.74, 141.56, 135.97, 135.30, 130.12, 129.98, 129.96, 129.85, 129.79, 129.67, 127.38, 126.56, 126.52, 119.18, 118.84, 62.06, 61.71, 61.01, 60.99, 53.10, 51.42, 45.69, 42.76, 21.55, 21.53, 14.36.

IR (ATR): $\tilde{\nu}$ (cm^{-1}) = 3003, 2985, 2938, 1712, 1609, 1448, 1405, 1275, 1159, 1102, 1029.

HRMS (ESI$^+$) calcd. for $C_{22}H_{27}N_2O_5S^+$ [M+H]$^+$: 431.1635, found: 431.1621.

***N*-(2-hydroxyethyl)-4-methyl-*N*-(2-(*p*-tolyl)allyl)benzenesulfonamide (S12a)**

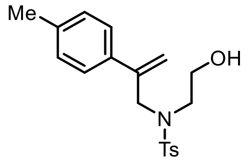

Prepared according to **General Procedure B (step B1)** using **S6a'** (2.66 g, 8.00 mmol, 1.00 equiv.), *p*-tolylboronic acid (1.31 g, 9.60 mmol, 1.20 equiv.), Na_2CO_3 (2.54 g, 24.0 mmol, 3.00 equiv.), $Pd(PPh_3)_4$ (277 mg, 0.24 mmol, 3 mol%), 1,4-dioxane/H_2O (36 mL/4 mL). Purification by flash column chromatography (pentane/ethyl acetate, 5:1 to 3:1 to 1:1 and CH_2Cl_2/ethyl acetate, 10:1 to 4:1) afforded alcohol **S12a** as a white solid (1.21 g, 3.51 mmol, 44%).

M.P.: 104-106 °C

^{1}H NMR (500 MHz, $CDCl_3$) δ (ppm) = 7.68 (d, J = 8.3 Hz, 2H), 7.36 (d, J = 8.2 Hz, 2H), 7.31 (d, J = 8.0 Hz, 2H), 7.15 (d, J = 7.9 Hz, 2H), 5.46 (s, 1H), 5.17 (d, J = 1.2 Hz, 1H), 4.22 (s, 2H), 3.55 (app. q, J = 5.6 Hz, 2H), 3.15 (t, J = 5.4 Hz, 2H), 2.44 (s, 3H), 2.35 (s, 3H), 2.03 (t, J = 6.1 Hz, 1H).

^{13}C NMR (126 MHz, $CDCl_3$) δ (ppm) = 143.73, 142.81, 138.15, 135.28, 134.89, 129.82, 129.31, 127.54, 126.39, 116.03, 61.01, 53.82, 50.14, 21.58, 21.21.

IR (ATR): $\tilde{\nu}$ (cm^{-1}) = 3515, 3006, 2989, 1598, 1515, 1454, 1335, 12761261, 1159, 1088, 1019.

HRMS (ESI$^+$) calcd. for $C_{19}H_{24}NO_3S^+$ [M+H]$^+$: 346.1471, found: 346.1460.

***N*-(2-(methoxyimino)ethyl)-4-methyl-*N*-(2-(*p*-tolyl)allyl)benzenesulfonamide (S12b)**

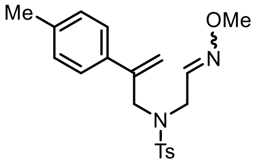

Prepared according to **General Procedure B (step B2)** using alcohol **S12a** (1.02 g, 2.96 mmol, 1.00 equiv.), CH_2Cl_2 (6.0 mL), Dess-Martin periodinane (1.88 g, 4.44 mmol, 1.50 equiv.), $NH_2OMe{\cdot}HCl$ (497 mg, 5.92 mmol, 2.00 equiv.), NaOAc (971 mg, 11.84 mmol, 4.00 equiv.), EtOH (29.6 mL), CH_2Cl_2 (3.0 mL). Purification by flash column chromatography (pentane/ethyl acetate, 8:1 to 3:1) afforded oxime **S12b** as a colorless oil (944 mg, 2.54 mmol, 86%, *E/Z* = 70:30).

^{1}H NMR (500 MHz, $CDCl_3$) δ (ppm) = 7.69-7.64 (m, 2H), 7.37 (d, *J* = 8.2 Hz, 0.6H), 7.33-7.27 (m, 3.4H), 7.16 (d, *J* = 7.8 Hz, 0.6H), 7.14 (d, *J* = 8.1 Hz, 1.4H), 7.03 (t, *J* = 5.8 Hz, 0.7H), 6.32 (t, *J* = 4.0 Hz, 0.3H), 5.46 (s, 0.3H), 5.45 (s, 0.7H), 5.20 (s, 0.7H), 5.16 (s, 0.3H), 4.22 (s, 1.4H), 4.19 (s, 0.6H), 3.90 (d, *J* = 4.0 Hz, 0.6H), 3.81-3.79 (m, 2.3H), 3.75 (s, 2.1H), 2.44 (s, 0.9H), 2.43 (s, 2.1H), 2.35 (s, 3H).

^{13}C NMR (126 MHz, $CDCl_3$) δ (ppm) = 148.11, 145.41, 143.88, 143.73, 142.16, 142.03, 138.21, 138.03, 136.19, 135.52, 135.33, 134.86, 129.96, 129.84, 129.32, 129.20, 127.54, 127.50, 126.54, 126.52, 116.61, 116.28, 62.10, 61.74, 53.37, 51.66, 45.66, 42.88, 21.64, 21.63, 21.26.

IR (ATR): $\tilde{\nu}$ (cm^{-1}) = 2934, 2819, 1628, 1598, 1515, 1446, 1341, 1160, 1091, 1032.

HRMS (ESI^+) calcd. for $C_{20}H_{25}N_2O_3S^+$ $[M+H]^+$: 373.1580, found: 373.1567.

***N*-(2-hydroxyethyl)-*N*-(2-(4-methoxyphenyl)allyl)-4-methylbenzenesulfonamide (S13a)**

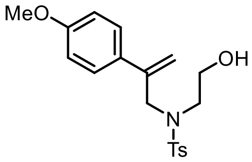

Prepared according to **General Procedure B (step B1)** using **S6a'** (2.00 g, 6.00 mmol, 1.00 equiv.), 4-methoxyphenylboronic acid (1.09 g, 7.20 mmol, 1.20 equiv.), Na_2CO_3 (1.91 g, 18.0 mmol, 3.00 equiv.), $Pd(PPh_3)_4$ (208 mg, 0.18 mmol, 3 mol%), 1,4-dioxane/H_2O (27 mL/3 mL). Purification by flash column chromatography (pentane/ethyl acetate, 5:1 to 3:1 to 1:1 and CH_2Cl_2/ethyl acetate, 10:1 to 4:1) afforded alcohol **S13a** as a white solid (1.20 g, 3.32 mmol, 55%).

M.P.: 85-87 °C

^{1}H NMR (500 MHz, $CDCl_3$) δ (ppm) = 7.68 (d, *J* = 8.3 Hz, 2H), 7.43 (d, *J* = 8.8 Hz, 2H), 7.30 (d, *J* = 7.8 Hz, 2H), 6.87 (d, *J* = 8.8 Hz, 2H), 5.41 (d, *J* = 0.9 Hz, 1H), 5.11 (d, *J* = 1.2 Hz, 1H), 4.19 (d, *J* =

1.1 Hz, 2H), 3.81 (s, 3H), 3.53 (app. q, J = 5.7 Hz, 2H), 3.13 (t, J = 5.5 Hz, 2H), 2.43 (s, 3H), 2.12 (t, J = 6.1 Hz, 1H).

^{13}C NMR (126 MHz, $CDCl_3$) δ (ppm) = 159.73, 143.80, 142.27, 135.20, 130.08, 129.87, 127.77, 127.59, 115.30, 114.02, 61.12, 55.35, 54.08, 50.18, 21.61.

IR (ATR): $\tilde{\nu}$ (cm^{-1}) = 3516, 3006, 1608, 1518, 1464, 1330, 1276, 1260, 1188, 1155, 1105, 1071, 1033.

HRMS (ESI^+) calcd. for $C_{19}H_{24}NO_4S^+$ $[M+H]^+$: 362.1421, found: 362.1421.

***N*-(2-(methoxyimino)ethyl)-*N*-(2-(4-methoxyphenyl)allyl)-4-methylbenzenesulfonamide (S13b)**

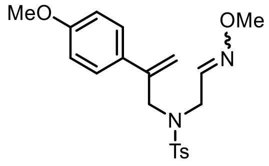

Prepared according to **General Procedure B (step B2)** using alcohol **S13a** (950 mg, 2.63 mmol, 1.00 equiv.), CH_2Cl_2 (5.2 mL), Dess-Martin periodinane (1.67 g, 3.95 mmol, 1.50 equiv.), $NH_2OMe{\cdot}HCl$ (442 mg, 5.26 mmol, 2.00 equiv.), NaOAc (863 mg, 10.52 mmol, 4.00 equiv.), EtOH (26.3 mL), CH_2Cl_2 (2.6 mL). Purification by flash column chromatography (pentane/ethyl acetate, 8:1 to 3:1) afforded oxime **S13b** as a colorless oil (792 mg, 2.04 mmol, 78%, *E/Z* = 70:30).

^{1}H NMR (500 MHz, $CDCl_3$) δ (ppm) = 7.68-7.64 (m, 2H), 7.43 (d, J = 8.8 Hz, 0.6H), 7.37 (d, J = 8.8 Hz, 1.4H), 7.31 (d, J = 7.9 Hz, 0.6H), 7.29 (d, J = 7.8 Hz, 1.4H), 7.01 (t, J = 5.8 Hz, 0.7H), 6.88 (d, J = 8.9 Hz, 0.6H), 6.86 (d, J = 9.0 Hz, 1.4H), 6.30 (t, J = 4.0 Hz, 0.3H), 5.41 (s, 0.3H), 5.41 (s, 0.7H), 5.14 (s, 0.7H), 5.10 (s, 0.3H), 4.20 (s, 1.4H), 4.17 (s, 0.6H), 3.88 (d, J = 4.1 Hz, 0.6H), 3.82 (s, 0.9H), 3.81 (s, 2.1H), 3.80 (s, 0.9H), 3.79 (d, J = 5.8 Hz, 1.4H), 3.74 (s, 2.1H), 2.43 (s, 0.9H), 2.43 (s, 2.1H).

^{13}C NMR (126 MHz, $CDCl_3$) δ (ppm) = 159.77, 159.67, 148.13, 145.44, 143.92, 143.76, 141.59, 141.44, 136.13, 135.44, 130.52, 130.05, 129.99, 129.86, 127.87, 127.54, 127.50, 115.85, 115.60, 114.00, 113.89, 62.11, 61.74, 55.38, 53.51, 51.81, 45.60, 42.85, 21.64, 21.63.

IR (ATR): $\tilde{\nu}$ (cm^{-1}) = 2937, 1607, 1514, 1459, 1443, 1339, 1276, 1251, 1183, 1159, 1091, 1030.

HRMS (ESI^+) calcd. for $C_{20}H_{25}N_2O_4S^+$ $[M+H]^+$: 389.1530, found: 389.1515.

***N*-(2-(furan-3-yl)allyl)-*N*-(2-hydroxyethyl)-4-methylbenzenesulfonamide (S14a)**

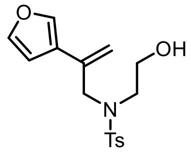

Prepared according to **General Procedure B (step B1)** using **S6a'** (2.66 g, 8.00 mmol, 1.00 equiv.), 3-furanylboronic acid (1.08 g, 9.60 mmol, 1.20 equiv.), Na_2CO_3 (2.54 g, 24.0 mmol, 3.00 equiv.), $Pd(PPh_3)_4$ (277 mg, 0.24 mmol, 3 mol%), 1,4-dioxane/H_2O (36 mL/4 mL). Purification by flash column chromatography (pentane/ethyl acetate, 5:1 to 3:1 to 1:1 and CH_2Cl_2/ethyl acetate, 10:1 to 4:1) afforded alcohol **S14a** as a pale-yellow solid (1.53 g, 4.77 mmol, 60%).

M.P.: 86-88 °C

^{1}H NMR (500 MHz, $CDCl_3$) δ (ppm) = 7.82 (t, *J* = 1.3 Hz, 1H), 7.71 (d, *J* = 8.3 Hz, 2H), 7.38 (t, *J* = 1.7 Hz, 1H), 7.33 (d, *J* = 8.2 Hz, 2H), 6.55 (dd, *J* = 1.9, 1.0 Hz, 1H), 5.38 (s, 1H), 5.05 (s, 1H), 3.99 (s, 2H), 3.56 (t, *J* = 5.6 Hz, 2H), 3.15 (t, *J* = 5.6 Hz, 2H), 2.43 (s, 3H), 2.21 (br. s, 1H).

^{13}C NMR (126 MHz, $CDCl_3$) δ (ppm) = 143.99, 143.45, 140.48, 134.98, 134.71, 129.99, 127.51, 123.69, 114.86, 108.06, 61.44, 54.63, 50.57, 21.62.

IR (ATR): $\tilde{\nu}$ (cm^{-1}) = 3006, 2989, 1756, 1598, 1457, 1343, 1276, 1261, 1163, 1089.

HRMS (ESI$^+$) calcd. for $C_{16}H_{20}NO_4S^+$ [M+H]$^+$: 322.1108, found: 322.1095.

***N*-(2-(furan-3-yl)allyl)-*N*-(2-(methoxyimino)ethyl)-4-methylbenzenesulfonamide (S14b)**

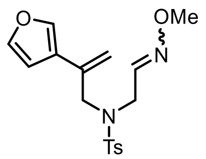

Prepared according to **General Procedure B (step B2)** using alcohol **S14a** (1.45 g, 4.50 mmol, 1.00 equiv.), CH_2Cl_2 (9.0 mL), Dess-Martin periodinane (2.87 g, 6.77 mmol, 1.50 equiv.), $NH_2OMe{\cdot}HCl$ (756 mg, 9.00 mmol, 2.00 equiv.), NaOAc (1.48 g, 18.00 mmol, 4.00 equiv.), EtOH (45.0 mL), CH_2Cl_2 (4.5 mL). Purification by flash column chromatography (pentane/ethyl acetate, 8:1 to 3:1) afforded oxime **S14b** as a white solid (934 mg, 2.68 mmol, 60%, *E/Z* = 75:25).

M.P.: 85-87 °C

^{1}H NMR (500 MHz, $CDCl_3$) δ (ppm) = 7.84 (s, 0.25H), 7.75 (s, 0.75H), 7.72-7.65 (m, 2H), 7.37-7.29 (m, 3H), 7.00 (t, *J* = 5.7 Hz, 0.75H), 6.53 (s, 0.25H), 6.52 (s, 0.75H), 6.40 (t, *J* = 4.0 Hz, 0.25H), 5.40 (s, 0.75H), 5.37 (s, 0.25H), 5.07 (s, 0.75H), 5.03 (s, 0.25H), 4.03 (s, 1.5H), 3.97 (s, 0.5H), 3.92 (d, *J* = 4.0 Hz, 0.5H), 3.82 (d, *J* = 5.8 Hz, 1.5H), 3.78 (s, 0.75H), 3.70 (s, 2.25H), 2.41 (s, 3H).

^{13}C NMR (126 MHz, $CDCl_3$) δ (ppm) = 147.85, 145.03, 144.04, 143.88, 143.24, 143.14, 140.57, 140.24, 135.89, 135.11, 133.59, 133.55, 130.02, 129.90, 127.39, 127.32, 123.65, 123.55, 115.43, 115.08, 108.13, 108.04, 62.01, 61.62, 53.73, 51.83, 45.49, 43.10, 21.52.

IR (ATR): $\tilde{\nu}$ (cm^{-1}) = 2938, 1638, 1598, 1446, 1338, 1161, 1093, 1066, 1028.

HRMS (ESI$^+$) calcd. for $C_{17}H_{21}N_2O_4S^+$ [M+H]$^+$: 349.1217, found: 349.1210.

***N*-(2-hydroxyethyl)-4-methyl-*N*-(2-(thiophen-2-yl)allyl)benzenesulfonamide (S15a)**

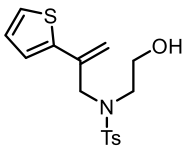

Prepared according to **General Procedure B (step B1)** using **S6a'** (2.66 g, 8.00 mmol, 1.00 equiv.), 2-thienylboronic acid (1.23 g, 9.60 mmol, 1.20 equiv.), Na_2CO_3 (2.54 g, 24.0 mmol, 3.00 equiv.), $Pd(PPh_3)_4$ (277 mg, 0.24 mmol, 3 mol%), 1,4-dioxane/H_2O (36 mL/4 mL). Purification by flash column chromatography (pentane/ethyl acetate, 5:1 to 3:1 to 1:1 and CH_2Cl_2/ethyl acetate, 10:1 to 4:1) afforded alcohol **S15a** as a white solid (1.85 g, 5.49 mmol, 69%).

M.P.: 85-87 °C

^{1}H NMR (500 MHz, $CDCl_3$) δ (ppm) = 7.75 (d, *J* = 8.3 Hz, 2H), 7.39-7.35 (m, 3H), 7.24 (dd, *J* = 5.2, 1.1 Hz, 1H), 7.04 (dd, *J* = 5.1, 3.6 Hz, 1H), 5.56 (s, 1H), 5.11 (s, 1H), 4.19 (s, 2H), 3.63 (app. q, *J* = 5.6 Hz, 2H), 3.22 (t, *J* = 5.4 Hz, 2H), 2.48 (s, 3H), 2.06 (t, *J* = 6.1 Hz, 1H).

^{13}C NMR (126 MHz, $CDCl_3$) δ (ppm) = 143.92, 141.53, 136.60, 135.13, 129.94, 127.88, 127.49, 125.31, 125.17, 115.16, 61.26, 54.17, 50.45, 21.60.

IR (ATR): $\tilde{\nu}$ (cm^{-1}) = 3006, 2989, 1455, 1342, 1276, 1261, 1163, 1090.

HRMS (ESI$^+$) calcd. for $C_{16}H_{20}NO_3S_2^+$ [M+H]$^+$: 338.0879, found: 338.0890.

***N*-(2-(methoxyimino)ethyl)-4-methyl-*N*-(2-(thiophen-2-yl)allyl)benzenesulfonamide (S15b)**

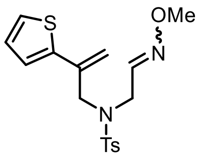

Prepared according to **General Procedure B (step B2)** using alcohol **S15a** (1.63 g, 4.84 mmol, 1.00 equiv.), CH_2Cl_2 (9.7 mL), Dess-Martin periodinane (3.08 g, 7.26 mmol, 1.50 equiv.), $NH_2OMe{\cdot}HCl$ (813 mg, 9.68 mmol, 2.00 equiv.), NaOAc (1.59 g, 19.36 mmol, 4.00 equiv.), EtOH (48.4 mL), CH_2Cl_2 (4.8 mL). Purification by flash column chromatography (pentane/ethyl acetate, 8:1 to 3:1) afforded oxime **S15b** as a colorless oil (1.17 g, 3.21 mmol, 66%, *E/Z* = 75:25).

^{1}H NMR (500 MHz, $CDCl_3$) δ (ppm) = 7.70 (d, *J* = 8.3 Hz, 2H), 7.34-7.29 (m, 2.25H), 7.25 (dd, *J* = 3.7, 1.1 Hz, 0.75H), 7.20-7.16 (m, 1H), 7.04 (t, *J* = 5.8 Hz, 0.75H), 7.00-6.95 (m, 1H), 6.42 (t, *J* = 4.1 Hz, 0.25H), 5.55 (s, 0.75H), 5.52 (s, 0.25H), 5.12 (s, 0.75H), 5.06 (s, 0.25H), 4.18 (s, 1.5H), 4.13 (s, 0.5H), 3.96 (d, *J* = 4.1 Hz, 0.5H), 3.86 (d, *J* = 5.8 Hz, 1.5H), 3.79 (s, 0.75H), 3.71 (s, 2.25H), 2.42 (s, 0.75H), 2.42 (s, 2.25H).

^{13}C NMR (126 MHz, $CDCl_3$) δ (ppm) = 147.73, 144.98, 144.00, 143.84, 141.55, 141.32, 135.97, 135.60, 135.22, 130.00, 129.86, 127.78, 127.68, 127.40, 127.35, 125.48, 125.13, 125.02, 124.92, 115.72, 115.35, 62.02, 61.64, 53.44, 51.59, 45.67, 43.07, 21.54, 21.53.

IR (ATR): $\tilde{\nu}$ (cm^{-1}) = 2937, 2901, 1622, 1597, 1440, 1338, 1159, 1091, 1033.

HRMS (ESI^+) calcd. for $C_{17}H_{21}N_2O_3S_2^+$ $[M+H]^+$: 365.0988, found: 365.0979.

***N*-(2-hydroxyethyl)-*N*-(2-(6-methoxypyridin-3-yl)allyl)-4-methylbenzenesulfonamide (S16a)**

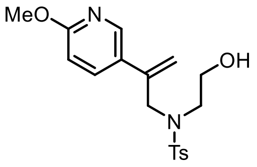

Prepared according to **General Procedure B (step B1)** using **S6a'** (2.66 g, 8.00 mmol, 1.00 equiv.), 2-methoxy-5-pyridineboronic acid (1.47 g, 9.60 mmol, 1.20 equiv.), Na_2CO_3 (2.54 g, 24.0 mmol, 3.00 equiv.), $Pd(PPh_3)_4$ (277 mg, 0.24 mmol, 3 mol%), 1,4-dioxane/H_2O (36 mL/4 mL). Purification by flash column chromatography (pentane/ethyl acetate, 3:1 to 1:1 to 1:3 and CH_2Cl_2/ethyl acetate, 7:1 to 2:1) afforded alcohol **S16a** as a white solid (1.60 g, 4.42 mmol, 55%).

M.P.: 80-82 °C

^{1}H NMR (500 MHz, $CDCl_3$) δ (ppm) = 8.24 (dd, *J* = 2.6, 0.7 Hz, 1H), 7.68-7.64 (m, 3H), 7.28 (d, *J* = 8.1 Hz, 2H), 6.68 (dd, *J* = 8.7, 0.7 Hz, 1H), 5.41 (s, 1H), 5.18 (s, 1H), 4.16 (s, 2H), 3.91 (s, 3H), 3.55 (app. q, *J* = 5.7 Hz, 2H), 3.15 (t, *J* = 5.7 Hz, 2H), 2.42-2.39 (m, 4H).

^{13}C NMR (126 MHz, $CDCl_3$) δ (ppm) = 164.03, 144.87, 143.88, 139.99, 136.84, 135.26, 129.89, 127.49, 126.93, 116.30, 110.57, 61.02, 53.73, 53.62, 50.09, 21.59.

IR (ATR): $\tilde{\nu}$ (cm^{-1}) = 3006, 2989, 1601, 1496, 1459, 1330, 1276, 1261, 1159, 1023.

HRMS (ESI^+) calcd. for $C_{18}H_{23}N_2O_4S^+$ $[M+H]^+$: 363.1373, found: 363.1357.

***N*-(2-(methoxyimino)ethyl)-*N*-(2-(6-methoxypyridin-3-yl)allyl)-4-methylbenzenesulfonamide (S16b)**

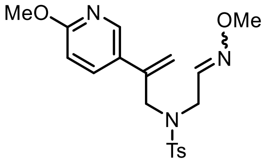

Prepared according to **General Procedure B (step B2)** using alcohol **S16a** (1.02 g, 2.82 mmol, 1.00 equiv.), CH_2Cl_2 (5.6 mL), Dess-Martin periodinane (1.80 g, 4.23 mmol, 1.50 equiv.), $NH_2OMe{\cdot}HCl$ (474 mg, 5.64 mmol, 2.00 equiv.), NaOAc (925 mg, 11.28 mmol, 4.00 equiv.), EtOH (28.2 mL), CH_2Cl_2 (2.8 mL). Purification by flash column chromatography (pentane/ethyl acetate, 5:1 to 1:1) afforded oxime **S16b** as a colorless oil (814 mg, 2.09 mmol, 74%, *E/Z* = 70:30).

^{1}H NMR (500 MHz, $CDCl_3$) δ (ppm) = 8.24 (dd, *J* = 2.6, 0.7 Hz, 0.3H), 8.18 (dd, *J* = 2.6, 0.8 Hz, 0.7H), 7.67-7.58 (m, 3H), 7.26 (d, *J* = 8.5 Hz, 0.6H), 7.24 (d, *J* = 8.5 Hz, 1.4H), 6.97 (t, *J* = 5.8 Hz, 0.7H), 6.66 (d, *J* = 8.7 Hz, 0.3H), 6.63 (d, *J* = 8.7 Hz, 0.7H), 6.29 (t, *J* = 4.1 Hz, 0.3H), 5.39 (s, 0.3H), 5.38 (s, 0.7H), 5.17 (s, 0.7H), 5.12 (s, 0.3H), 4.13 (s, 1.4H), 4.10 (s, 0.6H), 3.88 (s, 0.9H), 3.87 (s, 2.1H), 3.85 (d, *J* = 4.3 Hz, 0.6H), 3.76-3.72 (m, 2.3H), 3.67 (s, 2.1H), 2.36 (s, 3H).

^{13}C NMR (126 MHz, $CDCl_3$) δ (ppm) = 163.85, 163.78, 147.38, 144.83, 144.81, 143.88, 143.72, 139.11, 138.94, 136.69, 136.61, 135.74, 135.03, 129.84, 129.72, 127.23, 126.89, 126.46, 116.64, 116.47, 110.35, 110.23, 61.88, 61.52, 53.34, 53.32, 53.03, 51.45, 45.49, 42.60, 21.37, 21.36.

IR (ATR): $\tilde{\nu}$ (cm^{-1}) = 2943, 1630, 1602, 1495, 1370, 1342, 1289, 1161, 1092, 1027.

HRMS (ESI^+) calcd. for $C_{19}H_{24}N_3O_4S^+$ $[M+H]^+$: 390.1482, found: 390.1470.

***N*-(2-hydroxyethyl)-*N*-(2-(2-methoxypyrimidin-5-yl)allyl)-4-methylbenzenesulfonamide (S17a)**

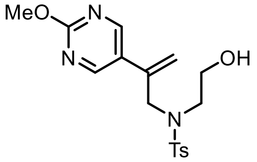

Prepared according to **General Procedure B (step B1)** using **S6a'** (2.66 g, 8.00 mmol, 1.00 equiv.), 2-methoxypyrimidine-5-boronic acid (1.48 g, 9.60 mmol, 1.20 equiv.), Na_2CO_3 (2.54 g, 24.0 mmol, 3.00 equiv.), $Pd(PPh_3)_4$ (277 mg, 0.24 mmol, 3 mol%), 1,4-dioxane/H_2O (36 mL/4 mL). Purification by flash column chromatography (pentane/ethyl acetate, 1:1 to 100% ethyl acetate) afforded alcohol **S17a** as a white solid (1.70 g, 4.68 mmol, 59%).

M.P.: 138-140 °C

^{1}**H NMR** (500 MHz, $CDCl_3$) δ (ppm) = 8.61 (s, 2H), 7.68 (d, *J* = 8.3 Hz, 2H), 7.32 (d, *J* = 8.1 Hz, 2H), 5.49 (s, 1H), 5.30 (s, 1H), 4.20 (s, 2H), 4.03 (s, 3H), 3.61 (app. q, *J* = 5.6 Hz, 2H), 3.19 (t, *J* = 5.6 Hz, 2H), 2.44 (s, 3H), 2.20 (t, *J* = 5.7 Hz, 1H).

^{13}**C NMR** (126 MHz, $CDCl_3$) δ (ppm) = 165.20, 157.16, 144.00, 137.41, 135.20, 129.92, 127.41, 125.38, 117.64, 60.96, 55.12, 53.46, 50.15, 21.58.

IR (ATR): $\tilde{\nu}$ (cm^{-1}) = 3006, 2989, 1594, 1550, 1477, 1417, 1333, 1276, 1261, 1159, 1037.

HRMS (ESI^+) calcd. for $C_{17}H_{22}N_3O_4S^+$ $[M+H]^+$: 364.1326, found: 364.1310.

***N*-(2-(methoxyimino)ethyl)-*N*-(2-(2-methoxypyrimidin-5-yl)allyl)-4-methylbenzene-sulfonamide (S17b)**

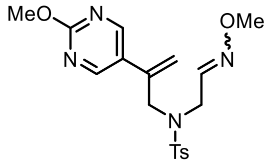

Prepared according to **General Procedure B (step B2)** using alcohol **S17a** (1.37 g, 3.77 mmol, 1.00 equiv.), CH_2Cl_2 (7.6 mL), Dess-Martin periodinane (2.40 g, 5.66 mmol, 1.50 equiv.), $NH_2OMe \cdot HCl$ (634 mg, 7.55 mmol, 2.00 equiv.), NaOAc (1.24 g, 15.10 mmol, 4.00 equiv.), EtOH (37.7 mL), CH_2Cl_2 (3.8 mL). Purification by flash column chromatography (pentane/ethyl acetate, 2:1 to 1:2) afforded oxime **S17b** as a colorless oil (1.25 g, 3.21 mmol, 85%, *E/Z* = 70:30).

^{1}**H NMR** (500 MHz, $CDCl_3$) δ (ppm) = 8.51 (s, 0.6H), 8.45 (s, 1.4H), 7.56-7.51 (m, 2H), 7.22-7.16 (m, 2H), 6.87 (t, J = 5.9 Hz, 0.7H), 6.22 (t, J = 4.2 Hz, 0.3H), 5.38 (s, 0.3H), 5.36 (s, 0.7H), 5.19 (d, J = 1.4 Hz, 0.7H), 5.15 (s, 0.3H), 4.05 (s, 1.4H), 4.03 (s, 0.6H), 3.88 (s, 0.9H), 3.87 (s, 2.1H), 3.77 (d, J = 4.3 Hz, 0.6H), 3.68-3.63 (m, 2.3H), 3.59 (s, 2.1H), 2.29 (s, 3H).

^{13}**C NMR** (126 MHz, $CDCl_3$) δ (ppm) = 165.03, 164.97, 156.94, 156.88, 146.74, 144.39, 143.96, 143.81, 136.39, 136.16, 135.32, 134.66, 129.79, 129.69, 127.06, 125.04, 124.66, 117.94, 117.88, 61.79, 61.43, 54.75, 54.72, 52.62, 51.17, 45.50, 42.43, 21.25, 21.24.

IR (ATR): $\tilde{\nu}$ (cm^{-1}) = 2960, 1633, 1592, 1548, 1474, 1415, 1333,1315, 1160, 1092, 1032.

HRMS (ESI^+) calcd. for $C_{18}H_{23}N_4O_4S^+$ $[M+H]^+$: 391.1435, found: 391.1421.

***N*-(2-(benzo[*d*][1,3]dioxol-5-yl)allyl)-*N*-(2-hydroxyethyl)-4-methylbenzenesulfonamide (S18a)**

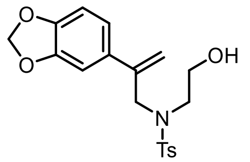

Prepared according to **General Procedure B (step B1)** using **S6a'** (2.66 g, 8.00 mmol, 1.00 equiv.), 3,4-(methylenedioxy)phenylboronic acid (1.59 g, 9.60 mmol, 1.20 equiv.), Na_2CO_3 (2.54 g, 24.0 mmol, 3.00 equiv.), $Pd(PPh_3)_4$ (277 mg, 0.24 mmol, 3 mol%), 1,4-dioxane/H_2O (36 mL/4 mL). Purification by flash column chromatography (pentane/ethyl acetate, 5:1 to 3:1 to 1:1 and CH_2Cl_2/ethyl acetate, 10:1 to 4:1) afforded alcohol **S18a** as a white solid (1.56 g, 4.16 mmol, 52%).

M.P.: 106-108 °C

^{1}H NMR (500 MHz, $CDCl_3$) δ (ppm) = 7.66 (d, *J* = 8.4 Hz, 2H), 7.29 (d, *J* = 8.0 Hz, 2H), 6.95 (dd, *J* = 8.1, 1.9 Hz, 1H), 6.91 (d, *J* = 1.8 Hz, 1H), 6.74 (dd, *J* = 8.1, 1.3 Hz, 1H), 5.93 (s, 2H), 5.37 (s, 1H), 5.12 (s, 1H), 4.15 (s, 2H), 3.54 (app. q, *J* = 5.6 Hz, 2H), 3.15 (t, *J* = 5.6 Hz, 2H), 2.42 (s, 3H), 2.31-2.25 (m, 1H).

^{13}C NMR (126 MHz, $CDCl_3$) δ (ppm) = 147.88, 147.67, 143.78, 142.38, 135.25, 131.92, 129.82, 127.49, 120.29, 115.89, 108.26, 106.91, 101.21, 61.01, 53.90, 50.05, 21.55.

IR (ATR): $\tilde{\nu}$ (cm^{-1}) = 3006, 2989, 1599, 1504, 1491, 1443, 1335, 1276, 1260, 1234, 1158, 1037.

HRMS (ESI^+) calcd. for $C_{19}H_{22}NO_5S^+$ $[M+H]^+$: 376.1213, found: 376.1213.

***N*-(2-(benzo[*d*][1,3]dioxol-5-yl)allyl)-*N*-(2-(methoxyimino)ethyl)-4-methylbenzenesulfonamide (S18b)**

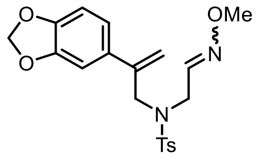

Prepared according to **General Procedure B (step B2)** using alcohol **S18a** (1.11 g, 2.96 mmol, 1.00 equiv.), CH_2Cl_2 (6.0 mL), Dess-Martin periodinane (1.88 g, 4.44 mmol, 1.50 equiv.), $NH_2OMe{\cdot}HCl$ (497 mg, 5.92 mmol, 2.00 equiv.), NaOAc (971 mg, 11.84 mmol, 4.00 equiv.), EtOH (29.6 mL), CH_2Cl_2 (3.0 mL). Purification by flash column chromatography (pentane/ethyl acetate, 8:1 to 3:1) afforded oxime **S18b** as a colorless oil (897 mg, 2.23 mmol, 75%, *E/Z* = 70:30).

^{1}H NMR (500 MHz, $CDCl_3$) δ (ppm) = 7.68-7.64 (m, 2H), 7.31 (d, *J* = 7.9 Hz, 0.6H), 7.28 (d, *J* = 8.0 Hz, 1.4H), 7.03 (t, *J* = 5.8 Hz, 0.7H), 6.98-6.86 (m, 2H), 6.76 (d, *J* = 8.1 Hz, 0.3H), 6.74 (d, *J* = 8.1

Hz, 0.7H), 6.35 (t, *J* = 4.1 Hz, 0.3H), 5.94 (s, 0.6H), 5.93 (s, 1.4H), 5.38 (d, *J* = 0.6 Hz, 0.3H), 5.37 (d, *J* = 0.9 Hz, 0.7H), 5.15 (d, *J* = 1.1 Hz, 0.7H), 5.11 (d, *J* = 1.1 Hz, 0.3H), 4.15 (s, 1.4H), 4.13 (s, 0.6H), 3.89 (d, *J* = 4.1 Hz, 0.6H), 3.81-3.78 (m, 2.3H), 3.74 (s, 2.1H), 2.42 (s, 0.9H), 2.42 (s, 2.1H).

^{13}C NMR (126 MHz, $CDCl_3$) δ (ppm) = 147.98, 147.89, 147.78, 147.72, 147.58, 145.31, 143.91, 143.76, 141.78, 141.59, 136.08, 135.40, 132.31, 131.81, 129.94, 129.82, 127.45, 127.42, 120.44, 120.37, 116.43, 116.16, 108.26, 108.17, 107.14, 106.99, 101.22, 101.17, 62.06, 61.70, 53.43, 51.76, 45.60, 42.81, 21.58, 21.57.

IR (ATR): $\tilde{\nu}$ (cm^{-1}) = 2937, 1599, 1504, 1491, 1442, 1341, 1231, 1159, 1091, 1034.

HRMS (ESI$^+$) calcd. for $C_{20}H_{23}N_2O_5S^+$ [M+H]$^+$: 403.1322, found: 403.1310.

***N*-(2-(4-(4-((2,4-dimethyl-3-oxopentan-2-yl)oxy)benzoyl)phenyl)allyl)-*N*-(2-hydroxyethyl)-4-methylbenzenesulfonamide (S19a)**

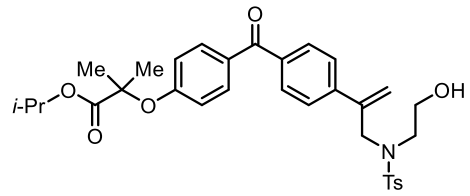

Prepared according to **General Procedure B (step B1)** using **S6a'** (2.66 g, 8.00 mmol, 1.00 equiv.), (4-(4-((1-isopropoxy-2-methyl-1-oxopropan-2-yl)oxy)benzoyl)phenyl)boronic acid[3] (3.55 g, 9.60 mmol, 1.20 equiv.), Na_2CO_3 (2.54 g, 24.0 mmol, 3.00 equiv.), $Pd(PPh_3)_4$ (277 mg, 0.24 mmol, 3 mol%), 1,4-dioxane/H_2O (36 mL/4 mL). Purification by flash column chromatography (pentane/ethyl acetate, 5:1 to 1:1 to 1:2 and CH_2Cl_2/ethyl acetate, 8:1 to 3:1) afforded alcohol **S19a** as a white solid (2.44 g, 4.21 mmol, 53%).

M.P.: 93-95 °C

^{1}H NMR (500 MHz, $CDCl_3$) δ (ppm) = 7.70 (d, *J* = 8.8 Hz, 2H), 7.63 (d, *J* = 8.4 Hz, 2H), 7.61 (d, *J* = 8.4 Hz, 2H), 7.49 (d, *J* = 8.6 Hz, 2H), 7.22 (d, *J* = 8.0 Hz, 2H), 6.82 (d, *J* = 8.9 Hz, 2H), 5.52 (s, 1H), 5.29 (s, 1H), 5.02 (p, *J* = 6.3 Hz, 1H), 4.22 (s, 2H), 3.50 (t, *J* = 5.9 Hz, 2H), 3.14 (t, *J* = 6.0 Hz, 2H), 2.67 (s, 1H), 2.32 (s, 3H), 1.60 (s, 6H), 1.15 (s, 3H), 1.13 (s, 3H).

^{13}C NMR (126 MHz, $CDCl_3$) δ (ppm) = 194.75, 172.92, 159.47, 143.60, 142.04, 141.53, 137.28, 135.28, 131.83, 130.30, 129.86, 129.67, 127.22, 126.16, 118.43, 117.12, 79.25, 69.18, 60.66, 53.17, 49.71, 25.21, 21.36, 21.33.

IR (ATR): $\tilde{\nu}$ (cm^{-1}) = 2988, 1727, 1647, 1599, 1504, 1452, 1384, 1276, 1260, 1179, 1148, 1101.

HRMS (ESI$^+$) calcd. for $C_{32}H_{38}NO_7S^+$ $[M+H]^+$: 580.2363, found: 580.2369.

***N*-(2-(4-(4-((2,4-dimethyl-3-oxopentan-2-yl)oxy)benzoyl)phenyl)allyl)-*N*-(2-(methoxyimino)ethyl)-4-methylbenzenesulfonamide (S19b)**

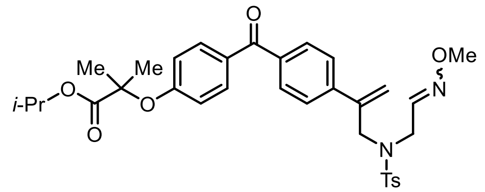

Prepared according to **General Procedure B (step B2)** using alcohol **S19a** (1.47 g, 2.54 mmol, 1.00 equiv.), CH_2Cl_2 (5.1 mL), Dess-Martin periodinane (1.61 g, 3.81 mmol, 1.50 equiv.), $NH_2OMe{\cdot}HCl$ (424 mg, 5.08 mmol, 2.00 equiv.), NaOAc (833 mg, 10.16 mmol, 4.00 equiv.), EtOH (25.4 mL), CH_2Cl_2 (2.5 mL). Purification by flash column chromatography (pentane/ethyl acetate, 8:1 to 2:1) afforded oxime **S19b** as a colorless oil (1.12 g, 1.85 mmol, 73%, *E/Z* = 73:27).

^{1}H NMR (500 MHz, $CDCl_3$) δ (ppm) = 7.74-7.58 (m, 6H), 7.53 (d, *J* = 8.7 Hz, 0.54H), 7.47 (d, *J* = 8.5 Hz, 1.46H), 7.27-7.20 (m, 2H), 6.96 (t, *J* = 5.9 Hz, 0.73H), 6.84-6.81 (m, 2H), 6.29 (t, *J* = 4.1 Hz, 0.27H), 5.55 (s, 0.27H), 5.54 (s, 0.73H), 5.31 (s, 0.73H), 5.28 (s, 0.27H), 5.06-4.99 (m, 1H), 4.21 (s, 1.46H), 4.19 (s, 0.54H), 3.87-3.65 (m, 5H), 2.34 (s, 3H), 1.61 (s, 6H), 1.17-1.09 (m, 6H).

^{13}C NMR (126 MHz, $CDCl_3$) δ (ppm) = 194.68, 194.63, 172.93, 159.50, 159.48, 147.48, 144.91, 143.91, 143.75, 141.60, 141.51, 141.30, 141.14, 137.52, 137.41, 135.76, 135.08, 131.86, 130.42, 130.39, 129.92, 129.87, 129.82, 129.75, 127.27, 126.33, 126.27, 119.16, 118.78, 117.10, 79.27, 69.17, 61.92, 61.54, 53.03, 51.37, 45.56, 42.73, 25.26, 21.41, 21.40.

IR (ATR): $\tilde{\nu}$ (cm^{-1}) = 3005, 2988, 1729, 1651, 1598, 1503, 1464, 1385, 1276, 1260, 1177, 1149, 1100, 1031.

HRMS (ESI$^+$) calcd. for $C_{33}H_{39}N_2O_7S^+$ $[M+H]^+$: 607.2472, found: 607.2477.

Ethyl 2-(4-(3-((*N*-(2-hydroxyethyl)-4-methylphenyl)sulfonamido)prop-1-en-2-yl)phenoxy)-2-methylpropanoate (S20a)

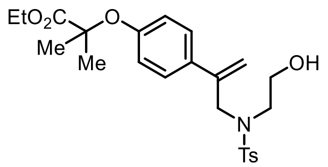

Prepared according to **General Procedure B (step B1)** using **S6a'** (1.67 g, 5.00 mmol, 1.00 equiv.), (4-((1-ethoxy-2-methyl-1-oxopropan-2-yl)oxy)phenyl)boronic acid[3] (1.45 g, 5.75 mmol, 1.15 equiv.),

Na_2CO_3 (1.59 g, 15.0 mmol, 3.00 equiv.), $Pd(PPh_3)_4$ (173 mg, 0.15 mmol, 3 mol%), 1,4-dioxane/H_2O (22.5 mL/2.5 mL). Purification by flash column chromatography (pentane/ethyl acetate, 5:1 to 1:1 and CH_2Cl_2/ethyl acetate, 8:1 to 3:1) afforded alcohol **S20a** as a colorless oil (1.27 g, 2.75 mmol, 55%).

^{1}H NMR (500 MHz, $CDCl_3$) δ (ppm) = 7.60 (d, *J* = 8.3 Hz, 2H), 7.29 (d, *J* = 8.8 Hz, 2H), 7.23 (d, *J* = 8.1 Hz, 2H), 6.73 (d, *J* = 8.8 Hz, 2H), 5.34 (s, 1H), 5.08 (s, 1H), 4.16 (q, *J* = 7.1 Hz, 2H), 4.12 (s, 2H), 3.46 (app. q, *J* = 5.7 Hz, 2H), 3.08 (t, *J* = 5.8 Hz, 2H), 2.42 (t, *J* = 5.9 Hz, 1H), 2.35 (s, 3H), 1.54 (s, 6H), 1.17 (t, *J* = 7.1 Hz, 3H).

^{13}C NMR (126 MHz, $CDCl_3$) δ (ppm) = 173.97, 155.41, 143.57, 141.87, 135.22, 131.36, 129.68, 127.31, 127.16, 118.62, 115.35, 79.00, 61.37, 60.71, 53.41, 49.78, 25.26, 21.37, 13.94.

IR (ATR): $\tilde{\nu}$ (cm^{-1}) = 2988, 1731, 1604, 1509, 1454, 1383, 1334, 1276, 1261, 1242, 1148, 1089.

HRMS (ESI^+) calcd. for $C_{24}H_{32}NO_6S^+$ $[M+H]^+$: 462.1945, found: 462.1945.

Ethyl 2-(4-(3-((*N*-(2-(methoxyimino)ethyl)-4-methylphenyl)sulfonamido)prop-1-en-2-yl)phenoxy)-2-methylpropanoate (S20b)

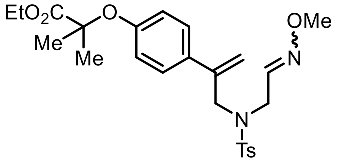

Prepared according to **General Procedure B (step B2)** using alcohol **S20a** (1.38 g, 3.00 mmol, 1.00 equiv.), CH_2Cl_2 (6.0 mL), Dess-Martin periodinane (1.91 g, 4.50 mmol, 1.50 equiv.), $NH_2OMe\cdot HCl$ (504 mg, 6.00 mmol, 2.00 equiv.), NaOAc (984 mg, 12.00 mmol, 4.00 equiv.), EtOH (30.0 mL), CH_2Cl_2 (3.0 mL). Purification by flash column chromatography (pentane/ethyl acetate, 8:1 to 3:1) afforded oxime **S20b** as a colorless oil (893 mg, 1.83 mmol, 61%, *E/Z* = 75:25).

^{1}H NMR (500 MHz, $CDCl_3$) δ (ppm) = 7.67-7.63 (m, 2H), 7.37 (d, *J* = 8.8 Hz, 0.5H), 7.33-7.26 (m, 3.5H), 7.00 (t, *J* = 5.8 Hz, 0.75H), 6.82-6.77 (m, 2H), 6.30 (t, *J* = 4.0 Hz, 0.25H), 5.42 (s, 0.25H), 5.41 (s, 0.75H), 5.16 (d, *J* = 1.2 Hz, 0.75H), 5.11 (d, *J* = 1.1 Hz, 0.25H), 4.25-4.20 (m, 2H), 4.18 (s, 1.5H), 4.15 (s, 0.5H), 3.88 (d, *J* = 4.1 Hz, 0.5H), 3.79-3.75 (m, 2.25H), 3.71 (s, 2.25H), 2.41 (s, 3H), 1.61 (s, 1.5H), 1.60 (s, 4.5H), 1.26-1.21 (m, 3H).

^{13}C NMR (126 MHz, $CDCl_3$) δ (ppm) = 174.02, 174.00, 155.58, 155.46, 147.86, 145.21, 143.82, 143.66, 141.29, 141.12, 135.91, 135.22, 131.58, 131.11, 129.86, 129.74, 127.38, 127.34, 127.32, 118.58, 118.55, 116.06, 115.73, 79.02, 61.91, 61.53, 61.41, 61.39, 53.19, 51.50, 45.47, 42.72, 25.33, 21.46, 21.45, 14.03.

IR (ATR): $\tilde{\nu}$ (cm^{-1}) = 3005, 2989, 1731, 1605, 1510, 1462, 1342, 1276, 1261, 1159, 1138, 1091.

HRMS (ESI^+) calcd. for $C_{25}H_{33}N_2O_6S^+$ $[M+H]^+$: 489.2054, found: 489.2058.

_N_-(2-hydroxyethyl)-4-methyl-_N_-(2-(4-(5-(_p_-tolyl)-3-(trifluoromethyl)-1_H_-pyrazol-1-yl)phenyl)allyl)benzenesulfonamide (S21a)

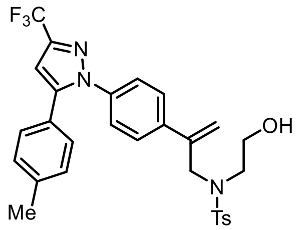

Prepared according to **General Procedure B (step B1)** using **S6a'** (2.66 g, 8.00 mmol, 1.00 equiv.), (4-(5-(-tolyl)-3-(trifluoromethyl)-1*H*-pyrazol-1-yl)phenyl)boronic acid[4] (3.32 g, 9.60 mmol, 1.20 equiv.), Na_2CO_3 (2.54 g, 24.0 mmol, 3.00 equiv.), $Pd(PPh_3)_4$ (277 mg, 0.24 mmol, 3 mol%), 1,4-dioxane/H_2O (36 mL/4 mL). Purification by flash column chromatography (pentane/ethyl acetate, 5:1 to 3:1 to 1:1 and CH_2Cl_2/ethyl acetate, 10:1 to 4:1) afforded alcohol **S21a** as a pale-yellow oil (1.83 g, 3.30 mmol, 41%).

^{1}H NMR (500 MHz, $CDCl_3$) δ (ppm) = 7.68 (d, *J* = 8.0 Hz, 2H), 7.46 (d, *J* = 8.5 Hz, 2H), 7.31 (d, *J* = 8.3 Hz, 2H), 7.29 (d, *J* = 8.5 Hz, 2H), 7.17 (d, *J* = 8.1 Hz, 2H), 7.13 (d, *J* = 8.0 Hz, 2H), 6.74 (s, 1H), 5.55 (s, 1H), 5.31 (s, 1H), 4.24 (s, 2H), 3.54 (app. q, *J* = 5.7 Hz, 2H), 3.17 (t, *J* = 5.7 Hz, 2H), 2.42 (s, 3H), 2.40-2.33 (m, 4H).

^{13}C NMR (126 MHz, $CDCl_3$) δ (ppm) = 144.90, 143.92, 143.17 (q, *J* = 38.1 Hz), 141.79, 139.32, 138.95, 137.84, 135.16, 129.88, 129.53, 128.65, 127.43, 127.16, 126.07, 125.38, 121.36 (q, *J* = 268.9 Hz), 117.81, 105.42 (q, *J* = 2.6 Hz), 60.91, 53.58, 49.97, 21.46, 21.28.

^{19}F NMR (471 MHz, $CDCl_3$) δ (ppm) = −62.07.

IR (ATR): $\tilde{\nu}$ (cm^{-1}) = 2926, 1599, 1509, 1472, 1449, 1376, 1334, 1271, 1235, 1157, 1131, 1096.

HRMS (ESI^+) calcd. for $C_{29}H_{29}F_3N_3O_3S^+$ $[M+H]^+$: 556.1876, found: 556.1856.

_N_-(2-(methoxyimino)ethyl)-4-methyl-_N_-(2-(4-(5-(_p_-tolyl)-3-(trifluoromethyl)-1_H_-pyrazol-1-yl)phenyl)allyl)benzenesulfonamide (S21b)

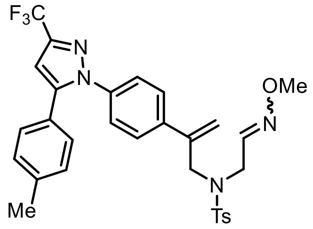

Prepared according to **General Procedure B (step B2)** using alcohol **S21a** (1.62 g, 2.92 mmol, 1.00 equiv.), CH_2Cl_2 (5.8 mL), Dess-Martin periodinane (1.86 g, 4.38 mmol, 1.50 equiv.), $NH_2OMe{\cdot}HCl$ (491 mg, 5.84 mmol, 2.00 equiv.), NaOAc (958 mg, 11.68 mmol, 4.00 equiv.), EtOH (29.2 mL), CH_2Cl_2 (2.9 mL). Purification by flash column chromatography (pentane/ethyl acetate, 8:1 to 3:1) afforded oxime **S21b** as a colorless oil (1.45 g, 2.49 mmol, 85%, *E/Z* = 70:30).

^{1}H NMR (500 MHz, $CDCl_3$) δ (ppm) = 7.66-7.62 (m, 2H), 7.46 (d, *J* = 8.7 Hz, 0.6H), 7.40 (d, *J* = 8.6 Hz, 1.4H), 7.30-7.22 (m, 4H), 7.15-7.09 (m, 4H), 6.99 (t, *J* = 5.8 Hz, 0.7H), 6.71 (s, 1H), 6.30 (t, *J* = 4.1 Hz, 0.3H), 5.52 (s, 0.3H), 5.51 (s, 0.7H), 5.30 (s, 0.7H), 5.25 (s, 0.3H), 4.20 (s, 1.4H), 4.18 (s, 0.6H), 3.89 (d, *J* = 4.1 Hz, 0.6H), 3.79-3.75 (m, 2.3H), 3.70 (s, 2.1H), 2.38 (s, 0.9H), 2.37 (s, 2.1H), 2.32 (s, 3H).

^{13}C NMR (126 MHz, $CDCl_3$) δ (ppm) = 147.54, 144.96, 144.77, 144.74, 143.95, 143.80, 142.99 (q, *J* = 38.0 Hz), 142.97 (q, *J* = 38.5 Hz), 141.06, 140.88, 139.17, 139.12, 138.89, 138.80, 137.93, 137.51, 135.70, 134.99, 129.86, 129.75, 129.42, 129.39, 128.51, 127.23, 127.22, 127.15, 125.97, 125.95, 125.21, 125.10, 121.33 (q, *J* = 268.9 Hz), 118.35, 118.02, 105.27, 105.25, 61.82, 61.43, 53.10, 51.50, 45.48, 42.60, 21.26, 21.23, 21.07.

^{19}F NMR (471 MHz, $CDCl_3$) δ (ppm) = −61.96.

IR (ATR): $\tilde{\nu}$ (cm^{-1}) = 2937, 1599, 1509, 1472, 1448, 1376, 1344, 1236, 1159, 1132, 1095, 1033.

HRMS (ESI$^+$) calcd. for $C_{30}H_{30}F_3N_4O_3S^+$ [M+H]$^+$: 583.1985, found: 583.1963.

Ethyl 2-(4-(2-(4-(3-((*N*-(2-hydroxyethyl)-4-methylphenyl)sulfonamido)prop-1-en-2-yl)benzamido)ethyl)phenoxy)-2-methylpropanoate (S22a)

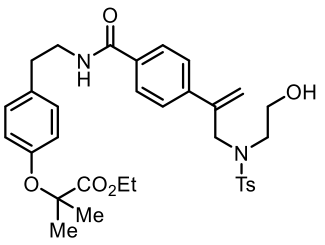

Prepared according to **General Procedure B (step B1)** using **S6a'** (2.66 g, 8.00 mmol, 1.00 equiv.), (4-((4-((1-ethoxy-2-methyl-1-oxopropan-2-yl)oxy)phenethyl)carbamoyl)phenyl)boronic acid [3] (3.83 g, 9.60 mmol, 1.20 equiv.), Na_2CO_3 (2.54 g, 24.0 mmol, 3.00 equiv.), $Pd(PPh_3)_4$ (277 mg, 0.24 mmol, 3 mol%), 1,4-dioxane/H_2O (36 mL/4 mL). Purification by flash column chromatography (pentane/ethyl acetate, 5:1 to 1:1 to 1:3 and CH_2Cl_2/ethyl acetate, 4:1 to 1:1) afforded alcohol **S22a** as a colorless oil (2.89 g, 4.75 mmol, 59%).

^{1}**H NMR** (500 MHz, $CDCl_3$) δ (ppm) = 7.60 (d, *J* = 4.7 Hz, 2H), 7.58 (d, *J* = 4.6 Hz, 2H), 7.37 (d, *J* = 8.2 Hz, 2H), 7.21 (d, *J* = 8.1 Hz, 2H), 7.01 (d, *J* = 8.6 Hz, 2H), 6.95 (t, *J* = 5.8 Hz, 1H), 6.72 (d, *J* = 8.5 Hz, 2H), 5.43 (s, 1H), 5.22 (s, 1H), 4.18-4.11 (m, 4H), 3.52 (q, *J* = 6.3 Hz, 2H), 3.43 (q, *J* = 5.9 Hz, 2H), 3.09 (t, *J* = 6.1 Hz, 2H), 3.06 (t, *J* = 5.7 Hz, 1H), 2.76 (t, *J* = 7.3 Hz, 2H), 2.32 (s, 3H), 1.50 (s, 6H), 1.16 (t, *J* = 7.1 Hz, 3H).

^{13}**C NMR** (126 MHz, $CDCl_3$) δ (ppm) = 174.16, 167.15, 153.72, 143.61, 141.93, 140.78, 135.24, 133.82, 132.64, 129.69, 129.29, 127.15, 127.03, 126.32, 119.39, 117.89, 78.98, 61.28, 60.50, 53.18, 49.62, 41.26, 34.60, 25.18, 21.32, 13.90.

IR (ATR): $\tilde{\nu}$ (cm^{-1}) = 3005, 2988, 1730, 1641, 1539, 1506, 1450, 1276, 1262, 1234, 1151, 1089.

HRMS (ESI^+) calcd. for $C_{33}H_{41}N_2O_7S^+$ $[M+H]^+$: 609.2629, found: 609.2643.

Ethyl 2-(4-(2-(4-(3-((*N*-(2-(methoxyimino)ethyl)-4-methylphenyl)sulfonamido)prop-1-en-2-yl)benzamido)ethyl)phenoxy)-2-methylpropanoate (S22b)

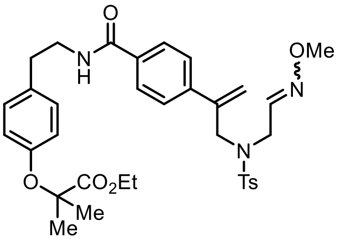

Prepared according to **General Procedure B (step B2)** using alcohol **S22a** (2.16 g, 3.55 mmol, 1.00 equiv.), CH_2Cl_2 (7.1 mL), Dess-Martin periodinane (2.26 g, 5.33 mmol, 1.50 equiv.), $NH_2OMe\cdot HCl$ (593 mg, 7.10 mmol, 2.00 equiv.), NaOAc (1.16 g, 14.20 mmol, 4.00 equiv.), EtOH (35.5 mL), CH_2Cl_2 (3.6 mL). Purification by flash column chromatography (pentane/ethyl acetate, 5:1 to 1:1) afforded oxime **S22b** as a colorless oil (1.26 g, 1.98 mmol, 56%, *E/Z* = 75:25).

^{1}**H NMR** (500 MHz, $CDCl_3$) δ (ppm) = 7.67-7.54 (m, 4H), 7.46-7.33 (m, 2H), 7.27-7.19 (m, 2H), 7.06-6.98 (m, 2H), 6.92 (t, *J* = 5.9, 0.75H), 6.78-6.67 (m, 3H), 6.22 (t, *J* = 4.2, 0.25H), 5.51-5.40 (m, 1H), 5.30-5.15 (m, 1H), 4.19-4.11 (m, 4H), 3.85-3.49 (m, 7H), 2.80 (t, *J* = 7.3 Hz, 2H), 2.35 (s, 3H), 1.55-1.47 (m, 6H), 1.19-1.13 (m, 3H).

^{13}**C NMR** (126 MHz, $CDCl_3$) δ (ppm) = 174.14, 166.96, 166.93, 153.85, 147.37, 144.79, 143.95, 143.80, 141.39, 141.20, 140.85, 140.41, 135.68, 135.00, 134.15, 134.01, 132.60, 129.89, 129.77, 129.33, 127.23, 127.20, 127.10, 127.00, 126.51, 126.46, 119.36, 118.68, 118.31, 78.98, 61.90, 61.53, 61.27, 53.00, 51.36, 45.52, 42.67, 41.24, 34.69, 25.24, 21.42, 21.40, 13.96.

IR (ATR): $\tilde{\nu}$ (cm^{-1}) = 3005, 1731, 1644, 1610, 1537, 1505, 1460, 1337, 1276, 1261, 1159, 1091.

HRMS (ESI$^+$) calcd. for $C_{34}H_{42}N_3O_7S^+$ [M+H]$^+$: 636.2738, found: 636.2740.

Methyl 2-(1-(4-(3-((*N*-(2-hydroxyethyl)-4-methylphenyl)sulfonamido)prop-1-en-2-yl)benzoyl)-5-methoxy-2-methyl-1*H*-indol-3-yl)acetate (S23a)

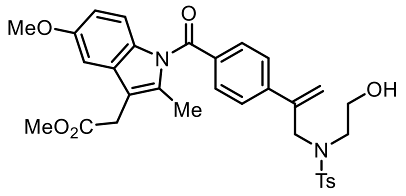

Prepared according to **General Procedure B (step B1)** using **S6a'** (2.66 g, 8.00 mmol, 1.00 equiv.), (4-(5-methoxy-3-(2-methoxy-2-oxoethyl)-2-methyl-1*H*-indole-1-carbonyl)phenyl)boronic acid[3] (3.66 g, 9.60 mmol, 1.20 equiv.), Na_2CO_3 (2.54 g, 24.0 mmol, 3.00 equiv.), $Pd(PPh_3)_4$ (277 mg, 0.24 mmol, 3 mol%), 1,4-dioxane/H_2O (36 mL/4 mL). Purification by flash column chromatography (pentane/ethyl acetate, 5:1 to 1:1 to 1:3 and CH_2Cl_2/ethyl acetate, 4:1 to 2:1) afforded alcohol **S23a** as a yellow oil (1.60 g, 2.71 mmol, 34%).

^{1}H NMR (500 MHz, $CDCl_3$) δ (ppm) = 7.66 (d, J = 8.2 Hz, 2H), 7.63 (d, J = 8.3 Hz, 2H), 7.55 (d, J = 8.4 Hz, 2H), 7.26 (d, J = 8.1 Hz, 2H), 6.95 (d, J = 2.6 Hz, 1H), 6.90 (d, J = 9.1 Hz, 1H), 6.65 (dd, J = 9.0, 2.6 Hz, 1H), 5.61 (s, 1H), 5.36 (s, 1H), 4.25 (s, 2H), 3.79 (s, 3H), 3.67 (s, 3H), 3.65 (s, 2H), 3.52 (t, J = 6.0 Hz, 2H), 3.16 (t, J = 5.8 Hz, 2H), 2.49 (s, 1H), 2.37 (s, 3H), 2.34 (s, 3H).

^{13}C NMR (126 MHz, $CDCl_3$) δ (ppm) = 171.36, 168.91, 155.89, 143.78, 142.22, 141.92, 135.83, 135.19, 134.87, 130.84, 130.49, 129.85, 129.78, 127.30, 126.70, 118.76, 114.97, 112.23, 111.48, 101.17, 60.80, 55.60, 53.38, 52.04, 49.90, 30.03, 21.40, 13.27.

IR (ATR): $\tilde{\nu}$ (cm^{-1}) = 3005, 2989, 1736, 1678, 1605, 1477, 1457, 1321, 1275, 1261, 1224, 1158.

HRMS (ESI$^+$) calcd. for $C_{32}H_{35}N_2O_7S^+$ [M+H]$^+$: 591.2159, found: 591.2146.

Methyl 2-(5-methoxy-1-(4-(3-((*N*-(2-(methoxyimino)ethyl)-4-methylphenyl)sulfonamido)prop-1-en-2-yl)benzoyl)-2-methyl-1*H*-indol-3-yl)acetate (S23b)

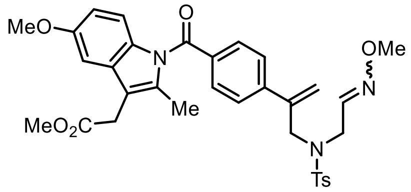

Prepared according to **General Procedure B (step B2)** using alcohol **S23a** (1.53 g, 2.59 mmol, 1.00 equiv.), CH_2Cl_2 (5.2 mL), Dess-Martin periodinane (1.65 g, 3.89 mmol, 1.50 equiv.), $NH_2OMe·HCl$ (433 mg, 5.18 mmol, 2.00 equiv.), NaOAc (850 mg, 10.36 mmol, 4.00 equiv.), EtOH

(25.9 mL), CH_2Cl_2 (2.6 mL). Purification by flash column chromatography (pentane/ethyl acetate, 2:1 to 1:2) afforded oxime **S23b** as a yellow oil (900 mg, 1.46 mmol, 56%, *E/Z* = 55:45).

^{1}H NMR (500 MHz, $CDCl_3$) δ (ppm) = 7.92-7.74 (m, 6H), 7.55-7.48 (m, 2H), 7.22 (t, *J* = 5.9 Hz, 0.55H), 7.20-7.14 (m, 2H), 6.92-6.86 (m, 1H), 6.55 (t, *J* = 4.1Hz, 0.45 H), 5.87 (s, 0.45H), 5.86 (s, 0.55H), 5.62 (s, 0.55H), 5.58 (s, 0.45H), 4.48 (s, 1.1H), 4.46 (s, 0.9H), 4.16-4.01 (m, 6H), 3.96-3.86 (m, 7H), 2.62 (s, 3H), 2.60 (s, 3H).

^{13}C NMR (126 MHz, $CDCl_3$) δ (ppm) = 171.30, 168.88, 168.84, 155.90, 147.45, 144.95, 144.01, 143.87, 142.39, 141.94, 141.33, 141.13, 135.86, 135.83, 135.72, 135.05, 135.01, 134.87, 130.85, 130.50, 130.49, 129.93, 129.89, 129.83, 129.81, 127.28, 126.83, 126.79, 119.45, 119.10, 114.97, 112.22, 112.20, 111.48, 101.19, 101.13, 61.98, 61.60, 55.58, 53.02, 52.02, 51.46, 45.67, 42.70, 30.05, 21.43, 13.28.

IR (ATR): $\tilde{\nu}$ (cm^{-1}) = 2989, 1736, 1679, 1605, 1477, 1456, 1353, 1317, 1276, 1261, 1224, 1160, 1067.

HRMS (ESI^+) calcd. for $C_{33}H_{36}N_3O_7S^+$ $[M+H]^+$: 618.2268, found: 618.2261.

***N*-(2-(cyclohex-1-en-1-yl)allyl)-*N*-(2-hydroxyethyl)-4-methylbenzenesulfonamide (S24a)**

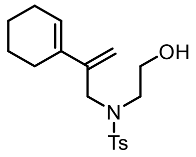

Prepared according to **General Procedure B (step B1)** using **S6a'** (2.66 g, 8.00 mmol, 1.00 equiv.), 1-cyclohexen-1-yl-boronic acid (1.21 g, 9.60 mmol, 1.20 equiv.), Na_2CO_3 (2.54 g, 24.0 mmol, 3.00 equiv.), $Pd(PPh_3)_4$ (277 mg, 0.24 mmol, 3 mol%), 1,4-dioxane/H_2O (36 mL/4 mL). Purification by flash column chromatography (pentane/ethyl acetate, 5:1 to 3:1 to 1:1 and CH_2Cl_2/ethyl acetate, 10:1 to 4:1) afforded alcohol **S24a** as a colorless oil (1.73 g, 5.16 mmol, 65%).

^{1}H NMR (500 MHz, $CDCl_3$) δ (ppm) = 7.68 (d, *J* = 8.3 Hz, 2H), 7.30 (d, *J* = 8.0 Hz, 2H), 6.11 (t, *J* = 4.1 Hz, 1H), 5.11 (s, 1H), 4.91 (s, 1H), 3.91 (s, 2H), 3.57 (app. q, *J* = 5.0 Hz, 2H), 3.10 (t, *J* = 5.5 Hz, 2H), 2.43 (s, 1H), 2.40 (s, 3H), 2.14-2.08 (m, 4H), 1.67-1.61 (m, 2H), 1.56-1.51 (m, 2H).

^{13}C NMR (126 MHz, $CDCl_3$) δ (ppm) = 143.67, 142.69, 135.01, 133.74, 129.80, 127.47, 127.15, 113.73, 61.29, 53.35, 50.14, 26.03, 25.85, 22.73, 22.00, 21.52.

IR (ATR): $\tilde{\nu}$ (cm^{-1}) = 2927, 2860, 1598, 1448, 1333, 1158, 1114, 1088, 1046, 1020.

HRMS (ESI^+) calcd. for $C_{18}H_{26}NO_3S^+$ $[M+H]^+$: 336.1628, found: 336.1616.

***N*-(2-(cyclohex-1-en-1-yl)allyl)-*N*-(2-(methoxyimino)ethyl)-4-methylbenzenesulfonamide (S24b)**

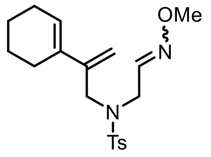

Prepared according to **General Procedure B (step B2)** using alcohol **S24a** (1.46 g, 4.36 mmol, 1.00 equiv.), CH_2Cl_2 (8.7 mL), Dess-Martin periodinane (2.77 g, 6.54 mmol, 1.50 equiv.), $NH_2OMe \cdot HCl$ (732 mg, 8.72 mmol, 2.00 equiv.), NaOAc (1.43 g, 17.44 mmol, 4.00 equiv.), EtOH (43.6 mL), CH_2Cl_2 (4.4 mL). Purification by flash column chromatography (pentane/ethyl acetate, 8:1 to 3:1) afforded oxime **S24b** as a colorless oil (1.00 g, 2.76 mmol, 63%, *E/Z* = 70:30).

^{1}H NMR (500 MHz, $CDCl_3$) δ (ppm) = 7.70-7.63 (m, 2H), 7.33-7.26 (m, 2H), 7.07 (t, *J* = 5.7 Hz, 0.7H), 6.48 (t, *J* = 3.9 Hz, 0.3H), 6.11 (t, *J* = 4.1 Hz, 0.3H), 6.00 (t, *J* = 3.4 Hz, 0.7H), 5.12 (s, 0.7H), 5.09 (s, 0.3H), 4.93 (s, 0.7H), 4.89 (s, 0.3H), 3.94 (s, 1.4H), 3.90 (s, 0.6H), 3.86 (d, *J* = 4.0 Hz, 0.6H), 3.78 (s, 0.9H), 3.76 (d, *J* = 5.8 Hz, 1.4H), 3.72 (s, 2.1H), 2.40 (s, 3H), 2.14-2.06 (m, 4H), 1.66-1.60 (m, 2H), 1.56-1.51 (m, 2H).

^{13}C NMR (126 MHz, $CDCl_3$) δ (ppm) = 148.53, 145.79, 143.76, 143.62, 141.90, 141.89, 135.98, 135.27, 133.76, 133.62, 129.89, 129.78, 127.42, 127.37, 127.29, 126.64, 114.32, 113.62, 61.99, 61.56, 52.64, 51.02, 45.58, 42.99, 25.96, 25.87, 25.81, 22.76, 22.71, 22.03, 21.53.

IR (ATR): $\tilde{\nu}$ (cm^{-1}) = 2929, 2860, 1636, 1598, 1448, 1340, 1159, 1091, 1031.

HRMS (ESI$^+$) calcd. for $C_{19}H_{27}N_2O_3S^+$ [M+H]$^+$: 363.1737, found: 363.1727.

***N*-(2-(cyclopent-1-en-1-yl)allyl)-*N*-(2-hydroxyethyl)-4-methylbenzenesulfonamide (S25a)**

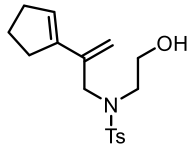

Prepared according to **General Procedure B (step B1)** using **S6a'** (2.66 g, 8.00 mmol, 1.00 equiv.), cyclopenten-1-ylboronic acid (1.08 g, 9.60 mmol, 1.20 equiv.), Na_2CO_3 (2.54 g, 24.0 mmol, 3.00 equiv.), $Pd(PPh_3)_4$ (277 mg, 0.24 mmol, 3 mol%), 1,4-dioxane/H_2O (36 mL/4 mL). Purification by flash column chromatography (pentane/ethyl acetate, 5:1 to 3:1 to 1:1 and CH_2Cl_2/ethyl acetate, 10:1 to 4:1) afforded alcohol **S25a** as a colorless oil (1.08 g, 3.36 mmol, 42%).

^{1}H NMR (500 MHz, $CDCl_3$) δ (ppm) = 7.69 (d, *J* = 8.4 Hz, 2H), 7.31 (d, *J* = 8.0 Hz, 2H), 6.13 (s, 1H), 5.05 (s, 1H), 5.02 (s, 1H), 3.94 (s, 2H), 3.58 (app. q, *J* = 5.5 Hz, 2H), 3.13 (t, *J* = 5.5 Hz, 2H), 2.45 (t, *J* = 7.6 Hz, 4H), 2.41 (s, 3H), 2.37 (t, *J* = 6.0 Hz, 1H), 1.87 (p, *J* = 7.6 Hz, 2H).

^{13}C NMR (126 MHz, $CDCl_3$) δ (ppm) = 143.73, 140.84, 138.93, 135.14, 129.86, 129.75, 127.45, 115.72, 61.39, 53.72, 50.56, 33.73, 33.02, 22.73, 21.56.

IR (ATR): $\tilde{\nu}$ (cm^{-1}) = 2936, 2845, 1630, 1597, 1447, 1333, 1306, 1088, 1038, 1020.

HRMS (ESI^+) calcd. for $C_{17}H_{24}NO_3S^+$ $[M+H]^+$: 322.1471, found: 322.1460.

***N*-(2-(cyclopent-1-en-1-yl)allyl)-*N*-(2-(methoxyimino)ethyl)-4-methylbenzenesulfonamide (S25b)**

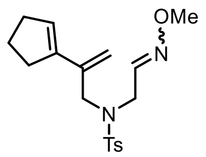

Prepared according to **General Procedure B (step B2)** using alcohol **S25a** (628 mg, 1.96 mmol, 1.00 equiv.), CH_2Cl_2 (4.0 mL), Dess-Martin periodinane (1.24 g, 2.92 mmol, 1.50 equiv.), $NH_2OMe \cdot HCl$ (330 mg, 3.93 mmol, 2.00 equiv.), NaOAc (643 mg, 7.84 mmol, 4.00 equiv.), EtOH (19.6 mL), CH_2Cl_2 (2.0 mL). Purification by flash column chromatography (pentane/ethyl acetate, 8:1 to 3:1) afforded oxime **S25b** as a colorless oil (348 mg, 1.00 mmol, 51%, *E/Z* = 70:30).

^{1}H NMR (400 MHz, $CDCl_3$) δ (ppm) = 7.70-7.65 (m, 2H), 7.33-7.26 (m, 2H), 7.05 (t, *J* = 5.8 Hz, 0.7H), 6.47 (t, *J* = 4.0 Hz, 0.3H), 6.13 (s, 0.3H), 6.01 (s, 0.7H), 5.06-4.98 (m, 2H), 3.96 (s, 1.4H), 3.92 (s, 0.6H), 3.88 (d, *J* = 4.0 Hz, 0.6H), 3.79 (d, *J* = 5.8 Hz, 1.4H), 3.77 (s, 0.9H), 3.70 (s, 2.1H), 2.45-2.38 (m, 7H), 1.90-1.80 (m, 2H).

^{13}C NMR (101 MHz, $CDCl_3$) δ (ppm) = 148.32, 145.49, 143.78, 143.64, 140.69, 140.57, 137.82, 136.06, 135.32, 129.91, 129.85, 129.79, 129.08, 127.33, 127.28, 116.28, 115.51, 61.97, 61.56, 52.88, 51.05, 45.74, 43.19, 33.69, 33.64, 32.89, 32.84, 22.69, 22.65, 21.50.

IR (ATR): $\tilde{\nu}$ (cm^{-1}) = 2940, 1597, 1443, 1345, 1276, 1161, 1092, 1033.

HRMS (ESI^+) calcd. for $C_{18}H_{25}N_2O_3S^+$ $[M+H]^+$: 349.1580, found: 349.1578.

***N*-(2-(cyclopent-1-en-1-yl)allyl)-*N*-(2-(2-(furan-2-carbonyl)hydrazineylidene)ethyl)-4-methylbenzenesulfonamide (S50b)**

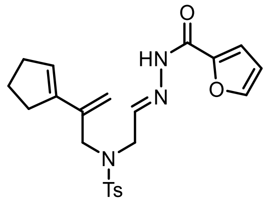

Prepared according to **General Procedure B (step B2)** using alcohol **S25a** (830 mg, 2.59 mmol, 1.00 equiv.), CH_2Cl_2 (5.2 mL), Dess-Martin periodinane (1.65 g, 3.89 mmol, 1.50 equiv.), 2-furoic acid hydrazide (390 mg, 3.10 mmol, 1.20 equiv.), EtOH (25.9 mL), CH_2Cl_2 (2.6 mL). Purification by flash column chromatography (pentane/ethyl acetate, 2:1 to 1:3) and recrystallization afforded oxime **S50b** as a white solid (460 mg, 1.08 mmol, 42%, *E*-isomer).

M.P.: 152-154 °C

^{1}H NMR (500 MHz, $CDCl_3$) δ (ppm) = 9.33 (s, 1H), 7.71 (d, *J* = 8.3 Hz, 2H), 7.45 (br. s, 2H), 7.33 (d, *J* = 7.9 Hz, 2H), 7.26 (br. s, 1H), 6.52 (dd, *J* = 3.6, 1.7 Hz, 1H), 6.01 (s, 1H), 5.05 (s, 1H), 5.01 (s, 1H), 3.97 (s, 2H), 3.90 (d, *J* = 5.1 Hz, 2H), 2.46-2.38 (m, 7H), 1.83 (p, *J* = 7.5 Hz, 2H).

^{13}C NMR (126 MHz, $CDCl_3$) δ (ppm) = 154.47, 147.95, 146.44, 144.68, 144.03, 140.86, 138.01, 135.05, 130.02, 129.36, 127.59, 116.42, 115.88, 112.56, 52.24, 48.98, 33.77, 33.03, 22.78, 21.66.

IR (ATR): $\tilde{\nu}$ (cm^{-1}) = 3258, 2933, 2848, 1660, 1590, 1571, 1542, 1473, 1325, 1292, 1196, 1156, 1090, 1042.

HRMS (ESI$^+$) calcd. for $C_{22}H_{26}N_3O_4S^+$ [M+H]$^+$: 428.1639, found: 428.1626.

1.8. General Procedure C for Cyclization Precursor Synthesis

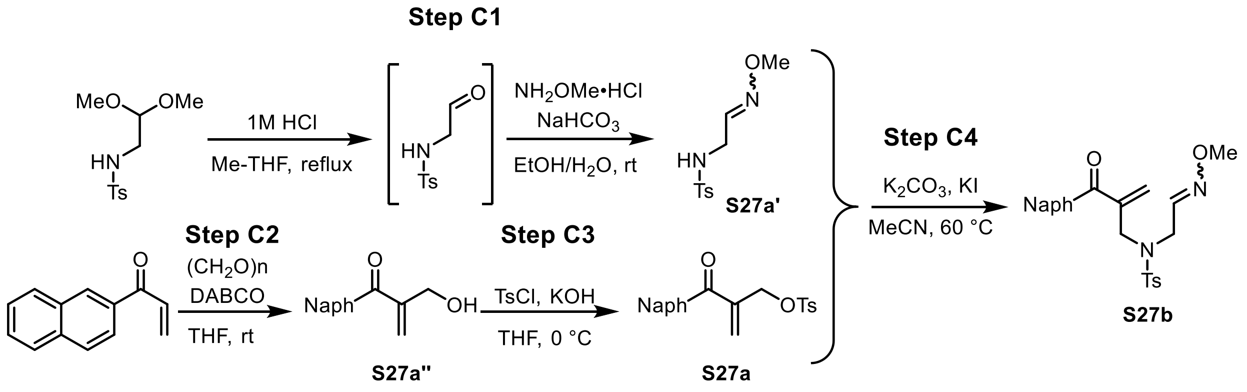

Step C1: In an oven-dried 1 L round-bottom flask was dissolved *N*-(2,2-dimethoxyethyl)-4-methylbenzenesulfonamide[5] (10.36 g, 40.0 mmol, 1.00 equiv.) in 2-MeTHF (200 mL) and 1 M aqueous HCl (200 mL) at room temperature. After stirring under reflux for 5 h, the reaction was allowed to cold down to room temperature and extracted with ethyl acetate (300 mL). The combined organic layers were washed with H_2O (200 mL) and brine (200 mL), dried over anhydrous $MgSO_4$, filtered and concentrated under reduced pressure. The crude product (colorless liquid) was used in the next step without further purification.

The crude aldehyde was then dissolved in ethanol (60 mL) and H_2O (40 mL) in a 250 mL round-bottom flask before $NaHCO_3$ (6.72 g, 80.0 mmol, 2.00 equiv.) and methoxyamine hydrochloride (5.04 g, 60.0 mmol, 1.50 equiv.) were added. The reaction was stirred at room temperature for 12 h. The reaction was quenched with sat. aq. $NaHCO_3$ solution (300 mL) and extracted with CH_2Cl_2 (3 × 100 mL). The combined organic layers were dried over anhydrous $MgSO_4$, filtered and concentrated under reduced pressure. The crude product was purified by flash column chromatography (pentane/ethyl acetate, 5:1 to 1:1) to afford **S27a'** as a white solid (7.60 g, 31.4 mmol, 79%, *E/Z*=55:45).

Step C2: In an oven-dried 100 mL round-bottom flask, 1-(naphthalen-2-yl)prop-2-en-1-one[6] (4.86 g, 26.7 mmol, 1.00 equiv.) was dissolved in anhydrous THF (26.7 mL). Paraformaldehyde (882 mg, 29.4 mmol, 1.10 equiv.) was then added, followed by the addition of DABCO (299 mg, 2.67 mmol, 0.10 equiv.). After being stirred at room temperature overnight, the reaction mixture was quenched with brine (100 mL). The product was extracted with ethyl acetate (3 × 50 mL). The combined organic layers were dried over anhydrous $MgSO_4$, filtered, and concentrated under reduced pressure. The crude product was purified by flash column chromatography (pentane/ethyl acetate, 5:1 to 1:1 and pentane/Et_2O, 1:1 to 1:4) to afford **S27a''** as a white solid (2.24 g, 10.57 mmol, 40%).

Note: The good quality of paraformaldehyde is crucial for the good outcome of the reaction. When using deteriorated paraformaldehyde, we observed a lower yield and with the formation of different side-products.

Step C3: An oven-dried 100 mL round-bottom flask was charged with **S27a''** (960 mg, 4.50 mmol, 1.00 equiv.) and TsCl (1.04 g, 5.46 mmol, 1.20 equiv.) in anhydrous THF (15.0 mL, 0.3 M) at 0 °C. KOH powder (756 mg, 13.5 mmol, 3.00 equiv.) was added and the reaction was stirred at 0 °C for 15 min until completion (judged by TLC). The reaction mixture was then quenched with H_2O (100 mL) and extracted with ethyl acetate (3 × 30 mL). The combined organic layers were dried over anhydrous $MgSO_4$ and concentrated under reduced pressure. The crude product was purified by flash column chromatography (pentane/ethyl acetate, 9:1 to 3:1) to afford **S27a** as a colorless oil (780 mg, 2.13 mmol, 47%).

Step C4: An oven-dried 100 mL round-bottom flask was charged with **S27a** (484 mg, 2.0 mmol, 1.00 equiv.) in anhydrous CH_3CN (10.0 mL, 0.2 M). Oxime **S27a'** (732 mg, 2.0 mmol, 1.00 equiv.) was added to the solution, followed by the addition of K_2CO_3 (552 mg, 4.0 mmol, 2.0 equiv.) and KI (33.2 mg, 0.2 mmol, 0.1 equiv.). The reaction mixture was then stirred at 60 °C for 12 h (judged by TLC). The reaction was filtered through anhydrous $MgSO_4$ and washed with CH_2Cl_2. The solvent was removed under reduced pressure and the crude product was purified by flash column chromatography (pentane/ethyl acetate, 6:1 to 2:1) to afford **S27b** as a colorless oil (860 mg, 1.97 mmol, 99%, *E/Z*=60:40).

***N*-(2-(methoxyimino)ethyl)-4-methylbenzenesulfonamide (S27a')**

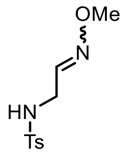

M.P.: 72-74 °C

^{1}H NMR (500 MHz, $CDCl_3$) δ (ppm) = 7.74-7.69 (m, 2H), 7.27 (d, *J* = 8.1 Hz, 2H), 7.19 (t, *J* = 5.0 Hz, 0.55H), 6.59 (t, *J* = 4.2 Hz, 0.45H), 5.69 (t, *J* = 6.4 Hz, 0.45H), 5.51 (t, *J* = 5.9 Hz, 0.55H), 3.77 (s, 1.35H), 3.75 (dd, *J* = 6.3, 4.2 Hz, 0.9H), 3.70 (s, 1.65H), 3.66 (app. t, *J* = 5.4 Hz, 1.1H), 2.38 (s, 3H).

^{13}C NMR (126 MHz, $CDCl_3$) δ (ppm) = 147.50, 144.91, 143.77, 143.72, 136.36, 136.32, 129.80, 129.75, 127.14, 127.08, 62.03, 61.66, 41.90, 38.66, 21.46, 21.44.

IR (ATR): $\tilde{\nu}$ (cm^{-1}) = 3279, 2940, 1598, 1433, 1324, 1306, 1276, 1156, 1092, 1037.

HRMS (ESI$^+$) calcd. for $C_{10}H_{15}N_2O_3S^+$ [M+H]$^+$: 243.0798, found: 243.0801.

2-(Hydroxymethyl)-1-(naphthalen-2-yl)prop-2-en-1-one (S27a'')

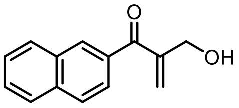

M.P.: 43-45 °C

^{1}H NMR (500 MHz, $CDCl_3$) δ (ppm) = 8.28 (d, *J* = 1.7 Hz, 1H), 7.94-7.83 (m, 4H), 7.60 (ddd, *J* = 8.2, 6.9, 1.4 Hz, 1H), 7.55 (ddd, *J* = 8.2, 6.8, 1.3 Hz, 1H), 6.20 (s, 1H), 5.87 (s, 1H), 4.58 (d, *J* = 6.2 Hz, 2H), 2.78 (t, *J* = 6.3 Hz, 1H).

^{13}C NMR (126 MHz, $CDCl_3$) δ (ppm) = 198.06, 146.58, 135.41, 134.61, 132.29, 131.38, 129.51, 128.48, 127.89, 127.16, 126.96, 125.26, 63.35.

IR (ATR): $\tilde{\nu}$ (cm^{-1}) = 3413, 1645, 1623, 1596, 1467, 1387, 1353, 1312, 1166, 1124, 1063, 1020.

HRMS (ESI$^+$) calcd. for $C_{14}H_{13}O_2^+$ [M+H]$^+$: 213.0910, found: 213.0913.

2-(2-Naphthoyl)allyl 4-methylbenzenesulfonate (S27a)

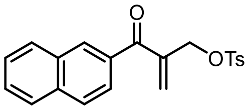

^{1}H NMR (500 MHz, $CDCl_3$) δ (ppm) = 8.19 (d, *J* = 1.7 Hz, 1H), 7.93-7.86 (m, 3H), 7.83 (d, *J* = 8.2 Hz, 2H), 7.78 (dd, *J* = 8.5, 1.7 Hz, 1H), 7.61 (ddd, *J* = 8.2, 6.8, 1.4 Hz, 1H), 7.56 (ddd, *J* = 8.1, 6.8, 1.3 Hz, 1H), 7.33 (d, *J* = 8.0 Hz, 2H), 6.28 (s, 1H), 5.97 (s, 1H), 4.98 (s, 2H), 2.42 (s, 3H).

^{13}C NMR (126 MHz, $CDCl_3$) δ (ppm) = 195.06, 145.15, 140.90, 135.47, 133.94, 132.85, 132.23, 131.43, 130.04, 129.53, 129.39, 128.63, 128.55, 128.15, 127.90, 127.05, 125.13, 68.67, 21.75.

IR (ATR): $\tilde{\nu}$ (cm^{-1}) = 3007, 1654, 1466, 1361, 1276, 1261, 1176.

HRMS (ESI$^+$) calcd. for $C_{21}H_{19}O_4S^+$ [M+H]$^+$: 367.0999, found: 367.1007.

***N*-(2-(2-naphthoyl)allyl)-*N*-(2-(methoxyimino)ethyl)-4-methylbenzenesulfonamide (S27b)**

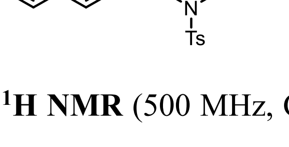

^{1}H NMR (500 MHz, $CDCl_3$) δ (ppm) = 8.24 (d, *J* = 1.7 Hz, 0.4H), 8.23 (d, *J* = 1.8 Hz, 0.6H), 7.96-7.87 (m, 3H), 7.83 (d, *J* = 1.7 Hz, 0.6H), 7.81 (d, *J* = 1.7 Hz, 0.4H), 7.76-7.71 (m, 2H), 7.63-7.54 (m,

2H), 7.33-7.28 (m, 2H), 7.25 (t, J = 6.0 Hz, 0.6H), 6.65 (t, J = 4.3 Hz, 0.4H), 6.28 (s, 0.4H), 6.27 (s, 0.6H), 5.97 (s, 0.4H), 5.96 (s, 0.6H), 4.23 (s, 0.8H), 4.21 (s, 1.2H), 4.16 (d, J = 4.4 Hz, 0.8H), 4.01 (d, J = 6.1 Hz, 1.2H), 3.83 (s, 1.2H), 3.74 (s, 1.8H), 2.41 (s, 1.2H), 2.40 (s, 1.8H).

^{13}C NMR (126 MHz, $CDCl_3$) δ (ppm) = 196.62, 196.52, 147.34, 145.12, 144.12, 144.02, 143.11, 142.84, 136.13, 135.97, 135.48, 135.44, 134.42, 134.32, 132.31, 131.49, 131.46, 130.11, 130.04, 129.59, 129.56, 128.67, 128.57, 128.52, 128.50, 128.44, 127.92, 127.90, 127.46, 127.01, 126.96, 125.37, 125.32, 62.28, 61.92, 50.01, 48.68, 47.98, 44.61, 21.65, 21.64.

IR (ATR): $\tilde{\nu}$ (cm^{-1}) = 2985, 1650, 1625, 1439, 1345, 1275, 1261, 1161, 1122, 1092, 1032.

HRMS (ESI$^+$) calcd. for $C_{24}H_{24}N_2O_4SNa^+$ [M+Na]$^+$: 459.1349, found: 459.1353.

2-Benzoylallyl 4-methylbenzenesulfonate (S28a)

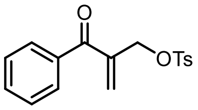

Prepared according to **General Procedure C (step C3)** using 2-(hydroxymethyl)-1-phenylprop-2-en-1-one[7] (780 mg, 4.80 mmol, 1.00 equiv.), THF (16.0 mL), TsCl (1.10 g, 5.77 mmol, 1.20 equiv.), KOH (810 mg, 14.46 mmol, 3.00 equiv.). Purification by flash column chromatography (pentane/ethyl acetate, 8:1 to 2:1) afforded **S28a** as a colorless oil (834 mg, 2.64 mmol, 55%).

^{1}H NMR (500 MHz, $CDCl_3$) δ (ppm) = 7.80 (d, J = 8.3 Hz, 2H), 7.68-7.64 (m, 2H), 7.57-7.52 (m, 1H), 7.44-7.40 (m, 2H), 7.34 (d, J = 8.0 Hz, 2H), 6.23 (s, 1H), 5.90 (s, 1H), 4.90 (s, 2H), 2.43 (s, 3H).

^{13}C NMR (126 MHz, $CDCl_3$) δ (ppm) = 195.11, 145.14, 140.67, 136.72, 132.83, 132.79, 130.02, 129.58, 129.50, 128.45, 128.11, 68.44, 21.72.

IR (ATR): $\tilde{\nu}$ (cm^{-1}) = 1654, 1596, 1447, 1360, 1276, 1260, 1188, 1174, 1096, 1033, 1004.

HRMS (ESI$^+$) calcd. for $C_{17}H_{16}O_4SNa^+$ [M+Na]$^+$: 339.0662, found: 339.0652.

***N*-(2-benzoylallyl)-*N*-(2-(methoxyimino)ethyl)-4-methylbenzenesulfonamide (S28b)**

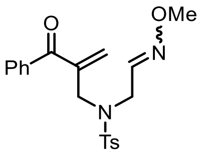

Prepared according to **General Procedure C (step C4)** using oxime **S27a'** (345 mg, 1.42 mmol, 1.00 equiv.), MeCN (5.7 mL, 0.25 M), **S28a** (450 mg, 1.42 mmol, 1.00 equiv.), K_2CO_3 (392 mg, 2.84 mmol, 2.00 equiv.), KI (24.0 mg, 0.14 mmol, 0.1 equiv.). Purification by flash column chromatography

(pentane/ethyl acetate, 5:1 to 2:1) afforded alcohol **S28b** as a colorless oil (420 mg, 1.09 mmol, 77%, *E/Z* = 60:40).

^{1}H NMR (500 MHz, $CDCl_3$) δ (ppm) = 7.74-7.69 (m, 4H), 7.57-7.53 (m, 1H), 7.46-7.41 (m, 2H), 7.34-7.39 (m, 2H), 7.20 (t, J = 6.0 Hz, 0.6H), 6.61 (t, J = 4.3 Hz, 0.4H), 6.24 (s, 0.4H), 6.22 (s, 0.6H), 5.90 (s, 0.4H), 5.89 (s, 0.6H), 4.16 (s, 0.8H), 4.14 (s, 1.2H), 4.10 (d, J = 4.4 Hz, 0.8H), 3.96 (d, J = 6.0 Hz, 1.2H), 3.81 (s, 1.2H), 3.73 (s, 1.8H), 2.42 (s, 3H).

^{13}C NMR (126 MHz, $CDCl_3$) δ (ppm) = 196.69, 196.60, 147.28, 145.04, 144.11, 144.01, 142.93, 142.68, 137.26, 137.14, 136.11, 135.94, 132.75, 132.64, 130.11, 130.03, 129.67, 129.65, 128.93, 128.89, 128.46, 128.39, 127.47, 127.45, 62.24, 61.88, 49.83, 48.50, 47.90, 44.57, 21.67, 21.65.

IR (ATR): $\tilde{\nu}$ (cm^{-1}) = 3006, 2989, 1654, 1597, 1447, 1343, 1276, 1261, 1160, 1092, 1032.

HRMS (ESI^+) calcd. for $C_{20}H_{23}N_2O_4S^+$ $[M+H]^+$: 387.1373, found: 387.1368.

1.9. General Procedure D for Cyclization Precursor Synthesis

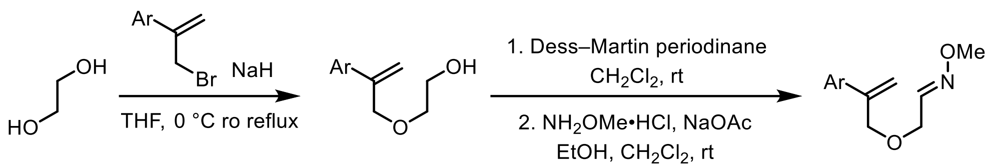

Step D1: An oven-dried round-bottom flask containing NaH (1.20 equiv.) was sealed and subjected to three N_2 evacuation/refill cycles before anhydrous THF (0.3 M) was added at 0 °C. Ethylene glycol (1.9 equiv.) was then added, and the reaction was stirred at 0 °C for 30 min before adding bromide (1.0 equiv.). The reaction mixture was allowed to warm up to room temperature and heated at reflux overnight. After cooling down, the reaction was quenched with sat. aq. NH_4Cl solution and extracted with CH_2Cl_2. The combined organic layers were dried over anhydrous $MgSO_4$, filtered and concentrated under reduced pressure. The crude product was purified by flash column chromatography.

Step D2: The alcohol (1.0 equiv.) was dissolved in CH_2Cl_2 (0.5 M) in an oven-dried round-bottom flask. Dess-Martin periodinane (1.5 equiv.) was then added and the reaction mixture was stirred in an open flask at room temperature for 0.5-1 h until completion (judged by TLC). The reaction was quenched with sat. aq. $NaHCO_3$ solution and extracted with CH_2Cl_2. The combined organic layers were dried over anhydrous $MgSO_4$, filtered, and concentrated under reduced pressure. The crude aldehyde was then dissolved in ethanol (0.1 M) and CH_2Cl_2 (1.0 M) in an oven-dried round-bottom flask before sodium acetate (4.0 equiv.) and methoxyamine hydrochloride (2.0 equiv.) were added. The reaction was stirred at room temperature for 1 h (judged by TLC). The reaction was quenched with sat. aq. $NaHCO_3$ solution and extracted with CH_2Cl_2. The combined organic layers were dried over anhydrous $MgSO_4$, filtered, concentrated under reduced pressure and purified by flash column chromatography.

Notes: In the final step (amination), the purpose of adding CH_2Cl_2 (1.0 M) is to wash the aldehyde stuck to the wall of the round-bottom flask into the reaction solution.

2-((2-(4-Chlorophenyl)allyl)oxy)ethan-1-ol (S31a)

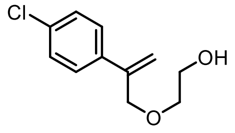

Prepared according to **General Procedure D (step D1)** using NaH (480 mg, 60 % purity, 12.0 mmol, 1.20 equiv.), THF (33.0 mL), ethylene glycol (1.06 mL, 19.0 mmol, 1.9 equiv.) and 1-(3-bromoprop-1-en-2-yl)-4-chlorobenzene[8] (2.30 g, 10.0 mmol, 1.00 equiv.). Purification by flash column

chromatography (pentane/ethyl acetate, 5:1 to 2:1) afforded alcohol **S31a** as a colorless oil (1.27 g, 5.99 mmol, 60%).

^{1}H NMR (500 MHz, $CDCl_3$) δ (ppm) = 7.39 (d, *J* = 8.6 Hz, 2H), 7.29 (d, *J* = 8.6 Hz, 2H), 5.52 (d, *J* = 1.1 Hz, 1H), 5.34 (d, *J* = 1.3 Hz, 1H), 4.38 (s, 2H), 3.72-3.66 (m, 2H), 3.59-3.53 (m, 2H), 2.20 (s, 1H).

^{13}C NMR (126 MHz, $CDCl_3$) δ (ppm) = 143.06, 136.99, 133.77, 128.64, 127.47, 115.39, 73.09, 71.26, 61.81.

IR (ATR): $\tilde{\nu}$ (cm^{-1}) = 3395, 2861, 1626, 1498, 1441, 1275, 1261, 1064, 1025.

HRMS (ESI^+) calcd. for $C_{11}H_{13}ClO_2Na^+$ $[M+Na]^+$: 235.0496, found: 235.0499.

2-((2-(4-Chlorophenyl)allyl)oxy)acetaldehyde *O*-methyl oxime (S31b)

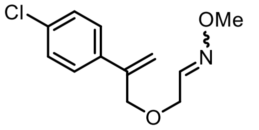

Prepared according to **General Procedure D (step D2)** using alcohol **S31a** (1.18 g, 5.57 mmol, 1.00 equiv.), CH_2Cl_2 (11.2 mL), Dess-Martin periodinane (3.54 g, 8.36 mmol, 1.50 equiv.), $NH_2OMe{\cdot}HCl$ (936 mg, 11.14 mmol, 2.00 equiv.), NaOAc (1.83 g, 22.28 mmol, 4.00 equiv.), EtOH (55.7 mL), CH_2Cl_2 (5.6 mL). Purification by flash column chromatography (pentane/ethyl acetate, 8:1 to 3:1) afforded oxime **S31b** as a colorless oil (784 mg, 3.28 mmol, 59%, *E/Z* = 60:40).

^{1}H NMR (500 MHz, $CDCl_3$) δ (ppm) = 7.42 (t, *J* = 5.8 Hz, 0.6H), 7.41-7.36 (m, 2H), 7.31 (d, *J* = 2.0 Hz, 1.2H), 7.30 (d, *J* = 2.0 Hz, 0.8H), 6.81 (t, *J* = 3.6 Hz, 0.4H), 5.55 (d, *J* = 1.0 Hz, 0.4H), 5.54 (d, *J* = 1.2 Hz, 0.6H), 5.36 (d, *J* = 1.4 Hz, 1H), 4.36 (s, 2H), 4.29 (d, *J* = 3.7 Hz, 0.8H), 4.10 (d, *J* = 5.7 Hz, 1.2H), 3.87 (s, 1.2H), 3.86 (s, 1.8H).

^{13}C NMR (126 MHz, $CDCl_3$) δ (ppm) = 149.91, 146.84, 142.74, 142.61, 136.99, 136.90, 133.88, 133.83, 128.69, 128.66, 127.52, 127.50, 115.89, 115.83, 73.21, 72.42, 66.67, 64.27, 62.18, 61.84.

IR (ATR): $\tilde{\nu}$ (cm^{-1}) = 3006, 1622, 1493, 1455, 1367, 1275, 1261, 1090, 1021.

HRMS (ESI^+) calcd. for $C_{12}H_{14}ClNO_2Na^+$ $[M+Na]^+$: 262.0605, found: 262.0609.

2-((2-Phenylallyl)oxy)ethan-1-ol (S32a)

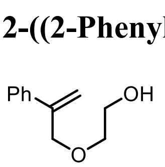

Prepared according to **General Procedure D (step D1)** using NaH (480 mg, 60 % purity, 12.0 mmol, 1.20 equiv.), THF (33.0 mL), ethylene glycol (1.06 mL, 19.0 mmol, 1.9 equiv.) and α-

(bromomethyl)styrene (1.96 g, 10.0 mmol, 1.00 equiv.). Purification by flash column chromatography (pentane/ethyl acetate, 5:1 to 2:1) afforded alcohol **S32a** as a colorless oil (1.0 g, 5.62 mmol, 56%).

^{1}H NMR (500 MHz, $CDCl_3$) δ (ppm) = 7.49-7.45 (m, 2H), 7.38-7.33 (m, 2H), 7.32-7.28 (m, 1H), 5.55 (d, J = 1.4 Hz, 1H), 5.35 (d, J = 1.4 Hz, 1H), 4.43 (s, 2H), 3.70 (app. q, J = 4.8 Hz, 2H), 3.62-3.56 (m, 2H), 2.34 (t, J = 5.8 Hz, 1H).

^{13}C NMR (126 MHz, $CDCl_3$) δ (ppm) = 144.15, 138.60, 128.47, 127.93, 126.10, 114.66, 73.13, 71.26, 61.79.

IR (ATR): $\tilde{\nu}$ (cm^{-1}) = 3397, 2864, 1631, 1495, 1446, 1276, 1261, 1065, 1027.

HRMS (ESI$^+$) calcd. for $C_{11}H_{14}O_2Na^+$ [M+Na]$^+$: 201.0886, found: 201.0881.

2-((2-Phenylallyl)oxy)acetaldehyde *O*-benzyl oxime (S32b)

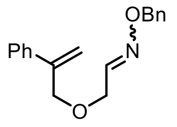

Prepared according to **General Procedure D (step D2)** using alcohol **S32a** (780 mg, 4.38 mmol, 1.00 equiv.), CH_2Cl_2 (8.8 mL), Dess-Martin periodinane (2.79 g, 6.57 mmol, 1.50 equiv.), $NH_2OBn{\cdot}HCl$ (1.40 g, 8.76 mmol, 2.00 equiv.), NaOAc (1.44 g, 17.52 mmol, 4.00 equiv.), EtOH (43.8 mL), CH_2Cl_2 (4.4 mL). Purification by flash column chromatography (pentane/ethyl acetate, 8:1 to 3:1) afforded oxime **S32b** as a colorless oil (510 mg, 1.81 mmol, 41%, *E/Z* = 50:50).

^{1}H NMR (500 MHz, $CDCl_3$) δ (ppm) = 7.60 (t, J = 5.8 Hz, 0.5H), 7.55-7.51 (m, 2H), 7.47-7.35 (m, 8H), 6.98 (t, J = 3.6 Hz, 0.5H), 5.63 (d, J = 1.2 Hz, 0.5H), 5.62 (d, J = 1.2 Hz, 0.5H), 5.42 (d, J = 1.3 Hz, 0.5H), 5.40 (d, J = 1.3 Hz, 0.5H), 5.20 (s, 2H), 4.47-4.44 (m, 3H), 4.20 (d, J = 5.8 Hz, 1H).

^{13}C NMR (126 MHz, $CDCl_3$) δ (ppm) = 150.70, 147.57, 143.72, 143.61, 138.55, 138.47, 137.69, 137.46, 128.46, 128.44, 128.29, 128.08, 127.97, 127.92, 126.10, 126.08, 115.05, 114.98, 76.30, 76.04, 73.19, 72.27, 66.59, 64.52.

IR (ATR): $\tilde{\nu}$ (cm^{-1}) = 3006, 1632, 1496, 1454, 1366, 1276, 1261, 1088, 1020.

HRMS (ESI$^+$) calcd. for $C_{18}H_{20}NO_2^+$ [M+H]$^+$: 282.1489, found: 282.1480.

1.10 General Procedure E for Cyclization Precursor Synthesis

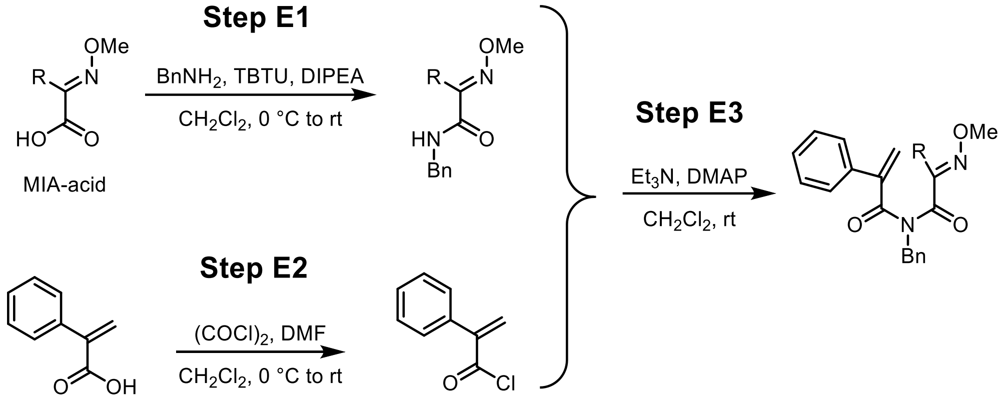

Step E1: In an oven-dried round-bottom flask MIA-acid (1.00 equiv.) and 2-(1H-benzotriazole-1-yl)-1,1,3,3-tetramethyluronium tetrafluoroborate (TBTU, 1.20 equiv.) were dissolved in anhydrous CH_2Cl_2 (0.2 M) at 0 °C. DIPEA (2.50 equiv.) was then added at 0 °C, followed by the addition of $BnNH_2$ (1.20 equiv.). The reaction mixture was allowed to warm up to room temperature and stirred for 2 h until completion (judged by TLC). The reaction was then quenched with 1 M aqueous HCl and extracted with CH_2Cl_2. The combined extracts were washed with brine, dried over anhydrous $MgSO_4$, and concentrated *in vacuo*. The crude product was purified by flash column chromatography to afford amide.

Step E2 and E3:

An oven-dried round-bottom flask containing 2-phenylacrylic acid (3.00 equiv.) was sealed and subjected to three N_2 evacuation/refill cycles before pre-sparged anhydrous CH_2Cl_2 (0.25 M with respect to 2-phenylacrylic acid) was added. Oxalyl chloride (4.00 equiv.) was then added at 0 °C, followed by the addition of DMF (3-5 drops). The reaction mixture was allowed to warm up to room temperature and stirred for 1 h. The reaction was then concentrated under reduced pressure to afford the crude acyl chloride and used directly for the next step with further purification.

In an oven-dried round-bottom flask the crude acyl chloride (3.00 equiv.) was dissolved in anhydrous CH_2Cl_2 (0.2 M with respect to amide/sulfonamide) at 0 °C. Et_3N (4.00 equiv.) was then added at 0 °C, followed by the addition of amide or sulfonamide (1.00 equiv.) and DMAP (0.50 equiv.). The reaction mixture was allowed to warm up to room temperature and stirred 4-12 h until completion of the reaction (TLC). The reaction was quenched with brine and extracted with CH_2Cl_2. The combined extracts were dried over anhydrous $MgSO_4$ and concentrated *in vacuo*. The crude product was purified by flash column chromatography to afford the desired product.

An alternative synthetic route:

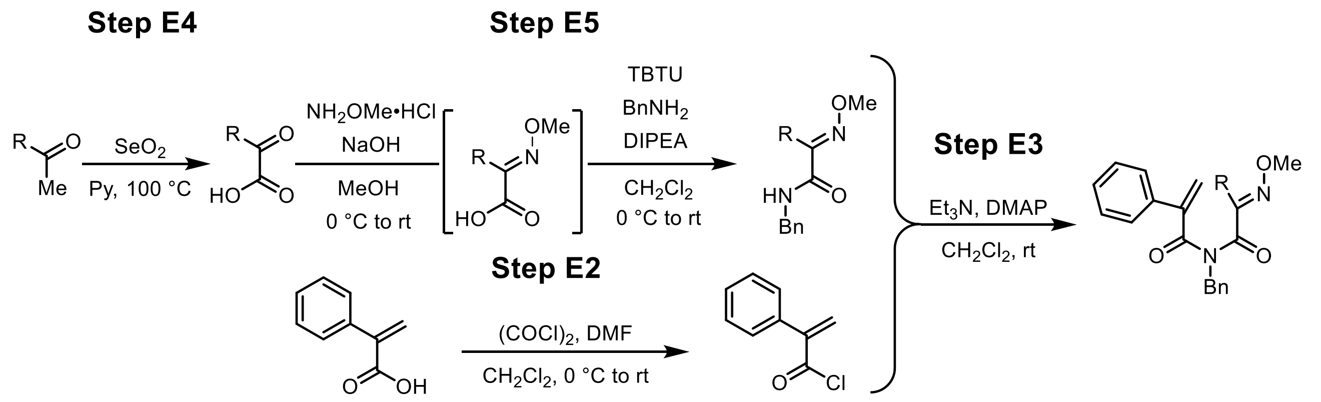

Step E4:

An oven-dried round-bottom flask containing aryl methyl ketone (1.00 equiv.) was sealed and subjected to three N_2 evacuation/refill cycles before anhydrous pyridine (1.0 M) was added. The SeO_2 (1.50 equiv.) was added to the solution. The reaction mixture was then stirred at 100 °C for 5-10 h until completion (judged by TLC). The solution containing precipitated selenium was filtered through anhydrous $MgSO_4$ and washed by ethyl acetate. The combined filtrate was treated with 1 M aqueous HCl to remove pyridine, the organic layer was separated, and the aqueous layer was extracted with ethyl acetate. The combined organic layers were dried through anhydrous $MgSO_4$ and concentrated under reduced pressure and the crude product was purified by flash column chromatography.

Step E5:

In an oven-dried round-bottom flask α-keto acids (1.00 equiv.) was dissolved in anhydrous MeOH (0.5 M) at 0 °C. NaOH (3.00 equiv.) and methoxyamine hydrochloride (2.00 equiv.) were then added at 0 °C and the reaction was stirred at room temperature for 2 h. The reaction was quenched with 1 M aqueous HCl and extracted with ethyl acetate. The combined organic layers were washed with brine, dried over anhydrous $MgSO_4$, filtered and concentrated under reduced pressure to afford the crude MIA-acid and used directly for the next step with further purification.

Note: ^{1}H NMR analysis indicated that the crude MIA-acid has a purity of >95%.

In an oven-dried round-bottom flask crude MIA-acid (1.00 equiv.) and 2-(1H-benzotriazole-1-yl)-1,1,3,3-tetramethyluronium tetrafluoroborate (TBTU, 1.20 equiv.) were dissolved in anhydrous CH_2Cl_2 (0.2 M) at 0 °C. DIPEA (2.50 equiv.) was then added at 0 °C, followed by the addition of $BnNH_2$ (1.20 equiv.). The reaction mixture was allowed to warm up to room temperature and stirred for 2 h until completion (judged by TLC). The reaction was then quenched with 1 M aqueous HCl and extracted with CH_2Cl_2. The combined extracts were washed with brine, dried over anhydrous $MgSO_4$,

and concentrated *in vacuo*. The crude product was purified by flash column chromatography to afford amide.

***N*-(2-(methoxyimino)ethyl)-2-phenyl-*N*-tosylacrylamide (S34b)**

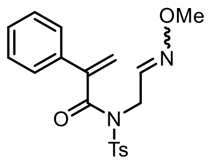

Prepared according to **General Procedure E (step E2 and E3)** using 2-phenylacrylic acid (1.33 g, 9.00 mmol, 3.00 equiv.), oxalyl chloride (1.03 mL, 12.00 mmol, 4.00 equiv.), CH_2Cl_2 (36.0 mL), DMF (3-5 drops), then CH_2Cl_2 (15.0 mL), Et_3N (1.67 mL, 12.00 mmol, 4.00 equiv.), sulfonamide **S27a'** (726 mg, 3.00 mmol, 1.00 equiv.) and DMAP (183 mg, 1.50 mmol, 0.50 equiv.). Purification by flash column chromatography (pentane/ethyl acetate, 6:1 to 2:1) afforded **S34b** as a pale-yellow oil (950 mg, 2.55 mmol, 85%, *E/Z* = 46:54).

^{1}H NMR (500 MHz, $CDCl_3$) δ (ppm) = 7.84 (d, J = 8.5 Hz, 0.92H), 7.80 (d, J = 8.4 Hz, 1.08H), 7.35-7.24 (m, 7H), 7.22 (t, J = 4.6 Hz, 0.46H), 6.46 (t, J = 3.9 Hz, 0.54H), 5.75 (s, 0.46H), 5.73 (s, 0.54H), 5.41 (s, 0.54H), 5.38 (s, 0.46H), 4.52 (d, J = 3.9 Hz, 1.08H), 4.47 (d, J = 4.6 Hz, 0.92H), 3.81 (s, 1.62H), 3.73 (s, 1.38H), 2.44 (s, 1.62H), 2.43 (s, 1.38H).

^{13}C NMR (126 MHz, $CDCl_3$) δ (ppm) = 169.97, 169.94, 146.90, 145.48, 145.24, 144.52, 144.36, 143.93, 135.73, 135.55, 134.53, 134.44, 129.70, 129.44, 129.29, 129.13, 129.10, 129.03, 128.98, 128.77, 126.15, 125.99, 118.26, 117.65, 62.29, 61.91, 45.53, 43.40, 21.79, 21.77.

IR (ATR): $\tilde{\nu}$ (cm^{-1}) = 1692, 1596, 1496, 1446, 1406, 1355, 1165, 1132, 1086, 1061, 1034, 912, 814.

HRMS (ESI$^+$) calcd. for $C_{19}H_{20}N_2O_4SNa^+$ [M+Na]$^+$: 395.1036, found: 395.1024.

***N*-benzyl-2-(methoxyimino)-*N*-(2-phenylallyl)acetamide (S35b)**

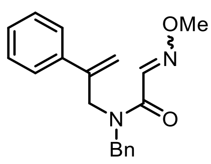

Prepared according to **General Procedure E (step E1)** using 2-(methoxyimino)acetic acid (MIA(H)-acid)[9] (103 mg, 1.00 mmol, 1.00 equiv.), TBTU (385 mg, 1.20 mmol, 1.20 equiv.), CH_2Cl_2 (5.0 mL), DIPEA (0.44 mL, 2.50 mmol, 2.50 equiv.), and *N*-benzyl-2-phenylprop-2-en-1-amine[10] (268 mg, 1.20 mmol, 1.20 equiv.). Purification by flash column chromatography (pentane/ethyl acetate, 8:1 to 3:1) afforded **S35b** as a pale-yellow oil (298 mg, 0.97 mmol, 97%, *E/Z* = 55:45).

^{1}H NMR (500 MHz, $CDCl_3$) δ (ppm) = 7.76 (s, 0.55H), 7.75 (s, 0.45H), 7.47-7.44 (m, 1H), 7.38-7.27 (m, 8H), 7.21-7.18 (m, 1H), 5.53 (d, *J* = 1.0 Hz, 0.45H), 5.52 (d, *J* = 1.6 Hz, 0.55H), 5.15 (app. t, *J* = 1.8 Hz, 0.55H), 5.12 (app. q, *J* = 1.4 Hz, 0.45H), 4.71 (s, 1.1H), 4.63 (s, 0.9H), 4.55 (s, 0.9H), 4.40 (app. t, *J* = 1.7 Hz, 1.1H), 3.97 (s, 1.65H), 3.92 (s, 1.35H).

^{13}C NMR (126 MHz, $CDCl_3$) δ (ppm) = 162.79, 162.42, 142.87, 142.63, 142.25, 142.12, 138.66, 138.23, 136.66, 136.20, 128.89, 128.77, 128.67, 128.53, 128.39, 128.19, 127.84, 127.76, 127.09, 126.34, 126.07, 115.45, 113.61, 62.94, 62.86, 50.32, 49.91, 48.93, 47.81.

IR (ATR): $\tilde{\nu}$ (cm^{-1}) = 1640, 1597, 1495, 1427, 1360, 1260, 1221, 1177, 1067, 1029, 908, 749.

HRMS (ESI^+) calcd. for $C_{19}H_{21}N_2O_2^+$ $[M+H]^+$: 309.1598, found: 309.1584.

(*E*)-*N*-benzyl-2-(methoxyimino)acetamide (S36a)

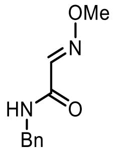

Prepared according to **General Procedure E (step E1)** using 2-(methoxyimino)acetic acid (MIA(H)-acid)[9] (1.03 g, 10.0 mmol, 1.00 equiv.), TBTU (3.85 g, 12.0 mmol, 1.20 equiv.), CH_2Cl_2 (50.0 mL), DIPEA (4.35 mL, 25.0 mmol, 2.50 equiv.), and $BnNH_2$ (1.30 mL, 12.0 mmol, 1.20 equiv.). Purification by flash column chromatography (CH_2Cl_2/ethyl acetate, 50:1 to 10:1) afforded **S36a** as a white solid (1.60 g, 8.33 mmol, 83%, *E*-isomer).

M.P.: 120-122 °C

^{1}H NMR (400 MHz, $CDCl_3$) δ (ppm) = 7.44 (s, 1H), 7.38-7.29 (m, 5H), 6.79 (br. s, 1H), 4.53 (d, *J* = 6.0 Hz, 2H), 3.95 (s, 3H).

^{13}C NMR (101 MHz, $CDCl_3$) δ (ppm) = 161.69, 142.94, 137.87, 128.92, 127.99, 127.82, 63.20, 43.32.

IR (ATR): $\tilde{\nu}$ (cm^{-1}) = 3224, 2941, 1682, 1647, 1599, 1563, 1455, 1432, 1362, 1245, 1081, 1025.

HRMS (ESI^+) calcd. for $C_{10}H_{13}N_2O_2^+$ $[M+H]^+$: 193.0972, found: 193.0963.

(*E*)-*N*-benzyl-*N*-(2-(methoxyimino)acetyl)-2-phenylacrylamide (S36b)

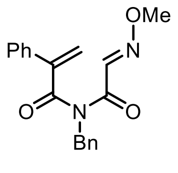

Prepared according to **General Procedure E (step E2 and E3)** using 2-phenylacrylic acid (1.33 g, 9.00 mmol, 3.00 equiv.), oxalyl chloride (1.03 mL, 12.0 mmol, 4.00 equiv.), CH_2Cl_2 (36.0 mL), DMF (3-5 drops), then CH_2Cl_2 (15.0 mL), Et_3N (1.67 mL, 12.00 mmol, 4.00 equiv.), amide **S36a** (576 mg, 3.00 mmol, 1.00 equiv.) and DMAP (183 mg, 1.50 mmol, 0.50 equiv.). Purification by flash column chromatography (pentane/ethyl acetate, 6:1 to 3:1) afforded **S36b** as a colorless oil (668 mg, 2.07 mmol, 69%, *E*-isomer).

^{1}H NMR (500 MHz, $CDCl_3$) δ (ppm) = 7.48 (s, 1H), 7.45-7.42 (m, 4H), 7.36-7.27 (m, 6H), 5.76 (s, 1H), 5.51 (s, 1H), 5.04 (s, 2H), 3.63 (s, 3H).

^{13}C NMR (126 MHz, $CDCl_3$) δ (ppm) = 172.16, 166.16, 147.55, 143.97, 136.60, 135.03, 129.08, 128.76, 128.63, 128.58, 127.81, 127.17, 121.19, 62.98, 48.64.

IR (ATR): $\tilde{\nu}$ (cm^{-1}) = 1752, 1694, 1661, 1497, 1448, 1385, 1350, 1276, 1261, 1180, 1033, 750.

HRMS (ESI^+) calcd. for $C_{19}H_{19}N_2O_3^+$ $[M+H]^+$: 323.1390, found: 323.1380.

***N*-benzyl-2-(methoxyimino)-*N*-(2-phenylallyl)propenamide (S37b)**

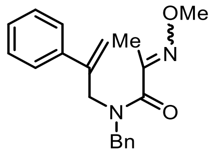

Prepared according to **General Procedure E (step E1)** using 2-(methoxyimino)propanoic acid (MIA(Me)-acid)[11] (234 mg, 2.00 mmol, 1.00 equiv.), TBTU (770 mg, 2.40 mmol, 1.20 equiv.), CH_2Cl_2 (10.0 mL), DIPEA (0.87 mL, 5.00 mmol, 2.50 equiv.), and N-benzyl-2-phenylprop-2-en-1-amine[10] (535 mg, 2.40 mmol, 1.20 equiv.). Purification by flash column chromatography (pentane/ethyl acetate, 8:1 to 3:1) afforded **S37b** as a colorless oil (580 mg, 1.80 mmol, 90%).

Note: The oxime product was isolated as a mixture of *E/Z* isomers in an approximate ratio of 55:45. However, the specific configuration could not be unambiguously assigned based on the available spectroscopic data.

^{1}H NMR (500 MHz, $CDCl_3$) δ (ppm) = 7.50-7.25 (m, 10H), 5.52 (s, 0.55H), 5.46 (s, 0.45H), 5.14 (s, 0.45H), 5.13 (s, 0.55H), 4.70 (s, 0.9H), 4.56 (s, 1.1H), 4.51 (s, 0.9H), 4.49 (s, 1.1H), 3.91 (s, 1.35H), 3.85 (s, 1.65H), 2.02 (s, 1.35H), 1.92 (s, 1.65H).

^{13}C NMR (126 MHz, $CDCl_3$) δ (ppm) = 167.03, 166.64, 151.21, 151.17, 143.72, 142.79, 139.05, 138.46, 136.81, 136.56, 128.70, 128.64, 128.57, 128.42, 128.26, 128.17, 128.09, 127.77, 127.71, 127.53, 126.41, 126.26, 114.89, 114.36, 62.26, 62.17, 51.35, 50.87, 48.02, 47.10, 12.91.

IR (ATR): $\tilde{\nu}$ (cm^{-1}) = 1638, 1495, 1452, 1362, 1276, 1196, 1166, 1043, 903, 747.

HRMS (ESI$^+$) calcd. for $C_{20}H_{23}N_2O_2^+$ [M+H]$^+$: 323.1754, found: 323.1743.

***N*-benzyl-*N*-(2-(methoxyimino)propanoyl)-2-phenylacrylamide (S38b)**

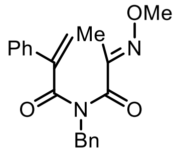

Prepared according to **General Procedure E (step E2 and E3)** using 2-phenylacrylic acid (1.33 g, 9.00 mmol, 3.00 equiv.), oxalyl chloride (1.03 mL, 12.0 mmol, 4.00 equiv.), CH_2Cl_2 (36.0 mL), DMF (3-5 drops), then CH_2Cl_2 (15.0 mL), Et_3N (1.67 mL, 12.00 mmol, 4.00 equiv.), (*E*)-*N*-benzyl-2-(methoxyimino)propanamide[12] (618 mg, 3.00 mmol, 1.00 equiv.) and DMAP (183 mg, 1.50 mmol, 0.50 equiv.). Purification by flash column chromatography (pentane/ethyl acetate, 6:1 to 2:1) afforded **S38b** as a colorless oil (554 mg, 1.65 mmol, 55%).

Note: The oxime product was isolated as a single stereoisomer. However, the *E/Z* configuration could not be unambiguously determined on the basis of the available spectroscopic data.

^{1}H NMR (500 MHz, $CDCl_3$) δ (ppm) = 7.53-7.47 (m, 4H), 7.36-7.26 (m, 6H), 5.70 (s, 1H), 5.33 (s, 1H), 5.06 (s, 2H), 3.44 (s, 3H), 1.82 (s, 3H).

^{13}C NMR (126 MHz, $CDCl_3$) δ (ppm) = 172.21, 169.73, 153.58, 148.25, 136.94, 135.25, 128.97, 128.83, 128.58, 128.46, 127.73, 127.18, 119.46, 62.54, 48.81, 12.05.

IR (ATR): $\tilde{\nu}$ (cm^{-1}) = 1707, 1658, 1608, 1496, 1431, 1385, 1348, 1224, 1152, 1076, 1048, 994, 734.
HRMS (ESI$^+$) calcd. for $C_{20}H_{21}N_2O_3^+$ [M+H]$^+$: 337.1547, found: 337.1541.

***N*-benzyl-2-(methoxyimino)-3-phenylpropanamide (S39a)**

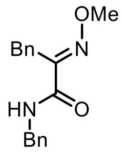

Prepared according to **General Procedure E (step E1)** using 2-(methoxyimino)-3-phenylpropanoic acid (MIA(Bn)-acid)[11] (1.93 g, 10.0 mmol, 1.00 equiv.), TBTU (3.85 g, 12.0 mmol, 1.20 equiv.), CH_2Cl_2 (50.0 mL), DIPEA (4.35 mL, 25.0 mmol, 2.50 equiv.), and $BnNH_2$ (1.30 mL, 12.0 mmol, 1.20 equiv.). Purification by flash column chromatography (pentane/ethyl acetate, 6:1 to 2:1) afforded **S39a** as a white solid (2.65 g, 9.40 mmol, 94%).

Note: The oxime product was isolated as a single stereoisomer. However, the *E/Z* configuration could not be unambiguously determined on the basis of the available spectroscopic data.

M.P.: 36-38 °C

^{1}H NMR (500 MHz, $CDCl_3$) δ (ppm) = 7.37-7.20 (m, 10H), 7.06 (t, *J* = 6.1 Hz, 1H), 4.52 (d, *J* = 6.0 Hz, 2H), 3.992 (s, 2H), 3.989 (s, 3H).

^{13}C NMR (126 MHz, $CDCl_3$) δ (ppm) = 162.64, 152.09, 138.12, 136.37, 129.38, 128.78, 128.51, 127.85, 127.59, 126.48, 63.02, 43.51, 29.96.

IR (ATR): $\tilde{\nu}$ (cm^{-1}) = 2937, 1664, 1517, 1494, 1454, 1212, 1041, 745, 696.

HRMS (ESI^{+}) calcd. for $C_{17}H_{19}N_2O_2^{+}$ $[M+H]^{+}$: 283.1441, found: 283.1427.

***N*-benzyl-*N*-(2-(methoxyimino)-3-phenylpropanoyl)-2-phenylacrylamide (S39b)**

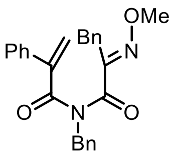

Prepared according to **General Procedure E (step E2 and E3)** using 2-phenylacrylic acid (1.33 g, 9.00 mmol, 3.00 equiv.), oxalyl chloride (1.03 mL, 12.00 mmol, 4.00 equiv.), CH_2Cl_2 (36.0 mL), DMF (3-5 drops), then CH_2Cl_2 (15.0 mL), Et_3N (1.67 mL, 12.00 mmol, 4.00 equiv.), amide **S39a** (846 mg, 3.00 mmol, 1.00 equiv.) and DMAP (183 mg, 1.50 mmol, 0.50 equiv.). Purification by flash column chromatography (pentane/ethyl acetate, 6:1 to 2:1) afforded **S39b** as a colorless oil (995 mg, 2.42 mmol, 81%).

Note: The oxime product was isolated as a single stereoisomer. However, the *E/Z* configuration could not be unambiguously determined on the basis of the available spectroscopic data.

^{1}H NMR (500 MHz, $CDCl_3$) δ (ppm) = 7.52-7.46 (m, 2H), 7.43-7.39 (m, 2H), 7.37-7.26 (m, 9H), 7.25-7.21 (m, 2H), 5.08 (s, 2H), 5.01 (s, 1H), 4.96 (s, 1H), 3.83 (s, 2H), 3.41 (s, 3H).

^{13}C NMR (126 MHz, $CDCl_3$) δ (ppm) = 172.32, 168.93, 153.21, 146.61, 137.01, 135.12, 134.32, 130.05, 128.74, 128.66, 128.53, 128.41, 128.17, 127.64, 127.03, 126.88, 120.20, 62.65, 49.26, 31.42.

IR (ATR): $\tilde{\nu}$ (cm^{-1}) = 2939, 1704, 1655, 1495, 1347, 1228, 1168, 1042, 920, 743, 696.

HRMS (ESI^{+}) calcd. for $C_{26}H_{25}N_2O_3^{+}$ $[M+H]^{+}$: 413.1860, found: 413.1845.

***N*-benzyl-2-cyclopropyl-2-(methoxyimino)acetamide (S40a)**

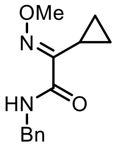

Prepared according to **General Procedure E (step E5)** using 2-cyclopropyl-2-oxoacetic acid (1.14 g, 10.00 mmol, 1.00 equiv.), $NH_2OMe\cdot HCl$ (1.68 g, 20.00 mmol, 2.00 equiv.), NaOH (1.20 g, 30.00 mmol, 3.00 equiv.) and MeOH (20.0 mL), then TBTU (3.85 g, 12.00 mmol, 1.20 equiv.), CH_2Cl_2 (50.0 mL), DIPEA (4.35 mL, 25.00 mmol, 2.50 equiv.), and $BnNH_2$ (1.31 mL, 12.00 mmol, 1.20 equiv.). Purification by flash column chromatography (pentane/ethyl acetate, 6:1 to 2:1) afforded **S40a** as a white solid (1.07 g, 4.61 mmol, 46%).

Note: The oxime product was isolated as a single stereoisomer. However, the *E/Z* configuration could not be unambiguously determined on the basis of the available spectroscopic data.

M.P.: 51-53 °C

^{1}H NMR (500 MHz, $CDCl_3$) δ (ppm) = 7.37 – 7.25 (m, 5H), 7.04 (s, 1H), 4.45 (d, *J* = 6.1 Hz, 2H), 3.92 (s, 3H), 2.21 (tt, *J* = 8.8, 5.5 Hz, 1H), 1.56 – 1.51 (m, 2H), 0.92 – 0.84 (m, 2H).

^{13}C NMR (126 MHz, $CDCl_3$) δ (ppm) = 162.56, 153.65, 138.22, 128.75, 127.77, 127.52, 62.75, 43.10, 8.56, 7.18.

IR (ATR): $\tilde{\nu}$ (cm^{-1}) = 1662, 1513, 1455, 1276, 1261, 1047.

HRMS (ESI$^+$) calcd. for $C_{13}H_{17}N_2O_2^+$ [M+H]$^+$: 233.1285, found: 233.1277.

***N*-benzyl-*N*-(2-cyclopropyl-2-(methoxyimino)acetyl)-2-phenylacrylamide (S40b)**

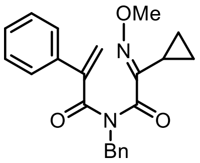

Prepared according to **General Procedure E (step E2 and E3)** using 2-phenylacrylic acid (1.33 g, 9.00 mmol, 3.00 equiv.), oxalyl chloride (1.03 mL, 12.00 mmol, 4.00 equiv.), CH_2Cl_2 (36.0 mL), DMF (3-5 drops), then CH_2Cl_2 (15.0 mL), Et_3N (1.67 mL, 12.00 mmol, 4.00 equiv.), amide **S40a** (696 mg, 3.00 mmol, 1.00 equiv.) and DMAP (183 mg, 1.50 mmol, 0.50 equiv.). Purification by flash column chromatography (pentane/ethyl acetate, 6:1 to 3:1 and pentane/CH_2Cl_2, 4:1 to 0:1) afforded **S40b** as a colorless oil (0.98 g, 2.71 mmol, 90%).

Note: The oxime product was isolated as a single stereoisomer. However, the *E/Z* configuration could not be unambiguously determined on the basis of the available spectroscopic data.

^{1}H NMR (500 MHz, $CDCl_3$) δ (ppm) = 7.63 – 7.60 (m, 2H), 7.46 – 7.43 (m, 2H), 7.39 – 7.31 (m, 5H), 7.30 – 7.26 (m, 1H), 5.74 (s, 1H), 5.32 (s, 1H), 5.02 (s, 2H), 3.40 (s, 3H), 1.94 (tt, *J* = 8.7, 5.4 Hz, 1H), 1.15 (dt, *J* = 6.3, 3.2 Hz, 2H), 0.90 – 0.80 (m, 2H).

^{13}C NMR (126 MHz, $CDCl_3$) δ (ppm) = 172.34, 167.41, 156.44, 147.43, 136.95, 135.15, 128.82, 128.58, 128.54, 128.36, 127.63, 127.01, 119.64, 62.32, 48.23, 9.15, 6.03.

IR (ATR): $\tilde{\nu}$ (cm^{-1}) = 1707, 1659, 1496, 1427, 1386, 1346, 1276, 1261, 1235, 1165.

HRMS (ESI^+) calcd. for $C_{22}H_{23}N_2O_3^+$ $[M+H]^+$: 363.1703, found: 363.1694.

***N*-benzyl-2-(methoxyimino)-2-phenylacetamide (S41a)**

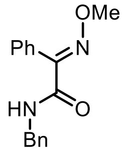

Prepared according to **General Procedure E (step E1)** using 2-(methoxyimino)-2-phenylacetic acid (MIA(Ph)-acid)[13] (1.79 g, 10.0 mmol, 1.00 equiv.), TBTU (3.85 g, 12.0 mmol, 1.20 equiv.), CH_2Cl_2 (50.0 mL), DIPEA (4.35 mL, 25.0 mmol, 2.50 equiv.), and $BnNH_2$ (1.30 mL, 12.0 mmol, 1.20 equiv.). Purification by flash column chromatography (pentane/ethyl acetate, 6:1 to 2:1) afforded **S41a** as a colorless oil (1.76 g, 6.57 mmol, 66%).

Note: The oxime product was isolated as a single stereoisomer. However, the *E/Z* configuration could not be unambiguously determined on the basis of the available spectroscopic data.

^{1}H NMR (500 MHz, $CDCl_3$) δ (ppm) = 7.66-7.63 (m, 2H), 7.40-7.28 (m, 8H), 6.35 (t, *J* = 5.9 Hz, 1H), 4.62 (d, *J* = 5.9 Hz, 2H), 4.01 (s, 3H).

^{13}C NMR (126 MHz, $CDCl_3$) δ (ppm) = 163.09, 152.76, 137.58, 131.36, 130.19, 128.81, 128.71, 127.87, 127.74, 126.84, 62.96, 43.48.

IR (ATR): $\tilde{\nu}$ (cm^{-1}) = 3274, 1646, 1530, 1497, 1455, 1445, 1259, 1185, 1048, 1029, 968, 879, 729.

HRMS (ESI^+) calcd. for $C_{16}H_{17}N_2O_2^+$ $[M+H]^+$: 269.1285, found: 269.1272.

***N*-benzyl-*N*-(2-(methoxyimino)-2-phenylacetyl)-2-phenylacrylamide (S41b)**

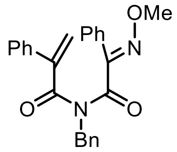

Prepared according to **General Procedure E (step E2 and E3)** using 2-phenylacrylic acid (1.33 g, 9.00 mmol, 3.00 equiv.), oxalyl chloride (1.03 mL, 12.00 mmol, 4.00 equiv.), CH_2Cl_2 (36.0 mL), DMF (3-5 drops), then CH_2Cl_2 (15.0 mL), Et_3N (1.67 mL, 12.00 mmol, 4.00 equiv.), amide **S41a** (804 mg, 3.00 mmol, 1.00 equiv.) and DMAP (183 mg, 1.50 mmol, 0.50 equiv.). Purification by flash column chromatography (pentane/ethyl acetate, 6:1 to 2:1) afforded **S41b** as a colorless oil (1.02 g, 2.56 mmol, 85%).

Note: The oxime product was isolated as a single stereoisomer. However, the *E/Z* configuration could not be unambiguously determined on the basis of the available spectroscopic data.

^{1}H NMR (500 MHz, $CDCl_3$) δ (ppm) = 7.53-7.27 (m, 15H), 5.51 (s, 1H), 5.31 (s, 1H), 5.05 (s, 2H), 3.74 (s, 3H).

^{13}C NMR (126 MHz, $CDCl_3$) δ (ppm) = 171.96, 165.99, 151.73, 143.42, 136.74, 134.52, 131.39, 130.14, 128.93, 128.71, 128.66, 128.63, 128.36, 127.76, 126.37, 126.27, 121.27, 62.75, 47.16.

IR (ATR): $\tilde{\nu}$ (cm^{-1}) = 1688, 1657, 1496, 1445, 1348, 1312, 1277, 1249, 1169, 1046, 1029, 946, 910, 880, 750, 727.

HRMS (ESI$^+$) calcd. for $C_{25}H_{23}N_2O_3^+$ [M+H]$^+$: 399.1703, found: 399.1690.

1-(4-(5-(*p*-Tolyl)-3-(trifluoromethyl)-1*H*-pyrazol-1-yl)phenyl)ethan-1-one (S42a')

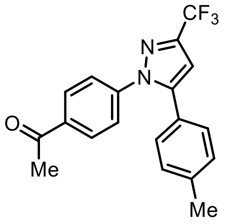

A 250 mL oven-dried round-bottom flask charged with 1-(4-bromophenyl)-5-(*p*-tolyl)-3-(trifluoromethyl)-1*H*-pyrazole [14] (11.4 g, 30.00 mmol, 1.00 equiv.) was sealed and subjected to three cycles of evacuation and nitrogen backfilling. Anhydrous THF (100.0 mL, 0.3 M) was then added, and the reaction mixture was cooled to –78 °C. *n*-BuLi (13.2 mL, 2.5 M in hexane, 33.00 mmol, 1.10 equiv.) was added dropwise at –78 °C, and the mixture was stirred at this temperature for 10 minutes. *N*-methoxy-*N*-methylacetamide (3.98 mL, 39.00 mmol, 1.30 equiv.) was subsequently added at –78 °C, and the reaction was allowed to warm to room temperature over 1 h. The reaction was then quenched with brine and extracted with ethyl acetate. The combined organic layers were dried over anhydrous $MgSO_4$, filtered, and concentrated under reduced pressure. The crude product was purified by flash column chromatography (pentane/ethyl acetate, 9:1 to 4:1) to afford the **S42a'** as a white solid (7.51 g, 21.83 mmol, 73%).

M.P.: 82-84 °C

^{1}H NMR (500 MHz, $CDCl_3$) δ (ppm) = 7.94 (d, J = 8.6 Hz, 2H), 7.42 (d, J = 8.6 Hz, 2H), 7.16 (d, J = 7.8 Hz, 2H), 7.11 (d, J = 8.3 Hz, 2H), 6.73 (s, 1H), 2.60 (s, 3H), 2.37 (s, 3H).

^{13}C NMR (126 MHz, $CDCl_3$) δ (ppm) = 197.02, 145.23, 143.98 (q, J = 38.4 Hz), 142.94, 139.68, 136.47, 129.74, 129.33, 128.85, 126.12, 125.22, 121.28 (q, J = 269.0 Hz), 106.25 (q, J = 2.1 Hz), 26.79, 21.43.

^{19}F NMR (471 MHz, $CDCl_3$) δ (ppm) = -62.35 (s).

IR (ATR): $\tilde{\nu}$ (cm^{-1}) = 1686, 1603, 1472, 1413, 1374, 1264, 1235, 1161, 1132, 1097.

HRMS (ESI^+) calcd. for $C_{19}H_{16}F_3N_2O^+$ $[M+H]^+$: 345.1209, found: 345.1213.

2-Oxo-2-(4-(5-(*p*-tolyl)-3-(trifluoromethyl)-1*H*-pyrazol-1-yl)phenyl)acetic acid (S42a")

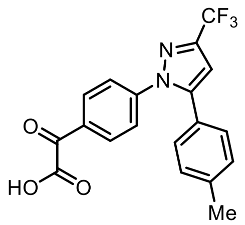

Prepared according to **General Procedure E (step E4)** using **S42a'** (4.51 g, 13.10 mmol, 1.00 equiv.), SeO_2 (2.18 g, 19.64 mmol, 1.50 equiv.) and pyridine (13.1 mL). Purification by flash column chromatography (pentane/ethyl acetate, 10:1 to 1:2) afforded **S42a"** as a white solid (2.85 g, 7.62 mmol, 58%).

M.P.: 148-150 °C

^{1}H NMR (500 MHz, $CDCl_3$) δ (ppm) = 8.97 (s, 1H), 8.27 (d, J = 8.7 Hz, 2H), 7.49 (d, J = 8.8 Hz, 2H), 7.18 (d, J = 7.9 Hz, 2H), 7.12 (d, J = 8.2 Hz, 2H), 6.76 (s, 1H), 2.38 (s, 3H).

^{13}C NMR (126 MHz, $CDCl_3$) δ (ppm) = 183.63, 162.11, 145.66, 144.50, 144.38 (q, J = 38.8 Hz), 140.08, 132.22, 131.18, 129.91, 128.87, 125.77, 125.30, 121.09 (q, J = 269.4 Hz), 106.72, 21.45.

^{19}F NMR (471 MHz, $CDCl_3$) δ (ppm) = -62.43 (s).

IR (ATR): $\tilde{\nu}$ (cm^{-1}) = 2926, 1737, 1691, 1603, 1473, 1374, 1236, 1164, 1136.

HRMS (ESI^+) calcd. for $C_{19}H_{14}F_3N_2O_3^+$ $[M+H]^+$: 375.0951, found: 375.0957.

***N*-Benzyl-2-(methoxyimino)-2-(4-(5-(*p*-tolyl)-3-(trifluoromethyl)-1*H*-pyrazol-1-yl)phenyl)acetamide (S42a)**

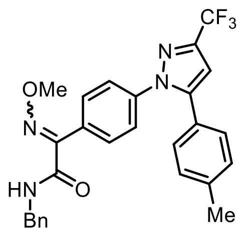

Prepared according to **General Procedure E (step E5)** using **S42a”** (561 mg, 1.50 mmol, 1.00 equiv.), $NH_2OMe{\cdot}HCl$ (252 mg, 3.00 mmol, 2.00 equiv.), NaOH (180 mg, 4.50 mmol, 3.00 equiv.) and MeOH (3.0 mL), then TBTU (578 mg, 1.80 mmol, 1.20 equiv.), CH_2Cl_2 (7.5 mL), DIPEA (0.65 mL, 3.75 mmol, 2.50 equiv.), and $BnNH_2$ (0.20 mL, 1.83 mmol, 1.20 equiv.). Purification by flash column chromatography (pentane/ethyl acetate, 6:1 to 2:1) afforded **S42a** as a colorless oil (280 mg, 0.57 mmol, 38%).

Note: The oxime product was isolated as a mixture of *E/Z* isomers in an approximate ratio of 70:30. However, the specific configuration could not be unambiguously assigned based on the available spectroscopic data.

^{1}H NMR (500 MHz, $CDCl_3$) δ (ppm) = 7.61 (d, J = 8.7 Hz, 1.4H), 7.51 (d, J = 8.6 Hz, 0.6H), 7.37 – 7.25 (m, 7H), 7.18 – 7.09 (m, 4.3H), 6.76-6.70 (m, 1.7H), 4.58 (d, J = 5.9 Hz, 1.4H), 4.53 (d, J = 6.0 Hz, 0.6H), 3.98 (s, 2.1H), 3.96 (s, 0.9H), 2.36 (s, 3H).

^{13}C NMR (126 MHz, $CDCl_3$) δ (ppm) = 162.62, 162.47, 151.48, 148.80, 144.97, 144.91, 143.43 (q, J = 38.5 Hz),143.38 (q, J = 38.5 Hz), 140.34, 139.87, 139.41, 139.35, 137.97, 137.46, 131.19, 130.90, 129.59, 128.78, 128.73, 128.72, 128.45, 127.85, 127.70, 127.66, 127.64, 127.40, 126.14, 126.03, 125.25, 124.21, 121.35 (q, J = 268.9 Hz), 121.32 (q, J = 269.2 Hz), 105.79, 105.77, 63.44, 63.01, 43.58, 43.31, 21.29.

^{19}F NMR (471 MHz, $CDCl_3$) δ (ppm) = -62.12 (s), -62.14 (s).

IR (ATR): $\tilde{\nu}$ (cm^{-1}) = 3288, 1652, 1509, 1472, 1376, 1271, 1236, 1161, 1132, 1050.

HRMS (ESI$^+$) calcd. for $C_{27}H_{24}F_3N_4O_2^+$ [M+H]$^+$: 493.1846, found: 493.1855.

***N*-benzyl-*N*-(2-(methoxyimino)-2-(4-(5-(*p*-tolyl)-3-(trifluoromethyl)-1*H*-pyrazol-1-yl)phenyl)acetyl)-2-phenylacrylamide (S42b)**

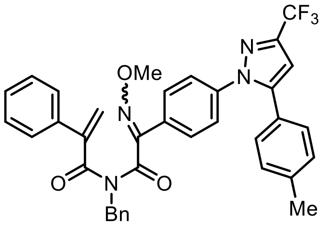

Prepared according to **General Procedure E (step E2 and E3)** using 2-phenylacrylic acid (434 mg, 2.93 mmol, 3.00 equiv.), oxalyl chloride (0.33 mL, 3.92 mmol, 4.00 equiv.), CH_2Cl_2 (11.7 mL), DMF (3-5 drops), then CH_2Cl_2 (5.0 mL), Et_3N (0.55 mL, 3.92 mmol, 4.00 equiv.), amide **S42a** (480 mg, 0.98 mmol, 1.00 equiv.) and DMAP (60 mg, 0.49 mmol, 0.50 equiv.). Purification by flash column chromatography (pentane/ethyl acetate, 6:1 to 3:1) afforded **S42b** as a colorless oil (530 mg, 0.85 mmol, 87%).

Note: The oxime product was isolated as a mixture of *E/Z* isomers in an approximate ratio of 75:25. However, the specific configuration could not be unambiguously assigned based on the available spectroscopic data.

^{1}H NMR (500 MHz, $CDCl_3$) δ (ppm) = 7.59 – 7.27 (m, 14H), 7.18 – 7.09 (m, 4H), 6.73 (s, 0.75H), 6.73 (s, 0.25H), 5.57 (s, 0.25H), 5.50 (s, 0.75H), 5.33 – 4.97 (m, 3H), 3.72 (s, 2.25H), 3.51 (s, 0.75H), 2.37 (s, 3H).

^{13}C NMR (126 MHz, $CDCl_3$) δ (ppm) = 172.28, 171.62, 168.10, 165.43, 150.43, 150.38, 146.76, 144.95, 144.94, 143.48 (q, J = 38.5 Hz), 143.42 (q, J = 38.2 Hz), 140.29, 140.19, 139.36, 139.34, 136.72, 136.45, 134.65, 134.10, 131.26, 130.23, 129.51, 128.95, 128.92, 128.77, 128.68, 128.55, 128.51, 128.39, 128.22, 127.70, 127.67, 126.79, 126.75, 126.09, 126.01, 125.28, 124.44, 121.30 (d, J = 268.9 Hz), 121.28 (q, J = 268.9 Hz), 121.14, 119.91, 105.83, 105.73, 63.11, 62.76, 48.59, 47.02, 21.20.

^{19}F NMR (471 MHz, $CDCl_3$) δ (ppm) = -62.22 (s), -62.27 (s).

IR (ATR): $\tilde{\nu}$ (cm^{-1}) = 1690, 1662, 1472, 1376, 1350, 1237, 1165, 1133, 1098, 1048.

HRMS (ESI^+) calcd. for $C_{36}H_{30}F_3N_4O_3^+$ $[M+H]^+$: 623.2265, found: 623.2271.

2-(6-Methoxypyridin-3-yl)-2-oxoacetic acid (S43a')

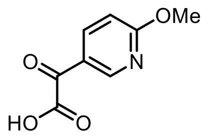

Prepared according to **General Procedure E (step E4)** using 5-acetyl-2-methoxypyridine (4.53 g, 30.00 mmol, 1.00 equiv.), SeO_2 (5.00 g, 45.00 mmol, 1.50 equiv.) and pyridine (30.0 mL). Purification by recrystallization (Et_2O) afforded **S43a'** as a white solid (4.20 g, 23.2 mmol, 77%).

Note: This α-keto acid is relatively water-soluble; thus, extraction from its 1 M aqueous HCl aqueous solution required eight successive portions of ethyl acetate, with a total volume of approximately 1 L, to achieve efficient transfer into the organic phase.

M.P.: 151-153 °C

^{1}H NMR (500 MHz, DMSO) δ (ppm) = 8.81 (dd, J = 2.5, 0.7 Hz, 1H), 8.21 (dd, J = 8.8, 2.5 Hz, 1H), 7.00 (dd, J = 8.8, 0.7 Hz, 1H), 3.97 (s, 3H).

^{13}C NMR (126 MHz, DMSO) δ (ppm) = 185.91, 167.04, 165.01, 151.26, 139.61, 122.62, 111.45, 54.28.

IR (ATR): $\tilde{\nu}$ (cm^{-1}) = 1723, 1674, 1608, 1557, 1501, 1388, 1304, 1276, 1217, 1128.

HRMS (ESI$^+$) calcd. for $C_8H_8NO_4^+$ [M+H]$^+$: 182.0448, found: 182.0450.

***N*-benzyl-2-(methoxyimino)-2-(6-methoxypyridin-3-yl)acetamide (S43a)**

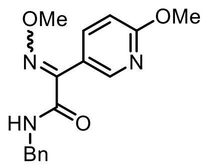

Prepared according to **General Procedure E (step E5)** using **S43a’** (1.09 g, 6.00 mmol, 1.00 equiv.), $NH_2OMe{\cdot}HCl$ (1.01 g, 12.00 mmol, 2.00 equiv.), NaOH (720 mg, 18.00 mmol, 3.00 equiv.) and MeOH (12.0 mL), then TBTU (2.31 g, 7.20 mmol, 1.20 equiv.), CH_2Cl_2 (30.0 mL), DIPEA (2.61 mL, 15.00 mmol, 2.50 equiv.), and $BnNH_2$ (0.79 mL, 7.20 mmol, 1.20 equiv.). Purification by flash column chromatography (pentane/ethyl acetate, 10:1 to 1:1) afforded **S43a** as a colorless oil (1.00 g, 3.34 mmol, 56%).

Note: The oxime product was isolated as a mixture of *E/Z* isomers in an approximate ratio of 80:20. However, the specific configuration could not be unambiguously assigned based on the available spectroscopic data.

^{1}H NMR (500 MHz, $CDCl_3$) δ (ppm) = 8.41 (dd, J = 2.3, 0.8 Hz, 0.2H), 8.33 (dd, J = 2.4, 0.8 Hz, 0.8H), 7.87 (dd, J = 8.7, 2.5 Hz, 0.8H), 7.77 (dd, J = 8.7, 2.4 Hz, 0.2H), 7.38 – 7.16 (m, 5.2H), 6.76 – 6.68 (m, 1.8H), 4.60 (d, J = 5.8 Hz, 1.6H), 4.54 (d, J = 6.0 Hz, 0.4H), 3.98 (s, 2.4H), 3.97 (s, 0.6H), 3.94 (s, 0.6H), 3.92 (s, 2.4H).

^{13}C NMR (126 MHz, $CDCl_3$) δ (ppm) = 165.09, 164.35, 162.61, 162.27, 150.15, 149.05, 147.46, 146.20, 140.32, 138.07, 137.48, 136.84, 128.84, 127.95, 127.85, 127.76, 127.70, 121.28, 118.01, 111.04, 109.98, 63.48, 63.04, 53.79, 53.71, 43.68, 43.54.

IR (ATR): $\tilde{\nu}$ (cm^{-1}) = 1649, 1603, 1559, 1493, 1455, 1373, 1288, 1259, 1048.

HRMS (ESI$^+$) calcd. for $C_{16}H_{18}N_3O_3^+$ [M+H]$^+$: 300.1343, found: 300.1344.

***N*-benzyl-*N*-(2-(methoxyimino)-2-(6-methoxypyridin-3-yl)acetyl)-2-phenylacrylamide (S43b)**

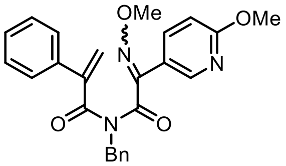

Prepared according to **General Procedure E (step E2 and E3)** using 2-phenylacrylic acid (1.38 g, 9.33 mmol, 3.00 equiv.), oxalyl chloride (1.05 mL, 12.44 mmol, 4.00 equiv.), CH_2Cl_2 (37.0 mL), DMF (3-5 drops), then CH_2Cl_2 (15.5 mL), Et_3N (1.73 mL, 12.44 mmol, 4.00 equiv.), amide **S43a** (930 mg, 3.11 mmol, 1.00 equiv.) and DMAP (190 mg, 1.56 mmol, 0.50 equiv.). Purification by flash column chromatography (pentane/ethyl acetate, 6:1 to 2:1) afforded **S43b** as a colorless oil (1.02 g, 2.38 mmol, 76%).

Note: The oxime product was isolated as a mixture of *E/Z* isomers in a ratio greater than 10:1. However, the specific configuration could not be unambiguously assigned based on the available spectroscopic data, and only the NMR data of the major isomer are reported.

^{1}H NMR (500 MHz, $CDCl_3$) δ (ppm) = 8.26 (d, *J* = 2.5 Hz, 1H), 7.85 – 7.76 (m, 1H), 7.42 – 7.28 (m, 10H), 6.75 (d, *J* = 8.8 Hz, 1H), 5.59 (s, 1H), 5.34 (s, 1H), 5.08 (s, 2H), 3.99 (s, 3H), 3.74 (s, 3H).

^{13}C NMR (126 MHz, $CDCl_3$) δ (ppm) = 171.87, 165.47, 165.03, 149.27, 145.66, 143.60, 136.61, 136.06, 134.37, 129.02, 128.68, 128.29, 127.81, 126.23, 121.30, 121.21, 111.20, 62.76, 53.83, 47.14.

IR (ATR): $\tilde{\nu}$ (cm^{-1}) = 1688, 1657, 1604, 1495, 1373, 1349, 1289, 1260, 1170, 1048.

HRMS (ESI$^+$) calcd. for $C_{25}H_{24}N_3O_4^+$ [M+H]$^+$: 430.1761, found: 430.1766.

***N*-benzyl-2-(furan-2-yl)-2-(methoxyimino)acetamide (S44a)**

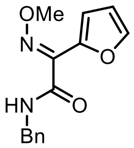

Prepared according to **General Procedure E (step E5)** using 2-(furan-2-yl)-2-oxoacetic acid (1.12 g, 8.00 mmol, 1.00 equiv.), $NH_2OMe{\cdot}HCl$ (1.34 g, 16.00 mmol, 2.00 equiv.), NaOH (960 mg, 24.00 mmol, 3.00 equiv.) and MeOH (16.0 mL), then TBTU (3.08 g, 9.60 mmol, 1.20 equiv.), CH_2Cl_2 (40.0 mL), DIPEA (3.48 mL, 20.00 mmol, 2.50 equiv.), and $BnNH_2$ (1.05 mL, 9.60 mmol, 1.20 equiv.). Purification by flash column chromatography (pentane/ethyl acetate, 10:1 to 1:1) afforded **S44a** as a colorless oil (690 mg, 2.67 mmol, 33%).

Note: The oxime product was isolated as a single stereoisomer. However, the *E/Z* configuration could not be unambiguously determined on the basis of the available spectroscopic data.

^{1}H NMR (500 MHz, $CDCl_3$) δ (ppm) = 7.38 (dd, *J* = 1.9, 0.8 Hz, 1H), 7.31 – 7.22 (m, 5H), 6.93 (t, *J* = 6.0 Hz, 1H), 6.67 (dd, *J* = 3.5, 0.8 Hz, 1H), 6.37 (dd, *J* = 3.5, 1.8 Hz, 1H), 4.51 (d, *J* = 6.0 Hz, 2H), 3.93 (s, 3H).

^{13}C NMR (126 MHz, $CDCl_3$) δ (ppm) = 160.63, 145.83, 144.35, 144.34, 137.37, 128.55, 127.53, 127.44, 113.86, 111.64, 63.02, 43.25.

IR (ATR): $\tilde{\nu}$ (cm^{-1}) = 1652, 1532, 1455, 1332, 1275, 1261, 1159, 1046.

HRMS (ESI^+) calcd. for $C_{14}H_{15}N_2O_3^+$ $[M+H]^+$: 259.1077, found: 259.1069.

***N*-benzyl-*N*-(2-(furan-2-yl)-2-(methoxyimino)acetyl)-2-phenylacrylamide (S44b)**

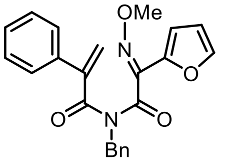

Prepared according to **General Procedure E (step E2 and E3)** using 2-phenylacrylic acid (0.93 g, 6.28 mmol, 3.00 equiv.), oxalyl chloride (0.71 mL, 8.36 mmol, 4.00 equiv.), CH_2Cl_2 (25.0 mL), DMF (3-5 drops), then CH_2Cl_2 (10.5 mL), Et_3N (1.17 mL, 8.36 mmol, 4.00 equiv.), amide **S44a** (540 mg, 2.09 mmol, 1.00 equiv.) and DMAP (127 mg, 1.04 mmol, 0.50 equiv.). Purification by flash column chromatography (pentane/ethyl acetate, 6:1 to 2:1) afforded **S44b** as a colorless oil (696 mg, 1.79 mmol, 86%).

Note: The oxime product was isolated as a single stereoisomer. However, the *E/Z* configuration could not be unambiguously determined on the basis of the available spectroscopic data.

^{1}H NMR (500 MHz, $CDCl_3$) δ (ppm) = 7.47 (dd, *J* = 1.8, 0.8 Hz, 1H), 7.41 – 7.37 (m, 4H), 7.35 – 7.31 (m, 5H), 7.30 – 7.26 (m, 1H), 6.48 (s, 1H), 6.43 (dd, *J* = 3.5, 1.8 Hz, 1H), 5.59 (s, 1H), 5.38 (s, 1H), 5.08 (s, 2H), 3.64 (s, 3H).

^{13}C NMR (126 MHz, $CDCl_3$) δ (ppm) = 171.73, 163.89, 145.97, 144.52, 144.10, 143.75, 136.58, 134.69, 128.85, 128.61, 128.60, 128.50, 127.79, 126.44, 121.93, 112.30, 111.84, 62.97, 47.24.

IR (ATR): $\tilde{\nu}$ (cm^{-1}) = 1693, 1659, 1497, 1433, 1349, 1276, 1260, 1173, 1155, 1047.

HRMS (ESI^+) calcd. for $C_{23}H_{21}N_2O_4^+$ $[M+H]^+$: 389.1496, found: 389.1488.

1.11. General Procedure F for Intramolecular [2+2] Cyclization

An oven-dried round-bottom flask containing substrate (1.0 equiv.) and [Ir(dF(CF_3)ppy)$_2$(dtbbpy)](PF_6) (1 mol%) was sealed and subjected to three N_2 evacuation/refill cycles before anhydrous solvent pre-sparged with N_2 (0.1 M) was added. The flask was placed at a distance of approximately 1 cm from a 40 W KSPR160L-427 nm Kessil light (100% intensity) and the reaction was stirred under continuous irradiation under a nitrogen atmosphere for 1-4 h until complete (judged by TLC analysis). The solvent was removed in vacuo and the crude product was purified by flash column chromatography.

Notes:

(1) For some substrates, when thioxanthone (0.1 equiv.) is used as a photosensitizer, the light source also changes to 40W KSPR160L-390 nm Kessil light (100% intensity).

(2) The reaction was set up under ambient (room temperature) conditions, but due to irradiation with blue LEDs, the actual temperature of the reaction mixture was higher than room temperature. Fan-assisted cooling was used to maintain the temperature at around 40 °C (The solution temperature was monitored with a thermometer probe placed 1 cm below the solvent surface).

(3) Most substrates react completely after 2 h, so it is recommended to monitor the reaction hourly (by TLC, the product is slightly more polar than the starting material). Substrates requiring extended reaction times (more than 4 h) are specifically indicated in the subsequent detailed data descriptions.

(4) The major and minor products (e.g., compounds **4** and **4a**) were occasionally challenging to separate using flash column chromatography with a pentane/ethyl acetate eluent system. However, effective separation was achieved by employing an alternative eluent system.

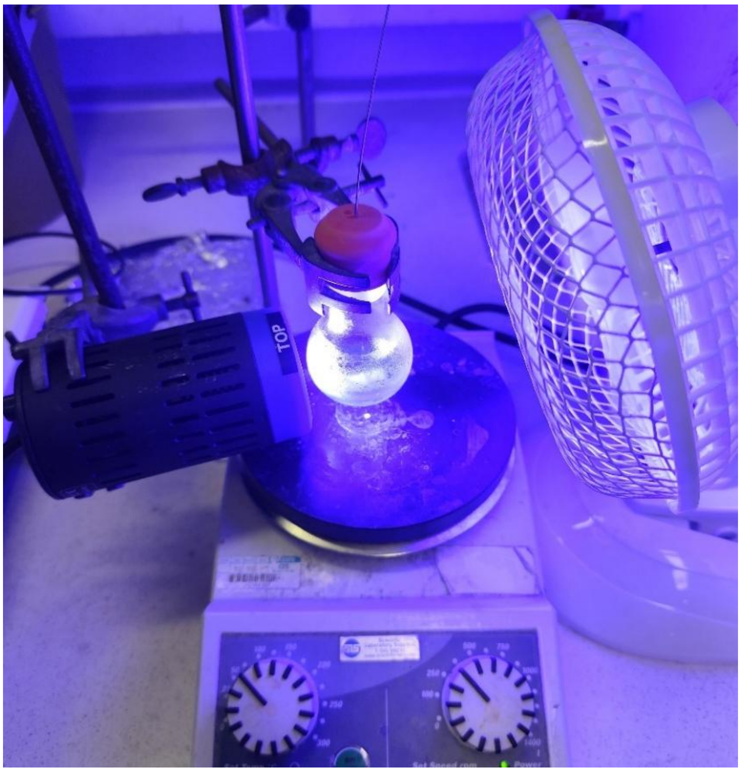

Photocatalysis reaction setup

6-Methoxy-1-phenyl-3-tosyl-3,6-diazabicyclo[3.1.1]heptane (4)

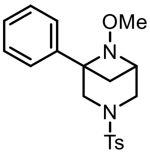

Prepared according to **General Procedure F** using oxime **S4b** (196 mg, 0.55 mmol, 1.00 equiv.), CH_3CN (5.5 mL), [Ir(dF(CF_3)ppy)$_2$(dtbbpy)](PF_6) (6.1 mg, 5.4 µmol, 1 mol%). Purification by flash column chromatography (pentane/ethyl acetate, 6:1 to 3:1 and CH_2Cl_2/ethyl acetate, 20:1 to 7:1) afforded **4** as a white solid (133 mg, 0.37 mmol, 68%) and **4a** as a colorless oil (24.8 mg, 0.069 mmol, 13%).

Gram scale: Prepared according to **General Procedure F** using oxime **S4b** (1.43 g, 4.00 mmol, 1.00 equiv.), CH_3CN (40.0 mL), [Ir(dF(CF_3)ppy)$_2$(dtbbpy)](PF_6) (22.4 mg, 0.02 mmol, 0.5 mol%). Purification by flash column chromatography (pentane/ethyl acetate, 6:1 to 3:1 and CH_2Cl_2/ethyl acetate, 20:1 to 7:1) afforded **4** as a white solid (874 mg, 2.44 mmol, 61%) and **4a** as a colorless oil (171 mg, 0.48 mmol, 12%).

Note: For gram-scale reactions, the reaction time is 20 hours.

M.P.: 130-132 °C

^{1}**H NMR** (500 MHz, $CDCl_3$) δ (ppm) = 7.73 (d, *J* = 8.3 Hz, 2H), 7.33-7.28 (m, 4H), 7.27-7.22 (m, 3H), 3.96 (ddt, *J* = 6.1, 2.5, 1.0 Hz, 1H), 3.63 (d, *J* = 10.9 Hz, 1H), 3.59-3.54 (m, 2H), 3.50 (dd, *J* = 10.9, 2.6 Hz, 1H), 3.29 (s, 3H), 2.41 (s, 3H), 2.15 (d, *J* = 8.7 Hz, 1H), 1.99 (dd, *J* = 9.4, 6.0 Hz, 1H).

^{13}**C NMR** (126 MHz, $CDCl_3$) δ (ppm) = 143.30, 141.81, 134.54, 129.50, 128.48, 127.75, 127.44, 124.96, 72.93, 59.95, 59.40, 46.92, 41.53, 27.82, 21.58.

IR (ATR): $\tilde{\nu}$ (cm^{-1}) = 3408, 2948, 1651, 1598, 1448, 1338, 1276, 1261, 1162, 1023, 1010.

HRMS (ESI^+) calcd. for $C_{19}H_{23}N_2O_3S^+$ $[M+H]^+$: 359.1424, found: 359.1416.

6-Methoxy-1-phenyl-3-tosyl-3,6-diazabicyclo[3.2.0]heptane (4a)

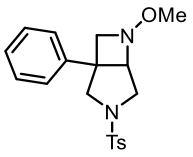

^{1}**H NMR** (500 MHz, DMSO-d_6, 70 °C) δ (ppm) = 7.71 (d, *J* = 8.3 Hz, 2H), 7.42 (d, *J* = 8.0 Hz, 2H), 7.33-7.27 (m, 2H), 7.24-7.20 (m, 1H), 7.18-7.15 (m, 2H), 4.37 (d, *J* = 5.1 Hz, 1H), 3.96-3.86 (m, 2H), 3.78 (d, *J* = 10.0 Hz, 1H), 3.69 (d, *J* = 9.0 Hz, 1H), 3.33 (s, 3H), 3.05 (dd, *J* = 11.0, 5.3 Hz, 1H), 2.77 (d, *J* = 10.0 Hz, 1H), 2.39 (s, 3H).

^{13}**C NMR** (126 MHz, DMSO-d_6, 70 °C) δ (ppm) = 143.24, 141.51, 132.40, 129.39, 128.27, 127.28, 126.43, 125.32, 75.36, 65.19, 59.06, 57.77, 50.32, 46.85, 20.61.

Note: Most of these 1,2-fused azetidine products exhibit significant signal broadening in NMR spectra at room temperature due to inherent steric hindrance. High-temperature NMR measurements provide well-resolved spectra.

IR (ATR): $\tilde{\nu}$ (cm^{-1}) = 2941, 1598, 1497, 1448, 1345, 1276, 1164, 1091, 1054, 1027, 1004.

HRMS (ESI^+) calcd. for $C_{19}H_{23}N_2O_3S^+$ $[M+H]^+$: 359.1424, found: 359.1407.

1-(4-Fluorophenyl)-6-methoxy-3-tosyl-3,6-diazabicyclo[3.1.1]heptane (6)

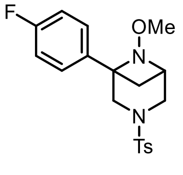

Prepared according to **General Procedure F** using oxime **S6b** (724 mg, 1.93 mmol, 1.00 equiv.), CH_3CN (19.3 mL), [Ir(dF(CF_3)ppy)$_2$(dtbbpy)](PF_6) (21.6 mg, 0.019 mmol, 1 mol%). Purification by flash column chromatography (pentane/ethyl acetate, 6:1 to 2:1 and CH_2Cl_2/ethyl acetate, 20:1 to 6:1)

afforded **6** as a colorless oil (446mg, 1.19 mmol, 62%) and **6a** as a colorless oil (78 mg, 0.21 mmol, 11%).

^{1}H NMR (500 MHz, $CDCl_3$) δ (ppm) = 7.71 (d, J = 8.4 Hz, 2H), 7.29-7.26 (m, 2H), 7.21-7.16 (m, 2H), 6.99-6.91 (m, 2H), 3.95-3.89 (m, 1H), 3.57 (d, J = 10.8 Hz, 1H), 3.55-3.50 (m, 2H), 3.46 (dd, J = 10.9, 2.6 Hz, 1H), 3.23 (s, 3H), 2.36 (s, 3H), 2.08 (d, J = 8.7 Hz, 1H), 1.91 (dd, J = 9.4, 6.0 Hz, 1H).

^{13}C NMR (126 MHz, $CDCl_3$) δ (ppm) = 162.08 (d, J = 246.2 Hz), 143.23, 137.63 (d, J = 3.1 Hz), 134.31, 129.39, 127.25, 126.67 (d, J = 8.1 Hz), 115.15 (d, J = 21.4 Hz), 72.16, 59.76, 59.21, 46.68, 41.28, 27.70, 21.37.

^{19}F NMR (471 MHz, $CDCl_3$) δ (ppm) = −114.52 (tt, J = 8.5, 5.3 Hz).

IR (ATR): $\tilde{\nu}$ (cm^{-1}) = 2948, 1608, 1598, 1510, 1461, 1337, 1222, 1158, 1095, 1051, 1011.

HRMS (ESI^+) calcd. for $C_{19}H_{22}FN_2O_3S^+$ $[M+H]^+$: 377.1330, found: 377.1319.

1-(4-Fluorophenyl)-6-methoxy-3-tosyl-3,6-diazabicyclo[3.2.0]heptane (6a)

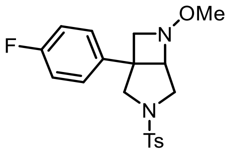

^{1}H NMR (500 MHz, DMSO-d_6, 75 °C) δ (ppm) = 7.71 (d, J = 8.3 Hz, 2H), 7.42 (d, J = 7.8 Hz, 2H), 7.24-7.20 (m, 2H), 7.12-7.07 (m, 2H), 4.37 (d, J = 5.2 Hz, 1H), 3.94-3.87 (m, 2H), 3.78 (d, J = 10.0 Hz, 1H), 3.67 (d, J = 9.1 Hz, 1H), 3.33 (d, J = 0.6 Hz, 3H), 3.06 (dd, J = 11.1, 5.3 Hz, 1H), 2.75 (d, J = 10.0 Hz, 1H), 2.39 (s, 3H).

^{13}C NMR (126 MHz, DMSO-d_6, 75 °C) δ (ppm) = 160.76 (d, J = 243.7 Hz), 143.24, 137.60, 132.47, 129.38, 127.47 (d, J = 8.1 Hz), 127.27, 114.95 (d, J = 21.1 Hz), 75.37, 65.21, 59.05, 57.63, 50.31, 46.43, 20.59.

^{19}F NMR (470 MHz, DMSO-d_6, 75 °C) δ (ppm) = −115.88 (tt, J = 9.2, 4.8 Hz).

IR (ATR): $\tilde{\nu}$ (cm^{-1}) = 2939, 1598, 1512, 1466, 1344, 1227, 1160, 1094, 1039, 1001.

HRMS (ESI^+) calcd. for $C_{19}H_{22}FN_2O_3S^+$ $[M+H]^+$: 377.1330, found: 377.1318.

1-(4-Chlorophenyl)-6-methoxy-3-tosyl-3,6-diazabicyclo[3.1.1]heptane (7)

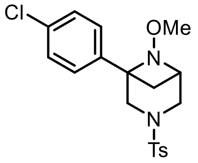

Prepared according to **General Procedure F** using oxime **S7b** (751 mg, 1.92 mmol, 1.00 equiv.), CH_3CN (19.2 mL), $[Ir(dF(CF_3)ppy)_2(dtbbpy)](PF_6)$ (21.5 mg, 0.019 mmol, 1 mol%). Purification by flash column chromatography (pentane/ethyl acetate, 6:1 to 2:1 and CH_2Cl_2/ethyl acetate, 20:1 to 6:1) afforded **7** as a colorless oil (462 mg, 1.18 mmol, 62%) and **7a** as a colorless oil (54 mg, 0.14 mmol, 7%).

^{1}H NMR (500 MHz, $CDCl_3$) δ (ppm) = 7.71 (d, J = 8.4 Hz, 2H), 7.27 (d, J = 8.5 Hz, 2H), 7.24 (d, J = 8.6 Hz, 2H), 7.16 (d, J = 8.6 Hz, 2H), 3.92 (ddt, J = 6.4, 2.5, 1.0 Hz, 1H), 3.57 (d, J = 10.9 Hz, 1H), 3.55-3.50 (m, 2H), 3.46 (dd, J = 10.9, 2.6 Hz, 1H), 3.23 (s, 3H), 2.36 (s, 3H), 2.08 (d, J = 9.4 Hz, 1H), 1.90 (dd, J = 9.4, 6.1 Hz, 1H).

^{13}C NMR (126 MHz, $CDCl_3$) δ (ppm) = 143.20, 140.19, 134.22, 133.31, 129.36, 128.41, 127.21, 126.37, 72.12, 59.75, 59.18, 46.44, 41.24, 27.65, 21.36.

IR (ATR): $\tilde{\nu}$ (cm^{-1}) = 2948, 2876, 1598, 1491, 1461, 1337, 1159, 1090, 1051, 1022, 1011.

HRMS (ESI$^+$) calcd. for $C_{19}H_{22}ClN_2O_3S^+$ $[M+H]^+$: 393.1034, found: 393.1021.

1-(4-Chlorophenyl)-6-methoxy-3-tosyl-3,6-diazabicyclo[3.2.0]heptane (7a)

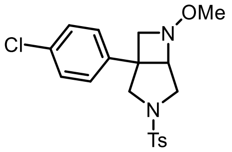

^{1}H NMR (500 MHz, DMSO-d$_6$, 75 °C) δ (ppm) = 7.70 (d, J = 7.9 Hz, 2H), 7.42 (d, J = 8.0 Hz, 2H), 7.34 (d, J = 8.5 Hz, 2H), 7.21 (d, J = 8.2 Hz, 2H), 4.37 (d, J = 5.2 Hz, 1H), 3.94-3.84 (m, 2H), 3.78 (d, J = 10.1 Hz, 1H), 3.65 (d, J = 9.1 Hz, 1H), 3.33 (s, 3H), 3.09-3.01 (m, 1H), 2.75 (dd, J = 9.9, 3.0 Hz, 1H), 2.40 (s, 3H).

^{13}C NMR (126 MHz, DMSO-d$_6$, 75 °C) δ (ppm) = 143.24, 140.34, 132.41, 131.28, 129.38, 128.18, 127.40, 127.25, 75.29, 65.08, 59.07, 57.41, 50.30, 46.51, 20.60.

IR (ATR): $\tilde{\nu}$ (cm^{-1}) = 2940, 2865, 1597, 1494, 1465, 1345, 1163, 1092, 1040, 1013.

HRMS (ESI$^+$) calcd. for $C_{19}H_{22}ClN_2O_3S^+$ $[M+H]^+$: 393.1034, found: 393.1020.

1-(3-Chlorophenyl)-6-methoxy-3-tosyl-3,6-diazabicyclo[3.1.1]heptane (8)

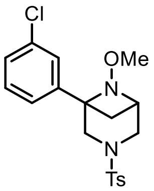

Prepared according to **General Procedure F** using oxime **S8b** (715 mg, 1.82 mmol, 1.00 equiv.), CH_3CN (18.2 mL), [Ir(dF(CF_3)ppy)$_2$(dtbbpy)](PF_6) (20.5 mg, 0.018 mmol, 1 mol%). Purification by flash column chromatography (pentane/ethyl acetate, 6:1 to 2:1 and CH_2Cl_2/ethyl acetate, 20:1 to 6:1) afforded **8** as a colorless oil (503 mg, 1.28 mmol, 70%) and **8a** as a colorless oil (87 mg, 0.22 mmol, 12%).

^{1}H NMR (500 MHz, $CDCl_3$) δ (ppm) = 7.69 (d, *J* = 8.3 Hz, 2H), 7.27-7.24 (m, 2H), 7.22-7.15 (m, 3H), 7.08 (dt, *J* = 7.2, 1.7 Hz, 1H), 3.93-3.88 (m, 1H), 3.57 (d, *J* = 10.8 Hz, 1H), 3.52 (dd, *J* = 6.1, 1.4 Hz, 1H), 3.50 (dd, *J* = 6.2, 1.4 Hz, 1H), 3.45 (dd, *J* = 11.0, 2.6 Hz, 1H), 3.22 (s, 3H), 2.35 (s, 3H), 2.08 (d, *J* = 9.4 Hz, 1H), 1.89 (dd, *J* = 9.4, 6.0 Hz, 1H).

^{13}C NMR (126 MHz, $CDCl_3$) δ (ppm) = 143.62, 143.18, 134.17, 134.12, 129.69, 129.35, 127.64, 127.18, 125.18, 123.05, 72.17, 59.69, 59.14, 46.39, 41.21, 27.64, 21.33.

IR (ATR): $\tilde{\nu}$ (cm^{-1}) = 2948, 2876, 1598, 1571, 1461, 1337, 1159, 1093, 1050, 1022, 1011.

HRMS (ESI^+) calcd. for $C_{19}H_{22}ClN_2O_3S^+$ $[M+H]^+$: 393.1034, found: 393.1020.

1-(3-Chlorophenyl)-6-methoxy-3-tosyl-3,6-diazabicyclo[3.2.0]heptane (8a)

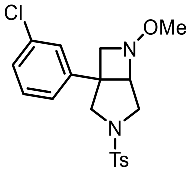

^{1}H NMR (500 MHz, DMSO-d$_6$, 75 °C) δ (ppm) = 7.71 (d, *J* = 8.2 Hz, 2H), 7.42 (d, *J* = 7.8 Hz, 2H), 7.35-7.25 (m, 2H), 7.24 (t, *J* = 2.1 Hz, 1H), 7.15 (dt, *J* = 7.6, 1.5 Hz, 1H), 4.40 (d, *J* = 5.2 Hz, 1H), 3.92-3.87 (m, 2H), 3.81 (d, *J* = 10.1 Hz, 1H), 3.67 (d, *J* = 9.2 Hz, 1H), 3.33 (s, 3H), 3.07 (dd, *J* = 11.0, 5.0 Hz, 1H), 2.76 (dd, *J* = 10.5, 2.3 Hz, 1H), 2.39 (s, 3H).

^{13}C NMR (126 MHz, DMSO-d$_6$, 75 °C) δ (ppm) = 143.84, 143.25, 133.17, 132.44, 130.09, 129.39, 127.26, 126.52, 125.49, 124.19, 75.18, 65.06, 59.08, 57.39, 50.27, 46.77, 20.61.

IR (ATR): $\tilde{\nu}$ (cm^{-1}) = 2939, 2864, 1598, 1570, 1466, 1346, 1161, 1092, 1043, 1001.

HRMS (ESI^+) calcd. for $C_{19}H_{22}ClN_2O_3S^+$ $[M+H]^+$: 393.1034, found: 393.1020.

4-(6-Methoxy-3-tosyl-3,6-diazabicyclo[3.1.1]heptan-1-yl)benzonitrile (9)

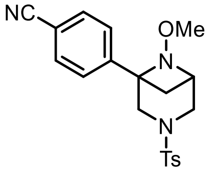

Prepared according to **General Procedure F** using oxime **S9b** (860 mg, 2.25 mmol, 1.00 equiv.), CH_3CN (22.5 mL), [Ir(dF(CF_3)ppy)$_2$(dtbbpy)](PF_6) (25.2 mg, 0.023 mmol, 1 mol%). Purification by flash column chromatography (pentane/ethyl acetate, 6:1 to 1:1 and CH_2Cl_2/ethyl acetate, 10:1 to 4:1) afforded **9** as a colorless oil (650 mg, 1.70 mmol, 76%) and **9a** as a colorless oil (24 mg, 0.063 mmol, 3%).

^{1}H NMR (500 MHz, $CDCl_3$) δ (ppm) = 7.63 (d, *J* = 8.2 Hz, 2H), 7.50 (d, *J* = 8.5 Hz, 2H), 7.28 (d, *J* = 8.5 Hz, 2H), 7.21 (d, *J* = 8.1 Hz, 2H), 3.88 (dd, *J* = 5.4, 2.7 Hz, 1H), 3.51 (d, *J* = 10.9 Hz, 1H), 3.47-3.43 (m, 2H), 3.40 (dd, *J* = 11.0, 2.6 Hz, 1H), 3.16 (s, 3H), 2.29 (s, 3H), 2.05 (d, *J* = 9.4 Hz, 1H), 1.83 (dd, *J* = 9.5, 6.1 Hz, 1H).

^{13}C NMR (126 MHz, $CDCl_3$) δ (ppm) = 146.48, 143.12, 133.86, 131.93, 129.23, 126.99, 125.69, 118.29, 111.06, 72.07, 59.45, 59.00, 45.97, 41.04, 27.35, 21.14.

IR (ATR): $\tilde{\nu}$ (cm^{-1}) = 2950, 2876, 2229, 1611, 1598, 1460, 1337, 1160, 1095, 1051, 1022, 1011.

HRMS (ESI$^+$) calcd. for $C_{20}H_{22}N_3O_3S^+$ [M+H]$^+$: 384.1376, found: 384.1358.

4-(6-Methoxy-3-tosyl-3,6-diazabicyclo[3.2.0]heptan-1-yl)benzonitrile (9a)

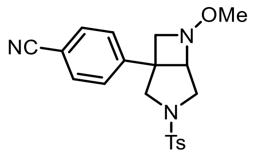

^{1}H NMR (500 MHz, DMSO-d$_6$, 75 °C) δ (ppm) = 7.75 (d, *J* = 8.4 Hz, 2H), 7.70 (d, *J* = 8.3 Hz, 2H), 7.44-7.38 (m, 4H), 4.42 (d, *J* = 5.2 Hz, 1H), 3.94-3.86 (m, 2H), 3.83 (d, *J* = 10.2 Hz, 1H), 3.67 (d, *J* = 9.2 Hz, 1H), 3.33 (s, 3H), 3.08 (dd, *J* = 11.0, 5.2 Hz, 1H), 2.78 (dd, *J* = 10.0, 2.3 Hz, 1H), 2.40 (s, 3H).

^{13}C NMR (126 MHz, DMSO-d$_6$, 75 °C) δ (ppm) = 146.74, 143.29, 132.43, 132.17, 129.41, 127.27, 126.72, 118.15, 109.60, 75.25, 64.94, 59.13, 57.04, 50.12, 47.18, 20.61.

IR (ATR): $\tilde{\nu}$ (cm^{-1}) = 2939, 2867, 2228, 1609, 1598, 1465, 1343, 1161, 1093, 1040, 1002.

HRMS (ESI$^+$) calcd. for $C_{20}H_{22}N_3O_3S^+$ [M+H]$^+$: 384.1376, found: 384.1362.

6-Methoxy-3-tosyl-1-(4-(trifluoromethyl)phenyl)-3,6-diazabicyclo[3.1.1]heptane (10)

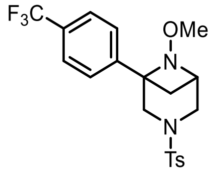

Prepared according to **General Procedure F** using oxime **S10b** (814 mg, 1.91 mmol, 1.00 equiv.), CH_3CN (19.1 mL), [Ir(dF(CF_3)ppy)$_2$(dtbbpy)](PF_6) (21.4 mg, 0.019 mmol, 1 mol%). Purification by flash column chromatography (pentane/ethyl acetate, 6:1 to 2:1 and CH_2Cl_2/ethyl acetate, 20:1 to 5:1) afforded **10** as a colorless oil (570 mg, 1.34 mmol, 70%) and **10a** as a colorless oil (73 mg, 0.17 mmol, 9%).

^{1}H NMR (500 MHz, $CDCl_3$) δ (ppm) = 7.71 (d, J = 8.4 Hz, 2H), 7.53 (d, J = 8.2 Hz, 2H), 7.35 (d, J = 8.1 Hz, 2H), 7.27 (d, J = 8.2 Hz, 2H), 3.94 (dt, J = 5.4, 1.7 Hz, 1H), 3.60 (d, J = 10.9 Hz, 1H), 3.56-3.52 (m, 2H), 3.48 (dd, J = 11.0, 2.6 Hz, 1H), 3.24 (s, 3H), 2.35 (s, 3H), 2.13 (d, J = 9.3 Hz, 1H), 1.93 (dd, J = 9.4, 6.1 Hz, 1H).

^{13}C NMR (126 MHz, $CDCl_3$) δ (ppm) = 145.66, 143.35, 134.22, 129.66 (q, J = 32.3 Hz), 129.42, 127.27, 125.46, 125.27 (q, J = 3.8 Hz), 124.00 (q, J = 272.1 Hz), 72.38, 59.68, 59.29, 46.45, 41.31, 27.63, 21.30.

^{19}F NMR (471 MHz, $CDCl_3$) δ (ppm) = −62.52 (s).

IR (ATR): $\tilde{\nu}$ (cm^{-1}) = 2949, 1621, 1598, 1462, 1410, 1232, 1159, 1118, 1072, 1011.

HRMS (ESI^+) calcd. for $C_{20}H_{22}F_3N_2O_3S^+$ $[M+H]^+$: 427.1298, found: 427.1287.

6-Methoxy-3-tosyl-1-(4-(trifluoromethyl)phenyl)-3,6-diazabicyclo[3.2.0]heptane (10a)

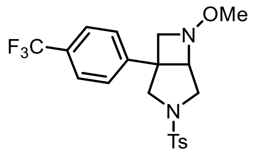

^{1}H NMR (500 MHz, DMSO-d$_6$, 75 °C) δ (ppm) = 7.71 (d, J = 8.3 Hz, 2H), 7.65 (d, J = 8.7 Hz, 2H), 7.45-7.40 (m, 4H), 4.43 (d, J = 5.1 Hz, 1H), 3.95-3.89 (m, 2H), 3.84 (d, J = 10.1 Hz, 1H), 3.70 (d, J = 9.2 Hz, 1H), 3.34 (s, 3H), 3.09 (dd, J = 11.2, 5.3 Hz, 1H), 2.80 (d, J = 9.5 Hz, 1H), 2.39 (s, 3H).

^{13}C NMR (126 MHz, DMSO-d$_6$, 75 °C) δ (ppm) = 145.97, 143.25, 132.45, 129.39, 127.39 (q, J = 32.4 Hz), 127.27, 126.44, 125.09 (q, J = 3.9 Hz), 123.84 (q, J = 271.8 Hz), 75.31, 64.99, 59.10, 57.23, 50.52, 46.94, 20.57.

^{19}F NMR (470 MHz, DMSO-d$_6$, 75 °C) δ (ppm) = −61.15 (s).

IR (ATR): $\tilde{\nu}$ (cm^{-1}) = 2941, 1619, 1598, 1466, 1411, 1326, 1163, 1121, 1068, 1018.

HRMS (ESI^+) calcd. for $C_{20}H_{22}F_3N_2O_3S^+$ $[M+H]^+$: 427.1298, found: 427.1288.

Ethyl 4-(6-methoxy-3-tosyl-3,6-diazabicyclo[3.1.1]heptan-1-yl)benzoate (11)

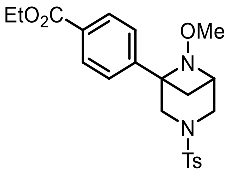

Prepared according to **General Procedure F** using oxime **S11b** (880 mg, 2.05 mmol, 1.00 equiv.), CH_3CN (20.5 mL), [Ir(dF(CF_3)ppy)$_2$(dtbbpy)](PF_6) (23.0 mg, 0.020 mmol, 1 mol%). Purification by flash column chromatography (pentane/ethyl acetate, 6:1 to 2:1 and CH_2Cl_2/ethyl acetate, 20:1 to 5:1) afforded **11** as a colorless oil (671 mg, 1.56 mmol, 76%) and **11a** as a colorless oil (20 mg, 0.047 mmol, 2%).

^{1}H NMR (500 MHz, $CDCl_3$) δ (ppm) = 7.96 (d, *J* = 8.4 Hz, 2H), 7.70 (d, *J* = 8.3 Hz, 2H), 7.31-7.25 (m, 4H), 4.31 (q, *J* = 7.2 Hz, 2H), 3.94 (dt, *J* = 5.5, 2.2 Hz, 1H), 3.60 (d, *J* = 10.9 Hz, 1H), 3.55-3.51 (m, 2H), 3.47 (dd, *J* = 11.0, 2.6 Hz, 1H), 3.25 (s, 3H), 2.37 (s, 3H), 2.14 (d, *J* = 9.4 Hz, 1H), 1.95 (dd, *J* = 9.4, 6.0 Hz, 1H), 1.33 (t, *J* = 7.1 Hz, 3H).

^{13}C NMR (126 MHz, $CDCl_3$) δ (ppm) = 166.13, 146.45, 143.30, 134.33, 129.81, 129.66, 129.45, 127.32, 124.99, 72.69, 60.94, 59.83, 59.34, 46.46, 41.36, 27.74, 21.47, 14.28.

IR (ATR): $\tilde{\nu}$ (cm^{-1}) = 2950, 1713, 1613, 1463, 1446, 1339, 1272, 1161, 1104, 1021.

HRMS (ESI$^+$) calcd. for $C_{22}H_{27}N_2O_5S^+$ [M+H]$^+$: 431.1635, found: 431.1619.

Ethyl 4-(6-methoxy-3-tosyl-3,6-diazabicyclo[3.2.0]heptan-1-yl)benzoate (11a)

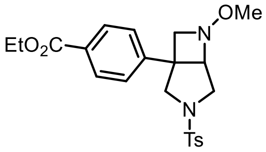

^{1}H NMR (500 MHz, DMSO-d$_6$, 75 °C) δ (ppm) = 7.88 (d, *J* = 8.3 Hz, 2H), 7.70 (d, *J* = 8.2 Hz, 2H), 7.41 (d, *J* = 7.8 Hz, 2H), 7.31 (d, *J* = 8.3 Hz, 2H), 4.41 (d, *J* = 5.1 Hz, 1H), 4.31 (q, *J* = 7.1 Hz, 2H), 3.93-3.88 (m, 2H), 3.82 (d, *J* = 10.1 Hz, 1H), 3.68 (d, *J* = 9.1 Hz, 1H), 3.33 (s, 3H), 3.09 (dd, *J* = 10.9, 5.2 Hz, 1H), 2.79 (d, *J* = 9.8 Hz, 1H), 2.39 (s, 3H), 1.31 (t, *J* = 7.1 Hz, 3H).

^{13}C NMR (126 MHz, DMSO-d$_6$, 75 °C) δ (ppm) = 165.11, 146.58, 143.29, 132.49, 129.42, 129.11, 128.40, 127.27, 125.81, 75.39, 65.01, 60.31, 59.13, 57.39, 50.55, 47.02, 20.61, 13.77.

IR (ATR): $\tilde{\nu}$ (cm^{-1}) = 2942, 1714, 1612, 1465, 1347, 1277, 1164, 1103, 1020.

HRMS (ESI$^+$) calcd. for $C_{22}H_{27}N_2O_5S^+$ [M+H]$^+$: 431.1635, found: 431.1619.

6-Methoxy-1-(*p*-tolyl)-3-tosyl-3,6-diazabicyclo[3.1.1]heptane (12)

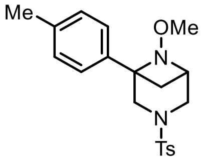

Prepared according to **General Procedure F** using oxime **S12b** (814 mg, 2.19 mmol, 1.00 equiv.), CH_3CN (21.9 mL), [Ir(dF(CF_3)ppy)$_2$(dtbbpy)](PF_6) (24.6 mg, 0.022 mmol, 1 mol%). Purification by flash column chromatography (pentane/ethyl acetate, 6:1 to 2:1 and CH_2Cl_2/ethyl acetate, 20:1 to 6:1) afforded **12** as a colorless oil (505 mg, 1.36 mmol, 62%) and **12a** as a colorless oil (107 mg, 0.29 mmol, 13%).

^{1}H NMR (500 MHz, $CDCl_3$) δ (ppm) = 7.74 (d, J = 8.4 Hz, 2H), 7.28 (d, J = 8.1 Hz, 2H), 7.15 (d, J = 8.3 Hz, 2H), 7.11 (d, J = 8.1 Hz, 2H), 3.93 (dt, J = 5.2, 2.1 Hz, 1H), 3.63 (d, J = 10.9 Hz, 1H), 3.61-3.55 (m, 2H), 3.48 (dd, J = 10.9, 2.6 Hz, 1H), 3.27 (s, 3H), 2.38 (s, 3H), 2.29 (s, 3H), 2.08 (d, J = 9.4 Hz, 1H), 1.94 (dd, J = 9.4, 6.0 Hz, 1H).

^{13}C NMR (126 MHz, $CDCl_3$) δ (ppm) = 142.99, 138.68, 137.06, 134.32, 129.24, 128.86, 127.15, 124.63, 72.41, 59.69, 59.07, 46.66, 41.29, 27.61, 21.26, 20.88.

IR (ATR): $\tilde{\nu}$ (cm^{-1}) = 2945, 1598, 1515, 1454, 1337, 1159, 1092, 1051, 1022, 1011.

HRMS (ESI$^+$) calcd. for $C_{20}H_{25}N_2O_3S^+$ [M+H]$^+$: 373.1580, found: 373.1562.

6-Methoxy-1-(p-tolyl)-3-tosyl-3,6-diazabicyclo[3.2.0]heptane (12a)

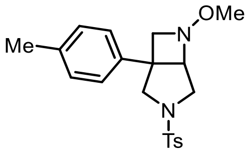

^{1}H NMR (500 MHz, DMSO-d$_6$, 75 °C) δ (ppm) = 7.71 (d, J = 8.1 Hz, 2H), 7.42 (d, J = 8.6 Hz, 2H), 7.10 (d, J = 8.4 Hz, 2H), 7.04 (d, J = 8.1 Hz, 2H), 4.34 (d, J = 5.1 Hz, 1H), 3.92-3.85 (m, 2H), 3.75 (d, J = 10.0 Hz, 1H), 3.66 (d, J = 9.0 Hz, 1H), 3.33 (d, J = 0.8 Hz, 3H), 3.03 (dd, J = 10.8, 4.0 Hz, 1H), 2.74 (d, J = 9.6 Hz, 1H), 2.39 (s, 3H), 2.26 (s, 3H).

^{13}C NMR (126 MHz, DMSO-d$_6$, 75 °C) δ (ppm) = 143.18, 138.48, 135.61, 132.40, 129.34, 128.78, 127.24, 125.16, 75.39, 65.19, 59.02, 57.83, 50.54, 46.52, 20.58, 20.11.

IR (ATR): $\tilde{\nu}$ (cm^{-1}) = 2938, 1597, 1517, 1465, 1451, 1345, 1161, 1100, 1092, 1041, 1003.

HRMS (ESI$^+$) calcd. for $C_{20}H_{25}N_2O_3S^+$ [M+H]$^+$: 373.1580, found: 373.1563.

6-Methoxy-1-(4-methoxyphenyl)-3-tosyl-3,6-diazabicyclo[3.1.1]heptane (13)

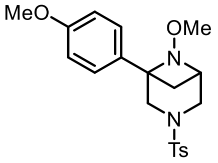

Prepared according to **General Procedure F** using oxime **S13b** (710 mg, 1.83 mmol, 1.00 equiv.), CH_3CN (18.3 mL), $[Ir(dF(CF_3)ppy)_2(dtbbpy)](PF_6)$ (20.5 mg, 0.018 mmol, 1 mol%). Purification by flash column chromatography (pentane/ethyl acetate, 6:1 to 2:1 and CH_2Cl_2/ethyl acetate, 20:1 to 6:1) afforded **13** as a colorless oil (398 mg, 1.03 mmol, 56%) and **13a** as a colorless oil (138 mg, 0.36 mmol, 19%).

^{1}H NMR (500 MHz, $CDCl_3$) δ (ppm) = 7.73 (d, J = 8.3 Hz, 2H), 7.28 (d, J = 7.9 Hz, 2H), 7.16 (d, J = 8.8 Hz, 2H), 6.83 (d, J = 8.9 Hz, 2H), 3.94-3.90 (m, 1H), 3.73 (s, 3H), 3.60 (d, J = 10.9 Hz, 1H), 3.58-3.52 (m, 2H), 3.46 (dd, J = 10.9, 2.6 Hz, 1H), 3.25 (s, 3H), 2.38 (s, 3H), 2.07 (d, J = 9.3 Hz, 1H), 1.93 (dd, J = 9.4, 6.0 Hz, 1H).

^{13}C NMR (126 MHz, $CDCl_3$) δ (ppm) = 158.98, 143.12, 134.40, 133.86, 129.35, 127.26, 126.03, 113.69, 72.24, 59.84, 59.15, 55.15, 46.72, 41.35, 27.80, 21.39.

IR (ATR): $\tilde{\nu}$ (cm^{-1}) = 2946, 1613, 1514, 1462, 1337, 1303, 1248, 1160, 1094, 1023.

HRMS (ESI$^+$) calcd. for $C_{20}H_{25}N_2O_4S^+$ [M+H]$^+$: 389.1530, found: 389.1513.

6-Methoxy-1-(4-methoxyphenyl)-3-tosyl-3,6-diazabicyclo[3.2.0]heptane (13a)

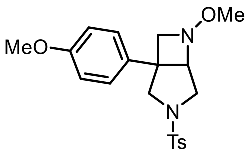

^{1}H NMR (500 MHz, DMSO-d$_6$, 75 °C) δ (ppm) = 7.71 (d, J = 7.9 Hz, 2H), 7.42 (d, J = 8.5 Hz, 2H), 7.09 (d, J = 8.7 Hz, 2H), 6.86 (d, J = 8.9 Hz, 2H), 4.32 (d, J = 5.1 Hz, 1H), 3.90 (d, J = 11.1 Hz, 1H), 3.87 (d, J = 9.0 Hz, 1H), 3.74 (d, J = 10.2 Hz, 1H), 3.73 (s, 3H), 3.66 (d, J = 9.0 Hz, 1H), 3.33 (s, 3H), 3.06-2.98 (m, 1H), 2.73 (d, J = 9.6 Hz, 1H), 2.39 (s, 3H).

^{13}C NMR (126 MHz, DMSO-d$_6$, 75 °C) δ (ppm) = 157.89, 143.19, 133.46, 132.39, 129.36, 127.26, 126.48, 113.83, 75.41, 65.29, 59.01, 57.85, 54.85, 50.41, 46.26, 20.58.

IR (ATR): $\tilde{\nu}$ (cm^{-1}) = 2939, 1612, 1598, 1516, 1464, 1345, 1297, 1249, 1181, 1097, 1030.

HRMS (ESI$^+$) calcd. for $C_{20}H_{25}N_2O_4S^+$ [M+H]$^+$: 389.1530, found: 389.1513.

1-(Furan-3-yl)-6-methoxy-3-tosyl-3,6-diazabicyclo[3.1.1]heptane (14)

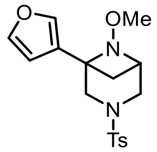

Prepared according to **General Procedure F** using oxime **S14b** (810 mg, 2.33 mmol, 1.00 equiv.), CH_3CN (23.3 mL), [Ir(dF(CF_3)ppy)$_2$(dtbbpy)](PF_6) (26.1 mg, 0.023 mmol, 1 mol%). Purification by flash column chromatography (pentane/ethyl acetate, 6:1 to 2:1 and CH_2Cl_2/ethyl acetate, 20:1 to 6:1) afforded **14** as a colorless oil (357 mg, 1.03 mmol, 44%) and **14a** as a colorless oil (195 mg, 0.56 mmol, 24%).

^{1}H NMR (500 MHz, $CDCl_3$) δ (ppm) = 7.70 (d, J = 8.0 Hz, 2H), 7.29 (t, J = 1.7 Hz, 1H), 7.28 (dd, J = 1.6, 1.0 Hz, 1H), 7.24 (d, J = 7.9 Hz, 2H), 6.32 (dd, J = 1.9, 0.9 Hz, 1H), 3.82 (ddt, J = 5.7, 2.8, 1.4 Hz, 1H), 3.58-3.53 (m, 2H), 3.49 (dd, J = 10.9, 1.7 Hz, 1H), 3.39 (dd, J = 10.9, 2.6 Hz, 1H), 3.13 (s, 3H), 2.33 (s, 3H), 1.94 (dd, J = 9.5, 1.2 Hz, 1H), 1.90 (dd, J = 9.5, 5.7 Hz, 1H).

^{13}C NMR (126 MHz, $CDCl_3$) δ (ppm) = 143.19, 143.14, 138.55, 134.27, 129.33, 127.20, 126.61, 108.20, 67.08, 60.40, 59.40, 45.02, 41.29, 28.47, 21.31.

IR (ATR): $\tilde{\nu}$ (cm^{-1}) = 2949, 1598, 1502, 1463, 1337, 1159, 1053, 1021, 1011.

HRMS (ESI$^+$) calcd. for $C_{17}H_{21}N_2O_4S^+$ [M+H]$^+$: 349.1217, found: 349.1204.

1-(Furan-3-yl)-6-methoxy-3-tosyl-3,6-diazabicyclo[3.2.0]heptane (14a)

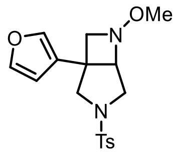

^{1}H NMR (500 MHz, DMSO-d$_6$, 75 °C) δ (ppm) = 7.73 (d, J = 8.2 Hz, 2H), 7.56-7.53 (m, 2H), 7.43 (d, J = 8.5 Hz, 2H), 6.38 (d, J = 0.9 Hz, 1H), 4.16 (d, J = 5.2 Hz, 1H), 3.85 (d, J = 11.1 Hz, 1H), 3.77 (dd, J = 9.0, 1.4 Hz, 1H), 3.68 (dd, J = 10.1, 2.7 Hz, 1H), 3.64 (d, J = 9.2 Hz, 1H), 3.33 (s, 3H), 2.97 (ddd, J = 10.8, 5.4, 2.3 Hz, 1H), 2.88 (d, J = 10.2 Hz, 1H), 2.40 (d, J = 2.3 Hz, 3H).

^{13}C NMR (126 MHz, DMSO-d$_6$, 75 °C) δ (ppm) = 143.52, 143.21, 138.59, 132.61, 129.36, 127.25, 125.79, 108.90, 75.98, 64.97, 59.04, 56.37, 50.02, 40.02, 20.59.

IR (ATR): $\tilde{\nu}$ (cm^{-1}) = 2940, 1597, 1504, 1465, 1343, 1160, 1046, 1023, 999.

HRMS (ESI$^+$) calcd. for $C_{17}H_{21}N_2O_4S^+$ [M+H]$^+$: 349.1217, found: 349.1204.

6-Methoxy-1-(thiophen-2-yl)-3-tosyl-3,6-diazabicyclo[3.1.1]heptane (15)

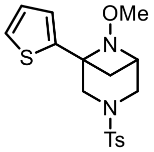

Prepared according to **General Procedure F** using oxime **S15b** (1.00 g, 2.75 mmol, 1.00 equiv.), CH_3CN (27.5 mL), [Ir(dF(CF_3)ppy)$_2$(dtbbpy)](PF_6) (30.8 mg, 0.027 mmol, 1 mol%). Purification by flash column chromatography (pentane/ethyl acetate, 6:1 to 2:1 and CH_2Cl_2/ethyl acetate, 20:1 to 6:1) afforded **15** as a pale-yellow oil (776 mg, 2.13 mmol, 78%).

^{1}H NMR (500 MHz, $CDCl_3$) δ (ppm) = 7.73 (d, J = 8.3 Hz, 2H), 7.27 (d, J = 7.9 Hz, 2H), 7.18 (dd, J = 5.1, 1.2 Hz, 1H), 6.90 (dd, J = 5.1, 3.6 Hz, 1H), 6.85 (dd, J = 3.6, 1.2 Hz, 1H), 3.87 (ddt, J = 5.8, 2.7, 1.1 Hz, 1H), 3.79 (d, J = 10.8 Hz, 1H), 3.64 (dd, J = 10.7, 1.2 Hz, 1H), 3.53 (dt, J = 10.8, 1.4 Hz, 1H), 3.43 (dd, J = 10.9, 2.7 Hz, 1H), 3.22 (s, 3H), 2.36 (s, 3H), 2.08 (dd, J = 9.5, 1.0 Hz, 1H), 2.00 (dd, J = 9.5, 6.0 Hz, 1H).

^{13}C NMR (126 MHz, $CDCl_3$) δ (ppm) = 145.43, 143.20, 134.22, 129.37, 127.23, 126.92, 125.02, 122.67, 69.87, 60.76, 59.17, 45.30, 41.15, 30.63, 21.37.

IR (ATR): $\tilde{\nu}$ (cm^{-1}) = 2956, 2811, 1597, 1439, 1336, 1248, 1159, 1091, 1050, 1022, 1009.

HRMS (ESI$^+$) calcd. for $C_{17}H_{21}N_2O_3S_2^+$ [M+H]$^+$: 365.0988, found: 365.0978.

6-Methoxy-1-(6-methoxypyridin-3-yl)-3-tosyl-3,6-diazabicyclo[3.1.1]heptane (16)

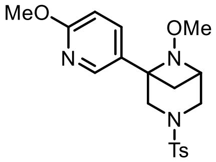

Prepared according to **General Procedure F** using oxime **S16b** (650 mg, 1.67 mmol, 1.00 equiv.), CH_3CN (16.7 mL), thioxanthone (35.4 mg, 0.17 mmol, 0.10 equiv.), 40W KSPR160L-390 nm Kessil light (100% intensity). Purification by flash column chromatography (pentane/ethyl acetate, 4:1 to 1:2 and CH_2Cl_2/ethyl acetate, 5:1 to 1:1) afforded **16** as a colorless oil (355 mg, 0.91 mmol, 55%) and **16a** as a colorless oil (103 mg, 0.26 mmol, 16%).

^{1}H NMR (500 MHz, $CDCl_3$) δ (ppm) = 8.07 (dd, J = 2.6, 0.7 Hz, 1H), 7.74 (d, J = 8.3 Hz, 2H), 7.47 (dd, J = 8.6, 2.5 Hz, 1H), 7.32 (d, J = 8.1 Hz, 2H), 6.70 (dd, J = 8.6, 0.7 Hz, 1H), 4.00-3.95 (m, 1H), 3.91 (s, 3H), 3.61 (d, J = 10.9 Hz, 1H), 3.57 (dd, J = 4.5, 1.5 Hz, 1H), 3.55 (dd, J = 4.2, 1.1 Hz, 1H), 3.51 (dd, J = 11.0, 2.6 Hz, 1H), 3.26 (s, 3H), 2.43 (s, 3H), 2.14 (d, J = 9.3 Hz, 1H), 1.98 (dd, J = 9.4, 6.0 Hz, 1H).

^{13}**C NMR** (126 MHz, $CDCl_3$) δ (ppm) = 163.57, 143.56, 143.20, 135.60, 134.21, 129.92, 129.35, 127.22, 110.45, 70.69, 59.85, 59.56, 53.29, 46.31, 41.23, 27.52, 21.35.

IR (ATR): $\tilde{\nu}$ (cm^{-1}) = 2948, 1606, 1570, 1493, 1461, 1377, 1337, 1285, 1254, 1159, 1092, 1022, 1010.

HRMS (ESI^+) calcd. for $C_{19}H_{24}N_3O_4S^+$ $[M+H]^+$: 390.1482, found: 390.1468.

6-Methoxy-1-(6-methoxypyridin-3-yl)-3-tosyl-3,6-diazabicyclo[3.2.0]heptane (16a)

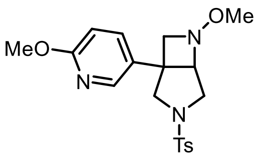

^{1}**H NMR** (500 MHz, DMSO-d$_6$, 75 °C) δ (ppm) = 8.00 (d, *J* = 2.6 Hz, 1H), 7.71 (d, *J* = 8.2 Hz, 2H), 7.53 (dd, *J* = 8.6, 2.6 Hz, 1H), 7.42 (d, *J* = 8.0 Hz, 2H), 6.74 (d, *J* = 8.5 Hz, 1H), 4.39 (d, *J* = 5.2 Hz, 1H), 3.90 (d, *J* = 11.5 Hz, 1H), 3.87 (d, *J* = 9.2 Hz, 1H), 3.83 (s, 3H), 3.79 (d, *J* = 10.1 Hz, 1H), 3.67 (d, *J* = 9.2 Hz, 1H), 3.33 (s, 3H), 3.04 (dd, *J* = 11.0, 5.2 Hz, 1H), 2.76 (d, *J* = 9.9 Hz, 1H), 2.40 (s, 3H).

^{13}**C NMR** (126 MHz, DMSO-d$_6$, 75 °C) δ (ppm) = 162.34, 143.83, 143.23, 136.71, 132.44, 129.82, 129.38, 127.27, 109.98, 75.06, 65.01, 59.05, 57.31, 52.72, 50.18, 44.63, 20.60.

IR (ATR): $\tilde{\nu}$ (cm^{-1}) = 2944, 1607, 1570, 1496, 1463, 1379, 1345, 1288, 1254, 1161, 1091, 1023.

HRMS (ESI^+) calcd. for $C_{19}H_{24}N_3O_4S^+$ $[M+H]^+$: 390.1482, found: 390.1468.

6-Methoxy-1-(2-methoxypyrimidin-5-yl)-3-tosyl-3,6-diazabicyclo[3.1.1]heptane (17)

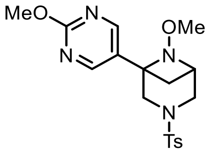

Prepared according to **General Procedure F** using oxime **S17b** (920 mg, 2.36 mmol, 1.00 equiv.), CH_3CN (23.6 mL), thioxanthone (50.0 mg, 0.24 mmol, 0.10 equiv.), 40W KSPR160L-390 nm Kessil light (100% intensity). Purification by flash column chromatography (pentane/ethyl acetate, 2:1 to 1:3 and CH_2Cl_2/ethyl acetate, 5:1 to 1:2) afforded **17** as a colorless oil (530 mg, 1.36 mmol, 58%) and **17a** as a pale-yellow oil (196 mg, 0.50 mmol, 21%).

^{1}**H NMR** (500 MHz, $CDCl_3$) δ (ppm) = 8.29 (s, 2H), 7.59 (d, *J* = 8.3 Hz, 2H), 7.17 (d, *J* = 8.4 Hz, 2H), 3.86 (dt, *J* = 5.7, 2.2 Hz, 1H), 3.82 (s, 3H), 3.50 (d, *J* = 10.9 Hz, 1H), 3.43 (dd, *J* = 10.9, 1.2 Hz, 1H), 3.40 (dd, *J* = 10.9, 1.7 Hz, 1H), 3.35 (dd, *J* = 11.1, 2.6 Hz, 1H), 3.09 (s, 3H), 2.26 (s, 3H), 2.02 (d, *J* = 9.5 Hz, 1H), 1.84 (dd, *J* = 9.5, 6.1 Hz, 1H).

^{13}**C NMR** (126 MHz, $CDCl_3$) δ (ppm) = 164.96, 156.27, 143.18, 133.87, 129.25, 127.87, 127.06, 68.94, 59.77, 59.74, 54.67, 45.51, 40.95, 27.45, 21.19.

IR (ATR): $\tilde{\nu}$ (cm^{-1}) = 2937, 1599, 1556, 1473, 1413, 1332, 1159, 1091, 1022, 1010.

HRMS (ESI^+) calcd. for $C_{18}H_{23}N_4O_4S^+$ $[M+H]^+$: 391.1435, found: 391.1418.

6-Methoxy-1-(2-methoxypyrimidin-5-yl)-3-tosyl-3,6-diazabicyclo[3.2.0]heptane (17a)

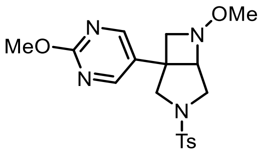

^{1}**H NMR** (500 MHz, DMSO-d_6, 75 °C) δ (ppm) = 8.47 (s, 2H), 7.71 (d, *J* = 8.2 Hz, 2H), 7.42 (d, *J* = 8.5 Hz, 2H), 4.49 (d, *J* = 4.9 Hz, 1H), 3.96-3.90 (m, 1H), 3.90 (s, 3H), 3.88 (d, *J* = 10.3 Hz, 1H), 3.85 (d, *J* = 10.2 Hz, 1H), 3.72 (d, *J* = 9.2 Hz, 1H), 3.33 (d, *J* = 0.7 Hz, 3H), 3.10-3.04 (m, 1H), 2.83 (dt, *J* = 10.1, 2.0 Hz, 1H), 2.39 (s, 3H).

^{13}**C NMR** (126 MHz, DMSO-d_6, 75 °C) δ (ppm) = 163.94, 156.97, 143.23, 132.55, 129.36, 127.93, 127.26, 74.75, 64.72, 59.07, 56.74, 54.13, 49.76, 43.01, 20.59.

IR (ATR): $\tilde{\nu}$ (cm^{-1}) = 2940, 1599, 1557, 1471, 1415, 1327, 1160, 1092, 1032, 1002.

HRMS (ESI^+) calcd. for $C_{18}H_{23}N_4O_4S^+$ $[M+H]^+$: 391.1435, found: 391.1419.

1-(Benzo[*d*][1,3]dioxol-5-yl)-6-methoxy-3-tosyl-3,6-diazabicyclo[3.1.1]heptane (18)

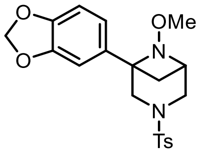

Prepared according to **General Procedure F** using oxime **S18b** (823 mg, 2.05 mmol, 1.00 equiv.), CH_3CN (20.5 mL), $[Ir(dF(CF_3)ppy)_2(dtbbpy)](PF_6)$ (23.0 mg, 0.020 mmol, 1 mol%). Purification by flash column chromatography (pentane/ethyl acetate, 6:1 to 2:1 and CH_2Cl_2/ethyl acetate, 20:1 to 6:1) afforded **18** as a colorless oil (563 mg, 1.40 mmol, 68%) and **18a** as a colorless oil (100 mg, 0.25 mmol, 12%).

^{1}**H NMR** (500 MHz, $CDCl_3$) δ (ppm) = 7.69 (d, *J* = 8.4 Hz, 2H), 7.24 (d, *J* = 8.4 Hz, 2H), 6.72 (d, *J* = 1.8 Hz, 1H), 6.67 (d, *J* = 8.0 Hz, 1H), 6.62 (dd, *J* = 8.1, 1.8 Hz, 1H), 5.84 (s, 2H), 3.87 (dd, *J* = 5.7, 2.5 Hz, 1H), 3.55-3.46 (m, 3H), 3.41 (dd, *J* = 11.0, 2.6 Hz, 1H), 3.20 (s, 3H), 2.34 (s, 3H), 2.01 (d, *J* = 9.3 Hz, 1H), 1.88 (dd, *J* = 9.4, 6.0 Hz, 1H).

^{13}**C NMR** (126 MHz, $CDCl_3$) δ (ppm) = 147.47, 146.79, 143.05, 135.65, 134.24, 129.26, 127.14, 117.97, 107.91, 105.59, 100.90, 72.31, 59.66, 58.90, 46.73, 41.19, 27.66, 21.27.

IR (ATR): $\tilde{\nu}$ (cm^{-1}) = 2947, 2878, 1598, 1505, 1488, 1440, 1337, 1230, 1159, 1092, 1037.

HRMS (ESI^+) calcd. for $C_{20}H_{23}N_2O_5S^+$ $[M+H]^+$: 403.1322, found: 403.1309.

1-(Benzo[*d*][1,3]dioxol-5-yl)-6-methoxy-3-tosyl-3,6-diazabicyclo[3.2.0]heptane (18a)

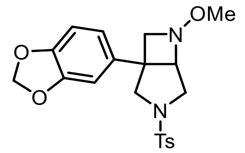

^{1}**H NMR** (500 MHz, DMSO-d_6, 75 °C) δ (ppm) = 7.70 (d, *J* = 8.3 Hz, 2H), 7.42 (d, *J* = 8.5 Hz, 2H), 6.79 (d, *J* = 8.0 Hz, 1H), 6.73 (d, *J* = 1.8 Hz, 1H), 6.62 (dd, *J* = 8.0, 1.9 Hz, 1H), 5.96 (s, 2H), 4.32 (d, *J* = 5.1 Hz, 1H), 3.91-3.83 (m, 2H), 3.73 (d, *J* = 10.0 Hz, 1H), 3.64 (d, *J* = 9.1 Hz, 1H), 3.32 (s, 3H), 3.04 (dd, *J* = 11.2, 5.3 Hz, 1H), 2.71 (d, *J* = 9.9 Hz, 1H), 2.40 (s, 3H).

^{13}**C NMR** (126 MHz, DMSO-d_6, 75 °C) δ (ppm) = 147.28, 145.72, 143.20, 135.39, 132.39, 129.35, 127.25, 118.42, 107.79, 106.13, 100.62, 75.31, 65.28, 59.01, 57.95, 50.51, 46.76, 20.59.

IR (ATR): $\tilde{\nu}$ (cm^{-1}) = 2939, 2894, 1598, 1506, 1490, 1440, 1345, 1233, 1163, 1096, 1036.

HRMS (ESI^+) calcd. for $C_{20}H_{23}N_2O_5S^+$ $[M+H]^+$: 403.1322, found: 403.1305.

Isopropyl 2-(4-(4-(6-methoxy-3-tosyl-3,6-diazabicyclo[3.1.1]heptan-1-yl)benzoyl)phenoxy)-2-methylpropanoate (19)

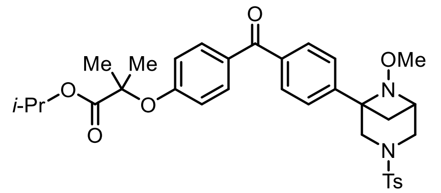

Prepared according to **General Procedure F** using oxime **S19b** (980 mg, 1.62 mmol, 1.00 equiv.), CH_3CN (16.2 mL), $[Ir(dF(CF_3)ppy)_2(dtbbpy)](PF_6)$ (18.1 mg, 0.016 mmol, 1 mol%). Purification by flash column chromatography (pentane/ethyl acetate, 5:1 to 1:1 and CH_2Cl_2/ethyl acetate, 9:1 to 4:1) afforded **19** as a colorless oil (638 mg, 1.05 mmol, 65%) and **19a** as a colorless oil (30 mg, 0.05 mmol, 3%).

^{1}**H NMR** (500 MHz, $CDCl_3$) δ (ppm) = 7.76 (d, *J* = 6.4 Hz, 2H), 7.74 (d, *J* = 6.9 Hz, 2H), 7.72 (d, *J* = 8.2 Hz, 2H), 7.37 (d, *J* = 8.2 Hz, 2H), 7.33 (d, *J* = 8.0 Hz, 2H), 6.87 (d, *J* = 8.9 Hz, 2H), 5.09 (hept, *J* = 6.3 Hz, 1H), 4.03-3.99 (m, 1H), 3.68 (d, *J* = 10.9 Hz, 1H), 3.63-3.57 (m, 2H), 3.53 (dd, *J* = 11.0, 2.6

Hz, 1H), 3.32 (s, 3H), 2.43 (s, 3H), 2.21 (d, J = 9.4 Hz, 1H), 2.03 (dd, J = 9.0, 6.4 Hz, 1H), 1.66 (s, 6H), 1.21 (s, 3H), 1.20 (s, 3H).

^{13}C NMR (126 MHz, $CDCl_3$) δ (ppm) = 194.84, 173.01, 159.57, 145.55, 143.30, 137.43, 134.32, 131.95, 130.41, 129.86, 129.45, 127.33, 124.86, 117.15, 79.33, 72.70, 69.25, 59.84, 59.36, 46.51, 41.37, 27.77, 25.34, 25.30, 21.47.

IR (ATR): $\tilde{\nu}$ (cm^{-1}) = 2986, 1729, 1652, 1464, 1339, 1276, 1259, 1159, 1100, 1022, 1011.

HRMS (ESI^{+}) calcd. for $C_{33}H_{39}N_2O_7S^+$ [M+H]$^+$: 607.2472, found: 607.2481.

Isopropyl 2-(4-(4-(6-methoxy-3-tosyl-3,6-diazabicyclo[3.2.0]heptan-1-yl)benzoyl)phenoxy)-2-methylpropanoate (19a)

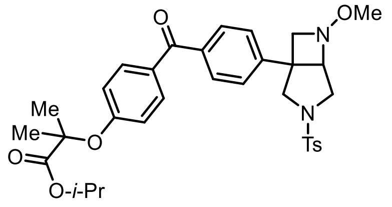

^{1}H NMR (500 MHz, DMSO-d$_6$, 75 °C) δ (ppm) = 7.72 (d, J = 8.4 Hz, 2H), 7.69 (d, J = 8.9 Hz, 2H), 7.62 (d, J = 8.4 Hz, 2H), 7.43 (d, J = 8.4 Hz, 2H), 7.35 (d, J = 8.2 Hz, 2H), 6.91 (d, J = 8.9 Hz, 2H), 4.99 (hept, J = 6.2 Hz, 1H), 4.45 (d, J = 5.1 Hz, 1H), 3.97-3.88 (m, 2H), 3.84 (d, J = 10.1 Hz, 1H), 3.73 (d, J = 9.2 Hz, 1H), 3.35 (s, 3H), 3.11-3.05 (m, 1H), 2.82 (d, J = 9.5 Hz, 1H), 2.40 (s, 3H), 1.61 (s, 6H), 1.18 (s, 3H), 1.17 (s, 3H).

^{13}C NMR (126 MHz, DMSO-d$_6$, 75 °C) δ (ppm) = 193.48, 171.73, 158.86, 145.44, 143.26, 136.01, 132.41, 131.23, 129.96, 129.42, 129.24, 127.28, 125.59, 117.30, 79.04, 75.35, 68.55, 65.07, 59.11, 57.33, 47.02, 24.80, 20.88, 20.60.

IR (ATR): $\tilde{\nu}$ (cm^{-1}) = 2989, 1726, 1652, 1464, 1340, 1276, 1261, 1164, 1102, 1023, 996.

HRMS (ESI^{+}) calcd. for $C_{33}H_{39}N_2O_7S^+$ [M+H]$^+$: 607.2472, found: 607.2459.

Ethyl 2-(4-(6-methoxy-3-tosyl-3,6-diazabicyclo[3.1.1]heptan-1-yl)phenoxy)-2-methylpropanoate (20)

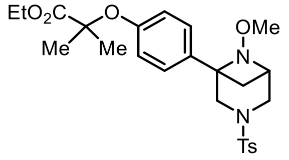

Prepared according to **General Procedure F** using oxime **S20b** (890 mg, 1.82 mmol, 1.00 equiv.), CH_3CN (18.2 mL), [Ir(dF(CF_3)ppy)$_2$(dtbbpy)](PF_6) (20.4 mg, 0.018 mmol, 1 mol%). Purification by flash column chromatography (pentane/ethyl acetate, 6:1 to 1:1 and CH_2Cl_2/ethyl acetate, 20:1 to 5:1) afforded **20** as a colorless oil (479 mg, 0.98 mmol, 54%) and **20a** as a colorless oil (106 mg, 0.22 mmol, 12%).

^{1}H NMR (500 MHz, $CDCl_3$) δ (ppm) = 7.69 (d, *J* = 8.2 Hz, 2H), 7.26 (d, *J* = 8.1 Hz, 2H), 7.08 (d, *J* = 8.7 Hz, 2H), 6.74 (d, *J* = 8.7 Hz, 2H), 4.17 (q, *J* = 7.2 Hz, 2H), 3.90 (dd, *J* = 5.5, 2.5 Hz, 1H), 3.55 (d, *J* = 10.9 Hz, 1H), 3.52 (d, *J* = 7.9 Hz, 1H), 3.50 (d, *J* = 7.8 Hz, 1H), 3.43 (dd, *J* = 10.9, 2.6 Hz, 1H), 3.22 (s, 3H), 2.36 (s, 3H), 2.05 (d, *J* = 9.3 Hz, 1H), 1.91 (dd, *J* = 9.4, 6.0 Hz, 1H), 1.52 (s, 6H), 1.19 (t, *J* = 7.2 Hz, 3H).

^{13}C NMR (126 MHz, $CDCl_3$) δ (ppm) = 174.05, 154.87, 143.15, 135.29, 134.43, 129.38, 127.28, 125.74, 118.84, 79.02, 72.33, 61.35, 59.83, 59.21, 46.75, 41.35, 27.70, 25.27, 25.22, 21.43, 14.01.

IR (ATR): $\tilde{\nu}$ (cm^{-1}) = 2986, 1731, 1610, 1510, 1464, 1338, 1276, 1261, 1160, 1022, 1011.

HRMS (ESI$^+$) calcd. for $C_{25}H_{33}N_2O_6S^+$ [M+H]$^+$: 489.2054, found: 489.2053.

Ethyl 2-(4-(6-methoxy-3-tosyl-3,6-diazabicyclo[3.2.0]heptan-1-yl)phenoxy)-2-methylpropanoate (20a)

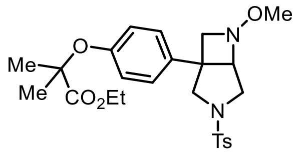

^{1}H NMR (500 MHz, DMSO-d$_6$, 75 °C) δ (ppm) = 7.71 (d, *J* = 8.9 Hz, 2H), 7.42 (d, *J* = 8.2 Hz, 2H), 7.08 (d, *J* = 8.9 Hz, 2H), 6.78-6.73 (m, 2H), 4.32 (dd, *J* = 5.1, 2.4 Hz, 1H), 4.16 (q, *J* = 7.0 Hz, 2H), 3.90 (d, *J* = 11.1 Hz, 1H), 3.86 (dd, *J* = 9.1, 2.1 Hz, 1H), 3.73 (dd, *J* = 10.1, 2.5 Hz, 1H), 3.66 (d, *J* = 9.0 Hz, 1H), 3.32 (s, 3H), 3.01 (d, *J* = 10.7 Hz, 1H), 2.72 (d, *J* = 11.3 Hz, 1H), 2.39 (s, 3H), 1.50 (s, 6H), 1.17 (t, *J* = 7.1 Hz, 3H).

^{13}C NMR (126 MHz, DMSO-d$_6$, 75 °C) δ (ppm) = 172.68, 153.63, 143.21, 135.03, 132.34, 129.38, 127.29, 126.33, 118.97, 78.61, 75.37, 65.27, 60.60, 59.02, 57.64, 50.21, 46.30, 24.74, 20.61, 13.47.

IR (ATR): $\tilde{\nu}$ (cm^{-1}) = 2989, 1730, 1654, 1512, 1467, 1344, 1276, 1261, 1165, 1024, 1003.

HRMS (ESI$^+$) calcd. for $C_{25}H_{33}N_2O_6S^+$ [M+H]$^+$: 489.2054, found: 489.2051.

6-Methoxy-1-(4-(5-(*p*-tolyl)-3-(trifluoromethyl)-1*H*-pyrazol-1-yl)phenyl)-3-tosyl-3,6-diazabicyclo[3.1.1]heptane (21)

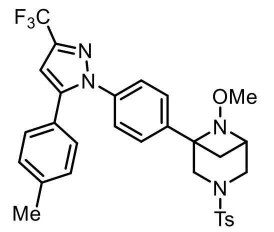

Prepared according to **General Procedure F** using oxime **S21b** (1.12 g, 1.92 mmol, 1.00 equiv.), CH_3CN (19.2 mL), [Ir(dF(CF_3)ppy)$_2$(dtbbpy)](PF_6) (21.6 mg, 0.019 mmol, 1 mol%). Purification by flash column chromatography (pentane/ethyl acetate, 6:1 to 1:1 and CH_2Cl_2/ethyl acetate, 20:1 to 6:1) afforded **21** as a colorless oil (860 mg, 1.48 mmol, 77%).

^{1}H NMR (500 MHz, $CDCl_3$) δ (ppm) = 7.74 (d, J = 8.3 Hz, 2H), 7.30 (d, J = 7.8 Hz, 2H), 7.26 (d, J = 8.9 Hz, 2H), 7.23 (d, J = 9.0 Hz, 2H), 7.11 (d, J = 7.9 Hz, 2H), 7.08 (d, J = 8.4 Hz, 2H), 6.68 (s, 1H), 3.96 (dt, J = 6.0, 2.2 Hz, 1H), 3.62-3.56 (m, 2H), 3.55 (d, J = 10.8 Hz, 1H), 3.49 (dd, J = 11.0, 2.6 Hz, 1H), 3.26 (s, 3H), 2.39 (s, 3H), 2.32 (s, 3H), 2.13 (d, J = 9.3 Hz, 1H), 1.96 (dd, J = 9.4, 6.0 Hz, 1H).

^{13}C NMR (126 MHz, $CDCl_3$) δ (ppm) = 144.71, 143.30, 142.98 (q, J = 38.0 Hz), 141.90, 139.09, 138.64, 134.30, 129.41, 128.53, 127.24, 125.98, 125.71, 125.17, 121.27 (q, J = 268.9 Hz), 105.34 (q, J = 2.5 Hz), 72.33, 59.72, 59.26, 46.53, 41.26, 27.58, 21.33, 21.12.

^{19}F NMR (471 MHz, $CDCl_3$) δ (ppm) = −62.05 (s).

IR (ATR): $\tilde{\nu}$ (cm^{-1}) = 2950, 1598, 1509, 1471, 1449, 1377, 1339, 1271, 1259, 1131, 1096, 1022.

HRMS (ESI$^+$) calcd. for $C_{30}H_{30}F_3N_4O_3S^+$ [M+H]$^+$: 583.1985, found: 583.1968.

Ethyl 2-(4-(2-(4-(6-methoxy-3-tosyl-3,6-diazabicyclo[3.1.1]heptan-1-yl)benzamido)ethyl)phenoxy)-2-methylpropanoate (22)

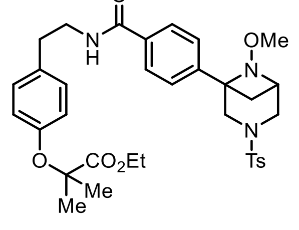

Prepared according to **General Procedure F** using oxime **S22b** (782 mg, 1.23 mmol, 1.00 equiv.), CH_3CN (12.3 mL), [Ir(dF(CF_3)ppy)$_2$(dtbbpy)](PF_6) (13.8 mg, 0.012 mmol, 1 mol%). Purification by flash column chromatography (pentane/ethyl acetate, 3:1 to 1:2 and CH_2Cl_2/ethyl acetate, 6:1 to 1:1) afforded **22** as a colorless oil (554 mg, 0.87 mmol, 71%).

^{1}H NMR (500 MHz, $CDCl_3$) δ (ppm) = 7.69-7.64 (m, 4H), 7.27 (d, J = 8.4 Hz, 2H), 7.22 (d, J = 8.2 Hz, 2H), 7.02 (d, J = 8.5 Hz, 2H), 6.75-6.69 (m, 3H), 4.17 (q, J = 6.9 Hz, 2H), 3.94-3.89 (m, 1H),

3.59-3.49 (m, 5H), 3.44 (dt, *J* = 11.0, 2.1 Hz, 1H), 3.23 (s, 3H), 2.78 (t, *J* = 7.2 Hz, 2H), 2.37 (s, 3H), 2.08 (d, *J* = 9.3 Hz, 1H), 1.90 (dd, *J* = 9.4, 6.2 Hz, 1H), 1.52 (s, 6H), 1.18 (t, *J* = 6.9 Hz, 3H).

^{13}C NMR (126 MHz, $CDCl_3$) δ (ppm) = 174.12, 166.87, 153.81, 144.76, 143.31, 134.11, 133.83, 132.57, 129.40, 129.32, 127.19, 127.05, 124.99, 119.38, 78.98, 72.44, 61.27, 59.76, 59.19, 46.40, 41.28, 41.17, 34.61, 27.63, 25.22, 21.38, 13.95.

IR (ATR): $\tilde{\nu}$ (cm^{-1}) = 2987, 1731, 1643, 1539, 1506, 1463, 1337, 1276, 1261, 1232, 1159, 1141, 1022.

HRMS (ESI^+) calcd. for $C_{34}H_{42}N_3O_7S^+$ $[M+H]^+$: 636.2738, found: 636.2760.

Methyl 2-(5-methoxy-1-(4-(6-methoxy-3-tosyl-3,6-diazabicyclo[3.1.1]heptan-1-yl)benzoyl)-2-methyl-1H-indol-3-yl)acetate (23)

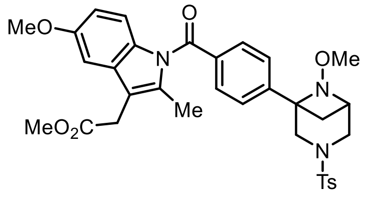

Prepared according to **General Procedure F** using oxime **S23b** (650 mg, 1.05 mmol, 1.00 equiv.), CH_3CN (10.5 mL), [Ir(dF(CF_3)ppy)$_2$(dtbbpy)](PF_6) (11.8 mg, 0.011 mmol, 1 mol%). Purification by flash column chromatography (pentane/ethyl acetate, 3:1 to 1:1 and CH_2Cl_2/ethyl acetate, 9:1 to 3:1) afforded **23** as a colorless oil (396 mg, 0.64 mmol, 61%).

^{1}H NMR (500 MHz, $CDCl_3$) δ (ppm) = 7.73 (d, *J* = 8.3 Hz, 2H), 7.65 (d, *J* = 8.4 Hz, 2H), 7.35 (d, *J* = 8.4 Hz, 2H), 7.30 (d, *J* = 8.0 Hz, 2H), 6.94 (d, *J* = 2.6 Hz, 1H), 6.89 (d, *J* = 9.0 Hz, 1H), 6.64 (dd, *J* = 9.0, 2.6 Hz, 1H), 3.98 (dt, *J* = 4.8, 2.1 Hz, 1H), 3.80 (s, 3H), 3.66 (s, 3H), 3.65-3.58 (m, 4H), 3.55 (dd, *J* = 10.8, 1.7 Hz, 1H), 3.50 (dd, *J* = 11.0, 2.6 Hz, 1H), 3.29 (s, 3H), 2.40 (s, 3H), 2.32 (s, 3H), 2.20 (d, *J* = 9.4 Hz, 1H), 2.01 (dd, *J* = 9.4, 6.1 Hz, 1H).

^{13}C NMR (126 MHz, $CDCl_3$) δ (ppm) = 171.32, 168.83, 155.91, 146.60, 143.36, 135.87, 134.85, 134.28, 130.84, 130.52, 129.89, 129.48, 127.29, 125.37, 114.94, 112.24, 111.47, 101.18, 72.68, 59.81, 59.38, 55.62, 52.05, 46.51, 41.31, 30.04, 27.68, 21.46, 13.34.

IR (ATR): $\tilde{\nu}$ (cm^{-1}) = 2989, 1737, 1609, 1477, 1457, 1354, 1275, 1261, 1224, 1161, 1070, 1012.

HRMS (ESI^+) calcd. for $C_{33}H_{36}N_3O_7S^+$ $[M+H]^+$: 618.2268, found: 618.2269.

1-(Cyclohex-1-en-1-yl)-6-methoxy-3-tosyl-3,6-diazabicyclo[3.1.1]heptane (24)

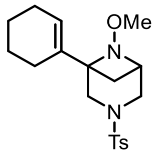

Prepared according to **General Procedure F** using oxime **S24b** (724 mg, 2.00 mmol, 1.00 equiv.), CH_3CN (20.0 mL), [Ir(dF(CF_3)ppy)$_2$(dtbbpy)](PF_6) (22.4 mg, 0.02 mmol, 1 mol%). Purification by flash column chromatography (pentane/ethyl acetate, 5:1 to 1:1) afforded **24** as a colorless oil (540 mg, 1.49 mmol, 75%).

^{1}H NMR (500 MHz, $CDCl_3$) δ (ppm) = 7.63 (d, *J* = 8.3 Hz, 2H), 7.21 (d, *J* = 8.5 Hz, 2H), 5.47 (tt, *J* = 3.8, 1.7 Hz, 1H), 3.72 (ddt, *J* = 5.8, 3.1, 1.6 Hz, 1H), 3.38 (dd, *J* = 10.8, 1.7 Hz, 1H), 3.34 (dd, *J* = 10.7, 1.0 Hz, 1H), 3.30 (d, *J* = 3.6 Hz, 1H), 3.28 (dd, *J* = 7.2, 3.6 Hz, 1H), 3.10 (s, 3H), 2.31 (s, 3H), 1.92-1.86 (m, 2H), 1.82-1.75 (m, 2H), 1.73-1.66 (m, 2H), 1.53-1.38 (m, 4H).

^{13}C NMR (126 MHz, $CDCl_3$) δ (ppm) = 142.90, 137.30, 134.42, 129.21, 127.11, 122.61, 73.93, 59.31, 58.29, 45.26, 41.45, 25.87, 24.67, 23.87, 22.29, 21.90, 21.29.

IR (ATR): $\tilde{\nu}$ (cm^{-1}) = 2930, 2877, 1598, 1439, 1336, 1159, 1092, 1049, 1023.

HRMS (ESI$^+$) calcd. for $C_{19}H_{27}N_2O_3S^+$ [M+H]$^+$: 363.1737, found: 363.1721.

1-(Cyclopent-1-en-1-yl)-6-methoxy-3-tosyl-3,6-diazabicyclo[3.1.1]heptane (25)

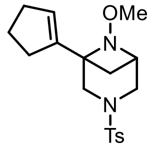

Prepared according to **General Procedure F** using oxime **S25b** (220 mg, 0.63 mmol, 1.00 equiv.), CH_3CN (6.3 mL), [Ir(dF(CF_3)ppy)$_2$(dtbbpy)](PF_6) (7.1 mg, 6.3 μmol, 1 mol%). Purification by flash column chromatography (pentane/ethyl acetate, 5:1 to 2:1) afforded **25** as a colorless oil (151 mg, 0.43 mmol, 69%).

^{1}H NMR (500 MHz, $CDCl_3$) δ (ppm) = 7.70 (d, *J* = 8.4 Hz, 2H), 7.27 (d, *J* = 8.0 Hz, 2H), 5.51 (t, *J* = 2.1 Hz, 1H), 3.81-3.77 (m, 1H), 3.49-3.45 (m, 2H), 3.44 (d, *J* = 10.9 Hz, 1H), 3.36 (dd, *J* = 10.9, 2.6 Hz, 1H), 3.16 (s, 3H), 2.38 (s, 3H), 2.31-2.22 (m, 4H), 1.87-1.76 (m, 4H).

^{13}C NMR (126 MHz, $CDCl_3$) δ (ppm) = 143.74, 143.11, 134.59, 129.39, 127.33, 126.30, 70.65, 60.02, 59.30, 44.81, 41.59, 32.61, 31.75, 26.51, 23.11, 21.48.

IR (ATR): $\tilde{\nu}$ (cm^{-1}) = 2944, 1598, 1441, 1336, 1304, 1276, 1261, 1159, 1093, 1053, 1023.

HRMS (ESI$^+$) calcd. for $C_{18}H_{25}N_2O_3S^+$ [M+H]$^+$: 349.1580, found: 349.1571.

6-Methoxy-3-tosyl-1-vinyl-3,6-diazabicyclo[3.1.1]heptane (26)

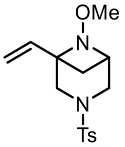

Prepared according to **General Procedure F** using oxime **S26b** (1.08 g, 3.50 mmol, 1.00 equiv.), CH_3CN (35.0 mL), [Ir(dF(CF_3)ppy)$_2$(dtbbpy)](PF_6) (39.3 mg, 0.035 mmol, 1 mol%). Purification by flash column chromatography (pentane/ethyl acetate, 5:1 to 1:1 and CH_2Cl_2/ethyl acetate, 9:1 to 4:1) afforded **26** as a colorless oil (916 mg, 2.97 mmol, 85%).

Note: When thioxanthone is used as the photosensitizer (10 mol%), the yield of this substrate decreases (70%), and we recommend terminating the reaction after 2 hours, as prolonged reaction times lead to the formation of minor unidentified side products.

^{1}H NMR (500 MHz, $CDCl_3$) δ (ppm) = 7.71 (d, J = 8.4 Hz, 2H), 7.28 (d, J = 8.0 Hz, 2H), 5.76 (dd, J = 17.5, 10.8 Hz, 1H), 5.23 (dd, J = 17.5, 1.1 Hz, 1H), 5.12 (dd, J = 10.9, 1.2 Hz, 1H), 3.83-3.77 (m, 1H), 3.47 (dd, J = 10.2, 1.6 Hz, 1H), 3.41 (d, J = 10.8 Hz, 1H), 3.40-3.36 (m, 2H), 3.18 (s, 3H), 2.39 (s, 3H), 1.84 (dd, J = 9.4, 1.0 Hz, 1H), 1.78 (dd, J = 9.5, 5.8 Hz, 1H).

^{13}C NMR (126 MHz, $CDCl_3$) δ (ppm) = 143.23, 137.78, 134.51, 129.45, 127.39, 116.16, 71.41, 60.44, 59.34, 45.01, 41.68, 27.54, 21.54.

IR (ATR): $\tilde{\nu}$ (cm^{-1}) = 2948, 2877, 1598, 1462, 1336, 1276, 1260, 1158, 1092, 1053, 1022.

HRMS (ESI$^+$) calcd. for $C_{15}H_{21}N_2O_3S^+$ [M+H]$^+$: 309.1267, found: 309.1267.

(6-Methoxy-3-tosyl-3,6-diazabicyclo[3.1.1]heptan-1-yl)(naphthalen-2-yl)methanone (27)

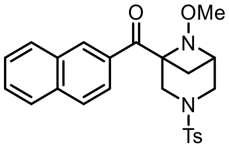

Prepared according to **General Procedure F** using oxime **S27b** (315 mg, 0.72 mmol, 1.00 equiv.), CH_3CN (7.2 mL), [Ir(dF(CF_3)ppy)$_2$(dtbbpy)](PF_6) (8.1 mg, 7.2 μmol, 1 mol%). Purification by flash column chromatography (pentane/ethyl acetate, 6:1 to 2:1) afforded **27** as a white solid (69 mg, 0.16 mmol, 22%) and **27a** as a colorless oil (47 mg, 0.11 mmol, 15%).

Note: The reaction time of this substrate is 5 h, and 22% of the raw material is recovered. Long reaction time (e.g. 24 h) will lead to the decomposition of the product.

M.P.: 150-152 °C

^{1}H NMR (500 MHz, $CDCl_3$) δ (ppm) = 8.48 (d, *J* = 1.8 Hz, 1H), 7.96 (dd, *J* = 8.7, 1.7 Hz, 1H), 7.92 (d, *J* = 8.1 Hz, 1H), 7.90-7.85 (m, 2H), 7.75 (d, *J* = 8.1 Hz, 2H), 7.62 (ddd, *J* = 8.1, 6.9, 1.3 Hz, 1H), 7.56 (td, *J* = 7.4, 1.2 Hz, 1H), 7.33 (d, *J* = 8.0 Hz, 2H), 3.98-3.94 (m, 2H), 3.70 (d, *J* = 11.3 Hz, 1H), 3.66 (dd, *J* = 11.0, 1.6 Hz, 1H), 3.57 (dd, *J* = 11.1, 2.5 Hz, 1H), 3.42 (s, 3H), 2.44 (s, 3H), 2.42 (d, *J* = 9.4 Hz, 1H), 2.37 (dd, *J* = 9.6, 5.7 Hz, 1H).

^{13}C NMR (126 MHz, CD_2Cl_2) δ (ppm) = 197.53, 144.14, 135.99, 134.68, 132.69, 131.89, 131.54, 130.08, 129.96, 129.30, 128.88, 128.14, 127.78, 127.34, 124.81, 77.92, 60.35, 59.83, 44.32, 42.22, 27.76, 21.67.

IR (ATR): $\tilde{\nu}$ (cm^{-1}) = 2967, 1674, 1629, 1597, 1465, 1354, 1276, 1261, 1159, 1094, 1029.

HRMS (ESI^{+}) calcd. for $C_{24}H_{25}N_2O_4S^+$ [M+H]$^{+}$: 437.1530, found: 437.1534.

(6-Methoxy-3-tosyl-3,6-diazabicyclo[3.2.0]heptan-1-yl)(naphthalen-2-yl)methanone (27a)

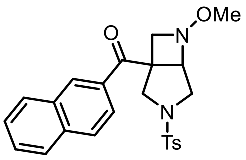

^{1}H NMR (500 MHz, DMSO-d$_6$, 75 °C) δ (ppm) = 8.26 (d, *J* = 1.8 Hz, 1H), 8.15 (dd, *J* = 8.1, 1.2 Hz, 1H), 8.00 (app. t, *J* = 7.7 Hz, 2H), 7.77 (dd, *J* = 8.7, 1.8 Hz, 1H), 7.71-7.67 (m, 3H), 7.64 (ddd, *J* = 8.1, 6.9, 1.3 Hz, 1H), 7.39 (d, *J* = 8.5 Hz, 2H), 4.52 (d, *J* = 4.6 Hz, 1H), 4.26 (d, *J* = 9.6 Hz, 1H), 4.21 (d, *J* = 10.7 Hz, 1H), 3.76 (d, *J* = 11.0 Hz, 1H), 3.72 (d, *J* = 9.6 Hz, 1H), 3.34 (s, 3H), 3.04-2.98 (m, 2H), 2.38 (s, 3H).

^{13}C NMR (126 MHz, DMSO-d$_6$, 75 °C) δ (ppm) = 196.54, 143.32, 134.99, 132.38, 131.86, 130.35, 130.21, 129.47, 129.41, 128.74, 128.44, 127.29, 127.24, 126.66, 123.46, 74.38, 62.41, 59.29, 54.29, 52.42, 51.64, 20.59.

IR (ATR): $\tilde{\nu}$ (cm^{-1}) = 2989, 1672, 1626, 1597, 1464, 1348, 1276, 1261, 1162, 1094, 1028.

HRMS (ESI^{+}) calcd. for $C_{24}H_{25}N_2O_4S^+$ [M+H]$^{+}$: 437.1530, found: 437.1536.

(6-Methoxy-3-tosyl-3,6-diazabicyclo[3.1.1]heptan-1-yl)(phenyl)methanone (28)

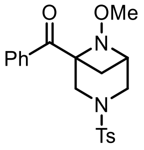

Prepared according to **General Procedure F** using oxime **S28b** (386 mg, 1.00 mmol, 1.00 equiv.), CH_3CN (10.0 mL), $[Ir(dF(CF_3)ppy)_2(dtbbpy)](PF_6)$ (11.2 mg, 0.01 mmol, 1 mol%). Purification by flash column chromatography (pentane/ethyl acetate, 6:1 to 2:1 and CH_2Cl_2/ethyl acetate, 20:1 to 6:1) afforded **28** as a colorless oil (55 mg, 0.14 mmol, 14%) and **28a** as a colorless oil (11 mg, 0.03 mmol, 3%).

Note: The reaction time of this substrate is 24 h, and 41% of the raw material is recovered.

^{1}H NMR (500 MHz, $CDCl_3$) δ (ppm) = 7.93-7.90 (m, 2H), 7.72 (d, *J* = 8.2 Hz, 2H), 7.60-7.54 (m, 1H), 7.47-7.41 (m, 2H), 7.32 (d, *J* = 8.0 Hz, 2H), 3.91 (ddt, *J* = 5.6, 2.9, 1.5 Hz, 1H), 3.87 (d, *J* = 11.2 Hz, 1H), 3.62-3.57 (m, 2H), 3.52 (dd, *J* = 11.1, 2.6 Hz, 1H), 3.38 (s, 3H), 2.43 (s, 3H), 2.32 (dd, *J* = 9.5, 1.3 Hz, 1H), 2.29 (dd, *J* = 9.6, 5.5 Hz, 1H).

^{13}C NMR (126 MHz, $CDCl_3$) δ (ppm) = 197.17, 143.62, 134.28, 133.97, 133.74, 129.64, 129.17, 128.82, 127.51, 77.64, 60.36, 59.61, 43.73, 41.78, 27.44, 21.63.

IR (ATR): $\tilde{\nu}$ (cm^{-1}) = 3005, 2988, 1681, 1597, 1449, 1340, 1276, 1261, 1161, 1023.

HRMS (ESI$^+$) calcd. for $C_{20}H_{23}N_2O_4S^+$ [M+H]$^+$: 387.1373, found: 387.1377.

(6-Methoxy-3-tosyl-3,6-diazabicyclo[3.2.0]heptan-1-yl)(phenyl)methanone (28a)

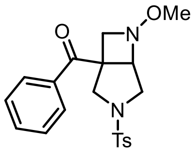

^{1}H NMR (500 MHz, DMSO-d$_6$, 75 °C) δ (ppm) = 7.70-7.64 (m, 5H), 7.54-7.50 (m, 2H), 7.41 (d, *J* = 7.9 Hz, 2H), 4.46 (d, *J* = 4.6 Hz, 1H), 4.09 (d, *J* = 9.6 Hz, 1H), 4.04 (d, *J* = 10.7 Hz, 1H), 3.72 (d, *J* = 11.1 Hz, 1H), 3.62 (d, *J* = 9.6 Hz, 1H), 3.31 (s, 3H), 3.01-2.95 (m, 2H), 2.40 (s, 3H).

^{13}C NMR (126 MHz, DMSO-d$_6$, 75 °C) δ (ppm) = 196.55, 143.35, 133.49, 133.09, 132.52, 129.43, 128.73, 128.25, 127.22, 74.27, 62.30, 59.29, 54.21, 52.32, 51.54, 20.62.

IR (ATR): $\tilde{\nu}$ (cm^{-1}) = 3005, 2989, 1677, 1599, 1463, 1348, 1276, 1261, 1164, 1093.

HRMS (ESI$^+$) calcd. for $C_{20}H_{23}N_2O_4S^+$ [M+H]$^+$: 387.1373, found: 387.1376.

4-((6-Methoxy-1-phenyl-3,6-diazabicyclo[3.1.1]heptan-3-yl)sulfonyl)benzonitrile (29)

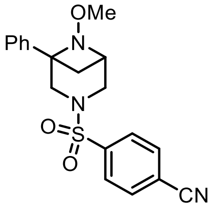

Prepared according to **General Procedure F** using oxime **S29b** (640 mg, 1.73 mmol, 1.00 equiv.), CH_3CN (17.3 mL), [Ir(dF(CF_3)ppy)$_2$(dtbbpy)](PF_6) (19.5 mg, 0.017 mmol, 1 mol%). Purification by flash column chromatography (pentane/ethyl acetate, 5:1 to 1:1 and CH_2Cl_2/ethyl acetate, 15:1 to 8:1) afforded **29** as a colorless oil (476 mg, 1.29 mmol, 74%) and **29a** as a colorless oil (60 mg, 0.16 mmol, 9%).

^{1}H NMR (500 MHz, $CDCl_3$) δ (ppm) = 7.95 (d, J = 8.5 Hz, 2H), 7.80 (d, J = 8.5 Hz, 2H), 7.33-7.28 (m, 2H), 7.27-7.20 (m, 3H), 3.97 (dt, J = 5.7, 2.2 Hz, 1H), 3.65 (d, J = 10.9 Hz, 1H), 3.56-3.48 (m, 3H), 3.26 (s, 3H), 2.15 (d, J = 9.5 Hz, 1H), 2.01 (dd, J = 9.5, 6.1 Hz, 1H).

^{13}C NMR (126 MHz, $CDCl_3$) δ (ppm) = 141.22, 141.19, 132.60, 128.38, 127.81, 127.72, 124.74, 117.35, 116.00, 72.58, 60.01, 59.01, 46.99, 41.63,27.63.

IR (ATR): $\tilde{\nu}$ (cm^{-1}) = 2950, 2233, 1448, 1398, 1343, 1284, 1162, 1092, 1050, 1012.

HRMS (ESI$^+$) calcd. for $C_{19}H_{20}N_3O_3S^+$ [M+H]$^+$: 370.1220, found: 370.1232.

4-((6-Methoxy-1-phenyl-3,6-diazabicyclo[3.2.0]heptan-3-yl)sulfonyl)benzonitrile (29a)

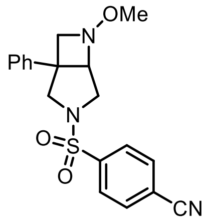

^{1}H NMR (500 MHz, DMSO-d$_6$, 75 °C) δ (ppm) = 8.07 (d, J = 8.7 Hz, 2H), 8.01 (d, J = 8.2 Hz, 2H), 7.31 (dd, J = 8.2, 7.0 Hz, 2H), 7.25-7.21 (m, 1H), 7.20-7.17 (m, 2H), 4.38 (d, J = 5.0 Hz, 1H), 3.95 (d, J = 11.2 Hz, 1H), 3.87 (d, J = 10.3 Hz, 1H), 3.84 (dd, J = 9.0, 1.1 Hz, 1H), 3.67 (d, J = 9.0 Hz, 1H), 3.31 (s, 3H), 3.22 (dd, J = 11.2, 5.1 Hz, 1H), 2.94 (d, J = 10.3 Hz, 1H).

^{13}C NMR (126 MHz, DMSO-d$_6$, 75 °C) δ (ppm) = 141.34, 140.06, 132.99, 128.26, 127.90, 126.47, 125.35, 117.18, 115.32, 75.66, 65.11, 59.11, 57.85, 50.80, 46.90.

IR (ATR): $\tilde{\nu}$ (cm^{-1}) = 2941, 2233, 1448, 1399, 1352, 1281, 1166, 1090, 1054, 1004.

HRMS (ESI$^+$) calcd. for $C_{19}H_{20}N_3O_3S^+$ [M+H]$^+$: 370.1220, found: 370.1234.

The synthetic route to compound 30 has been described in detail in Section 1.14 ("The synthesis of the analogue of DB04232") and is not repeated here.

1-(4-Chlorophenyl)-6-methoxy-3-oxa-6-azabicyclo[3.1.1]heptane (31)

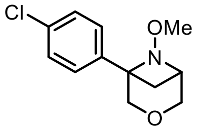

Prepared according to **General Procedure F** using oxime **S31b** (220 mg, 0.92 mmol, 1.00 equiv.), DMSO (9.2 mL), [Ir(dF(CF_3)ppy)$_2$(dtbbpy)](PF_6) (10.3 mg, 9.2 μmol, 1 mol%). Purification by flash column chromatography (pentane/ethyl acetate, 30:1 to 5:1) afforded **31** as a colorless oil (145 mg, 0.61 mmol, 66%).

Note: The reaction time of this substrate is 20 h.

^{1}H NMR (500 MHz, $CDCl_3$) δ (ppm) = 7.19 (d, J = 8.5 Hz, 2H), 7.11 (d, J = 8.6 Hz, 2H), 4.34-4.27 (m, 2H), 3.82 (dd, J = 6.1, 2.4 Hz, 1H), 3.57 (d, J = 10.5 Hz, 1H), 3.54 (dd, J = 10.6, 2.3 Hz, 1H), 3.51 (s, 3H), 2.27 (d, J = 8.8 Hz, 1H), 1.94 (dd, J = 8.7, 6.1 Hz, 1H).

^{13}C NMR (126 MHz, $CDCl_3$) δ (ppm) = 140.18, 133.30, 128.54, 126.56, 74.41, 65.84, 61.88, 60.56, 59.97, 26.08.

IR (ATR): $\tilde{\nu}$ (cm^{-1}) = 2931, 1496, 1451, 1362, 1277, 1260, 1009.

HRMS (ESI$^+$) calcd. for $C_{12}H_{15}ClNO_2^+$ [M+H]$^+$: 240.0786, found: 240.0784.

6-(Benzyloxy)-1-phenyl-3-oxa-6-azabicyclo[3.1.1]heptane (32)

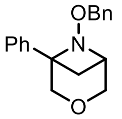

Prepared according to **General Procedure F** using oxime **S32b** (260 mg, 0.92 mmol, 1.00 equiv.), CH_3CN (9.2 mL), [Ir(dF(CF_3)ppy)$_2$(dtbbpy)](PF_6) (10.4 mg, 9.2 μmol, 1 mol%). Purification by flash column chromatography (pentane/ethyl acetate, 6:1 to 4:1 and CH_2Cl_2/ethyl acetate, 100:1 to 20:1) afforded **32** as a colorless oil (125 mg, 0.44 mmol, 48%) and **32a** as a colorless oil (20 mg, 0.07 mmol, 8%).

Note: The reaction time of this substrate is 20 h.

^{1}H NMR (500 MHz, $CDCl_3$) δ (ppm) = 7.46-7.43 (m, 2H), 7.40-7.28 (m, 8H), 4.93 (d, J = 11.6 Hz, 1H), 4.87 (d, J = 11.7 Hz, 1H), 4.61 (dd, J = 10.6, 1.2 Hz, 1H), 4.48 (d, J = 10.4 Hz, 1H), 3.79 (d, J = 10.5 Hz, 1H), 3.69 (dd, J = 6.0, 2.3 Hz, 1H), 3.65 (dd, J = 10.4, 2.3 Hz, 1H), 2.41 (d, J = 8.7 Hz, 1H), 2.12 (dd, J = 8.7, 6.0 Hz, 1H).

^{13}**C NMR** (126 MHz, $CDCl_3$) δ (ppm) = 141.79, 138.29, 128.55, 128.41, 128.37, 127.81, 127.48, 125.18, 75.32, 75.15, 66.32, 63.09, 61.19, 26.42.

IR (ATR): $\tilde{\nu}$ (cm^{-1}) = 2933, 2871, 1495, 1454, 1364, 1276, 1261, 1011.

HRMS (ESI^+) calcd. for $C_{18}H_{20}NO_2^+$ $[M+H]^+$: 282.1489, found: 282.1478.

6-(Benzyloxy)-1-phenyl-3-oxa-6-azabicyclo[3.2.0]heptane (32a)

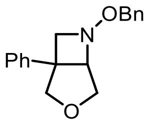

^{1}**H NMR** (500 MHz, DMSO-d_6, 75 °C) δ (ppm) = 7.38-7.31 (m, 6H), 7.30-7.23 (m, 4H), 4.58 (s, 2H), 4.42 (d, *J* = 4.4 Hz, 1H), 4.35 (br. s, 1H), 4.00 (d, *J* = 9.0 Hz, 1H), 3.82 (s, 2H), 3.64 (dd, *J* = 10.3, 4.4 Hz, 1H), 3.37 (d, *J* = 9.0 Hz, 1H).

^{13}**C NMR** (126 MHz, DMSO-d_6, 75 °C) δ (ppm) = 140.90, 138.01, 128.18, 127.80, 127.77, 127.12, 126.19, 125.76, 77.52, 76.13, 73.72, 69.19, 65.63, 48.77.

IR (ATR): $\tilde{\nu}$ (cm^{-1}) = 2929, 2852, 1496, 1453, 1366, 1276, 1260, 1007.

HRMS (ESI^+) calcd. for $C_{18}H_{20}NO_2^+$ $[M+H]^+$: 282.1489, found: 282.1476.

1-([1,1'-Biphenyl]-4-yl)-6-methoxy-6-azabicyclo[3.1.1]heptane (33)

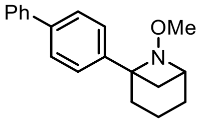

Prepared according to **General Procedure F** using oxime **S33b** (100 mg, 0.36 mmol, 1.00 equiv.), DMSO (3.6 mL), [Ir(dF(CF_3)ppy)$_2$(dtbbpy)](PF_6) (4.0 mg, 3.6 μmol, 1 mol%). Purification by flash column chromatography (pentane/ethyl acetate, 25:1 to 10:1) afforded **33** as a colorless oil (41 mg, 0.15 mmol, 41%).

Note: The reaction time of this substrate is 20 h and the solvent is DMSO.

^{1}**H NMR** (500 MHz, $CDCl_3$) δ (ppm) = 7.62-7.58 (m, 2H), 7.57 (d, *J* = 8.3 Hz, 2H), 7.46-7.42 (m, 2H), 7.41 (d, *J* = 8.3 Hz, 2H), 7.36-7.32 (m, 1H), 4.02 (dt, *J* = 5.5, 2.5 Hz, 1H), 3.62 (s, 3H), 2.44 (dtd, *J* = 14.1, 9.0, 1.7 Hz, 1H), 2.26 (dtt, *J* = 13.9, 8.8, 1.6 Hz, 1H), 2.15 (dd, *J* = 9.3, 1.0 Hz, 1H), 2.02-1.98 (m, 1H), 1.97-1.90 (m, 1H), 1.84-1.76 (m, 2H), 1.58 (dddd, *J* = 13.9, 9.3, 3.3, 1.3 Hz, 1H).

^{13}**C NMR** (126 MHz, $CDCl_3$) δ (ppm) = 145.97, 141.25, 139.59, 128.82, 127.20, 127.07, 125.25, 74.94, 61.69, 59.90, 27.72, 27.24, 19.85, 14.83.

IR (ATR): $\tilde{\nu}$ (cm^{-1}) = 2935, 1601, 1487, 1439, 1339, 1276, 1261, 1184, 1049, 1006.

HRMS (ESI$^+$) calcd. for $C_{19}H_{22}NO^+$ [M+H]$^+$: 280.1696, found: 280.1688.

6-Methoxy-1-phenyl-3-tosyl-3,6-diazabicyclo[3.1.1]heptan-2-one (34)

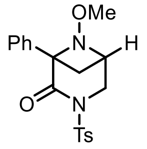

Prepared according to **General Procedure F** using oxime **S34b** (640 mg, 1.72 mmol, 1.00 equiv.), CH_3CN (17.2 mL), [Ir(dF(CF_3)ppy)$_2$(dtbbpy)](PF_6) (19.3 mg, 0.017 mmol, 1 mol%). Purification by flash column chromatography (pentane/ethyl acetate, 5:1 to 1:1) afforded **34** as a white solid (470 mg, 1.26 mmol, 73%).

M.P.: 148-150 °C

^{1}H NMR (500 MHz, $CDCl_3$) δ (ppm) = 7.92 (d, J = 8.4 Hz, 2H), 7.33-7.22 (m, 7H), 4.35-4.31 (m, 2H), 4.09-4.04 (m, 1H), 3.49 (s, 3H), 2.38 (s, 3H), 2.24-2.22 (m, 2H).

^{13}C NMR (126 MHz, $CDCl_3$) δ (ppm) = 169.73, 144.74, 136.45, 135.76, 129.23, 128.46, 127.96, 127.93, 126.37, 78.63, 61.03, 59.23, 45.95, 26.44, 21.59.

IR (ATR): $\tilde{\nu}$ (cm^{-1}) = 1715, 1596, 1461, 1448, 1353, 1240, 1188, 1167, 1113, 1060, 1037, 1020, 805.

HRMS (ESI$^+$) calcd. for $C_{19}H_{21}N_2O_4S^+$ [M+H]$^+$: 373.1217, found: 373.1207.

3-Benzyl-6-methoxy-5-phenyl-3,6-diazabicyclo[3.1.1]heptan-2-one (35)

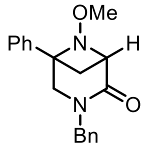

Prepared according to **General Procedure F** using oxime **S35b** (250 mg, 0.81 mmol, 1.00 equiv.), CH_3CN (8.1 mL), [Ir(dF(CF_3)ppy)$_2$(dtbbpy)](PF_6) (9.1 mg, 8.1 μmol, 1 mol%). Purification by flash column chromatography (pentane/ethyl acetate, 5:1 to 1:1) afforded **35** as a pale-yellow oil (205 mg, 0.67 mmol, 82%).

^{1}H NMR (500 MHz, $CDCl_3$) δ (ppm) = 7.36-7.25 (m, 10H), 4.80 (d, J = 14.9 Hz, 1H), 4.58 (d, J = 15.0 Hz, 1H), 4.28 (d, J = 5.6 Hz, 1H), 3.70 (d, J = 11.7 Hz, 1H), 3.62 (s, 3H), 3.33 (d, J = 11.9 Hz, 1H), 2.22 (dd, J = 9.2, 5.8 Hz, 1H), 2.13 (d, J = 9.3 Hz, 1H).

^{13}**C NMR** (126 MHz, $CDCl_3$) δ (ppm) =169.69, 140.67, 136.55, 128.51, 128.50, 128.03, 127.95, 127.40, 124.75, 72.96, 65.78, 60.67, 48.94, 48.10, 28.13.

IR (ATR): $\tilde{\nu}$ (cm^{-1}) = 1670, 1495, 1452, 1428, 1275, 1219, 1189, 1075, 1038, 1028, 758.

HRMS (ESI^+) calcd. for $C_{19}H_{21}N_2O_2^+$ $[M+H]^+$: 309.1598, found: 309.1588.

3-Benzyl-6-methoxy-1-phenyl-3,6-diazabicyclo[3.1.1]heptane-2,4-dione (36)

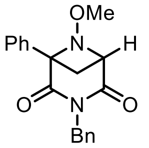

Prepared according to **General Procedure F** using oxime **S36b** (385 mg, 1.20 mmol, 1.00 equiv.), CH_3CN (12.0 mL), $[Ir(dF(CF_3)ppy)_2(dtbbpy)](PF_6)$ (13.5 mg, 0.012 mmol, 1 mol%). Purification by flash column chromatography (pentane/ethyl acetate, 5:1 to 2:1) afforded **36** as a white solid (301 mg, 0.93 mmol, 78%).

Note: Trace amounts of the side-product (1,2-fused azetidines) were observed in the crude NMR, but this minor isomer was not isolated through flash column chromatography.

M.P.: 92-94 °C

^{1}**H NMR** (500 MHz, $CDCl_3$) δ (ppm) = 7.46-7.37 (m, 5H), 7.36-7.26 (m, 5H), 5.02 (d, *J* = 14.3 Hz, 1H), 4.98 (d, *J* = 14.4 Hz, 1H), 4.56 (dd, *J* = 6.0, 0.8 Hz, 1H), 3.66 (s, 3H), 2.63 (d, *J* = 9.6 Hz, 1H), 2.51 (dd, *J* = 9.6, 6.0 Hz, 1H).

^{13}**C NMR** (126 MHz, $CDCl_3$) δ (ppm) = 170.88, 170.46, 136.67, 136.50, 128.67, 128.56, 128.46, 128.39, 127.54, 126.31, 79.54, 67.23, 61.44, 42.30, 30.84.

IR (ATR): $\tilde{\nu}$ (cm^{-1}) = 1752, 1691, 1498, 1448, 1429, 1385, 1350, 1313, 1283, 1142, 1036, 1025, 1003.

HRMS (ESI^+) calcd. for $C_{19}H_{19}N_2O_3^+$ $[M+H]^+$: 323.1390, found: 323.1379.

3-Benzyl-6-methoxy-1-methyl-5-phenyl-3,6-diazabicyclo[3.1.1]heptan-2-one (37)

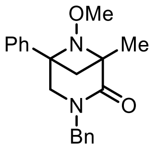

Prepared according to **General Procedure F** using oxime **S37b** (438 mg, 1.36 mmol, 1.00 equiv.), CH_3CN (13.6 mL), $[Ir(dF(CF_3)ppy)_2(dtbbpy)](PF_6)$ (15.3 mg, 0.014 mmol, 1 mol%). Purification by

flash column chromatography (pentane/ethyl acetate, 5:1 to 1:1) afforded **37** as a pale-yellow oil (340 mg, 1.06 mmol, 78%).

^{1}H NMR (500 MHz, $CDCl_3$) δ (ppm) 7.38-7.26 (m, 10H), 4.84 (d, *J* = 15.0 Hz, 1H), 4.58 (d, *J* = 14.9 Hz, 1H), 3.73 (d, *J* = 11.6 Hz, 1H), 3.69 (s, 3H), 3.34 (d, *J* = 11.6 Hz, 1H), 2.26 (d, *J* = 9.1 Hz, 1H), 2.02 (d, *J* = 9.1 Hz, 1H), 1.64 (s, 3H).

^{13}C NMR (126 MHz, $CDCl_3$) δ (ppm) = 171.44, 141.20, 136.87, 128.53, 128.51, 128.08, 127.86, 127.34, 124.88, 69.96, 69.80, 62.59, 50.33, 48.43, 34.05, 19.46.

IR (ATR): $\tilde{\nu}$ (cm^{-1}) = 1667, 1495, 1483, 1453, 1376, 1231, 1197, 1041, 911, 763, 732.

HRMS (ESI^+) calcd. for $C_{20}H_{23}N_2O_2^+$ $[M+H]^+$: 323.1754, found: 323.1739.

3-Benzyl-6-methoxy-1-methyl-5-phenyl-3,6-diazabicyclo[3.1.1]heptane-2,4-dione (38)

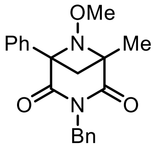

Prepared according to **General Procedure F** using oxime **S38b** (168 mg, 0.50 mmol, 1.00 equiv.), CH_3CN (5.0 mL), $[Ir(dF(CF_3)ppy)_2(dtbbpy)](PF_6)$ (5.6 mg, 5.0 µmol, 1 mol%). Purification by flash column chromatography (pentane/ethyl acetate, 5:1 to 2:1 and pentane/CH_2Cl_2, 3:1 to 1:1 to 100% CH_2Cl_2) afforded **38** as a colorless oil (110 mg, 0.33 mmol, 65%) and **38a** as a colorless oil (7.0 mg, 0.02 mmol, 4%).

^{1}H NMR (500 MHz, $CDCl_3$) δ (ppm) = 7.46-7.25 (m, 10H), 5.02 (d, *J* = 14.3 Hz, 1H), 4.99 (d, *J* = 14.3 Hz, 1H), 3.71 (s, 3H), 2.69 (d, *J* = 9.5 Hz, 1H), 2.30 (d, *J* = 9.6 Hz, 1H), 1.64 (s, 3H).

^{13}C NMR (126 MHz, $CDCl_3$) δ (ppm) = 172.35, 171.16, 136.85, 136.81, 128.71, 128.49, 128.46, 128.42, 127.50, 126.45, 76.95, 71.98, 63.20, 42.59, 36.59, 19.16.

IR (ATR): $\tilde{\nu}$ (cm^{-1}) = 1750, 1693, 1498, 1449, 1389, 1378, 1349, 1318, 1275, 1225, 1166, 1143, 1037, 1001, 750.

HRMS (ESI^+) calcd. for $C_{20}H_{21}N_2O_3^+$ $[M+H]^+$: 337.1547, found: 337.1534.

6-Methoxy-5-methyl-1-phenyl-3-tosyl-3,6-diazabicyclo[3.2.0]heptane-2,4-dione (38a)

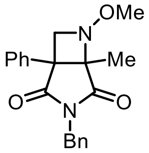

^{1}**H NMR** (500 MHz, $CDCl_3$) δ (ppm) = 7.46-6.99 (m, 10H), 4.89 (d, *J* = 14.2 Hz, 1H), 4.80 (d, *J* = 14.2 Hz, 1H), 4.31 (br. s, 1H), 3.88 (br. s, 1H), 3.53 (s, 3H), 1.22 (s, 3H).

^{13}**C NMR** (126 MHz, $CDCl_3$) δ (ppm) = 177.67, 135.70, 128.96, 128.74, 128.35, 128.21, 127.95, 127.30, 62.17, 49.89, 42.97.

IR (ATR): $\tilde{\nu}$ (cm^{-1}) = 1708, 1388, 1345, 1276, 1132, 750.

HRMS (ESI^+) calcd. for $C_{20}H_{21}N_2O_3^+$ $[M+H]^+$: 337.1547, found: 337.1534.

1,3-Dibenzyl-6-methoxy-5-phenyl-3,6-diazabicyclo[3.1.1]heptane-2,4-dione (39)

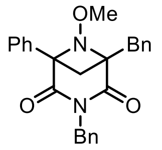

Prepared according to **General Procedure F** using oxime **S39b** (730 mg, 1.77 mmol, 1.00 equiv.), CH_3CN (17.7 mL), $[Ir(dF(CF_3)ppy)_2(dtbbpy)](PF_6)$ (19.9 mg, 0.018 mmol, 1 mol%). Purification by flash column chromatography (pentane/ethyl acetate, 6:1 to 2:1) afforded **39** as a colorless oil (497 mg, 1.21 mmol, 68%) and **39a** as a colorless oil (120 mg, 0.29 mmol, 16%).

^{1}**H NMR** (500 MHz, $CDCl_3$) δ (ppm) = 7.51-7.27 (m, 15H), 5.09 (d, *J* = 14.3 Hz, 1H), 5.04 (d, *J* = 14.3 Hz, 1H), 3.72 (s, 3H), 3.55 (d, *J* = 14.6 Hz, 1H), 3.28 (d, *J* = 14.5 Hz, 1H), 2.52 (d, *J* = 9.6 Hz, 1H), 2.17 (d, *J* = 9.7 Hz, 1H).

^{13}**C NMR** (126 MHz, $CDCl_3$) δ (ppm) = 172.01, 170.90, 136.81, 136.61, 135.54, 130.59, 128.67, 128.45, 128.39, 128.38, 128.31, 127.49, 126.88, 126.49, 76.63, 74.98, 63.00, 42.78, 37.48, 34.15.

IR (ATR): $\tilde{\nu}$ (cm^{-1}) = 1749, 1692, 1496, 1448, 1383, 1350, 1315, 1144, 1034, 1001, 734.

HRMS (ESI^+) calcd. for $C_{26}H_{25}N_2O_3^+$ $[M+H]^+$: 413.1860, found: 413.1845.

3,5-Dibenzyl-6-methoxy-1-phenyl-3,6-diazabicyclo[3.2.0]heptane-2,4-dione (39a)

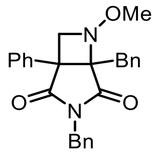

^{1}**H NMR** (500 MHz, $CDCl_3$) δ (ppm) = 7.39-7.25 (m, 8H), 7.20-7.16 (m, 2H), 7.03-6.98 (m, 1H), 6.86-6.80 (m, 2H), 6.35-6.30 (m, 2H), 4.83 (s, 2H), 4.37 (d, *J* = 10.1 Hz, 1H), 3.72 (d, *J* = 10.1 Hz, 1H), 3.65 (s, 3H), 3.46 (d, *J* = 14.0 Hz, 1H), 3.01 (d, *J* = 14.0 Hz, 1H).

^{13}C NMR (126 MHz, $CDCl_3$) δ (ppm) = 176.83, 172.74, 135.36, 132.63, 132.07, 130.20, 128.72, 128.71, 128.61, 128.26, 128.17, 127.88, 127.79, 126.63, 79.81, 65.45, 62.48, 49.01, 42.89, 38.00.

IR (ATR): $\tilde{\nu}$ (cm^{-1}) = 1706, 1497, 1455, 1389, 1348, 1133, 1029, 966, 751, 698.

HRMS (ESI^+) calcd. for $C_{26}H_{25}N_2O_3^+$ $[M+H]^+$: 413.1860, found: 413.1843.

3-benzyl-1-cyclopropyl-6-methoxy-5-phenyl-3,6-diazabicyclo[3.1.1]heptane-2,4-dione (40)

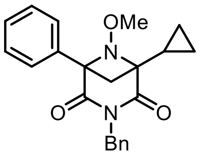

Prepared according to **General Procedure F** using oxime **S40b** (760 mg, 2.10 mmol, 1.00 equiv.), CH_3CN (21.0 mL), $[Ir(dF(CF_3)ppy)_2(dtbbpy)](PF_6)$ (23.6 mg, 0.021 mmol, 1 mol%). Purification by flash column chromatography (pentane/Et_2O, 6:1 to 2:1) afforded **40** as a colorless oil (501 mg, 1.38 mmol, 66%) and **40a** as a colorless oil (144 mg, 0.40 mmol, 19%).

^{1}H NMR (500 MHz, $CDCl_3$) δ (ppm) = 7.50-7.32 (m, 9H), 7.31-7.26 (m, 1H), 5.06 (d, J = 14.4 Hz, 1H), 5.03 (d, J = 14.4 Hz, 1H), 3.68 (s, 3H), 2.51 (d, J = 9.6 Hz, 1H), 2.03 (d, J = 9.6 Hz, 1H), 1.62 (tt, J = 8.5, 5.4 Hz, 1H), 0.79-0.66 (m, 2H), 0.60 (dtd, J = 9.6, 5.4, 4.4 Hz, 1H), 0.47 (dtd, J = 9.7, 5.6, 4.2 Hz, 1H).

^{13}C NMR (126 MHz, $CDCl_3$) δ (ppm) = 171.91, 170.85, 136.83, 136.65, 128.65, 128.36, 128.26, 127.39, 126.53, 75.66, 75.12, 62.35, 42.57, 33.29, 10.94, 1.48, 1.36.

IR (ATR): $\tilde{\nu}$ (cm^{-1}) = 1750, 1692, 1448, 1378, 1349, 1315, 1276, 1261, 1143, 1034.

HRMS (ESI^+) calcd. for $C_{22}H_{23}N_2O_3^+$ $[M+H]^+$: 363.1703, found: 363.1694.

3-Benzyl-5-cyclopropyl-6-methoxy-1-phenyl-3,6-diazabicyclo[3.2.0]heptane-2,4-dione (40a)

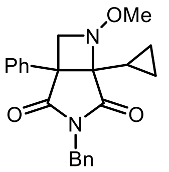

^{1}H NMR (500 MHz, $CDCl_3$) δ (ppm) = 7.46-7.42 (m, 2H), 7.40-7.29 (m, 6H), 7.26-7.23 (m, 2H), 4.88 (d, J = 14.1 Hz, 1H), 4.79 (d, J = 14.2 Hz, 1H), 4.52 (d, J = 10.4 Hz, 1H), 3.68 (d, J = 10.4 Hz, 1H), 3.54 (s, 3H), 0.94 – 0.83 (m, 1H), 0.62 (tt, J = 8.8, 4.4 Hz, 1H), 0.42 (tdd, J = 8.8, 6.0, 4.7 Hz, 1H), 0.38-0.32 (m, 1H), 0.33-0.24 (m, 1H).

^{13}**C NMR** (126 MHz, $CDCl_3$) δ 177.73, 172.30, 135.86, 132.39, 128.82, 128.68, 128.54, 128.17, 128.10, 128.04, 79.23, 61.79, 61.74, 51.03, 42.96, 11.51, 1.91, 1.38.

IR (ATR): $\tilde{\nu}$ (cm^{-1}) = 1776, 1704, 1498, 1457, 1430, 1386, 1345, 1276, 1127, 1074.

HRMS (ESI^+) calcd. for $C_{22}H_{22}N_2O_3Na^+$ $[M+Na]^+$: 385.1523, found: 385.1513.

3-Benzyl-6-methoxy-1,5-diphenyl-3,6-diazabicyclo[3.1.1]heptane-2,4-dione (41)

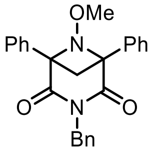

Prepared according to **General Procedure F** using oxime **S41b** (671 mg, 1.69 mmol, 1.00 equiv.), CH_3CN (16.9 mL), [Ir(dF(CF_3)ppy)$_2$(dtbbpy)](PF_6) (18.9 mg, 0.017 mmol, 1 mol%). Purification by flash column chromatography (pentane/CH_2Cl_2, 3:1 to 1:1 to 100% CH_2Cl_2) afforded **41** as a white solid (480 mg, 1.21 mmol, 72%) and **41a** as a colorless oil (110 mg, 0.28 mmol, 16%).

M.P.: 125-127 °C

^{1}**H NMR** (500 MHz, $CDCl_3$) δ (ppm) = 7.61-7.34 (m, 15H), 5.17 (s, 2H), 3.89 (s, 3H), 3.08 (d, J = 9.6 Hz, 1H), 2.72 (d, J = 9.7 Hz, 1H).

^{13}**C NMR** (126 MHz, $CDCl_3$) δ (ppm) = 170.67, 136.71, 136.26, 128.82, 128.49, 128.37, 128.29, 127.47, 126.62, 76.51, 62.93, 42.86, 35.77.

IR (ATR): $\tilde{\nu}$ (cm^{-1}) = 1750, 1693, 1497, 1448, 1382, 1351, 1320, 1276, 1266, 1209, 1145, 1034, 999, 908, 728.

HRMS (ESI^+) calcd. for $C_{25}H_{23}N_2O_3^+$ $[M+H]^+$: 399.1703, found: 399.1690.

3-Benzyl-6-methoxy-1,5-diphenyl-3,6-diazabicyclo[3.2.0]heptane-2,4-dione (41a)

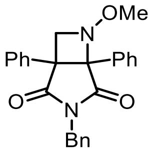

^{1}**H NMR** (500 MHz, $CDCl_3$) δ (ppm) = 7.59-7.56 (m, 2H), 7.44-7.34 (m, 3H), 7.18-7.04 (m, 10H), 5.07 (d, J = 14.0 Hz, 1H), 4.98 (d, J = 14.0 Hz, 1H), 4.61 (d, J = 10.3 Hz, 1H), 3.86 (d, J = 10.3 Hz, 1H), 3.65 (s, 3H).

^{13}**C NMR** (126 MHz, $CDCl_3$) δ (ppm) = 177.20, 172.66, 135.85, 134.54, 131.40, 128.91, 128.70, 128.35, 128.28, 128.19, 128.11, 128.03, 127.82, 127.14, 82.00, 63.05, 61.78, 52.50, 43.40.

IR (ATR): $\tilde{\nu}$ (cm^{-1}) = 1776, 1703, 1497, 1448, 1431, 1385, 1344, 1176, 1136, 1027, 909, 720.

HRMS (ESI^+) calcd. for $C_{25}H_{22}N_2O_3Na^+$ $[M+Na]^+$: 421.1523, found: 421.1505.

3-Benzyl-6-methoxy-1-phenyl-5-(4-(5-(*p*-tolyl)-3-(trifluoromethyl)-1*H*-pyrazol-1-yl)phenyl)-3,6-diazabicyclo[3.1.1]heptane-2,4-dione (42)

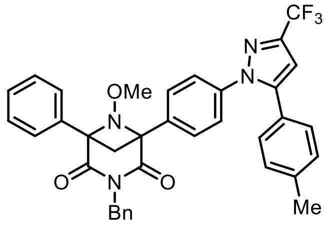

Prepared according to **General Procedure F** using oxime **S42b** (480 mg, 0.77 mmol, 1.00 equiv.), CH_3CN (7.7 mL), $[Ir(dF(CF_3)ppy)_2(dtbbpy)](PF_6)$ (8.7 mg, 7.8 μmol, 1 mol%). Purification by flash column chromatography (CH_2Cl_2/ethyl acetate, 50:1 to 10:1) afforded **42** as a colorless oil (335 mg, 0.54 mmol, 70%).

Note: Minor amounts of 1,2-fused azetidine product was detected in the crude ^1H NMR spectrum (NMR yield: 13%). However, due to difficulties in achieving complete separation by column chromatography, 1,2-fused azetidine product was not further purified and are therefore not reported herein.

^{1}H NMR (500 MHz, $CDCl_3$) δ (ppm) = 7.52-7.41 (m, 11H), 7.36-7.31 (m, 2H), 7.31-7.27 (m, 1H), 7.19 (s, 4H), 6.76 (s, 1H), 5.08 (s, 2H), 3.80 (s, 3H), 3.05 (d, *J* = 9.6 Hz, 1H), 2.64 (d, *J* = 9.6 Hz, 1H), 2.39 (s, 3H).

^{13}C NMR (126 MHz, $CDCl_3$) δ (ppm) = 170.62, 170.33, 144.91, 143.38 (q, *J* = 38.3 Hz), 139.47, 139.31, 136.69, 136.37, 136.15, 129.61, 128.81, 128.77, 128.54, 128.52, 127.75, 127.64, 126.67, 126.22, 125.11, 121.39 (q, *J* = 268.9 Hz), 105.73 (q, *J* = 2.2 Hz), 76.65, 76.10, 63.15, 43.04, 36.27, 21.36.

^{19}F NMR (471 MHz, $CDCl_3$) δ (ppm) = -62.10 (s).

IR (ATR): $\tilde{\nu}$ (cm^{-1}) = 1751, 1695, 1472, 1449, 1377, 1320, 1273, 1235, 1158, 1132.

HRMS (ESI^+) calcd. for $C_{36}H_{30}F_3N_4O_3^+$ $[M+H]^+$: 623.2265, found: 623.2265.

3-Benzyl-6-methoxy-1-(6-methoxypyridin-3-yl)-5-phenyl-3,6-diazabicyclo[3.1.1]heptane-2,4-dione (43)

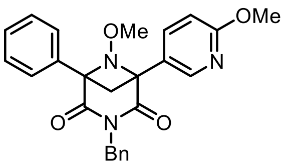

Prepared according to **General Procedure F** using oxime **S43b** (860 mg, 2.00 mmol, 1.00 equiv.), CH_3CN (20.0 mL), [Ir(dF(CF_3)ppy)$_2$(dtbbpy)](PF_6) (22.44 mg, 0.02 mmol, 1 mol%). Purification by flash column chromatography (CH_2Cl_2/Et_2O, 50:1 to 10:1) afforded **43** as a colorless oil (574 mg, 1.34 mmol, 67%).

Note: Minor amounts of 1,2-fused azetidine product was detected in the crude ^{1}H NMR spectrum (NMR yield: 12%). However, due to difficulties in achieving complete separation by column chromatography, 1,2-fused azetidine product was not further purified and are therefore not reported herein.

^{1}H NMR (500 MHz, $CDCl_3$) δ (ppm) = 8.31 (dd, J = 2.5, 0.8 Hz, 1H), 7.69 (dd, J = 8.7, 2.6 Hz, 1H), 7.50 – 7.43 (m, 6H), 7.42 – 7.38 (m, 1H), 7.35 – 7.26 (m, 3H), 6.83 (dd, J = 8.6, 0.7 Hz, 1H), 5.08 (s, 2H), 3.98 (s, 3H), 3.78 (s, 3H), 3.01 (d, J = 9.5 Hz, 1H), 2.65 (d, J = 9.6 Hz, 1H).

^{13}C NMR (126 MHz, $CDCl_3$) δ (ppm) = 170.49, 170.43, 164.17, 145.35, 137.55, 136.58, 136.10, 128.77, 128.59, 128.40, 128.36, 127.53, 126.55, 124.75, 110.56, 76.77, 74.71, 63.01, 53.52, 42.92, 35.71.

IR (ATR): $\tilde{\nu}$ (cm^{-1}) = 1751, 1693, 1608, 1497, 1380, 1317, 1285, 1260, 1146, 1020.

HRMS (ESI$^+$) calcd. for $C_{25}H_{24}N_3O_4^+$ [M+H]$^+$: 430.1761, found: 430.1765.

3-Benzyl-1-(furan-2-yl)-6-methoxy-5-phenyl-3,6-diazabicyclo[3.1.1]heptane-2,4-dione (44)

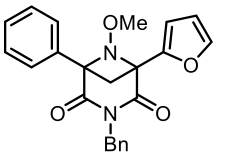

Prepared according to **General Procedure F** using oxime **S44b** (625 mg, 1.61 mmol, 1.00 equiv.), CH_3CN (16.1 mL), [Ir(dF(CF_3)ppy)$_2$(dtbbpy)](PF_6) (18.1 mg, 0.016 mmol, 1 mol%). Purification by flash column chromatography (pentane/ethyl acetate, 6:1 to 2:1) afforded **44** as a colorless oil (285 mg, 0.73 mmol, 46%).

Note: No 1,2-fused azetidine products were detected in the crude ^{1}H NMR spectrum.

^{1}**H NMR** (500 MHz, $CDCl_3$) δ (ppm) = 7.53 (dd, *J* = 1.8, 0.9 Hz, 1H), 7.51 – 7.44 (m, 4H), 7.43 – 7.40 (m, 3H), 7.37 – 7.33 (m, 2H), 7.32 – 7.27 (m, 1H), 6.92 (dd, *J* = 3.4, 0.9 Hz, 1H), 6.48 (dd, *J* = 3.4, 1.8 Hz, 1H), 5.11 (d, *J* = 14.3 Hz, 1H), 5.07 (d, *J* = 14.3 Hz, 1H), 3.68 (s, 3H), 3.00 (d, *J* = 9.6 Hz, 1H), 2.95 (d, *J* = 9.6 Hz, 1H).

^{13}**C NMR** (126 MHz, $CDCl_3$) δ (ppm) = 170.37, 168.89, 148.12, 143.31, 136.64, 136.28, 128.77, 128.62, 128.48, 128.43, 127.57, 126.47, 110.80, 110.78, 75.93, 71.11, 62.73, 42.78, 35.20.

IR (ATR): $\tilde{\nu}$ (cm^{-1}) = 1752, 1696, 1498, 1448, 1351, 1316, 1276, 1144, 1077, 1002.

HRMS (ESI^+) calcd. for $C_{23}H_{21}N_2O_4^+$ $[M+H]^+$: 389.1496, found: 389.1487.

1-Phenyl-3-tosyl-3,6-diazabicyclo[3.1.1]heptan-6-yl acetate (47)

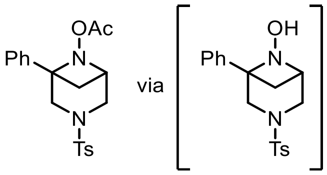

An oven-dried round-bottom flask containing oxime **S47b** (702 mg, 2.04 mmol, 1.00 equiv.) and $[Ir(dF(CF_3)ppy)_2(dtbbpy)](PF_6)$ (22.9 mg, 0.02 mmol, 1 mol%) was sealed and subjected to three N_2 evacuation/refill cycles before pre-sparged anhydrous CH_2Cl_2 (20.4 mL, 0.1 M) was added. The flask was placed close to a 40 W KSPR160L-427 nm Kessil light (100% intensity) at a distance of approximately 1 cm and the reaction was stirred under a nitrogen atmosphere with fan cooling for 2-2.5 h until complete (judged by TLC analysis). Et_3N (427 µL, 3.06 mmol, 1.50 equiv.) was then added, followed by the addition of DMAP (25 mg, 0.20 mmol, 0.10 equiv.) and Ac_2O (230 µL, 2.44 mmol, 1.20 equiv.). After being stirred at room temperature for 30 min, the reaction mixture was quenched with brine (100 mL). The product was extracted with CH_2Cl_2 (3 × 50 mL). The combined organic layers were dried over anhydrous $MgSO_4$ and concentrated under reduced pressure. The crude product was purified by flash column chromatography (pentane/ethyl acetate, 5:1 to 1:1) to afford **47** as a colorless oil (504 mg, 1.31 mmol, 64%).

Note: 1. Intermediate **45** will spontaneously rearrange into product **46** during the photochemical reaction. Therefore, reactions are recommended to be monitored by TLC every 30 minutes. Once the starting material is consumed, the light should be turned off and anhydride should be added immediately to protect the *N*-OH, avoiding the rearrangement. For example, in this case, the substrate is completely consumed after 2 h. By TLC, the polarity of intermediate **45** is slightly higher than that of the starting material, and a small amount of rearrangement byproduct **46** is found (shown below). At this point, the light was turned off, and Et_3N, DMAP, and anhydride were added to the reaction.

2. When thioxanthone is used as the photosensitizer, the starting material appears to require a longer time to be consumed (TLC indicated residual starting material even after 2.5 hours). However, extended reaction times lead to increased formation of compound **46** via rearrangement of the *N*–OH intermediate. Therefore, despite the cost advantage of thioxanthone, it is not recommended for use with this precursor.

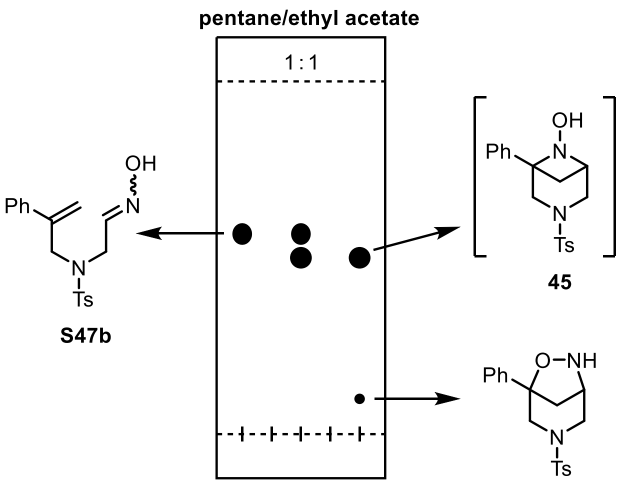

^{1}H NMR (500 MHz, $CDCl_3$) δ (ppm) = 7.76 (d, *J* = 8.3 Hz, 2H), 7.36-7.23 (m, 7H), 4.23 (ddt, *J* = 6.0, 2.7, 1.2 Hz, 1H), 3.86 (d, *J* = 11.3 Hz, 1H), 3.64 (dd, *J* = 11.5, 2.6 Hz, 1H), 3.53 (dd, *J* = 11.3, 1.2 Hz, 1H), 3.30 (dt, *J* = 11.5, 1.3 Hz, 1H), 2.40 (s, 3H), 2.38 (dd, *J* = 9.6, 1.0 Hz, 1H), 2.20 (dd, *J* = 9.5, 6.1 Hz, 1H), 1.46 (s, 3H).

^{13}C NMR (126 MHz, $CDCl_3$) δ (ppm) = 168.54, 143.84, 140.11, 133.89, 129.81, 128.69, 128.19, 127.70, 124.92, 73.85, 61.95, 47.46, 42.00, 28.96, 21.57, 18.44.

IR (ATR): $\tilde{\nu}$ (cm^{-1}) = 2968, 1755, 1597, 1495, 1448, 1341, 1211, 1163, 1092, 1029, 1014.

HRMS (ESI^+) calcd. for $C_{20}H_{23}N_2O_4S^+$ $[M+H]^+$: 387.1373, found: 387.1358.

5-Phenyl-3-tosyl-6-oxa-3,7-diazabicyclo[3.2.1]octane (46)

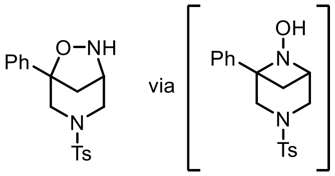

An oven-dried round-bottom flask containing oxime **S47b** (688 mg, 2.00 mmol, 1.00 equiv.) and $[Ir(dF(CF_3)ppy)_2(dtbbpy)](PF_6)$ (22.4 mg, 0.02 mmol, 1 mol%) was sealed and subjected to three N_2 evacuation/refill cycles before pre-sparged anhydrous MeCN (20.0 mL, 0.1 M) was added. The flask was placed close to a 40 W KSPR160L-427 nm Kessil light (100% intensity) at a distance of

approximately 1 cm and the reaction was stirred under a nitrogen atmosphere with fan cooling for 2-2.5 h until complete (judged by TLC analysis). The solvent was then removed in vacuo and the crude product was purified by flash column chromatography (pentane/ethyl acetate, 5:1 to 1:1) to afford intermediate **45** as a colorless oil (397 mg, 1.15 mmol, 58%).

Intermediate **45** was then dissolved in anhydrous 1,2-dichloroethane (10.0 mL) and stirred at 50 °C for 16 h. The solvent was then removed in vacuo and the crude product was purified by flash column chromatography (pentane/ethyl acetate, 1:1 to 100% ethyl acetate) to afford **46** as a white solid (230 mg, 0.67 mmol, 33%).

Note: Due to the insufficient stability of intermediate **45**, which can gradually rearrange at room temperature, it is necessary to quickly evaporate the solvent from the crude product (including keeping the water bath temperature below 30 °C) and perform rapid column chromatography. This is also why, in the synthesis of compound **47**, we added the anhydride directly to the reaction mixture after the photoreaction, rather than attempting to isolate intermediate **45**.

M.P.: 175-177 °C

^{1}H NMR (500 MHz, DMSO-d$_6$) δ (ppm) = 7.66 (d, J = 8.3 Hz, 2H), 7.41 (d, J = 8.1 Hz, 2H), 7.38 – 7.29 (m, 5H), 7.21 (br. s, 1H), 3.83 (dtd, J = 5.2, 3.6, 1.4 Hz, 1H), 3.62 (dd, J = 10.9, 3.5 Hz, 1H), 3.43 (dd, J = 11.3, 2.0 Hz, 1H), 3.33 (s, 3H), 2.83 (d, J = 11.2 Hz, 1H), 2.72 (d, J = 11.4 Hz, 1H), 2.41-2.36 (m, 4H), 1.99 (dd, J = 11.3, 5.1 Hz, 1H).

^{13}C NMR (126 MHz, DMSO-d$_6$) δ (ppm) = 143.15, 140.33, 134.00, 129.73, 128.29, 127.74, 127.21, 125.65, 80.45, 55.46, 54.53, 49.44, 42.67, 21.00.

IR (ATR): $\tilde{\nu}$ (cm^{-1}) = 2922, 1598, 1448, 1334, 1276, 1261, 1158, 1090, 974.

HRMS (ESI$^+$) calcd. for $C_{18}H_{21}N_2O_3S^+$ [M+H]$^+$: 345.1267, found: 345.1260.

6-(Benzyloxy)-1-phenyl-3-tosyl-3,6-diazabicyclo[3.1.1]heptane (48)

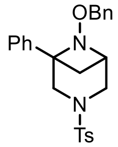

Prepared according to **General Procedure F** using oxime **S48b** (868 mg, 2.00 mmol, 1.00 equiv.), CH_3CN (20.0 mL), [Ir(dF(CF$_3$)ppy)$_2$(dtbbpy)](PF$_6$) (22.4 mg, 0.02 mmol, 1 mol%). Purification by flash column chromatography (pentane/ethyl acetate, 5:1 to 2:1) afforded a white solid (694 mg, 80% yield for a mixture of **48** (66%, major isomer) and fused product (14%, minor isomer), the ratio is 4.7:1

by ^{1}H NMR), Compound **48** could be exclusively obtained as a white solid via further recrystallization (pentane/ethyl acetate).

M.P.: 138-140 °C

^{1}H NMR (500 MHz, $CDCl_3$) δ (ppm) = 7.72 (d, J = 8.3 Hz, 2H), 7.36-7.24 (m, 8H), 7.19 (d, J = 8.0 Hz, 2H), 7.05 (dd, J = 7.6, 1.8 Hz, 2H), 4.60 (d, J = 11.5 Hz, 1H), 4.57 (d, J = 11.6 Hz, 1H), 3.75 (dt, J = 5.1, 1.7 Hz, 1H), 3.72 (d, J = 10.8 Hz, 1H), 3.67 (dd, J = 10.8, 1.0 Hz, 1H), 3.55 (dd, J = 10.8, 1.7 Hz, 1H), 3.50 (dd, J = 10.7, 2.6 Hz, 1H), 2.38 (s, 3H), 2.23 (d, J = 9.3 Hz, 1H), 2.02 (dd, J = 9.3, 6.0 Hz, 1H).

^{13}C NMR (126 MHz, $CDCl_3$) δ (ppm) = 143.20, 141.82, 137.54, 134.52, 129.51, 128.51, 128.31, 128.29, 127.77, 127.42, 125.06, 75.12, 73.33, 60.54, 46.96, 41.93, 28.17, 21.68.

IR (ATR): $\tilde{\nu}$ (cm^{-1}) = 3006, 1599, 1496, 1463, 1449, 1336, 1276, 1261, 1164, 1025.

HRMS (ESI^+) calcd. for $C_{25}H_{27}N_2O_3S^+$ $[M+H]^+$: 435.1737, found: 435.1720.

***N*-(1-phenyl-3-tosyl-3,6-diazabicyclo[3.1.1]heptan-6-yl)furan-2-carboxamide (49)**

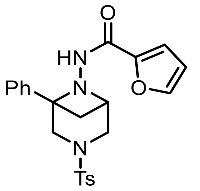

Prepared according to **General Procedure F** using oxime **S49b** (437 mg, 1.00 mmol, 1.00 equiv.), CH_3CN (10.0 mL), [Ir(dF(CF_3)ppy)$_2$(dtbbpy)](PF_6) (11.2 mg, 0.01 mmol, 1 mol%). Purification by flash column chromatography (pentane/ethyl acetate, 2:1 to 1:4) afforded **49** as a pale-yellow oil (248 mg, 0.57 mmol, 57%).

^{1}H NMR (500 MHz, $CDCl_3$) δ (ppm) = 7.76 (d, J = 8.0 Hz, 2H), 7.39-7.14 (m, 9H), 7.02 (app. s, 1H), 6.39 (app. s, 1H), 4.02 (dt, J = 5.7, 2.8 Hz, 1H), 3.78 (d, J = 12.1 Hz, 1H), 3.71 (dd, J = 11.9, 2.5 Hz, 1H), 3.62 (dd, J = 12.1, 1.5 Hz, 1H), 3.48 (dd, J = 12.0, 1.7 Hz, 1H), 2.39 (s, 3H), 2.32 (dd, J = 9.0, 6.1 Hz, 1H), 1.63 (d, J = 9.3 Hz, 1H).

^{13}C NMR (126 MHz, $CDCl_3$) δ (ppm) = 157.20, 146.50, 144.22, 143.97, 139.93, 134.06, 130.08, 128.58, 127.91, 127.31, 124.66, 115.41, 112.08, 72.94, 60.26, 46.83, 41.65, 32.73, 21.58.

IR (ATR): $\tilde{\nu}$ (cm^{-1}) = 3347, 2987, 1684, 1590, 1512, 1468, 1337, 1276, 1159, 1094, 1003.

HRMS (ESI^+) calcd. for $C_{23}H_{24}N_3O_4S^+$ $[M+H]^+$: 438.1482, found: 438.1464.

***N*-(1-(cyclopent-1-en-1-yl)-3-tosyl-3,6-diazabicyclo[3.1.1]heptan-6-yl)furan-2-carboxamide (50)**

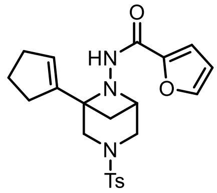

Prepared according to **General Procedure F** using oxime **S50b** (370 mg, 0.87 mmol, 1.00 equiv.), CH_3CN (8.7 mL), $[Ir(dF(CF_3)ppy)_2(dtbbpy)](PF_6)$ (9.7 mg, 8.6 μmol, 1 mol%). Purification by flash column chromatography (pentane/ethyl acetate, 3:1 to 1:2 and CH_2Cl_2/ethyl acetate, 9:1 to 4:1) afforded **50** as a yellow oil (201 mg, 0.47 mmol, 54%).

^{1}H NMR (500 MHz, $CDCl_3$) δ (ppm) = 7.60 (d, *J* = 8.3 Hz, 2H), 7.47 (dd, *J* = 1.8, 0.8 Hz, 1H), 7.36 (dd, *J* = 3.5, 0.9 Hz, 1H), 7.30 (d, *J* = 8.0 Hz, 2H), 6.39 (dd, *J* = 3.5, 1.8 Hz, 1H), 5.34 (t, *J* = 6.3 Hz, 1H), 4.46 (dt, *J* = 10.0, 1.4 Hz, 1H), 4.24 (d, *J* = 8.8 Hz, 1H), 3.72-3.65 (m, 1H), 3.46 (dd, *J* = 8.6, 4.2 Hz, 1H), 2.91 (d, *J* = 10.3 Hz, 1H), 2.65-2.55 (m, 2H), 2.42-2.29 (ddt, *J* = 12.9, 4.2, 2.1 Hz, 1H), 2.43-2.29 (m, 5H), 1.77-1.50 (m, 4H).

^{13}C NMR (126 MHz, $CDCl_3$) δ (ppm) = 159.79, 147.16, 144.59, 143.99, 141.14, 132.88, 129.86, 127.46, 126.07, 117.68, 111.39, 62.07, 53.42, 51.89, 48.04, 34.08, 33.96, 29.52, 24.59, 21.49.

IR (ATR): $\tilde{\nu}$ (cm^{-1}) = 2952, 2865, 1630, 1561, 1513, 1471, 1397, 1345, 1163, 1090, 1034.

HRMS (ESI^+) calcd. for $C_{22}H_{26}N_3O_4S^+$ $[M+H]^+$: 428.1639, found: 428.1624.

Benzyl (1-phenyl-3-tosyl-3,6-diazabicyclo[3.1.1]heptan-6-yl)carbamate (51)

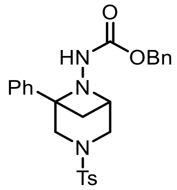

Prepared according to **General Procedure F** using oxime **S51b** (572 mg, 1.20 mmol, 1.00 equiv.), CH_3CN (12.0 mL), $[Ir(dF(CF_3)ppy)_2(dtbbpy)](PF_6)$ (13.4 mg, 0.012 mmol, 1 mol%). Purification by flash column chromatography (pentane/ethyl acetate, 5:1 to 1:1) afforded **51** as a colorless oil (400 mg, 0.84 mmol, 70%).

^{1}H NMR (500 MHz, $CDCl_3$) δ (ppm) = 7.74 (d, *J* = 8.3 Hz, 2H), 7.34-7.20 (m, 12H), 5.65 (s, 1H), 5.08 (br. s, 1H), 4.97 (d, *J* = 12.1 Hz, 1H), 3.93 (d, *J* = 6.1 Hz, 1H), 3.77 (d, *J* = 12.0 Hz, 1H), 3.62 (dd, *J* = 11.9, 2.6 Hz, 1H), 3.51 (d, *J* = 11.9 Hz, 1H), 3.45 (dd, *J* = 12.0, 1.8 Hz, 1H), 2.32-2.13(m, 4H), 1.57 (d, *J* = 9.3 Hz, 1H).

^{13}C NMR (126 MHz, $CDCl_3$) δ (ppm) = 155.56, 144.23, 140.02, 135.84, 133.58, 130.10, 128.44, 128.38, 128.13, 128.08, 127.78, 126.82, 124.70, 72.49, 66.94, 59.92, 46.65, 41.64, 32.44, 21.27.

IR (ATR): $\tilde{\nu}$ (cm^{-1}) = 3348, 2963, 2250, 1741, 1597, 1497, 1448, 1338, 1214, 1161, 1095, 1025.

HRMS (ESI^+) calcd. for $C_{26}H_{28}N_3O_4S^+$ $[M+H]^+$: 478.1795, found: 478.1772.

***N*-(1-phenyl-3-tosyl-3,6-diazabicyclo[3.1.1]heptan-6-yl)benzenesulfonamide (52)**

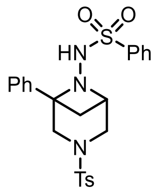

Prepared according to **General Procedure F** using oxime **S52b** (483 mg, 1.00 mmol, 1.00 equiv.), CH_3CN (10.0 mL), $[Ir(dF(CF_3)ppy)_2(dtbbpy)](PF_6)$ (11.2 mg, 0.01 mmol, 1 mol%). Purification by flash column chromatography (pentane/ethyl acetate, 5:1 to 1:1) afforded **52** as a colorless oil (310 mg, 0.64 mmol, 64%).

^{1}H NMR (500 MHz, $CDCl_3$) δ (ppm) = 7.84-7.77 (m, 4H), 7.58-7.54 (m, 1H), 7.46-7.41 (m, 4H), 7.21-7.10 (m, 5H), 5.34 (br. s, 1H), 3.66 (d, *J* = 11.7 Hz, 1H), 3.58 (d, *J* = 11.7 Hz, 1H), 3.46 (d, *J* = 12.5 Hz, 1H), 3.42-3.34 (m, 2H), 2.48 (s, 3H), 2.16-2.10 (m, 1H), 1.62-1.55 (m, 1H).

^{13}C NMR (126 MHz, $CDCl_3$) δ (ppm) = 145.02, 139.83, 138.80, 133.47, 133.36, 130.48, 129.08, 128.42, 128.16, 128.00, 127.03, 124.87, 72.52, 59.68, 47.53, 41.42, 32.55, 21.76.

IR (ATR): $\tilde{\nu}$ (cm^{-1}) = 3005, 1470, 1461, 1337, 1276, 1261, 1162, 1125.

HRMS (ESI^+) calcd. for $C_{24}H_{26}N_3O_4S_2^+$ $[M+H]^+$: 484.1359, found: 484.1366.

1.12 Procedures for Transformation of Product (Vinyl Group)

1.12.1 Hydrogenation of Alkene

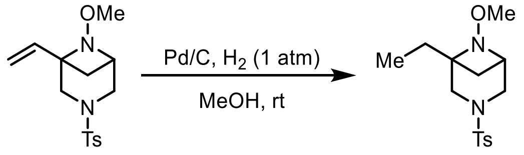

To a solution of alkene **26** (180 mg, 0.58 mmol, 1.00 equiv.) in MeOH (5.8 mL, 0.1 M) in an oven-dried 25 mL round-bottom flask was added 10% Pd/C (61.5 mg, 0.058 mmol, 0.10 equiv.). Then the vial was degassed by the freeze-pump-thaw (FPT) method followed by refilling of the vial with H_2 (1 atm) and the reaction mixture was stirred at room temperature for 2.5 h. The reaction was then purged by bubbling a gentle stream of $N_{2(g)}$ through the solution for 20 min and then the reaction mixture was filtered through a plug of $MgSO_4$ (washing with CH_2Cl_2). After removal of the solvent under reduced pressure, the residue was purified by column chromatography (CH_2Cl_2/ethyl acetate, 20:1 to 8:1) to afford **53** as a colorless oil (120 mg, 0.39 mmol, 67%).

1-Ethyl-6-methoxy-3-tosyl-3,6-diazabicyclo[3.1.1]heptane (53)

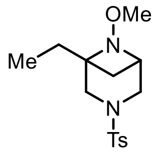

^{1}H NMR (500 MHz, $CDCl_3$) δ (ppm) = 7.70 (d, J = 8.4 Hz, 2H), 7.28 (d, J = 7.9 Hz, 2H), 3.78-3.75 (m, 1H), 3.44 (ddd, J = 10.8, 1.8, 0.9 Hz, 1H), 3.34 (dd, J = 10.8, 2.6 Hz, 1H), 3.30 (s, 1H), 3.29 (s, 1H), 3.16 (s, 3H), 2.39 (s, 3H), 1.65 (dd, J = 9.4, 5.8 Hz, 1H), 1.61 (dd, J = 9.4, 1.1 Hz, 1H), 1.51-1.43 (m, 1H), 1.45-1.35 (m, 1H), 0.83 (t, J = 7.6 Hz, 3H).

^{13}C NMR (126 MHz, $CDCl_3$) δ (ppm) = 143.12, 134.62, 129.40, 127.37, 72.02, 60.34, 59.26, 45.63, 41.80, 28.84, 24.78, 21.53, 8.22.

IR (ATR): $\tilde{\nu}$ (cm^{-1}) = 3006, 2976, 1598, 1461, 1338, 1276, 1261, 1162, 1095, 1058, 1012.

HRMS (ESI^+) calcd. for $C_{15}H_{23}N_2O_3S^+$ $[M+H]^+$: 311.1424, found: 311.1418.

1.12.2 Hydroboration-Oxidation of Alkene

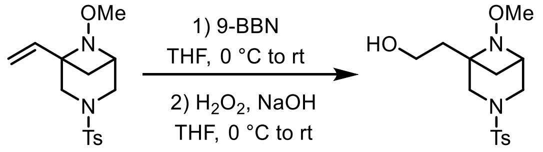

An oven-dried round-bottom flask containing alkene **26** (180 mg, 0.58 mmol, 1.00 equiv.) was sealed and subjected to three N_2 evacuation/refill cycles before pre-sparged anhydrous THF (5.8 mL, 0.1 M) was added. 9-BBN (3.5 mL, 0.5 M in THF, 1.75 mmol, 3.00 equiv.) was added to the solution at 0 °C. The mixture was stirred for 15 min at 0 °C and 4 h at room temperature. The reaction was then cooled to 0 °C, and then 3 N aqueous NaOH (6 mL) and 30% aqueous H_2O_2 (5 mL) were added. The reaction was allowed to warm to room temperature and stirring continued for 1 h before quenching with aqueous $Na_2S_2O_3$ (40 mL) at 0 °C and extracting with CH_2Cl_2 (3 × 20 mL). The organic layer was dried over MgSO4, filtered, and concentrated under reduced pressure. The crude mixture was purified by flash column chromatography (pentane/ethyl acetate, 1:1 to 1:9) to give the alcohol **54** as a colorless oil (161 mg, 0.49 mmol, 85%).

2-(6-Methoxy-3-tosyl-3,6-diazabicyclo[3.1.1]heptan-1-yl)ethan-1-ol (54)

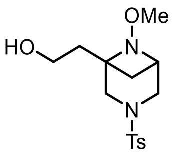

^{1}H NMR (400 MHz, $CDCl_3$) δ (ppm) = 7.68 (d, J = 8.3 Hz, 2H), 7.28 (d, J = 7.9 Hz, 2H), 3.89-3.80 (m, 2H), 3.70-3.60 (m, 2H), 3.43 (dd, J = 11.0, 1.8 Hz, 1H), 3.40-3.33 (m, 2H), 3.25 (d, J = 10.7 Hz, 1H), 3.17 (s, 3H), 2.38 (s, 3H), 1.97 (dd, J = 9.8, 6.1 Hz, 1H), 1.80-1.70 (m, 1H), 1.68 (d, J = 9.7 Hz, 1H), 1.39 (dd, J = 15.0, 4.3 Hz, 1H).

^{13}C NMR (101 MHz, $CDCl_3$) δ (ppm) = 143.32, 134.34, 129.47, 127.34, 71.56, 60.31, 59.49, 59.21, 46.36, 41.71, 35.00, 23.75, 21.52.

IR (ATR): $\tilde{\nu}$ (cm^{-1}) = 3006, 2988, 1598, 1462, 1335, 1276, 1261, 1159, 1092, 1050, 1023.

HRMS (ESI$^+$) calcd. for $C_{15}H_{23}N_2O_4S^+$ [M+H]$^+$: 327.1373, found: 327.1367.

1.12.3. Ozonolysis Followed by Amination of Alkene

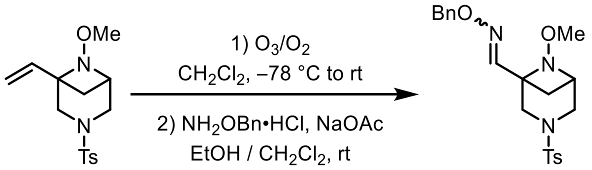

A solution of alkene **26** (200 mg, 0.65 mmol, 1.00 equiv.) in CH_2Cl_2 (13.0 mL, 0.05 M) was cooled to –78 °C before a stream of O_3-$O_{2(g)}$ was gently bubbled through the solution for ~10 min. The reaction was then purged by bubbling a gentle stream of $N_{2(g)}$ through the solution for 20 min, which was followed by the addition of Me_2S (0.29 mL, 3.90 mmol, 6.0 equiv.). The mixture was stirred at –78 °C

for 5 min followed by 2 h at room temperature. The solution was then concentrated under reduced pressure and the crude aldehyde was then dissolved in ethanol (6.5 mL, 0.1 M) and CH_2Cl_2 (0.65 mL, 1.0 M) in a 25 mL oven-dried round-bottom flask before sodium acetate (213 mg, 2.60 mmol, 4.00 equiv.) and *O*-benzylhydroxylamine hydrochloride (208 mg, 1.30 mmol, 2.00 equiv.) were added. The reaction was stirred at room temperature for 1 h until completion (judged by TLC). The reaction was quenched with sat. aq. $NaHCO_3$ solution (60 mL) and extracted with CH_2Cl_2 (3 × 20 mL). The combined organic layers were dried over anhydrous $MgSO_4$, filtered, concentrated under reduced pressure and purified by flash column chromatography (pentane/ethyl acetate, 5:1 to 1:1 and CH_2Cl_2/ethyl acetate, 20:1 to 9:1) to afford **55** as a colourless oil (170 mg, 0.41 mmol, 63%, *E/Z* = 75:25).

Note: After bubbling ozone into the reaction mixture at −78 °C for approximately 10 minutes, the solution may turn deep blue due to the presence of dissolved liquid ozone. This color change indicates an excess of ozone in the system and is commonly used to ensure the ozonolysis is complete.

6-Methoxy-3-tosyl-3,6-diazabicyclo[3.1.1]heptane-1-carbaldehyde *O*-benzyl oxime (55)

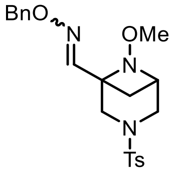

^{1}H NMR (500 MHz, $CDCl_3$) δ (ppm) = 7.75 (d, *J* = 8.3 Hz, 1.5H), 7.68 (d, *J* = 8.3 Hz, 0.5H), 7.36-7.28 (m, 7.25H), 7.23 (d, *J* = 7.9 Hz, 0.5H), 6.67 (s, 0.25H), 5.07 (s, 1.5H), 5.05 (d, *J* = 2.6 Hz, 0.5H), 3.86-3.77 (m, 1.25H), 3.71 (d, *J* = 11.3 Hz, 0.75H), 3.55-3.41 (m, 2.75H), 3.36 (dd, *J* = 11.1, 2.6 Hz, 0.25H), 3.25 (s, 0.75H), 3.17 (s, 2.25H), 2.41 (s, 2.25H), 2.39 (s, 0.75H), 2.05 (dd, *J* = 10.1, 0.9 Hz, 0.25H), 1.96 (dd, *J* = 9.5, 5.9 Hz, 0.75H), 1.92 (dd, *J* = 9.9, 6.0 Hz, 0.25H), 1.86 (dd, *J* = 9.6, 0.9 Hz, 0.75H).

^{13}C NMR (126 MHz, $CDCl_3$) δ (ppm) = 149.73, 149.28, 143.27, 143.21, 137.34, 136.92, 134.52, 134.42, 129.47, 129.41, 128.47, 128.46, 128.41, 128.06, 128.01, 127.99, 127.36, 127.32, 76.54, 76.41, 70.15, 69.31, 60.79, 60.54, 60.43, 59.73, 42.81, 41.76, 41.58, 27.32, 26.02, 21.50, 21.49.

IR (ATR): $\tilde{\nu}$ (cm^{-1}) = 3006, 2988, 1598, 1495, 1455, 1340, 1276, 1261, 1160, 1092, 1052, 1022, 1011.

HRMS (ESI^+) calcd. for $C_{21}H_{26}N_3O_4S^+$ $[M+H]^+$: 416.1639, found: 416.1634.

1.12.4. Ozonation Followed by Reduction of Alkene

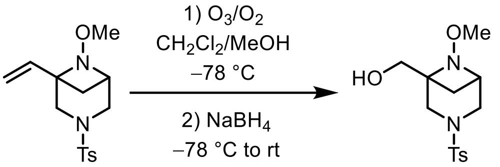

A solution of alkene **26** (430 mg, 1.40 mmol, 1.0 equiv.) in CH_2Cl_2/MeOH (28.0 mL, 3:1) was cooled to −78 °C before a stream of O_3-$O_{2(g)}$ was gently bubbled through the solution for ~10 min (the solution became blue). The reaction was then purged by bubbling a gentle stream of $N_{2(g)}$ through the solution for 20 min, which was followed by the portion-wise addition of $NaBH_4$ (212 mg, 5.60 mmol, 4.00 equiv.). The mixture was stirred at −78 °C for 15 min, followed by 1 h at room temperature before quenching with brine (60 mL) and extracting with EtOAc (3 × 20 mL). The combined organic layers were dried over anhydrous $MgSO_4$, filtered, concentrated under reduced pressure and purified by flash column chromatography (pentane/ethyl acetate, 3:1 to 1:1 to 100% ethyl acetate) to afford alcohol **56** as a colorless oil (370 mg, 1.19 mmol, 85%).

Note: After bubbling ozone into the reaction mixture at −78 °C for approximately 10 minutes, the solution may turn deep blue due to the presence of dissolved liquid ozone. This color change indicates an excess of ozone in the system and is commonly used to ensure the ozonolysis is complete.

(6-Methoxy-3-tosyl-3,6-diazabicyclo[3.1.1]heptan-1-yl)methanol (56)

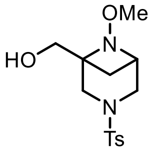

^{1}H NMR (400 MHz, $CDCl_3$) δ (ppm) = 7.68 (d, J = 8.3 Hz, 2H), 7.27 (d, J = 7.9 Hz, 2H), 3.81-3.78 (m, 1H), 3.48 (ddd, J = 10.9, 1.6, 0.9 Hz, 1H), 3.41 (dd, J = 4.1, 2.7 Hz, 1H), 3.38 (t, J = 2.9 Hz, 1H), 3.34-3.27 (m, 3H), 3.16 (s, 3H), 2.51 (dd, J = 8.2, 3.3 Hz, 1H), 2.38 (s, 3H), 1.88 (dd, J = 9.5, 5.9 Hz, 1H), 1.61 (d, J = 9.5 Hz, 1H).

^{13}C NMR (101 MHz, $CDCl_3$) δ (ppm) = 143.33, 134.29, 129.47, 127.33, 72.03, 63.10, 60.46, 59.48, 43.49, 42.18, 22.57, 21.50.

IR (ATR): $\tilde{\nu}$ (cm^{-1}) = 3006, 2989, 1462, 1336, 1276, 1261, 1160, 1092, 1055, 1024.

HRMS (ESI$^+$) calcd. for $C_{14}H_{21}N_2O_4S^+$ [M+H]$^+$: 313.1217, found: 313.1213.

1.12.5 Deoxygenation of Alcohol 56

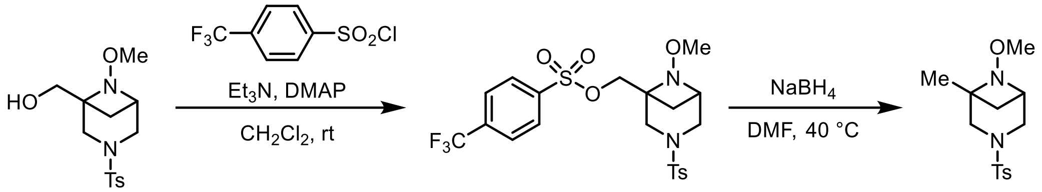

In an oven-dried 25 mL round-bottom flask under nitrogen atmosphere was dissolved alcohol **56** (102 mg, 0.33 mmol, 1.00 equiv.) in anhydrous CH_2Cl_2 (2.2 mL, 0.15 M) at room temperature. Et_3N (140 µL, 1.00 mmol, 3.00 equiv.) was then added, followed by the addition of 4-(trifluoromethyl)benzenesulfonyl chloride (245 mg, 1.00 mmol, 3.00 equiv.) and DMAP (8.0 mg, 0.066 mmol, 0.20 equiv.). After being stirred at room temperature for 1 h, the reaction mixture was quenched with sat. aq. NaCl solution (100 mL). The product was extracted with CH_2Cl_2 (3 × 40 mL). The combined organic layers were dried over anhydrous Na_2SO_4, filtered, and concentrated under reduced pressure. The crude product was purified by flash column chromatography (pentane/ethyl acetate, 5:1 to 1:1) to afford **57'** as a white solid (167 mg, 0.32 mmol, 97%).

(6-Methoxy-3-tosyl-3,6-diazabicyclo[3.1.1]heptan-1-yl)methyl 4-(trifluoromethyl) benzene-sulfonate (57')

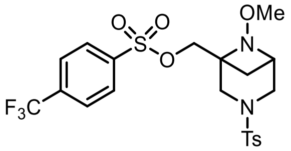

M.P.: 95-97 °C

^{1}H NMR (400 MHz, $CDCl_3$) δ (ppm) = 8.02 (d, *J* = 8.2 Hz, 2H), 7.83 (d, *J* = 8.3 Hz, 2H), 7.67 (d, *J* = 8.4 Hz, 2H), 7.29 (d, *J* = 8.0 Hz, 2H), 3.98 (d, *J* = 10.5 Hz, 1H), 3.92 (d, *J* = 10.5 Hz, 1H), 3.84-3.79 (m, 1H), 3.45 (dd, *J* = 11.0, 1.6 Hz, 1H), 3.40-3.33 (m, 3H), 3.10 (s, 3H), 2.41 (s, 3H), 1.76-1.71 (m, 2H).

^{13}C NMR (101 MHz, $CDCl_3$) δ (ppm) = 143.55, 139.20, 135.75 (q, *J* = 33.3 Hz), 134.25, 129.59, 128.60, 127.38, 126.64 (q, *J* = 3.7 Hz), 123.05 (q, *J* = 273.2 Hz), 71.65, 69.26, 60.58, 60.21, 42.83, 41.68, 24.04, 21.56.

^{19}F NMR (377 MHz, $CDCl_3$) δ (ppm) = −63.28 (s).

IR (ATR): $\tilde{\nu}$ (cm^{-1}) = 3006, 2989, 1461, 1323, 1276, 1261, 1183, 1162, 1136, 1063, 1015.

HRMS (ESI$^+$) calcd. for $C_{21}H_{24}F_3N_2O_6S_2^+$ [M+H]$^+$: 521.1022, found: 521.1004.

In an oven-dried 25 mL round-bottom flask under nitrogen atmosphere was dissolved **57'** (110 mg, 0.21 mmol, 1.00 equiv.) in anhydrous DMF (2.1 mL, 0.10 M) at room temperature. $NaBH_4$ (24 mg, 0.63 mmol, 3.00 equiv.) was then added. After being stirred at 40 °C for 15 h, the reaction mixture was quenched with sat. aq. NaCl solution (70 mL). The product was extracted with ethyl acetate (3 × 20 mL). The combined organic layers were dried over anhydrous Na_2SO_4, filtered and concentrated under reduced pressure. The crude product was purified by flash column chromatography (pentane/ethyl acetate, 5:1 to 1:1) to afford **57** as a white solid (56 mg, 0.19 mmol, 90%).

6-Methoxy-1-methyl-3-tosyl-3,6-diazabicyclo[3.1.1]heptane (57)

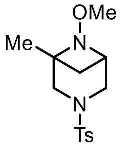

M.P.: 87-89 °C

^{1}H NMR (400 MHz, $CDCl_3$) δ (ppm) = 7.71 (d, *J* = 8.3 Hz, 2H), 7.28 (d, *J* = 8.1 Hz, 2H), 3.79 (dddd, *J* = 5.6, 2.7, 1.7, 1.0 Hz, 1H), 3.42 (ddd, *J* = 10.8, 1.8, 0.9 Hz, 1H), 3.33 (dd, *J* = 10.8, 2.6 Hz, 1H), 3.30 (dd, *J* = 10.6, 1.2 Hz, 1H), 3.24 (d, *J* = 10.6 Hz, 1H), 3.18 (s, 3H), 2.40 (s, 3H), 1.73 (dd, *J* = 9.4, 0.9 Hz, 1H), 1.65 (dd, *J* = 9.4, 5.8 Hz, 1H), 1.15 (s, 3H).

^{13}C NMR (101 MHz, $CDCl_3$) δ (ppm) = 143.17, 134.63, 129.44, 127.41, 68.45, 60.73, 59.75, 46.83, 41.33, 27.58, 22.97, 21.56.

IR (ATR): $\tilde{\nu}$ (cm^{-1}) = 2958, 2875, 1598, 1458, 1336, 1276, 1260, 1159, 1094, 1044, 1023.

HRMS (ESI$^+$) calcd. for $C_{14}H_{21}N_2O_3S^+$ $[M+H]^+$: 297.1267, found: 297.1257.

1.12.6 Ozonation Followed by Pinnick Oxidation of Alkene

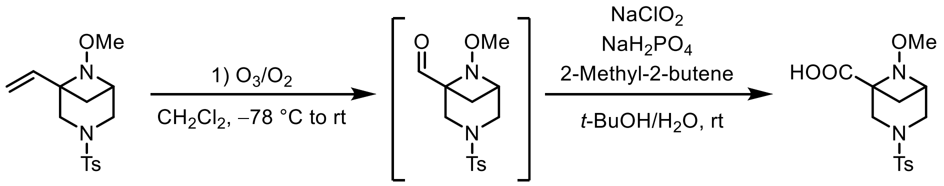

A solution of alkene **26** (860 mg, 2.80 mmol, 1.00 equiv.) in CH_2Cl_2 (35.0 mL, 0.08 M) was cooled to −78 °C before a stream of O_3-$O_{2(g)}$ was gently bubbled through the solution for ~10 min (the solution became blue). The reaction was then purged by bubbling a gentle stream of $N_{2(g)}$ through the solution for 20 min, which was followed by the addition of Me_2S (1.23 mL, 16.80 mmol, 6.00 equiv.). The mixture was stirred at –78 °C for 5 min followed by 1 h at room temperature. The solution was then concentrated under reduced pressure and the crude aldehyde was then dissolved in *t*-BuOH (40 mL)

and H_2O (10 mL) in an oven-dried 100 mL round-bottom flask before NaH_2PO_4 (1.68 g, 14.0 mmol, 5.00 equiv.), 2-methyl-2-butene (5.94 mL, 56.0 mmol, 20.0 equiv.) and $NaClO_2$ (1.58 g, 80% purity, 14.0 mmol, 5.00 equiv.) were added. The reaction was stirred at room temperature for 12 h and was quenched with 1 M aqueous HCl (120 mL) and extracted with ethyl acetate (3 × 40 mL). The combined organic layers were dried over anhydrous $MgSO_4$, filtered, concentrated under reduced pressure and purified by flash column chromatography (pentane/ethyl acetate, 5:1 to 1:1 to 100 % ethyl acetate) to afford carboxylic acid **58** as a white solid (782 mg, 3.40 mmol, 86%).

6-Methoxy-3-tosyl-3,6-diazabicyclo[3.1.1]heptane-1-carboxylic acid (58)

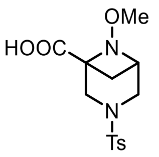

M.P.: 88-90 °C

^{1}H NMR (400 MHz, $CDCl_3$) δ (ppm) = 9.99 (br. s, 1H), 7.69 (d, J = 8.4 Hz, 2H), 7.29 (d, J = 8.1 Hz, 2H), 3.89-3.82 (m, 1H), 3.73 (d, J = 11.1 Hz, 1H), 3.53 (d, J = 11.0 Hz, 1H), 3.48 (dd, J = 11.0, 1.6 Hz, 1H), 3.40 (dd, J = 11.2, 2.6 Hz, 1H), 3.23 (s, 3H), 2.38 (s, 3H), 2.17 (dd, J = 9.7, 6.0 Hz, 1H), 1.93 (d, J = 9.7 Hz, 1H).

^{13}C NMR (101 MHz, $CDCl_3$) δ (ppm) = 173.16, 143.64, 133.94, 129.58, 127.33, 71.21, 60.30, 59.70, 42.19, 41.24, 25.45, 21.49.

IR (ATR): $\tilde{\nu}$ (cm^{-1}) = 3006, 2989, 1699, 1598, 1456, 1343, 1314, 1276, 1261, 1157, 1136, 1055, 1006.

HRMS (Nanospray) calcd. for $C_{14}H_{17}N_2O_5S^-$ [M-H]$^-$: 325.0864, found: 325.0855.

1.12.7 Synthesis of Carboxylic Acid Derivatives

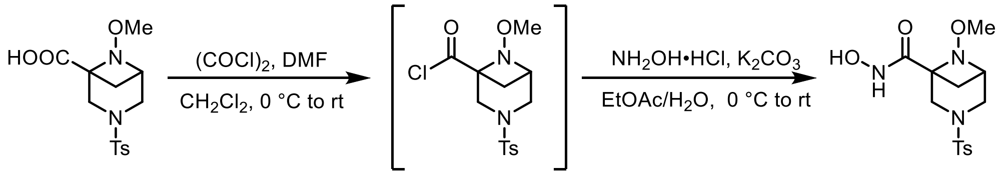

An oven-dried 50 mL round-bottom flask containing carboxylic acid **58** (240 mg, 0.73 mmol, 1.00 equiv.) was sealed and subjected to three N_2 evacuation/refill cycles before pre-sparged anhydrous CH_2Cl_2 (7.3 mL, 0.1 M) was added. Oxalyl chloride (126 μL, 1.47 mmol, 2.00 equiv.) was then added at 0 °C, followed by the addition of DMF (3 drops). The reaction mixture was allowed to warm up to room temperature and stirred for 3 h. Then the reaction was concentrated under reduced pressure to afford the crude acyl chloride and used directly for the next step with further purification.

In an oven-dried 25 mL round-bottom flask, hydroxylamine hydrochloride (76 mg, 1.09 mmol, 1.50 equiv.) was added to a biphasic mixture of K_2CO_3 (201 mg, 1.46 mmol, 2.00 equiv.) in EtOAc/H_2O solution (3.0 mL/1.5 mL). The reaction mixture was cooled to 0 °C, and crude acyl chloride dissolved in EtOAc (1.0 mL) was added. The mixture was warmed up to room temperature and stirred for 3 h until completion (judged by TLC). The reaction was quenched with brine (80 mL) and extracted with ethyl acetate (3 × 30 mL). The combined organic layers were washed with brine, dried over anhydrous $MgSO_4$, filtered and concentrated under reduced pressure. The crude product was purified by flash column chromatography (pentane/ethyl acetate, 5:1 to 1:1 to 100% ethyl acetate) to afford **59** as a white solid (135 mg, 0.40 mmol, 54%).

***N*-hydroxy-6-methoxy-3-tosyl-3,6-diazabicyclo[3.1.1]heptane-1-carboxamide (59)**

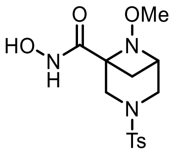

M.P.: 159-161 °C

^{1}H NMR (500 MHz, $CDCl_3$) δ (ppm) = 9.24 (s, 1H), 8.89 (br. s, 1H), 7.71 (d, *J* = 8.3 Hz, 2H), 7.32 (d, *J* = 8.1 Hz, 2H), 3.91 (d, *J* = 11.2 Hz, 1H), 3.87 (ddt, *J* = 5.6, 2.8, 1.2 Hz, 1H), 3.50 (dd, *J* = 11.2, 1.9 Hz, 1H), 3.43 (dd, *J* = 11.3, 2.7 Hz, 1H), 3.39 (dd, *J* = 11.2, 0.8 Hz, 1H), 3.27 (s, 3H), 2.42 (s, 3H), 1.99 (d, *J* = 9.5 Hz, 1H), 1.93 (dd, *J* = 9.6, 5.8 Hz, 1H).

^{13}C NMR (126 MHz, $CDCl_3$) δ (ppm) = 166.91, 143.73, 134.27, 129.71, 127.46, 70.78, 60.51, 59.85, 41.74, 41.30, 27.48, 21.64.

IR (ATR): $\tilde{\nu}$ (cm^{-1}) = 3006, 2988, 1666, 1598, 1494, 1460, 1336, 1276, 1261, 1158, 1091, 1022.

HRMS (ESI$^+$) calcd. for $C_{14}H_{20}N_3O_5S^+$ [M+H]$^+$: 342.1118, found: 342.1108.

1.12.8 Decarboxylation of Carboxylic Acid 58

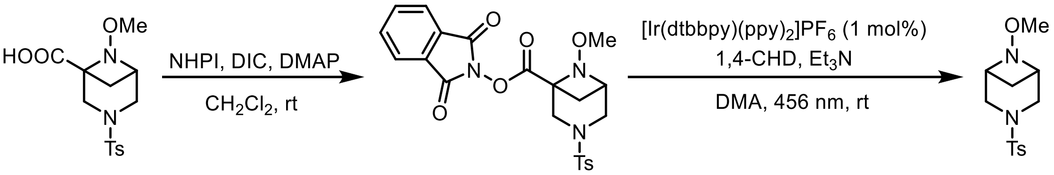

In an oven-dried 25 mL round-bottom flask, carboxylic acid **58** (65 mg, 0.20 mmol, 1.00 equiv.), *N*-hydroxyphthalimide (NHPI, 35.9 mg, 0.22 mmol, 1.10 equiv.) and DMAP (2.4 mg, 0.02 mmol, 0.10 equiv.) were dissolved in anhydrous CH_2Cl_2 (2.0 mL, 0.1 M) at room temperature. *N*,*N'*-Diisopropylcarbodiimide (DIC, 35 μL, 0.22 mmol, 1.10 equiv.) was then added and the reaction was stirred at room temperature for 30 min before concentrating under reduced pressure. The crude product

was purified by flash column chromatography (pentane/ethyl acetate, 5:1 to 1:1) to afford **60’** as a white solid (80.2 mg, 0.17 mmol, 85%).

Note: This redox-active ester is prone to decomposition on silica gel during column chromatography if it remains in the column for too long. Therefore, after loading the compound onto the top of the silica gel column, the purification should be completed within 10 minutes.

1,3-Dioxoisoindolin-2-yl 6-methoxy-3-tosyl-3,6-diazabicyclo[3.1.1]heptane-1-carboxylate (60’)

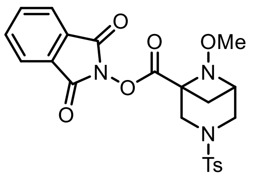

M.P.: 149-151 °C

^{1}H NMR (400 MHz, $CDCl_3$) δ (ppm) = 7.84 (dd, J = 5.5, 3.1 Hz, 2H), 7.77 (dd, J = 5.6, 3.1 Hz, 2H), 7.73 (d, J = 8.3 Hz, 2H), 7.31 (d, J = 8.0 Hz, 2H), 3.95-3.91 (m, 1H), 3.87 (d, J = 11.1 Hz, 1H), 3.71 (dd, J = 11.1, 1.0 Hz, 1H), 3.55 (dt, J = 11.1, 1.0 Hz, 1H), 3.46 (dd, J = 11.2, 2.6 Hz, 1H), 3.34 (s, 3H), 2.46-2.38 (m, 4H), 2.11 (d, J = 9.6 Hz, 1H).

^{13}C NMR (101 MHz, $CDCl_3$) δ (ppm) = 165.79, 161.42, 143.70, 135.04, 134.07, 129.69, 128.74, 127.42, 124.11, 70.11, 60.83, 60.78, 41.77, 41.17, 25.66, 21.55.

IR (ATR): $\tilde{\nu}$ (cm^{-1}) = 3006, 2989, 1812, 1783, 1744, 1597, 1467, 1345, 1333, 1275, 1261, 1186, 1160, 1120, 1059, 1008.

HRMS (ESI^+) calcd. for $C_{22}H_{22}N_3O_7S^+$ $[M+H]^+$: 472.1173, found: 472.1170.

The redox-active ester **60’** (100 mg, 0.21 mmol, 1.00 equiv.) and $[Ir(ppy)_2(dtbbpy)]PF_6$ (1.9 mg, 2.1 μmol, 1 mol%) were added to an oven-dried 25 mL round-bottom flask equipped with a stir bar and placed under an N_2 atmosphere. Dry, degassed DMA (4.2 mL, 0.05 M) was added, followed by triethylamine (59 μL, 0.42 mmol, 2.00 equiv.) and 1,4-cyclohexadiene (39 μL, 0.42 mmol, 2.00 equiv.). The flask was placed close to a 40 W KSPR160L-456 nm Kessil light (100% intensity) at a distance of approximately 1 cm (fan cooling). The reaction was stirred for 6 h, before being diluted with H_2O (40 mL), and extracted with Et_2O (3 × 20 mL). The combined organic layers were washed with brine, dried over anhydrous $MgSO_4$, filtered and concentrated under reduced pressure. The crude product was purified by flash column chromatography (pentane/ethyl acetate, 5:1 to 2:3) to afford **60** as a white solid (38 mg, 0.13 mmol, 64%).

6-Methoxy-3-tosyl-3,6-diazabicyclo[3.1.1]heptane (60)

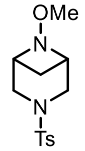

M.P.: 112-114 °C

^{1}H NMR (400 MHz, $CDCl_3$) δ (ppm) = 7.73 (d, J = 8.0 Hz, 2H), 7.30 (d, J = 8.0 Hz, 2H), 3.83 (d, J = 5.7 Hz, 2H), 3.54 (d, J = 11.0 Hz, 2H), 3.42 (dd, J = 10.9, 2.1 Hz, 2H), 3.15 (s, 3H), 2.41 (s, 3H), 1.98 (dt, J = 9.8, 5.8 Hz, 1H), 1.68 (d, J = 9.6 Hz, 1H).

^{13}C NMR (101 MHz, $CDCl_3$) δ (ppm) = 143.27, 134.61, 129.48, 127.47, 62.60, 59.15, 42.52, 21.67, 21.60.

IR (ATR): $\tilde{\nu}$ (cm^{-1}) =3006, 2954, 1716, 1597, 1494, 1463, 1335, 1276, 1260, 1162, 1125, 1093, 1058.

HRMS (ESI^+) calcd. for $C_{13}H_{19}N_2O_3S^+$ $[M+H]^+$: 283.1111, found: 283.1107.

1.13 Procedures for *N*-Deprotection and *N*-Functionalization

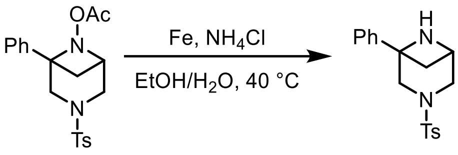

In an oven-dried 25 mL round-bottom flask, **47** (502 mg, 1.30 mmol, 1.00 equiv.) was dissolved in EtOH/H_2O (4 mL:1 mL, 0.25 M) at room temperature. Iron powder (728 mg, 13.0 mmol, 10.0 equiv.) and ammonium chloride (696 mg, 13.0 mmol, 10.0 equiv.) were then added and the reaction was stirred at 40 °C for 18 h. The reaction mixture was then allowed to cool to room temperature, filtered through a short pad of $MgSO_4$ (washed with CH_2Cl_2) and concentrated under reduced pressure. The crude product was purified by flash column chromatography (pentane/ethyl acetate, 1:1 to 100% ethyl acetate) to afford **61** as a white solid (361 mg, 1.10 mmol, 85%).

1-Phenyl-3-tosyl-3,6-diazabicyclo[3.1.1]heptane (61)

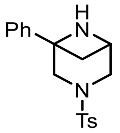

M.P.: 153-155 °C

^{1}H NMR (500 MHz, $CDCl_3$) δ (ppm) = 7.70 (d, *J* = 8.3 Hz, 2H), 7.33-7.28 (m, 4H), 7.25-7.21 (m, 1H), 7.17-7.15 (m, 2H), 3.82 (d, *J* = 11.1 Hz, 1H), 3.74-3.66 (m, 2H), 3.34 (dd, *J* = 10.8, 1.3 Hz, 1H), 3.18 (dd, *J* = 11.1, 1.4 Hz, 1H), 2.46 (dd, *J* = 8.9, 6.1 Hz, 1H), 2.41 (s, 3H), 2.00 (d, *J* = 8.8 Hz, 1H), 1.17 (br. s, 1H).

^{13}C NMR (126 MHz, $CDCl_3$) δ (ppm) = 143.64, 143.19, 134.00, 129.84, 128.40, 127.37, 127.33, 124.39, 64.89, 55.23, 51.45, 49.26, 35.08, 21.52.

IR (ATR): $\tilde{\nu}$ (cm^{-1}) =3407, 2939, 1597, 1448, 1334, 1276, 1162, 1091, 1024, 1009.

HRMS (ESI^+) calcd. for $C_{18}H_{21}N_2O_2S^+$ $[M+H]^+$: 329.1318, found: 329.1307.

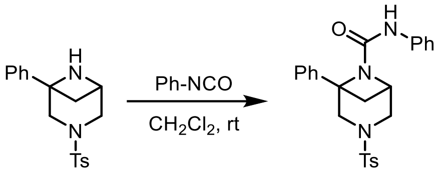

To an oven-dried 25 mL round-bottom flask was dissolved **61** (112 mg, 0.34 mmol, 1.00 equiv.) in anhydrous CH_2Cl_2 (3.4 mL) at room temperature. Phenyl isocyanate (61 mg, 0.51 mmol, 1.50 equiv.) was then added dropwise and the reaction was stirred at room temperature for 12 h until completion

of the reaction (TLC). The reaction was quenched with water (50 mL) and extracted with CH_2Cl_2 (3 × 20 mL). The combined extracts were washed with brine, dried over anhydrous $MgSO_4$, and concentrated *in vacuo*. The crude product was purified by flash column chromatography (pentane/ethyl acetate, 3:1 to 1:1) to afford **62** as a white solid (150 mg, 0.34 mmol, 99%).

***N*,1-diphenyl-3-tosyl-3,6-diazabicyclo[3.1.1]heptane-6-carboxamide (62)**

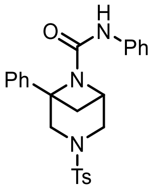

M.P.: 180-182 °C

^{1}H NMR (500 MHz, $CDCl_3$) δ (ppm) = 7.64 (d, J = 8.3 Hz, 2H), 7.50 (d, J = 7.2 Hz, 2H), 7.43 (t, J = 7.3 Hz, 2H), 7.40-7.36 (m, 1H), 7.16-7.10 (m, 2H), 7.03 (d, J = 8.0 Hz, 2H), 6.97 (t, J = 7.4 Hz, 1H), 6.82 (d, J = 7.5 Hz, 2H), 5.85 (s, 1H), 4.36 (dt, J = 6.5, 2.1 Hz, 1H), 4.13 (d, J = 10.8 Hz, 1H), 3.96 (dd, J = 10.9, 2.2 Hz, 1H), 3.91 (d, J = 10.8 Hz, 1H), 3.57 (dd, J = 10.9, 1.8 Hz, 1H), 2.51 (t, J = 7.7 Hz, 1H), 2.22 (s, 3H), 2.03 (d, J = 8.8 Hz, 1H).

^{13}C NMR (126 MHz, $CDCl_3$) δ (ppm) = 155.35, 143.73, 138.67, 137.60, 133.61, 129.76, 129.38, 129.08, 128.64, 127.21, 125.37, 123.23, 119.31, 69.30, 55.49, 47.43, 44.21, 36.72, 21.57.

IR (ATR): $\tilde{\nu}$ (cm^{-1}) = 3355, 1735, 1685, 1597, 1531, 1499, 1439, 1335, 1305, 1276, 1246, 1153, 1094.

HRMS (ESI$^+$) calcd. for $C_{25}H_{26}N_3O_3S^+$ [M+H]$^+$: 448.1689, found: 448.1677.

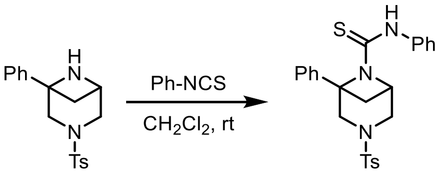

To an oven-dried 25 mL round-bottom flask was dissolved **61** (100 mg, 0.30 mmol, 1.00 equiv.) in anhydrous CH_2Cl_2 (3.0 mL) at room temperature. Phenyl isothiocyanate (54 µL, 0.45 mmol, 1.50 equiv.) was then added dropwise and the reaction was stirred at room temperature for 12 h until completion of the reaction (TLC). The reaction was quenched with water (50 mL) and extracted with CH_2Cl_2 (3 × 20 mL). The combined extracts were washed with brine, dried over anhydrous $MgSO_4$, and concentrated *in vacuo*. The crude product was purified by flash column chromatography (pentane/ethyl acetate, 3:1 to 1:1) to afford **63** as a colorless oil (118 mg, 0.25 mmol, 85%).

N,1-diphenyl-3-tosyl-3,6-diazabicyclo[3.1.1]heptane-6-carbothioamide (63)

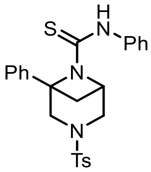

^{1}H NMR (500 MHz, $CDCl_3$) δ (ppm) = 7.76 (d, J = 8.3 Hz, 2H), 7.56 (d, J = 7.0 Hz, 2H), 7.51 (t, J = 7.5 Hz, 2H), 7.47-7.42 (m, 1H), 7.24 (d, J = 8.1 Hz, 2H), 7.20-7.13 (m, 2H), 7.08 (t, J = 7.4 Hz, 1H), 6.79 (d, J = 7.8 Hz, 2H), 6.52 (br. s, 1H), 4.71 (d, J = 6.5 Hz, 1H), 4.54 (br. s, 1H), 4.28 (d, J = 10.9 Hz, 1H), 3.84 (br. s, 1H), 3.62 (dd, J = 10.8, 2.1 Hz, 1H), 2.66 (t, J = 7.8 Hz, 1H), 2.37 (s, 3H), 2.15 (d, J = 9.1 Hz, 1H).

^{13}C NMR (126 MHz, $CDCl_3$) δ (ppm) = 180.41, 143.82, 137.64, 136.75, 133.77, 130.03, 129.94, 128.40, 127.53, 125.65, 125.49, 123.80, 71.11, 58.34, 46.53, 43.79, 37.06, 21.62.

IR (ATR): $\tilde{\nu}$ (cm^{-1}) = 3357, 2919, 1597, 1531, 1517, 1497, 1447, 1338, 1305, 1276, 1261, 1160, 1093.

HRMS (ESI^+) calcd. for $C_{25}H_{26}N_3O_2S_2^+$ $[M+H]^+$: 464.1461, found: 464.1443.

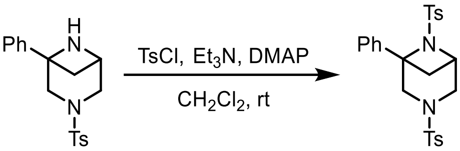

To an oven-dried 25 mL round-bottom flask was dissolved **61** (87.0 mg, 0.27 mmol, 1.00 equiv.) in anhydrous CH_2Cl_2 (2.7 mL) at room temperature. Et_3N (74 µL, 0.53 mmol, 2.00 equiv.) was then added, followed by the addition of TsCl (65.7 mg, 0.34 mmol, 1.30 equiv.) and DMAP (6.5 mg, 0.053 mmol, 0.20 equiv.). After being stirred at room temperature for 1 h, the reaction mixture was quenched with sat. aq. NaCl solution (50 mL). The product was extracted with CH_2Cl_2 (3 × 30 mL). The combined organic layers were dried over anhydrous $MgSO_4$ and concentrated under reduced pressure. The crude product was purified by flash column chromatography (pentane/ethyl acetate, 5:1 to 1:1) to afford **64** as a colorless oil (125 mg, 0.26 mmol, 98%).

1-Phenyl-3,6-ditosyl-3,6-diazabicyclo[3.1.1]heptane (64)

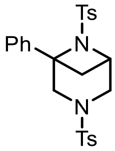

^{1}H NMR (500 MHz, $CDCl_3$) δ (ppm) = 7.80 (d, J = 8.3 Hz, 2H), 7.32 (d, J = 7.8 Hz, 2H), 7.25 (d, J = 8.3 Hz, 2H), 7.22-7.13 (m, 5H), 7.00 (d, J = 7.8 Hz, 2H), 4.34 (dt, J = 6.1, 1.9 Hz, 1H), 4.32 (dd, J =

10.8, 1.4 Hz, 1H), 4.05 (ddd, J = 11.0, 1.8, 0.9 Hz, 1H), 3.97 (d, J = 10.8 Hz, 1H), 3.69 (dd, J = 11.1, 2.2 Hz, 1H), 2.62-2.57 (m, 1H), 2.38 (s, 3H), 2.28 (s, 3H), 1.96 (d, J = 8.6 Hz, 1H).

^{13}C NMR (126 MHz, $CDCl_3$) δ (ppm) = 143.67, 143.39, 138.12, 136.49, 134.62, 129.74, 129.16, 128.67, 128.28, 127.57, 127.05, 126.01, 73.67, 59.55, 49.28, 46.48, 36.41, 21.64, 21.47.

IR (ATR): $\tilde{\nu}$ (cm^{-1}) = 2968, 1598, 1449, 1337, 1305, 1155, 1091, 1050, 1003.

HRMS (ESI$^+$) calcd. for $C_{25}H_{27}N_2O_4S_2^+$ [M+H]$^+$: 483.1407, found: 483.1401.

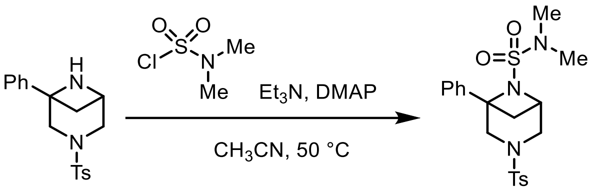

To an oven-dried 25 mL round-bottom flask was dissolved **61** (110 mg, 0.34 mmol, 1.00 equiv.) in anhydrous CH_3CN (3.4 mL) at room temperature. Et_3N (187 µL, 1.34 mmol, 4.00 equiv.) was then added, followed by the addition of *N*,*N*-dimethylsulfamoyl chloride (198 mg, 1.38 mmol, 4.00 equiv.) and DMAP (41.0 mg, 0.34 mmol, 1.00 equiv.). After being stirred at 50 °C for 16 h, the reaction mixture was quenched with sat. aq. NaCl solution (70 mL). The product was extracted with CH_2Cl_2 (3 × 25 mL). The combined organic layers were dried over anhydrous $MgSO_4$ and concentrated under reduced pressure. The crude product was purified by flash column chromatography (pentane/ethyl acetate, 5:1 to 1:1) to afford **65** as a pale-yellow solid (123 mg, 0.28 mmol, 84%).

***N*, *N*-dimethyl-1-phenyl-3-tosyl-3,6-diazabicyclo[3.1.1]heptane-6-sulfonamide (65)**

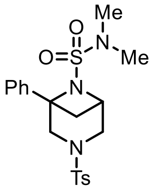

M.P.: 126-128 °C

^{1}H NMR (500 MHz, $CDCl_3$) δ (ppm) = 7.79 (d, J = 8.3 Hz, 2H), 7.48-7.45 (m, 2H), 7.40-7.31 (m, 5H), 4.39 (dd, J = 10.8, 1.4 Hz, 1H), 4.22 (dt, J = 6.3, 1.9 Hz, 1H), 3.97 (app. s, 1H), 3.94 (app. s, 1H), 3.65 (dd, J = 11.2, 2.3 Hz, 1H), 2.81 (dd, J = 8.5, 6.3 Hz, 1H), 2.40 (s, 3H), 2.30 (s, 6H), 2.00 (d, J = 8.7 Hz, 1H).

^{13}C NMR (126 MHz, $CDCl_3$) δ (ppm) = 143.57, 137.82, 134.71, 129.67, 129.16, 128.58, 127.58, 126.21, 72.29, 59.93, 47.12, 44.99, 37.14, 35.30, 21.66.

IR (ATR): $\tilde{\nu}$ (cm^{-1}) = 2987, 1449, 1340, 1276, 1261, 1160, 1145, 1104.

HRMS (ESI$^+$) calcd. for $C_{20}H_{26}N_3O_4S_2^+$ [M+H]$^+$: 436.1359, found: 436.1343.

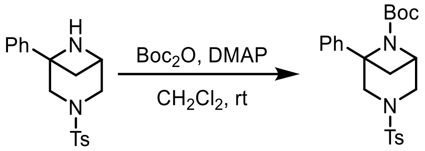

To an oven-dried 25 mL round-bottom flask was dissolved **61** (248 mg, 0.75 mmol, 1.00 equiv.) in anhydrous CH_2Cl_2 (7.5 mL) at room temperature. Boc_2O (245 mg, 1.12 mmol, 1.50 equiv.) was then added, followed by the addition of DMAP (18.4 mg, 0.15 mmol, 0.20 equiv.). After being stirred at room temperature for 1.5 h, the reaction mixture was quenched with sat. aq. NaCl solution (80 mL). The product was extracted with CH_2Cl_2 (3 × 25 mL). The combined organic layers were dried over anhydrous $MgSO_4$ and concentrated under reduced pressure. The crude product was purified by flash column chromatography (pentane/ethyl acetate, 5:1 to 1:1) to afford **66** as a white solid (240 mg, 0.56 mmol, 75%).

***Tert*-butyl 1-phenyl-3-tosyl-3,6-diazabicyclo[3.1.1]heptane-6-carboxylate (66)**

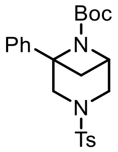

M.P.: 165-167 °C

^{1}H NMR (500 MHz, $CDCl_3$) δ (ppm) = 7.72 (d, *J* = 8.3 Hz, 2H), 7.37 (app. d, *J* = 7.7 Hz, 2H), 7.33-7.23 (m, 5H), 4.26-3.61 (m, 4H), 3.48 (dd, *J* = 10.7, 2.1 Hz, 1H), 2.42-2.33 (m, 4H), 1.91 (d, *J* = 8.8 Hz, 1H), 1.13 (s, 9H).

^{13}C NMR (126 MHz, $CDCl_3$) δ (ppm) = 155.38, 143.64, 139.60, 134.09, 129.84, 128.27, 127.81, 127.31, 125.25, 80.62, 69.71, 55.63, 48.15, 44.52, 35.50, 27.95, 21.41.

IR (ATR): $\tilde{\nu}$ (cm^{-1}) = 2979, 1791, 1735, 1703, 1449, 1340, 1227, 1163, 1090, 1057.

HRMS (ESI$^+$) calcd. for $C_{23}H_{28}N_2O_4SNa^+$ [M+Na]$^+$: 451.1662, found: 451.1662.

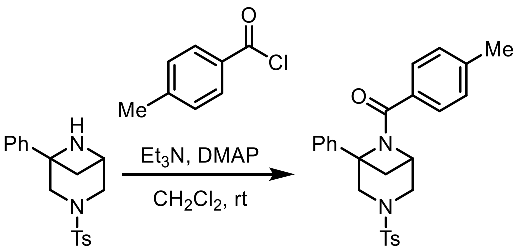

To an oven-dried 25 mL round-bottom flask was dissolved **61** (90.0 mg, 0.27 mmol, 1.00 equiv.) in anhydrous CH_2Cl_2 (2.7 mL) at room temperature. Et_3N (76 µL, 0.55 mmol, 2.00 equiv.) was then

added, followed by the addition of 4-methylbenzoyl chloride (47 μL, 0.36 mmol, 1.30 equiv.) and DMAP (6.7 mg, 0.055 mmol, 0.20 equiv.). After being stirred at room temperature for 1 h, the reaction mixture was quenched with sat. aq. NaCl solution (50 mL). The product was extracted with CH_2Cl_2 (3 × 20 mL). The combined organic layers were dried over anhydrous $MgSO_4$ and concentrated under reduced pressure. The crude product was purified by flash column chromatography (pentane/ethyl acetate, 5:1 to 1:1) to afford **67** as a white solid (120 mg, 0.27 mmol, 98%).

(1-Phenyl-3-tosyl-3,6-diazabicyclo[3.1.1]heptan-6-yl)(*p*-tolyl)methanone (67)

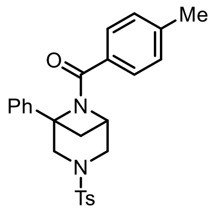

M.P.: 150-152 °C

^{1}H NMR (500 MHz, $CDCl_3$) δ (ppm) = 7.66 (d, *J* = 8.2 Hz, 2H), 7.43 (d, *J* = 7.6 Hz, 2H), 7.37-7.32 (m, 4H), 7.30-7.26 (m, 1H), 7.19-6.87 (m, 4H), 4.54 (dd, *J* = 6.6, 2.1 Hz, 1H), 4.43 (br. s, 1H), 3.97 (d, *J* = 10.6 Hz, 1H), 3.45 (dd, *J* = 11.0, 1.9 Hz, 1H), 2.98 (br. s, 1H), 2.66 (t, *J* = 7.7 Hz, 1H), 2.51 (s, 3H), 2.36 (s, 3H), 2.10 (d, *J* = 8.8 Hz, 1H).

^{13}C NMR (126 MHz, $CDCl_3$) δ (ppm) = 174.10, 143.64, 142.15, 139.51, 134.03, 131.21, 129.85, 129.05, 128.51, 127.97, 127.79, 127.60, 125.53, 70.73, 59.57, 48.87, 46.84, 35.33, 21.62, 21.56.

IR (ATR): $\tilde{\nu}$ (cm^{-1}) = 2988, 1649, 1533, 1496, 1448, 1340, 1276, 1261, 1163, 1098, 1031.

HRMS (ESI^+) calcd. for $C_{26}H_{27}N_2O_3S^+$ $[M+H]^+$: 447.1737, found: 447.1715.

1.14. Procedures for Analogue of DB04232 Synthesis

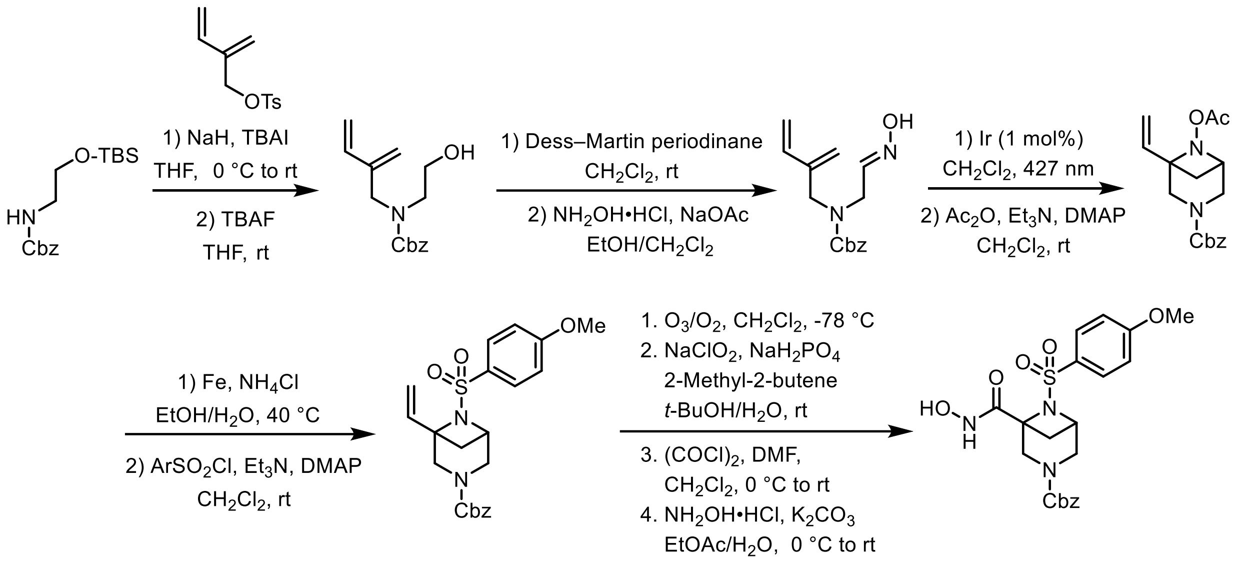

Benzyl (2-hydroxyethyl)(2-methylenebut-3-en-1-yl)carbamate (s30a)

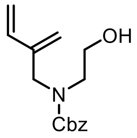

An oven-dried 100 mL round-bottom flask containing benzyl (2-((tert-butyldimethylsilyl)oxy)ethyl)carbamate [15] (4.06 g, 13.2 mmol, 1.00 equiv.) was sealed and subjected to three N_2 evacuation/refill cycles before pre-sparged anhydrous THF (33.0 mL, 0.4 M) was added at 0 °C. NaH (1.61 g, 60% purity, 39.6 mmol, 3.00 equiv.) was then added, and the reaction was stirred at 0 °C for 30 min before adding **S26a'** (3.13 g, 13.2 mmol, 1.00 equiv.) and tetrabutylammonium iodide (97.0 mg, 0.26 mmol, 2 mol%). The reaction mixture was allowed to warm up to room temperature and stirred overnight until completion (judged by TLC). The reaction was then quenched with sat. aq. NH_4Cl solution (200 mL) and extracted with ethyl acetate (3 × 50 mL). The combined organic layers were dried over anhydrous $MgSO_4$, filtered and concentrated under reduced pressure. The crude product was purified by flash column chromatography (pentane/ethyl acetate, 20:1 to 8:1) to afford the coupling product as a colorless oil, which was used for the next deprotection reaction without further purification.

The coupling product (1.00 equiv.) was dissolved in THF (26.4 mL, 0.5 M) in an oven-dried 100 mL round-bottom flask. Tetrabutylammonium fluoride (TBAF, 13.2 mL, 1.0 M solution in THF, 13.2 mmol, 1.00 equiv.) was then added and the reaction mixture was stirred in an open flask at room temperature for 0.5 h until completion (judged by TLC). The reaction was quenched with brine (200 mL) and extracted with ethyl acetate (3 × 60 mL). The combined organic layers were dried over

anhydrous $MgSO_4$ and concentrated under reduced pressure and purified by flash column chromatography (pentane/ethyl acetate, 5:1 to 1:1) to afford **S30a** as a colorless oil (2.92 g, 11.2 mmol, 85%).

Note: Two sets of peaks were observed in ^1H and ^{13}C NMR spectra due to carbamate rotamers.

^{1}H NMR (600 MHz, $CDCl_3$) δ (ppm) = 7.36-7.27 (m, 5H), 6.42-6.33 (m, 1H), 5.30-4.98 (m, 6H), 4.24 -4.13 (m, 2 H), 3.77-3.64 (m, 2H), 3.46-3.34 (m, 2H), 3.20 (br. s, 0.6 H), 2.61 (br. s, 0.4 H).

^{13}C NMR (151 MHz, $CDCl_3$) δ (ppm) = 157.53, 156.37, 141.31, 141.04, 136.77, 136.61, 136.43, 128.52, 128.46, 128.02, 127.85, 127.75, 116.74, 115.96, 114.80, 114.25, 67.44, 67.39, 61.40, 60.83, 49.91, 49.06, 48.94, 48.32.

IR (ATR): $\tilde{\nu}$ (cm^{-1}) = 3431, 2944, 1684, 1597, 1472, 1455, 1419, 1366, 1235, 1124, 1053.

HRMS (ESI^+) calcd. for $C_{15}H_{20}NO_3^+$ $[M+H]^+$: 262.1438, found: 262.1429.

Benzyl (2-(hydroxyimino)ethyl)(2-methylenebut-3-en-1-yl)carbamate (S30b)

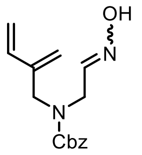

Alcohol **S30a** (2.57 g, 9.85 mmol, 1.00 equiv.) was dissolved in CH_2Cl_2 (20 mL, 0.5 M) in an oven-dried 100 mL round-bottom flask. Dess-Martin periodinane (6.26 g, 14.76 mmol, 1.50 equiv.) was then added and the reaction mixture was stirred in an open flask at room temperature for 0.5 h until completion (judged by TLC). The reaction was quenched with sat. aq. $NaHCO_3$ solution (200 mL) and extracted with CH_2Cl_2 (3 × 60 mL). The combined organic layers were dried over anhydrous $MgSO_4$ and concentrated under reduced pressure. The crude aldehyde was then dissolved in ethanol (100 mL, 0.1 M) and CH_2Cl_2 (10 mL, 1.0 M) in an oven-dried 250 mL round-bottom flask before sodium acetate (3.23 g, 39.4 mmol, 4.00 equiv.) and hydroxylamine hydrochloride (1.37 g, 19.7 mmol, 2.00 equiv.) were added. The reaction was stirred at room temperature for 1 h until completion (judged by TLC). The reaction was quenched with sat. aq. $NaHCO_3$ solution (200 mL) and extracted with CH_2Cl_2 (3 × 60 mL). The combined organic layers were dried over anhydrous $MgSO_4$, filtered, concentrated under reduced pressure and purified by flash column chromatography (pentane/ethyl acetate, 5:1 to 1:1) to afford **S30b** as a colorless oil (1.81 g, 6.61 mmol, 67%).

Note: Four sets of peaks were observed in ^1H and ^{13}C NMR spectra due to carbamate rotamers and *E/Z* isomers.

^{1}H NMR (500 MHz, $CDCl_3$) δ (ppm) = 9.04 (br. s, 1H), 7.53- 7.27 (m, 5.5 H), 6.84-6.63 (m, 0.5H), 6.42-6.29 (m, 1H), 5.41-4.98 (m, 6H), 4.25-3.93 (m, 4H).

^{13}C NMR (126 MHz, $CDCl_3$) δ (ppm) = 156.48, 156.45, 156.12, 156.02, 150.06, 149.64, 147.00, 140.91, 140.85, 140.60, 140.54, 136.65, 136.62, 136.33, 136.29, 128.55, 128.51, 128.16, 128.14, 128.00, 127.95, 127.91, 127.84, 118.44, 117.81, 117.11, 116.42, 115.44, 115.24, 114.62, 114.45, 67.77, 67.74, 67.72, 49.30, 48.86, 48.07, 47.44, 45.50, 44.66, 42.68, 41.91.

IR (ATR): $\tilde{\nu}$ (cm^{-1}) = 3349, 3090, 1687, 1596, 1456, 1417, 1368, 1275, 1235, 1125.

HRMS (ESI^+) calcd. for $C_{15}H_{19}N_2O_3^+$ $[M+H]^+$: 275.1390, found: 275.1382.

Benzyl 6-acetoxy-1-vinyl-3,6-diazabicyclo[3.1.1]heptane-3-carboxylate (30)

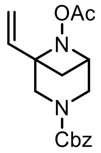

An oven-dried 50 mL round-bottom flask containing oxime **S30b** (700 mg, 2.55 mmol, 1.00 equiv.) and $[Ir(dF(CF_3)ppy)_2(dtbbpy)](PF_6)$ (28.6 mg, 0.026 mmol, 1 mol%) was sealed and subjected to three N_2 evacuation/refill cycles before pre-sparged anhydrous CH_2Cl_2 (25.5 mL, 0.1 M) was added. The flask was placed close to a 40 W KSPR160L-427 nm Kessil light (100% intensity) at a distance of approximately 1 cm (fan cooling) and the reaction stirred under nitrogen atmosphere for 1.5 h until complete (judged by TLC analysis). Et_3N (533 µL, 3.82 mmol, 1.50 equiv.) was then added, followed by the addition of DMAP (31.2 mg, 0.26 mmol, 0.10 equiv.) and Ac_2O (313 µL, 3.31 mmol, 1.30 equiv.). After being stirred at room temperature for 30 min, the reaction mixture was quenched with brine (200 mL). The product was extracted with CH_2Cl_2 (3 × 60 mL). The combined organic layers were dried over anhydrous $MgSO_4$ and concentrated under reduced pressure. The crude product was purified by flash column chromatography (pentane/ethyl acetate, 5:1 to 1:1) to afford **30** as a colorless oil (363 mg, 1.15 mmol, 45%).

Note: (1) In this case, the precursor **S30b** is completely consumed after 1.5 h (by TLC) and the rearrangement product was also observed. At this point, the light should be turned off and the anhydride should be added to the reaction immediately. Reactions are recommended to be monitored by TLC every 15 mins after 1 hour from the start of the reaction.

(2) Two sets of peaks were observed in ^{1}H and ^{13}C NMR spectra due to carbamate rotamers.

^{1}H NMR (500 MHz, $CDCl_3$) δ (ppm) = 7.34-7.21 (m, 5H), 5.85-5.75 (m, 1H), 5.29 (dd, J = 4.9, 1.1 Hz, 0.55H), 5.25 (dd, J = 4.9, 1.0 Hz, 0.45H), 5.15-5.08 (m, 3H), 4.09-4.06 (m, 0.45H), 4.04-3.98 (m,

0.55H), 3.62-3.50 (m, 4H), 2.10-2.00 (m, 1H), 1.94 (s, 1.65H), 1.93 (s, 1.35H), 1.82 (d, J = 2.7 Hz, 0.55H), 1.80 (d, J = 2.6 Hz, 0.45H).

^{13}C NMR (126 MHz, $CDCl_3$) δ (ppm) = 169.04, 169.02, 155.66, 155.62, 136.28, 136.22, 136.09, 128.29, 128.27, 127.87, 127.85, 127.73, 127.66, 116.72, 116.66, 71.76, 71.70, 66.92, 66.89, 60.94, 60.90, 45.23, 41.79, 41.63, 28.48, 28.44, 19.03.

IR (ATR): $\tilde{\nu}$ (cm^{-1}) = 2973, 2883, 1751, 1702, 1410, 1357, 1321, 1275, 1261, 1210, 1111.

HRMS (ESI$^+$) calcd. for $C_{17}H_{20}N_2O_4Na^+$ [M+Na]$^+$: 339.1315, found: 339.1306.

Benzyl 6-((4-methoxyphenyl)sulfonyl)-1-vinyl-3,6-diazabicyclo[3.1.1]heptane-3-carboxylate (68a)

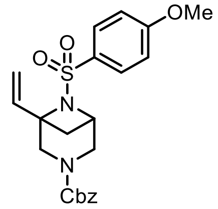

In an oven-dried 25 mL round-bottom flask was dissolved **30** (90 mg, 0.28 mmol, 1.00 equiv.) in EtOH/H_2O (1.1 mL/0.3 mL) at room temperature. Iron powder (157 mg, 2.8 mmol, 10.0 equiv.) and ammonium chloride (150 mg, 2.8 mmol, 10.0 equiv.) were then added and the reaction was stirred at 40 °C for 15 h. The reaction mixture was then allowed to cool to room temperature, filtered through a short pad of $MgSO_4$ (washed by CH_2Cl_2) and concentrated under reduced pressure. The crude product was used for next step without further purification.

In an oven-dried 25 mL round-bottom flask were dissolved crude *N*-H intermediate in anhydrous CH_2Cl_2 (2.8 mL, 0.1 M) at room temperature. Et_3N (78 µL, 0.56 mmol, 2.00 equiv.) was then added, followed by the addition of DMAP (6.8 mg, 0.056 mmol, 0.20 equiv.) and 4-methoxybenzenesulfonyl chloride (87.0 mg, 0.42 mmol, 1.50 equiv.). After being stirred at room temperature for 1 h, the reaction mixture was quenched with sat. aq. NaCl solution (60 mL). The product was extracted with CH_2Cl_2 (3 × 20 mL). The combined organic layers were dried over anhydrous $MgSO_4$ and concentrated under reduced pressure. The crude product was purified by flash column chromatography (pentane/ethyl acetate, 5:1 to 1:1) to afford **68a** as a colorless oil (51.4 mg, 0.12 mmol, 43% for two steps).

Note: Two sets of peaks were observed in ^{1}H and ^{13}C NMR spectra due to carbamate rotamers.

^{1}H NMR (500 MHz, $CDCl_3$) δ (ppm) = 7.77 (d, J = 4.1 Hz, 1.1H), 7.75 (d, J = 4.1 Hz, 0.9H), 7.38-7.27 (m, 5H), 6.93-6.87 (m, 2H), 5.97-5.85 (m, 1H), 5.21-5.13 (m, 3H), 5.05 (d, J = 12.5 Hz, 0.55H), 5.02 (d, J = 12.4 Hz, 0.45H), 4.23 (dt, J = 6.5, 2.0 Hz, 0.45H), 4.19 (dt, J = 6.5, 1.9 Hz, 0.55H), 4.13-

4.00 (m, 2H), 3.80 (s, 1.65H), 3.80 (s, 1.35H), 3.53-3.43 (m, 2H), 2.52-2.46 (m, 1H), 1.62 (d, J = 1.4 Hz, 0.55H), 1.60 (d, J = 1.4 Hz, 0.45H).

^{13}C NMR (126 MHz, $CDCl_3$) δ (ppm) = 163.10, 163.08, 155.85, 155.78, 136.48, 136.45, 135.61, 135.52, 132.20, 132.17, 129.63, 129.61, 128.48, 128.01, 127.99, 127.81, 127.76, 117.82, 117.70, 114.033, 114.025, 71.87, 71.80, 67.03, 67.02, 58.92, 58.82, 55.60, 55.59, 48.81, 48.64, 45.76, 45.66, 35.92, 35.82.

IR (ATR): $\tilde{\nu}$ (cm^{-1}) = 3007, 1701, 1595, 1498, 1443, 1406, 1309, 1275, 1260, 1151, 1098, 1021.

HRMS (ESI^+) calcd. for $C_{22}H_{24}N_2O_5SNa^+$ $[M+Na]^+$: 451.1298, found: 451.1284.

Benzyl 1-(hydroxycarbamoyl)-6-((4-methoxyphenyl)sulfonyl)-3,6-diazabicyclo[3.1.1]heptane-3-carboxylate (68)

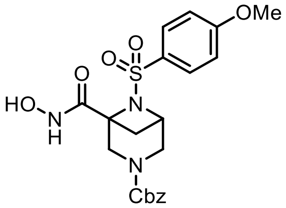

To a solution of alkene **68a** (188 mg, 0.44 mmol, 1.00 equiv.) in CH_2Cl_2 (8.8 mL, 0.05 M) cooled to −78 °C, a stream of O_3-$O_{2(g)}$ was gently bubbled through the solution for ~10 min (the solution became blue). The reaction was then purged by bubbling a gentle stream of $N_{2(g)}$ through the solution for 20 min, which was followed by the addition of Me_2S (193 µL, 2.63 mmol, 6.00 equiv.). The mixture was stirred at −78 °C for 5 min followed by 1 h at room temperature. The solution was then concentrated under reduced pressure and the crude aldehyde was then dissolved in *t*-BuOH (7.0 mL) and H_2O (1.8 mL) in a 50 mL round-bottom flask before NaH_2PO_4 (264 mg, 2.2 mmol, 5.00 equiv.), 2-methyl-2-butene (0.93 mL, 8.8 mmol, 20.0 equiv.) and $NaClO_2$ (249 mg, 80% purity, 2.2 mmol, 5.00 equiv.) was added. The reaction was stirred at room temperature for 12 h and was quenched with 1 M aqueous HCl (60 mL) and extracted with ethyl acetate (3 × 20 mL). The combined organic layers were dried over anhydrous $MgSO_4$, filtered, concentrated under reduced pressure and purified by flash column chromatography (pentane/ethyl acetate, 5:1 to 1:1 to 100 % ethyl acetate) to afford carboxylic acid as a colorless oil (108 mg, 0.24 mmol). The carboxylic acid was used for the next step without further purification.

An oven-dried 25 mL round-bottom flask containing carboxylic acid (108 mg, 0.24 mmol, 1.00 equiv.) was sealed and subjected to three N_2 evacuation/refill cycles before pre-sparged anhydrous CH_2Cl_2 (2.4 mL, 0.1 M) was added. Oxalyl chloride (41 µL, 0.48 mmol, 2.00 equiv.) was then added at 0 °C, followed by the addition of DMF (3 drops). The reaction mixture was allowed to warm up to room

temperature and stirred for 2 h. Then it was concentrated under reduced pressure to afford the crude acyl chloride and used for the next step without further purification.

In an oven-dried 25 mL round-bottom flask, hydroxylamine hydrochloride (50.0 mg, 0.72 mmol, 3.00 equiv.) was added to a biphasic mixture of K_2CO_3 (132 mg, 0.96 mmol, 4.00 equiv.) in EtOAc/H_2O solution (1.6 mL/0.8 mL). The reaction mixture was cooled to 0 °C, and crude acyl chloride dissolved in EtOAc (0.8 mL) was added. The mixture was warmed up to room temperature and stirred for 1 h until completion (judged by TLC). The reaction was quenched with brine (50 mL) and extracted with ethyl acetate (3 × 15 mL). The combined organic layers were washed with brine, dried over anhydrous $MgSO_4$, filtered and concentrated under reduced pressure. The crude product was purified by flash column chromatography (100% ethyl acetate to ethyl acetate/MeOH, 10:1) to afford **68** as a white solid (56 mg, 0.12 mmol, 28%).

Note: Two sets of peaks were observed in ^{1}H and ^{13}C NMR spectra due to carbamate rotamers.

M.P.: 88-90 °C

^{1}H NMR (500 MHz, $CDCl_3$) δ (ppm) = 10.29 (s, 0.6H), 10.22 (s, 0.4H), 8.19 (br. s, 1H), 7.84-7.79 (m, 2H), 7.40-7.27 (m, 5H), 6.98 (d, J = 9.0 Hz, 1.2H), 6.93 (d, J = 8.8 Hz, 0.8H), 5.14-5.06 (m, 1H), 5.00 (d, J = 12.4 Hz, 0.4H), 4.90 (d, J = 12.4 Hz, 0.6H), 4.15-4.00 (m, 2H), 3.88-3.75 (m, 5H), 3.41-3.35 (m, 1H), 2.69-2.61 (m, 1H), 1.86-1.79 (m, 1H).

^{13}C NMR (126 MHz, $CDCl_3$) δ (ppm) = 165.87, 165.77, 164.18, 164.14, 155.61, 155.57, 136.29, 136.03, 130.28, 130.09, 129.56, 129.16, 128.70, 128.67, 128.33, 128.29, 127.97, 127.92, 114.83, 114.82, 71.73, 71.42, 67.43, 59.67, 59.43, 55.93, 55.88, 46.50, 46.31, 44.62, 44.57, 35.82, 35.62.

IR (ATR): $\tilde{\nu}$ (cm^{-1}) = 3275, 3007, 1686, 1595, 1498, 1412, 1307, 1275, 1261, 1151, 1092, 1022.

HRMS (ESI^+) calcd. for $C_{21}H_{23}N_3O_7SNa^+$ $[M+Na]^+$: 484.1149, found: 484.1159.

1.15. Electrochemical Measurements of Compound 3

Cyclic voltammetry was performed at room temperature using MultiPalmSens 4. CV analysis with a platinum working electrode, a platinum counter electrode, an Ag/$AgNO_3$ (0.1 M) reference electrode, and a scan rate of 100 mV/s. The experimental setup was calibrated using ferrocene (Fc+/Fc) prior to each experiment. Samples were prepared with 0.1 mmol substrate in 10 mL *n*-Bu_4NPF_6 electrolyte (0.1 M in MeCN) and degassed by sparging with argon gas for 10 min prior to use. The potential ($E_{1/2}$) was determined and referenced to the SCE as described by Nicewicz.[16]

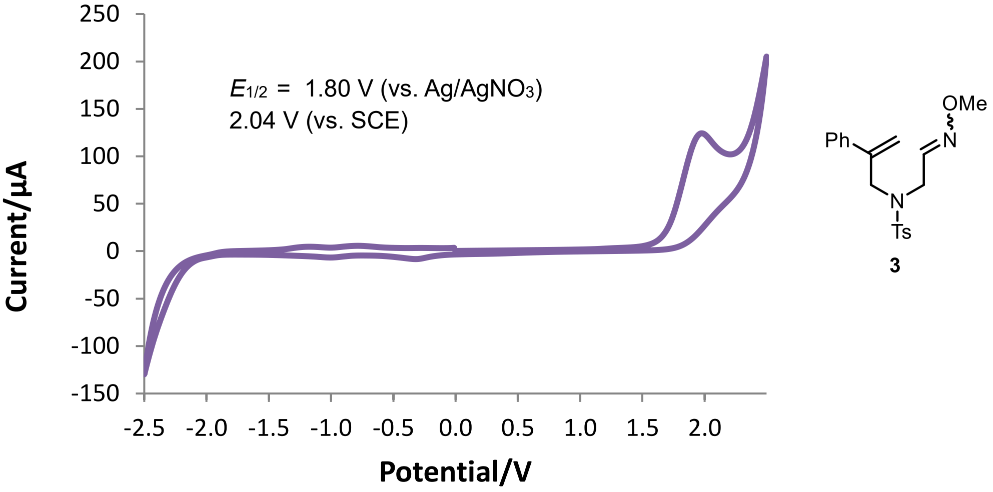

Supplementary Figure S3. Cyclic voltammogram of compound **3**.

The cyclic voltammogram shows an irreversible oxidation process with $E_{1/2}$ = +2.04 V (vs. SCE) of compound **3**. [Ir(dF(CF_3)ppy)$_2$(dtbbpy)]PF_6 ($E_{1/2}^{III*/II}$ = +1.21 V vs. SCE)[17] does not possess an excited state oxidation potential sufficient to oxidize **3**, thus, a photoredox process is unlikely.

2. NMR Spectra

Compound S4a

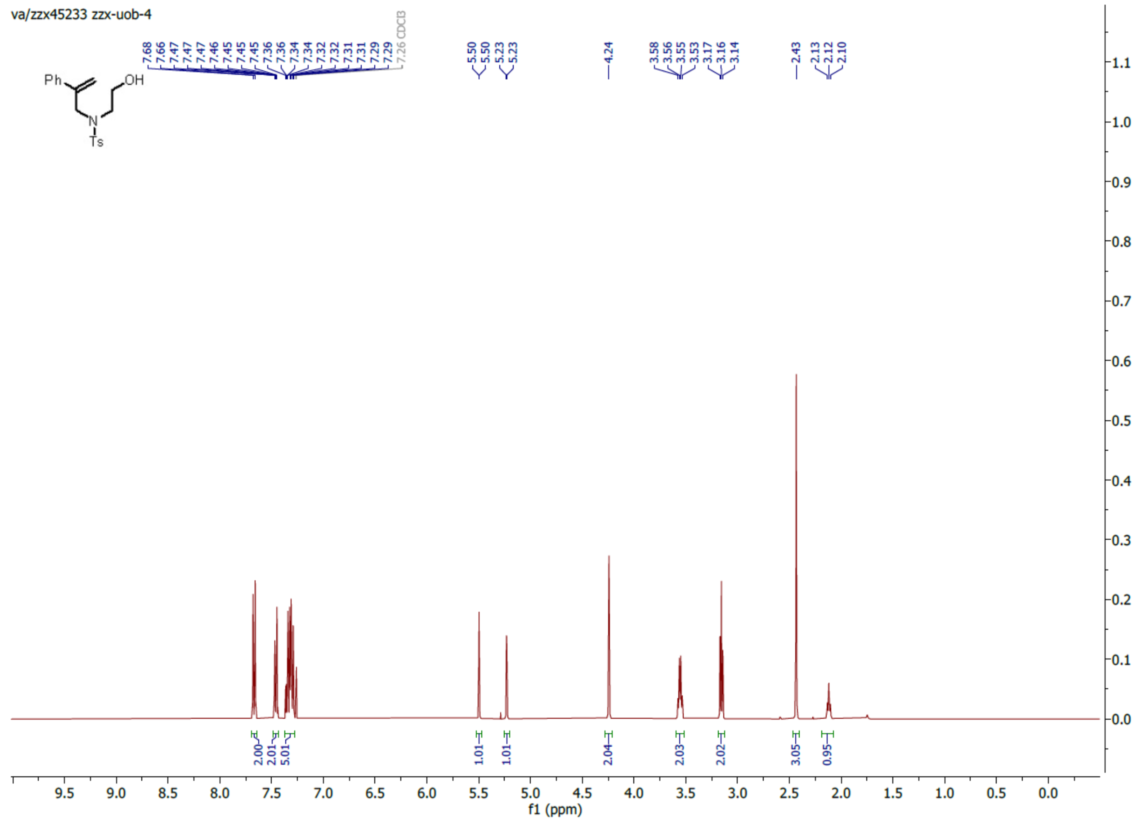

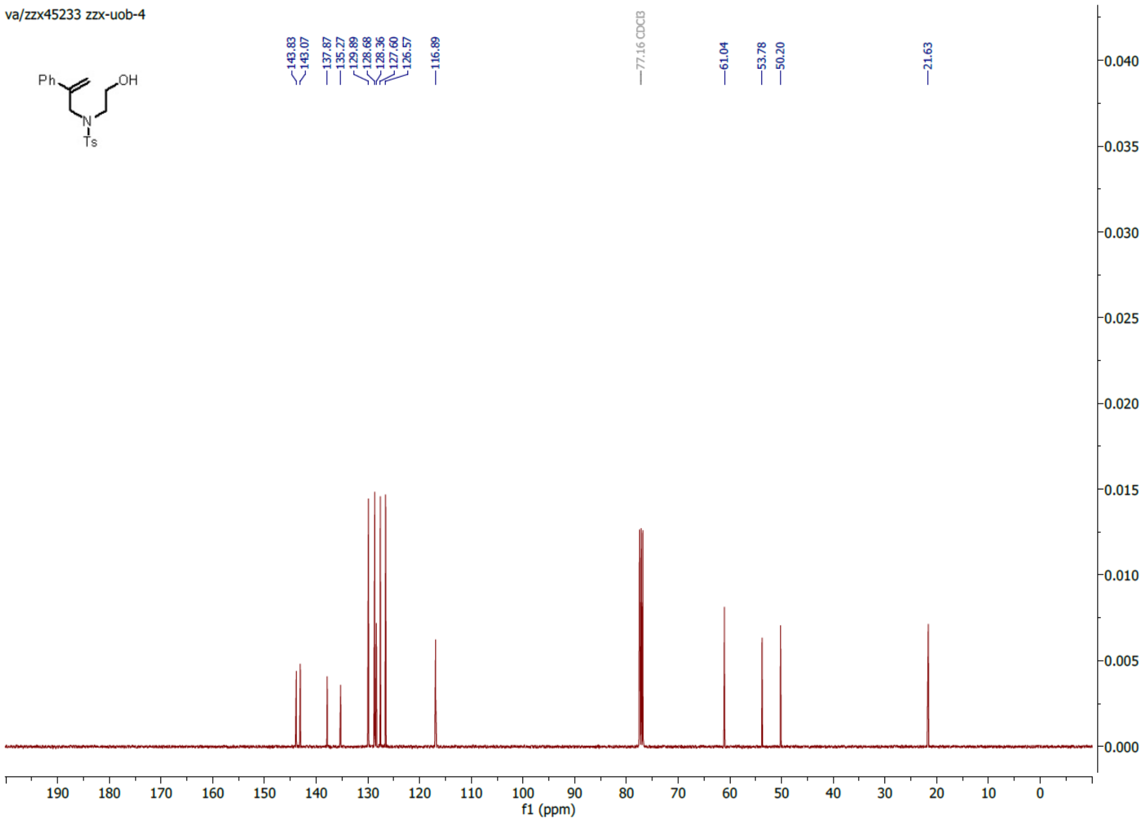

Compound 3

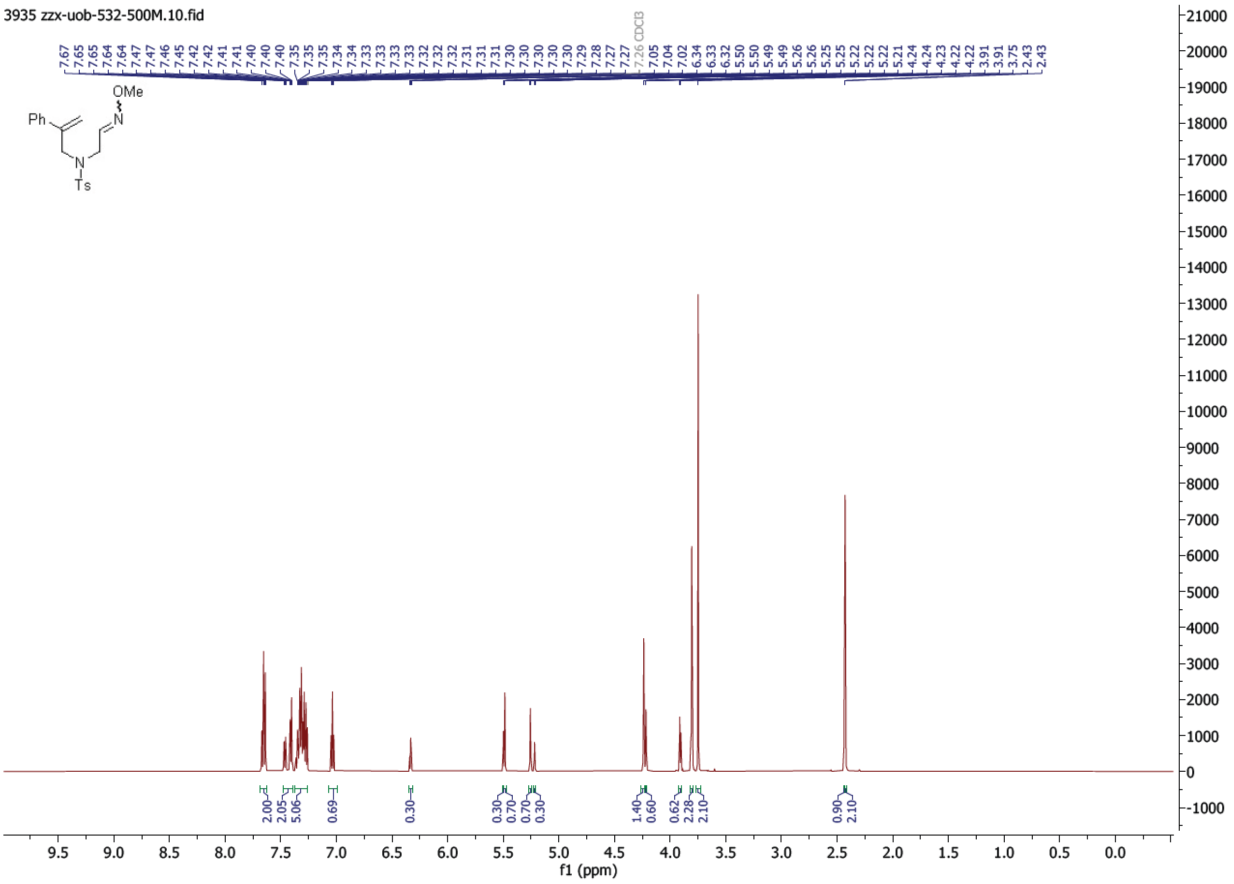

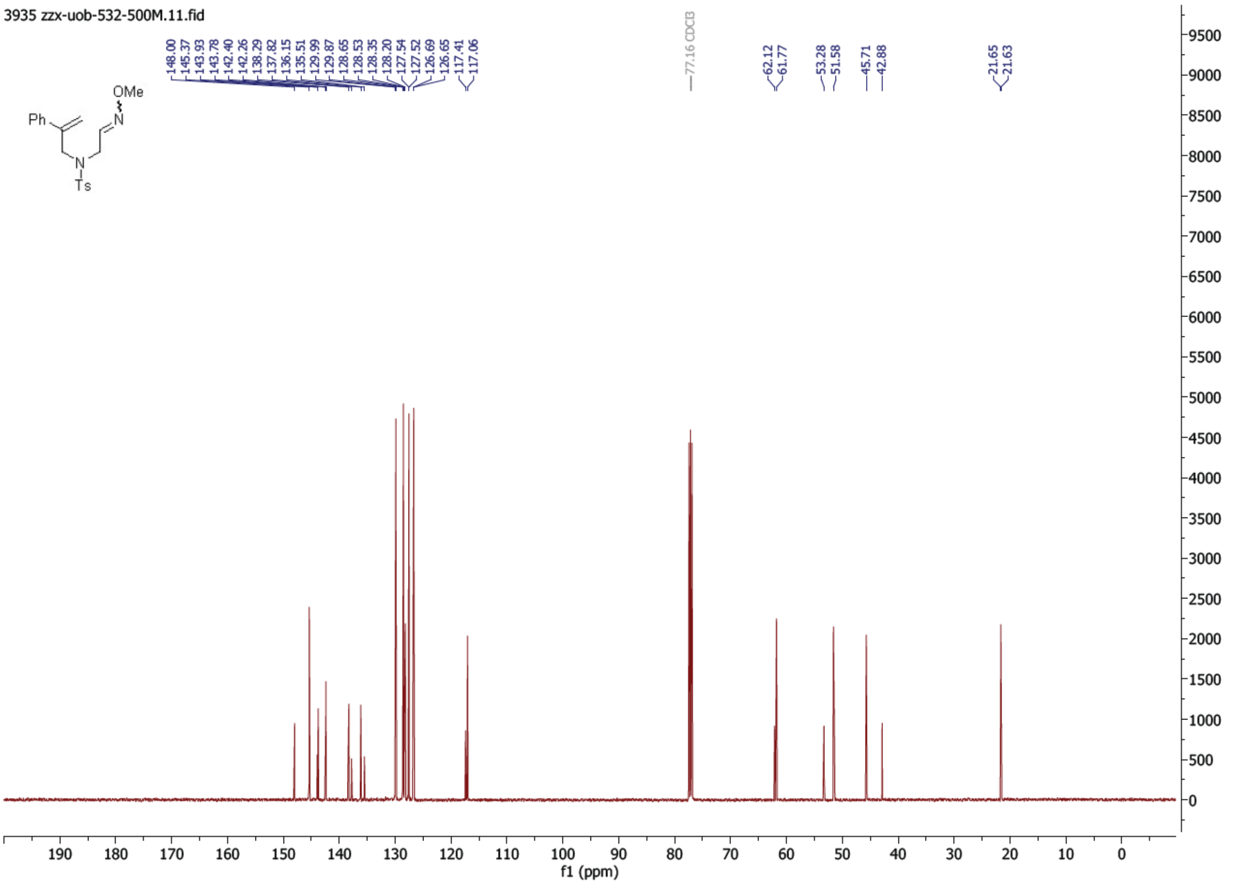

Compound S26a'

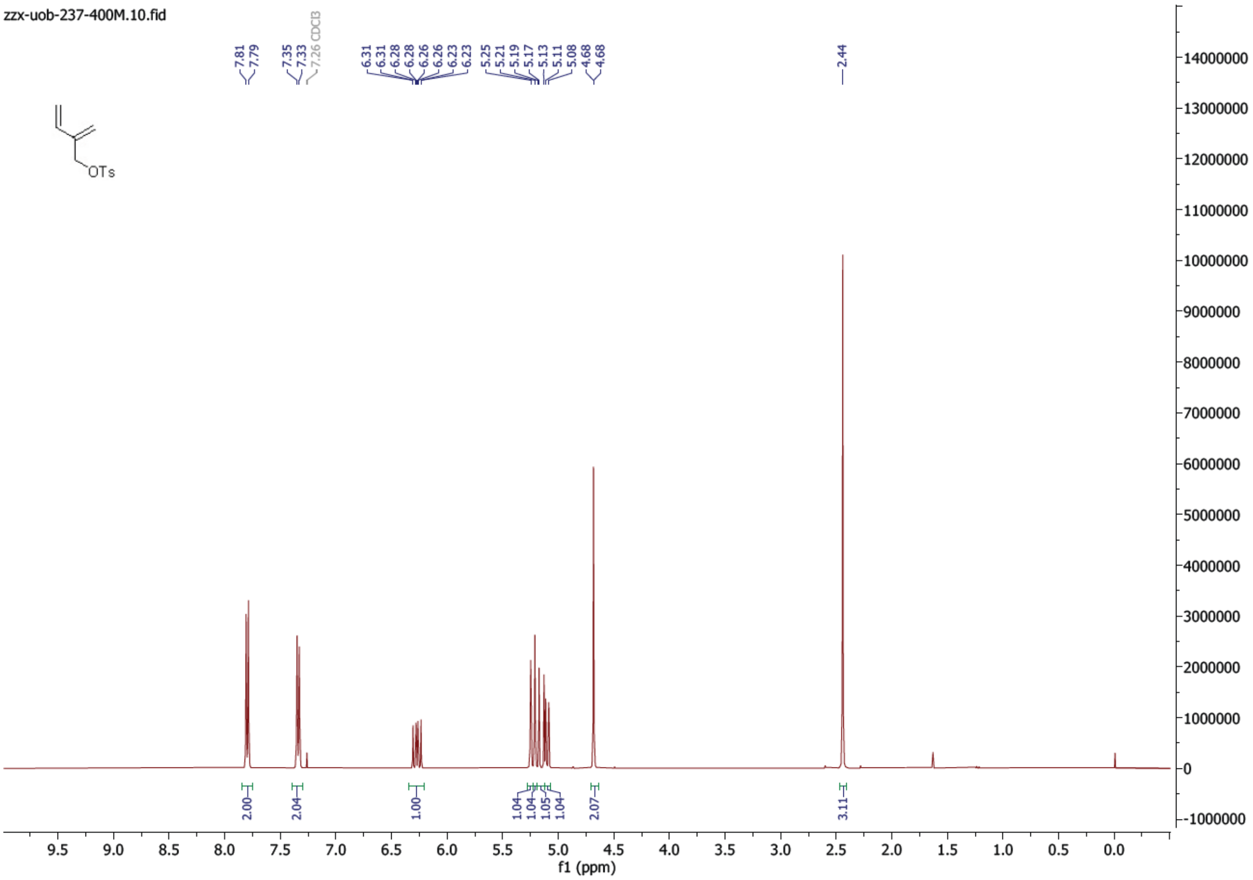

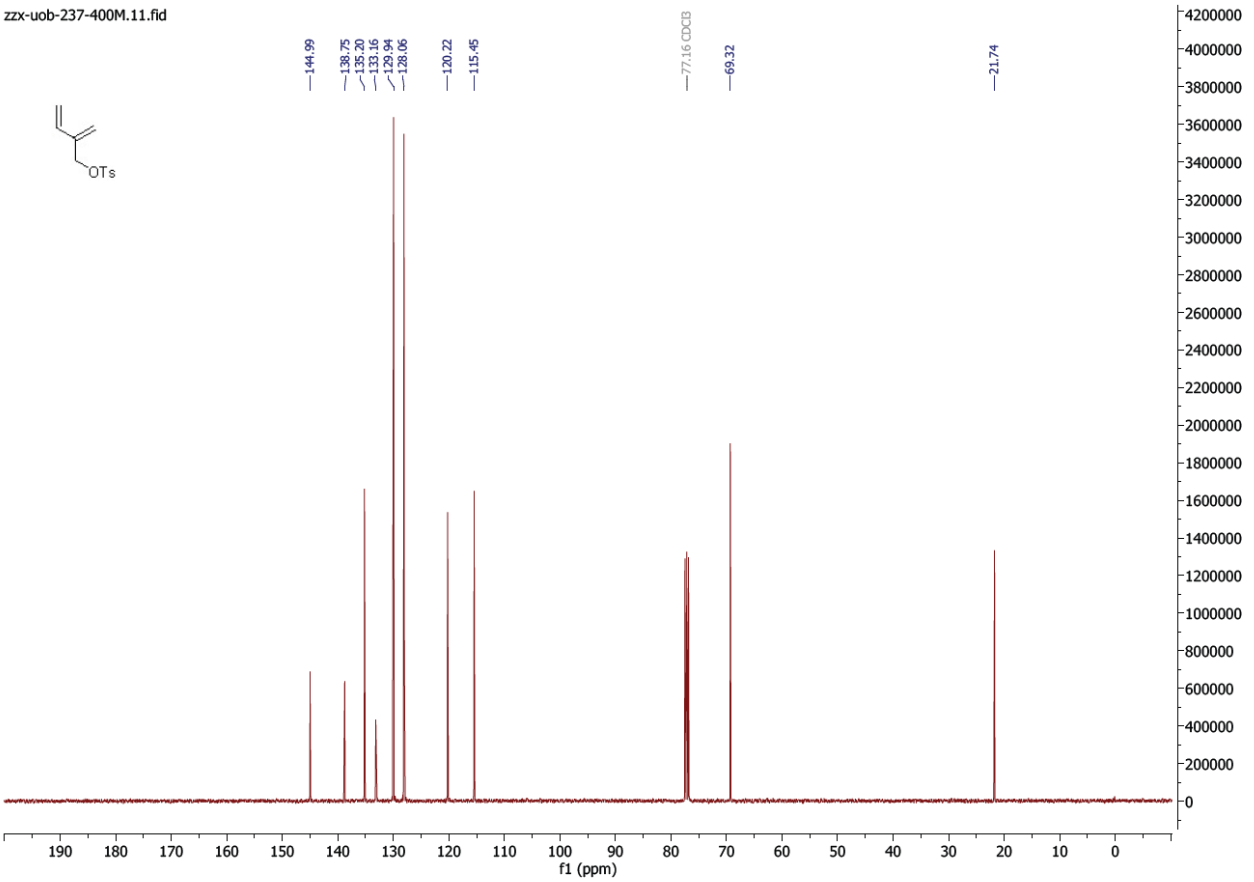

Compound S26a

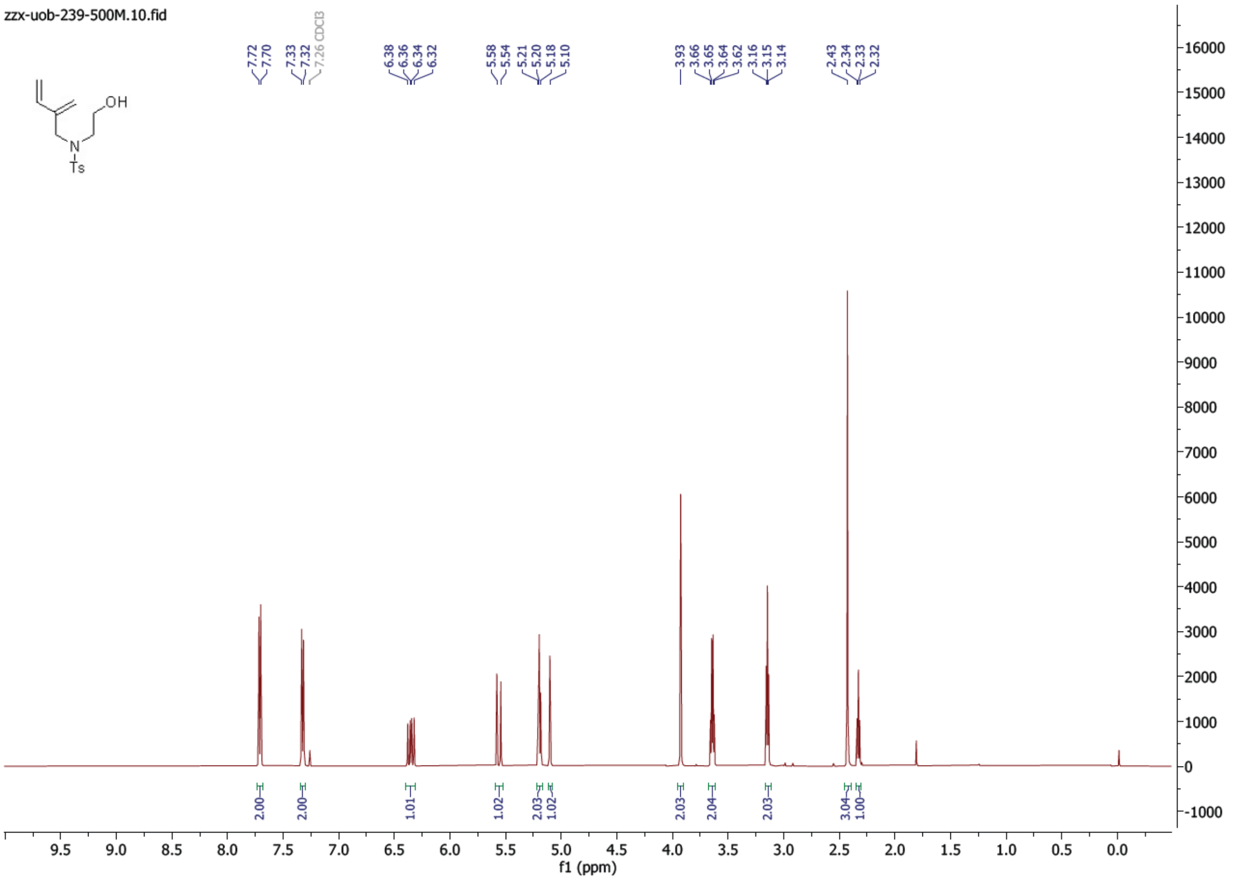

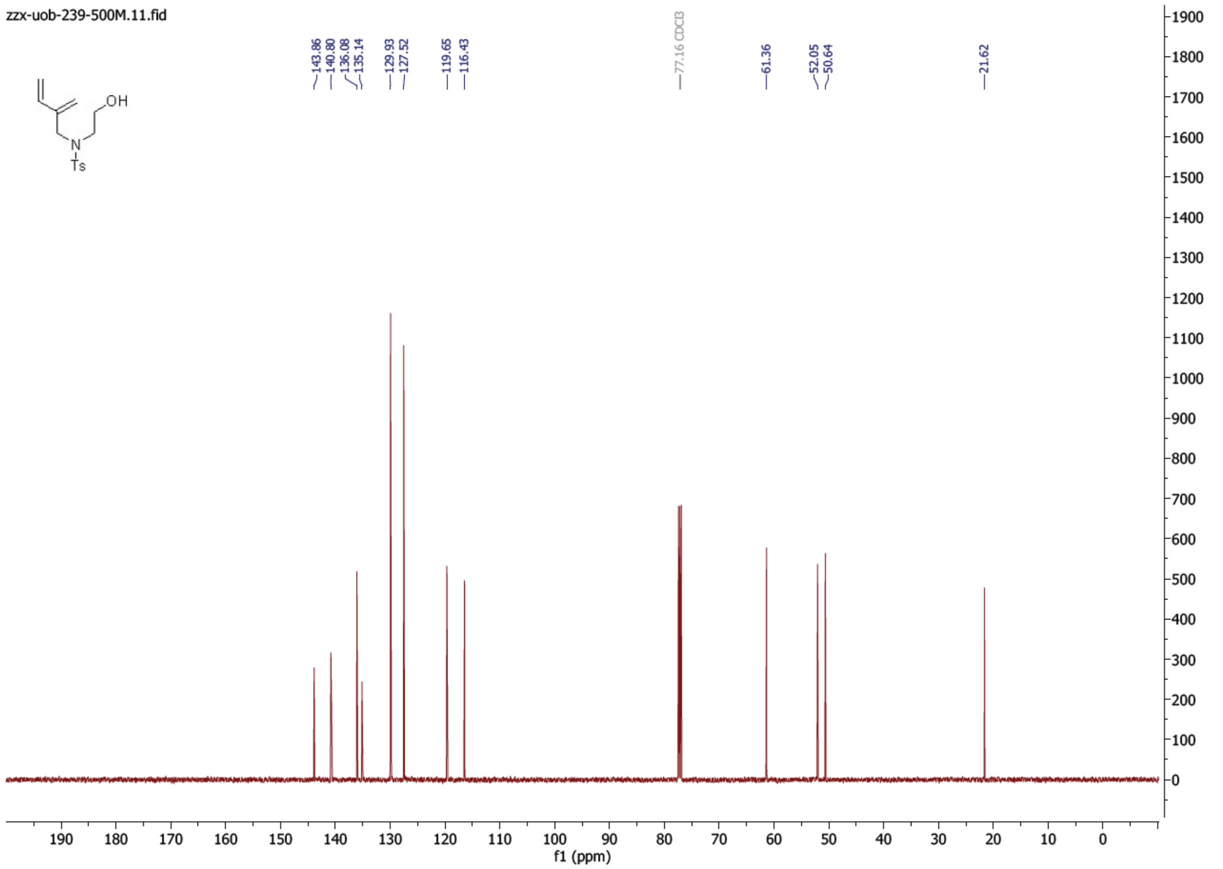

Compound S26b

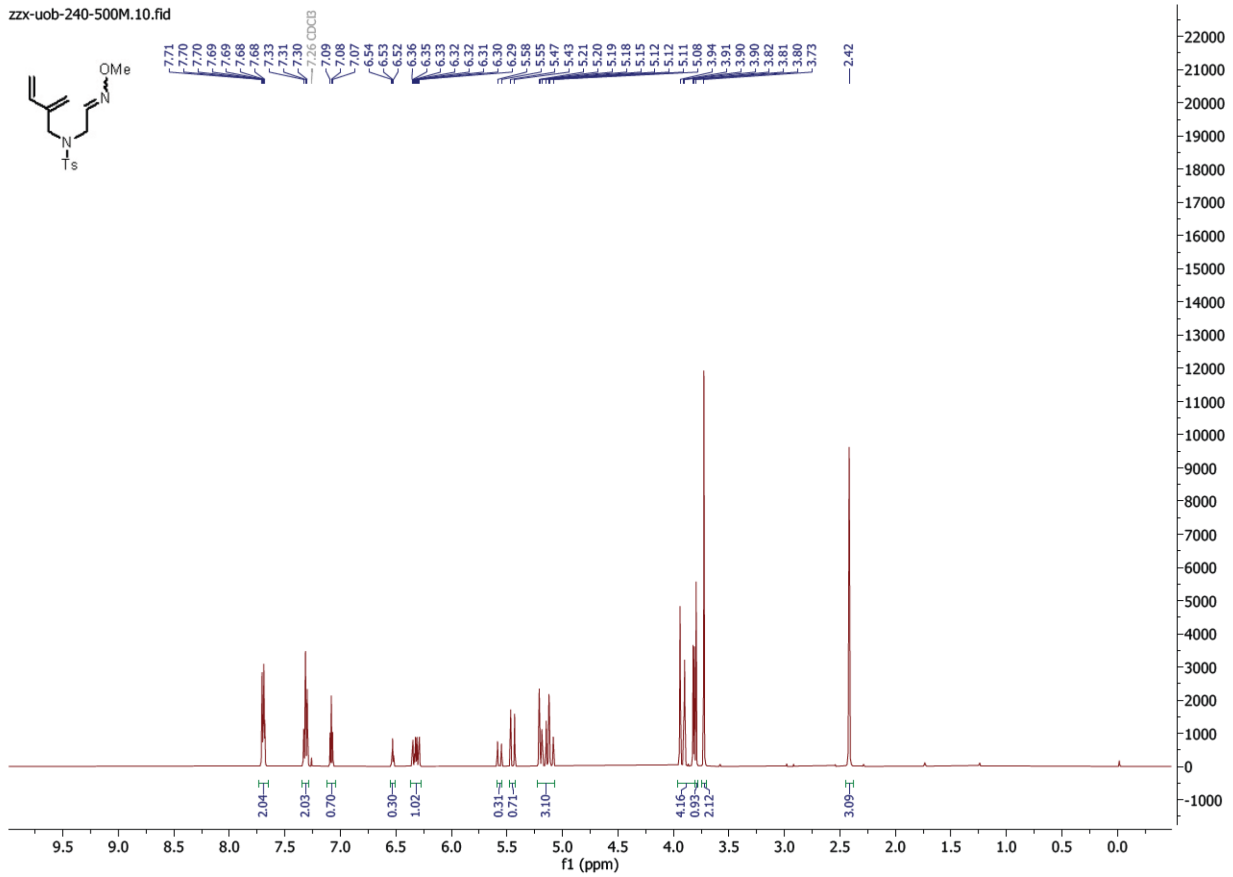

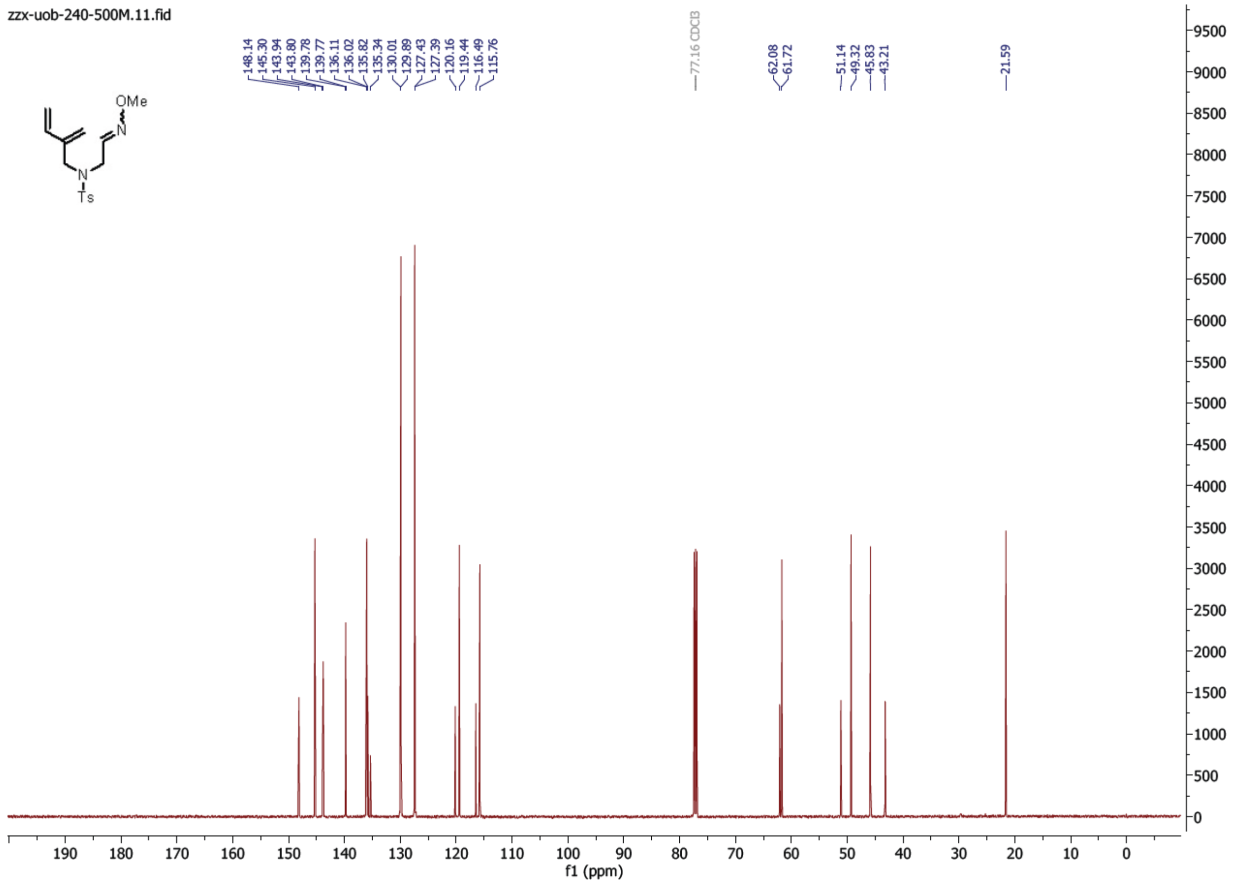

Compound S29a

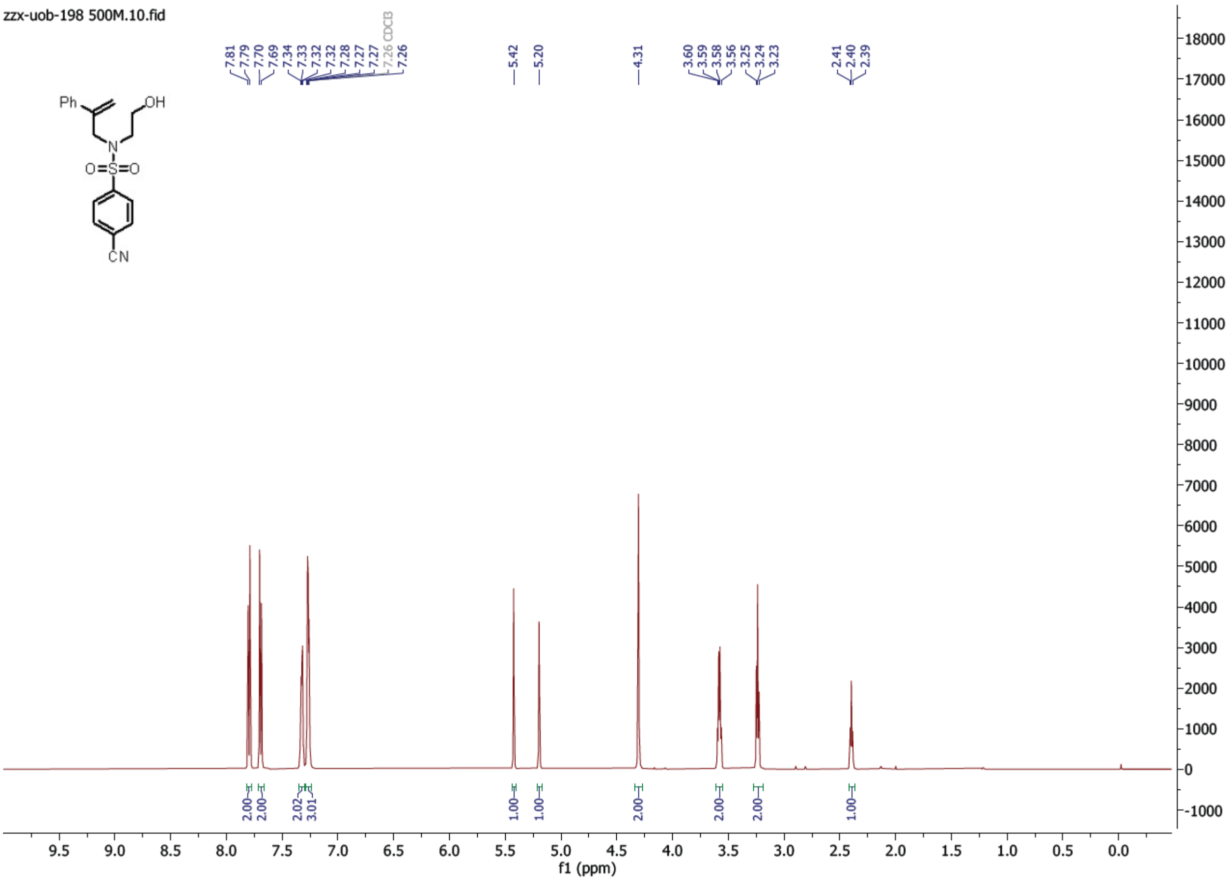

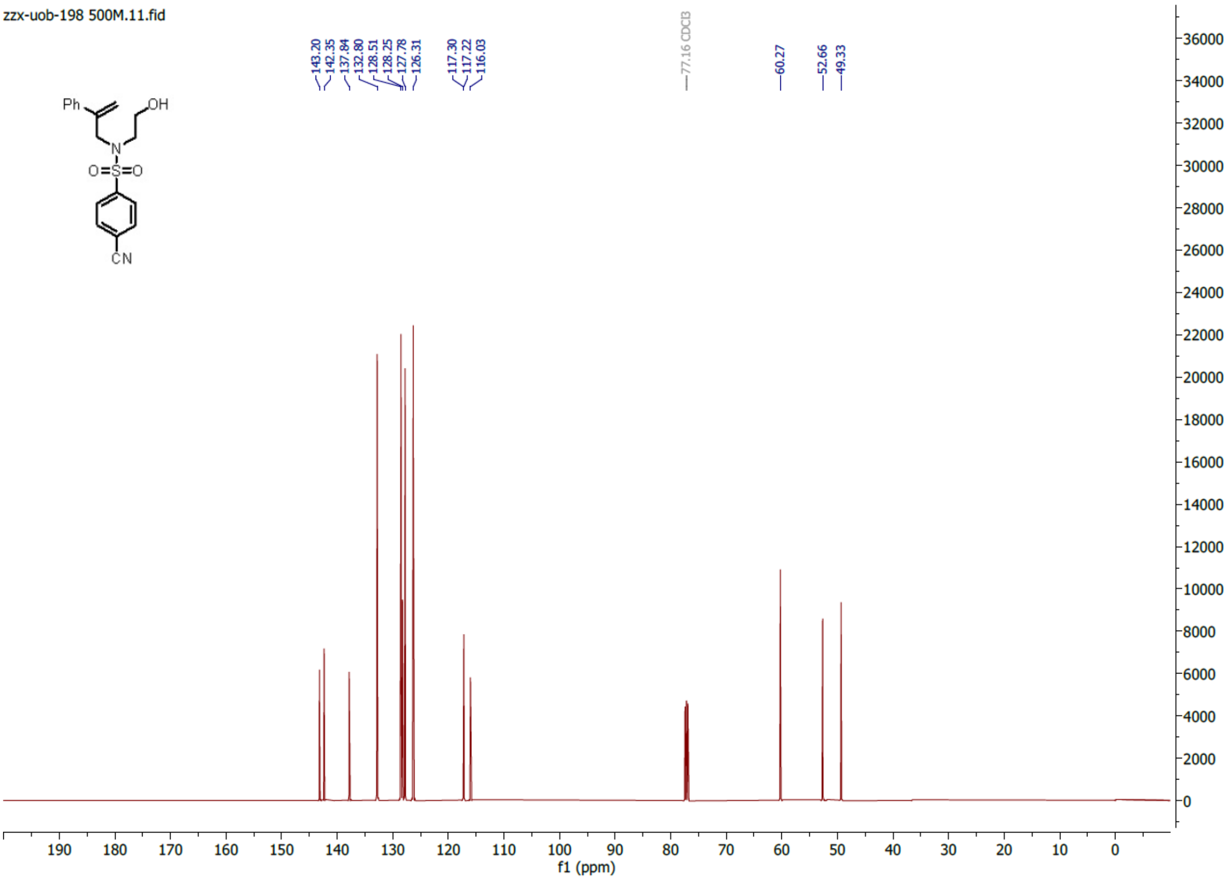

Compound S29b

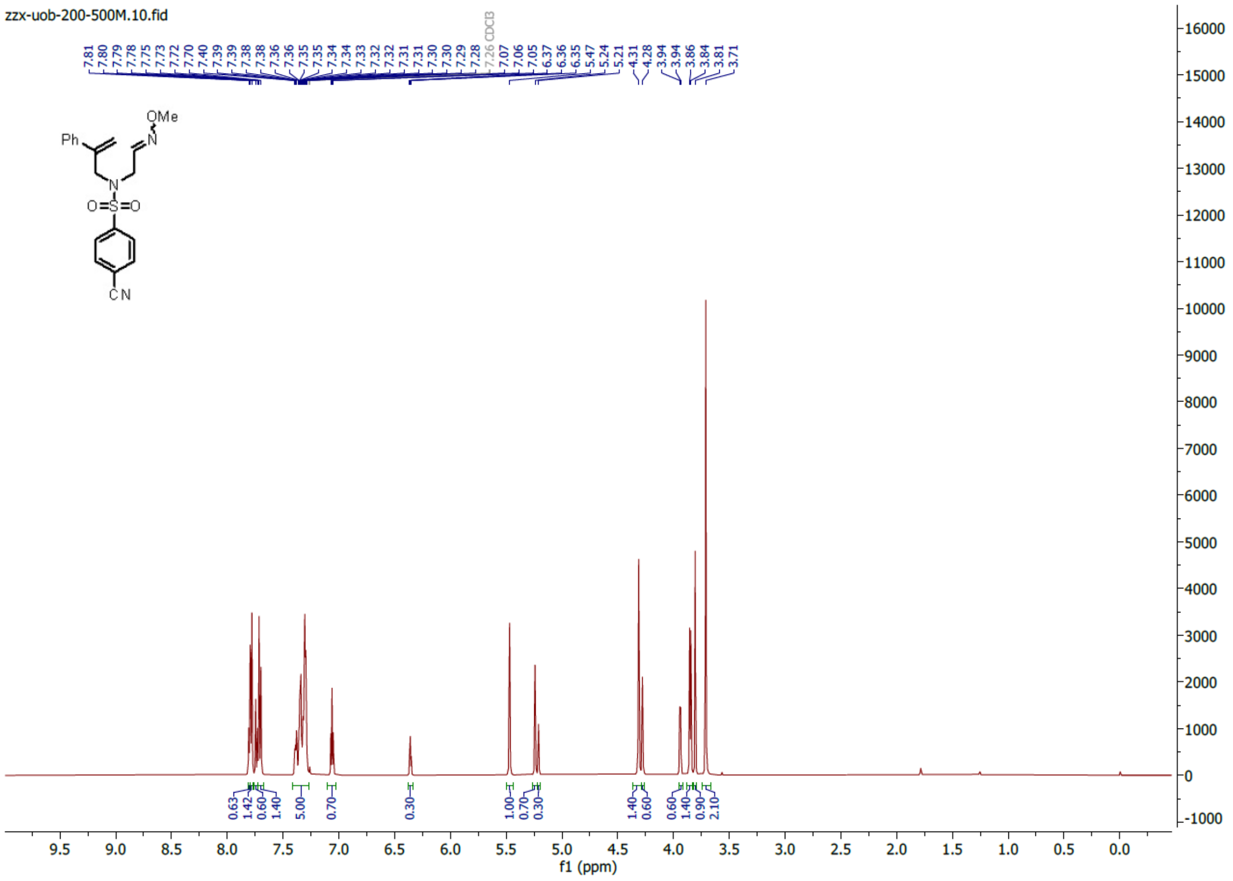

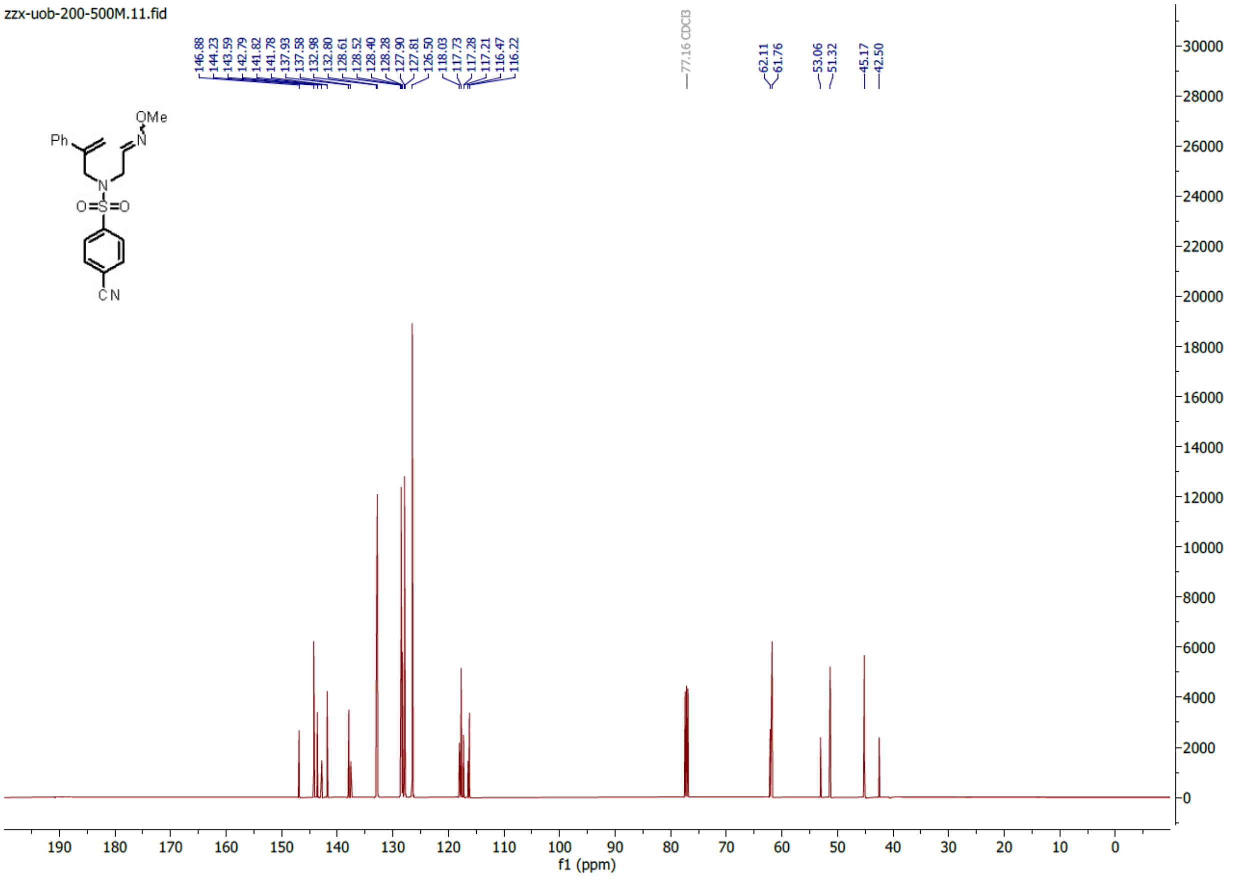

Compound S33b

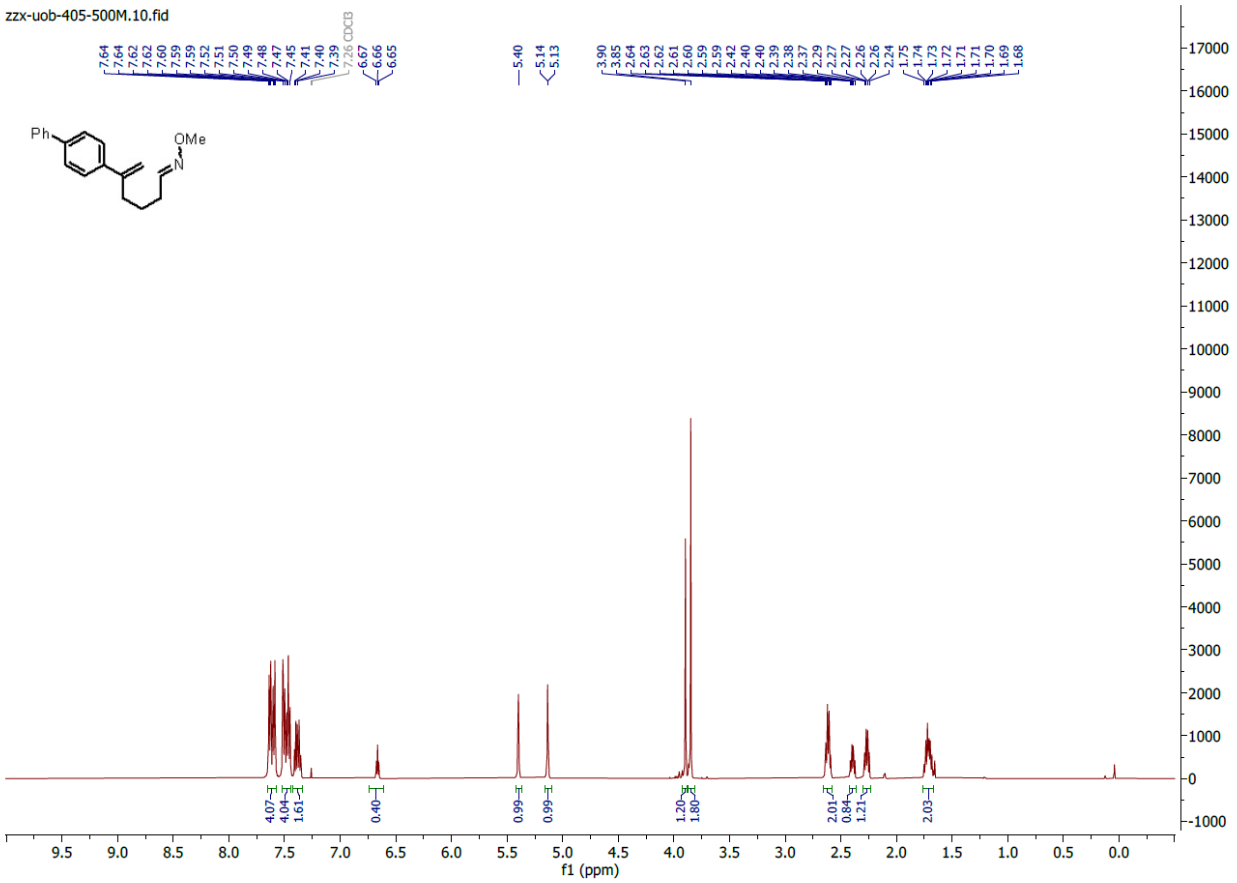

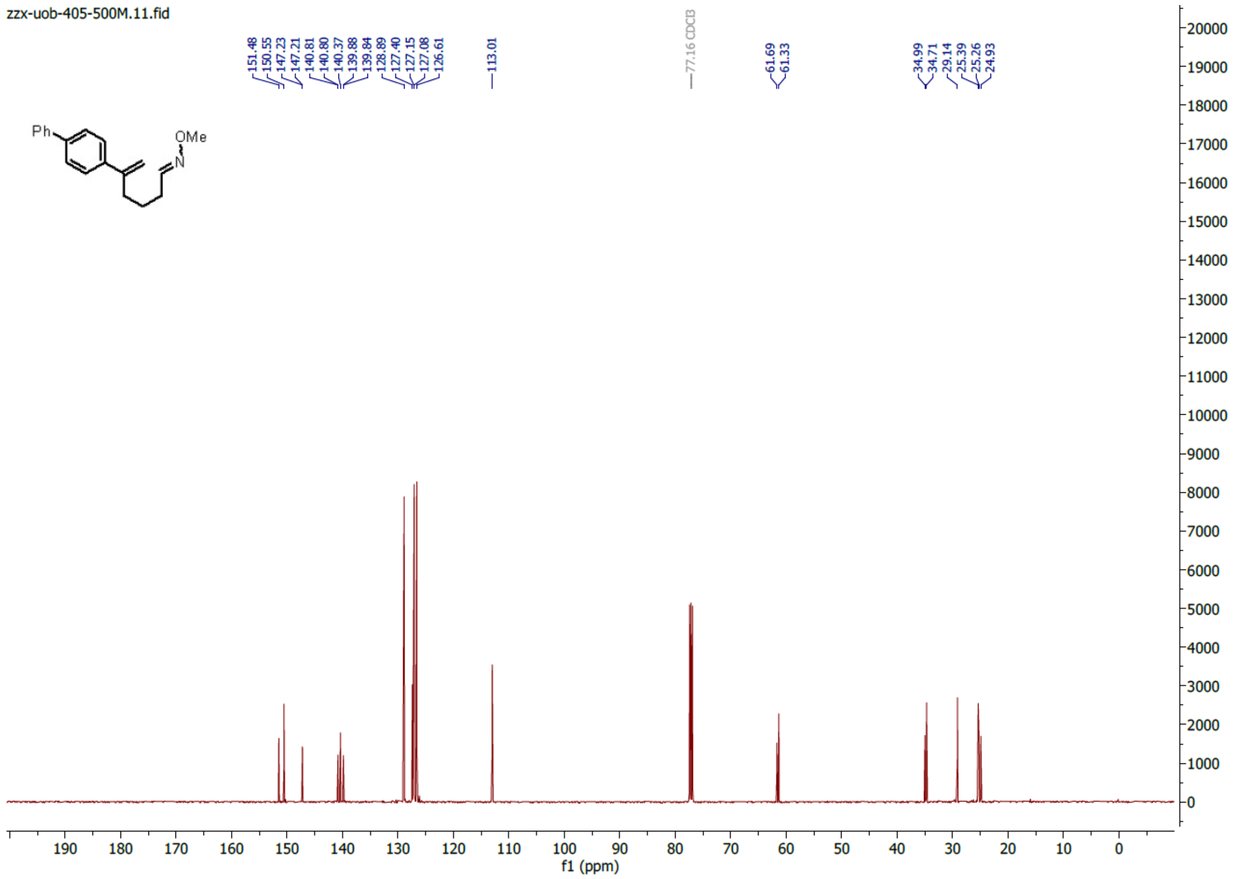

Compound S47b

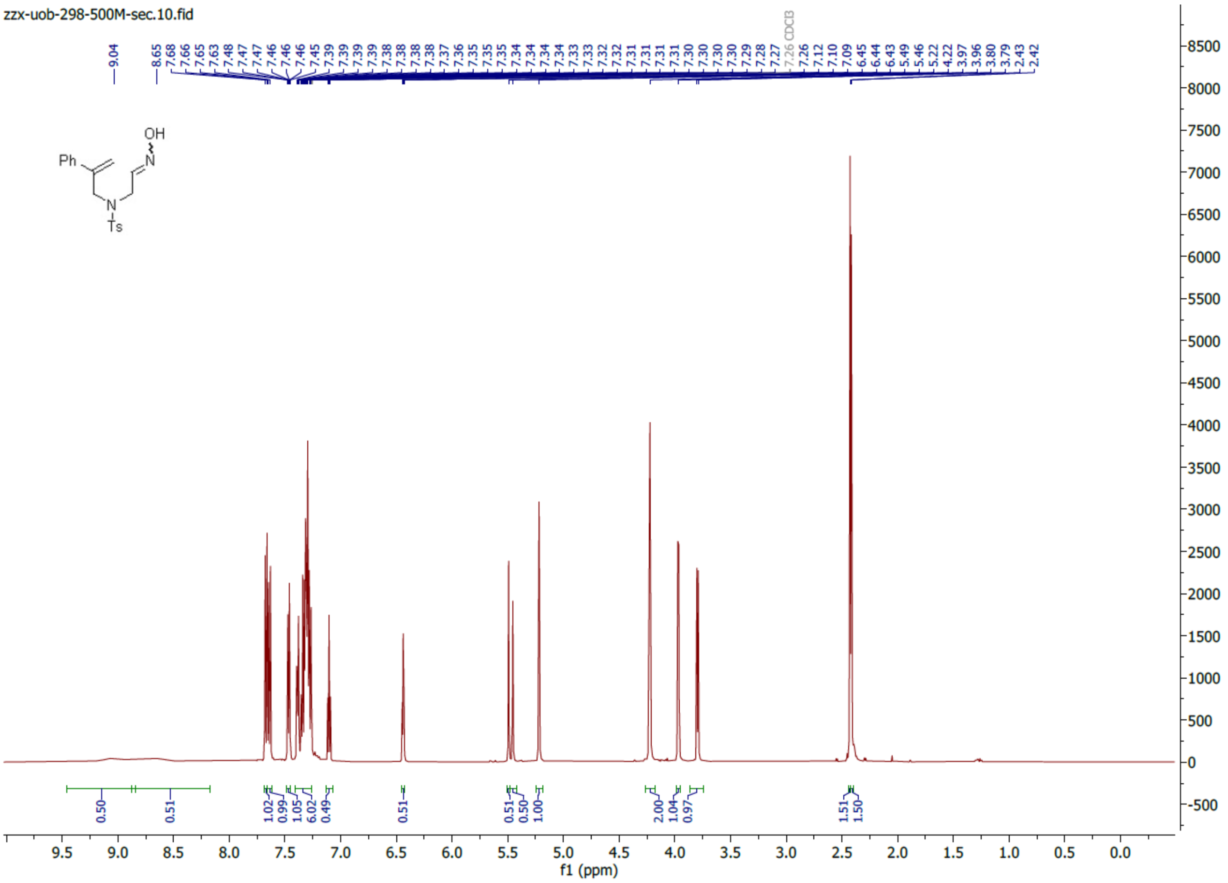

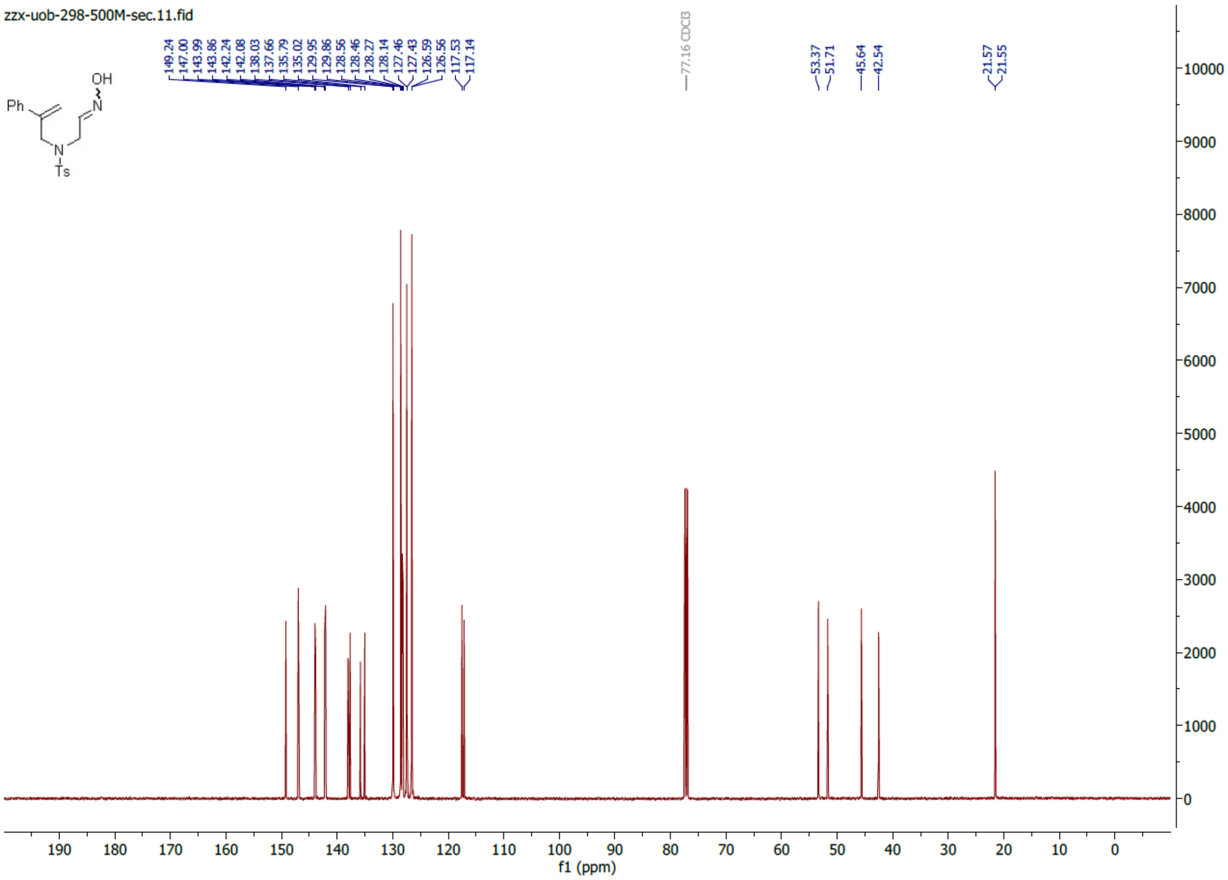

Compound S48b

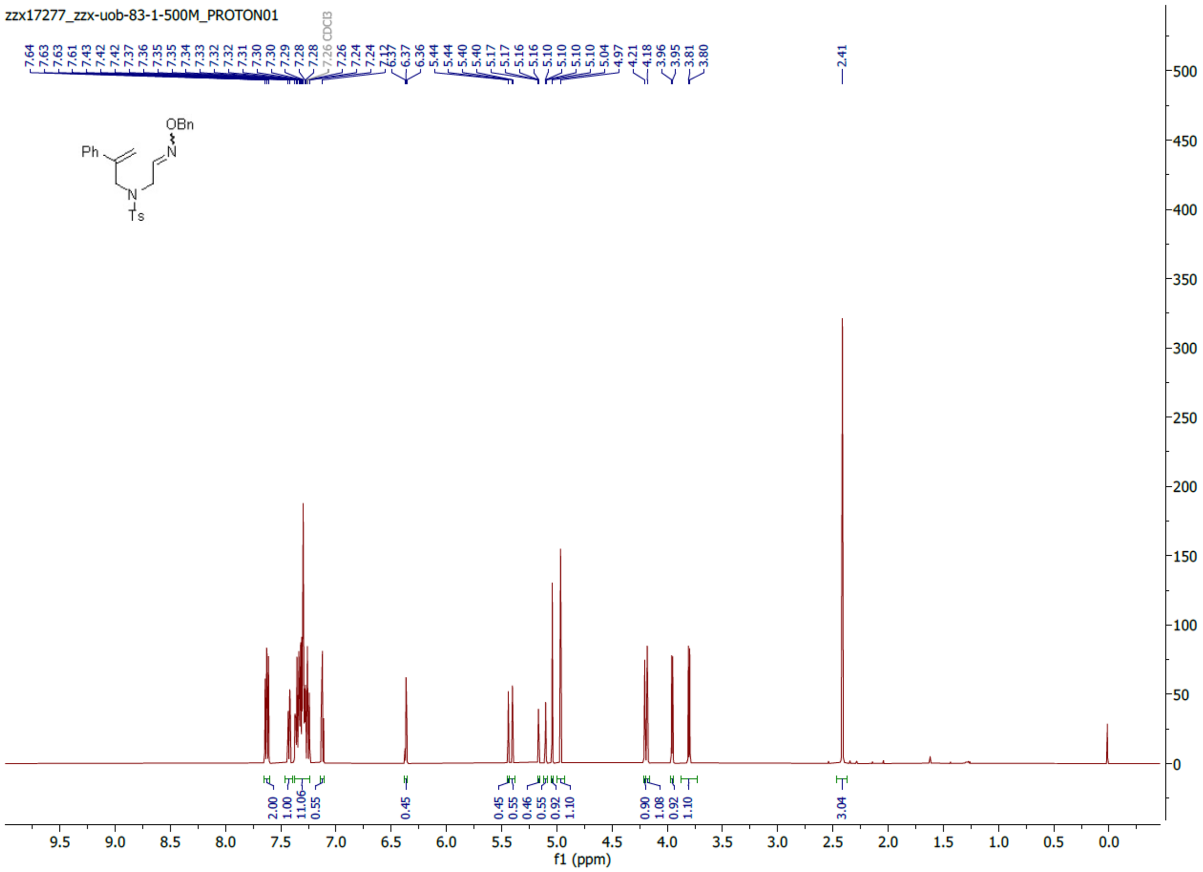

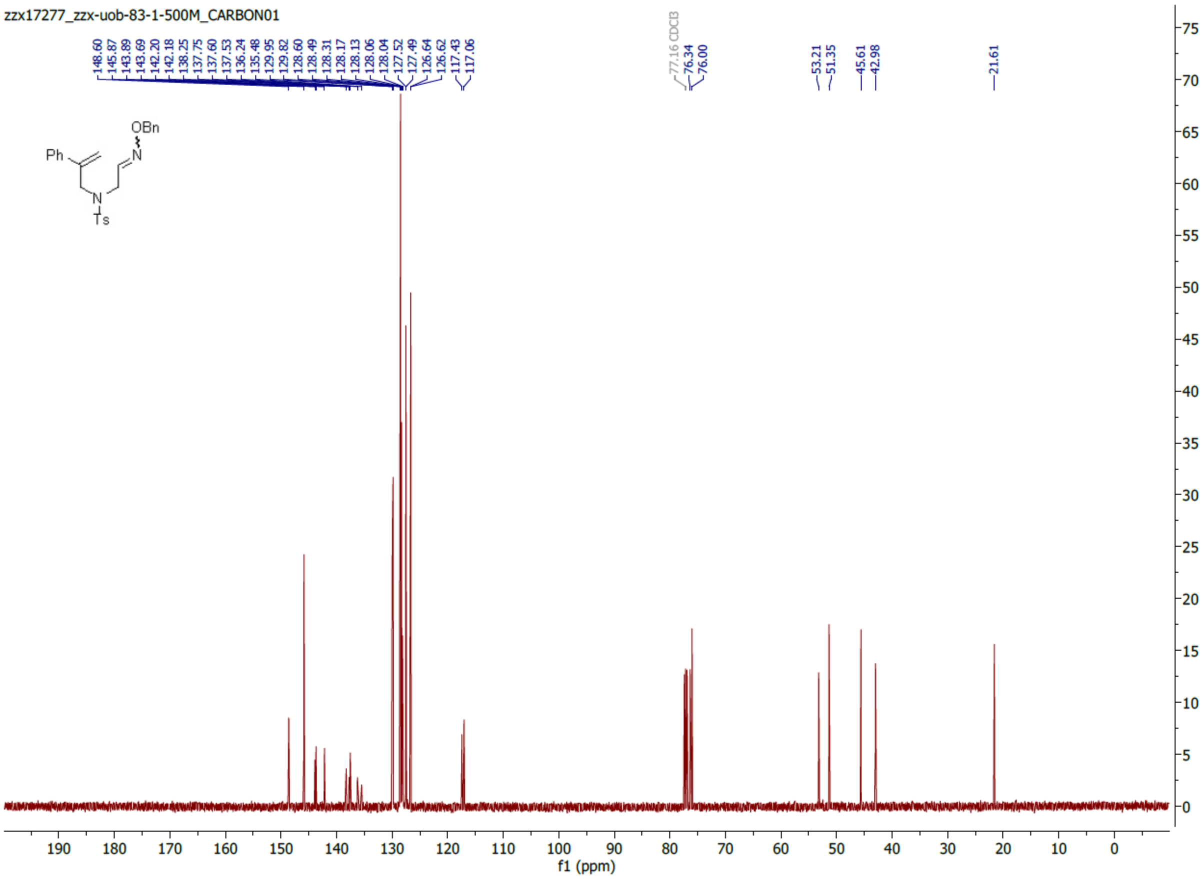

Compound S49b

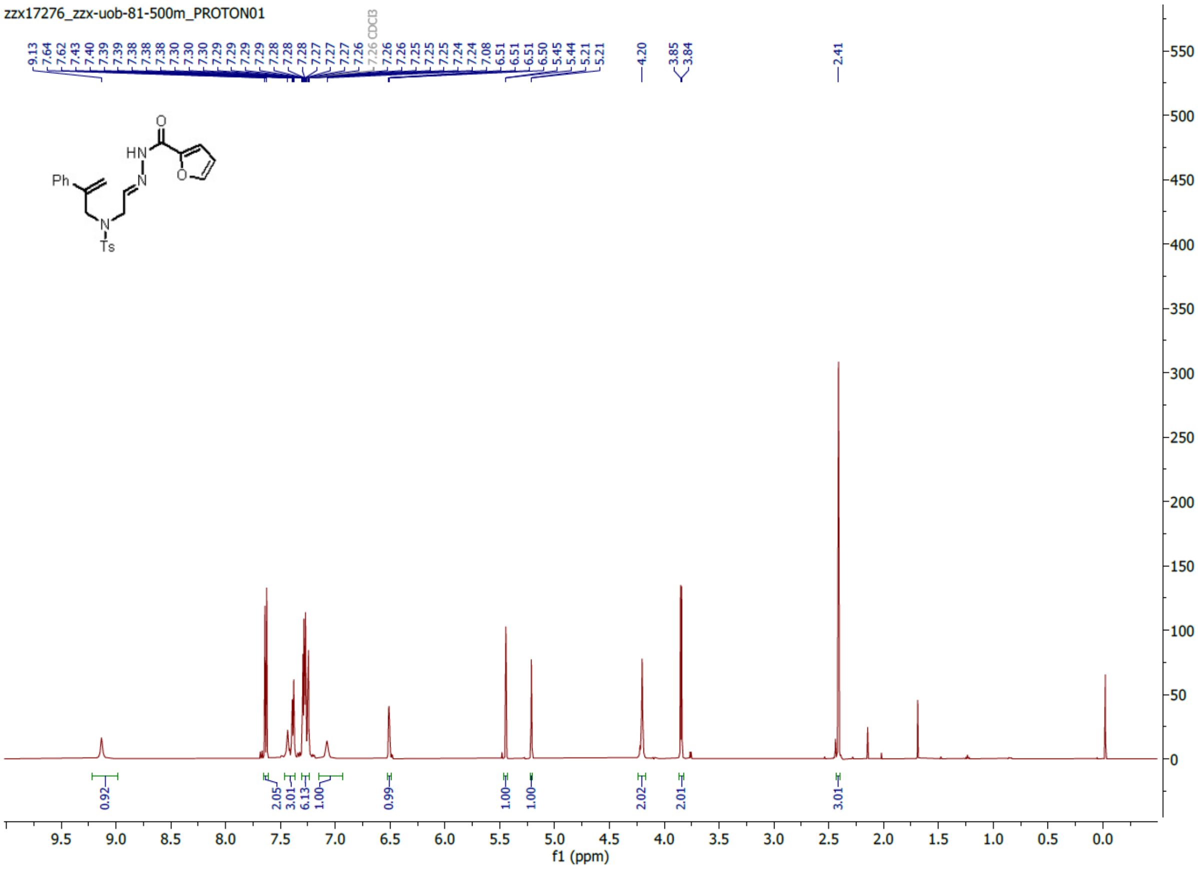

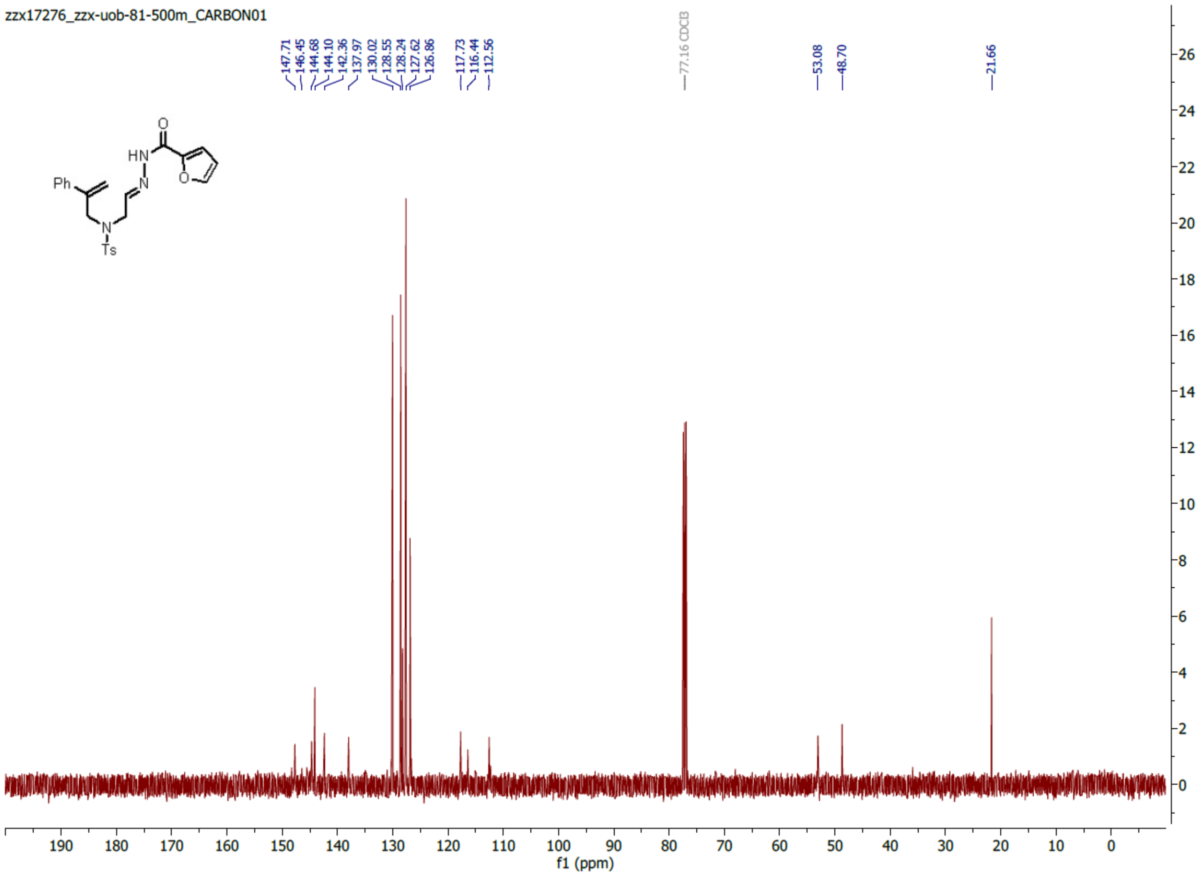

Compound S51b

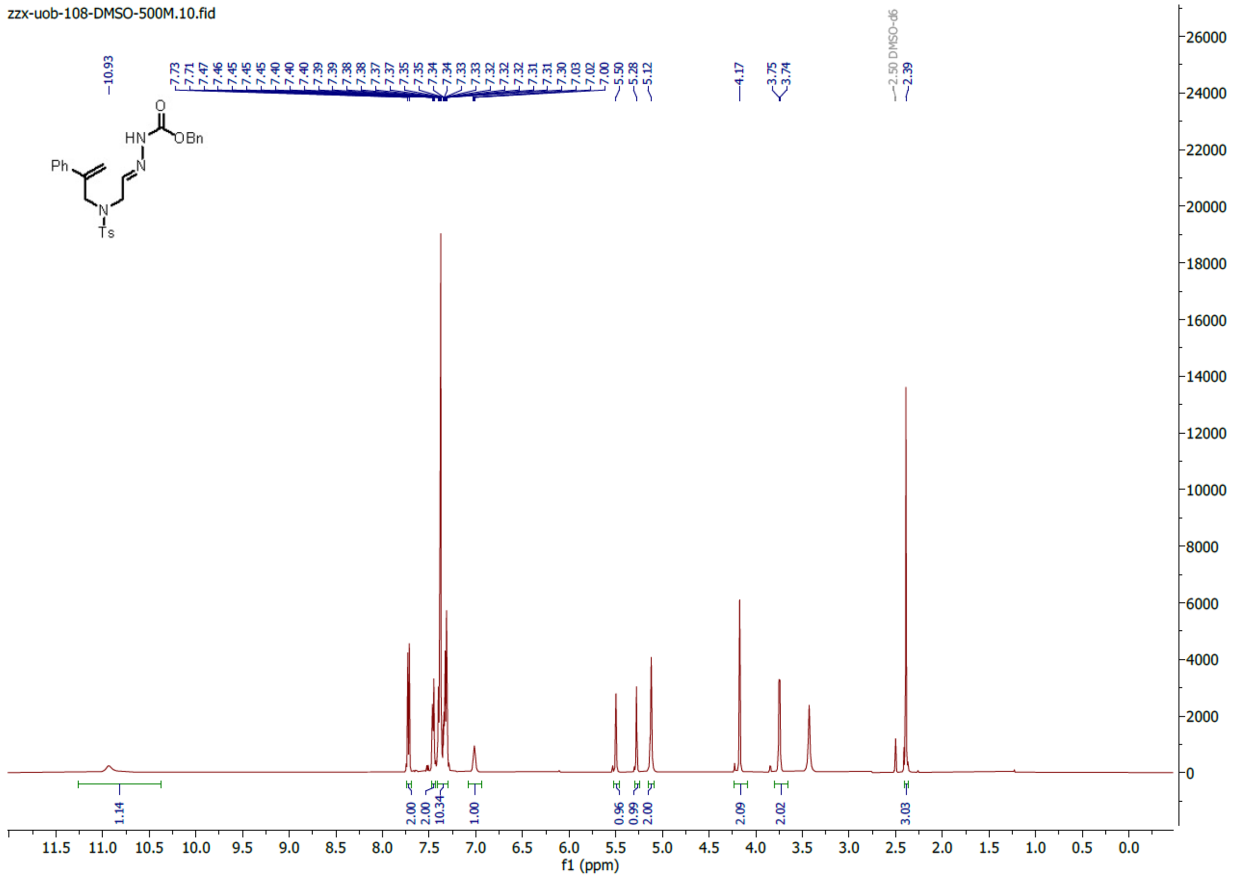

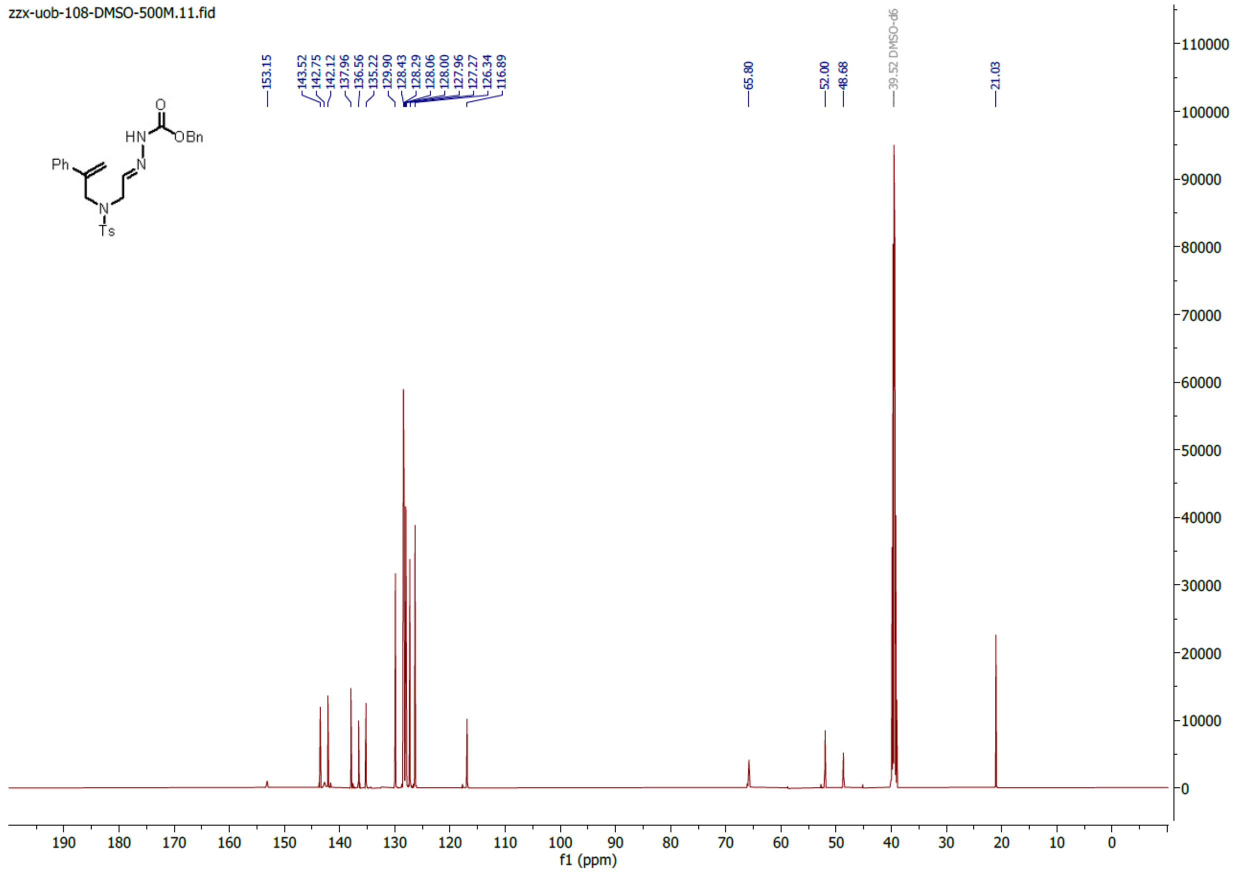

Compound S52b

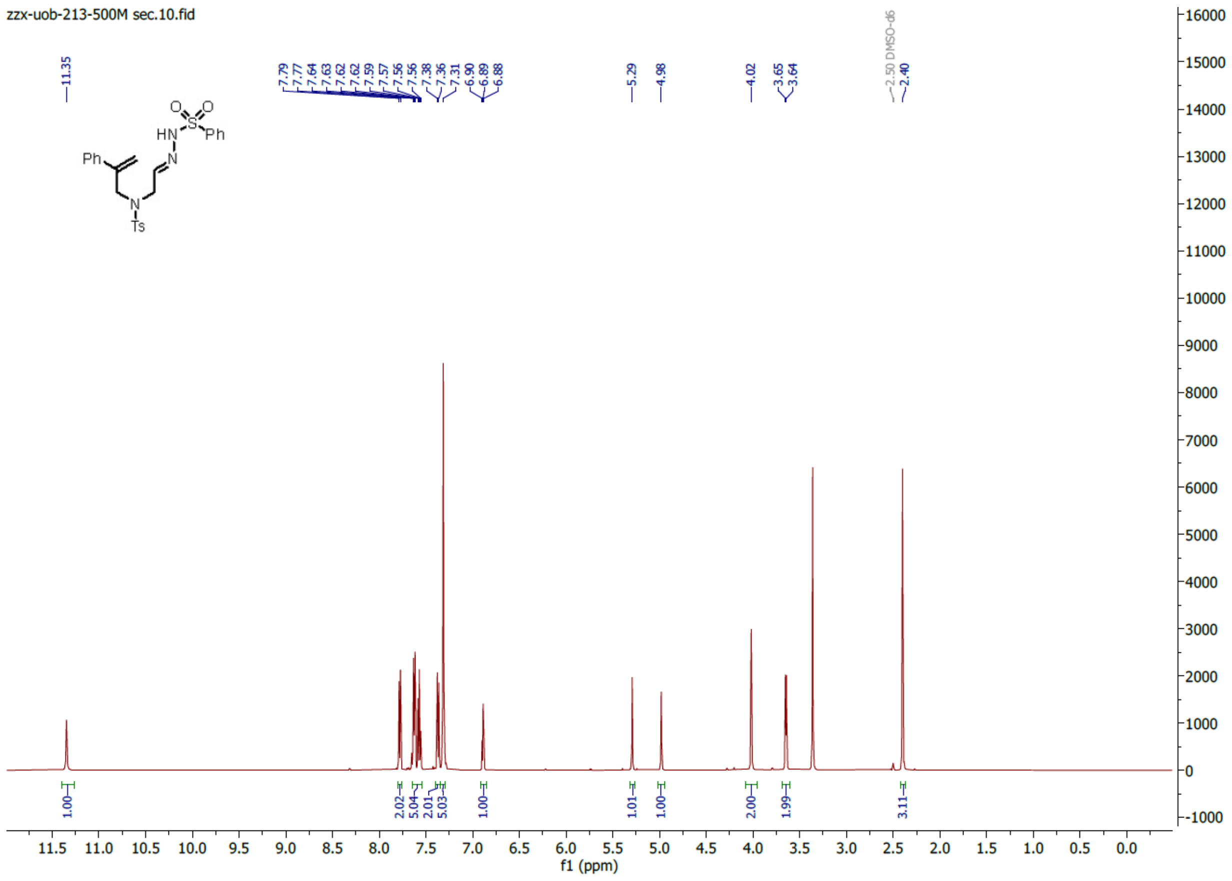

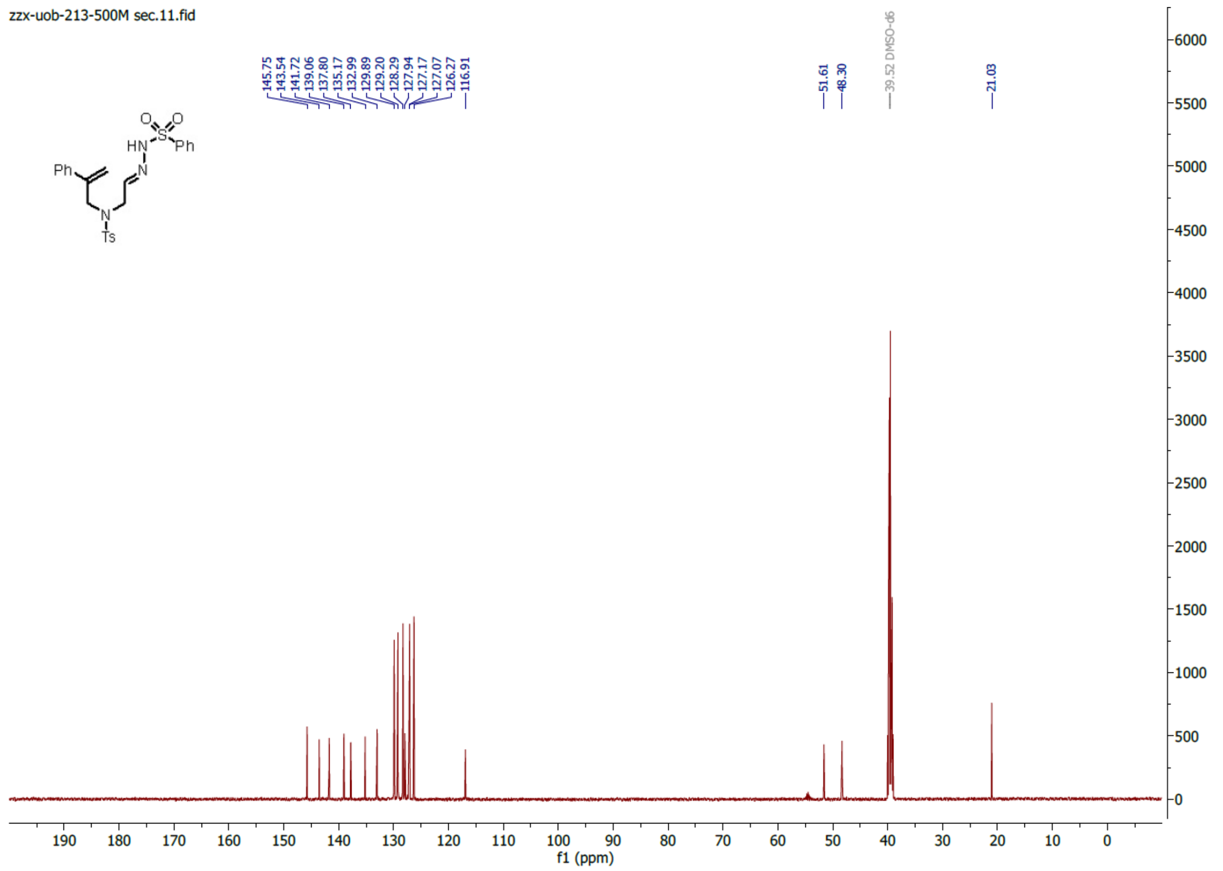

Compound S6a'

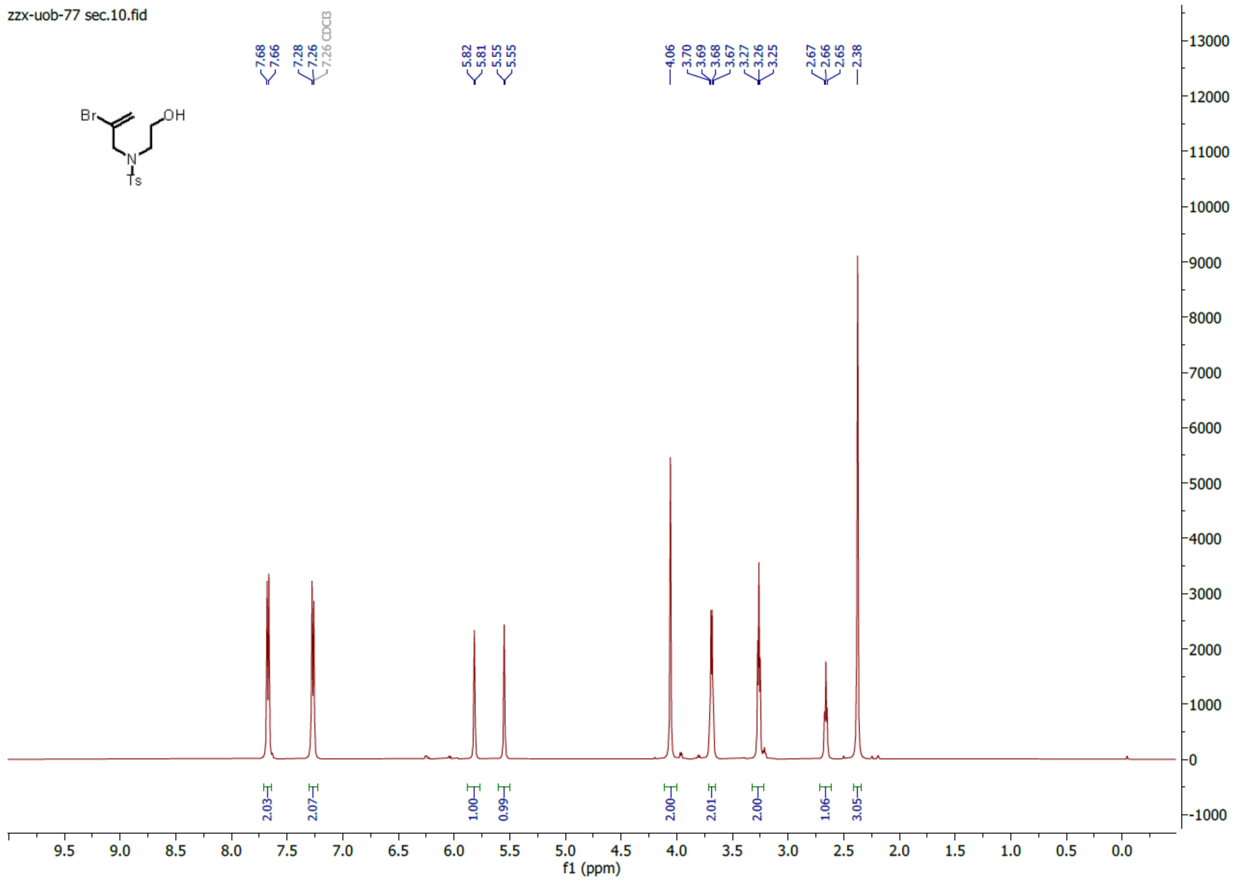

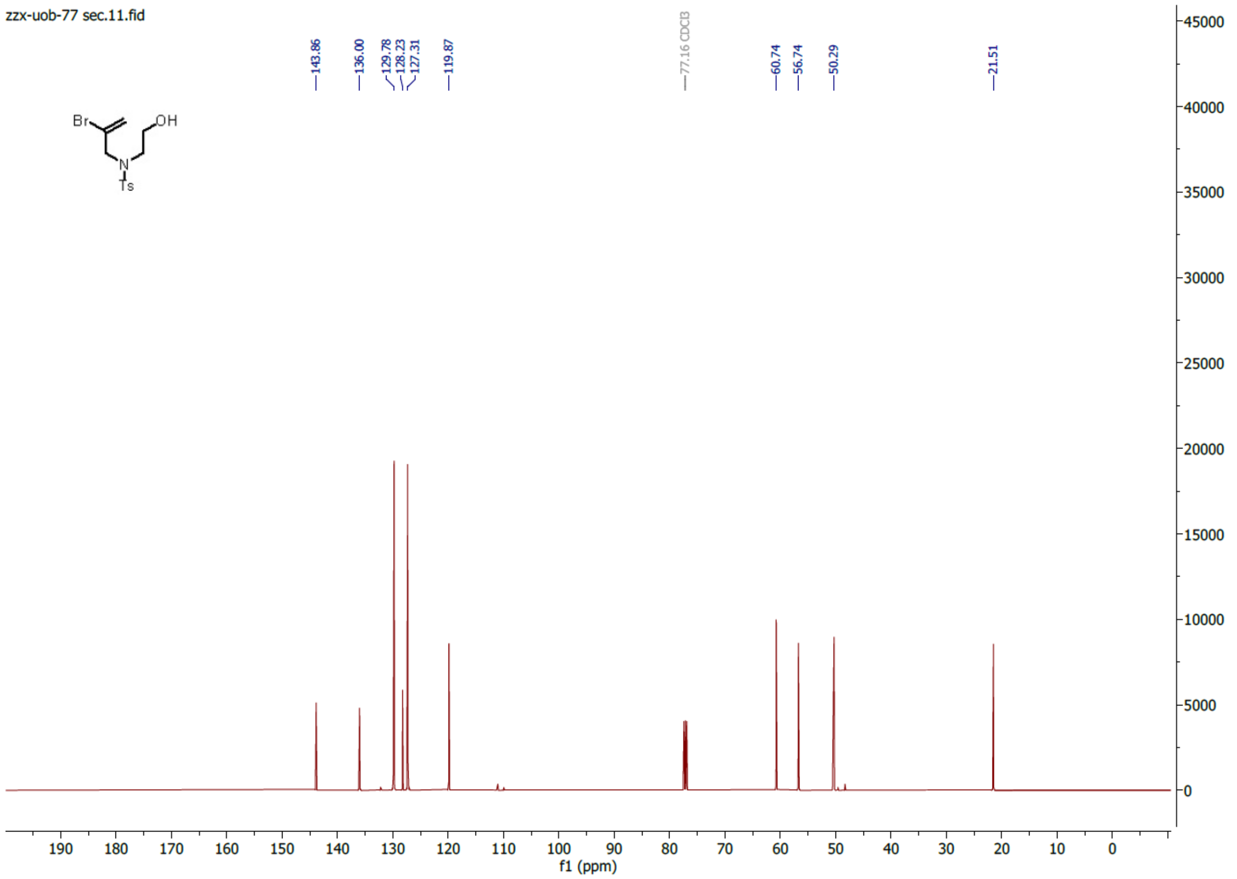

Compound S6a

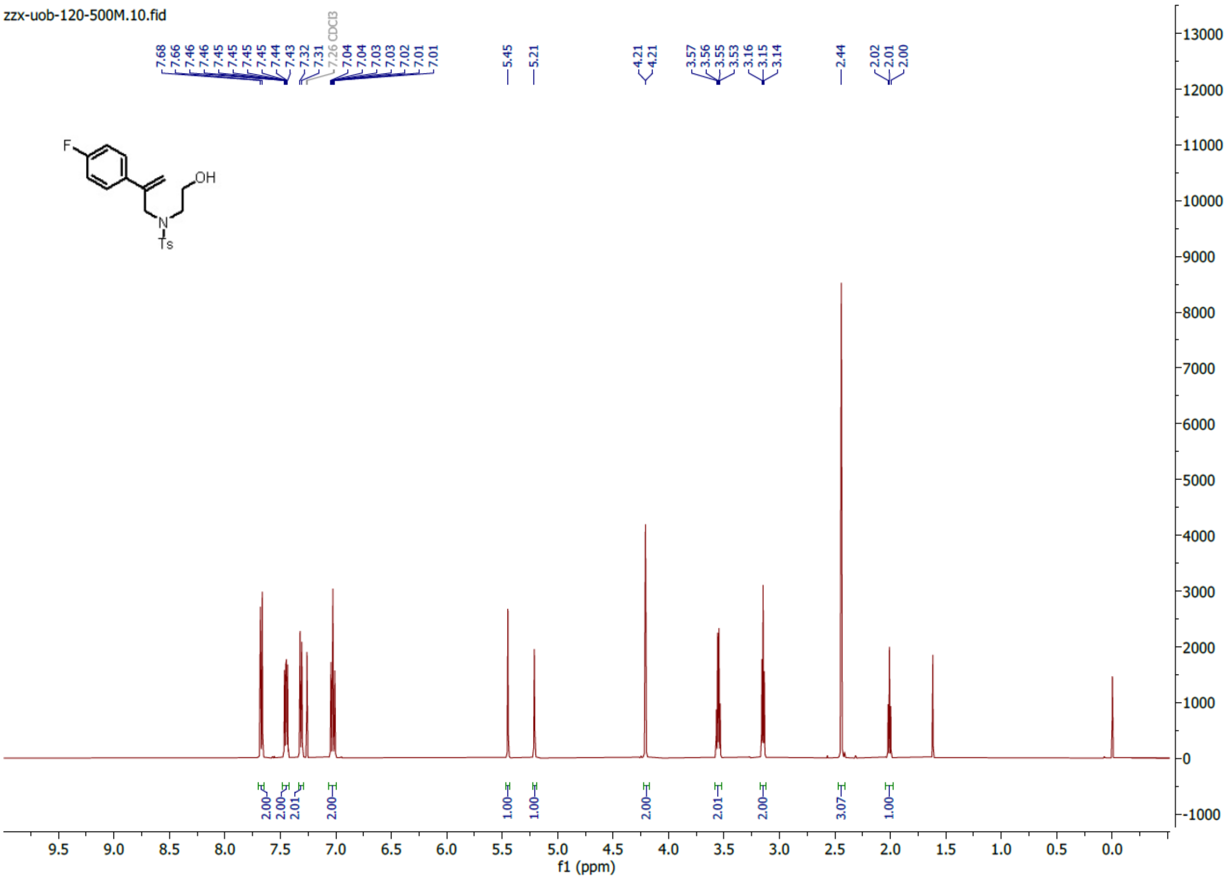

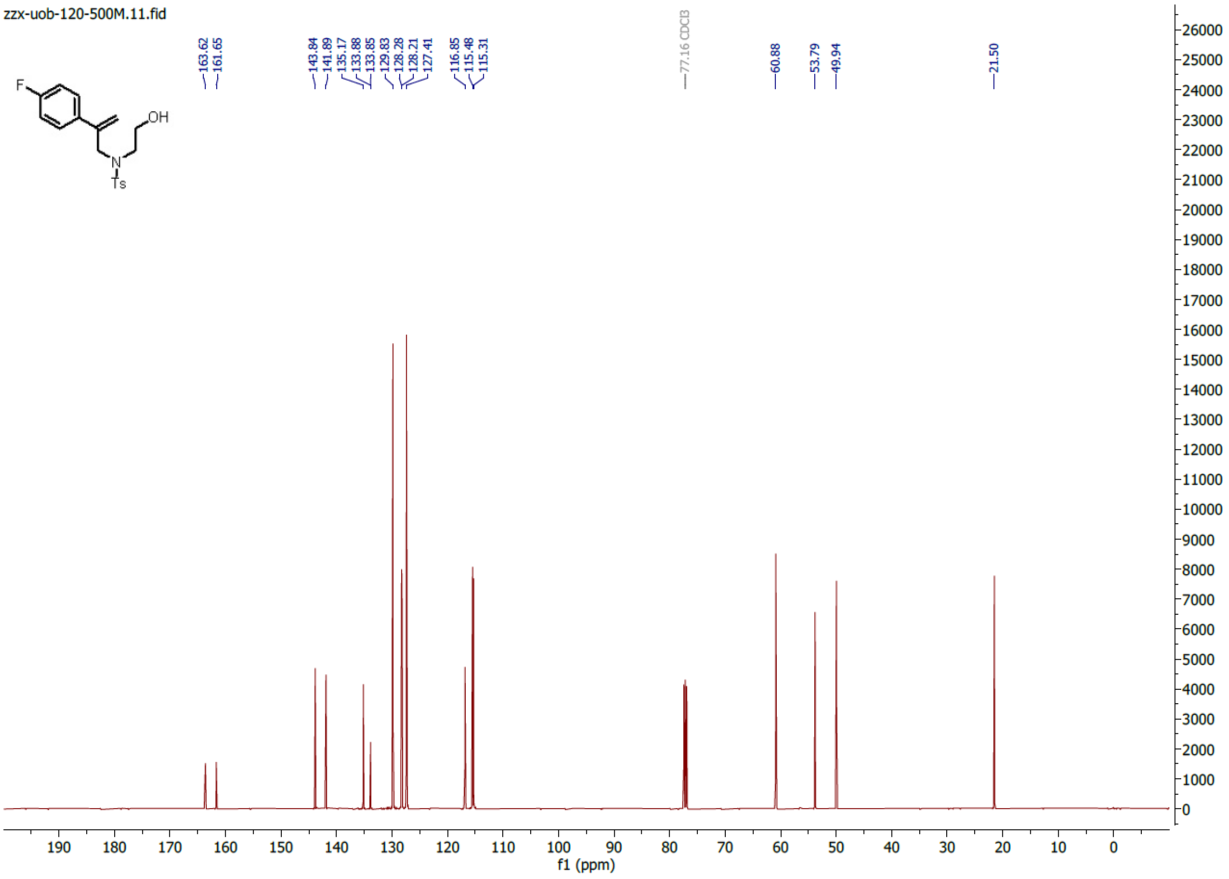

zzx-uob-120-F-500M.11.fid

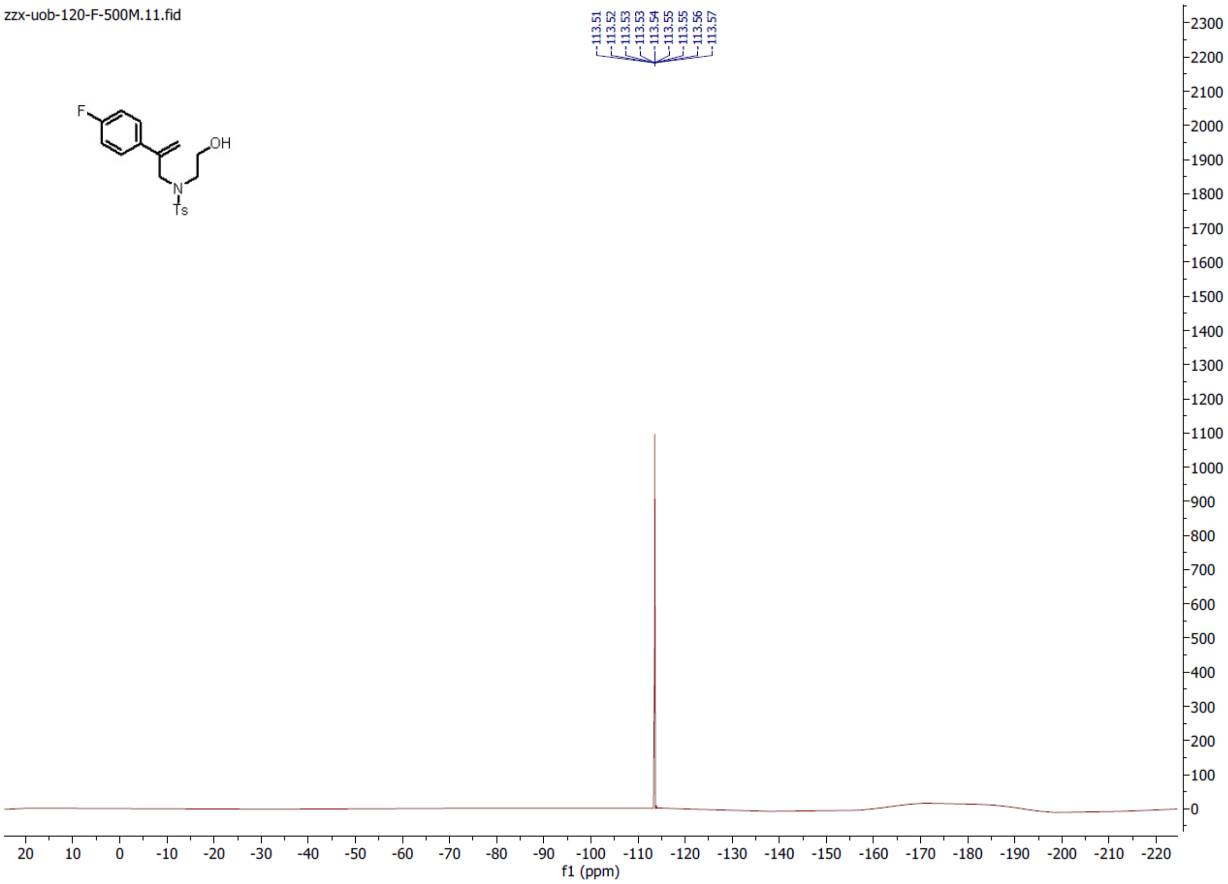

Compound S6b

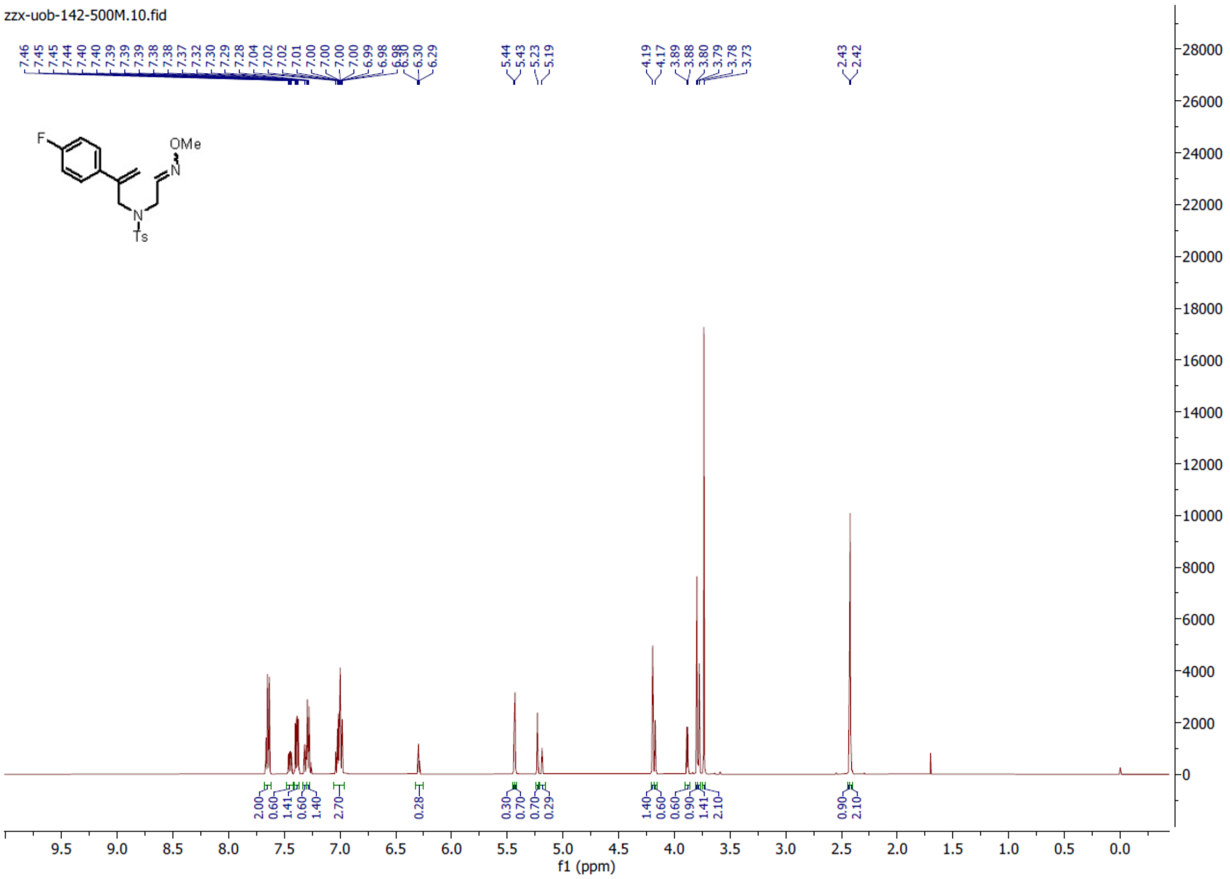

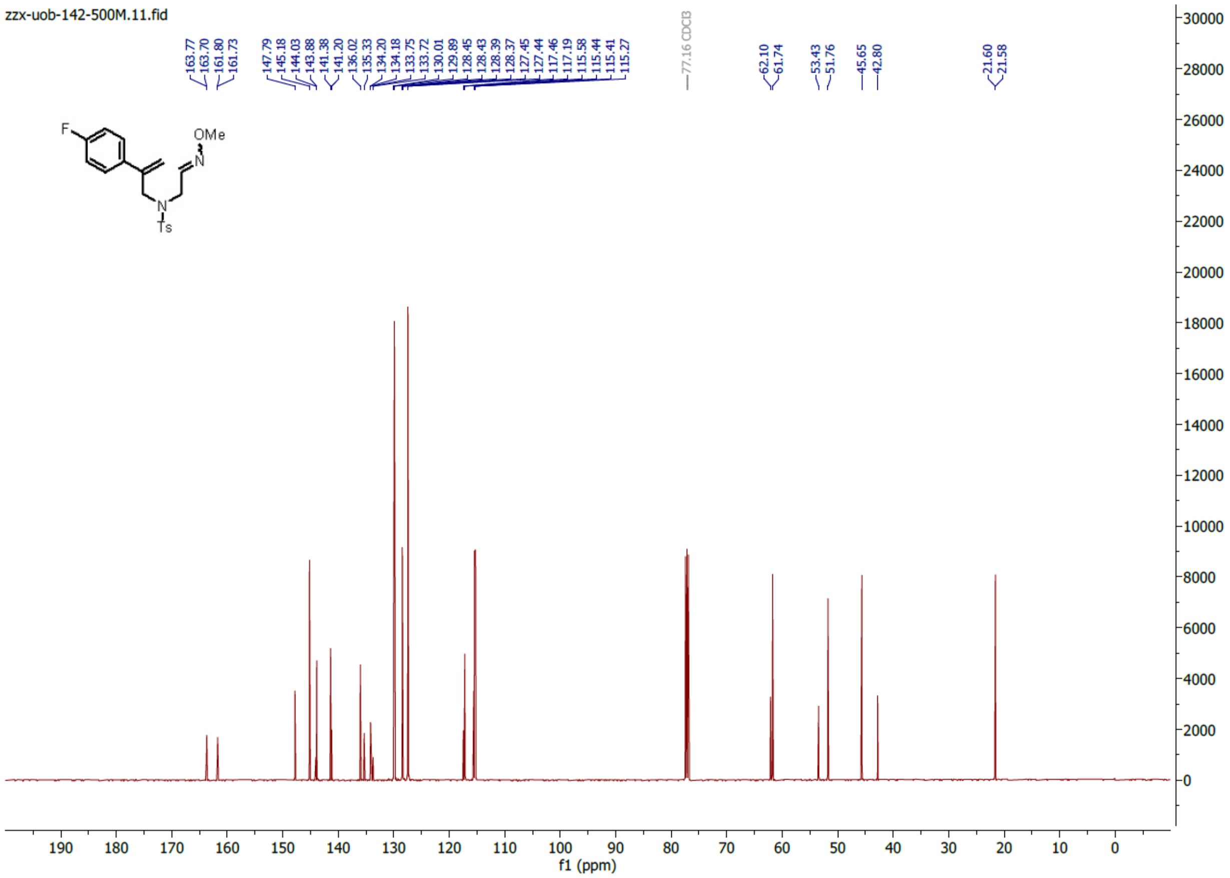

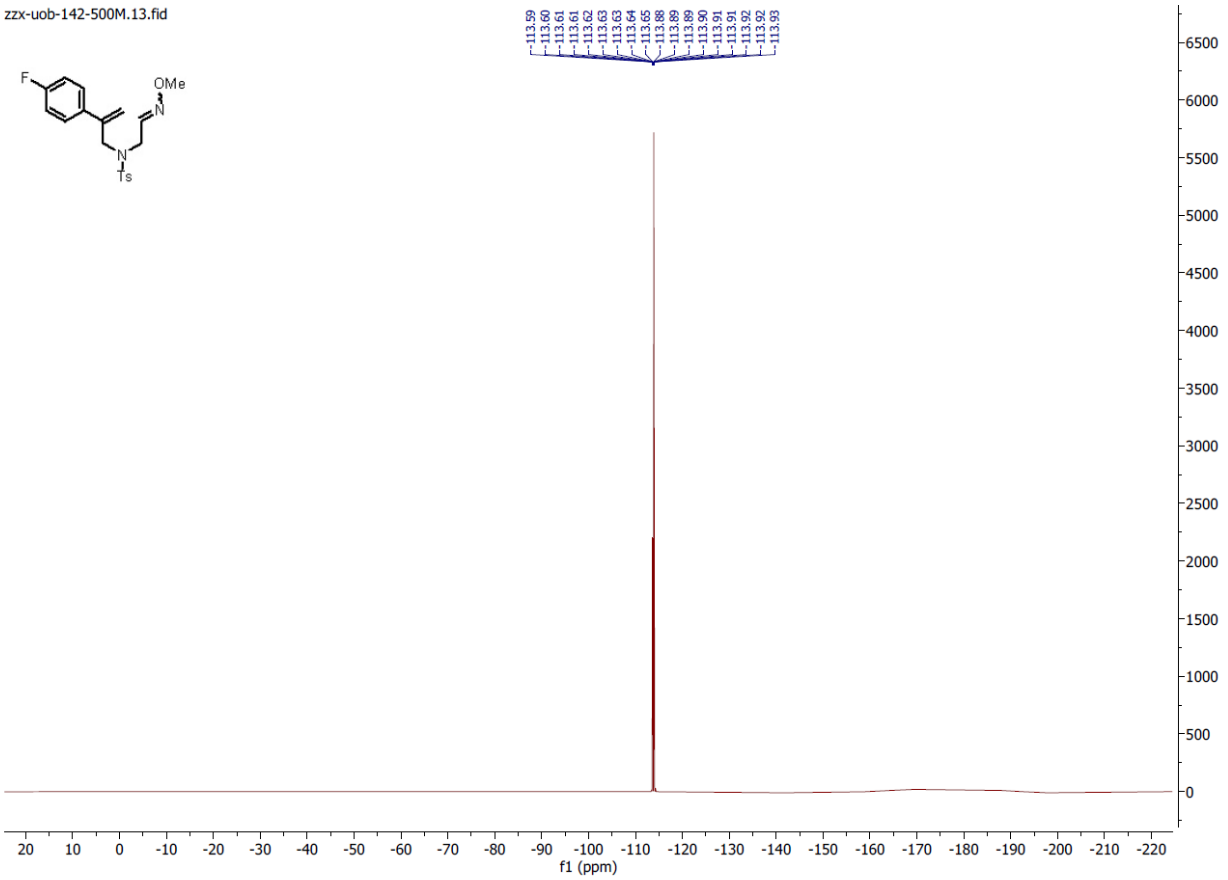
zzx-uob-142-500M.13.fid
F
OMe
N
N
Ts
-113.59
-113.60
-113.61
-113.61
-113.62
-113.63
-113.63
-113.64
-113.65
-113.88
-113.89
-113.89
-113.90
-113.91
-113.91
-113.92
-113.92
-113.93
f1 (ppm)

Compound S7a

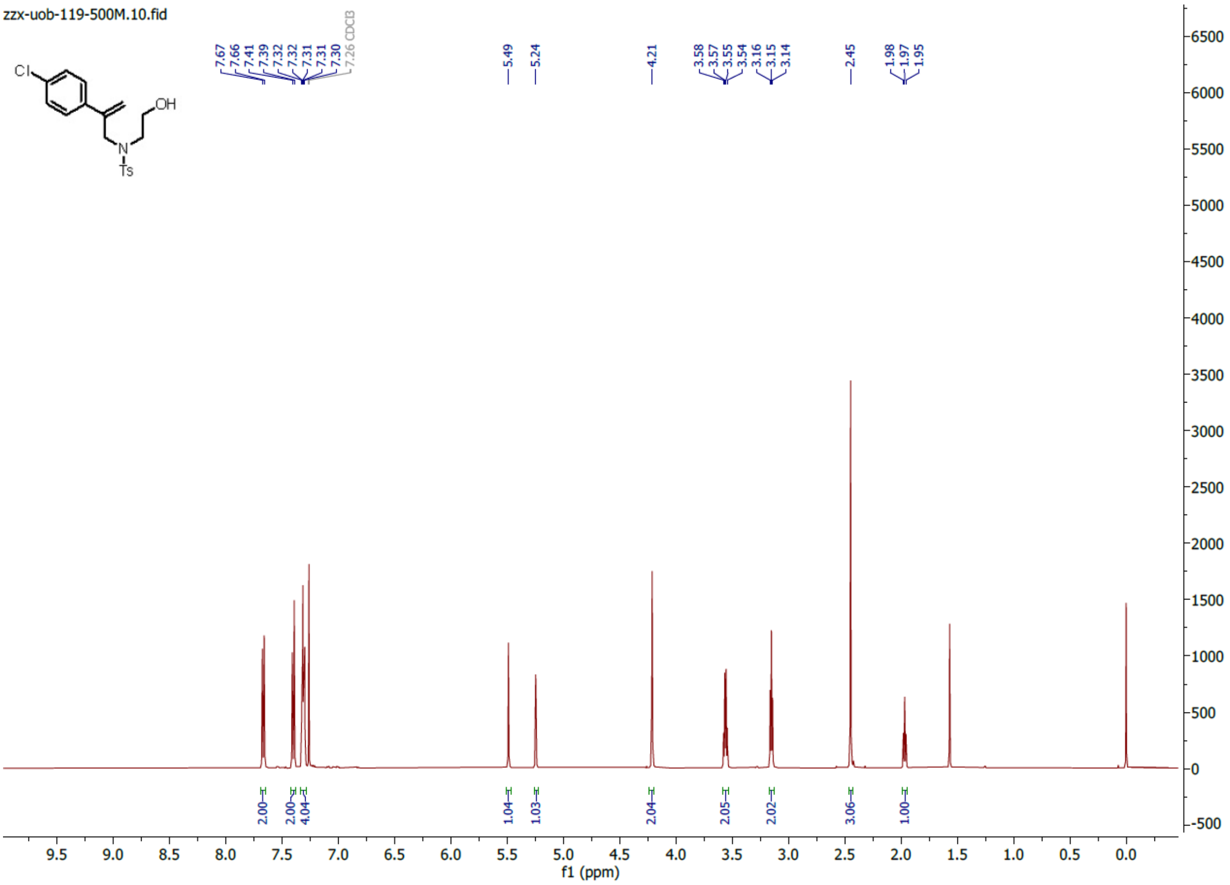

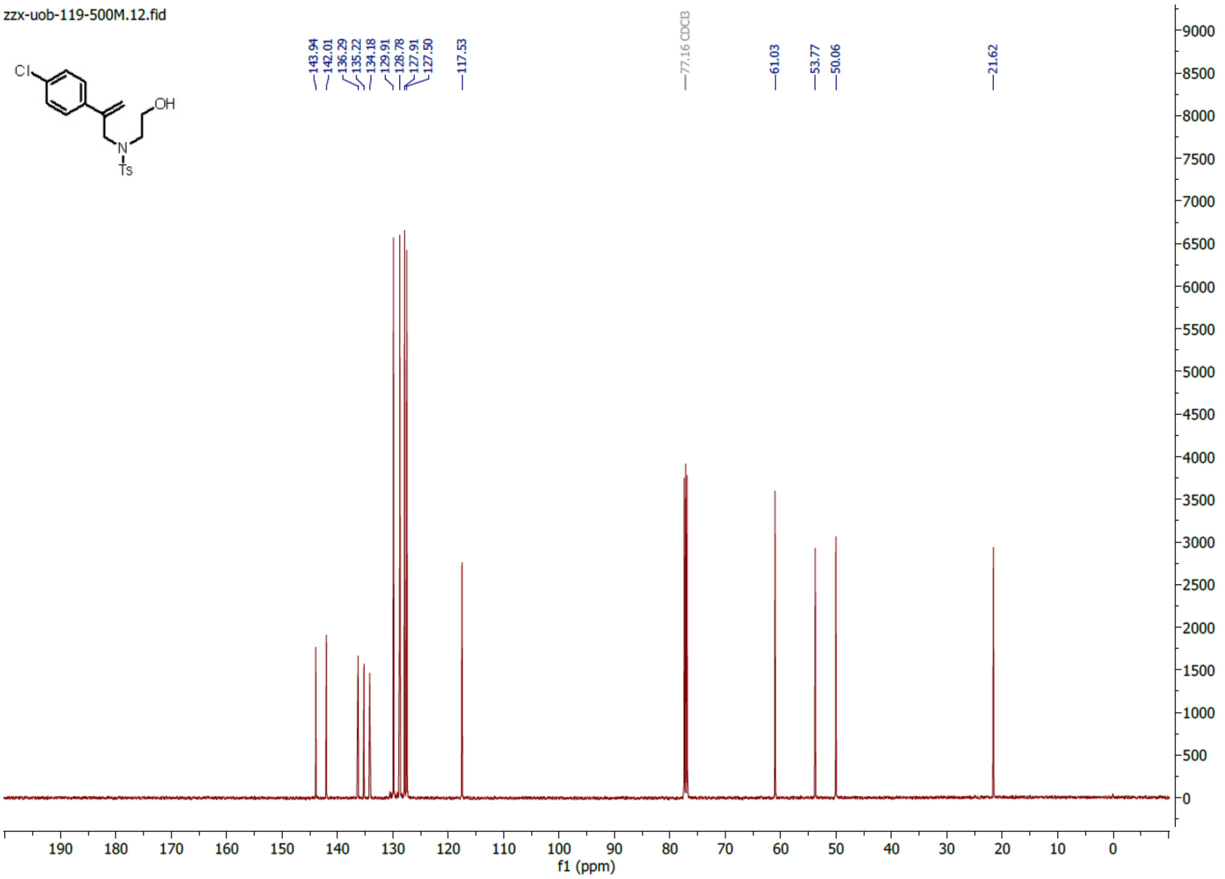

Compound S7b

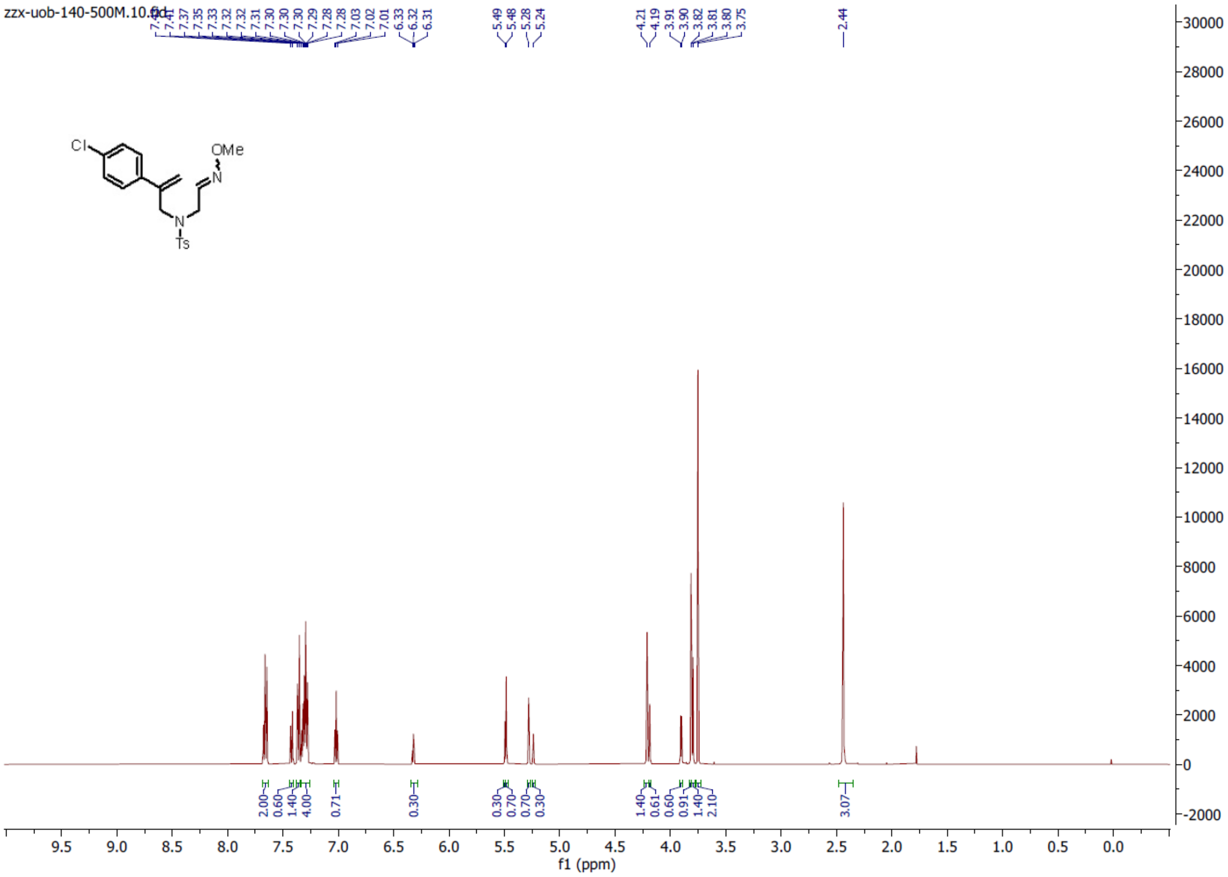

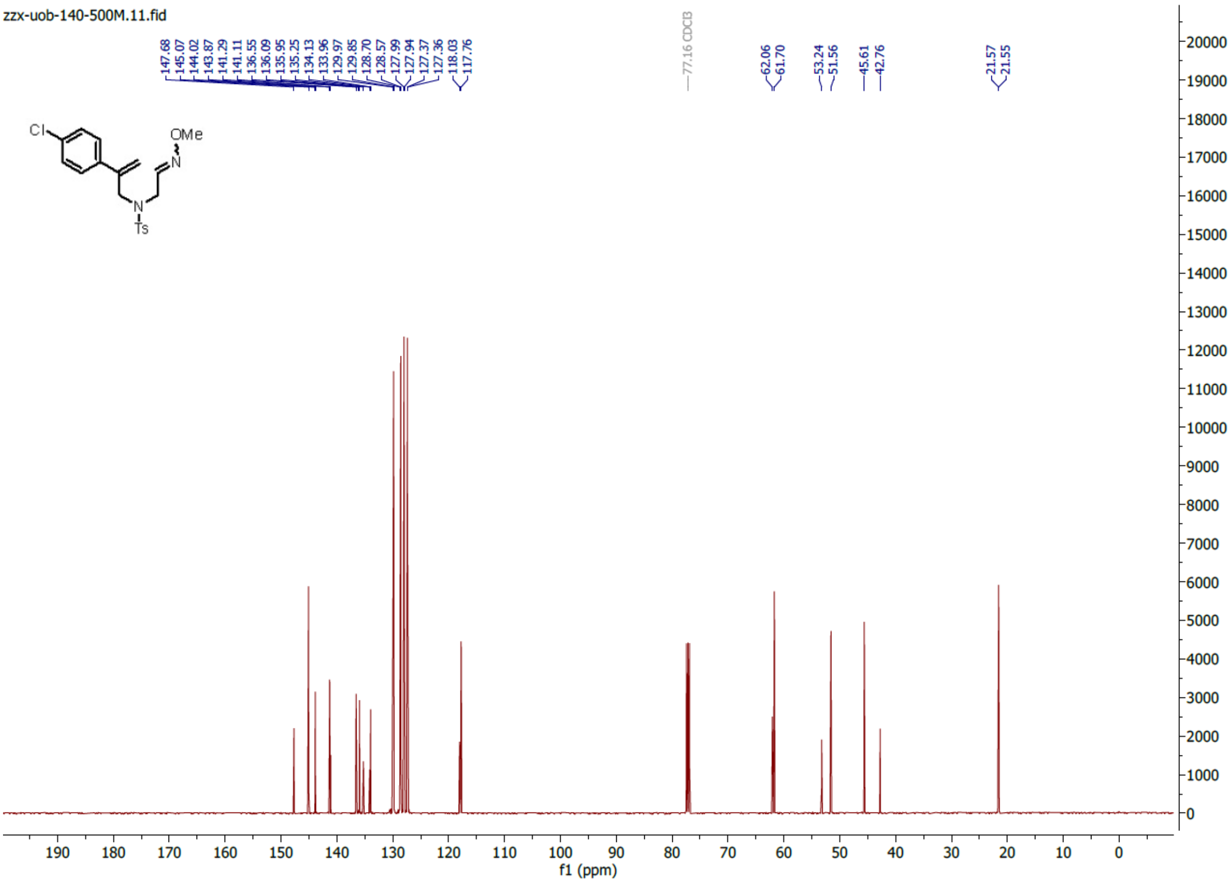

Compound S8a

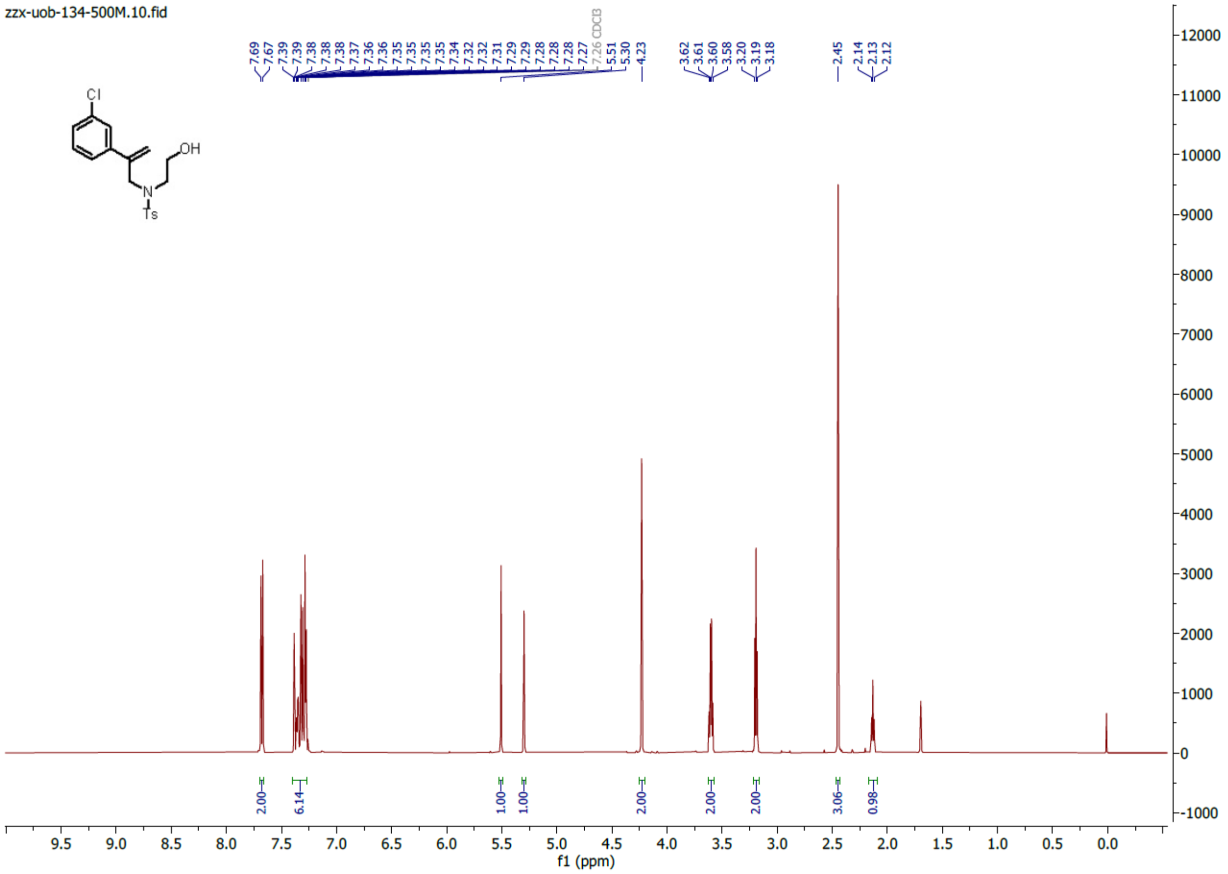

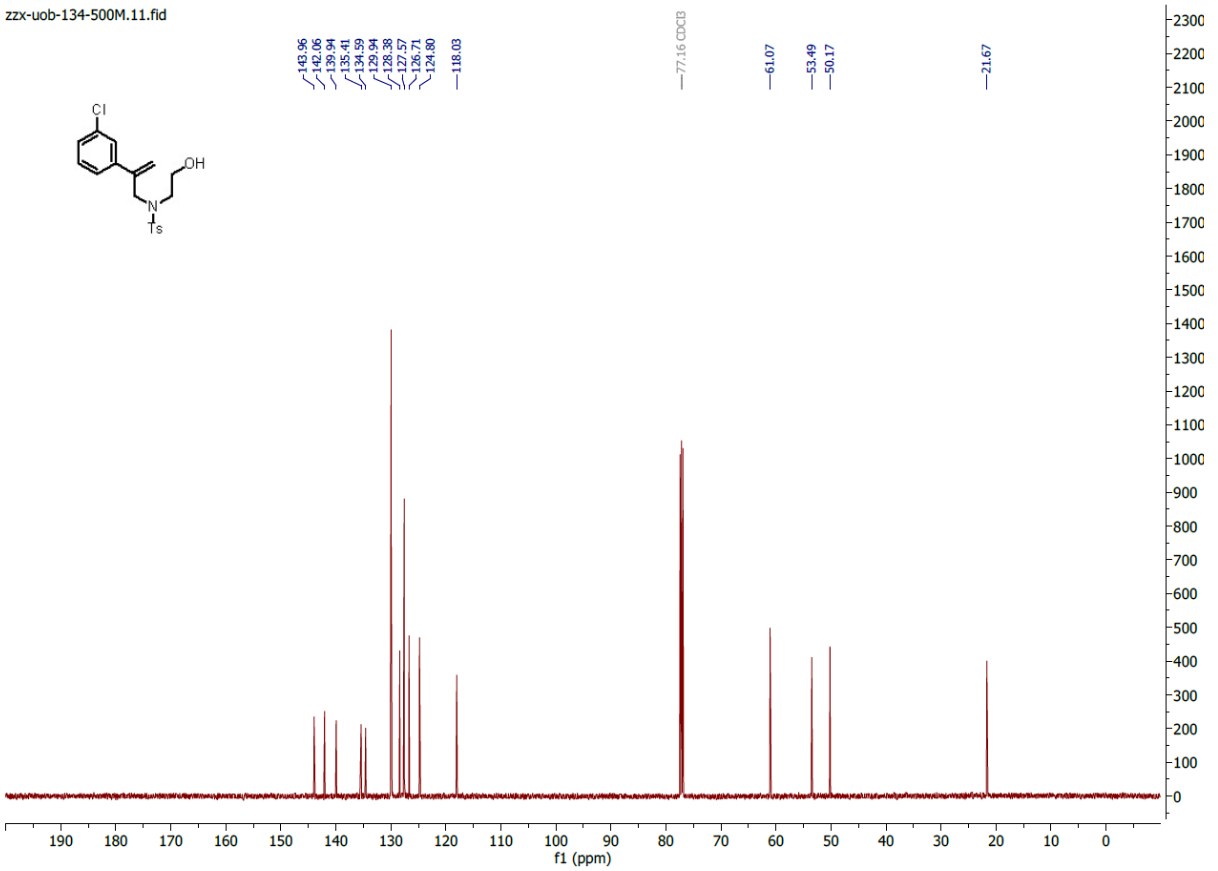

Compound S8b

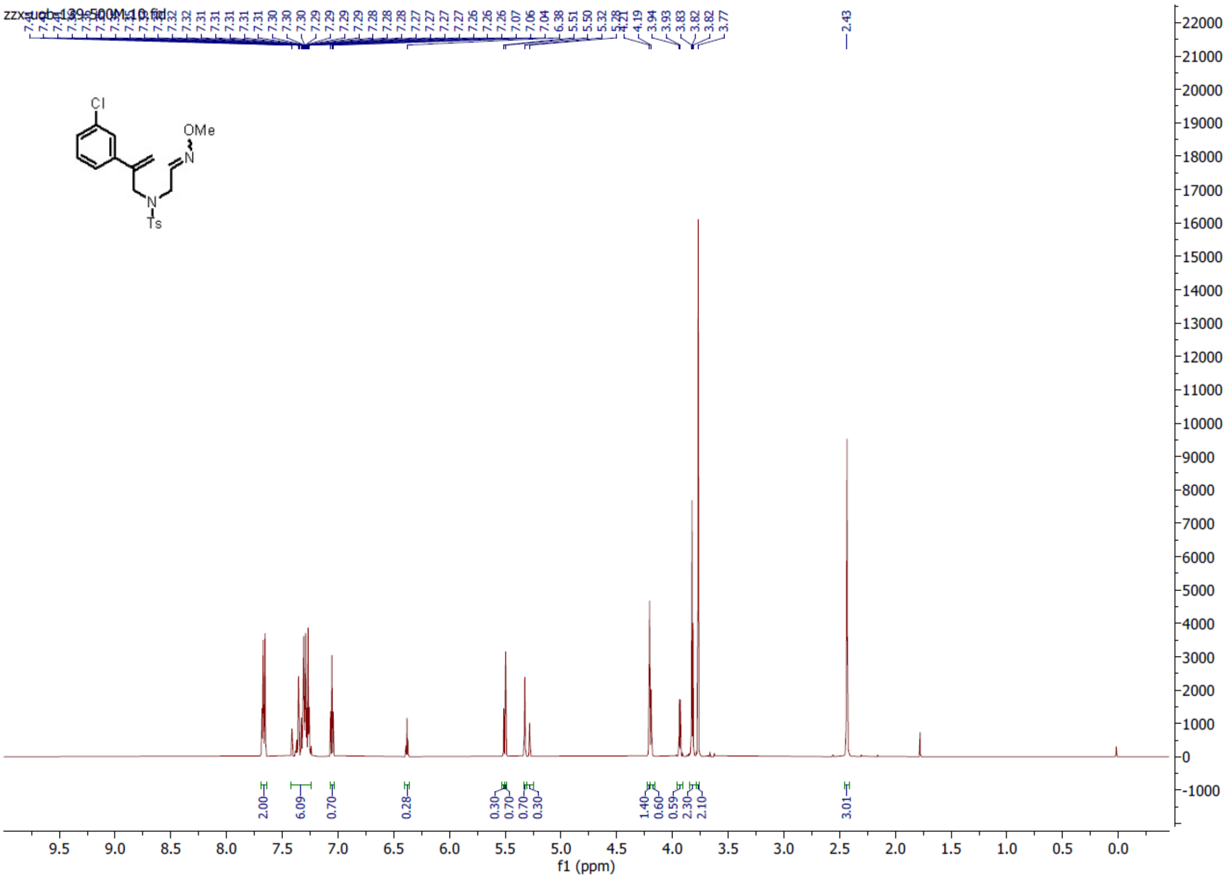

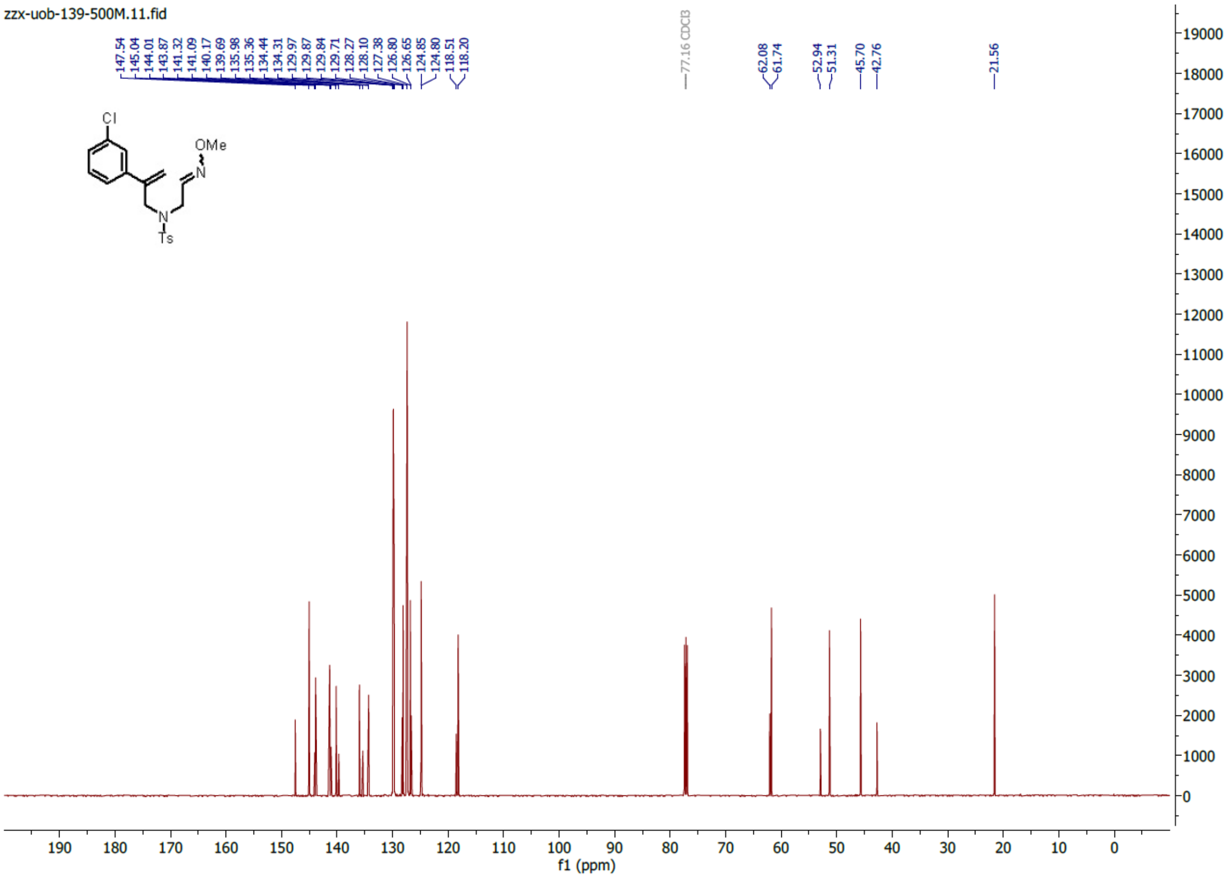

Compound S9a

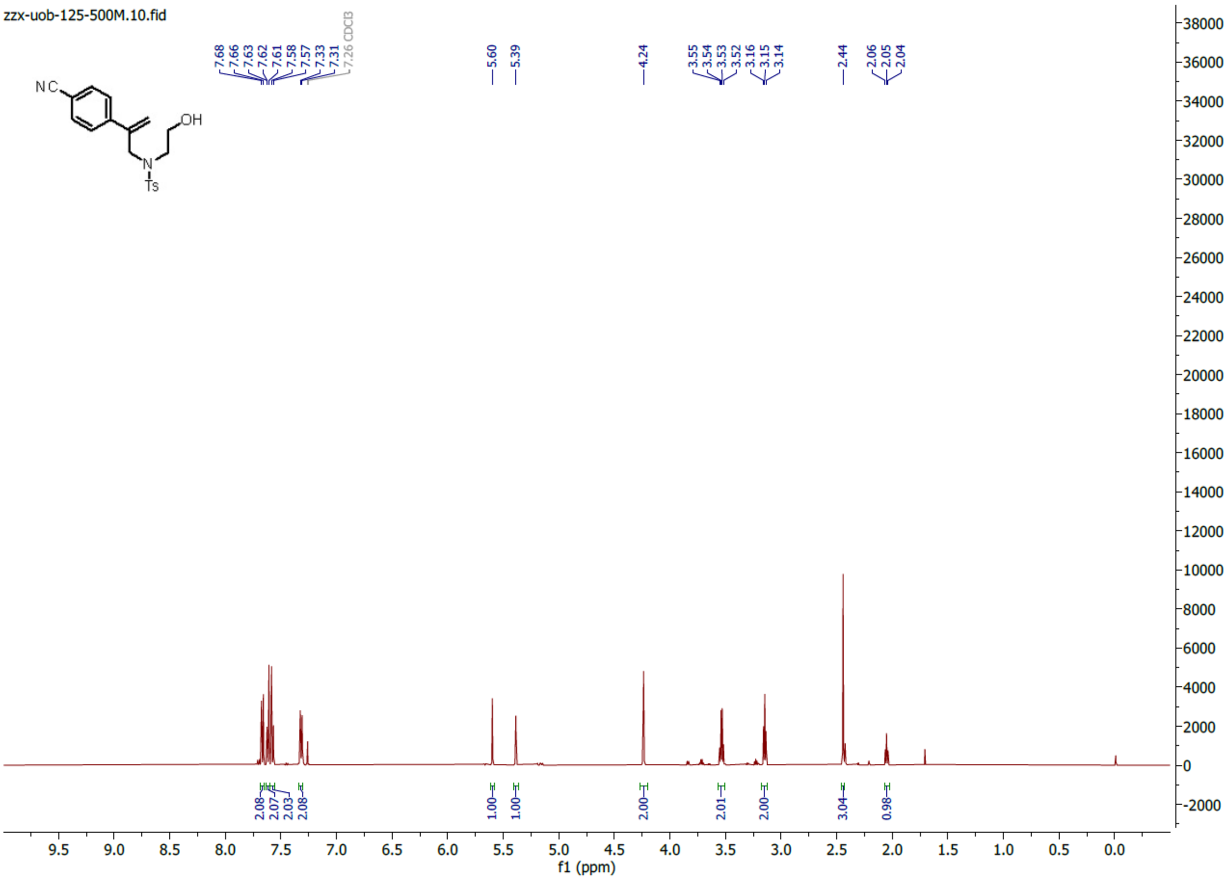

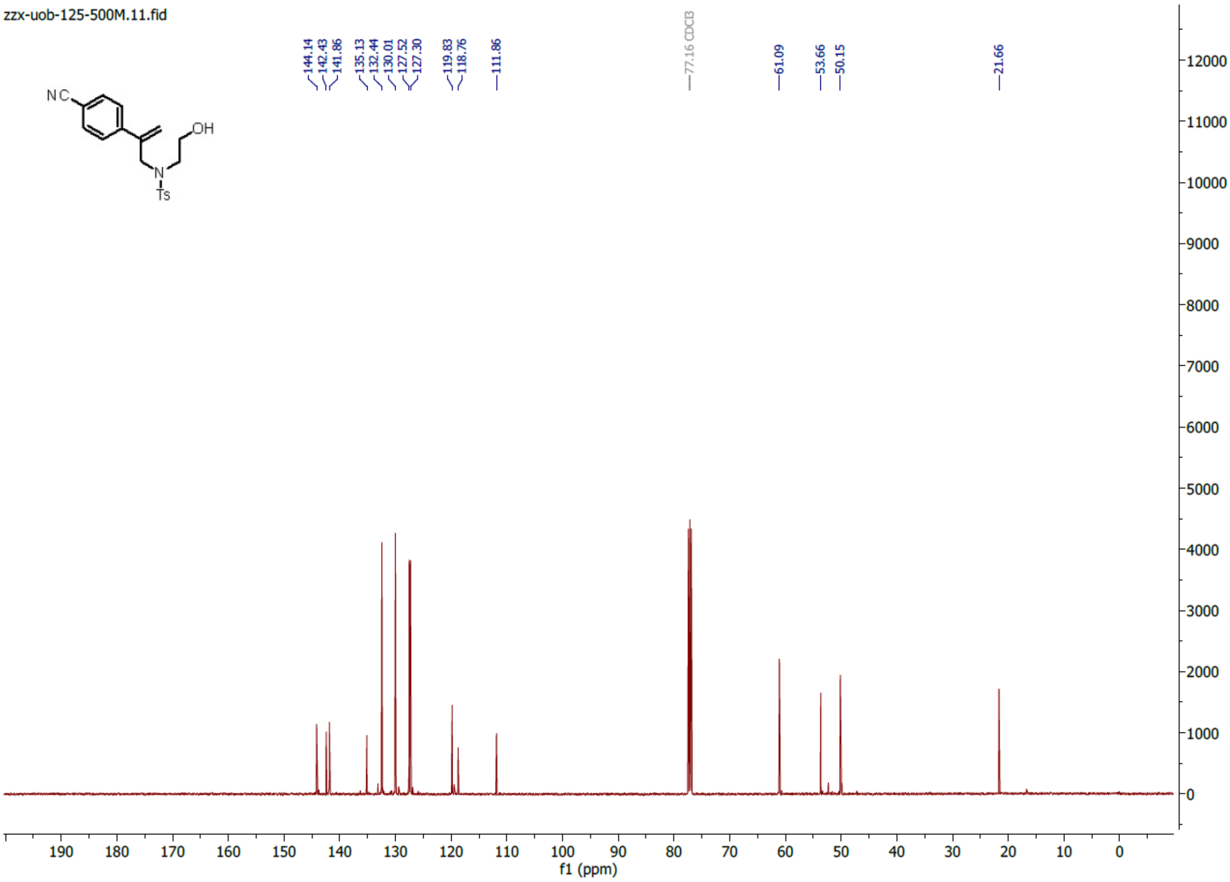

Compound S9b

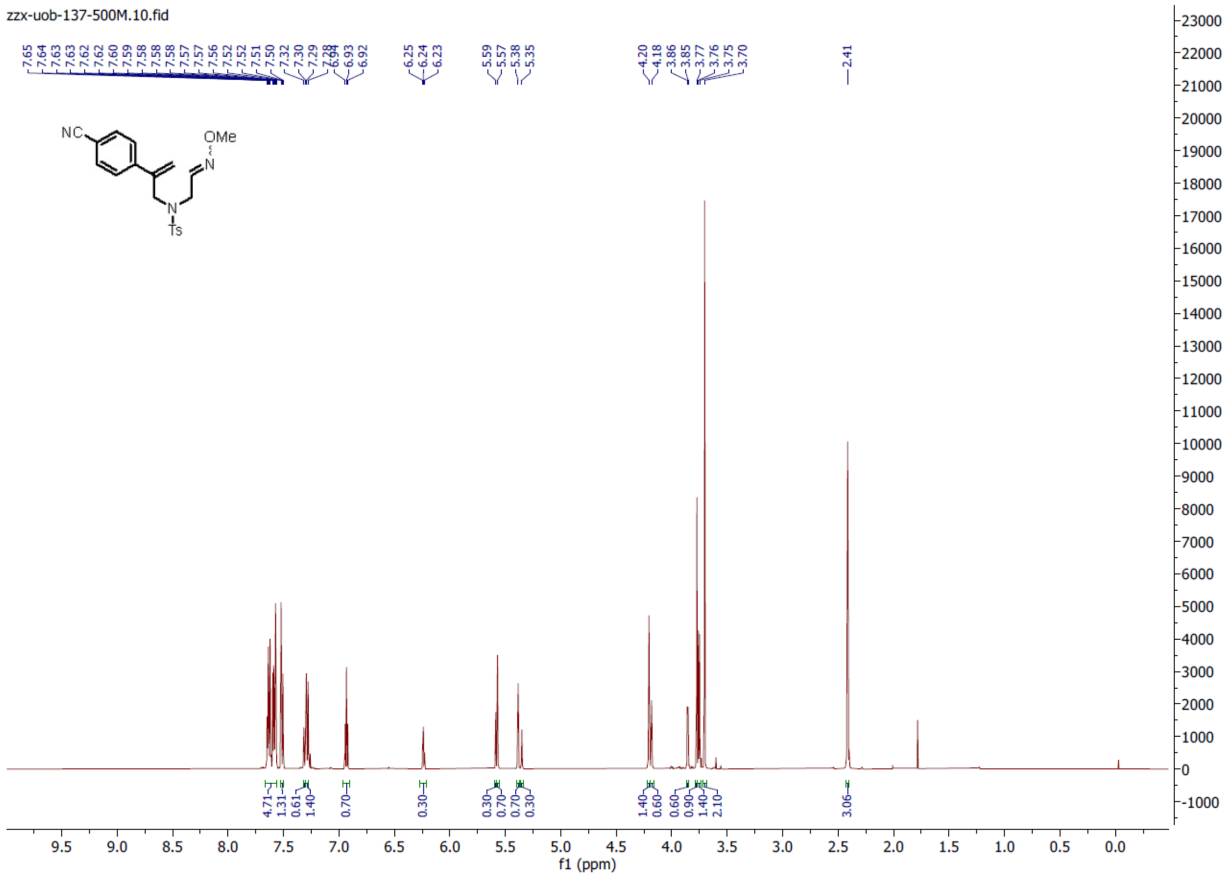

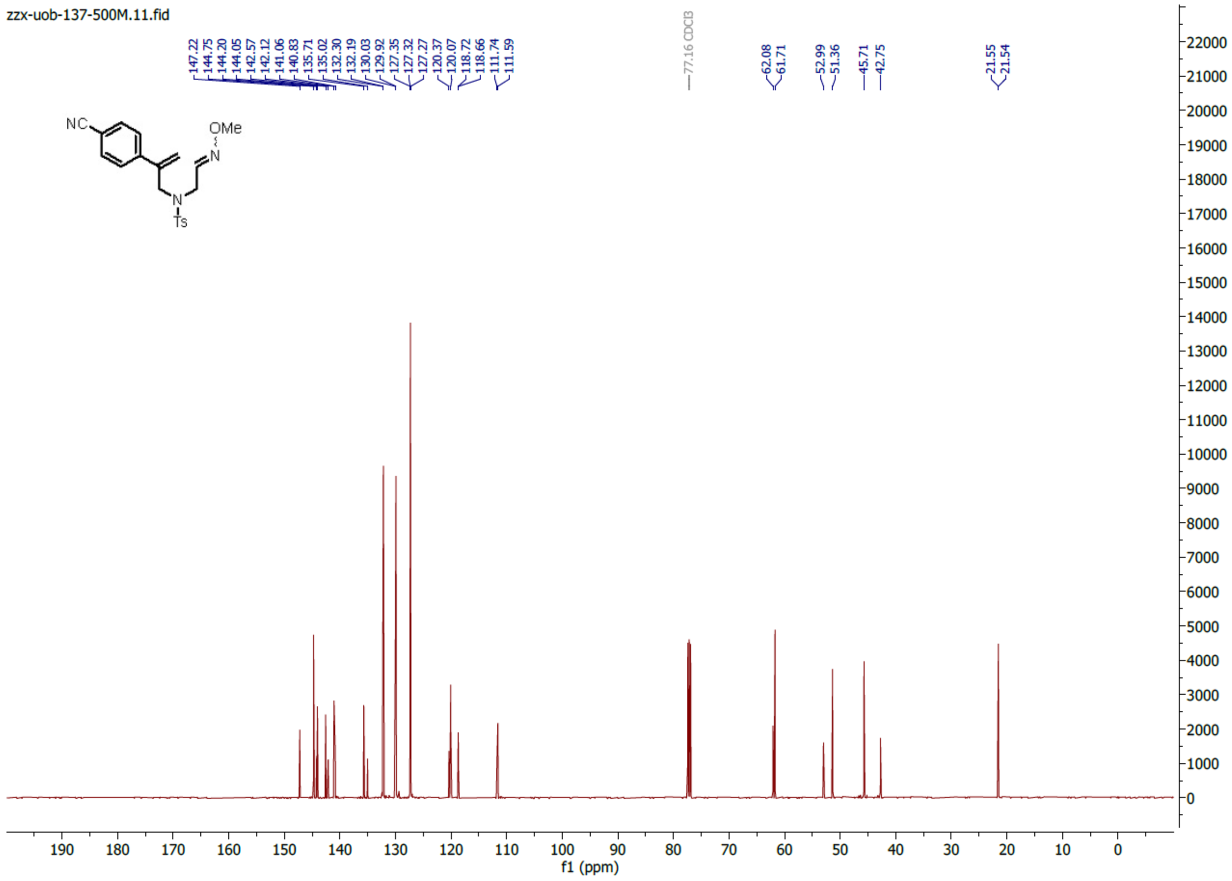

Compound S10a

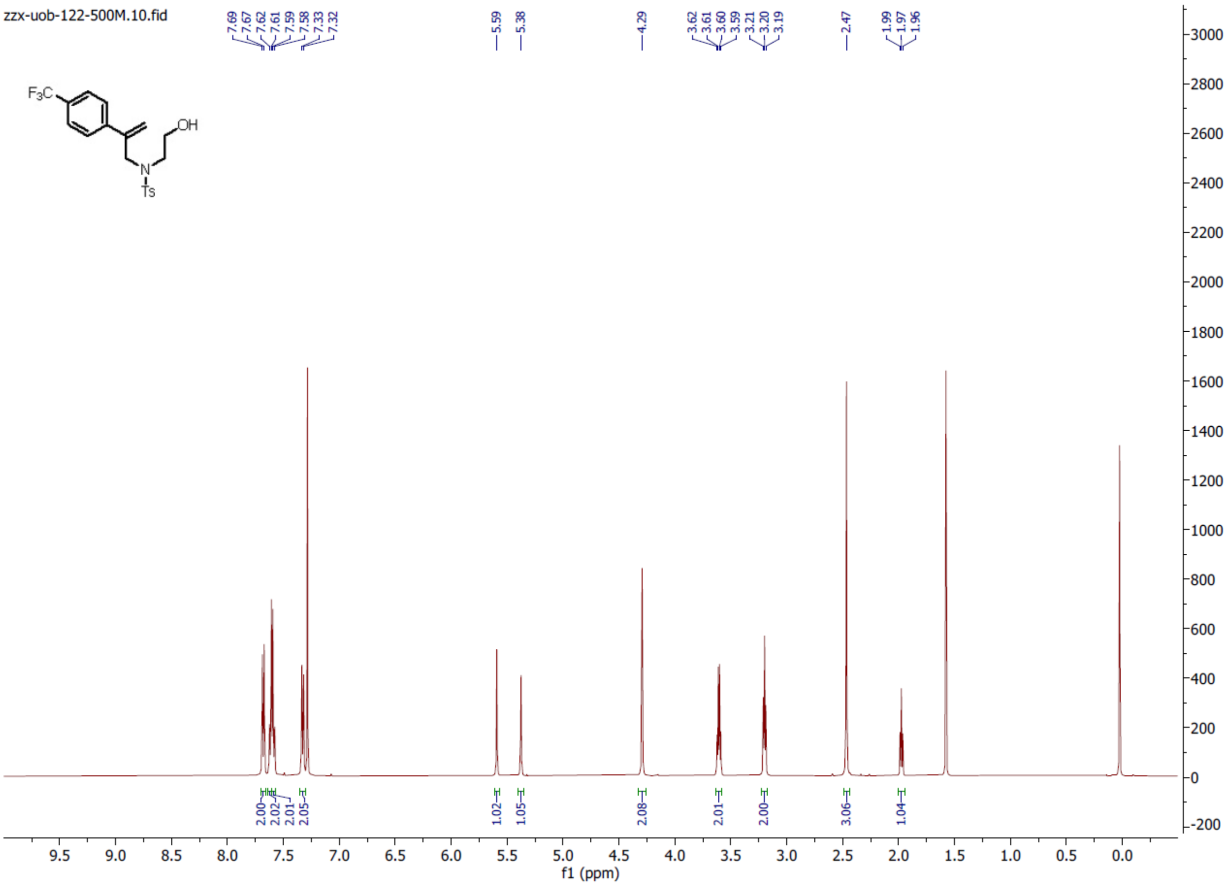

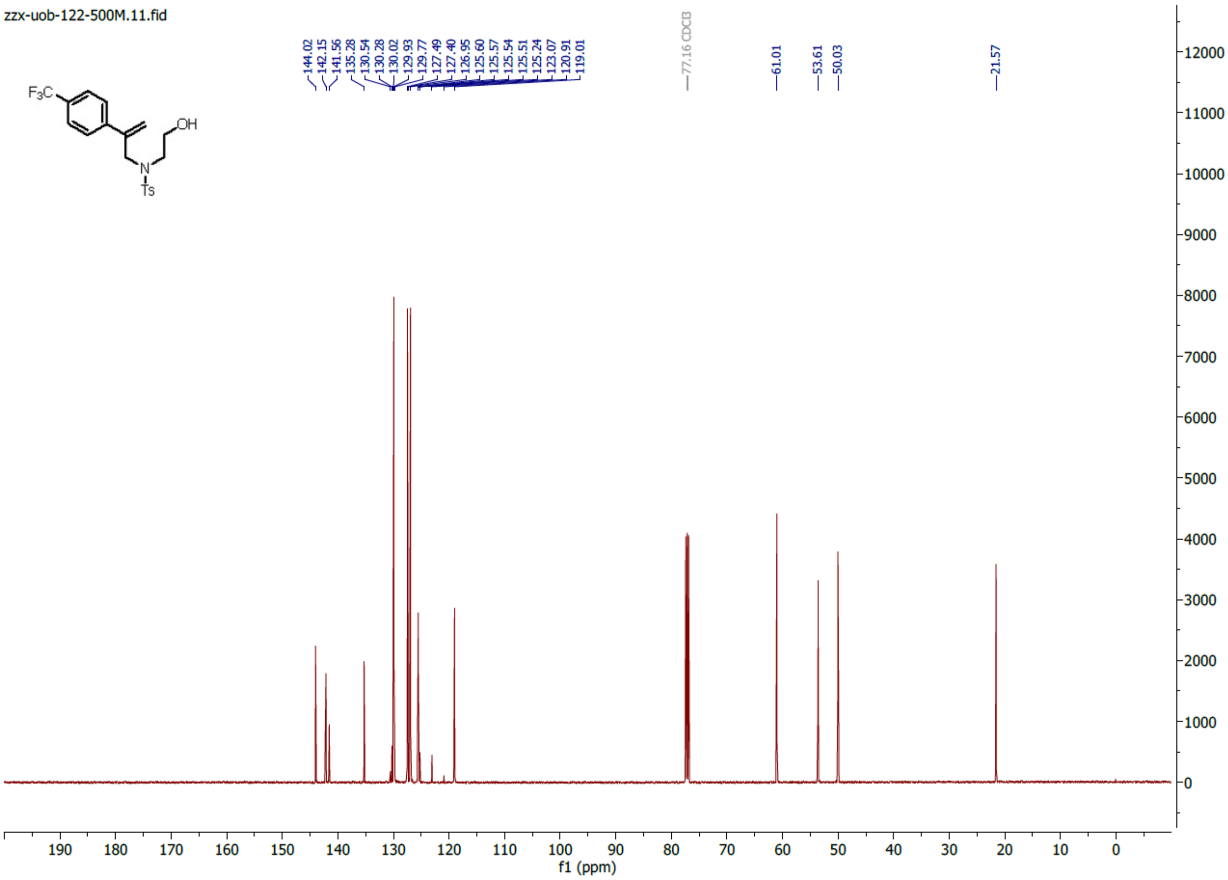

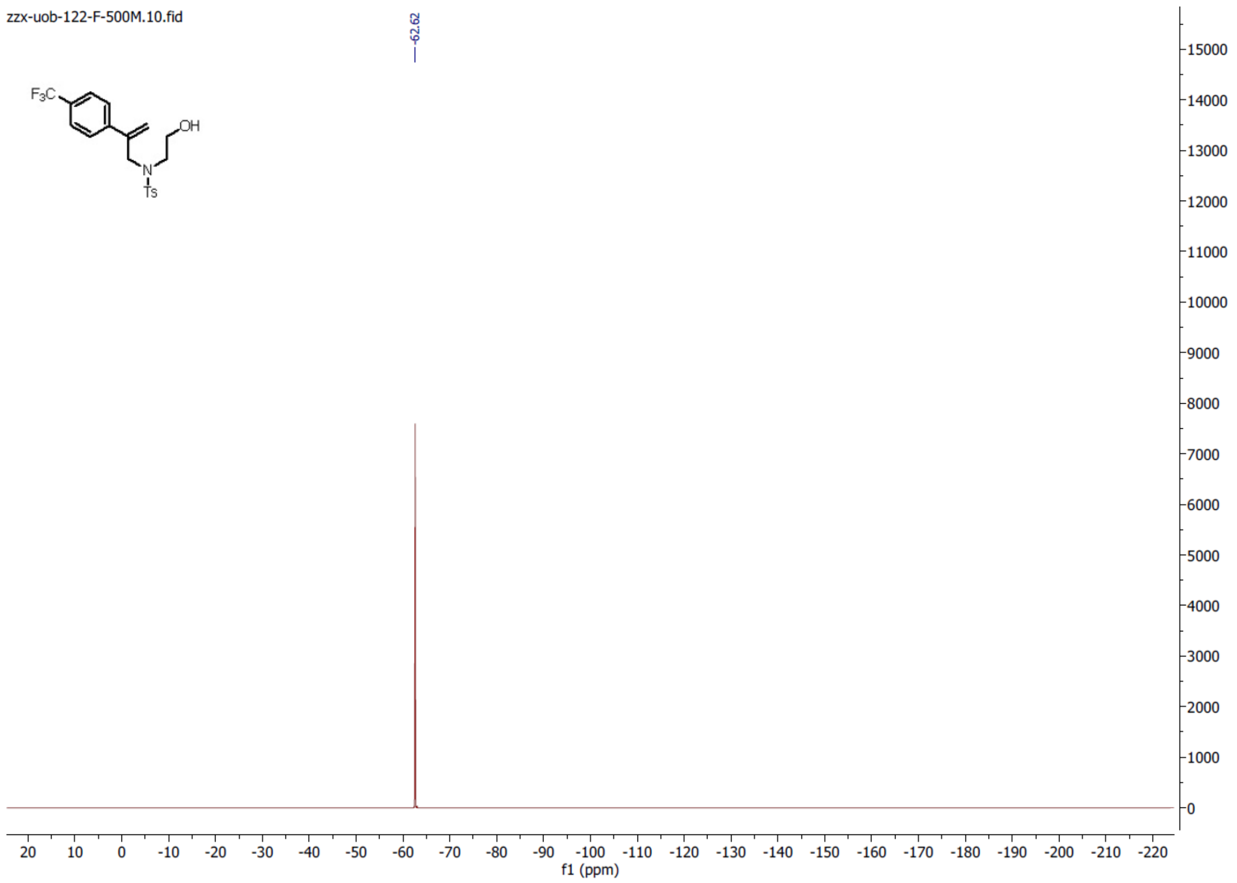
zzx-uob-122-F-500M.10.fid
-62.62
F3C
OH
N
Ts
15000
14000
13000
12000
11000
10000
9000
8000
7000
6000
5000
4000
3000
2000
1000
0
20 10 0 -10 -20 -30 -40 -50 -60 -70 -80 -90 -100 -110 -120 -130 -140 -150 -160 -170 -180 -190 -200 -210 -220
f1 (ppm)

Compound S10b

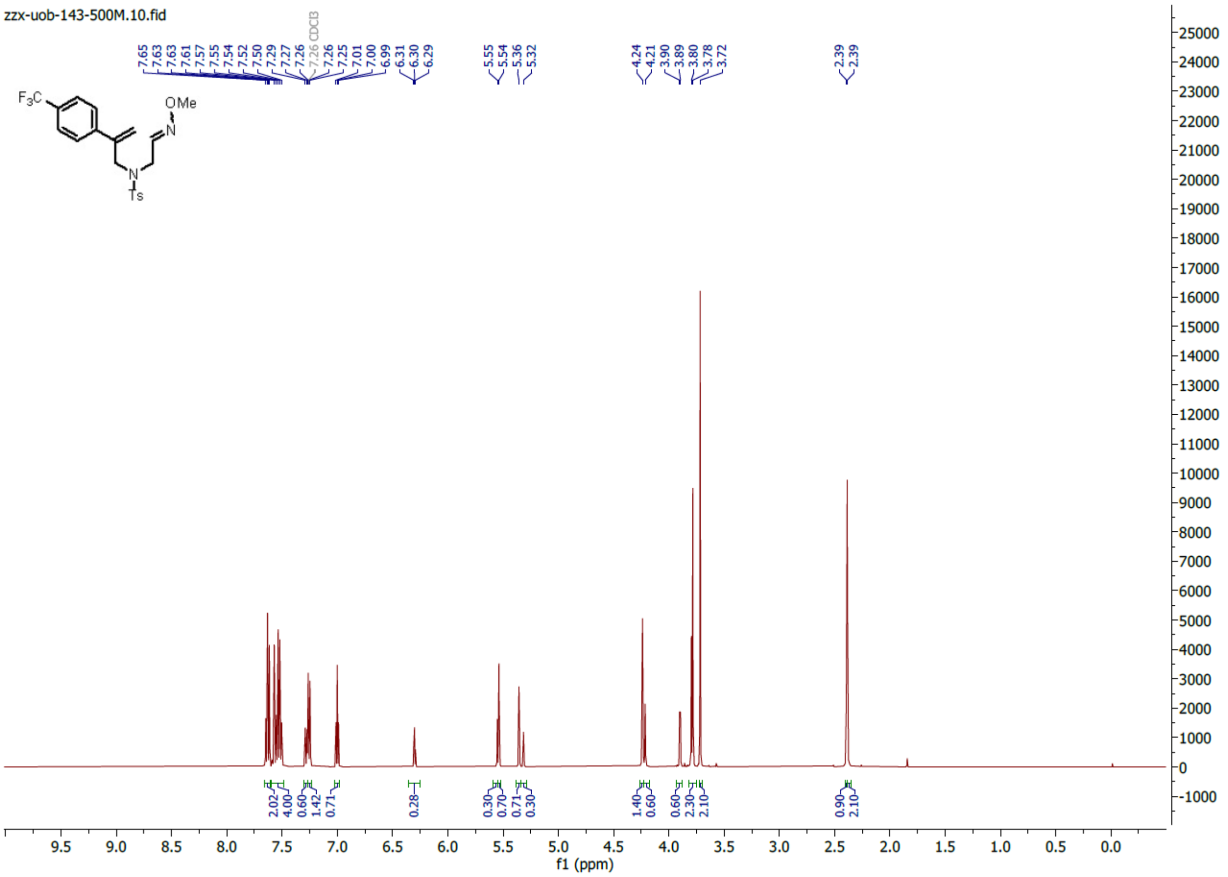

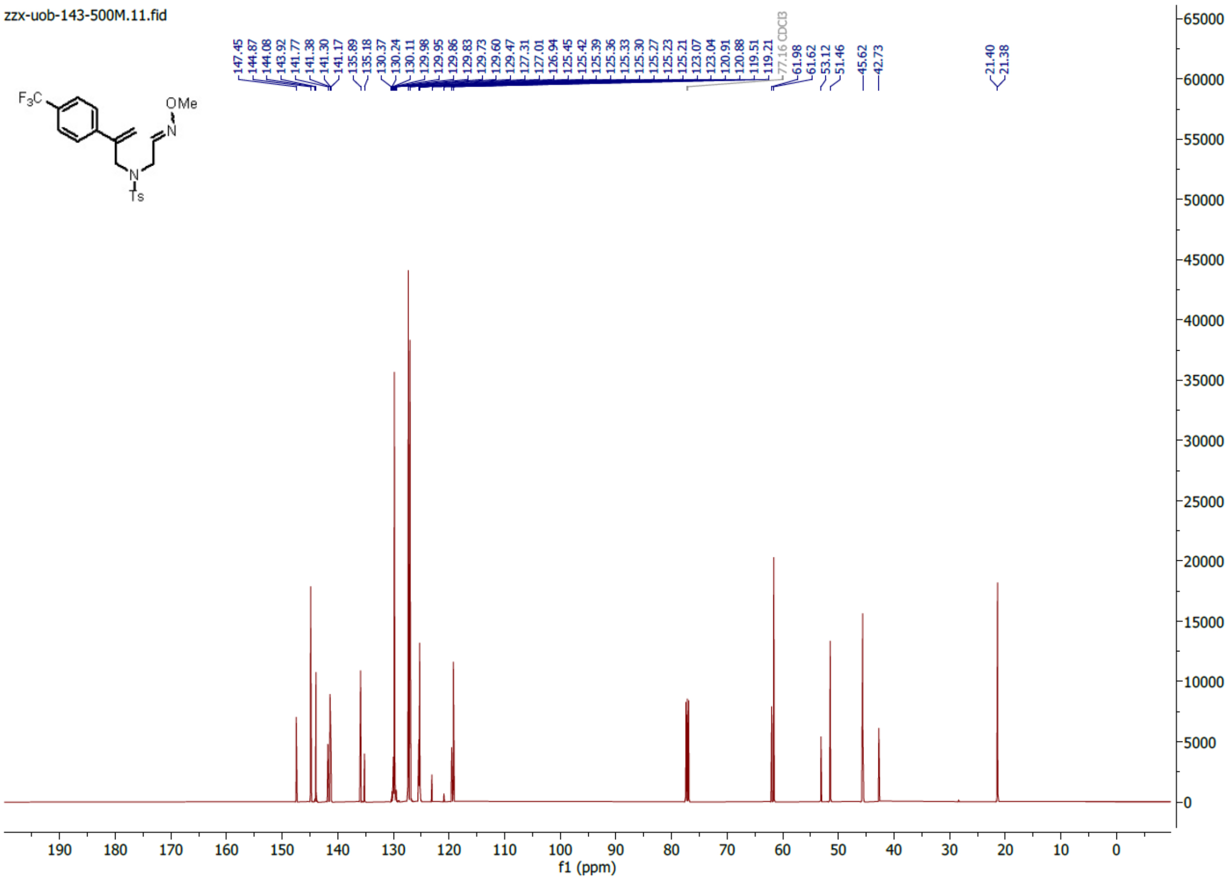

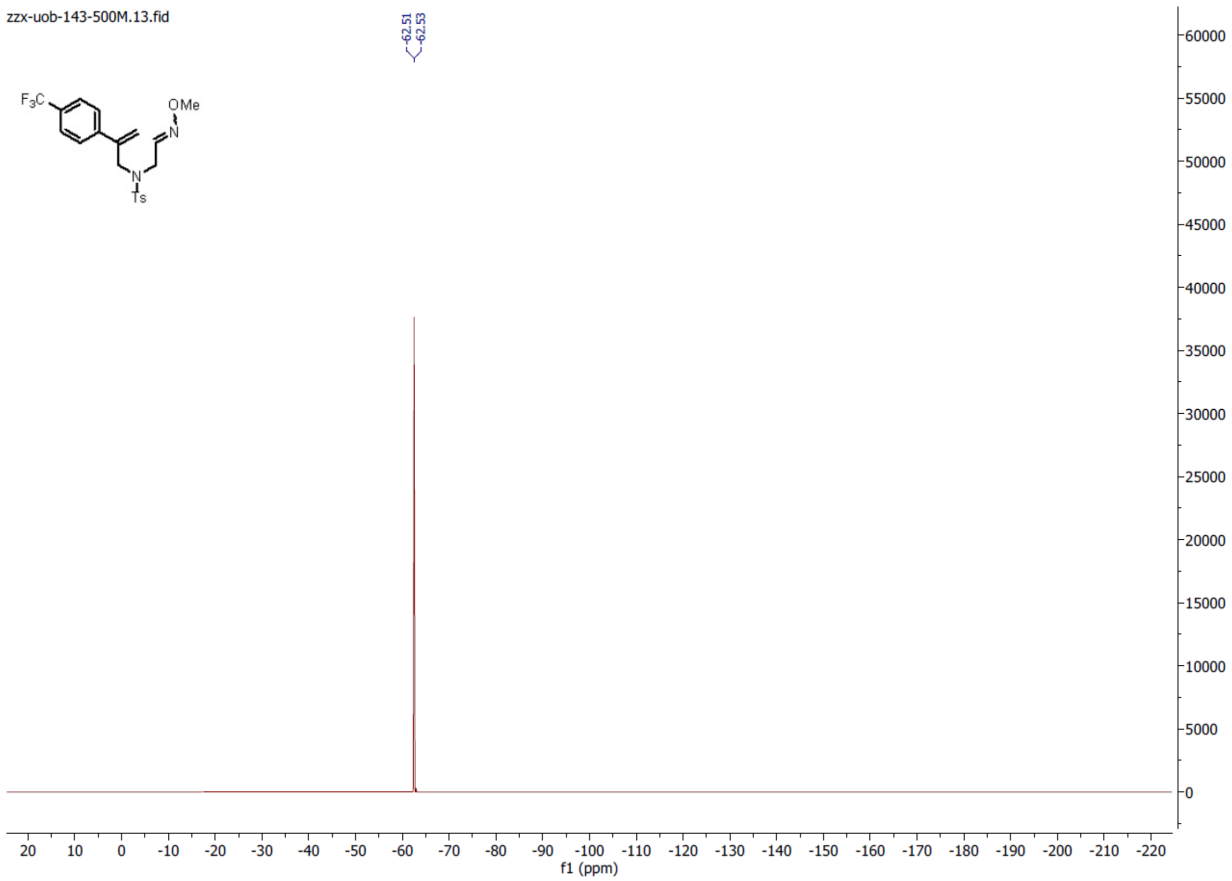

zzx-uob-143-500M.13.fid
-62.51
-62.53
F3C
OMe
N
N
Ts
60000
55000
50000
45000
40000
35000
30000
25000
20000
15000
10000
5000
0
20 10 0 -10 -20 -30 -40 -50 -60 -70 -80 -90 -100 -110 -120 -130 -140 -150 -160 -170 -180 -190 -200 -210 -220
f1 (ppm)

Compound S11a

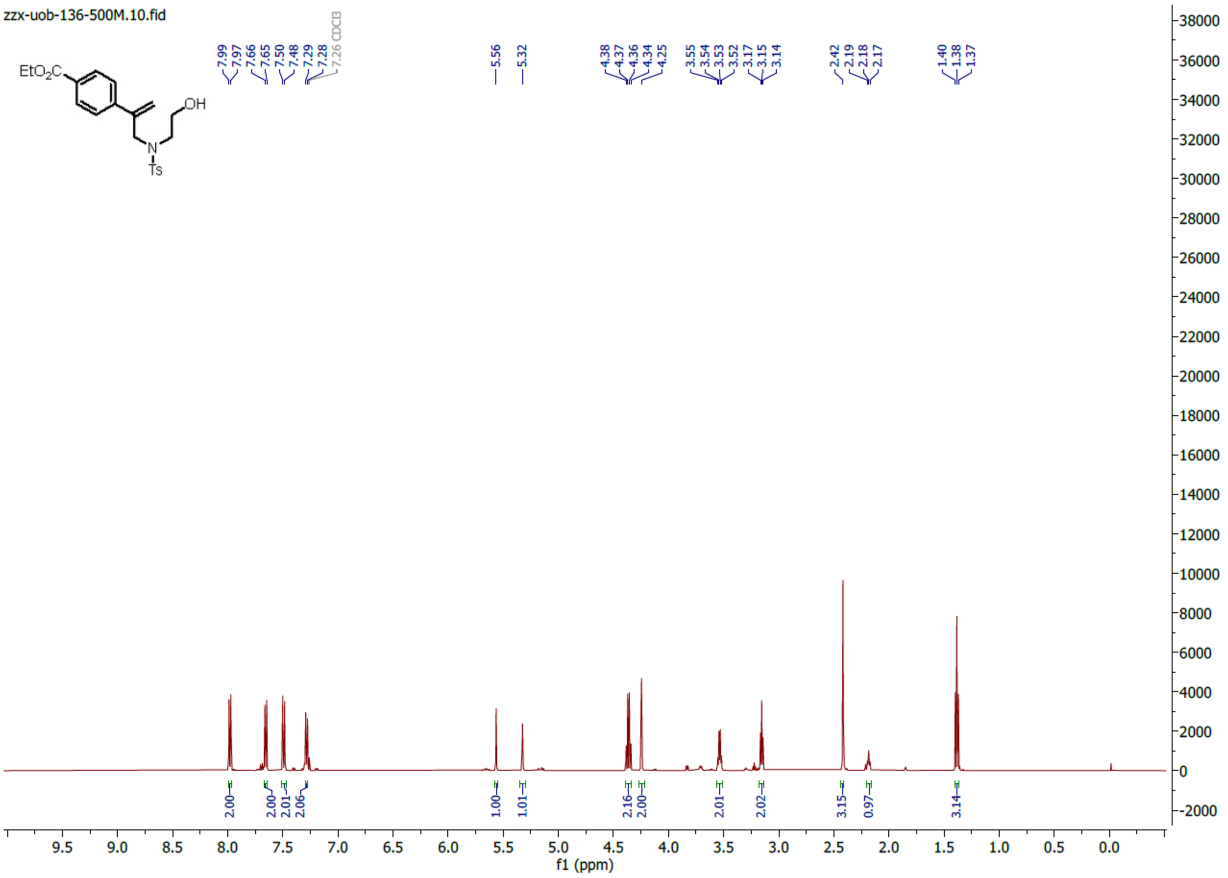

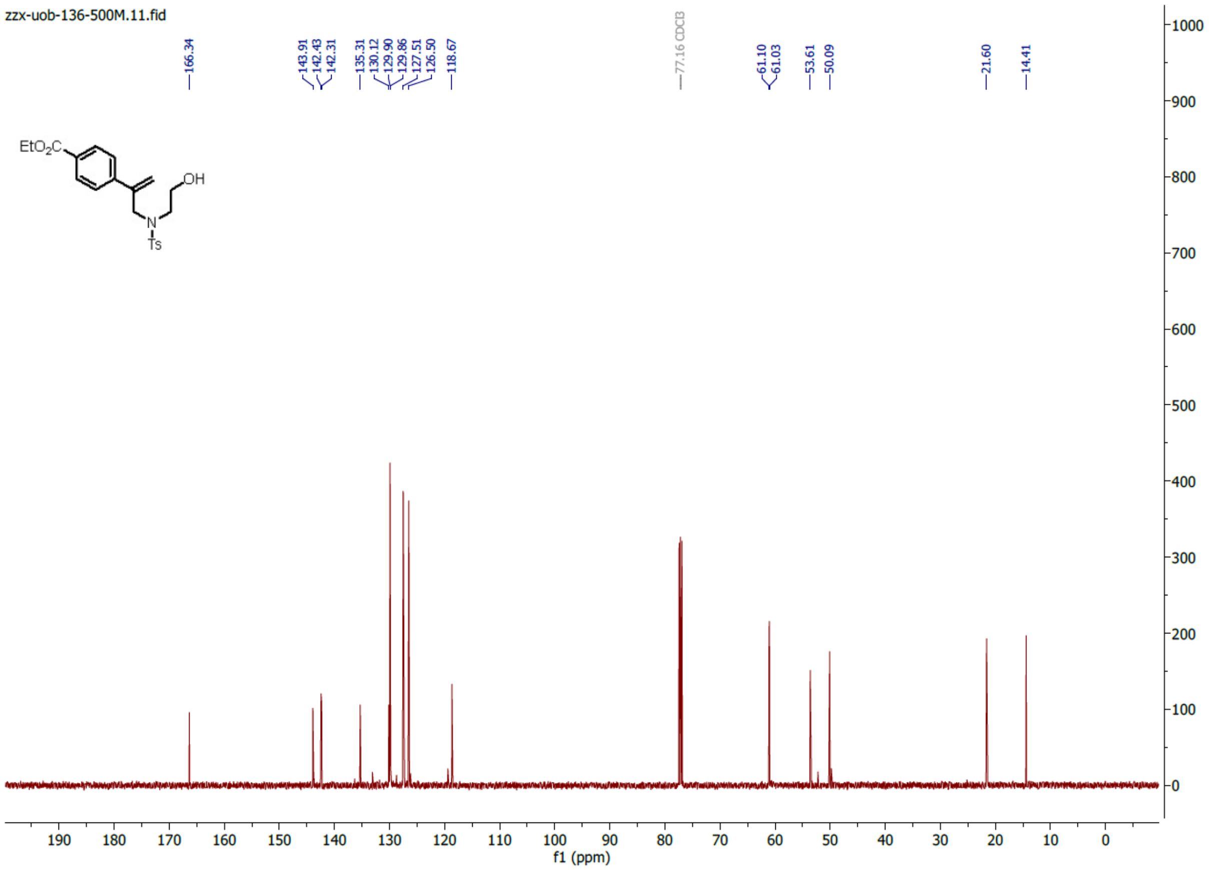

Compound S11b

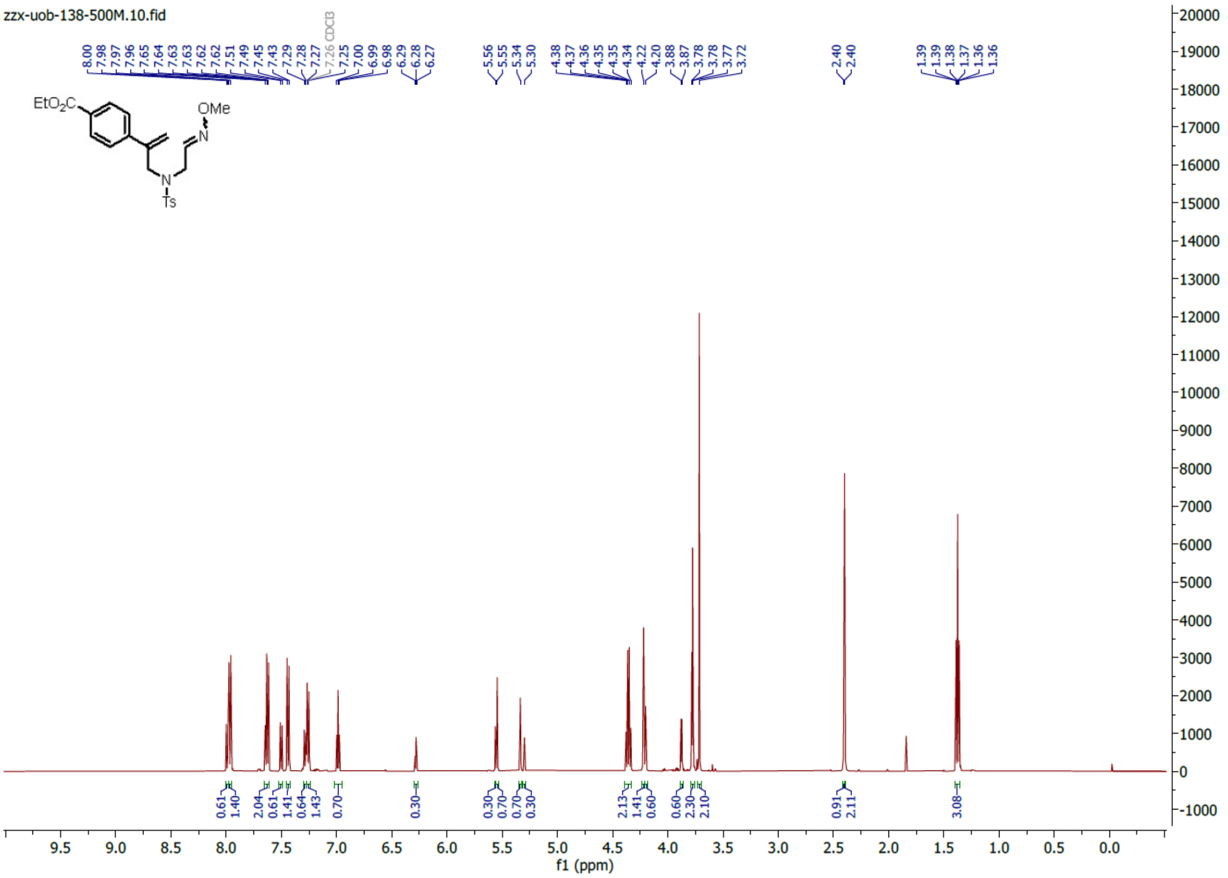

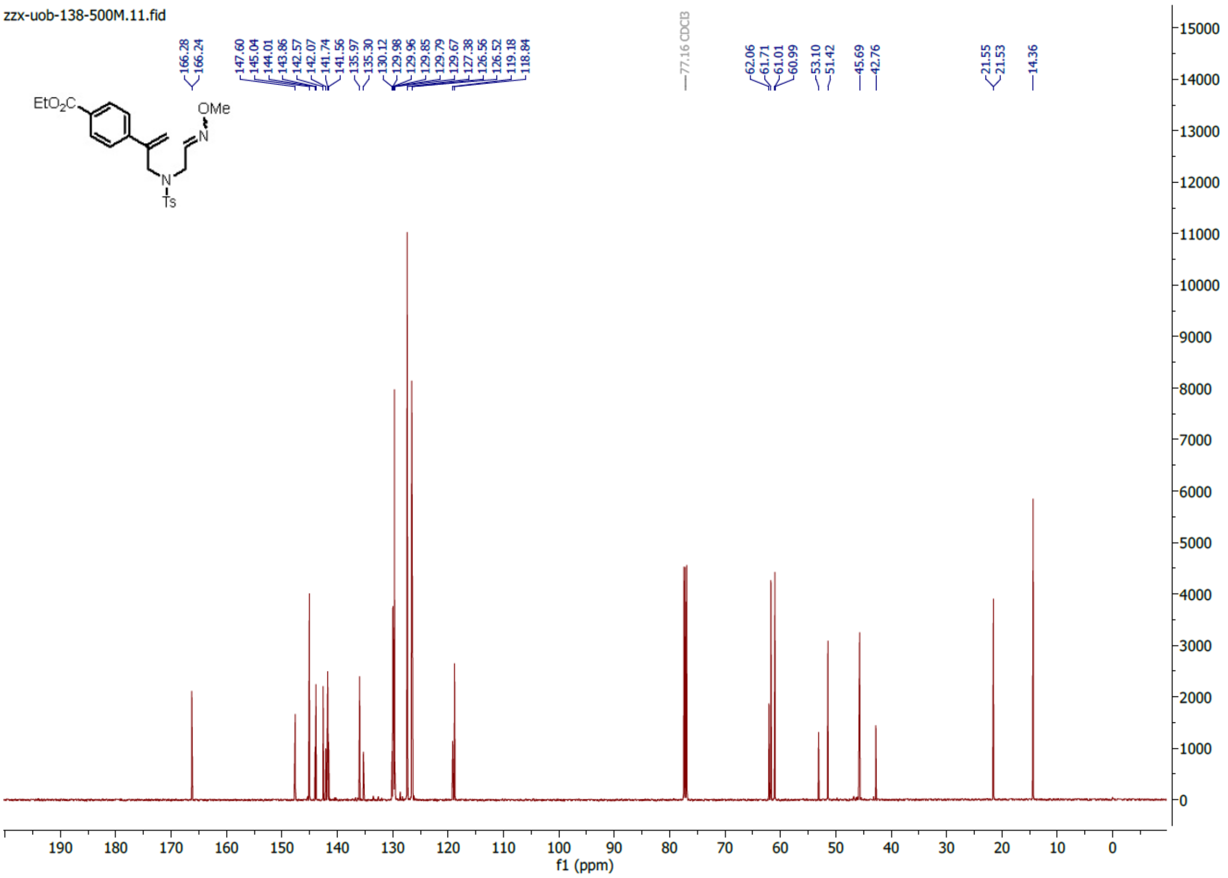

Compound S12a

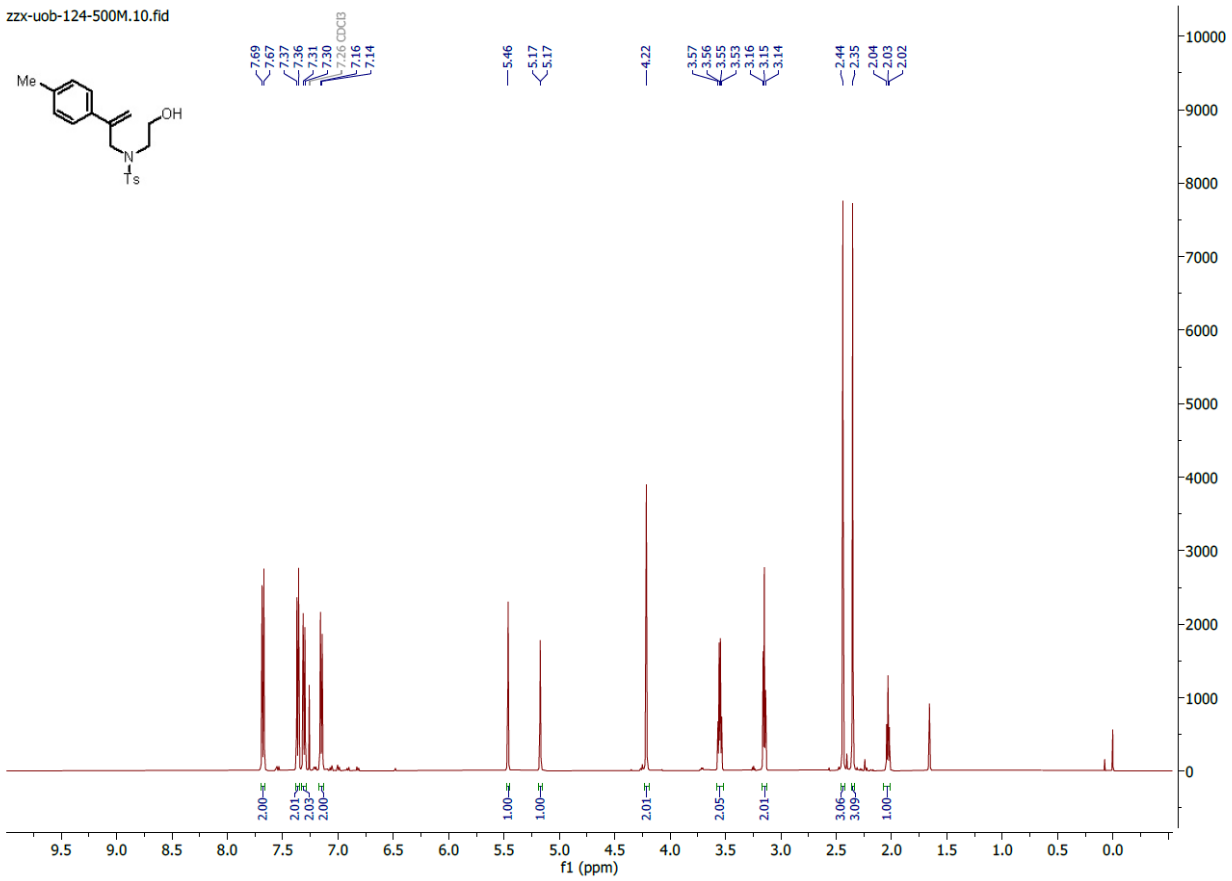

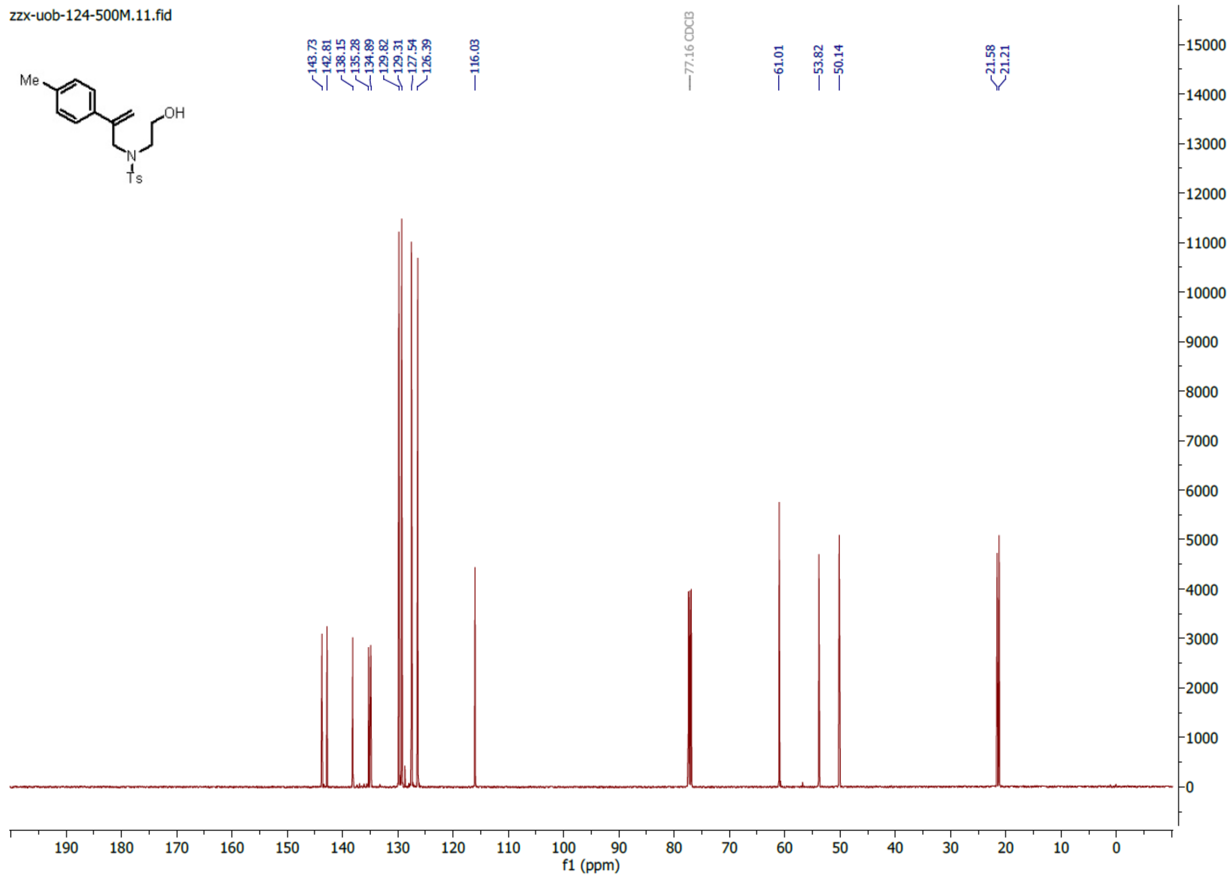

Compound S12b

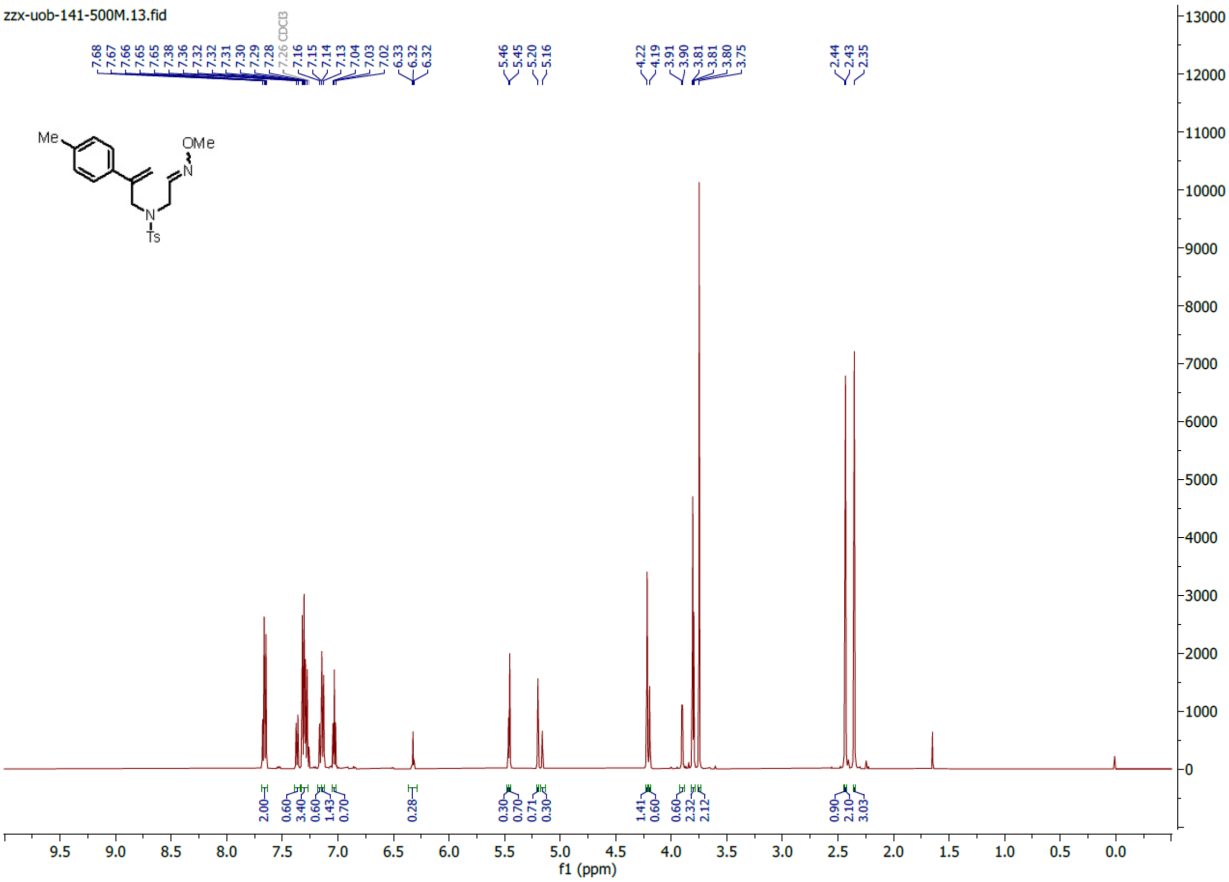

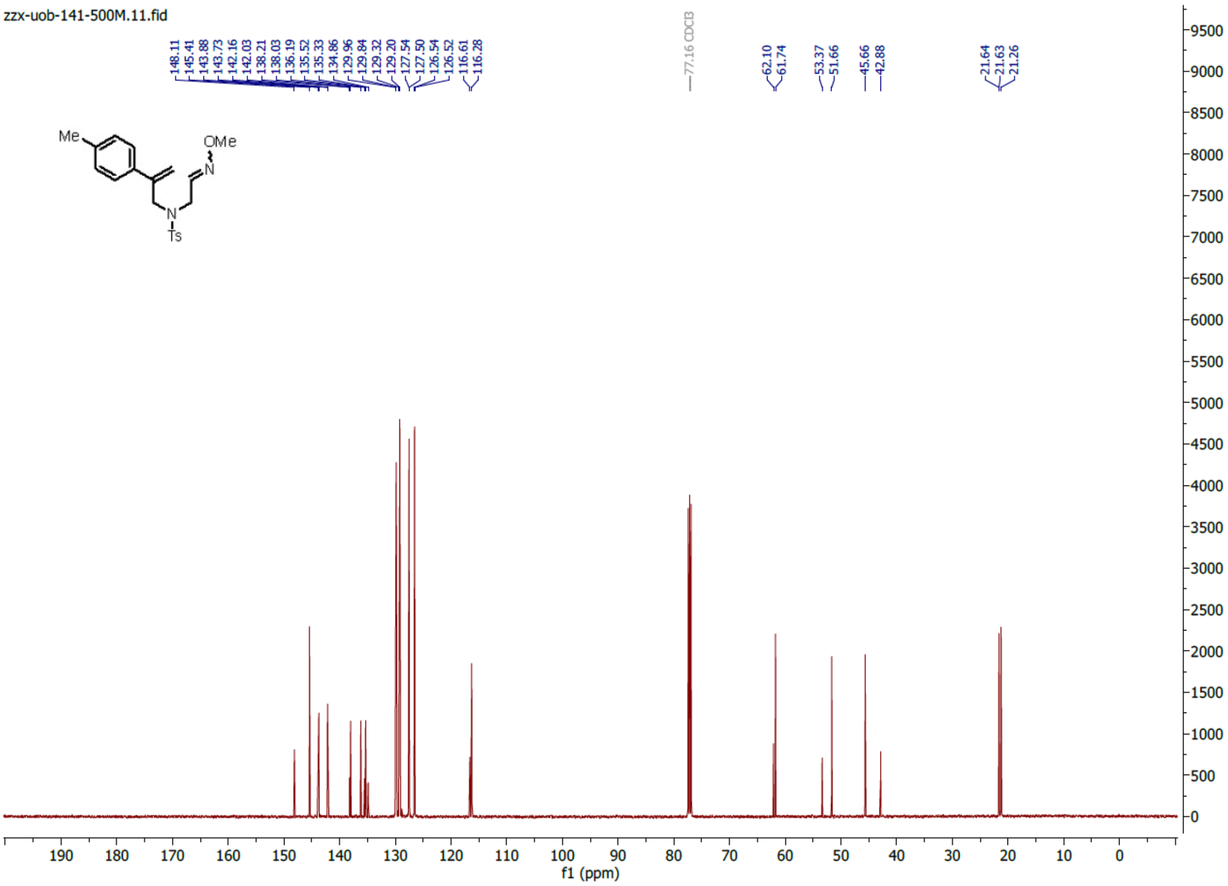

Compound S13a

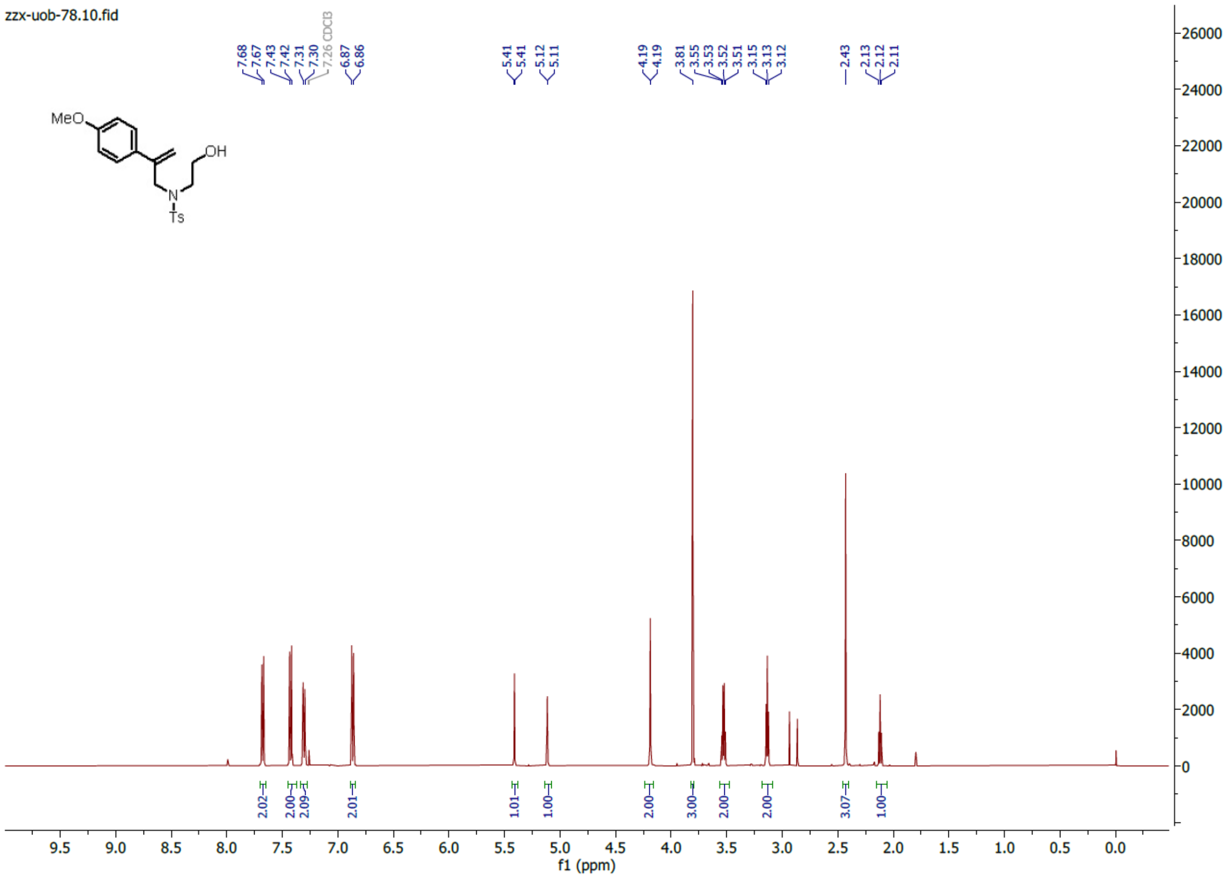

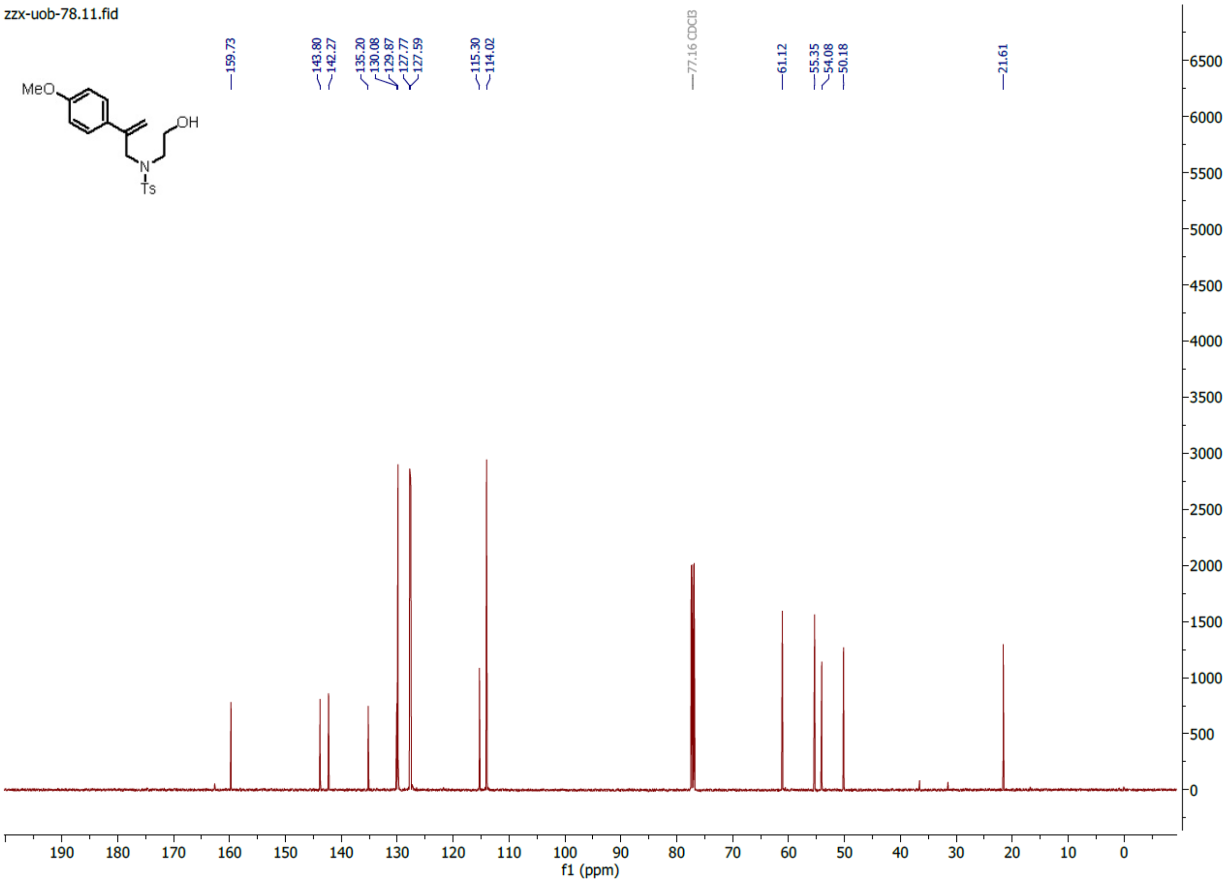

Compound S13b

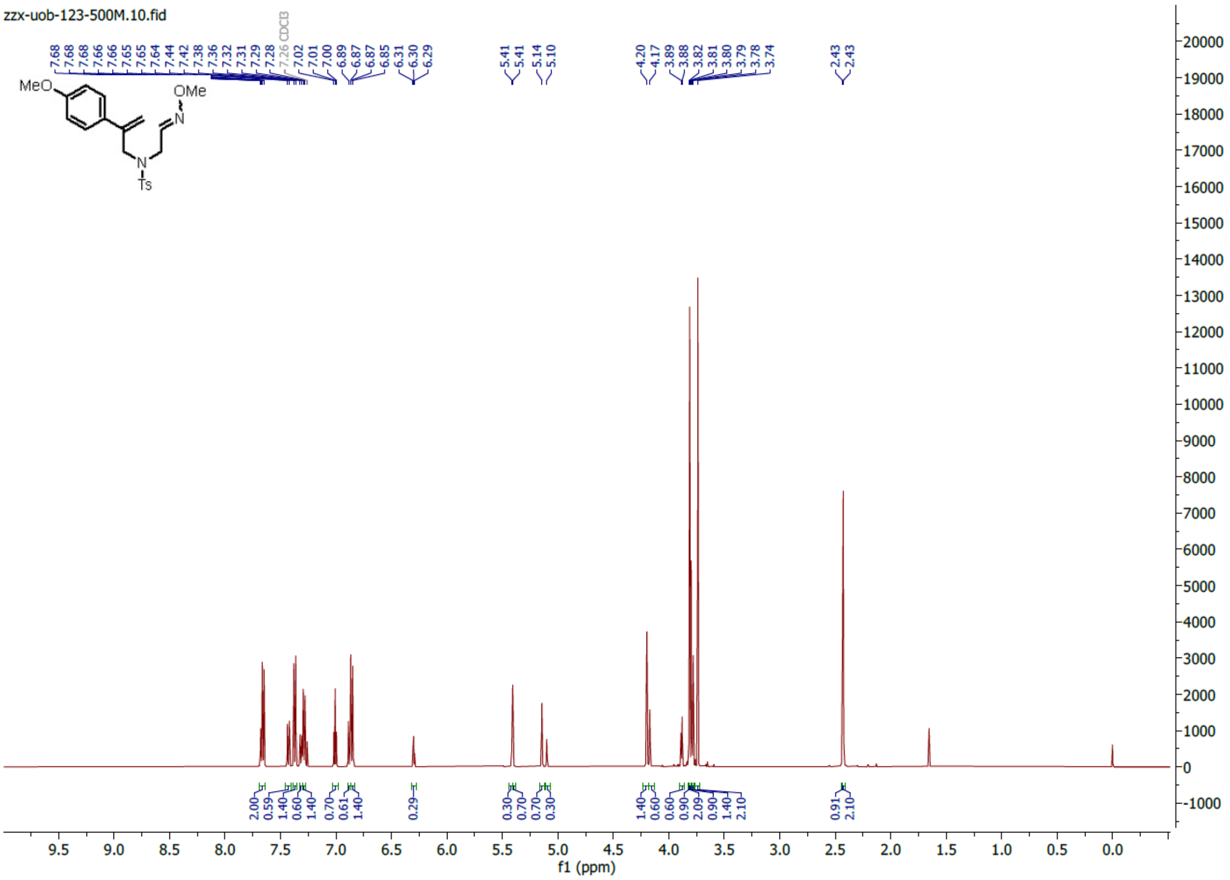

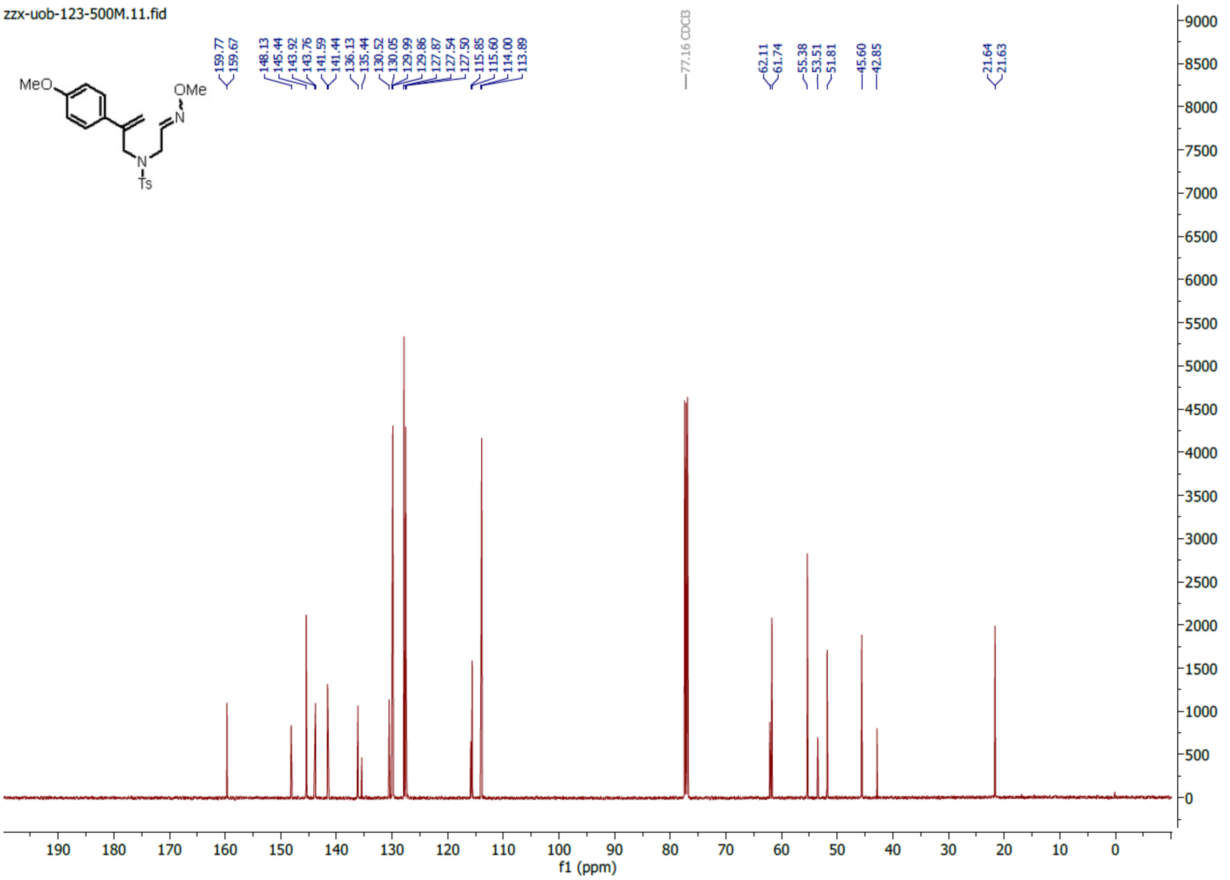

Compound S14a

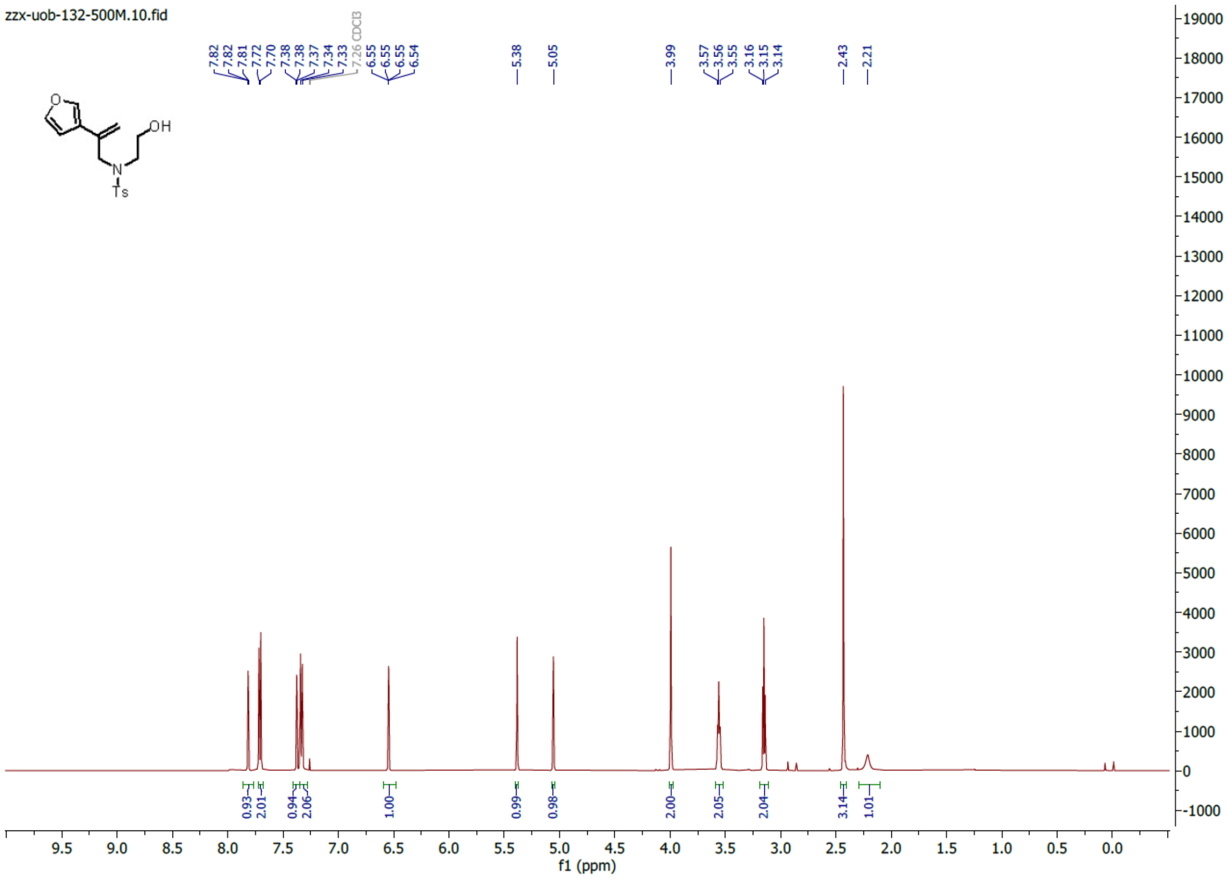

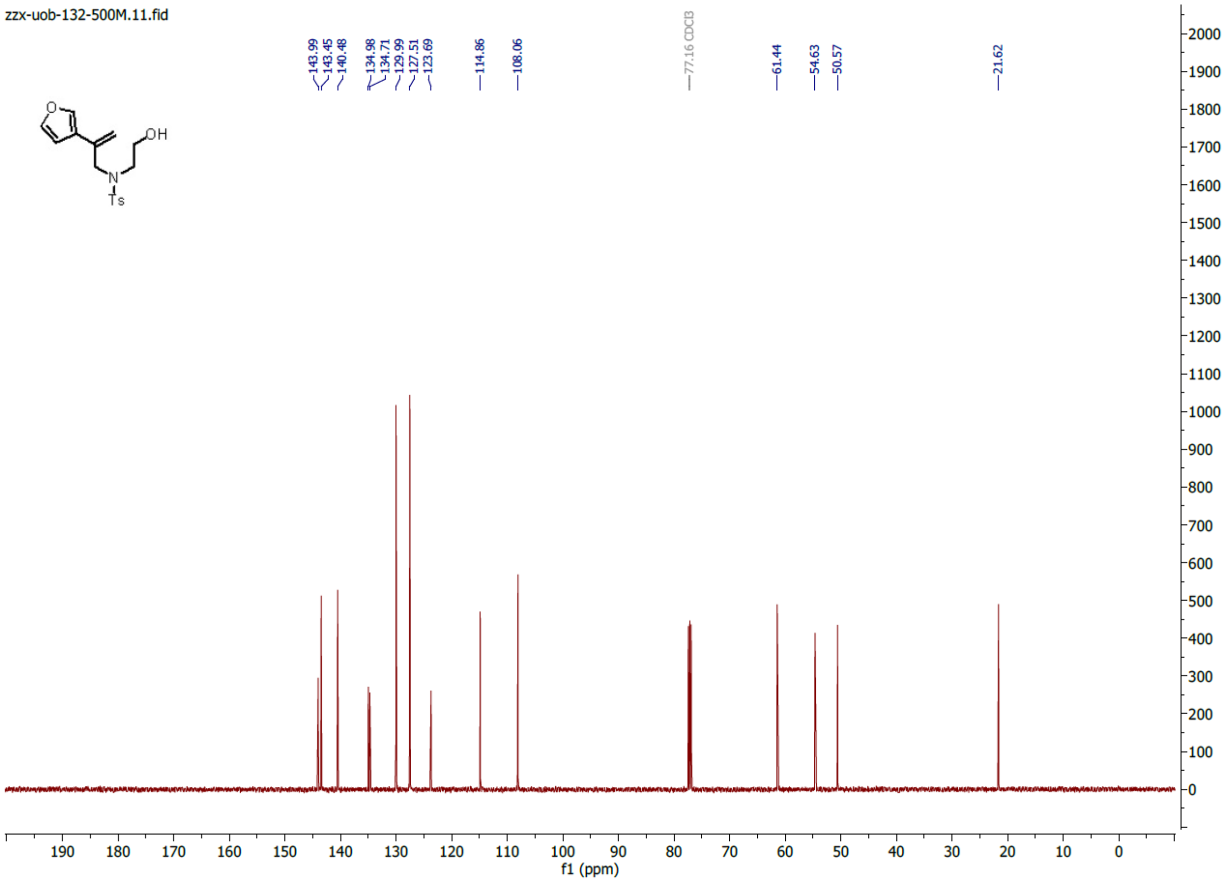

Compound S14b

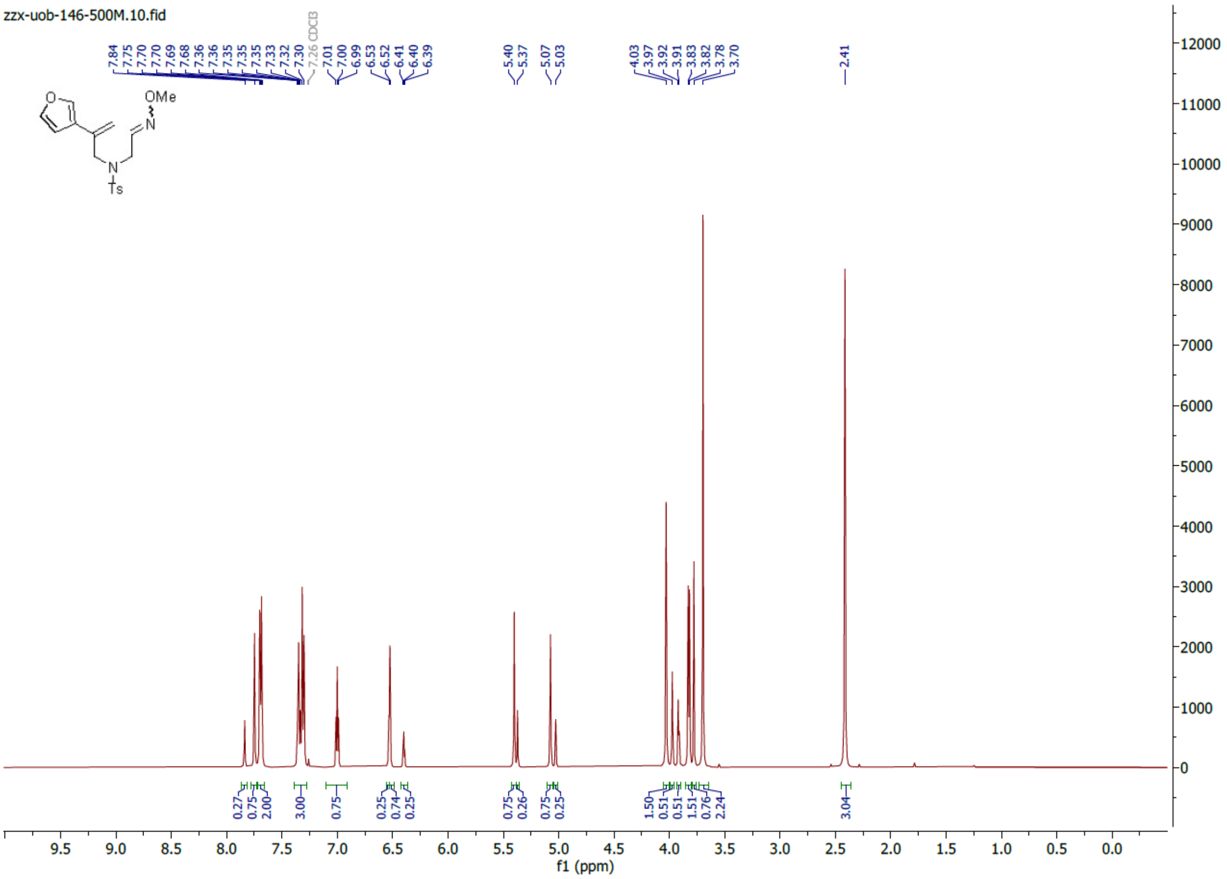

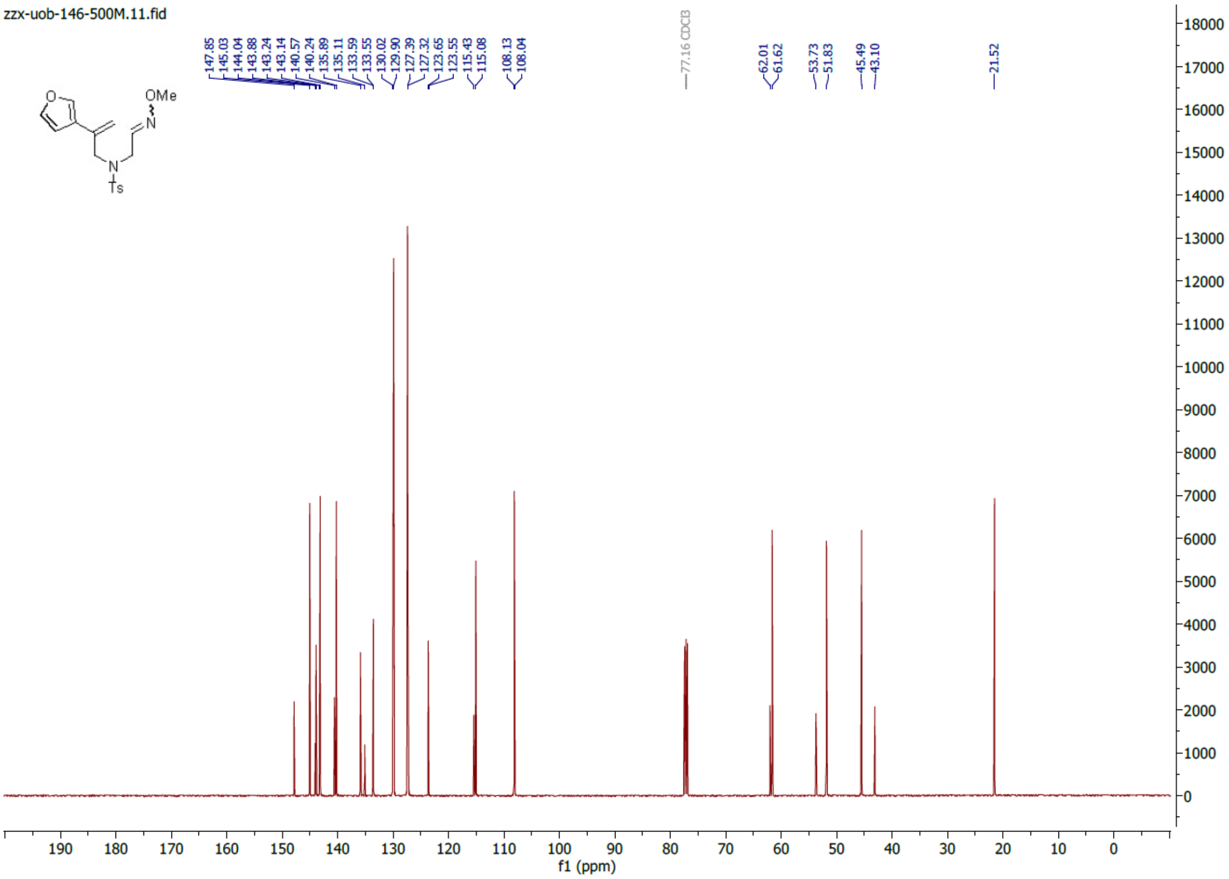

Compound S15a

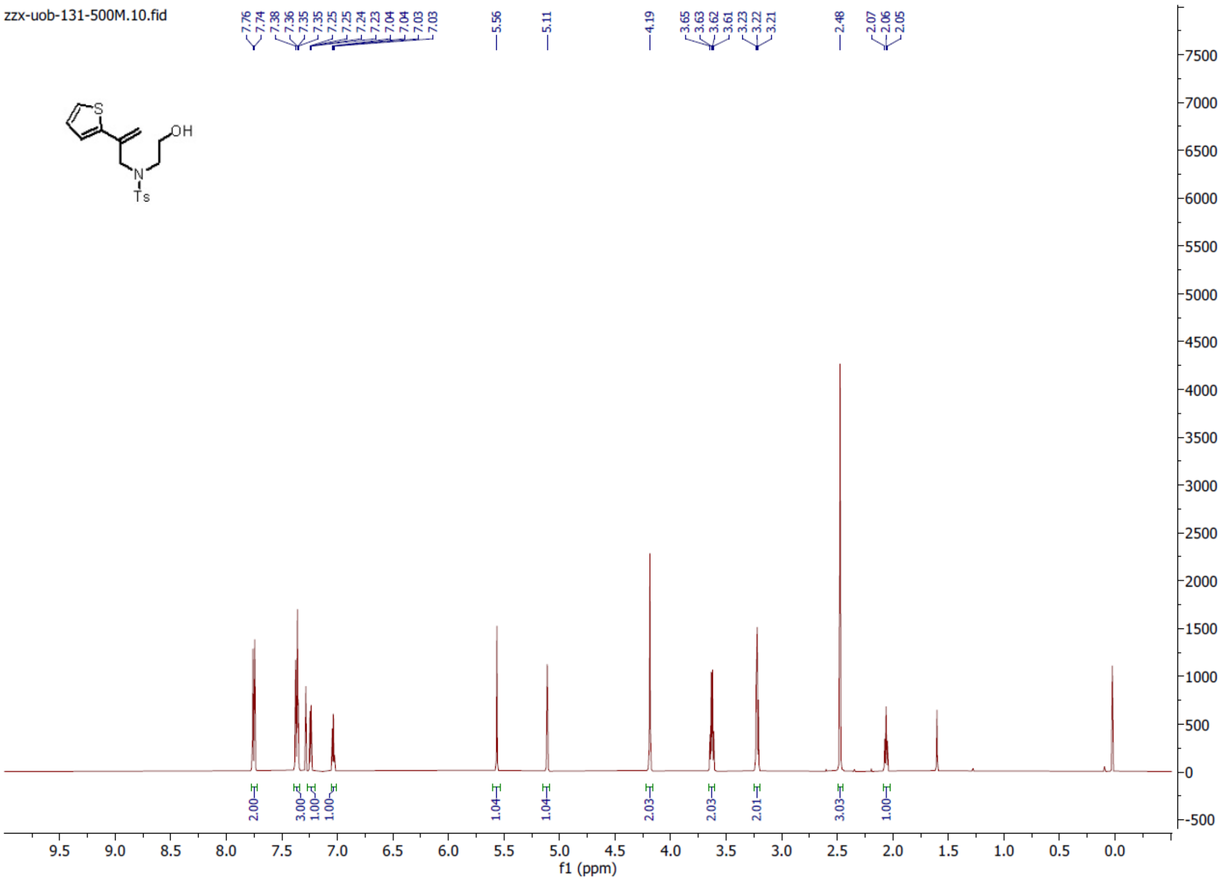

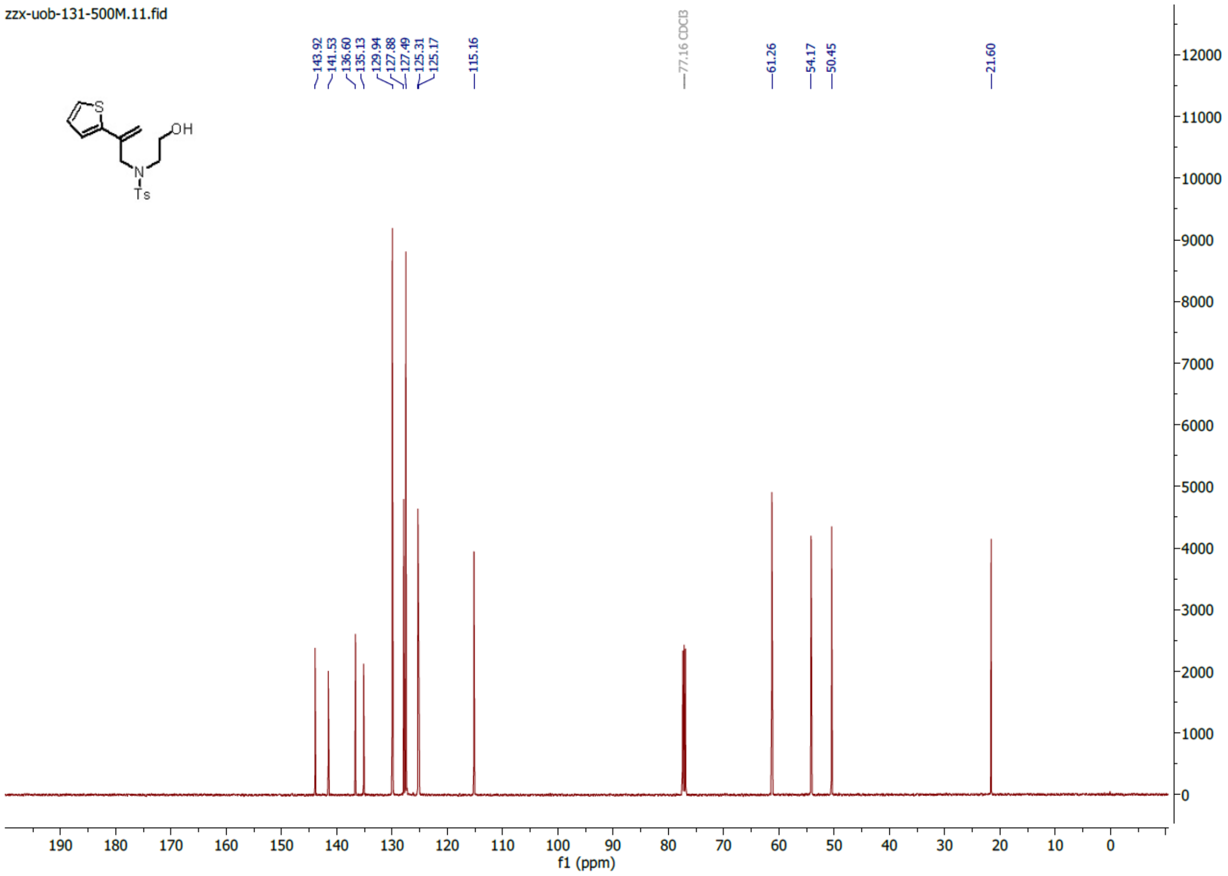

Compound S15b

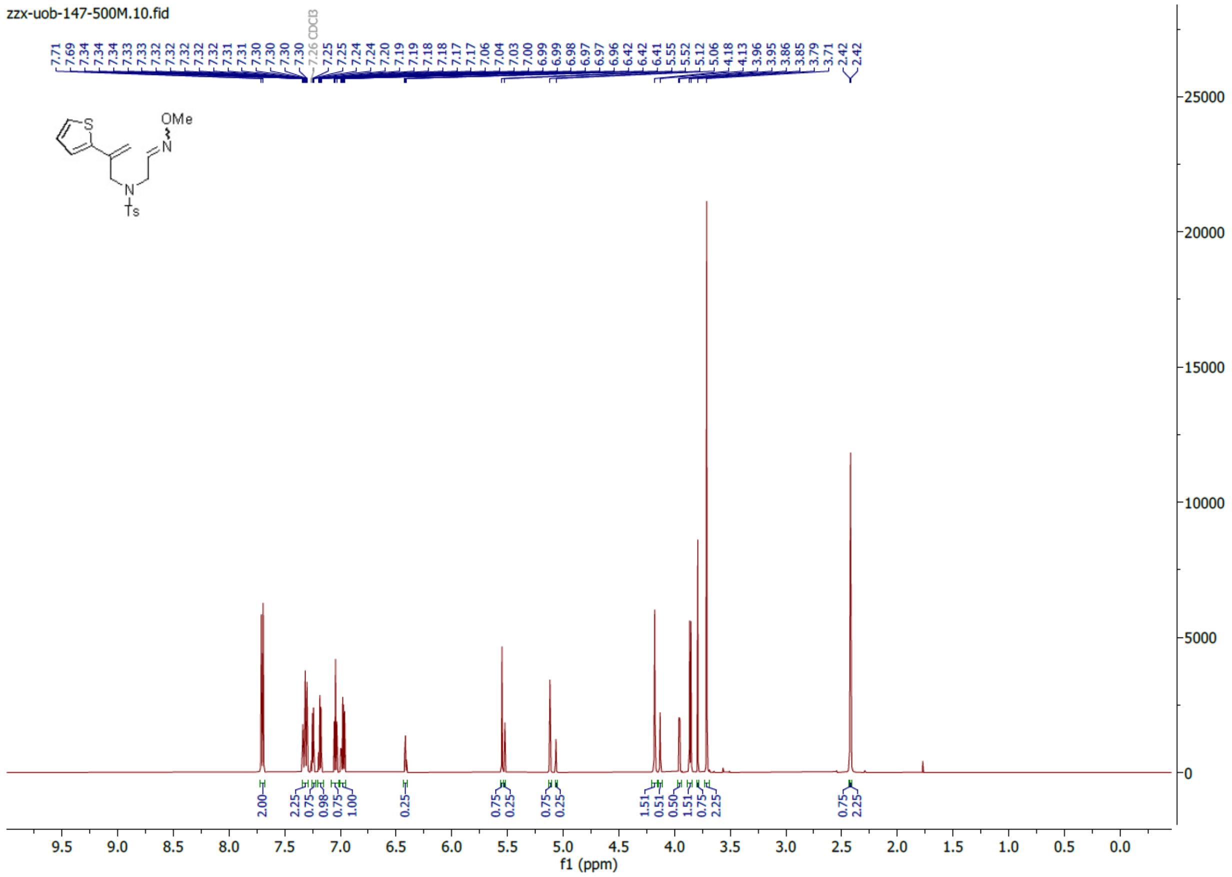

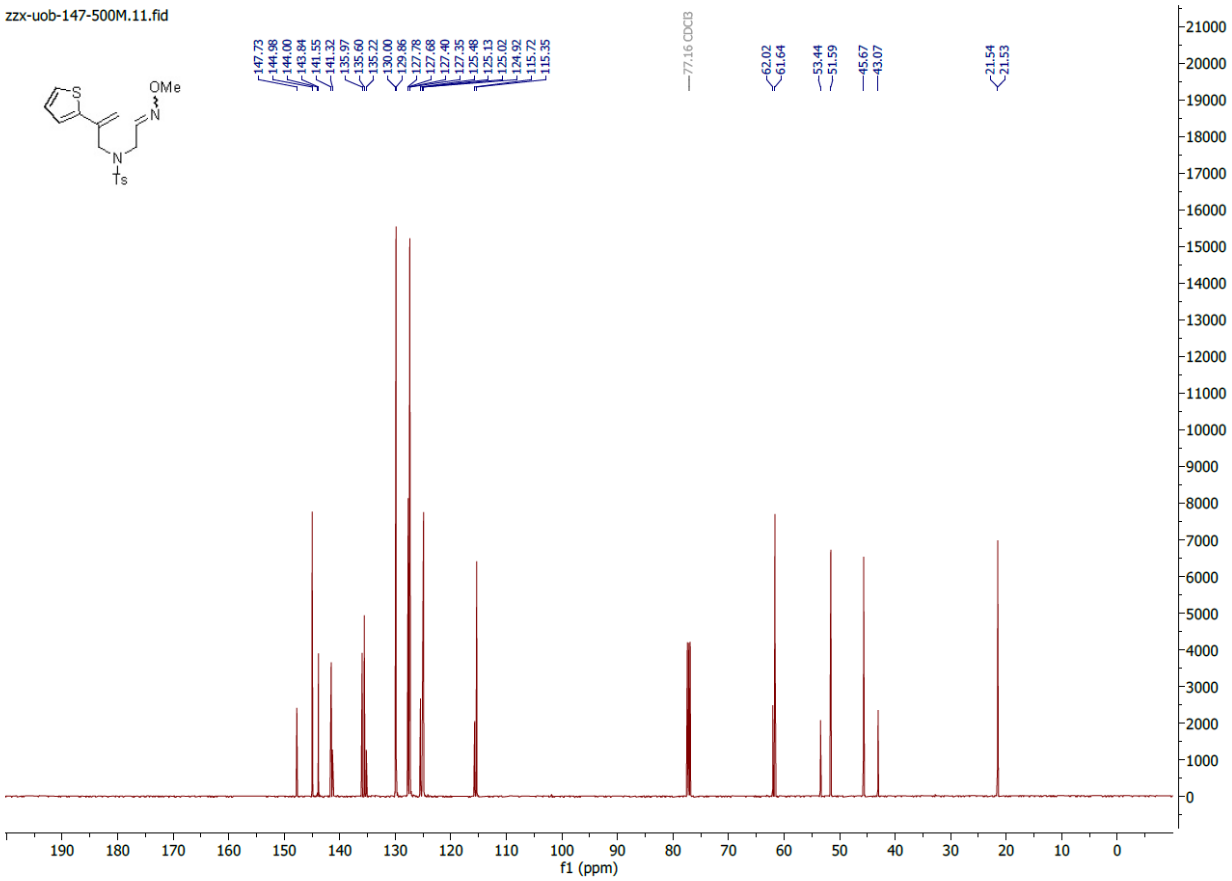

Compound S16a

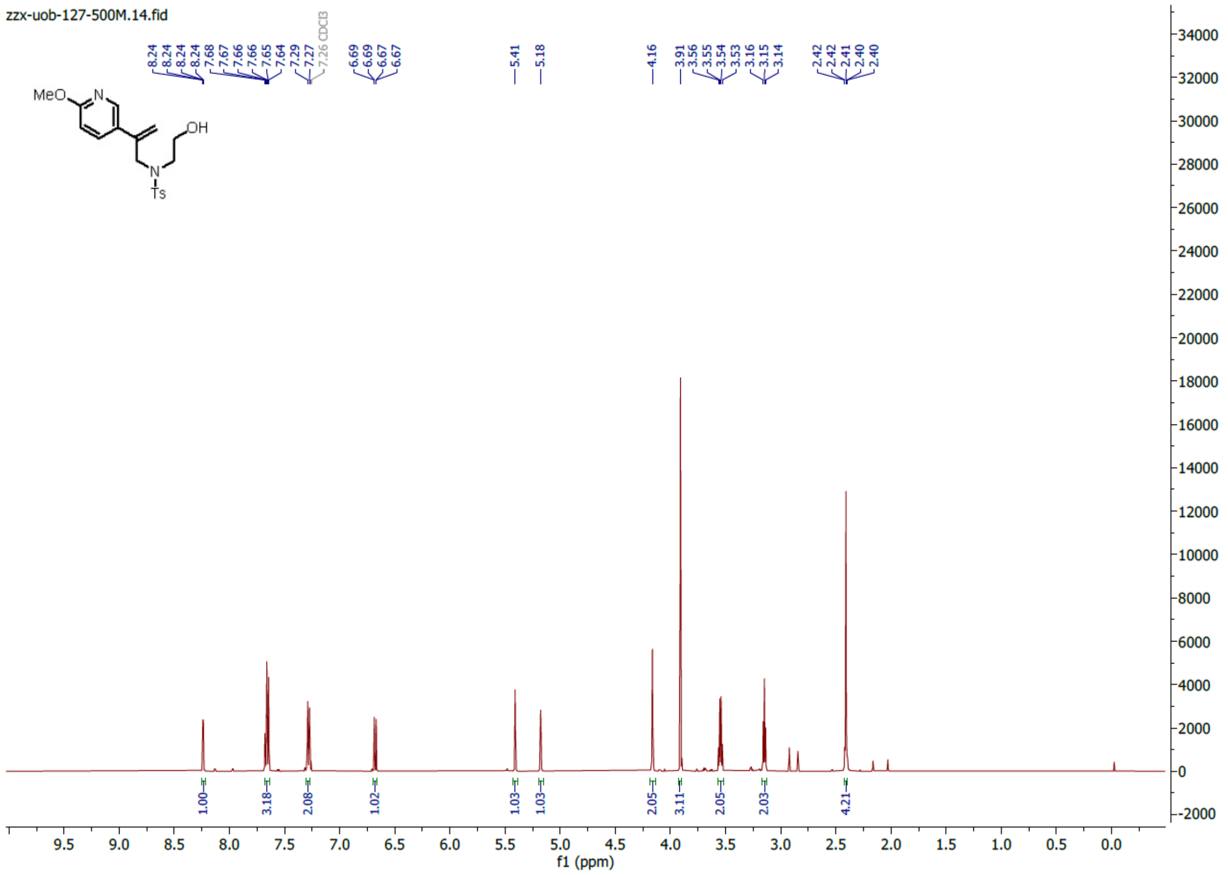

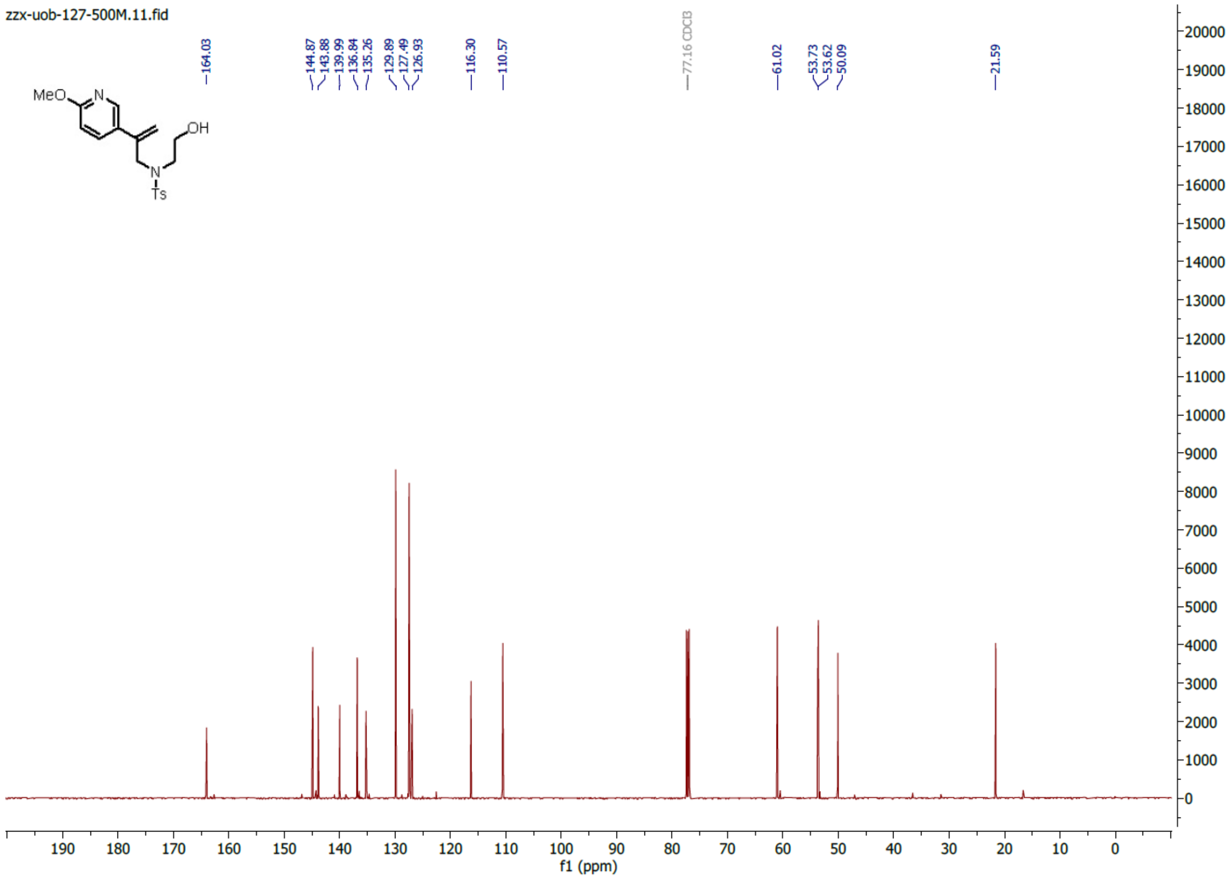

Compound S16b

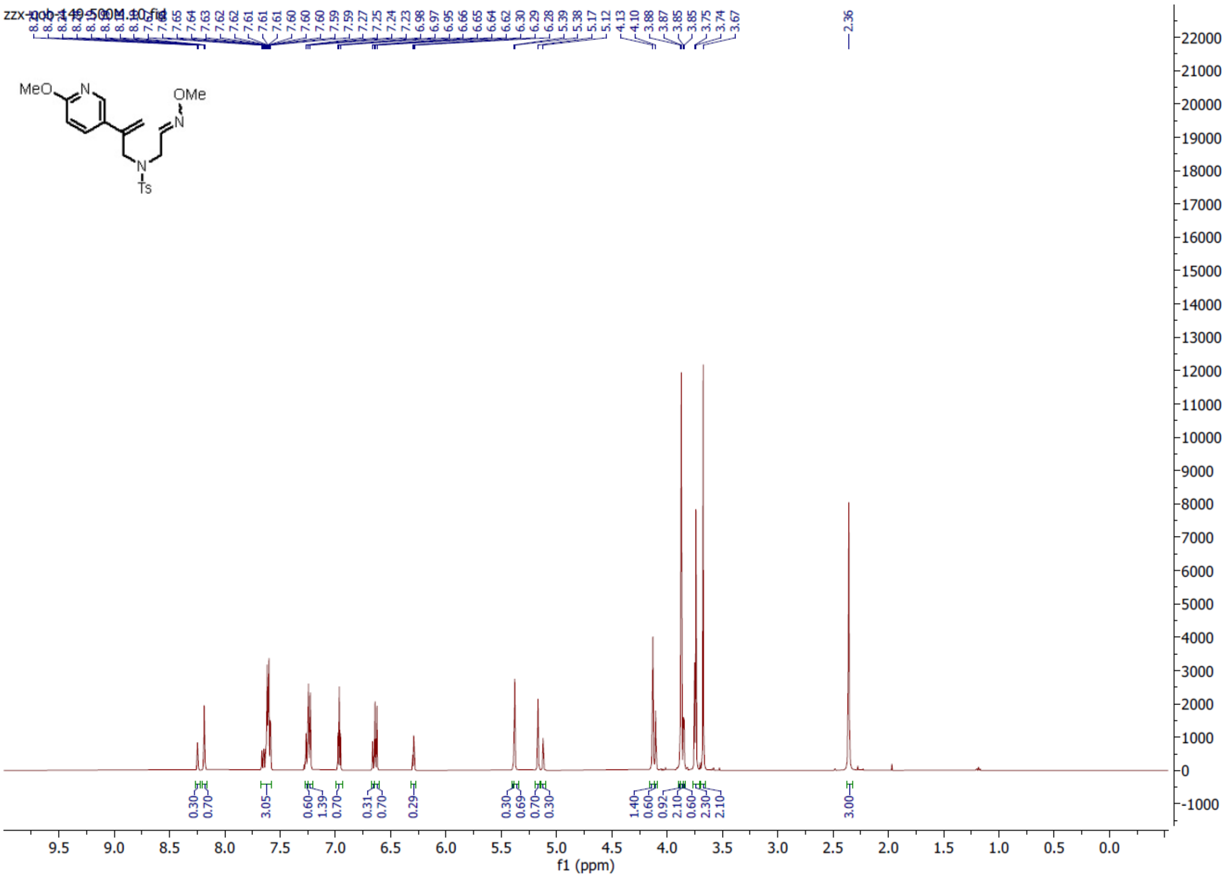

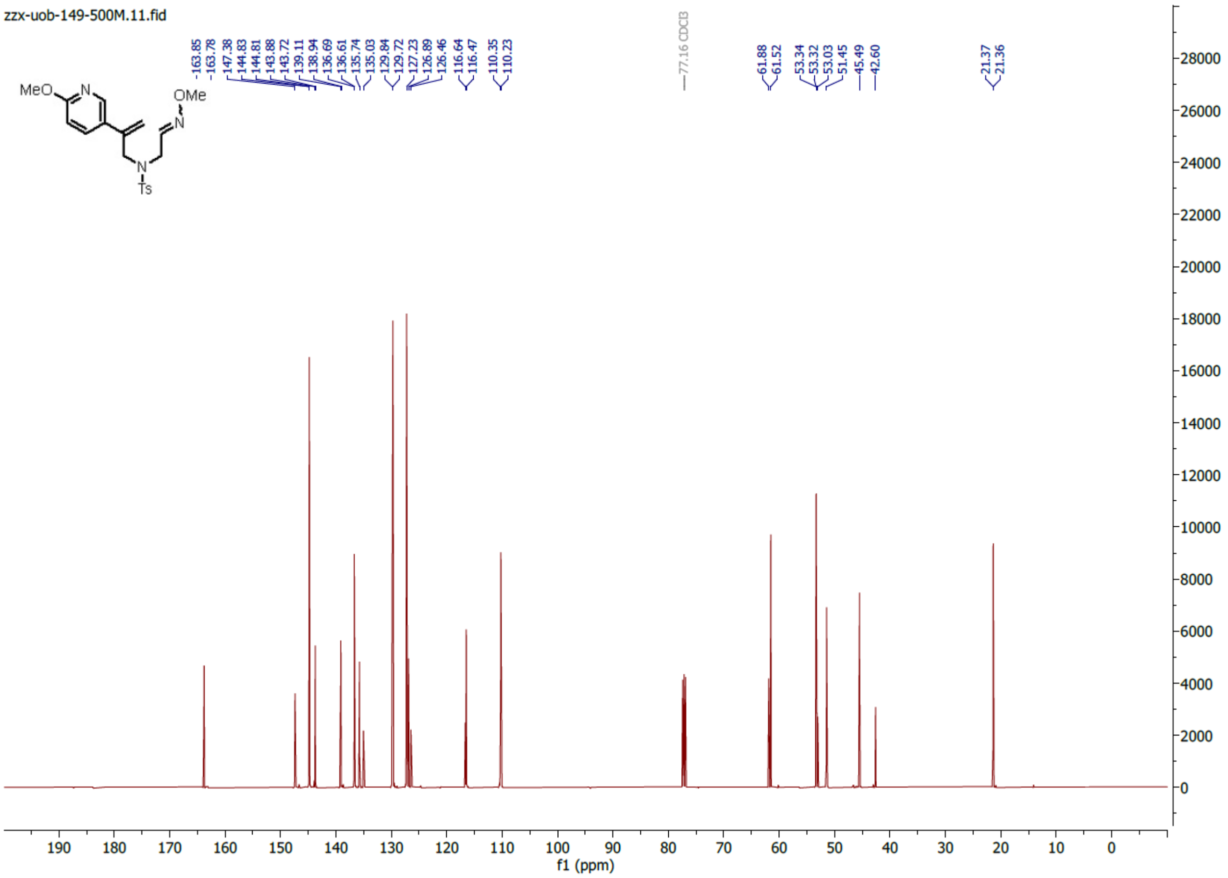

Compound S17a

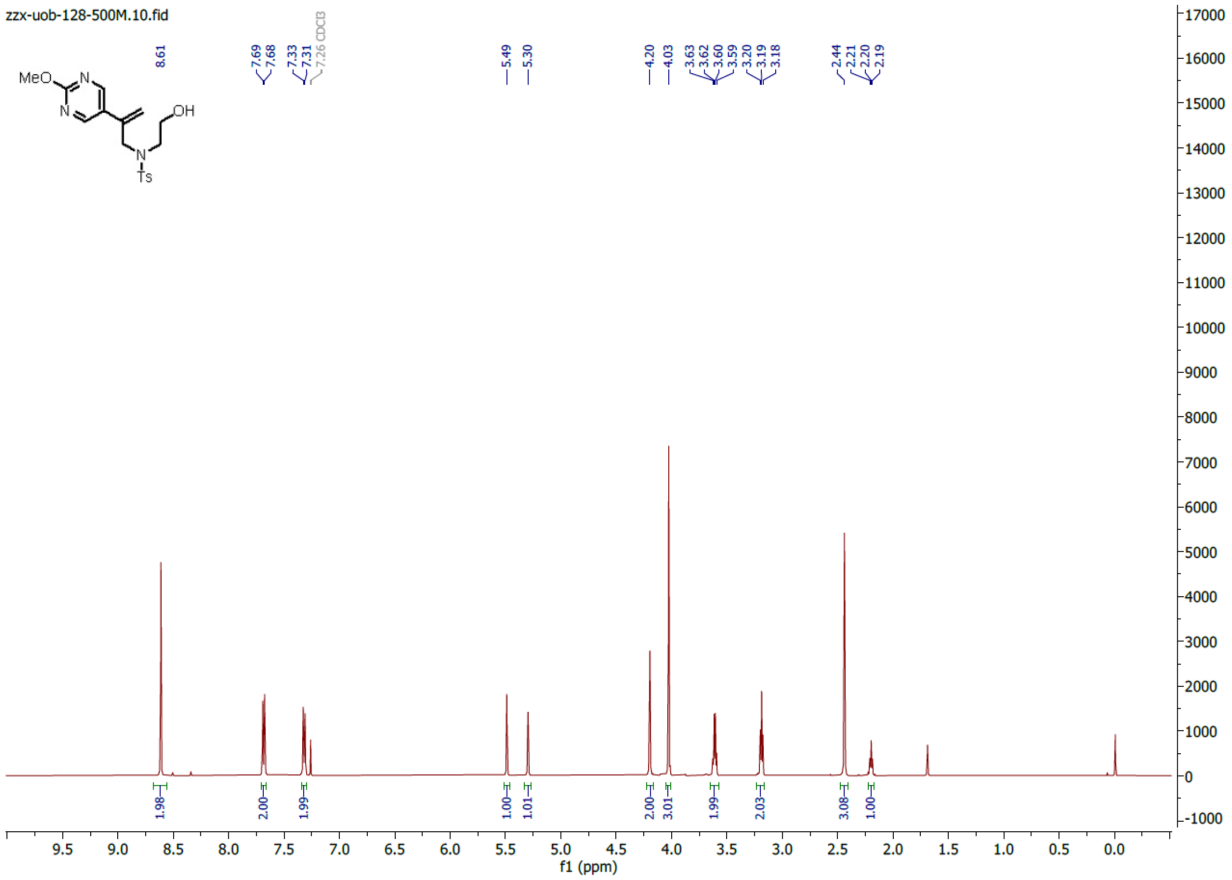

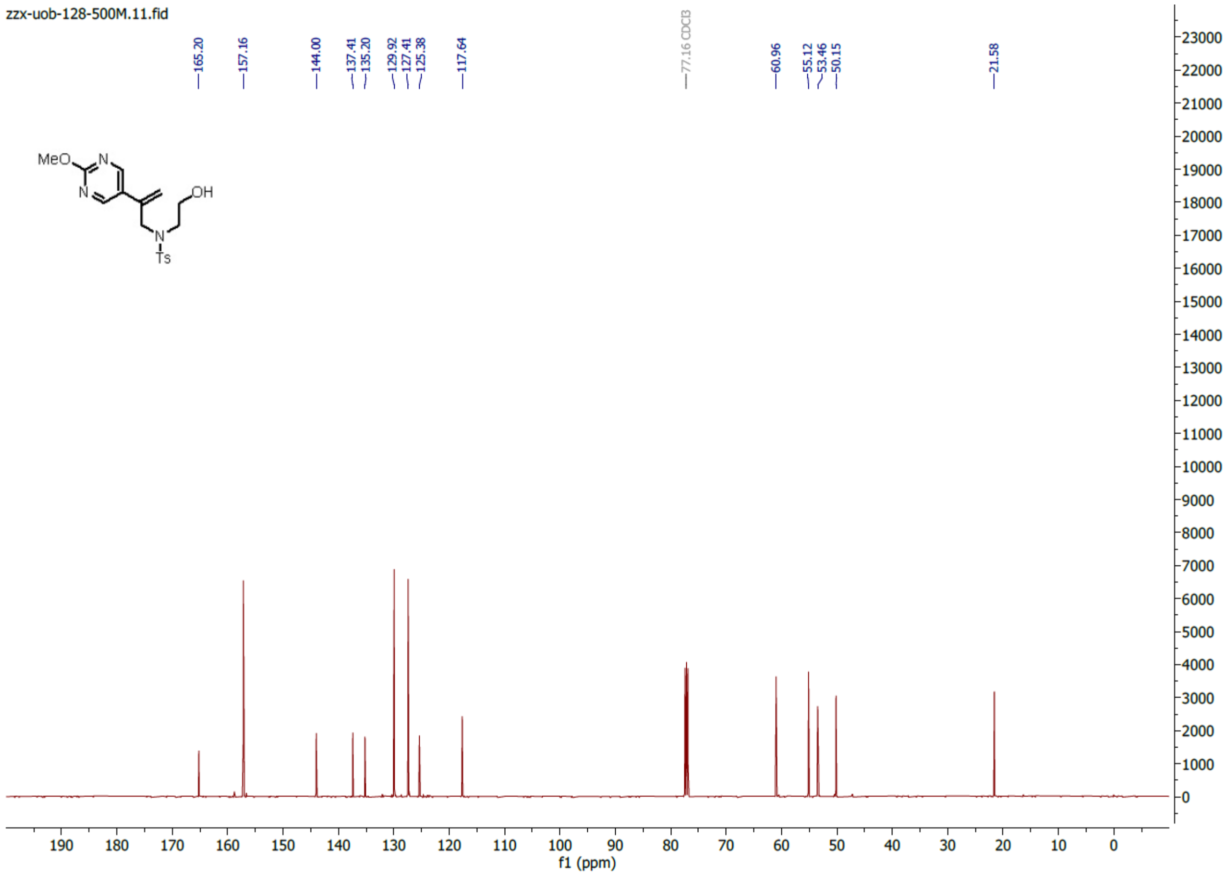

Compound S17b

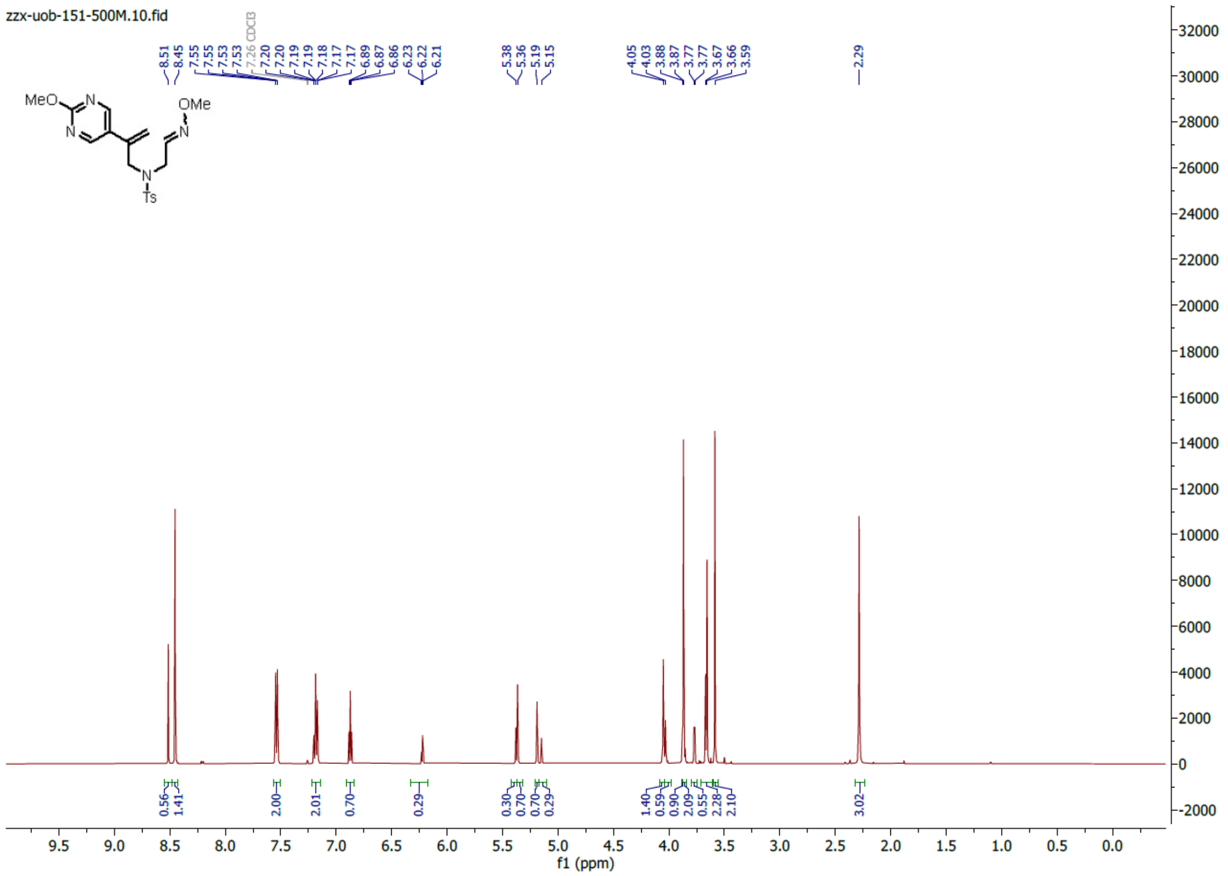

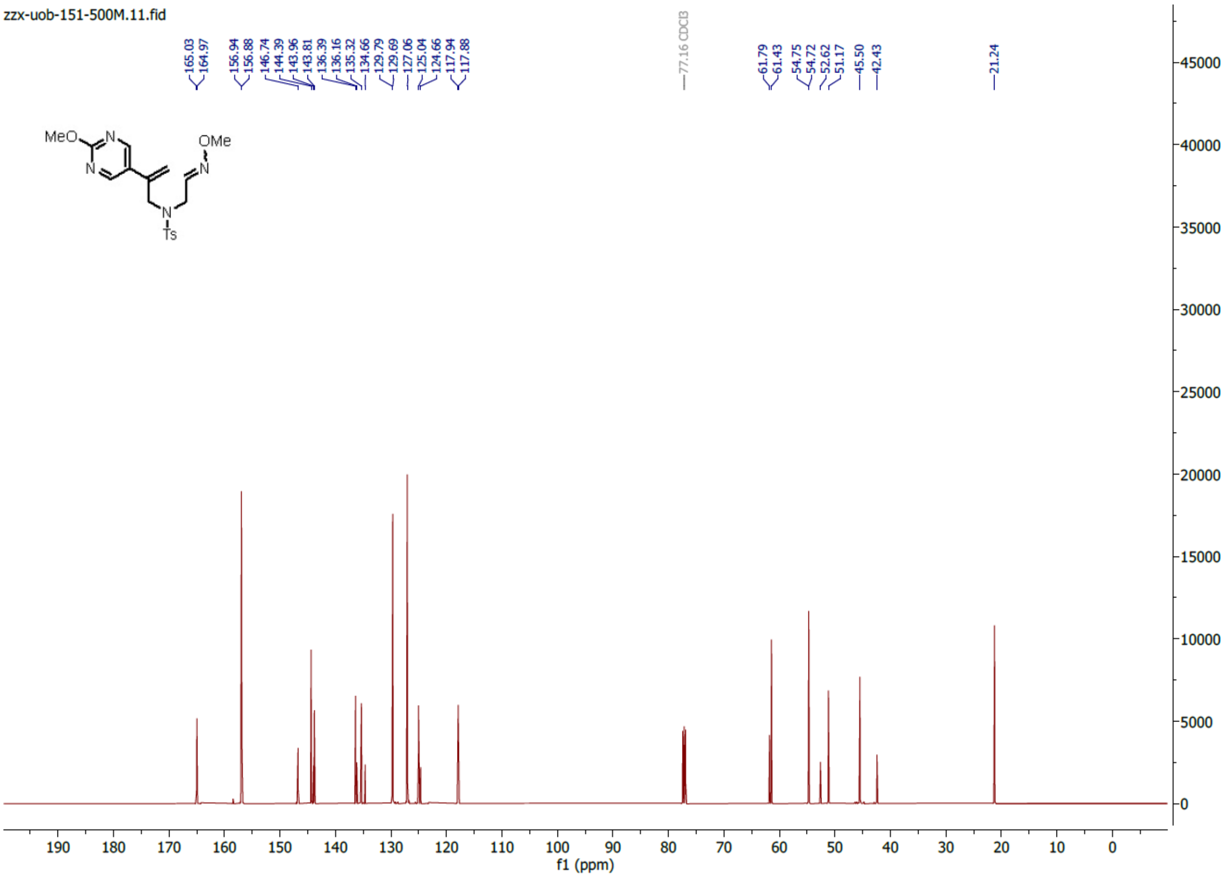

Compound S18a

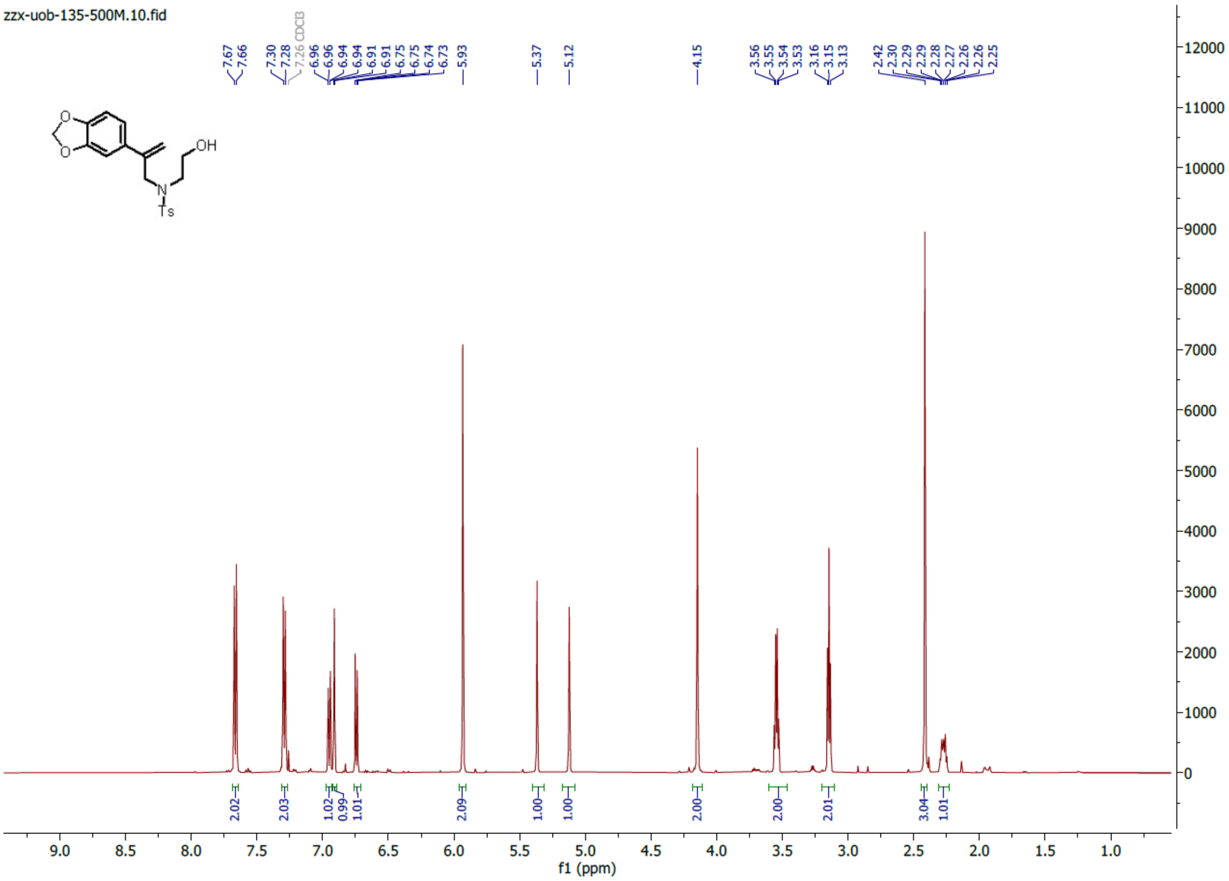

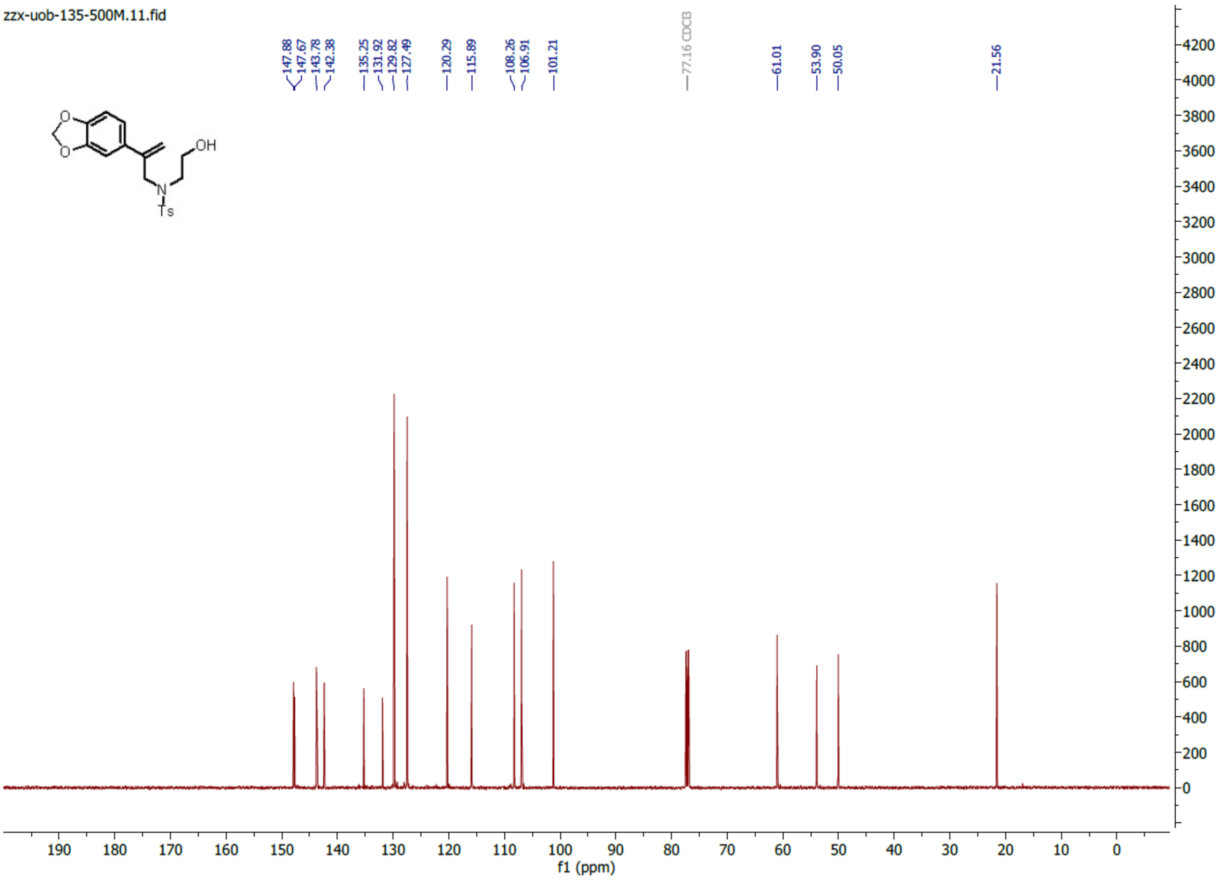

Compound S18b

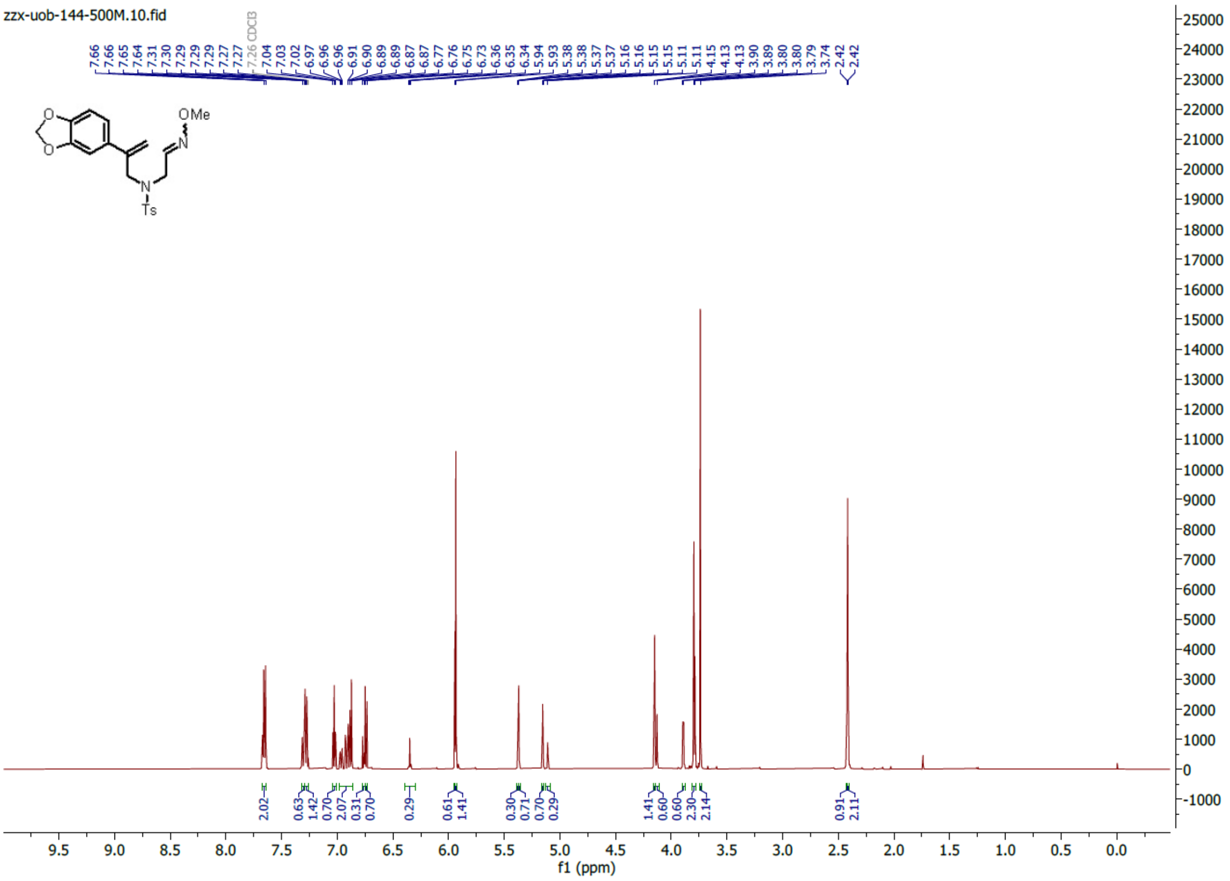

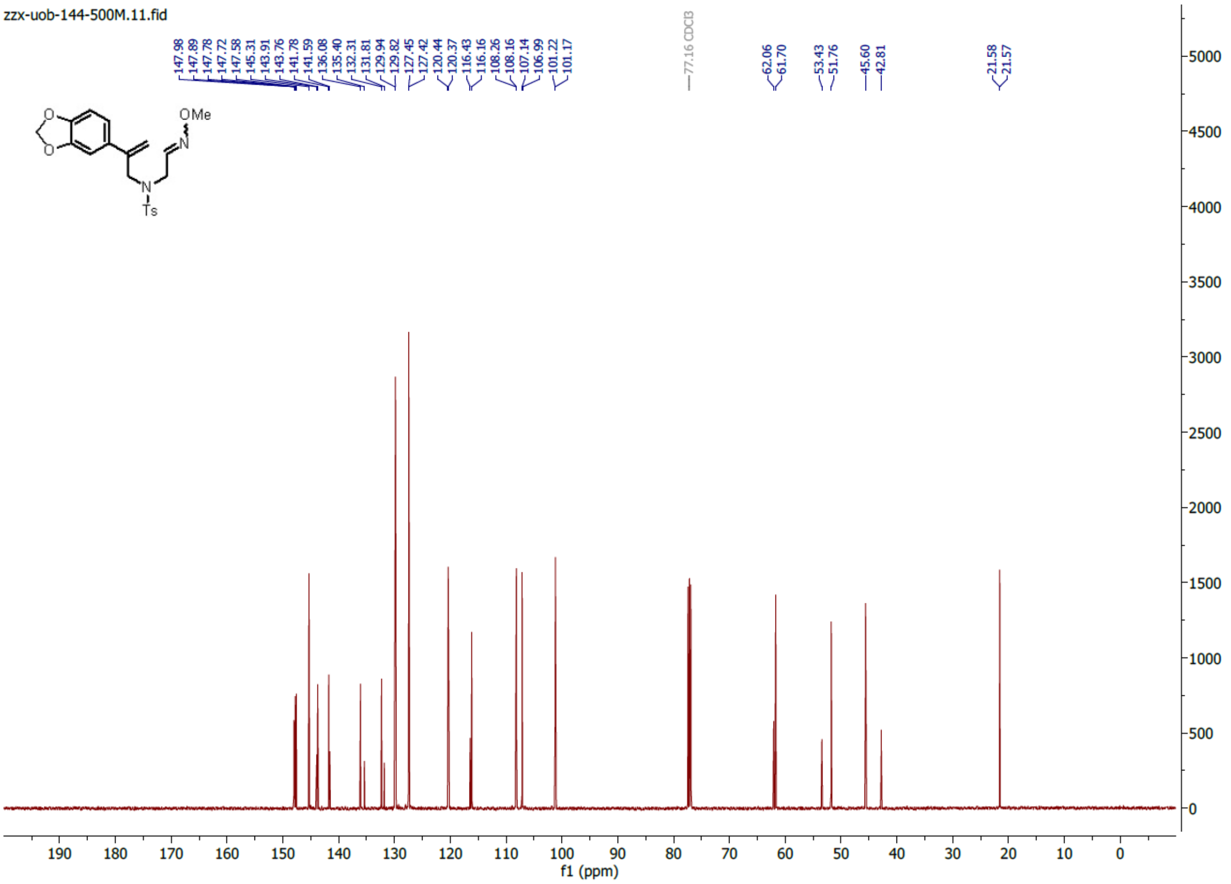

Compound S19a

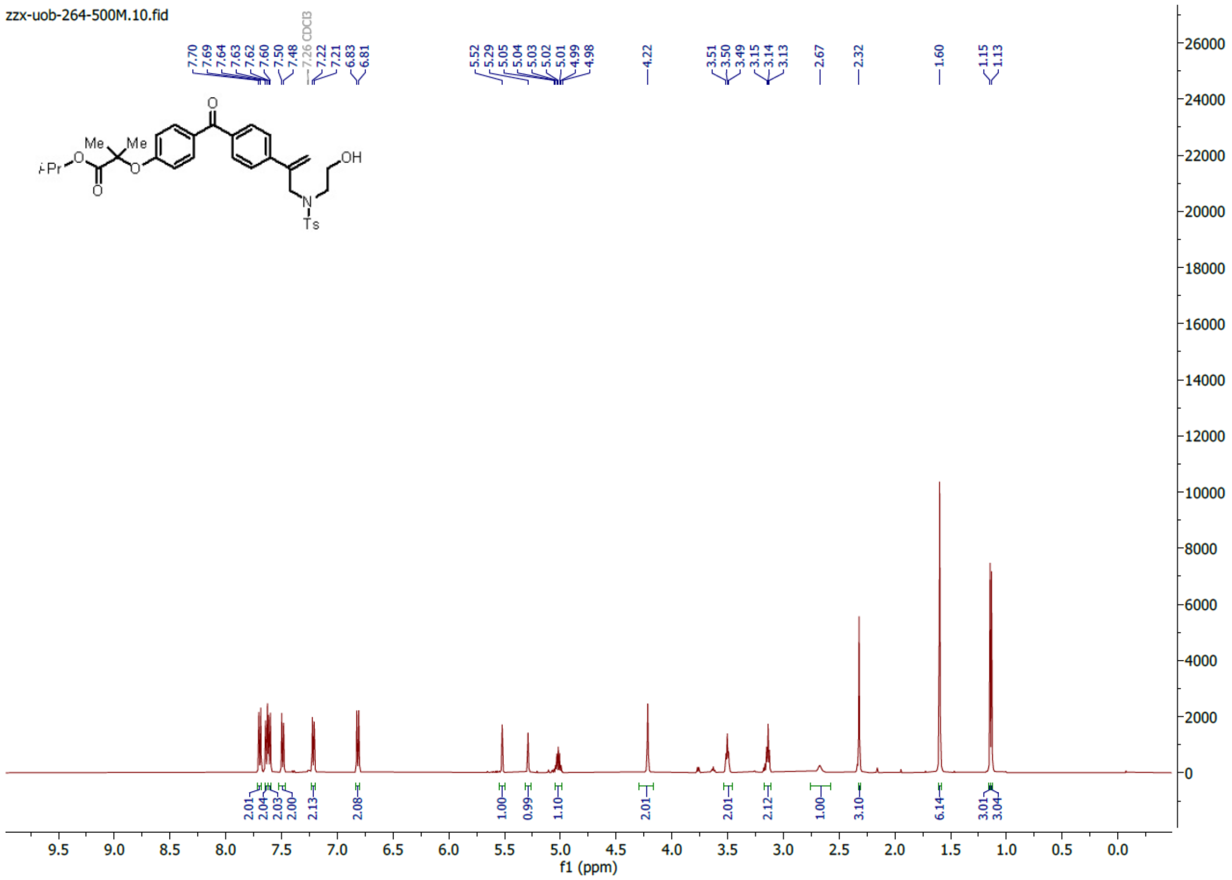

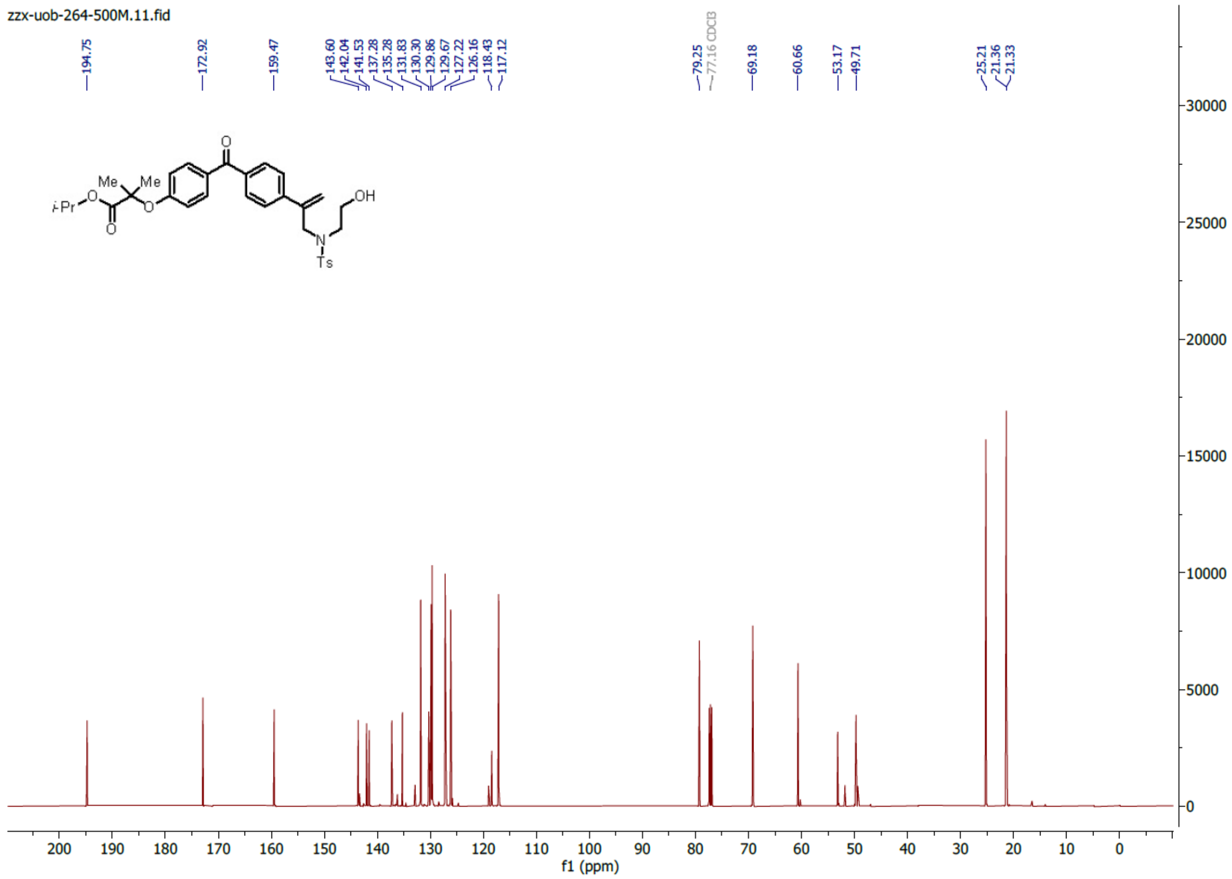

Compound S19b

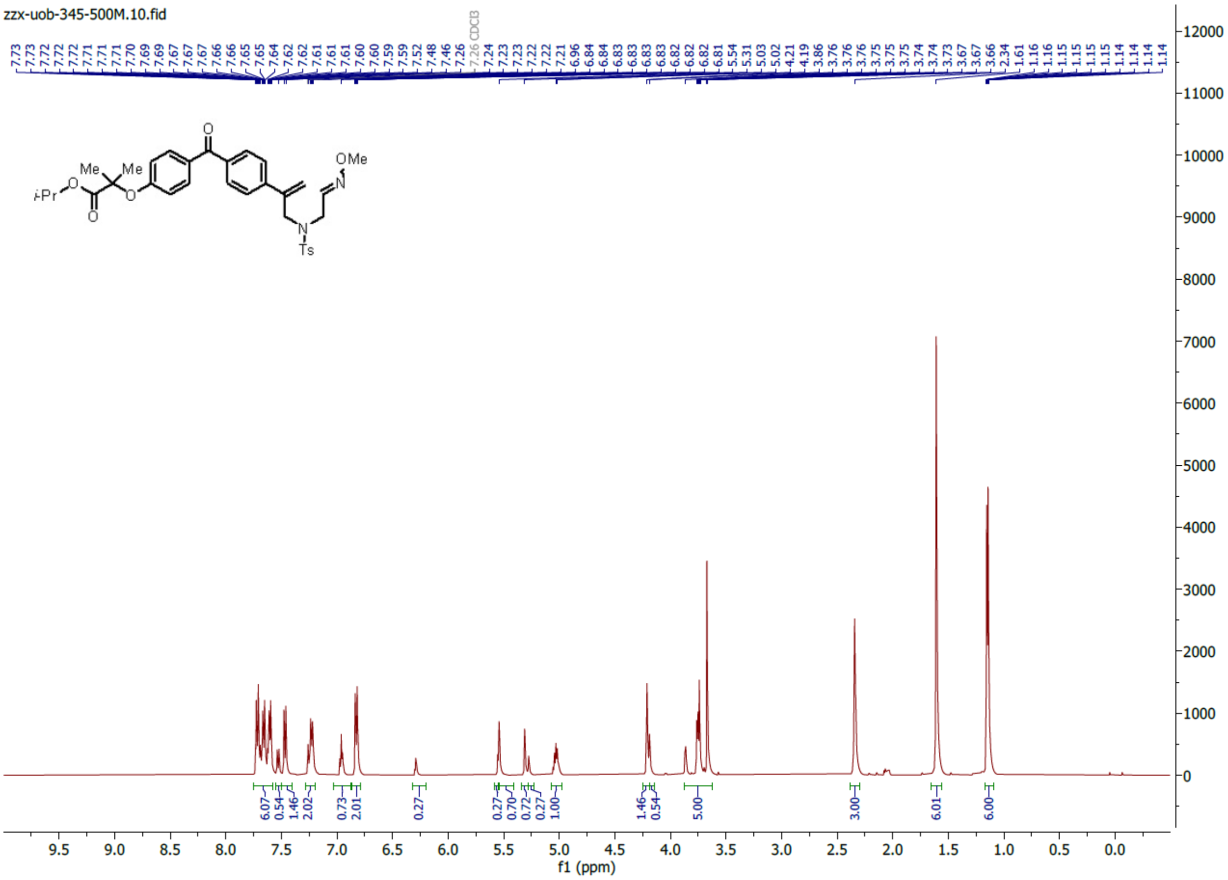

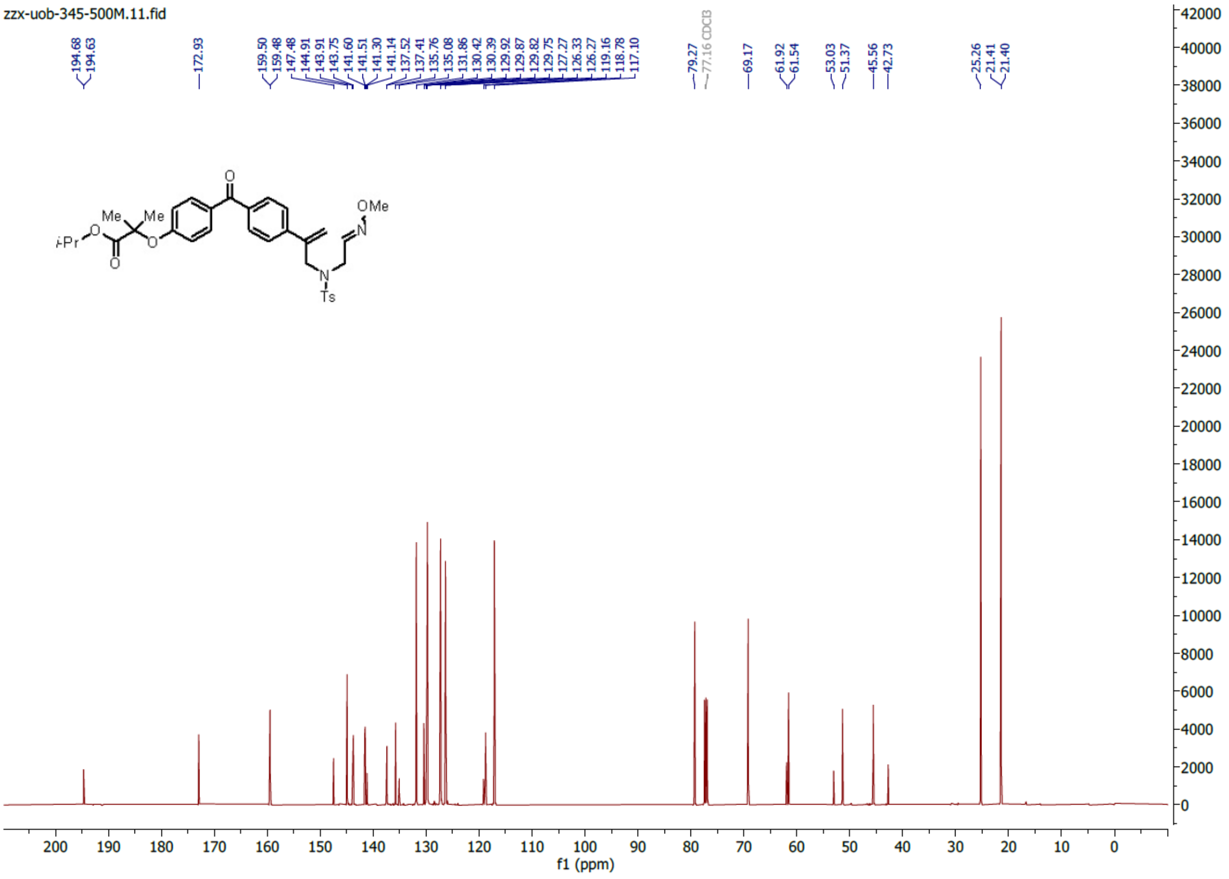

Compound S20a

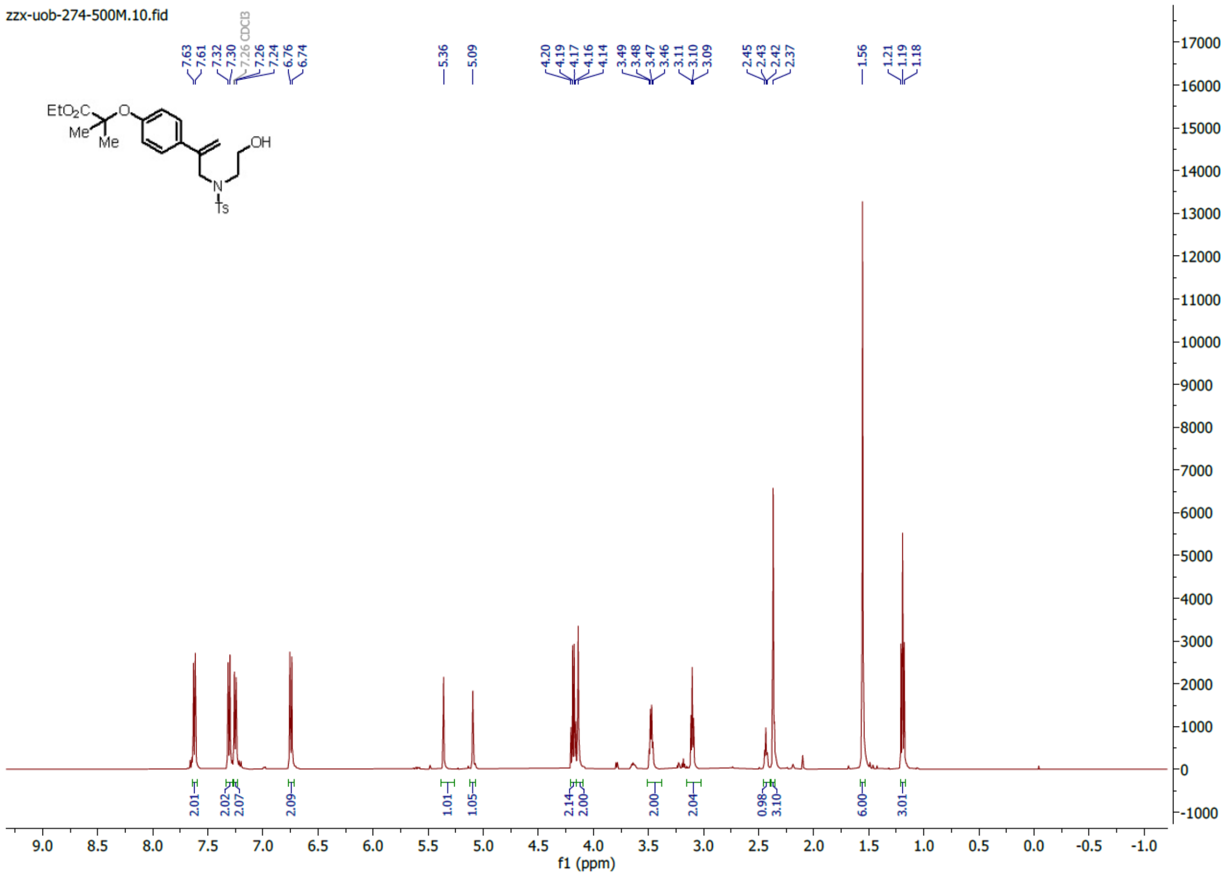

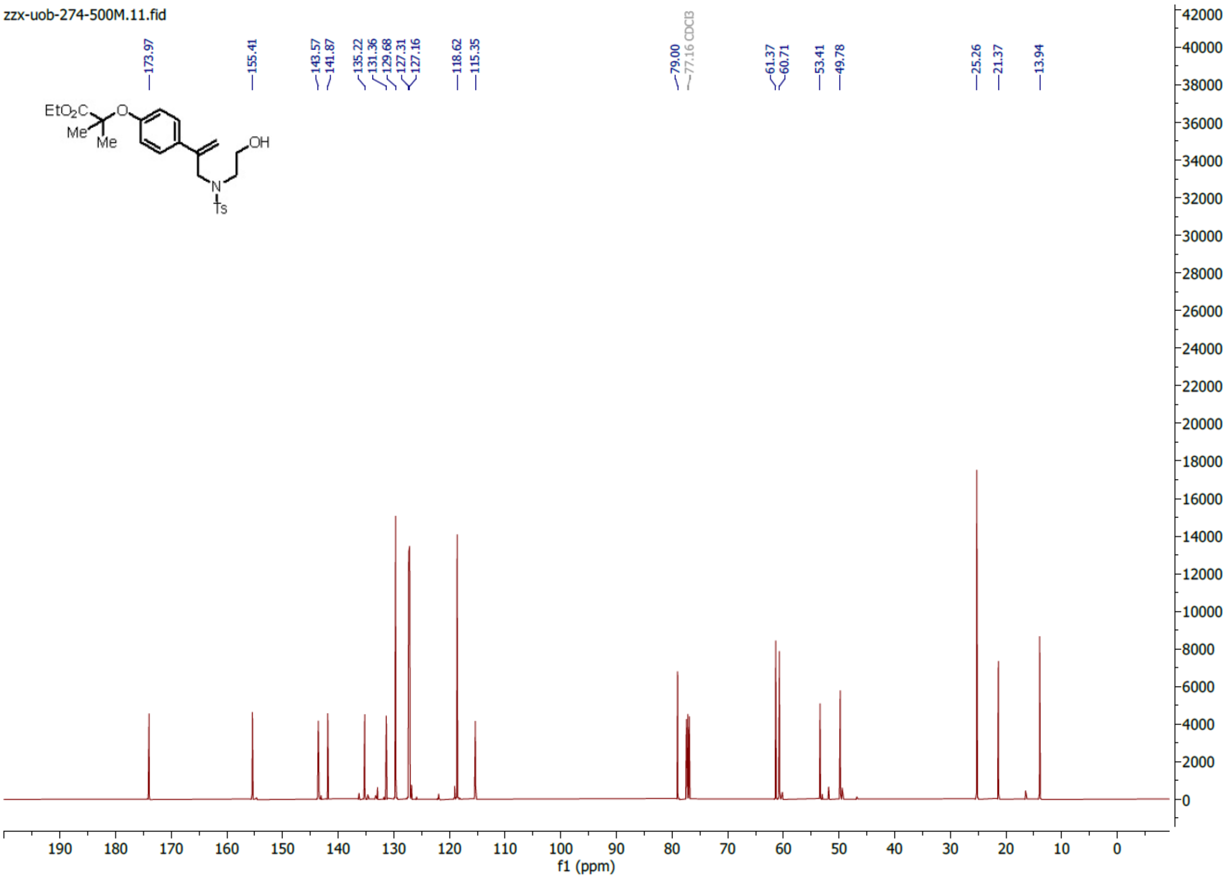

Compound S20b

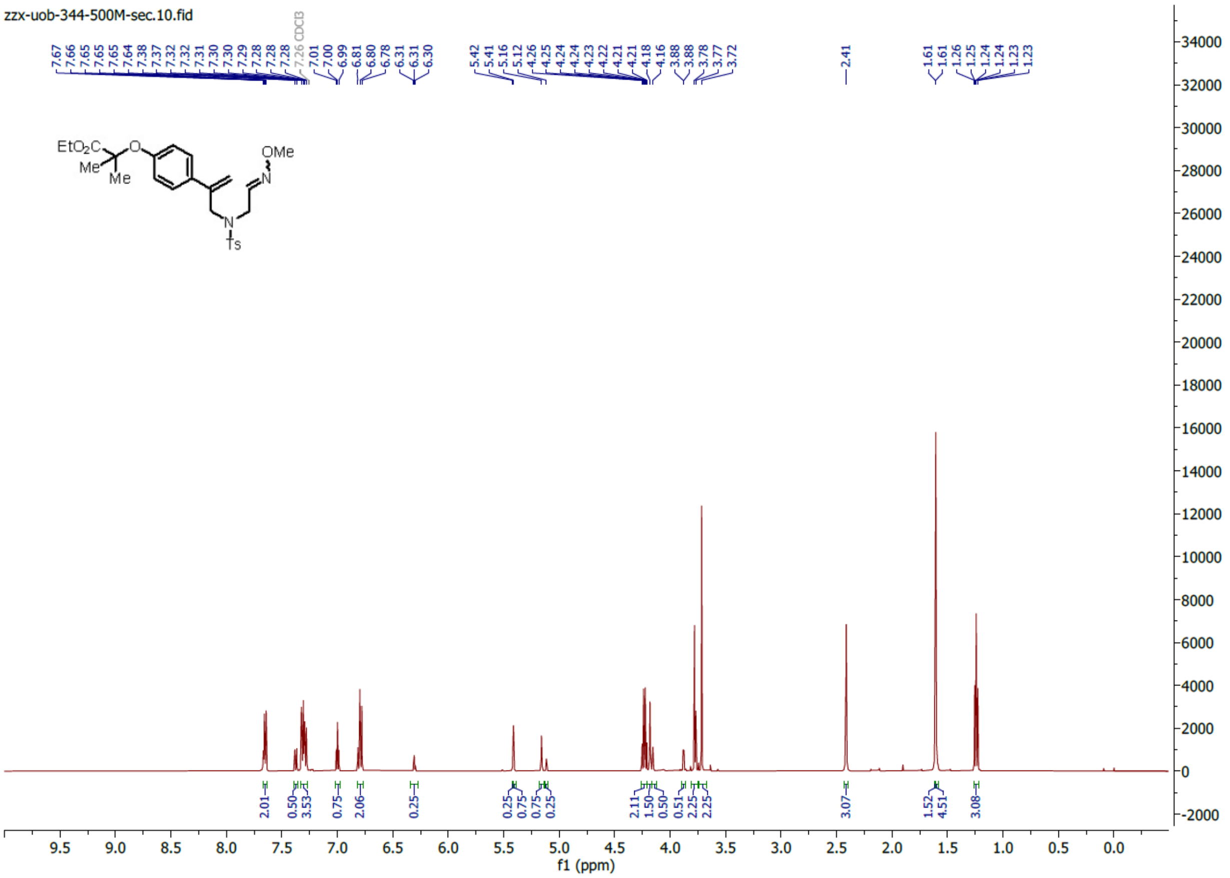

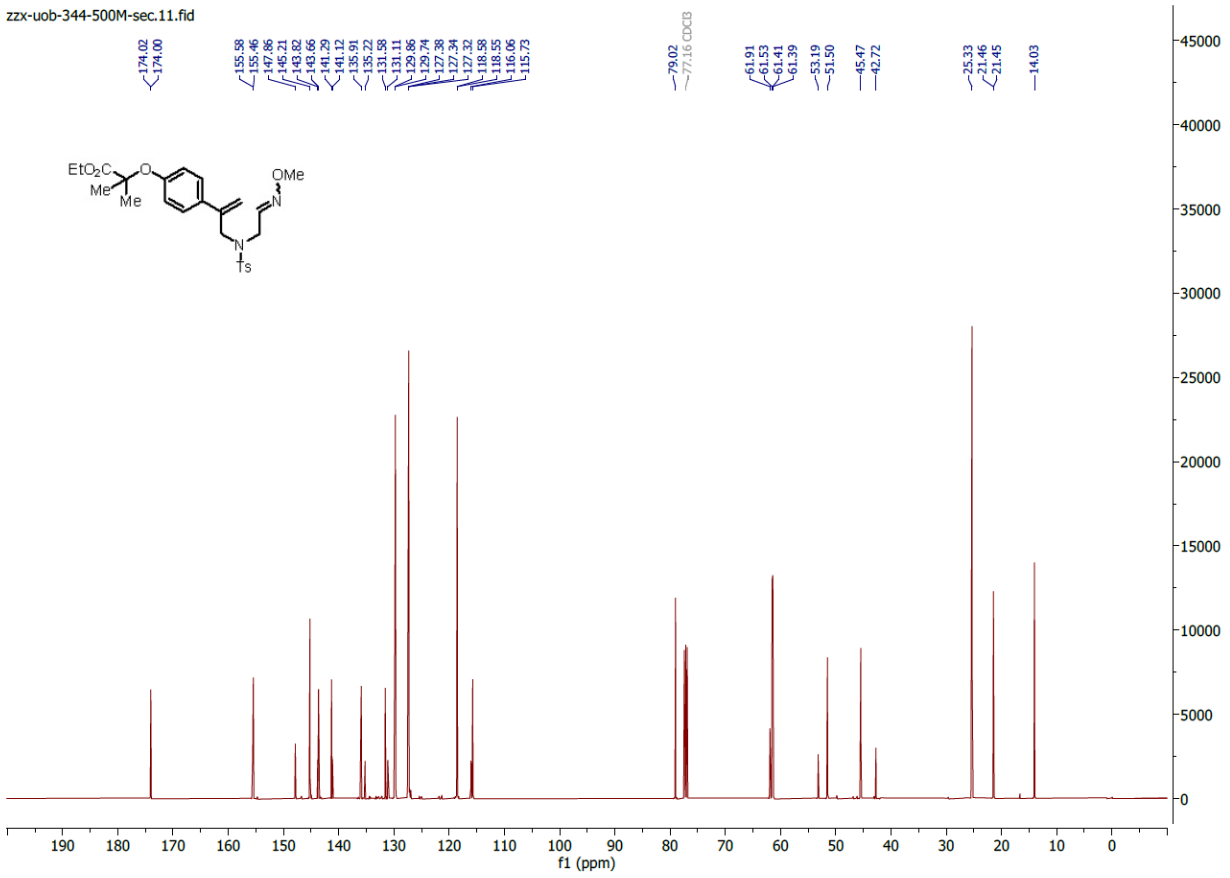

Compound S21a

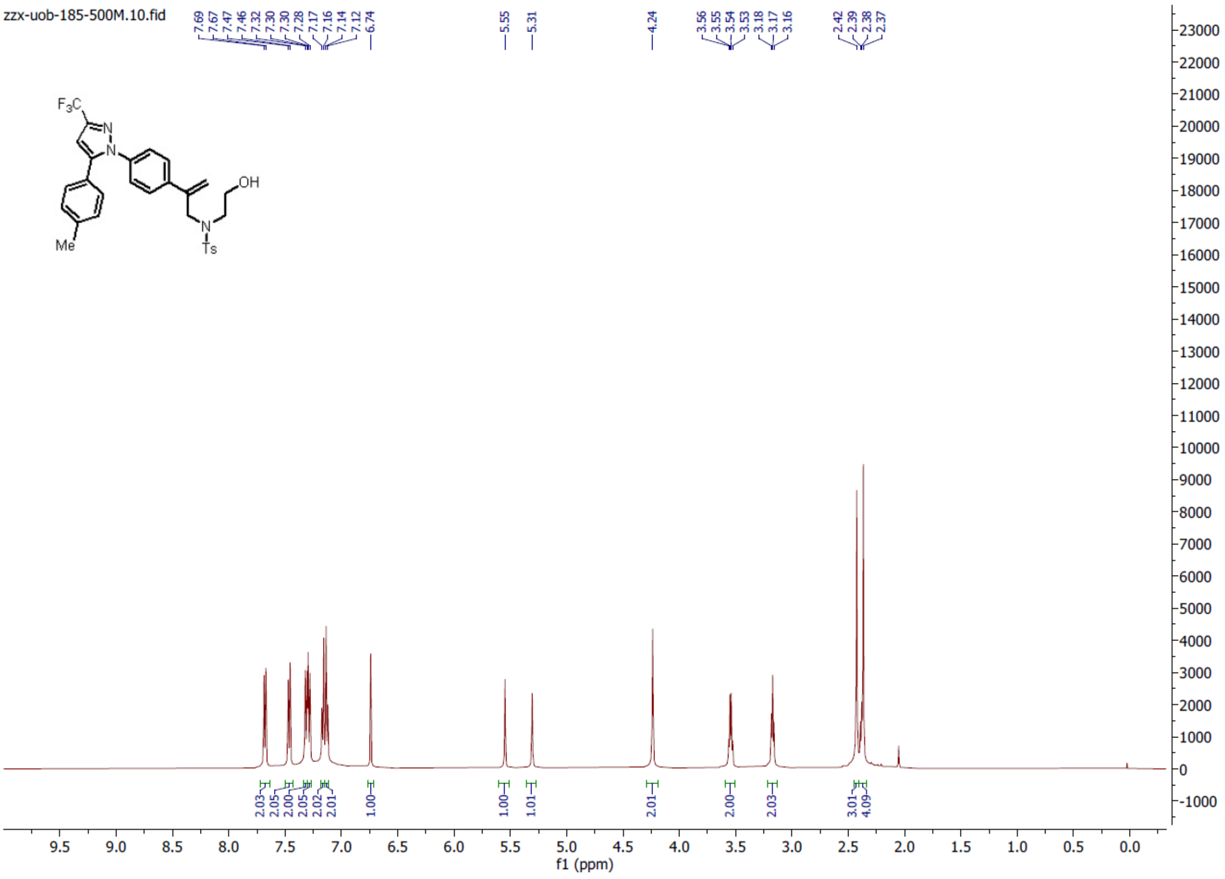

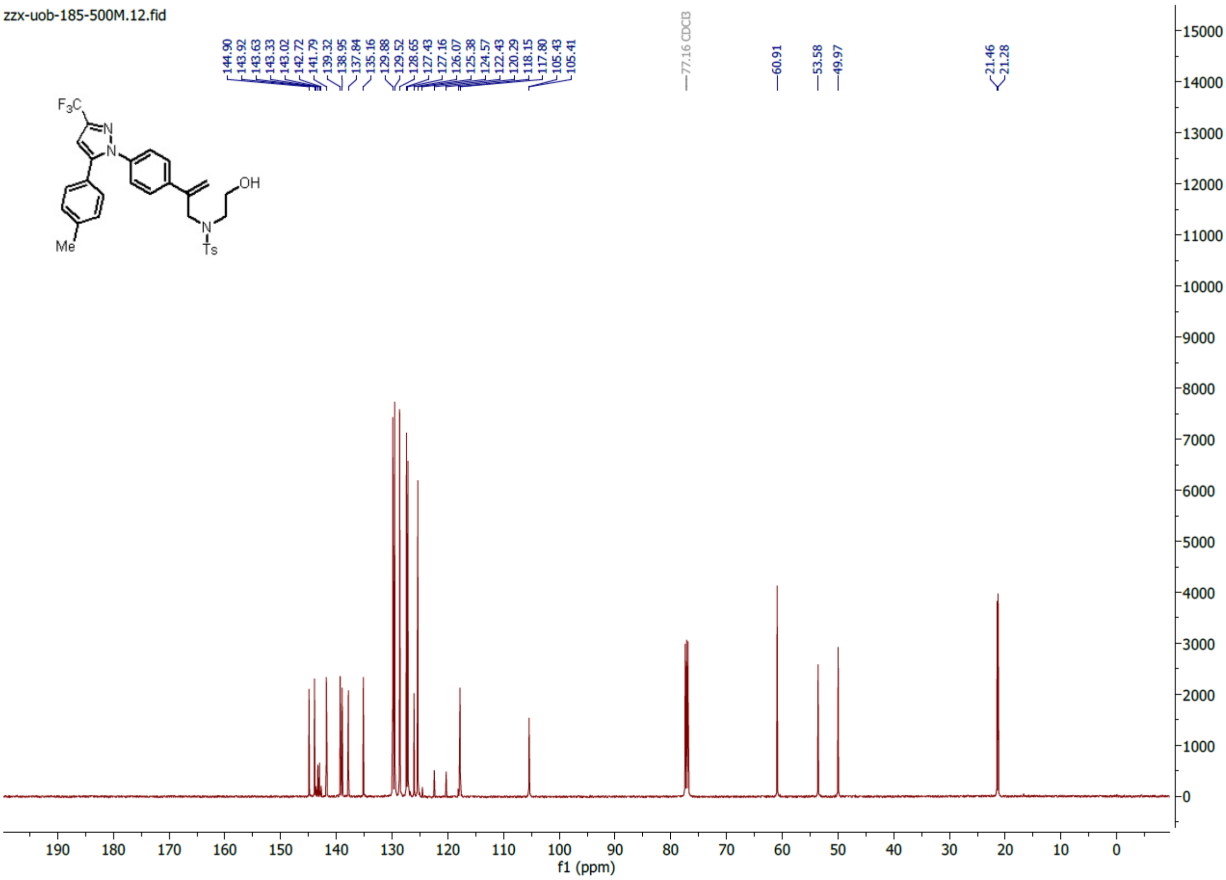

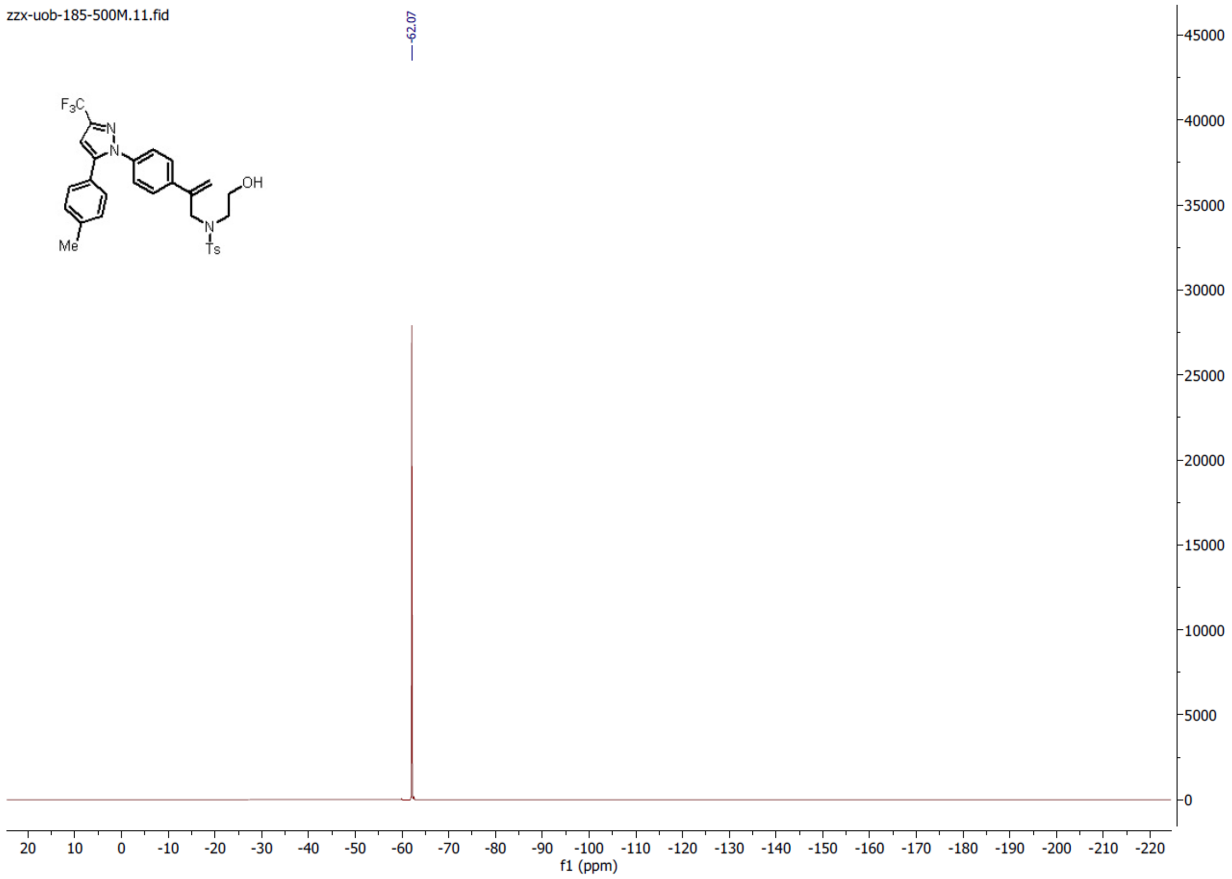
zzx-uob-185-500M.11.fid
-62.07
F3C
N
N
OH
N
Ts
Me
45000
40000
35000
30000
25000
20000
15000
10000
5000
0
20 10 0 -10 -20 -30 -40 -50 -60 -70 -80 -90 -100 -110 -120 -130 -140 -150 -160 -170 -180 -190 -200 -210 -220
f1 (ppm)

Compound S21b

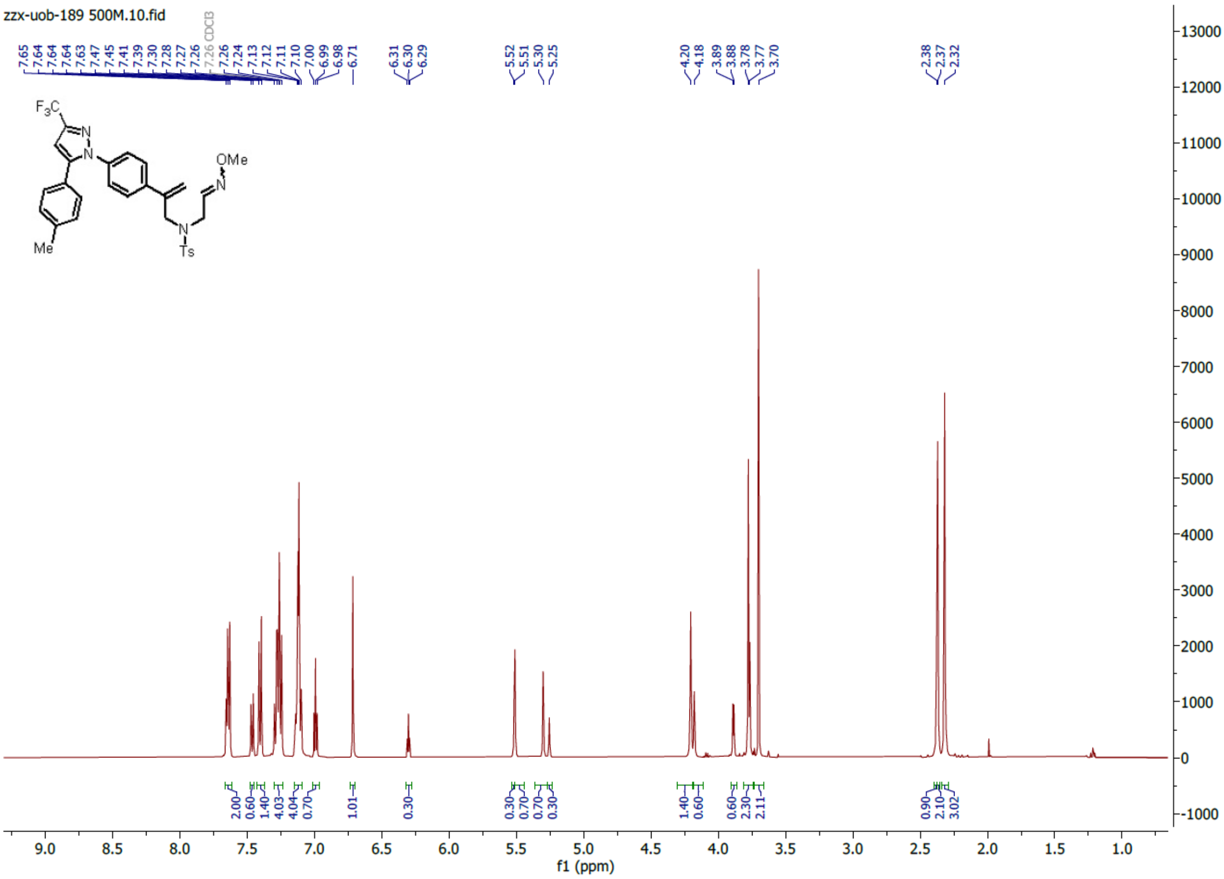

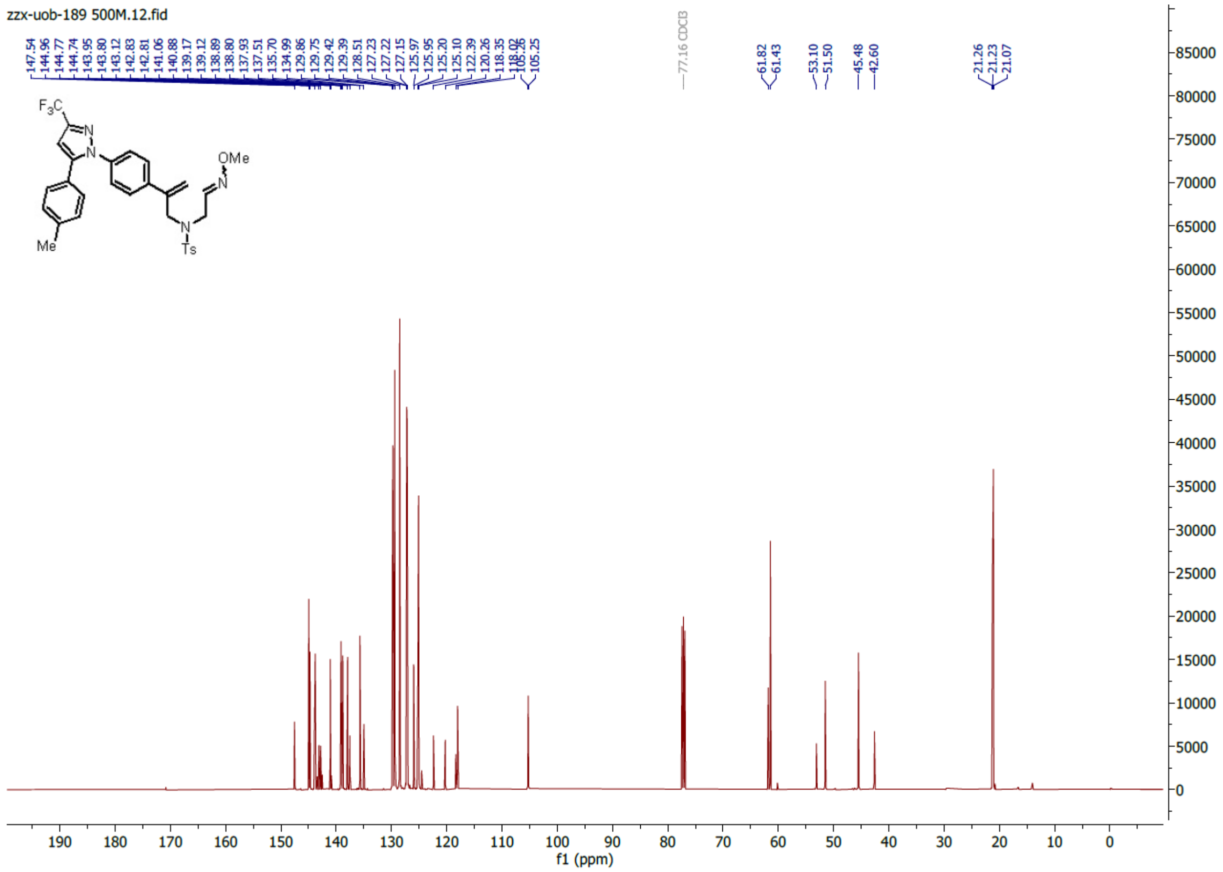

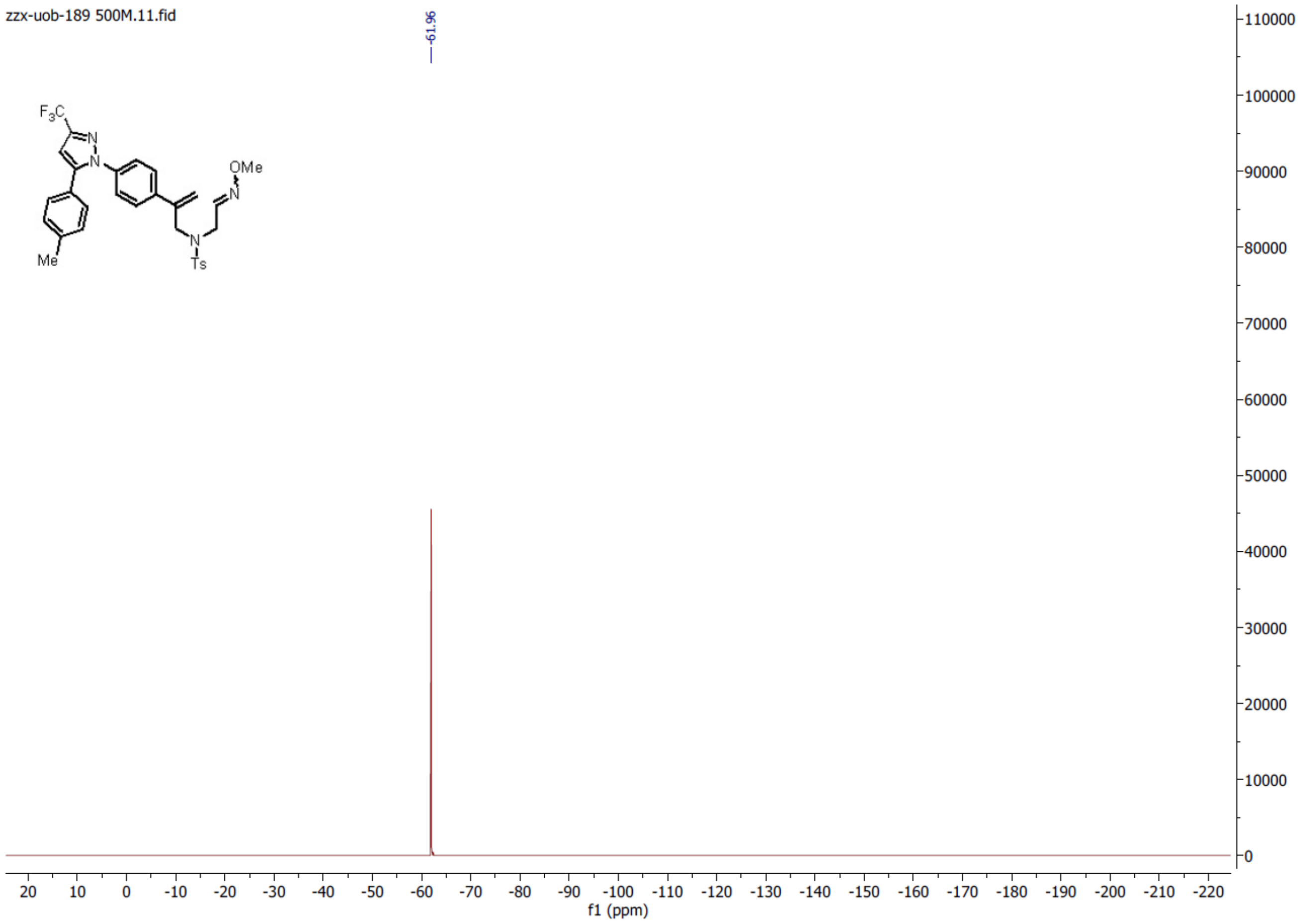
zzx-uob-189 500M.11.fid
-61.96
F3C
N
N
OMe
N
N
Ts
Me
110000
100000
90000
80000
70000
60000
50000
40000
30000
20000
10000
0
20 10 0 -10 -20 -30 -40 -50 -60 -70 -80 -90 -100 -110 -120 -130 -140 -150 -160 -170 -180 -190 -200 -210 -220
f1 (ppm)

Compound S22a

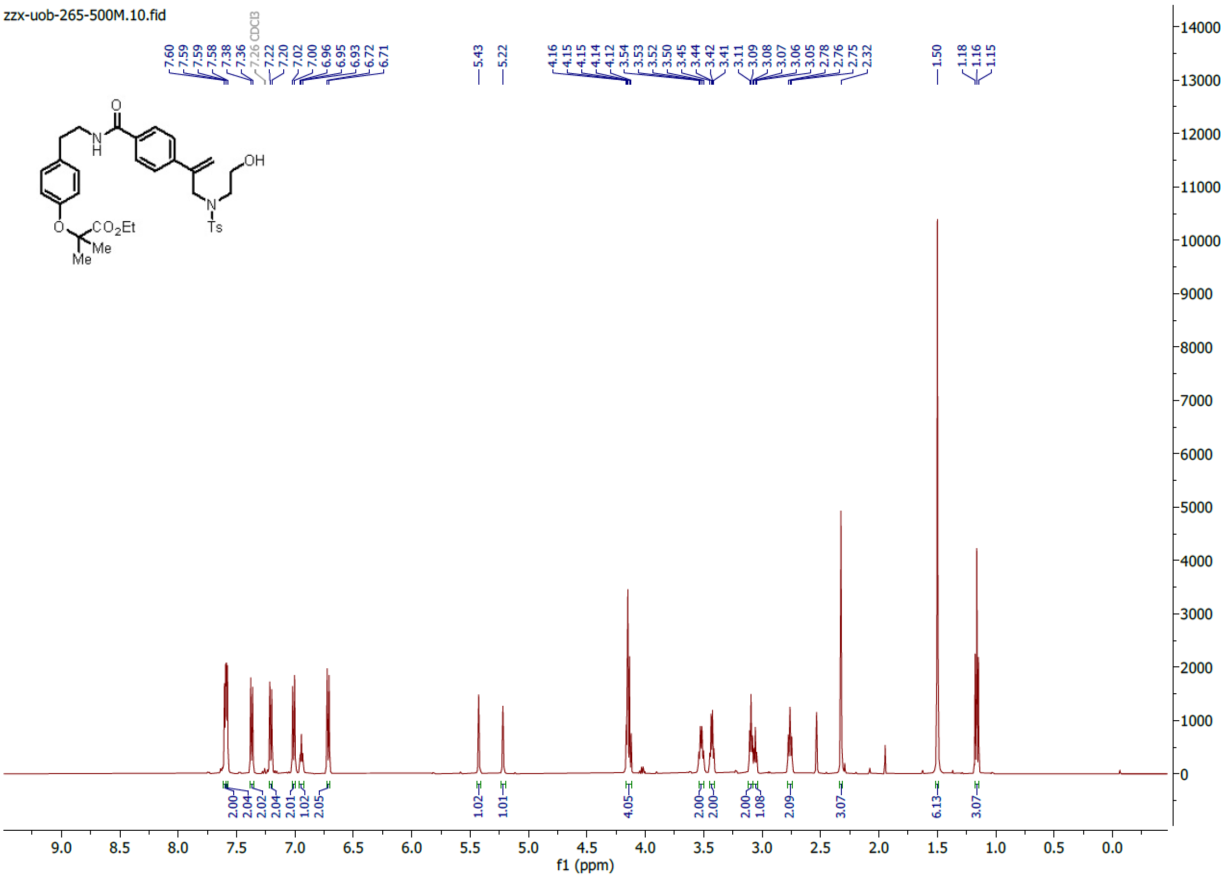

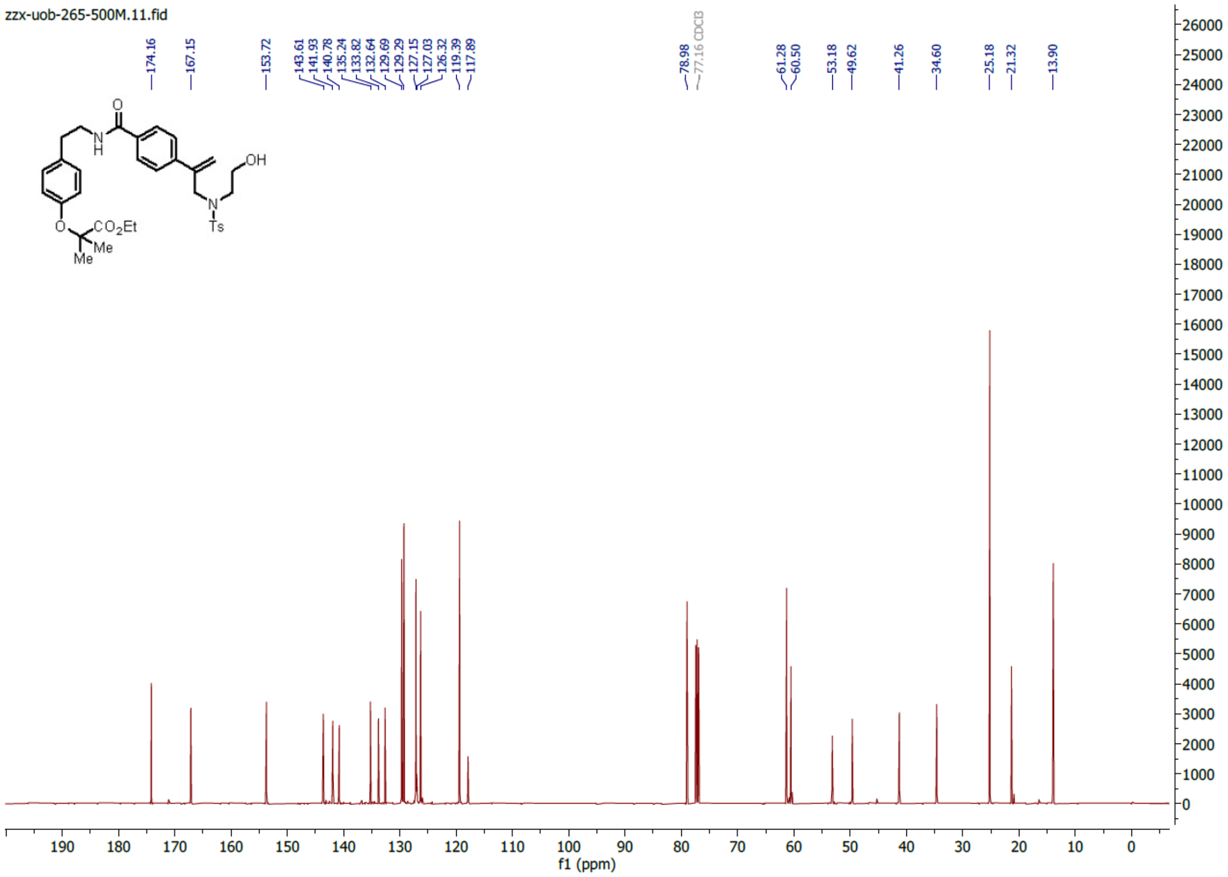

Compound S22b

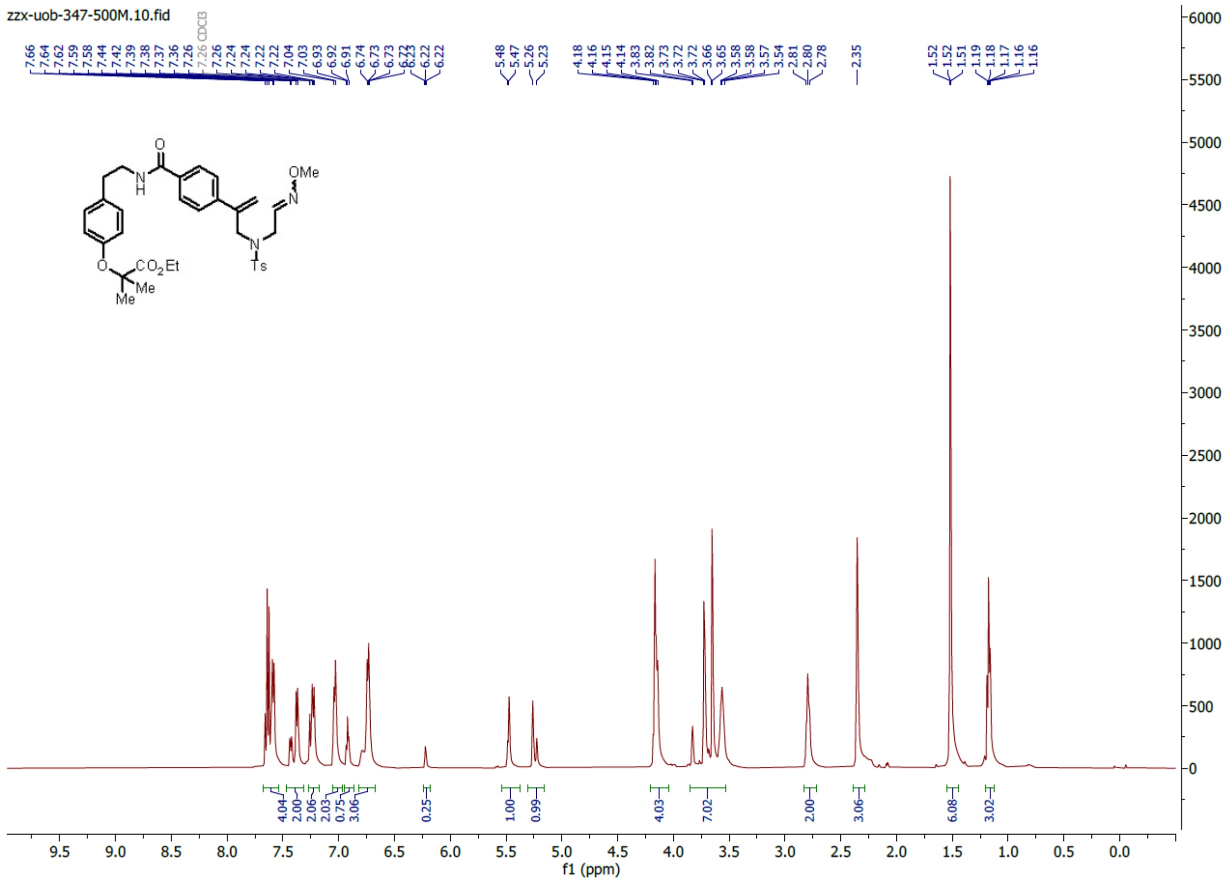

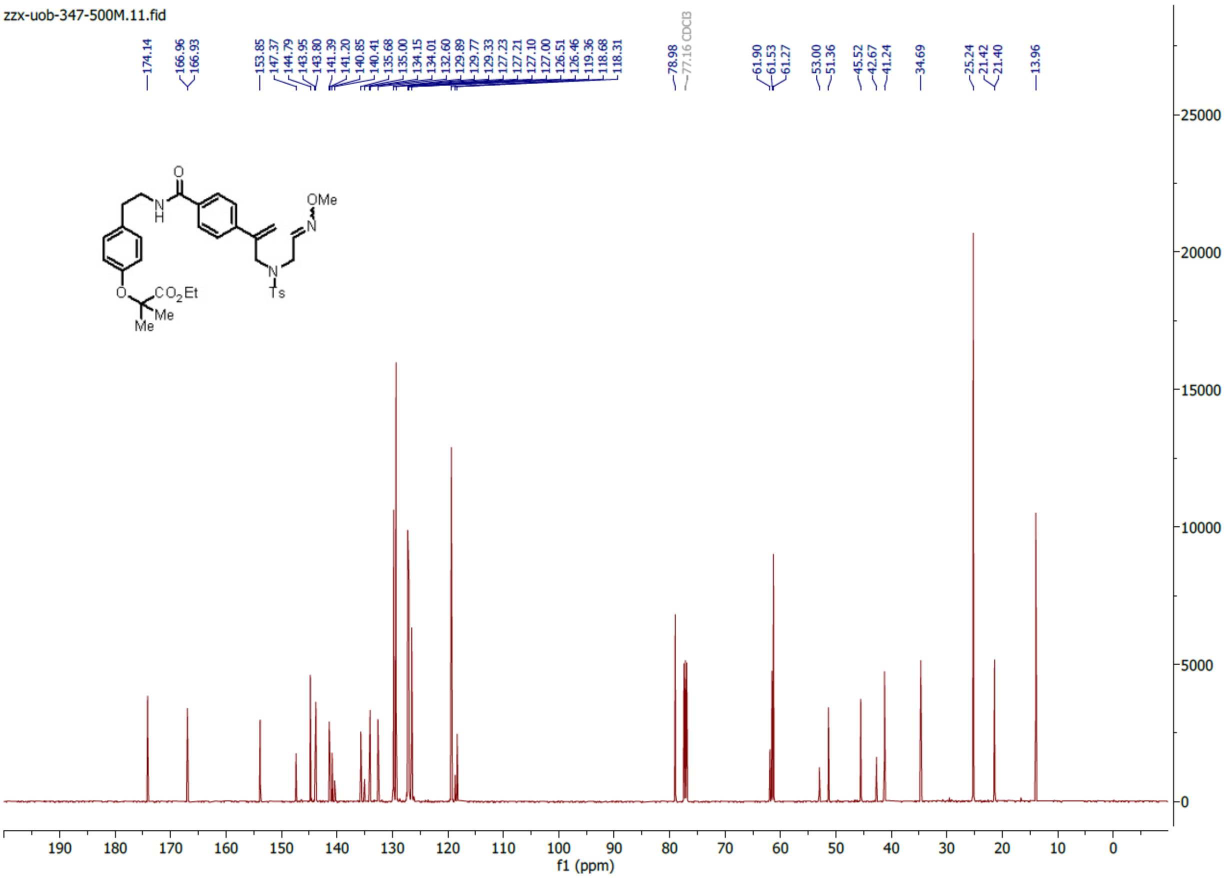

Compound S23a

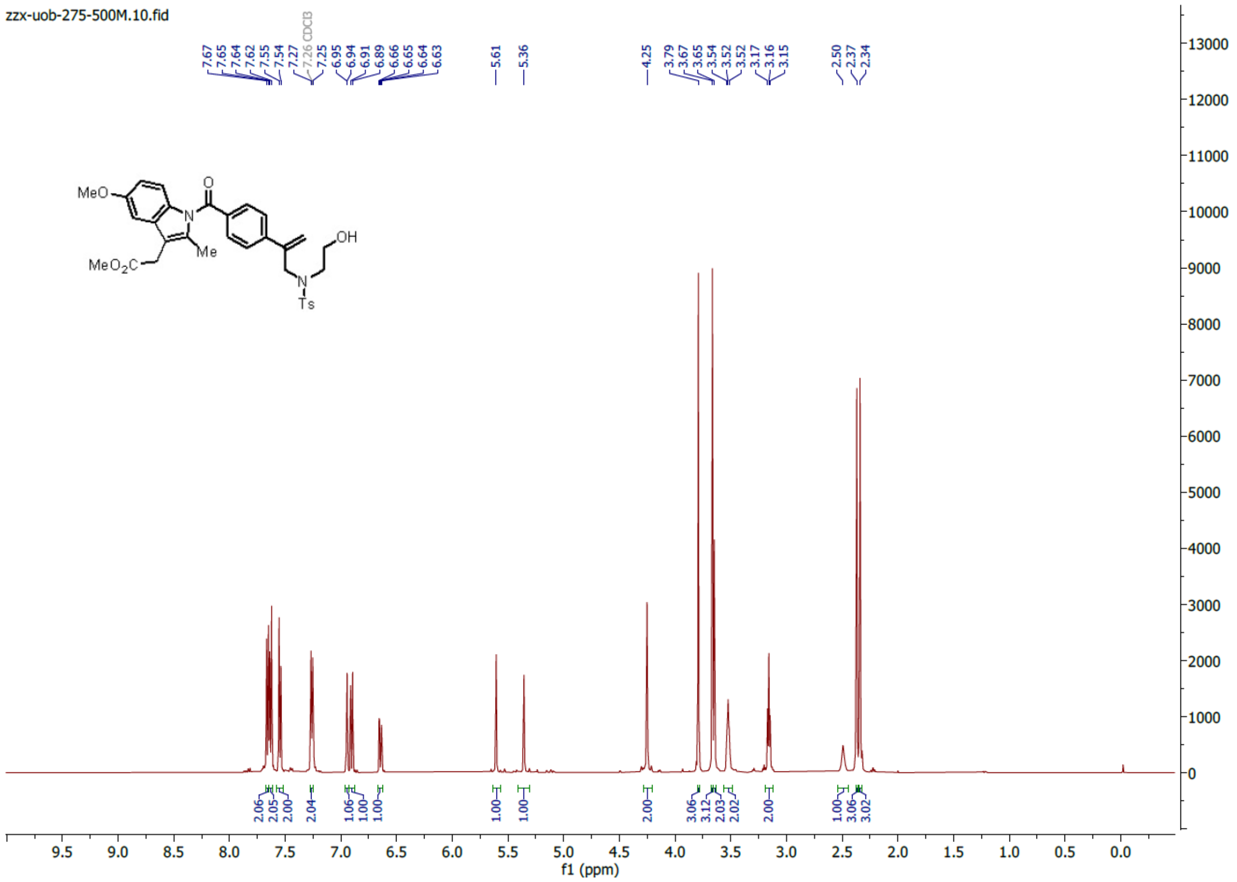

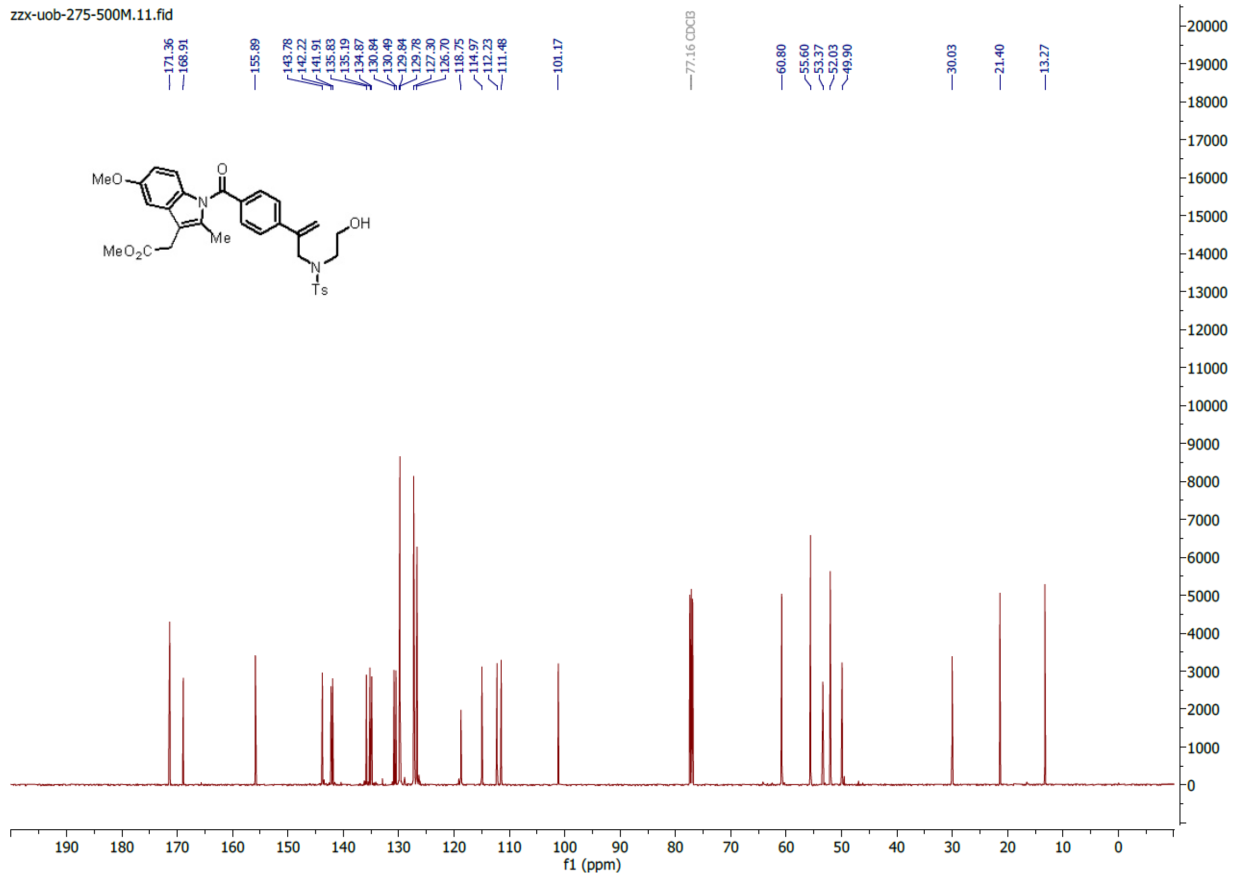

Compound S23b

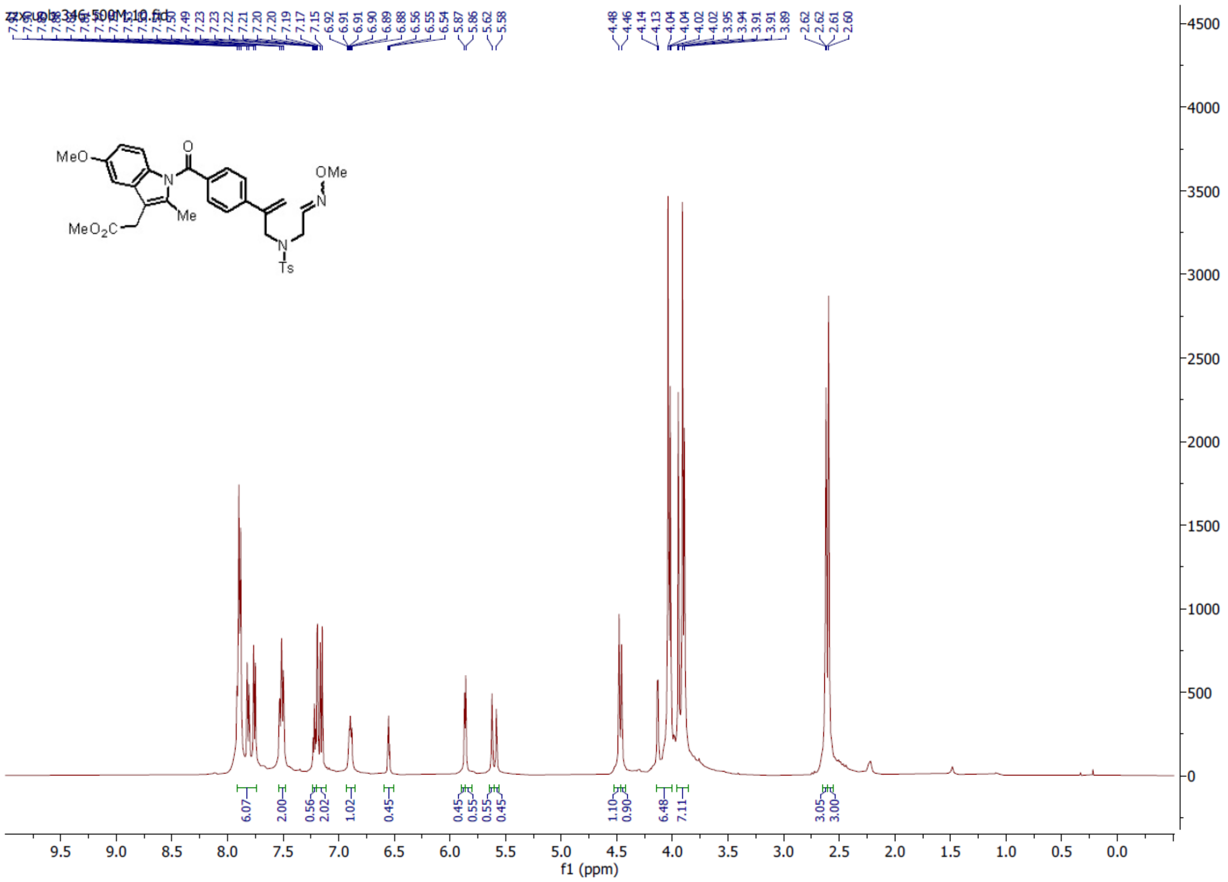

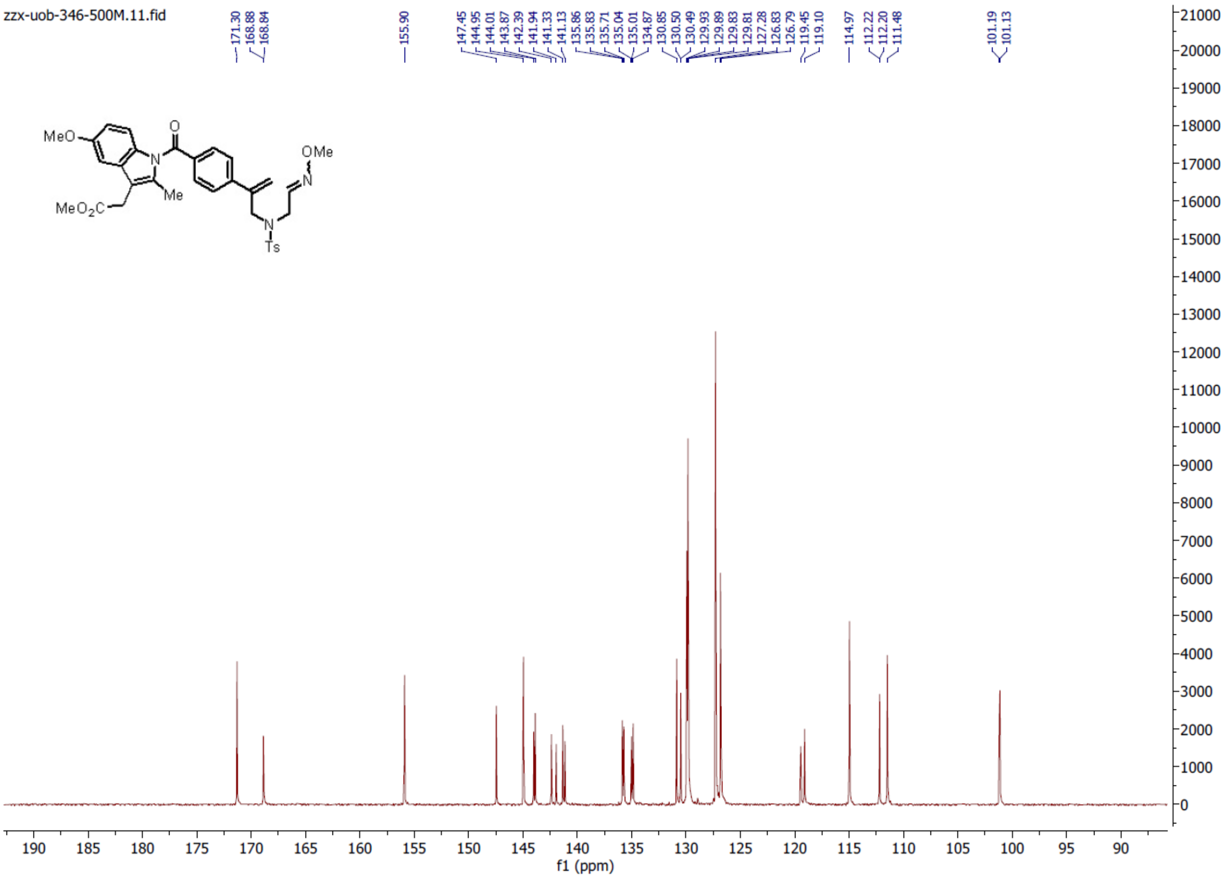

Compound S24a

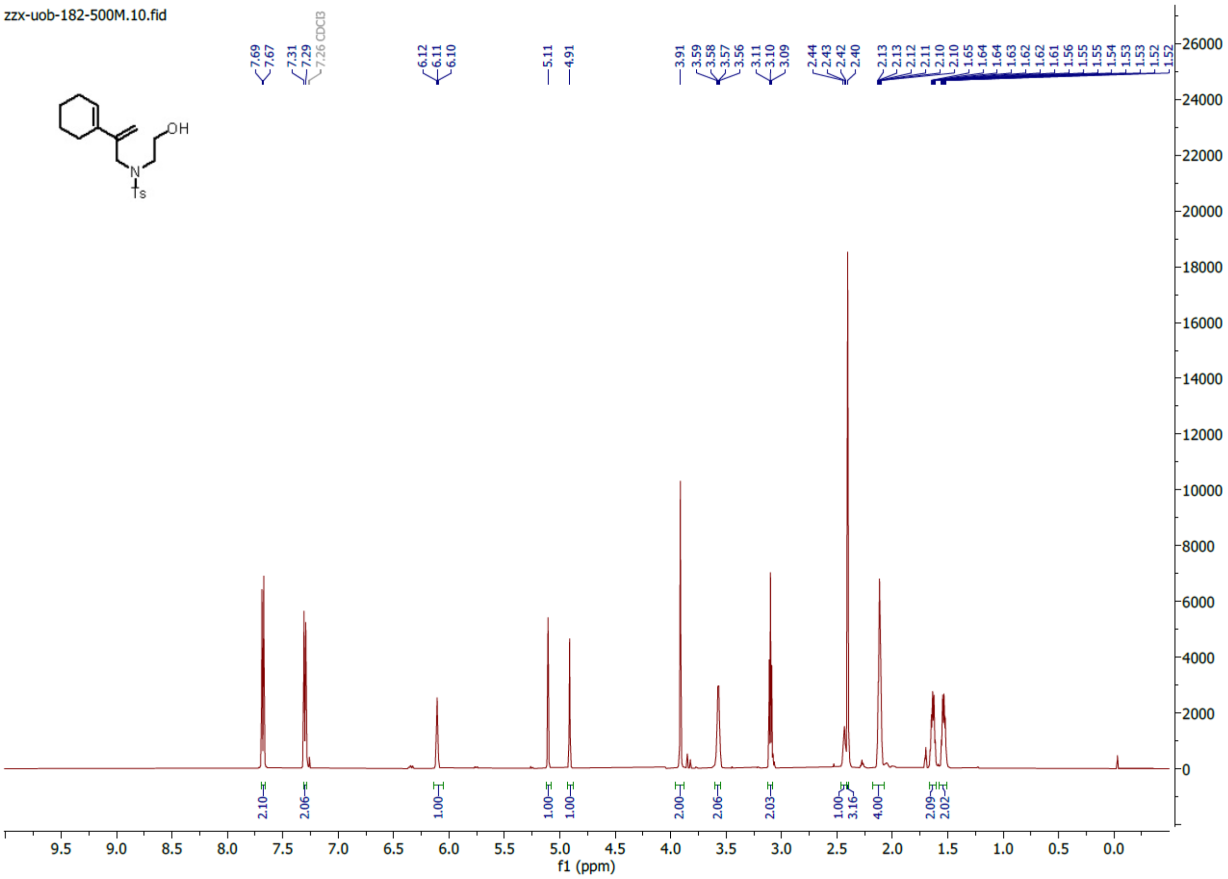

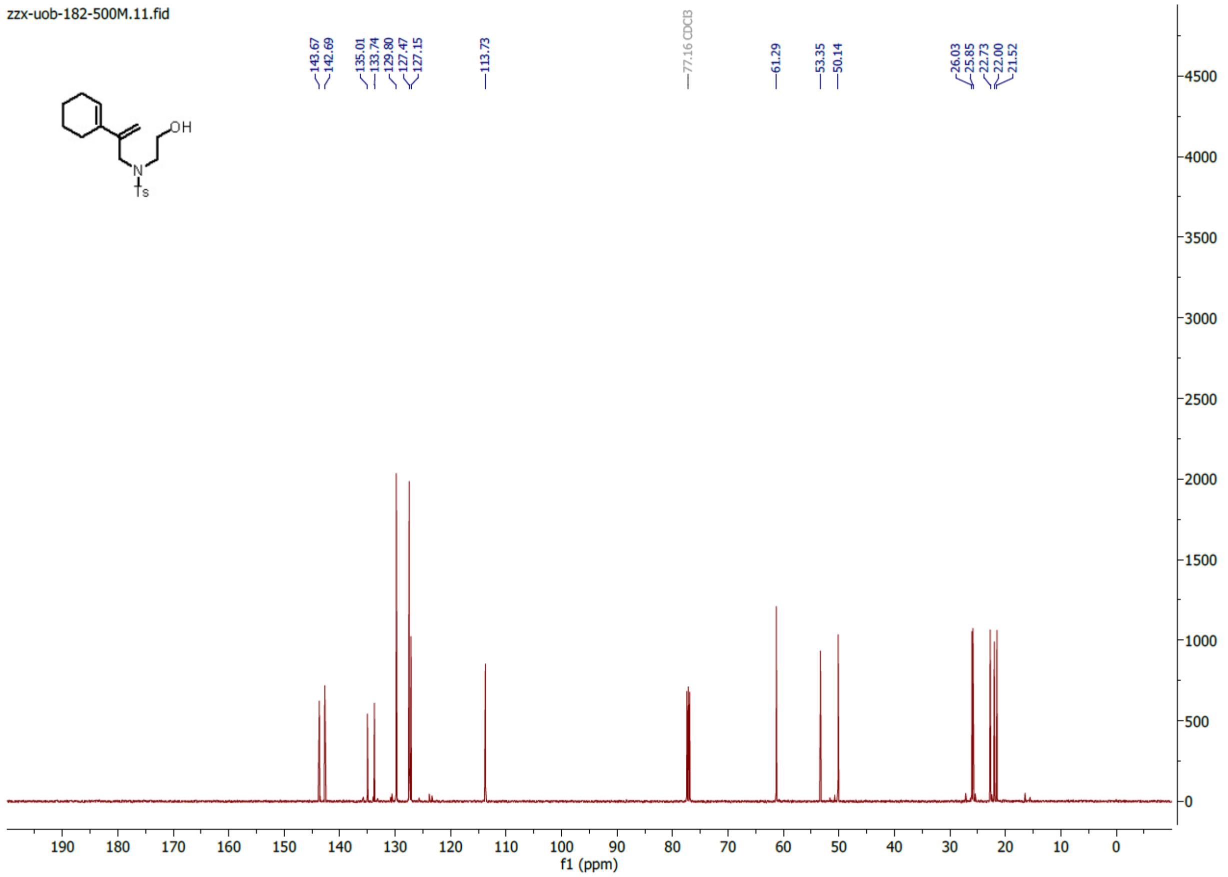

Compound S24b

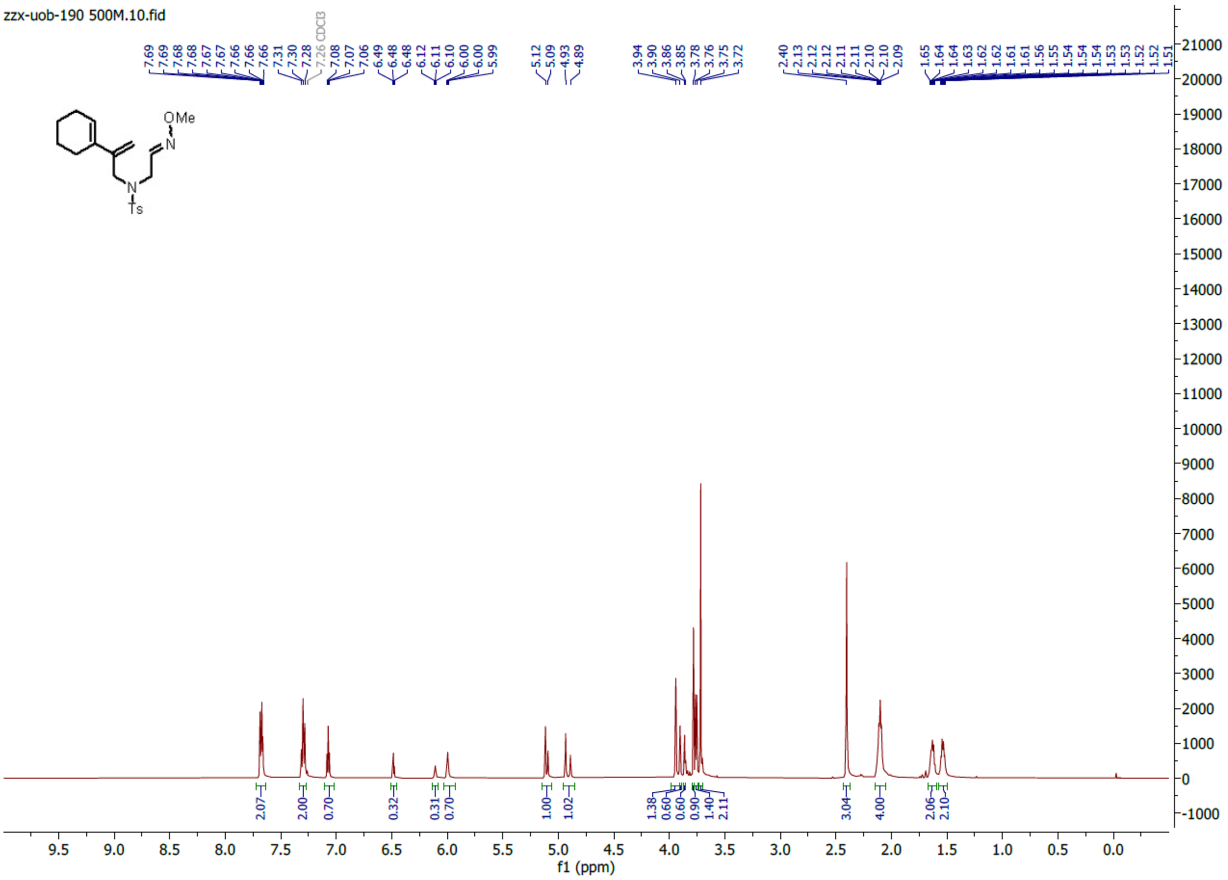

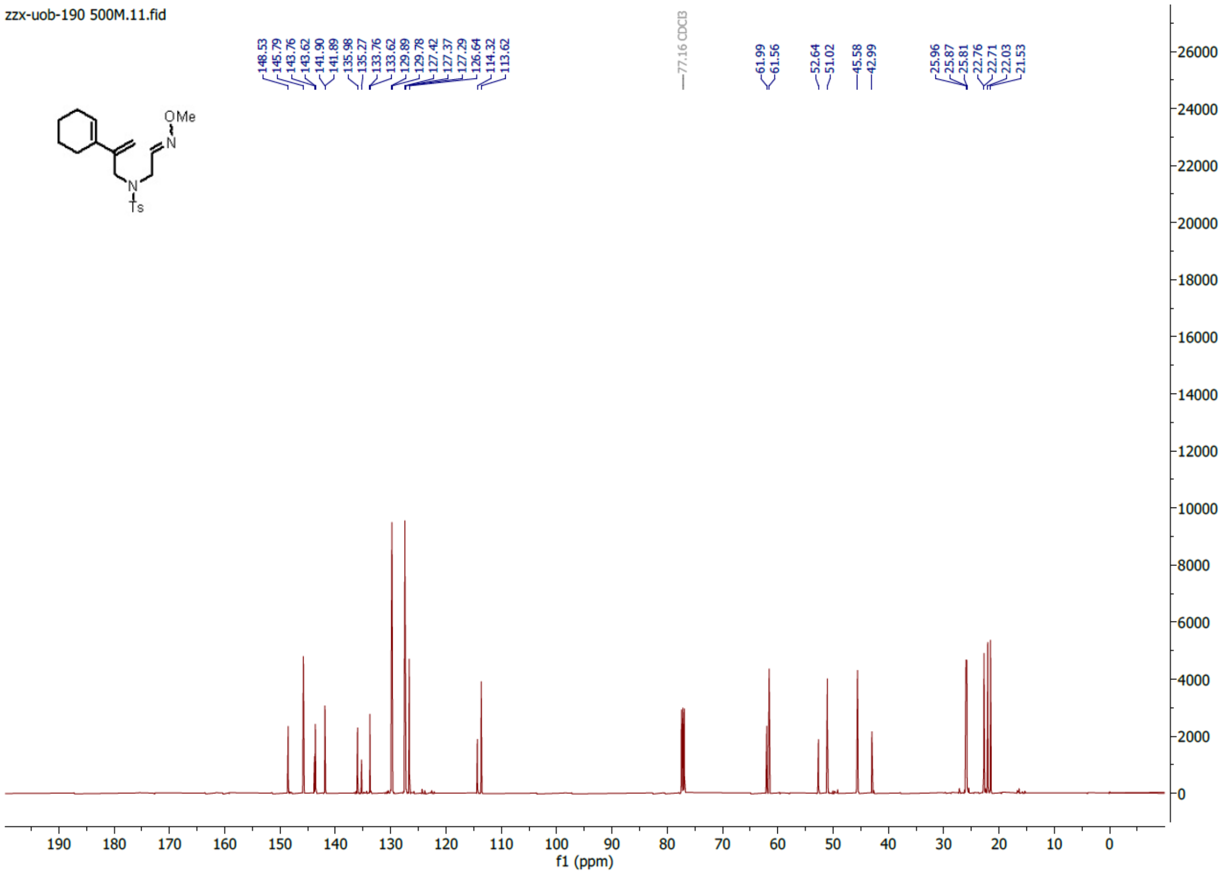

Compound S25a

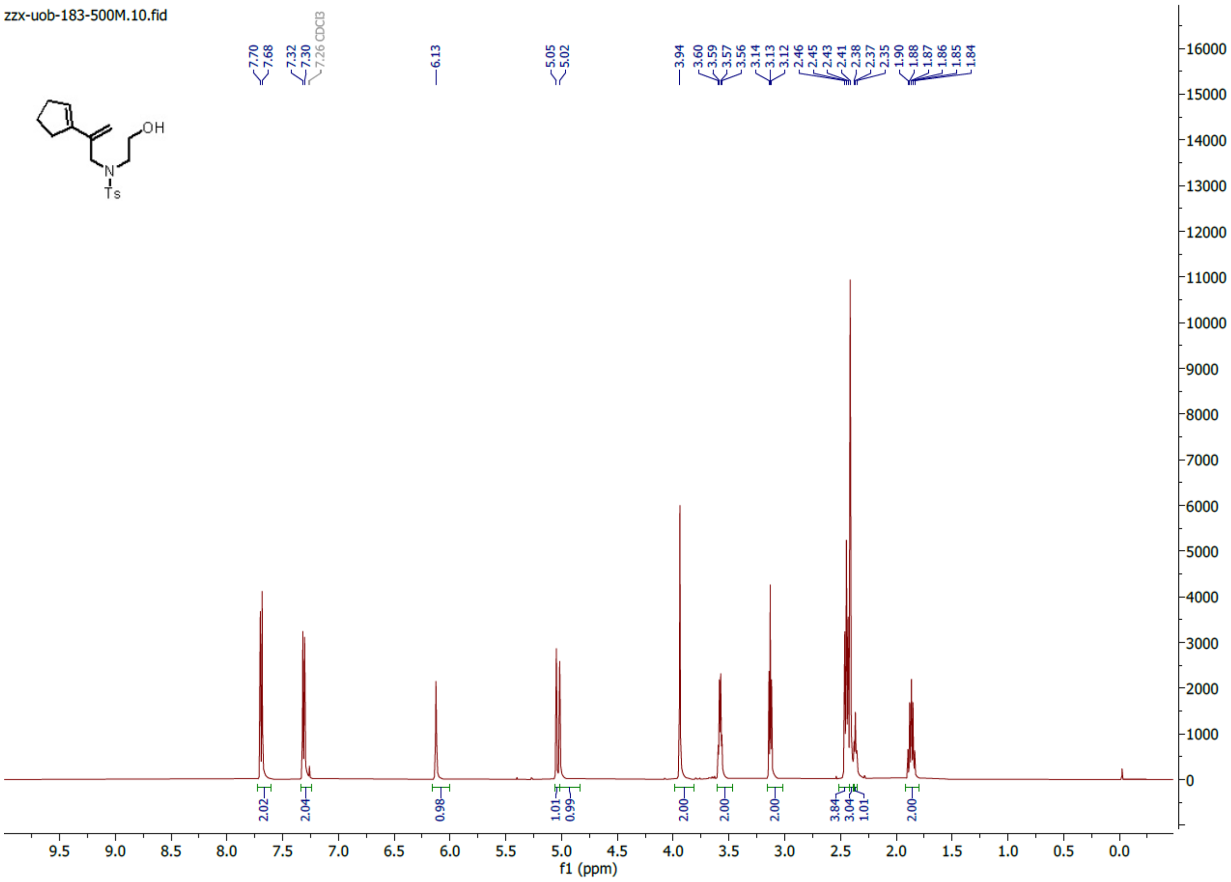

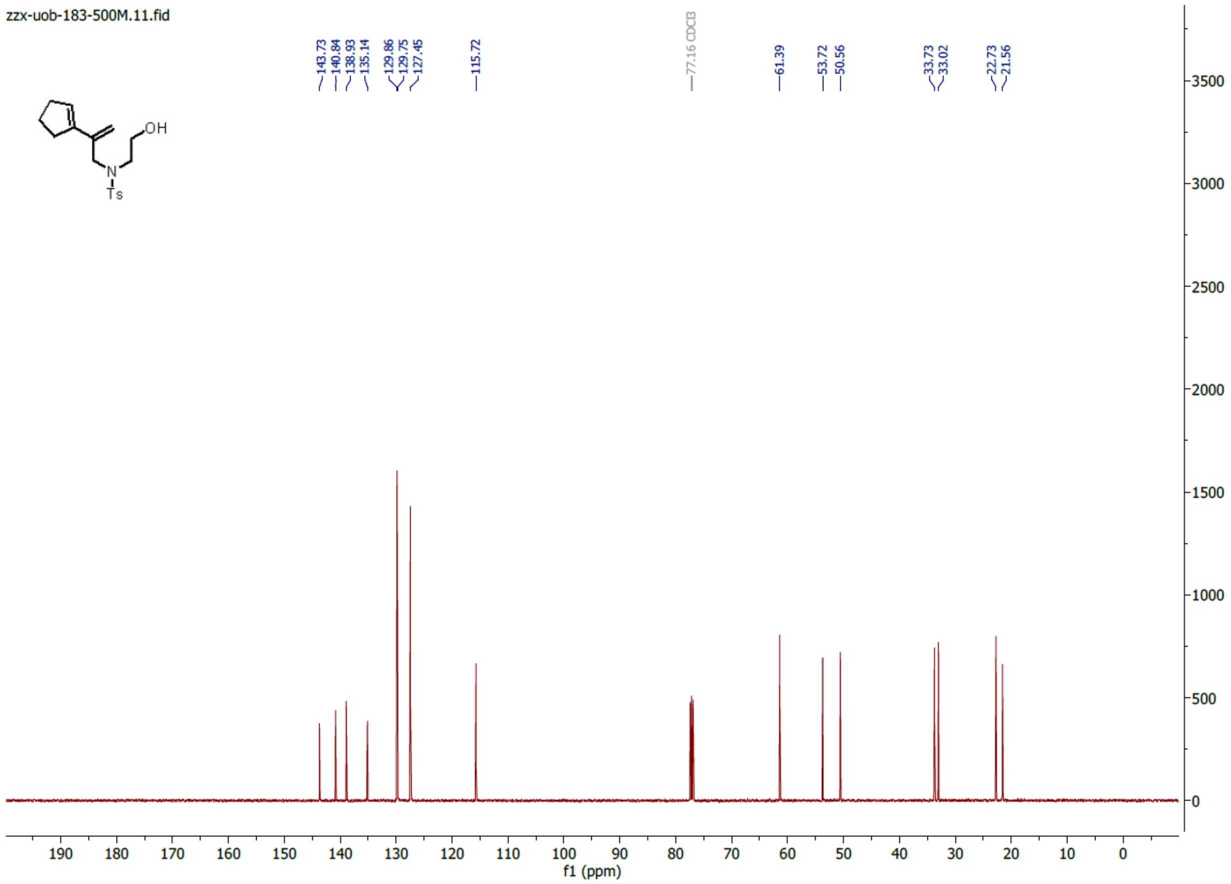

Compound S25b

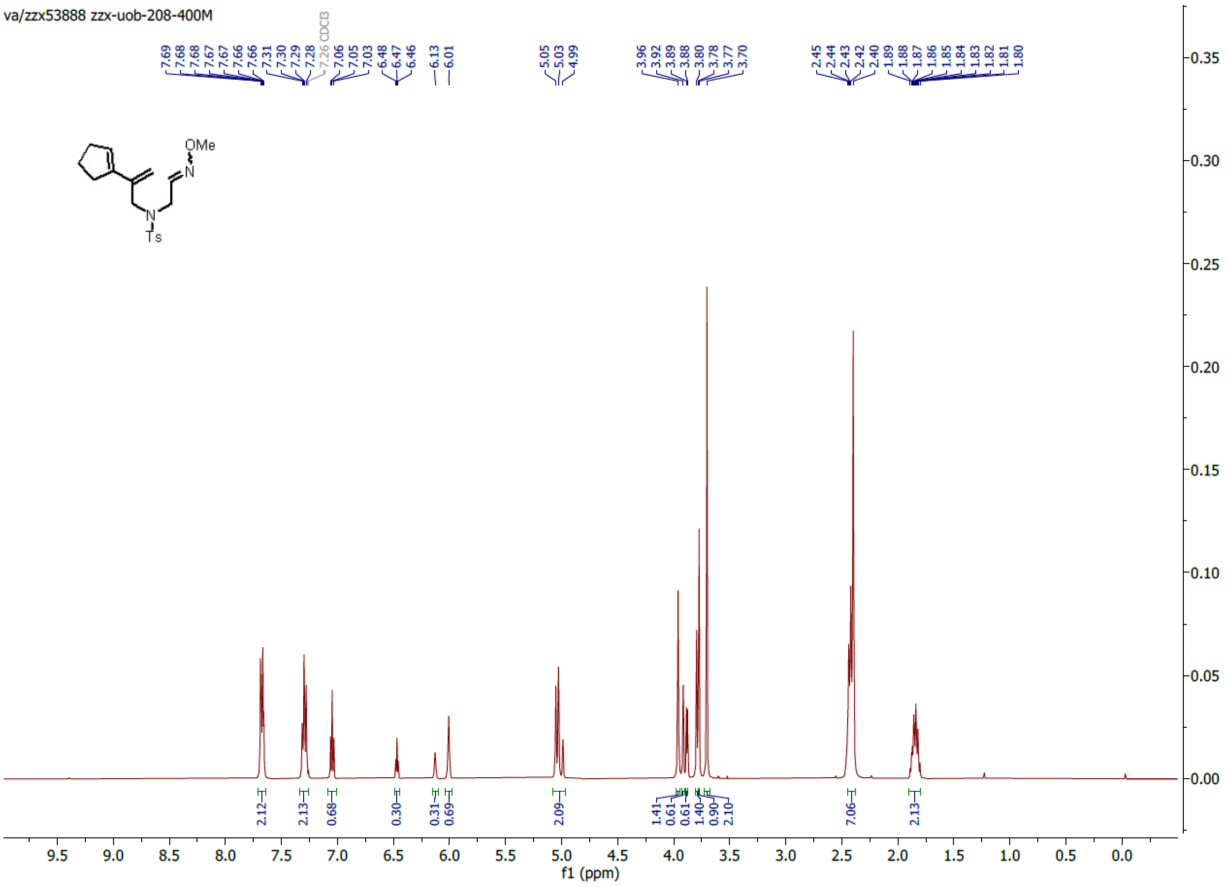

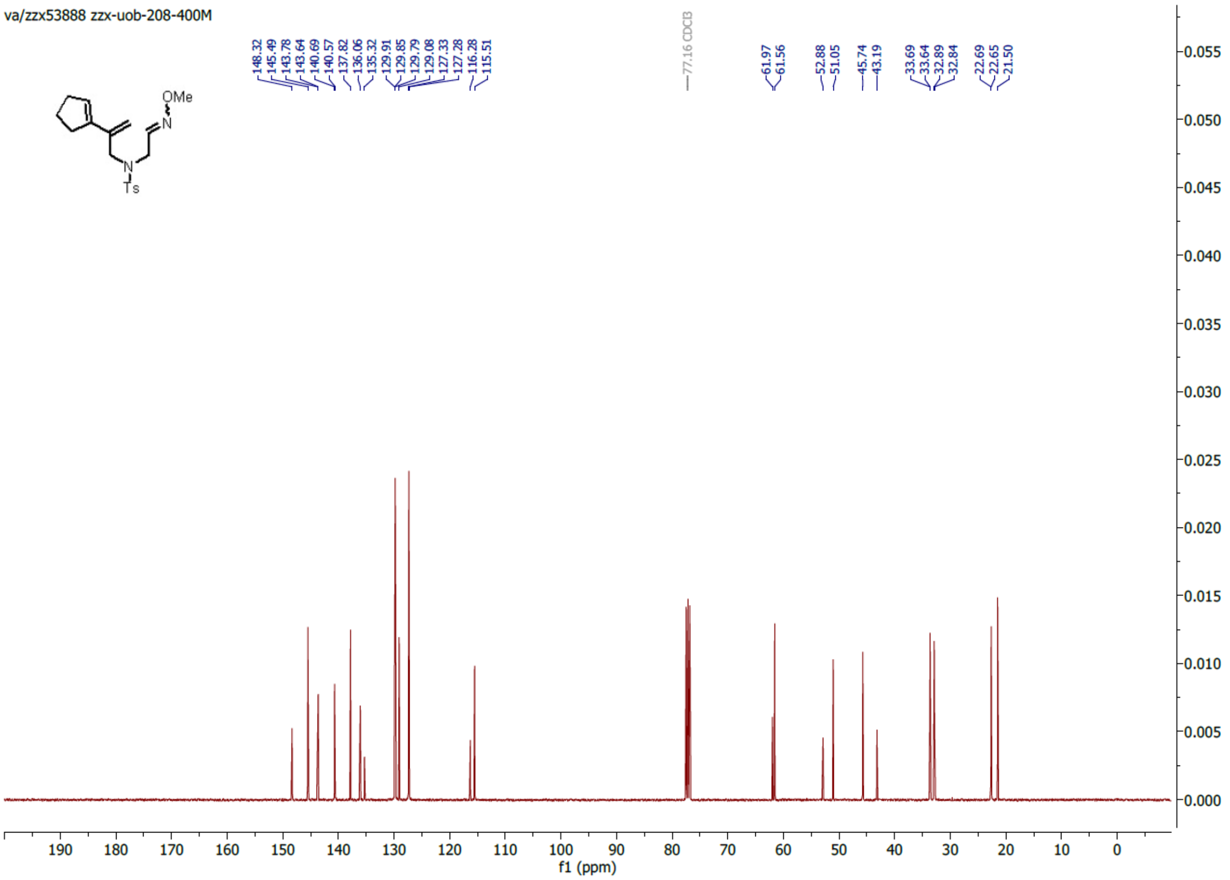

Compound S50b

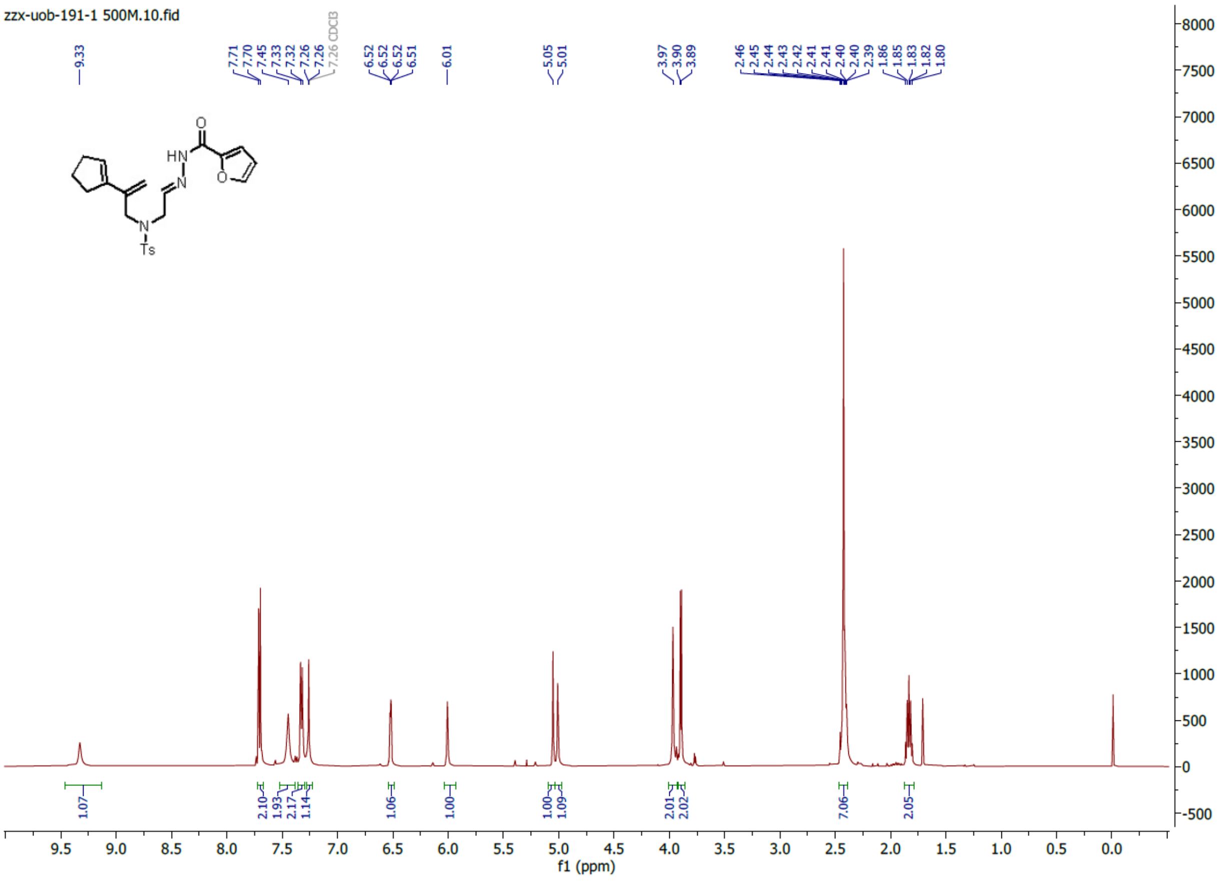

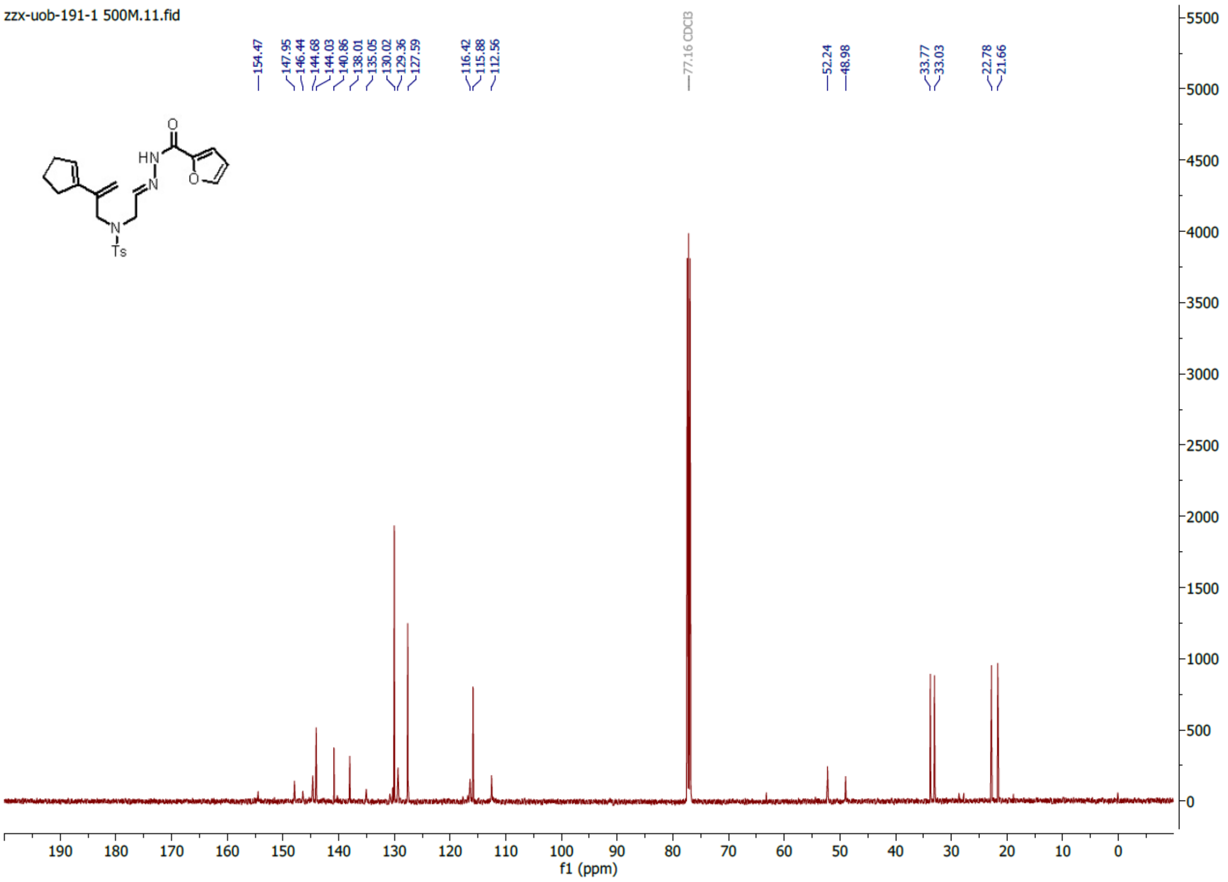

Compound S27a'

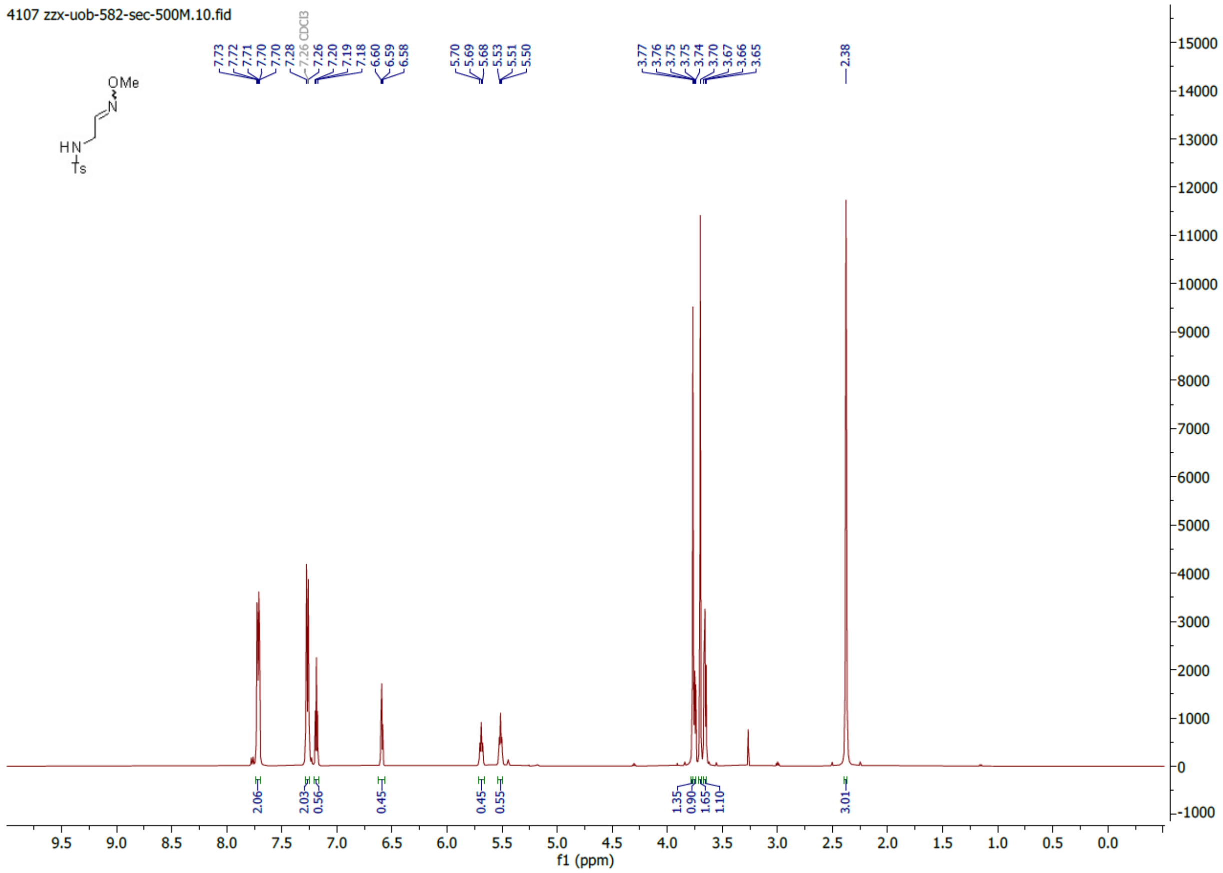

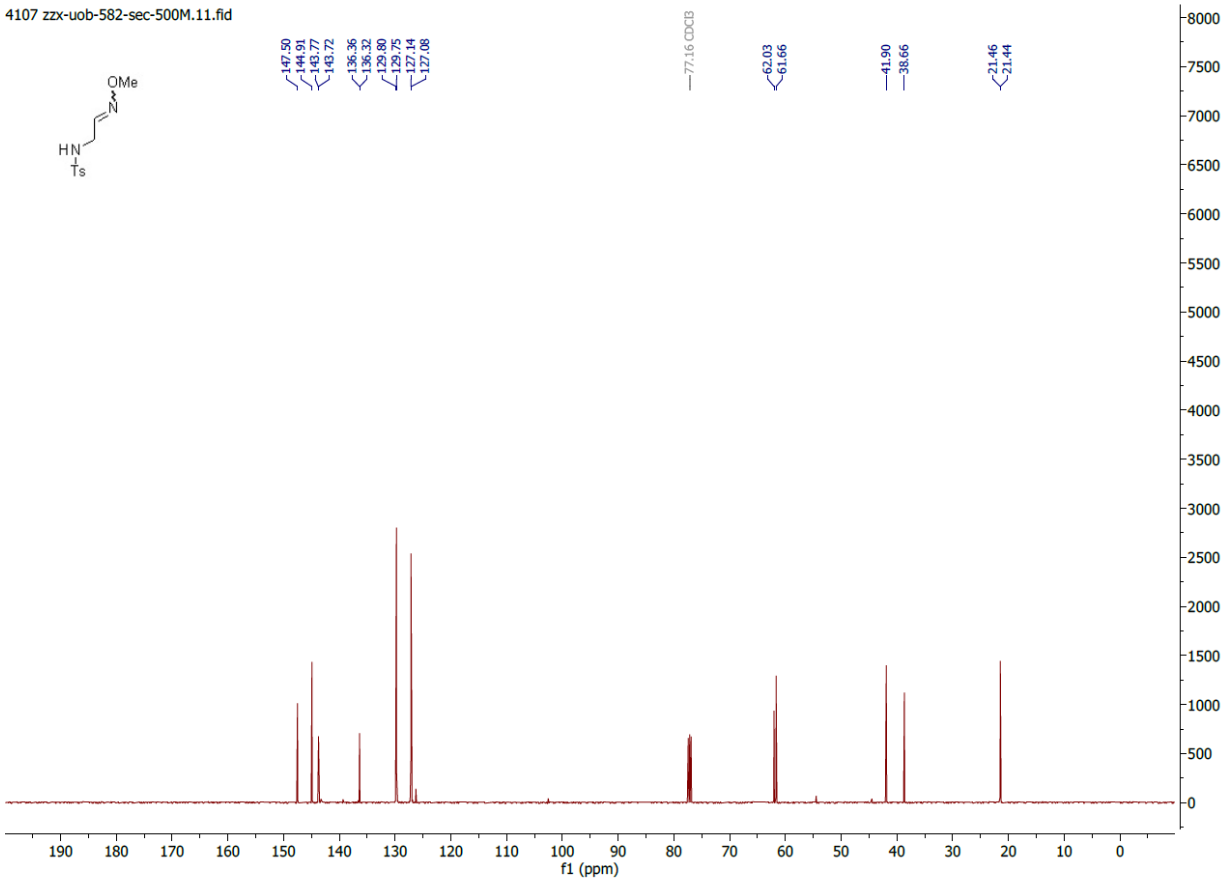

Compound S27a''

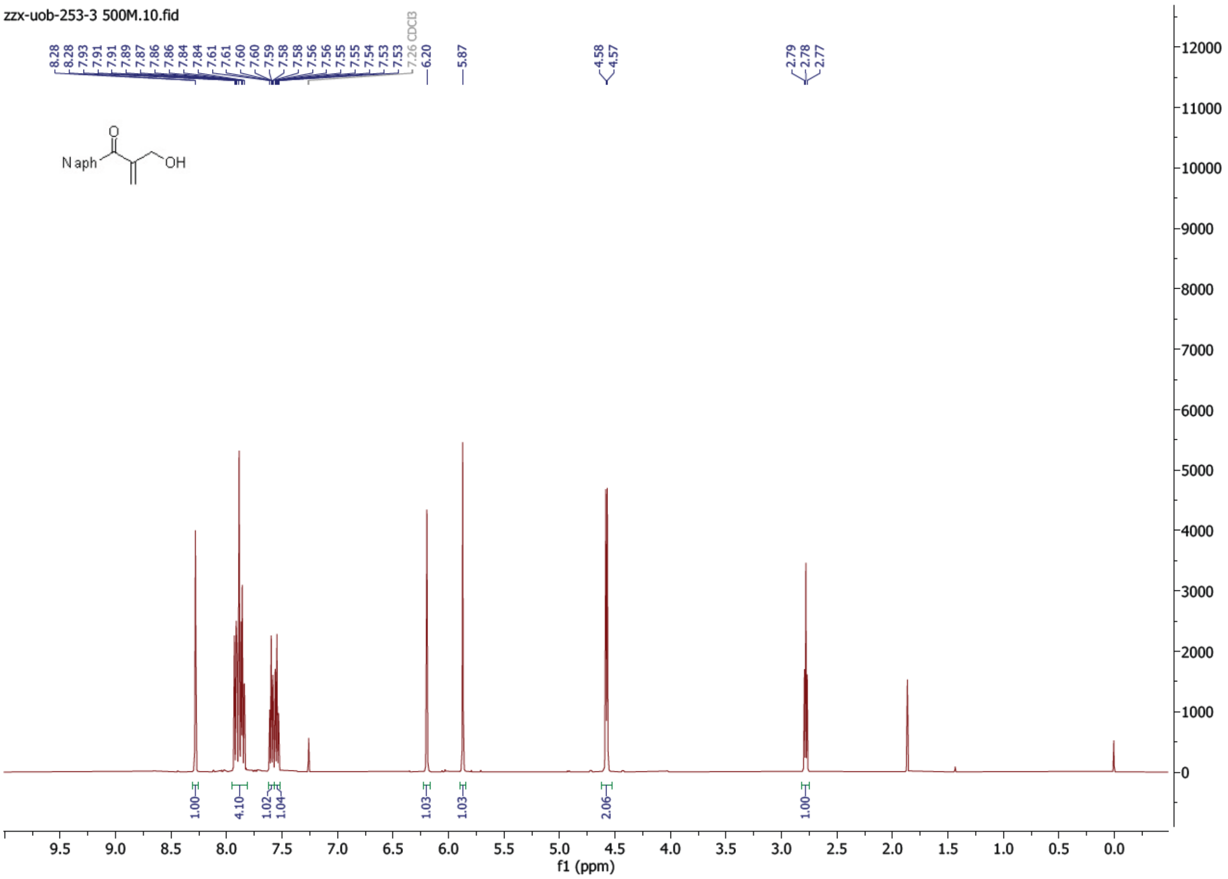

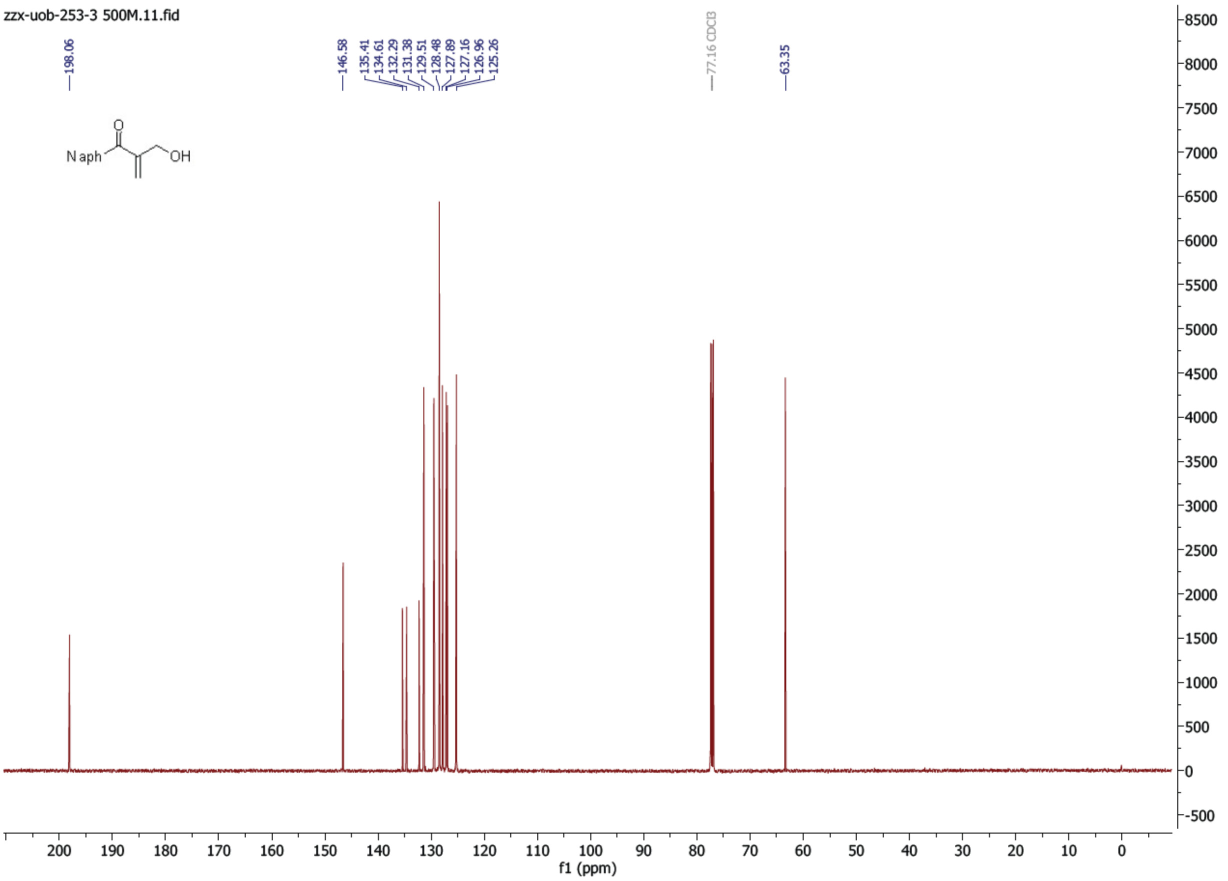

Compound S27a

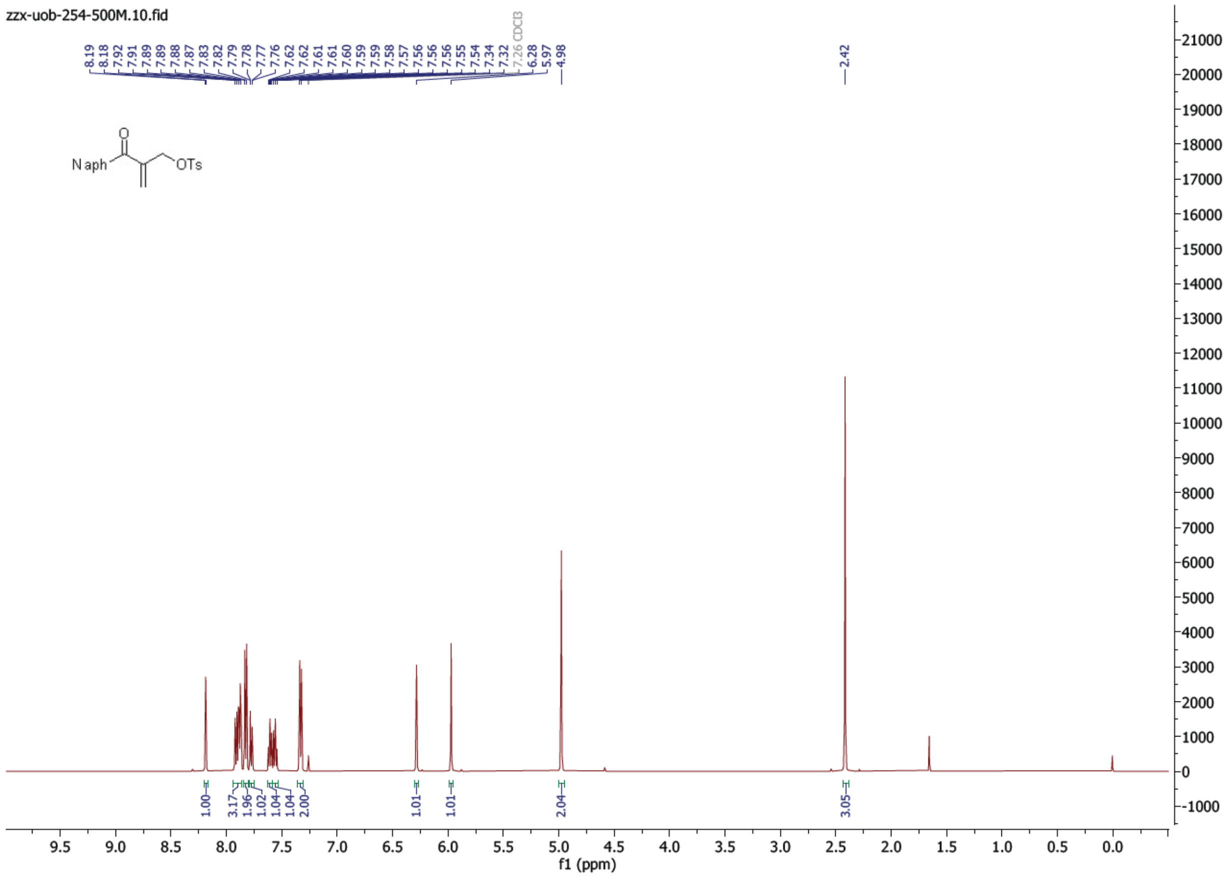

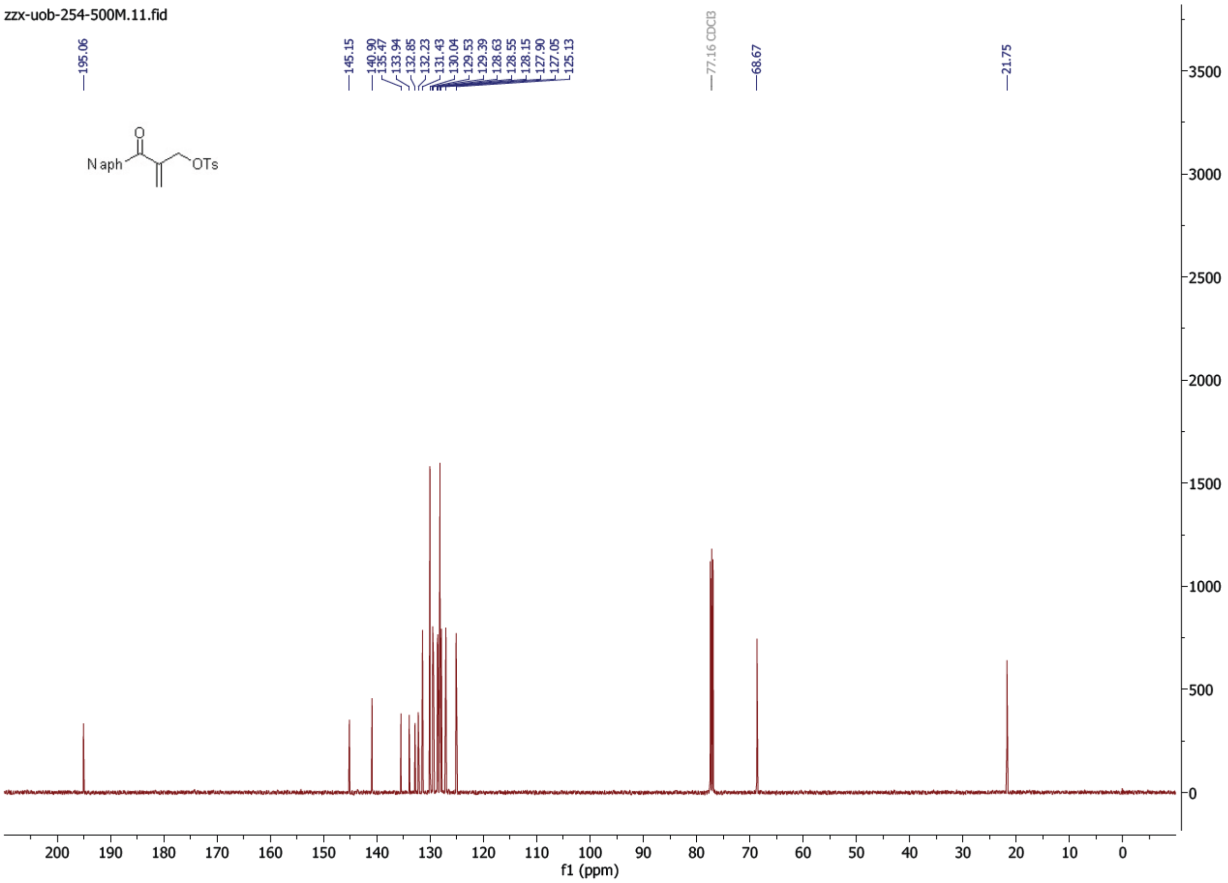

Compound S27b

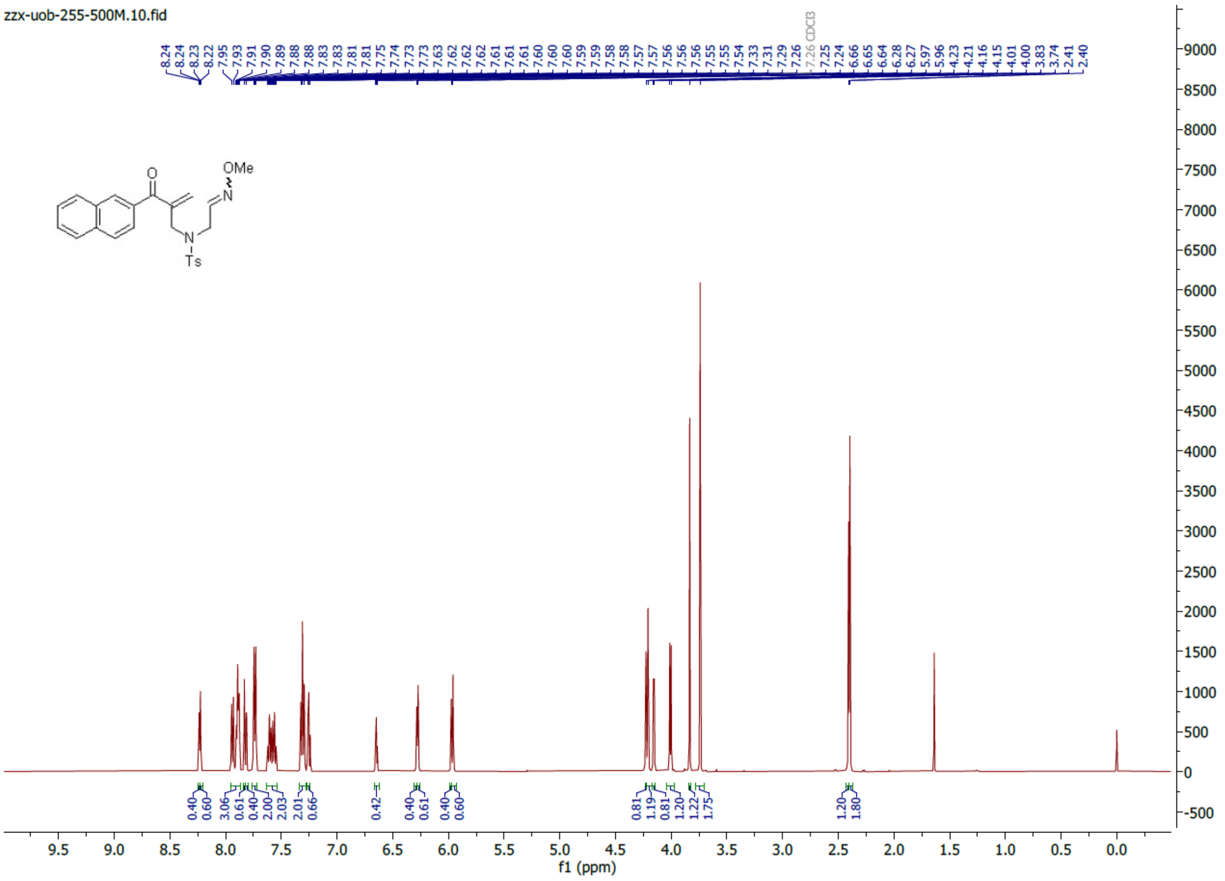

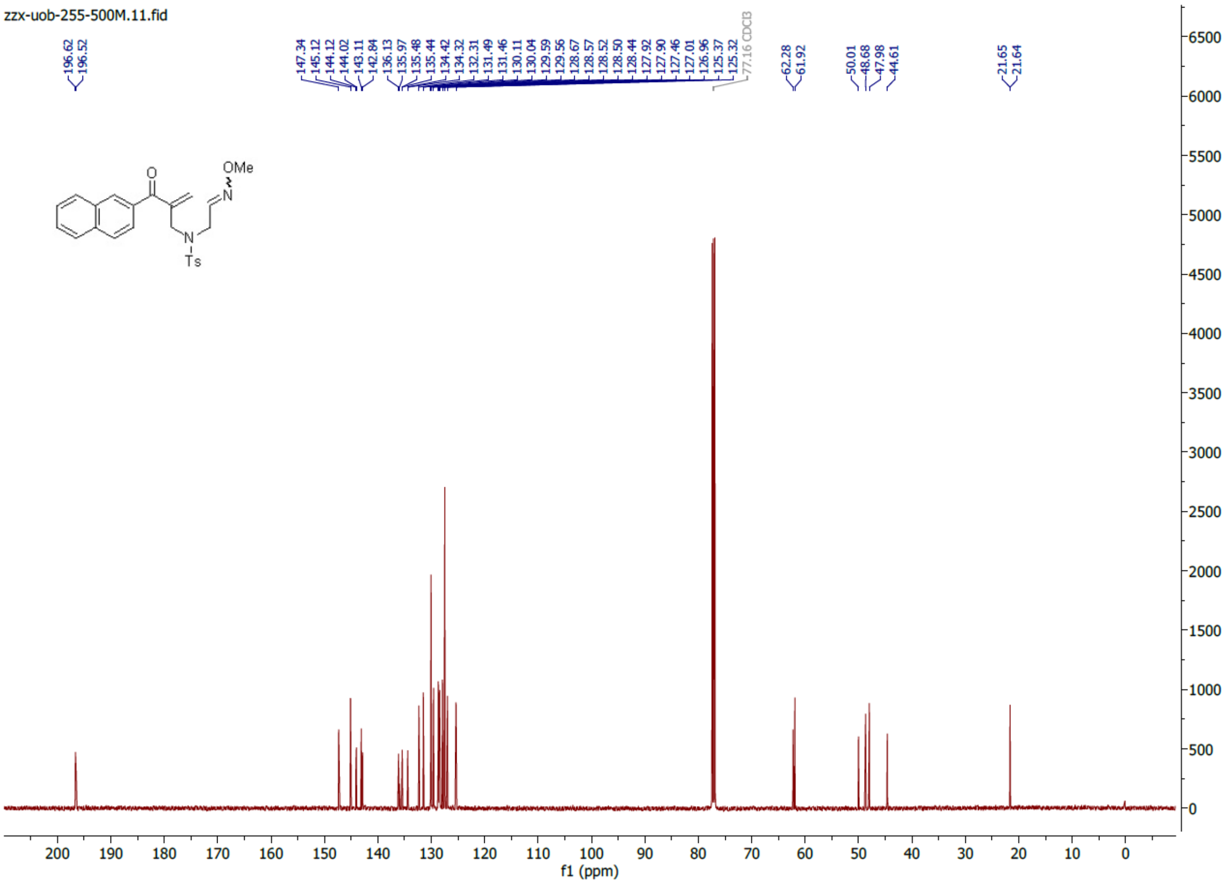

Compound S28a

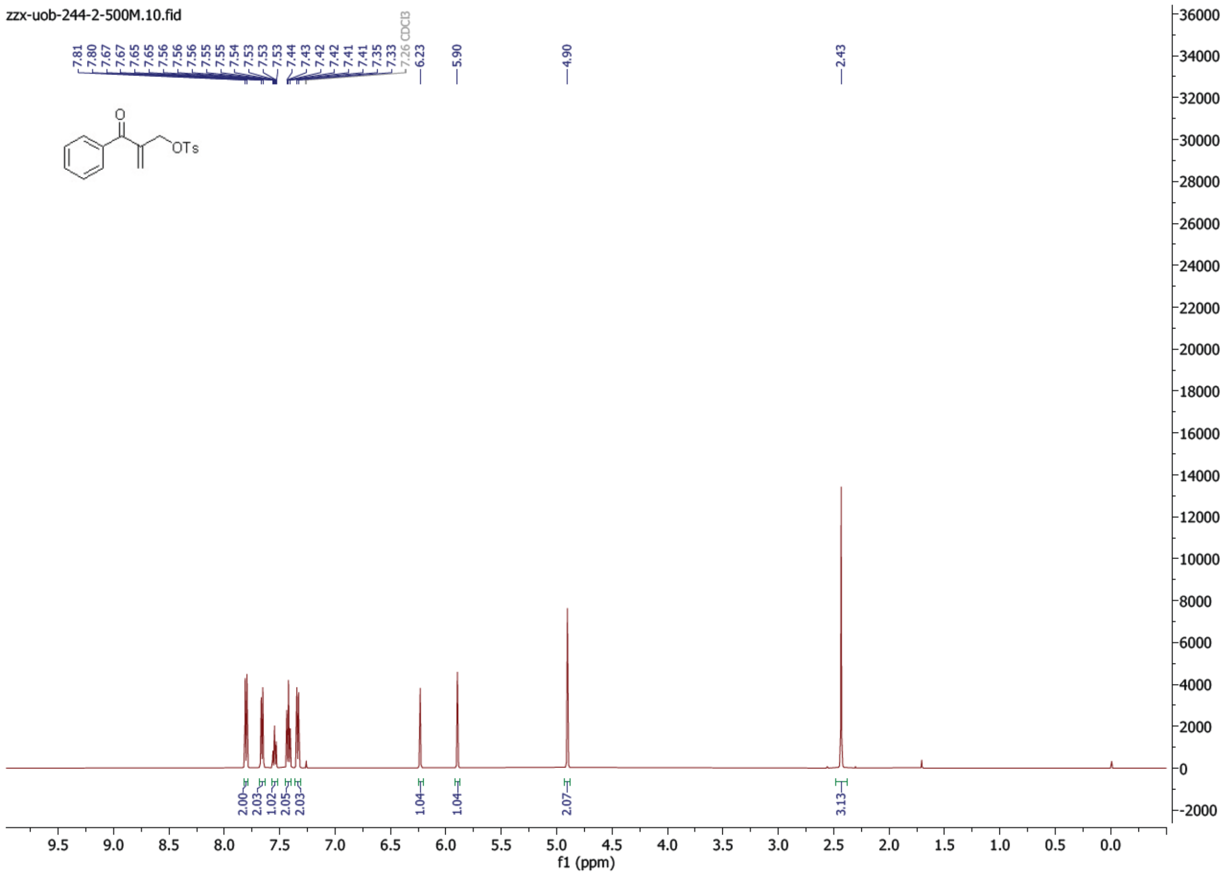

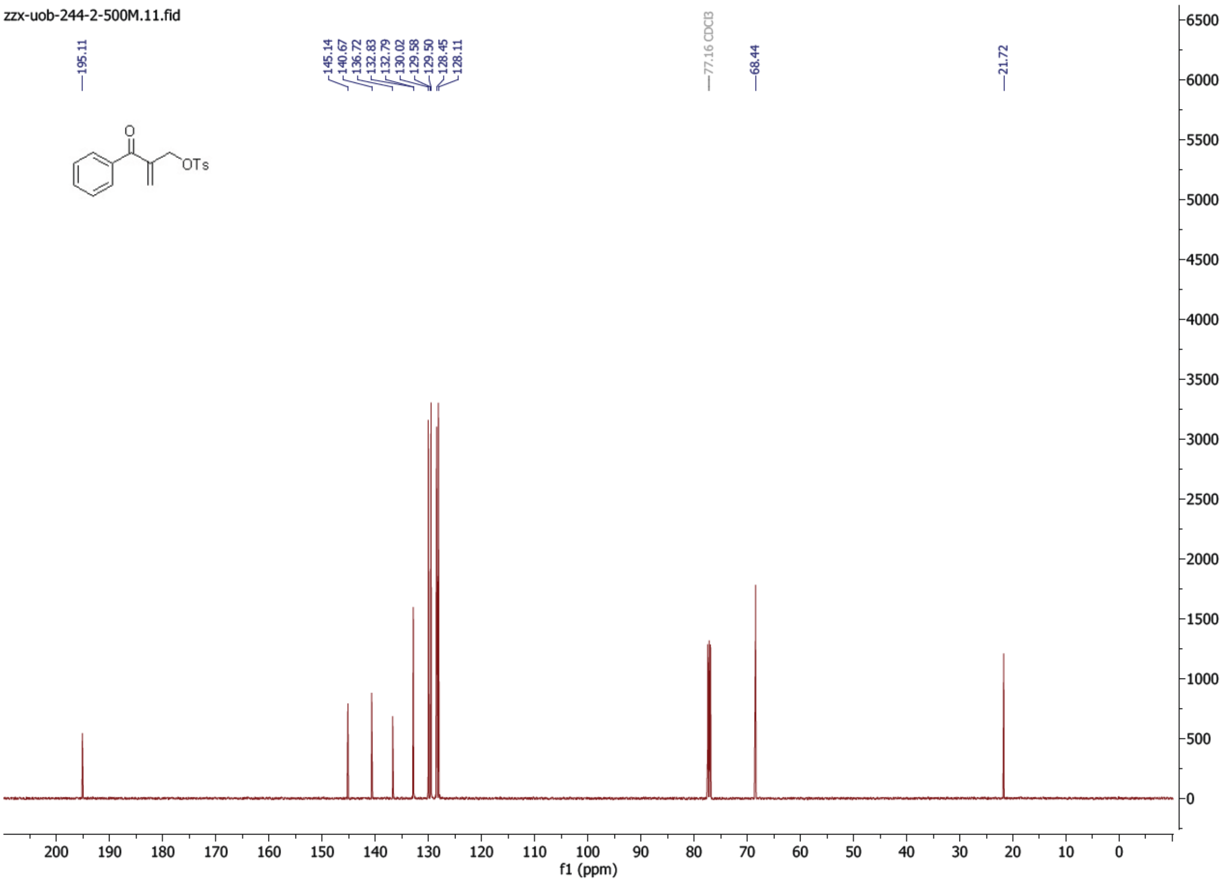

Compound S28b

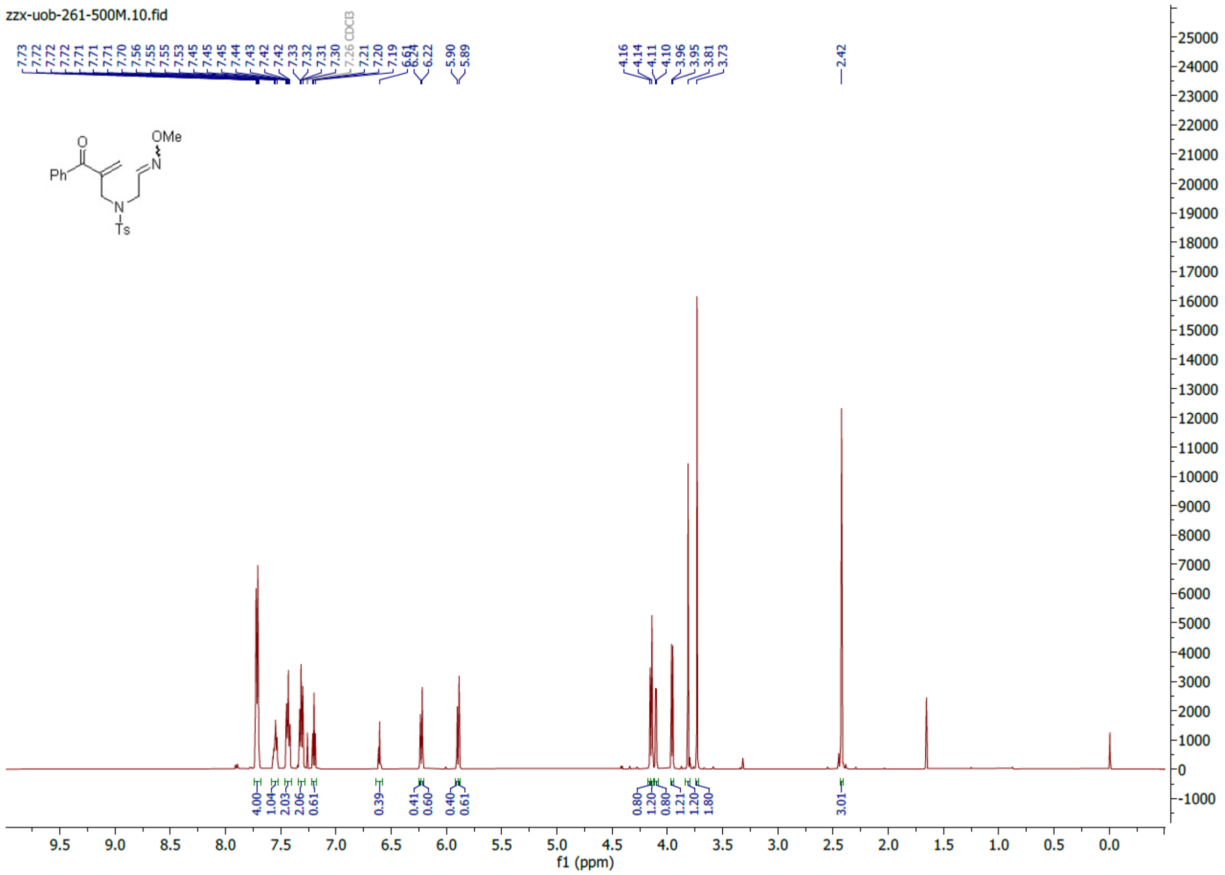

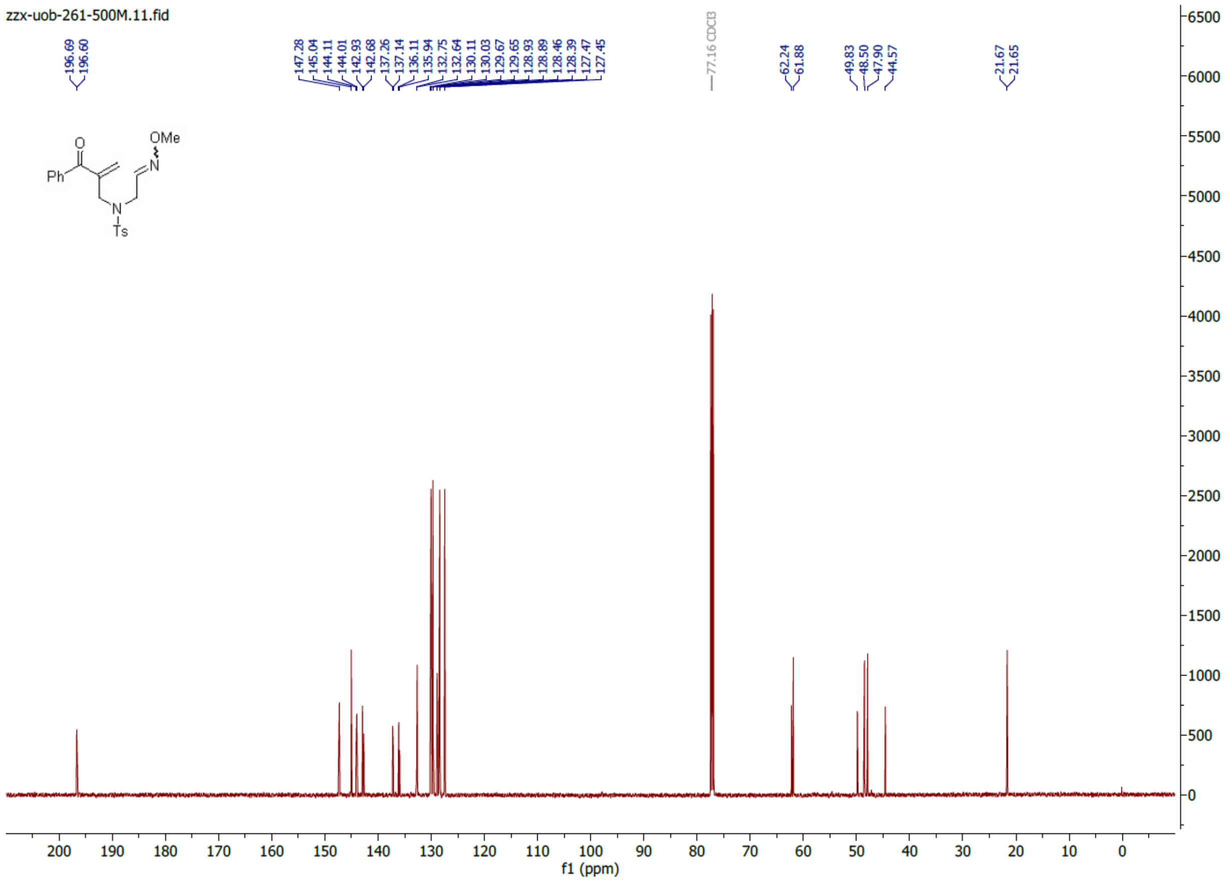

Compound S31a

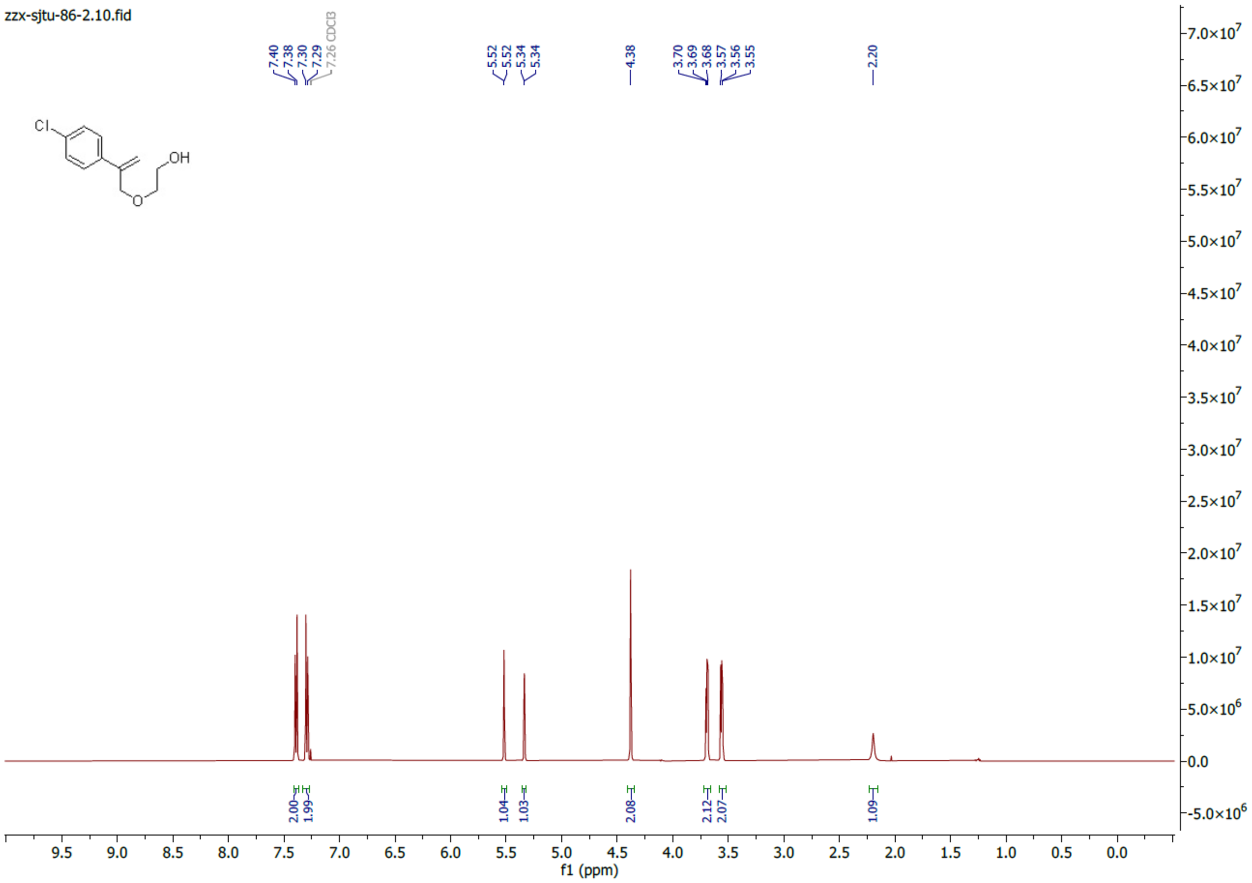

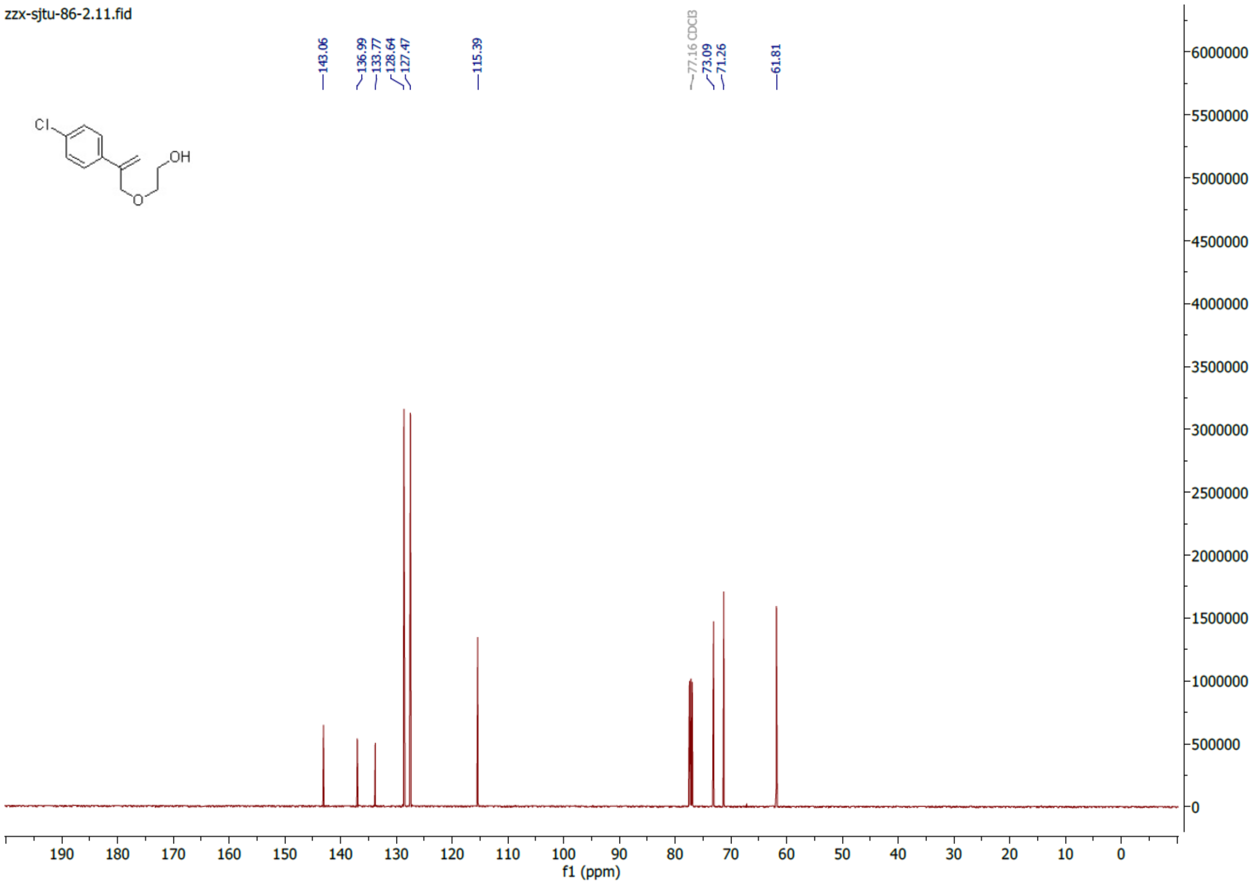

Compound S31b

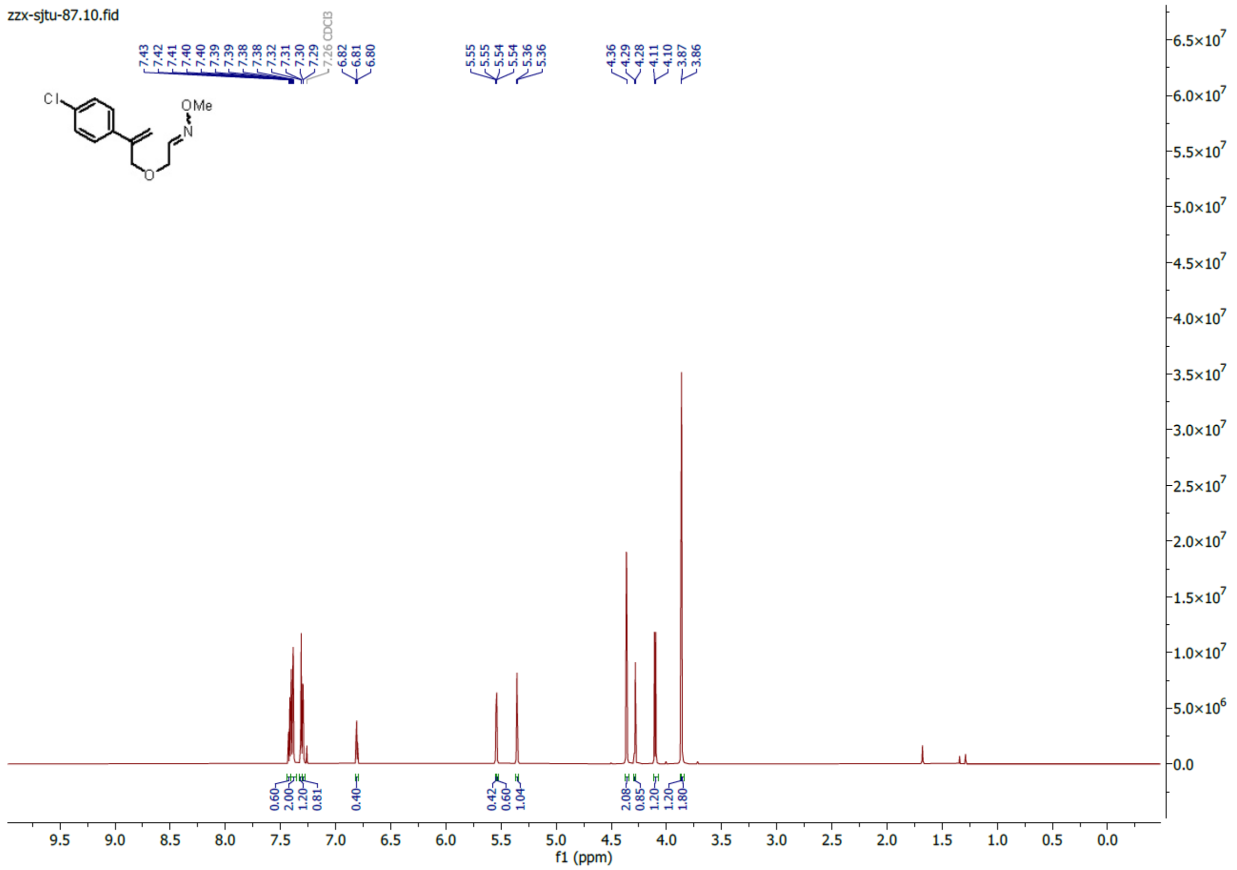

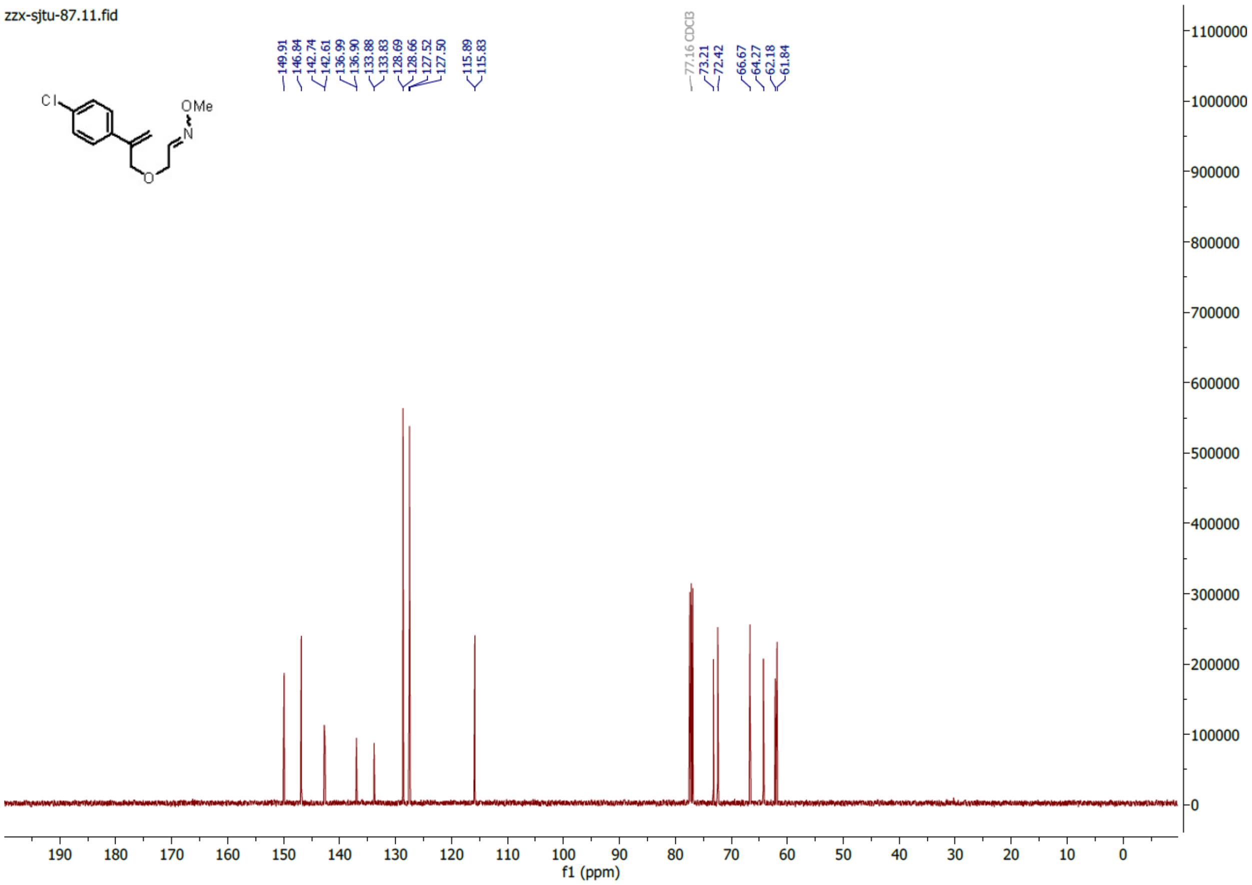

Compound S32a

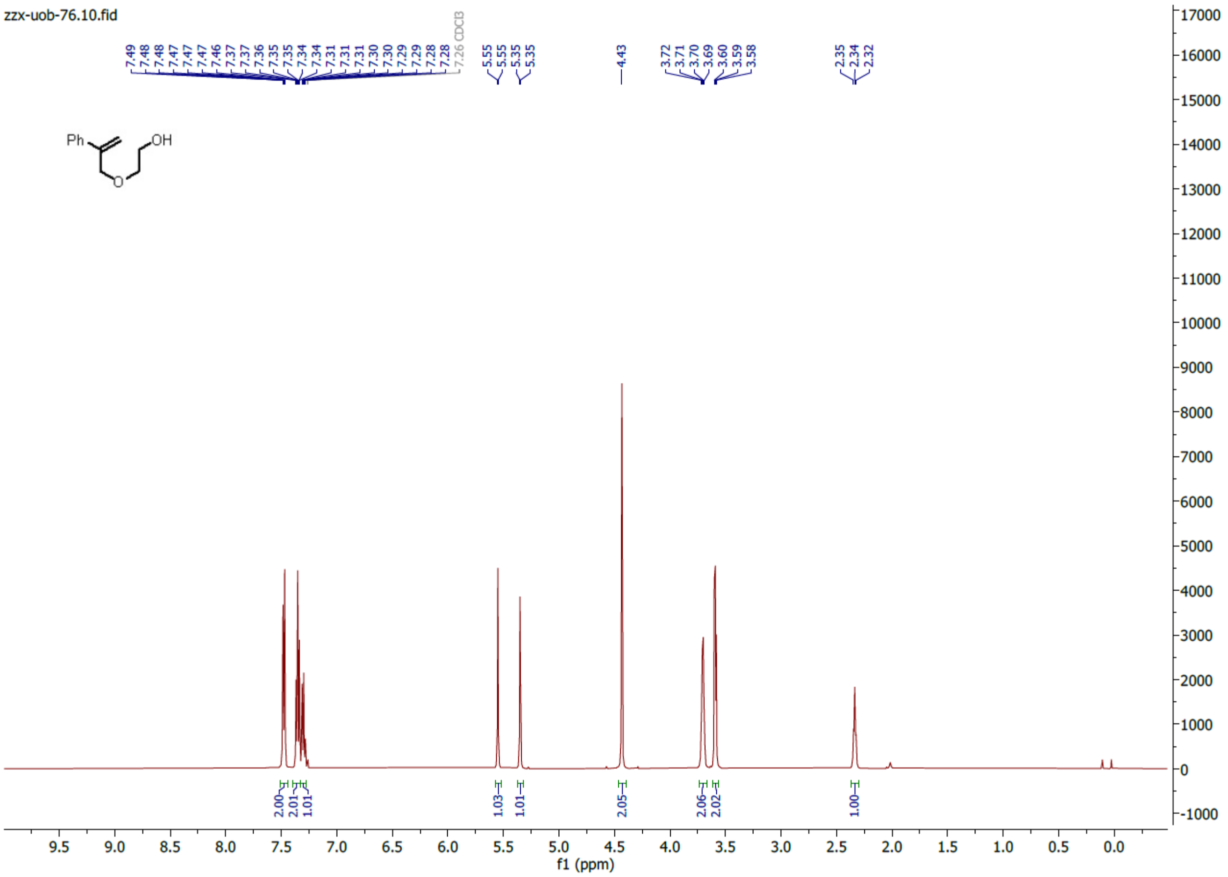

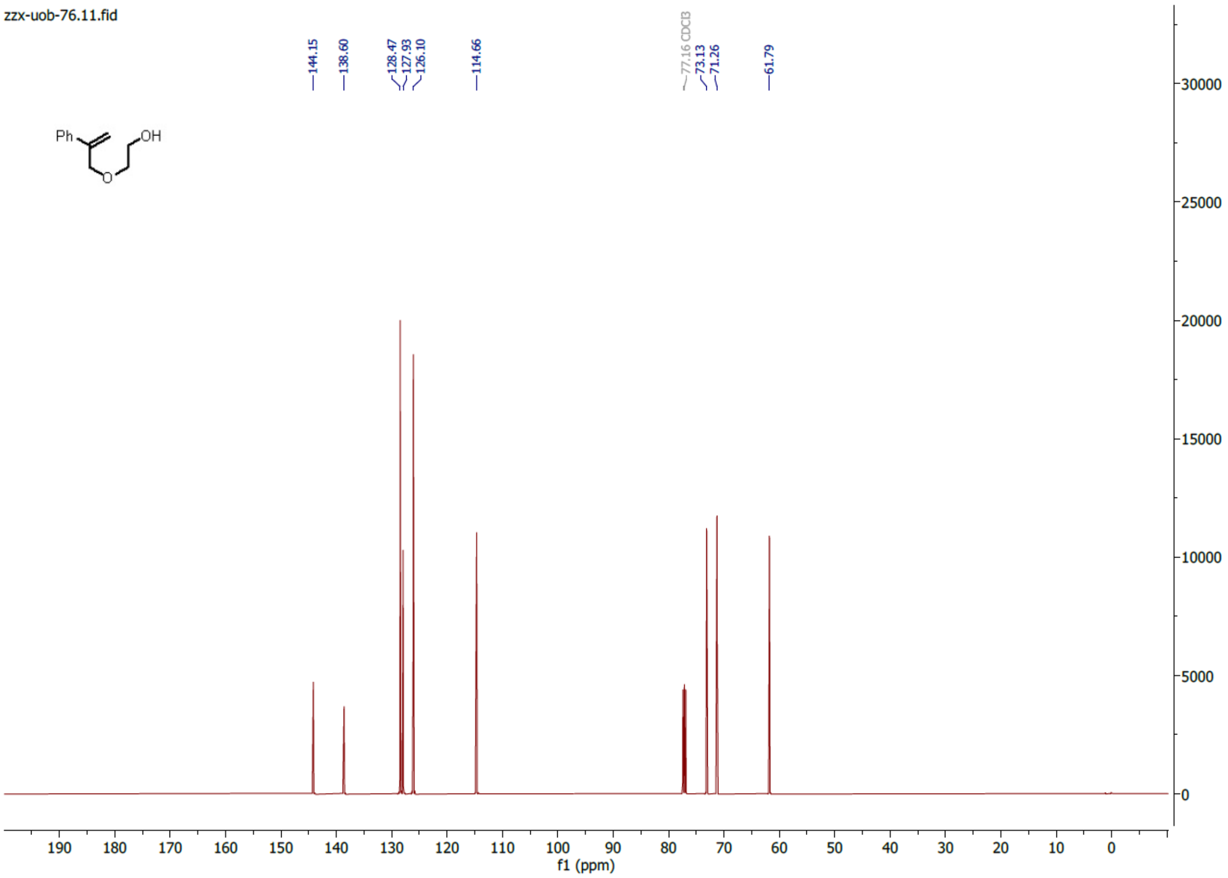

Compound S32b

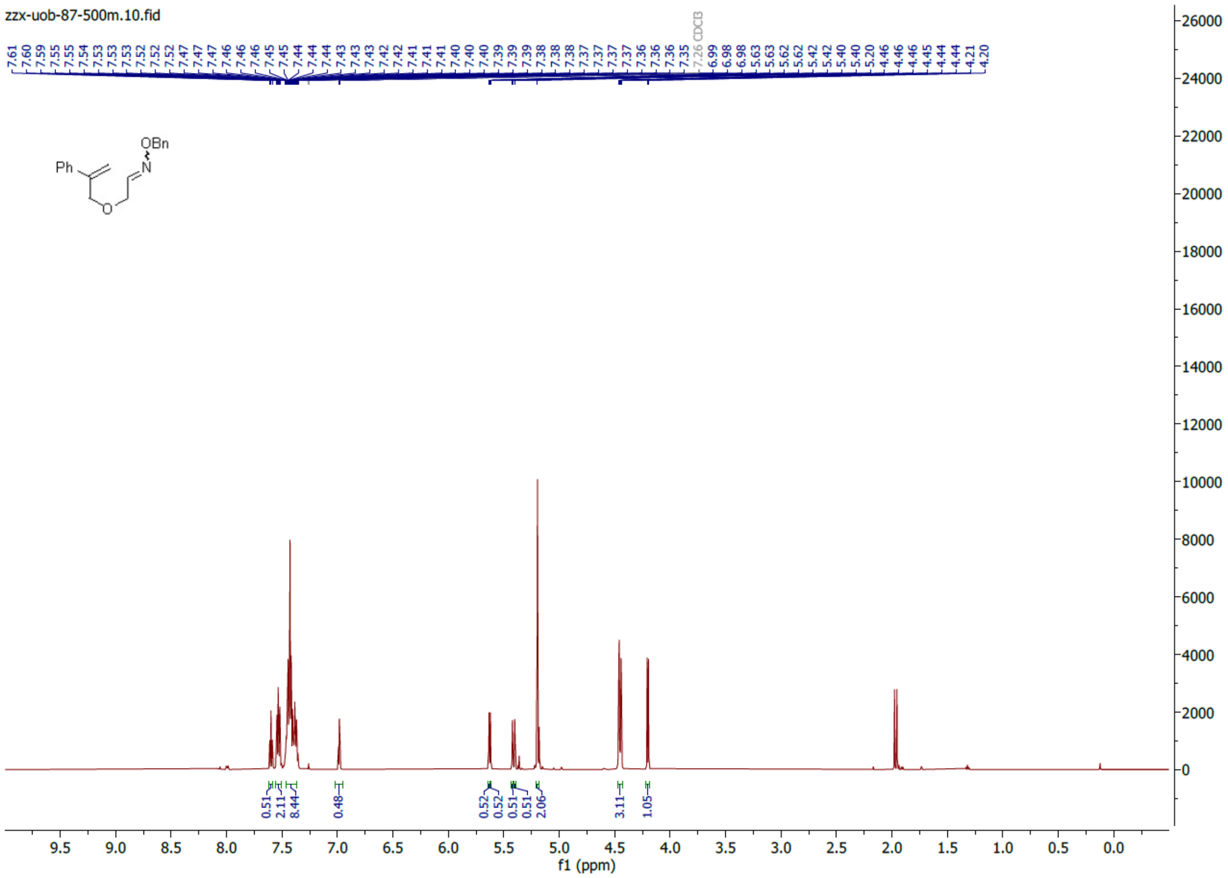

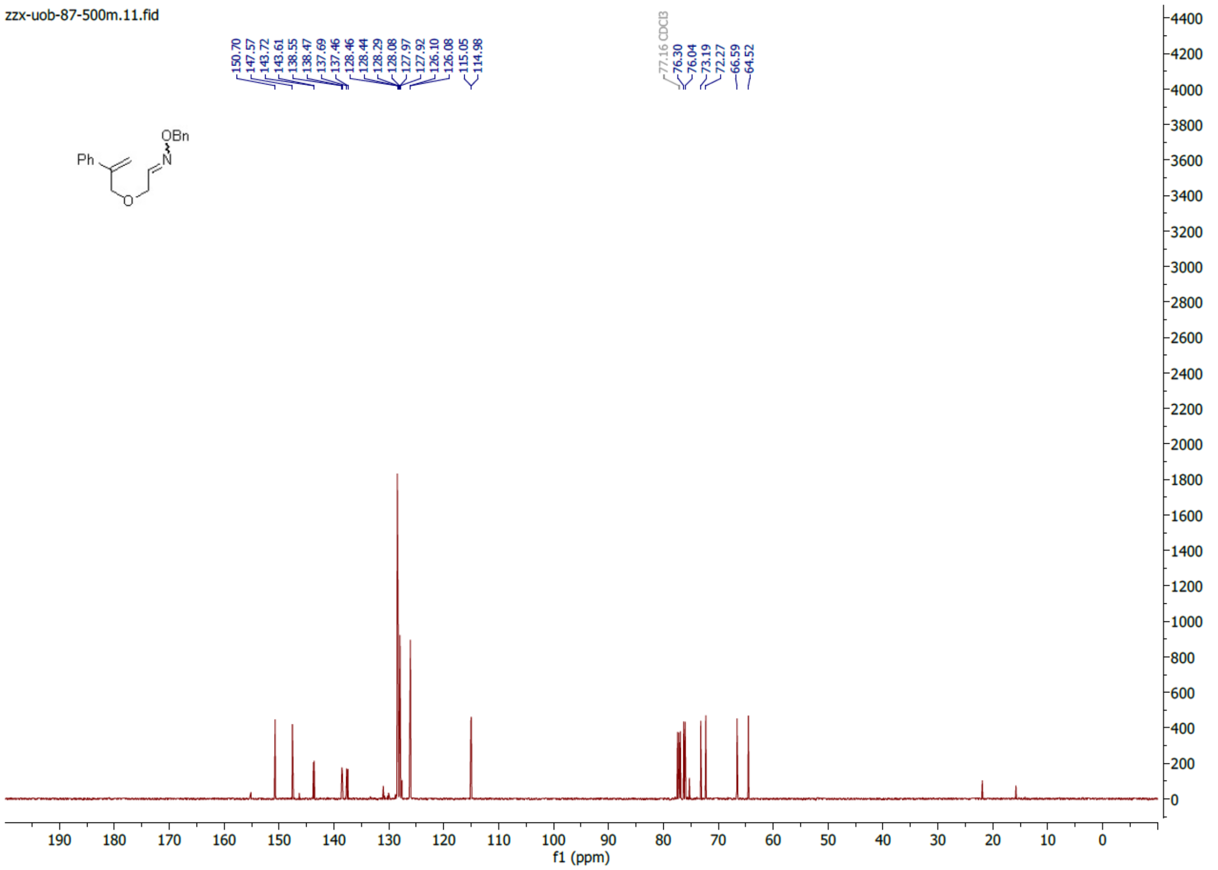

Compound S34b

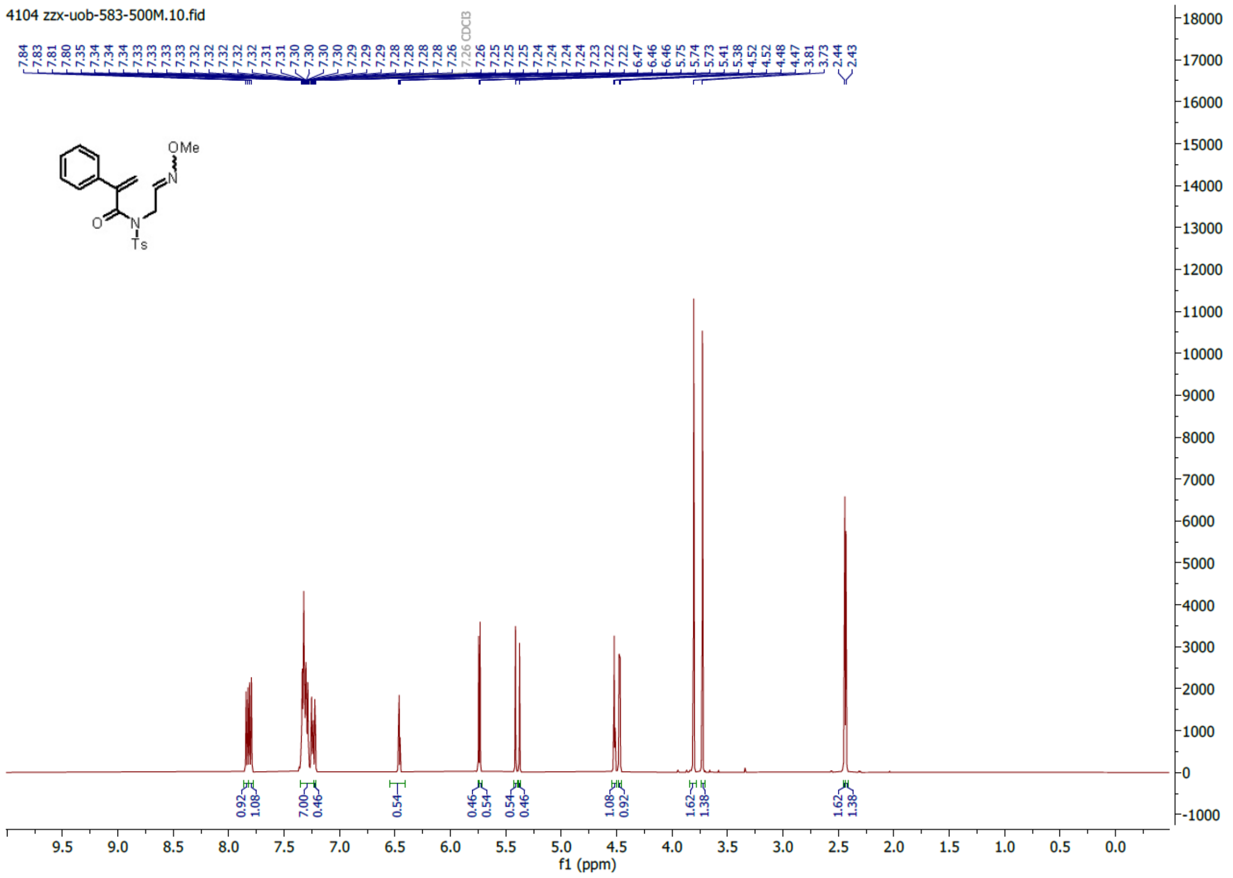

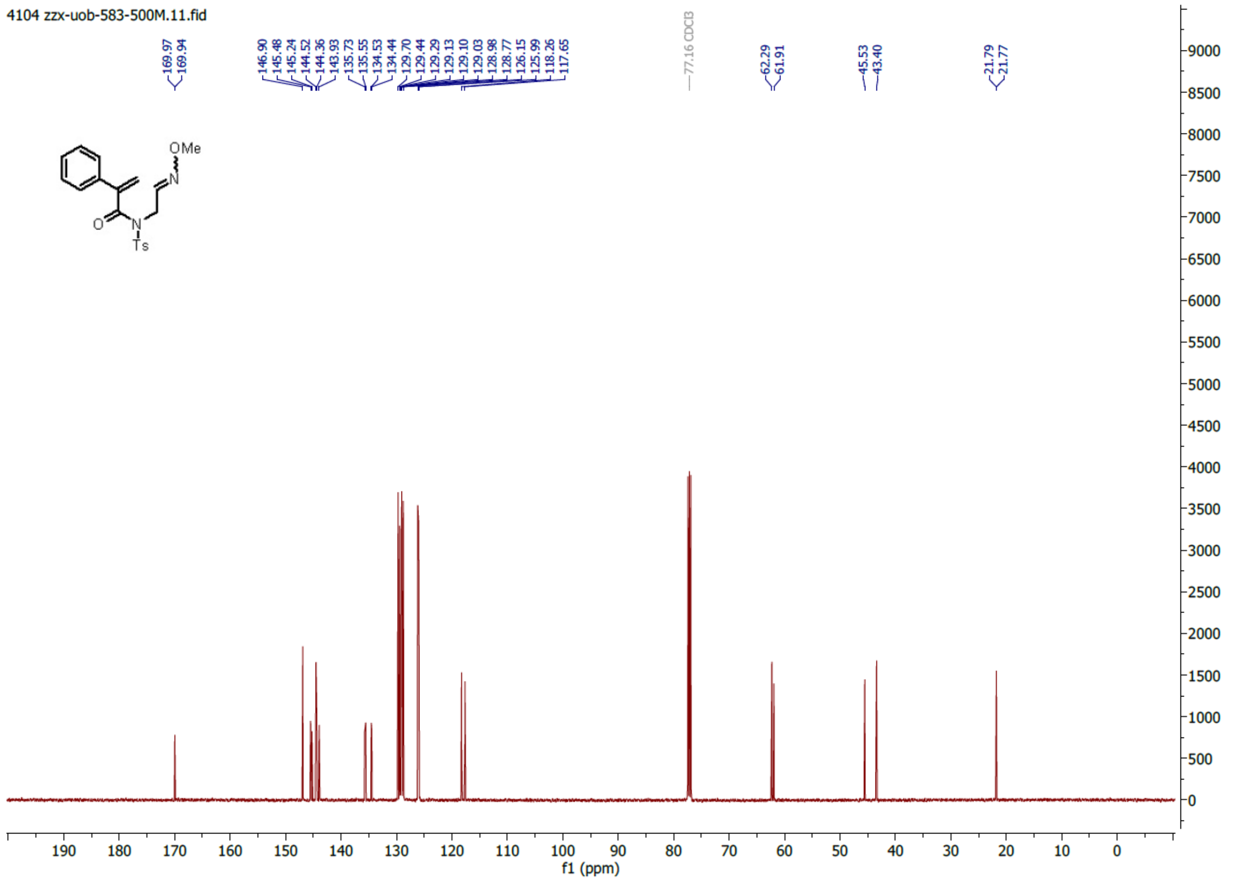

Compound S35b

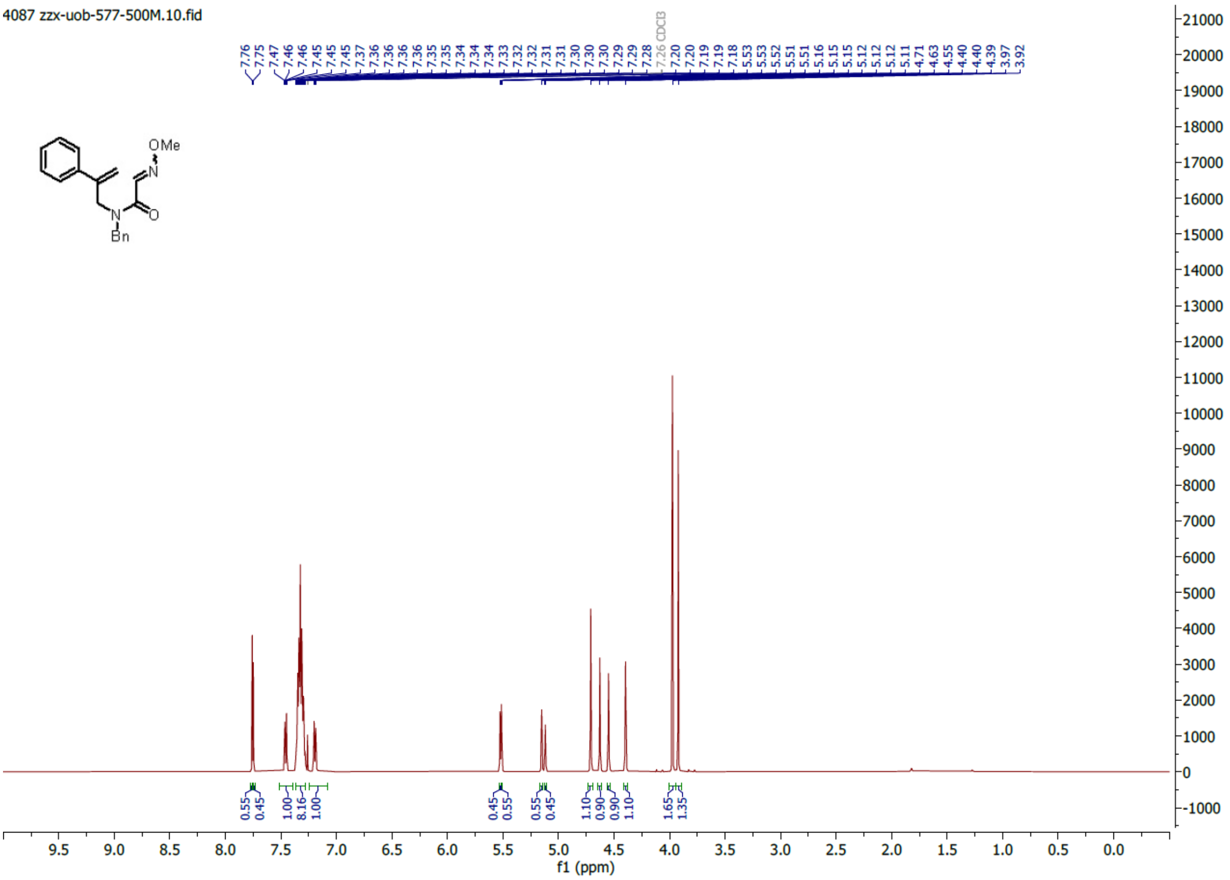

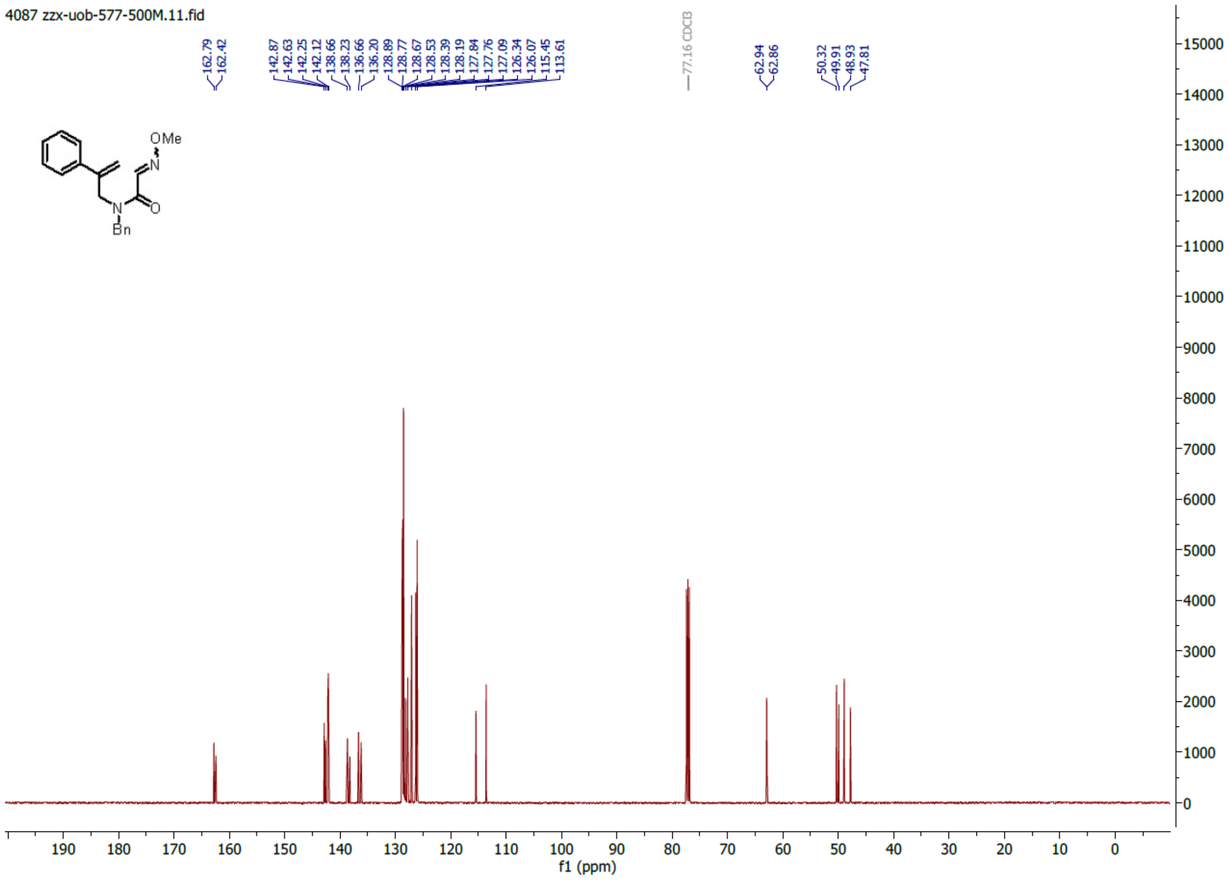

Compound S36a

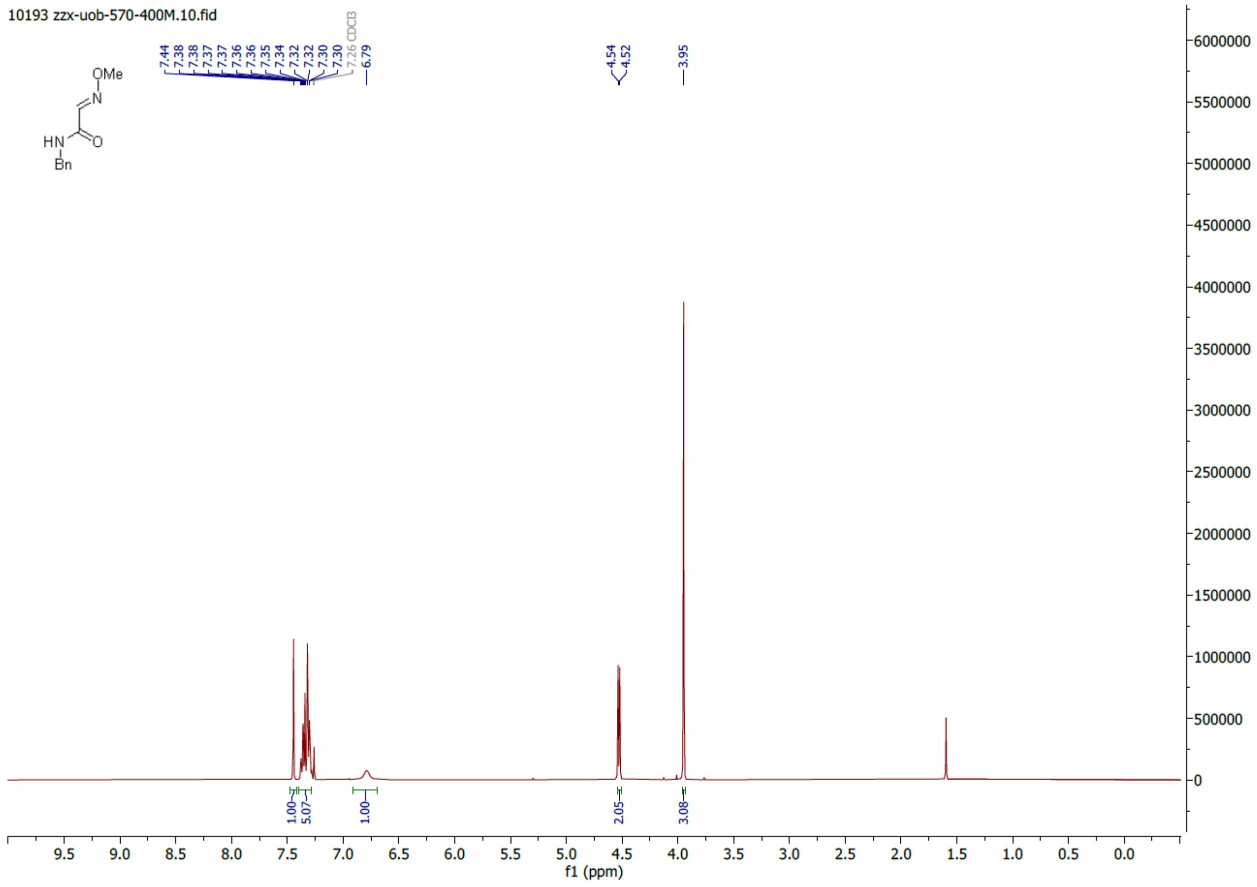

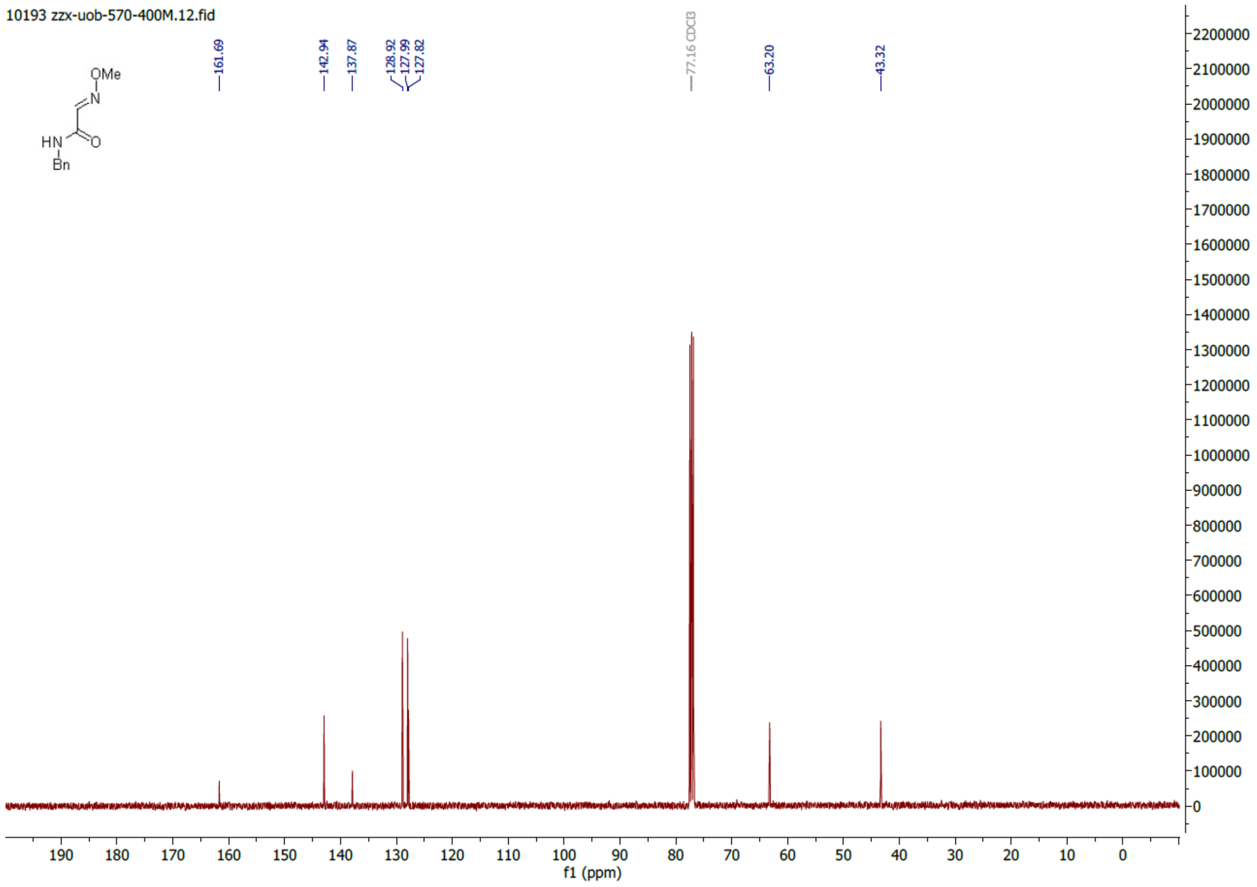

Compound S36b

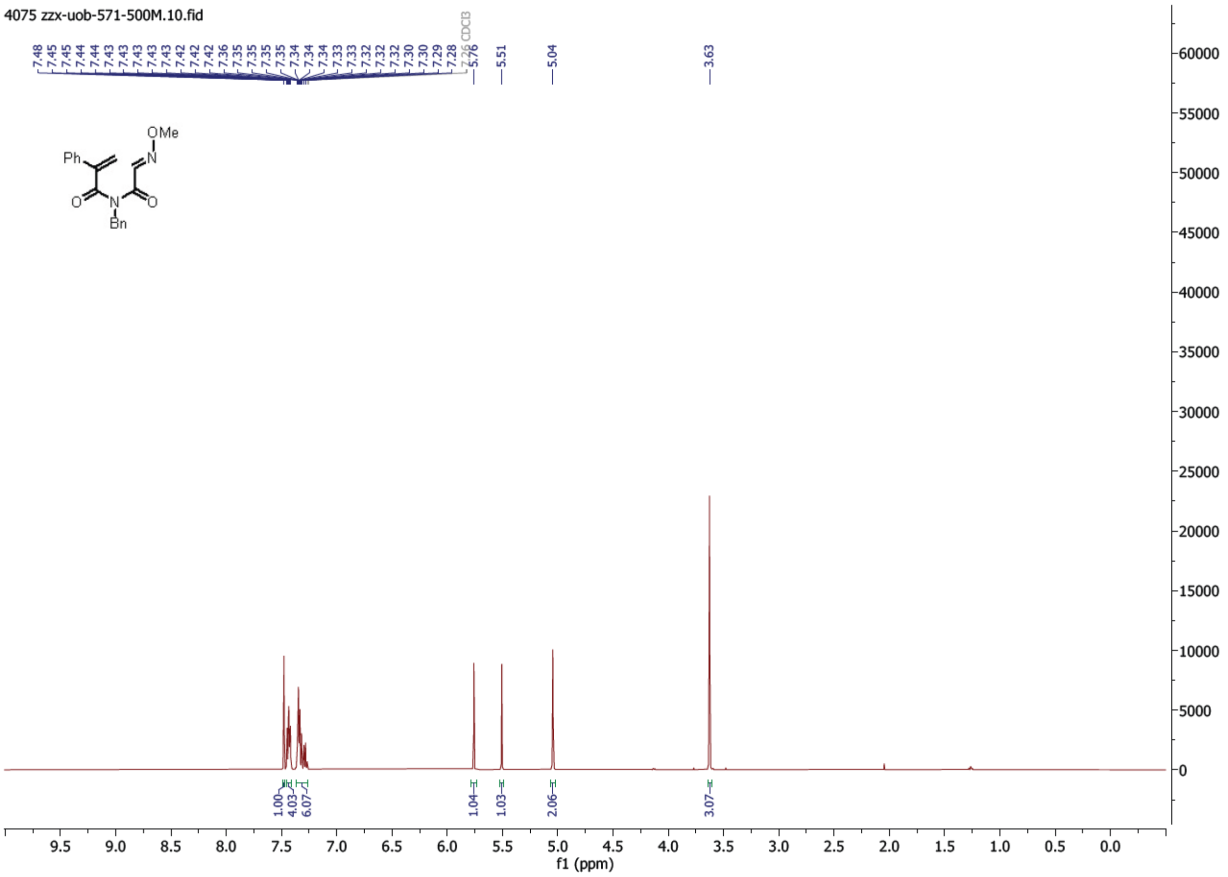

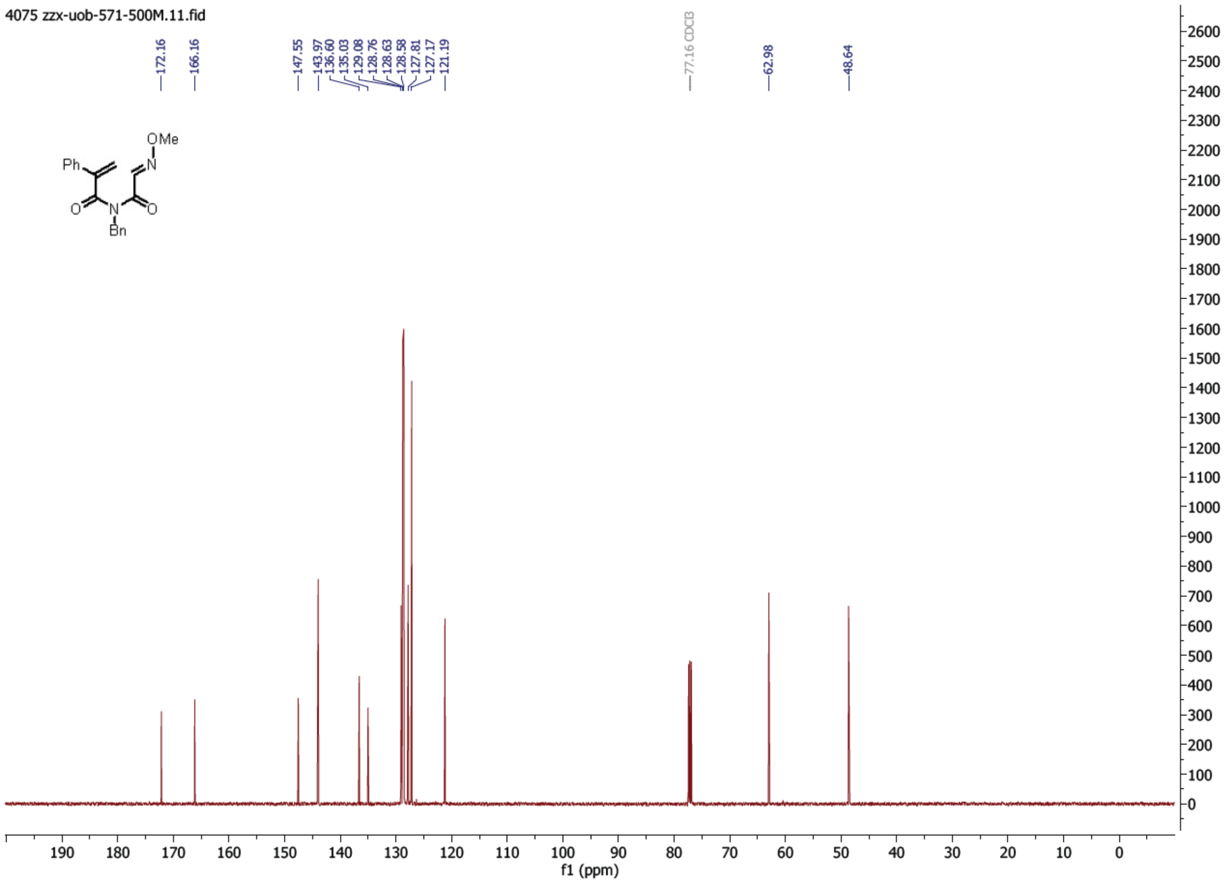

Compound S37b

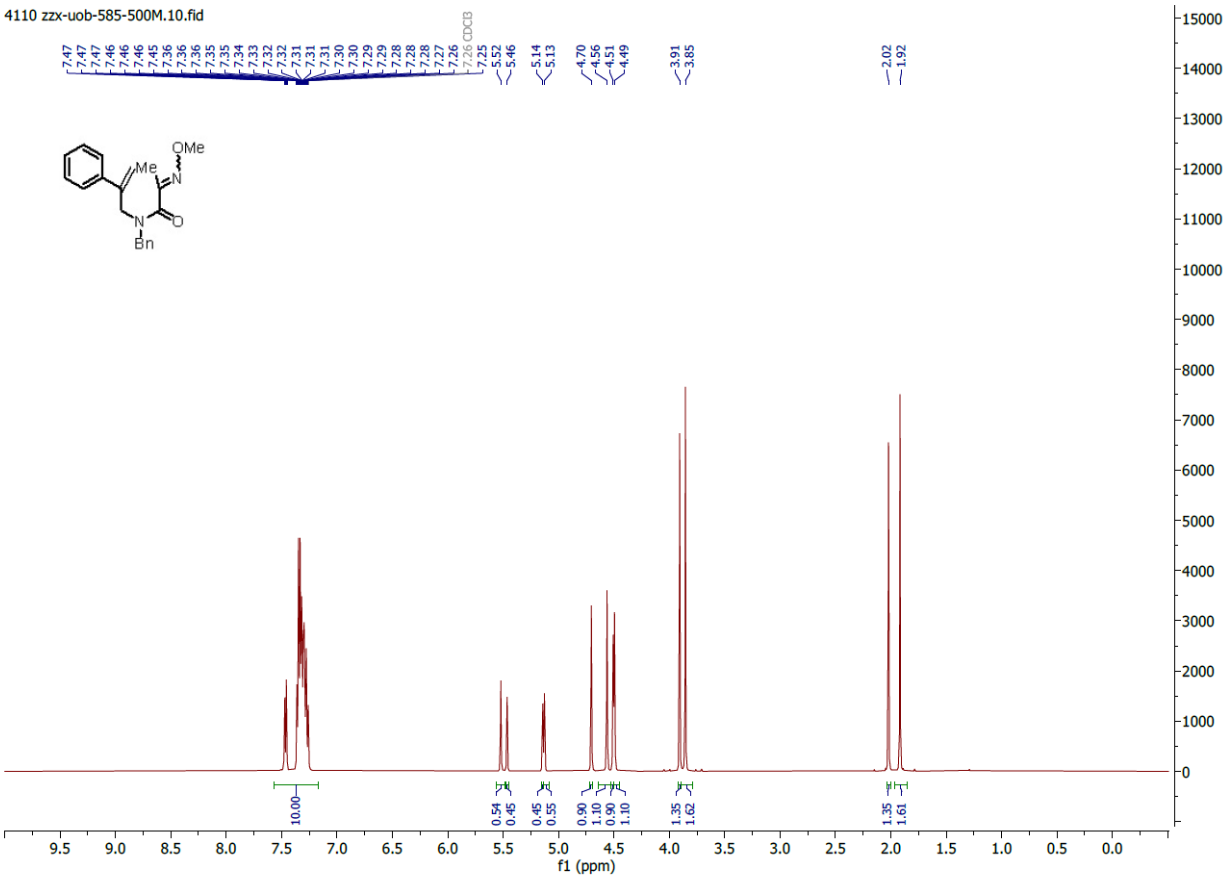

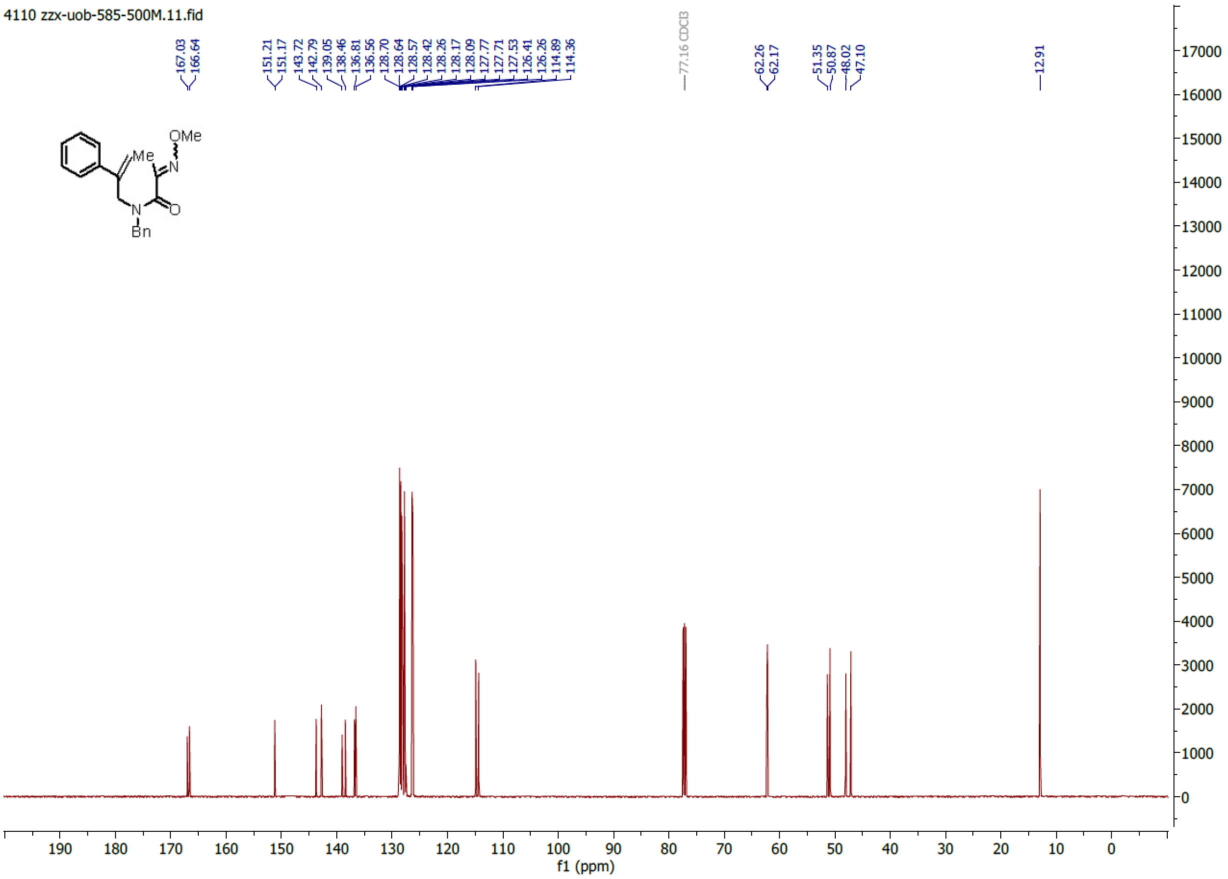

Compound S38b

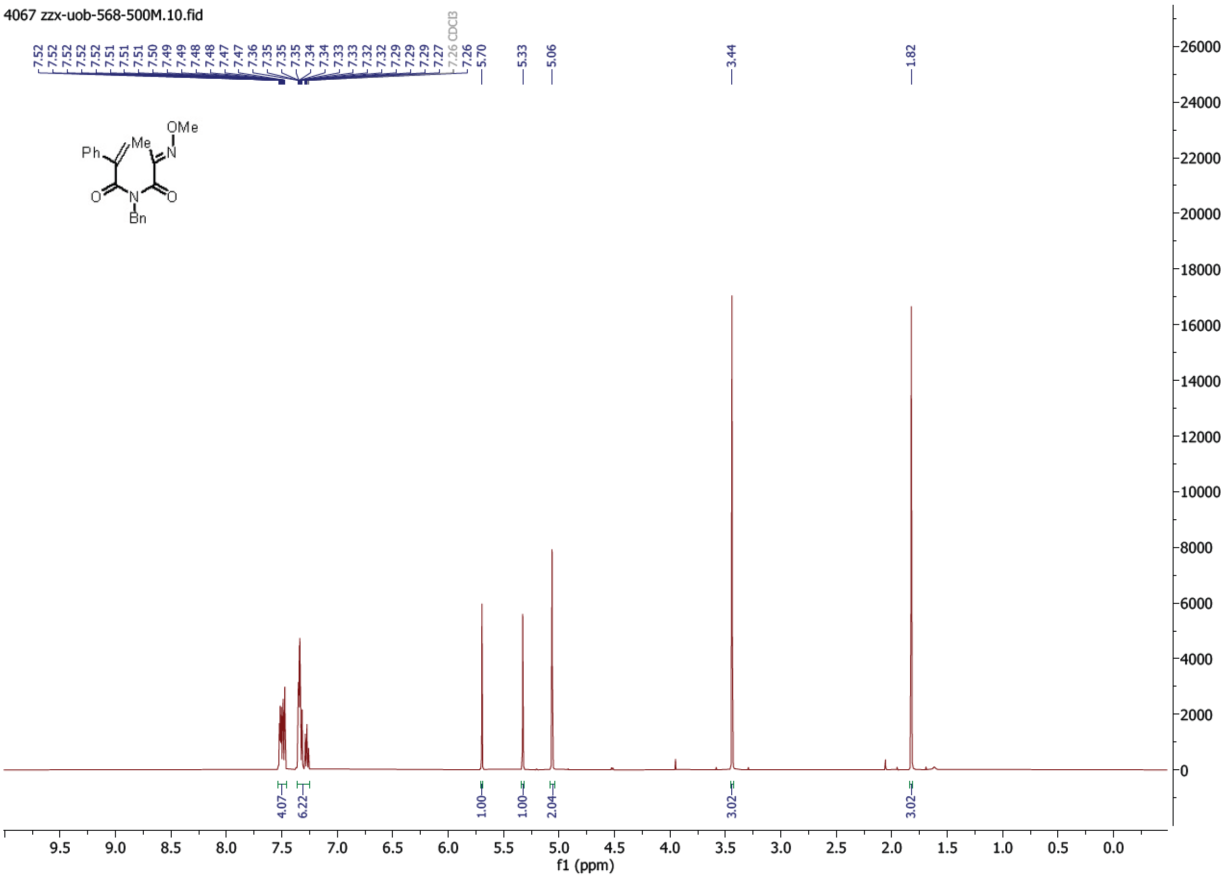

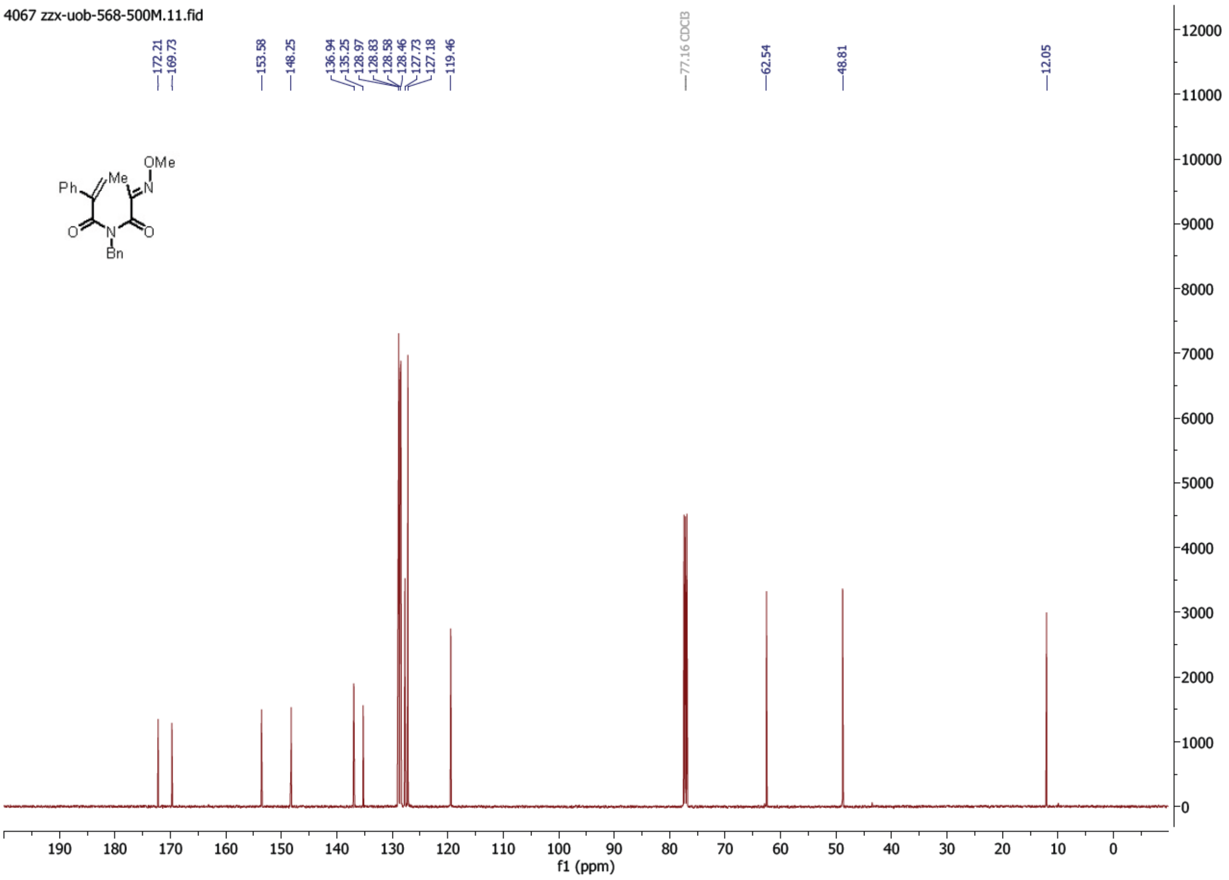

Compound S39a

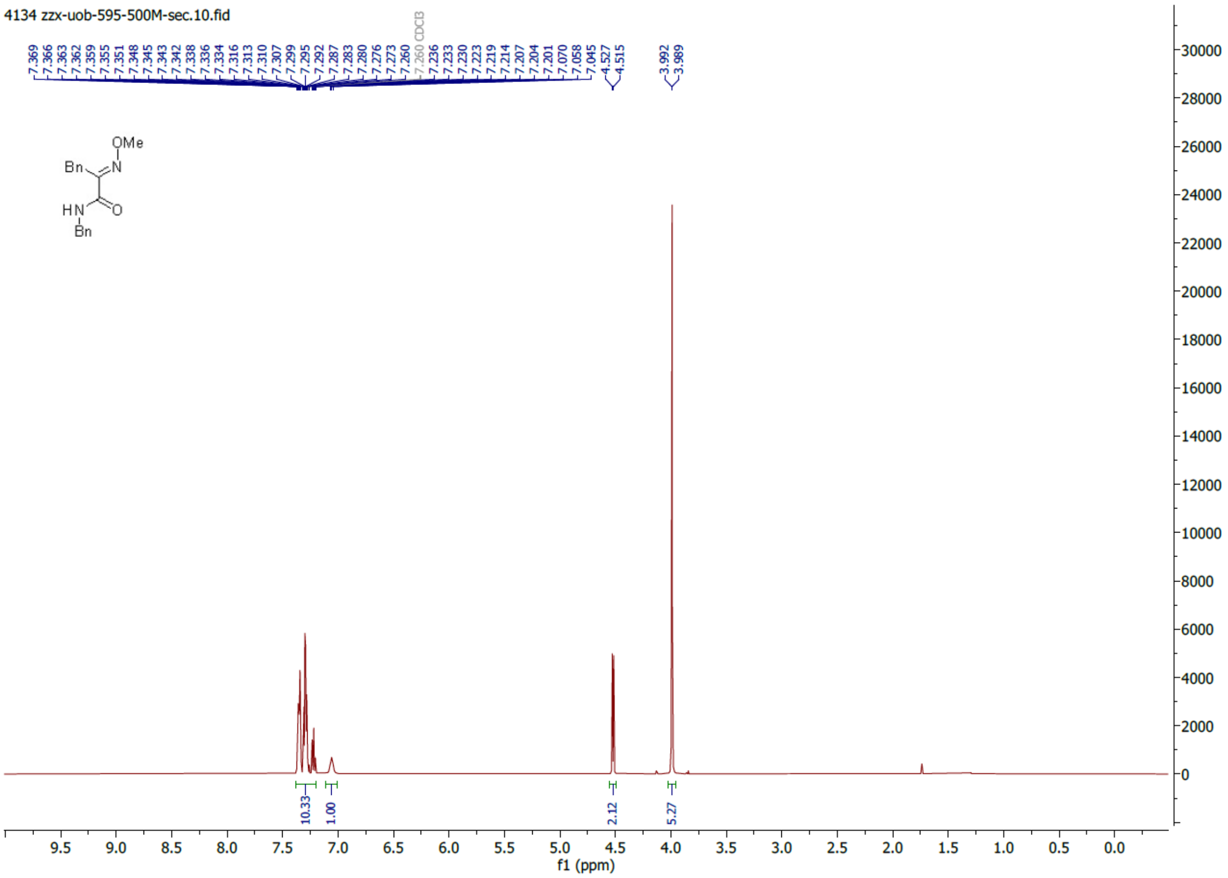

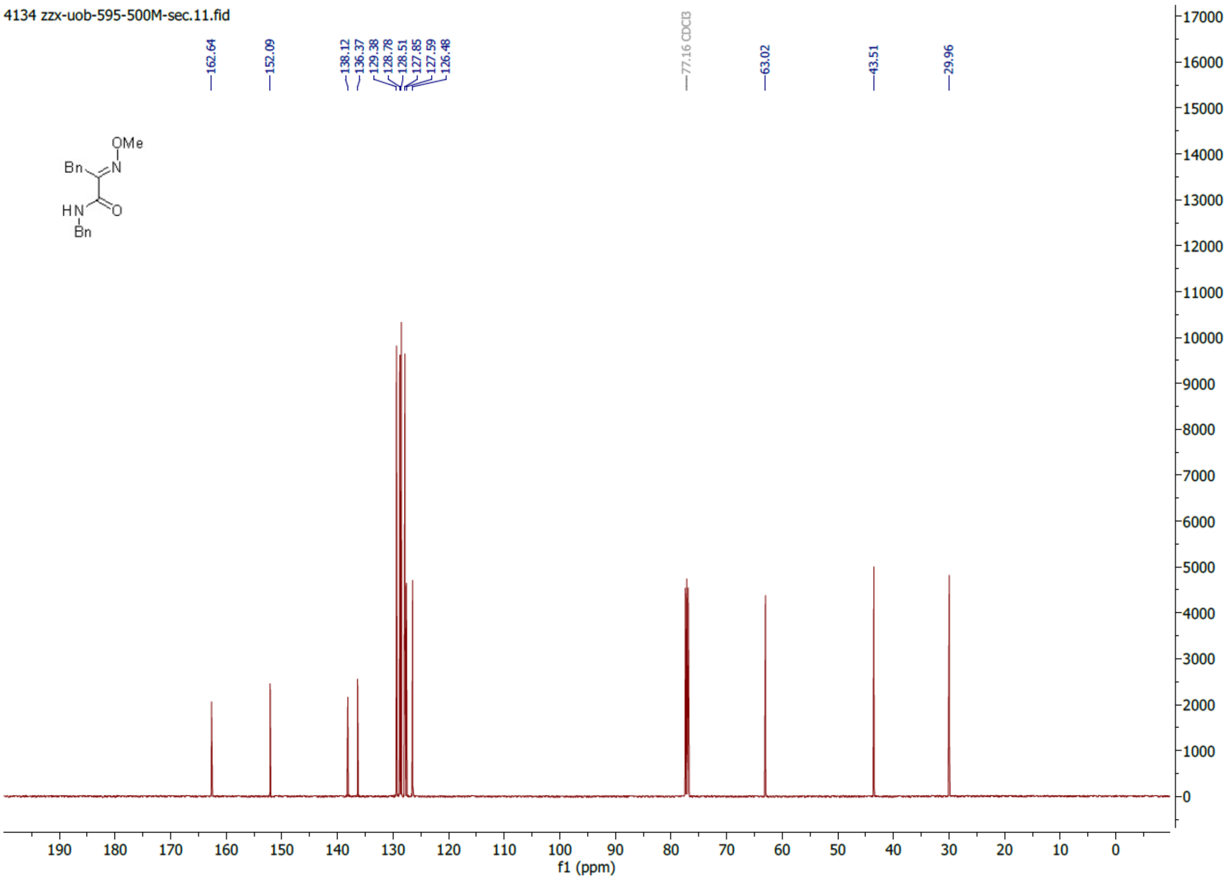

Compound S39b

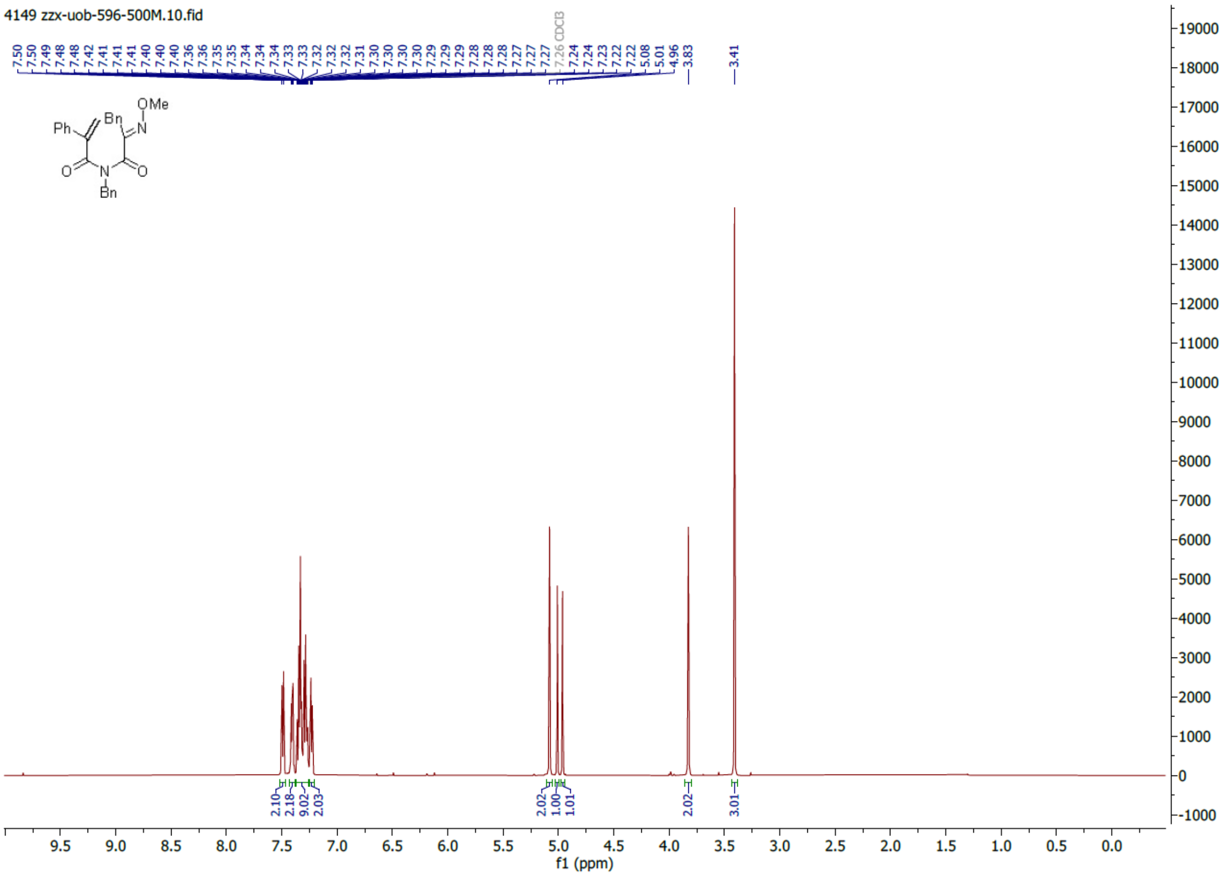

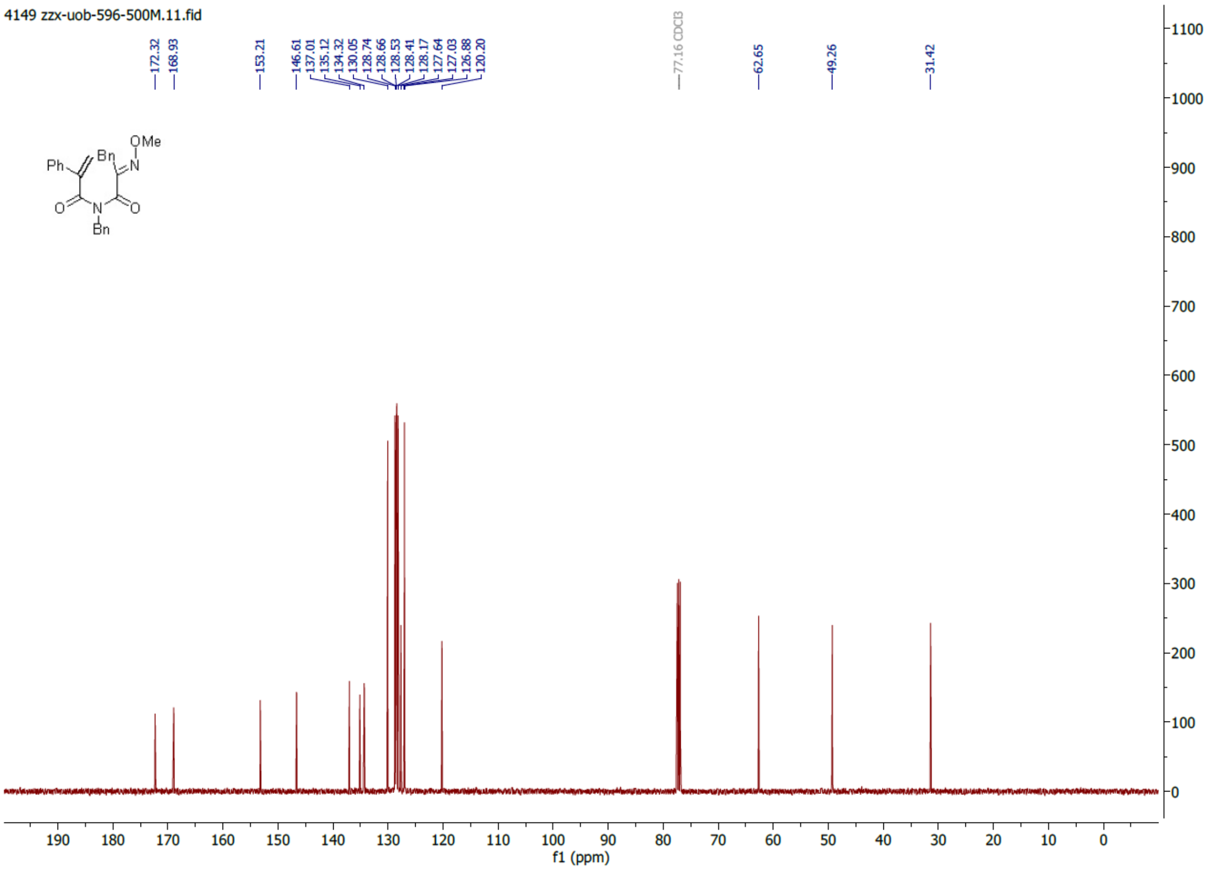

Compound S40a

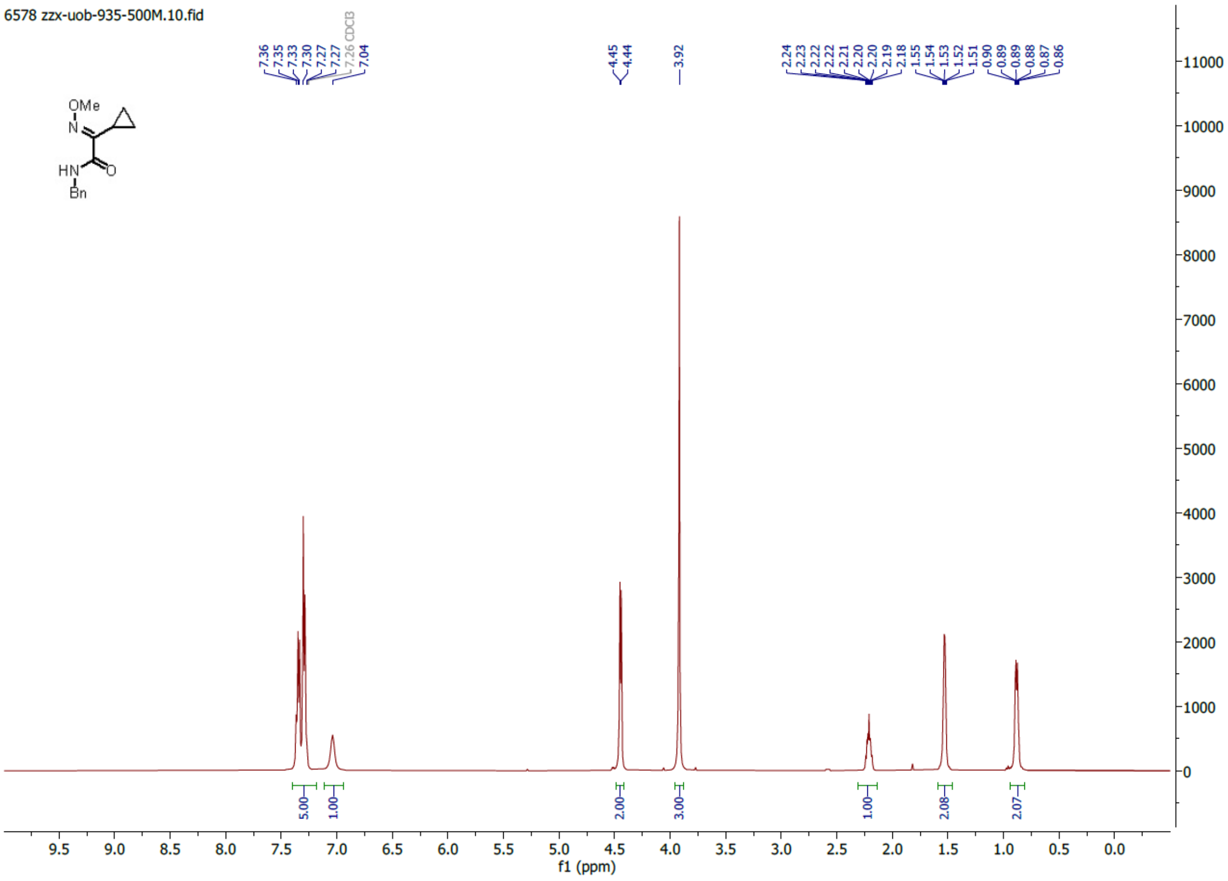

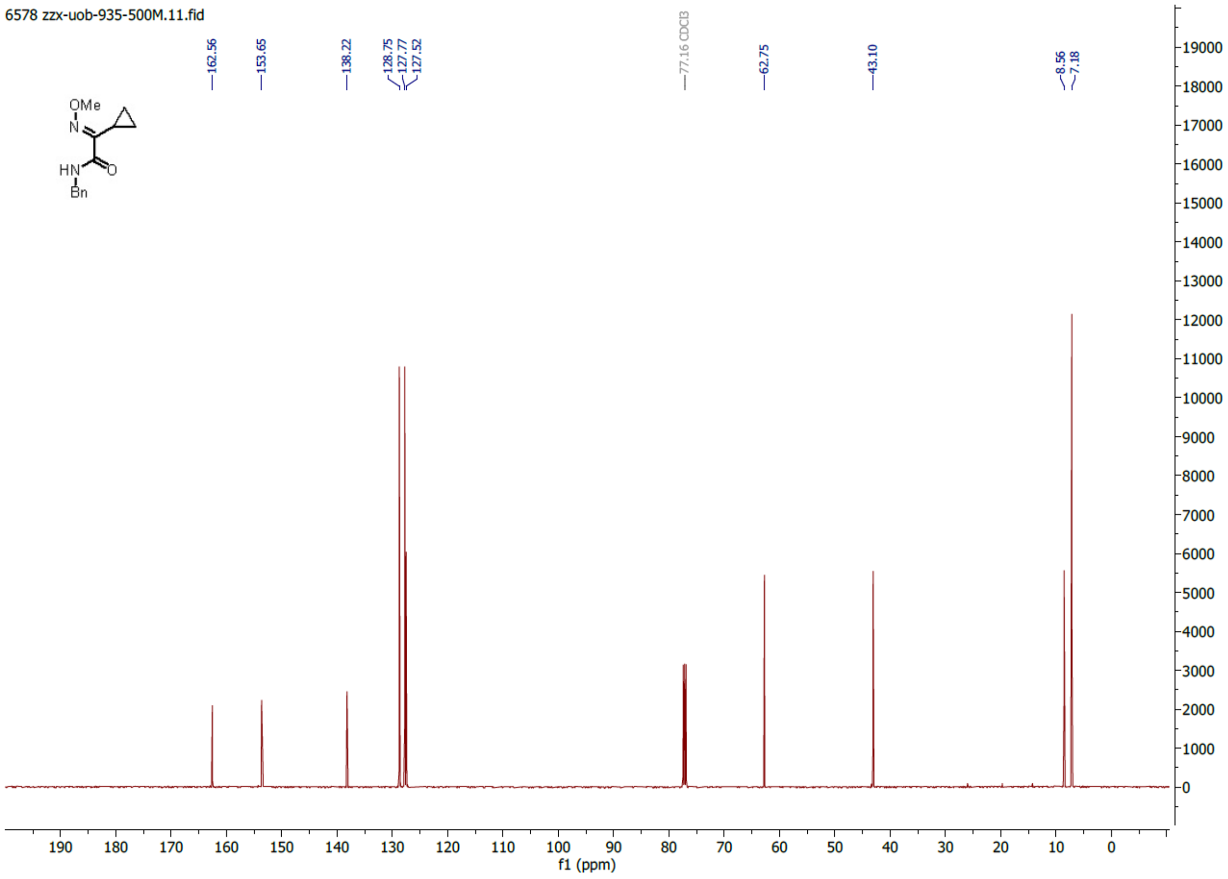

Compound S40b

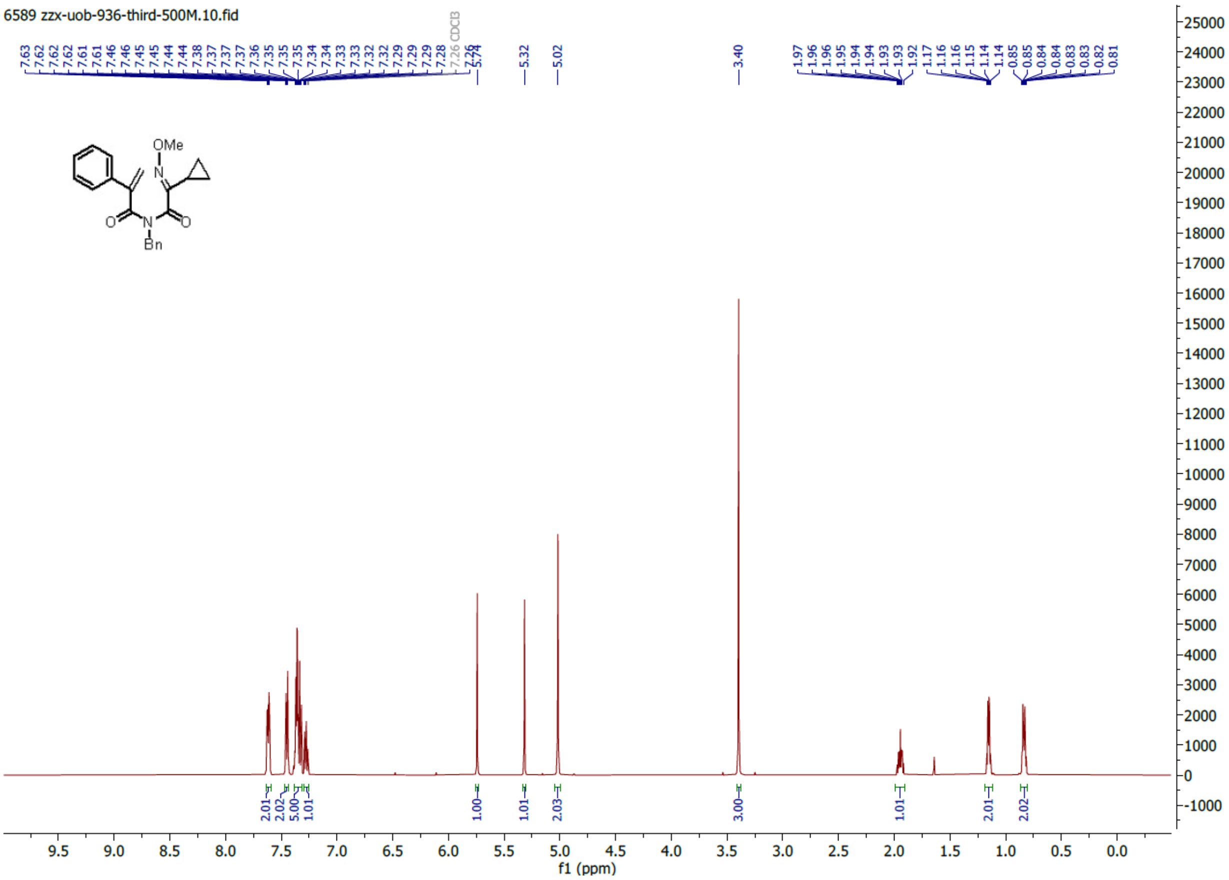

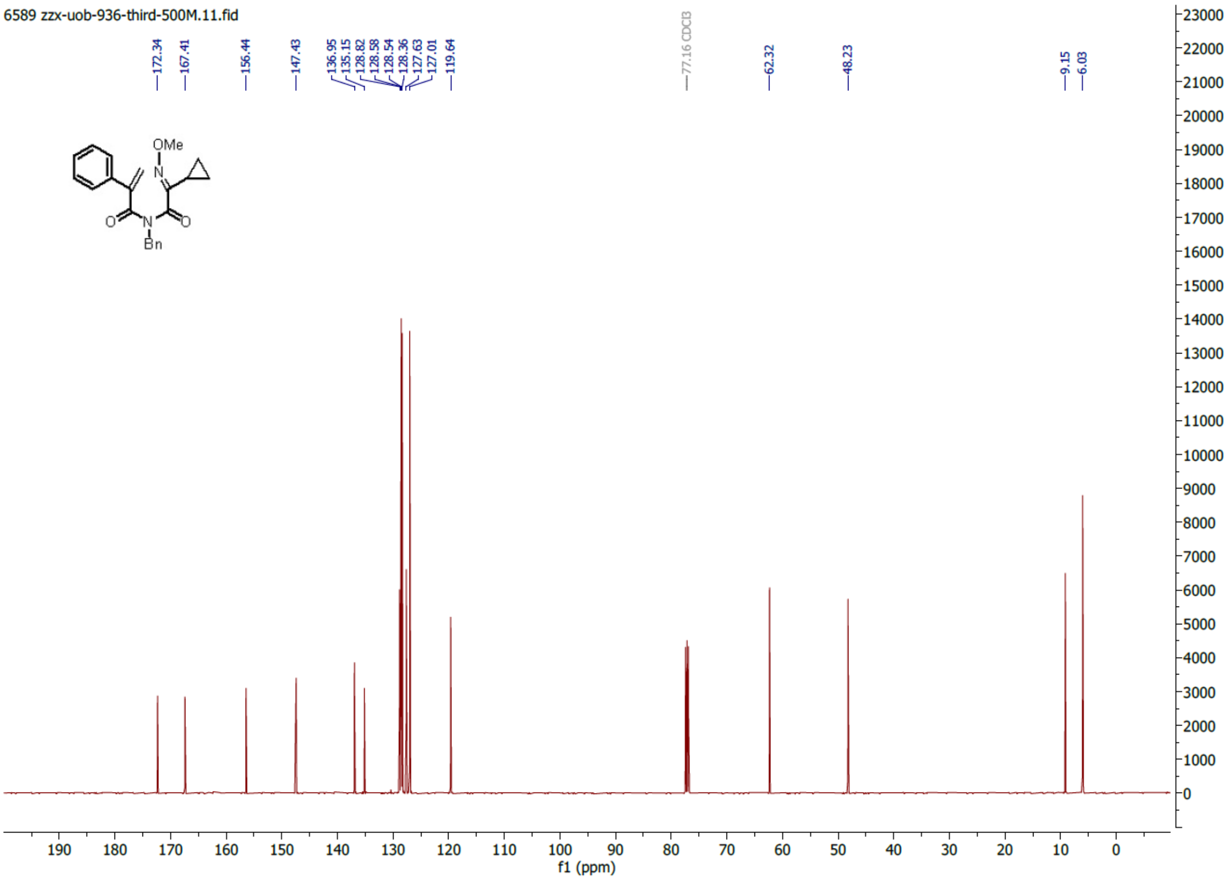

Compound S41a

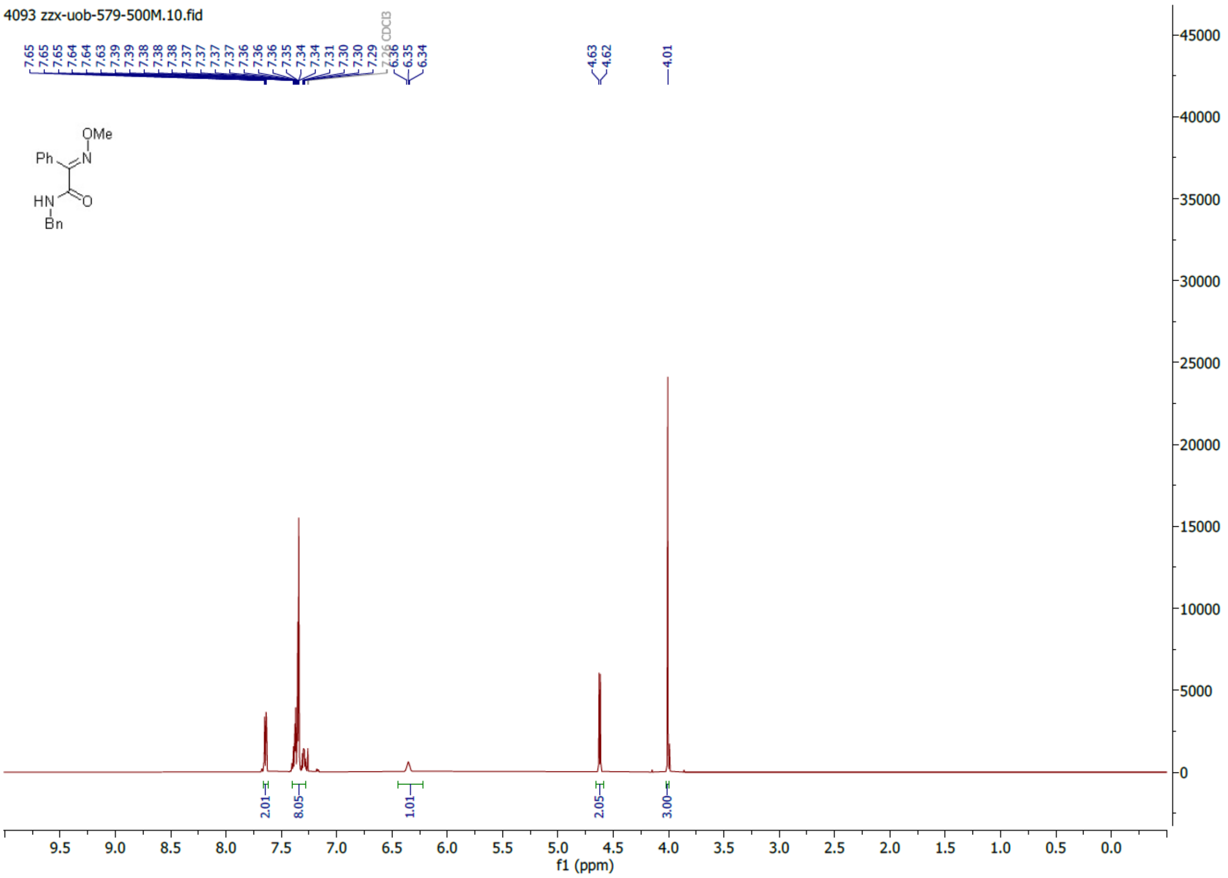

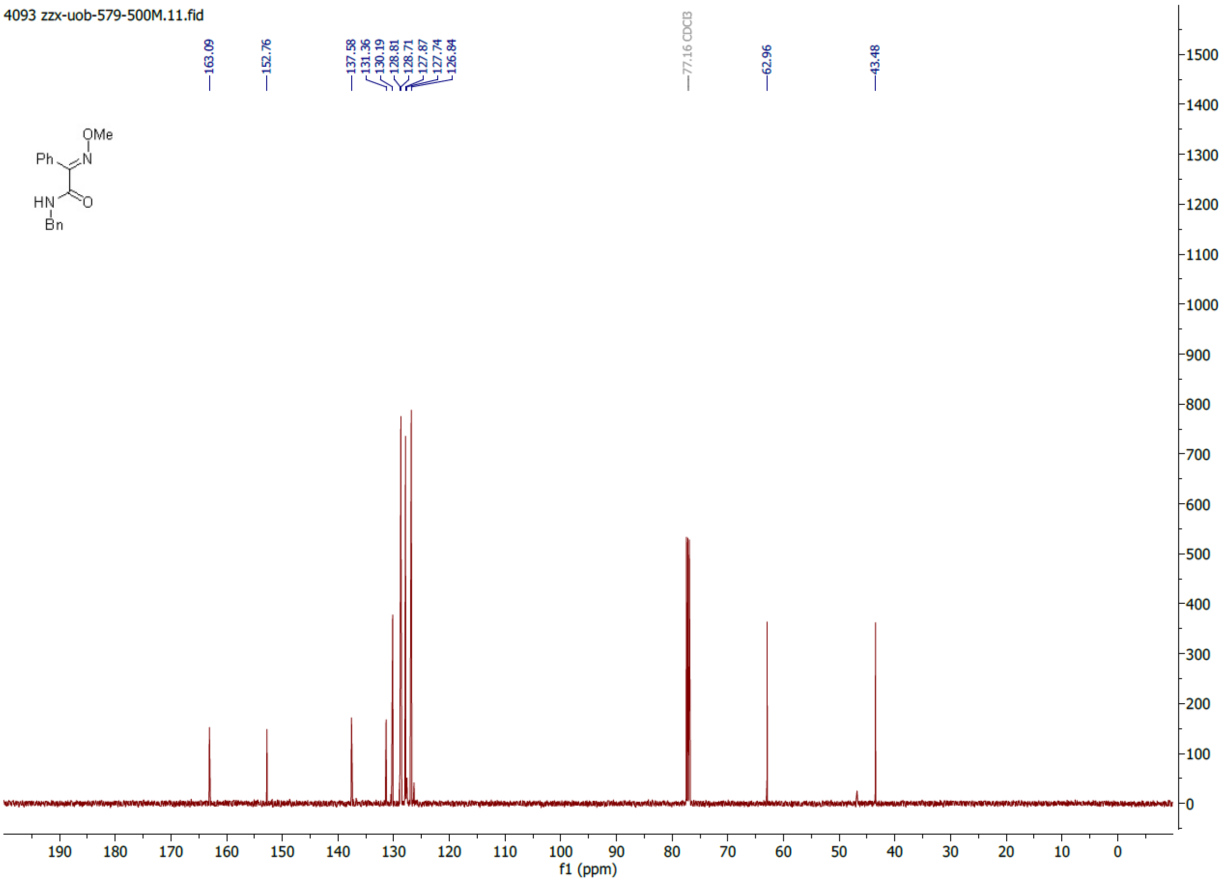

Compound S41b

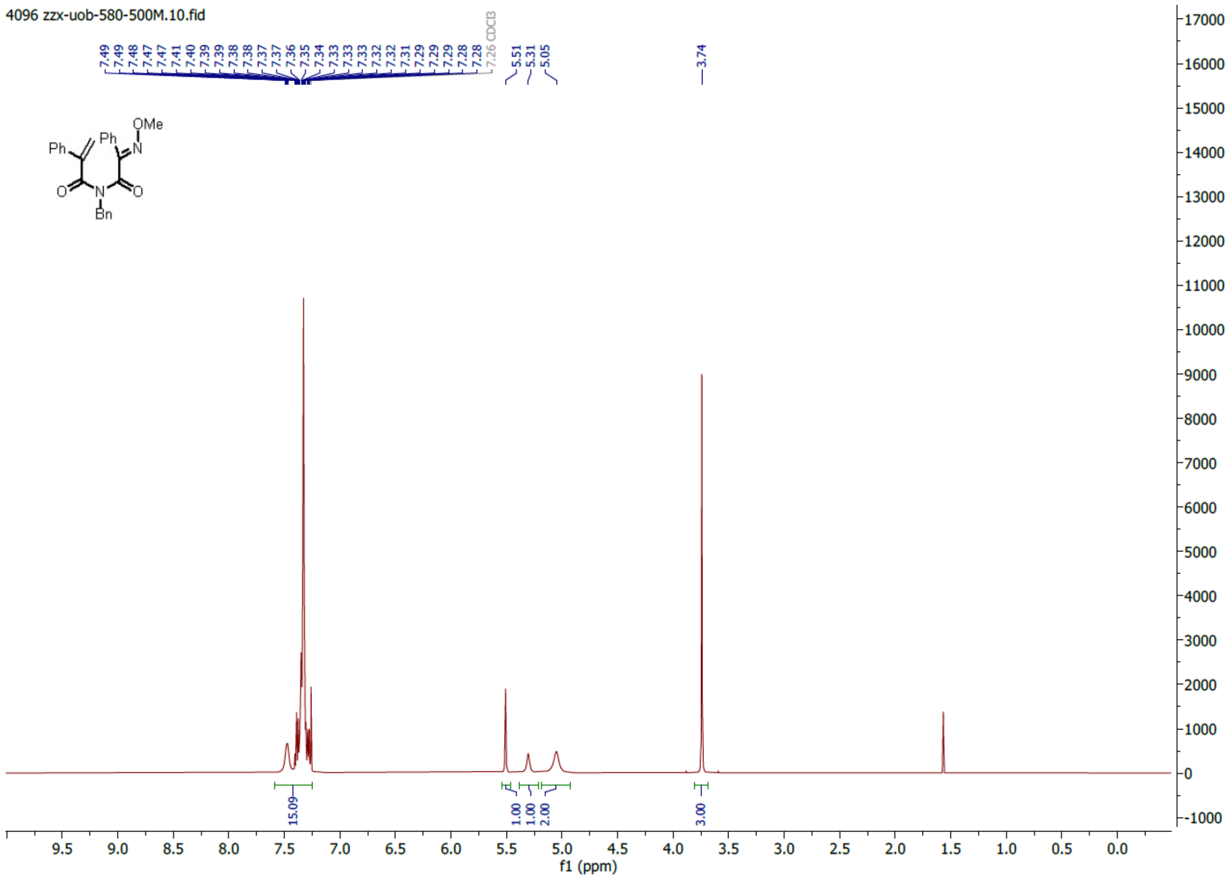

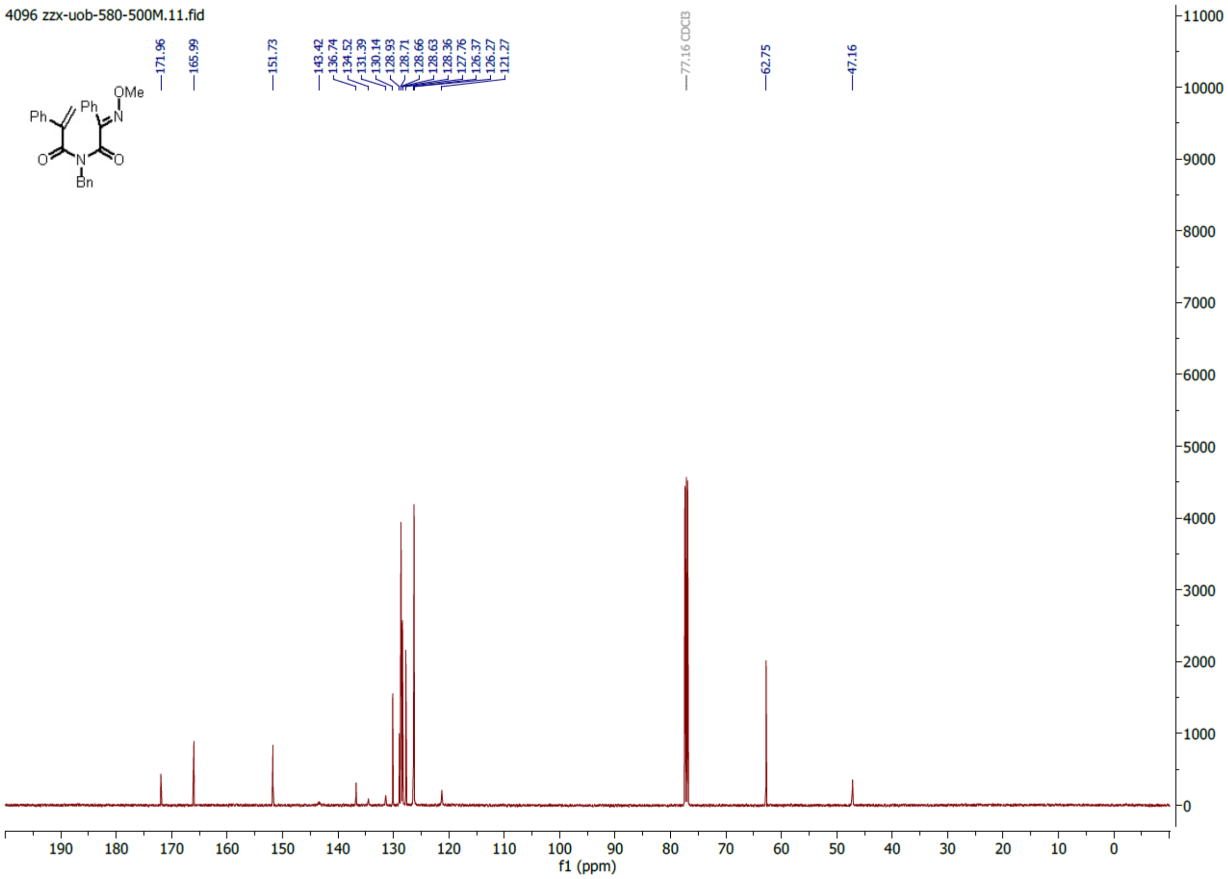

Compound S42a’

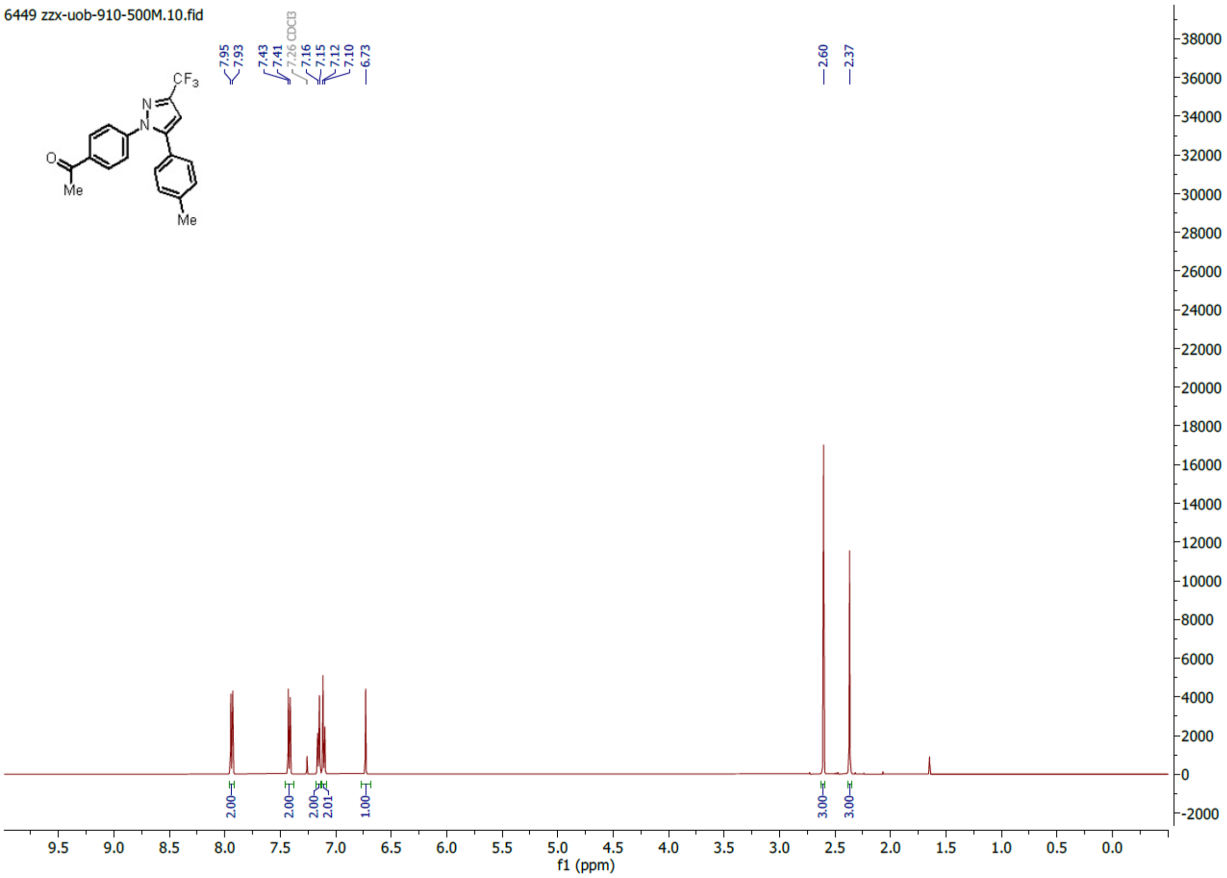

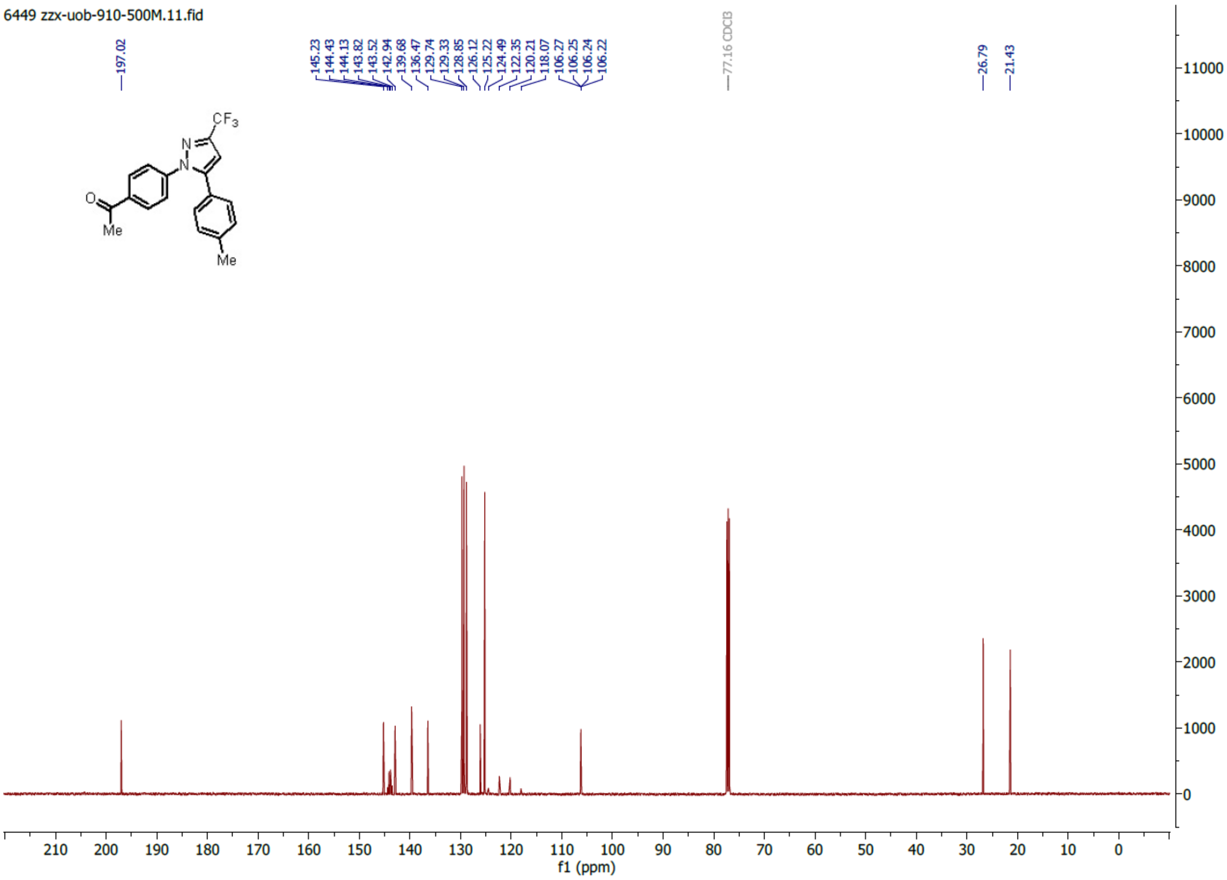

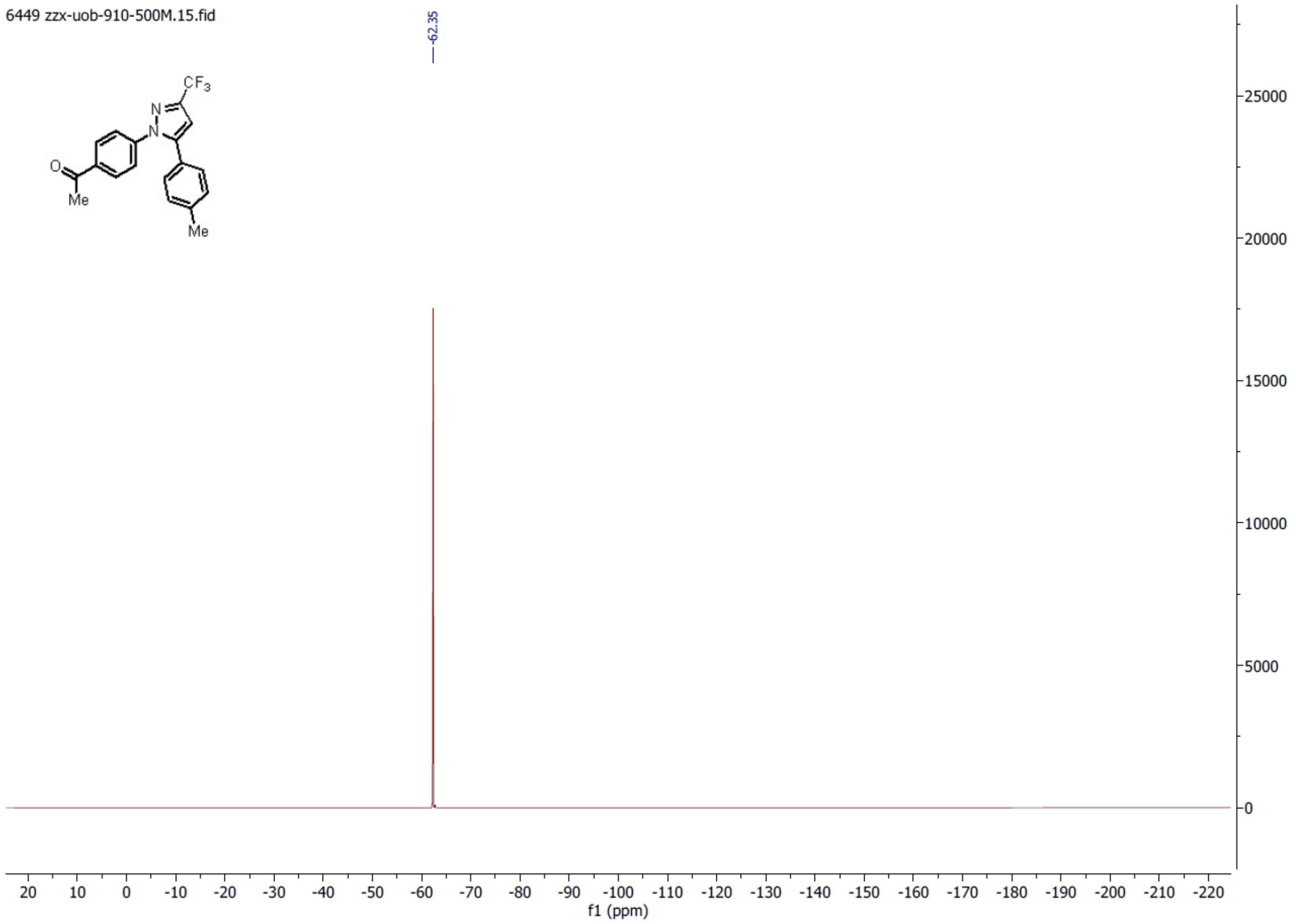
6449 zzx-uob-910-500M.15.fid
—62.35
CF3
N
N
O
Me
Me
25000
20000
15000
10000
5000
0
20 10 0 -10 -20 -30 -40 -50 -60 -70 -80 -90 -100 -110 -120 -130 -140 -150 -160 -170 -180 -190 -200 -210 -220
f1 (ppm)

Compound S42a’’

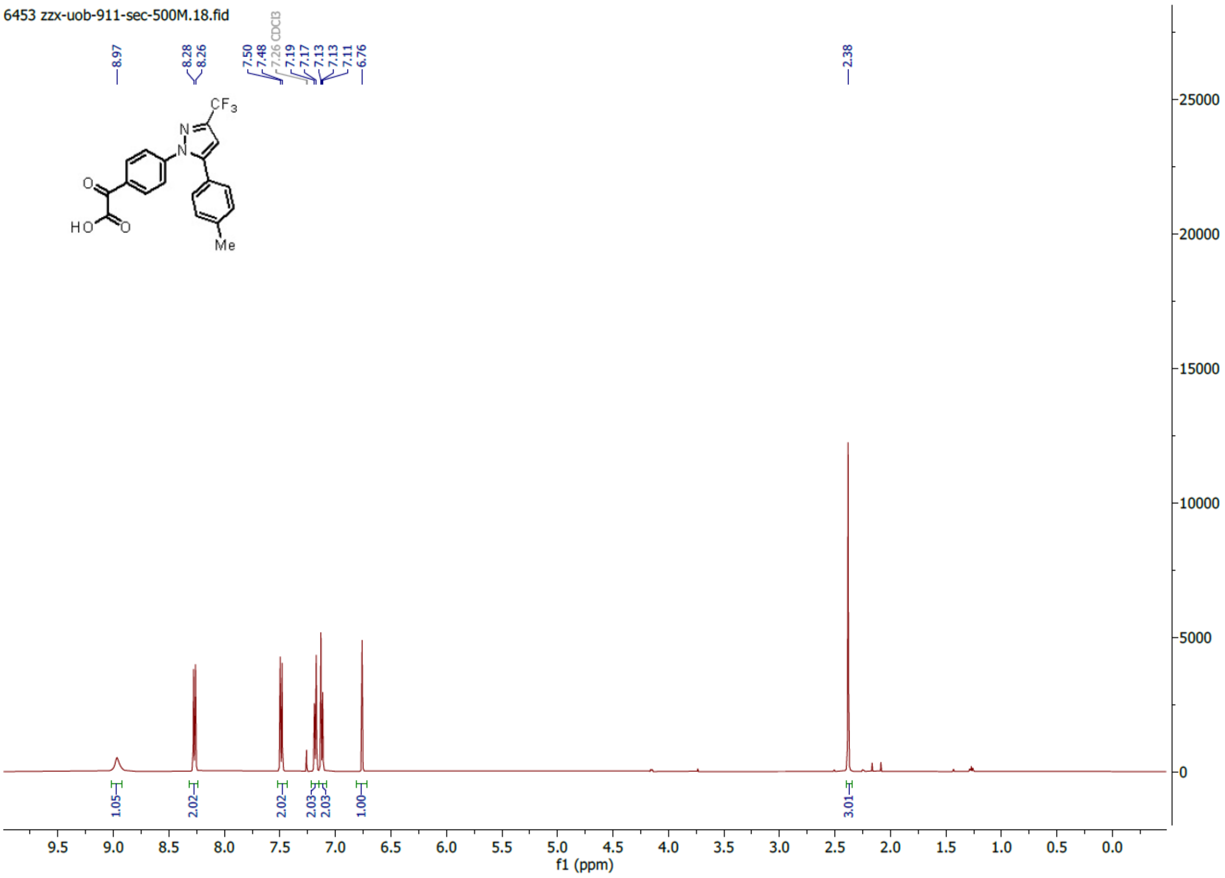

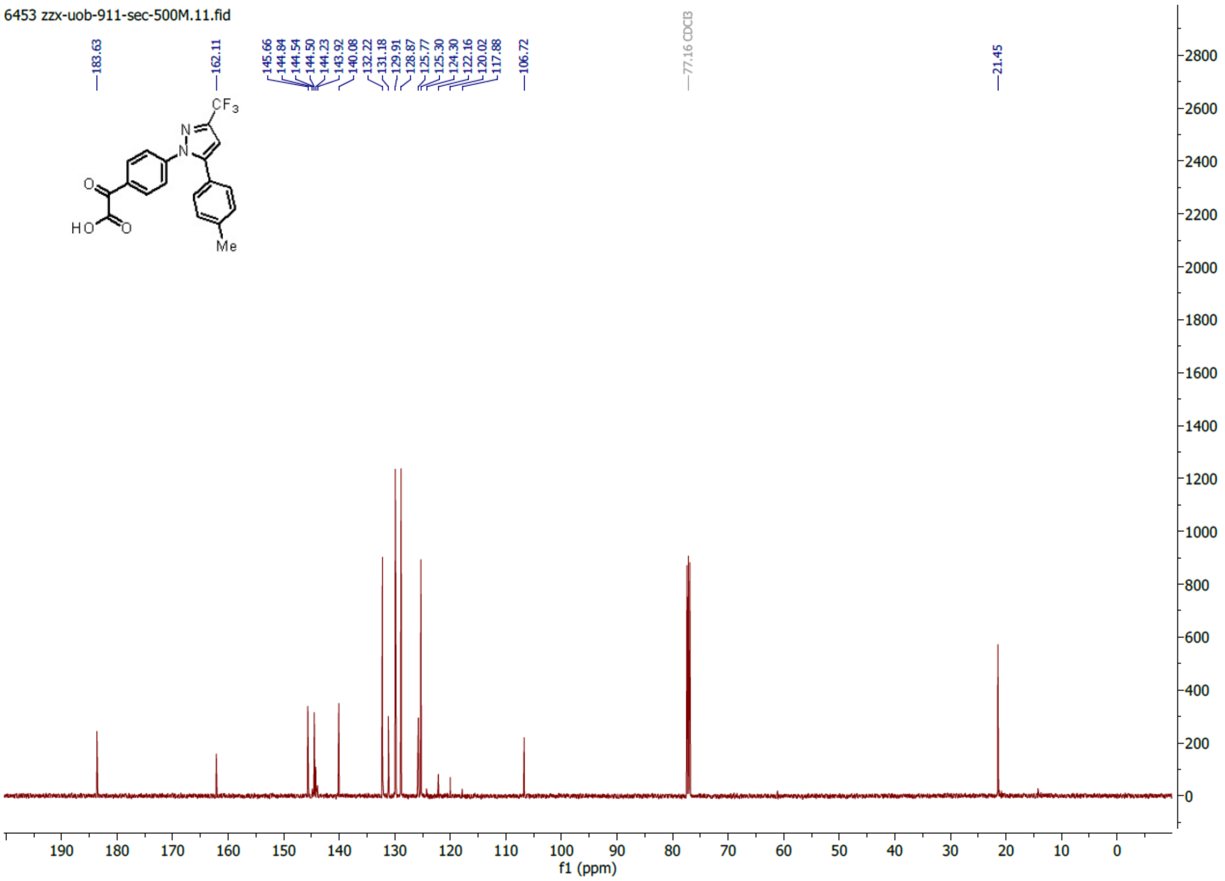

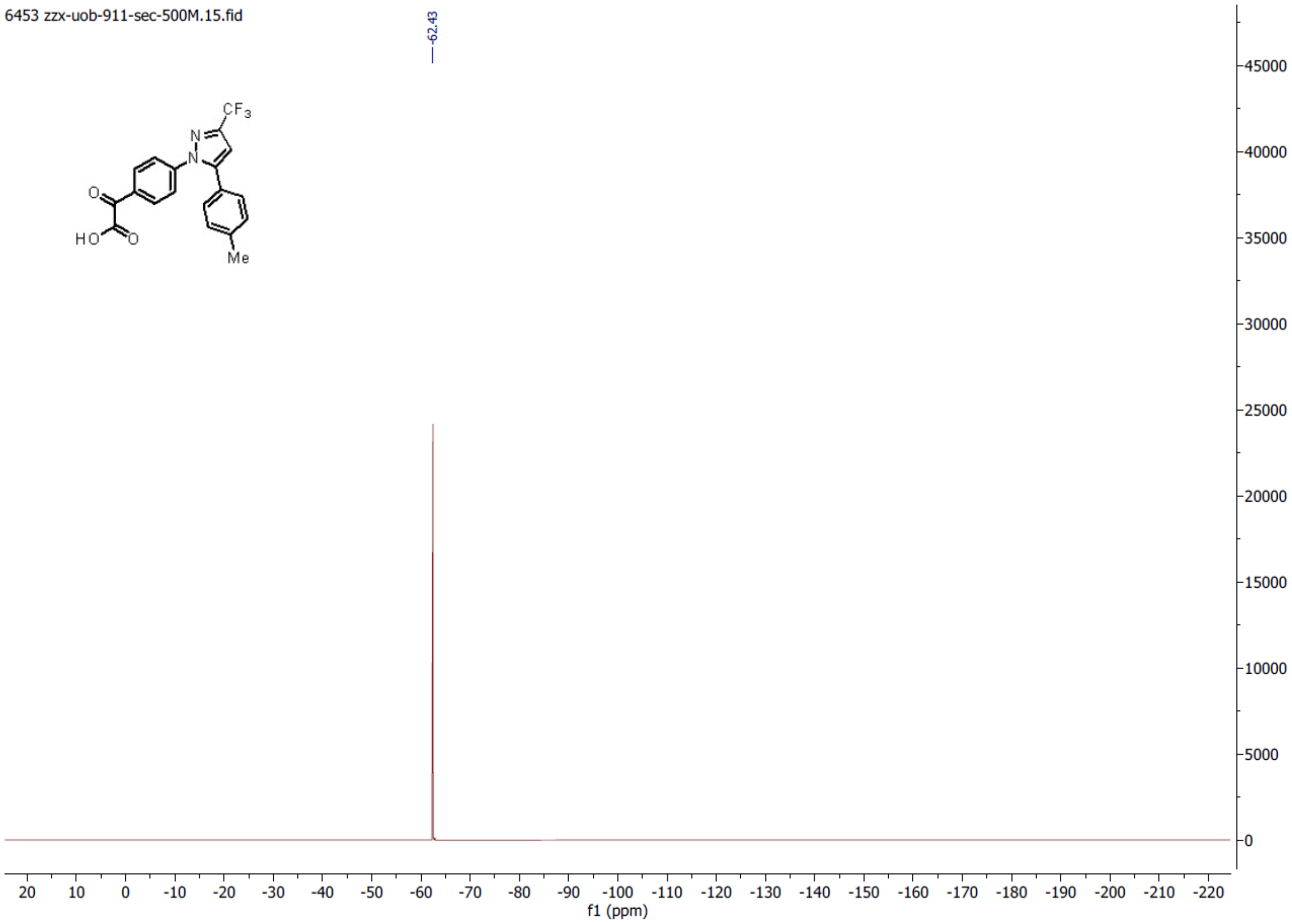
6453 zzx-uob-911-sec-500M.15.fid
-62.43
CF3
N
N
O
HO
O
Me
45000
40000
35000
30000
25000
20000
15000
10000
5000
0
20 10 0 -10 -20 -30 -40 -50 -60 -70 -80 -90 -100 -110 -120 -130 -140 -150 -160 -170 -180 -190 -200 -210 -220
f1 (ppm)

Compound S42a

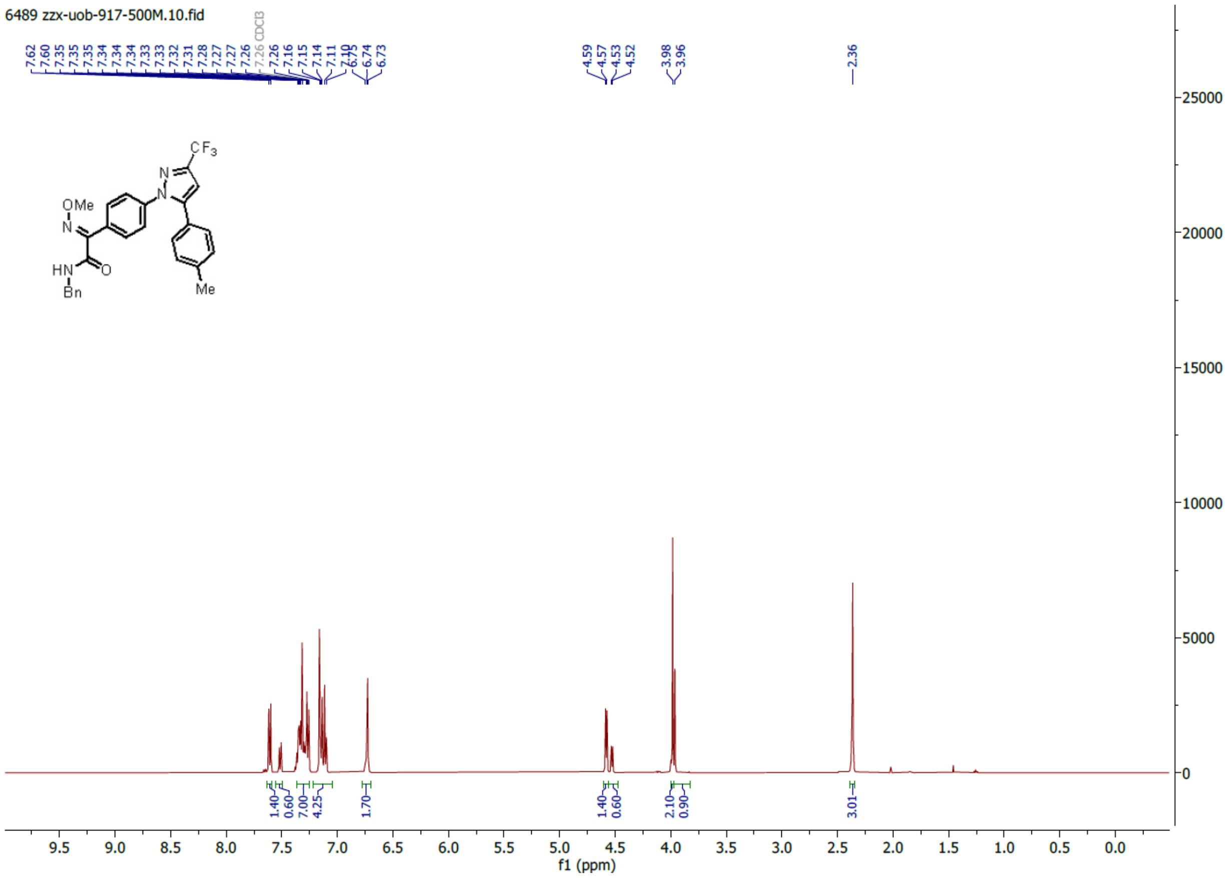

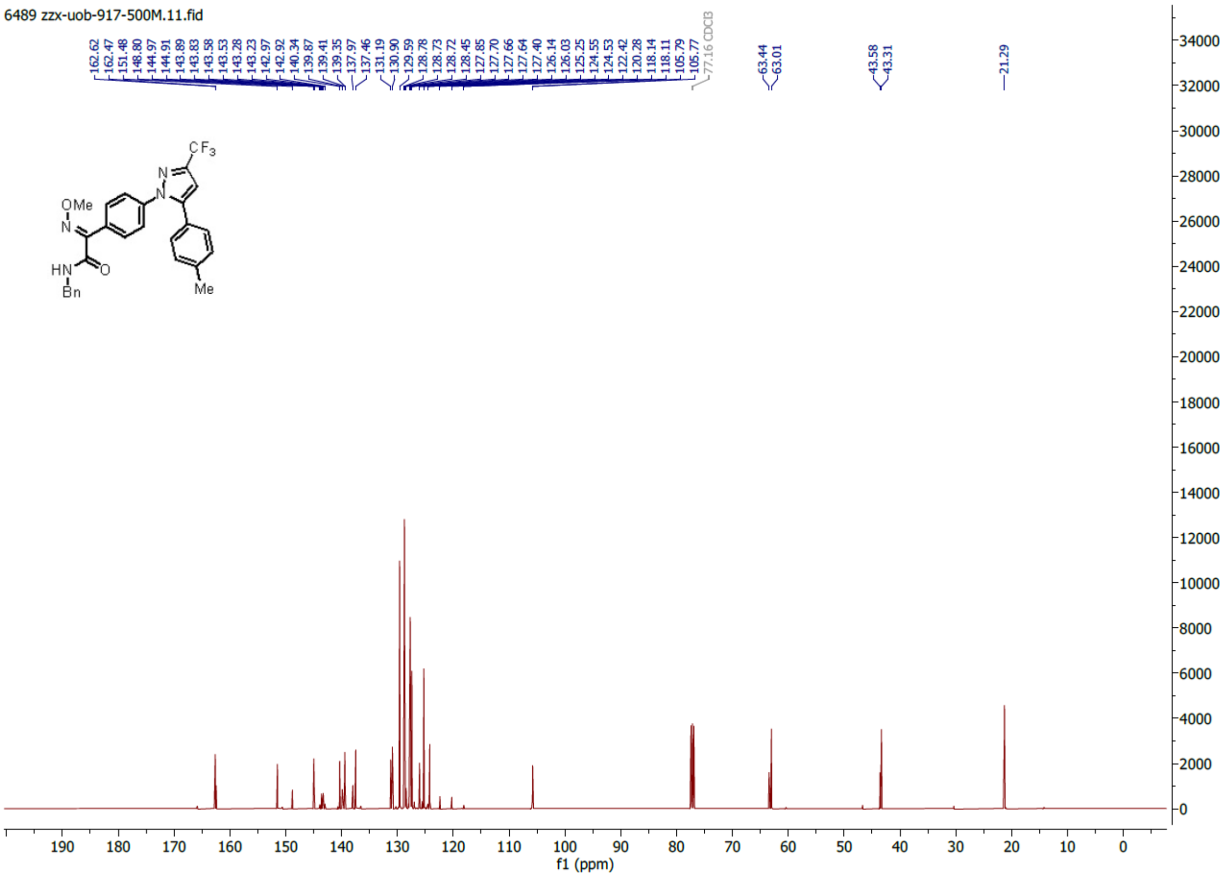

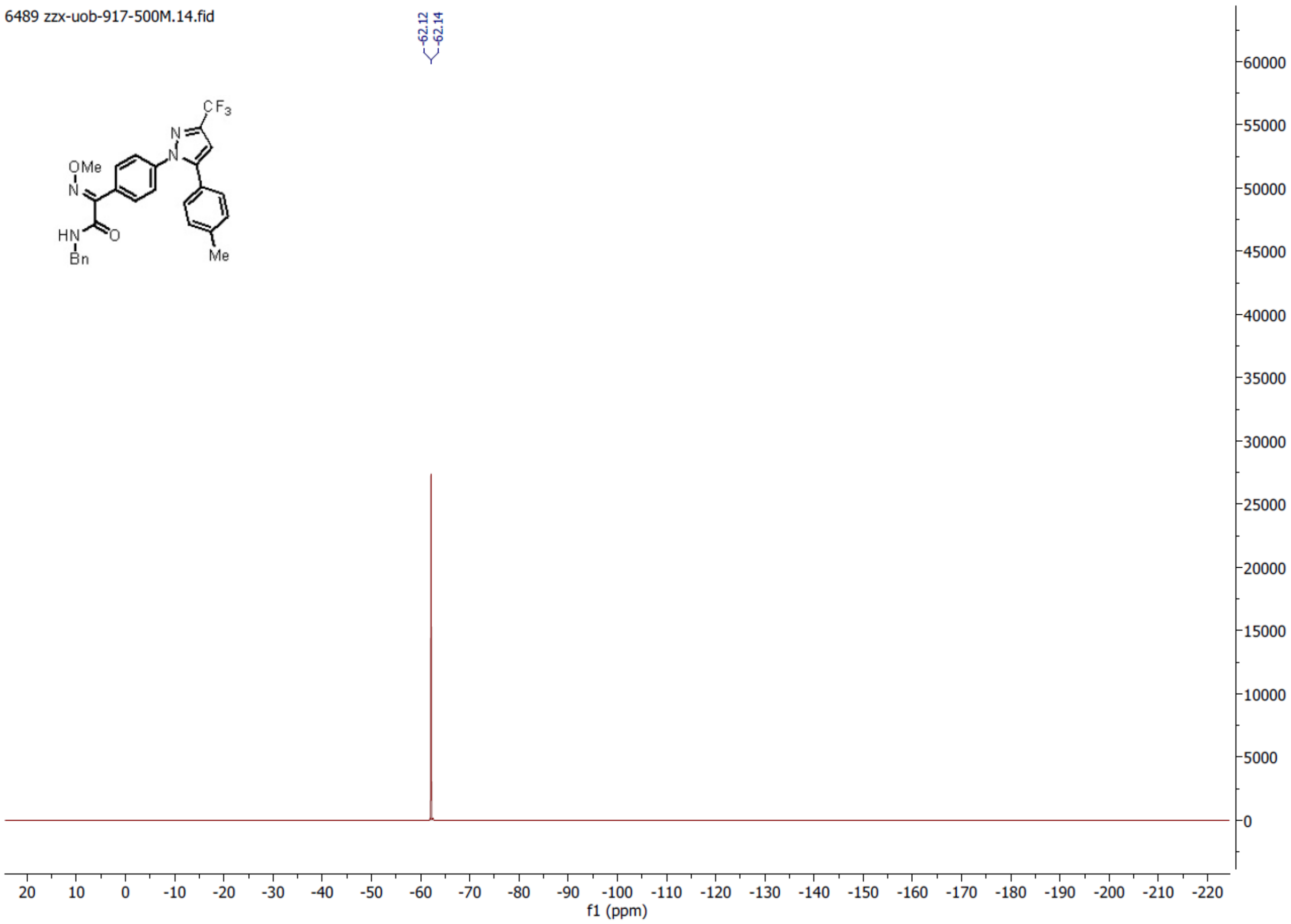
6489 zzx-uob-917-500M.14.fid
-62.12
-62.14
CF3
N
N
OMe
N
HN
O
Bn
Me
60000
55000
50000
45000
40000
35000
30000
25000
20000
15000
10000
5000
0
20 10 0 -10 -20 -30 -40 -50 -60 -70 -80 -90 -100 -110 -120 -130 -140 -150 -160 -170 -180 -190 -200 -210 -220
f1 (ppm)

Compound S42b

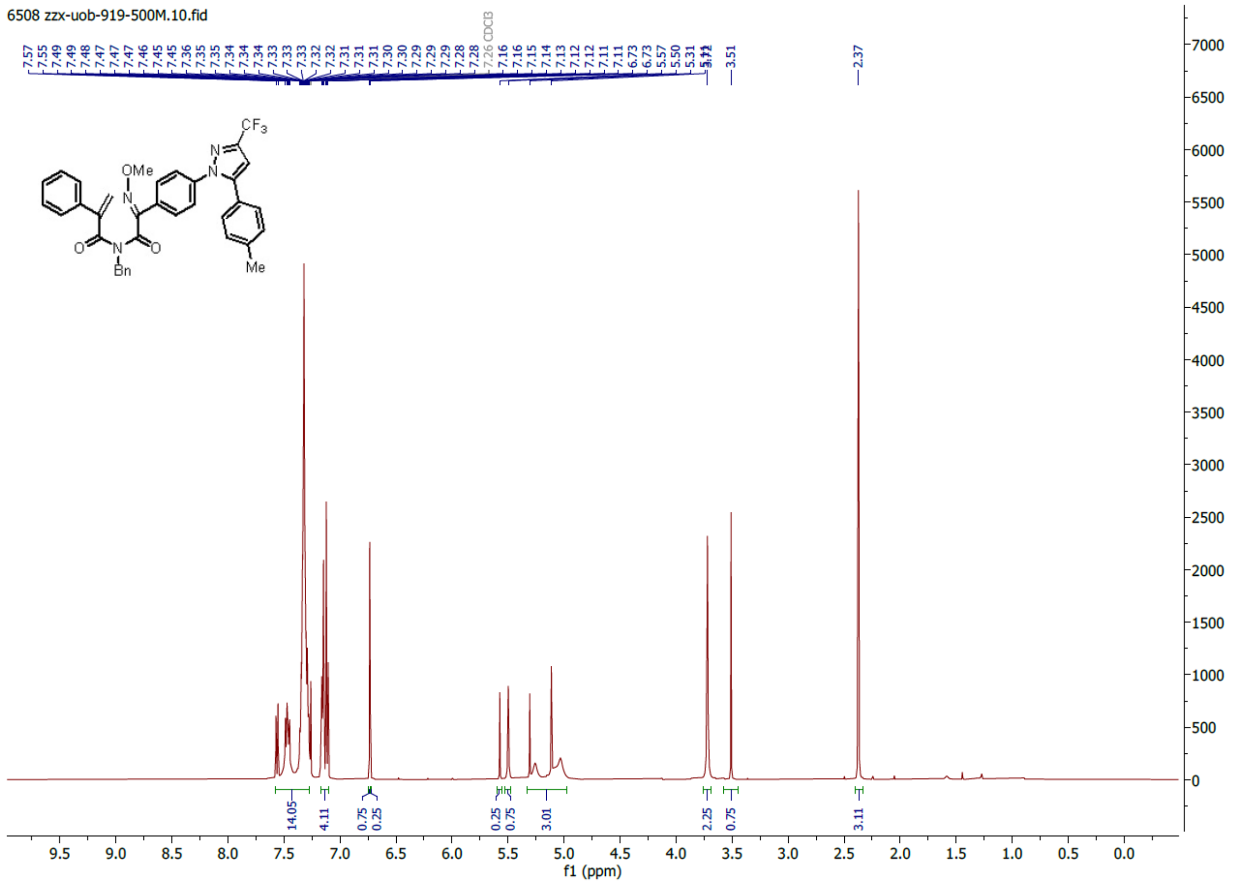

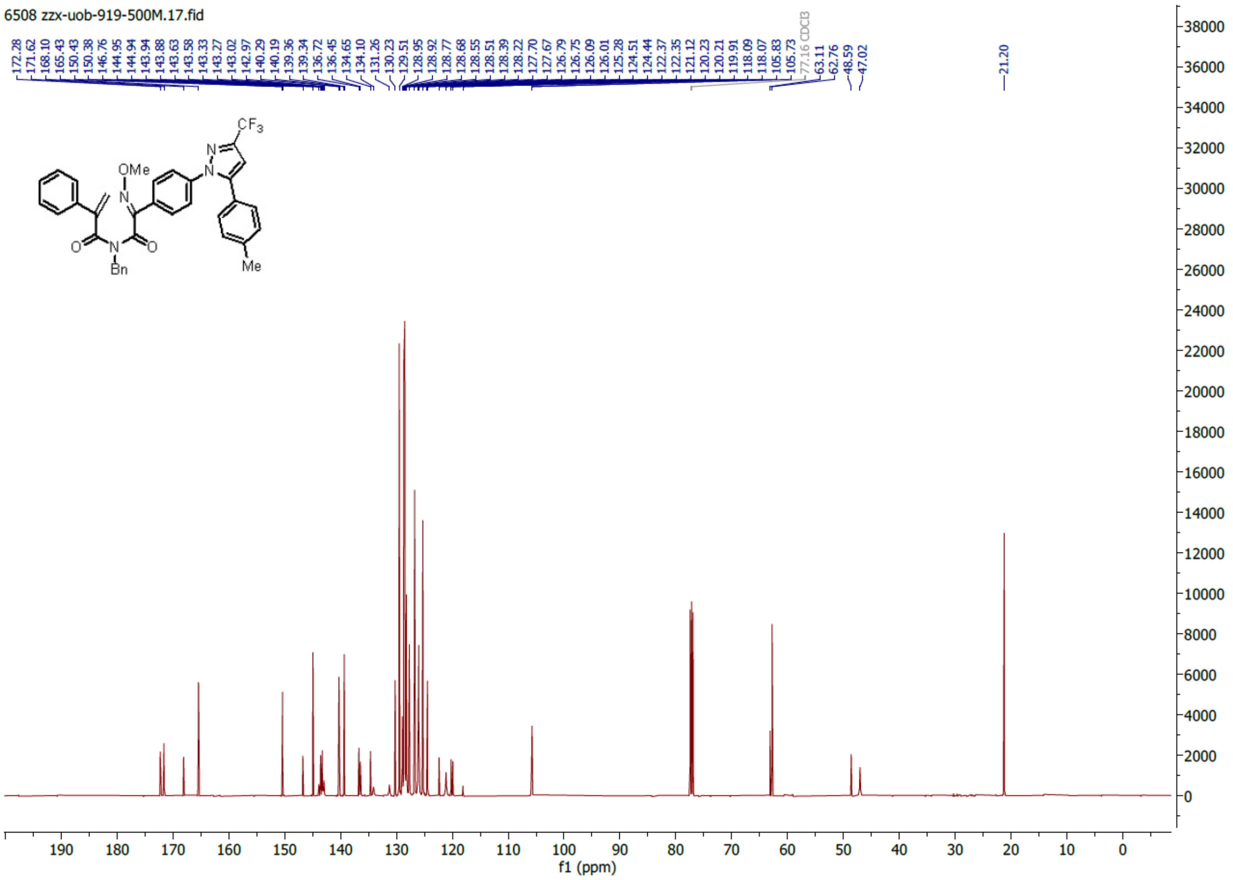

6508 zzx-uob-919-500M.15.fid

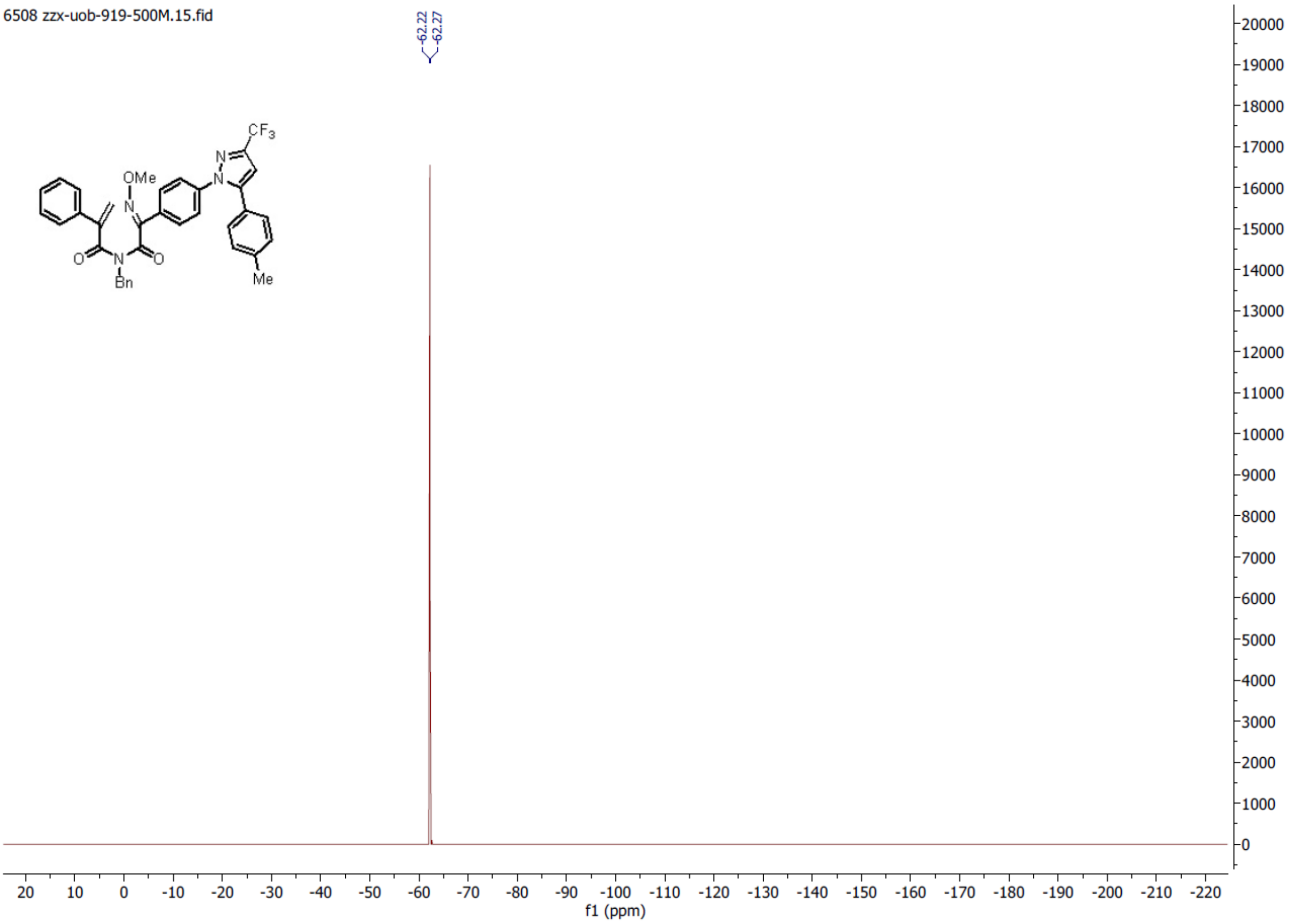

Compound S43a’

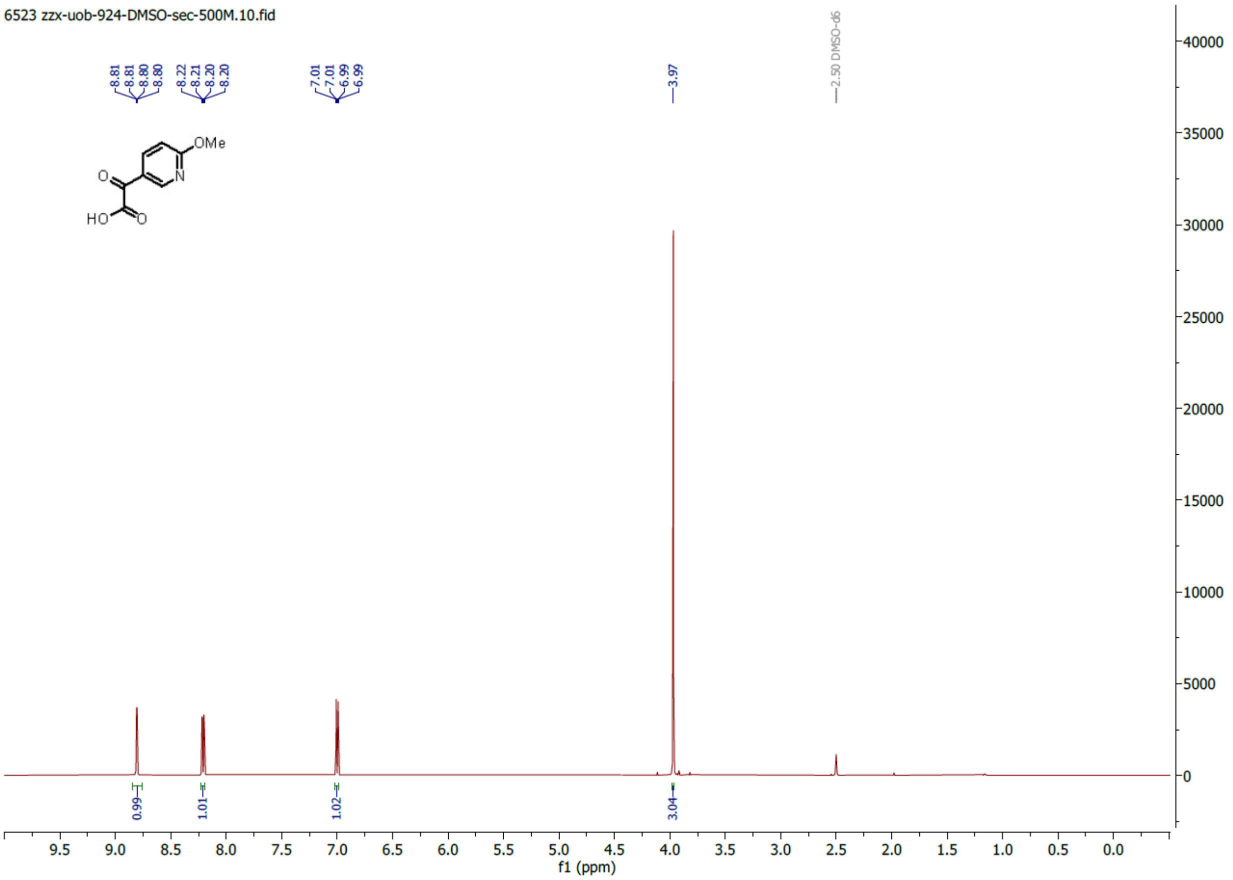

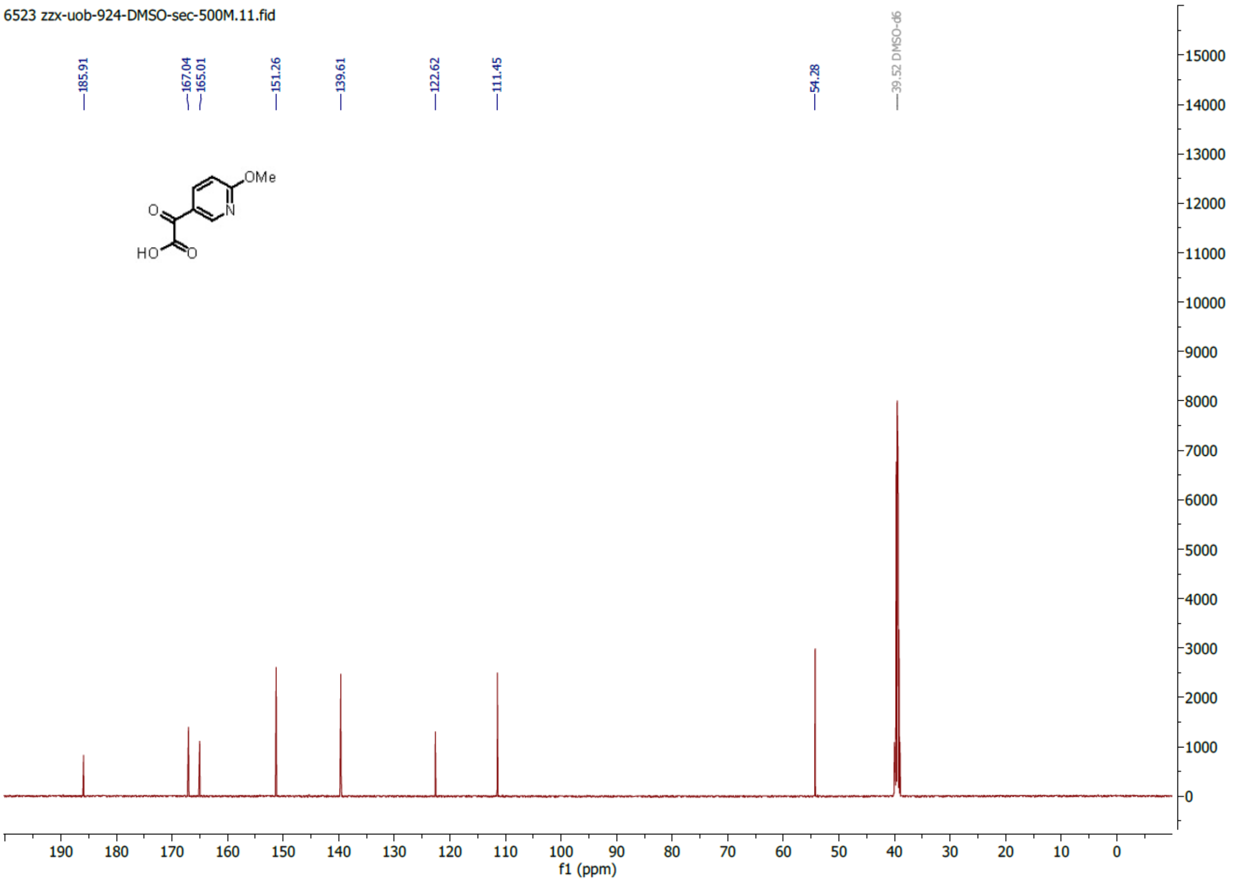

Compound S43a

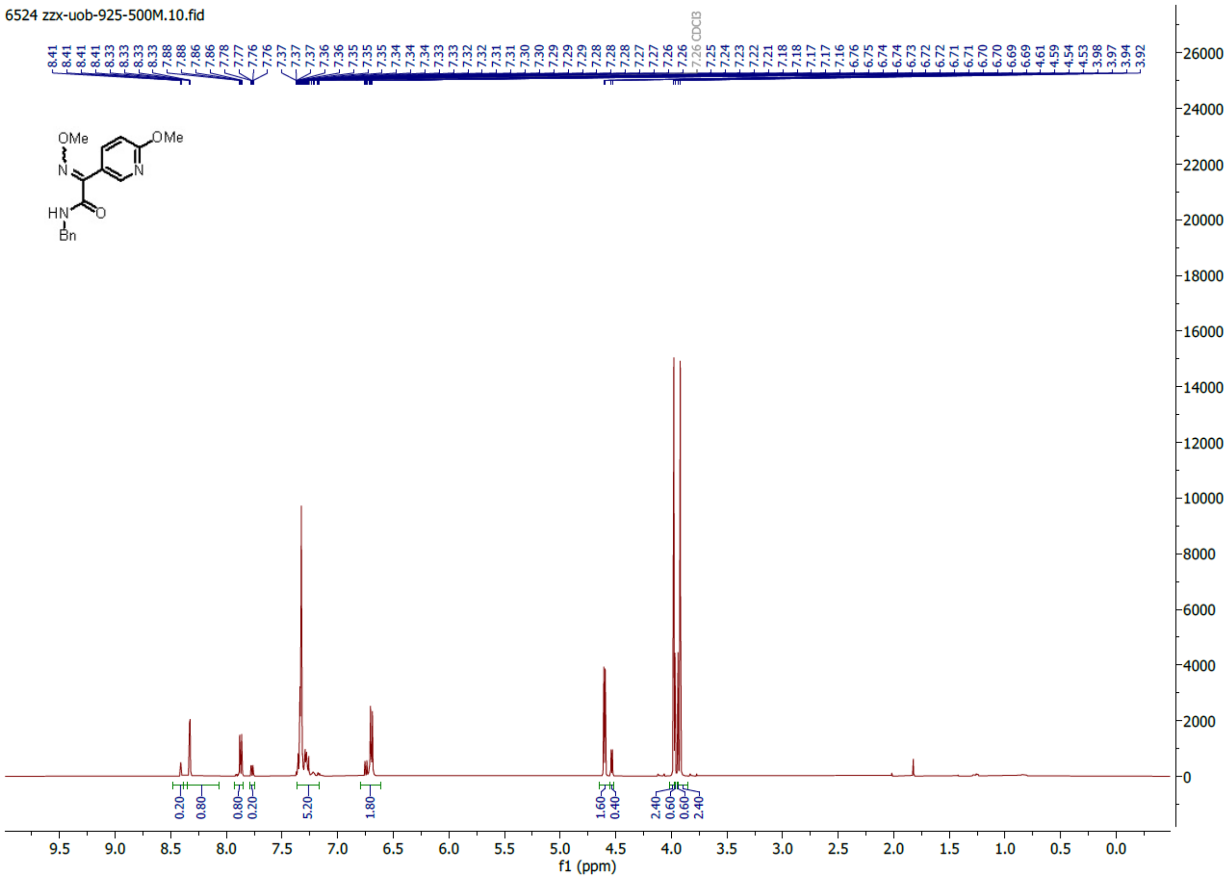

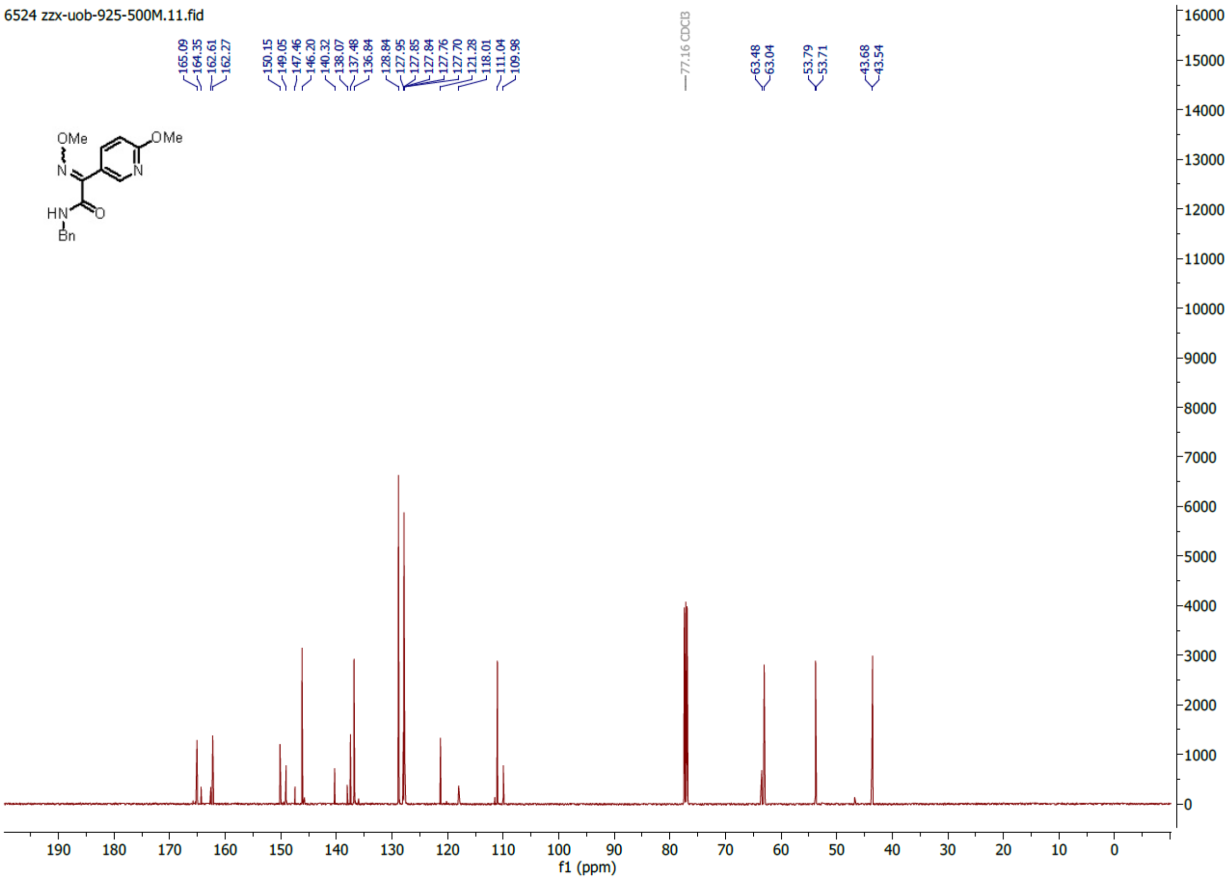

Compound S43b

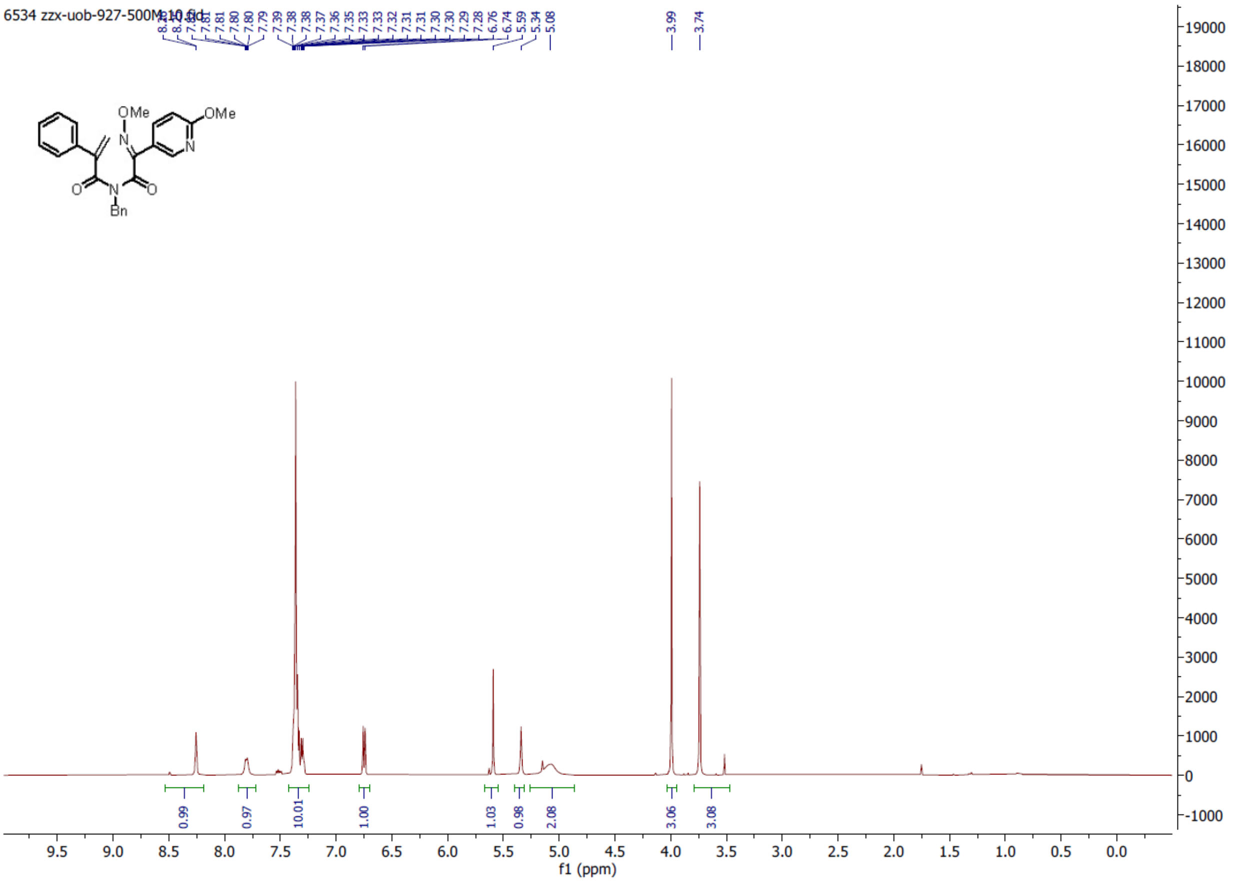

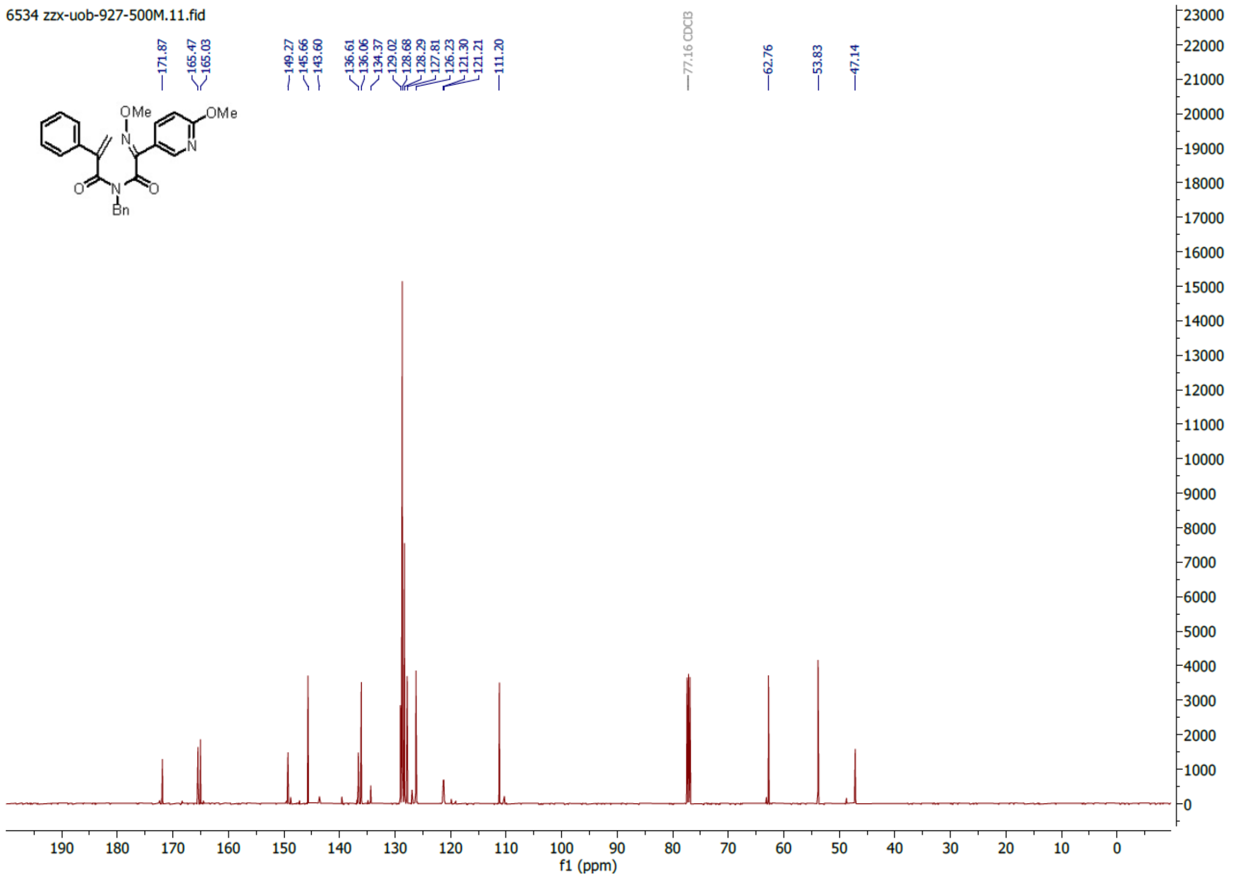

Compound S44a

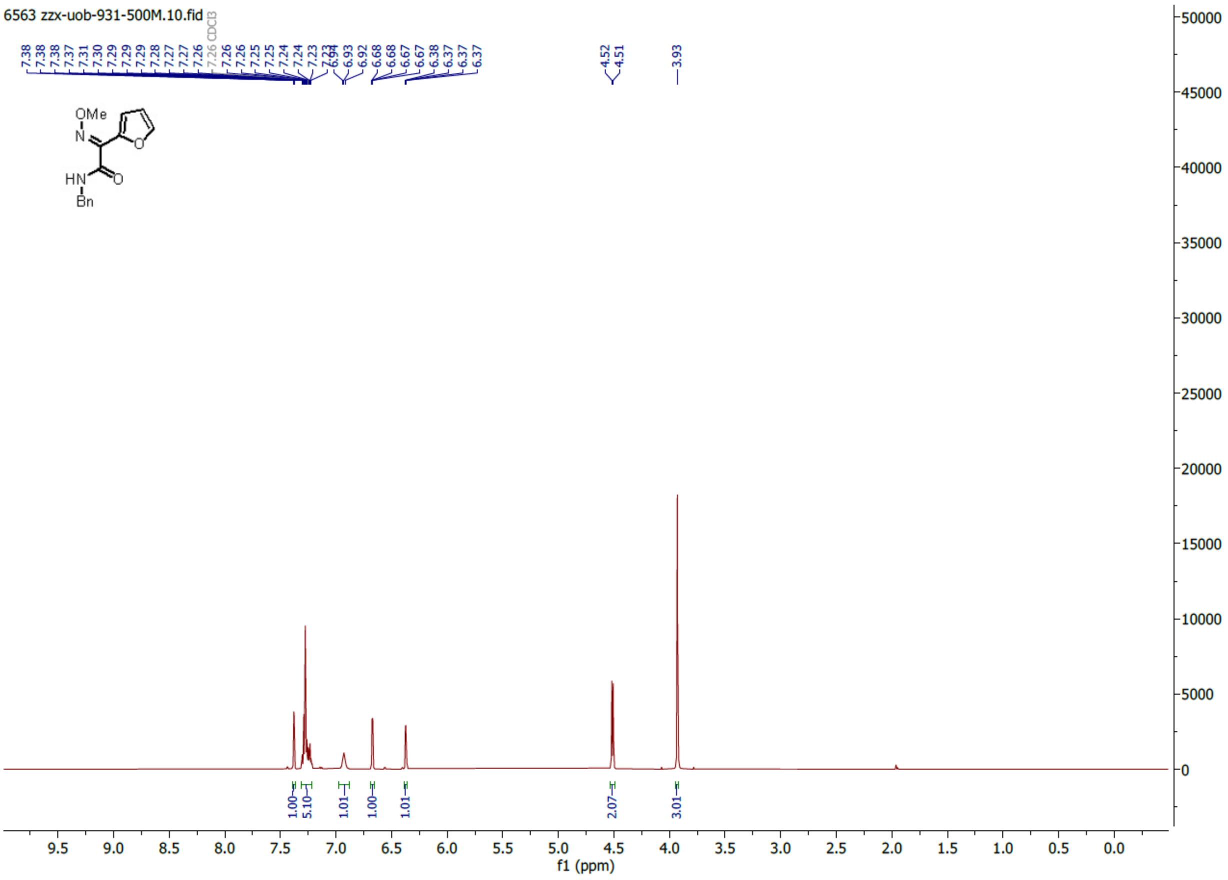

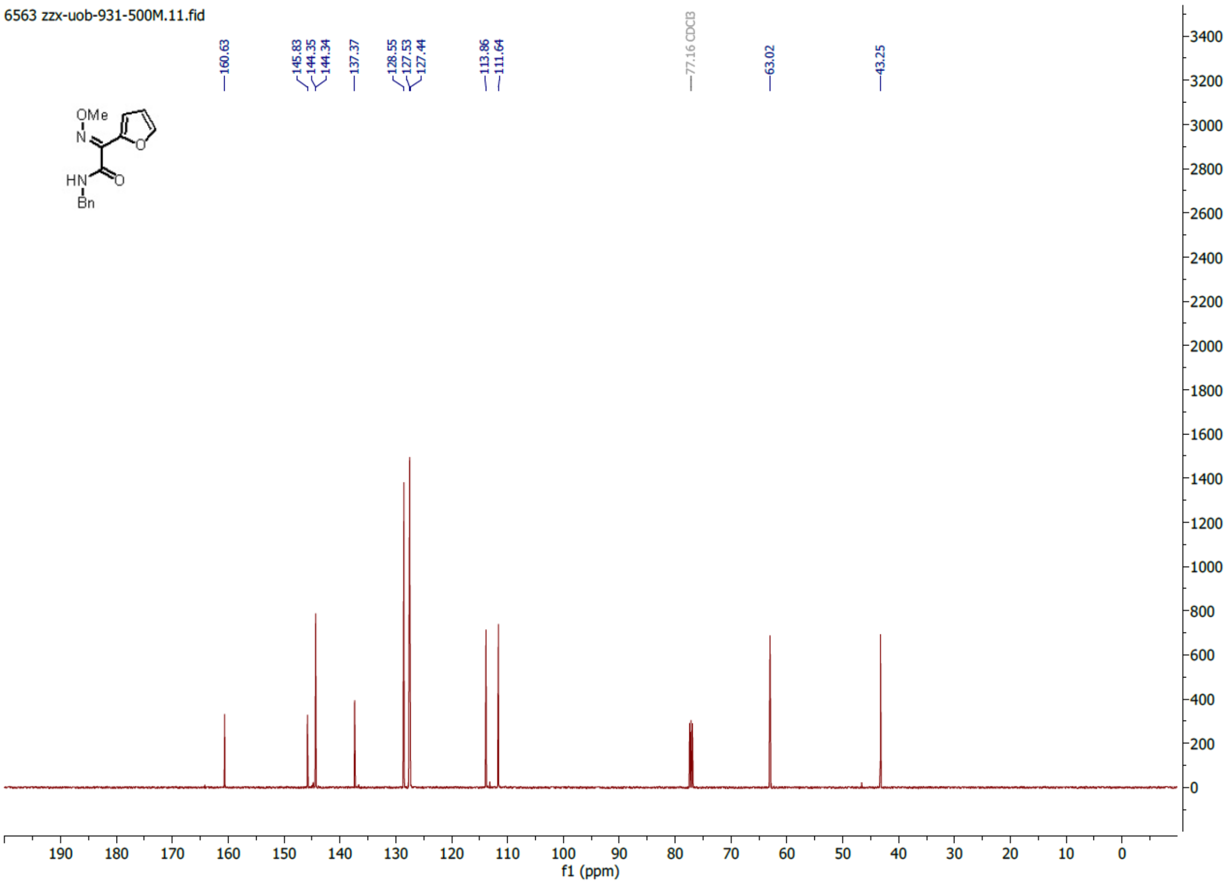

Compound S44b

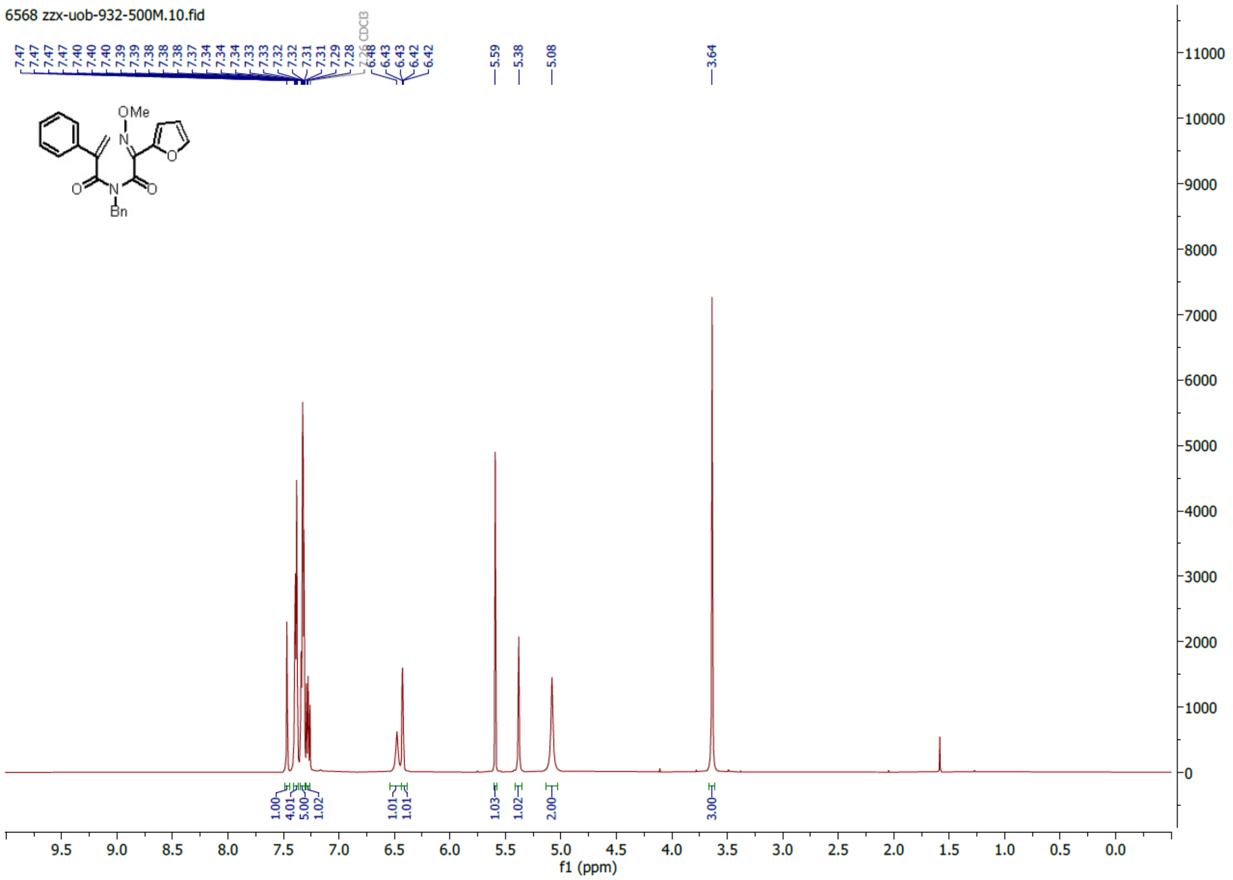

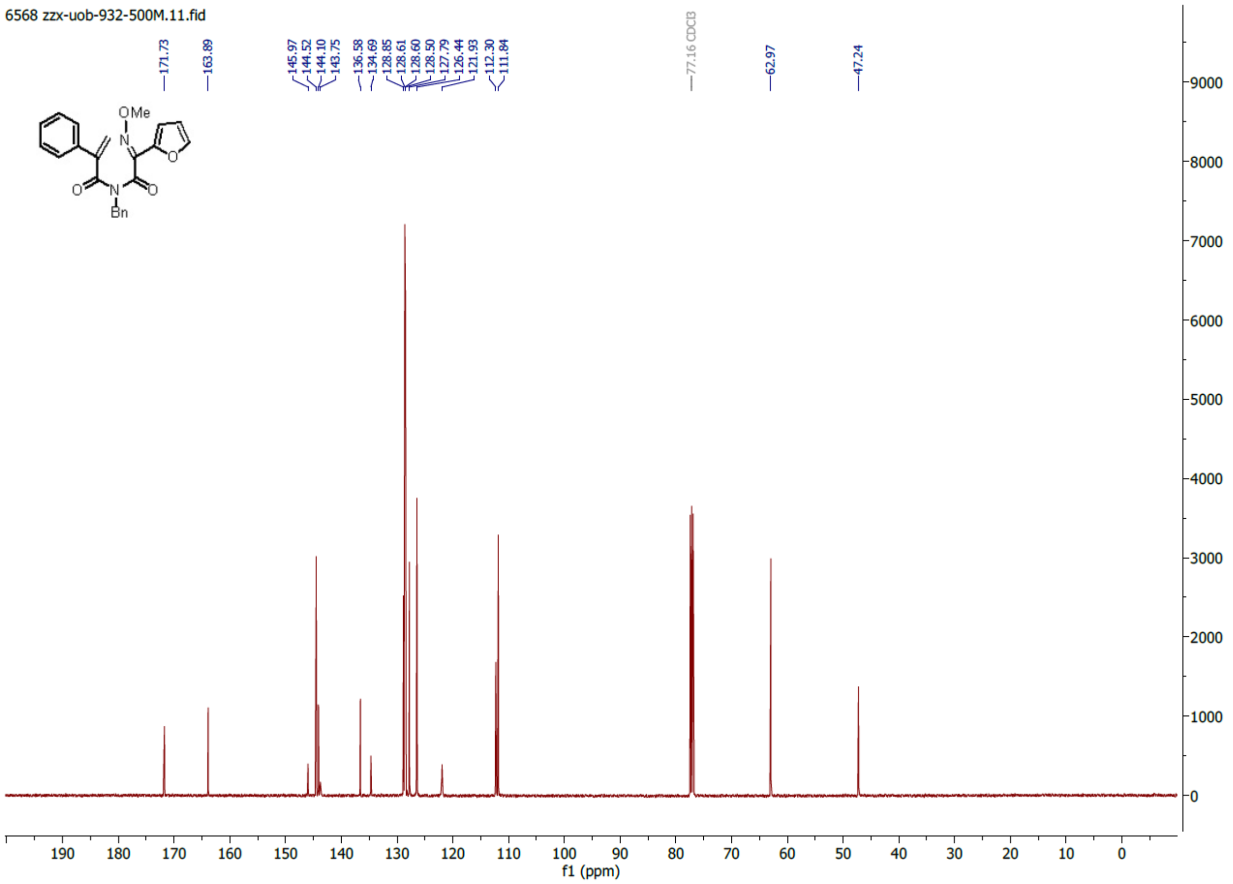

Compound 4

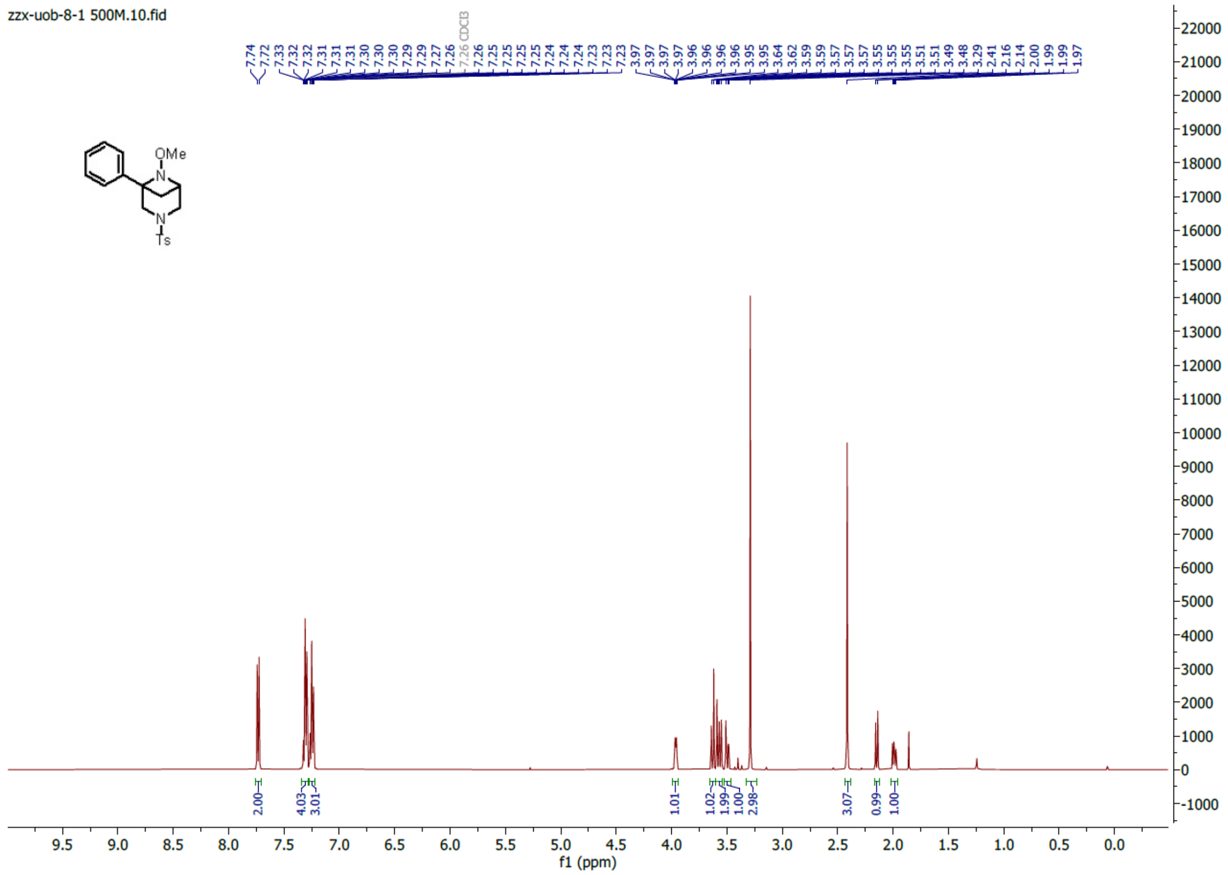

zzx-uob-8-1 500M.10.fid
7.74 7.72 7.33 7.32 7.32 7.31 7.31 7.31 7.30 7.30 7.30 7.29 7.29 7.27 7.26 CDCl3 7.26 7.25 7.25 7.25 7.25 7.24 7.24 7.24 7.23 7.23 7.23 7.23 3.97 3.97 3.97 3.97 3.96 3.96 3.96 3.96 3.95 3.95 3.64 3.62 3.59 3.59 3.57 3.57 3.57 3.55 3.55 3.55 3.51 3.51 3.49 3.48 3.29 2.41 2.16 2.14 2.00 1.99 1.99 1.97
OMe
N
N
Ts
2.00
4.03
3.01
1.01
1.02
1.99
1.00
2.98
3.07
0.99
1.00
f1 (ppm)

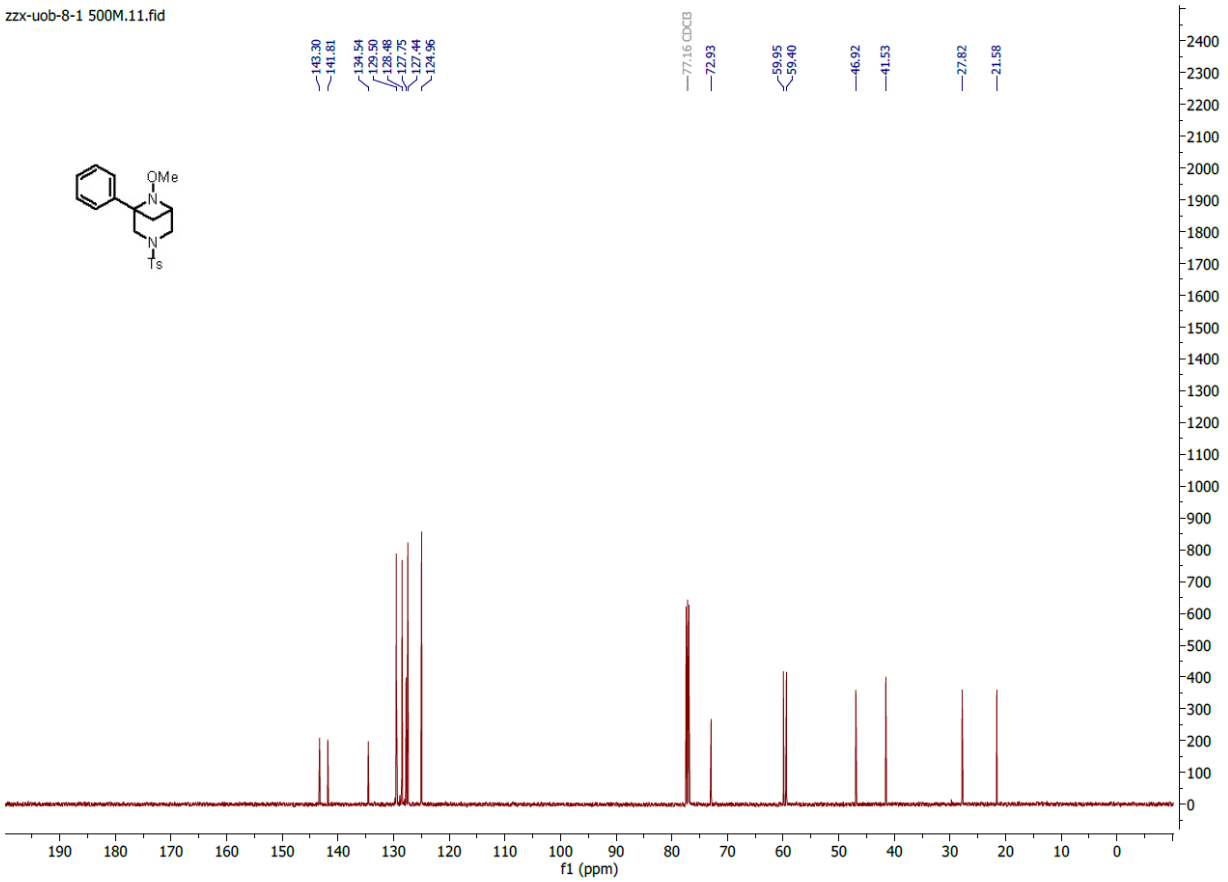

zzx-uob-8-1 500M.11.fid
143.30 141.81 134.54 129.50 128.48 127.75 127.44 124.96
77.16 CDCl3
72.93
59.95 59.40
46.92
41.53
27.82
21.58
OMe
N
N
Ts
f1 (ppm)

Compound 4a

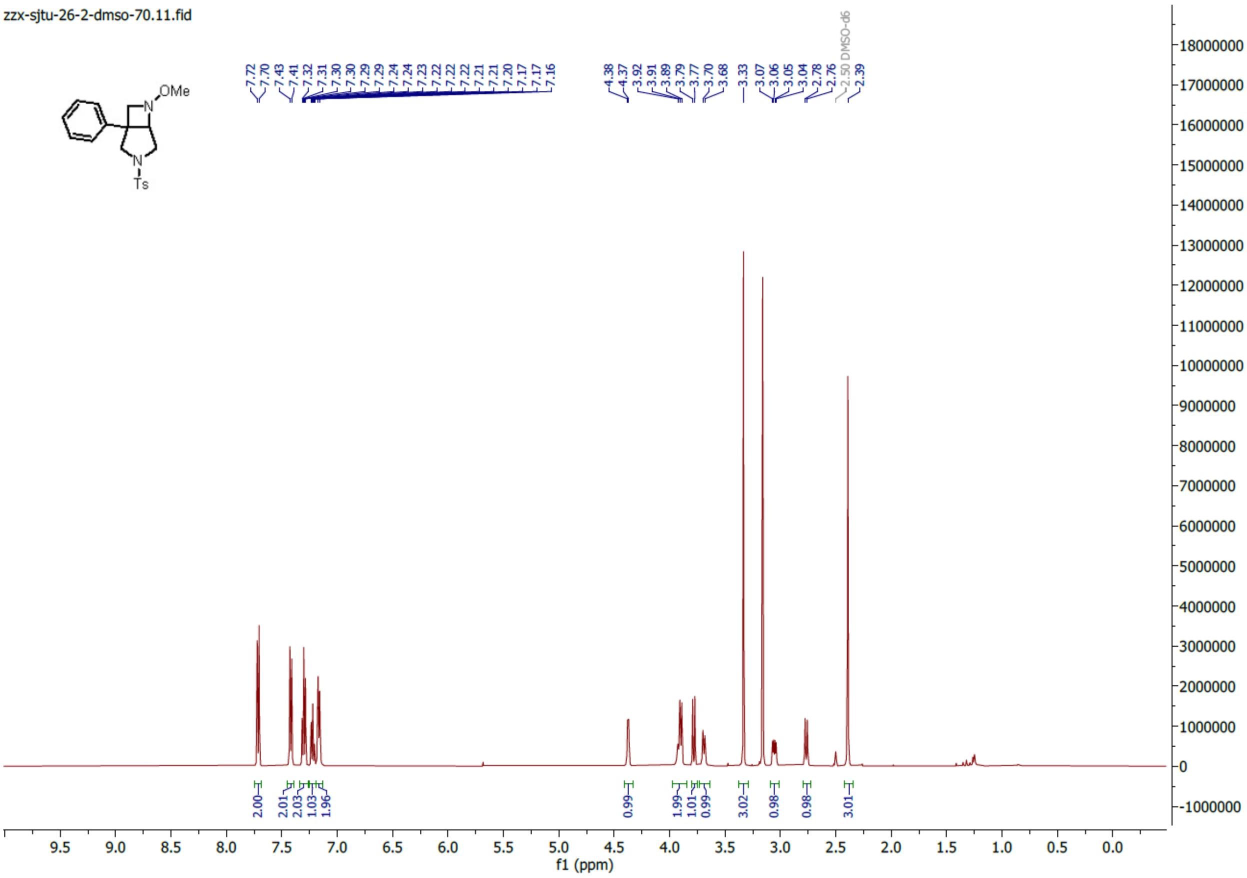
zzx-sjtu-26-2-dmso-70.11.fid
OMe
N
N
Ts
f1 (ppm)
2.50 DMSO-d6

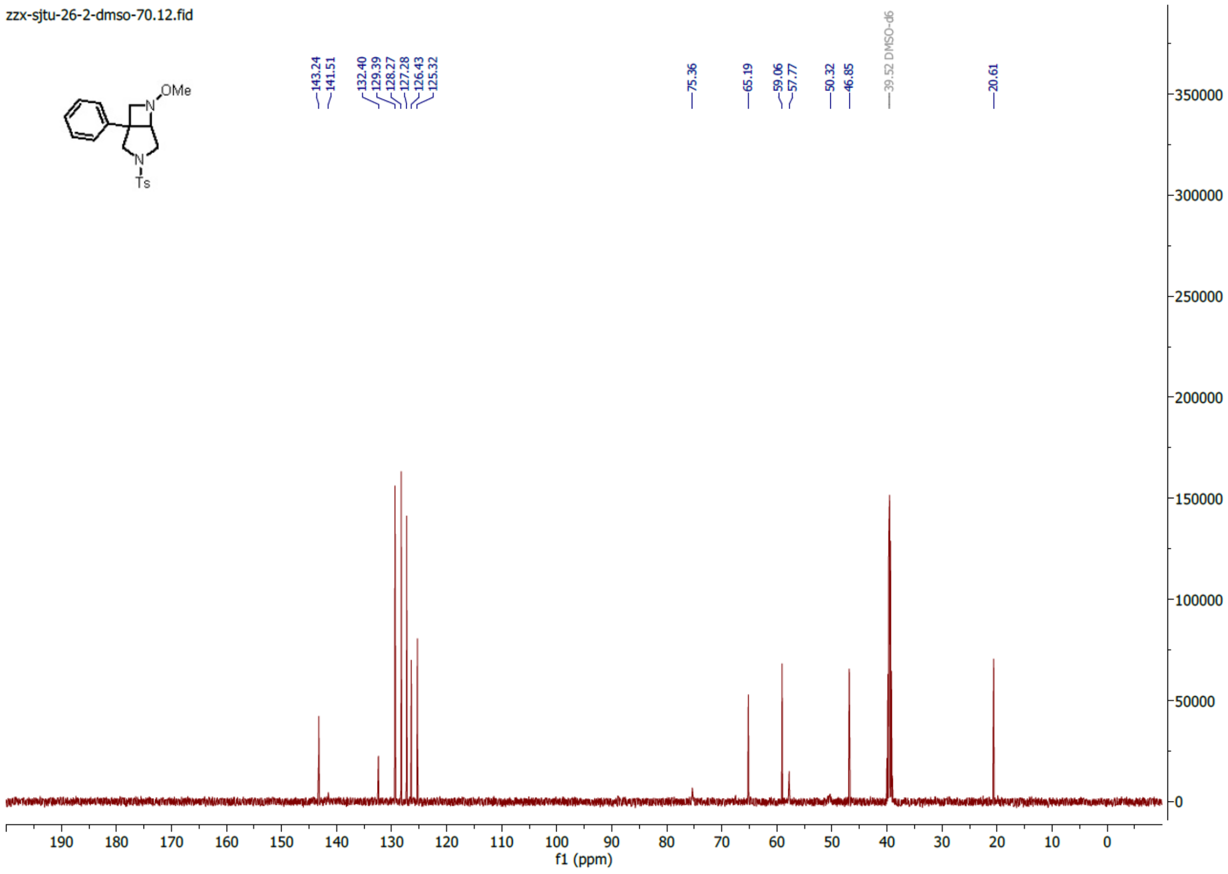
zzx-sjtu-26-2-dmso-70.12.fid
OMe
N
N
Ts
f1 (ppm)
39.52 DMSO-d6

Compound 6

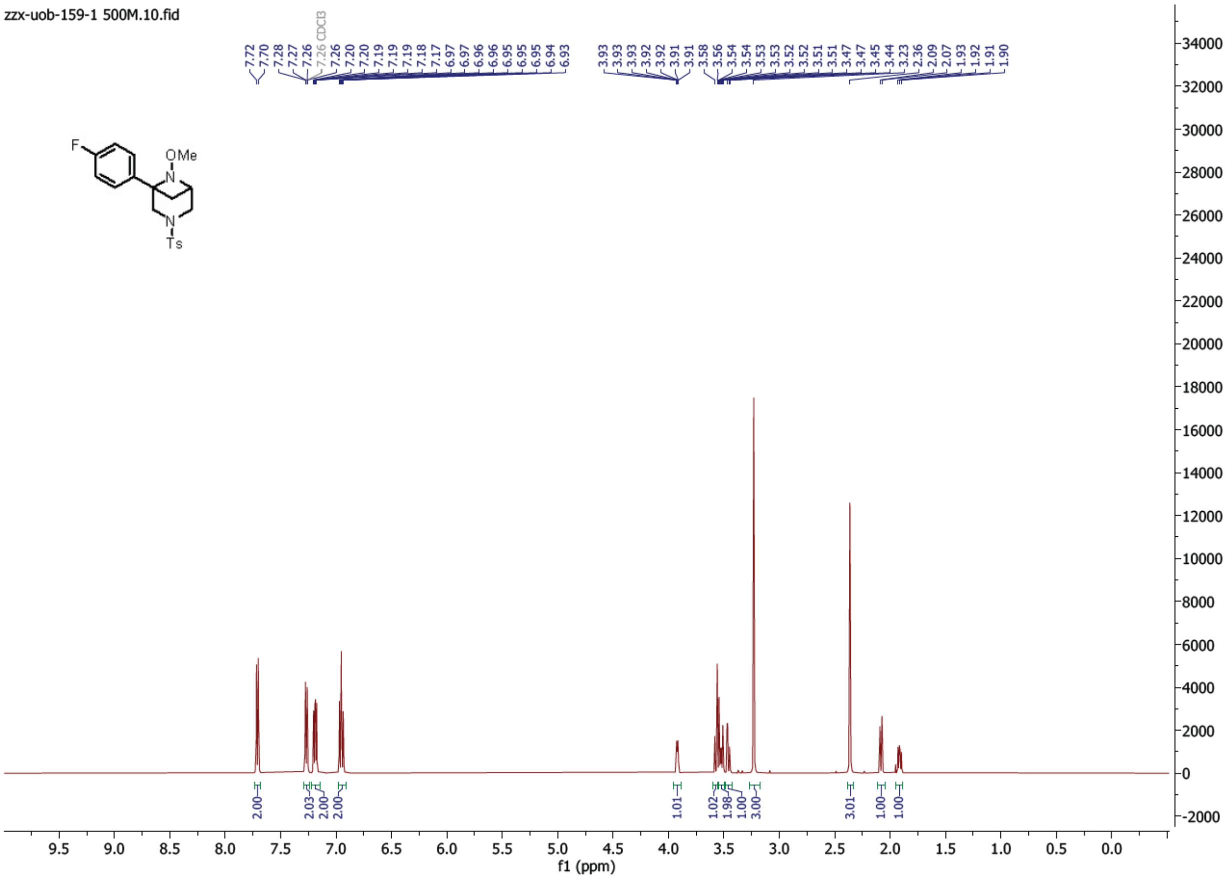
zzx-uob-159-1 500M.10.fid
7.72 7.70 7.28 7.27 7.26 7.26 CDCl3 7.26 7.20 7.20 7.19 7.19 7.19 7.18 7.17 6.97 6.97 6.96 6.96 6.95 6.95 6.95 6.94 6.93
3.93 3.93 3.93 3.92 3.92 3.91 3.91 3.58 3.56 3.54 3.54 3.53 3.53 3.52 3.52 3.51 3.51 3.47 3.47 3.45 3.44 3.23 2.36 2.09 2.07 1.93 1.92 1.91 1.90
F
OMe
N
N
Ts
2.00 2.03 2.00 2.00 1.01 1.02 1.98 1.00 3.00 3.01 1.00 1.00
f1 (ppm)

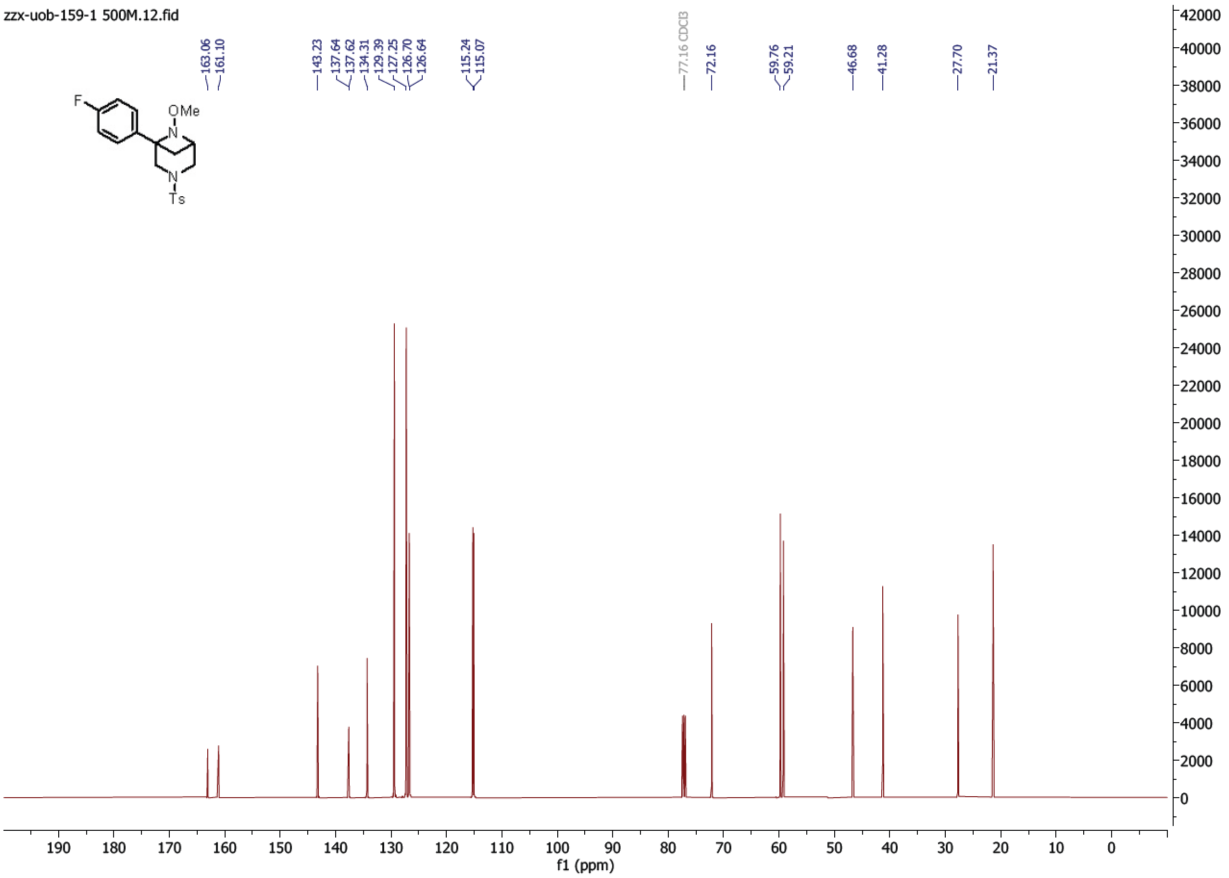
zzx-uob-159-1 500M.12.fid
163.06 161.10 143.23 137.64 137.62 134.31 129.39 127.25 126.70 126.64 115.24 115.07 77.16 CDCl3 72.16 59.76 59.21 46.68 41.28 27.70 21.37
F
OMe
N
N
Ts
f1 (ppm)

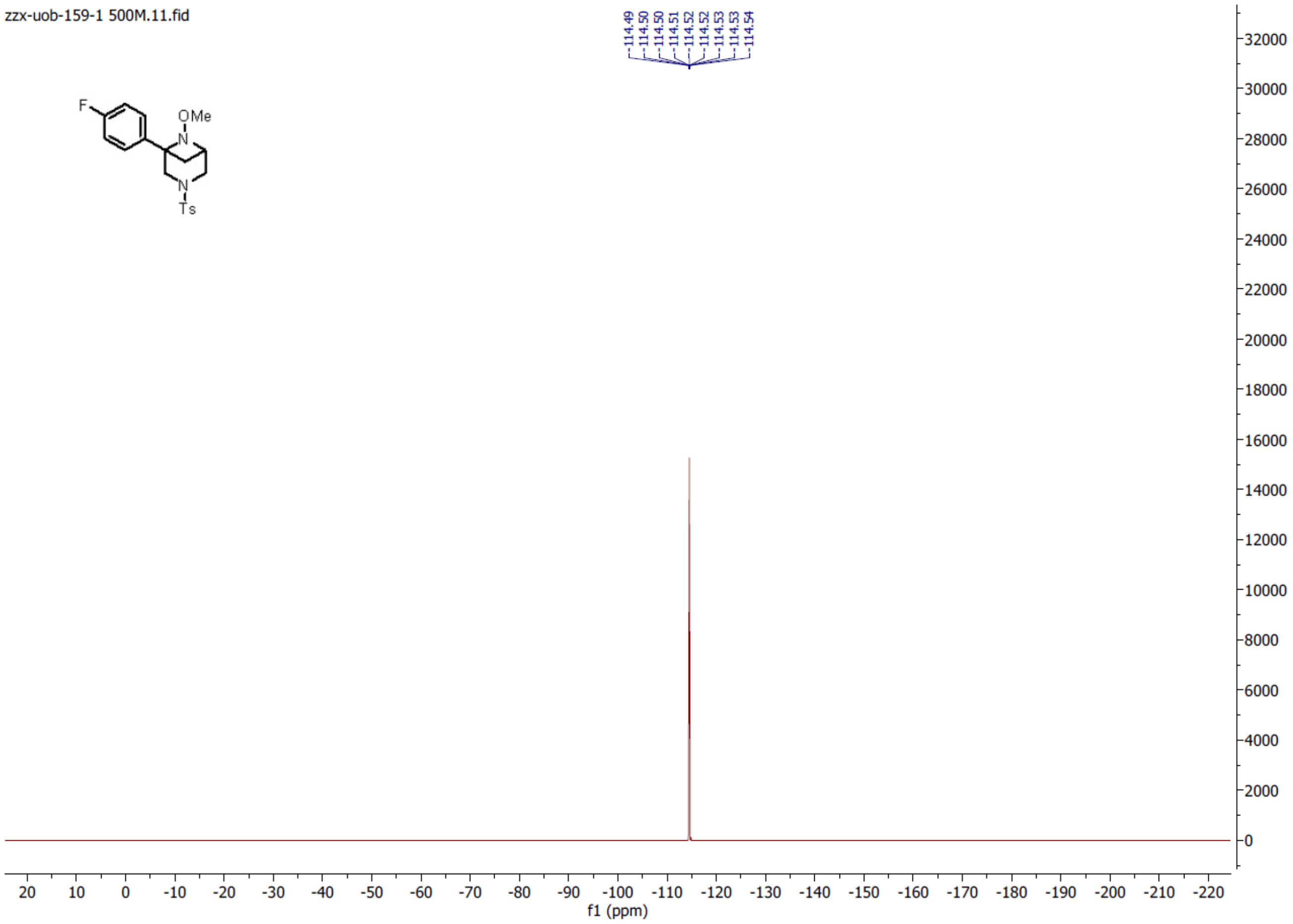
zzx-uob-159-1 500M.11.fid
-114.49
-114.50
-114.50
-114.51
-114.52
-114.52
-114.53
-114.53
-114.54
F
OMe
N
N
Ts
32000
30000
28000
26000
24000
22000
20000
18000
16000
14000
12000
10000
8000
6000
4000
2000
0
20 10 0 -10 -20 -30 -40 -50 -60 -70 -80 -90 -100 -110 -120 -130 -140 -150 -160 -170 -180 -190 -200 -210 -220
f1 (ppm)

Compound 6a

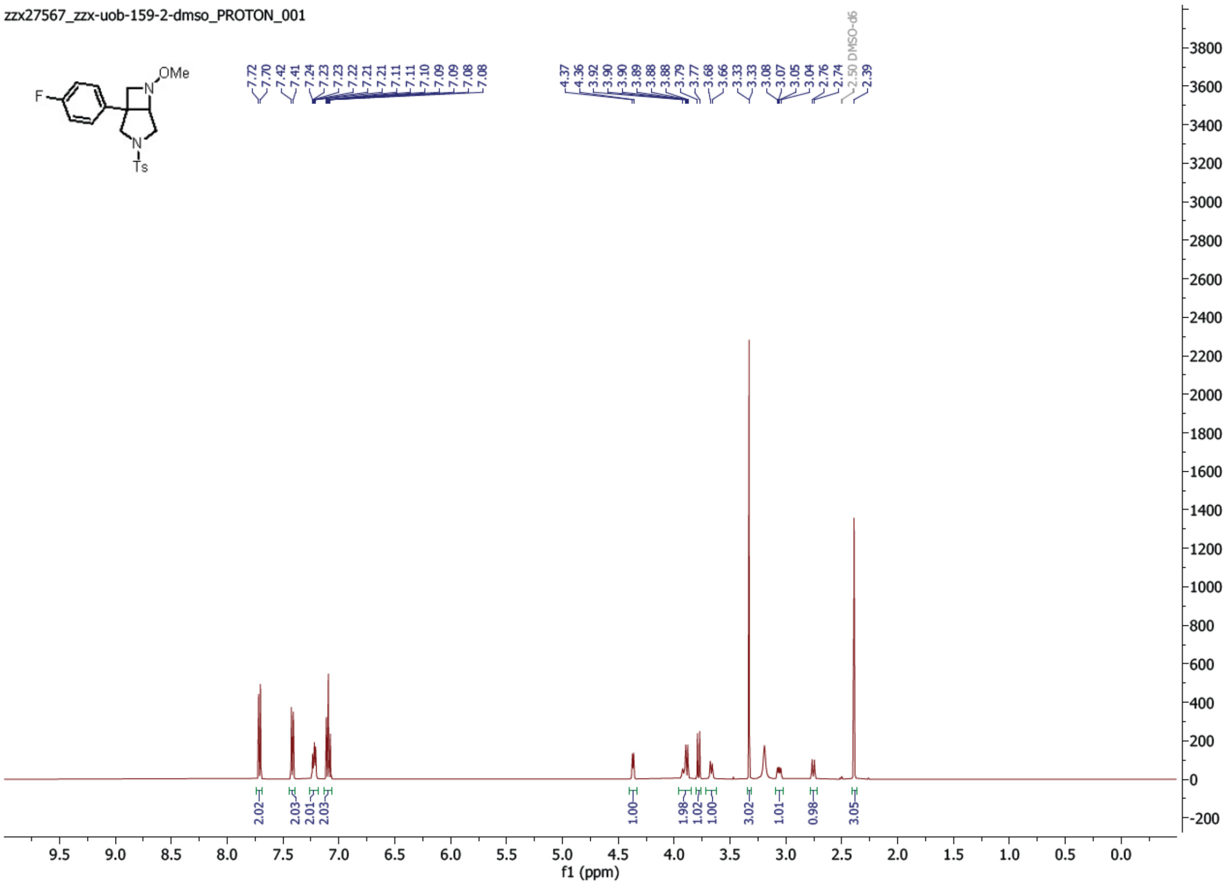
zzx27567_zzx-uob-159-2-dmso_PROTON_001
OMe
F
N
Ts
7.72
7.70
7.42
7.41
7.24
7.23
7.23
7.22
7.21
7.21
7.11
7.11
7.10
7.09
7.09
7.08
7.08
4.37
4.36
3.92
3.90
3.90
3.89
3.88
3.88
3.79
3.77
3.68
3.66
3.33
3.33
3.08
3.07
3.05
3.04
2.76
2.74
2.50 DMSO-d6
2.39
2.02
2.03
2.01
2.03
1.00
1.98
1.02
1.00
3.02
1.01
0.98
3.05
f1 (ppm)

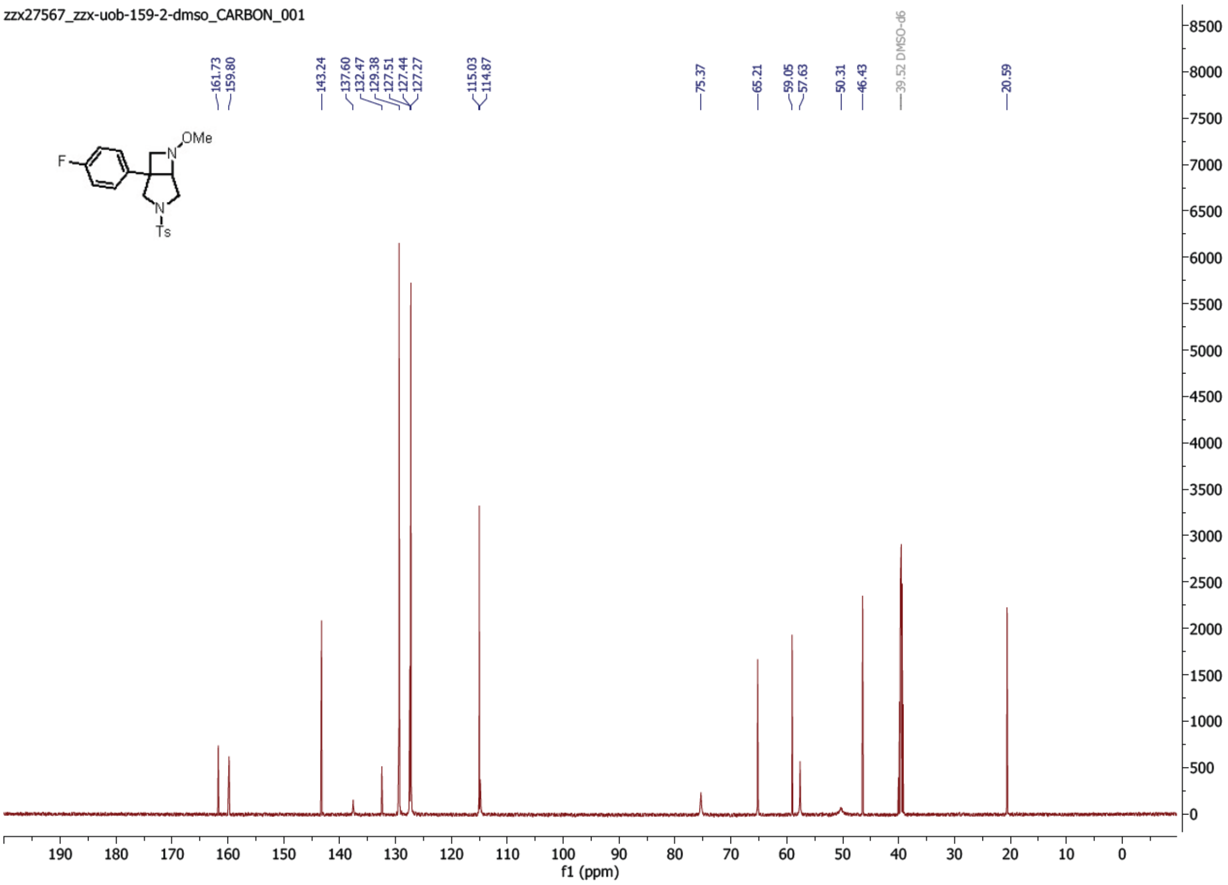
zzx27567_zzx-uob-159-2-dmso_CARBON_001
161.73
159.80
143.24
137.60
132.47
129.38
127.51
127.44
127.27
115.03
114.87
75.37
65.21
59.05
57.63
50.31
46.43
39.52 DMSO-d6
20.59
OMe
F
N
Ts
f1 (ppm)

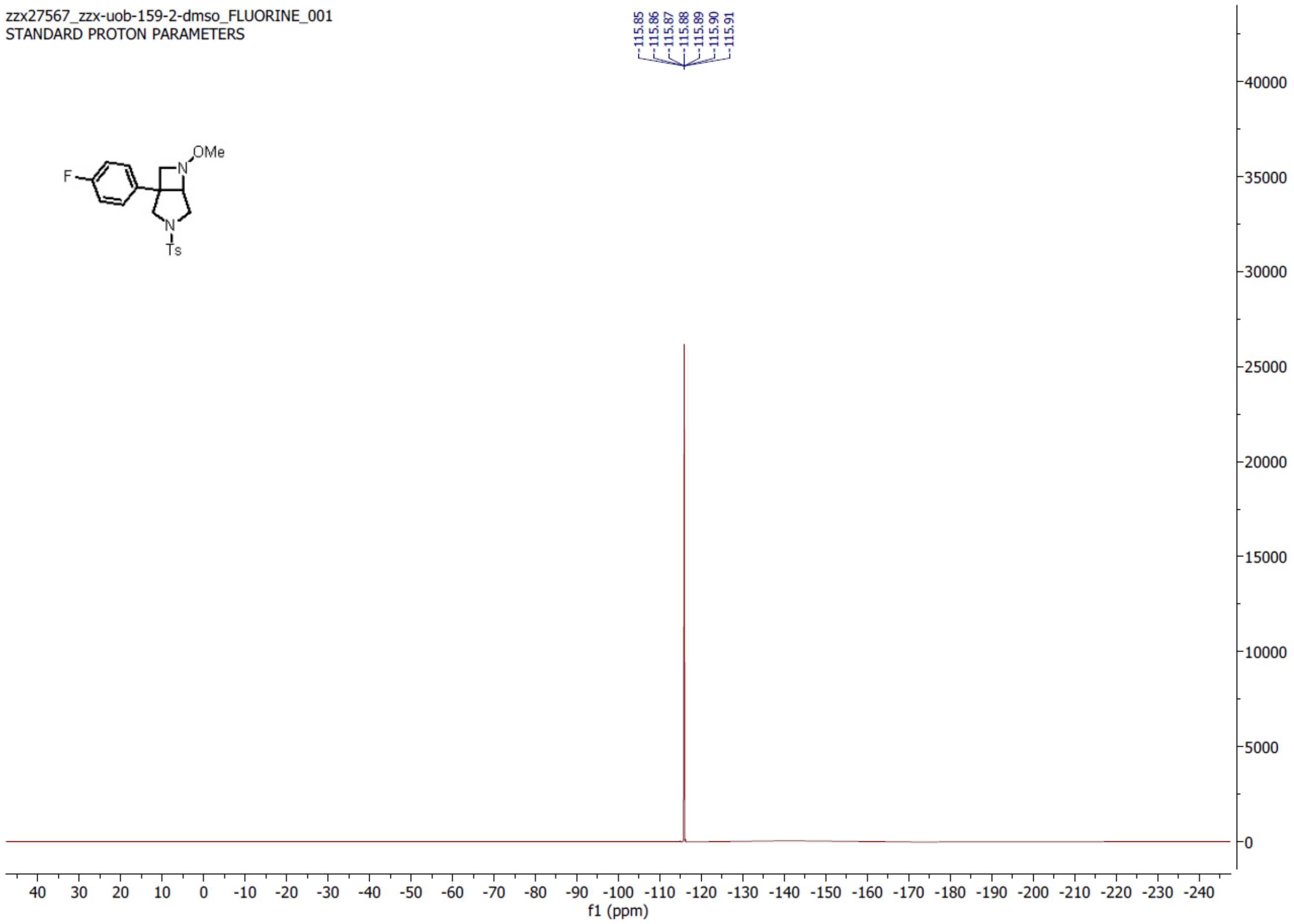
zzx27567_zzx-uob-159-2-dmso_FLUORINE_001
STANDARD PROTON PARAMETERS
-115.85
-115.86
-115.87
-115.88
-115.89
-115.90
-115.91
F
N
OMe
N
Ts
f1 (ppm)

Compound 7

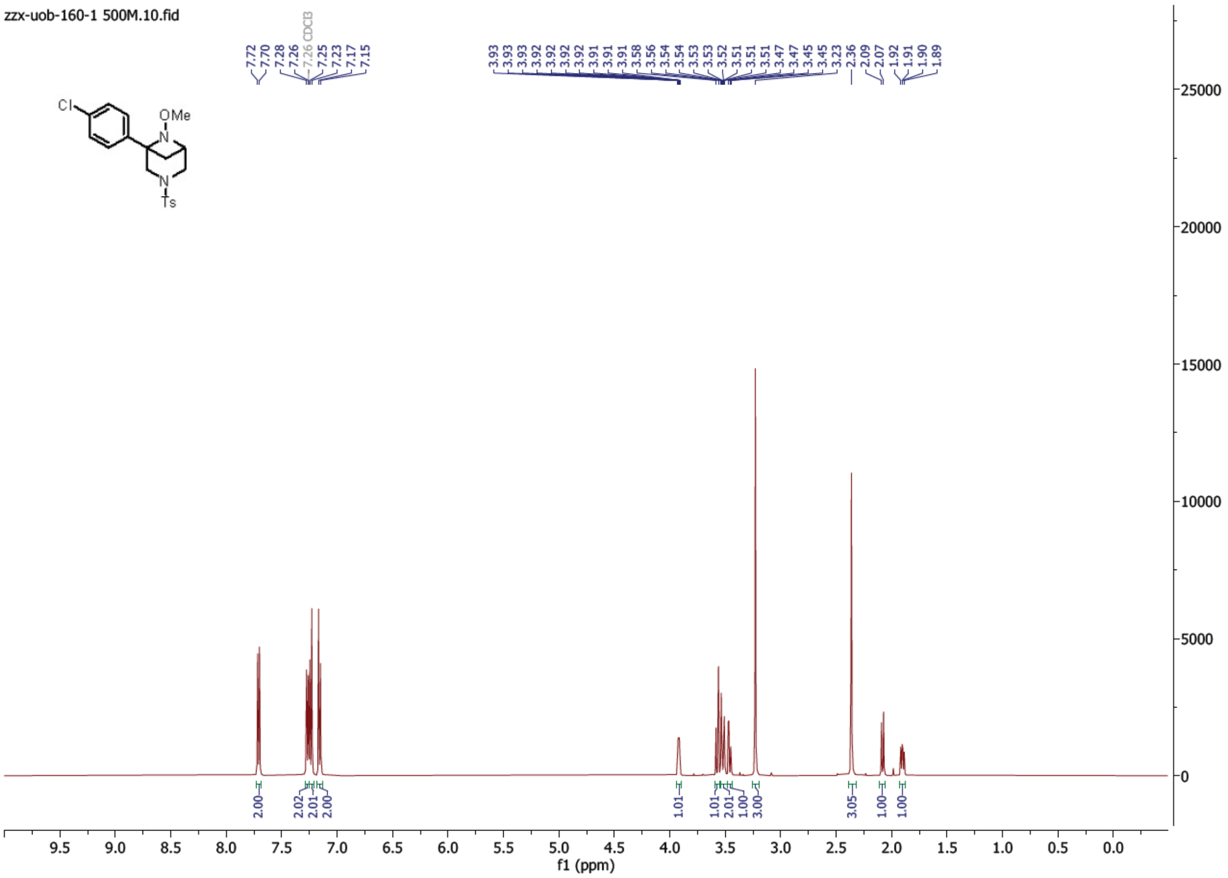

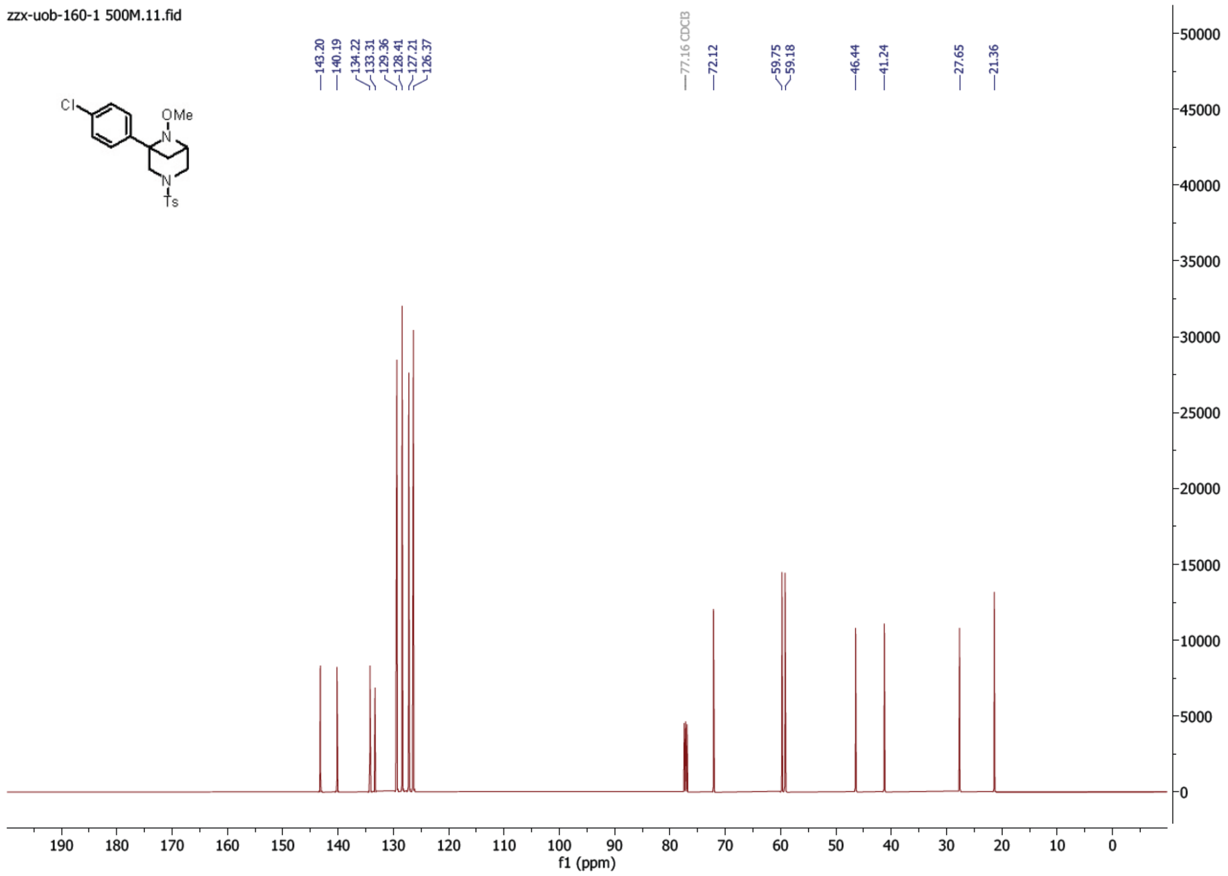

Compound 7a

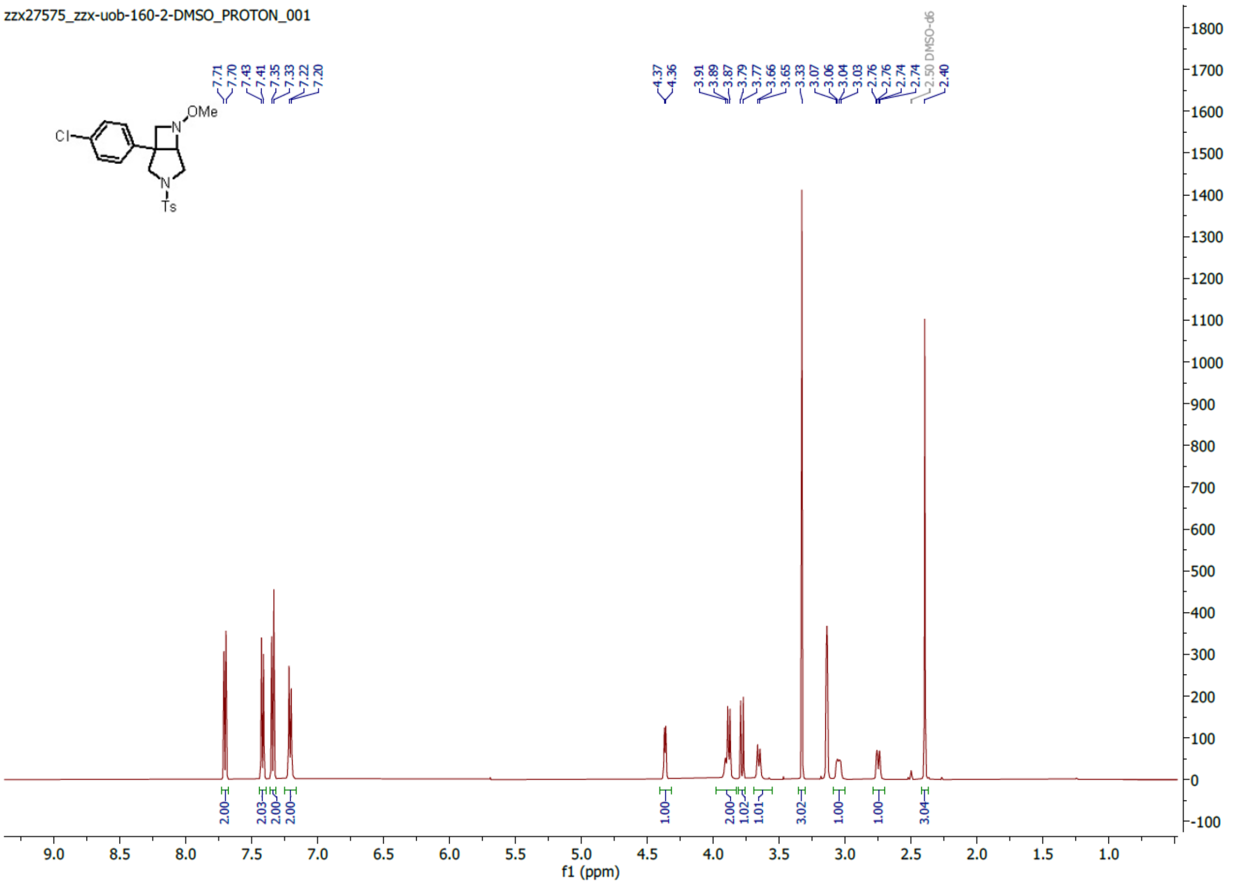
zzx27575_zzx-uob-160-2-DMSO_PROTON_001
Cl
N
OMe
N
Ts
7.71
7.70
7.43
7.41
7.35
7.33
7.22
7.20
4.37
4.36
3.91
3.89
3.87
3.79
3.77
3.66
3.65
3.33
3.07
3.06
3.04
3.03
2.76
2.76
2.74
2.74
2.50 DMSO-d6
2.40
2.00
2.03
2.00
2.00
1.00
2.00
1.02
1.01
3.02
1.00
1.00
3.04
f1 (ppm)

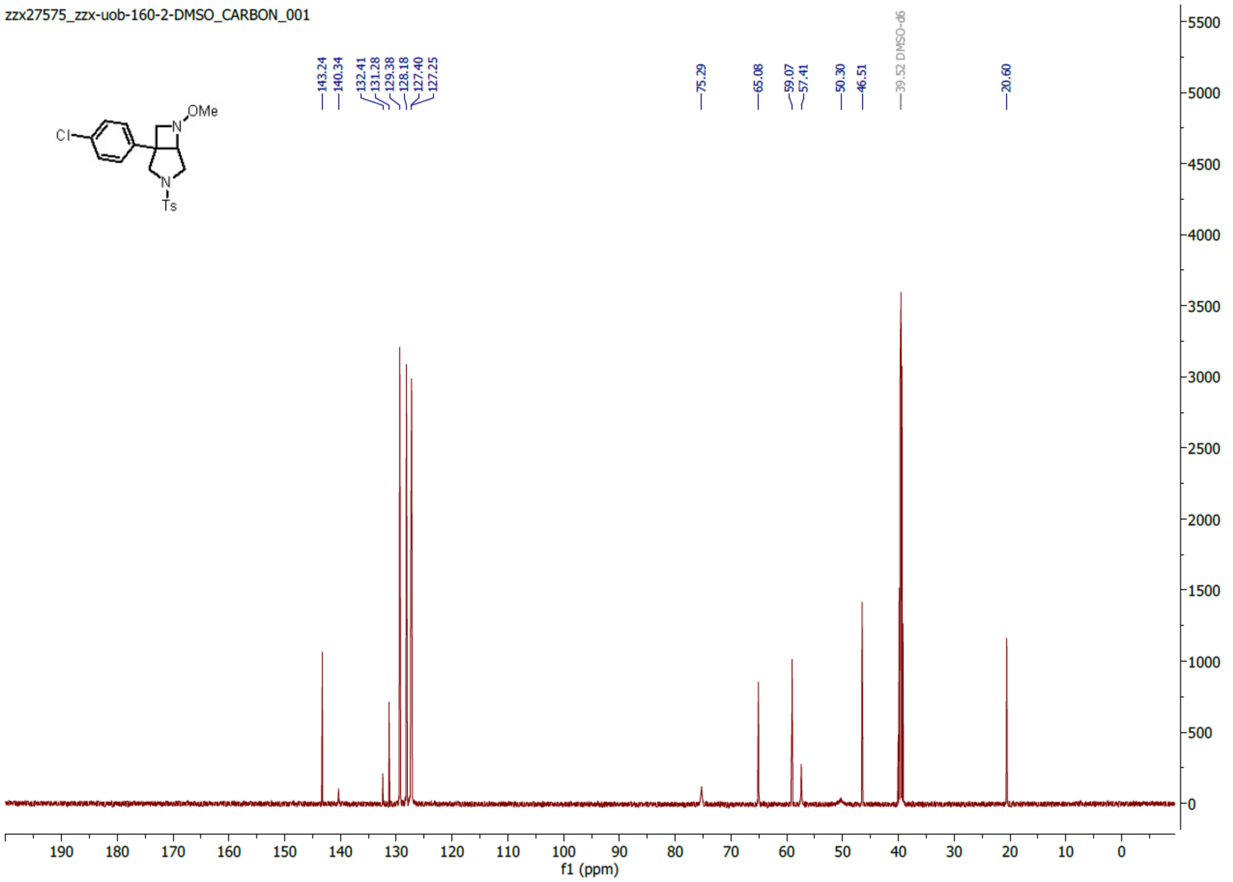
zzx27575_zzx-uob-160-2-DMSO_CARBON_001
Cl
N
OMe
N
Ts
143.24
140.34
132.41
131.28
129.38
128.18
127.40
127.25
75.29
65.08
59.07
57.41
50.30
46.51
39.52 DMSO-d6
20.60
f1 (ppm)

Compound 8

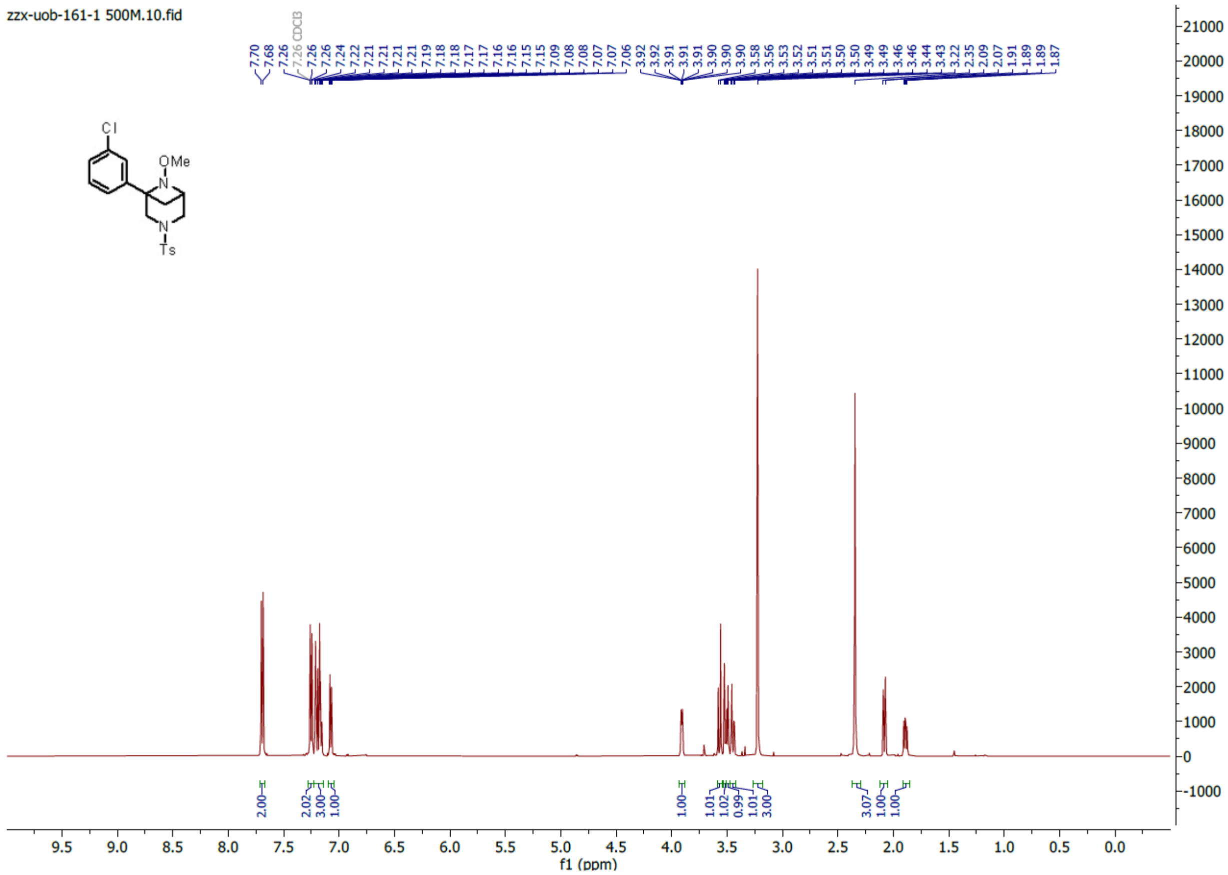
zzx-uob-161-1 500M.10.fid
f1 (ppm)

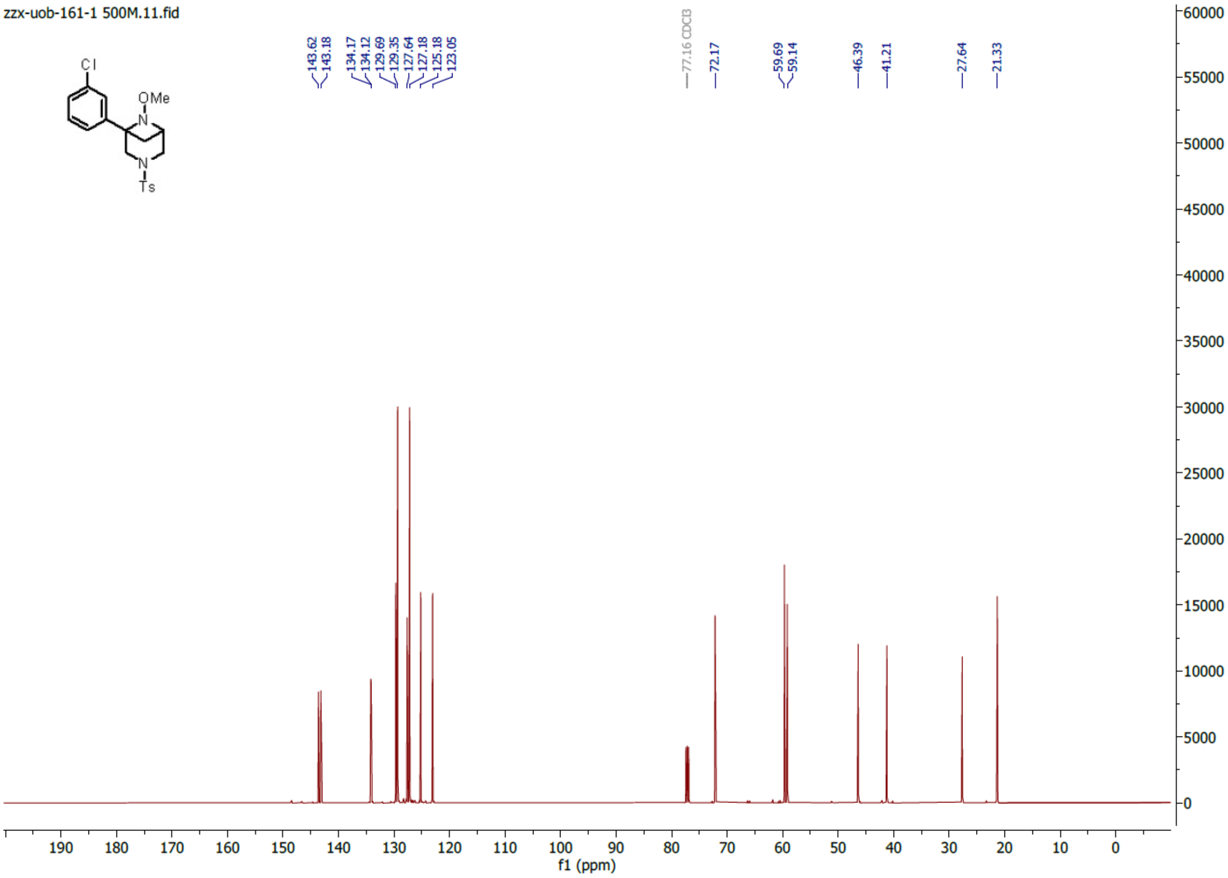
zzx-uob-161-1 500M.11.fid
143.62
143.18
134.17
134.12
129.69
129.35
127.64
127.18
125.18
123.05
77.16 CDCl3
72.17
59.69
59.14
46.39
41.21
27.64
21.33
f1 (ppm)

Compound 8a

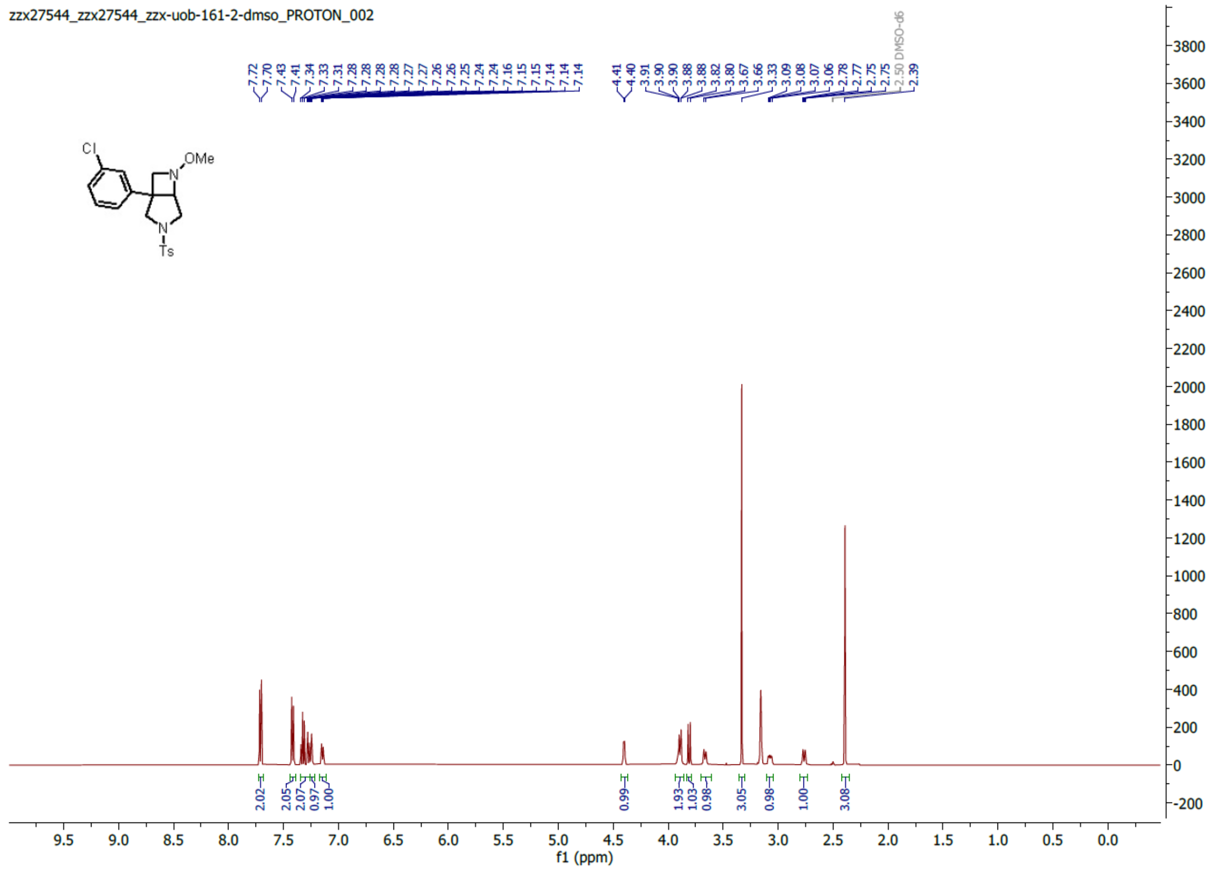
zzx27544_zzx27544_zzx-uob-161-2-dmso_PROTON_002
7.72
7.70
7.43
7.41
7.34
7.33
7.31
7.28
7.28
7.28
7.28
7.27
7.27
7.26
7.26
7.25
7.24
7.24
7.16
7.15
7.15
7.14
7.14
7.14
4.41
4.40
3.91
3.90
3.90
3.88
3.88
3.82
3.80
3.67
3.66
3.33
3.09
3.08
3.07
3.06
2.78
2.77
2.75
2.75
2.50 DMSO-d6
2.39
Cl
N
OMe
N
Ts
2.02
2.05
2.07
0.97
1.00
0.99
1.93
1.03
0.98
3.05
0.98
1.00
3.08
f1 (ppm)

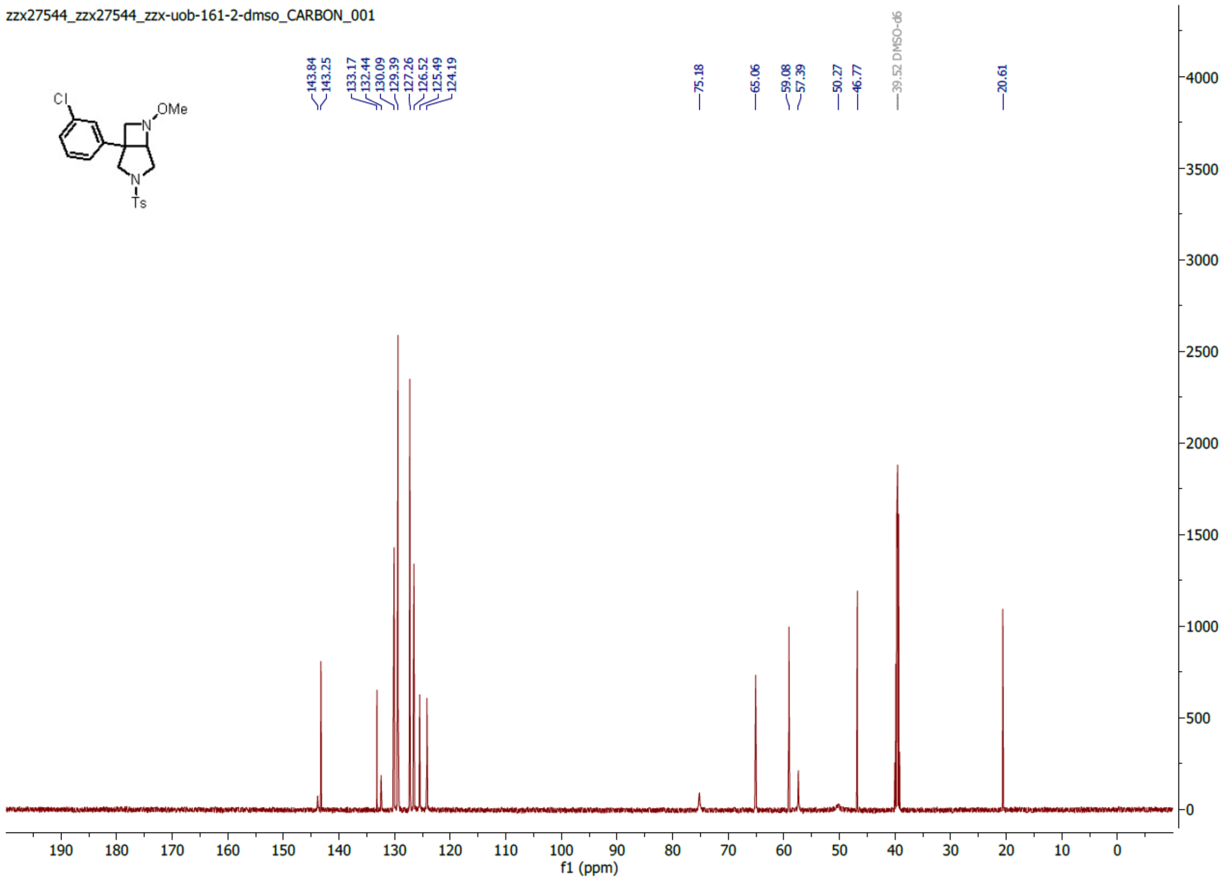
zzx27544_zzx27544_zzx-uob-161-2-dmso_CARBON_001
143.84
143.25
133.17
132.44
130.09
129.39
127.26
126.52
125.49
124.19
75.18
65.06
59.08
57.39
50.27
46.77
39.52 DMSO-d6
20.61
Cl
N
OMe
N
Ts
f1 (ppm)

Compound 9

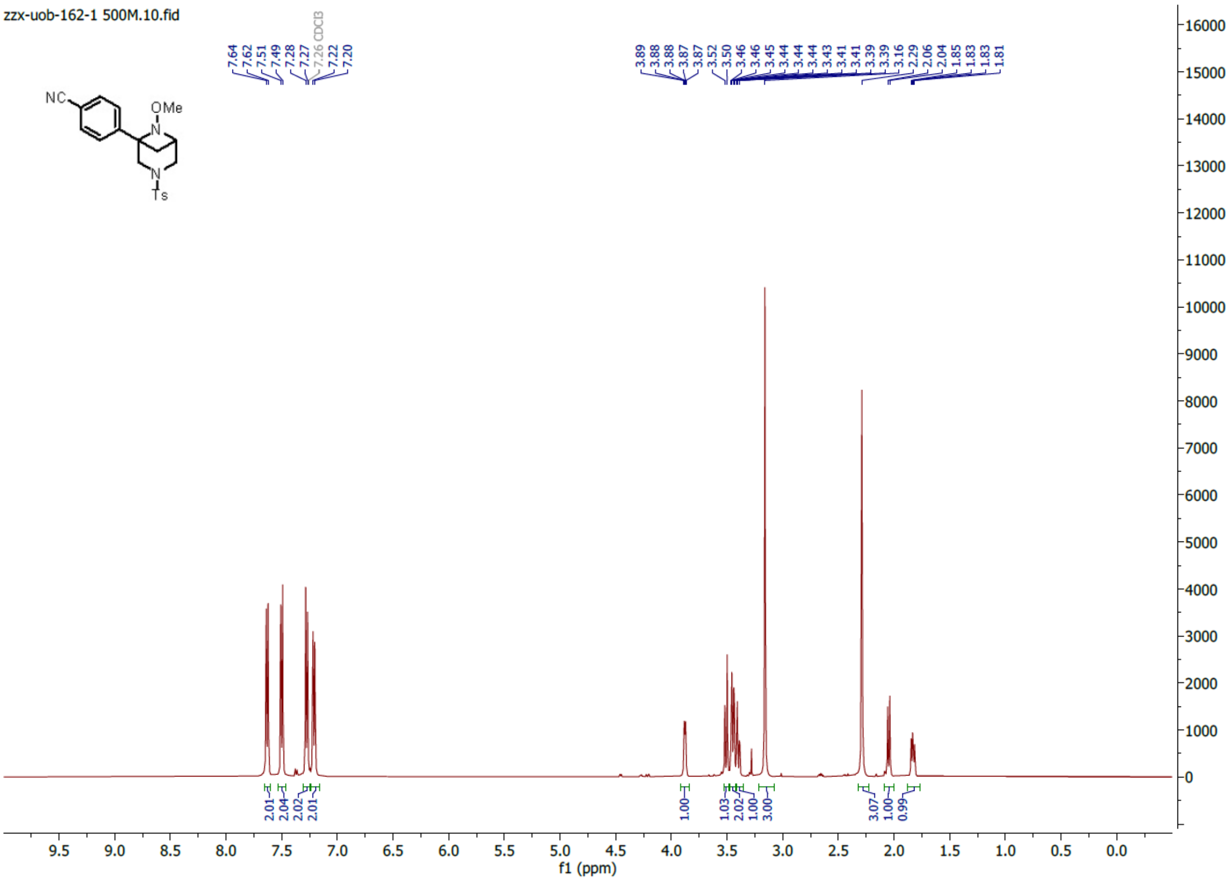

zzx-uob-162-1 500M.10.fid
NC
OMe
N
N
Ts
7.64 7.62 7.51 7.49 7.28 7.27 7.26 CDCl3 7.22 7.20
3.89 3.88 3.88 3.87 3.87 3.52 3.50 3.46 3.46 3.45 3.44 3.44 3.44 3.43 3.41 3.41 3.39 3.39 3.16 2.29 2.06 2.04 1.85 1.83 1.83 1.81
2.01 2.04 2.02 2.01 1.00 1.03 2.02 1.00 3.00 3.07 1.00 0.99
f1 (ppm)

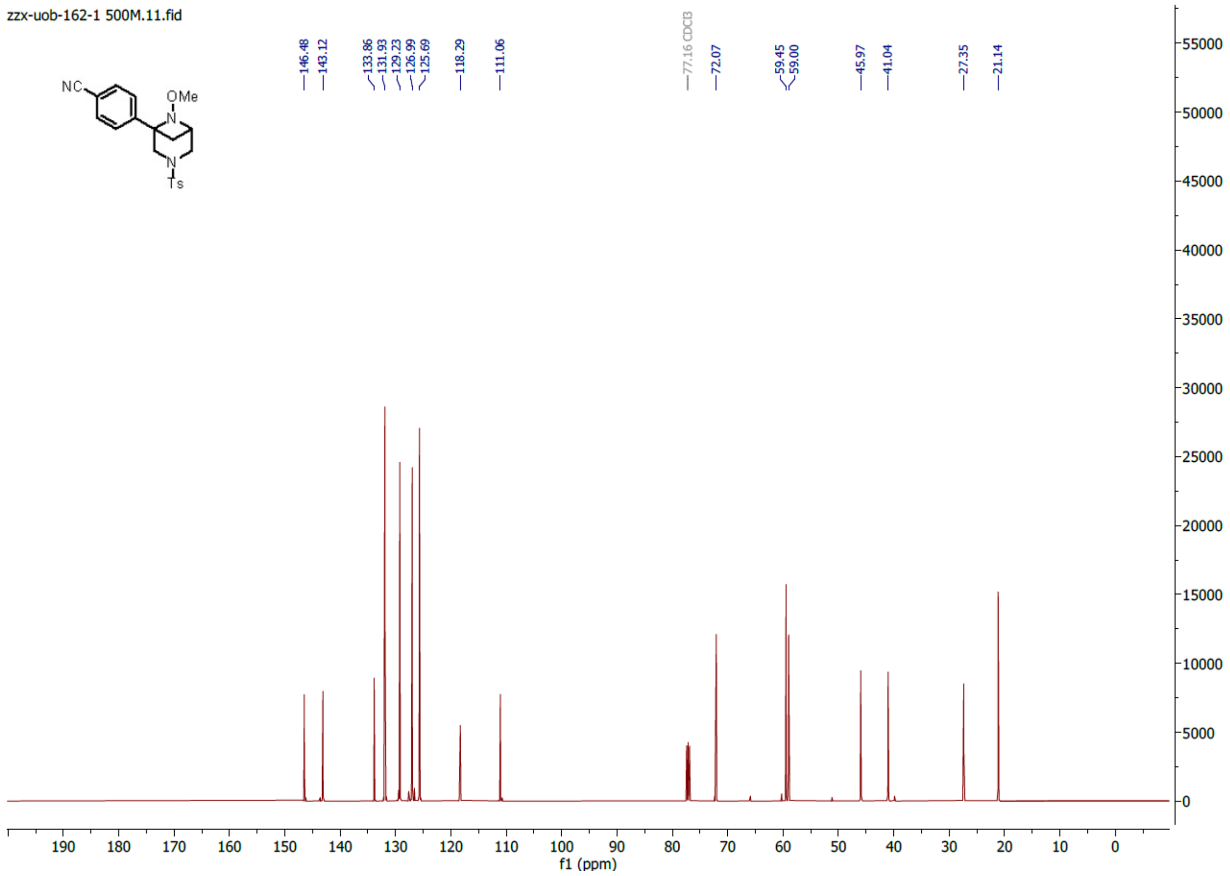

zzx-uob-162-1 500M.11.fid
NC
OMe
N
N
Ts
146.48 143.12 133.86 131.93 129.23 126.99 125.69 118.29 111.06
77.16 CDCl3 72.07 59.45 59.00 45.97 41.04 27.35 21.14
f1 (ppm)

Compound 9a

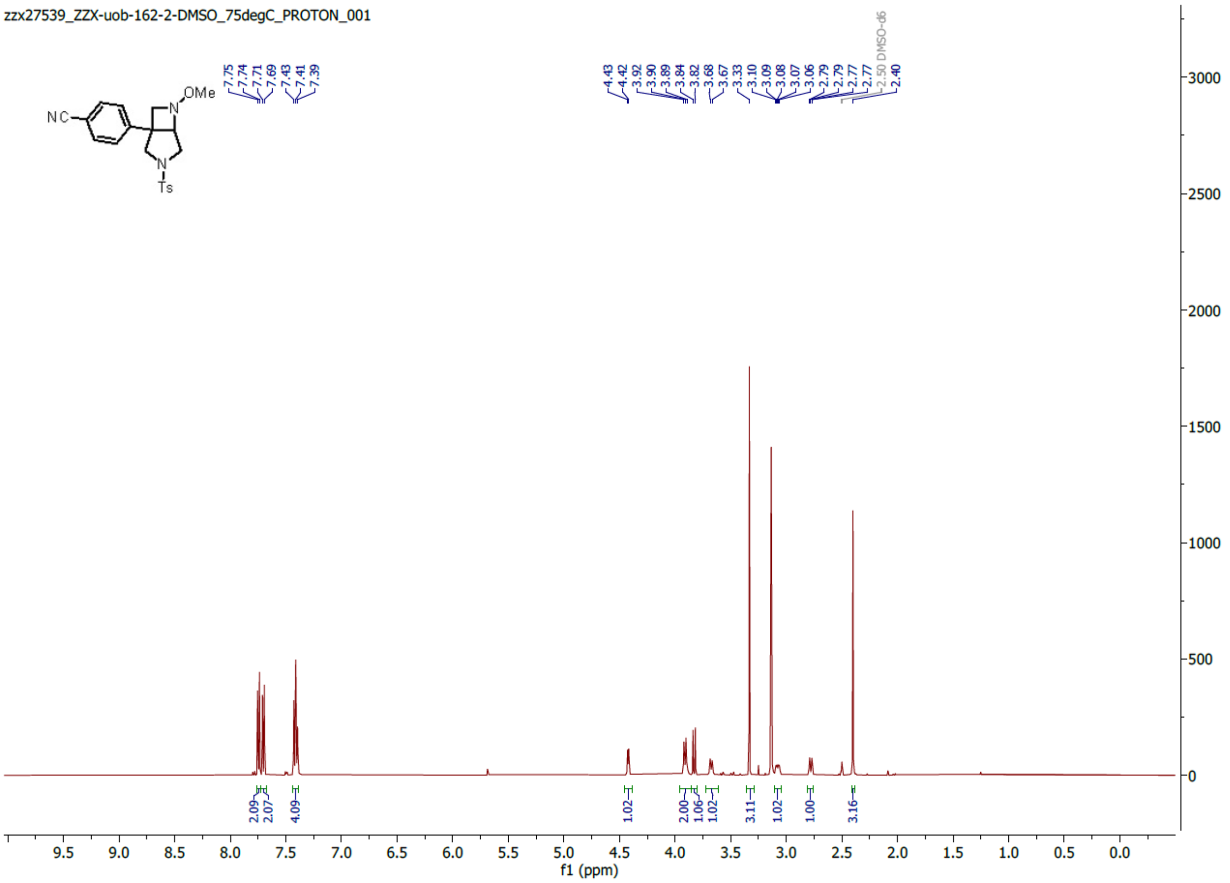

zzx27539_ZZX-uob-162-2-DMSO_75degC_PROTON_001
NC
N
OMe
N
Ts
7.75
7.74
7.71
7.69
7.43
7.41
7.39
4.43
4.42
3.92
3.90
3.89
3.84
3.82
3.68
3.67
3.33
3.10
3.09
3.08
3.07
3.06
2.79
2.79
2.77
2.77
2.50 DMSO-d6
2.40
2.09
2.07
4.09
1.02
2.00
1.06
1.02
3.11
1.02
1.00
3.16
f1 (ppm)

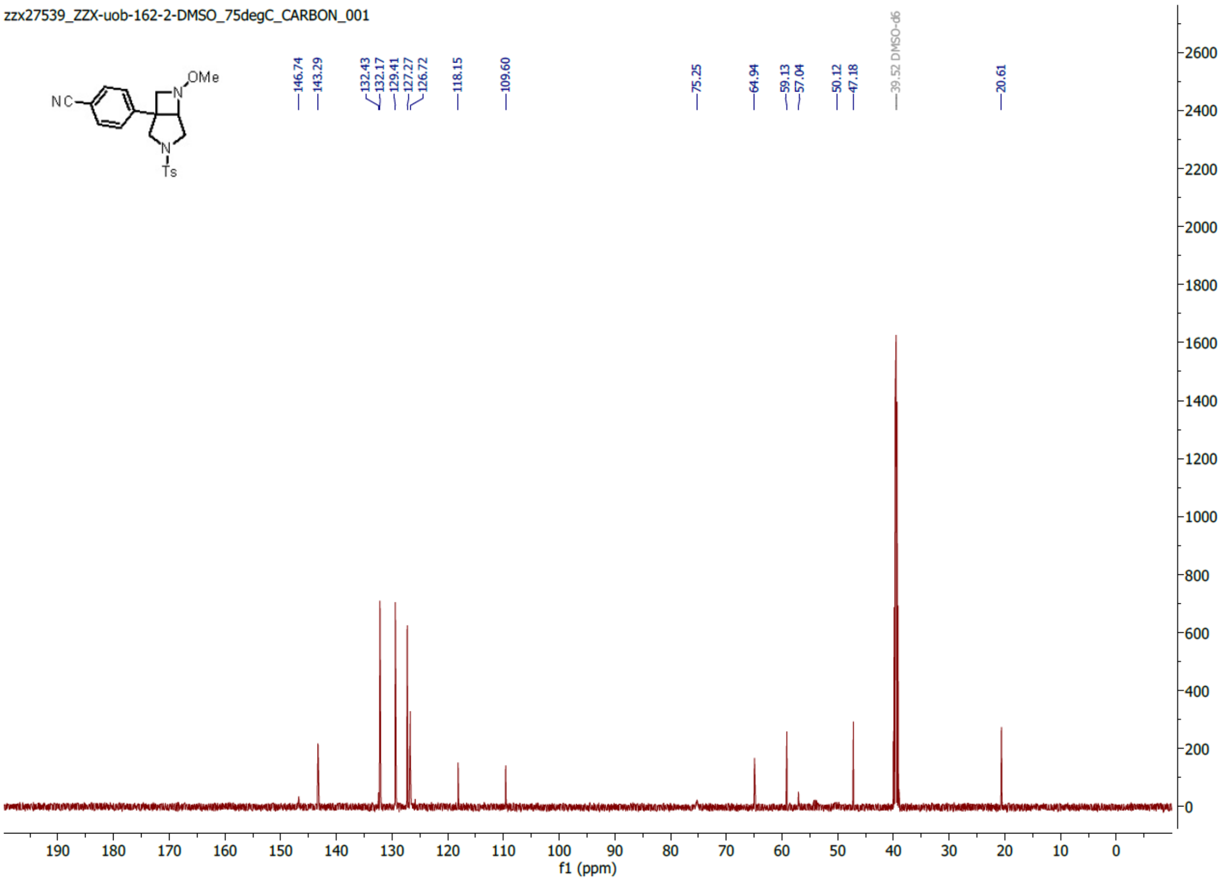

zzx27539_ZZX-uob-162-2-DMSO_75degC_CARBON_001
NC
N
OMe
N
Ts
146.74
143.29
132.43
132.17
129.41
127.27
126.72
118.15
109.60
75.25
64.94
59.13
57.04
50.12
47.18
39.52 DMSO-d6
20.61
f1 (ppm)

Compound 10

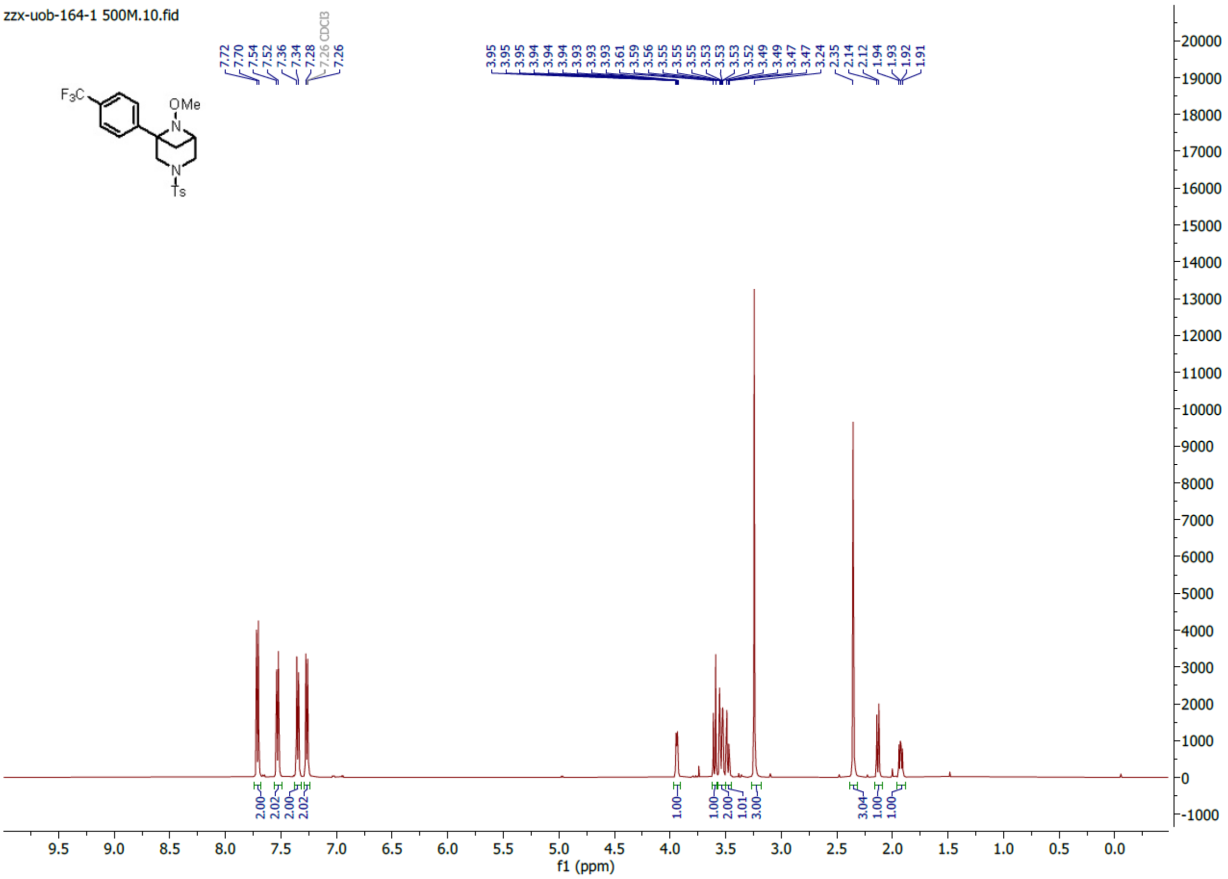
zzx-uob-164-1 500M.10.fid
7.72 7.70 7.54 7.52 7.36 7.34 7.28 7.26 CDCl3 7.26
3.95 3.95 3.95 3.94 3.94 3.94 3.93 3.93 3.93 3.93 3.61 3.59 3.56 3.56 3.55 3.55 3.55 3.53 3.53 3.53 3.52 3.49 3.49 3.47 3.47 3.24 2.35 2.14 2.12 1.94 1.93 1.92 1.91
F3C
OMe
N
N
Ts
2.00 2.02 2.00 2.02
1.00 1.00 2.00 1.01 3.00
3.04 1.00 1.00
f1 (ppm)

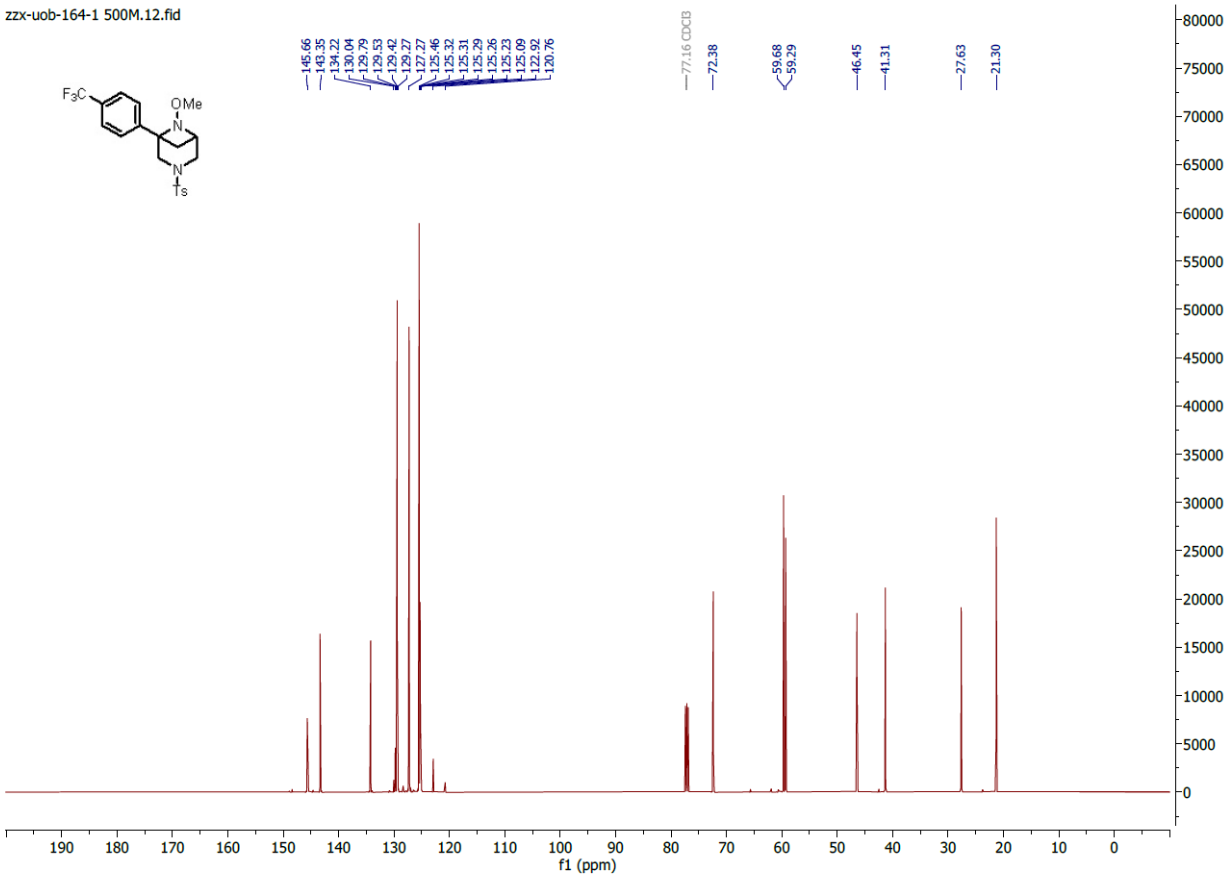
zzx-uob-164-1 500M.12.fid
145.66 143.35 134.22 130.04 129.79 129.53 129.42 129.27 127.27 125.46 125.32 125.31 125.29 125.26 125.23 125.09 122.92 120.76
77.16 CDCl3
72.38
59.68 59.29
46.45
41.31
27.63
21.30
F3C
OMe
N
N
Ts
f1 (ppm)

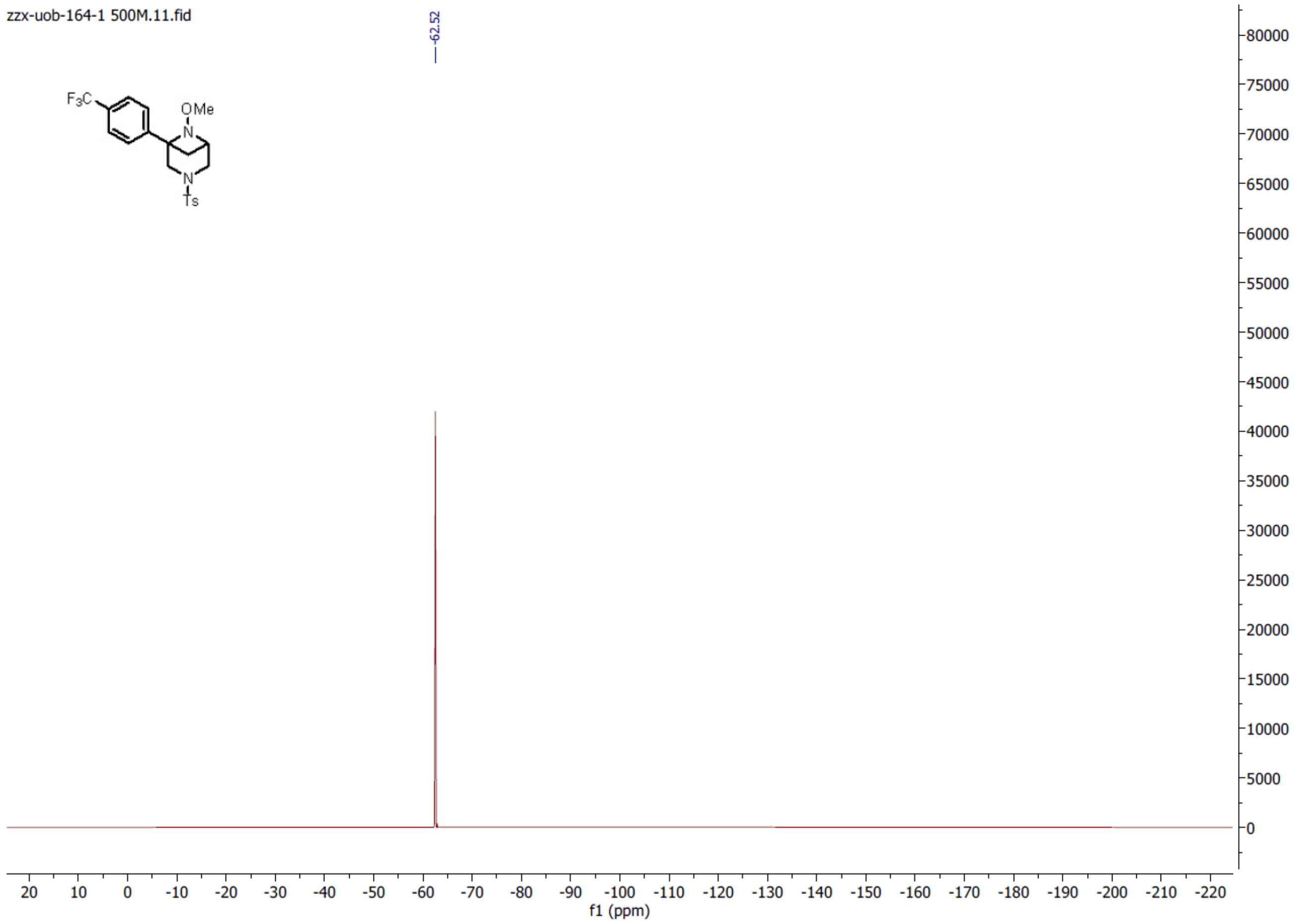
zzx-uob-164-1 500M.11.fid
-62.52
F3C
OMe
N
N
Ts
80000
75000
70000
65000
60000
55000
50000
45000
40000
35000
30000
25000
20000
15000
10000
5000
0
20 10 0 -10 -20 -30 -40 -50 -60 -70 -80 -90 -100 -110 -120 -130 -140 -150 -160 -170 -180 -190 -200 -210 -220
f1 (ppm)

Compound 10a

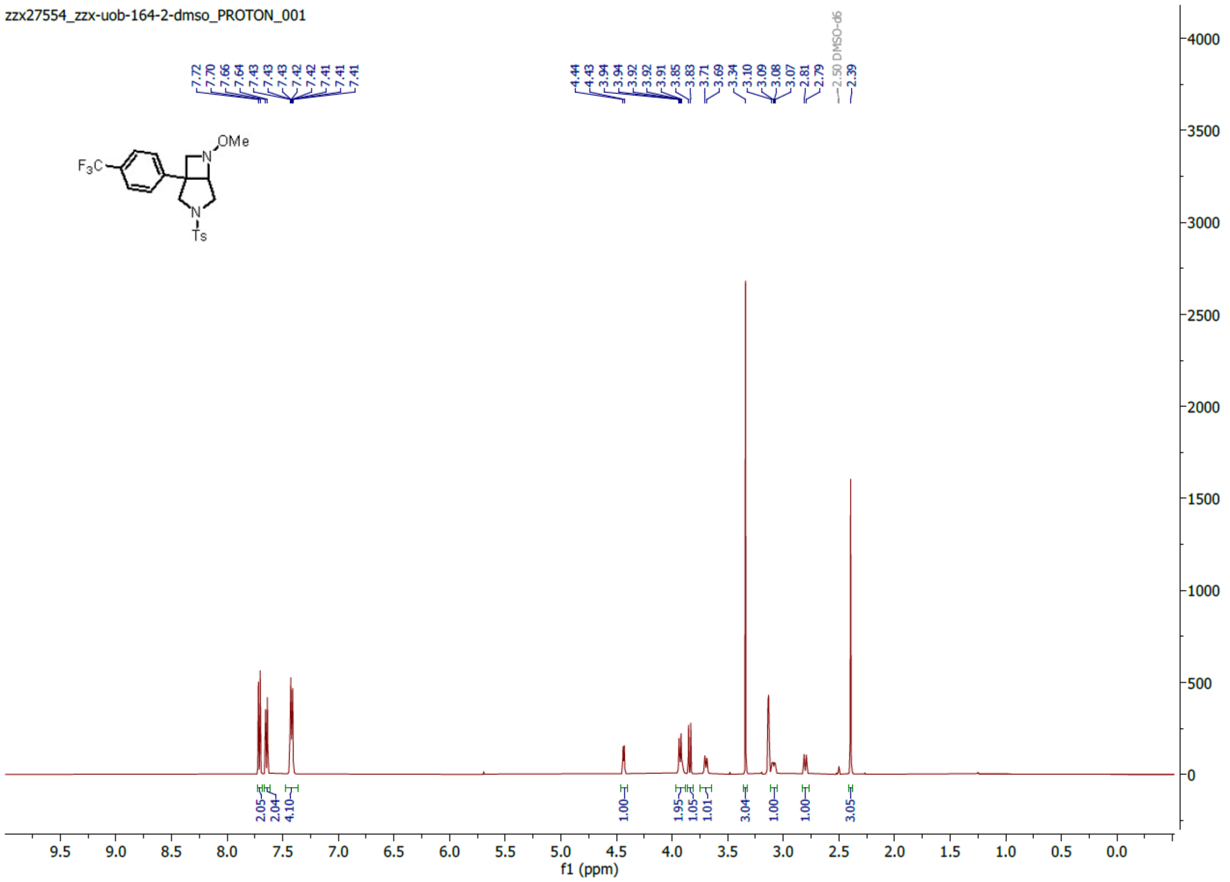

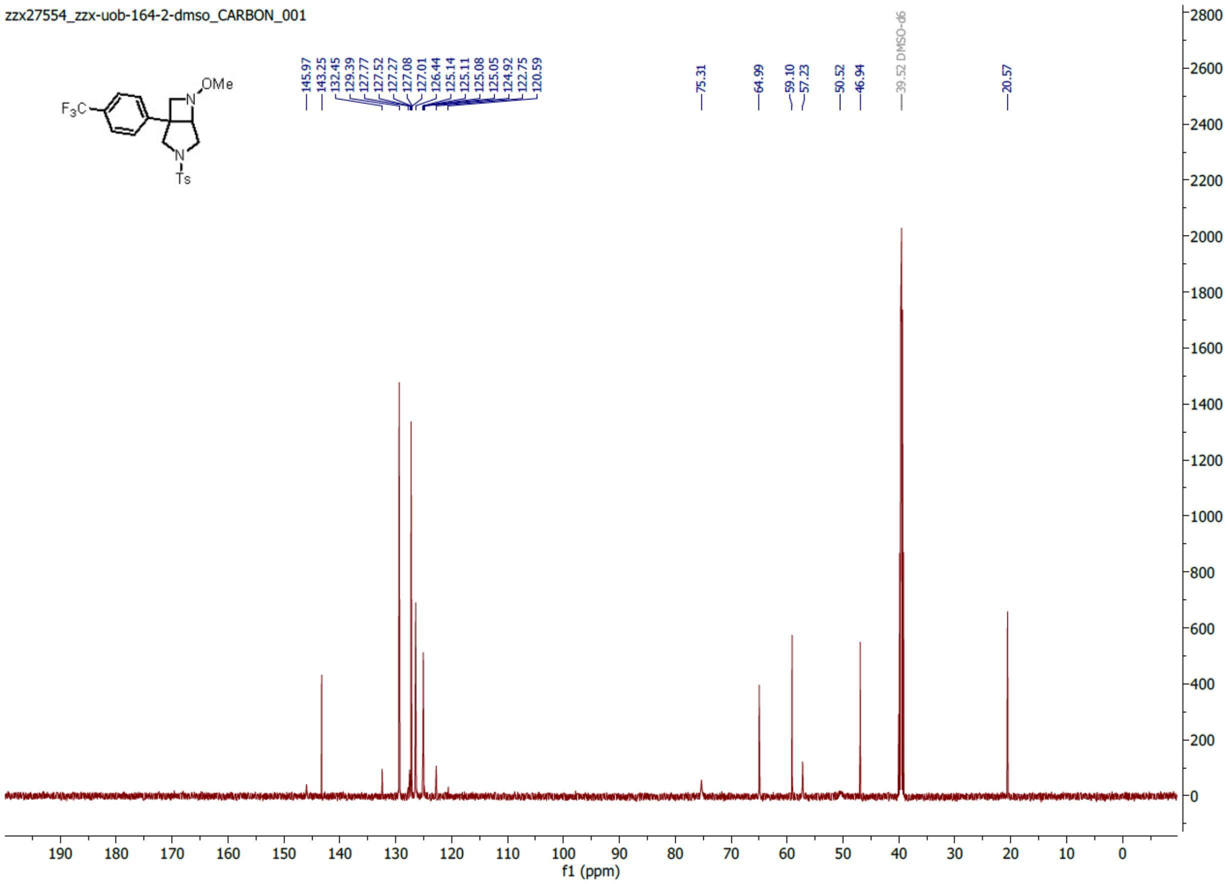

zzx27576_zzx-uob-164-2-DMSO-sec_FLUORINE_001
STANDARD PROTON PARAMETERS

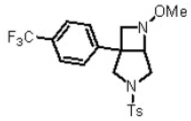

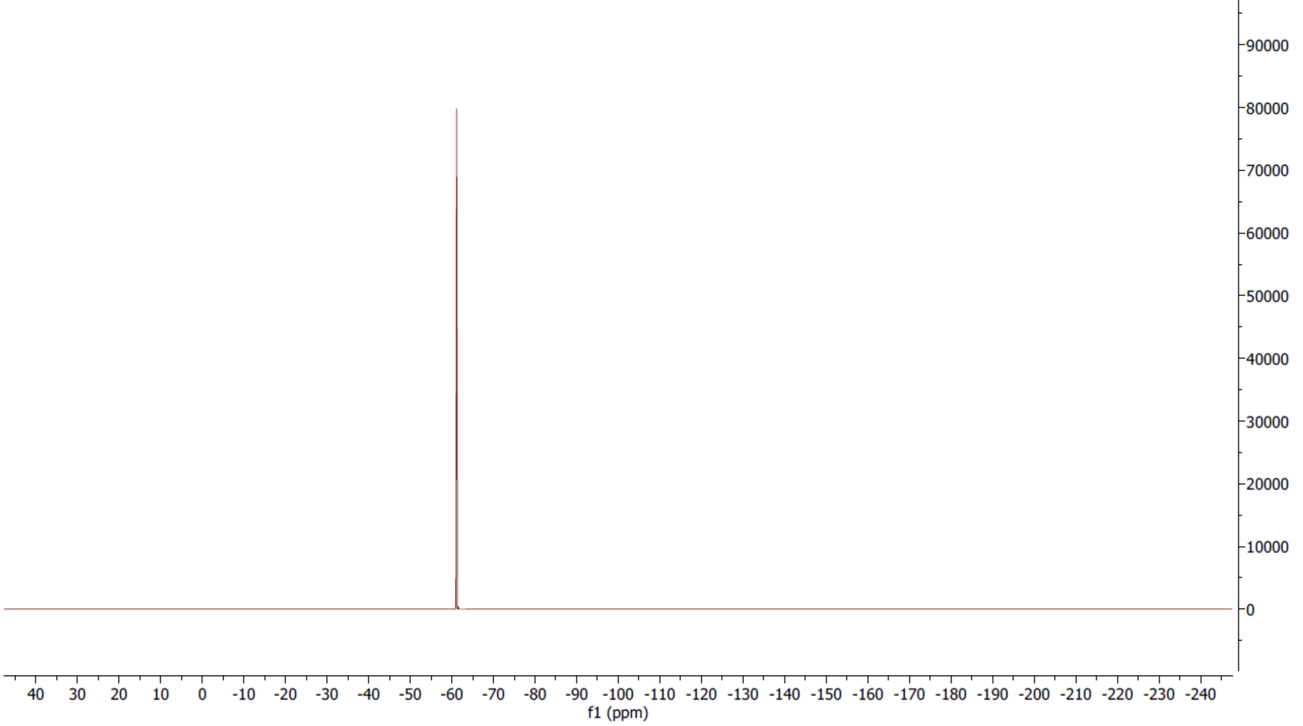

Compound 11

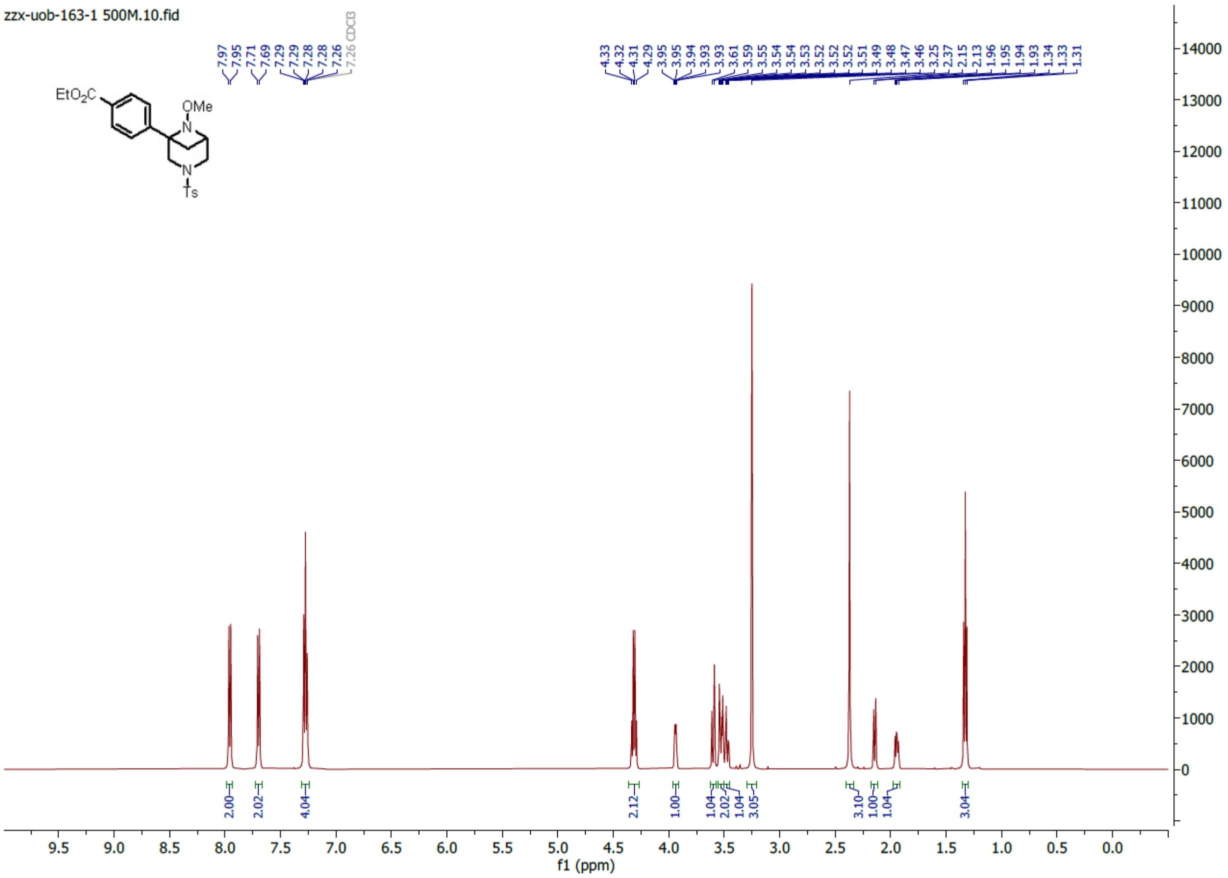
zzx-uob-163-1 500M.10.fid
EtO2C
OMe
N
N
Ts
7.97 7.95 7.71 7.69 7.29 7.29 7.28 7.28 7.26 7.26 CDCl3
4.33 4.32 4.31 4.29 3.95 3.95 3.94 3.93 3.93 3.61 3.59 3.55 3.54 3.54 3.53 3.52 3.52 3.52 3.51 3.49 3.48 3.47 3.46 3.25 2.37 2.15 2.13 1.96 1.95 1.94 1.93 1.34 1.33 1.31
2.00 2.02 4.04 2.12 1.00 1.04 2.02 1.04 3.05 3.10 1.00 1.04 3.04
f1 (ppm)

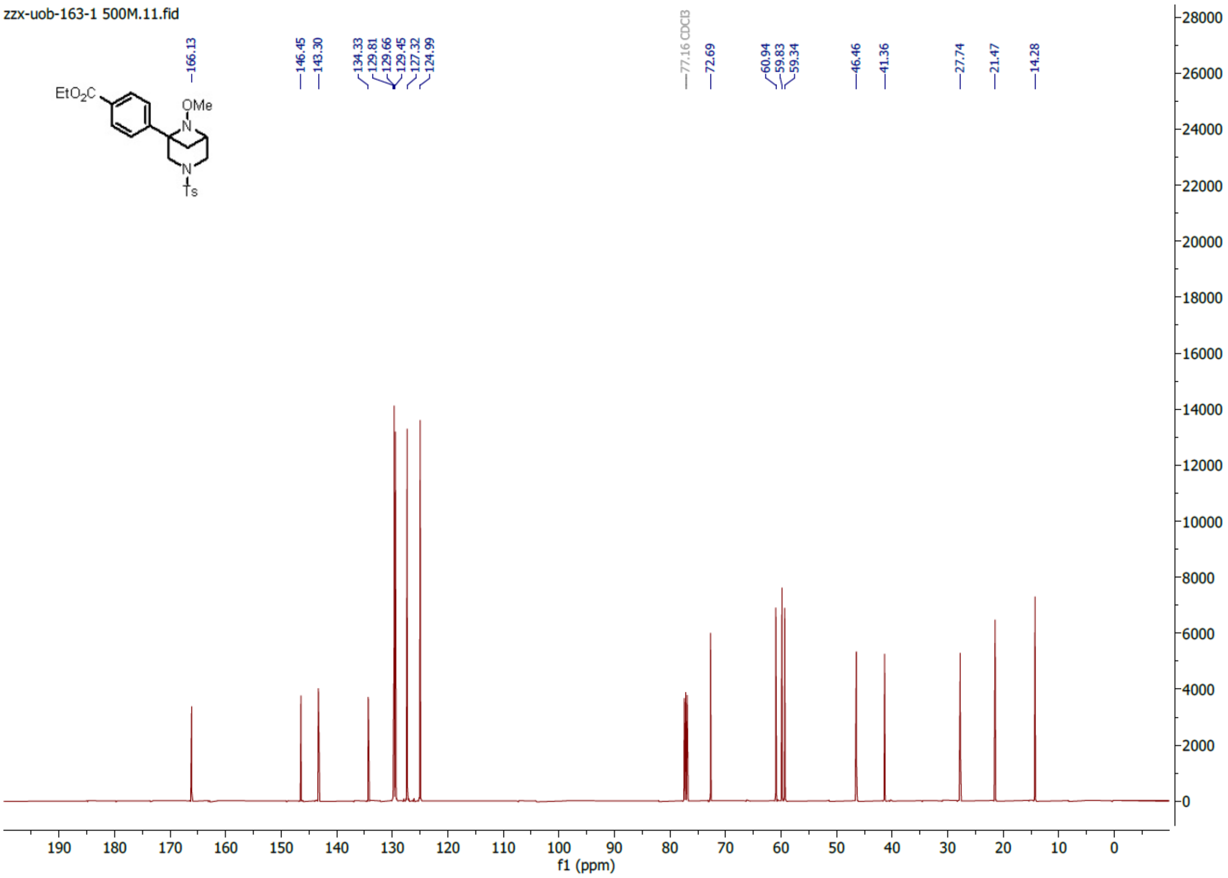
zzx-uob-163-1 500M.11.fid
EtO2C
OMe
N
N
Ts
166.13 146.45 143.30 134.33 129.81 129.66 129.45 127.32 124.99 77.16 CDCl3 72.69 60.94 59.83 59.34 46.46 41.36 27.74 21.47 14.28
f1 (ppm)

Compound 11a

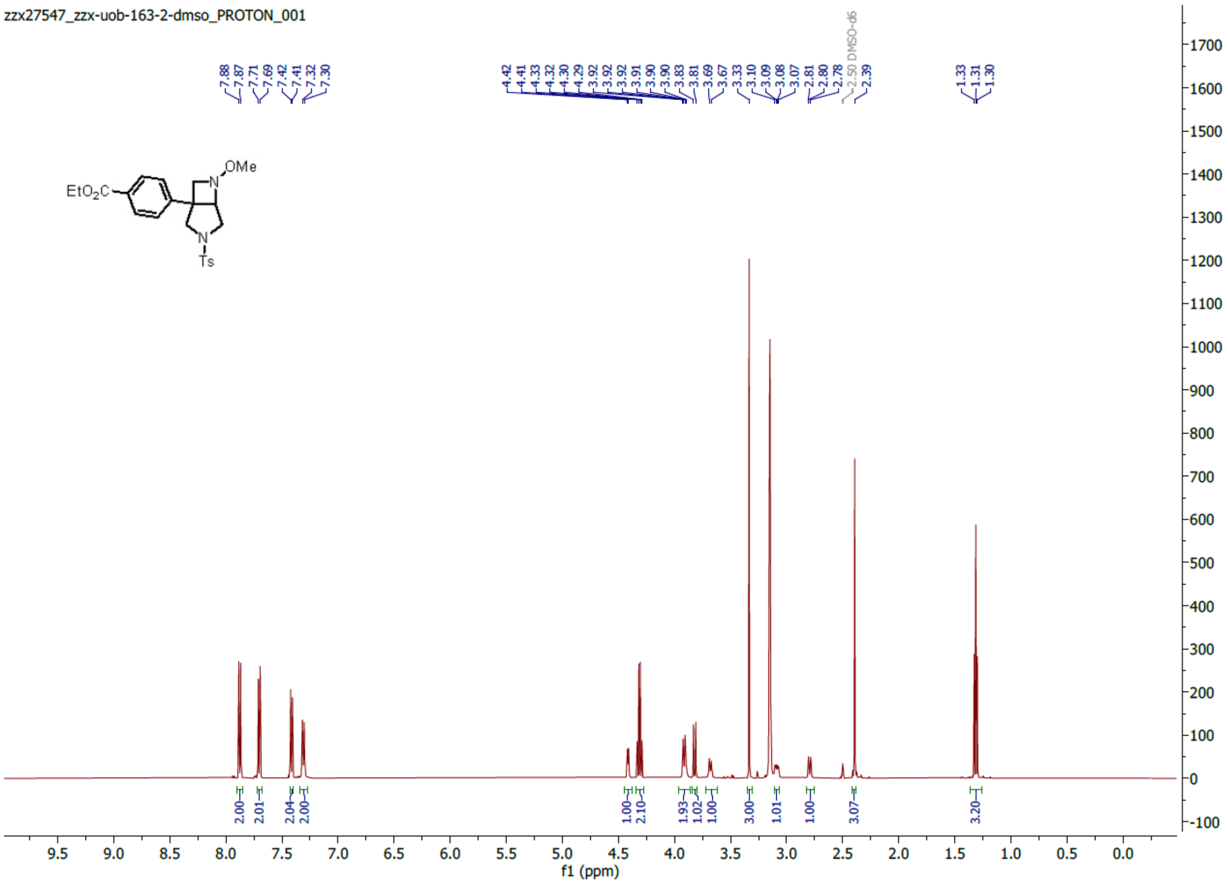

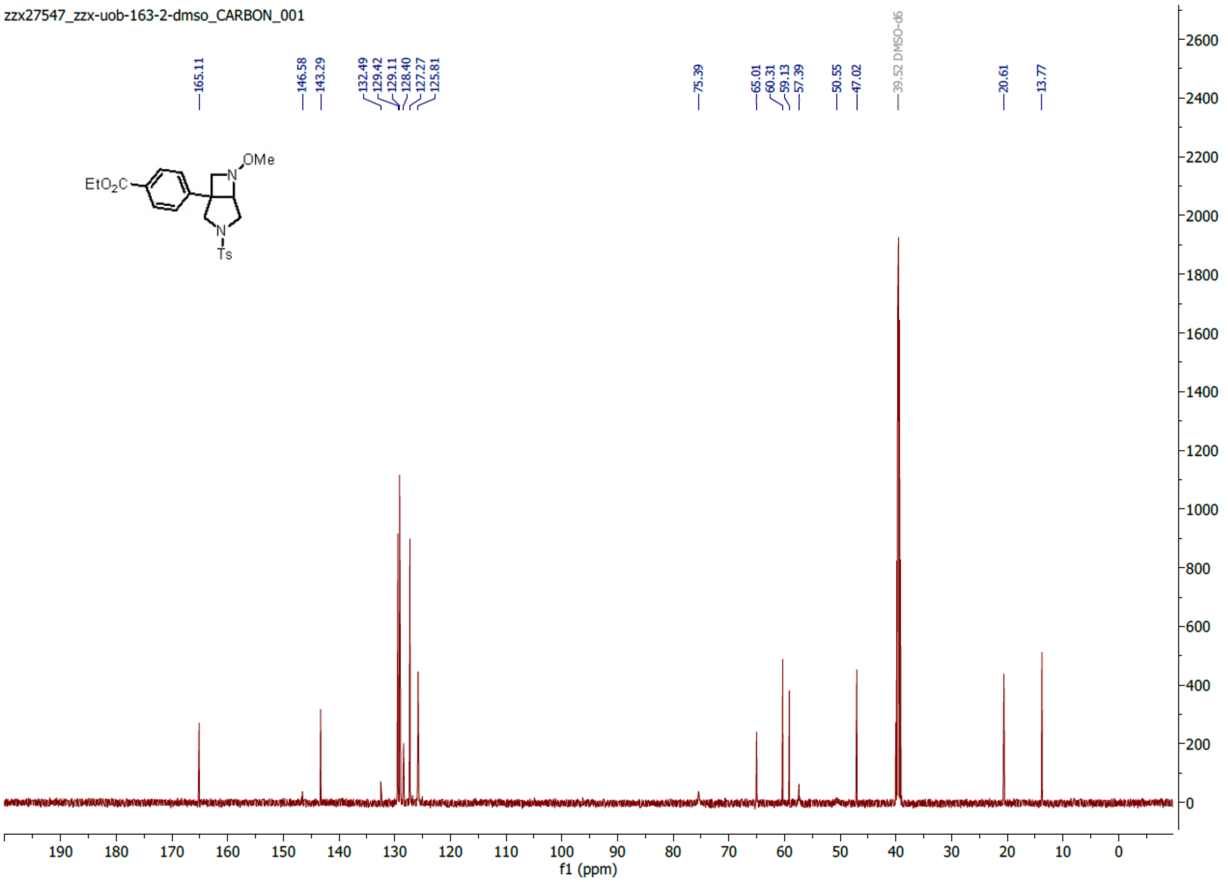

Compound 12

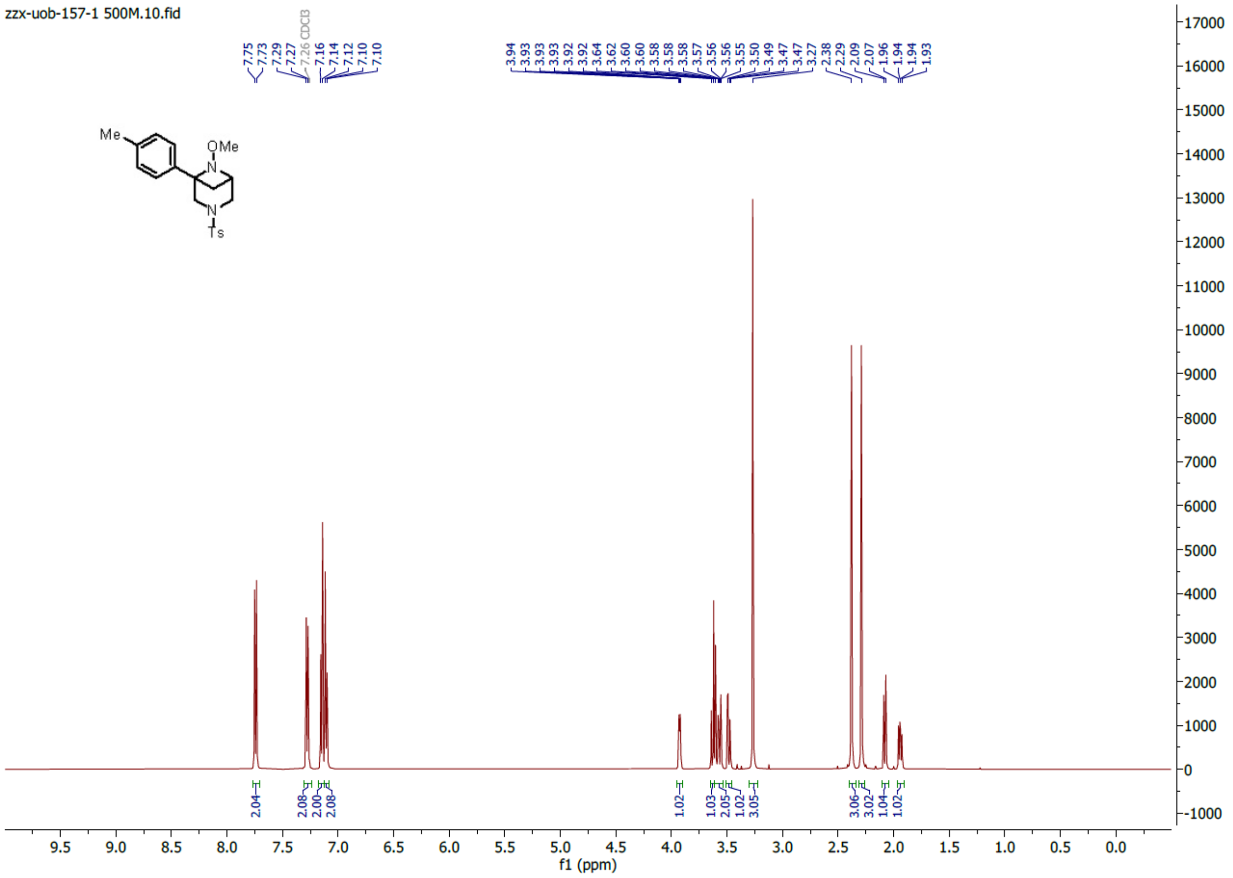

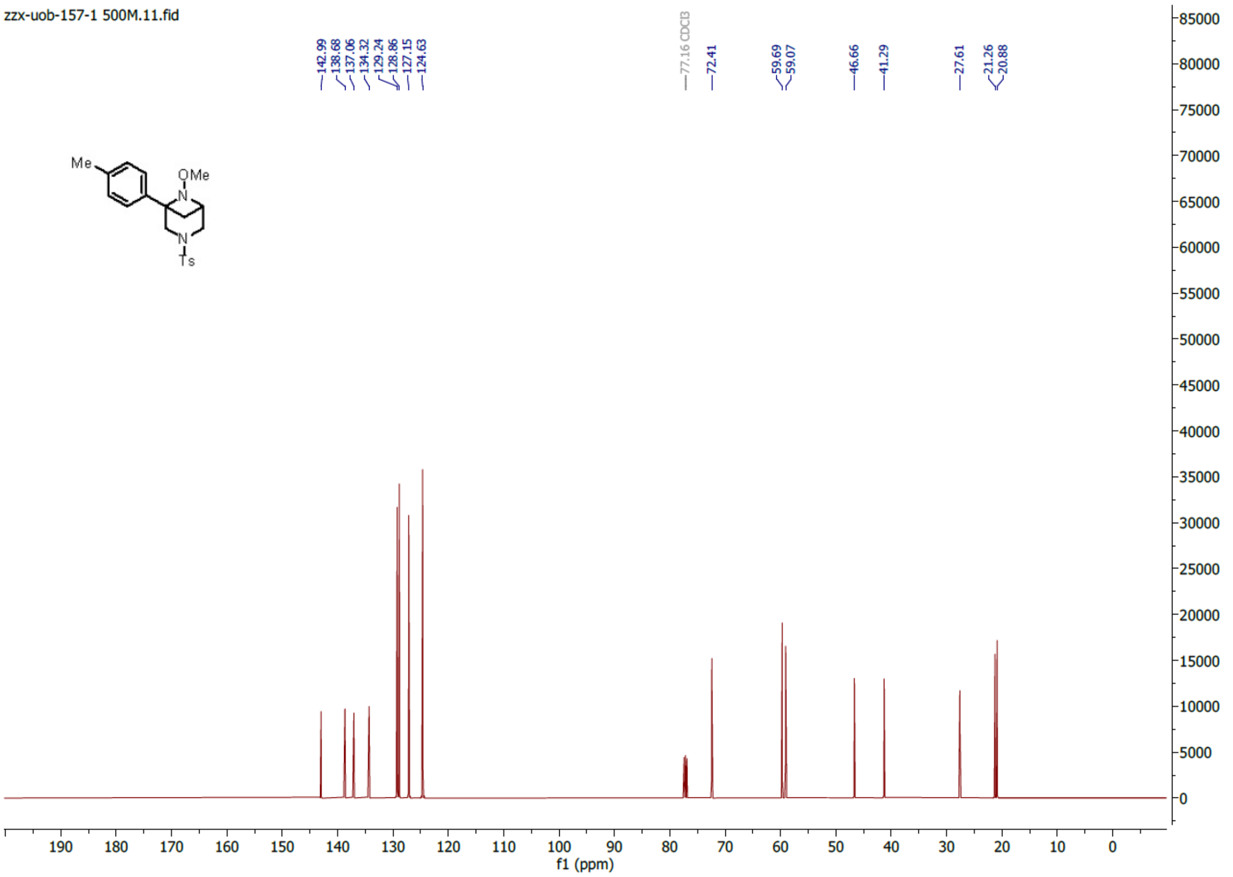

Compound 12a

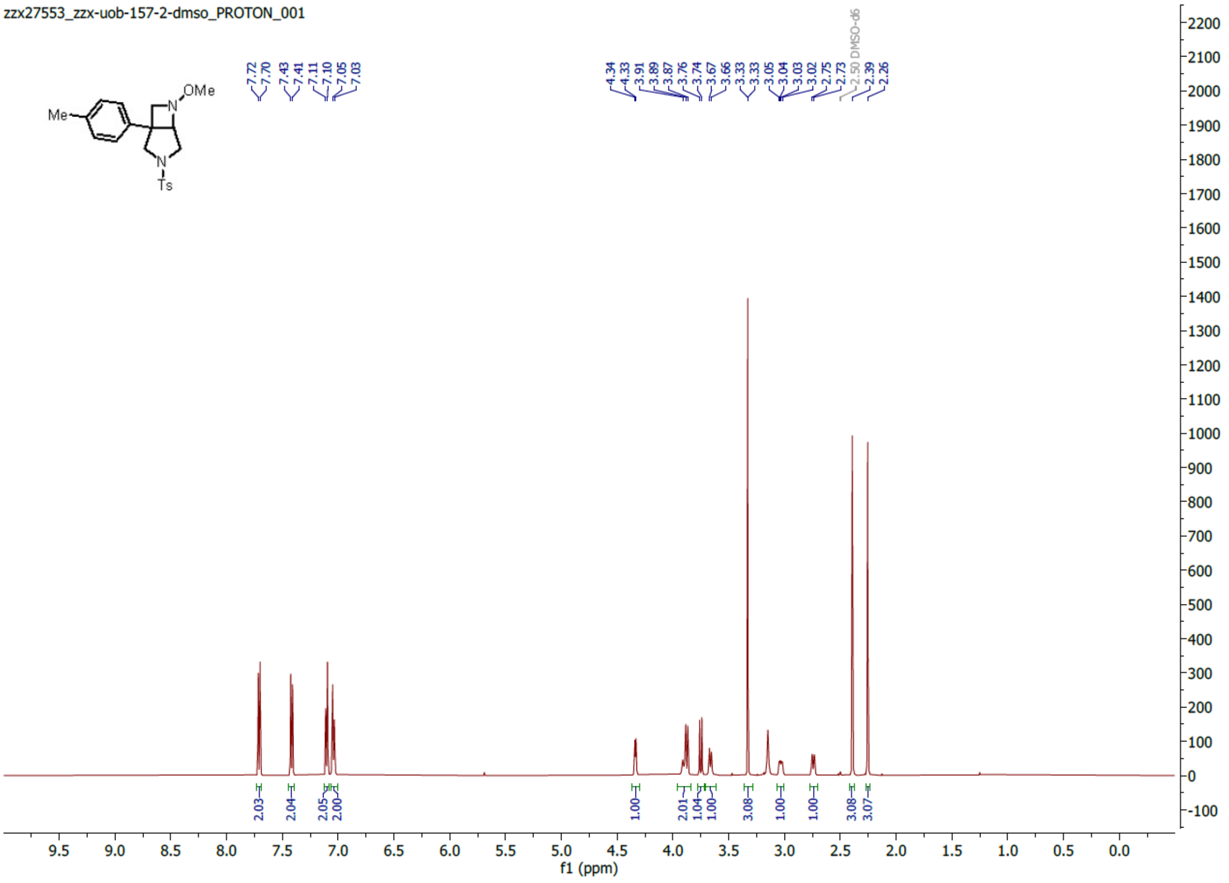

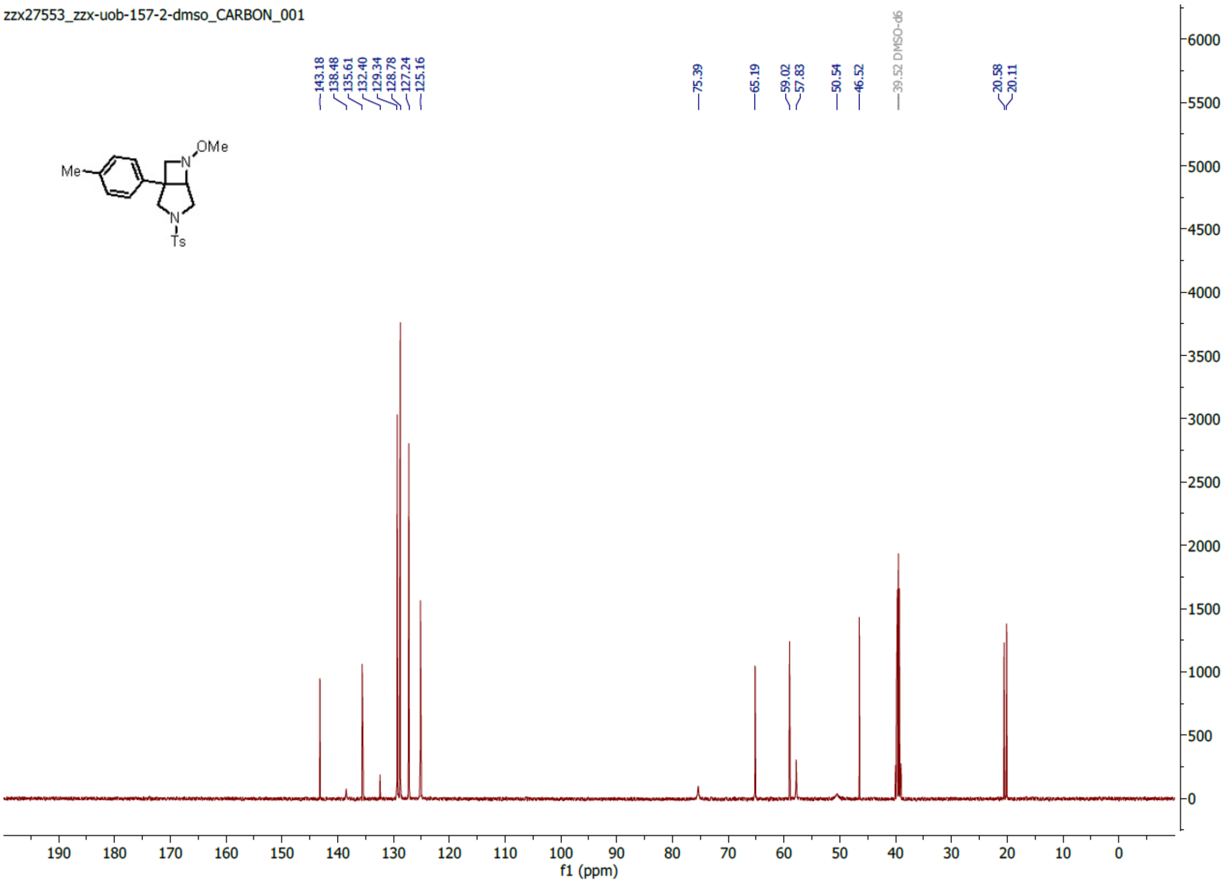

Compound 13

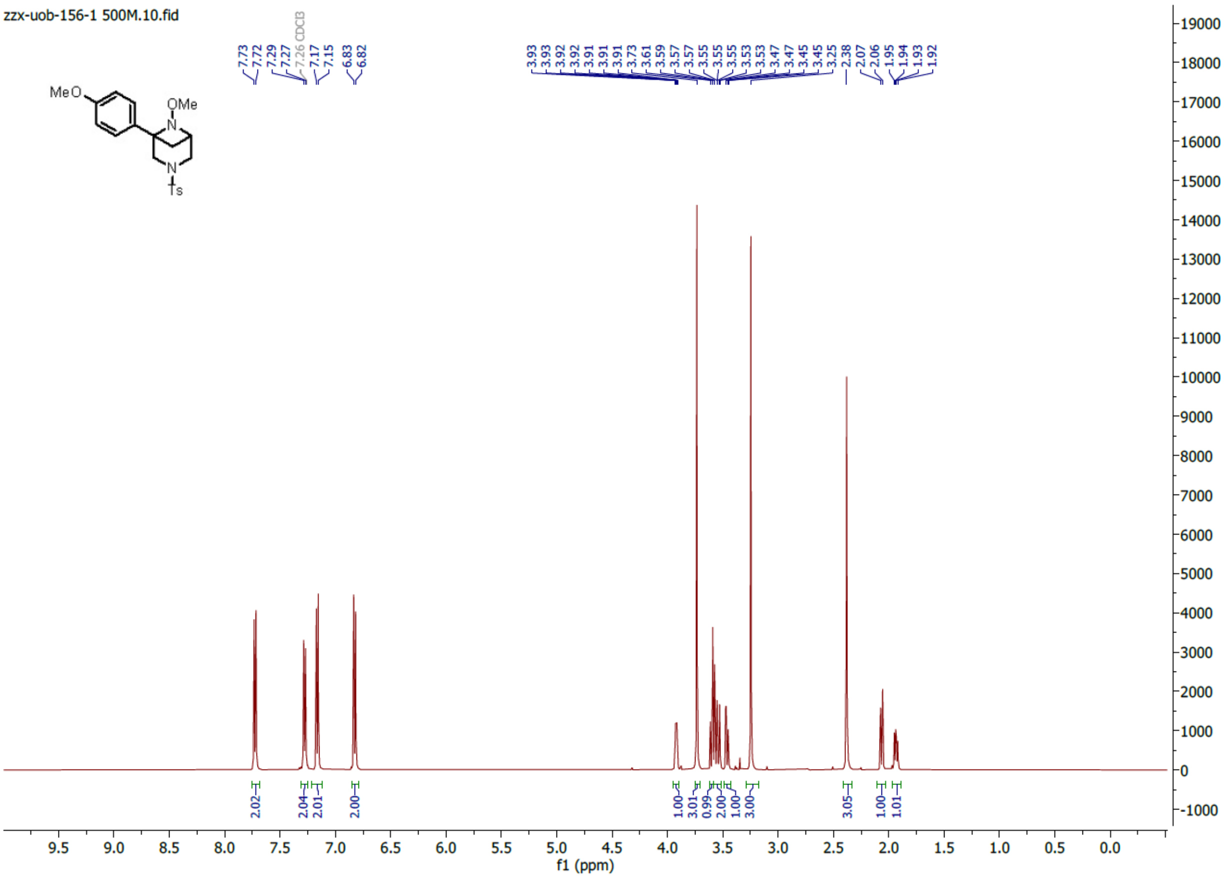
zzx-uob-156-1 500M.10.fid
7.73
7.72
7.29
7.27
7.26 CDCl3
7.17
7.15
6.83
6.82
3.93
3.93
3.92
3.92
3.91
3.91
3.91
3.73
3.61
3.59
3.57
3.57
3.55
3.55
3.55
3.53
3.53
3.47
3.45
3.25
2.38
2.07
2.06
1.95
1.94
1.93
1.92
MeO
OMe
N
N
Ts
2.02
2.04
2.01
2.00
1.00
3.01
0.99
2.00
1.00
3.00
3.05
1.00
1.01
f1 (ppm)

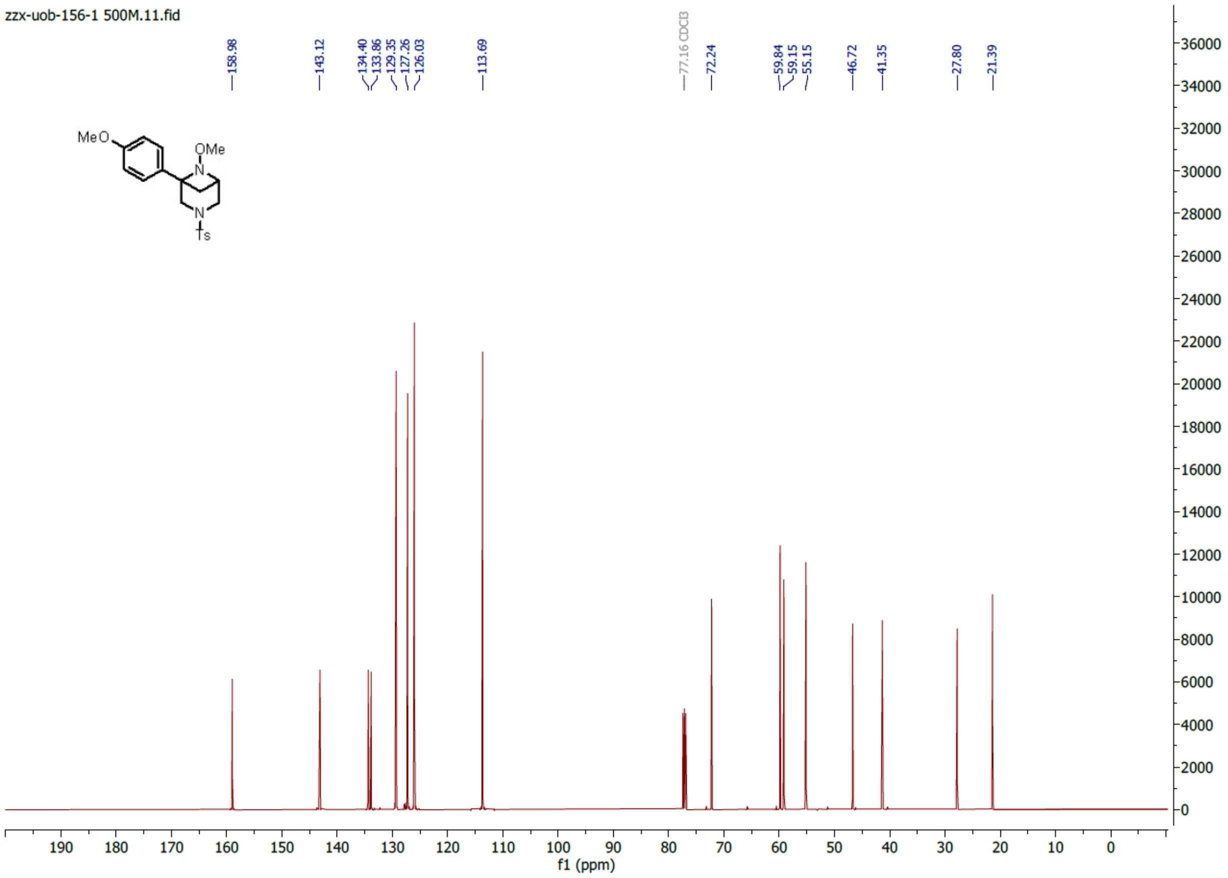
zzx-uob-156-1 500M.11.fid
158.98
143.12
134.40
133.86
129.35
127.26
126.03
113.69
77.16 CDCl3
72.24
59.84
59.15
55.15
46.72
41.35
27.80
21.39
MeO
OMe
N
N
Ts
f1 (ppm)

Compound 13a

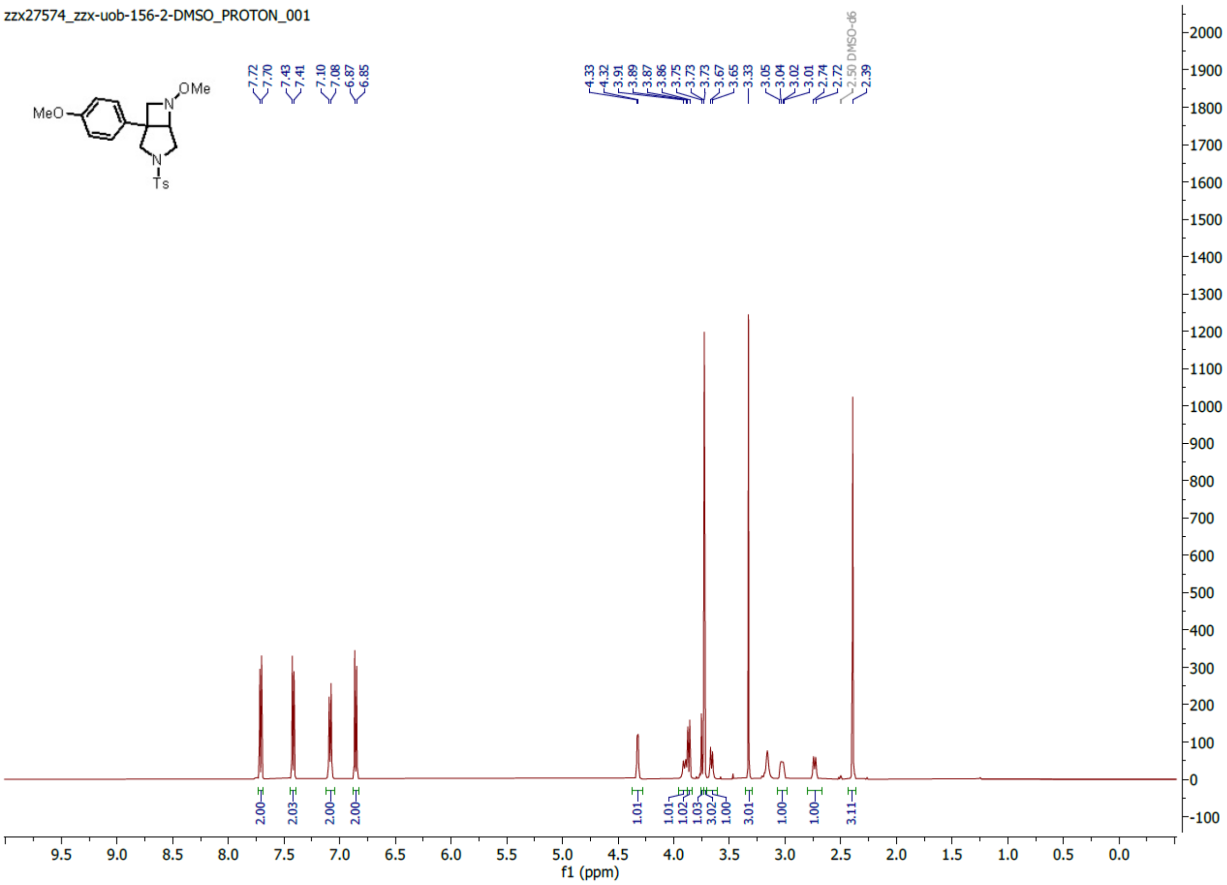

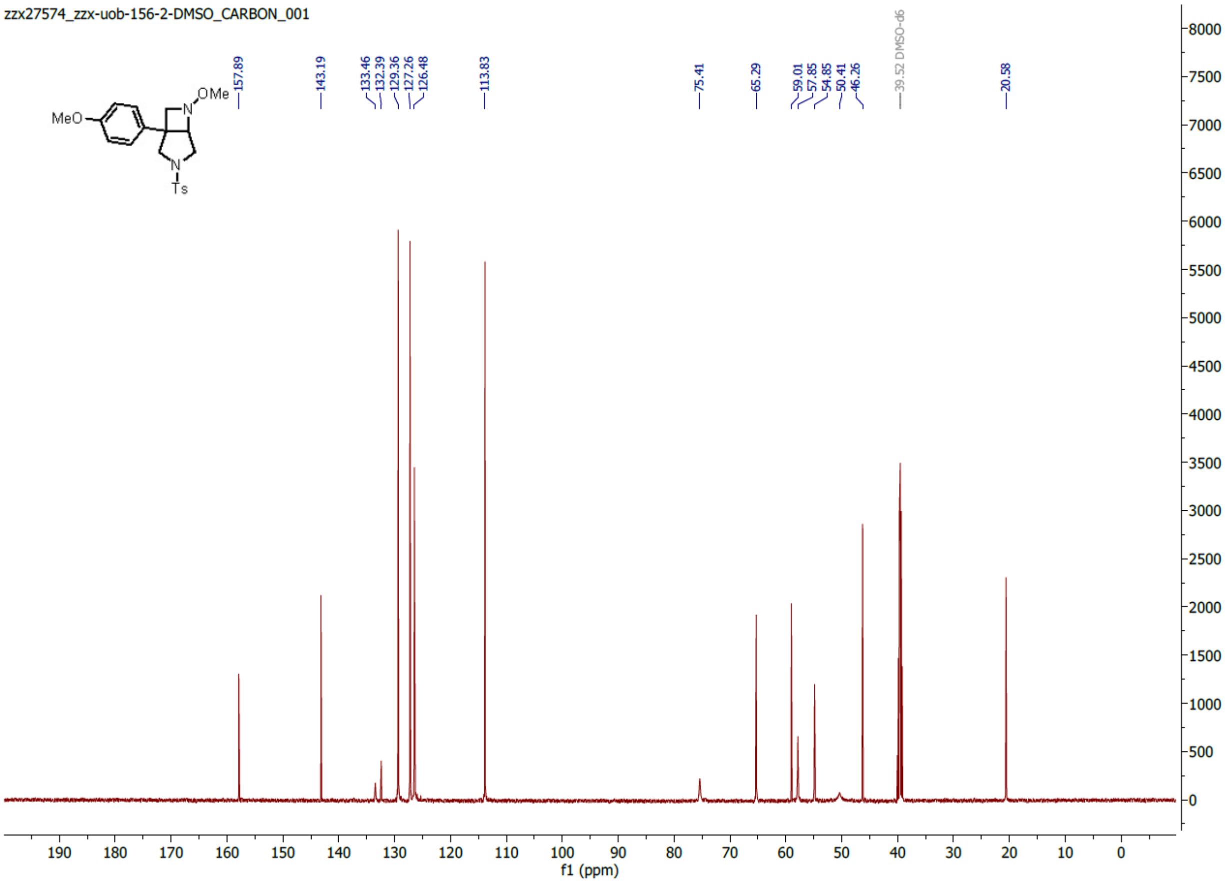

Compound 14

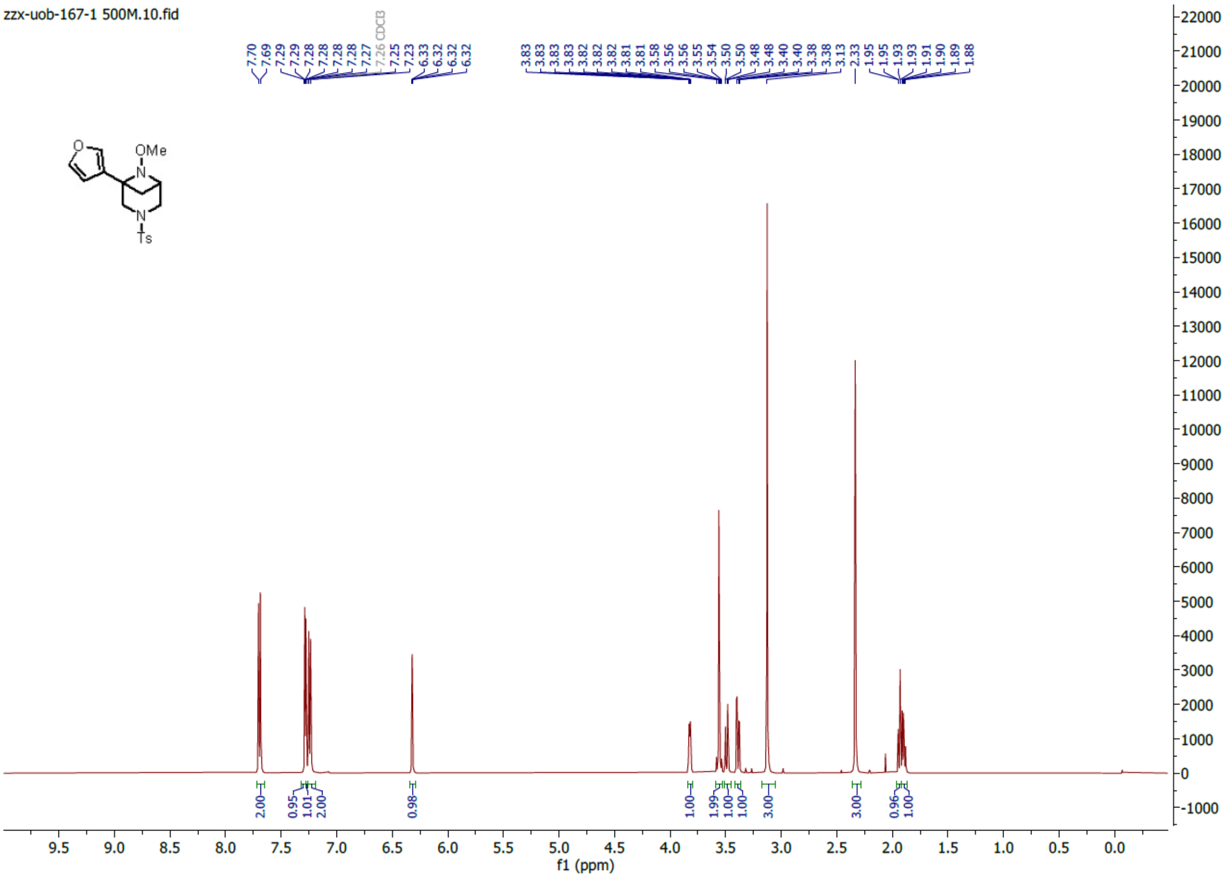
zzx-uob-167-1 500M.10.fid
7.70
7.69
7.29
7.29
7.28
7.28
7.28
7.28
7.27
7.26 CDCl3
7.25
7.23
6.33
6.32
6.32
6.32
3.83
3.83
3.83
3.83
3.82
3.82
3.82
3.81
3.81
3.58
3.56
3.56
3.55
3.54
3.50
3.50
3.48
3.48
3.40
3.40
3.38
3.38
3.13
2.33
1.95
1.95
1.93
1.93
1.91
1.90
1.89
1.88
OMe
N
N
Ts
O
2.00
0.95
1.01
2.00
0.98
1.00
1.99
1.00
1.00
3.00
3.00
0.96
1.00
f1 (ppm)

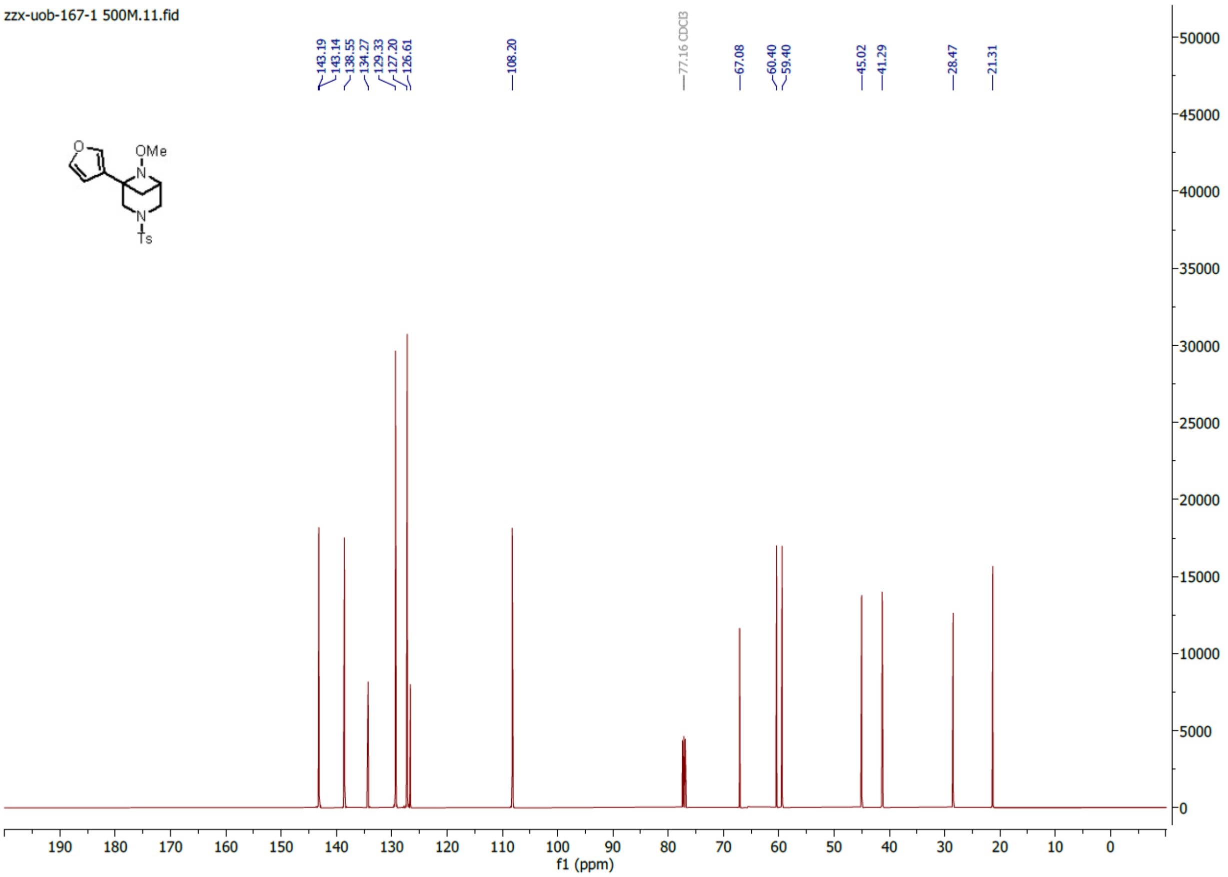
zzx-uob-167-1 500M.11.fid
143.19
143.14
138.55
134.27
129.33
127.20
126.61
108.20
77.16 CDCl3
67.08
60.40
59.40
45.02
41.29
28.47
21.31
OMe
N
N
Ts
O
f1 (ppm)

Compound 14a

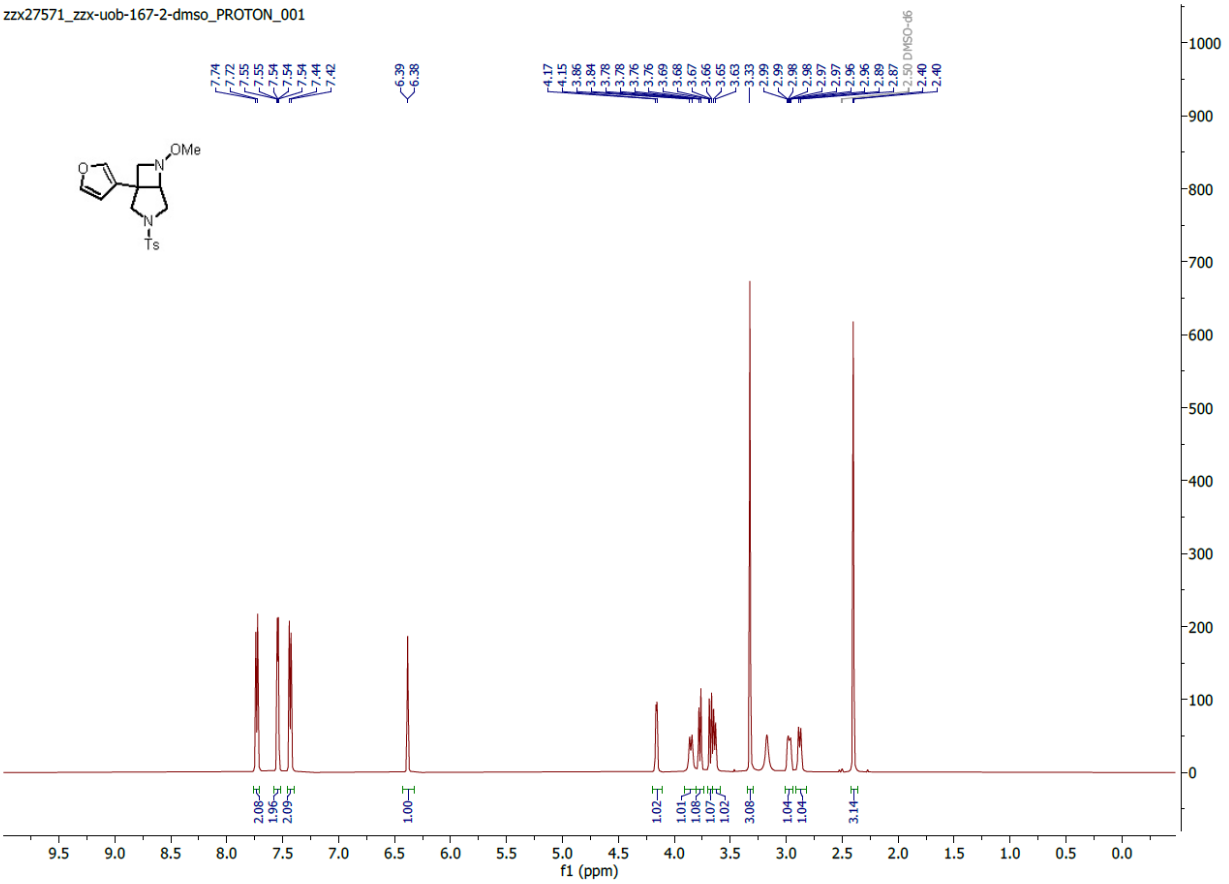

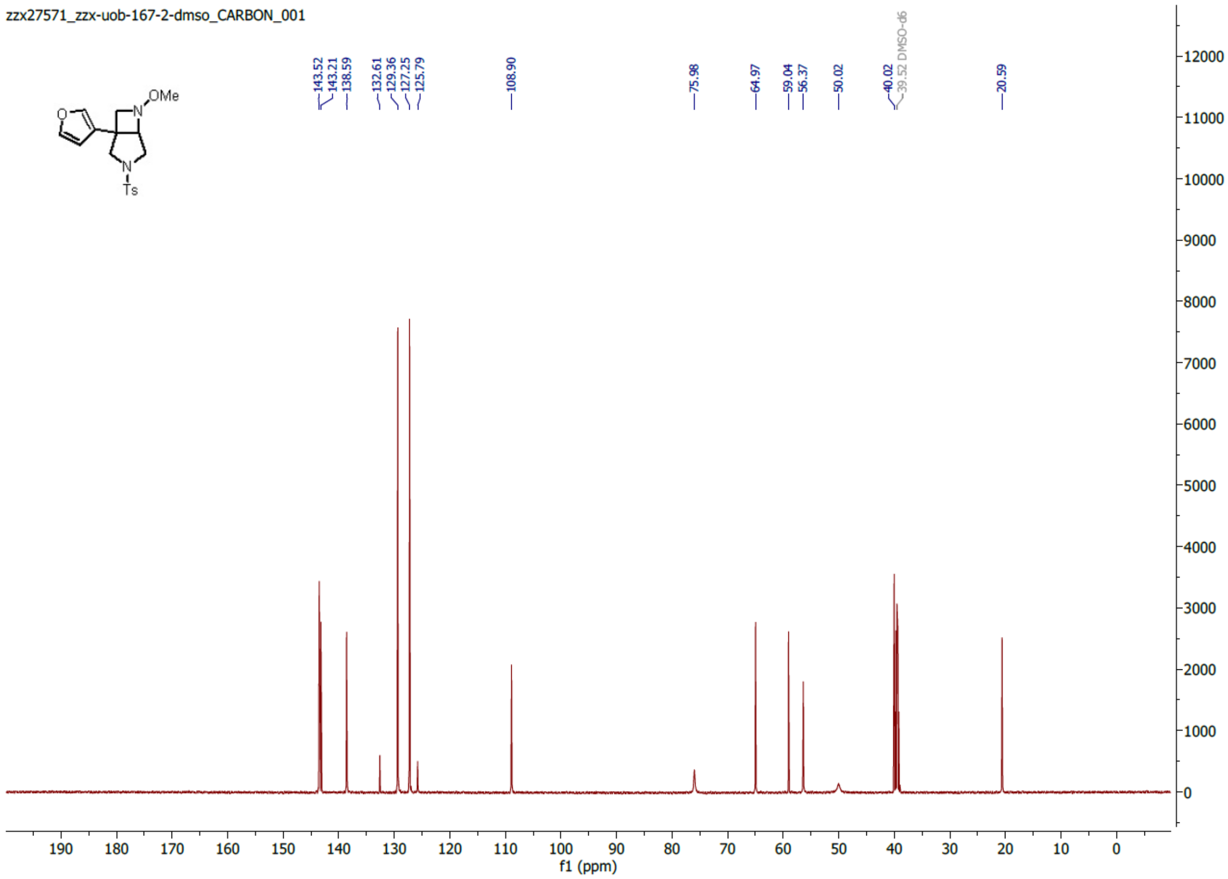

Compound 15

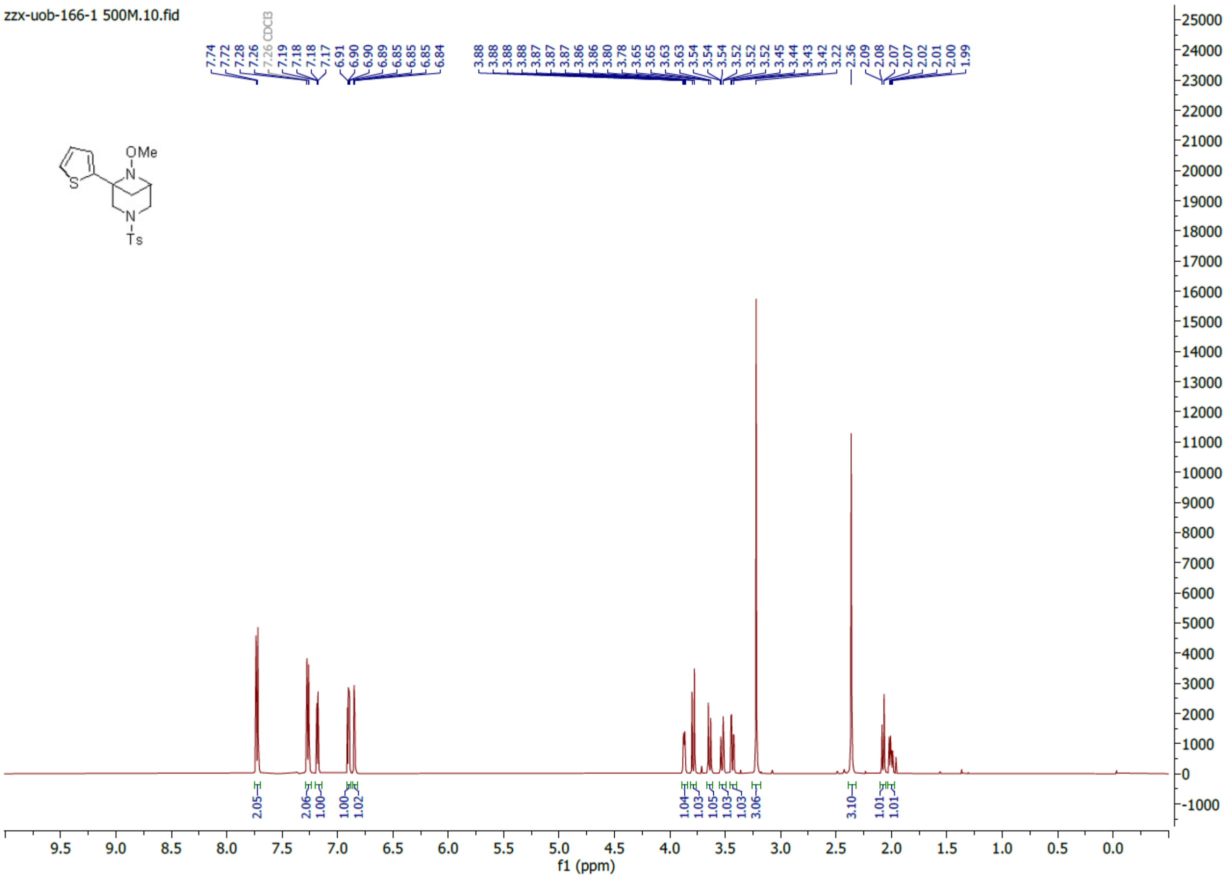
zzx-uob-166-1 500M.10.fid
OMe
N
S
N
Ts
f1 (ppm)

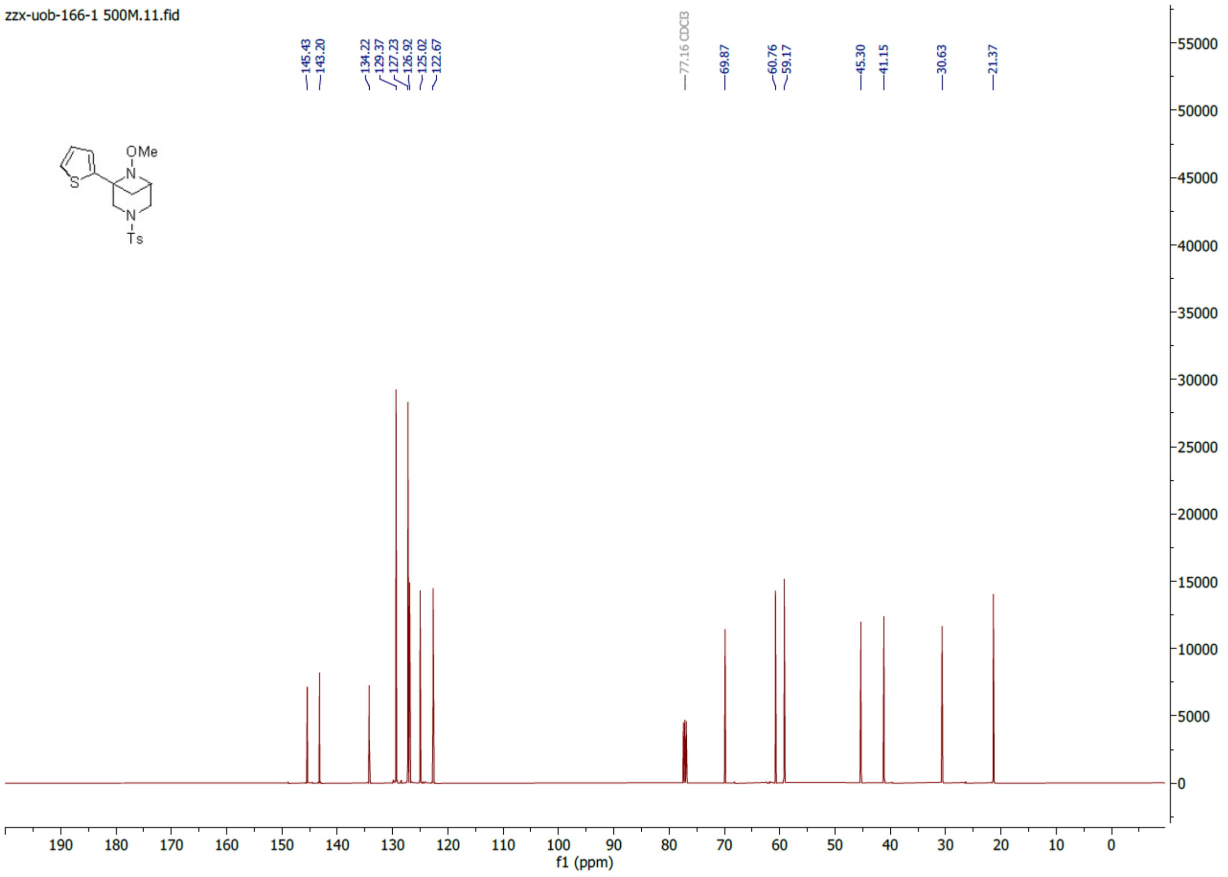
zzx-uob-166-1 500M.11.fid
145.43
143.20
134.22
129.37
127.23
126.92
125.02
122.67
77.16 CDCl3
69.87
60.76
59.17
45.30
41.15
30.63
21.37
OMe
N
S
N
Ts
f1 (ppm)

Compound 16

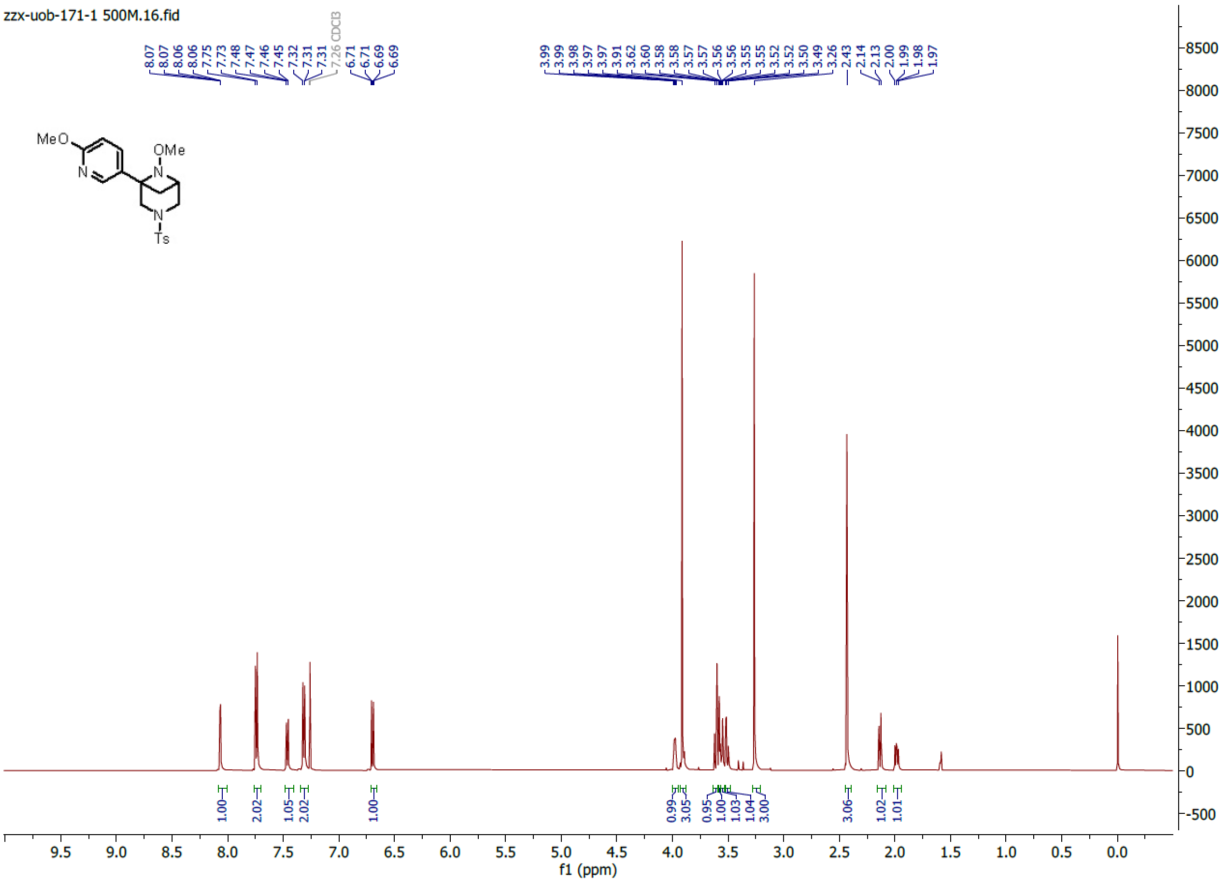
zzx-uob-171-1 500M.16.fid
8.07 8.07 8.06 8.06 7.75 7.73 7.48 7.47 7.46 7.45 7.32 7.31 7.31 7.26 CDCl3 6.71 6.71 6.69 6.69
3.99 3.99 3.98 3.97 3.97 3.91 3.62 3.60 3.58 3.58 3.57 3.57 3.56 3.56 3.55 3.55 3.52 3.52 3.50 3.49 3.26 2.43 2.14 2.13 2.00 1.99 1.98 1.97
MeO
N
OMe
N
N
Ts
1.00 2.02 1.05 2.02 1.00 0.99 3.05 0.95 1.00 1.03 1.04 3.00 3.06 1.02 1.01
f1 (ppm)

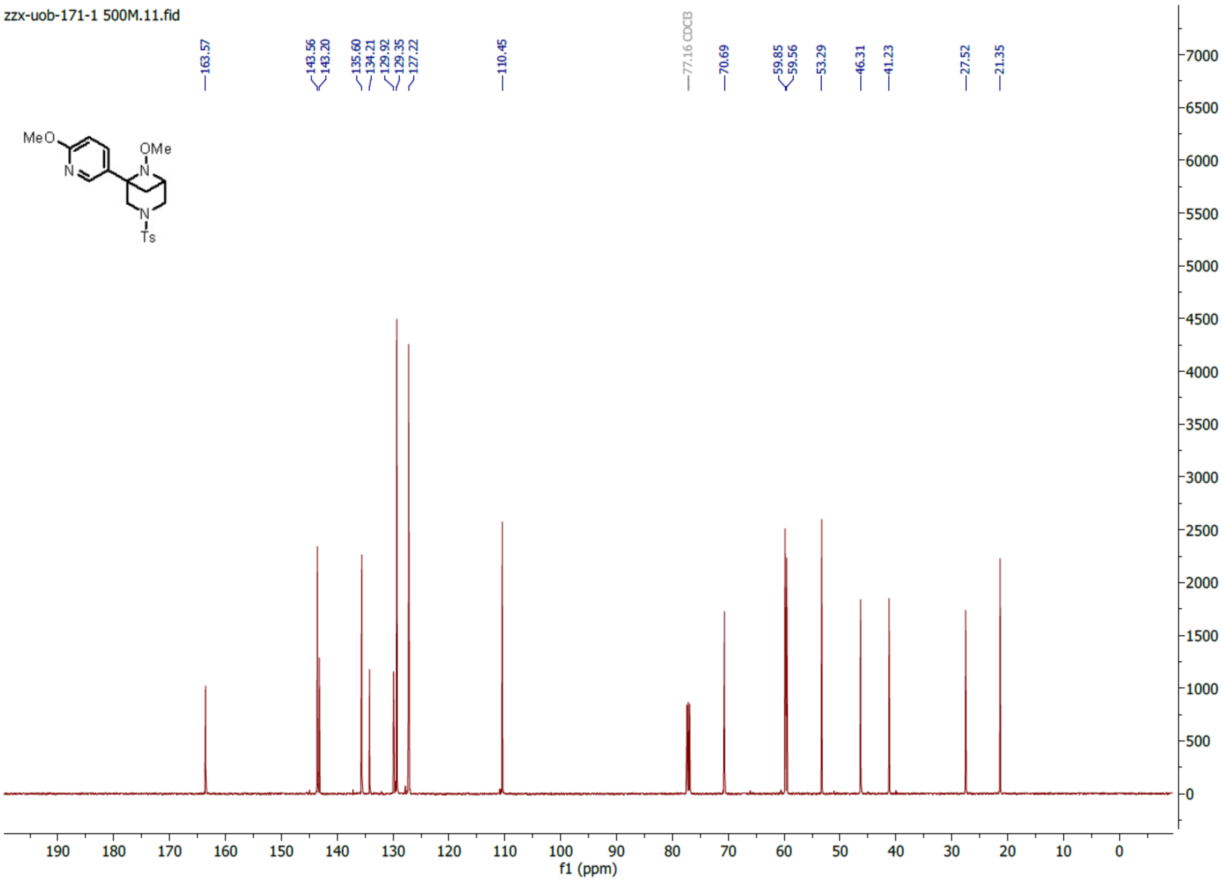
zzx-uob-171-1 500M.11.fid
163.57 143.56 143.20 135.60 134.21 129.92 129.35 127.22 110.45 77.16 CDCl3 70.69 59.85 59.56 53.29 46.31 41.23 27.52 21.35
MeO
N
OMe
N
N
Ts
f1 (ppm)

Compound 16a

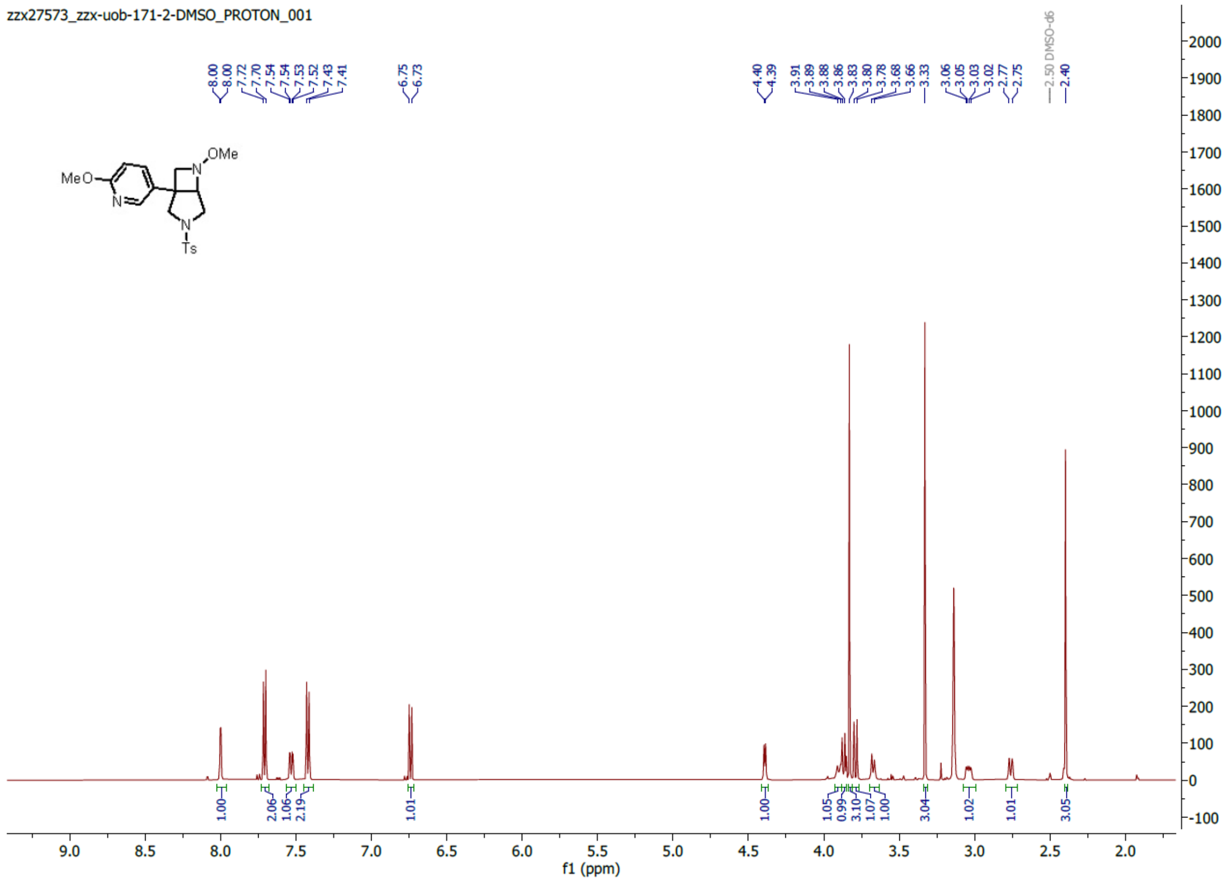

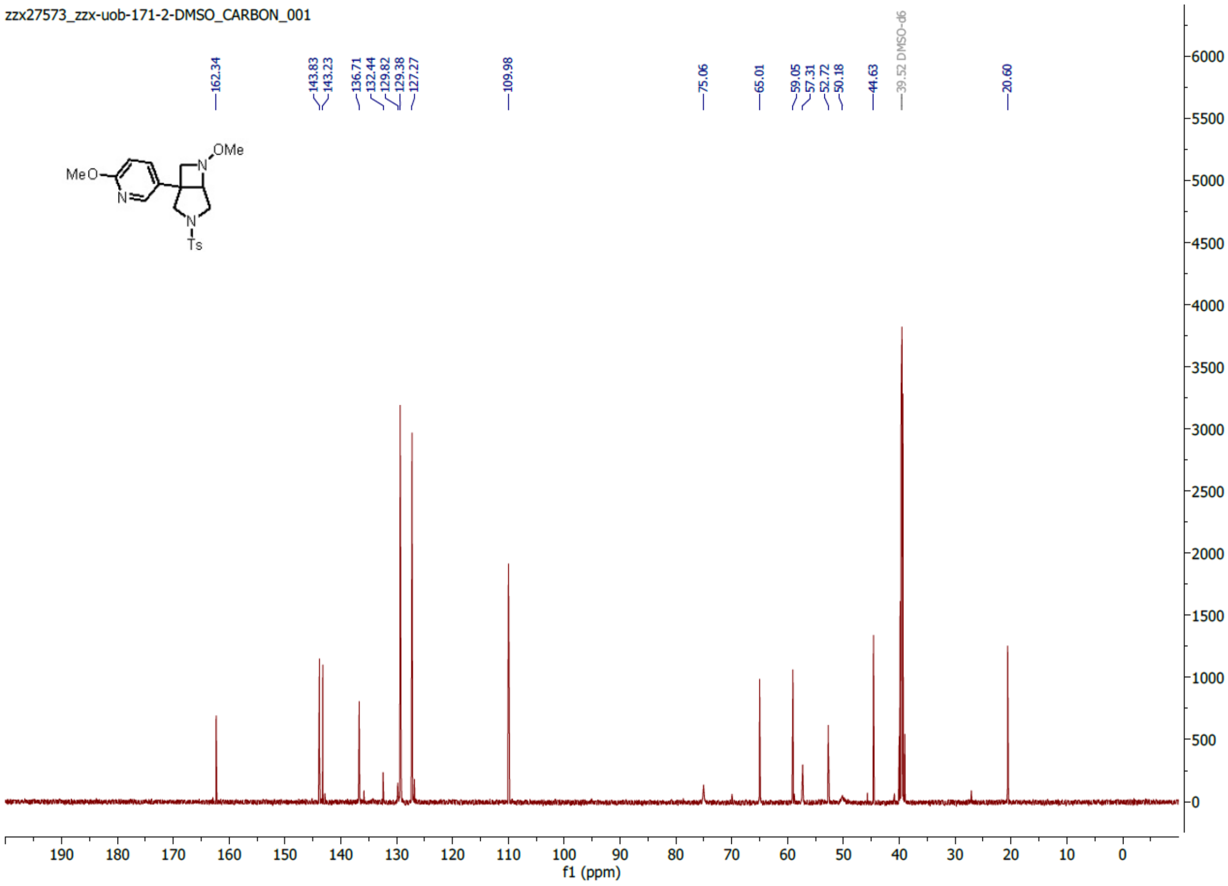

Compound 17

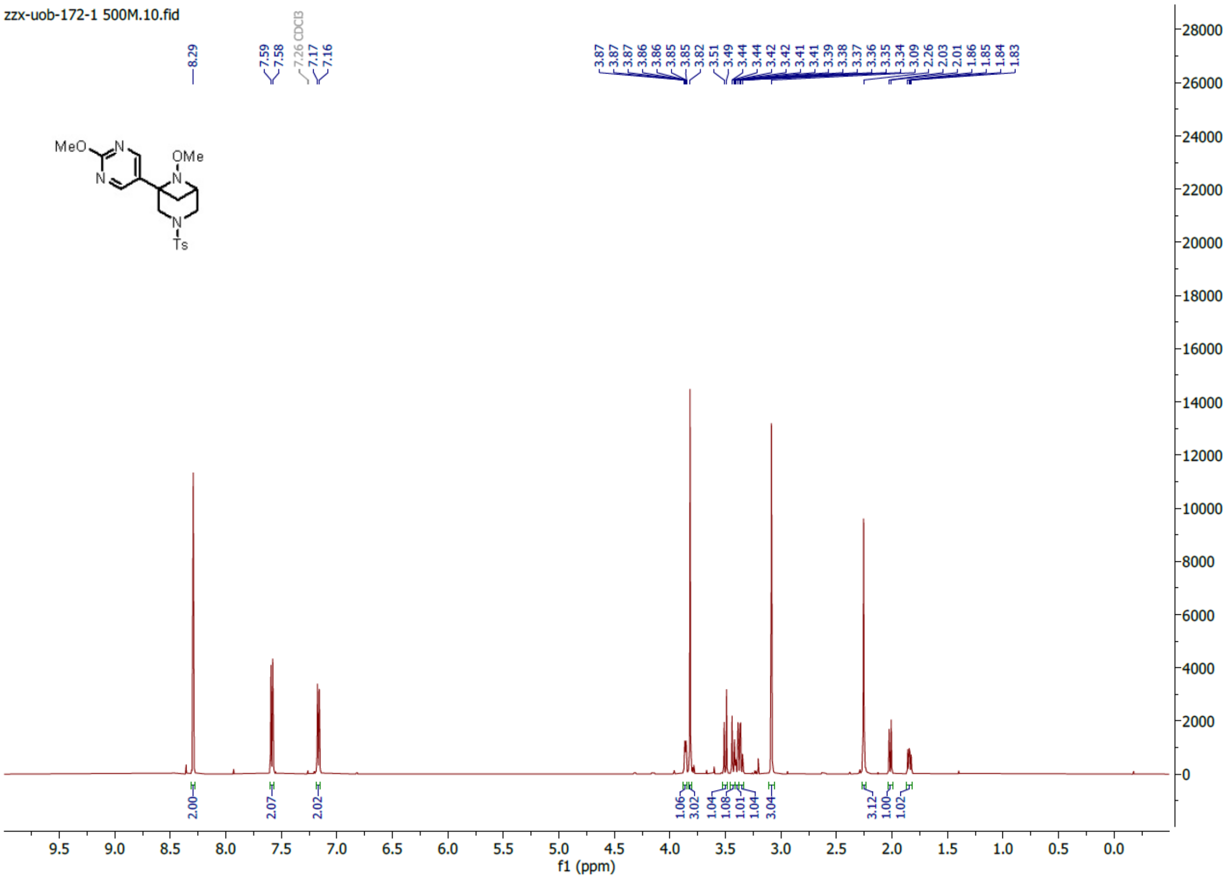

zzx-uob-172-1 500M.10.fid
MeO
N
N
OMe
N
N
Ts
f1 (ppm)

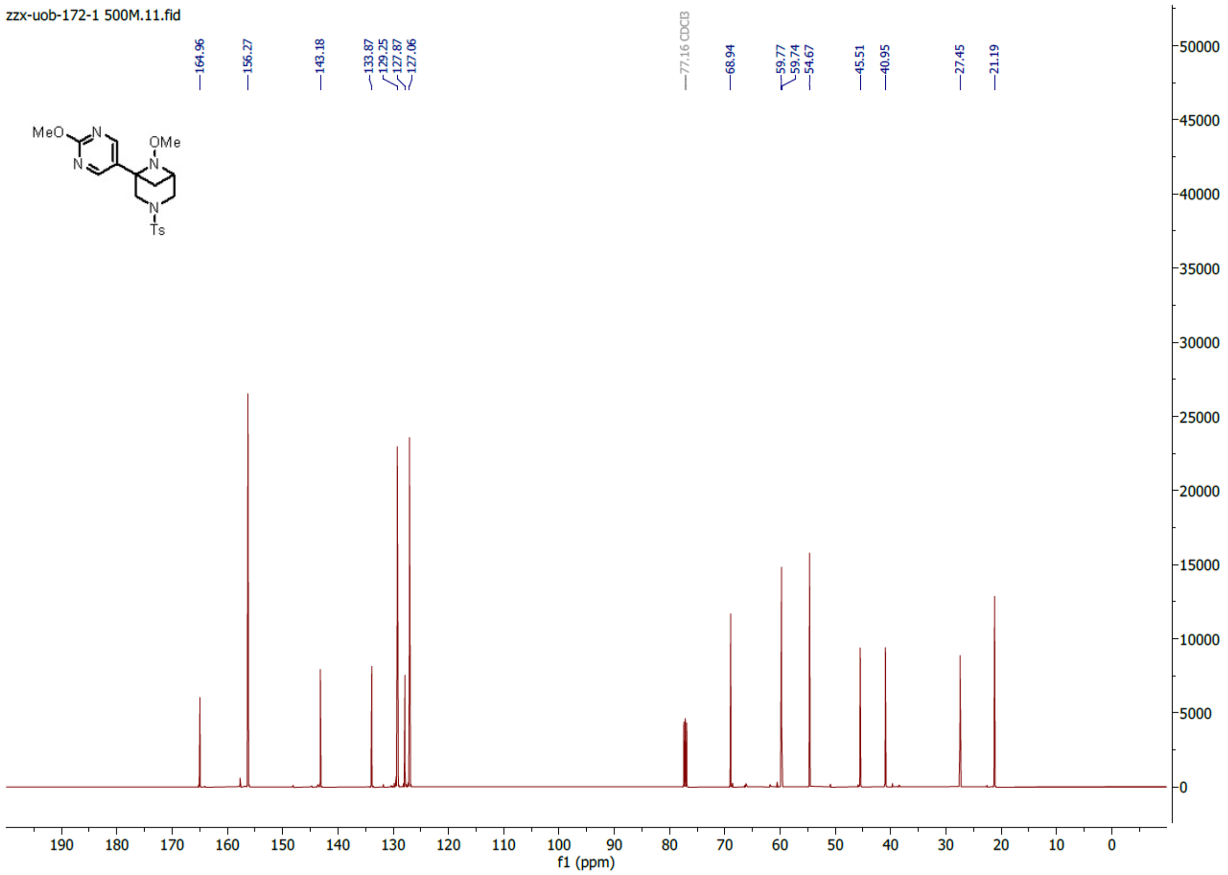

zzx-uob-172-1 500M.11.fid
MeO
N
N
OMe
N
N
Ts
f1 (ppm)

Compound 17a

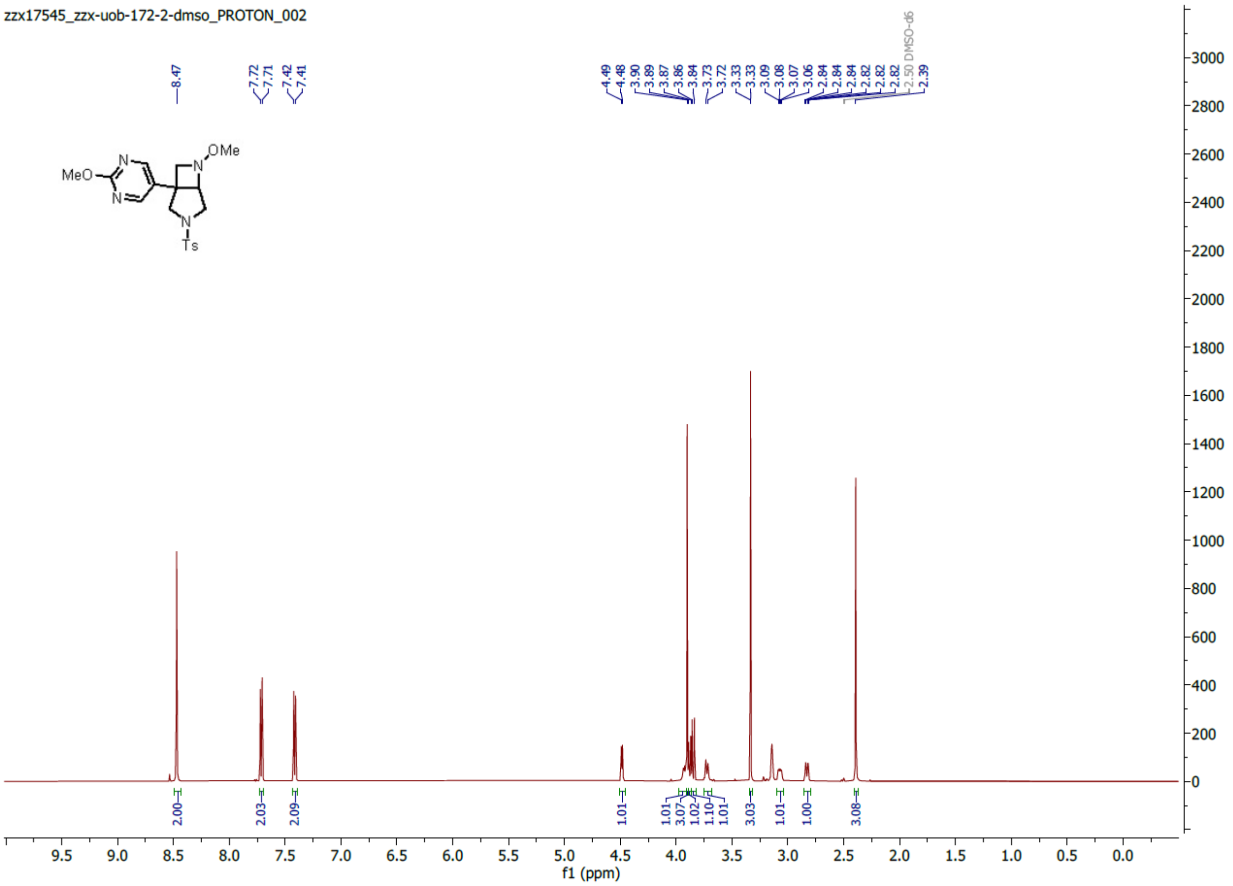

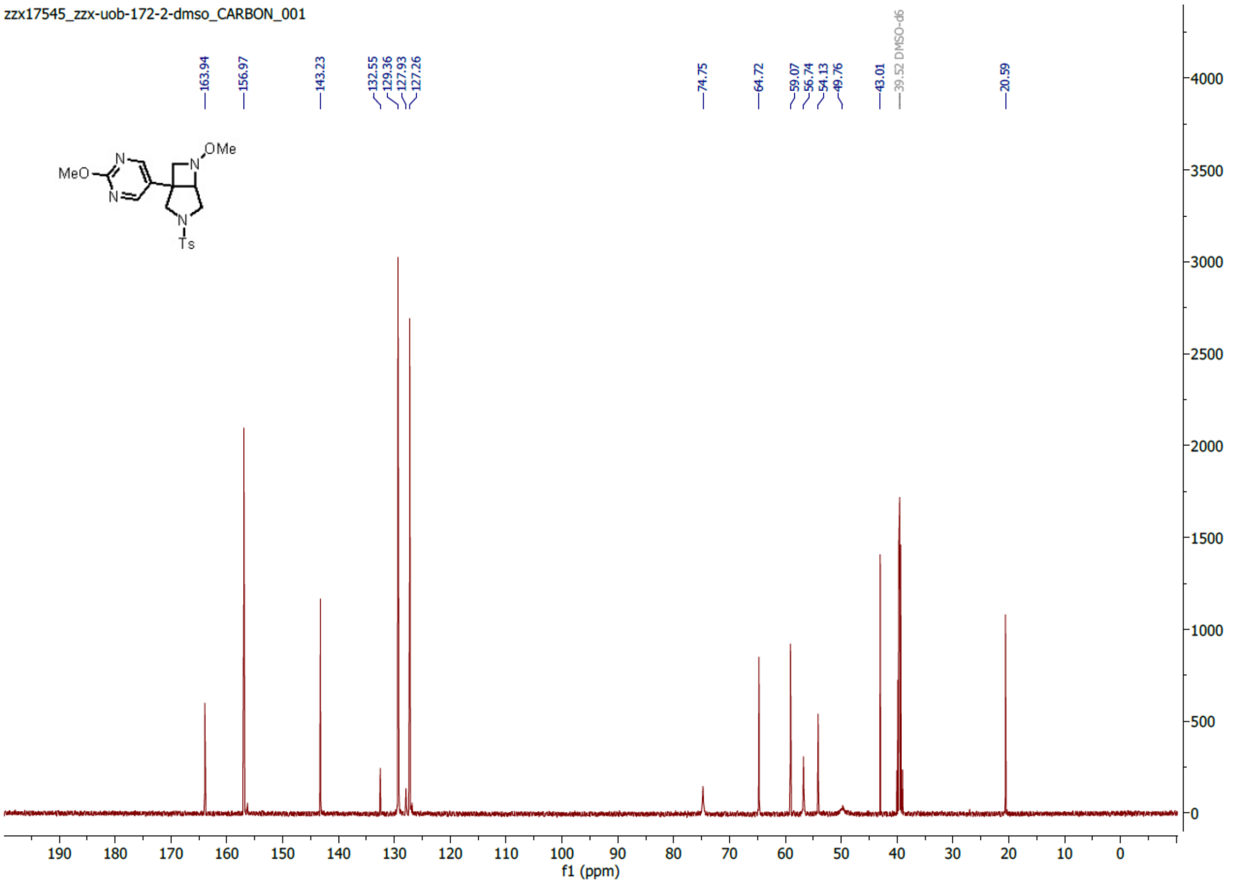

Compound 18

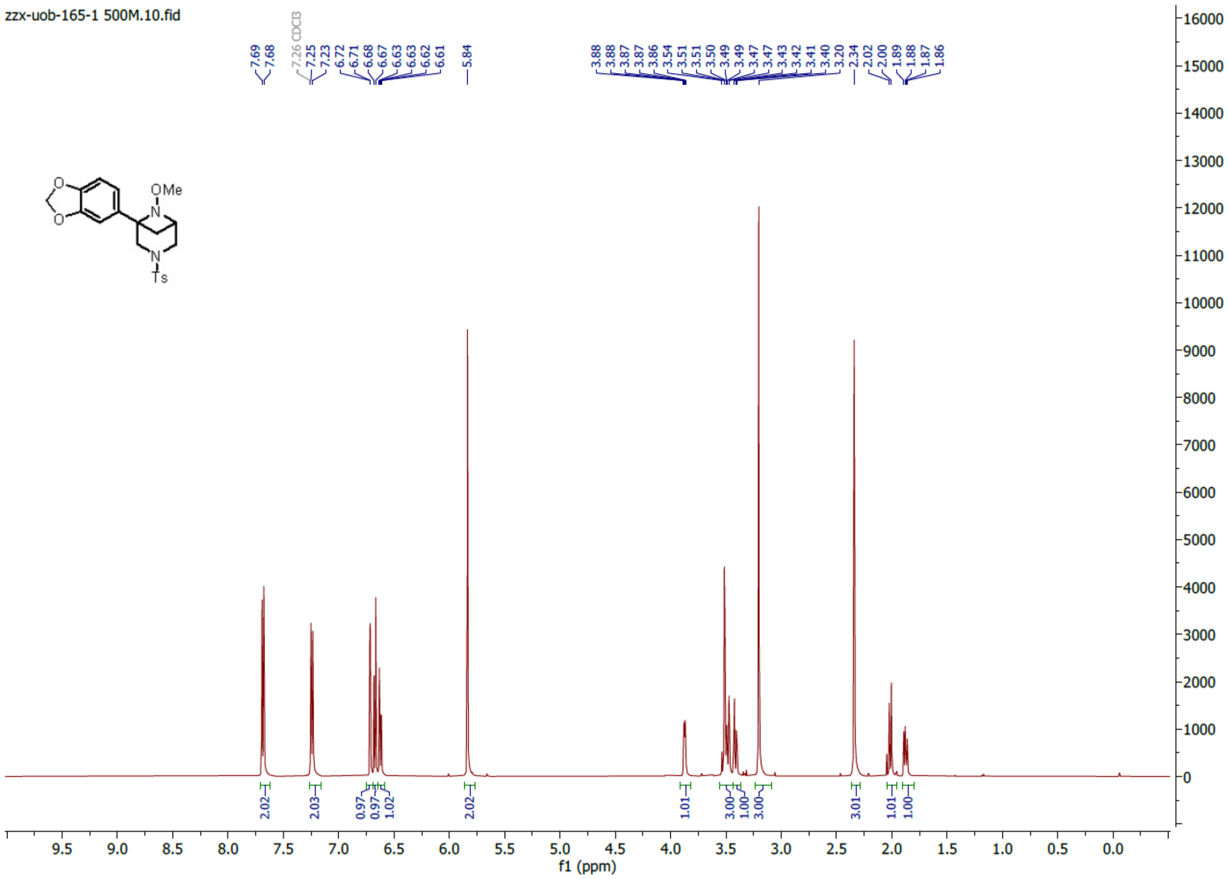

zzx-uob-165-1 500M.10.fid
7.69 7.68 7.26 CDCl3 7.25 7.23 6.72 6.71 6.68 6.67 6.63 6.63 6.62 6.61 5.84
3.88 3.88 3.87 3.87 3.86 3.54 3.51 3.51 3.50 3.49 3.49 3.47 3.47 3.43 3.42 3.41 3.40 3.20 2.34 2.02 2.00 1.89 1.88 1.87 1.86
OMe
N
O
O
N
Ts
2.02 2.03 0.97 0.97 1.02 2.02 1.01 3.00 1.00 3.00 3.01 1.01 1.00
f1 (ppm)

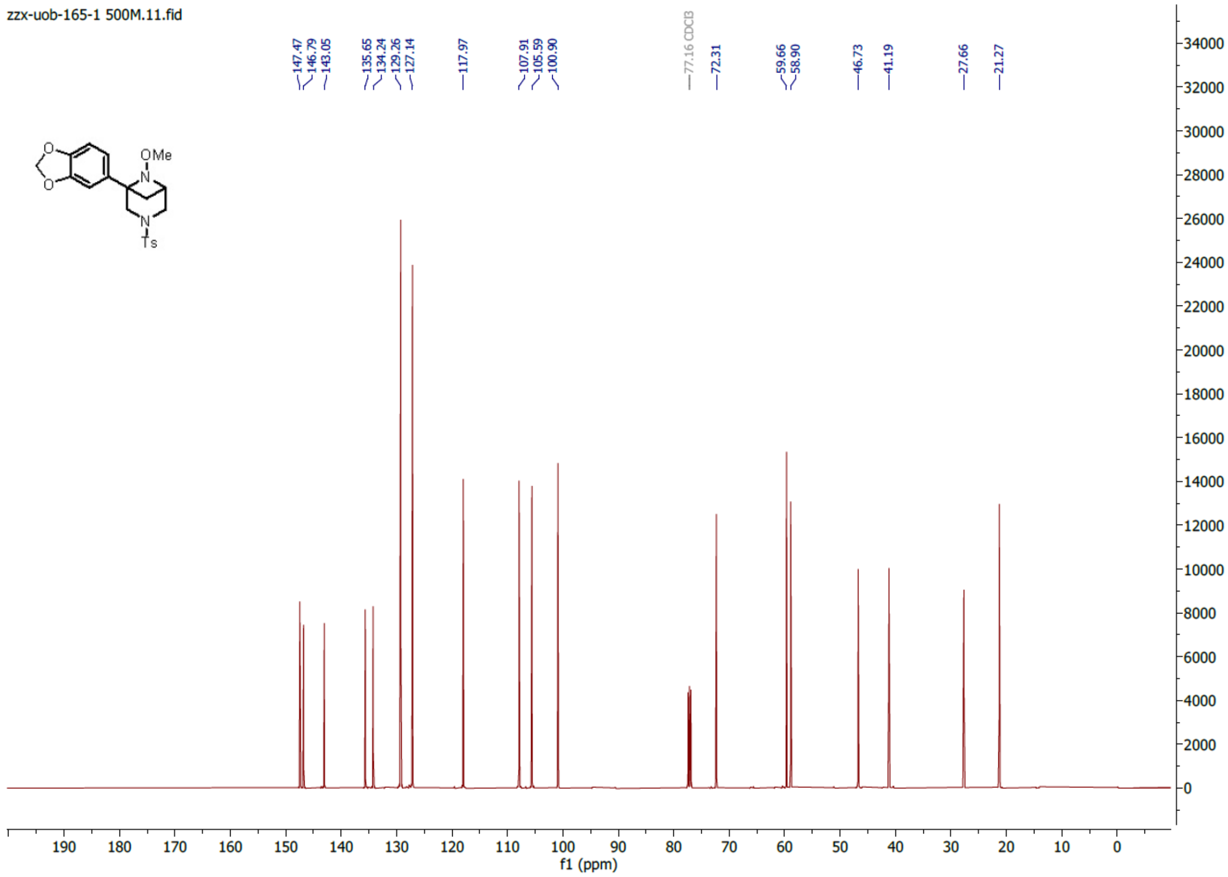

zzx-uob-165-1 500M.11.fid
147.47 146.79 143.05 135.65 134.24 129.26 127.14 117.97 107.91 105.59 100.90 77.16 CDCl3 72.31 59.66 58.90 46.73 41.19 27.66 21.27
OMe
N
O
O
N
Ts
f1 (ppm)

Compound 18a

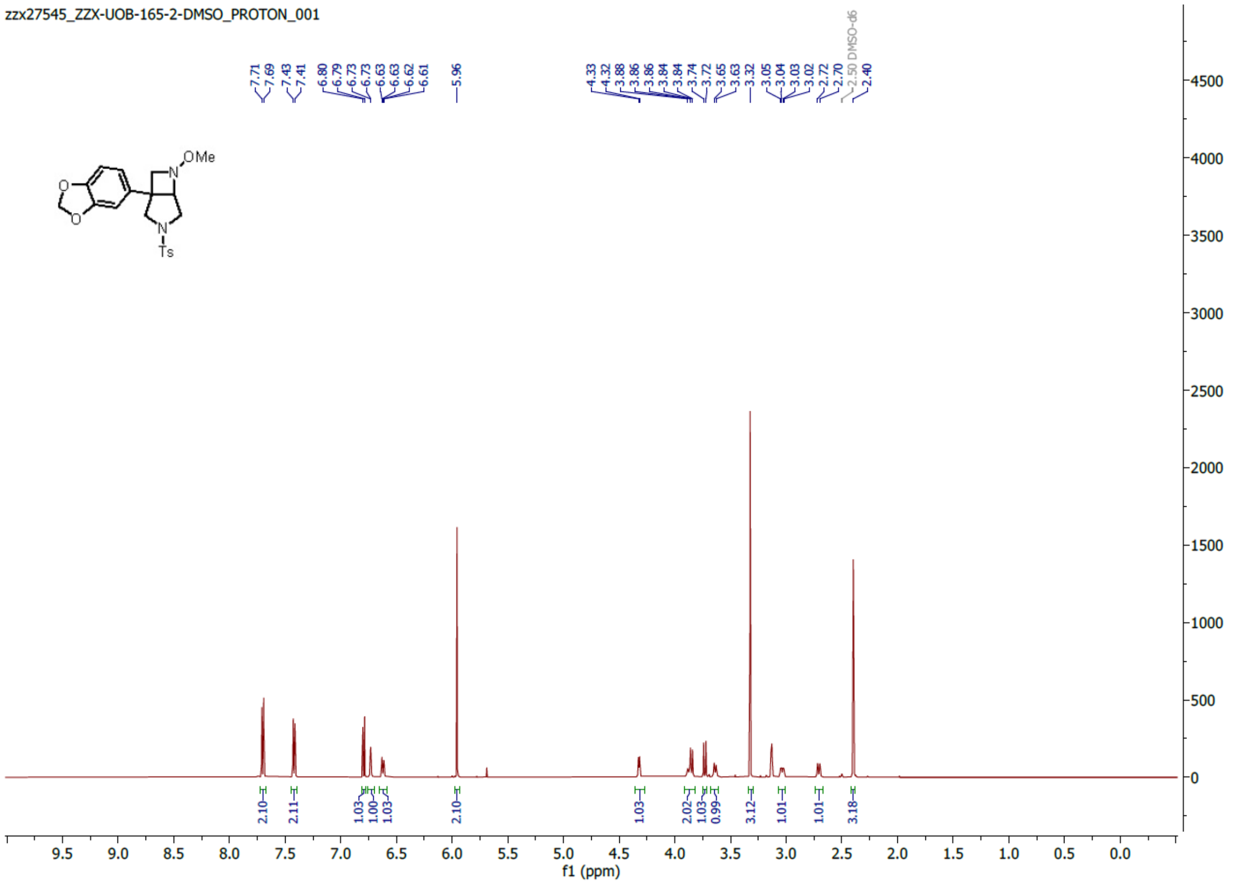

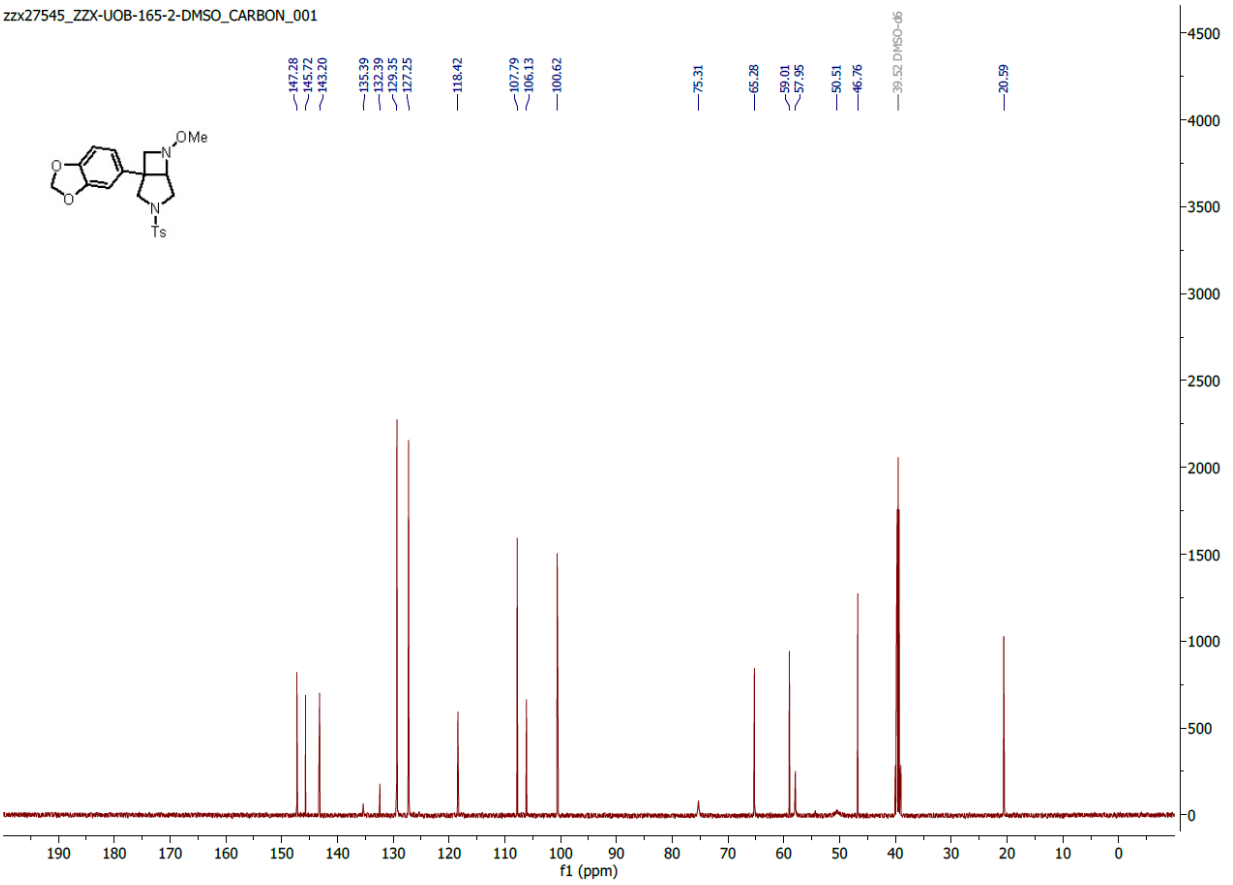

Compound 19

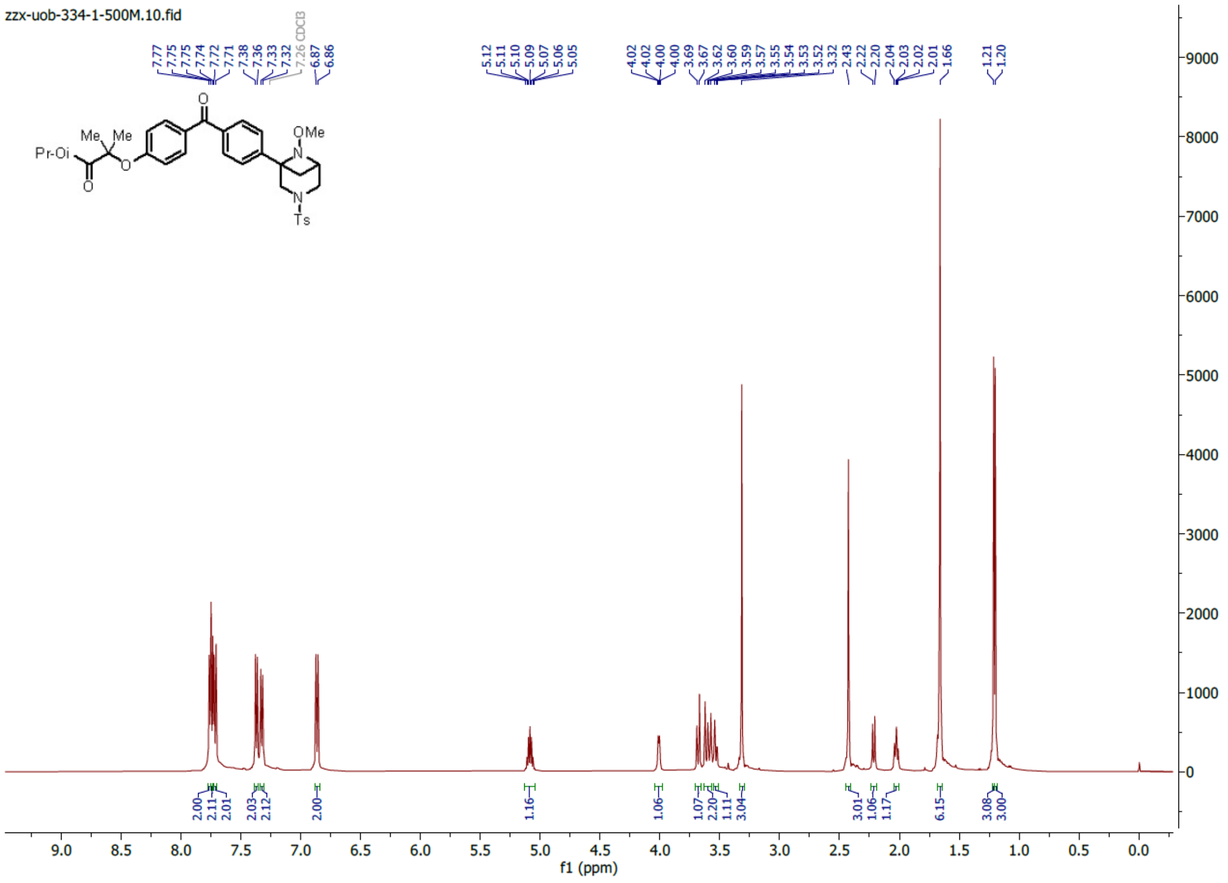
zzx-uob-334-1-500M.10.fid
7.77 7.75 7.75 7.74 7.72 7.71 7.38 7.36 7.33 7.32 7.26 CDCl3 6.87 6.86
5.12 5.11 5.10 5.09 5.07 5.06 5.05
4.02 4.02 4.00 4.00 3.69 3.67 3.62 3.60 3.59 3.57 3.55 3.54 3.53 3.52 3.32 2.43 2.22 2.20 2.04 2.03 2.02 2.01 1.66
1.21 1.20
Pr-Oi
Me Me
O
OMe
N
Ts
2.00 2.11 2.01 2.03 2.12 2.00 1.16 1.06 1.07 2.20 1.11 3.04 3.01 1.06 1.17 6.15 3.08 3.00
f1 (ppm)

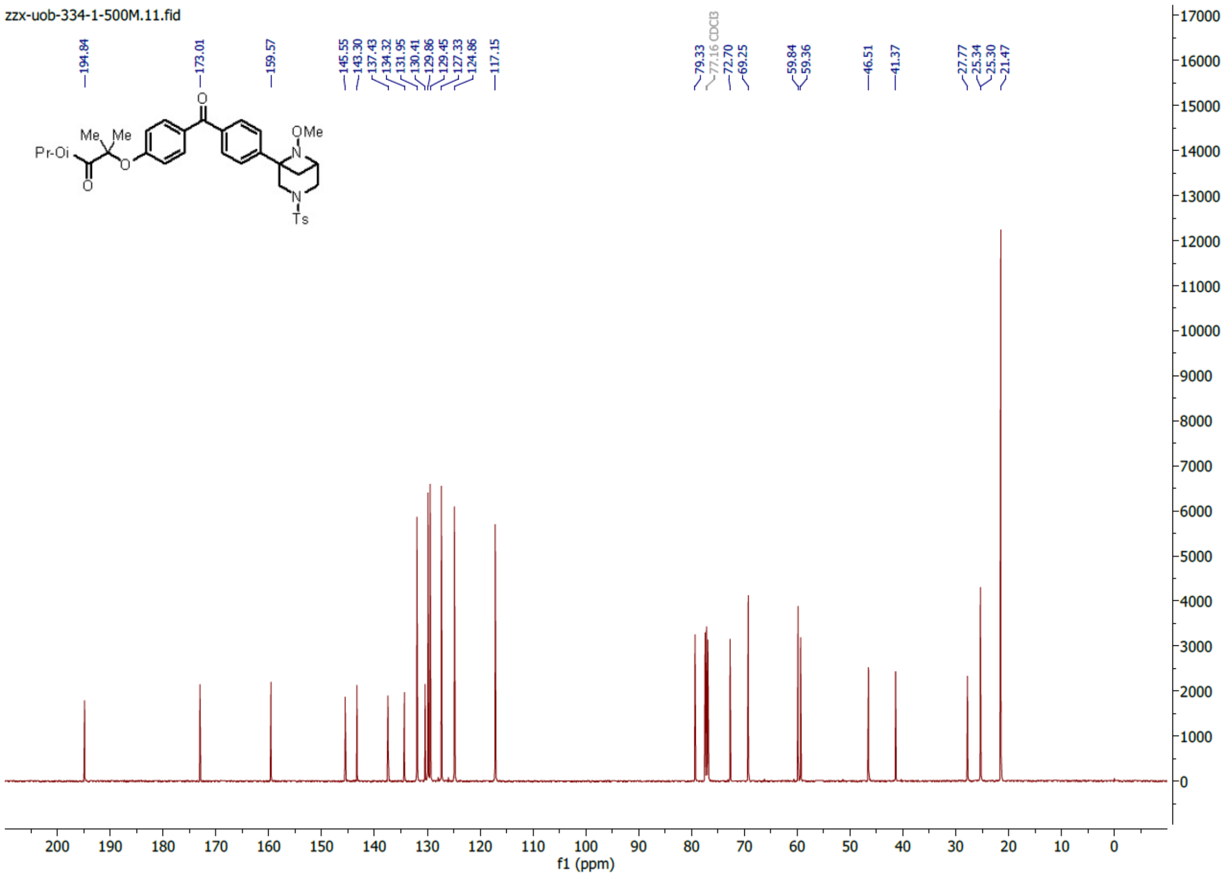
zzx-uob-334-1-500M.11.fid
194.84
173.01
159.57
145.55 143.30 137.43 134.32 131.95 130.41 129.86 129.45 127.33 124.86
117.15
79.33 77.16 CDCl3 72.70 69.25
59.84 59.36
46.51
41.37
27.77 25.34 25.30 21.47
Pr-Oi
Me Me
O
OMe
N
Ts
f1 (ppm)

Compound 19a

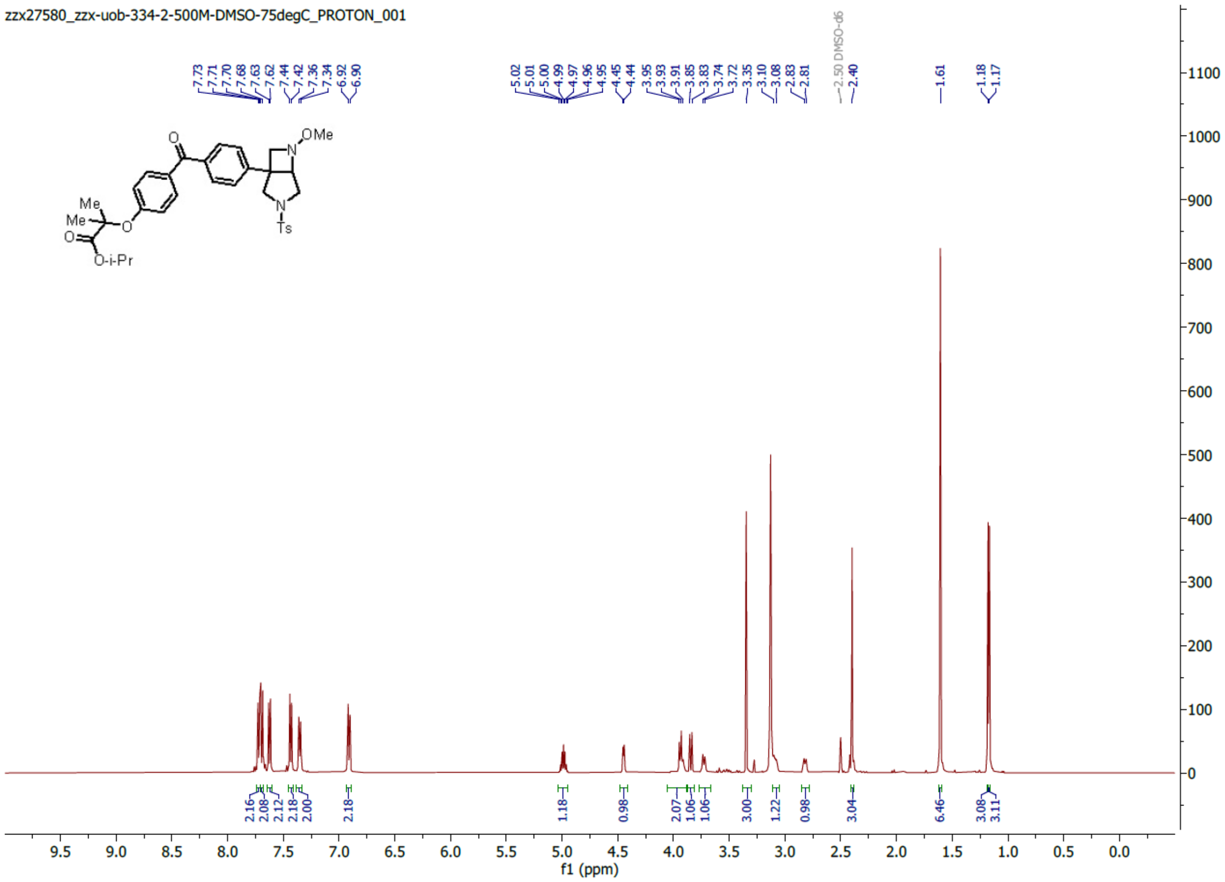

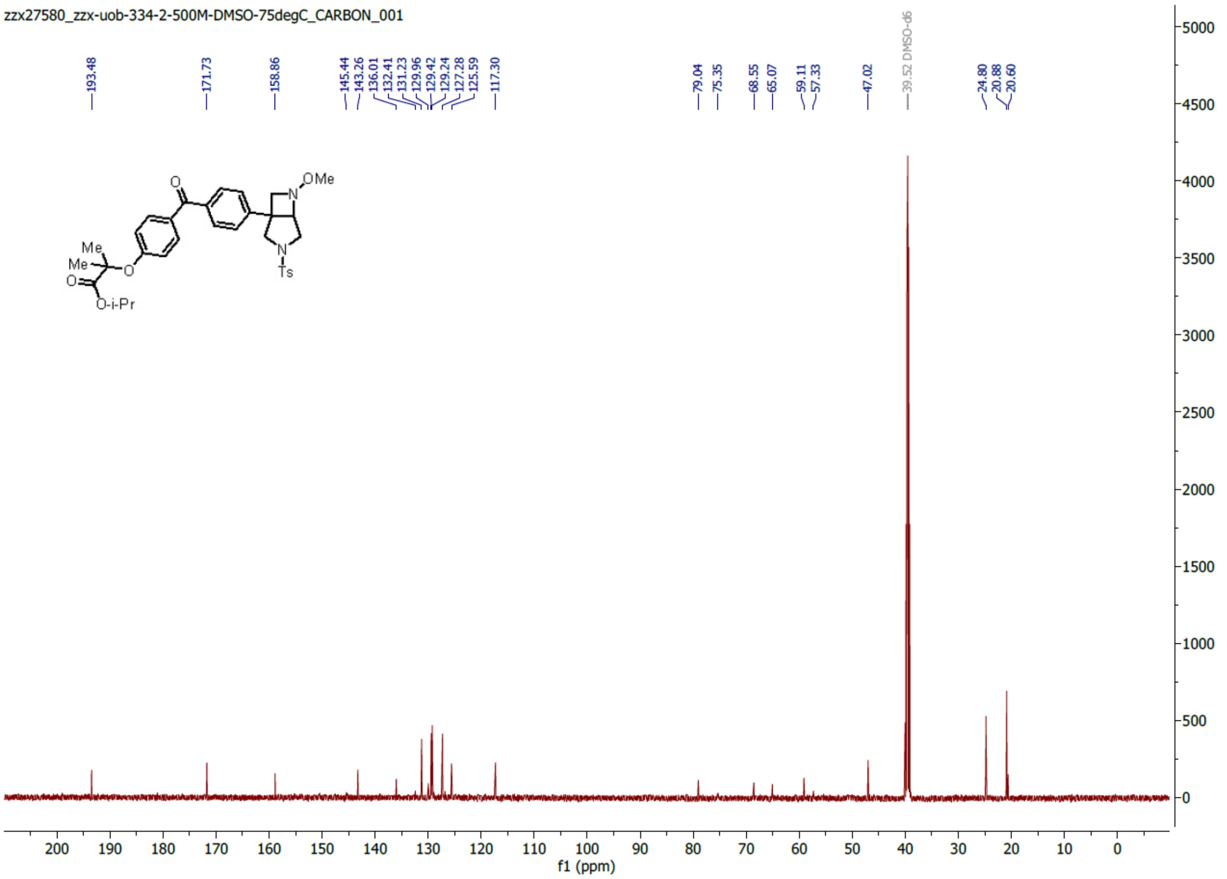

Compound 20

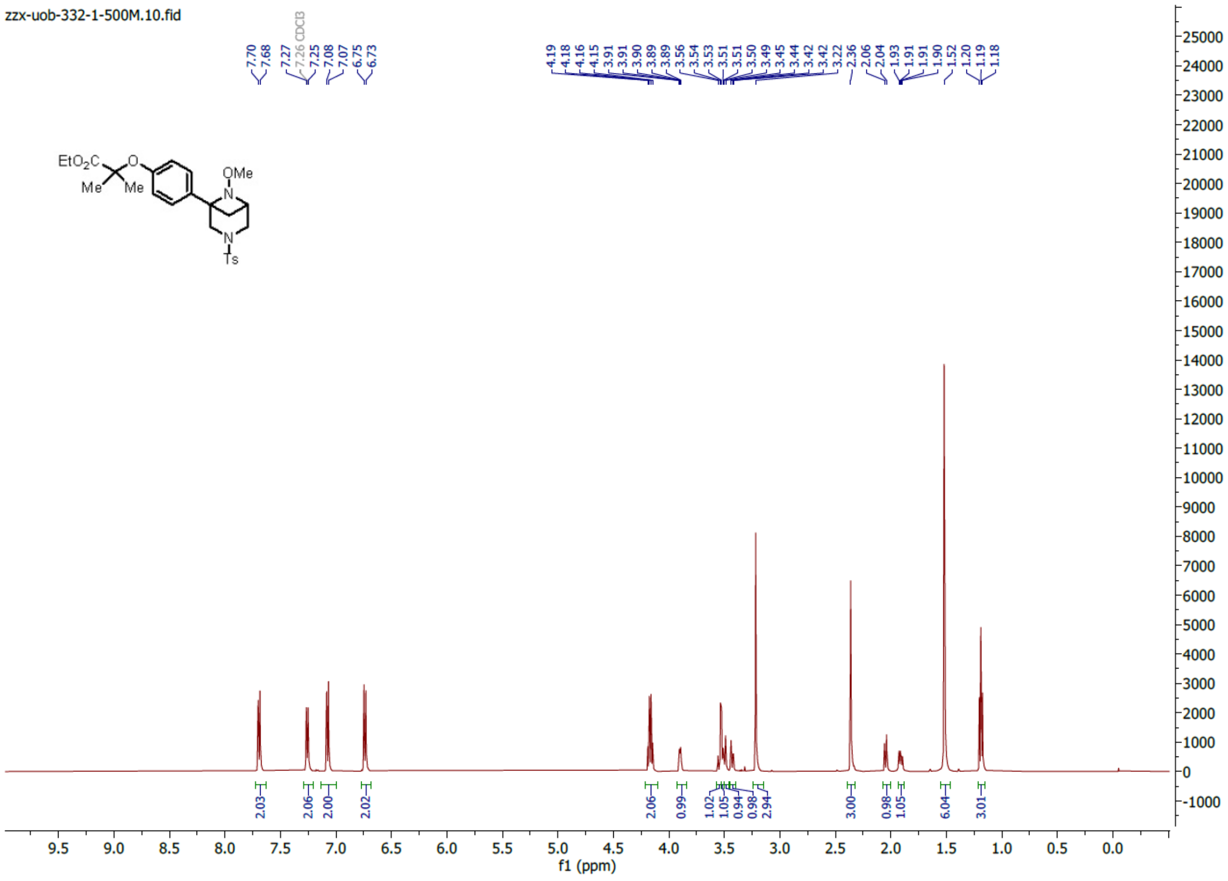

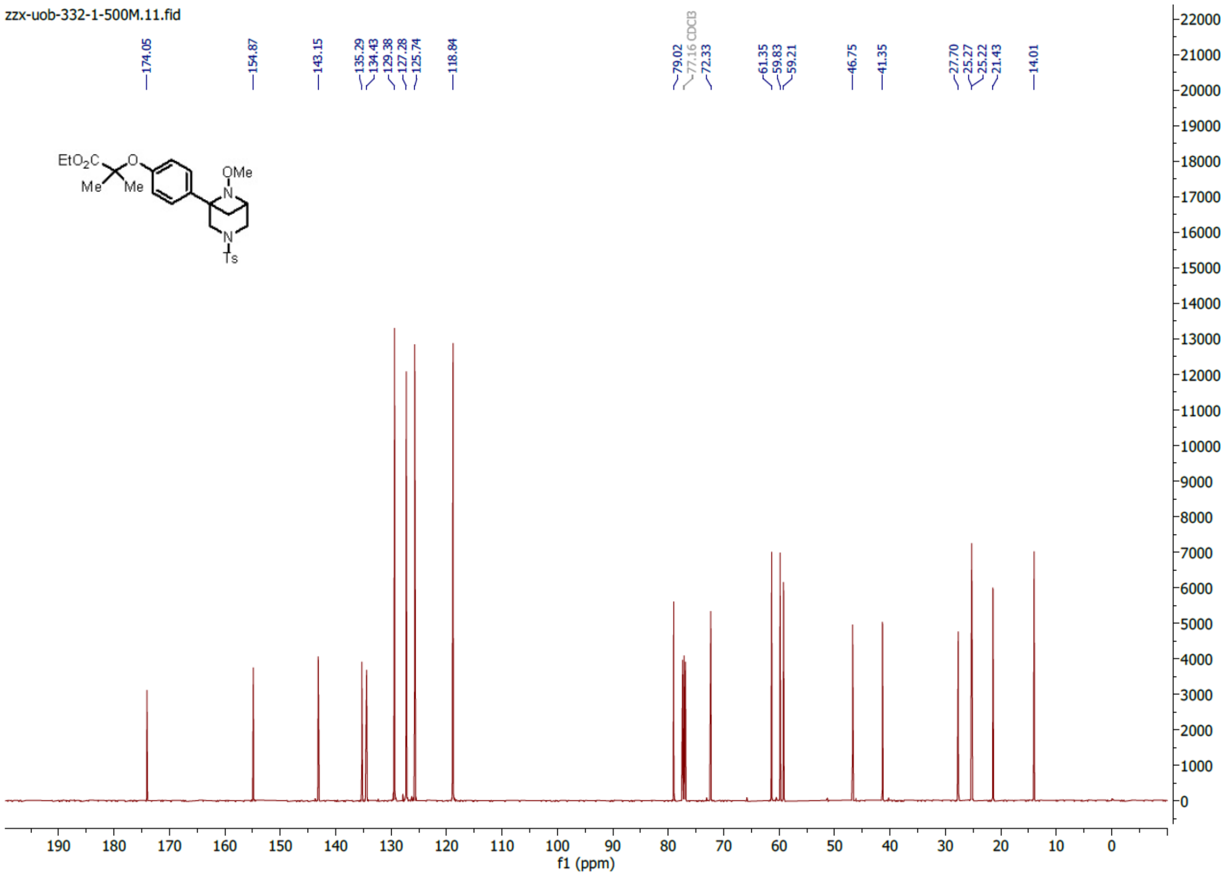

Compound 20a

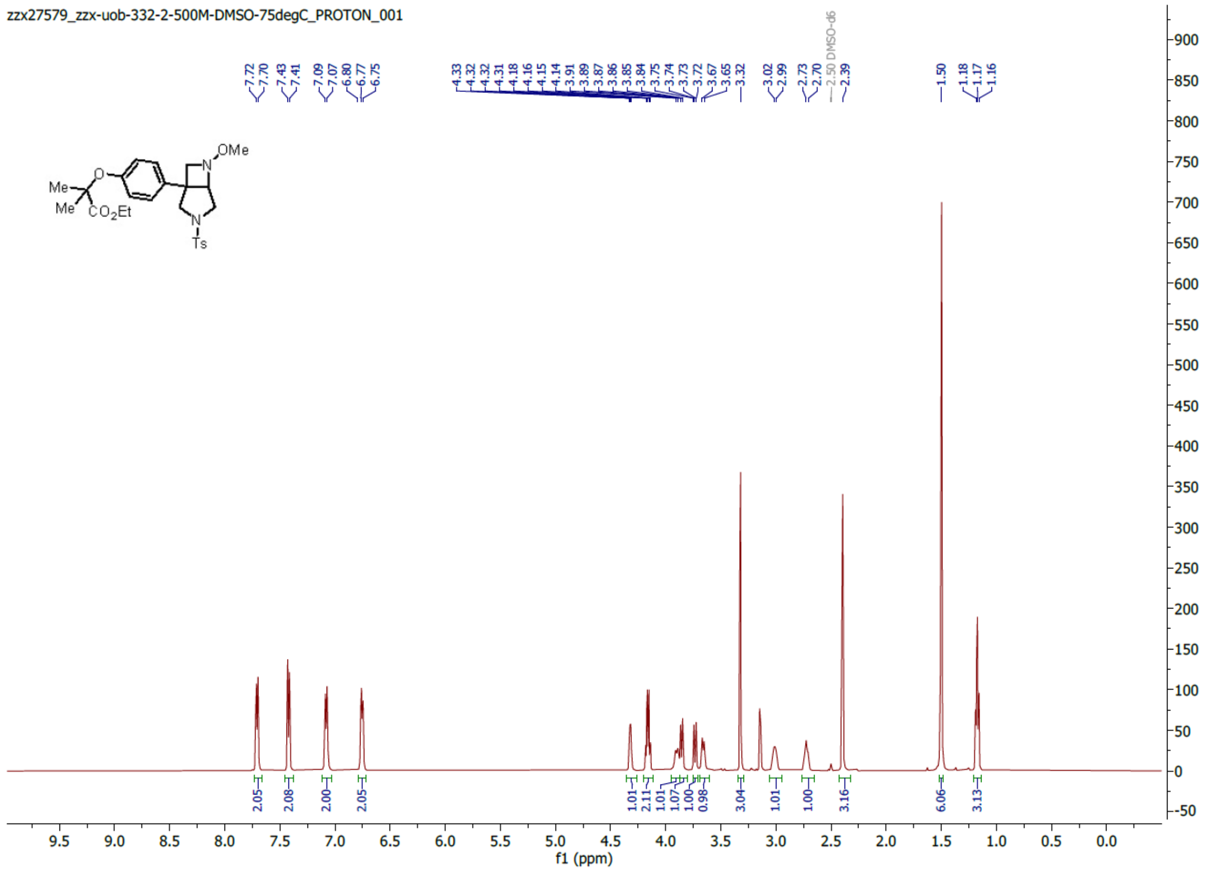

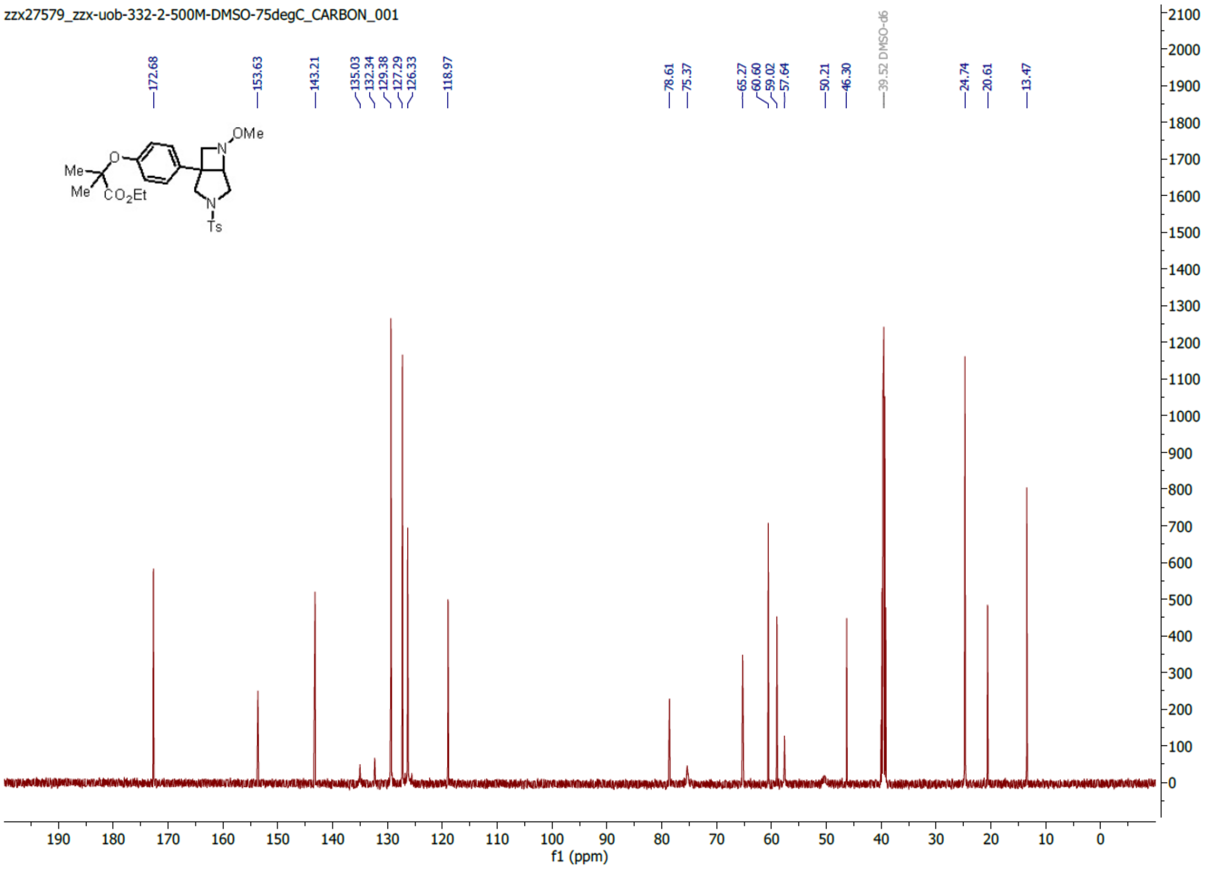

Compound 21

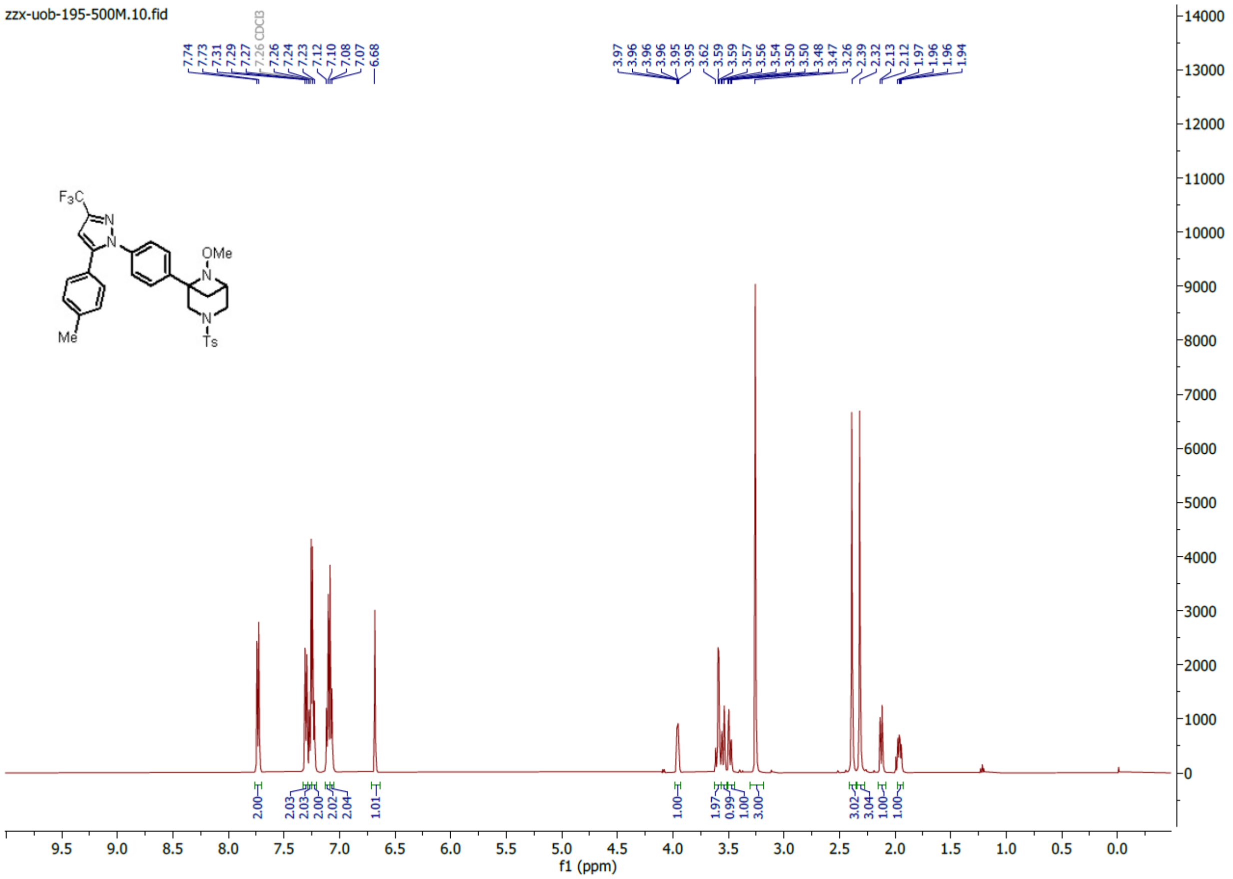
zzx-uob-195-500M.10.fid
7.74 7.73 7.31 7.29 7.27 7.26 CDCl3 7.26 7.24 7.23 7.12 7.10 7.08 7.07 6.68
3.97 3.96 3.96 3.96 3.95 3.95 3.62 3.59 3.59 3.57 3.56 3.54 3.50 3.50 3.48 3.47 3.26 2.39 2.32 2.13 2.12 1.97 1.96 1.96 1.94
F3C
N
N
OMe
N
N
Ts
Me
2.00
2.03
2.03
2.00
2.02
2.04
1.01
1.00
1.97
0.99
1.00
3.00
3.02
3.04
1.00
1.00
f1 (ppm)

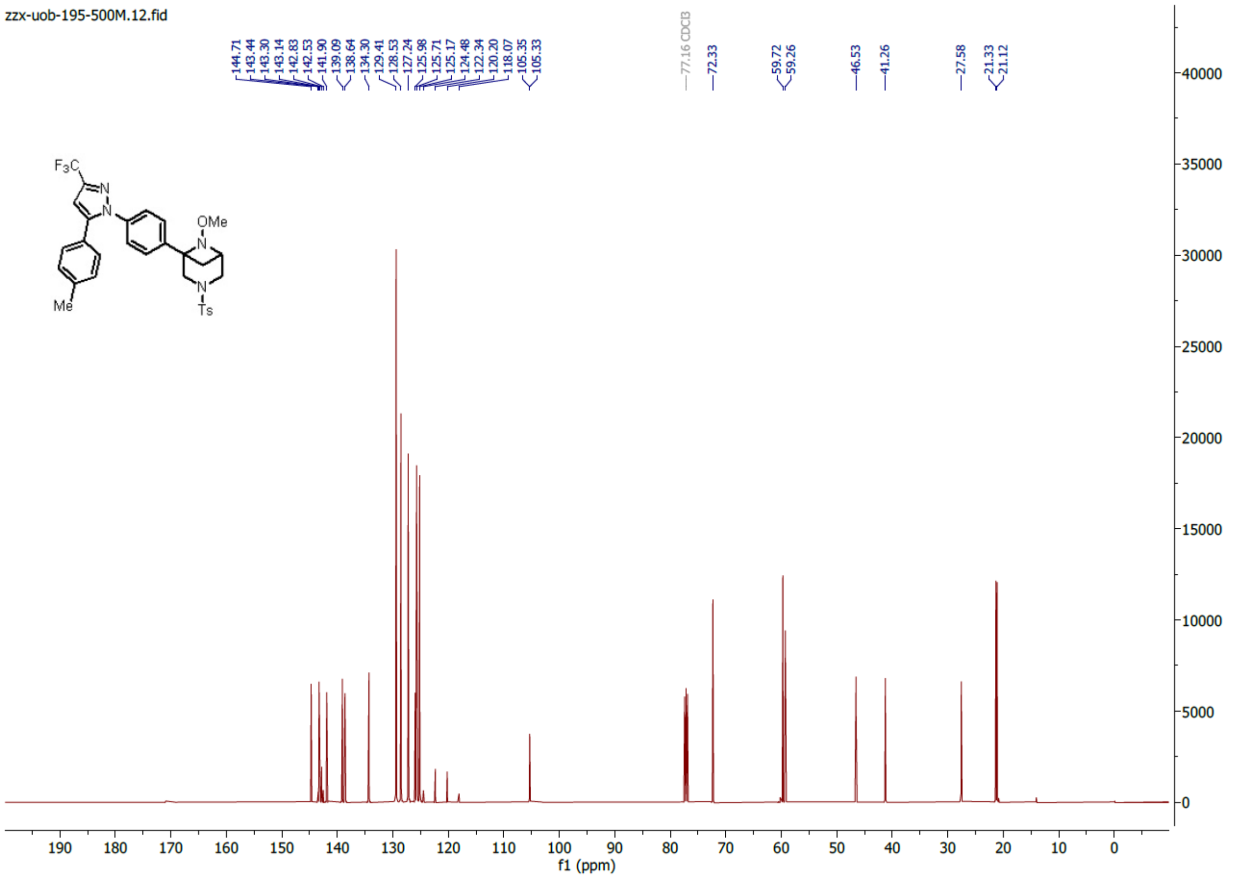
zzx-uob-195-500M.12.fid
144.71 143.44 143.30 143.14 142.83 142.53 141.90 139.09 138.64 134.30 129.41 128.53 127.24 125.98 125.71 125.17 124.48 122.34 120.20 118.07 105.35 105.33
77.16 CDCl3
72.33
59.72 59.26
46.53
41.26
27.58
21.33 21.12
F3C
N
N
OMe
N
N
Ts
Me
f1 (ppm)

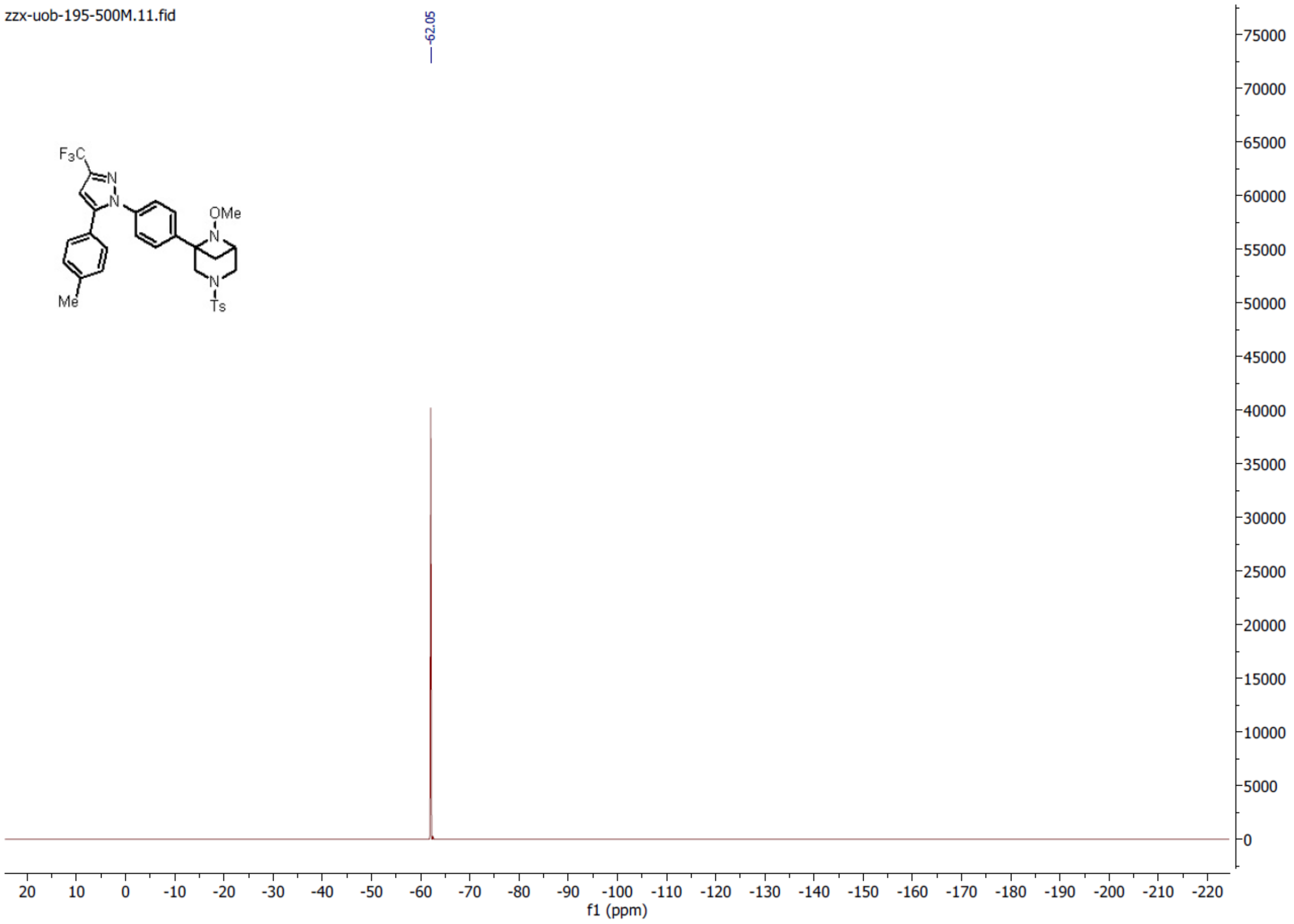

zzx-uob-195-500M.11.fid
-62.05
F3C
N
N
OMe
N
N
Ts
Me
75000
70000
65000
60000
55000
50000
45000
40000
35000
30000
25000
20000
15000
10000
5000
0
20 10 0 -10 -20 -30 -40 -50 -60 -70 -80 -90 -100 -110 -120 -130 -140 -150 -160 -170 -180 -190 -200 -210 -220
f1 (ppm)

Compound 22

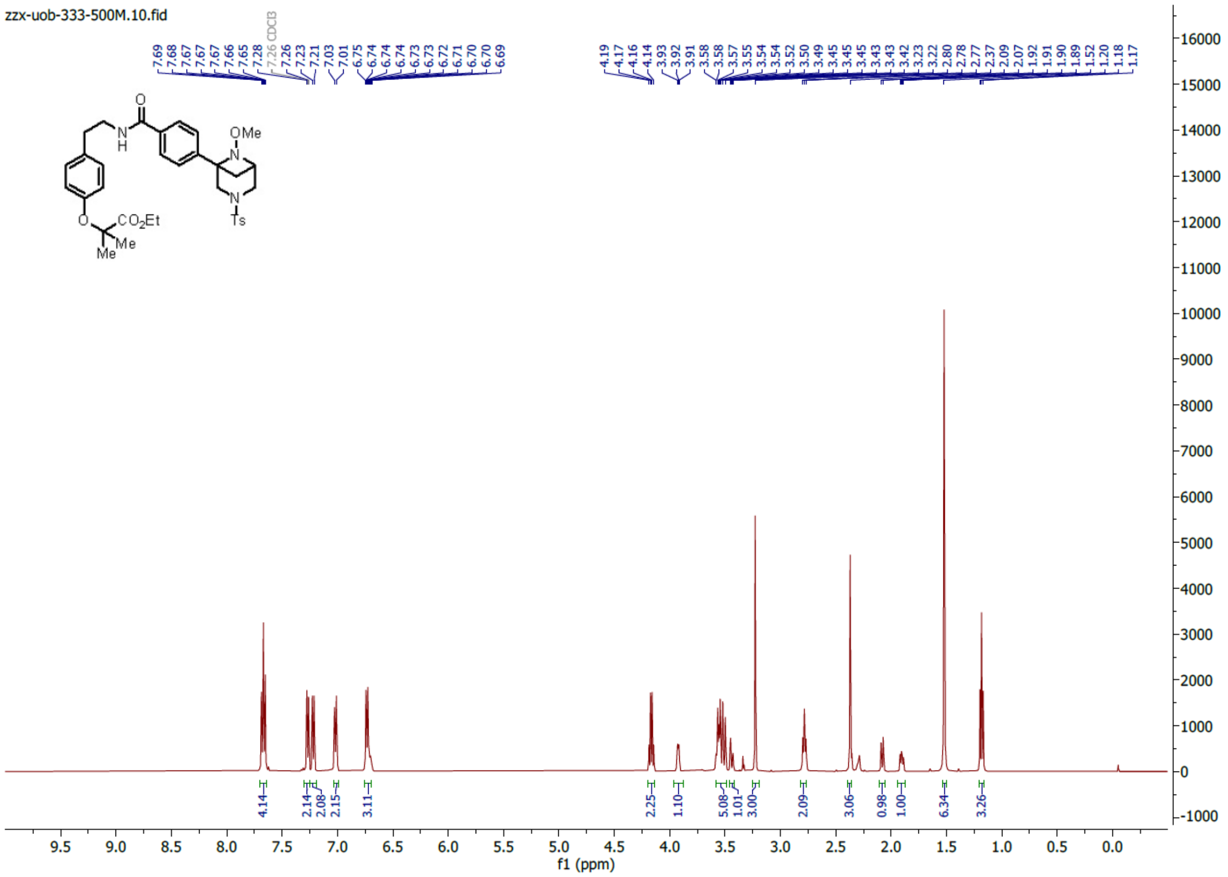

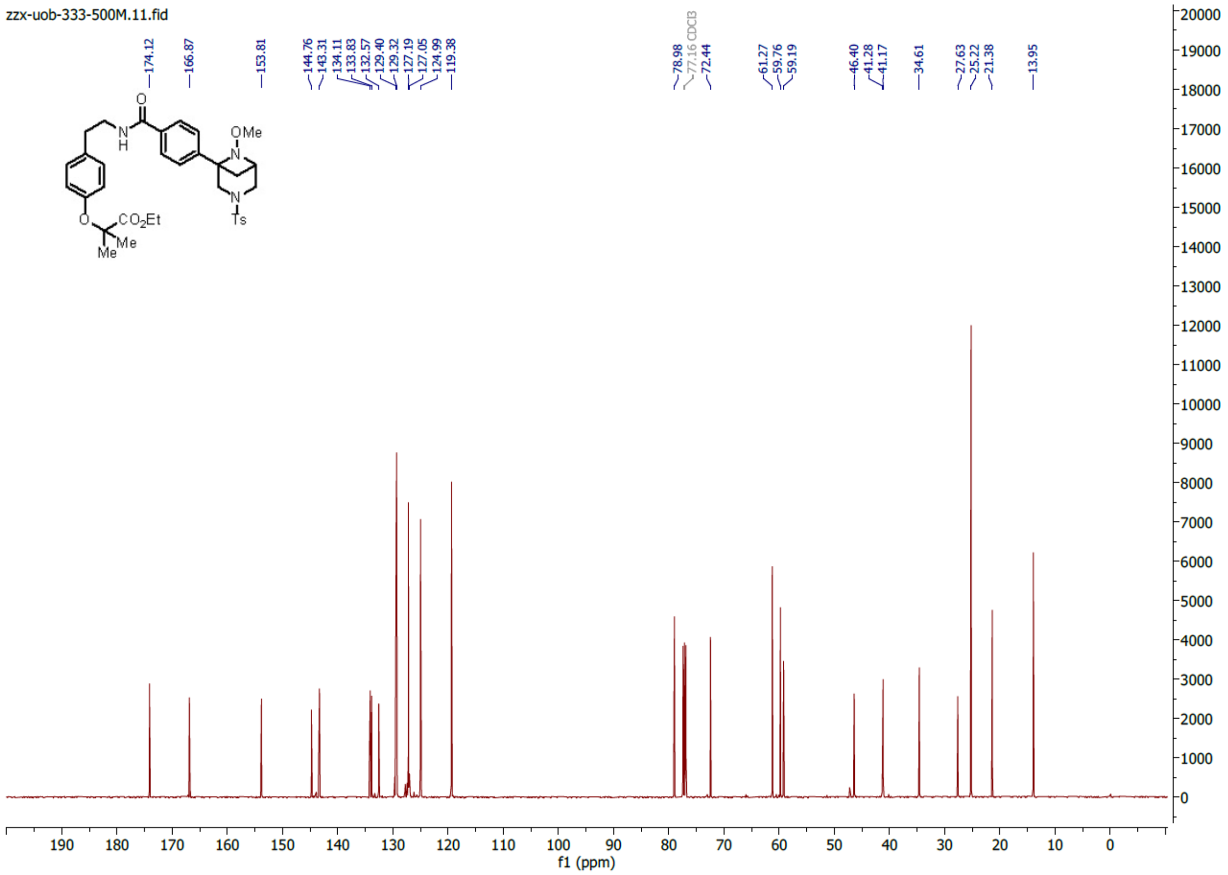

Compound 23

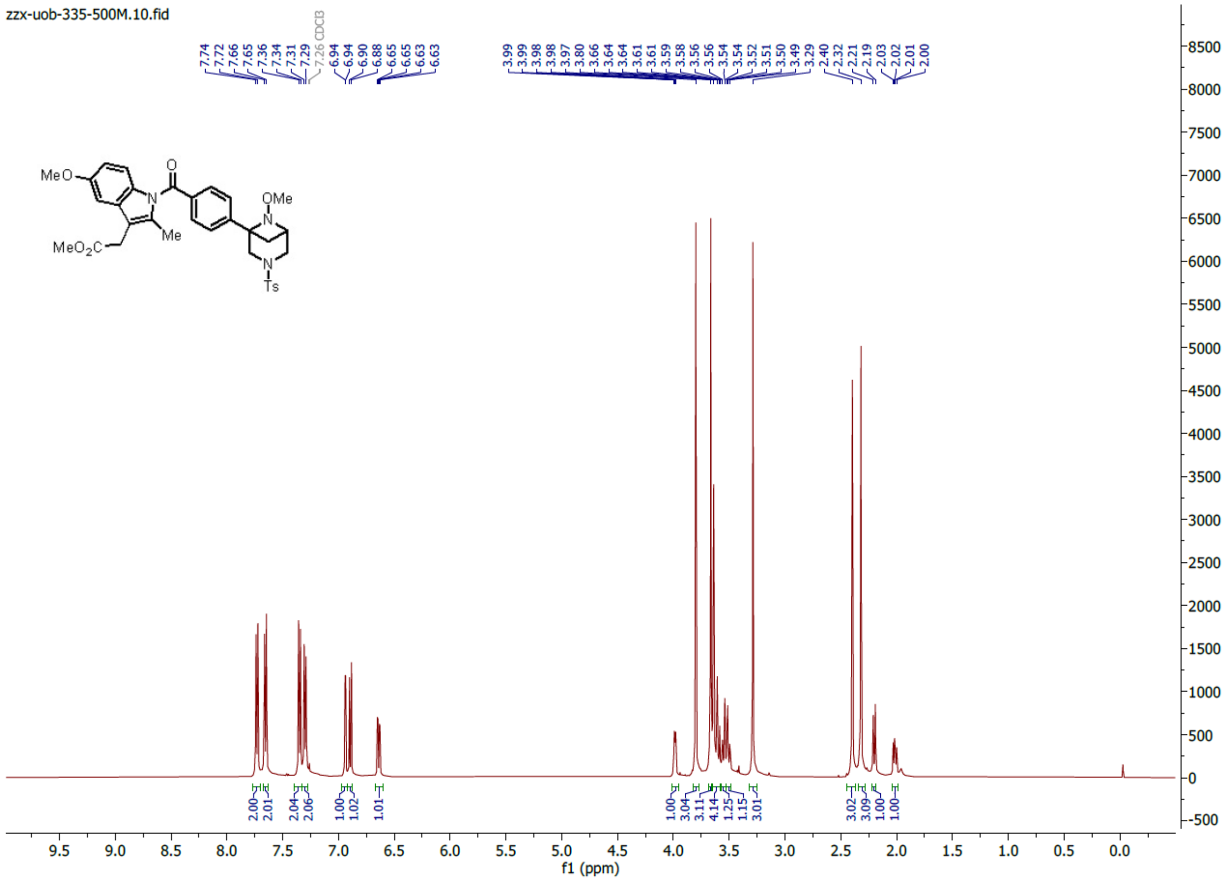
zzx-uob-335-500M.10.fid
7.74
7.72
7.66
7.65
7.36
7.34
7.31
7.29
7.26 CDCl3
6.94
6.94
6.90
6.88
6.65
6.65
6.63
6.63
3.99
3.99
3.98
3.98
3.97
3.80
3.66
3.64
3.64
3.61
3.61
3.59
3.58
3.56
3.56
3.54
3.54
3.52
3.51
3.50
3.49
3.29
2.40
2.32
2.21
2.19
2.03
2.02
2.01
2.00
MeO
O
N
OMe
Me
MeO2C
N
Ts
2.00
2.01
2.04
2.06
1.00
1.02
1.01
1.00
3.04
3.11
4.14
1.25
1.15
3.01
3.02
3.09
1.00
1.00
f1 (ppm)

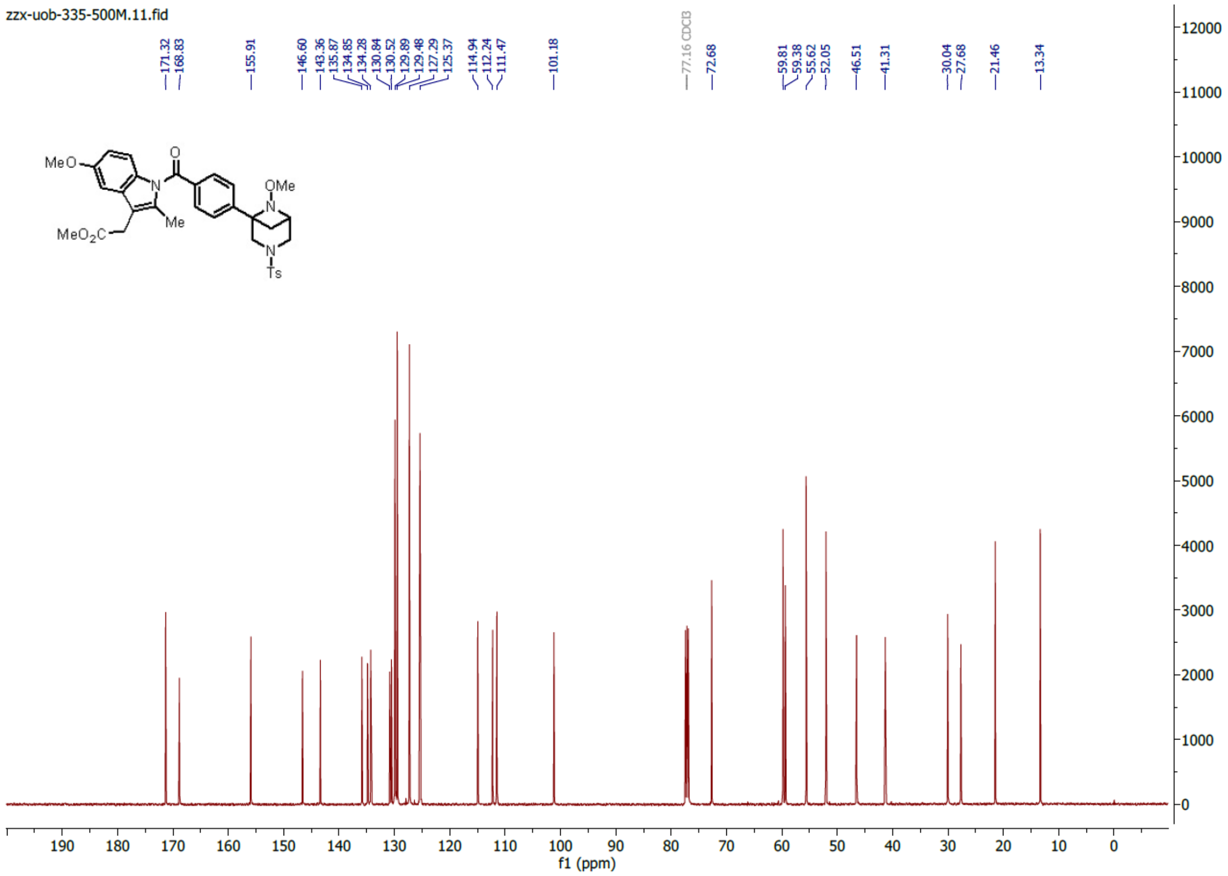
zzx-uob-335-500M.11.fid
171.32
168.83
155.91
146.60
143.36
135.87
134.85
134.28
130.84
130.52
129.89
129.48
127.29
125.37
114.94
112.24
111.47
101.18
77.16 CDCl3
72.68
59.81
59.38
55.62
52.05
46.51
41.31
30.04
27.68
21.46
13.34
MeO
O
N
OMe
Me
MeO2C
N
Ts
f1 (ppm)

Compound 24

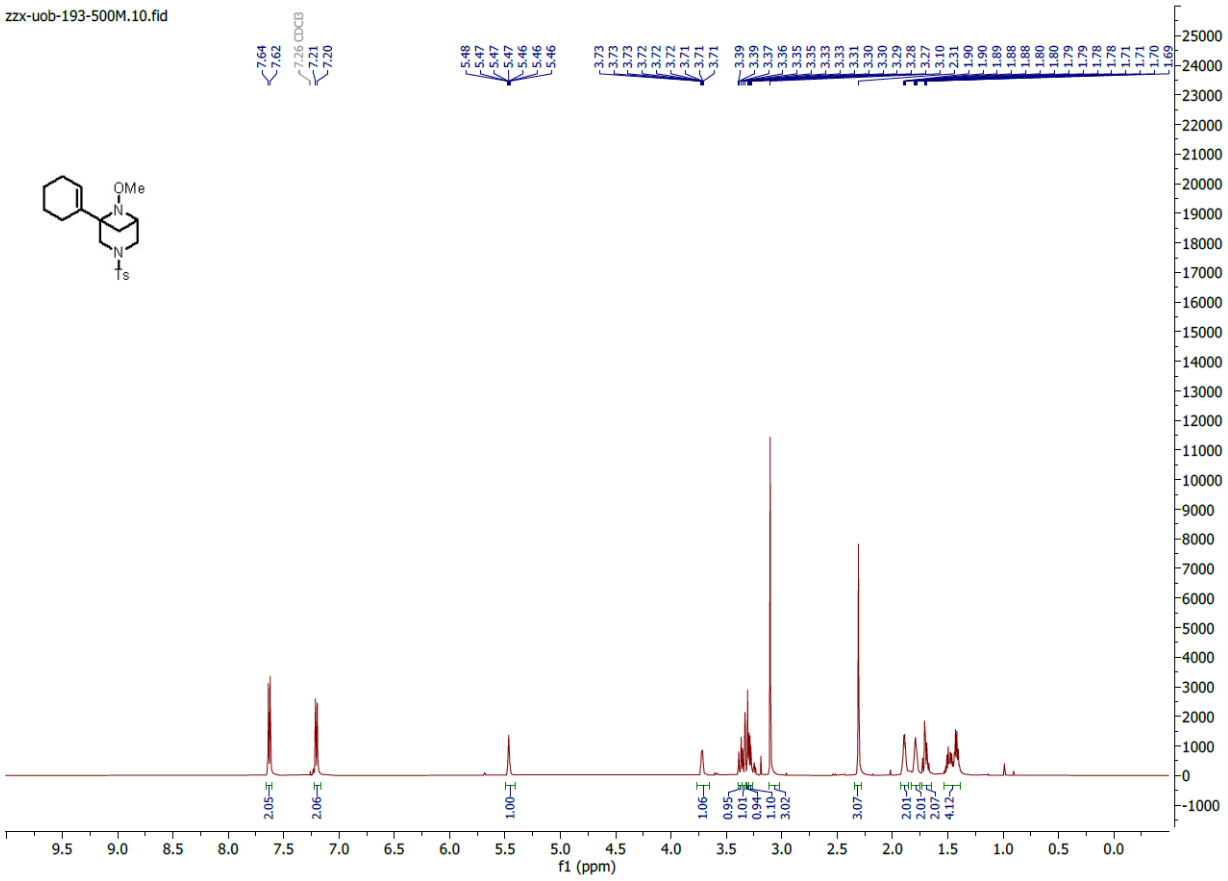
zzx-uob-193-500M.10.fid
7.64
7.62
7.26 CDCl3
7.21
7.20
5.48
5.47
5.47
5.47
5.46
5.46
5.46
3.73
3.73
3.73
3.72
3.72
3.72
3.71
3.71
3.71
3.39
3.39
3.37
3.36
3.35
3.35
3.33
3.33
3.31
3.30
3.30
3.29
3.28
3.27
3.10
2.31
1.90
1.90
1.89
1.88
1.88
1.80
1.80
1.79
1.79
1.78
1.78
1.71
1.71
1.70
1.69
OMe
N
N
Ts
2.05
2.06
1.00
1.06
0.95
1.01
0.94
1.10
3.02
3.07
2.01
2.01
2.07
4.12
f1 (ppm)

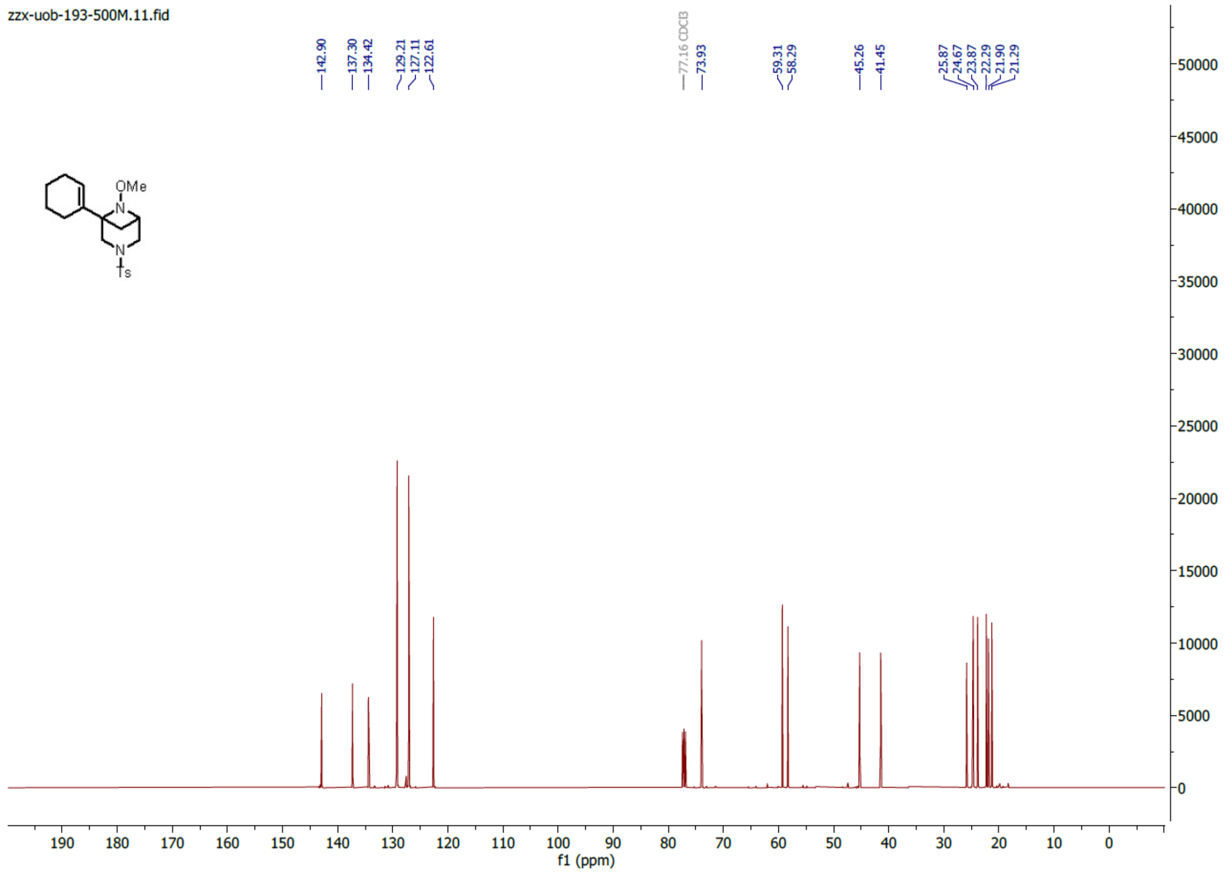
zzx-uob-193-500M.11.fid
142.90
137.30
134.42
129.21
127.11
122.61
77.16 CDCl3
73.93
59.31
58.29
45.26
41.45
25.87
24.67
23.87
22.29
21.90
21.29
OMe
N
N
Ts
f1 (ppm)

Compound 25

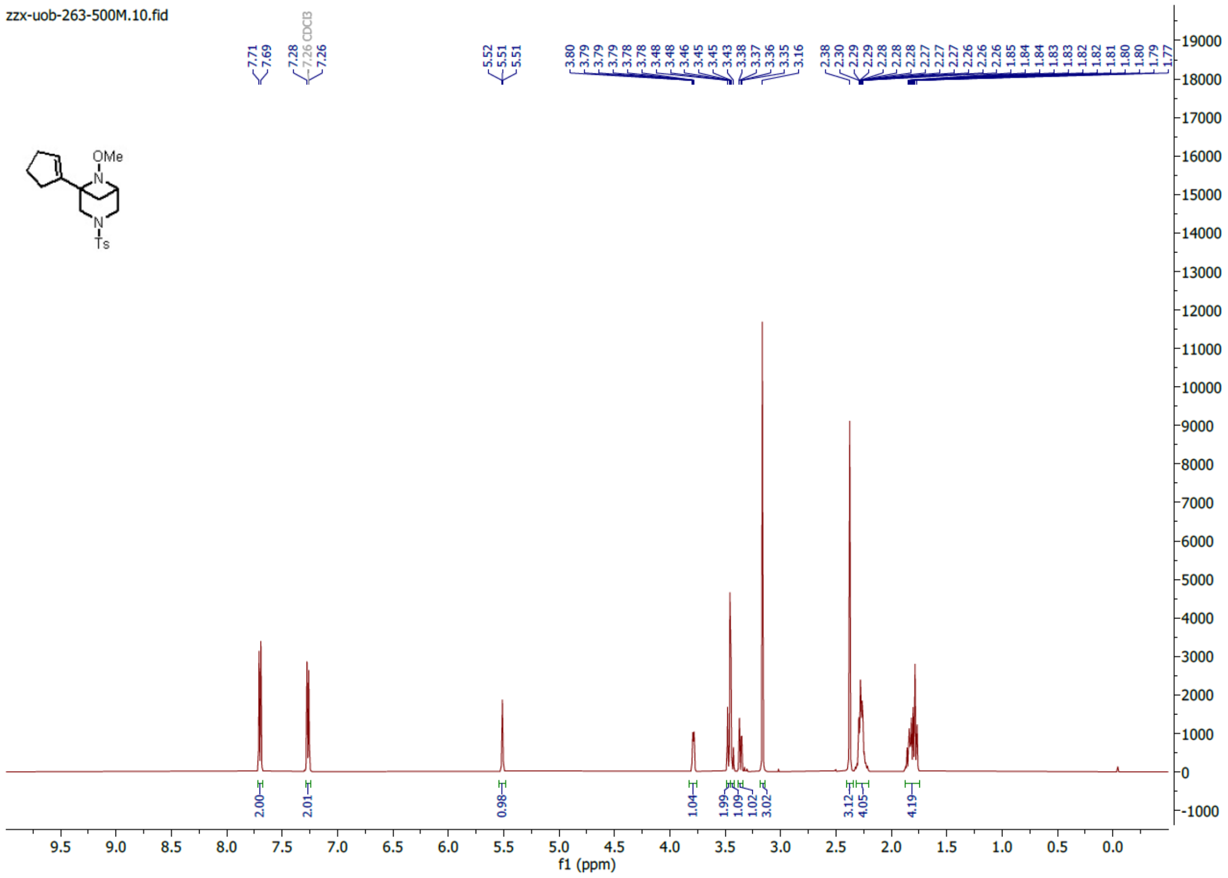
zzx-uob-263-500M.10.fid
OMe
N
N
Ts
f1 (ppm)

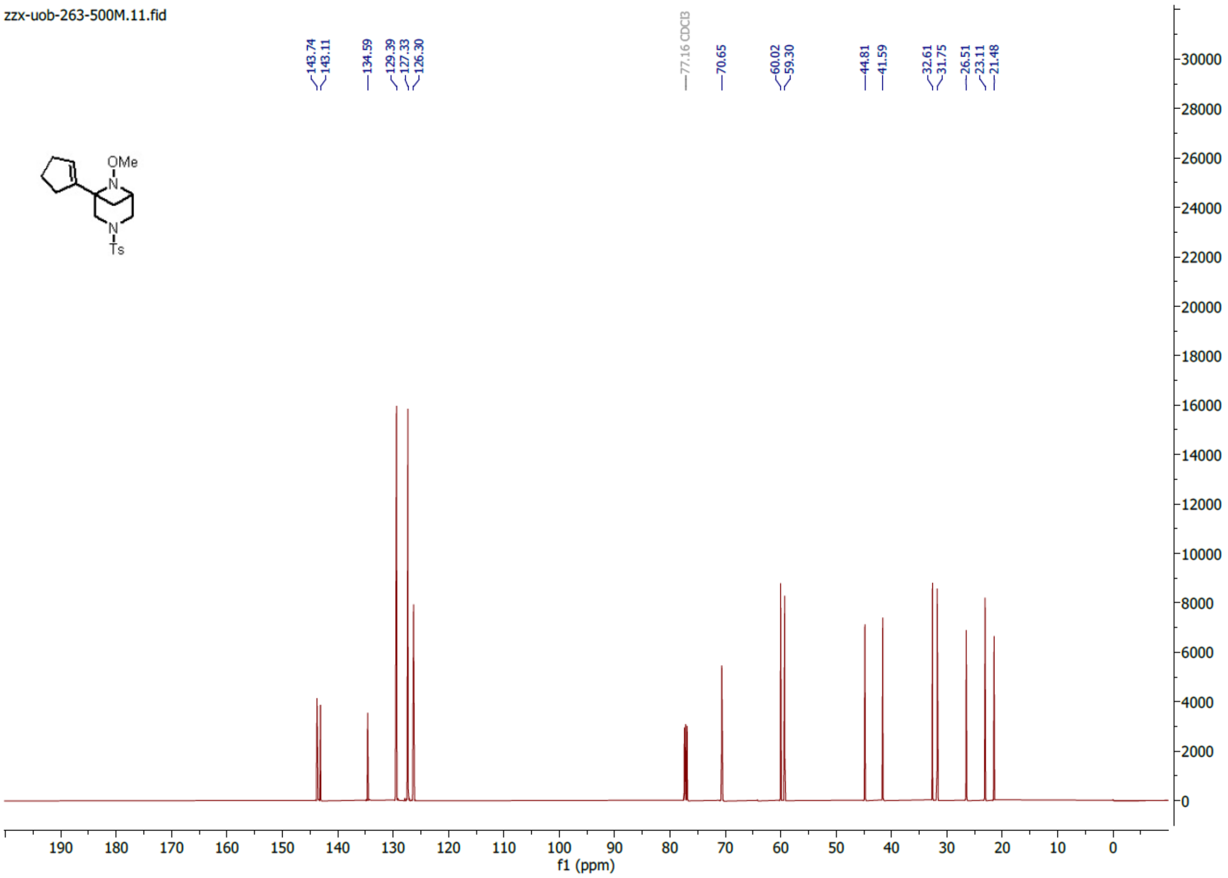
zzx-uob-263-500M.11.fid
OMe
N
N
Ts
f1 (ppm)

Compound 26

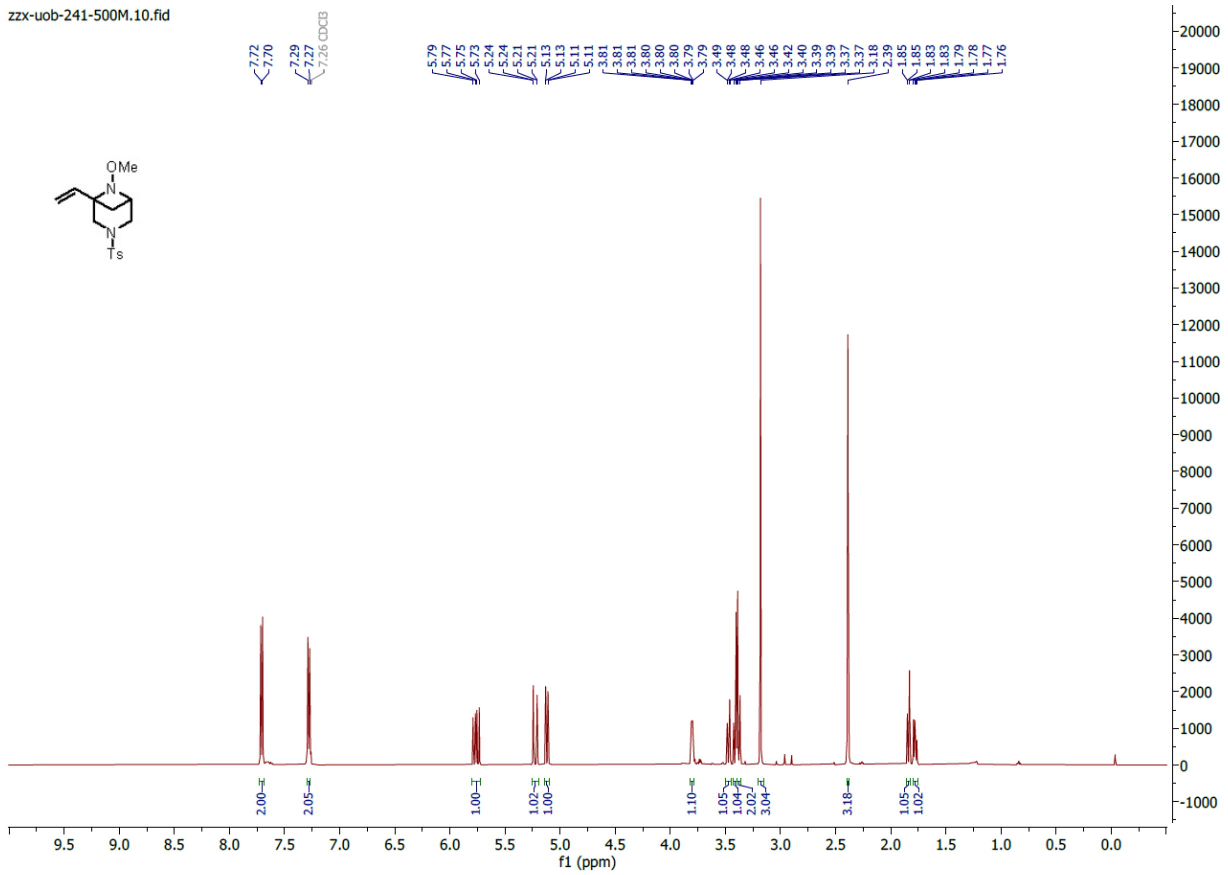
zzx-uob-241-500M.10.fid
OMe
N
N
Ts
f1 (ppm)

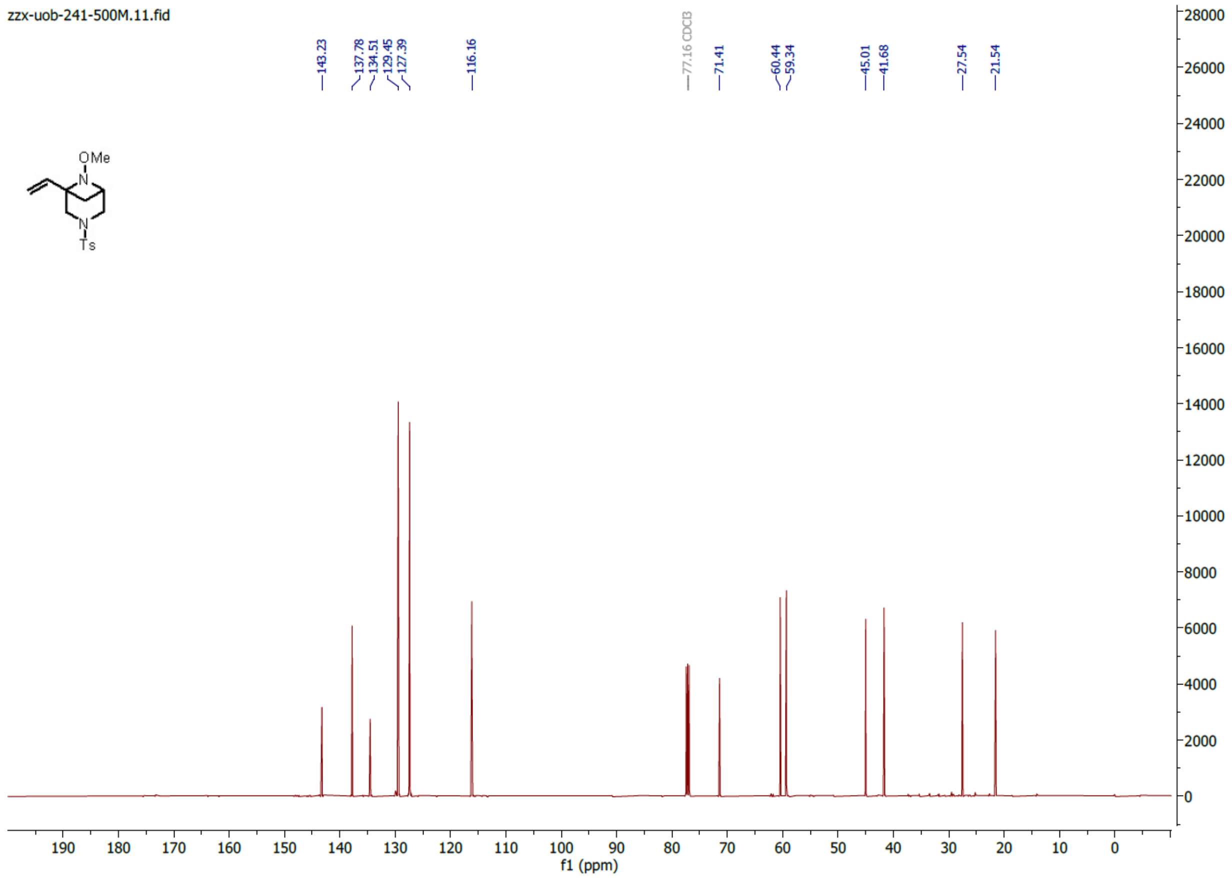
zzx-uob-241-500M.11.fid
143.23
137.78
134.51
129.45
127.39
116.16
77.16 CDCl3
71.41
60.44
59.34
45.01
41.68
27.54
21.54
OMe
N
N
Ts
f1 (ppm)

Compound 27

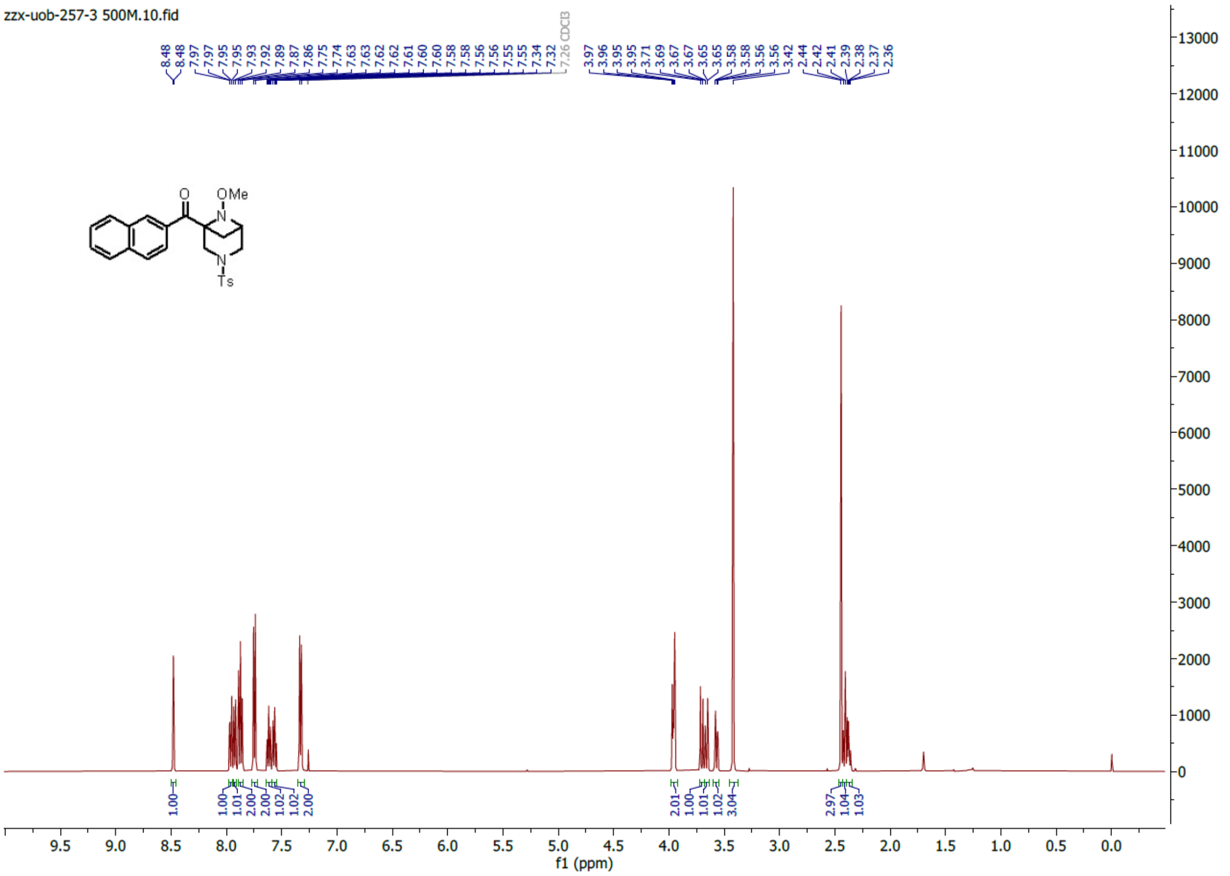
zzx-uob-257-3 500M.10.fid
f1 (ppm)
OMe
Ts

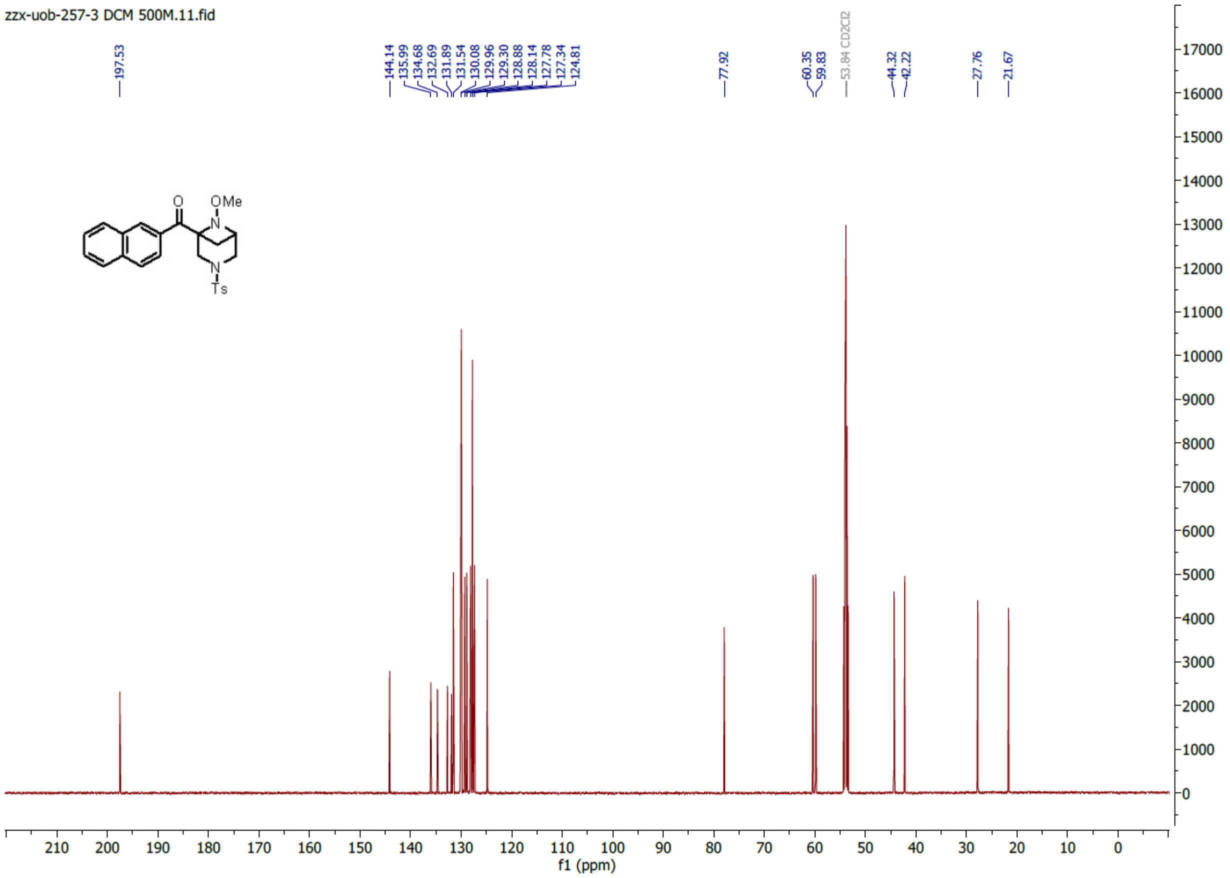
zzx-uob-257-3 DCM 500M.11.fid
f1 (ppm)
OMe
Ts

Compound 27a

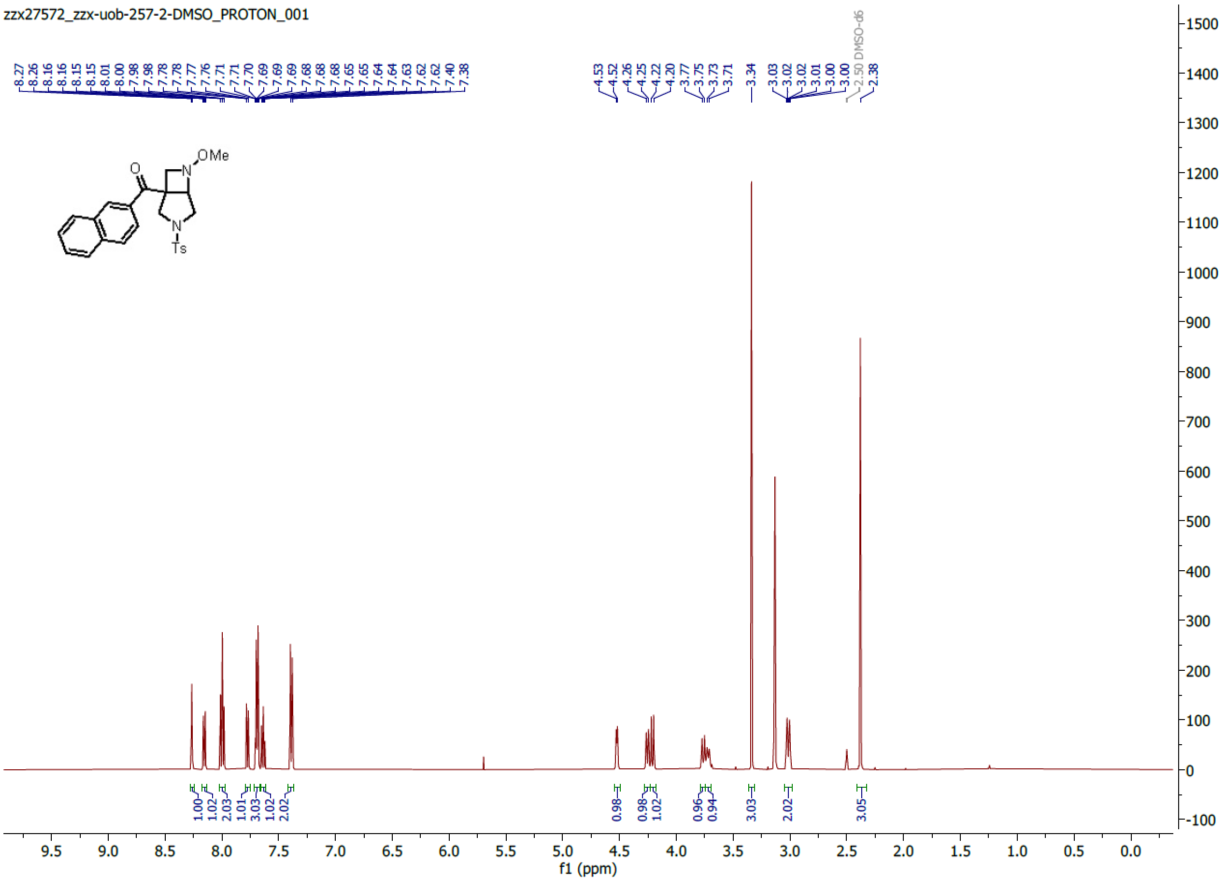

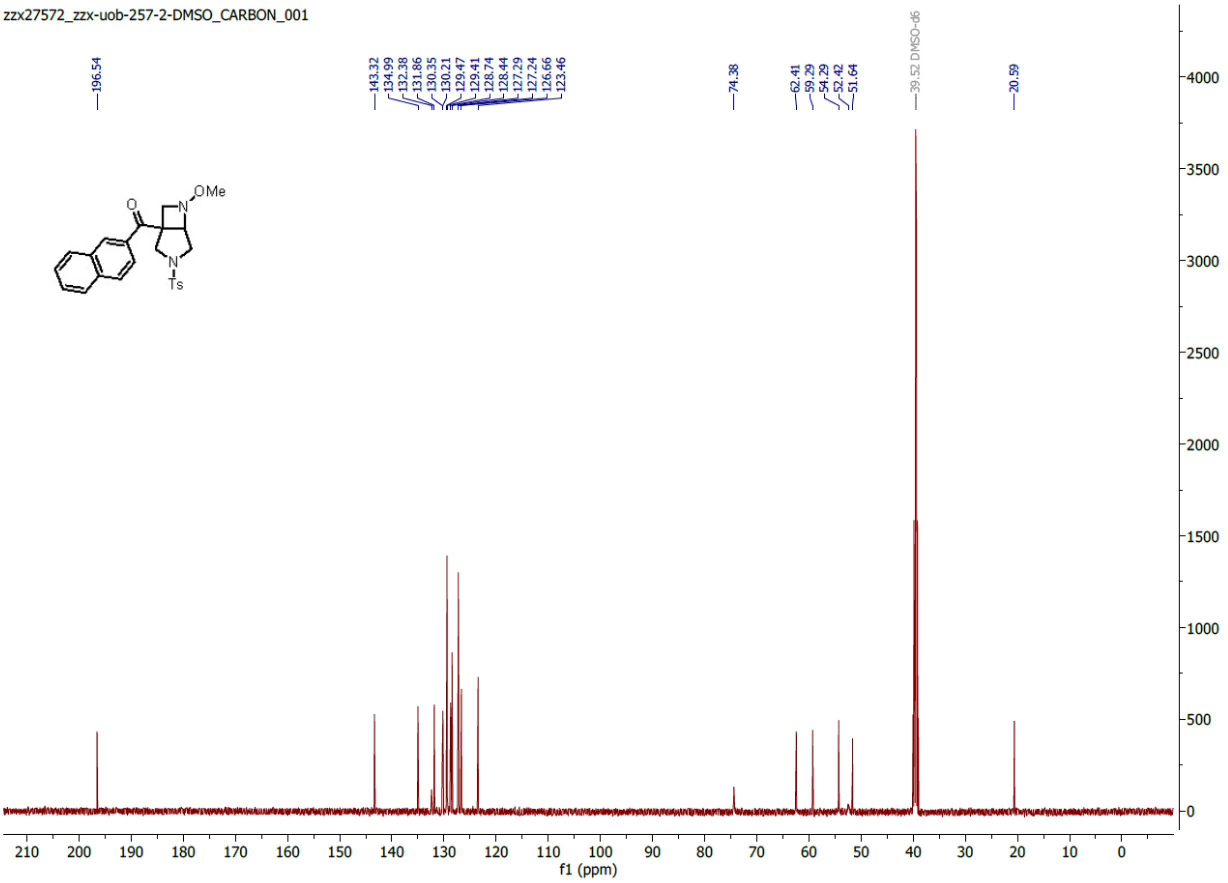

Compound 28

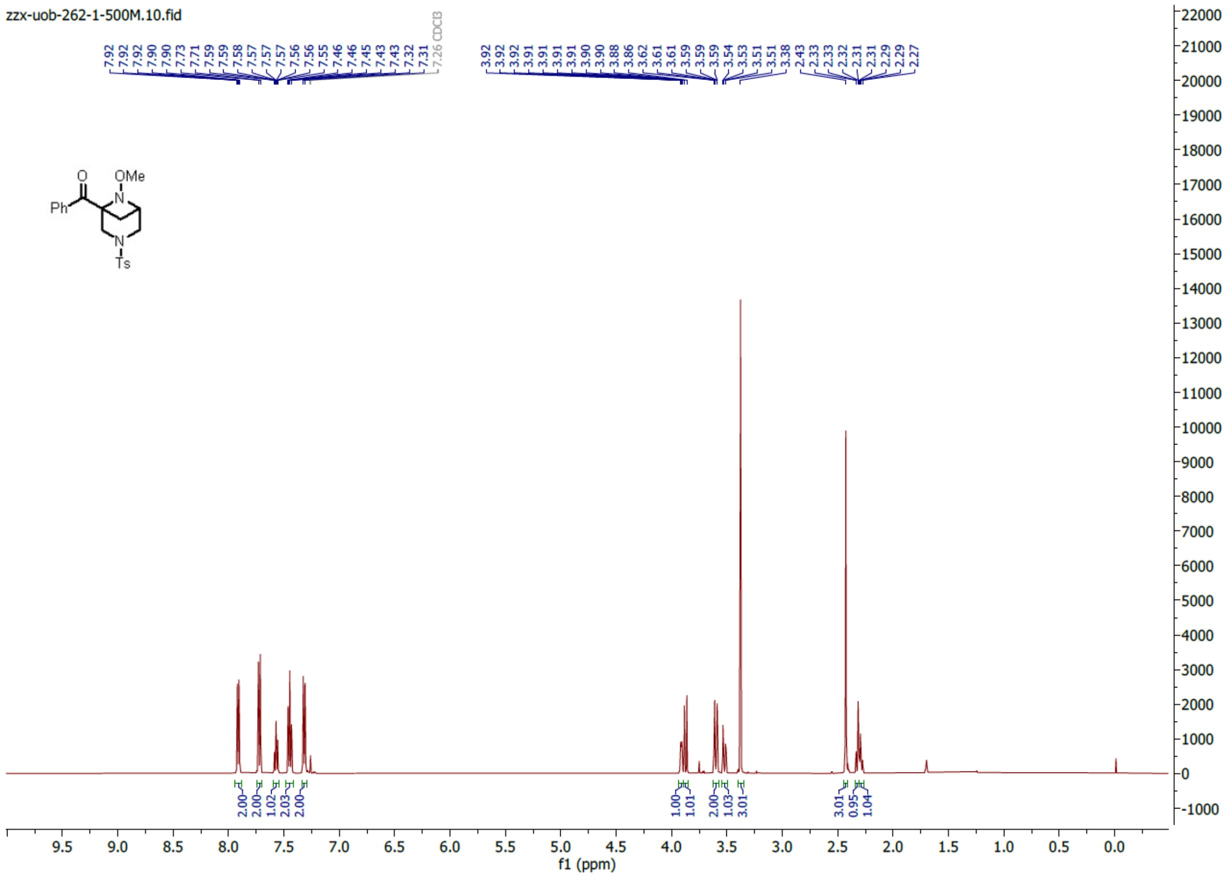
zzx-uob-262-1-500M.10.fid
7.92 7.92 7.92 7.90 7.90 7.73 7.71 7.59 7.59 7.58 7.57 7.57 7.56 7.56 7.55 7.46 7.45 7.43 7.43 7.32 7.31 7.26 CDCl3
3.92 3.92 3.92 3.91 3.91 3.91 3.91 3.90 3.90 3.88 3.86 3.62 3.61 3.61 3.59 3.59 3.59 3.54 3.53 3.51 3.51 3.38 2.43 2.33 2.33 2.32 2.31 2.31 2.29 2.29 2.27
OMe
Ph
O
N
N
Ts
2.00 2.00 1.02 2.03 2.00
1.00 1.01 2.00 1.03 3.01
3.01 0.95 1.04
f1 (ppm)

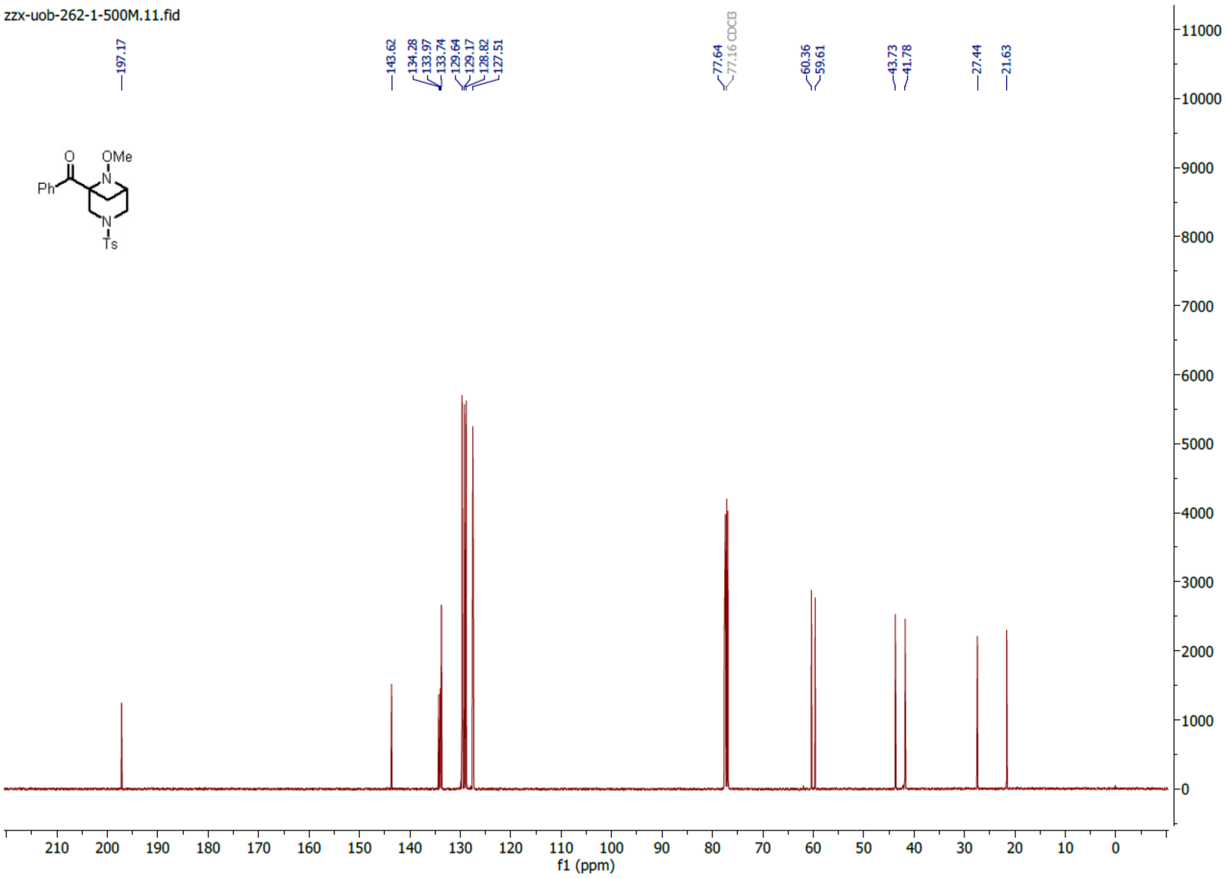
zzx-uob-262-1-500M.11.fid
197.17
143.62 134.28 133.97 133.74 129.64 129.17 128.82 127.51
77.64 77.16 CDCl3
60.36 59.61
43.73 41.78
27.44
21.63
OMe
Ph
O
N
N
Ts
f1 (ppm)

Compound 28a

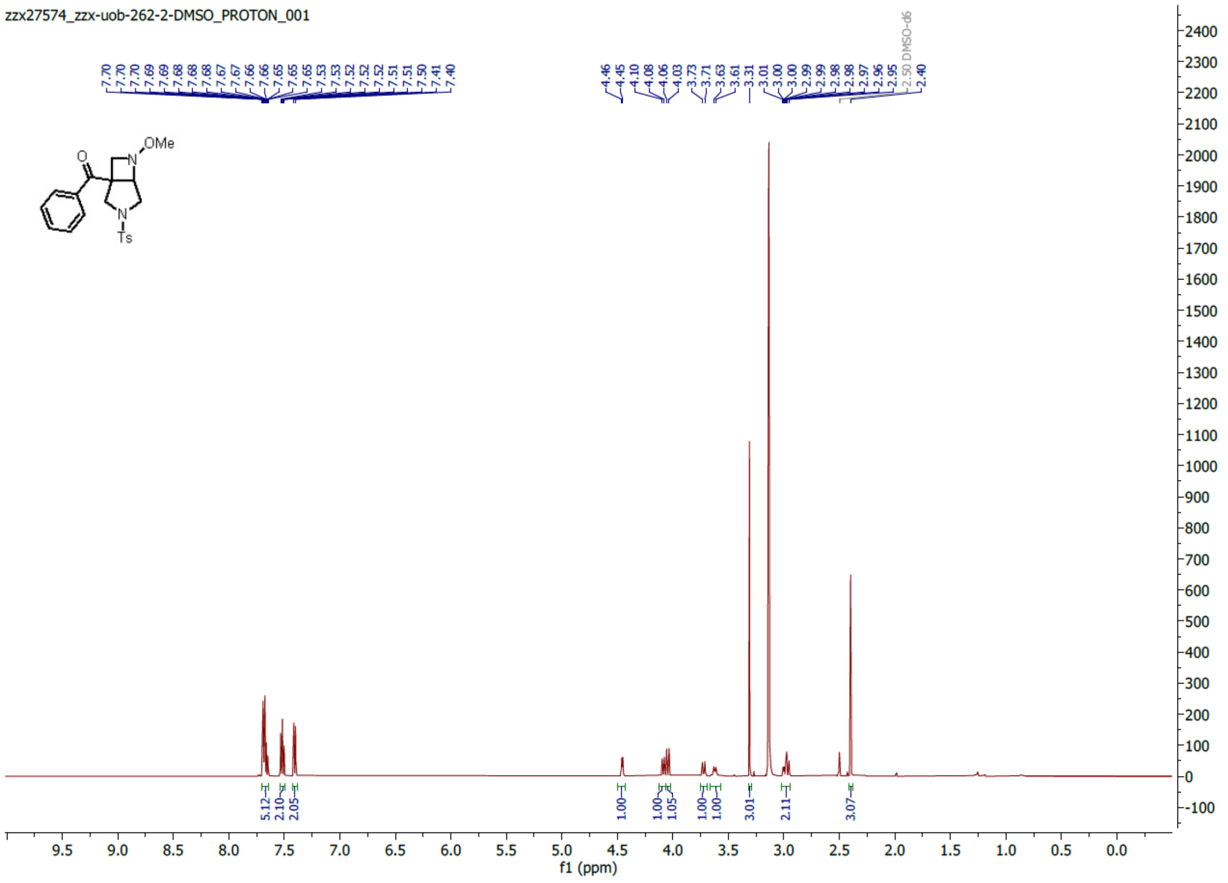

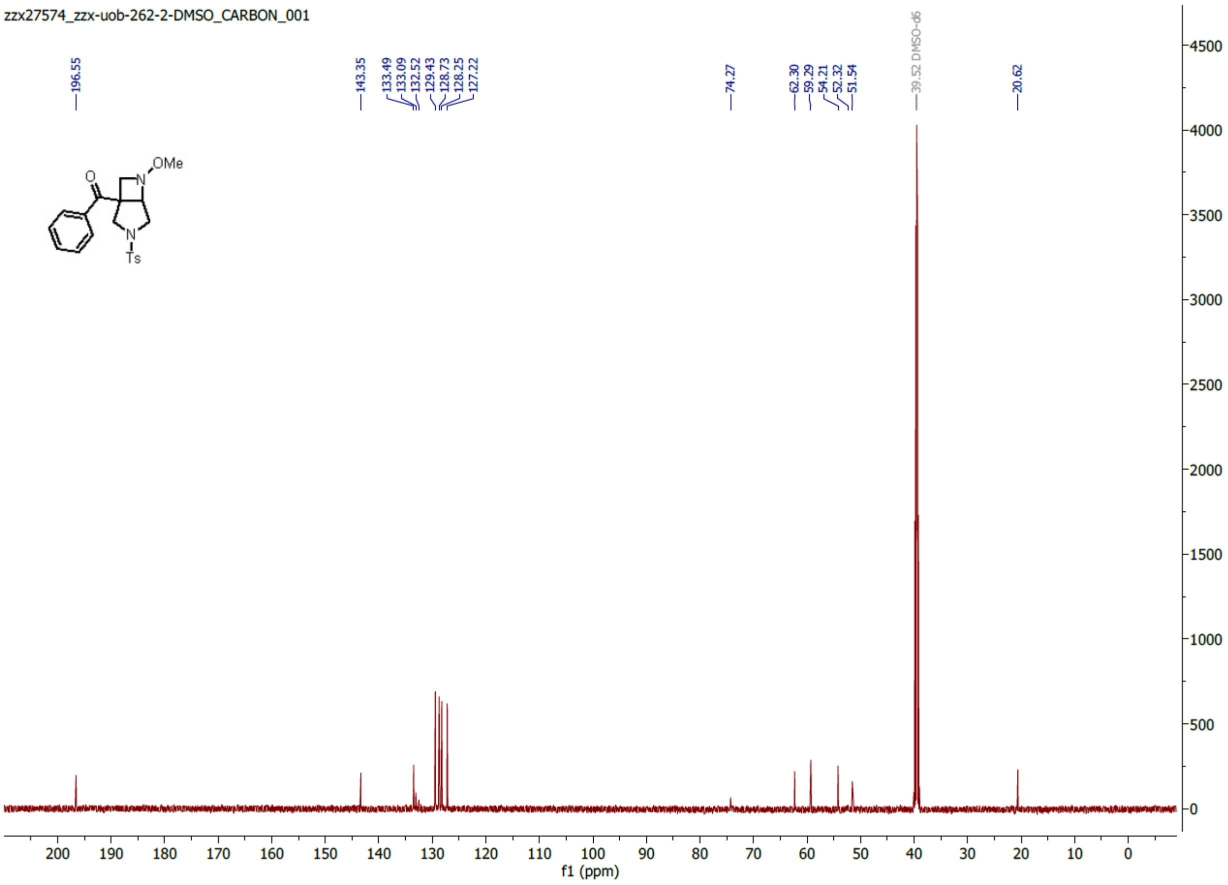

Compound 29

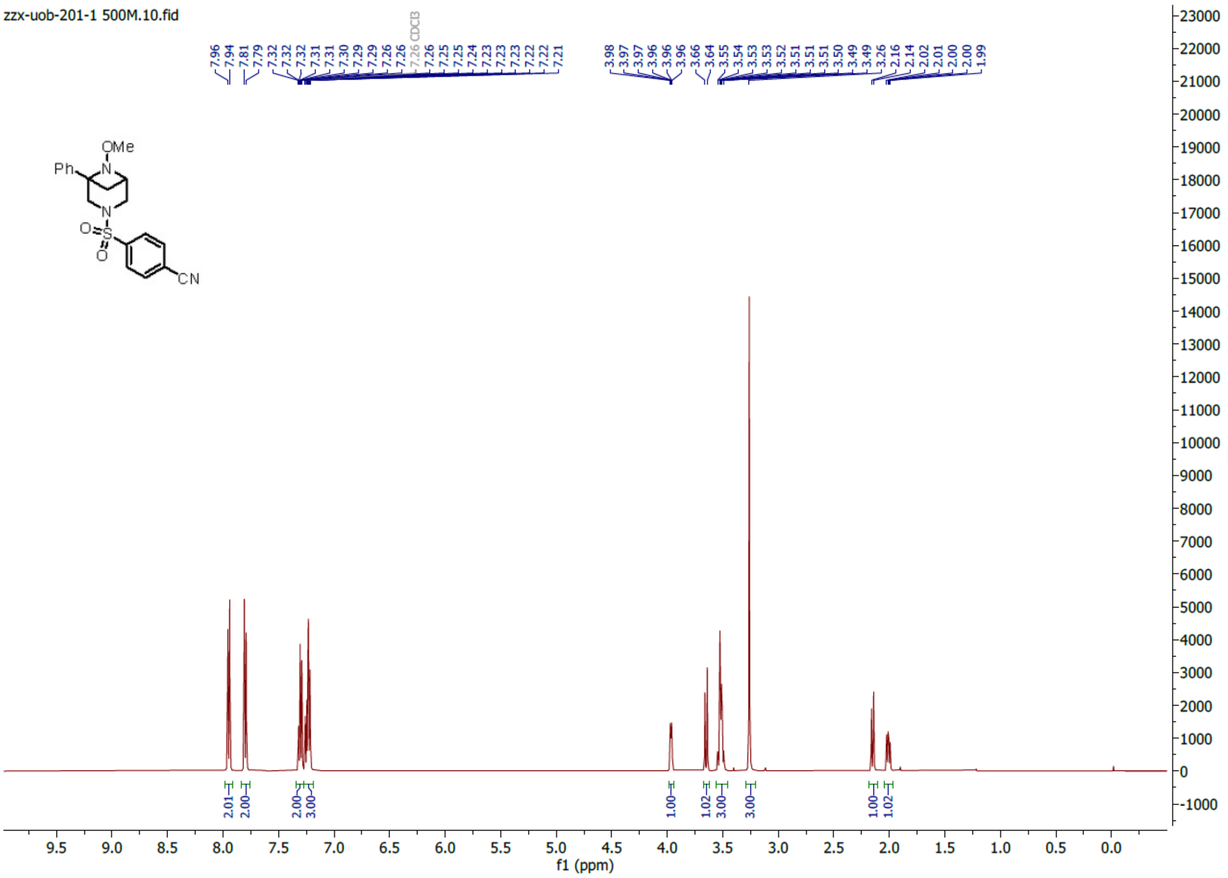
zzx-uob-201-1 500M.10.fid
OMe
Ph
N
N
O
S
O
CN
f1 (ppm)

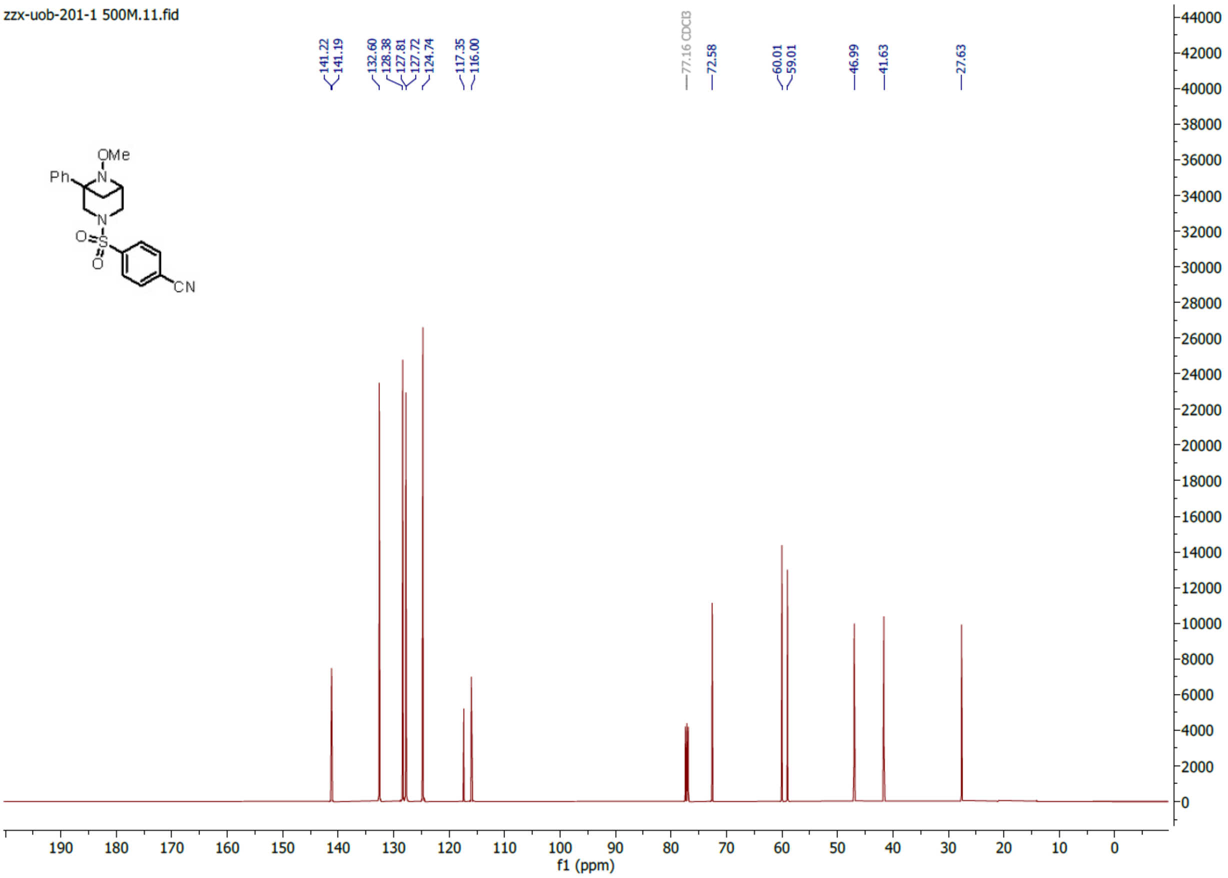
zzx-uob-201-1 500M.11.fid
OMe
Ph
N
N
O
S
O
CN
f1 (ppm)

Compound 29a

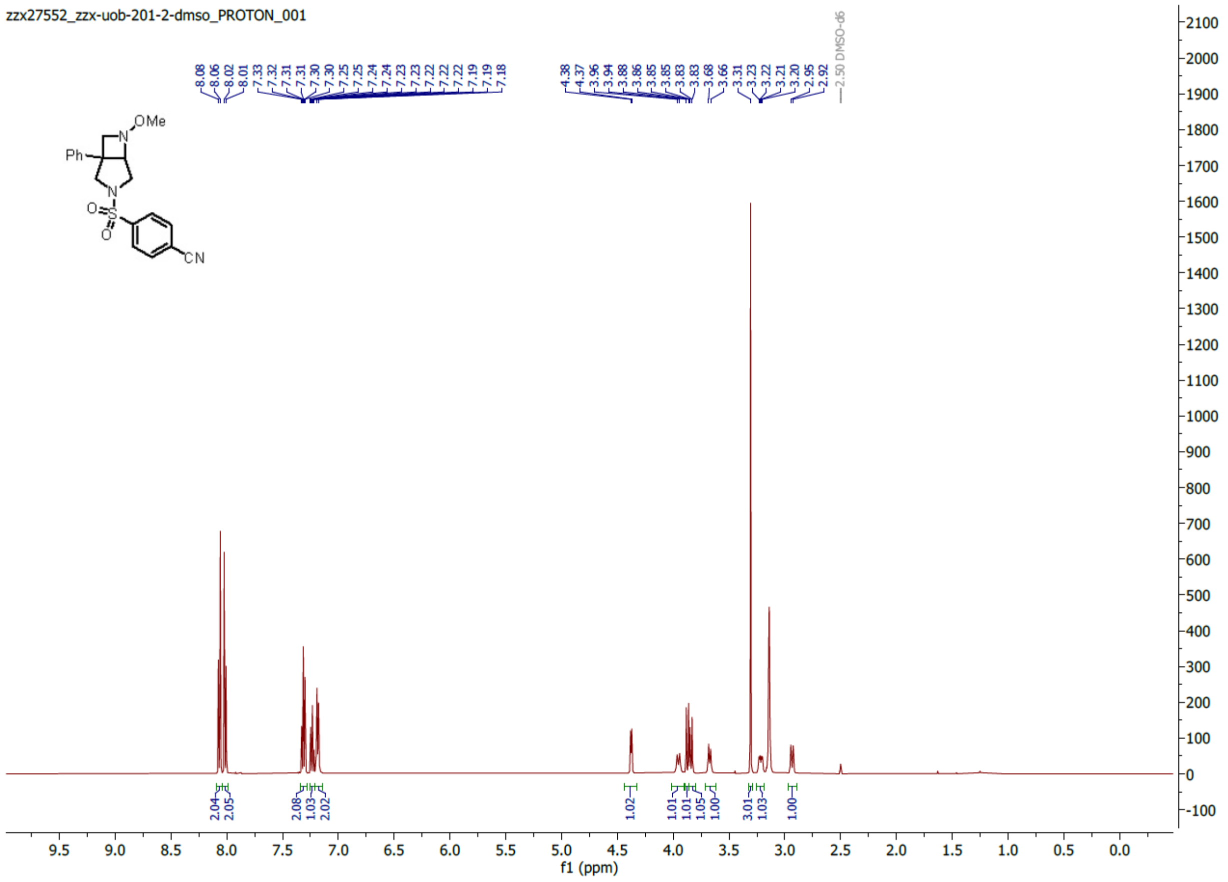

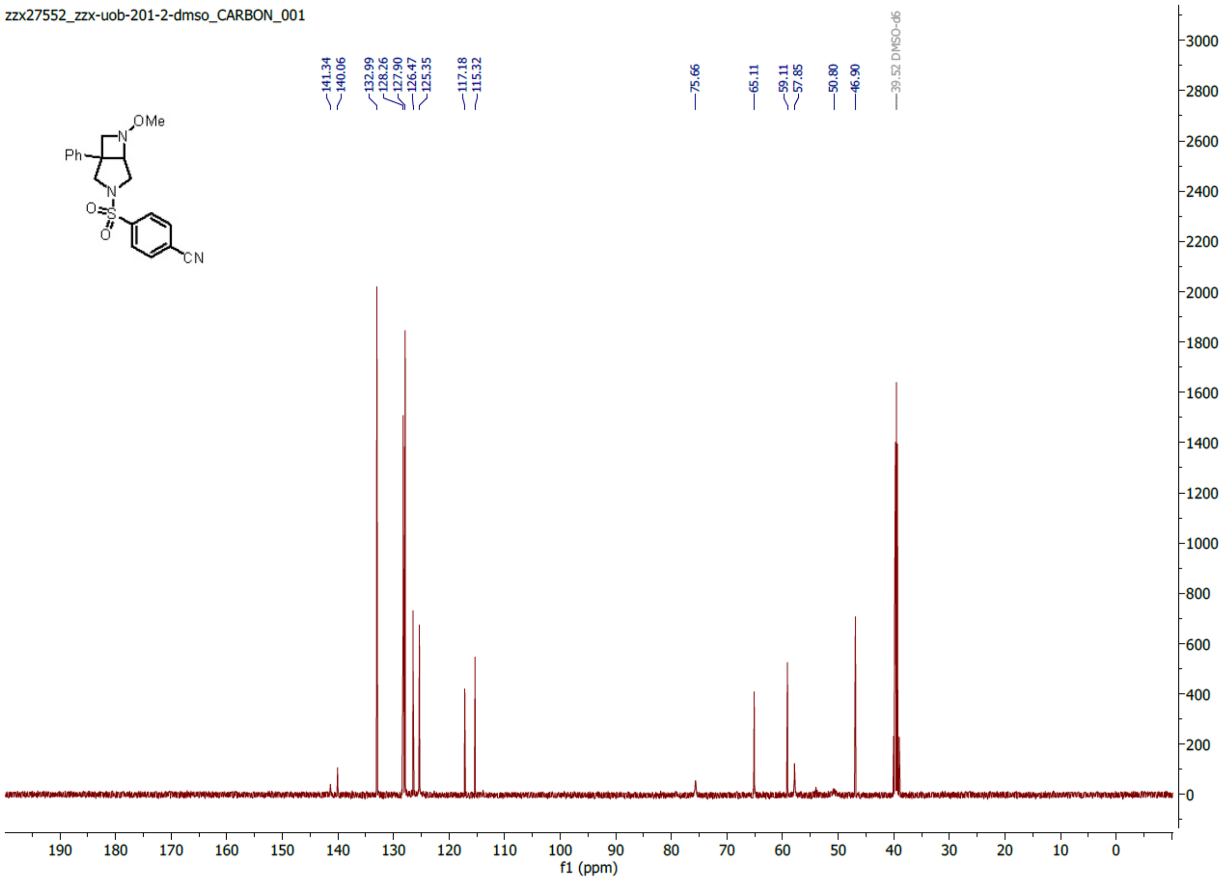

Compound 31

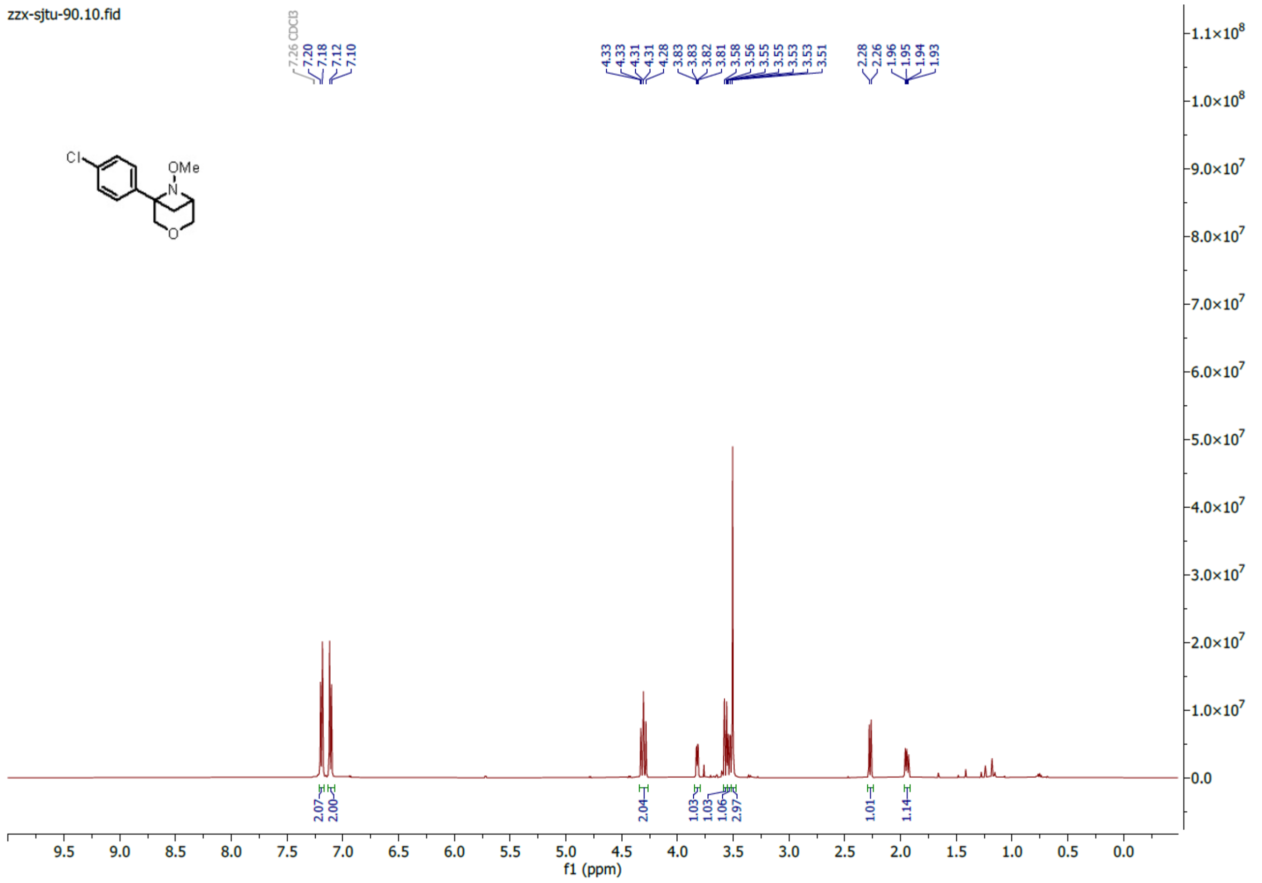
zzx-sjtu-90.10.fid
7.26 CDCl3
7.20
7.18
7.12
7.10
4.33
4.33
4.31
4.31
4.28
3.83
3.83
3.82
3.81
3.58
3.56
3.55
3.55
3.53
3.53
3.51
2.28
2.26
1.96
1.95
1.94
1.93
Cl
OMe
N
O
2.07
2.00
2.04
1.03
1.03
1.06
2.97
1.01
1.14
f1 (ppm)

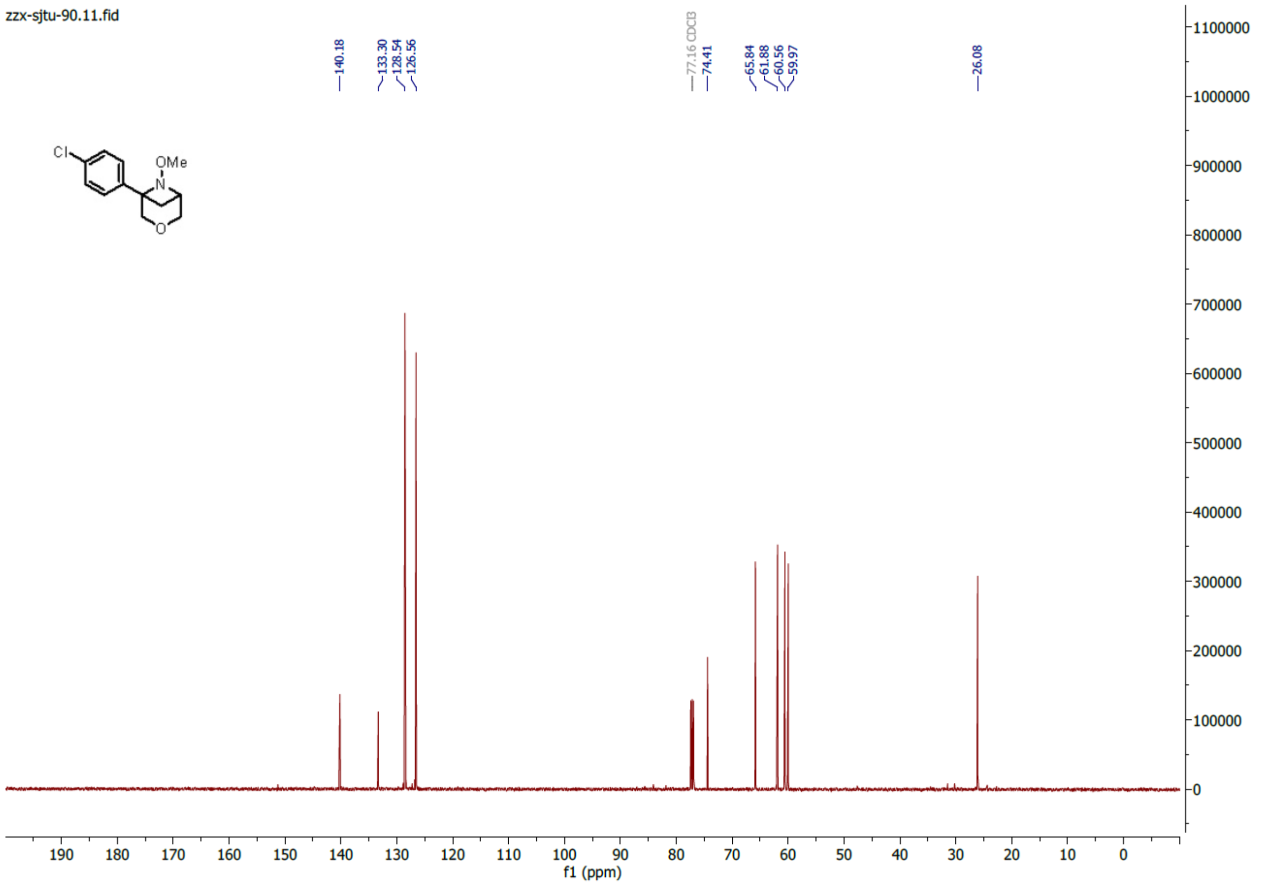
zzx-sjtu-90.11.fid
140.18
133.30
128.54
126.56
77.16 CDCl3
74.41
65.84
61.88
60.56
59.97
26.08
Cl
OMe
N
O
f1 (ppm)

Compound 32

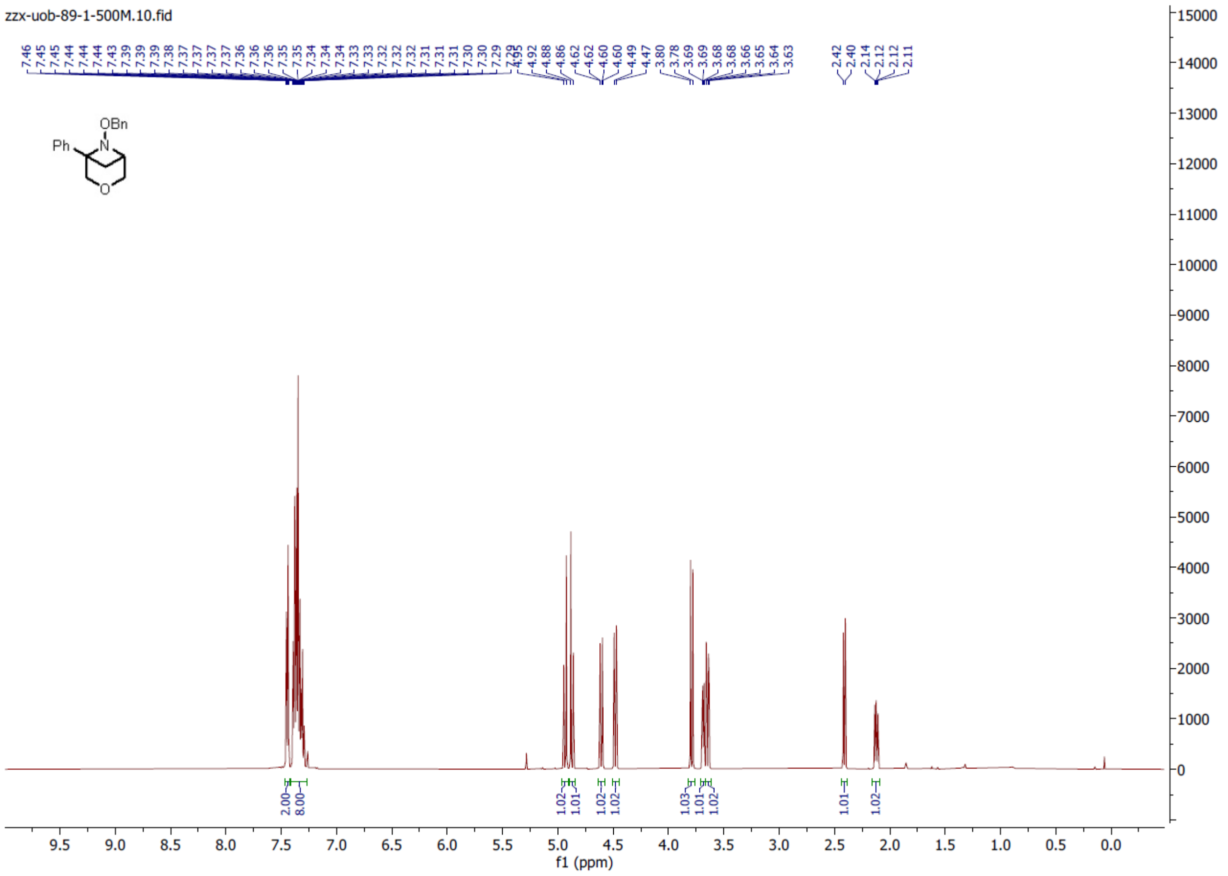
zzx-uob-89-1-500M.10.fid
OBn
Ph
N
O
2.00
8.00
1.02
1.01
1.02
1.02
1.03
1.01
1.02
1.01
1.02
f1 (ppm)

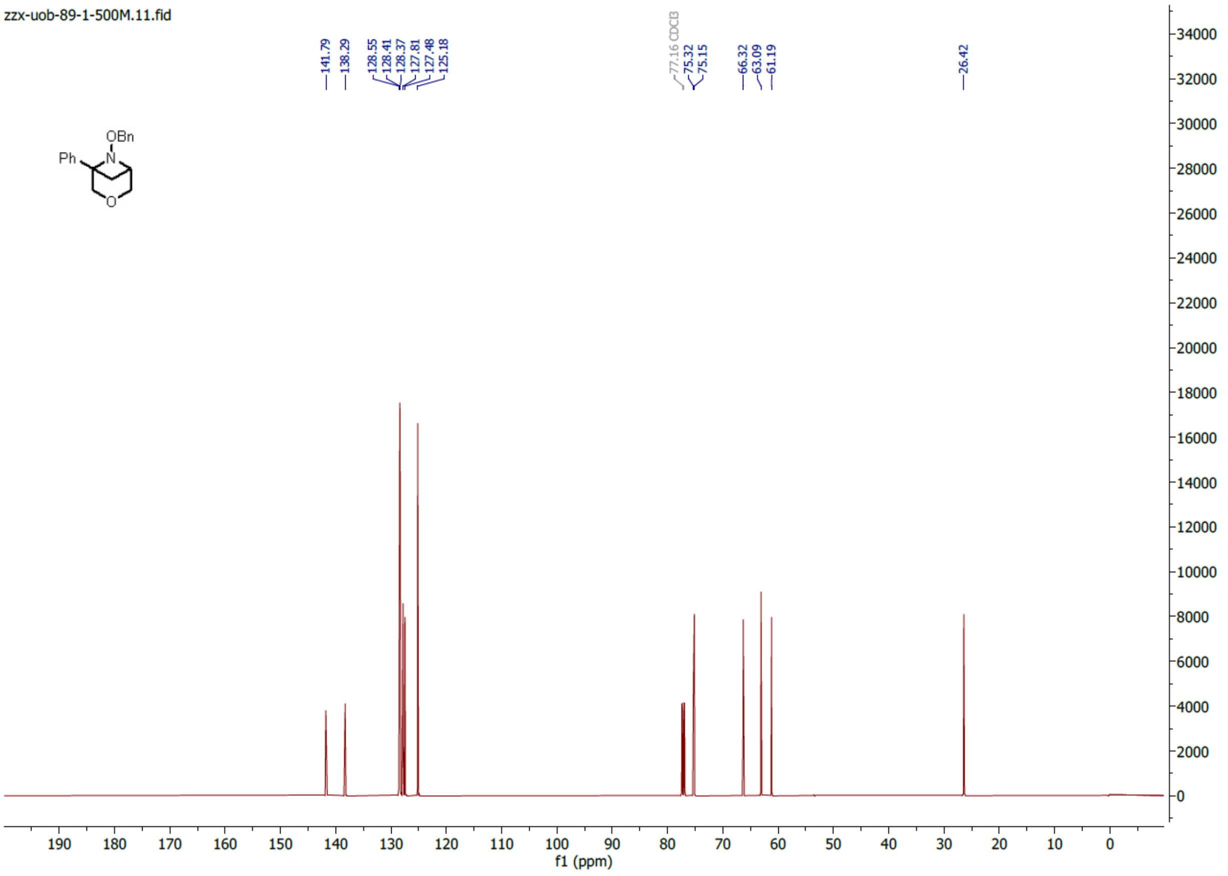
zzx-uob-89-1-500M.11.fid
141.79
138.29
128.55
128.41
128.37
127.81
127.48
125.18
77.16 CDCl3
75.32
75.15
66.32
63.09
61.19
26.42
OBn
Ph
N
O
f1 (ppm)

Compound 32a

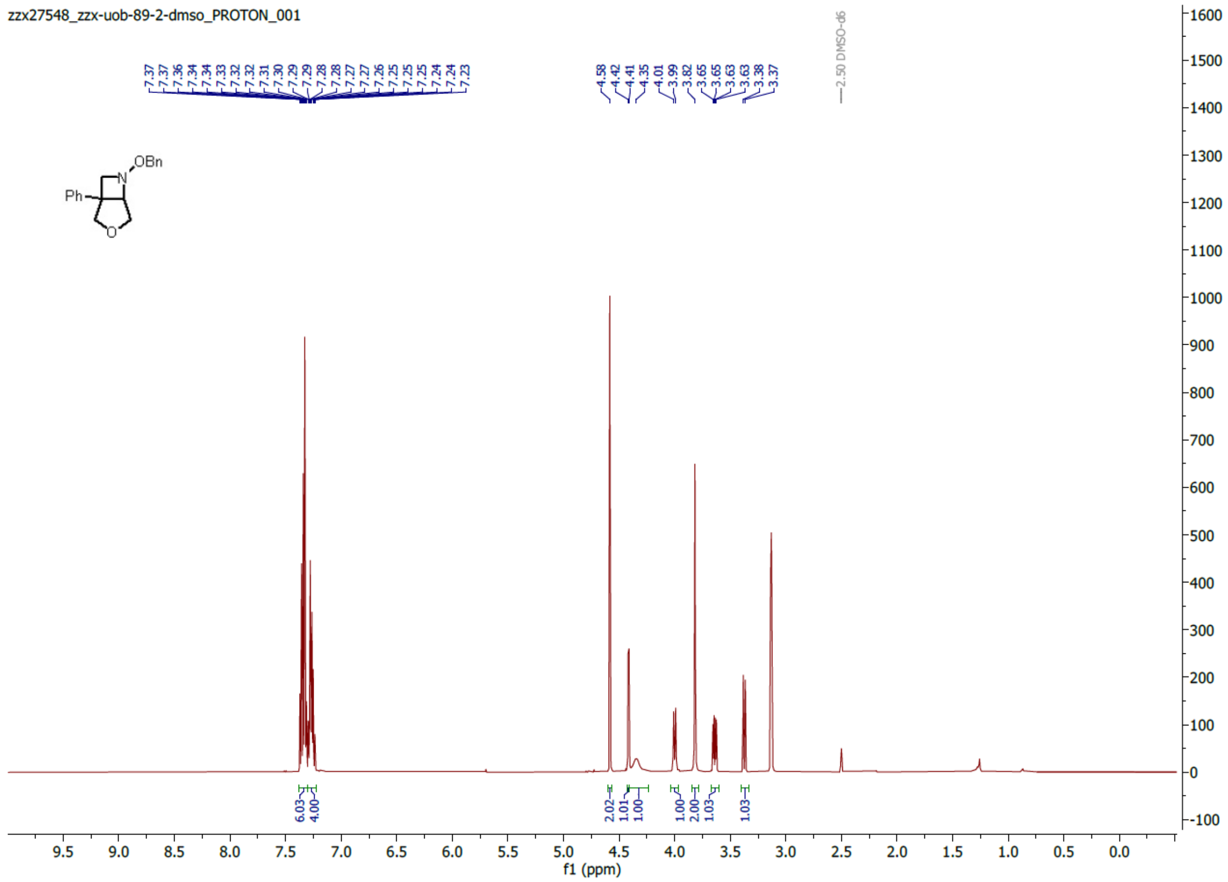

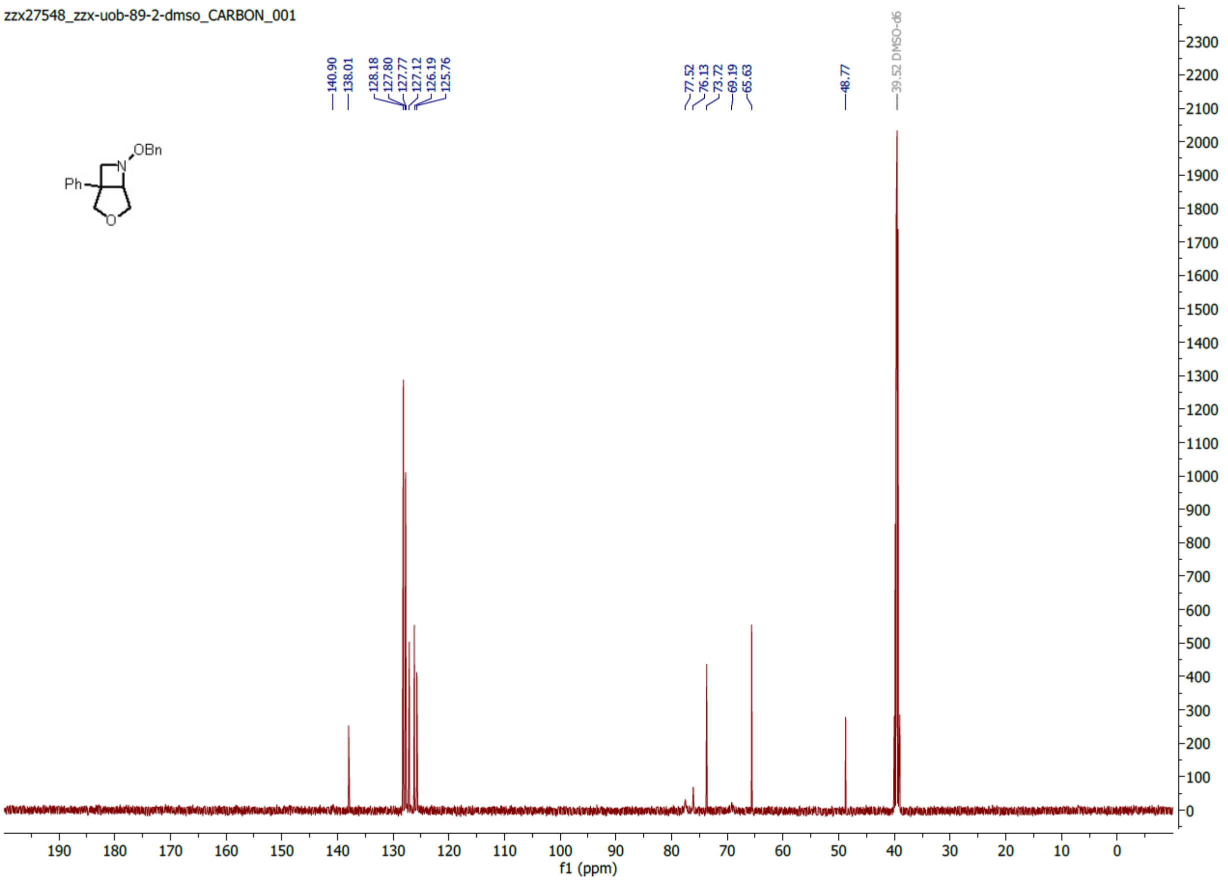

Compound 33

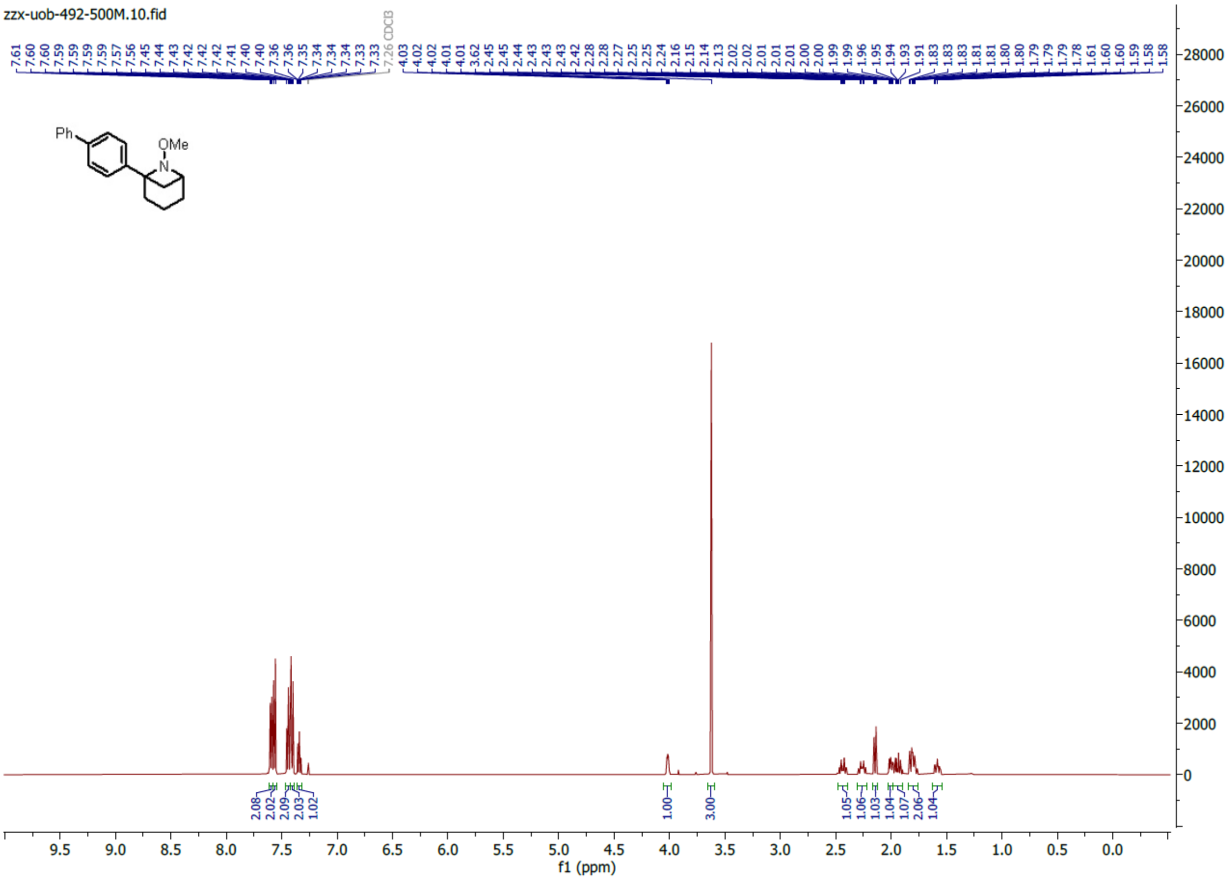

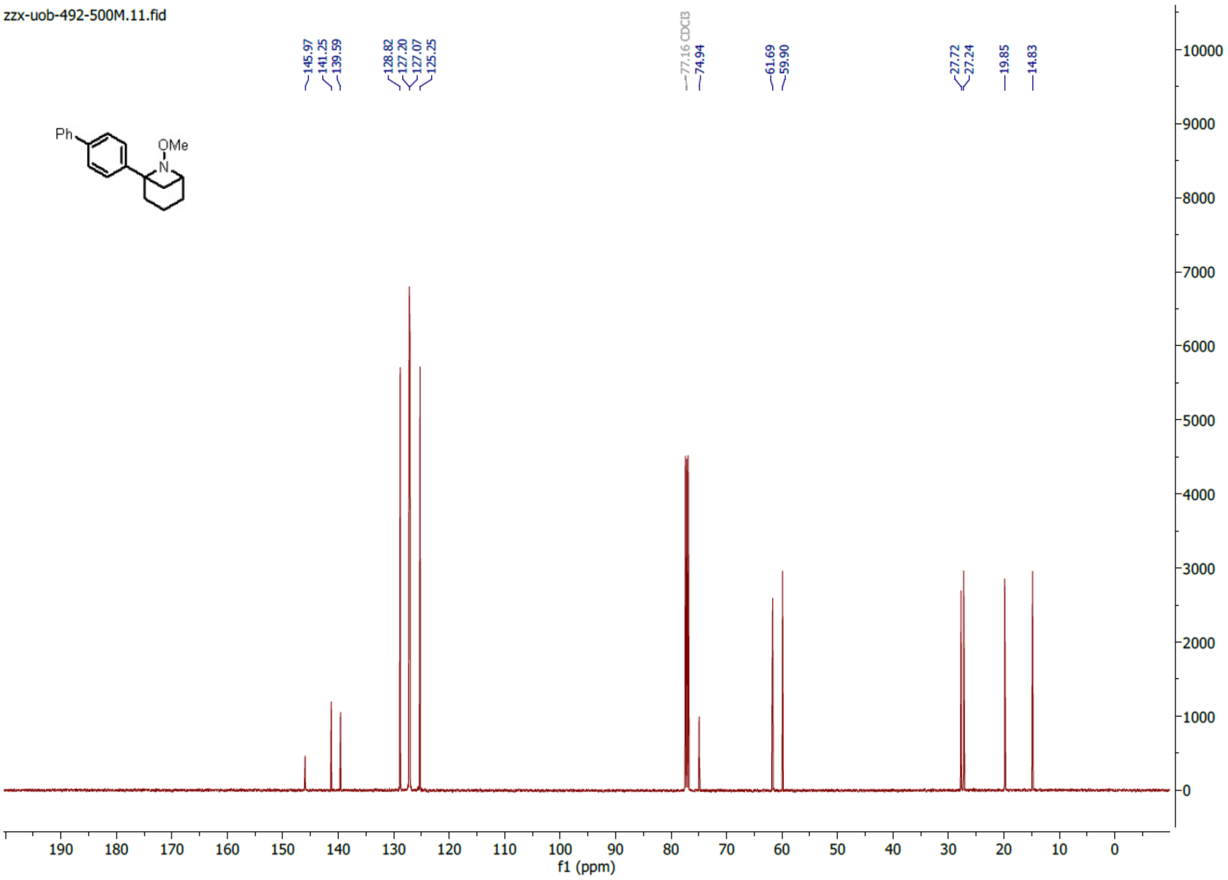

Compound 34

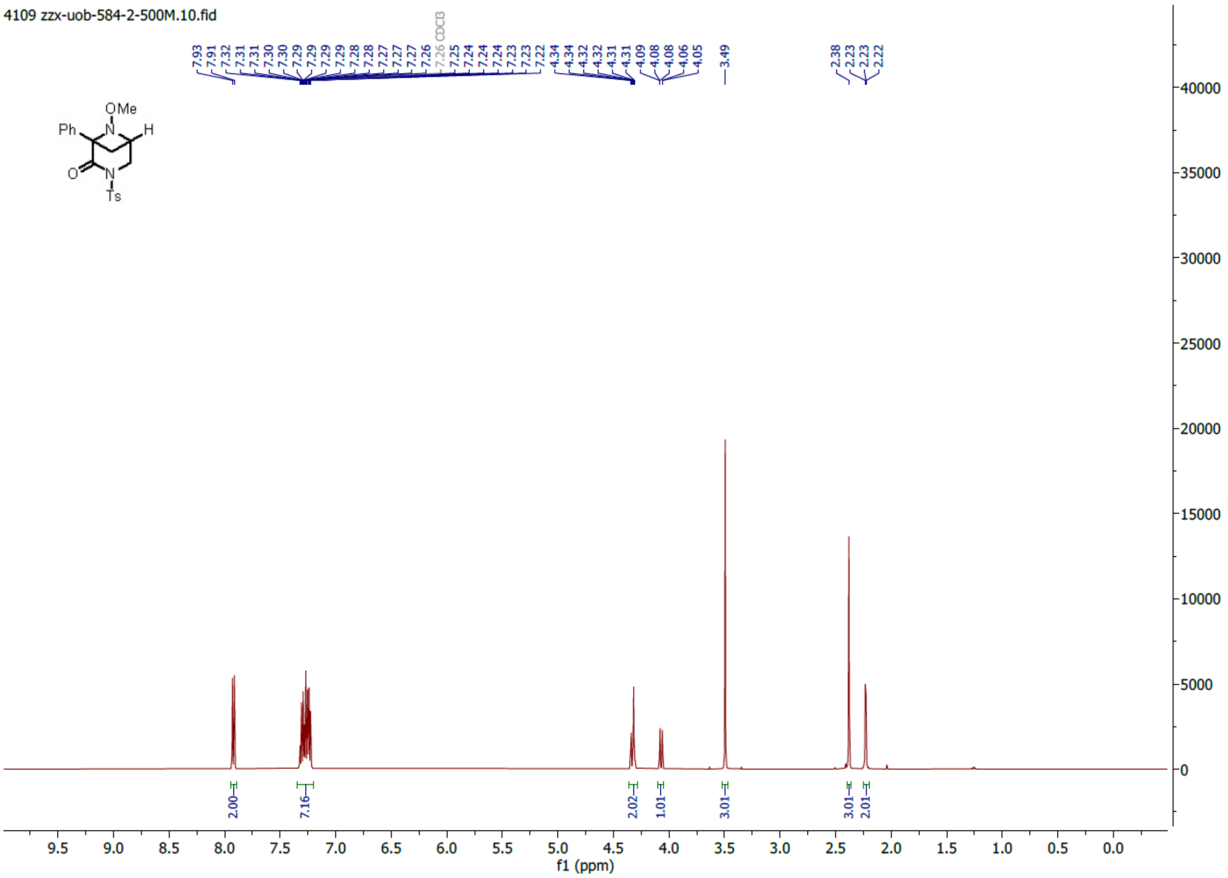
4109 zzx-uob-584-2-500M.10.fid
7.93 7.91 7.32 7.31 7.31 7.30 7.30 7.29 7.29 7.29 7.29 7.28 7.28 7.27 7.27 7.27 7.26 CDCl3 7.25 7.24 7.24 7.24 7.23 7.23 7.22 4.34 4.34 4.32 4.32 4.31 4.31 4.09 4.08 4.08 4.06 4.05 3.49 2.38 2.23 2.23 2.22
OMe
Ph
N
H
O
N
Ts
2.00 7.16 2.02 1.01 3.01 3.01 2.01
f1 (ppm)

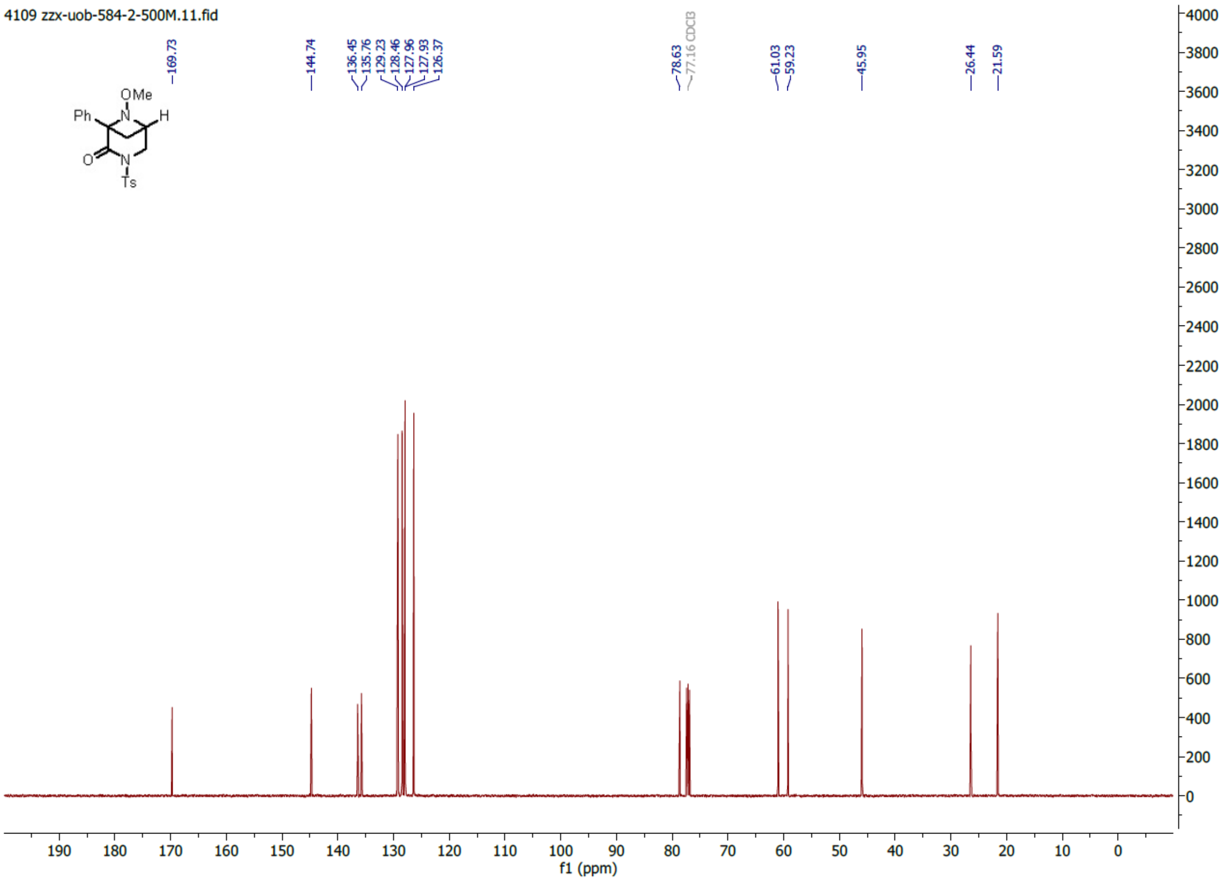
4109 zzx-uob-584-2-500M.11.fid
169.73 144.74 136.45 135.76 129.23 128.46 127.96 127.93 126.37 78.63 77.16 CDCl3 61.03 59.23 45.95 26.44 21.59
OMe
Ph
N
H
O
N
Ts
f1 (ppm)

Compound 35

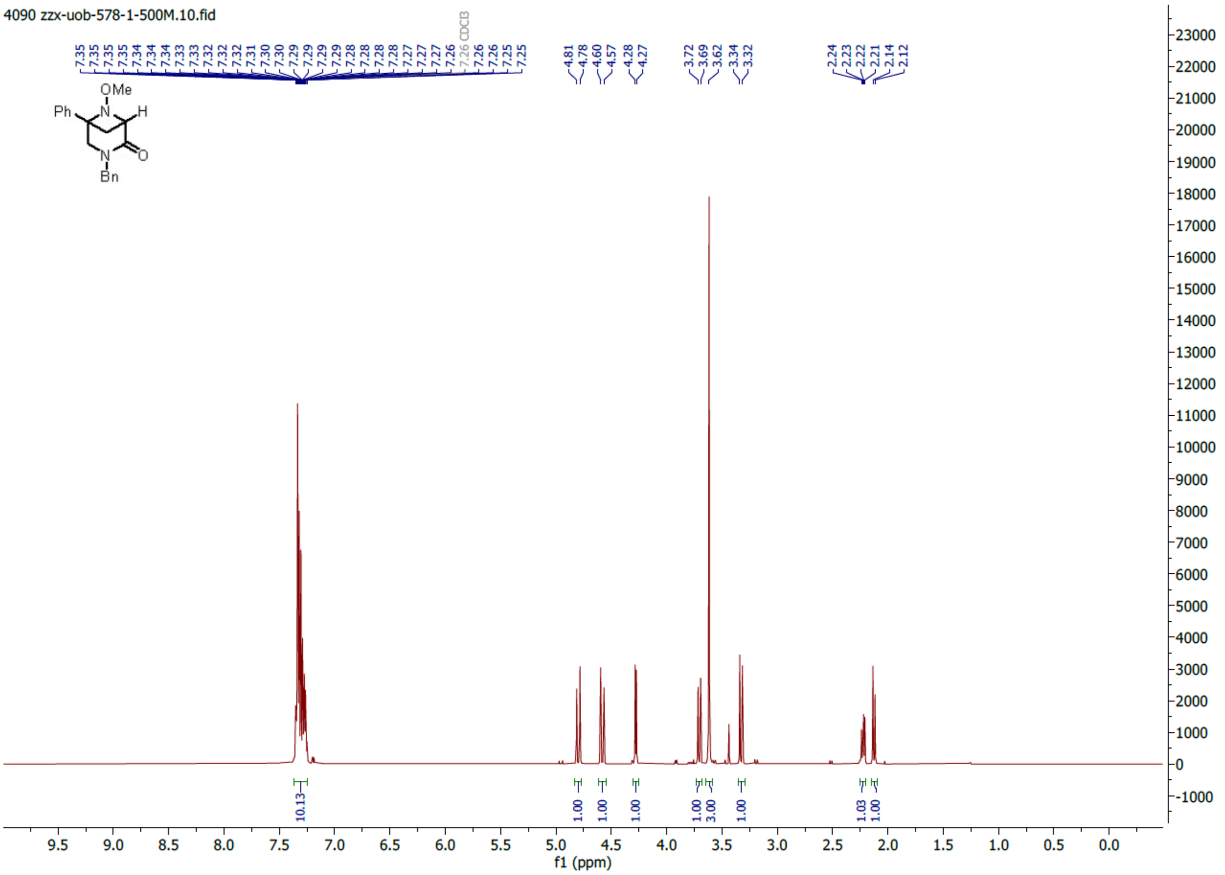

4090 zzx-uob-578-1-500M.10.fid
f1 (ppm)

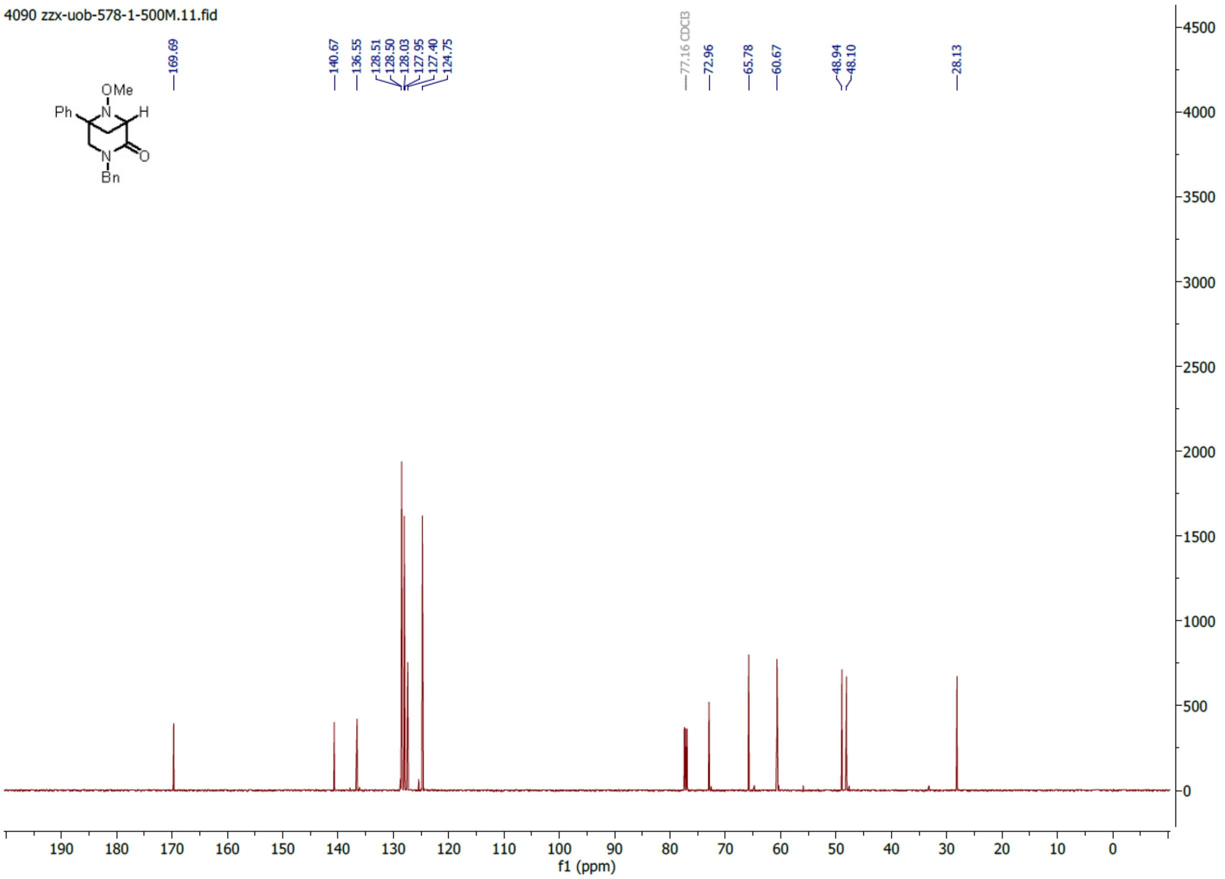

4090 zzx-uob-578-1-500M.11.fid
f1 (ppm)

Compound 36

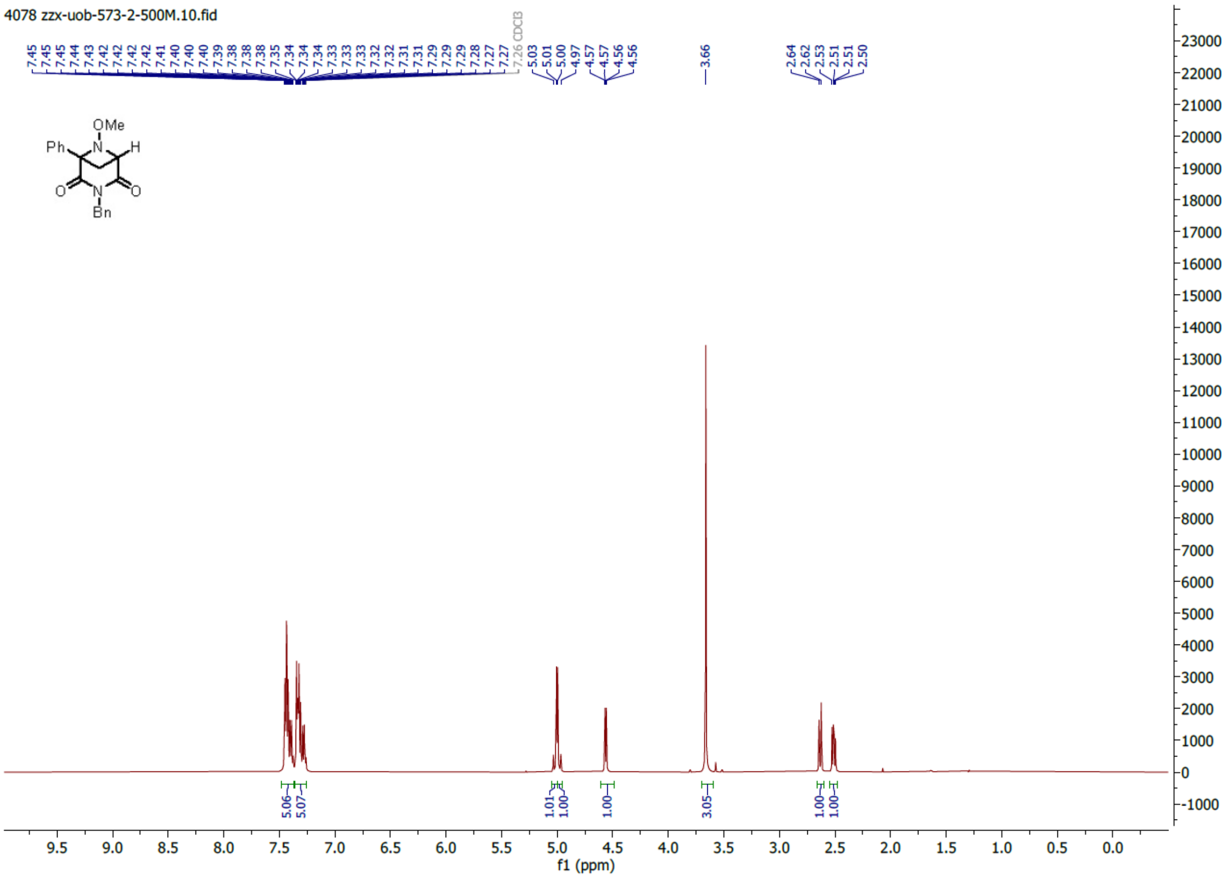

4078 zzx-uob-573-2-500M.10.fid
7.45
7.45
7.45
7.44
7.43
7.42
7.42
7.42
7.42
7.41
7.40
7.40
7.40
7.39
7.38
7.38
7.38
7.35
7.34
7.34
7.34
7.33
7.33
7.33
7.32
7.32
7.31
7.31
7.29
7.29
7.29
7.28
7.27
7.27
7.26 CDCl3
5.03
5.01
5.00
4.97
4.57
4.57
4.56
4.56
3.66
2.64
2.62
2.53
2.51
2.51
2.50
OMe
N
Ph
H
O
N
O
Bn
5.06
5.07
1.01
1.00
1.00
3.05
1.00
1.00
f1 (ppm)

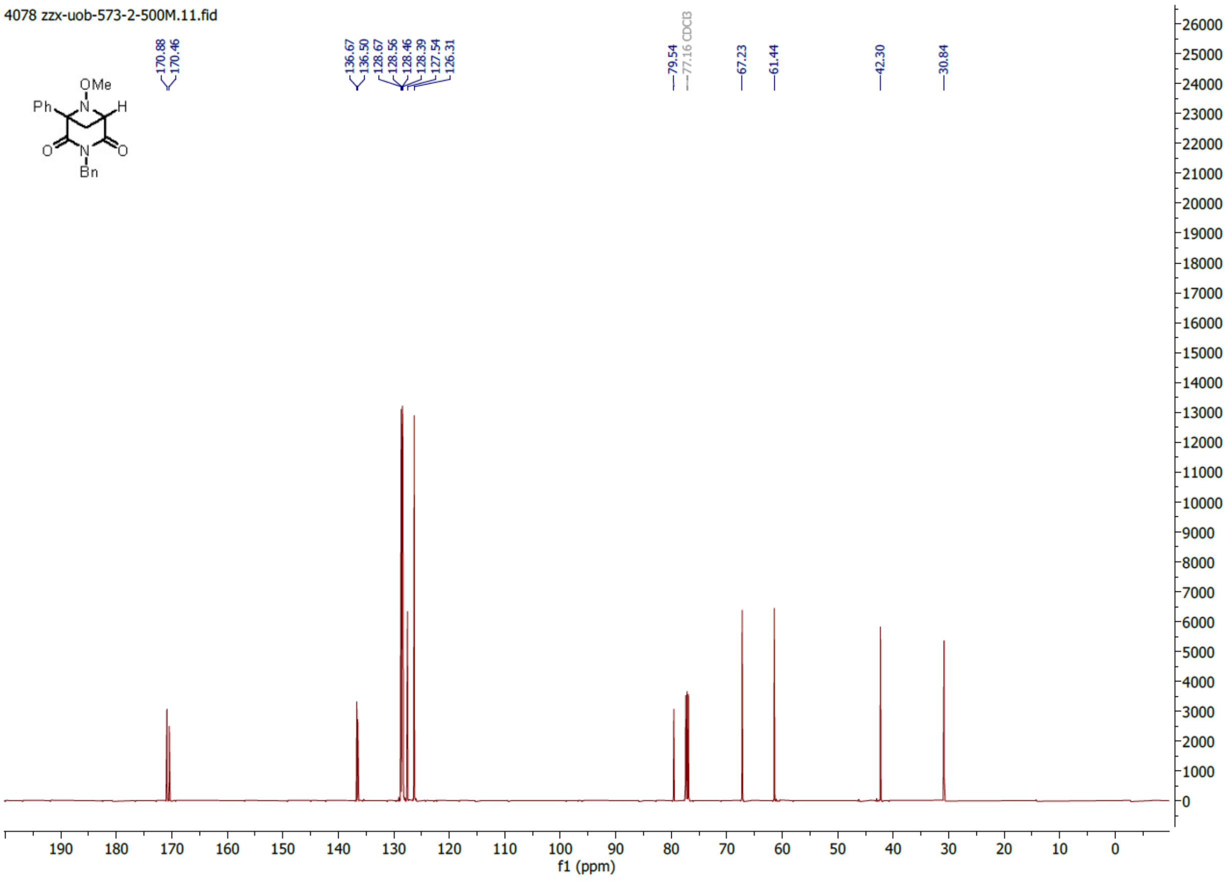

4078 zzx-uob-573-2-500M.11.fid
170.88
170.46
136.67
136.50
128.67
128.56
128.46
128.39
127.54
126.31
79.54
77.16 CDCl3
67.23
61.44
42.30
30.84
OMe
N
Ph
H
O
N
O
Bn
f1 (ppm)

Compound 37

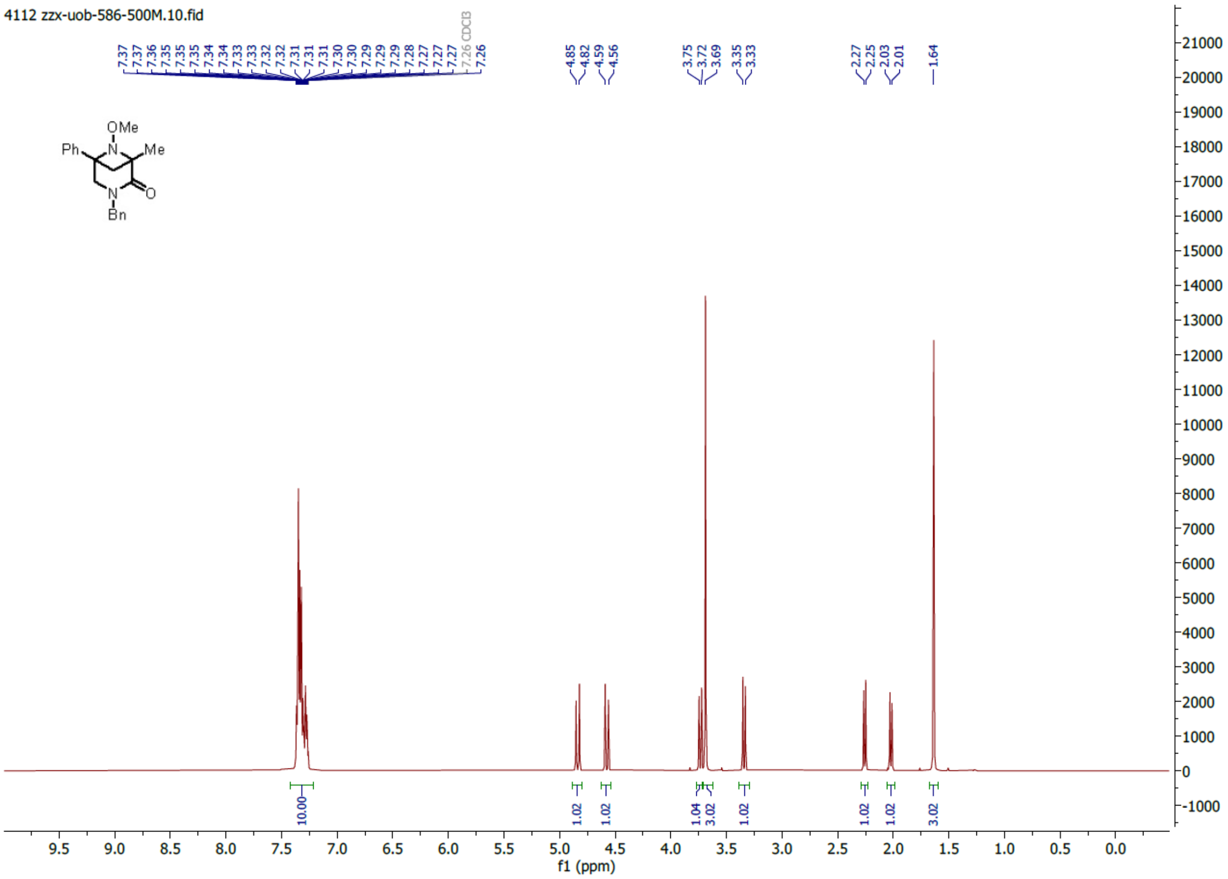
4112 zzx-uob-586-500M.10.fid
7.37 7.37 7.36 7.35 7.35 7.35 7.34 7.34 7.33 7.33 7.32 7.32 7.31 7.31 7.31 7.30 7.30 7.29 7.29 7.29 7.28 7.27 7.27 7.27 7.26 CDCl3 7.26
4.85 4.82 4.59 4.56
3.75 3.72 3.69 3.35 3.33
2.27 2.25 2.03 2.01
1.64
OMe
N
Ph
Me
N
O
Bn
10.00
1.02
1.02
1.04
3.02
1.02
1.02
1.02
3.02
f1 (ppm)

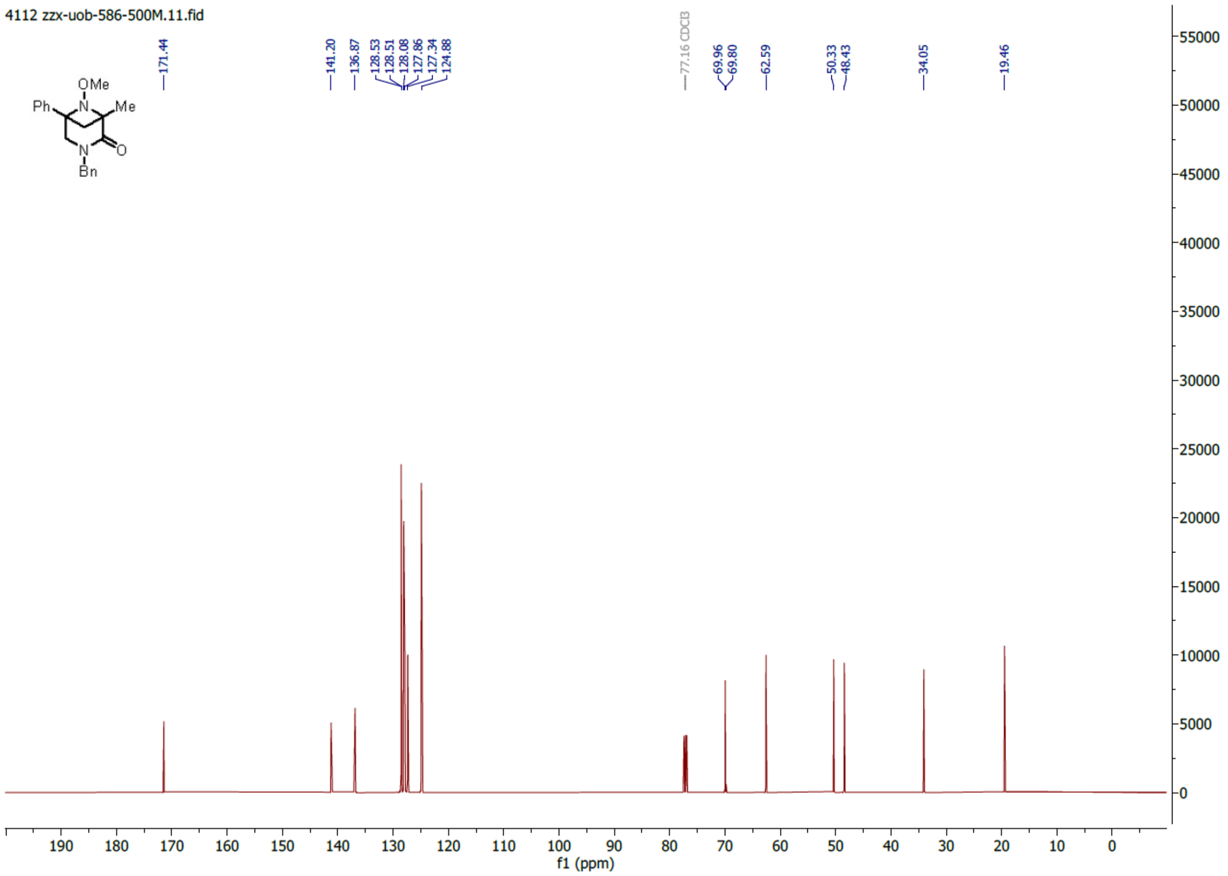
4112 zzx-uob-586-500M.11.fid
171.44
141.20 136.87 128.53 128.51 128.08 127.86 127.34 124.88
77.16 CDCl3
69.96 69.80
62.59
50.33 48.43
34.05
19.46
OMe
N
Ph
Me
N
O
Bn
f1 (ppm)

Compound 38

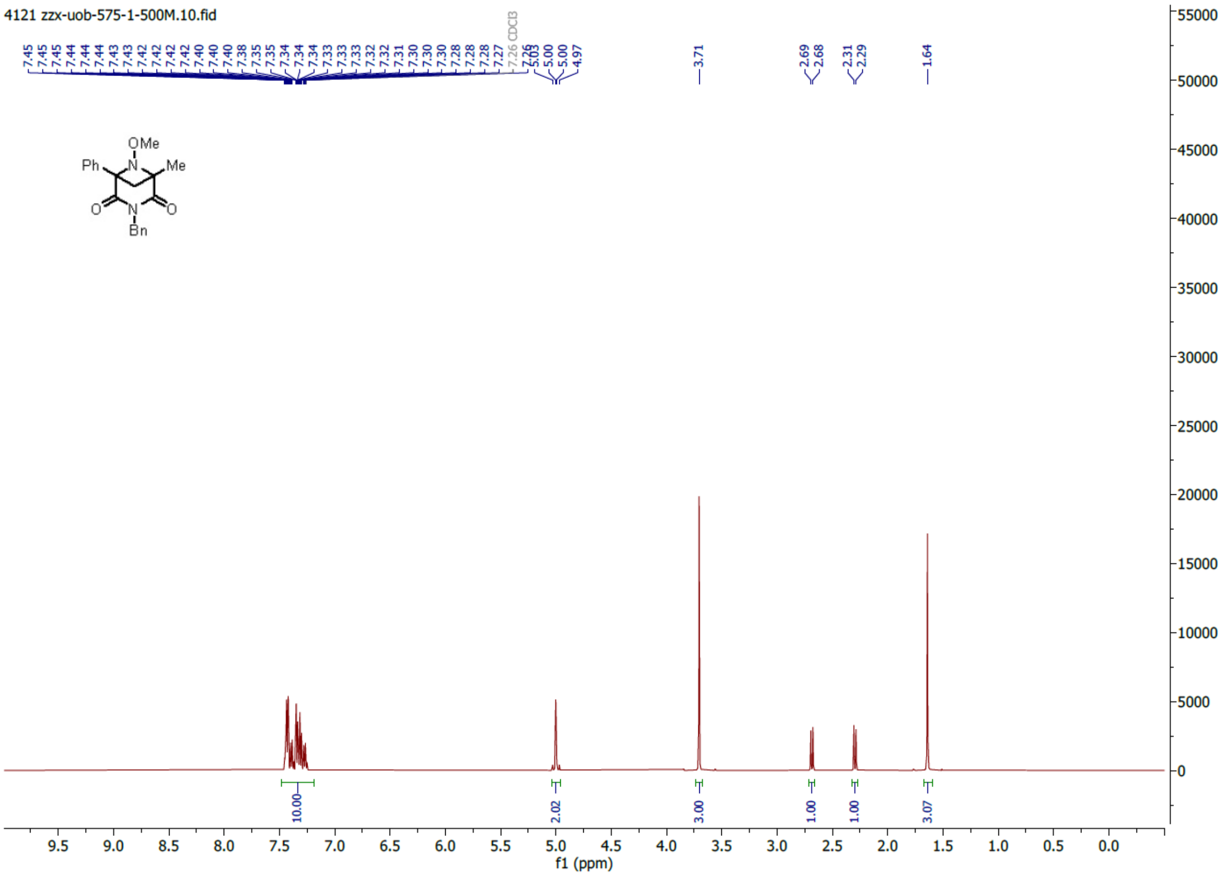

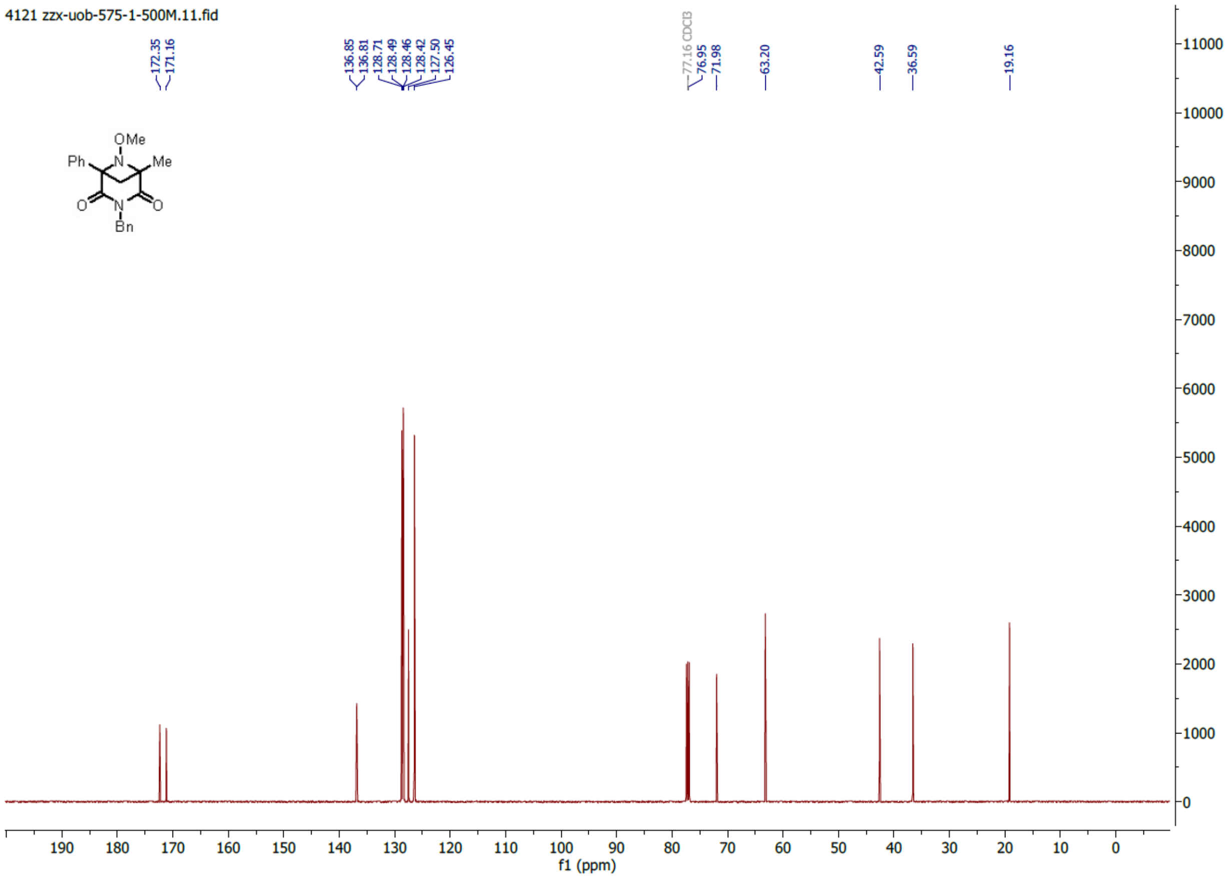

Compound 38a

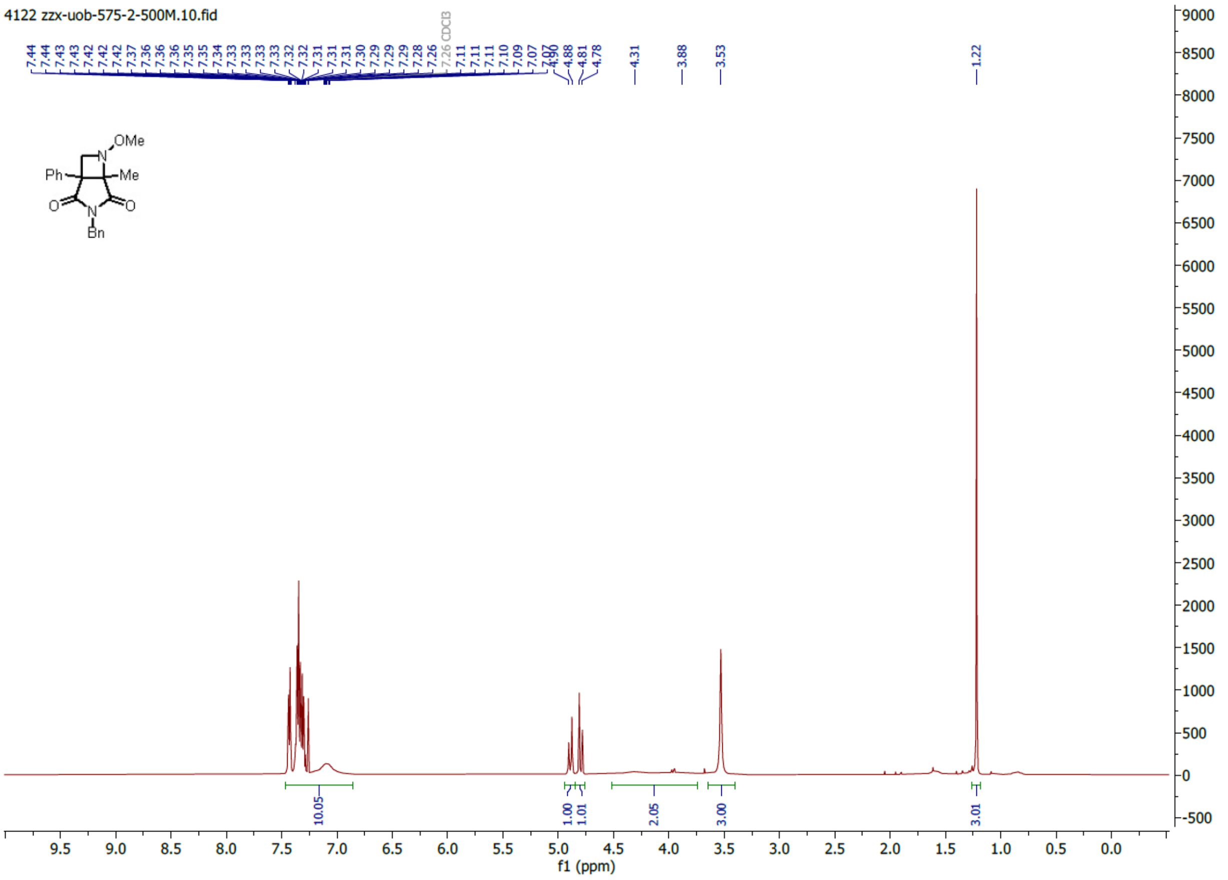

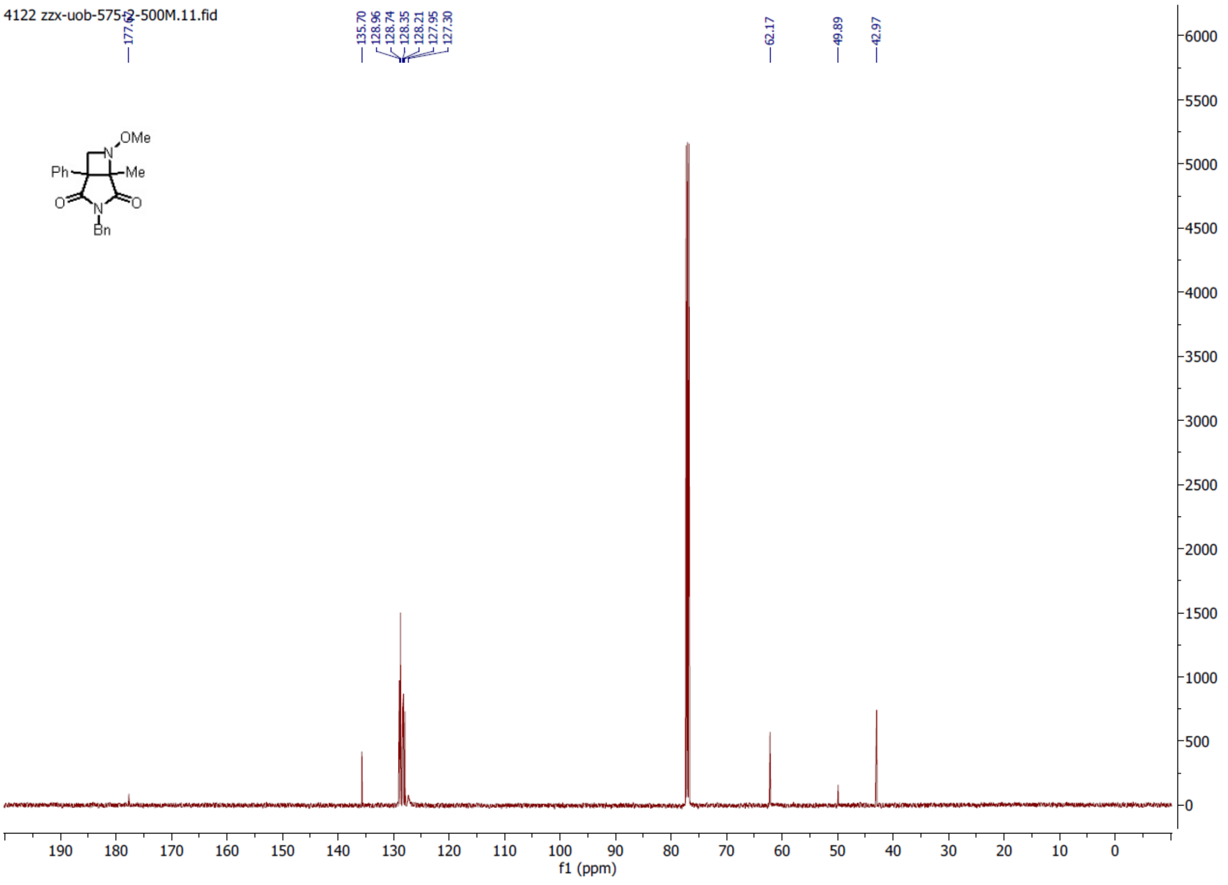

Compound 39

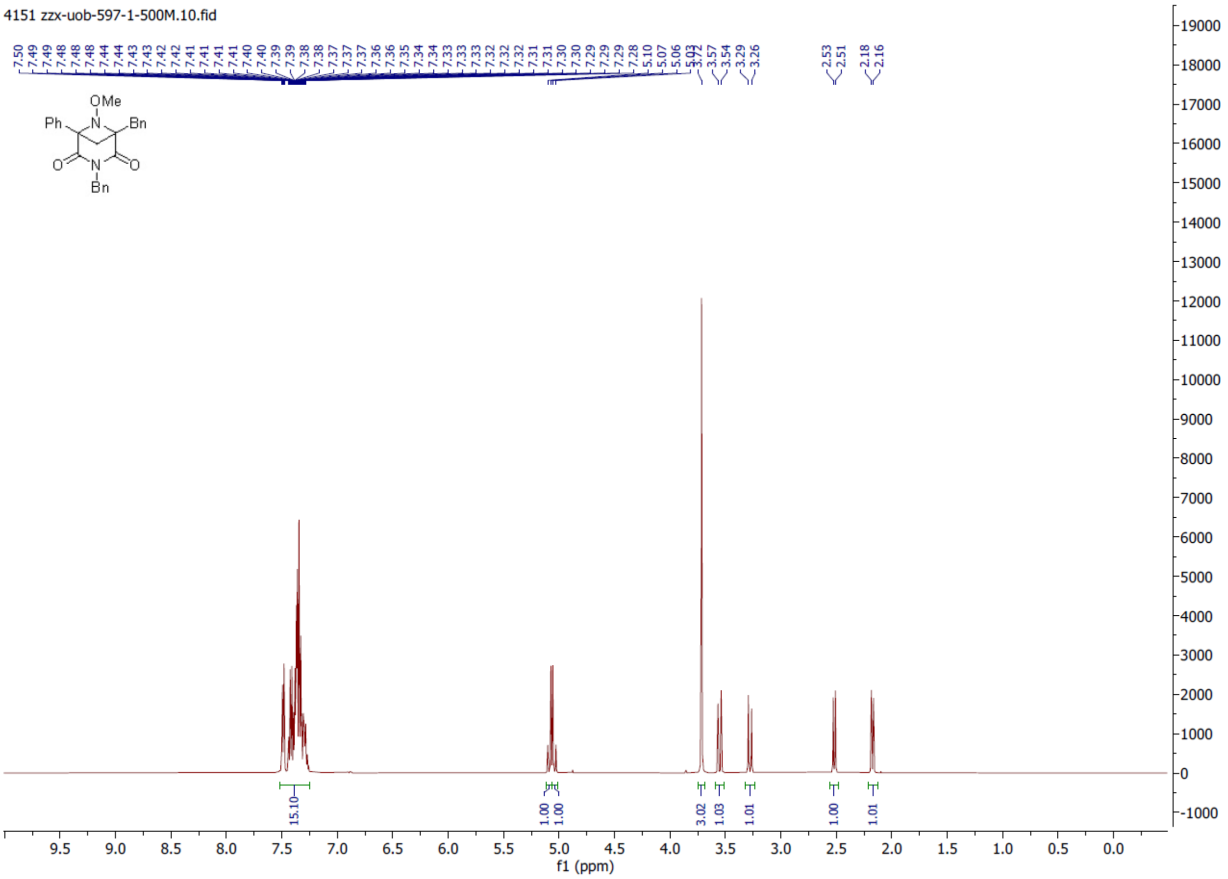

4151 zzx-uob-597-1-500M.10.fid
OMe
Ph
Bn
O
N
O
Bn
f1 (ppm)

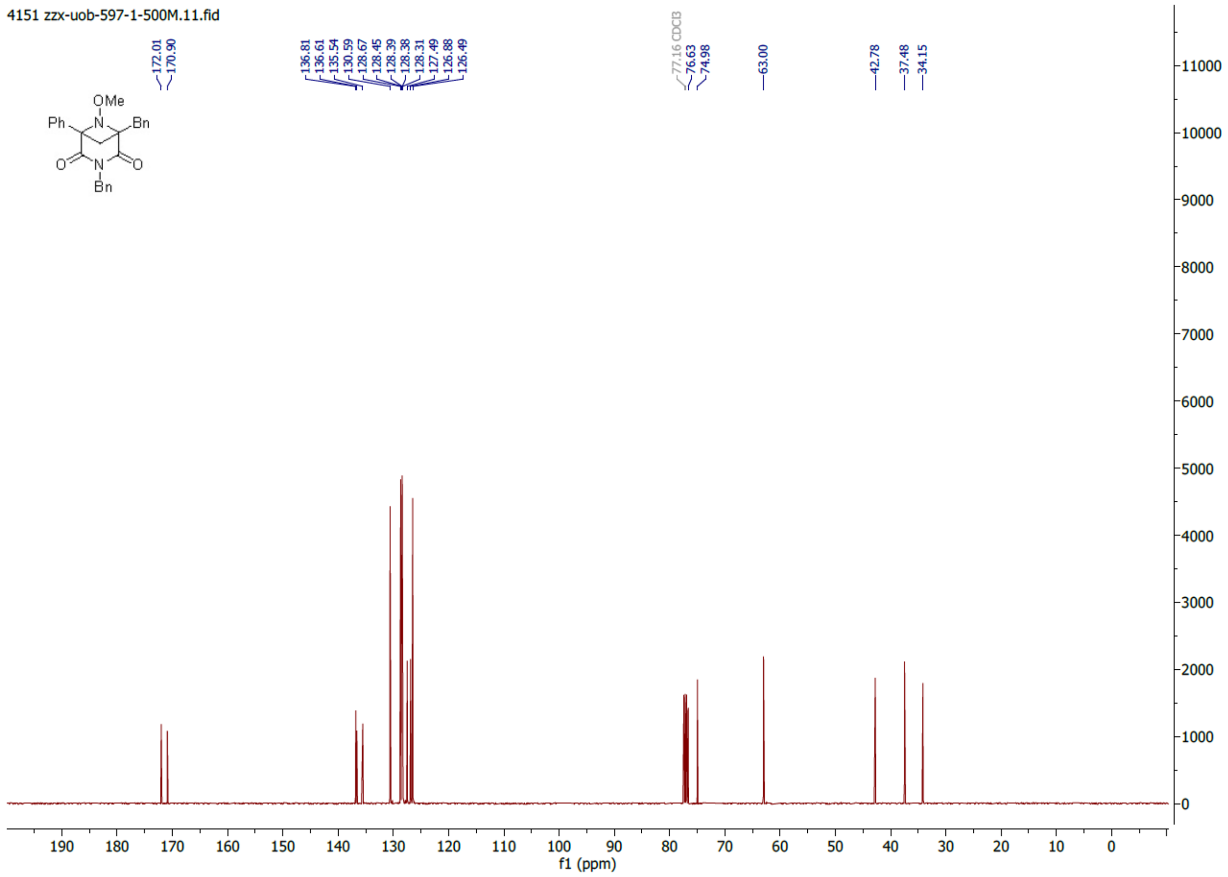

4151 zzx-uob-597-1-500M.11.fid
172.01
170.90
136.81
136.61
135.54
130.59
128.67
128.45
128.39
128.38
128.31
127.49
126.88
126.49
77.16 CDCl3
76.63
74.98
63.00
42.78
37.48
34.15
f1 (ppm)

Compound 39a

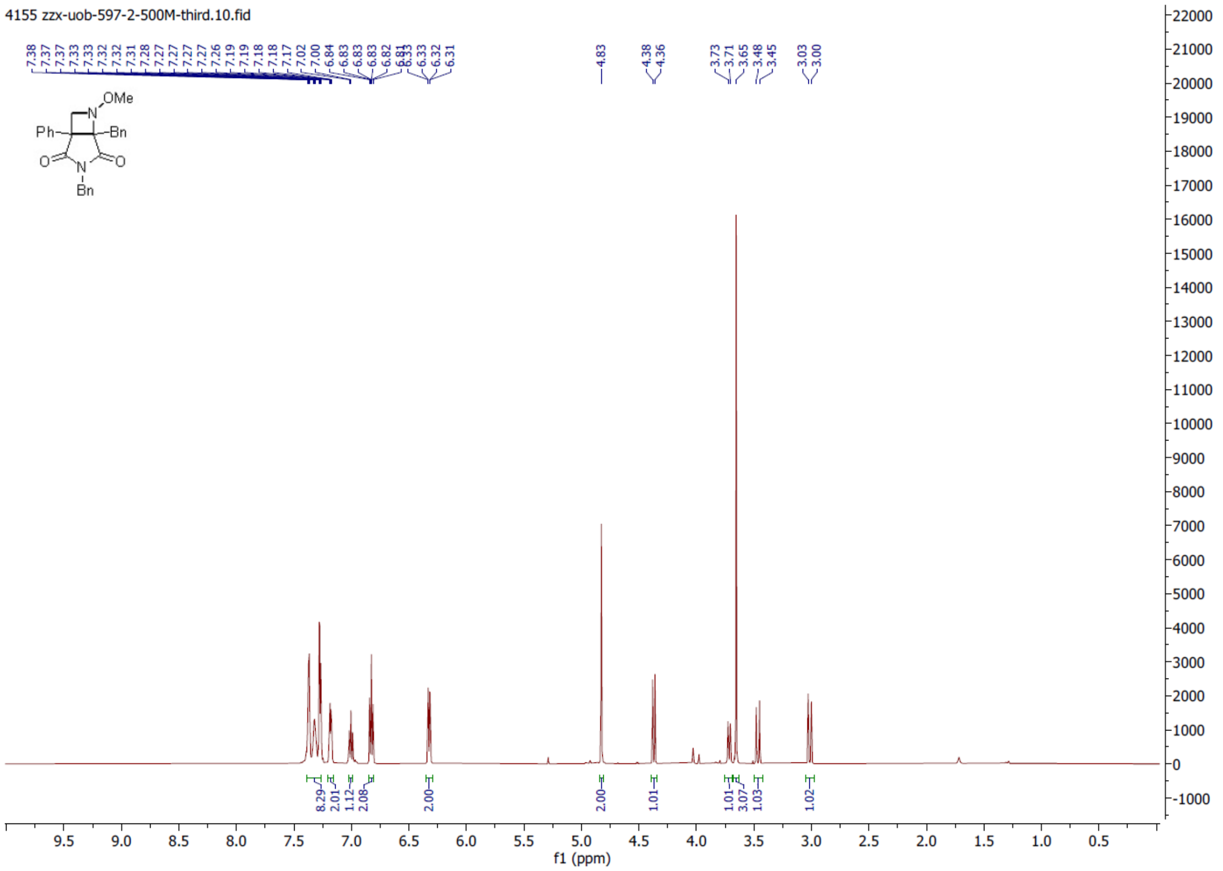

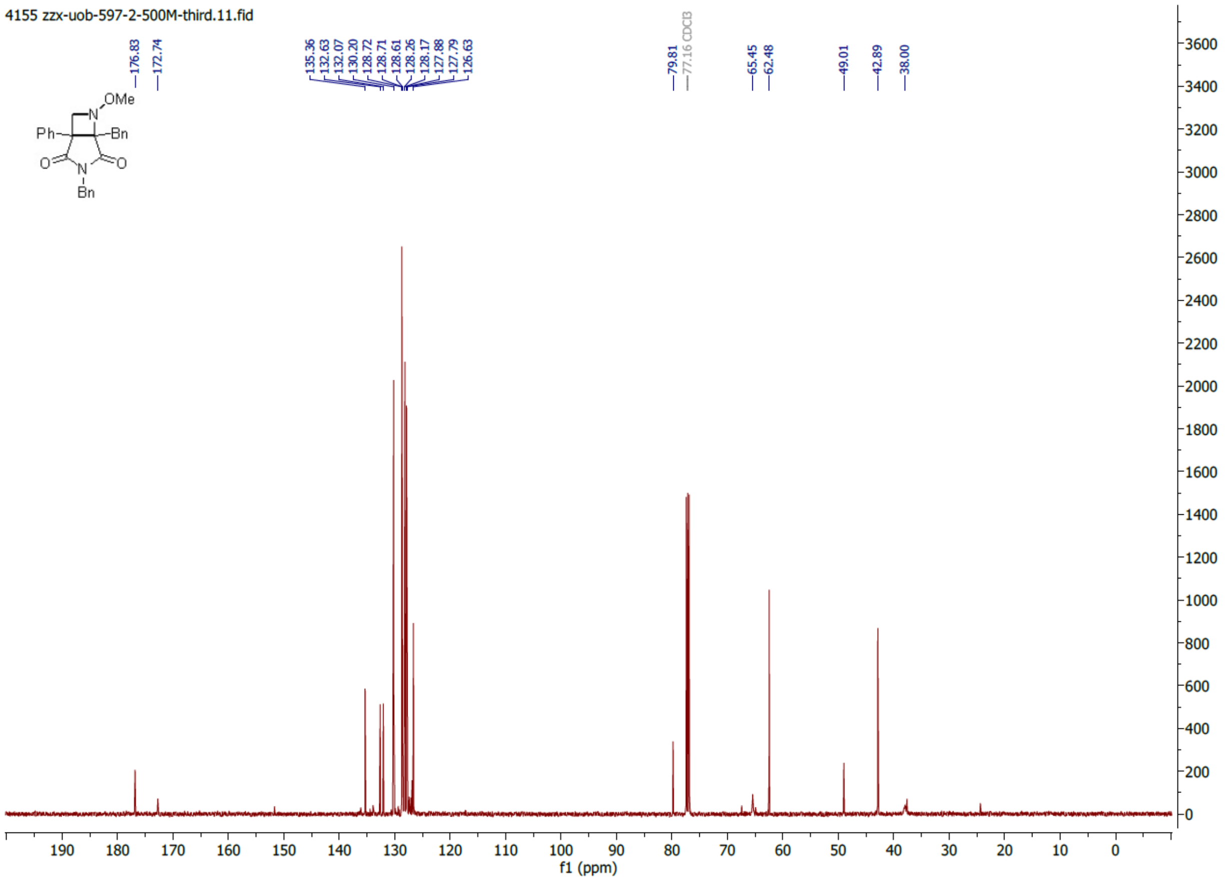

Compound 40

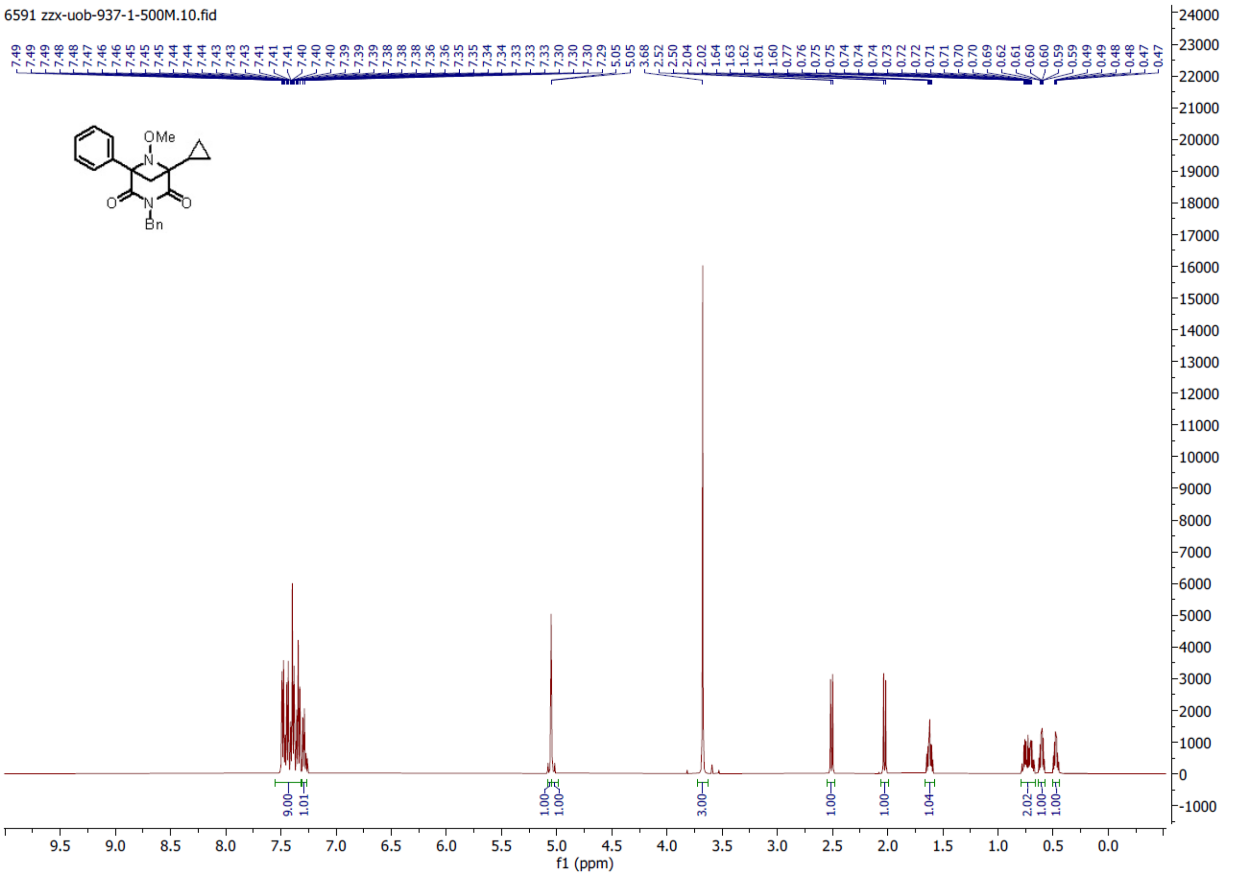

6591 zzx-uob-937-1-500M.10.fid
f1 (ppm)

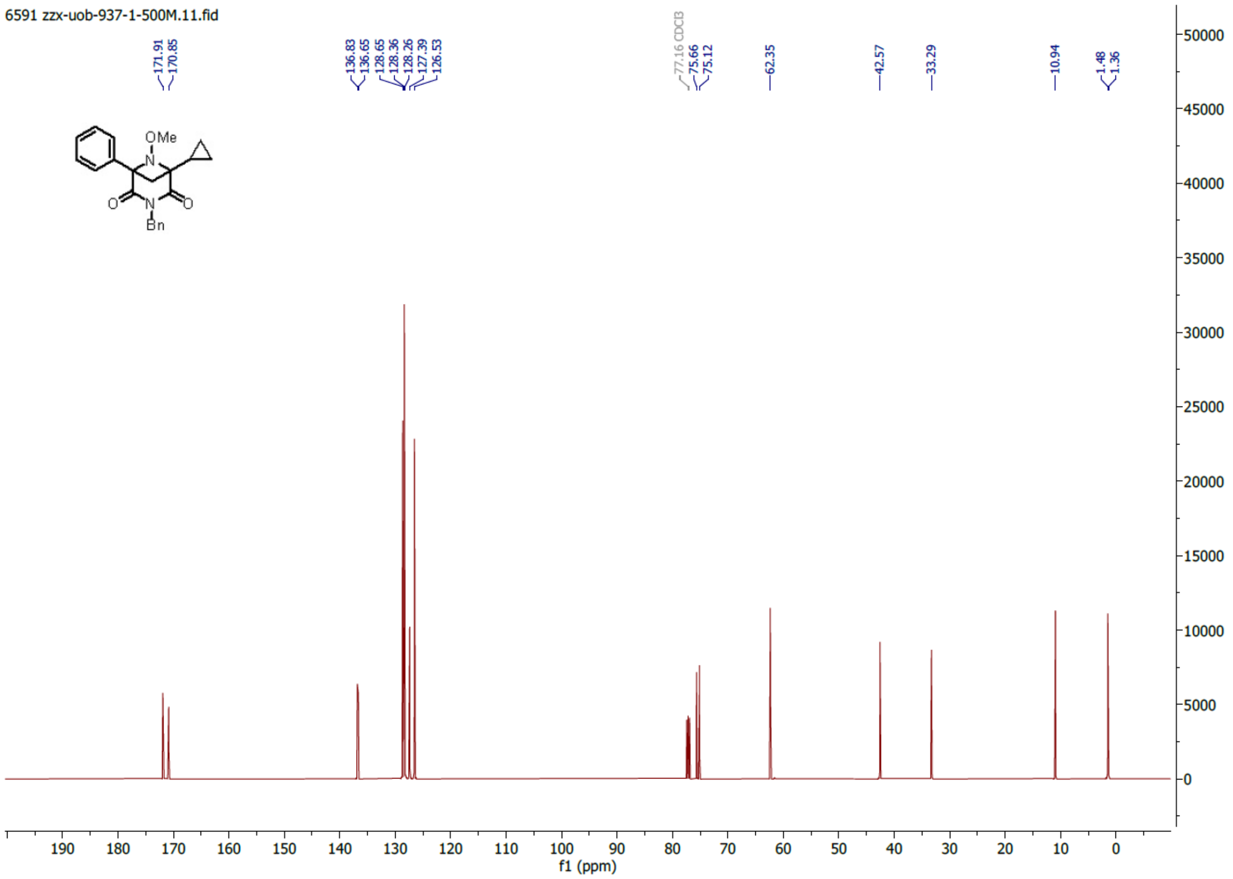

6591 zzx-uob-937-1-500M.11.fid
f1 (ppm)

Compound 40a

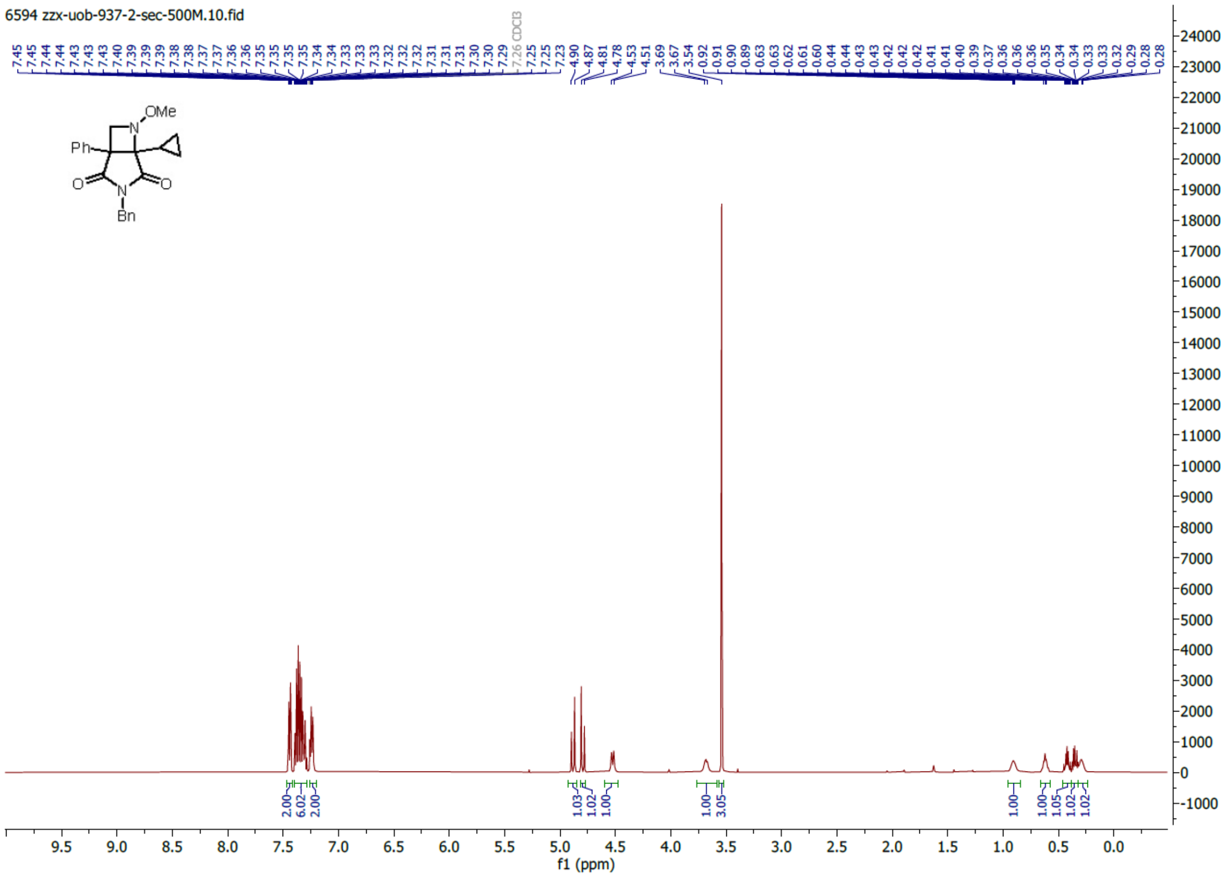

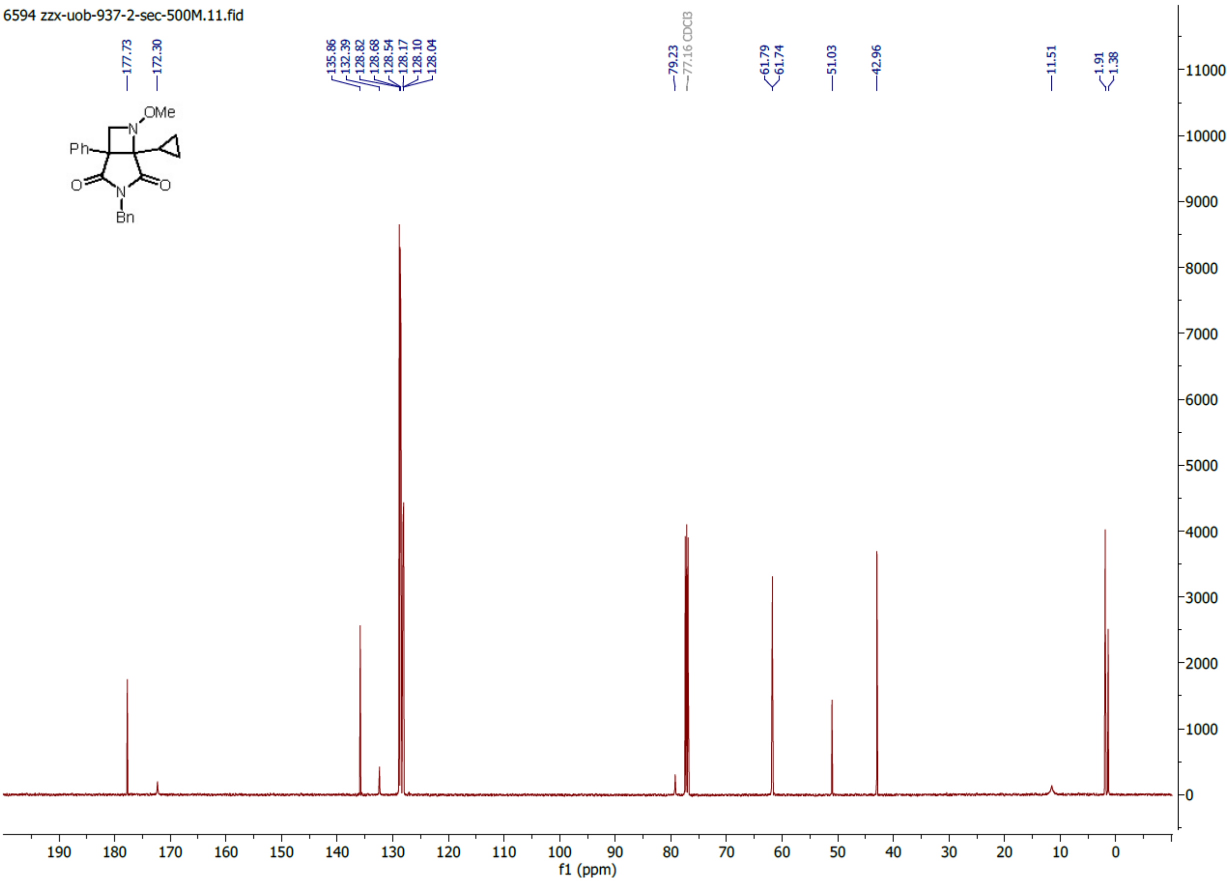

Compound 41

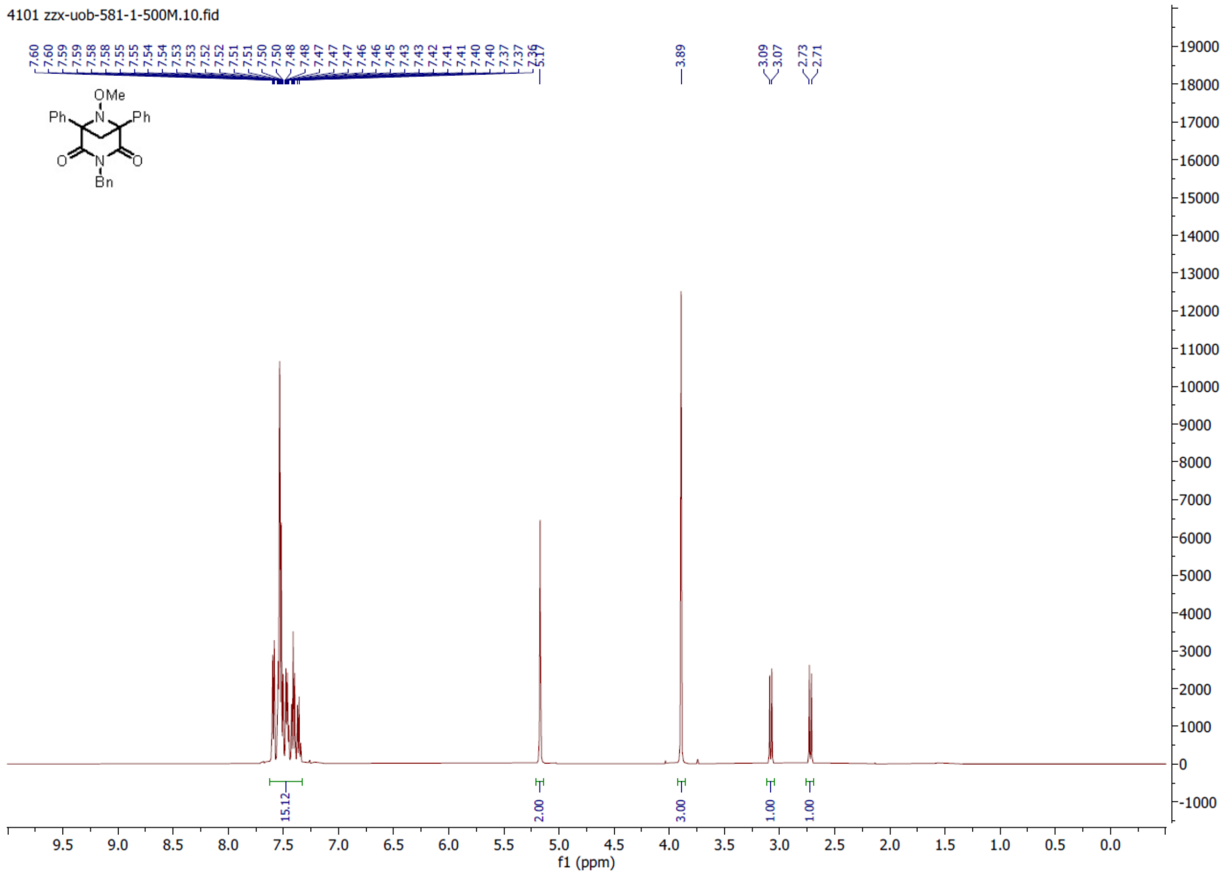
4101 zzx-uob-581-1-500M.10.fid
7.60 7.60 7.59 7.59 7.58 7.58 7.55 7.55 7.54 7.54 7.53 7.53 7.52 7.52 7.51 7.51 7.50 7.50 7.48 7.48 7.47 7.47 7.47 7.46 7.46 7.45 7.43 7.43 7.42 7.41 7.41 7.40 7.40 7.37 7.37 7.36 5.17
3.89
3.09 3.07
2.73 2.71
OMe
Ph
N
Ph
O
N
O
Bn
15.12
2.00
3.00
1.00
1.00
f1 (ppm)

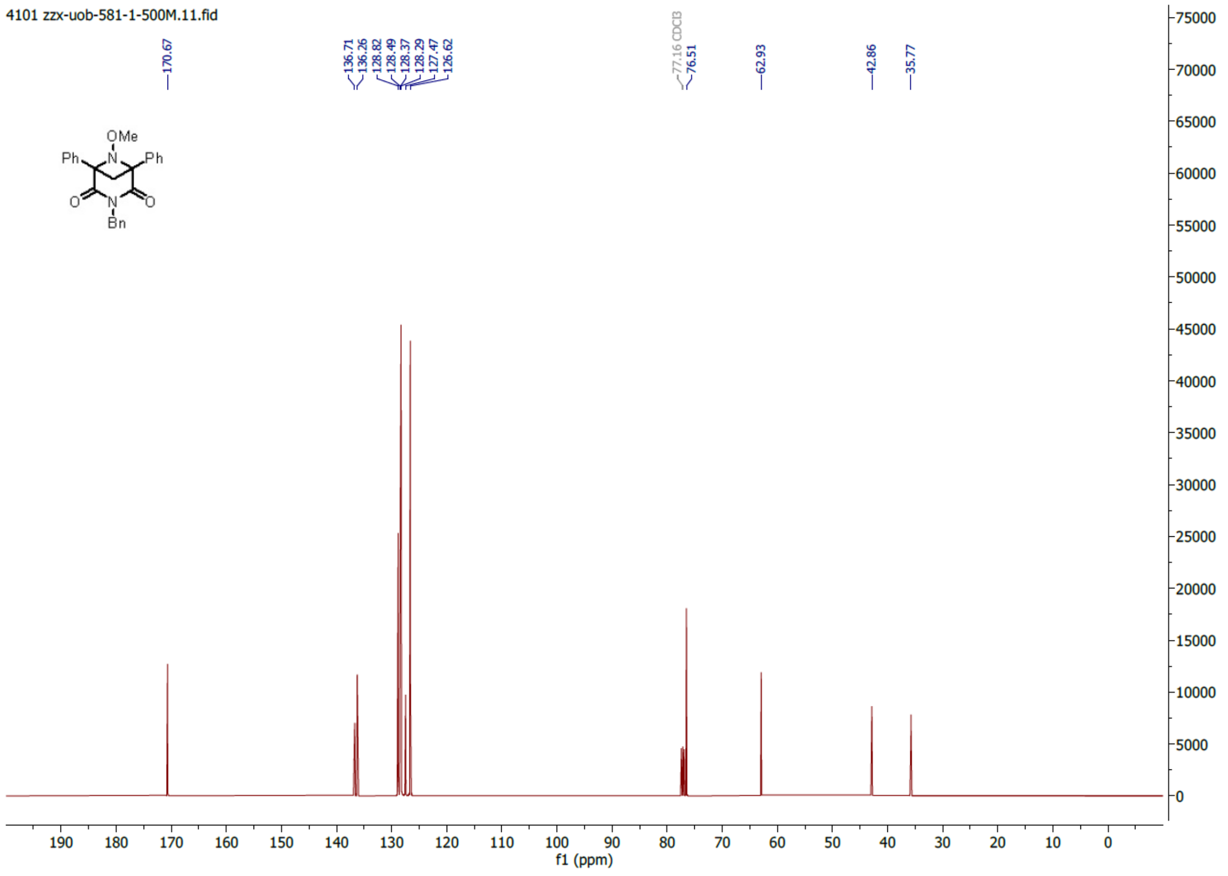
4101 zzx-uob-581-1-500M.11.fid
170.67
136.71 136.26 128.82 128.49 128.37 128.29 127.47 126.62
77.16 CDCl3
76.51
62.93
42.86
35.77
OMe
Ph
N
Ph
O
N
O
Bn
f1 (ppm)

Compound 41a

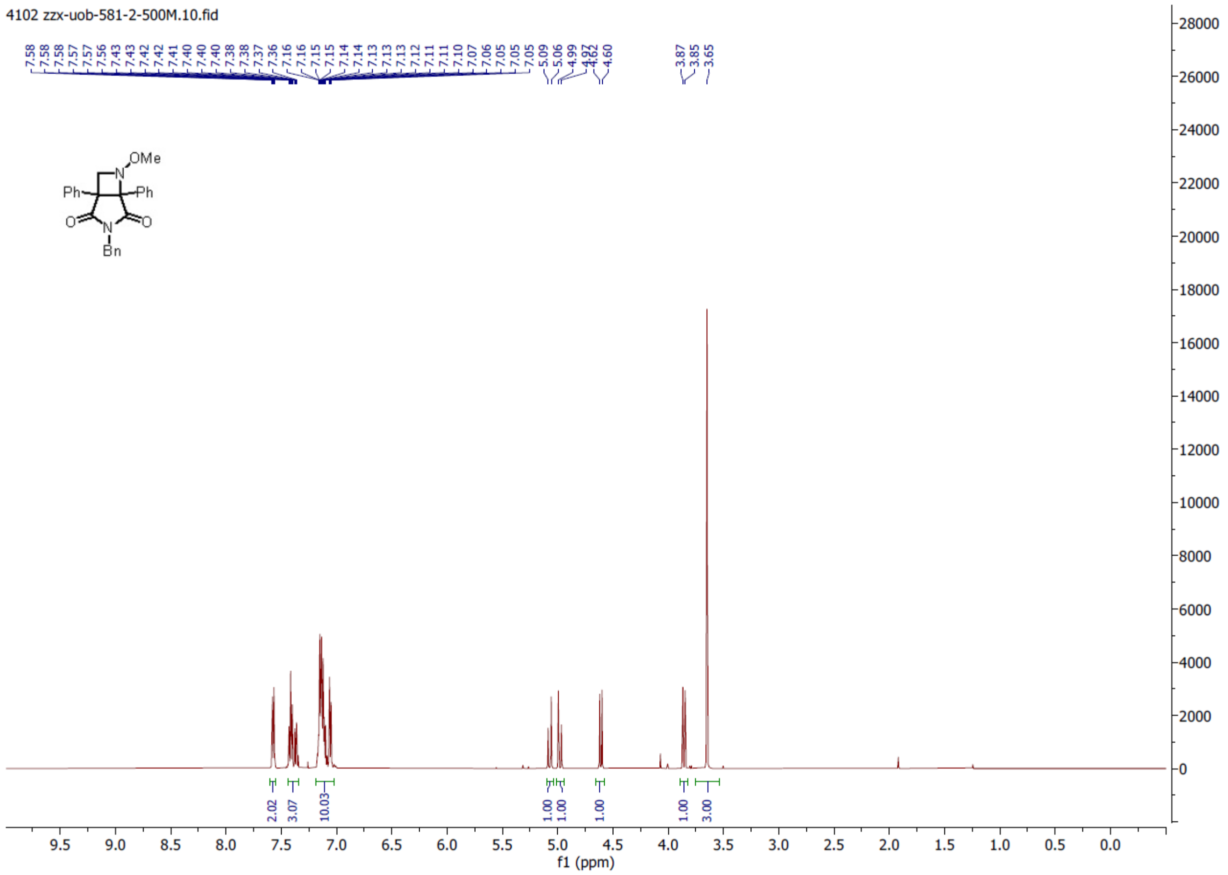

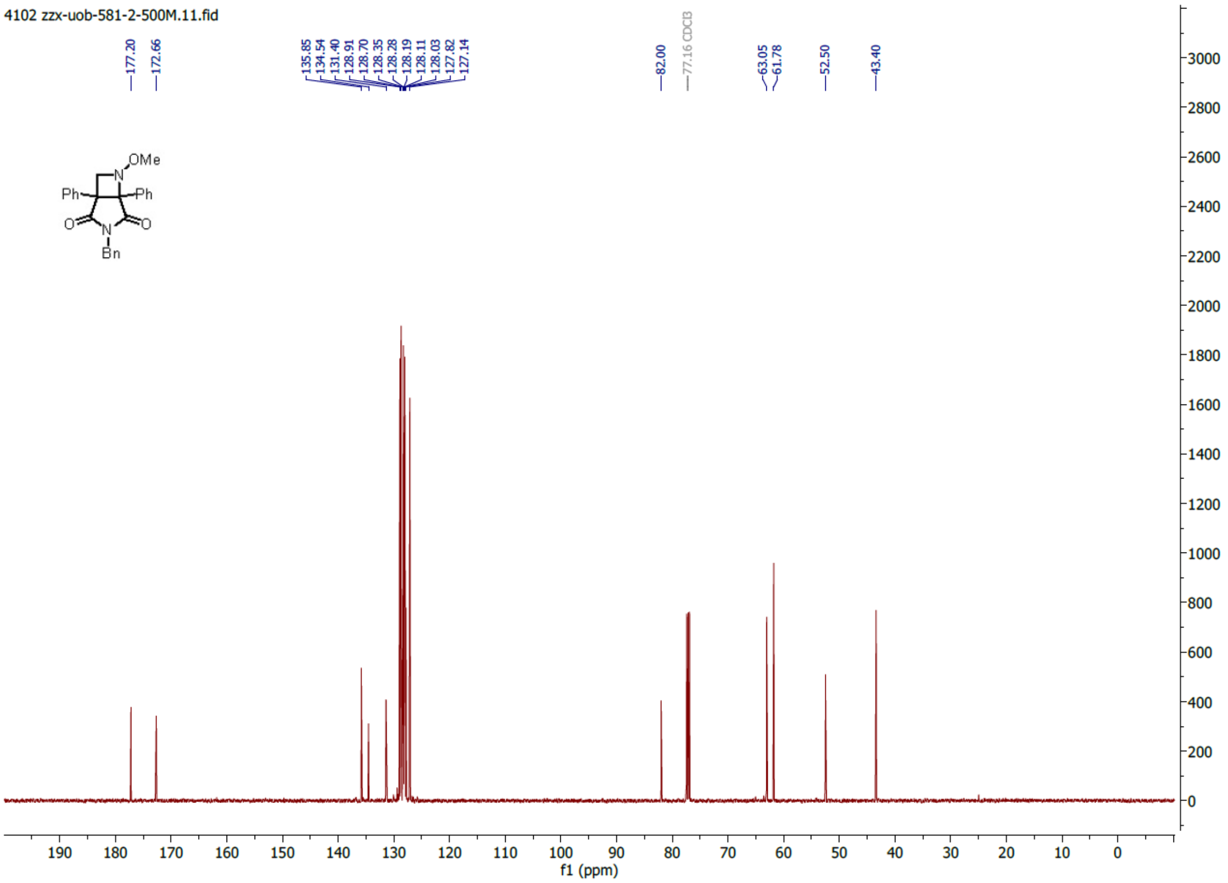

Compound 42

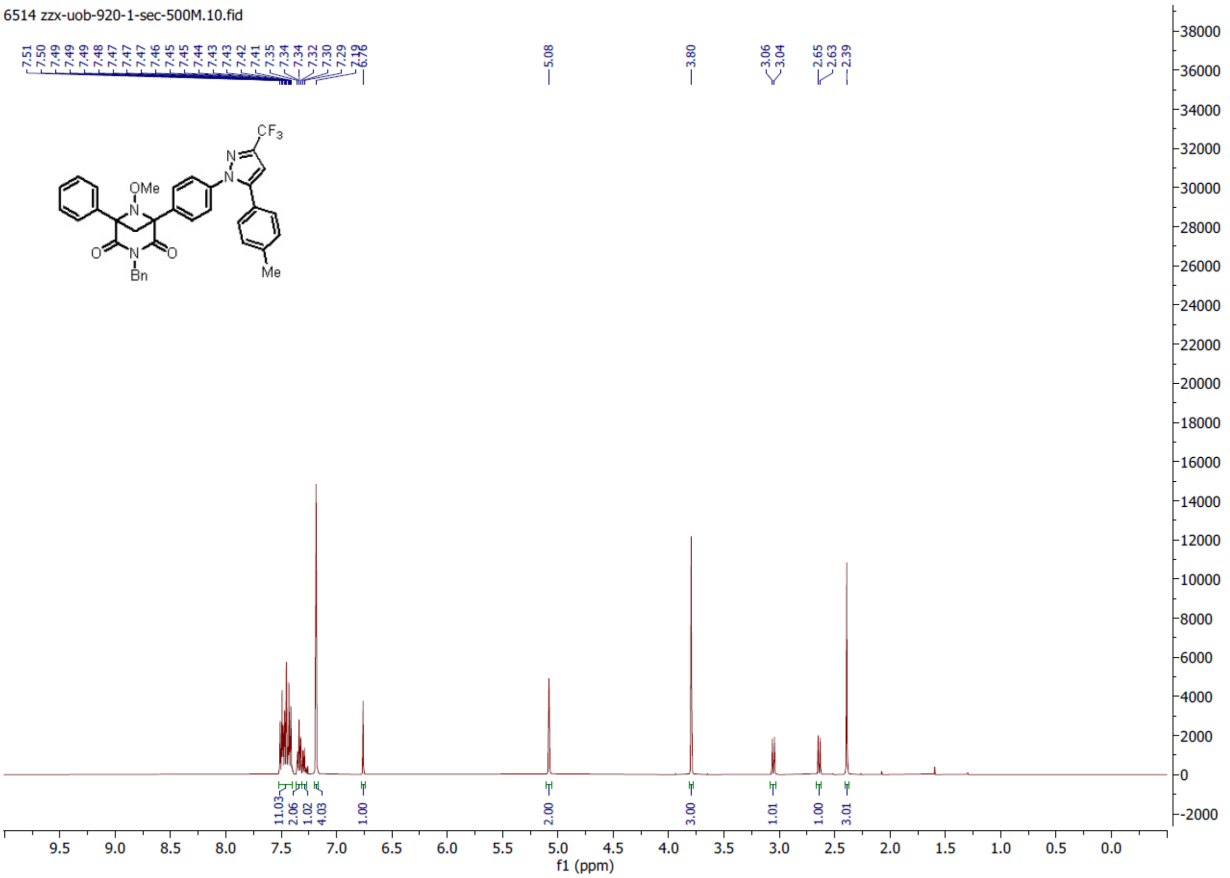

6514 zzx-uob-920-1-sec-500M.10.fid
7.51 7.50 7.49 7.49 7.49 7.48 7.47 7.47 7.46 7.45 7.45 7.44 7.43 7.43 7.42 7.41 7.35 7.34 7.34 7.32 7.30 7.29 7.19 6.76
5.08
3.80
3.06 3.04
2.65 2.63 2.39
CF3
N
N
OMe
N
O
N
O
Bn
Me
11.03 2.06 1.02 4.03 1.00 2.00 3.00 1.01 1.00 3.01
f1 (ppm)

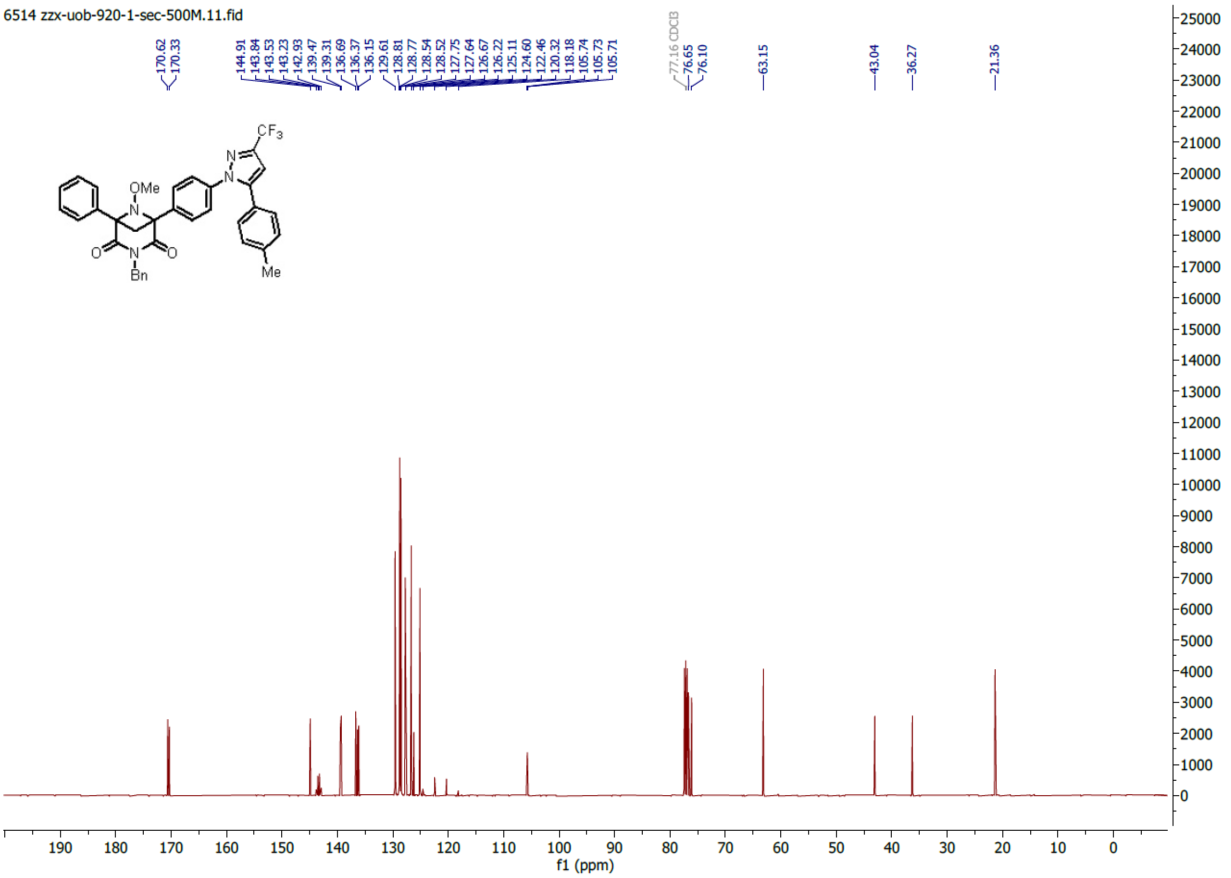

6514 zzx-uob-920-1-sec-500M.11.fid
170.62 170.33
144.91 143.84 143.53 143.23 142.93 139.47 139.31 136.69 136.37 136.15 129.61 128.81 128.77 128.54 128.52 127.75 127.64 126.67 126.22 125.11 124.60 122.46 120.32 118.18 105.74 105.73 105.71
77.16 CDCl3 76.65 76.10
63.15
43.04
36.27
21.36
CF3
N
N
OMe
N
O
N
O
Bn
Me
f1 (ppm)

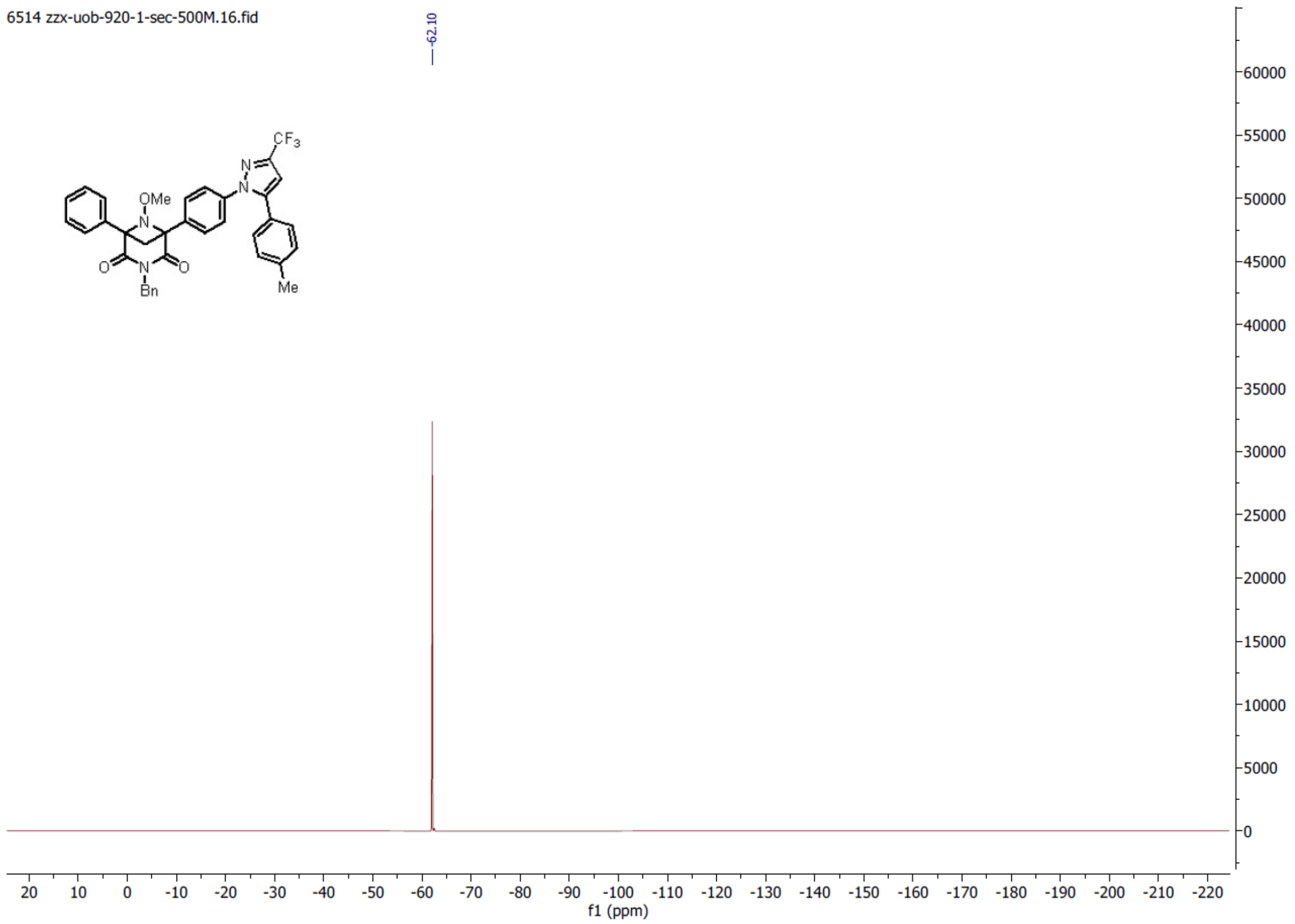
6514 zzx-uob-920-1-sec-500M.16.fid
-62.10
CF3
OMe
Bn
Me
f1 (ppm)

Compound 43

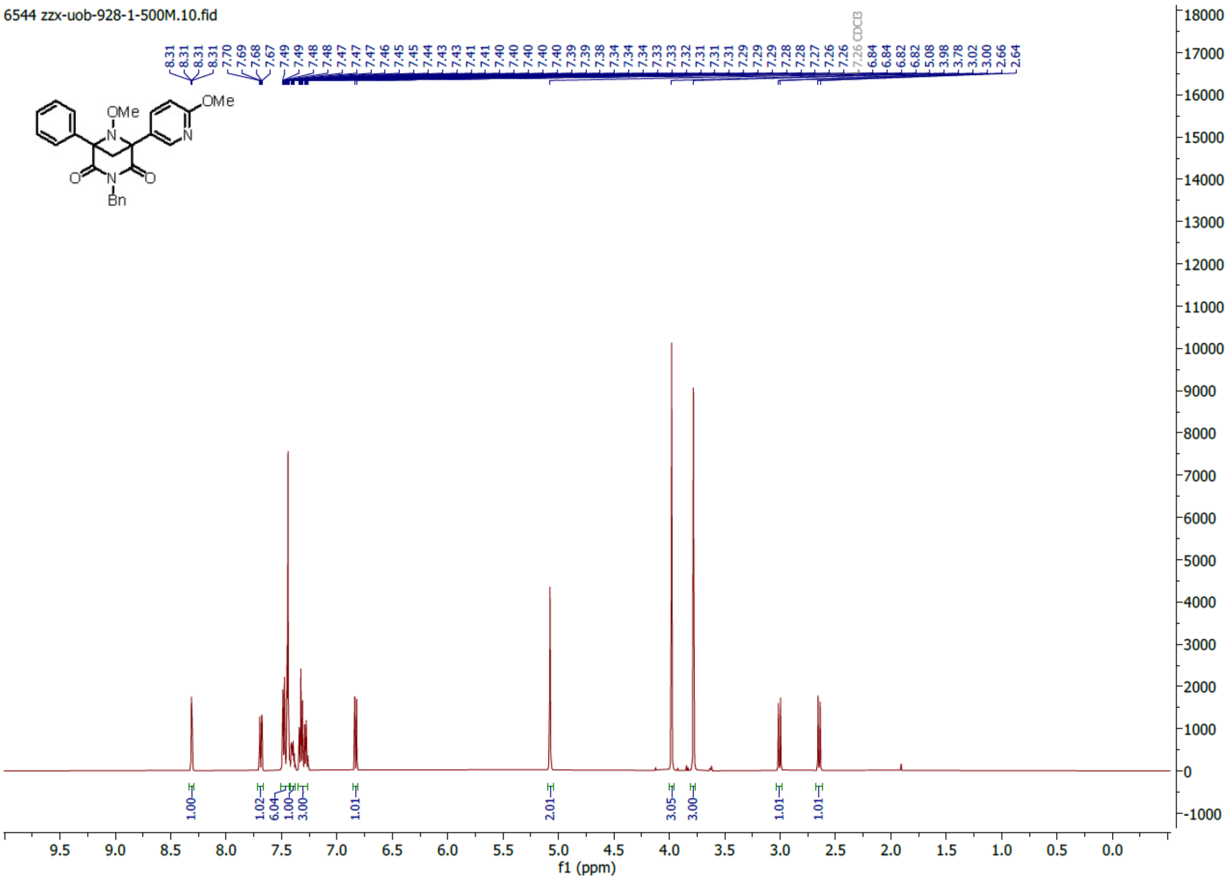
6544 zzx-uob-928-1-500M.10.fid
OMe
OMe
N
N
O
O
N
Bn
f1 (ppm)

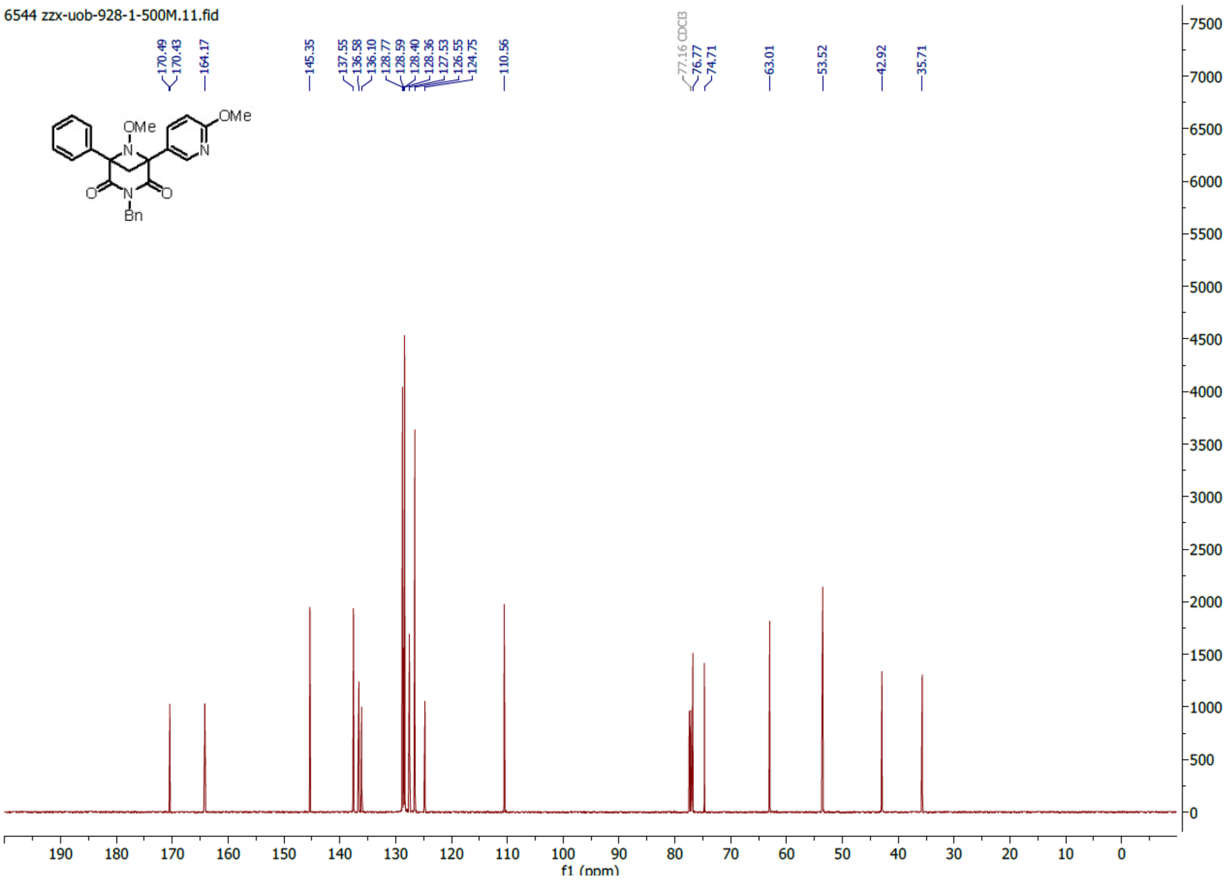
6544 zzx-uob-928-1-500M.11.fid
170.49
170.43
164.17
145.35
137.55
136.58
136.10
128.77
128.59
128.40
128.36
127.53
126.55
124.75
110.56
77.16 CDCl3
76.77
74.71
63.01
53.52
42.92
35.71
OMe
OMe
N
N
O
O
N
Bn
f1 (ppm)

Compound 44

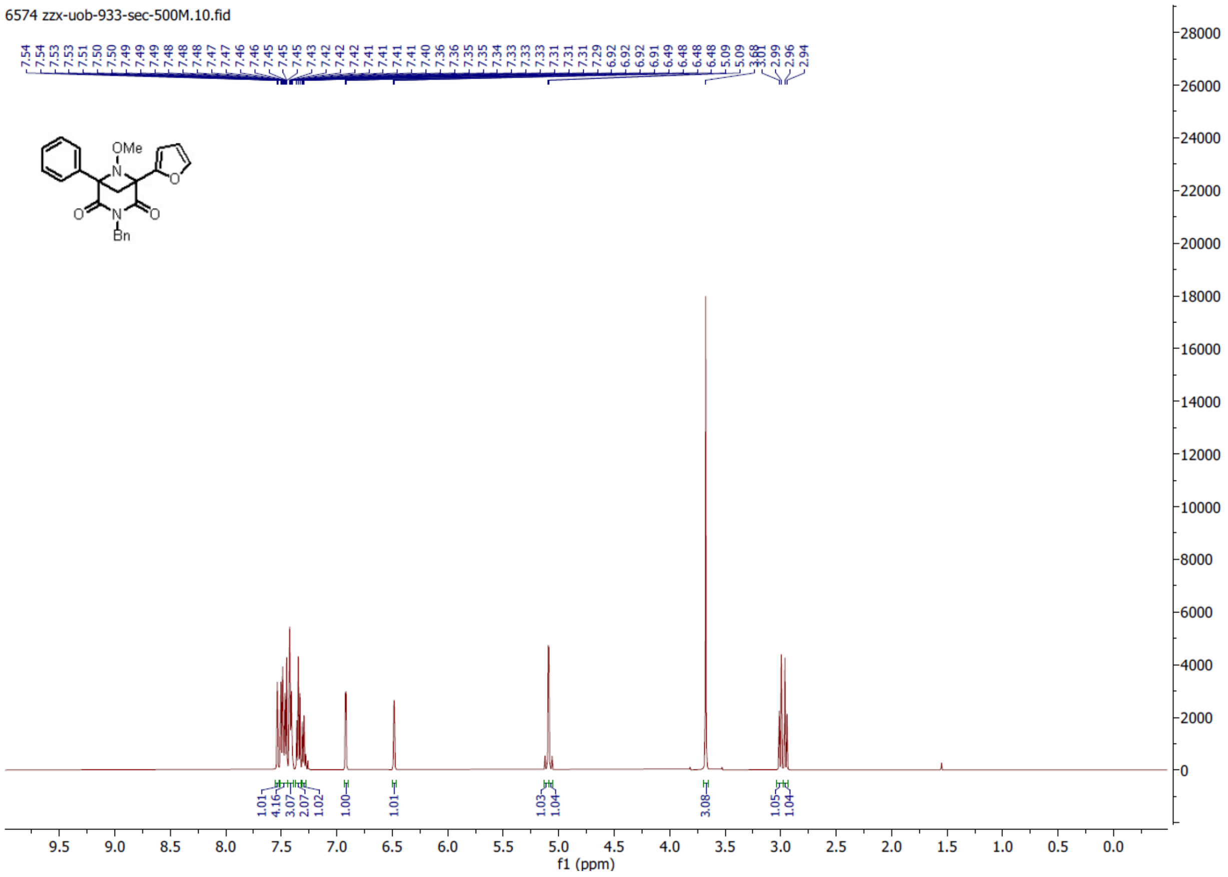
6574 zzx-uob-933-sec-500M.10.fid
OMe
N
O
O
N
Bn
O
f1 (ppm)

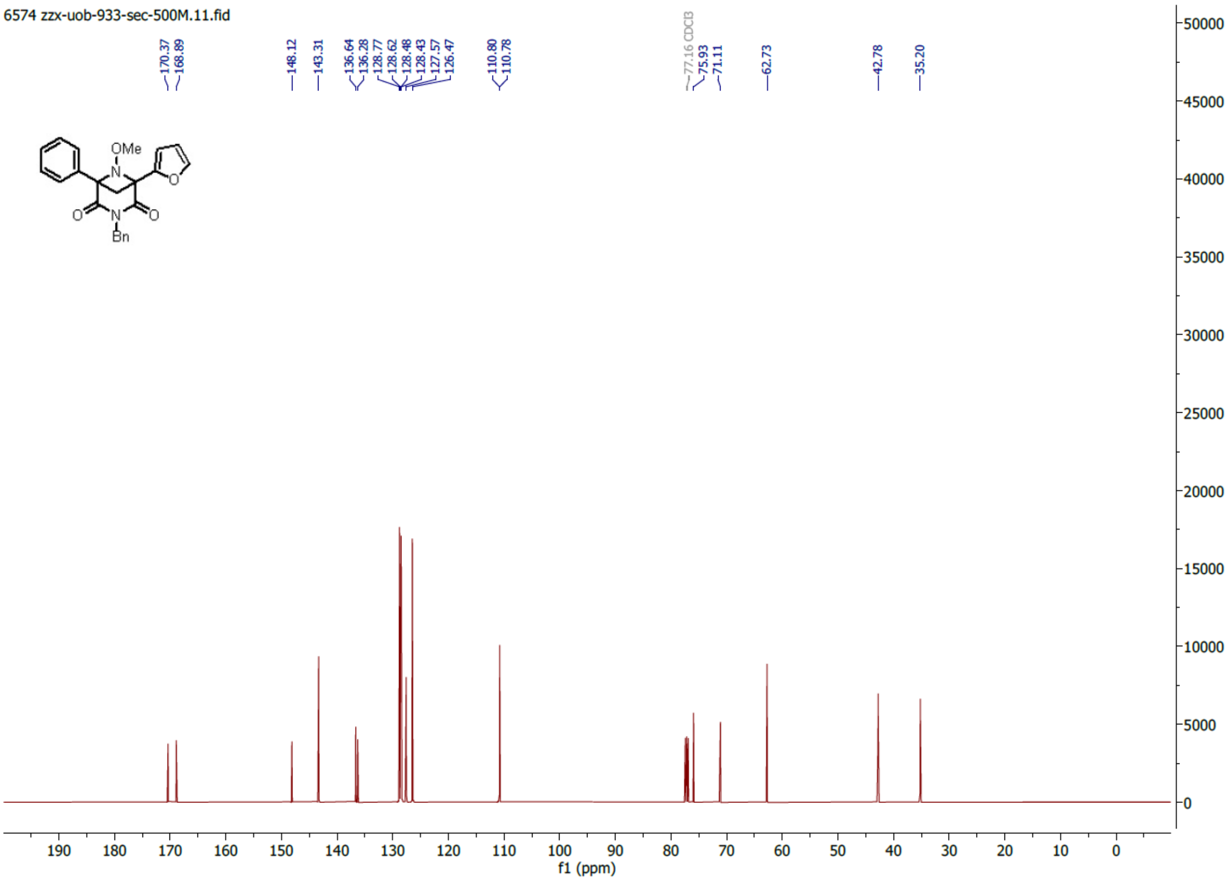
6574 zzx-uob-933-sec-500M.11.fid
170.37
168.89
148.12
143.31
136.64
136.28
128.77
128.62
128.48
128.43
127.57
126.47
110.80
110.78
77.16 CDCl3
75.93
71.11
62.73
42.78
35.20
OMe
N
O
O
N
Bn
O
f1 (ppm)

Compound 47

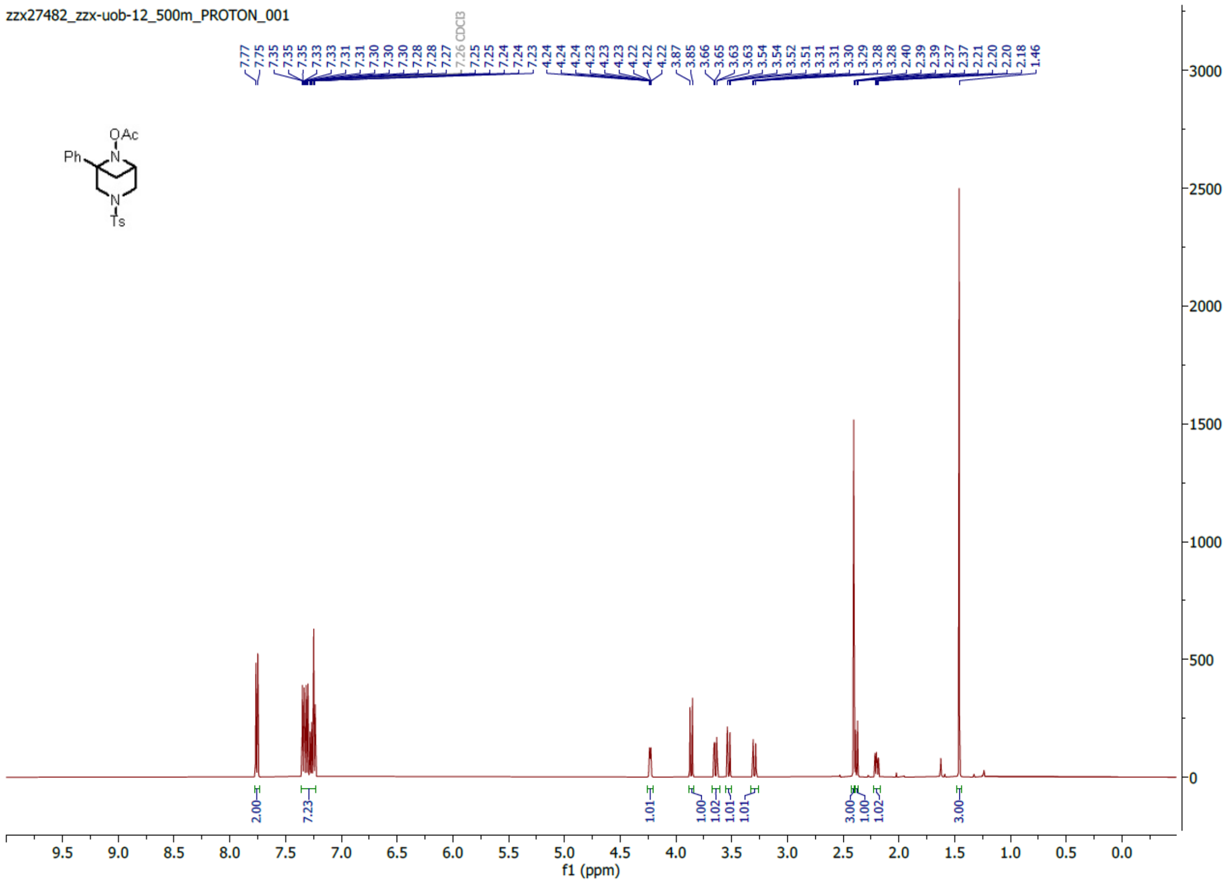

zzx27482_zzx-uob-12_500m_PROTON_001
7.77 7.75 7.35 7.35 7.35 7.33 7.33 7.31 7.31 7.30 7.30 7.30 7.28 7.28 7.27 7.26 CDCl3 7.25 7.25 7.24 7.24 7.23 4.24 4.24 4.24 4.23 4.23 4.23 4.22 4.22 4.22 3.87 3.85 3.66 3.65 3.63 3.63 3.54 3.54 3.52 3.51 3.31 3.31 3.30 3.29 3.28 3.28 2.40 2.39 2.39 2.37 2.37 2.21 2.20 2.20 2.18 1.46
OAc
Ph
N
N
Ts
2.00 7.23 1.01 1.00 1.02 1.01 1.01 3.00 1.00 1.02 3.00
f1 (ppm)

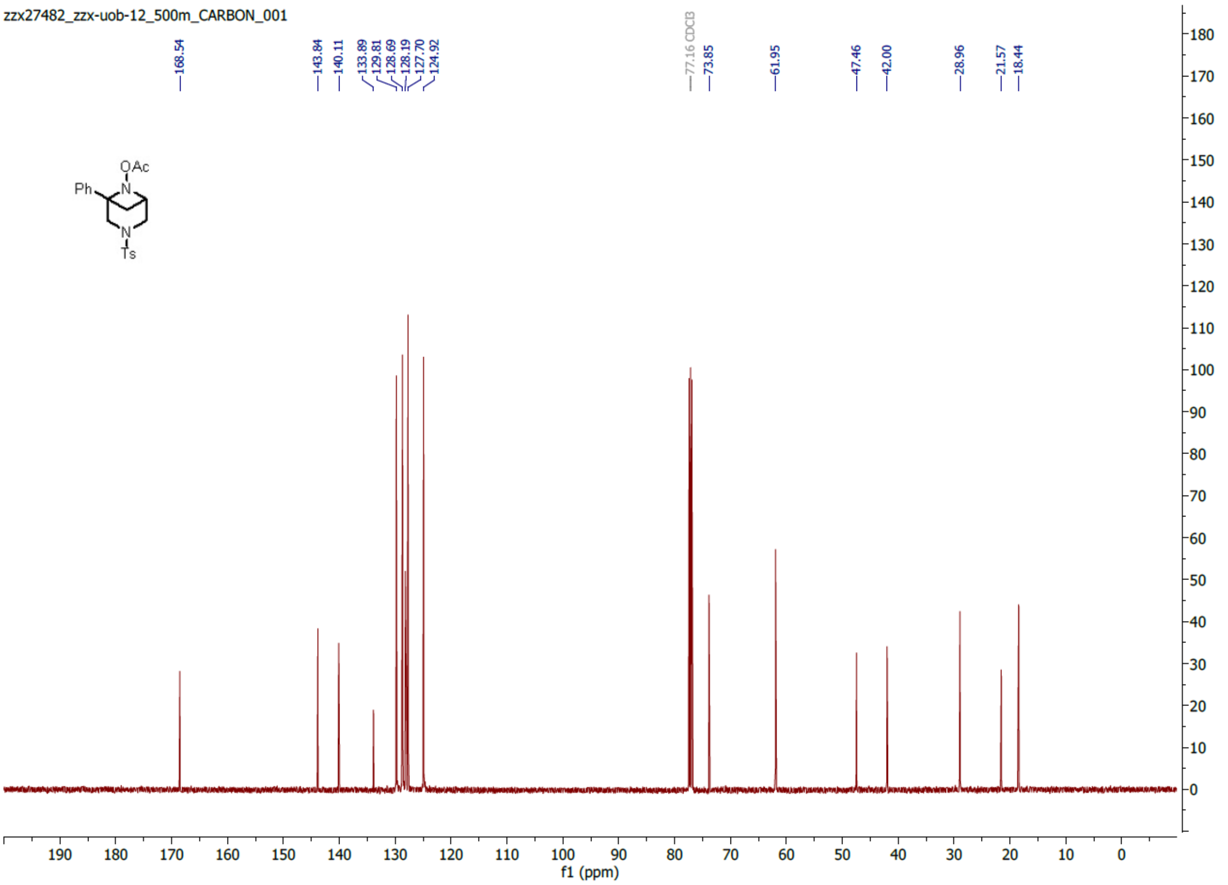

zzx27482_zzx-uob-12_500m_CARBON_001
168.54 143.84 140.11 133.89 129.81 128.69 128.19 127.70 124.92 77.16 CDCl3 73.85 61.95 47.46 42.00 28.96 21.57 18.44
OAc
Ph
N
N
Ts
f1 (ppm)

Compound 46

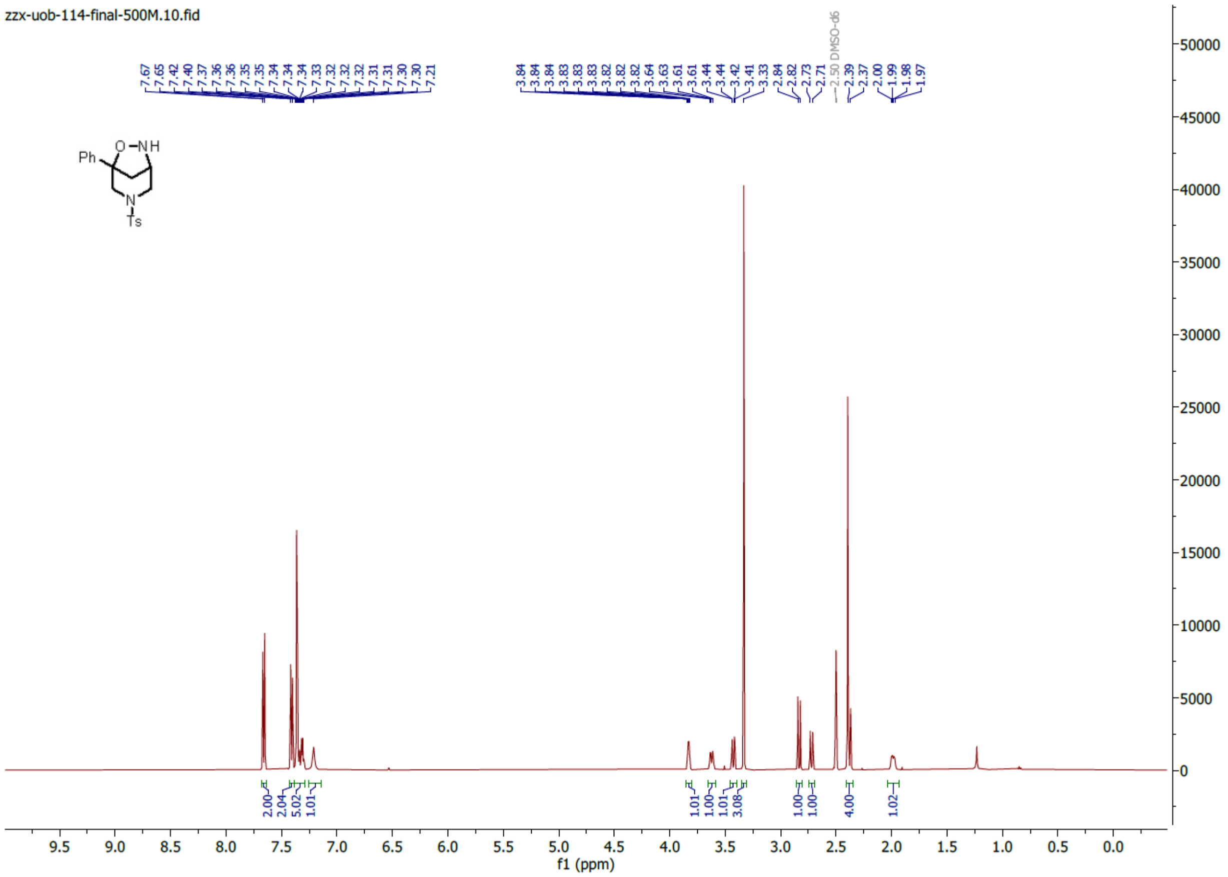

zzx-uob-114-final-500M.10.fid
Ph
O
NH
N
Ts
f1 (ppm)

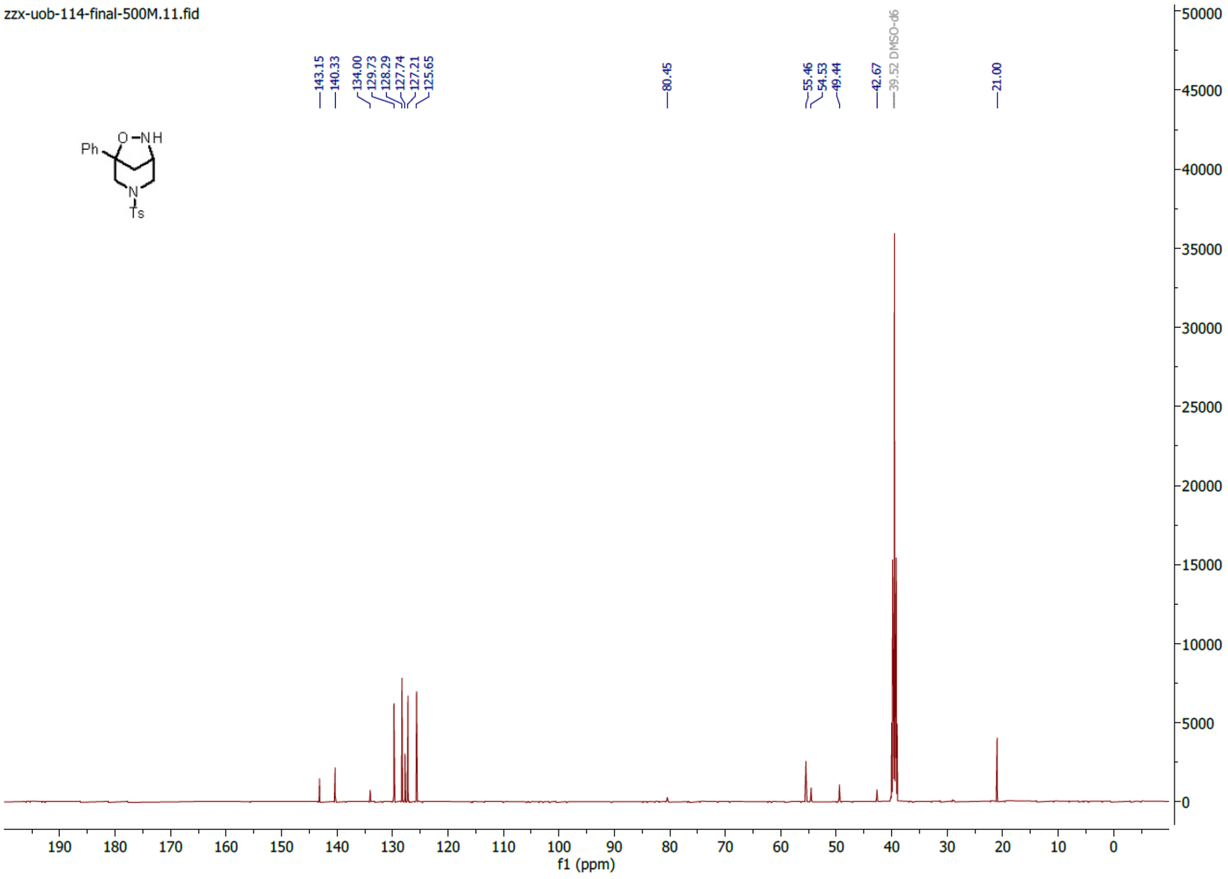

zzx-uob-114-final-500M.11.fid
143.15
140.33
134.00
129.73
128.29
127.74
127.21
125.65
80.45
55.46
54.53
49.44
42.67
39.52 DMSO-d6
21.00
Ph
O
NH
N
Ts
f1 (ppm)

Compound 48

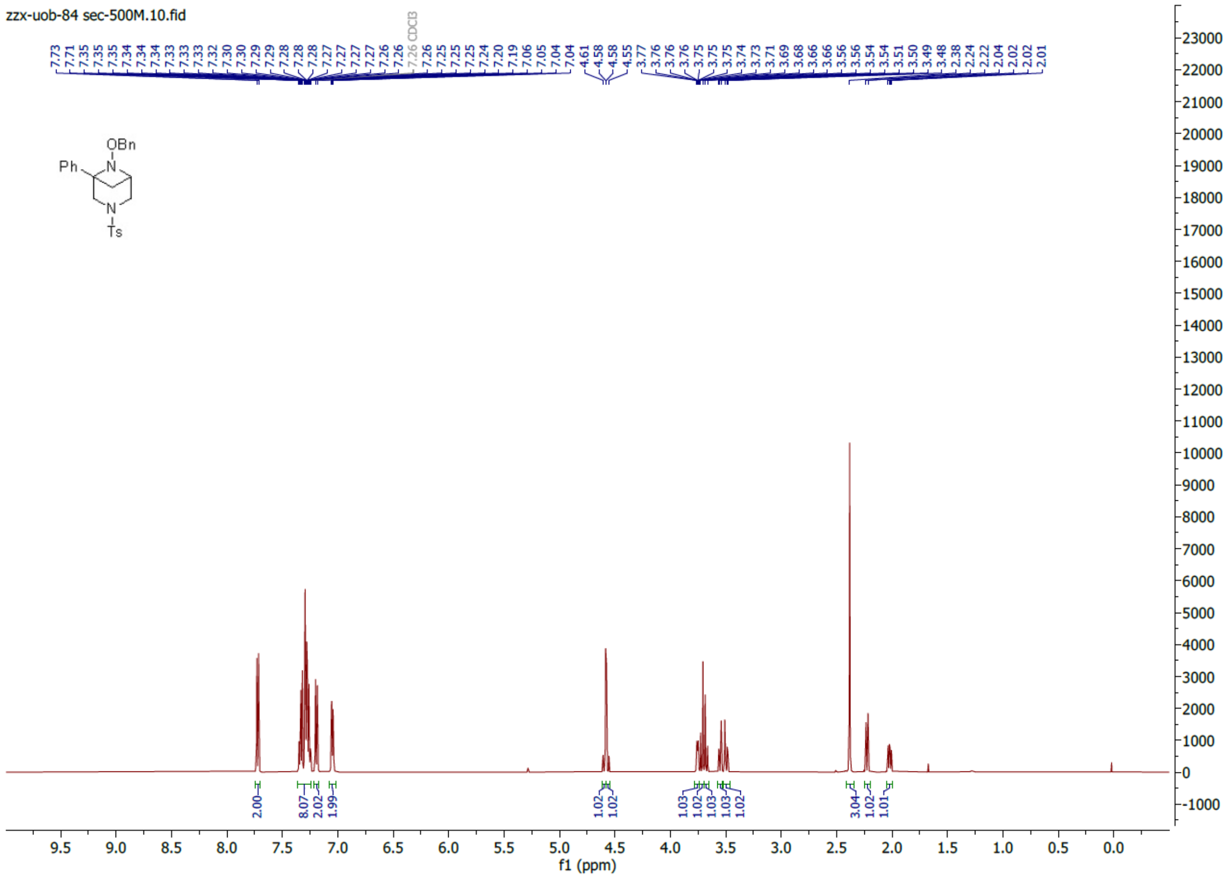

zzx-uob-84 sec-500M.10.fid
7.73 7.71 7.35 7.35 7.35 7.34 7.34 7.34 7.33 7.33 7.33 7.32 7.30 7.29 7.29 7.28 7.28 7.28 7.27 7.27 7.27 7.27 7.26 7.26 7.26 CDCl3 7.26 7.25 7.25 7.25 7.24 7.20 7.19 7.06 7.05 7.04 7.04 4.61 4.58 4.58 4.55 3.77 3.76 3.76 3.76 3.75 3.75 3.75 3.74 3.73 3.71 3.69 3.68 3.66 3.66 3.56 3.56 3.54 3.54 3.51 3.50 3.49 3.48 2.38 2.24 2.22 2.04 2.02 2.02 2.01
OBn
Ph
N
N
Ts
2.00 8.07 2.02 1.99 1.02 1.02 1.03 1.02 1.03 1.03 1.02 3.04 1.02 1.01
f1 (ppm)

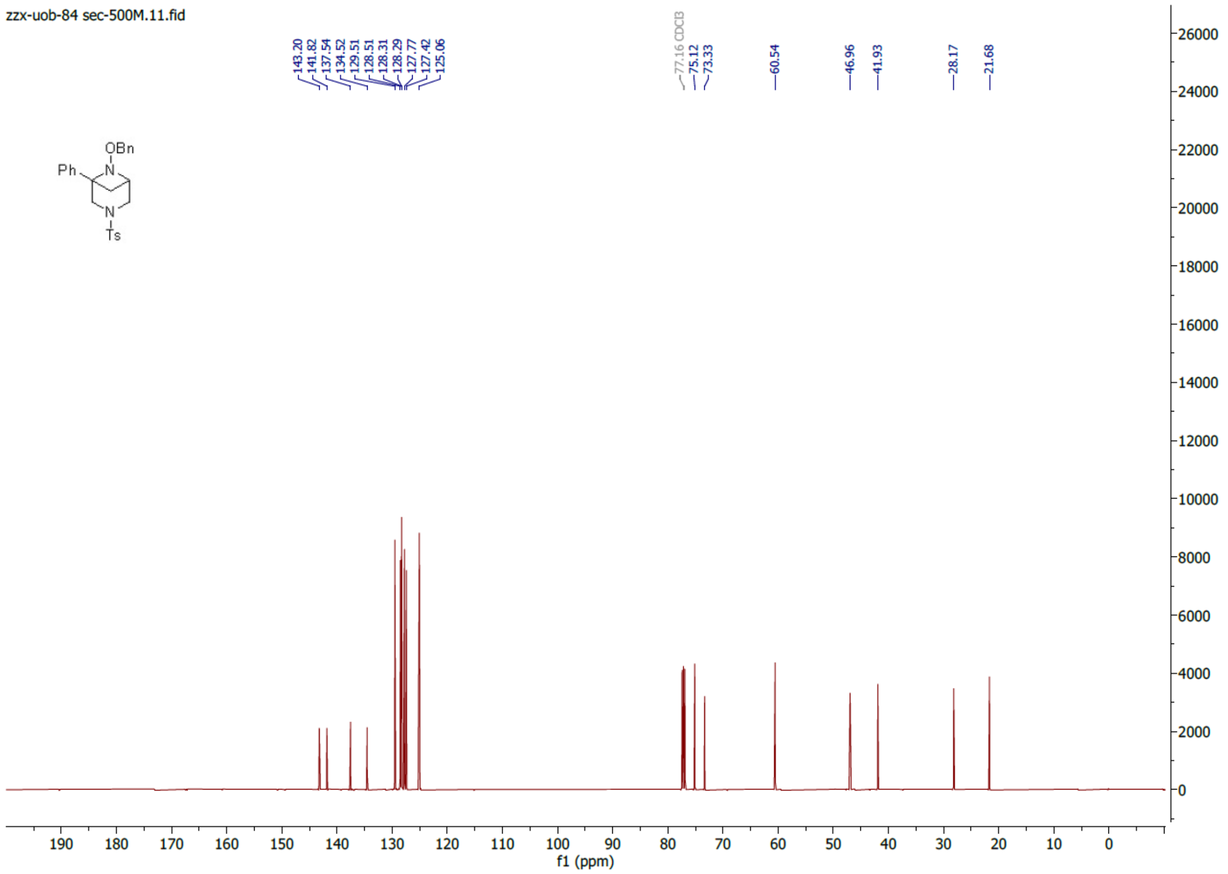

zzx-uob-84 sec-500M.11.fid
143.20 141.82 137.54 134.52 129.51 128.51 128.31 128.29 127.77 127.42 125.06
77.16 CDCl3 75.12 73.33
60.54
46.96
41.93
28.17
21.68
OBn
Ph
N
N
Ts
f1 (ppm)

Compound 49

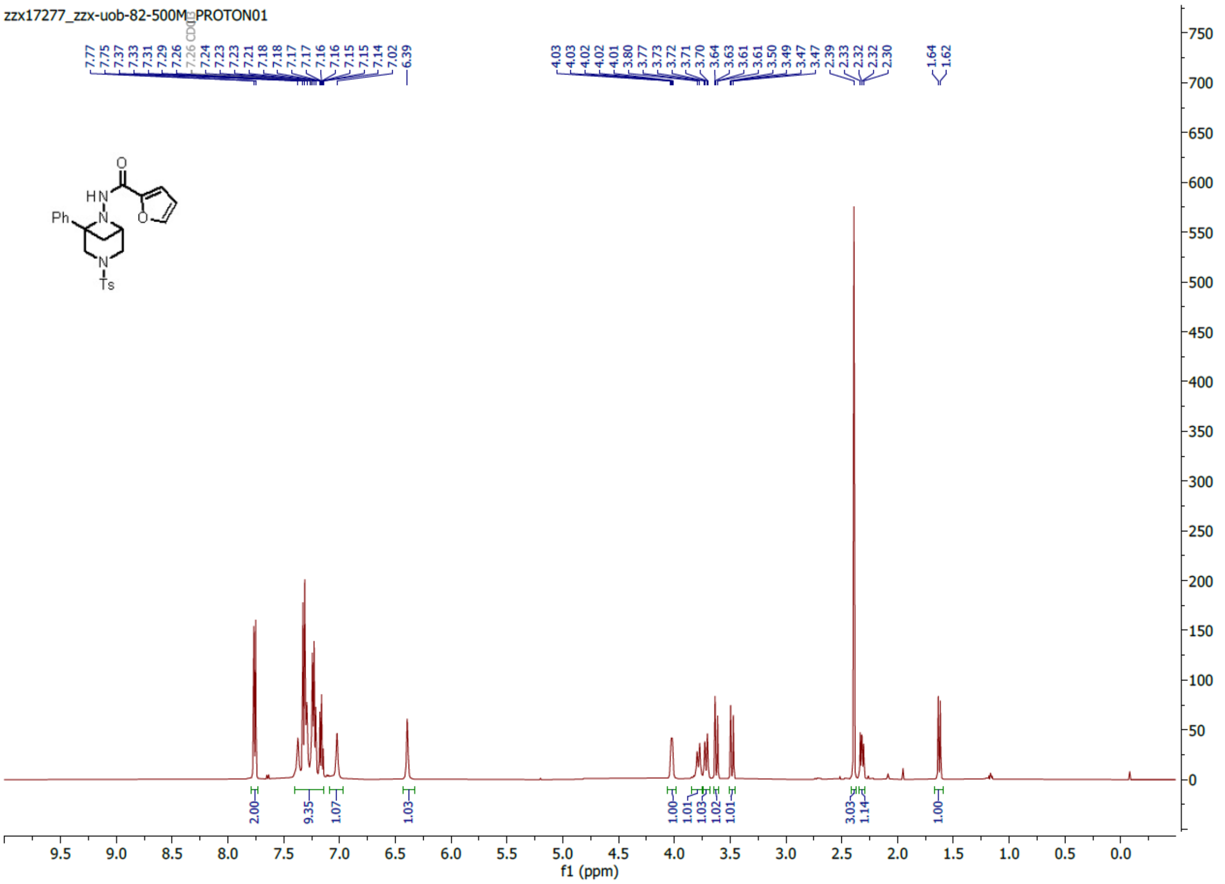
zzx17277_zzx-uob-82-500M_PROTON01
f1 (ppm)

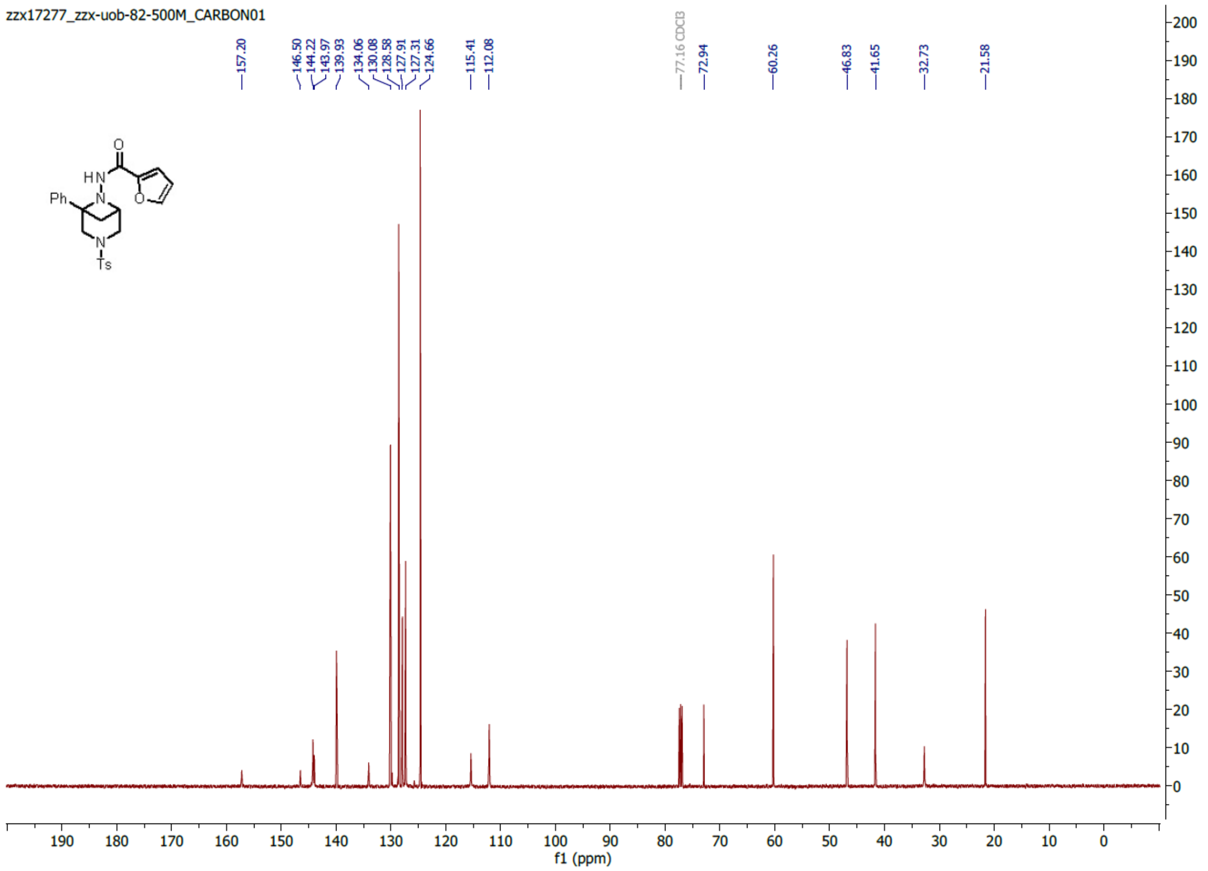
zzx17277_zzx-uob-82-500M_CARBON01
f1 (ppm)

Compound 50

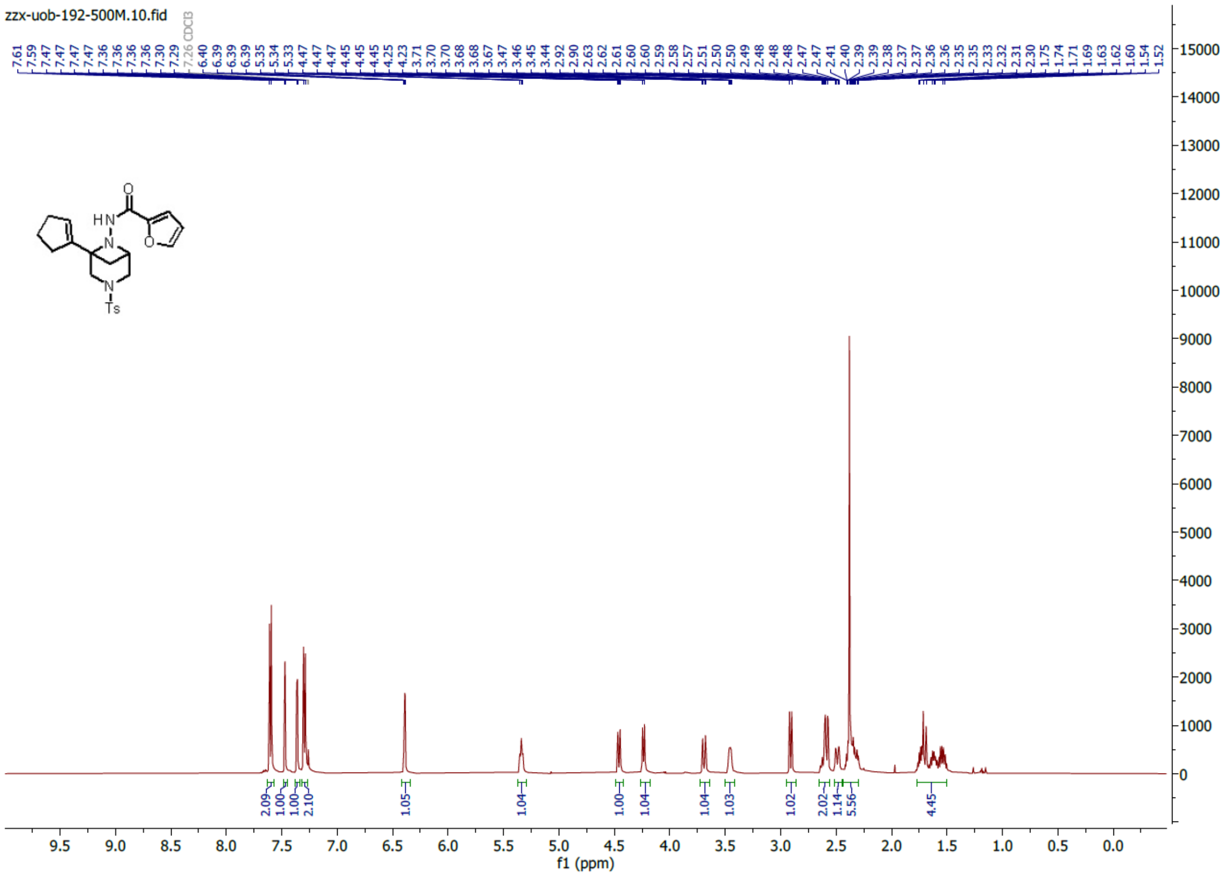
zzx-uob-192-500M.10.fid
7.61 7.59 7.47 7.47 7.47 7.47 7.36 7.36 7.36 7.36 7.30 7.29 7.26 CDCl3 6.40 6.39 6.39 6.39 6.39 5.35 5.34 5.33 4.47 4.47 4.47 4.45 4.45 4.45 4.25 4.23 3.71 3.70 3.70 3.68 3.68 3.67 3.47 3.46 3.45 3.44 2.92 2.90 2.63 2.62 2.61 2.60 2.60 2.59 2.58 2.57 2.51 2.50 2.50 2.49 2.48 2.48 2.48 2.47 2.47 2.41 2.40 2.39 2.39 2.38 2.37 2.37 2.36 2.36 2.35 2.35 2.33 2.32 2.31 2.30 1.75 1.74 1.71 1.69 1.63 1.62 1.60 1.54 1.52
HN
N
O
O
N
Ts
2.09 1.00 1.00 2.10 1.05 1.04 1.00 1.04 1.04 1.03 1.02 2.02 1.14 5.56 4.45
f1 (ppm)

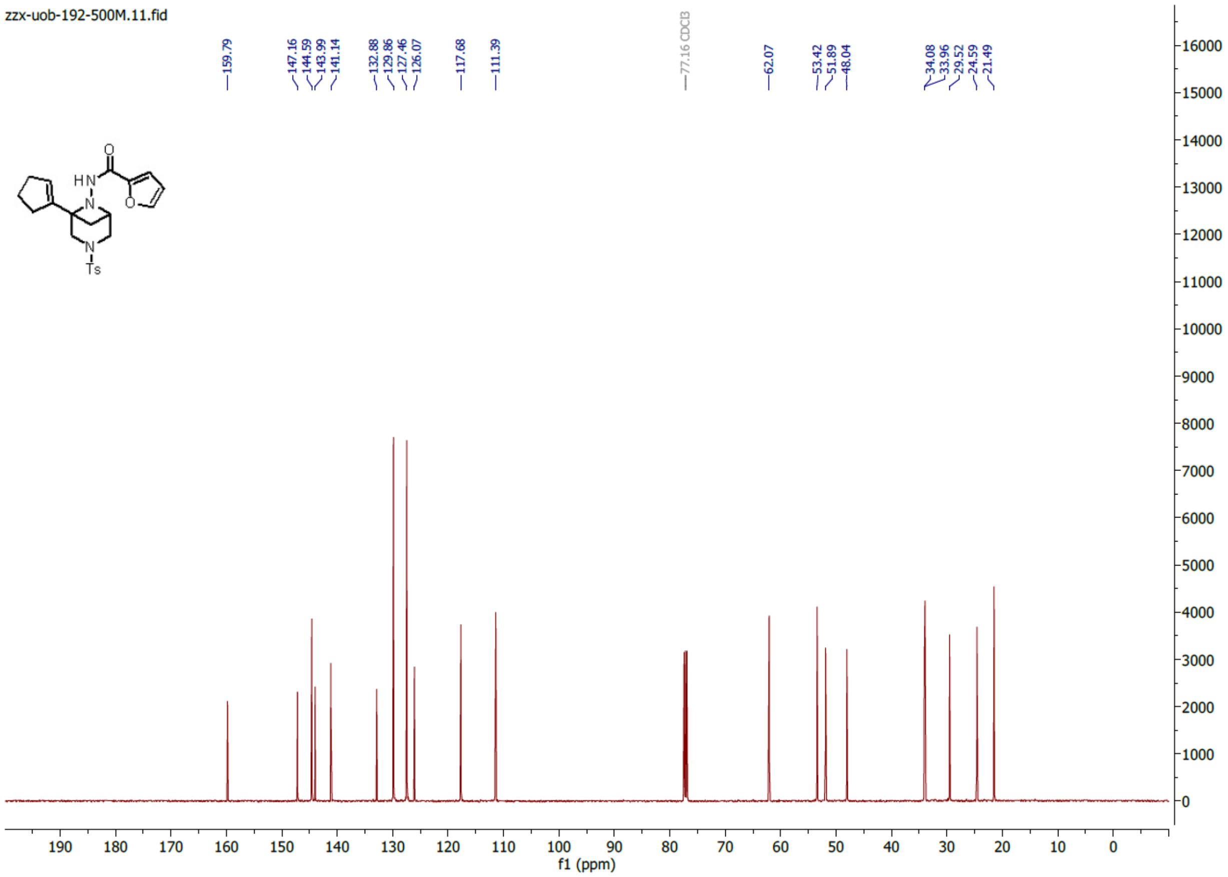
zzx-uob-192-500M.11.fid
159.79 147.16 144.59 143.99 141.14 132.88 129.86 127.46 126.07 117.68 111.39 77.16 CDCl3 62.07 53.42 51.89 48.04 34.08 33.96 29.52 24.59 21.49
HN
N
O
O
N
Ts
f1 (ppm)

Compound 51

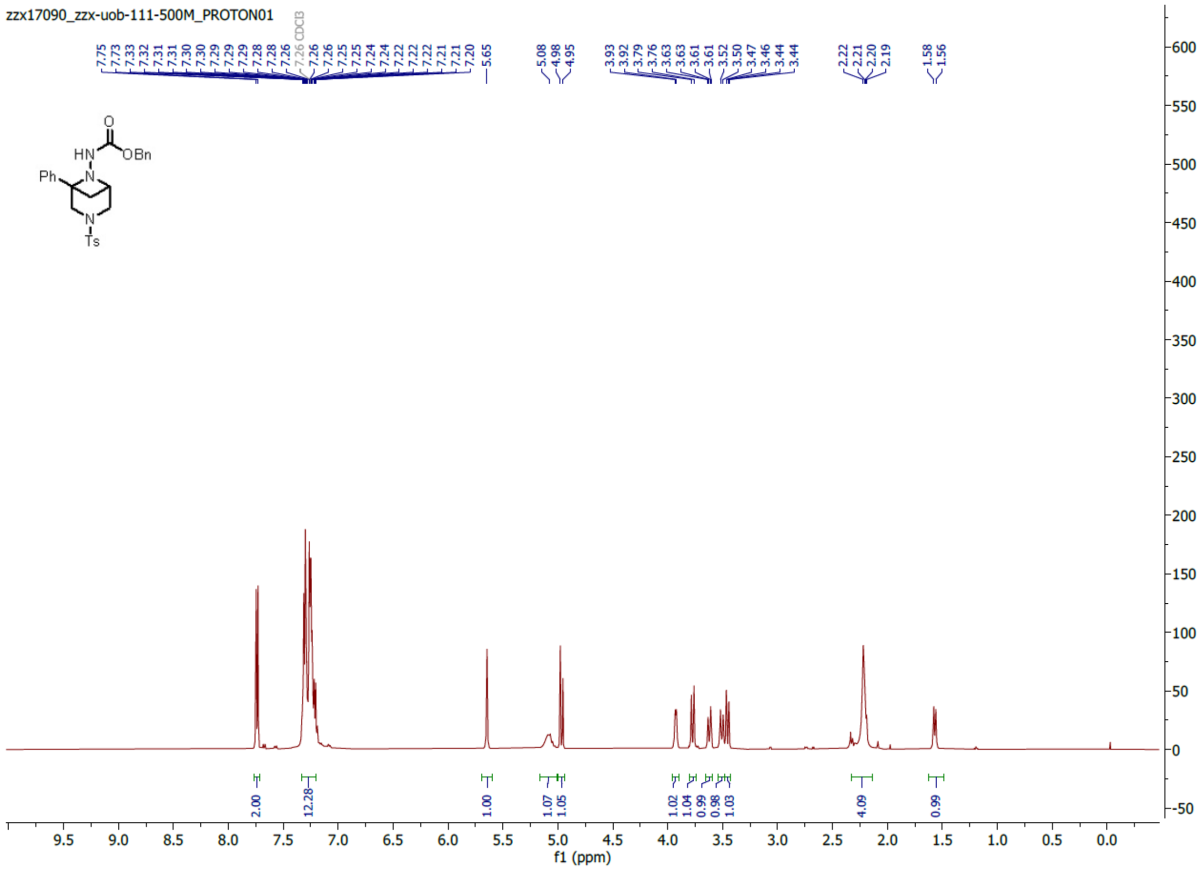

zzx17090_zzx-uob-111-500M_PROTON01
7.75
7.73
7.33
7.32
7.31
7.31
7.30
7.30
7.29
7.29
7.29
7.28
7.28
7.26
7.26 CDCl3
7.26
7.26
7.25
7.25
7.24
7.24
7.22
7.22
7.22
7.22
7.21
7.21
7.20
5.65
5.08
4.98
4.95
3.93
3.92
3.79
3.76
3.63
3.63
3.61
3.61
3.52
3.50
3.47
3.46
3.44
3.44
2.22
2.21
2.20
2.19
1.58
1.56
HN
O
OBn
Ph
N
N
Ts
2.00
12.28
1.00
1.07
1.05
1.02
1.04
0.99
0.98
1.03
4.09
0.99
f1 (ppm)

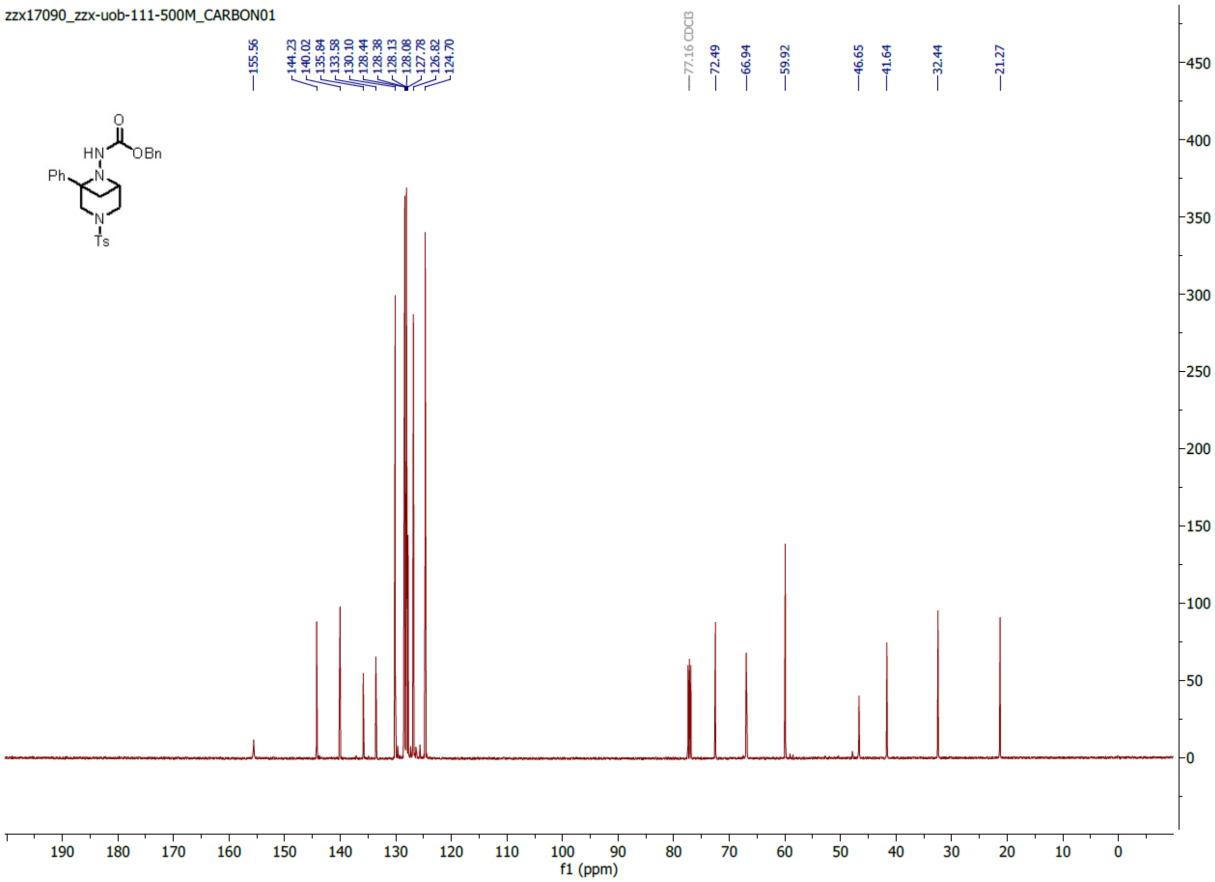

zzx17090_zzx-uob-111-500M_CARBON01
155.56
144.23
140.02
135.84
133.58
130.10
128.44
128.38
128.13
128.08
127.78
126.82
124.70
77.16 CDCl3
72.49
66.94
59.92
46.65
41.64
32.44
21.27
HN
O
OBn
Ph
N
N
Ts
f1 (ppm)

Compound 52

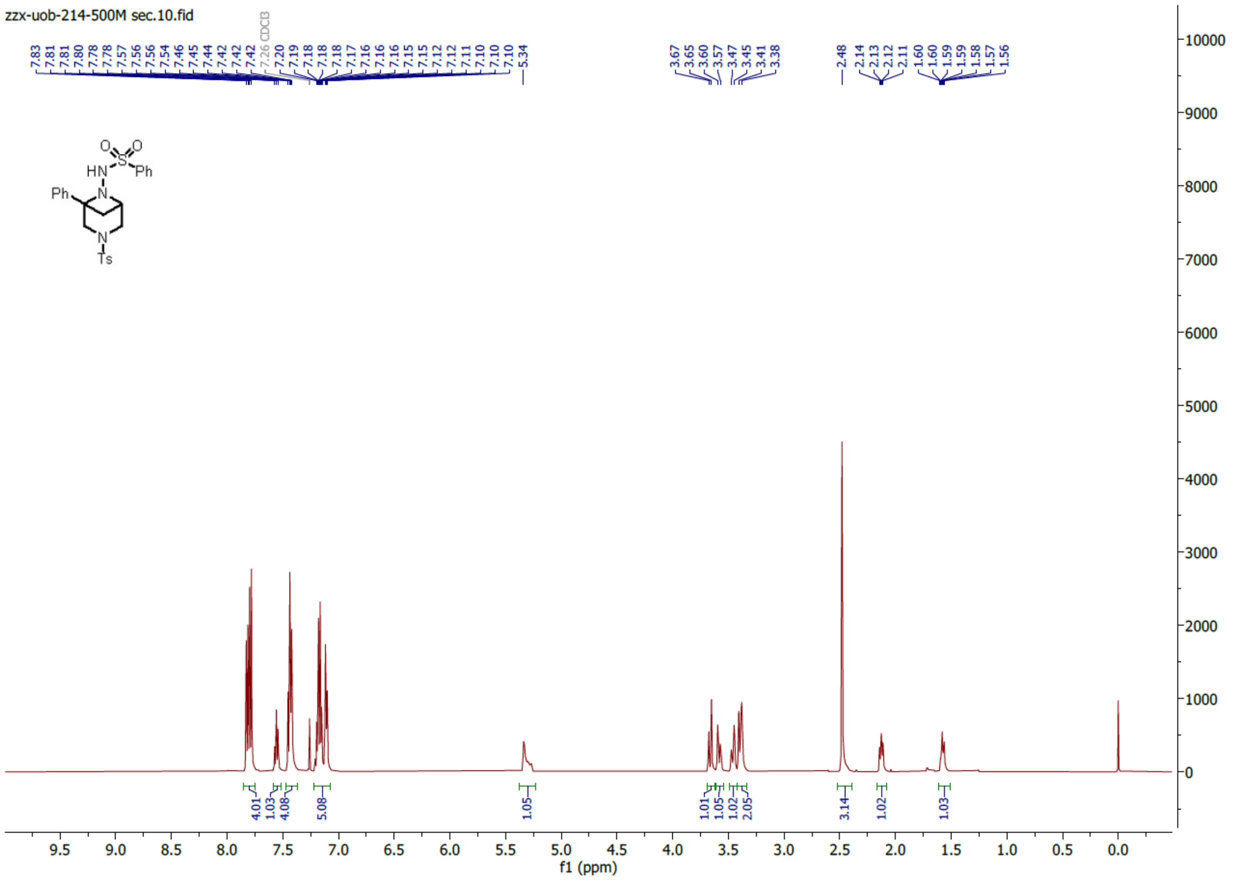

zzx-uob-214-500M sec.10.fid
7.83 7.81 7.81 7.80 7.78 7.78 7.57 7.56 7.56 7.54 7.46 7.45 7.44 7.42 7.42 7.42 7.42 7.26 CDCl3 7.20 7.19 7.18 7.18 7.18 7.17 7.16 7.16 7.15 7.15 7.12 7.12 7.11 7.10 7.10 7.10 5.34
3.67 3.65 3.60 3.57 3.47 3.45 3.41 3.38
2.48 2.14 2.13 2.12 2.11 1.60 1.60 1.59 1.59 1.58 1.57 1.56
4.01 1.03 4.08 5.08 1.05 1.01 1.05 1.02 2.05 3.14 1.02 1.03
f1 (ppm)

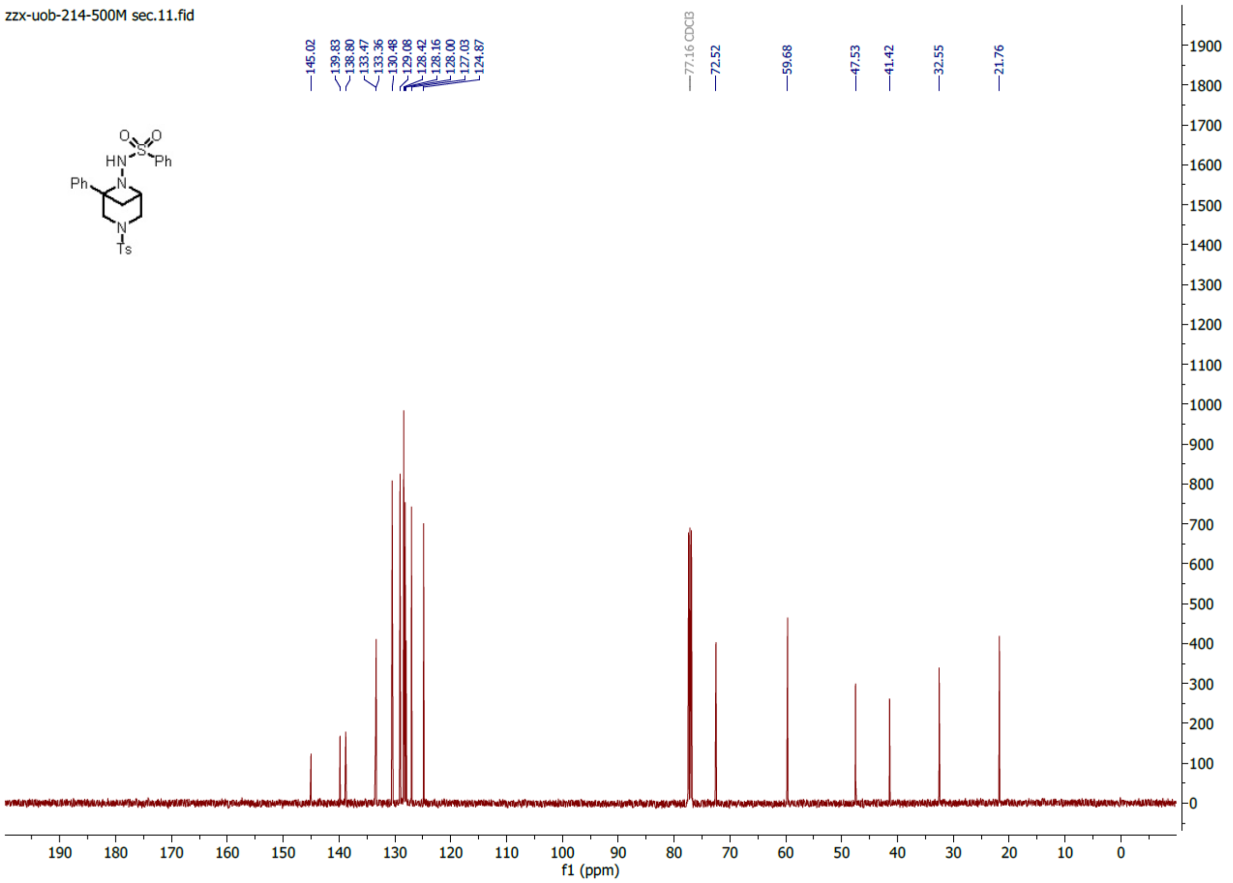

zzx-uob-214-500M sec.11.fid
145.02 139.83 138.80 133.47 133.36 130.48 129.08 128.42 128.16 128.00 127.03 124.87
77.16 CDCl3 72.52 59.68 47.53 41.42 32.55 21.76
f1 (ppm)

Compound 53

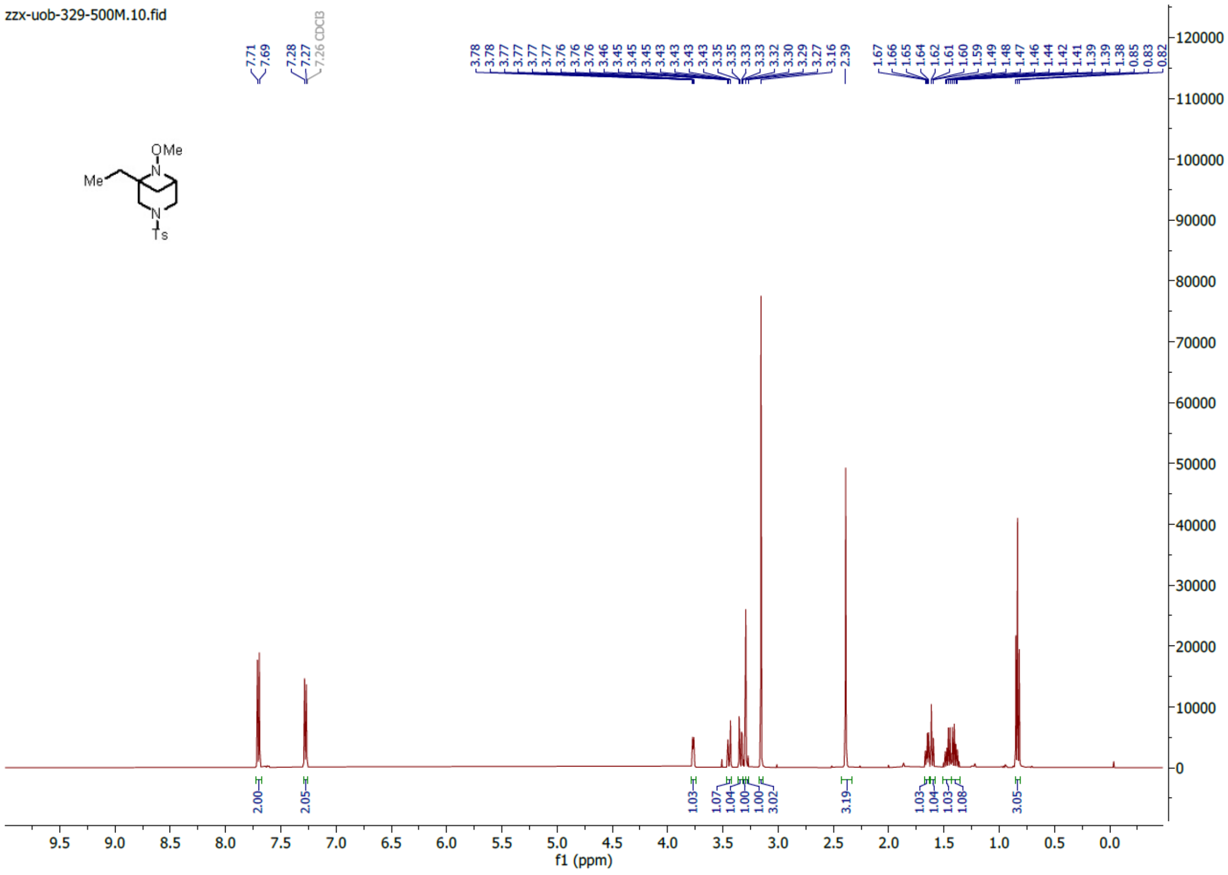
zzx-uob-329-500M.10.fid
OMe
Me
N
N
Ts
f1 (ppm)

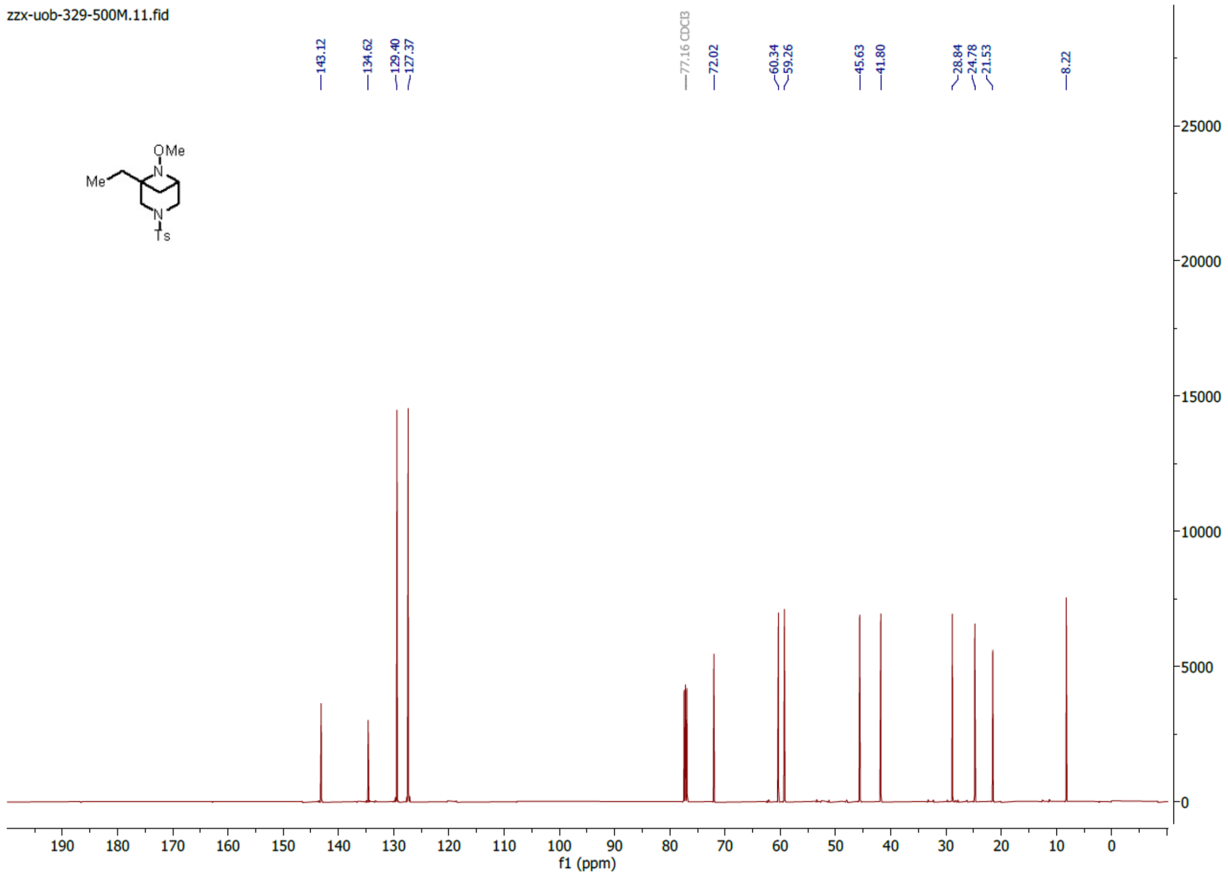
zzx-uob-329-500M.11.fid
143.12
134.62
129.40
127.37
77.16 CDCl3
72.02
60.34
59.26
45.63
41.80
28.84
24.78
21.53
8.22
OMe
Me
N
N
Ts
f1 (ppm)

Compound 54

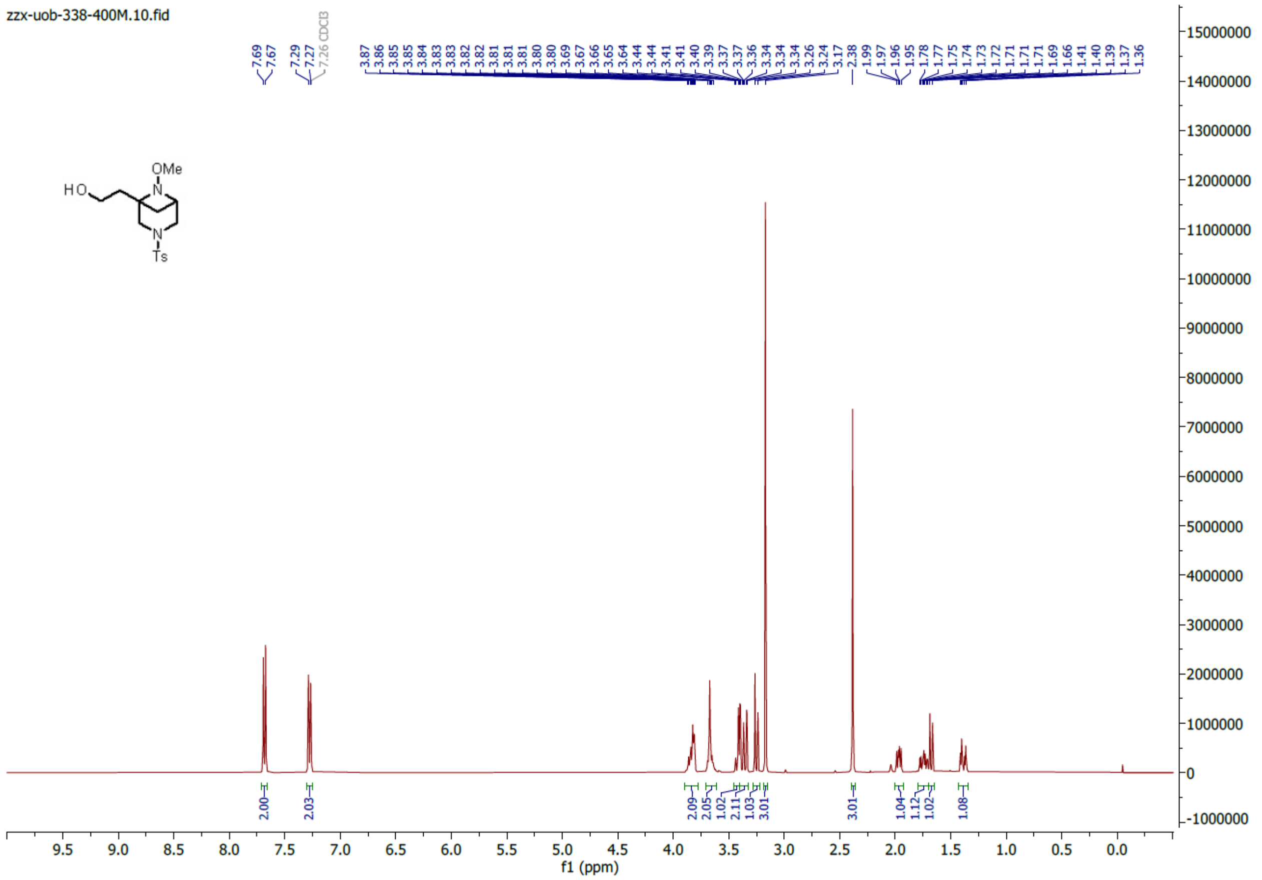

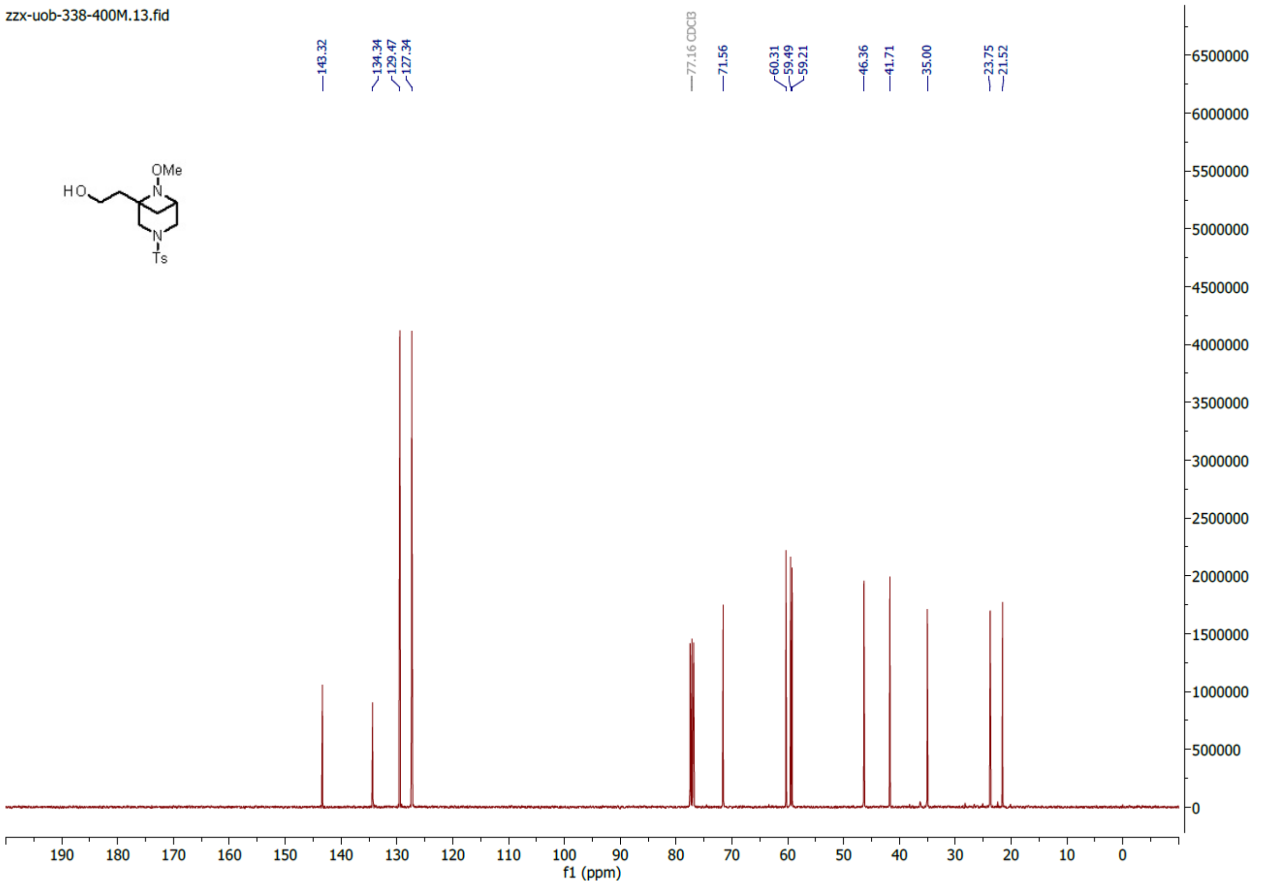

Compound 55

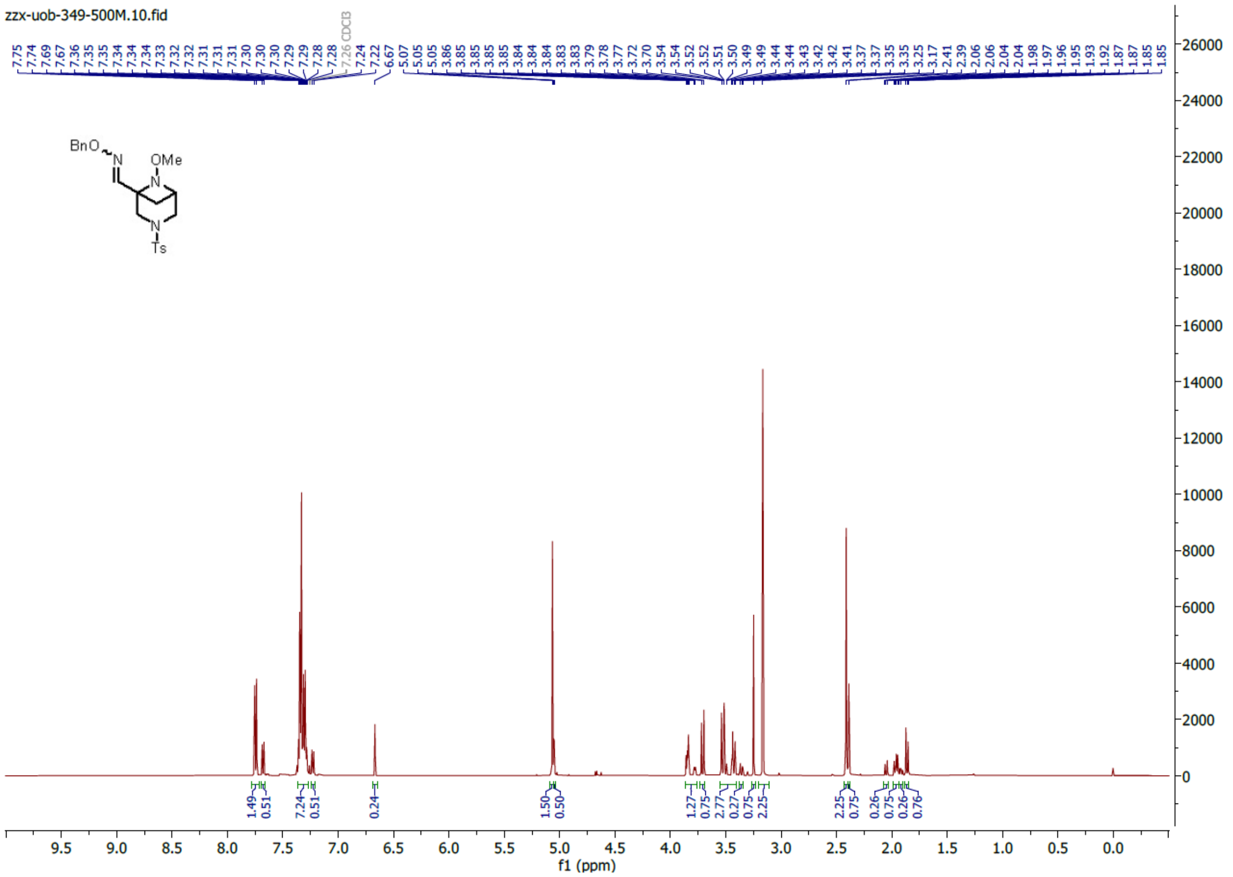

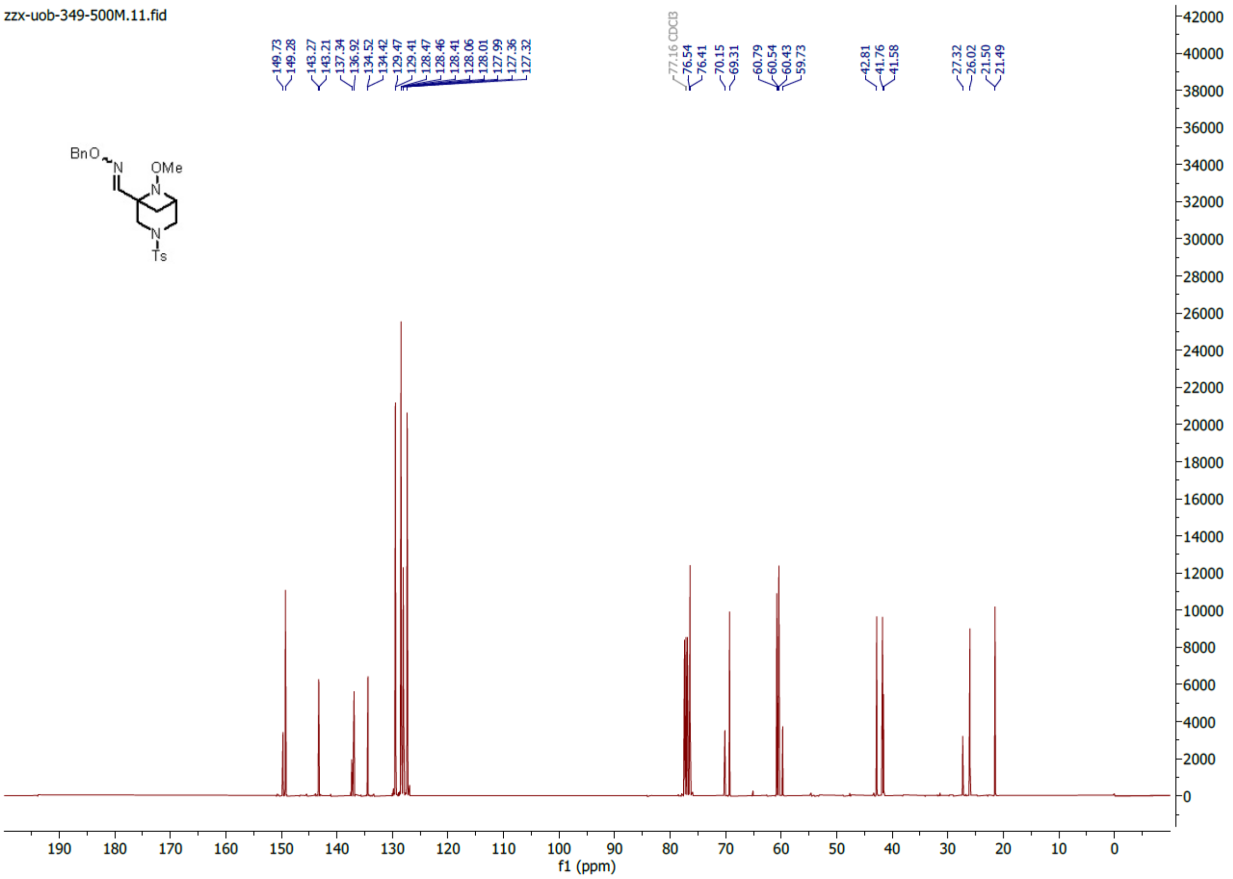

Compound 56

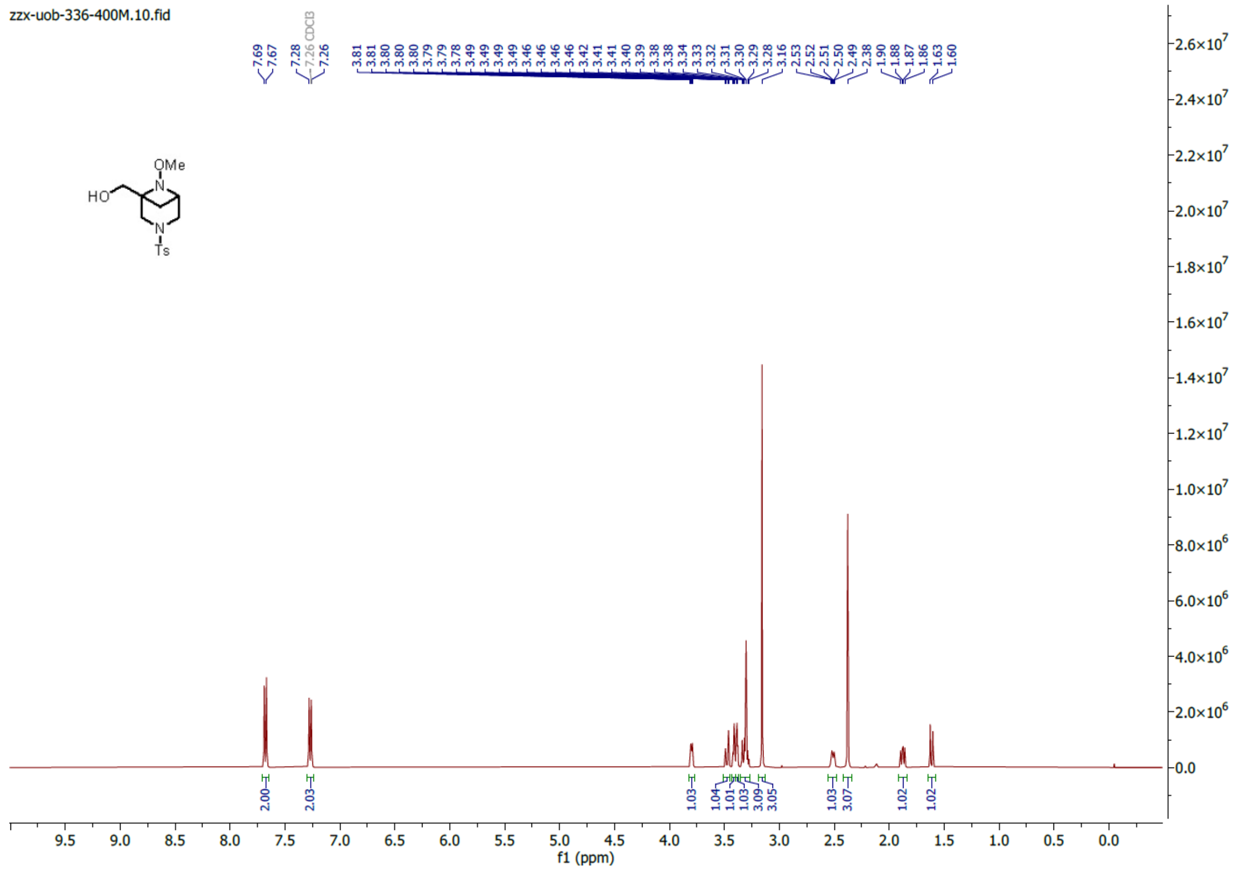

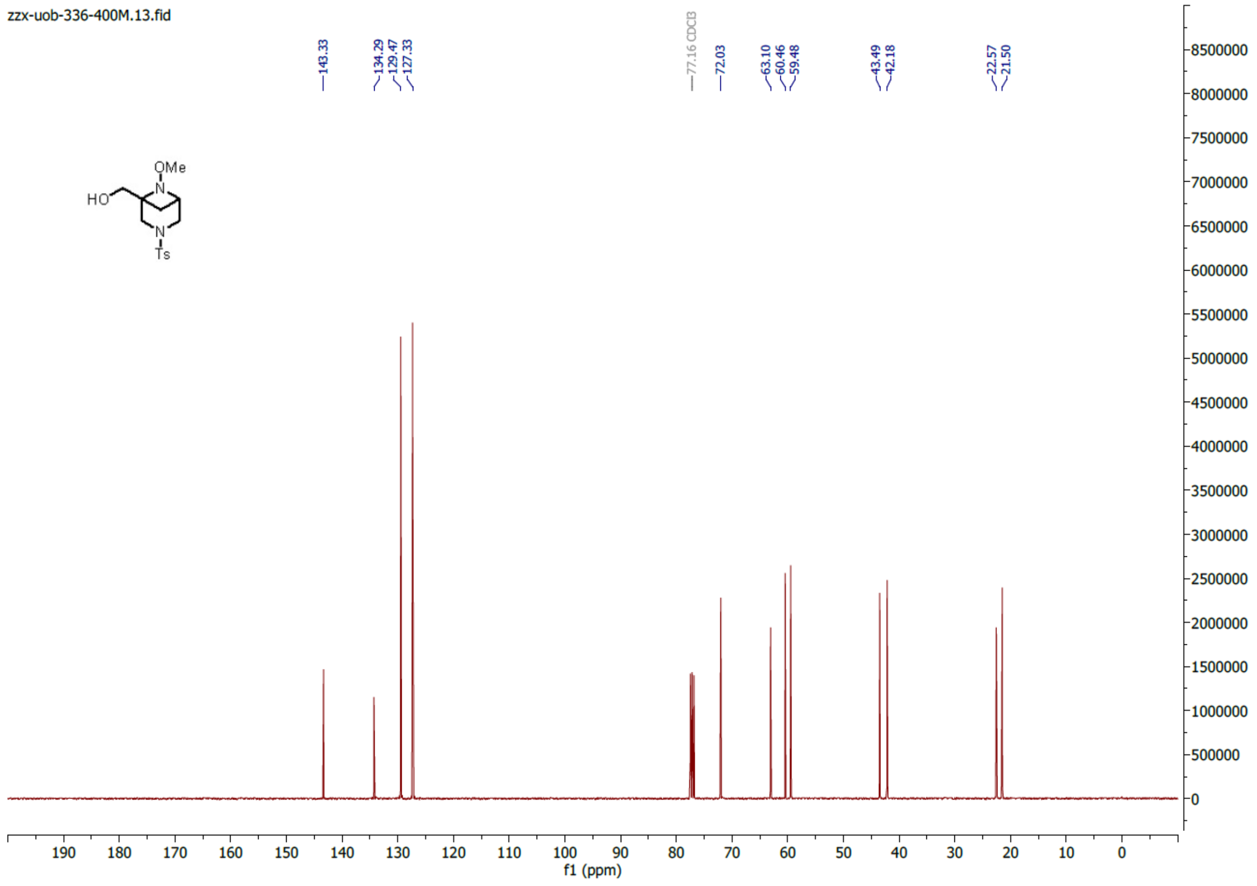

Compound 57’

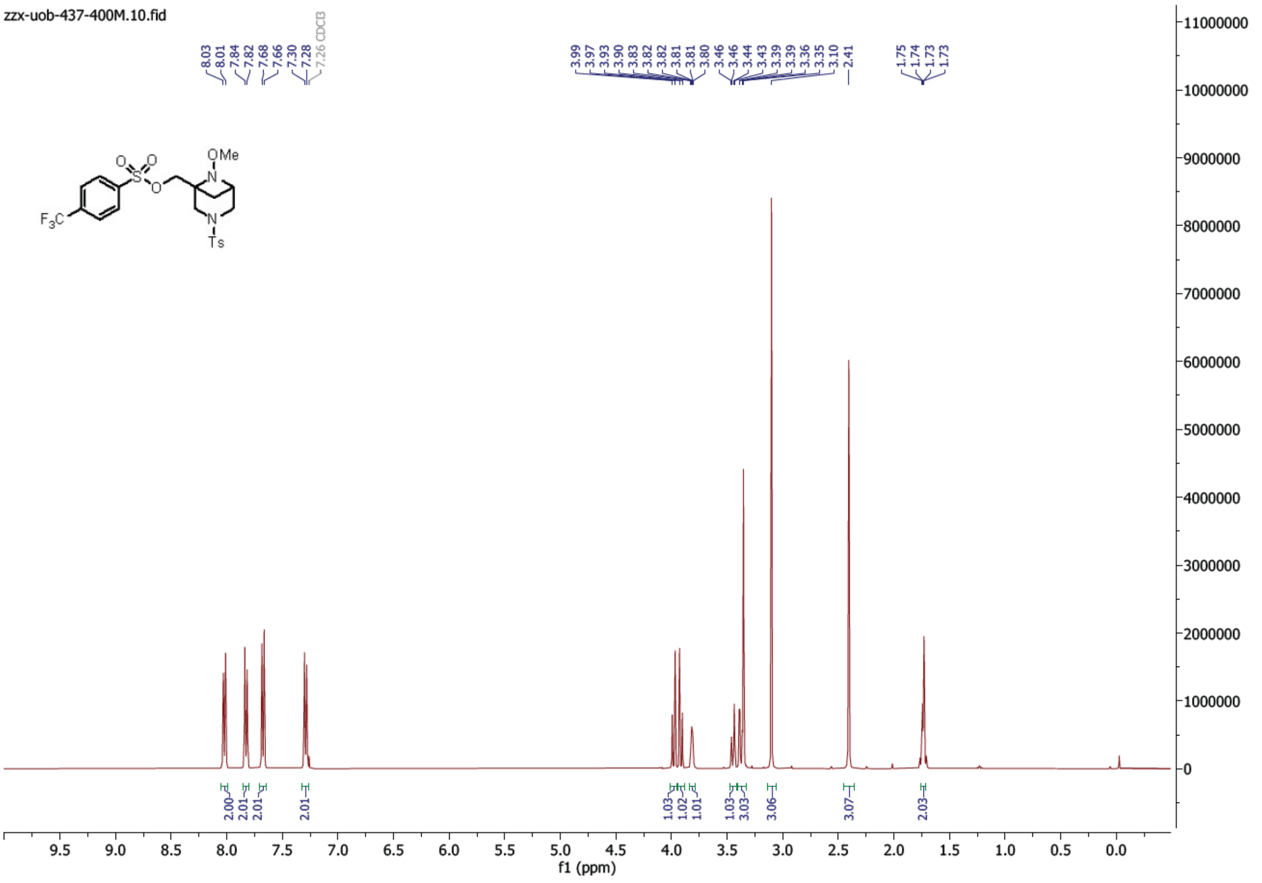
zzx-uob-437-400M.10.fid
8.03
8.01
7.84
7.82
7.68
7.66
7.30
7.28
7.26 CDCl3
3.99
3.97
3.93
3.90
3.83
3.82
3.82
3.81
3.81
3.80
3.46
3.44
3.43
3.39
3.39
3.36
3.35
3.10
2.41
1.75
1.74
1.73
1.73
OMe
N
F3C
S
O
O
O
N
Ts
2.00
2.01
2.01
2.01
1.03
1.02
1.01
1.03
3.03
3.06
3.07
2.03
f1 (ppm)

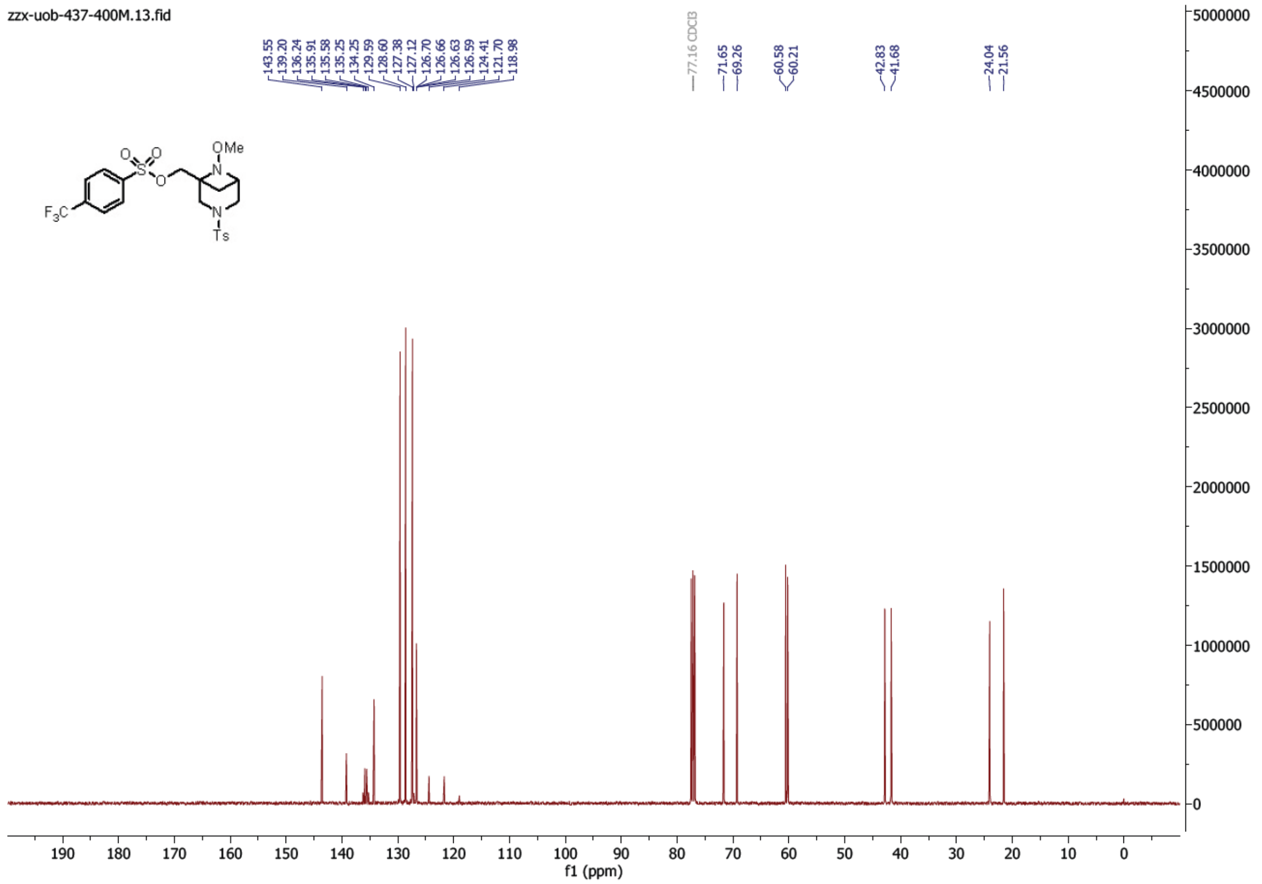
zzx-uob-437-400M.13.fid
143.55
139.20
136.24
135.91
135.58
135.25
134.25
129.59
128.60
127.38
127.12
126.70
126.66
126.63
126.59
124.41
121.70
118.98
77.16 CDCl3
71.65
69.26
60.58
60.21
42.83
41.68
24.04
21.56
OMe
N
F3C
S
O
O
O
N
Ts
f1 (ppm)

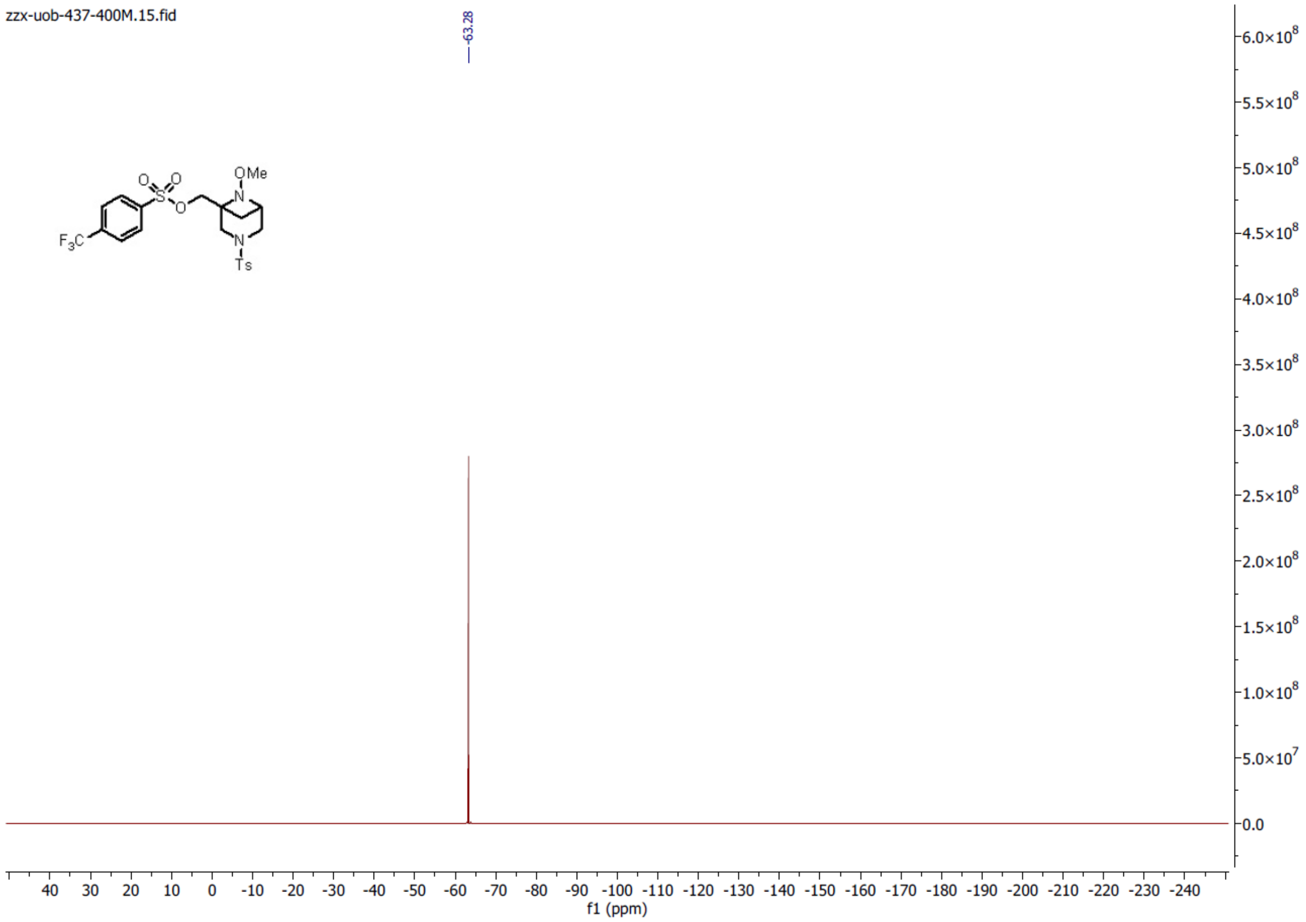
zzx-uob-437-400M.15.fid
-63.28
OMe
N
N
Ts
O
S
O
O
F3C
6.0×10^8
5.5×10^8
5.0×10^8
4.5×10^8
4.0×10^8
3.5×10^8
3.0×10^8
2.5×10^8
2.0×10^8
1.5×10^8
1.0×10^8
5.0×10^7
0.0
40 30 20 10 0 -10 -20 -30 -40 -50 -60 -70 -80 -90 -100 -110 -120 -130 -140 -150 -160 -170 -180 -190 -200 -210 -220 -230 -240
f1 (ppm)

Compound 57

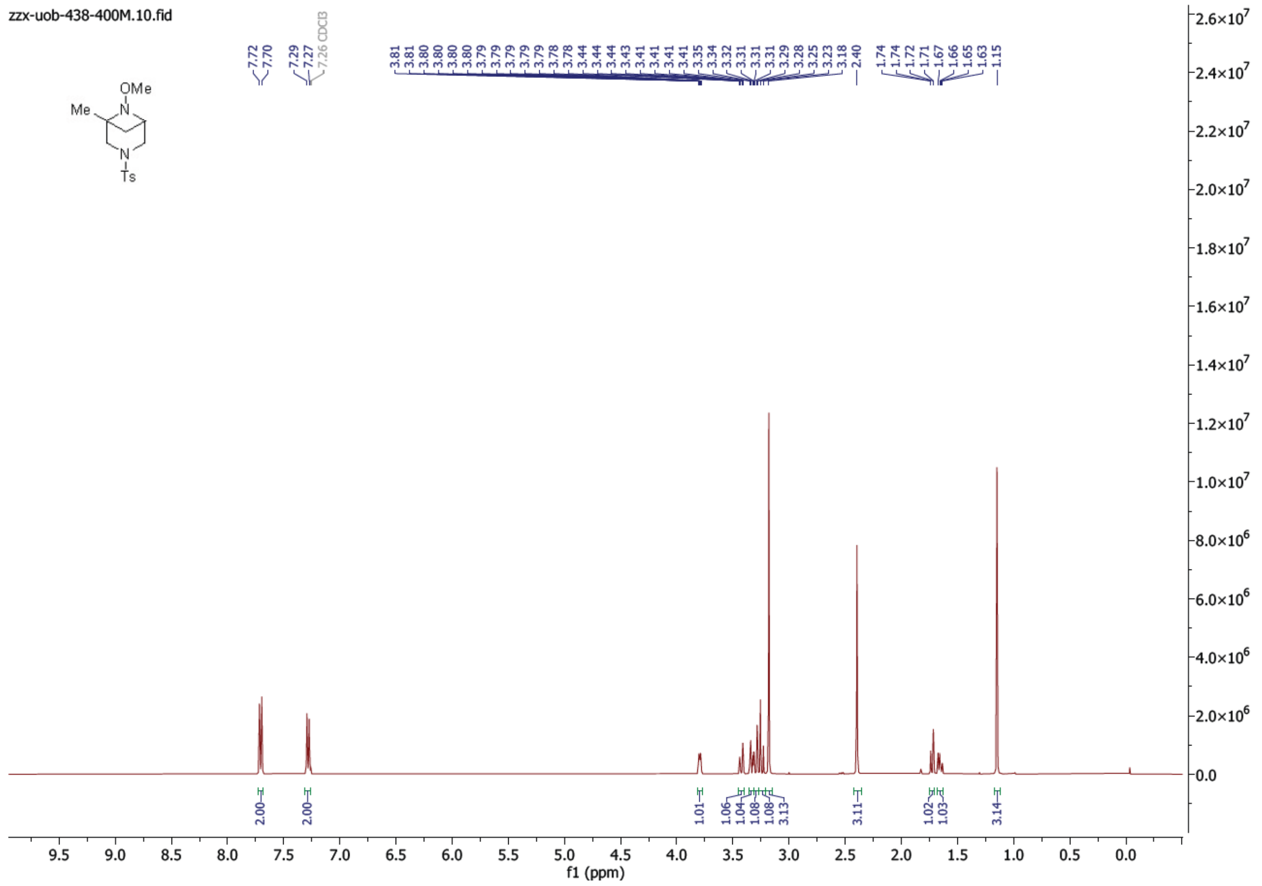
zzx-uob-438-400M.10.fid
OMe
Me
N
N
Ts
f1 (ppm)

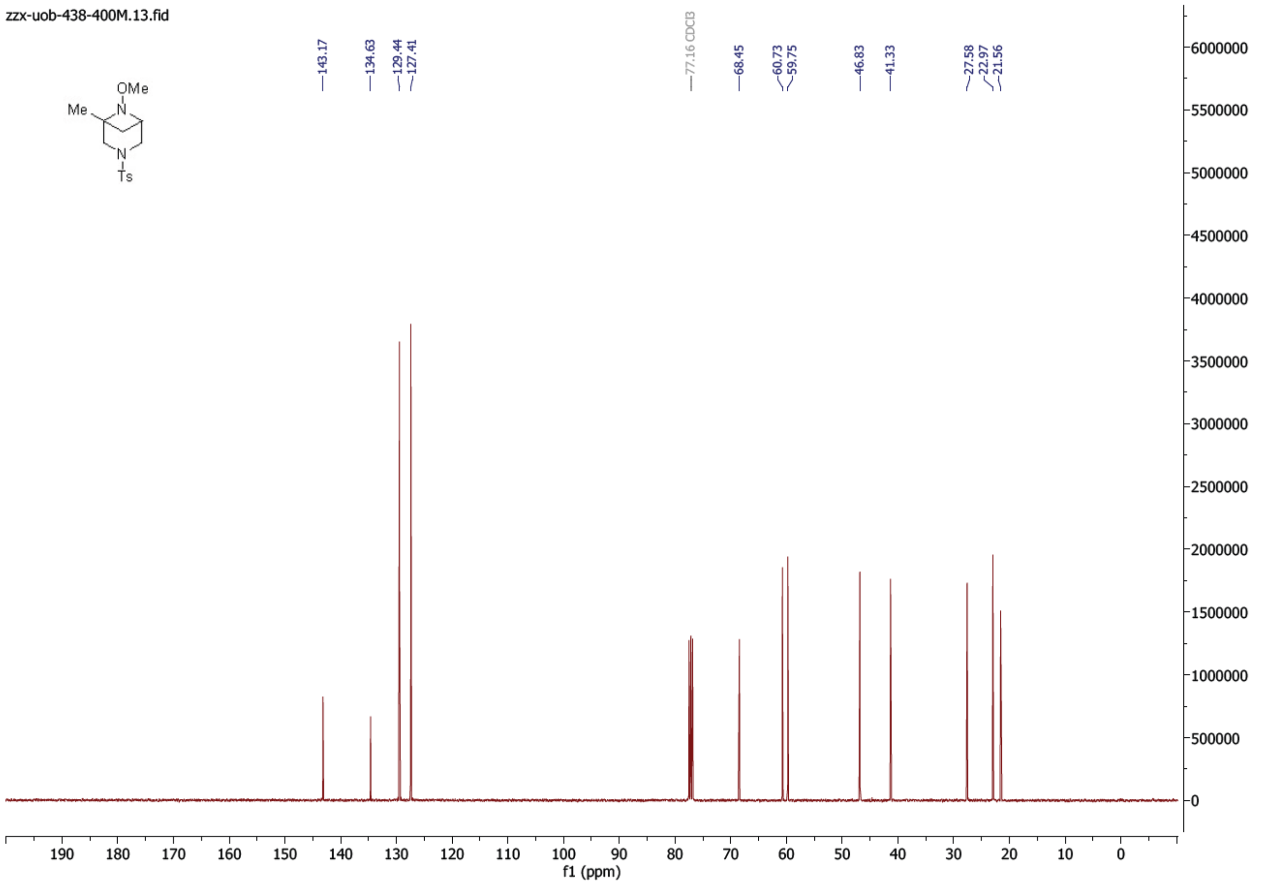
zzx-uob-438-400M.13.fid
OMe
Me
N
N
Ts
f1 (ppm)

Compound 58

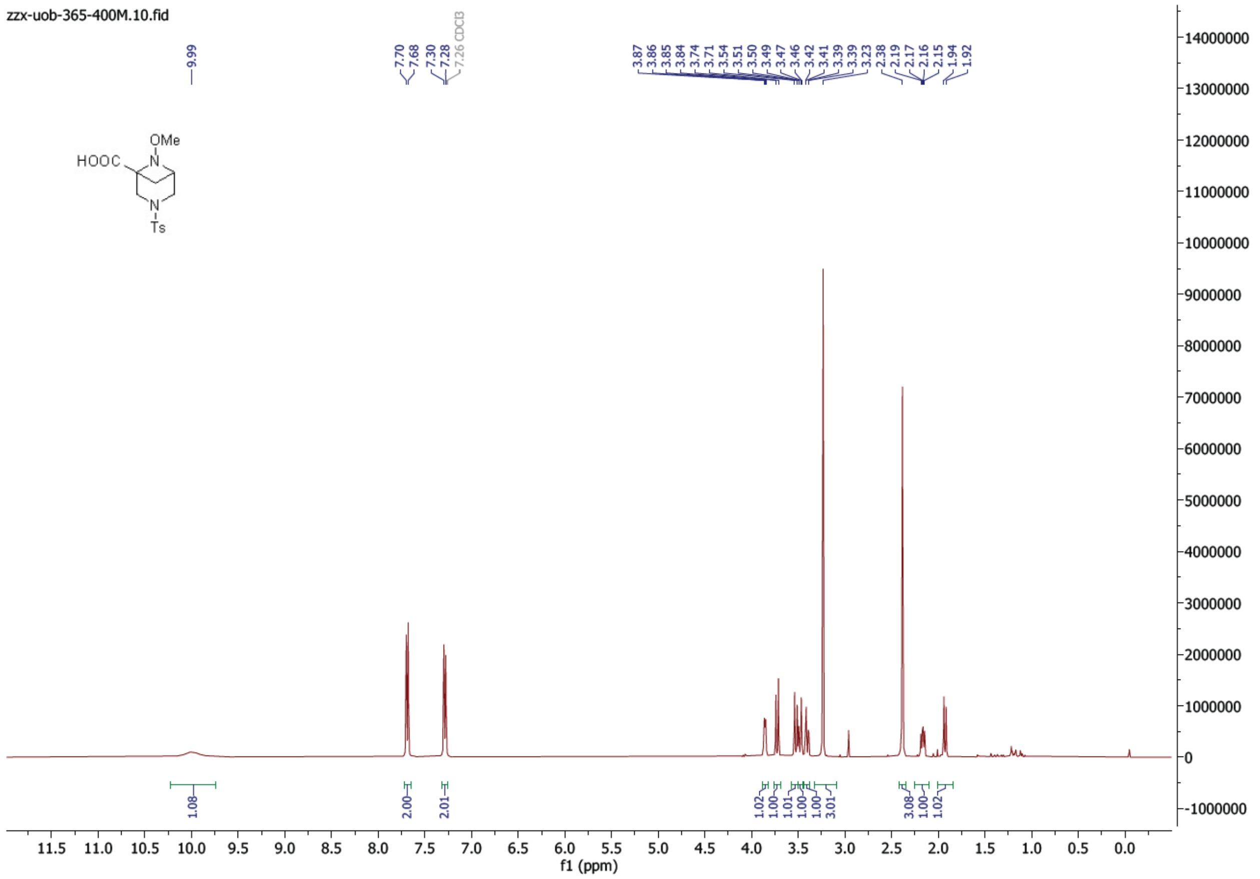

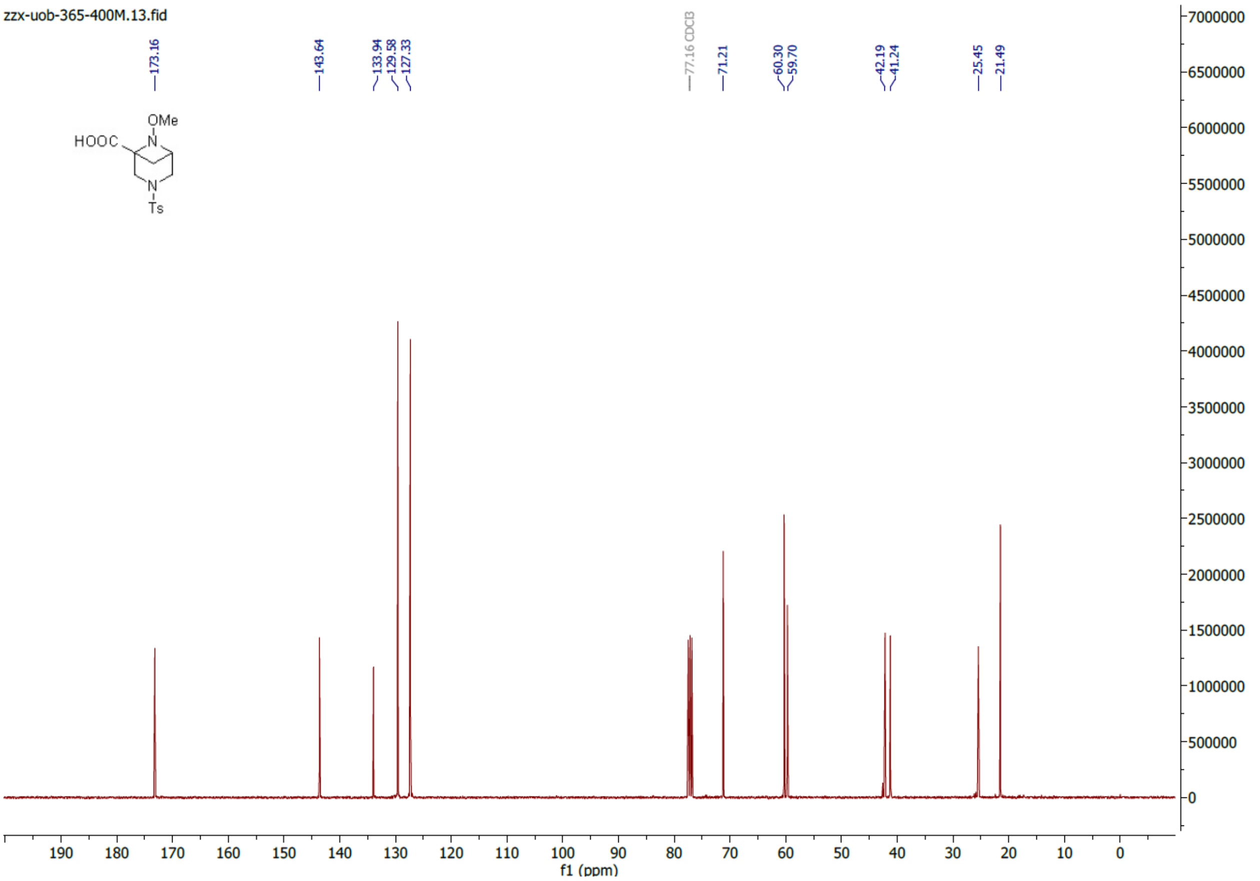

Compound 59

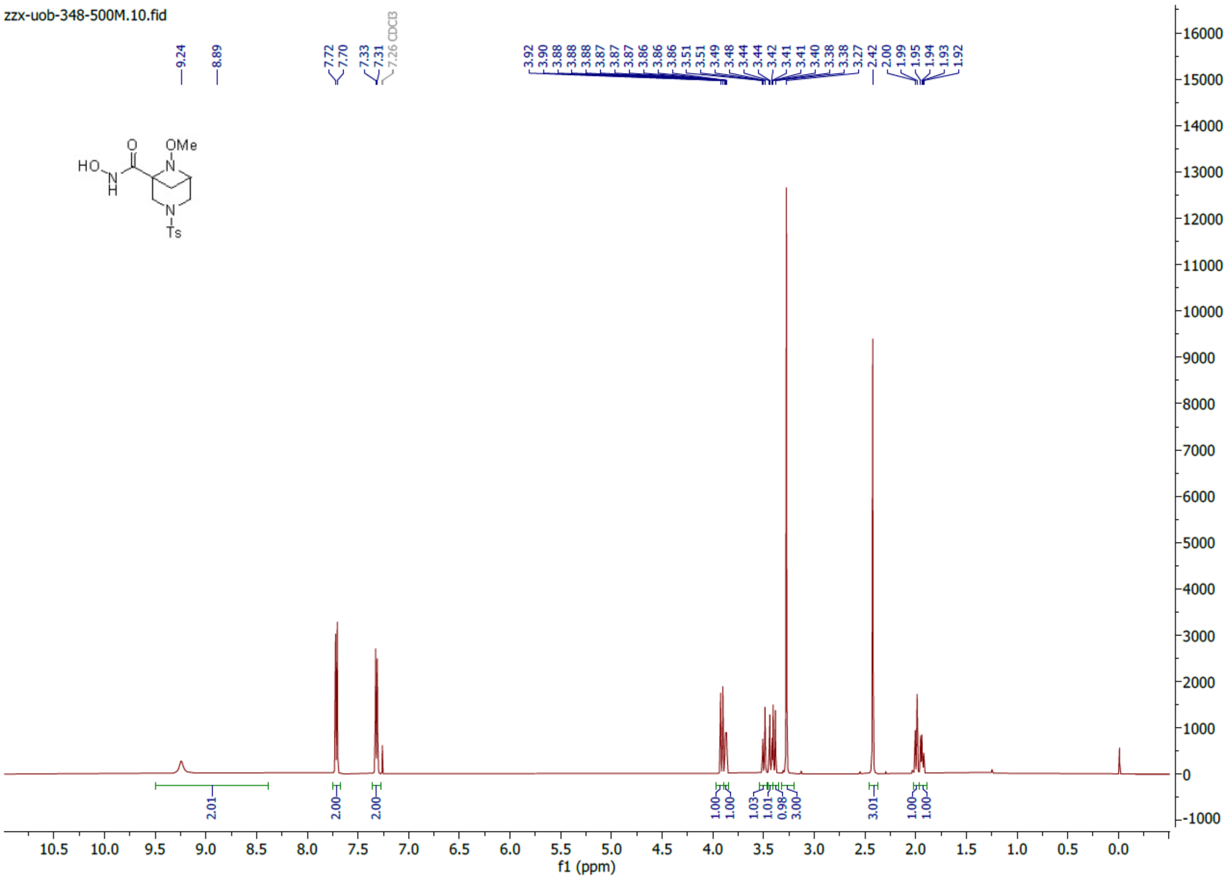
zzx-uob-348-500M.10.fid
9.24
8.89
7.72
7.70
7.33
7.31
7.26 CDCl3
3.92
3.90
3.88
3.88
3.88
3.87
3.87
3.87
3.86
3.86
3.86
3.51
3.51
3.49
3.48
3.44
3.44
3.42
3.41
3.41
3.40
3.38
3.38
3.27
2.42
2.00
1.99
1.95
1.94
1.93
1.92
HO
N
H
O
OMe
N
N
Ts
2.01
2.00
2.00
1.00
1.00
1.03
1.01
0.98
3.00
3.01
1.00
1.00
f1 (ppm)

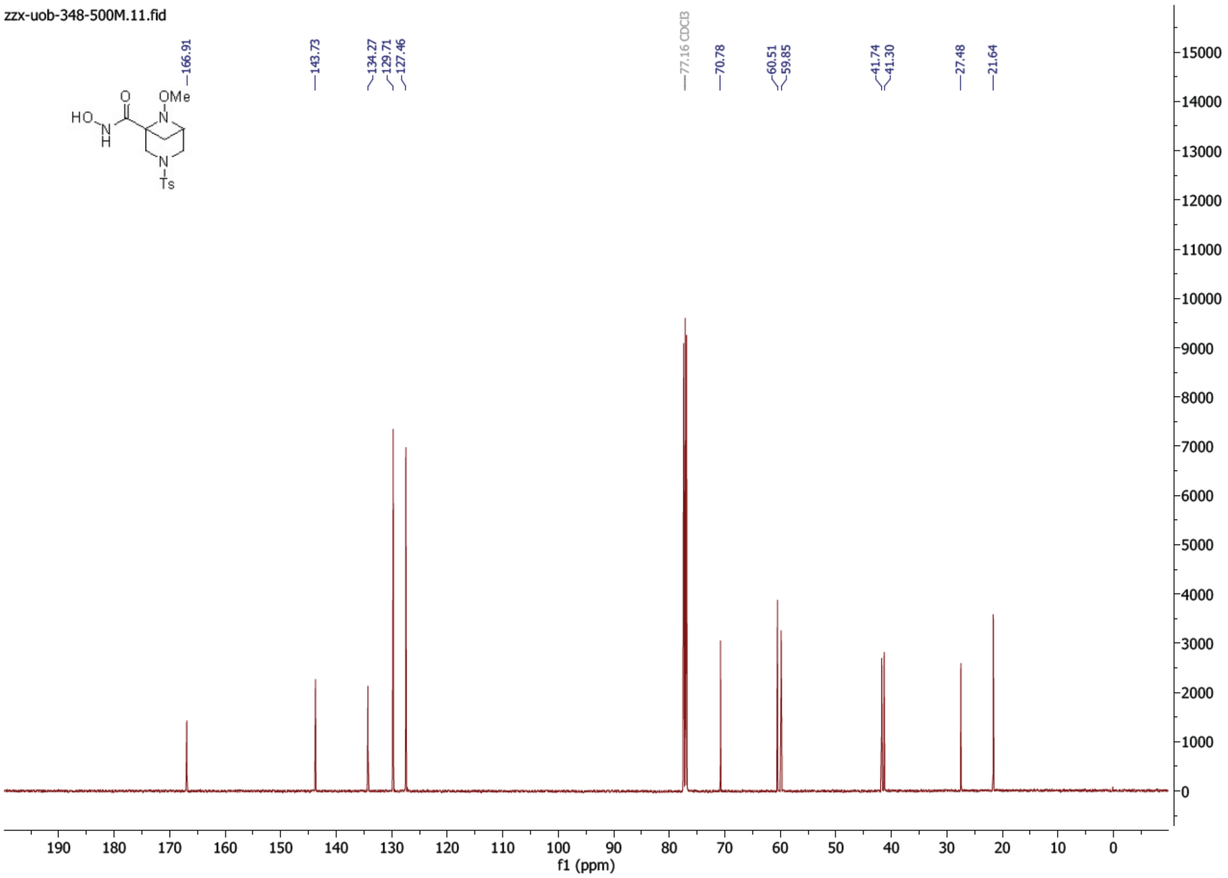
zzx-uob-348-500M.11.fid
166.91
143.73
134.27
129.71
127.46
77.16 CDCl3
70.78
60.51
59.85
41.74
41.30
27.48
21.64
HO
N
H
O
OMe
N
N
Ts
f1 (ppm)

Compound 60’

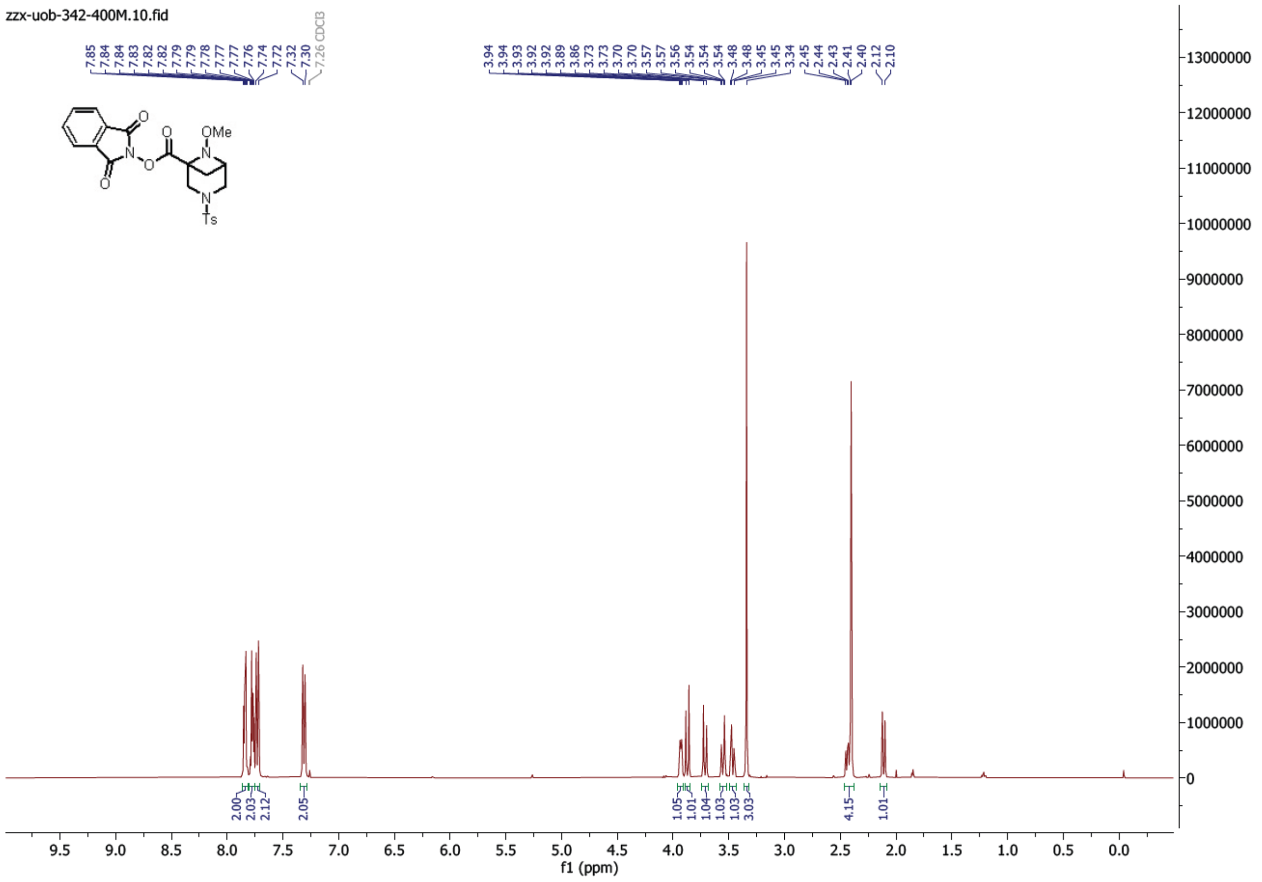

zzx-uob-342-400M.10.fid
7.85 7.84 7.83 7.82 7.82 7.79 7.79 7.78 7.77 7.77 7.76 7.74 7.72 7.32 7.30 7.26 CDCl3
3.94 3.94 3.93 3.92 3.92 3.89 3.86 3.73 3.73 3.70 3.70 3.57 3.57 3.56 3.54 3.54 3.54 3.48 3.48 3.45 3.45 3.34 2.45 2.44 2.43 2.41 2.40 2.12 2.10
OMe
N
O
Ts
2.00 2.03 2.12 2.05 1.05 1.01 1.04 1.03 1.03 3.03 4.15 1.01
f1 (ppm)

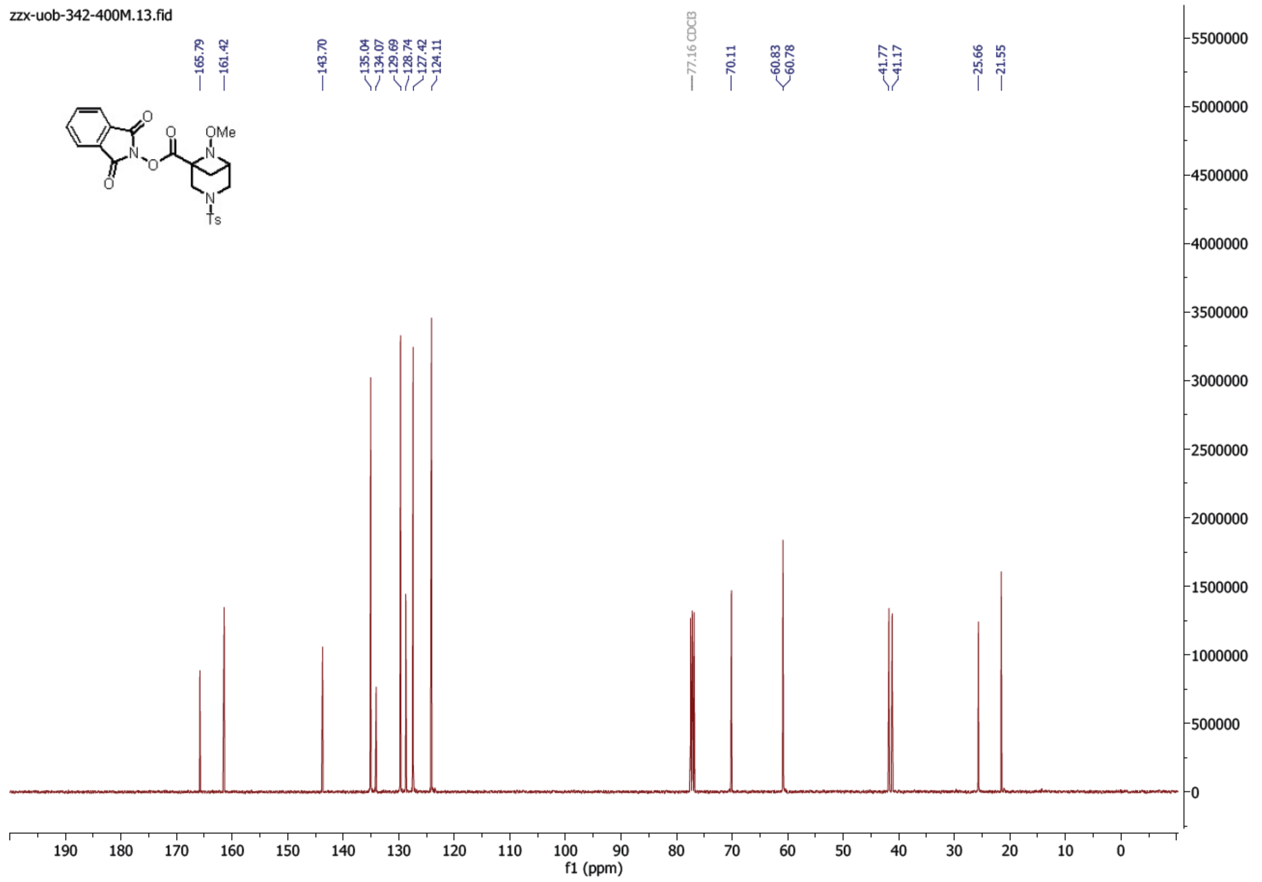

zzx-uob-342-400M.13.fid
165.79 161.42 143.70 135.04 134.07 129.69 128.74 127.42 124.11 77.16 CDCl3 70.11 60.83 60.78 41.77 41.17 25.66 21.55
OMe
N
O
Ts
f1 (ppm)

Compound 60

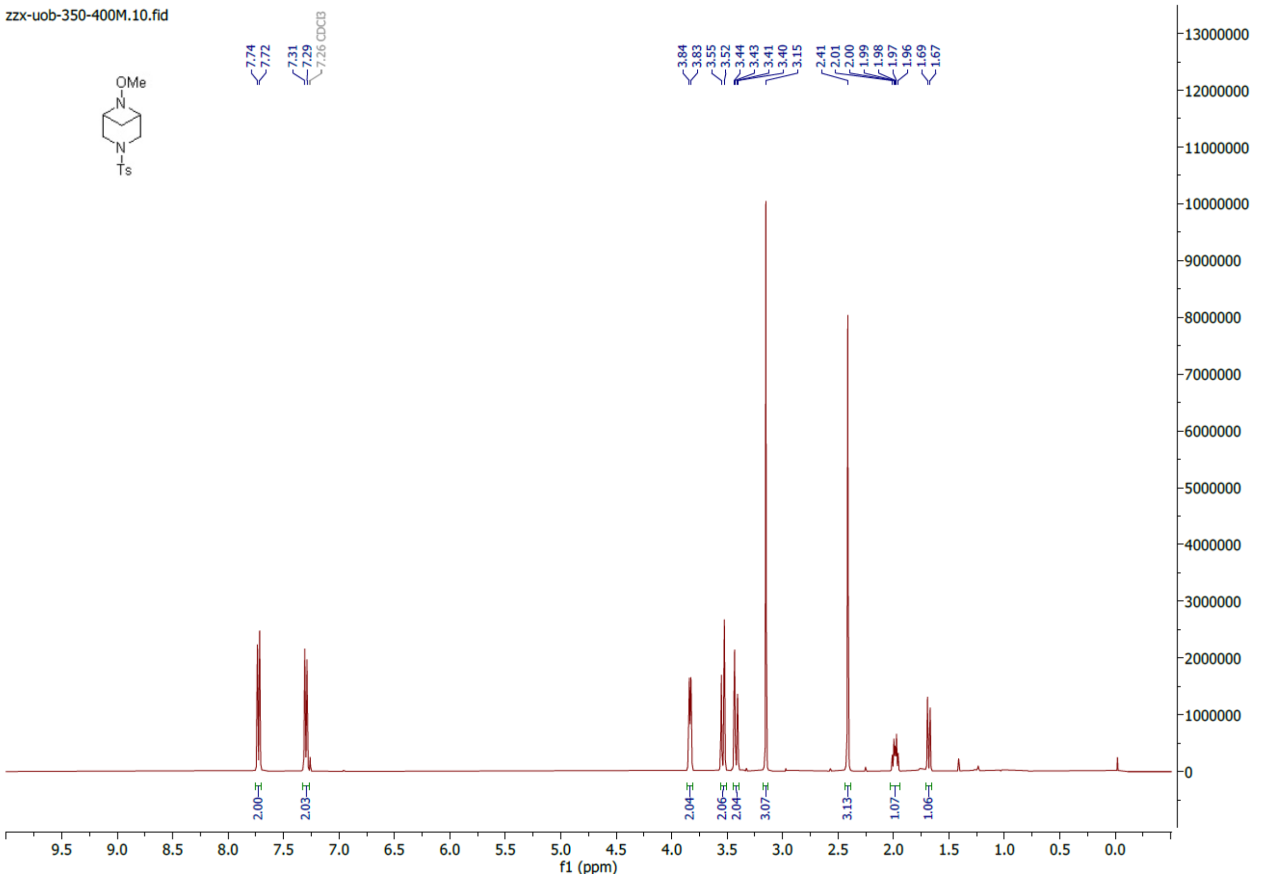
zzx-uob-350-400M.10.fid
OMe
N
N
Ts
7.74
7.72
7.31
7.29
7.26 CDCl3
3.84
3.83
3.55
3.52
3.44
3.43
3.41
3.40
3.15
2.41
2.01
2.00
1.99
1.98
1.97
1.96
1.69
1.67
2.00
2.03
2.04
2.06
2.04
3.07
3.13
1.07
1.06
f1 (ppm)

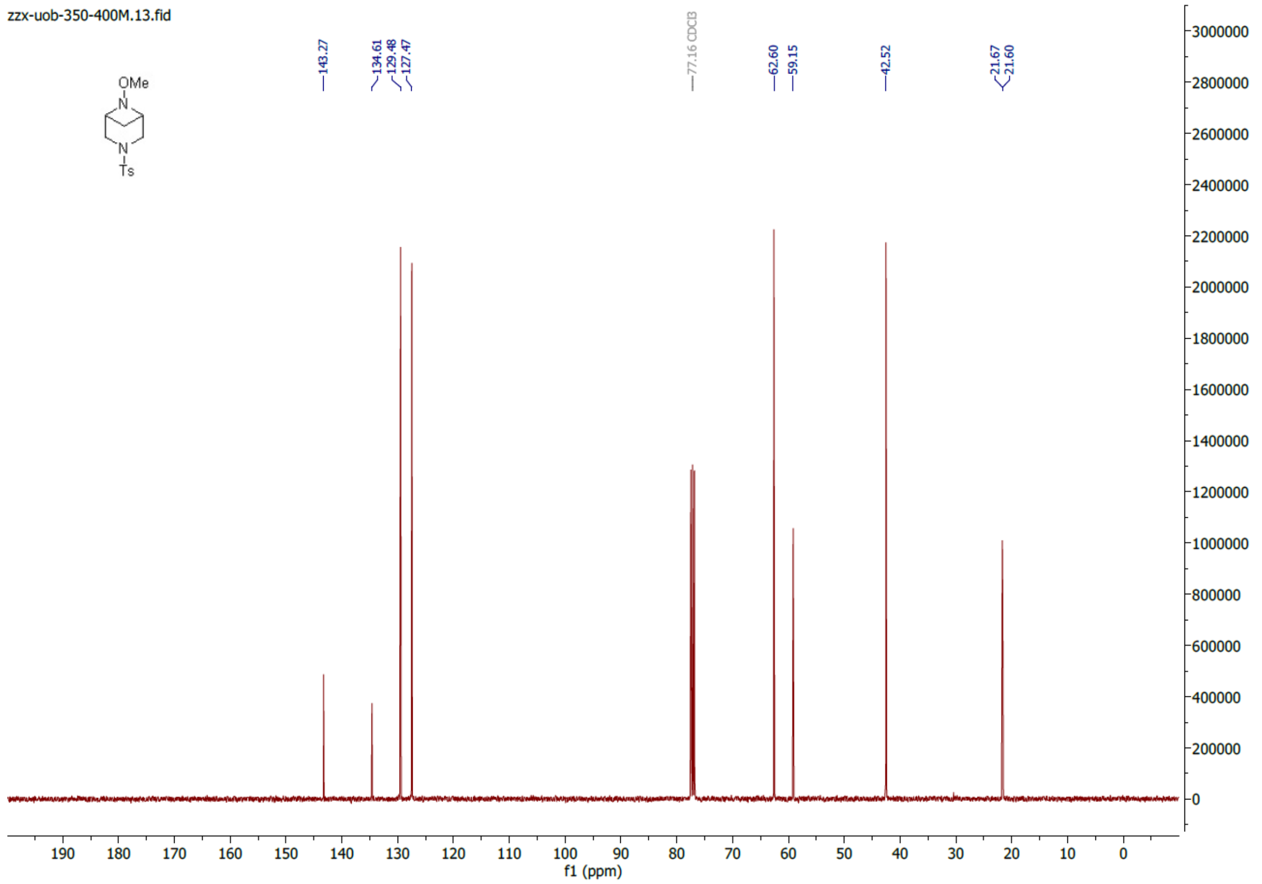
zzx-uob-350-400M.13.fid
OMe
N
N
Ts
143.27
134.61
129.48
127.47
77.16 CDCl3
62.60
59.15
42.52
21.67
21.60
f1 (ppm)

Compound 61

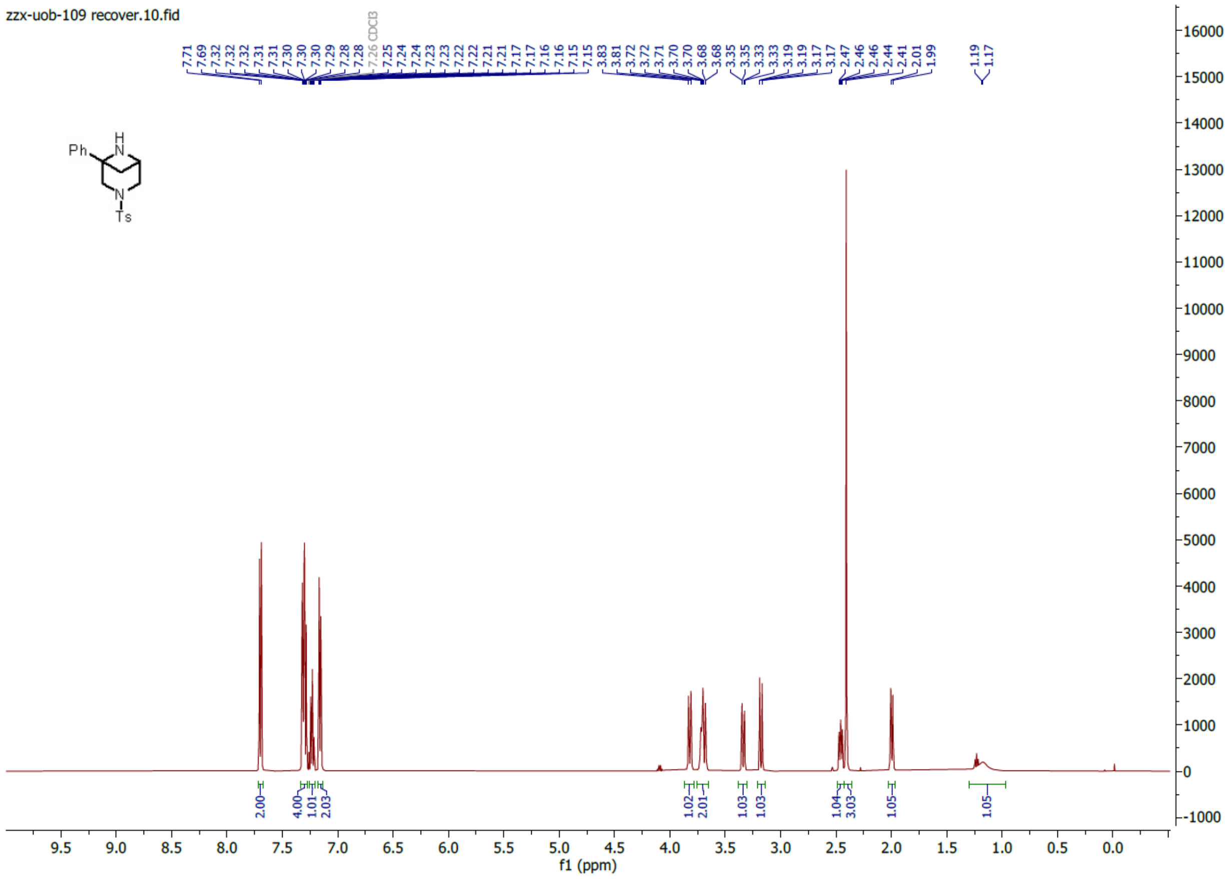
zzx-uob-109 recover.10.fid
f1 (ppm)

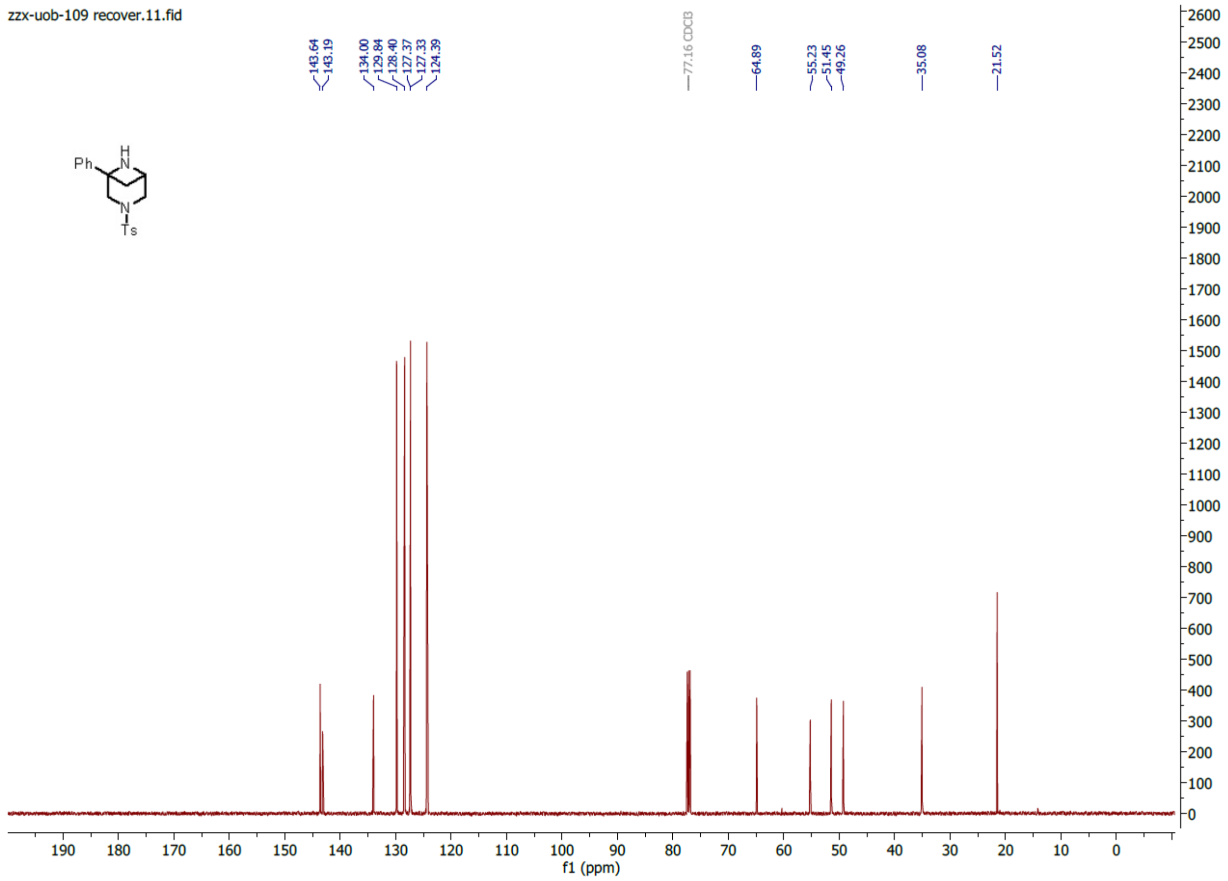
zzx-uob-109 recover.11.fid
f1 (ppm)

Compound 62

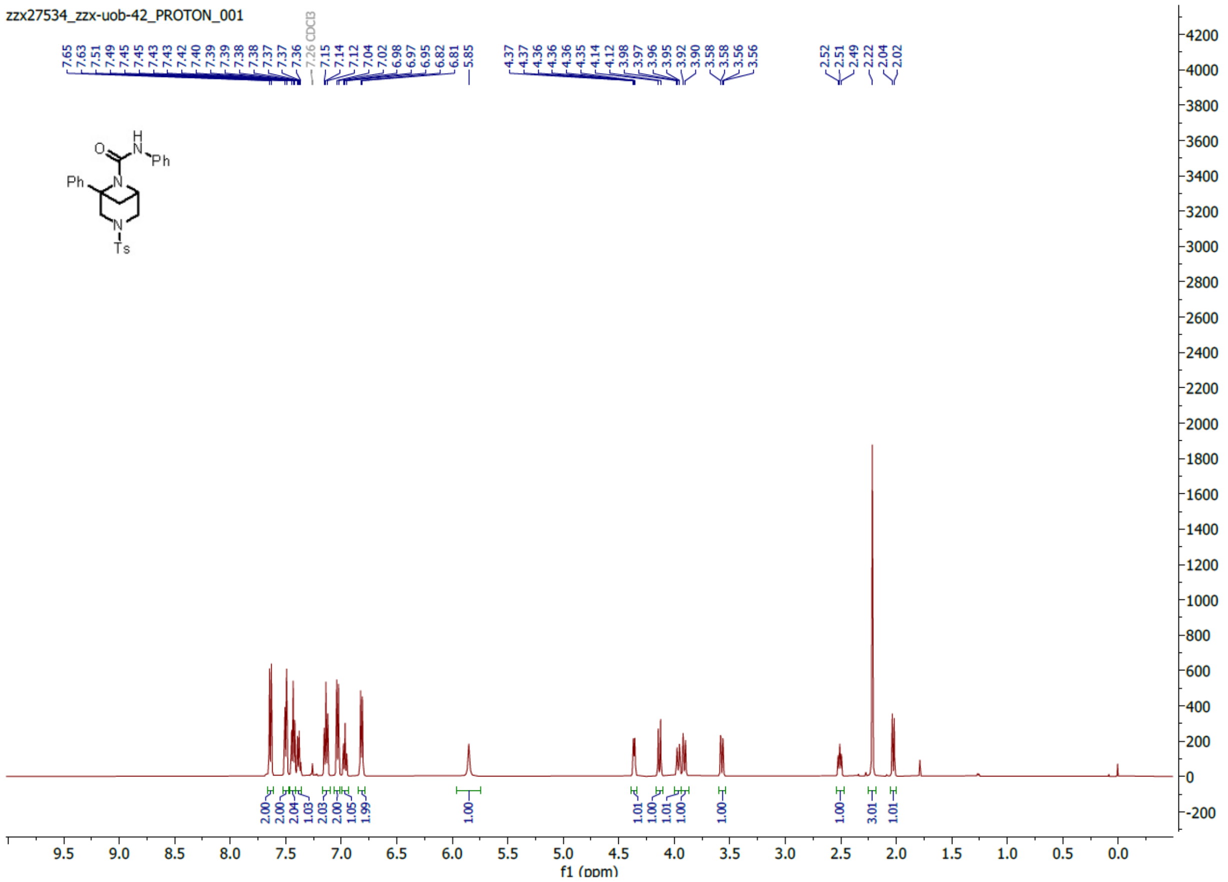
zzx27534_zzx-uob-42_PROTON_001
f1 (ppm)

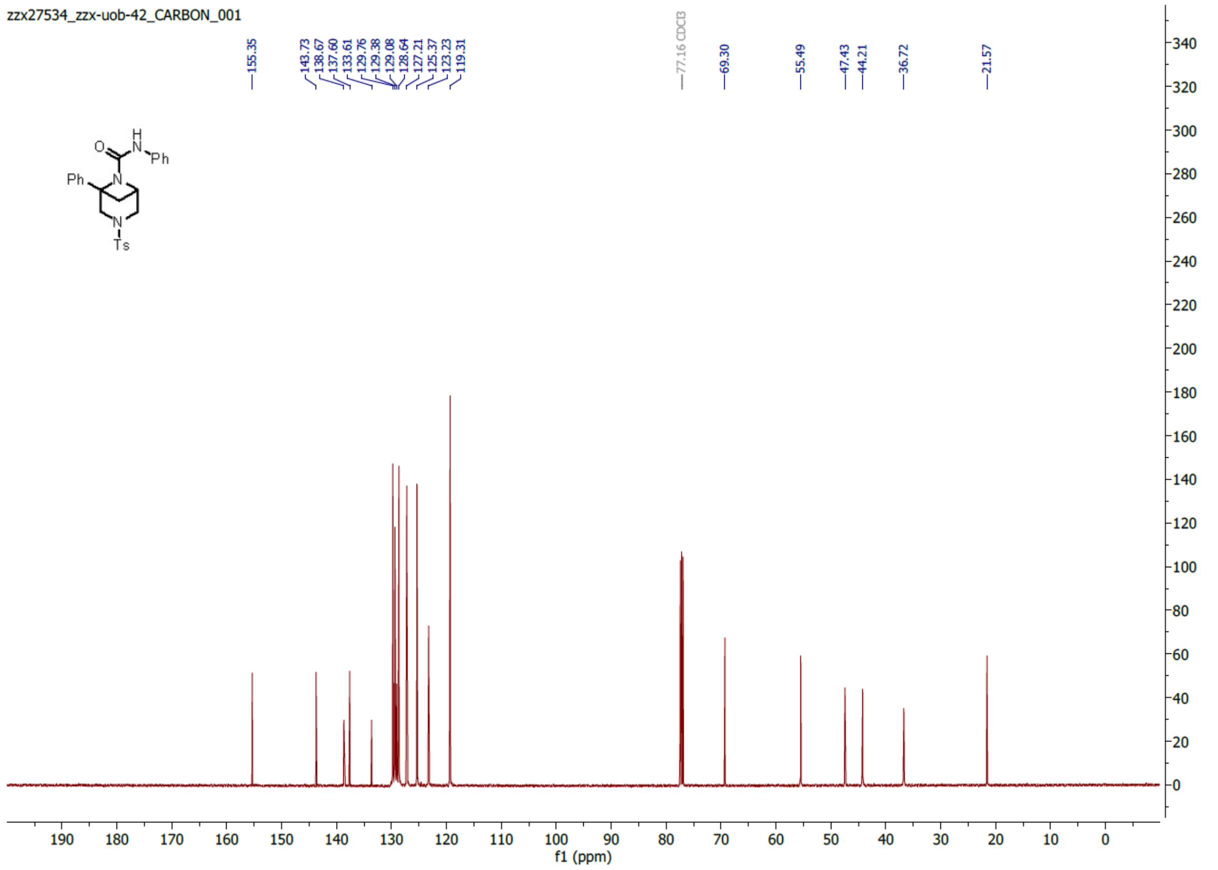
zzx27534_zzx-uob-42_CARBON_001
f1 (ppm)

Compound 63

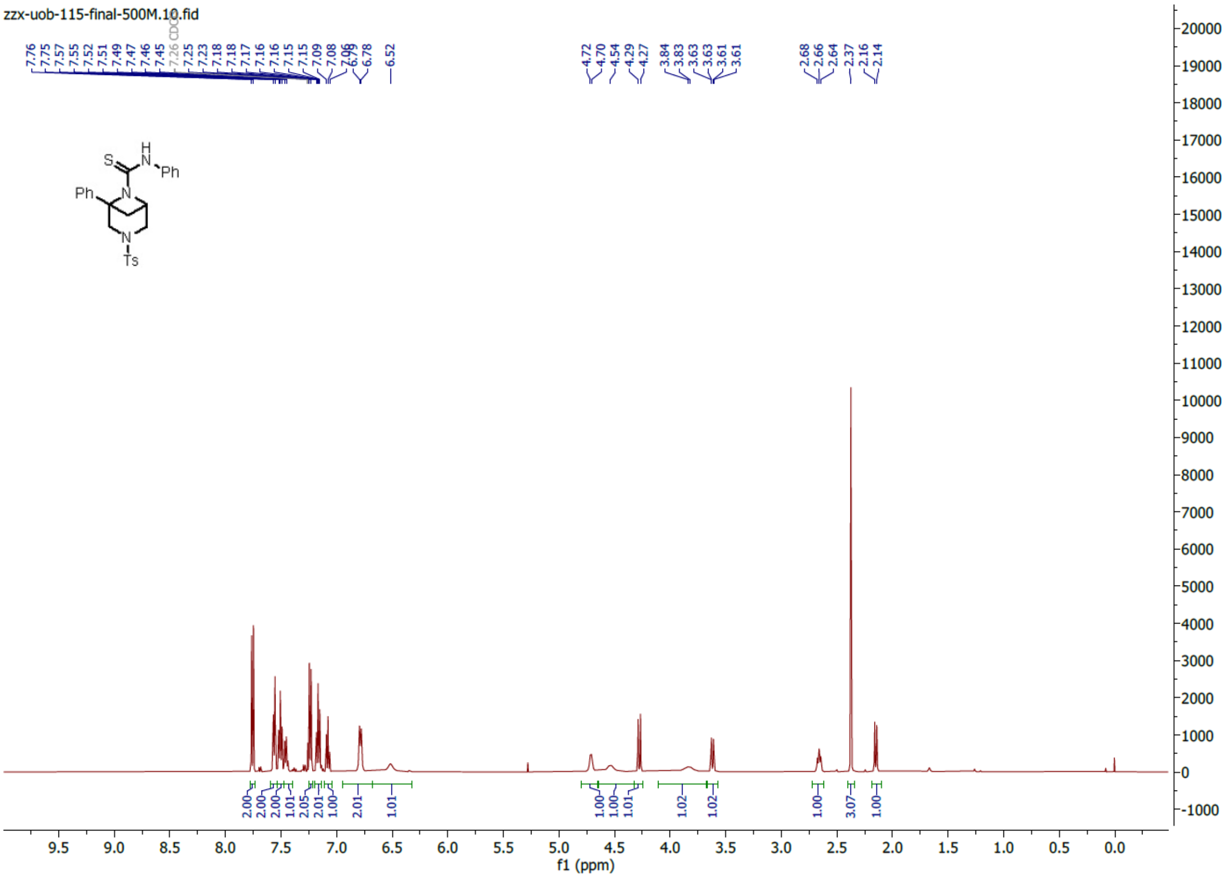
zzx-uob-115-final-500M.10.fid
f1 (ppm)

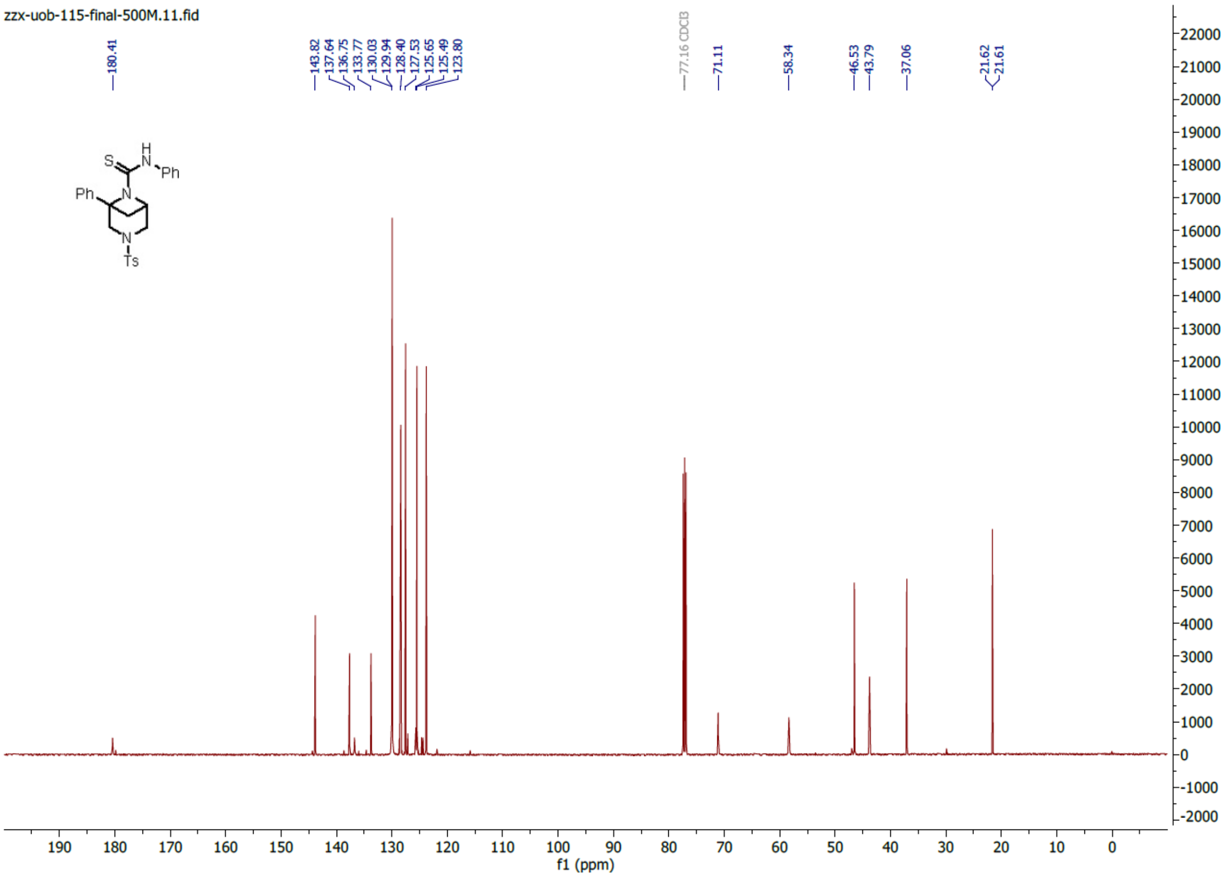
zzx-uob-115-final-500M.11.fid
f1 (ppm)

Compound 64

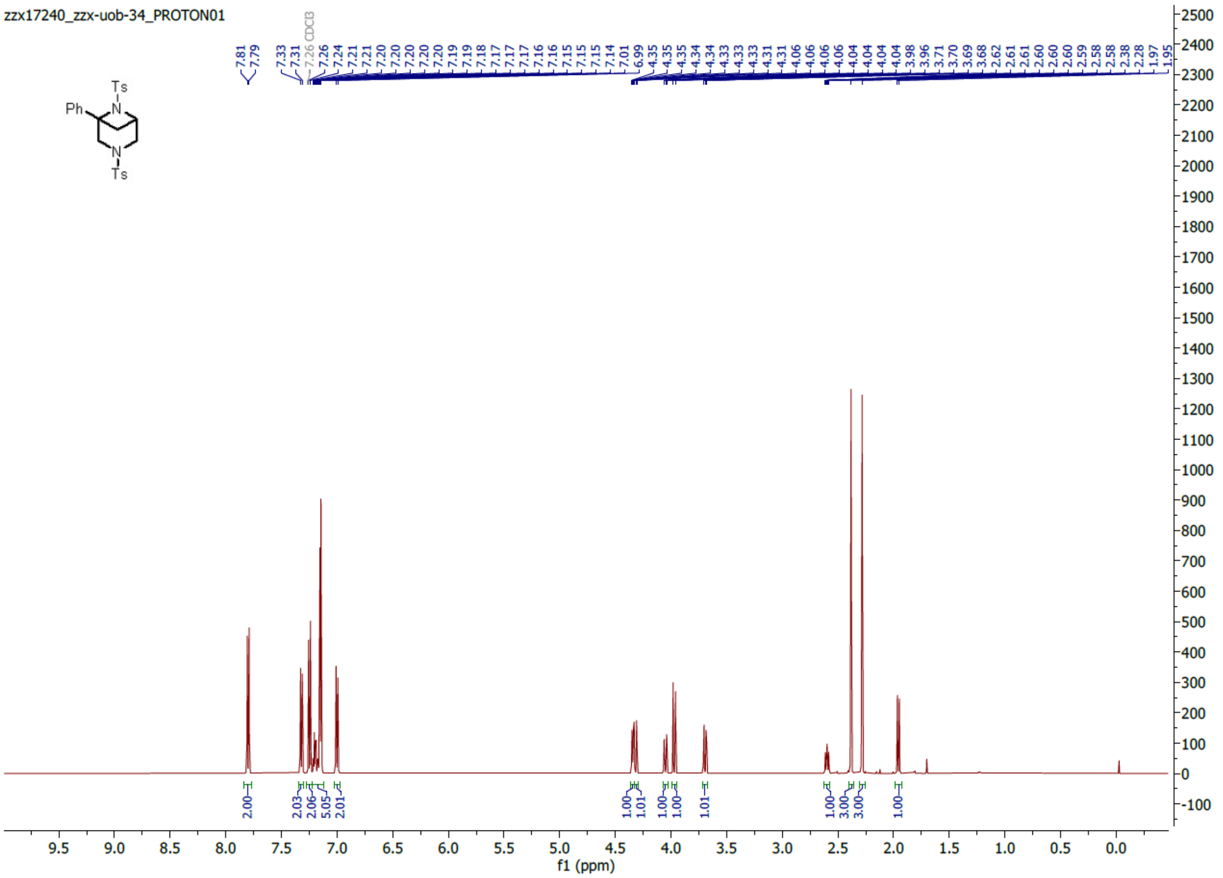
zzx17240_zzx-uob-34_PROTON01
7.81
7.79
7.33
7.31
7.26 CDCl3
7.26
7.24
7.21
7.21
7.20
7.20
7.20
7.20
7.20
7.19
7.19
7.18
7.17
7.17
7.17
7.16
7.15
7.15
7.15
7.14
7.01
6.99
4.35
4.35
4.35
4.34
4.34
4.33
4.33
4.33
4.31
4.31
4.06
4.06
4.06
4.04
4.04
4.04
4.04
3.98
3.96
3.71
3.70
3.69
3.68
2.62
2.61
2.61
2.60
2.60
2.60
2.59
2.58
2.58
2.38
2.28
1.97
1.95
Ts
Ph
N
N
Ts
2.00
2.03
2.06
5.05
2.01
1.00
1.01
1.00
1.00
1.01
1.00
3.00
3.00
1.00
f1 (ppm)

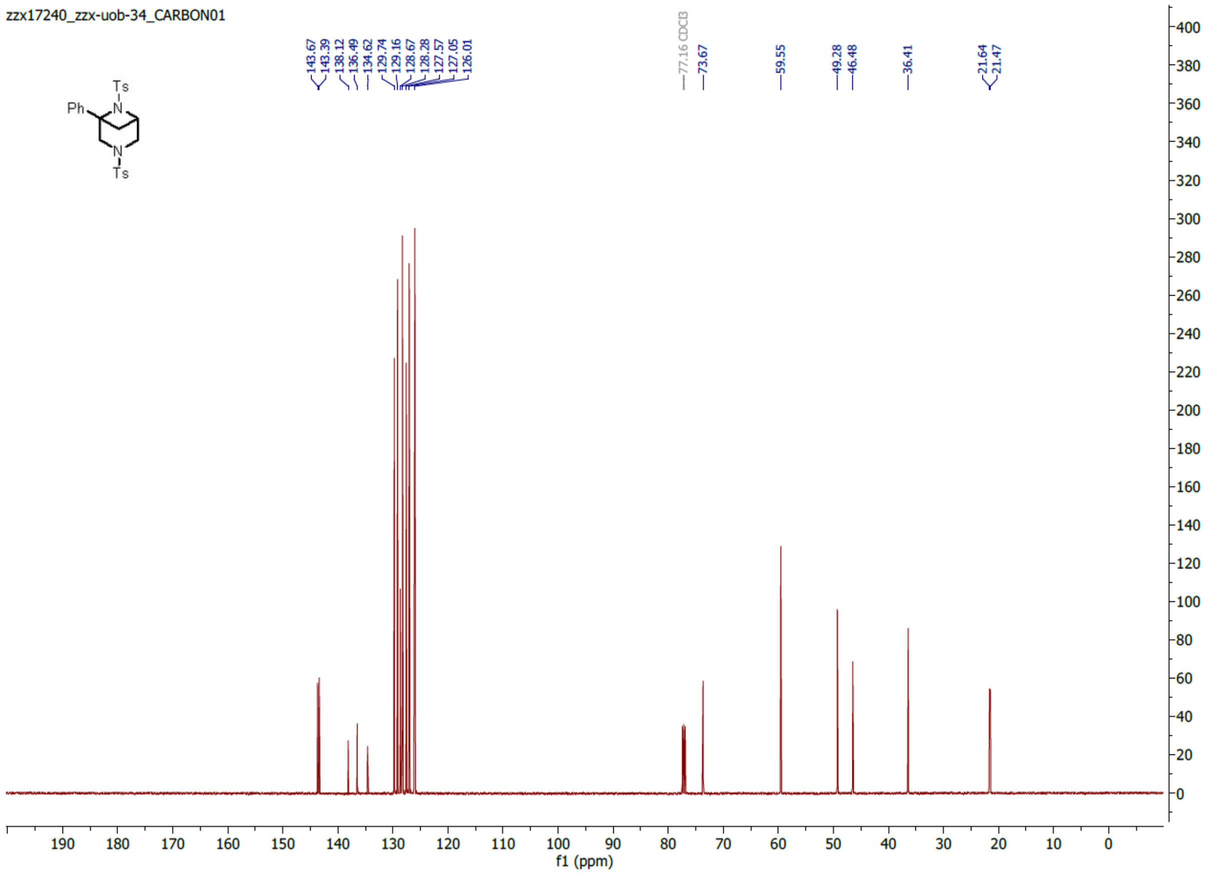
zzx17240_zzx-uob-34_CARBON01
143.67
143.39
138.12
136.49
134.62
129.74
129.16
128.67
128.28
127.57
127.05
126.01
77.16 CDCl3
73.67
59.55
49.28
46.48
36.41
21.64
21.47
Ts
Ph
N
N
Ts
f1 (ppm)

Compound 65

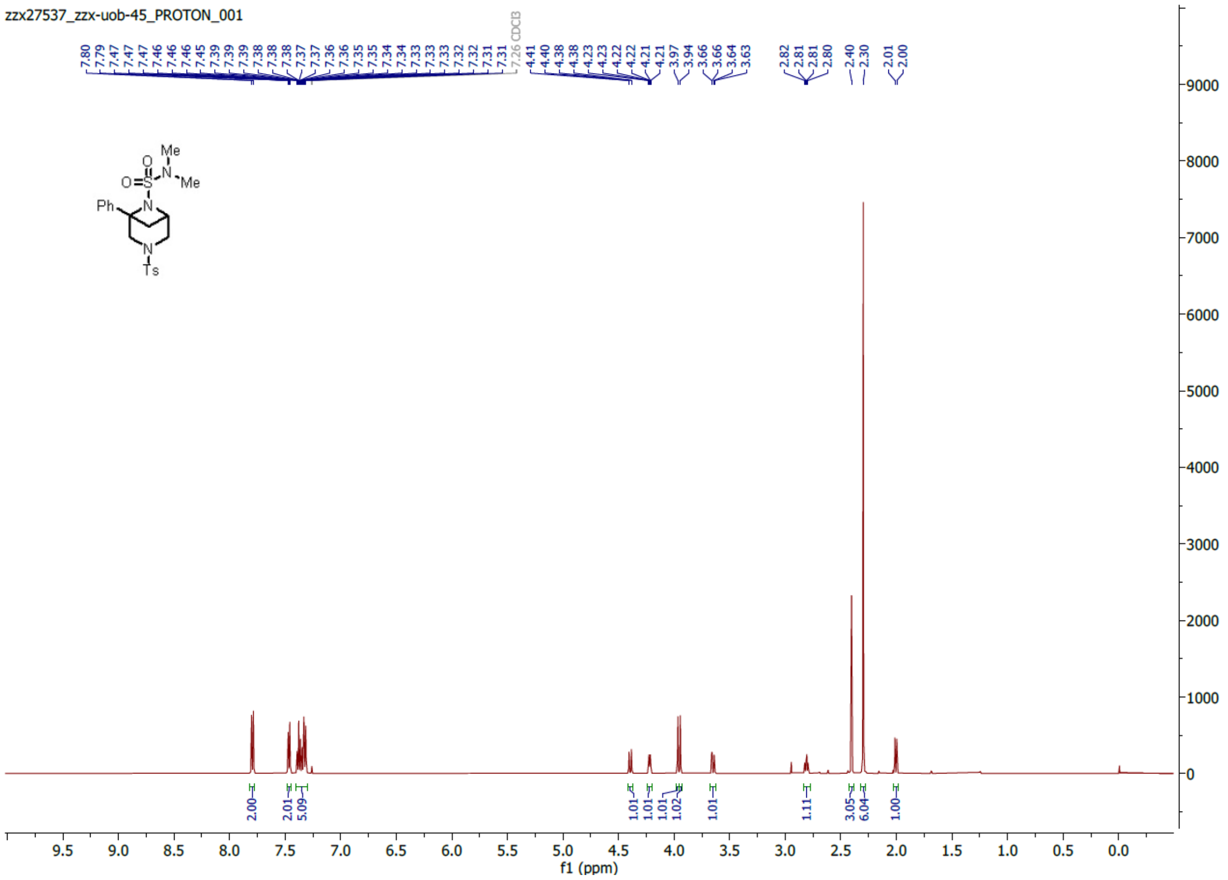

zzx27537_zzx-uob-45_PROTON_001
7.80 7.79 7.47 7.47 7.47 7.46 7.46 7.46 7.45 7.39 7.39 7.39 7.38 7.38 7.38 7.37 7.37 7.36 7.36 7.35 7.35 7.34 7.34 7.33 7.33 7.33 7.32 7.32 7.31 7.31
7.26 CDCl3
4.41 4.40 4.38 4.38 4.23 4.23 4.22 4.22 4.21 4.21 3.97 3.94 3.66 3.66 3.64 3.63
2.82 2.81 2.81 2.80 2.40 2.30 2.01 2.00
Me
O
O=S
N
Me
Ph
N
N
Ts
2.00 2.01 5.09 1.01 1.01 1.01 1.02 1.01 1.11 3.05 6.04 1.00
f1 (ppm)

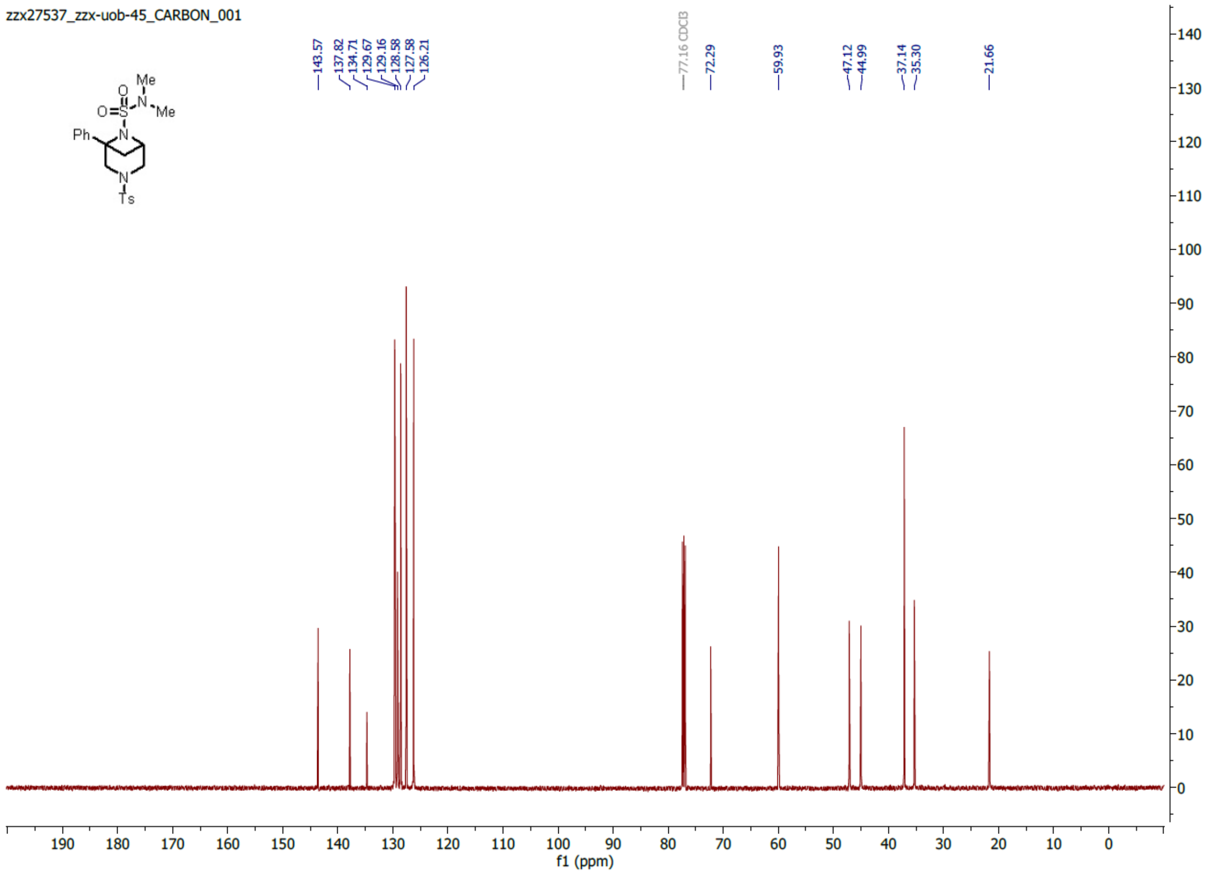

zzx27537_zzx-uob-45_CARBON_001
143.57 137.82 134.71 129.67 129.16 128.58 127.58 126.21
77.16 CDCl3
72.29 59.93 47.12 44.99 37.14 35.30 21.66
Me
O
O=S
N
Me
Ph
N
N
Ts
f1 (ppm)

Compound 66

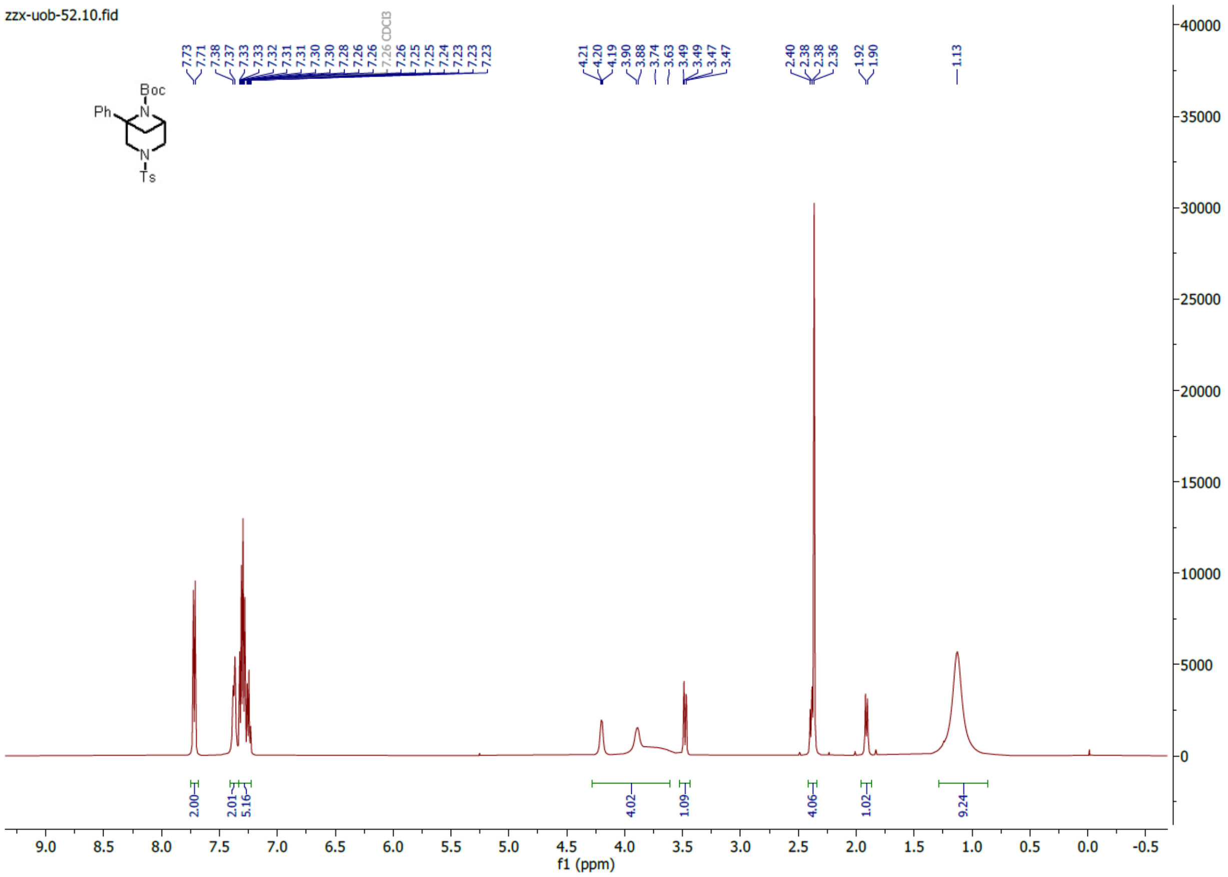
zzx-uob-52.10.fid
7.73
7.71
7.38
7.37
7.33
7.33
7.32
7.31
7.31
7.30
7.30
7.28
7.26
7.26
7.26 CDCl3
7.26
7.25
7.25
7.24
7.23
7.23
7.23
4.21
4.20
4.19
3.90
3.88
3.74
3.63
3.49
3.49
3.47
3.47
2.40
2.38
2.38
2.36
1.92
1.90
1.13
Boc
Ph
N
N
Ts
2.00
2.01
5.16
4.02
1.09
4.06
1.02
9.24
f1 (ppm)

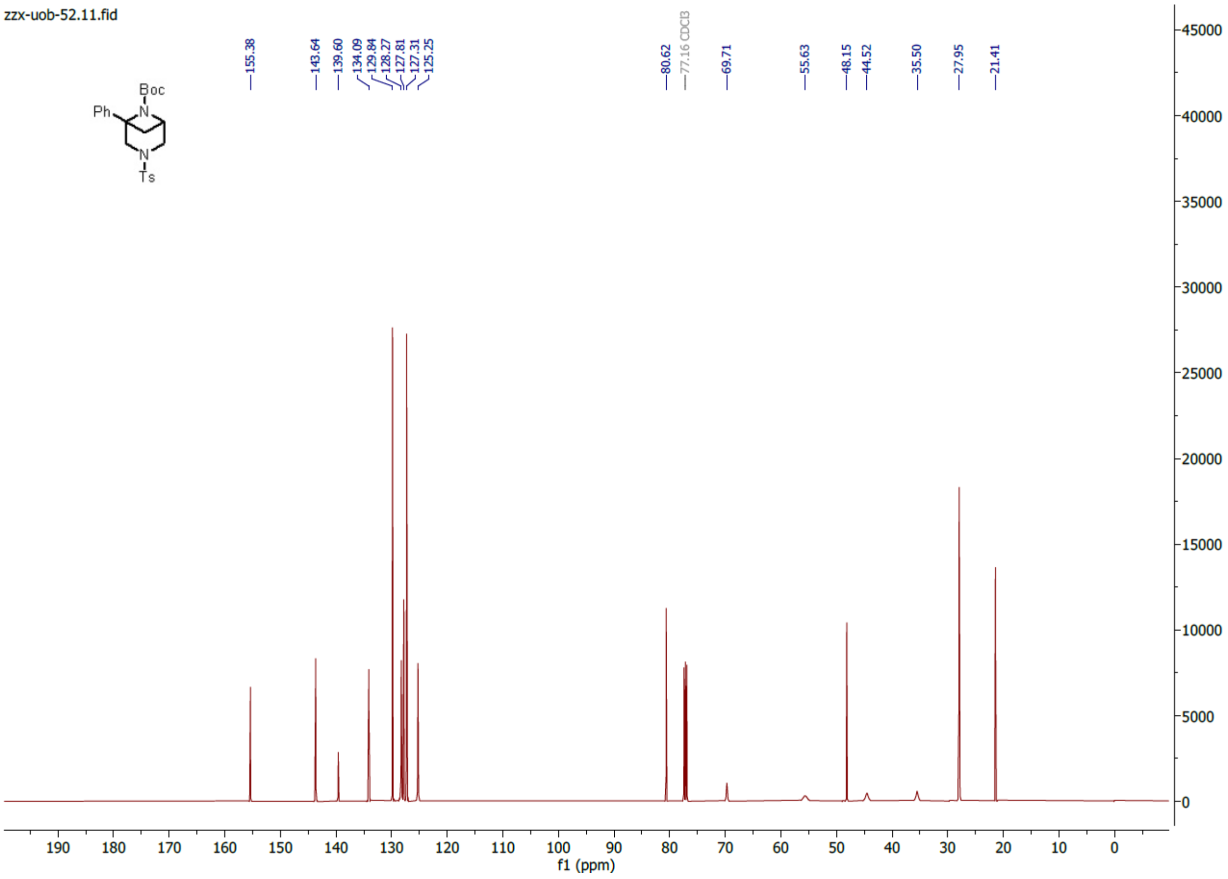
zzx-uob-52.11.fid
155.38
143.64
139.60
134.09
129.84
128.27
127.81
127.31
125.25
80.62
77.16 CDCl3
69.71
55.63
48.15
44.52
35.50
27.95
21.41
Boc
Ph
N
N
Ts
f1 (ppm)

Compound 67

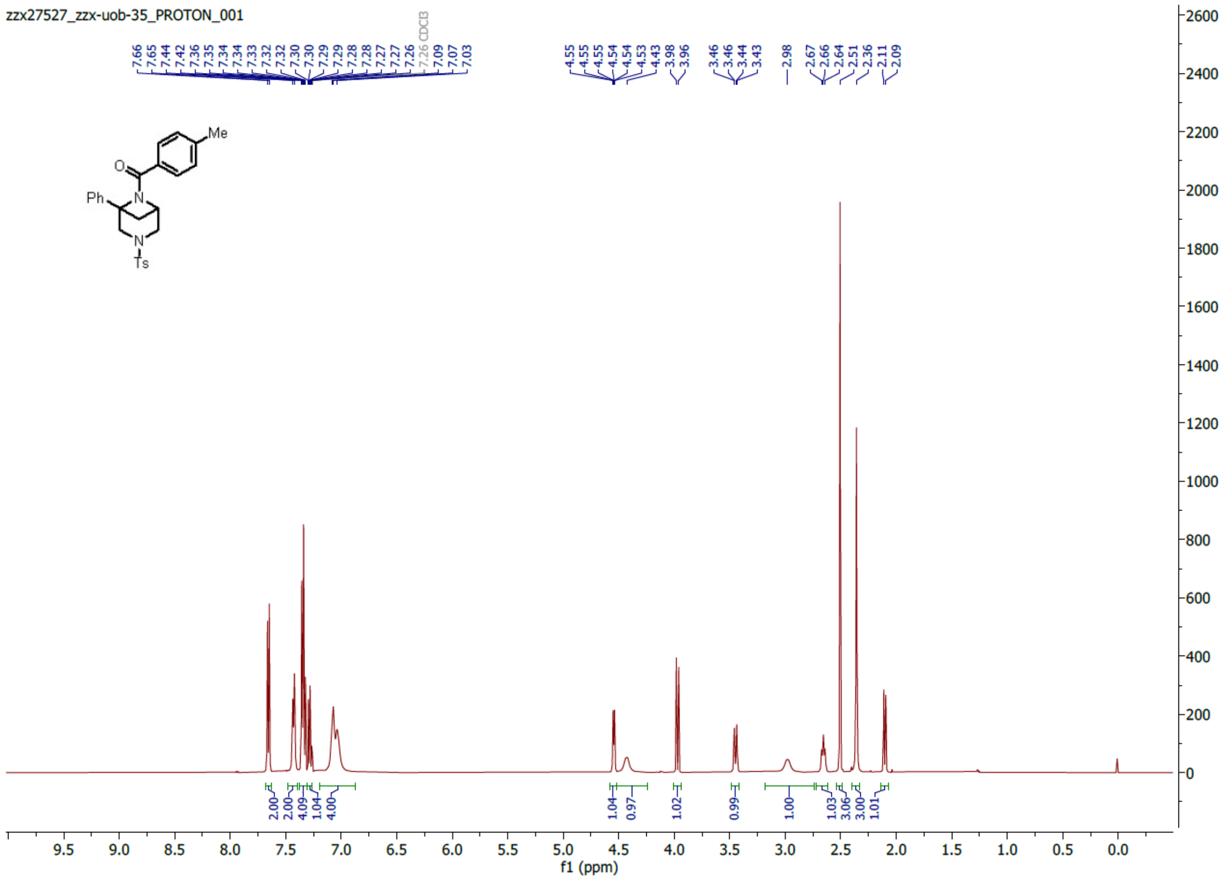
zzx27527_zzx-uob-35_PROTON_001
7.66 7.65 7.44 7.42 7.36 7.35 7.34 7.34 7.33 7.32 7.32 7.30 7.30 7.29 7.29 7.28 7.28 7.27 7.27 7.26 7.26 CDCl3 7.09 7.07 7.03
4.55 4.55 4.55 4.54 4.54 4.53 4.43 3.98 3.96
3.46 3.46 3.44 3.43
2.98 2.67 2.66 2.64 2.51 2.36 2.11 2.09
Me
O
Ph
N
N
Ts
2.00 2.00 4.09 1.04 4.00 1.04 0.97 1.02 0.99 1.00 1.03 3.06 3.00 1.01
f1 (ppm)

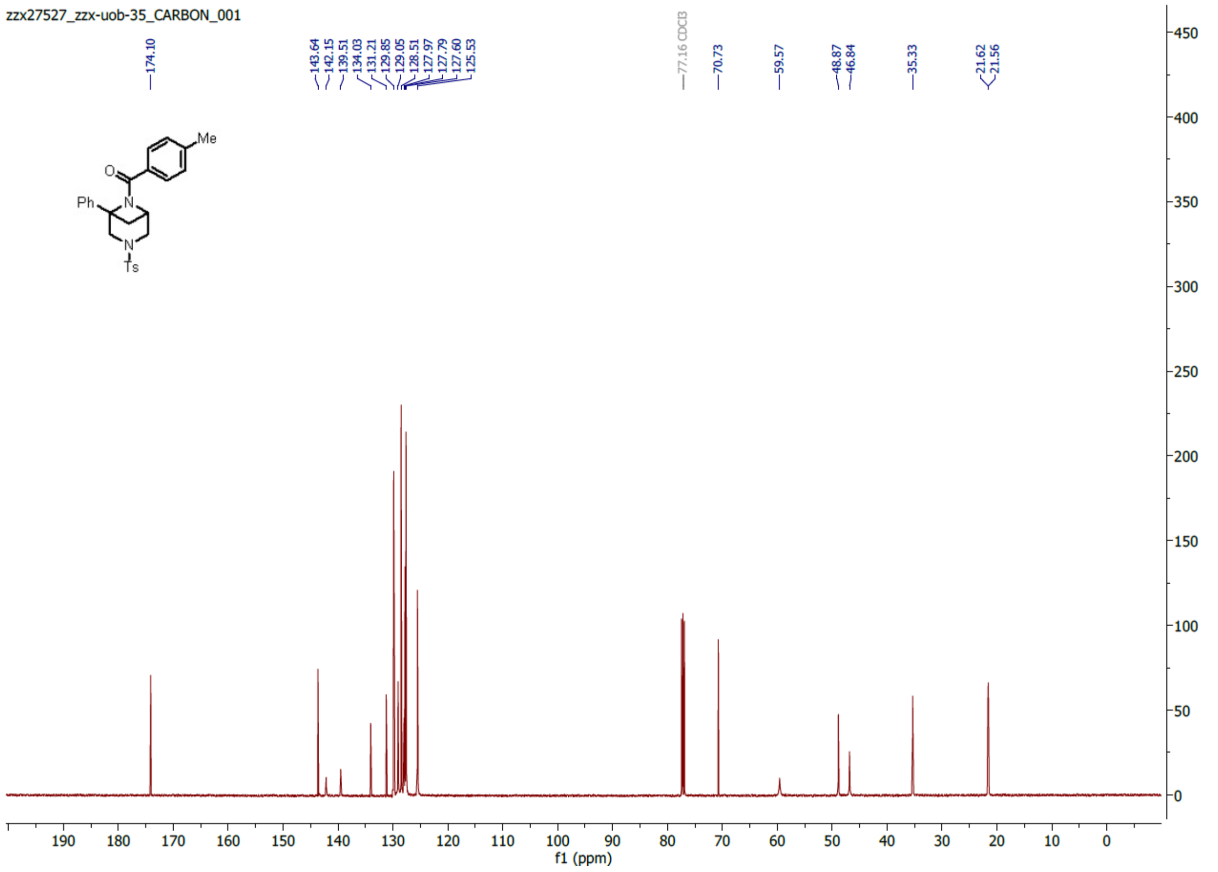
zzx27527_zzx-uob-35_CARBON_001
174.10
143.64 142.15 139.51 134.03 131.21 129.85 129.05 128.51 127.97 127.79 127.60 125.53
77.16 CDCl3
70.73
59.57
48.87 46.84
35.33
21.62 21.56
Me
O
Ph
N
N
Ts
f1 (ppm)

Compound S30a

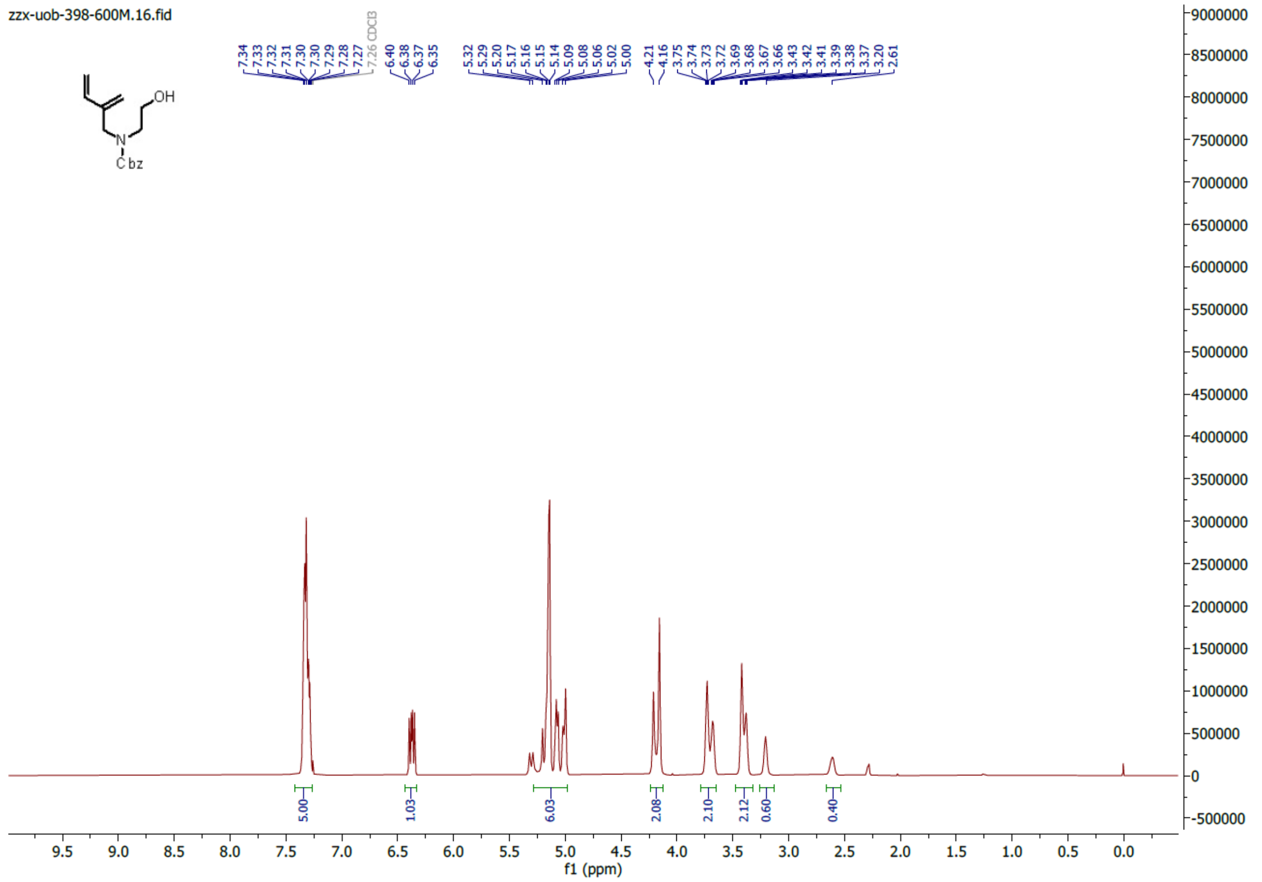

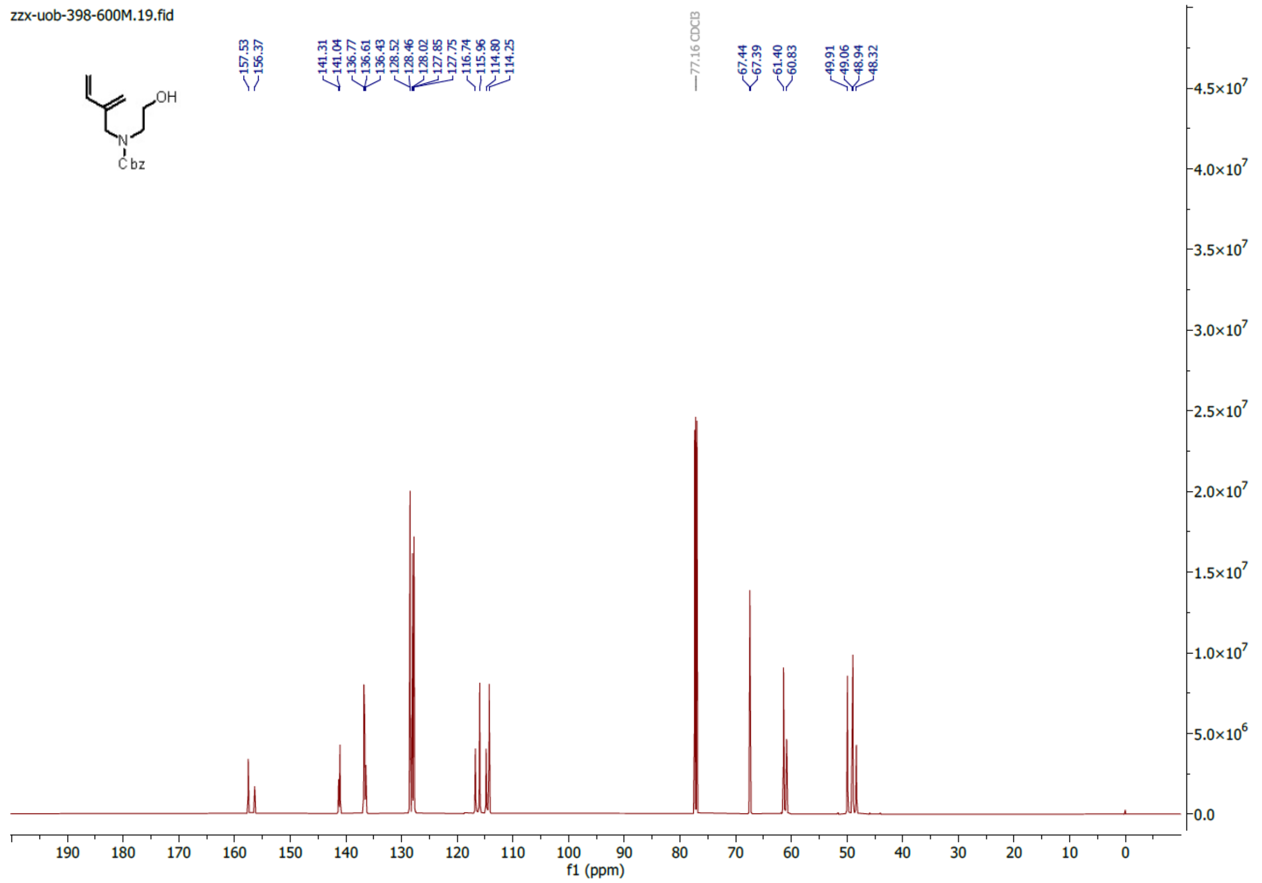

Compound S30b

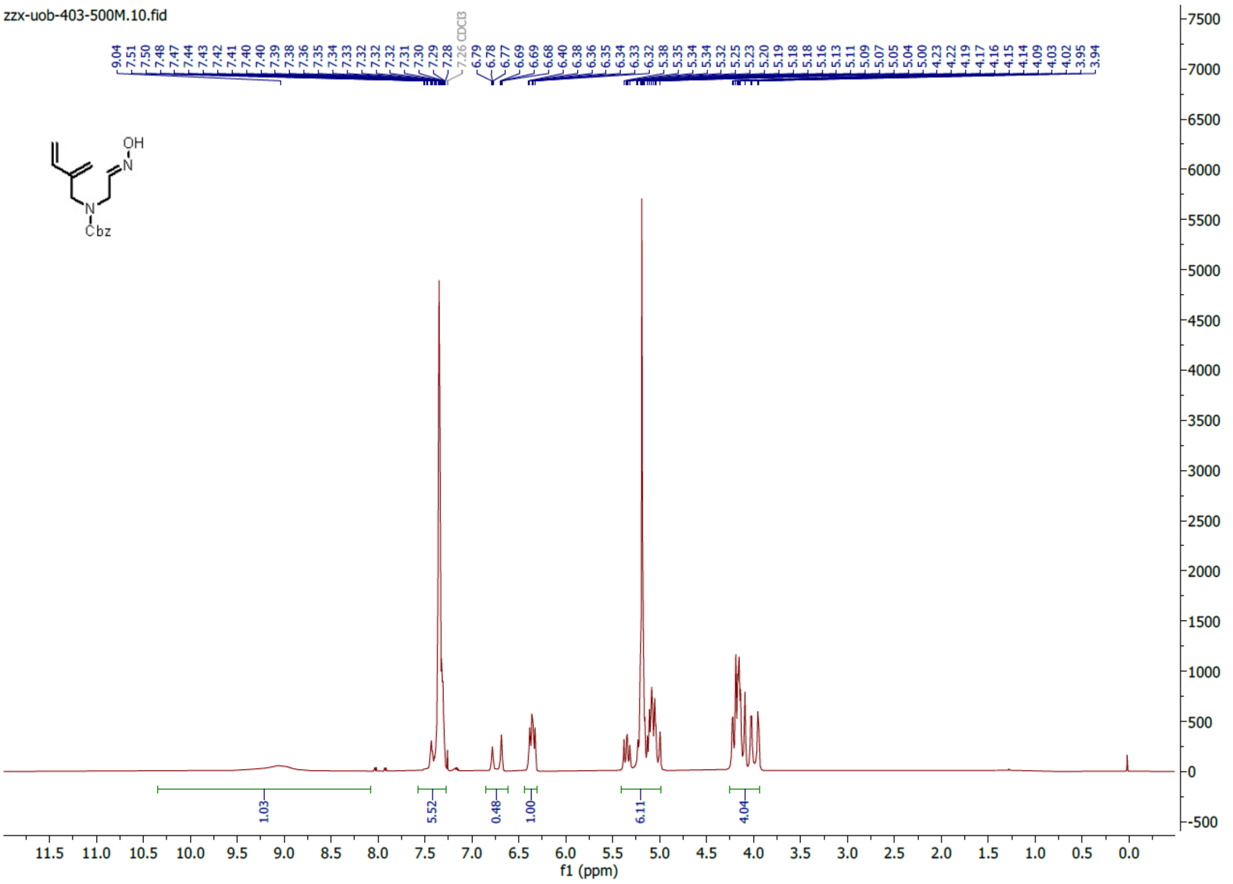

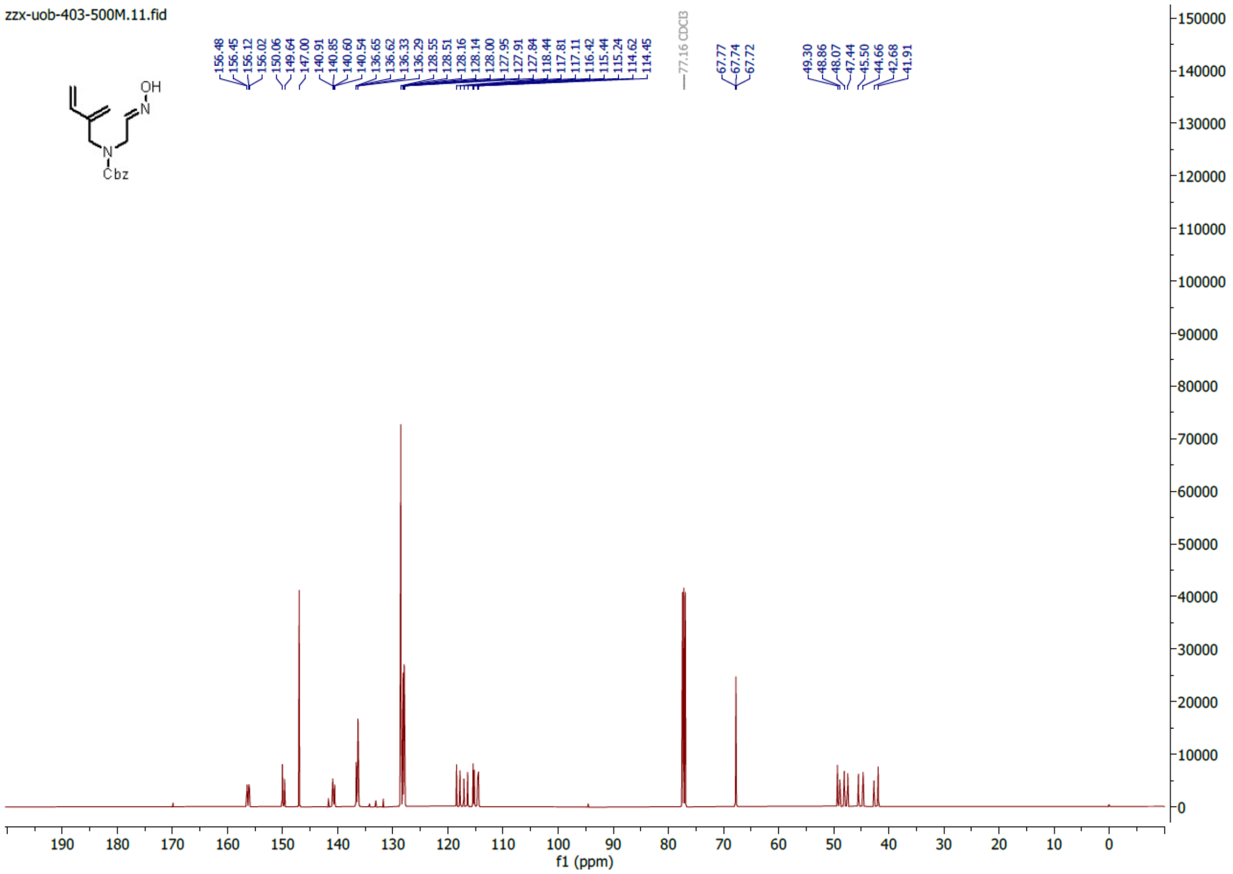

Compound 30

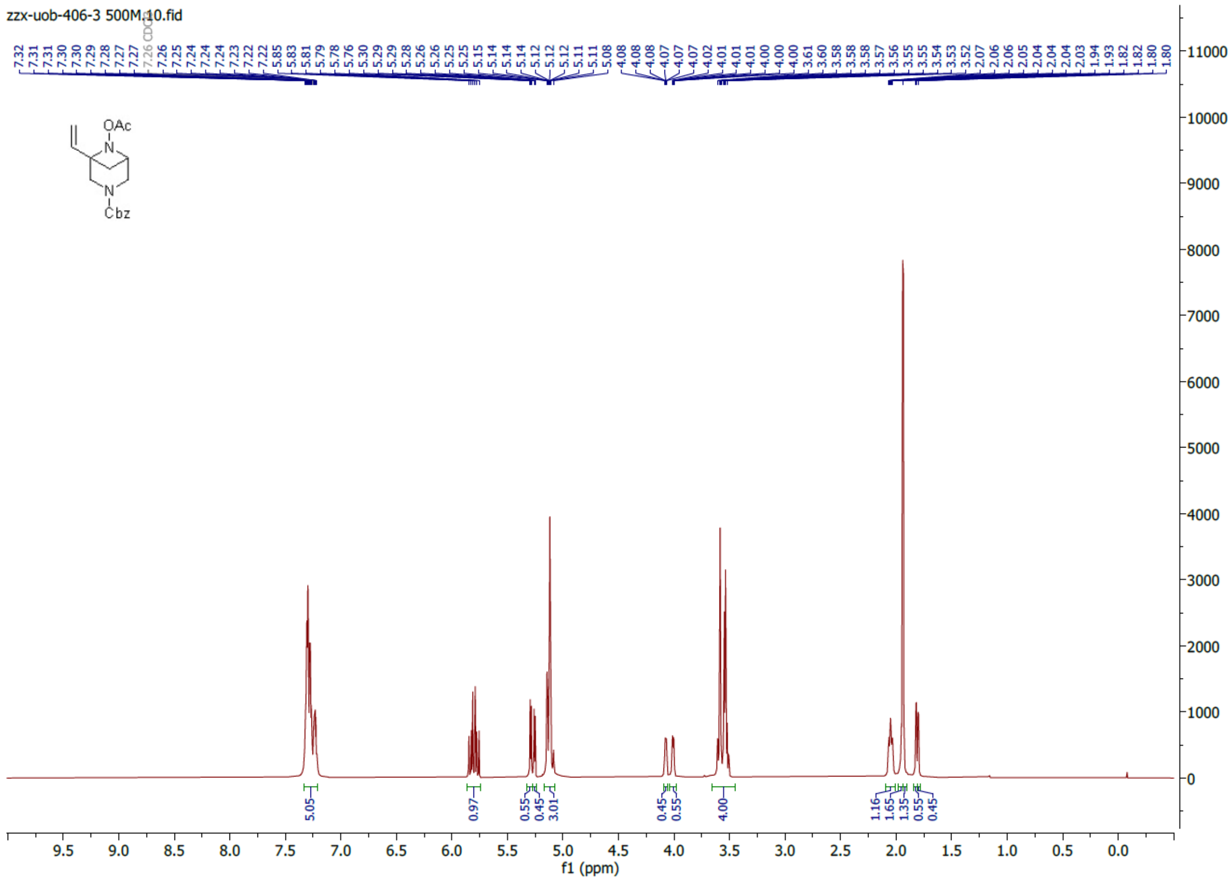

zzx-uob-406-3 500M.10.fid
OAc
N
N
Cbz
f1 (ppm)

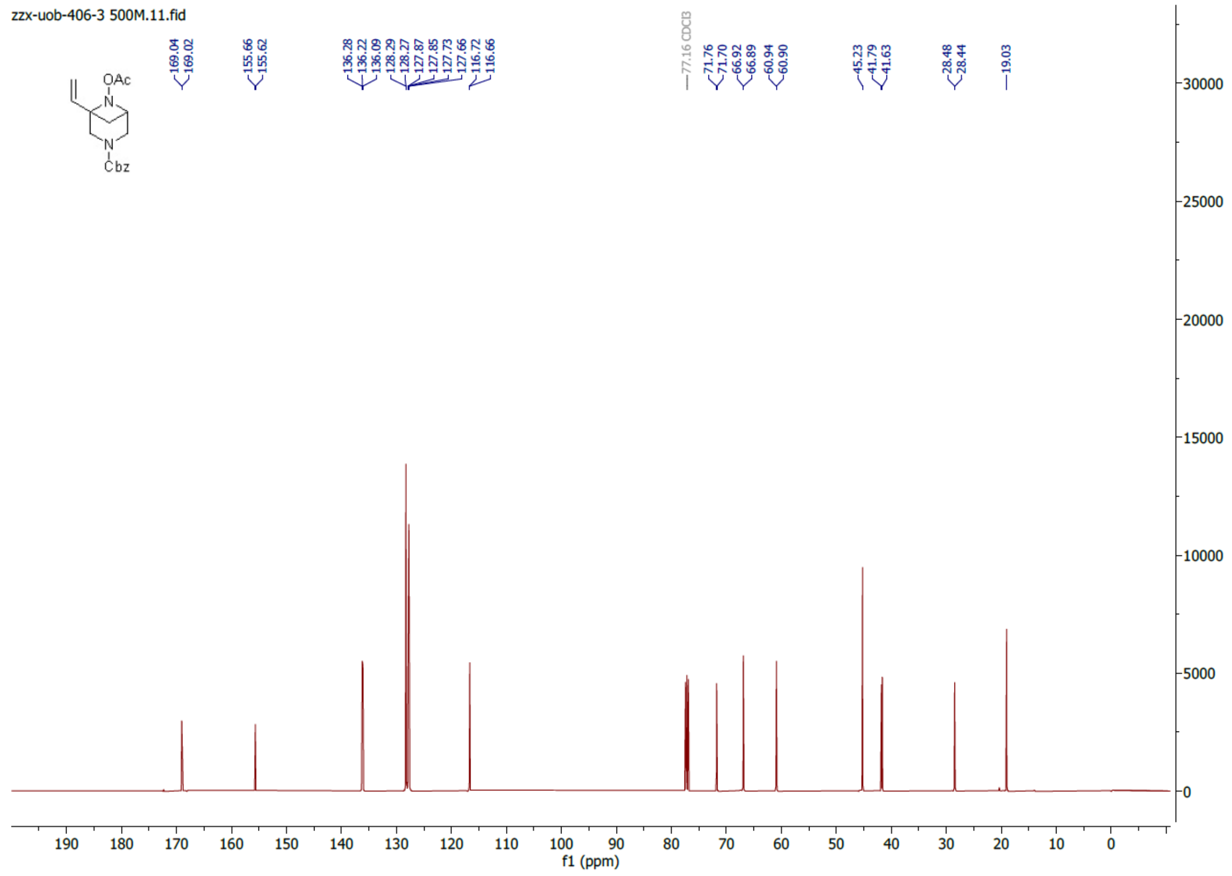

zzx-uob-406-3 500M.11.fid
OAc
N
N
Cbz
f1 (ppm)

Compound 68a

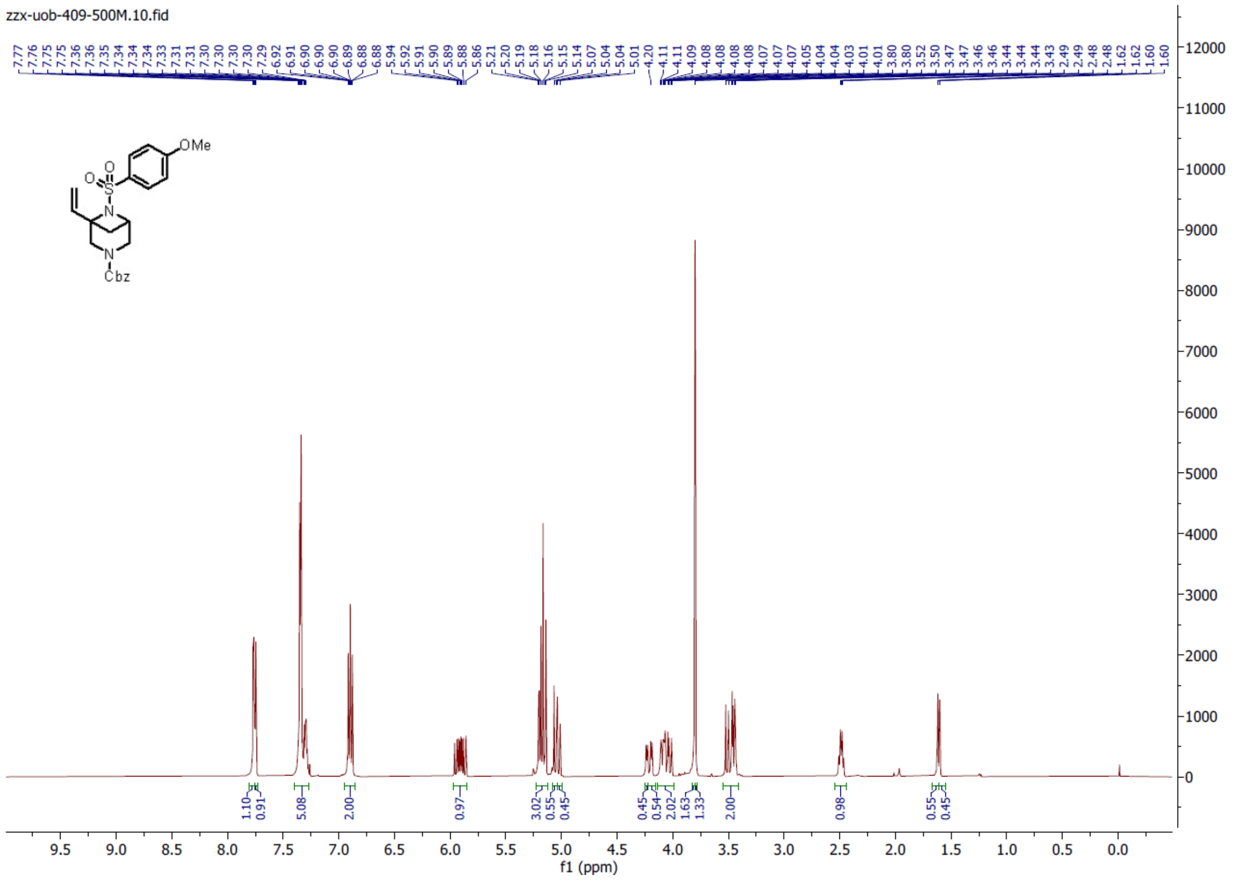

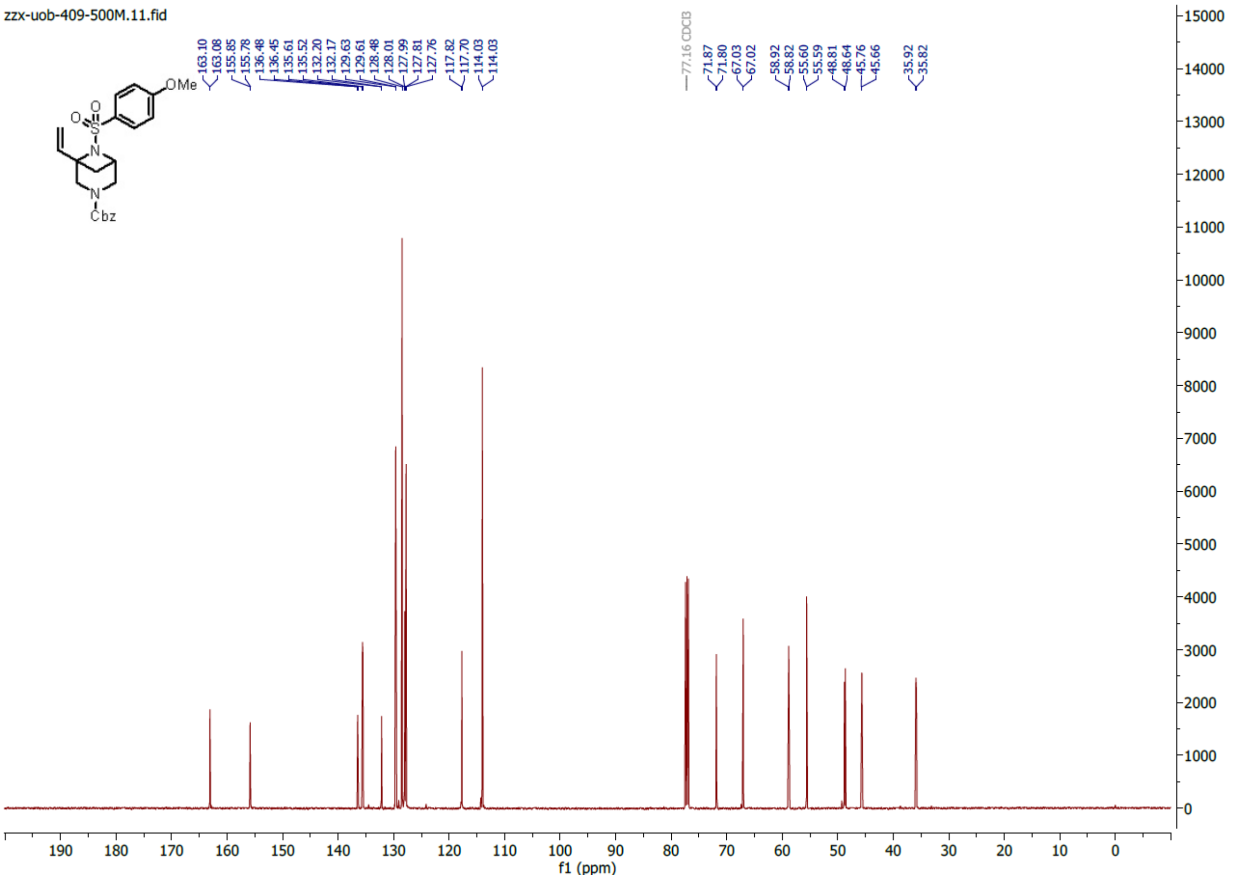

Compound 68

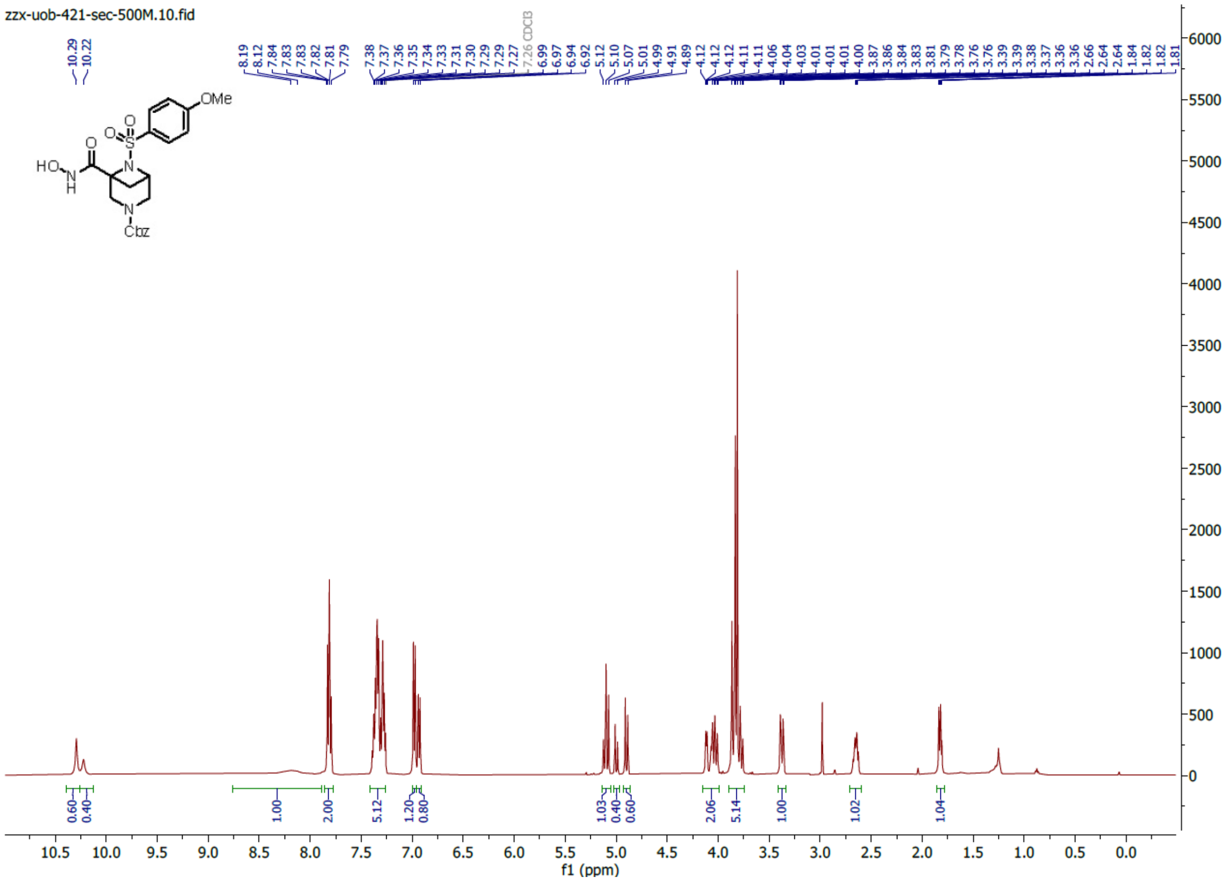

zzx-uob-421-sec-500M.10.fid
OMe
HO
Cbz
f1 (ppm)

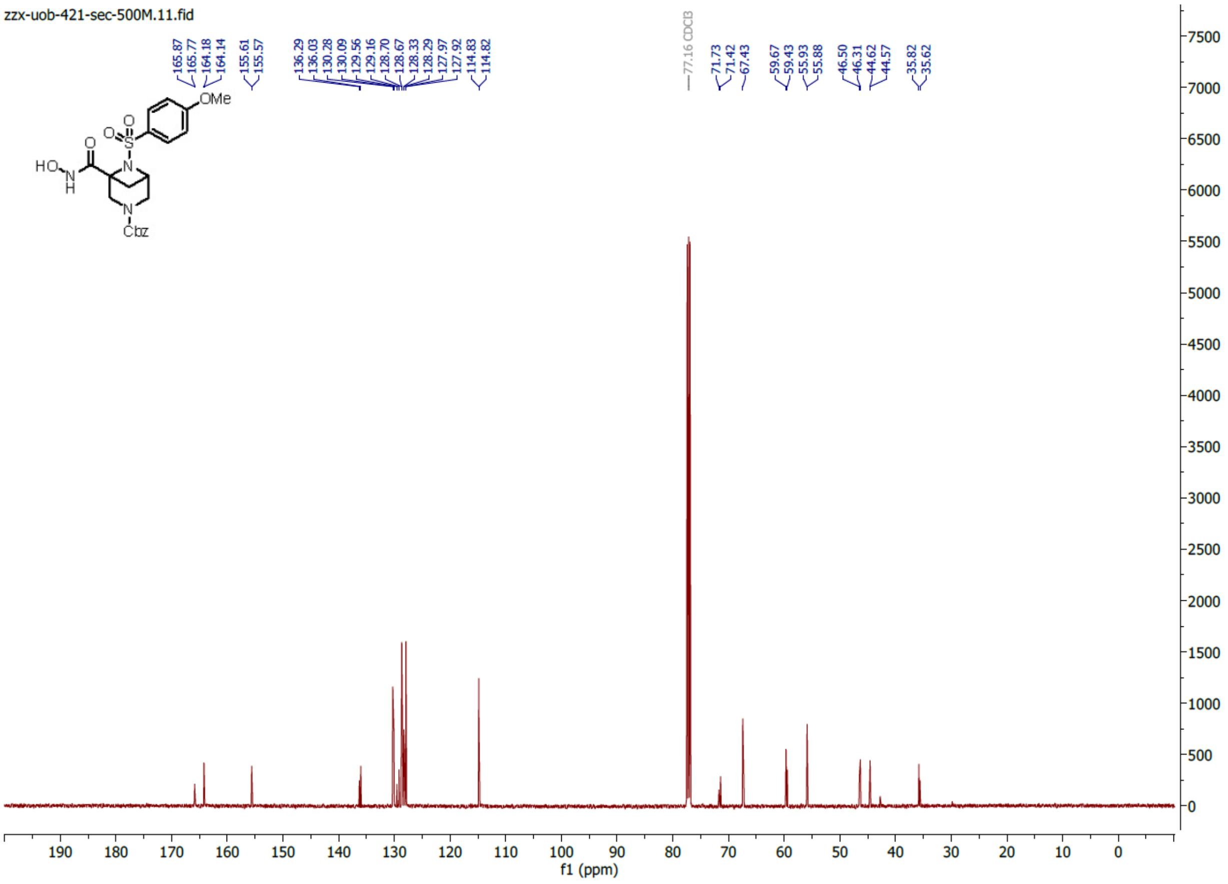

zzx-uob-421-sec-500M.11.fid
OMe
HO
Cbz
f1 (ppm)

3. Determination of LogP (pH 7.4)

LogP Assay

10 µL aliquots from a 10 mM DMSO stock solution of the test compound was dispensed into Eppendorf tubes in duplicate. Each Eppendorf was diluted with 490 µL of MeOH and vortexed to afford 200 µM samples. From each Eppendorf a 5 µL sample was injected into the HPLC system (HPLC conditions: isocratic mobile phase [methanol:10 mM PBS (pH 7.4) (75:25 v/v)], column temperature 30 °C, flow rate 1.0 mL/min, t_0 measured using unretained thiourea; LC-UV conditions: Shimadzu LC-20 instrument, X-Bridge C18, 5 µm, 4.6 mm × 250 mm column) and the retention time was recorded.

Data Analysis: The Log P value for each compound was calculated with the following equation:

$$\text{Log P} = 2.5494*\log (t_R - t_0)/t_0 + 2.7752.$$

The regression equation was obtained from a graph of Log k versus Log P for reference compounds.

The capacity factor (k) was determined by the expression: $k = (t_R - t_0)/t_0$

where t_R is the retention time of the test substance; t_0 is the dead time.

Reference substance	Log P
4-Acetylpyridine	0.5
Acetophenone	1.7
Chlorobenzene	2.8
Ethylbenzene	3.2
Phenanthrene	4.5
Triphenylamine	5.7

LogP values for the reference substances used to obtain the regression equation.

**Log P Results Summary**			
**Sample No.**	**Compound ID**	**Batch**	**Log P Result**
1	**DB04232**	**DB04232**-01	1.43
2	**68**	**68**-01	0.946

4. Assessment of Metabolic Stability in Human Liver Microsomes

Summary

Compound ID	Batch No.	Human					
		R^2	$T_{1/2}$ (min)	$CL_{int(mic)}$ (µL/min/mg)	$CL_{int(liver)}$ (mL/min/kg)	Remaining (T60)	Remaining (NCF60)
**DB04232**	**DB04232**	0.9840	36.0	38.5	34.6	30.6%	110.2%
**68**	**68**	0.9578	36.3	38.1	34.3	28.3%	86.9%
Testosterone	KWAKPA K	0.9986	16.7	82.9	74.6	8.0%	97.3%
Diclofenac	C2027110	0.9995	6.0	232.5	209.3	0.1%	96.1%
Propafenone	CDGO-101190	0.9211	6.4	216.4	194.8	0.1%	105.2%

Notes:

NCF: abbreviation of no co-factor. No NADPH was added to NCF samples (replaced by buffer) during the 1 h incubation. If the NCF remaining was less than 60%, then possibly a non-NADPH dependent metabolism had occurred.

R^2: correlation coefficient of the linear regression for the determination of kinetic constant (see raw data worksheet)

$T_{1/2}$: half-life

$CL_{int(mic)}$: microsome intrinsic clearance

$CL_{int(liver)}$: hepatic intrinsic clearance

$CL_{int(mic)} = 0.693/T_{1/2}$/mg microsome protein per mL

$CL_{int(liver)} = CL_{int(mic)} \times$ mg microsomal protein/g liver weight * g liver weight/kg body weight

Species	Liver Weight (g/kg Body Weight)[1-2]	Hepatic Blood Flow (Q_h) (mL/min/kg)[18,19]	Microsomal Protein (mg/g liver weight)
Mouse	88	90	45
Rat	40	55.2	45
Dog	32	30.9	45
Monkey	30	43.6	45
Human	20	20.7	45

Materials and Methods

Information of Test Compound

Compound ID	Batch No.	MW	FW	Purity%	Stock Conc. (mM)	Final Conc. (µM)
DB04232	DB04232	449.1257	449.1257	100	10	1
**68**	**68**	461.1257	461.1257	100	10	1

Information of Control Compound

Compound ID	Batch No.	MW	Stock Conc. (mM)	Final Conc. (µM)
Testosterone	KWAKPAK	288.42	10	1
Diclofenac	C2027110	295.14	10	1
Propafenone	CDGO-101190	341.44	10	1

The stock solutions of test and control compound were diluted to 100 µM with acetonitrile.

Information of Liver Microsomes

Species	Strain	Vendor	Cat. No.	Lot No.	Final Conc. (mg/mL)
Human	/	Corning	452117	38298	0.5

The microsomes were diluted to a concentration of 0.56 mg/mL using a 100 mM potassium phosphate buffer (PB Buffer).

Information of Cofactor

Name	Vendor	Cat. No.	Lot No.	Final Conc. (mM)
NADPH	BONTAC	BT04	BT04T123L001	1

An appropriate amount of NADPH powder was weighed and diluted to a concentration of 10 mM using a 10 mM $MgCl_2$ solution.

Stop Solution

Cold (4 °C) acetonitrile containing 250 nM tolbutamide and 250 nM labetalol as the internal standards (IS)

Metabolic Stability Assay

Empty incubation plates T60 and NCF60 were pre-warmed at 37 °C for 10 min. Working solutions of the liver microsomes (445 μL) were transferred into the pre-warmed incubation plates T60 and NCF60, followed by a further 10 min incubation at 37 °C. A 54 μL working solution of the liver microsomes was transferred to a Blank60 plate, followed by the addition of 6 μL NADPH cofactor and 180 μL of the stop solution into each well. Compound working solution (5 μL) was added to the incubation plates (T60 and NCF60) containing microsomes. For the NCF60 incubation plate, 50 μL of PB buffer was added and the plate was incubated at 37 °C for 1 h. Stop solution (180 μL) and NADPH working solution (6 μL) were added to a T0 plate. Then, a 54 μL aliquot was removed from the incubation plate T60 and transferred to the T0 plate. For the incubation plate T60, NADPH working solution (44 μL) was added, followed by a 1 h incubation at 37 °C. At 5, 15, 30, 45, and 60 min, 60 μL of each sample at each time point was transferred to a well containing 180 μL of stop solution, followed by mixing. All sampling plates were shaken for 10 min, then centrifuged at 3220 ×g for 20 min at 4 °C. The supernatant (80 μL) was transferred into 240 μL of pure water and mixed using a plate shaker for 10 min. Each bioanalysis plate was sealed and shaken for 10 min prior to LC-MS/MS analysis.

Data Analysis: The %Remaining of the compound after incubation was calculated using the following equation:

$$\%\text{Remaining}=\frac{\text{Peak area ratio of analyte to internal standard at each time point}}{\text{Peak area ratio of analyte to internal standard at zero time point}}\times 100$$

The equation of first-order kinetics was used to calculate $T_{1/2}$:

$$C_t = C_0 \times e^{-\mathrm{kt}}, \text{when } C_t = \frac{1}{2}C_0,\ T_{1/2} = \frac{ln2}{k_e} = \frac{0.693}{k_e}$$

Initial report

Compound ID & Species	Time (min)	Analyte Peak Area	IS Peak Area	Area Ratio	%Remaining	Time (min)	%Remaining	Ln (%Remaining)
DiclofenacHuman	Blank	0	260,521	0.000	0.0	NA	NA	NA
DiclofenacHuman	60	622	269,357	0.002	0.1	60	0.1	-2.4
DiclofenacHuman	45	3,537	267,090	0.013	0.5	45	0.5	-0.7
DiclofenacHuman	30	18,038	268,589	0.067	2.6	30	2.6	0.9
DiclofenacHuman	15	117,128	287,027	0.408	15.6	15	15.6	2.7
DiclofenacHuman	5	365,460	283,164	1.291	49.4	5	49.4	3.9
DiclofenacHuman	0	748,147	286,342	2.613	100.0	0	100.0	4.6
DiclofenacHuman	NCF60	733,522	292,212	2.510	96.1	NA	NA	NA
PropafenoneHuman	Blank	276	305,168	0.001	0.0	NA	NA	NA
PropafenoneHuman	60	3,095	310,920	0.010	0.1	60	0.1	-2.2
PropafenoneHuman	45	69,941	310,052	0.226	2.5	45	2.5	0.9
PropafenoneHuman	30	566,162	318,220	1.779	19.5	30	19.5	3.0
PropafenoneHuman	15	1,601,871	329,852	4.856	53.2	15	53.2	4.0
PropafenoneHuman	5	2,399,947	320,099	7.498	82.1	5	82.1	4.4
PropafenoneHuman	0	2,863,322	313,436	9.135	100.0	0	100.0	4.6
PropafenoneHuman	NCF60	3,080,196	320,552	9.609	105.2	NA	NA	NA

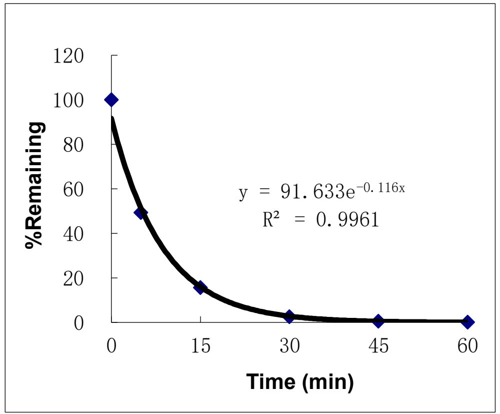

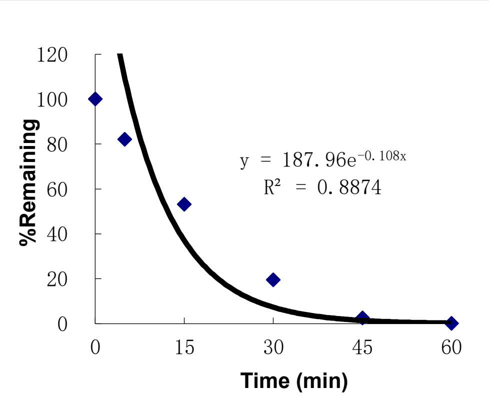

Compound ID & Species	Time (min)	Analyte Peak Area	IS Peak Area	Area Ratio	%Remaining	Time (min)	%Remaining	Ln (%Remaining)
TestosteroneHuman	Blank	1,491	688,383	0.002	0.3	NA	NA	NA
TestosteroneHuman	60	37,458	668,927	0.056	8.0	60	8.0	2.1
TestosteroneHuman	45	83,552	769,941	0.109	15.5	45	15.5	2.7
TestosteroneHuman	30	153,833	717,350	0.214	30.6	30	30.6	3.4
TestosteroneHuman	15	281,617	763,515	0.369	52.7	15	52.7	4.0
TestosteroneHuman	5	394,440	720,608	0.547	78.2	5	78.2	4.4
TestosteroneHuman	0	498,125	711,290	0.700	100.0	0	100.0	4.6
TestosteroneHuman	NCF60	520,045	763,468	0.681	97.3	NA	NA	NA
**DB04232Human**	Blank	778	257,046	0.003	0.1	NA	NA	NA
**DB04232Human**	60	271,183	266,169	1.019	30.6	60	30.6	3.4
**DB04232Human**	45	348,830	272,590	1.280	38.5	45	38.5	3.7
**DB04232Human**	30	487,848	247,556	1.971	59.3	30	59.3	4.1
**DB04232Human**	15	661,366	273,661	2.417	72.7	15	72.7	4.3
**DB04232Human**	5	723,902	265,507	2.726	82.0	5	82.0	4.4
**DB04232Human**	0	882,935	265,554	3.325	100.0	0	100.0	4.6
**DB04232Human**	NCF60	913,216	249,181	3.665	110.2	NA	NA	NA

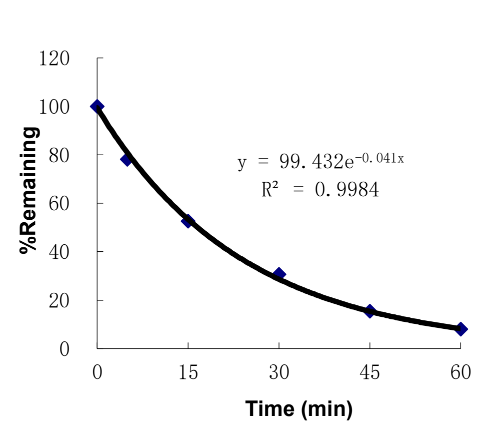

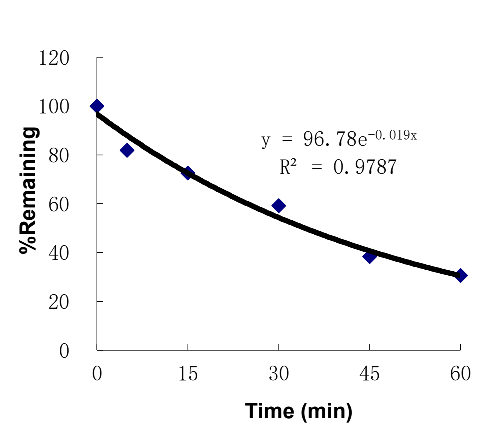

Compound ID & Species	Time (min)	Analyte Peak Area	IS Peak Area	Area Ratio	%Remaining	Time (min)	%Remaining	Ln (%Remaining)
**68 Human**	Blank	801	309,620	0.003	0.1	NA	NA	NA
**68 Human**	60	216,973	313,391	0.692	28.3	60	28.3	3.3
**68 Human**	45	294,931	329,170	0.896	36.6	45	36.6	3.6
**68 Human**	30	355,265	315,963	1.124	45.9	30	45.9	3.8
**68 Human**	15	462,274	322,179	1.435	58.6	15	58.6	4.1
**68 Human**	5	563,159	322,663	1.745	71.3	5	71.3	4.3
**68 Human**	0	708,621	289,379	2.449	100.0	0	100.0	4.6
**68 Human**	NCF60	687,866	323,402	2.127	86.9	NA	NA	NA

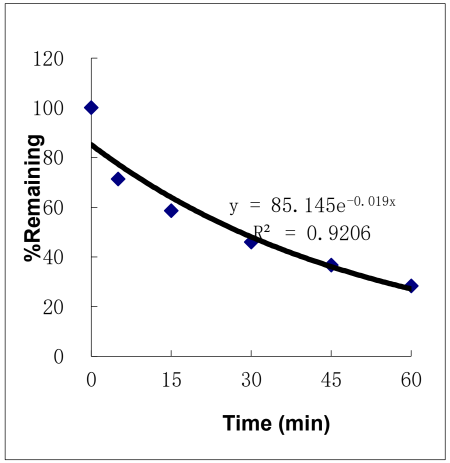

5. Computational Analysis

5.1. General Information

Two sets of calculations were performed to investigate two sets of questions. The respective method of choice is based on similar previous literature reports. The first one is for the structural analyses related to the investigation of bio-isosteres of nitrogen-containing heterocycles. The second set involves transition state analyses related to the exploration of the rule-of-5 radical cyclisation.

3D Structure and exit vector analyses: Calculations were conducted using the computational chemistry package ORCA (version 6.0.0).[20,21] Calculations involving geometry optimisations were carried out in the gas phase using the Quasi-Newton method in redundant internal coordinates,[22] with an initial model hessian from Almlöf,[23] and 'TightSCF' implementing the default parameters (a tolerance of 10^{-8} Hartrees for the SCF energy change and 5×10^{-6} Hartrees for the optimization steps). Density functional theory (DFT)[24] for structural analysis used the ω97X function,[25] with the dispersion correction protocol from Grimme's with Becke-Johnson damping (D3BJ).[26,27] This hybrid functional applied the resolution of identity chain of spheres exchange (RIJCOSX) approximation,[28,29] which applies the resolution of identity approximation for the Coulomb integrals (RI-J),[30] with the def2/J auxiliary basis set,[31] and the chain of spheres integration for the Hartree-Fock Exchange (COSX).[32] The DFT and COSX integration grids are determined by the 'DEFGRID2' keyword, which is the default. The Popel basis set,[33,34] 6-31++G(d,p) was used for calculations, this methodology was selected based on previous structural benchmarking of N-containing heterocycles for bioisostere identification.[35]

Initial geometry guesses and final conformation entropies were obtained from the CREST (version 3.0.2)[36-38] and xtb (version 6.6.1)[39] program, with an energy threshold of 6 kcal mol^{-1}. This initial conformational sampling utilized the semi-empirical tight binding method GFN2-xTB,[40] in the gas phase, where the lowest energy conformer in the ensemble was kept for further DFT calculations. Final structures were verified as minima by having no imaginary frequencies from analytical calculations of the force constant hessian at the ω97X-D3BJ/6-31++G(d,p) level of theory.

Transition state analyses (rule-of-5): For the DFT analyses of the energy barriers of radical cyclizations, calculations were conducted using the *Gaussian 09* software package (Revision E.01).[41] Calculations employed the unrestricted (U)ωB97X-D[42] functional with the def2-TZVP[43] basis set and the SMD[44] implicit solvation model for acetonitrile. Geometry optimizations were conducted employing redundant internal coordinates in default settings with thresholds of 4.5×10^{-4} Hartree/Bohr for maximum force, 3.0×10^{-4} Hartree/Bohr for RMS force, 1.8×10^{-3} Bohr for maximum displacement, and 1.2×10^{-3} Bohr for RMS displacement. Self-consistent field (SCF) convergence was performed using the exact quadratic convergence algorithm (SCF = XQC). Vibrational frequency analysis was used to classify stationary points as minima or transition states, with the latter characterised by a single large imaginary frequency and the former. Intrinsic reaction coordinate (IRC) calculations were carried out to verify that transition states correctly connect the expected reactants and products. Thermal corrections and entropy contributions were refined using Grimme's quasi-harmonic (QHA) model with a 100.0 cm^{-1} frequency cut-off, implemented via the *GoodVibes* program at the solution-phase standard state (T = 298.15 K, c = 1 $mol\cdot L^{-1}$). Computations using this level of theory were further assessed by comparing the Curtain-Hammet derived product

distribution from the difference in barriers (DDG‡) with the experimentally obtained results (*vide infra*). Moreover, the use of range separated hybrid functional and triple-z basis set are generally well behaved in open shell calculations for related systems.[45,46] Molecular visualizations were produced through Chimera.[47] The coordinates and thermochemical data are provided as an additional supplement.

5.2. Parameter Calculation

Several cheminformatic descriptors were extract from the RDKit software (version 2024.03.5),[48] utilising Python (version 3.12.5) in a Jupyter notebooks (version 6.6.3).[49] with data handling using NumPy (version 2.0.1)[50] and pandas (version 2.2.2) packages.[51] The descriptors extracted in this way included the molecular weight, computed molecular volume, plane of best fit (PBF, to characterise three-dimensional space),[52] BertzCT (a topological index for complexity),[53] 3D score (a metric for the three-dimensionality based on the summation of the normalised principal moments of inertia (PMI)),[54] MolLogP (atom-based calculation of LogP calculation based on Crippen's approach), and topological polar surface area (TPSA).[55] The conformational entropy was also obtained through a minima mining approach using the conformer-rotamer ensembles as obtained from metadynamics simulations with GFN2-xTB implemented in CREST,[56] due to the statistical nature, this calculation was repeated 10 times and the average and standard deviation are provided in cal mol^{-1} K^{-1}.

Several descriptors pertaining to geometric features were also extracted based on the coordinates produced from the DFT calculations (**Supplementary Figure S4**, exemplified on pyridine).

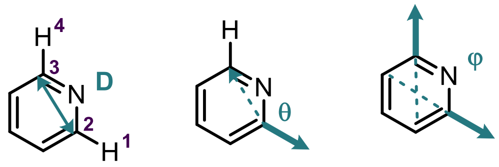

Supplementary Figure S4. Example of geometric descriptors used with pyridine as an example.

Here, D refers to the distance in angstroms between C2 and C3. The angle θ in degrees is the averaged angles between the vector C2-H1 with C2-C3 and H4-C3 with C3-C2, and φ is the exit angle based on the vectors C2-H1 and C3-H4. The angles (θ and φ) are calculated according to the 2-argument arctangent function of the normed scalar product and normed vector product:

$$cos\,cos\,(\theta) = \frac{\vec{u} \cdot \vec{v}}{|\vec{u}||\vec{v}|}$$

$$sin\,sin\,(\theta) = \frac{|\vec{u} \times \vec{v}|}{|\vec{u}||\vec{v}|}$$

$$\theta = atan2[sin(\theta), cos(\theta)]$$

Another quantity examining structural features of the molecules is the root-mean-square deviation (RMSD in angstrom) between pairs of structures. To calculate this value the hydrogens are removed and the systems truncated down to a simple core, the cores are overlapped and the distance between the same numbered atoms calculated. This procedure was implemented in Chimera.[47]

Finally using the truncated systems, the distortion energy was calculated in kcal mol^{-1}. This refers to the energy required to convert the core of one structure to another, for example the boat piperidine to a higher energy structure with the same geometry as the bicyclic structures core.

5.3. Parameter Analysis

The following descriptors were obtained for the structures of interest (**Supplementary Table S2 & S3**).

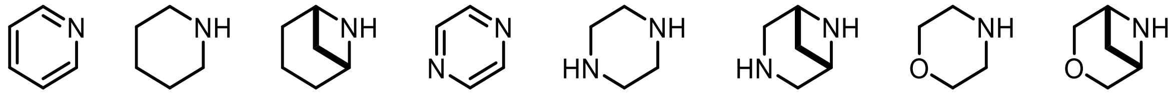

A B C D E F G H

Structure	MolWt	Vol	PBF	BertzCT	PMI1	PMI2	PMI3
**A**	79.10	79.47	0.00	256.33	83.66	87.01	170.67
**B**	85.15	96.86	0.58	282.93	112.49	115.07	199.50
**C**	97.16	104.84	0.74	362.26	130.82	154.64	198.46
**D**	80.09	75.19	0.00	196.21	78.64	85.33	163.97
**E**	86.14	92.59	0.59	220.41	107.69	112.56	193.66
**F**	98.15	100.81	0.72	309.48	124.48	153.29	192.09
**G**	87.12	89.22	0.59	227.21	103.50	110.21	188.93
**H**	99.13	97.26	0.76	303.95	122.30	150.21	188.38

Supplementary Table S2. chemical descriptors obtained for the following N-containing heterocycles.

Structure	3D Score	MolLogP	TPSA	S_{conf}	D (Å)	θ (°)	Φ (°)
**A**	1.00	1.08	12.89	0.00 ± 0.00	2.28	147.3	114.7
**B**	1.14	0.76	12.03	1.94 ± 0.00	2.43	142.3	104.6
**C**	1.44	0.90	12.03	1.19 ± 0.00	2.07	147.8	115.7
**D**	1.00	0.48	25.78	0.00 ± 0.00	2.26	149.2	118.4
**E**	1.14	-0.82	24.06	2.25 ± 0.16	2.42	142.9	105.8
**F**	1.45	-0.68	24.06	2.54 ± 0.16	2.04	149.6	119.1
**G**	1.13	-0.39	21.26	2.40 ± 0.05	2.41	143.6	107.1
**H**	1.45	-0.25	21.26	1.38 ± 0.00	2.05	150.8	121.7

Supplementary Table S3. chemical descriptors obtained for the following N-containing heterocycles including those derived from structural features.

The data indicates that the bridged structures have several desirable properties including an increased in the three dimensionality and the complexity, while retaining useful predicted solubility. As expected, the S_{conf} for the aromatic systems is 0 and drastically increases with piperidine, piperazine and morpholine. The bridged equivalent for piperidine and morpholine show a decrease in S_{conf}

indicating a more rigidified structure. In contrast the piperazine structures show larger error, but indicate the opposite trend, potentially a consequence from the number of low energy conformers which are able to be adopted due to the nitrogen atom. The structural parameters show that the vectors analysed indicate the bridged structures are more similar to the aromatic systems, potentially indicating their ability to act as bioisosteres. To investigate this further the cores were overlapped (**Supplementary Figure S5**).

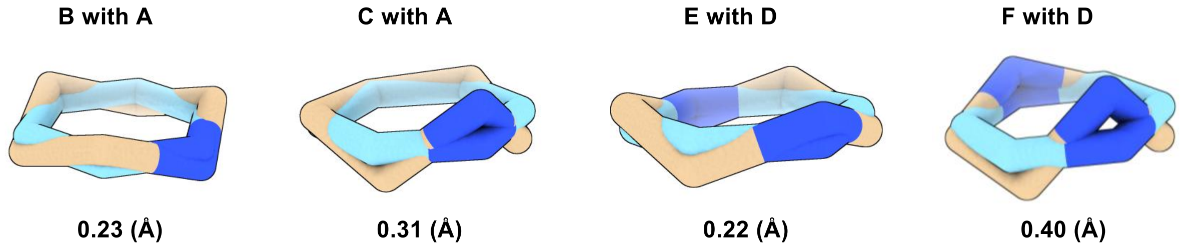

Supplementary Figure S5. examination of the root-mean-square deviation of overlapping core structures with the parent aromatic system (blue).

Although the geometric parameters studied show that the bridge structures have more in common with the aromatic system than the saturated heterocycles, the reverse is seen when considering the RMSD. From the depictions this is a consequence of the nitrogen heteroatom sitting out of the plane, overall implying that this unit provides alternative directionality.

Finally, the energy required to distort the piperidine, piperazine and morpholine to the conformations of the bridged structure was examined (**Supplementary Figure S6**).

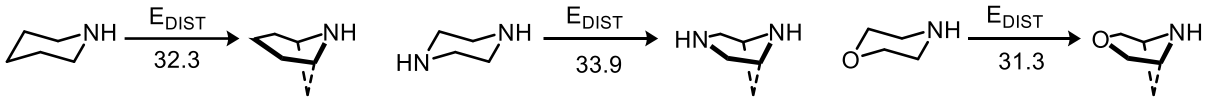

Supplementary Figure S6. Energy required to distort the system in kcal mol^{-1}, where the dotted lines indicate the structural component removed.

The calculations indicate that the bridged systems create a highly distorted boat core, which would be energetically unfavourable to obtain by the parent heterocycle.

5.4. Transition State Analyses for Radical Cyclization: Breaking the Rule of 5

Considering a Dexter Entergy transfer scenario,[57,58] the exchange of multiplicities between the substrate and the photocatalyst should occur within the catalyst's triplet excited energy (i.e., $E_T = 63.5$ kcal mol^{-1} for *fac*-[IrdF(ppy)$_3$]). To confirm which fragment (olefine vs oxime) goes to the triplet state (**SM*-1** vs **SM*-2**) upon sensitization of the model substrate **SM**, the triplet energies were estimated using the free webtool EnT Decker developed by Glorius and co-workers (**Supplementary Figure S7**).[59] The relatively low triplet energy of styrene fragment at 59 kcal mol^{-1} is accessible with the typical photosensitizes employed in this study. On the other hand, the triplet excited state of oxime 70 kcal mol^{-1} is beyond the triplet energies of the that can be supplied by the photocatalyst. This means that the intramolecular radical cyclization proceeds with the as styrene moiety as diradical attacking the p* of the oxime fragment. This is consistent with the previous observations reported in the literature that olefins are preferentially excited over the oxime moiety.[60] Moreover, forcing the

excitation of oxime for related substrates under high energy UV irradiation often lead to unproductive decay of the excited state (i.e. E/Z isomerization) rather than the desired radical cyclization.[60]

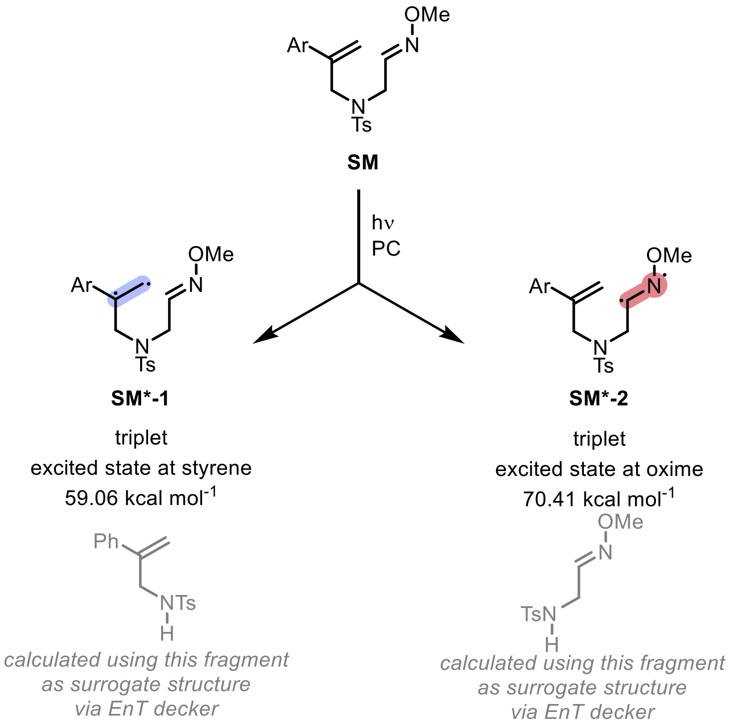

Supplementary Figure S7. Estimation of triplet exited state energies for the olefin and oxime fragment.

As the nature of the triplet excited state being the diradical at the styrene fragment is now established, the mode of cyclization is then examined (**Supplementary Figure S8**). All four possible cyclizations were considered depending on which radical (C1 vs C2) attacks which end of the oxime's p* namely: 6-exo-trig, 5-exo-trig, 6-endo-trig, and 7-endo-trig. Considering also various conformations of the transition states, the barriers for these cyclizations were calculated ($\Delta G^{\ddagger}$, kcal mol^{-1}). According to Baldwin's rules[61] and modified rules introduced by Beckwith[62], radical 5/6-exo-trig cyclizations are preferentially favoured over endo-trig. Indeed, both 6-exo-trig and 5-exo-trig cyclizations of our model substrate are kinetically accessible ($\Delta G^{\ddagger}$ = 11.9 kcal mol^{-1} and below), while the 6-endo-trig and 7-endo-trig cyclization have relatively high barriers ($\Delta G^{\ddagger}$ = 17.8 to 33.4 kcal mol^{-1}). It is generally observed that 5-exo-trig cyclization is more feasible over the 6-exo-trig cyclization (hence the rule-of-5)[63-65] due to stereo-electronic effects – that the 5-membered transition state has better orbital overlap compared to other geometries. Interestingly, among the series of transition states for our model substrates, **TS$_{6\text{-exo-trig}}$I** has the lowest barrier which indicates that this is the most kinetically feasible pathway (barrier difference, $\Delta\Delta G^{\ddagger}$, kcal mol^{-1} in **Supplementary Figure S8** are calculated based on this). Moreover, the difference between the barrier heights, $\Delta\Delta G^{\ddagger}$, of **TS$_{6\text{-exo-trig}}$I** and (the lowest barrier in the 5-exo-trig series) is 1.3 kcal mol^{-1}. Despite the fact that the angle of attack of the C2 radical to oxime in **TS$_{5\text{-exo-trig}}$II** (108°) is slightly closer to the Bürgi-Dunitz angle versus the angle of attack of the C1 radical to oxime **TS$_{6\text{-exo-trig}}$I** (102°). This signifies that the radical stabilization brought by the phenyl group adjacent to C2 is the dominant factor for the switch in regioselectivity. Indeed, when the phenyl group is swapped with methyl (**Supplementary Figure S9**), the regioselectivity follows the rule-of-5 radical cyclization as the barrier for **TS$_{6\text{-exo-trig}}$Me** ($\Delta G^{\ddagger}$ = 5.6 kcal mol^{-1}) is 3.4 kcal mol^{-1} higher than the barrier of for **TS$_{5\text{-exo-trig}}$Me** ($\Delta G^{\ddagger}$ = 2.1 kcal mol^{-1}).

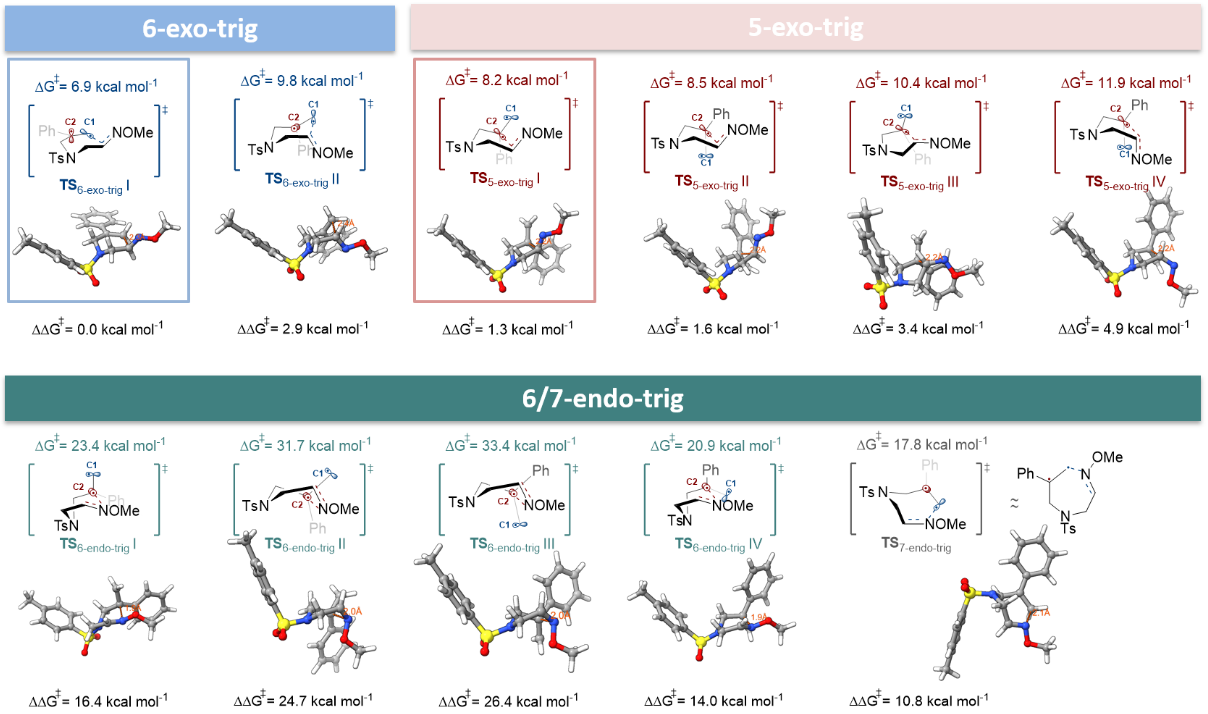

Supplementary Figure S8. Summary of Transition states for the initial radical cyclization.

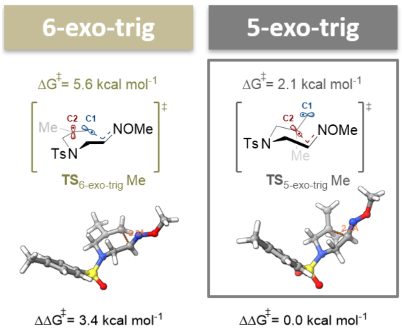

Supplementary Figure S9. Transition States for the Initial Radical Cyclization with the Ph Group Replaced by a Me Group

Considering a Curtin Hammett scenario applies (**Supplementary Equation S1**), the calculated $\Delta\Delta G^{\ddagger}$ of 1.3 kcal mol^{-1} translates to a product distribution of 90:10, favouring the anti-rule-of-5 product. Experimentally, 69% of anti-rule-of-5 product and 13% of rule-of-5 product were observed, culminating in a normalized product distribution of 84:16 favouring the anti-rule-of-5 product, which is in close agreement with the value obtained from DFT calculations.

$$\frac{P1}{P2} = e^{\frac{-\Delta\Delta G^{\ddagger}}{RT}} \text{ ; where R} = 1.987\times10^{-3} \text{ kcal/(mol·K) and T} = 298 \text{ K}$$

Supplementary Equation S1. Curtin-Hammett relation with product distribution.

6. Crystallographic Data (X-Ray)

Structure Report for compounds 36 and 62

Crystallographic data are presented in the Tables below. Single crystals of **36** and **62** were coated in perfluoropolyether oil and mounted on glass fibres. X-ray measurements were made using a Bruker D8 Venture dual source kappa CPAD diffractometer with Mo K_a radiation (l = 0.71073 Å).[66] Intensities were integrated[66] from several series of exposures, each exposure covering 0.5° in w or f. Absorption corrections were applied, based on multiple and symmetry-equivalent measurements.[67] The structures were solved by direct methods and refined by least squares on weighted F^2 values for all reflections (see **Supplementary Tables S4** and **S6**).[68]

All non-hydrogen atoms were assigned anisotropic displacement parameters and refined without positional constraints. For compound **62**, the Hydrogen atom H(1) was located in the electron density difference map and refined without distance restraints. For both structures, the positions of the methyl hydrogen atoms were assigned by a rotating group refinement with fixed, idealised C-H distances. All other hydrogen atoms were constrained to ideal geometries. All hydrogen atoms were assigned isotropic displacement parameters equal to 1.5 times (methyl hydrogen atoms) or 1.2 times (all other hydrogen atoms) that of their parent atom.

Refinement proceeded smoothly to give the residuals shown in **Supplementary Table S4**. Complex neutral-atom scattering factors were used.[69]

Supplementary Table S4. Crystal data and structure refinement for **36**

Identification code	36	
Empirical formula	$C_{19}H_{18}N_2O_3$	
Formula weight	322.35 g mol^{-1}	
Temperature	100(2) K	
Wavelength	0.71073 Å	
Crystal system	Monoclinic	
Space group	$P2_1/c$	
Unit cell dimensions	a = 8.7410(3) Å	a = 90°
	b = 21.8142(7) Å	b = 113.9100(10)°
	c = 9.1918(3) Å	g = 90°
Volume	1602.26(9) Å^3	
Z	4	
Density (calculated)	1.336 Mg/m^3	
Absorption coefficient	0.091 mm^{-1}	
F(000)	680	
Crystal size	0.5 x 0.5 x 0.5 mm	
q range for data collection	1.867 to 28.389°	
Index ranges	-11<=h<=11, -28<=k<=29, -12<=l<=12	
Reflections collected	32367	
Independent reflections	4005 [R_{int} = 0.0345]	

Completeness to q = 25.242°	99.9 %
Absorption correction	Semi-empirical from equivalents
Max. and min. transmission	0.9705 and 0.8864
Refinement method	Full-matrix least-squares on F^2
Data / restraints / parameters	4005 / 0 / 218
Goodness-of-fit on F^2	S = 1.027
R indices [for 3694reflections with I>2s(I)]	R_1 = 0.0373, wR_2 = 0.0929
R indices (for all 4005 data)	R_1 = 0.0401, wR_2 = 0.0950
Weighting scheme	$w^{-1} = s^2(F_o{}^2) + (aP)^2 + (bP)$, where $P = [\max(F_o{}^2, 0) + 2F_c{}^2]/3$ a = 0.0432, b = 0.6684
Largest diff. peak and hole	0.366 and -0.220 eÅ^{-3}

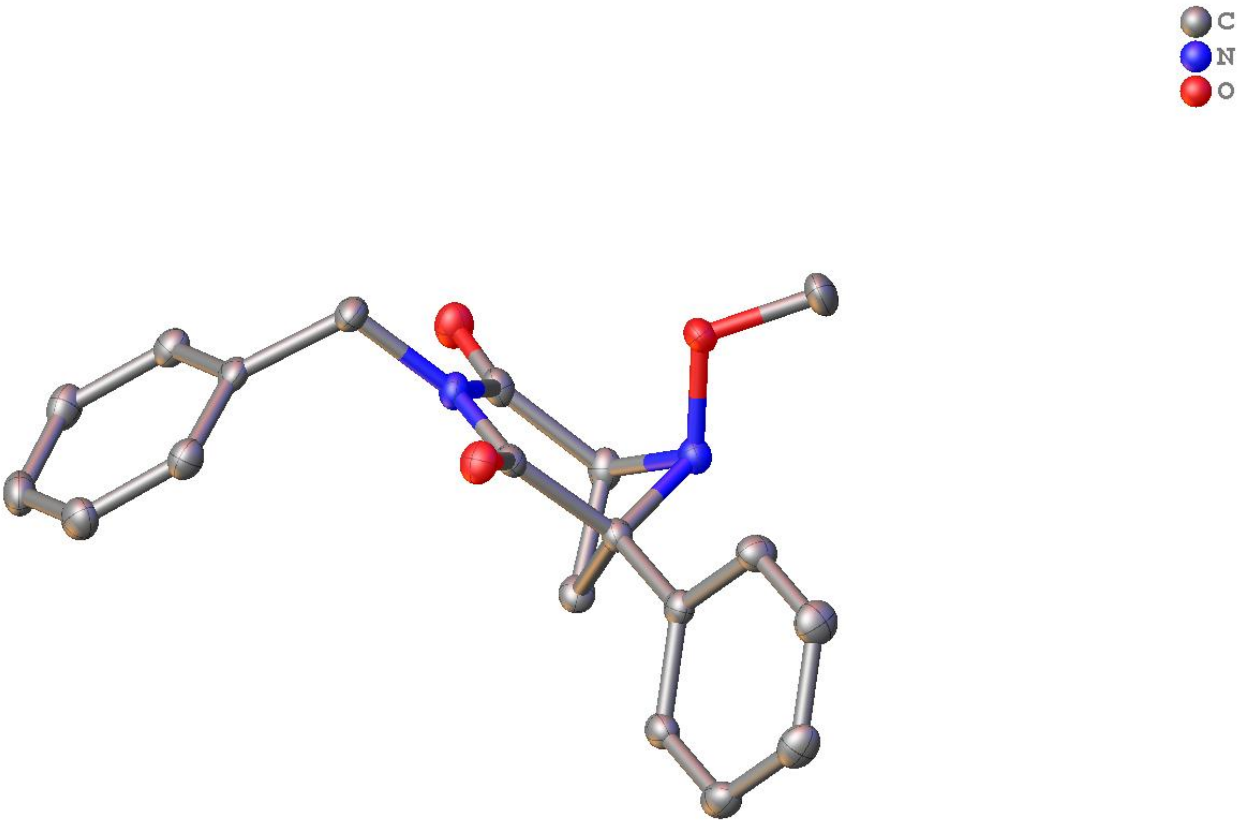

Supplementary Figure S10. ORTEP representation of **36**, CCDC 2451817

Supplementary Table S5. Bond lengths [Å] and angles [°] for **36**

C(1)-O(1)	1.2104(12)
C(1)-N(1)	1.3926(13)
C(1)-C(2)	1.5390(13)
C(2)-C(14)	1.4962(14)
C(2)-N(2)	1.5029(12)
C(2)-C(3)	1.5534(13)
C(3)-C(4)	1.5462(15)
C(4)-N(2)	1.4965(13)
C(4)-C(5)	1.5124(14)
C(5)-O(3)	1.2098(13)
C(5)-N(1)	1.3950(13)
C(6)-N(1)	1.4772(12)
C(6)-C(7)	1.5110(14)
C(7)-C(8)	1.3902(14)
C(7)-C(12)	1.3952(14)
C(8)-C(9)	1.3921(15)
C(9)-C(10)	1.3881(16)
C(10)-C(11)	1.3882(16)
C(11)-C(12)	1.3883(15)
C(13)-O(2)	1.4259(12)
C(14)-C(15)	1.3897(14)
C(14)-C(19)	1.3963(14)
C(15)-C(16)	1.3910(15)
C(16)-C(17)	1.3848(16)
C(17)-C(18)	1.3903(16)
C(18)-C(19)	1.3891(15)
N(2)-O(2)	1.4398(11)
O(1)-C(1)-N(1)	122.26(9)
O(1)-C(1)-C(2)	123.70(9)
N(1)-C(1)-C(2)	114.03(8)
C(14)-C(2)-N(2)	116.32(8)
C(14)-C(2)-C(1)	113.65(8)
N(2)-C(2)-C(1)	108.90(8)
C(14)-C(2)-C(3)	120.60(8)
N(2)-C(2)-C(3)	85.89(7)
C(1)-C(2)-C(3)	108.13(8)
C(4)-C(3)-C(2)	84.48(7)

N(2)-C(4)-C(5)	111.00(8)
N(2)-C(4)-C(3)	86.37(7)
C(5)-C(4)-C(3)	109.08(8)
O(3)-C(5)-N(1)	122.39(9)
O(3)-C(5)-C(4)	124.22(9)
N(1)-C(5)-C(4)	113.39(9)
N(1)-C(6)-C(7)	111.73(8)
C(8)-C(7)-C(12)	119.07(9)
C(8)-C(7)-C(6)	120.88(9)
C(12)-C(7)-C(6)	120.04(9)
C(7)-C(8)-C(9)	120.34(10)
C(10)-C(9)-C(8)	120.19(10)
C(9)-C(10)-C(11)	119.78(10)
C(10)-C(11)-C(12)	119.98(10)
C(11)-C(12)-C(7)	120.60(10)
C(15)-C(14)-C(19)	119.18(9)
C(15)-C(14)-C(2)	119.82(9)
C(19)-C(14)-C(2)	120.99(9)
C(14)-C(15)-C(16)	120.50(10)
C(17)-C(16)-C(15)	120.18(10)
C(16)-C(17)-C(18)	119.64(10)
C(19)-C(18)-C(17)	120.33(10)
C(18)-C(19)-C(14)	120.16(10)
C(1)-N(1)-C(5)	120.03(8)
C(1)-N(1)-C(6)	120.64(8)
C(5)-N(1)-C(6)	119.22(8)
O(2)-N(2)-C(4)	112.76(8)
O(2)-N(2)-C(2)	111.66(7)
C(4)-N(2)-C(2)	88.01(7)
C(13)-O(2)-N(2)	108.80(8)

Supplementary Table S6. Crystal data and structure refinement for **62**

Identification code	62	
Empirical formula	$C_{25}H_{25}N_3O_3S$	
Formula weight	447.54 g mol^{-1}	
Temperature	100(2) K	
Wavelength	0.71073 Å	
Crystal system	Monoclinic	
Space group	$P2_1/n$	
Unit cell dimensions	*a* = 8.5564(3) Å	*a* = 90°
	b = 23.2079(7) Å	*b* = 100.8000(10)°
	c = 11.3644(4) Å	*g* = 90°
Volume	2216.73(13) Å^3	
Z	4	
Density (calculated)	1.341 Mg/m^3	
Absorption coefficient	0.179 mm^{-1}	
F(000)	944	
Crystal size	0.533 x 0.429 x 0.32 mm	
q range for data collection	2.024 to 27.931°	
Index ranges	-11<=*h*<=11, -30<=*k*<=30, -14<=*l*<=14	
Reflections collected	82876	
Independent reflections	5296 [R_{int} = 0.0628]	
Completeness to *q* = 25.242°	100.0 %	
Absorption correction	Semi-empirical from equivalents	
Max. and min. transmission	0.7456 and 0.5912	
Refinement method	Full-matrix least-squares on F^2	
Data / restraints / parameters	5296 / 0 / 294	
Goodness-of-fit on F^2	*S* = 1.042	
R indices [for 4283reflections with I>2s(I)]	R_1 = 0.0391, wR_2 = 0.0871	
R indices (for all 5296 data)	R_1 = 0.0543, wR_2 = 0.0930	
Weighting scheme	$w^{-1} = s^2(F_o{}^2) + (aP)^2 + (bP)$,	
	where $P = [\max(F_o{}^2, 0) + 2F_c{}^2]/3$	
	a = 0.0385, *b* = 1.2941	
Largest diff. peak and hole	0.379 and -0.471 eÅ^{-3}	

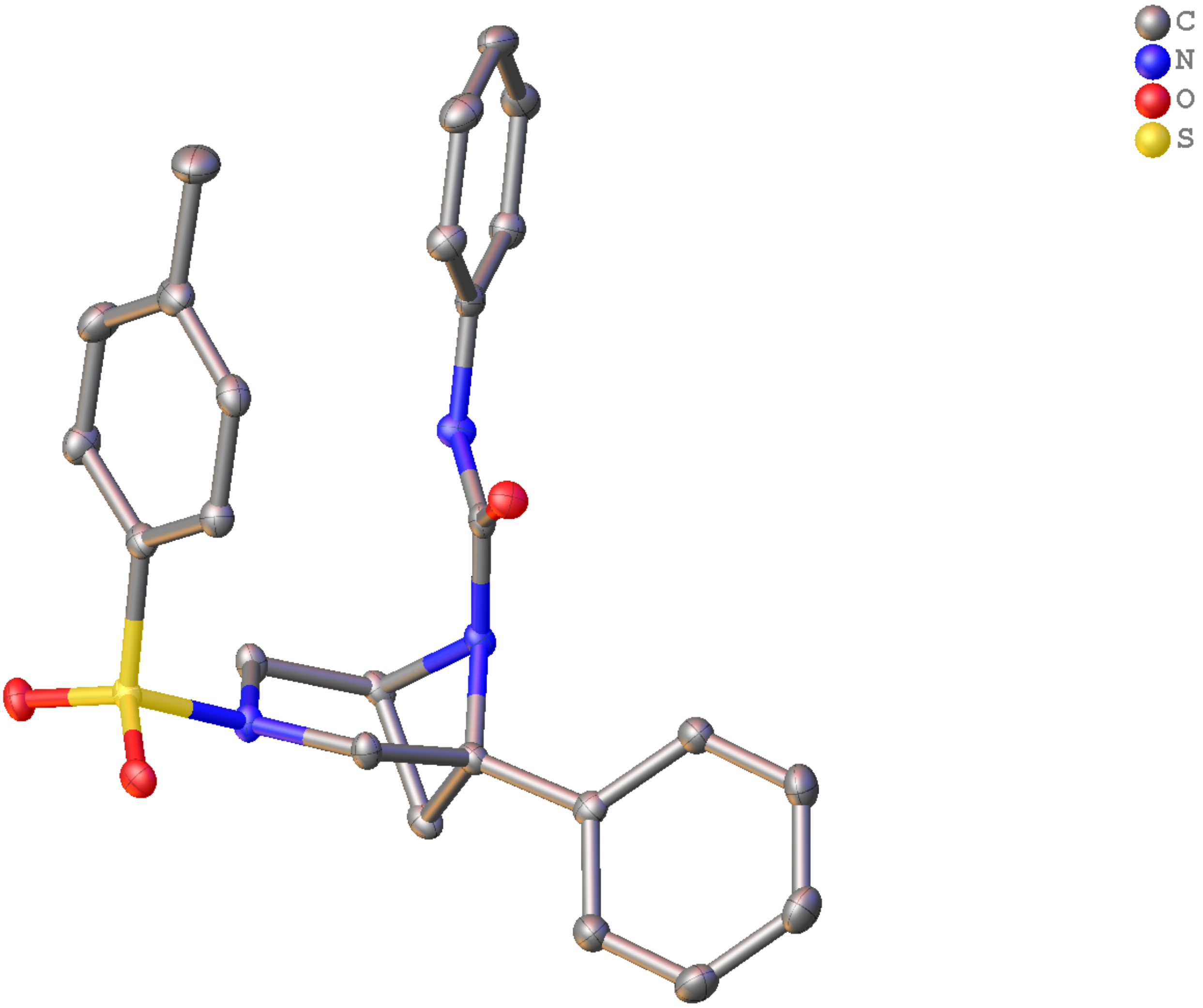

Supplementary Figure S11. ORTEP representation of **62**, CCDC 2451818

Supplementary Table S7. Bond lengths [Å] and angles [°] for **62**

S(1)-O(2)	1.4312(11)
S(1)-N(3)	1.6240(12)
S(1)-O(3)	1.4405(10)
S(1)-C(19)	1.7599(15)
N(1)-C(6)	1.4098(19)
N(1)-C(7)	1.3731(18)
N(1)-H(1)	0.880(18)
C(1)-H(1A)	0.9500
C(1)-C(2)	1.384(2)
C(1)-C(6)	1.395(2)
O(1)-C(7)	1.2206(17)
N(2)-C(7)	1.3889(18)
N(2)-C(8)	1.4909(18)

N(2)-C(10)	1.4976(17)
C(2)-H(2)	0.9500
C(2)-C(3)	1.385(2)
C(3)-H(3)	0.9500
C(3)-C(4)	1.387(2)
N(3)-C(11)	1.4876(17)
N(3)-C(12)	1.4823(18)
C(4)-H(4)	0.9500
C(4)-C(5)	1.388(2)
C(5)-H(5)	0.9500
C(5)-C(6)	1.392(2)
C(8)-C(9)	1.5515(19)
C(8)-C(12)	1.5229(19)
C(8)-C(13)	1.503(2)
C(9)-H(9A)	0.9900
C(9)-H(9B)	0.9900
C(9)-C(10)	1.534(2)
C(10)-H(10)	1.0000
C(10)-C(11)	1.518(2)
C(11)-H(11A)	0.9900
C(11)-H(11B)	0.9900
C(12)-H(12A)	0.9900
C(12)-H(12B)	0.9900
C(13)-C(14)	1.387(2)
C(13)-C(18)	1.394(2)
C(14)-H(14)	0.9500
C(14)-C(15)	1.391(2)
C(15)-H(15)	0.9500
C(15)-C(16)	1.382(2)
C(16)-H(16)	0.9500
C(16)-C(17)	1.385(2)
C(17)-H(17)	0.9500
C(17)-C(18)	1.386(2)
C(18)-H(18)	0.9500
C(19)-C(20)	1.391(2)
C(19)-C(24)	1.395(2)
C(20)-H(20)	0.9500
C(20)-C(21)	1.387(2)
C(21)-H(21)	0.9500
C(21)-C(22)	1.392(2)

C(22)-C(23)	1.391(2)
C(22)-C(25)	1.509(2)
C(23)-H(23)	0.9500
C(23)-C(24)	1.384(2)
C(24)-H(24)	0.9500
C(25)-H(25A)	0.9800
C(25)-H(25B)	0.9800
C(25)-H(25C)	0.9800
O(2)-S(1)-N(3)	107.16(6)
O(2)-S(1)-O(3)	120.00(6)
O(2)-S(1)-C(19)	108.35(7)
N(3)-S(1)-C(19)	107.84(7)
O(3)-S(1)-N(3)	105.27(6)
O(3)-S(1)-C(19)	107.68(6)
C(6)-N(1)-H(1)	114.1(11)
C(7)-N(1)-C(6)	125.33(12)
C(7)-N(1)-H(1)	116.4(11)
C(2)-C(1)-H(1A)	119.7
C(2)-C(1)-C(6)	120.60(14)
C(6)-C(1)-H(1A)	119.7
C(7)-N(2)-C(8)	124.94(11)
C(7)-N(2)-C(10)	124.78(12)
C(8)-N(2)-C(10)	88.79(10)
C(1)-C(2)-H(2)	119.9
C(1)-C(2)-C(3)	120.23(15)
C(3)-C(2)-H(2)	119.9
C(2)-C(3)-H(3)	120.4
C(2)-C(3)-C(4)	119.11(15)
C(4)-C(3)-H(3)	120.4
C(11)-N(3)-S(1)	117.31(9)
C(12)-N(3)-S(1)	116.10(9)
C(12)-N(3)-C(11)	120.09(11)
C(3)-C(4)-H(4)	119.3
C(3)-C(4)-C(5)	121.33(15)
C(5)-C(4)-H(4)	119.3
C(4)-C(5)-H(5)	120.3
C(4)-C(5)-C(6)	119.34(15)
C(6)-C(5)-H(5)	120.3
C(1)-C(6)-N(1)	117.68(13)

C(5)-C(6)-N(1)	122.92(13)
C(5)-C(6)-C(1)	119.38(14)
N(1)-C(7)-N(2)	111.62(12)
O(1)-C(7)-N(1)	124.99(13)
O(1)-C(7)-N(2)	123.35(13)
N(2)-C(8)-C(9)	86.42(10)
N(2)-C(8)-C(12)	109.72(11)
N(2)-C(8)-C(13)	118.95(12)
C(12)-C(8)-C(9)	109.35(11)
C(13)-C(8)-C(9)	118.66(12)
C(13)-C(8)-C(12)	111.31(12)
C(8)-C(9)-H(9A)	114.4
C(8)-C(9)-H(9B)	114.4
H(9A)-C(9)-H(9B)	111.6
C(10)-C(9)-C(8)	85.31(10)
C(10)-C(9)-H(9A)	114.4
C(10)-C(9)-H(9B)	114.4
N(2)-C(10)-C(9)	86.82(10)
N(2)-C(10)-H(10)	115.3
N(2)-C(10)-C(11)	109.85(11)
C(9)-C(10)-H(10)	115.3
C(11)-C(10)-C(9)	110.89(12)
C(11)-C(10)-H(10)	115.3
N(3)-C(11)-C(10)	108.33(11)
N(3)-C(11)-H(11A)	110.0
N(3)-C(11)-H(11B)	110.0
C(10)-C(11)-H(11A)	110.0
C(10)-C(11)-H(11B)	110.0
H(11A)-C(11)-H(11B)	108.4
N(3)-C(12)-C(8)	109.29(11)
N(3)-C(12)-H(12A)	109.8
N(3)-C(12)-H(12B)	109.8
C(8)-C(12)-H(12A)	109.8
C(8)-C(12)-H(12B)	109.8
H(12A)-C(12)-H(12B)	108.3
C(14)-C(13)-C(8)	122.61(13)
C(14)-C(13)-C(18)	119.16(14)
C(18)-C(13)-C(8)	118.24(13)
C(13)-C(14)-H(14)	120.0
C(13)-C(14)-C(15)	119.91(14)

C(15)-C(14)-H(14)	120.0
C(14)-C(15)-H(15)	119.7
C(16)-C(15)-C(14)	120.64(15)
C(16)-C(15)-H(15)	119.7
C(15)-C(16)-H(16)	120.1
C(15)-C(16)-C(17)	119.75(15)
C(17)-C(16)-H(16)	120.1
C(16)-C(17)-H(17)	120.1
C(16)-C(17)-C(18)	119.82(15)
C(18)-C(17)-H(17)	120.1
C(13)-C(18)-H(18)	119.6
C(17)-C(18)-C(13)	120.73(15)
C(17)-C(18)-H(18)	119.6
C(20)-C(19)-S(1)	119.28(11)
C(20)-C(19)-C(24)	121.03(14)
C(24)-C(19)-S(1)	119.64(11)
C(19)-C(20)-H(20)	120.6
C(21)-C(20)-C(19)	118.78(14)
C(21)-C(20)-H(20)	120.6
C(20)-C(21)-H(21)	119.3
C(20)-C(21)-C(22)	121.35(14)
C(22)-C(21)-H(21)	119.3
C(21)-C(22)-C(25)	121.18(15)
C(23)-C(22)-C(21)	118.63(14)
C(23)-C(22)-C(25)	120.18(14)
C(22)-C(23)-H(23)	119.3
C(24)-C(23)-C(22)	121.32(14)
C(24)-C(23)-H(23)	119.3
C(19)-C(24)-H(24)	120.6
C(23)-C(24)-C(19)	118.87(14)
C(23)-C(24)-H(24)	120.6
C(22)-C(25)-H(25A)	109.5
C(22)-C(25)-H(25B)	109.5
C(22)-C(25)-H(25C)	109.5
H(25A)-C(25)-H(25B)	109.5
H(25A)-C(25)-H(25C)	109.5
H(25B)-C(25)-H(25C)	109.5

7. References

1 Tercenio, Q. D. & Alexanian, E. J. Stereospecific Nickel-Catalyzed Reductive Cross-Coupling of Alkyl Tosylate and Allyl Alcohol Electrophiles. *Org. Lett.* **23**, 7215-7219 (2021).

2 Wang, X. *et al.* Access to Saturated Oxygen Heterocycles and Lactones via Electrochemical Sulfonylative Oxycyclization of Alkenes with Sulfonyl Hydrazides. *J. Org. Chem.* **88**, 2505-2520 (2023).

3 Hsu, C.-M. *et al.* Azetidines with All-Carbon Quaternary Centers: Merging Relay Catalysis with Strain Release Functionalization. *J. Am. Chem. Soc.* **145**, 19049-19059 (2023).

4 Chen, Y., Murray, P. R. D., Davies, A. T. & Willis, M. C. Direct Copper-Catalyzed Three-Component Synthesis of Sulfonamides. *J. Am. Chem. Soc.* **140**, 8781-8787 (2018).

5 Ma, Y.-R., Lv, X.-J., Dong, Q., Ming, Y.-C. & Liu, Y.-K. Brønsted-Acid-Catalyzed In Situ Formation of Acyclic Tertiary Enamides and Its Application to the Preparation of Diverse Nitrogen-Containing Heterocyclic Compounds. *Org. Lett.* **25**, 5929-5934 (2023).

6 Liu, D. *et al.* Formyl Radical Generation from α-Chloro N-Methoxyphthalimides Enables Selective Aldehyde Synthesis. *Angew. Chem. Int. Ed.* **62**, e202213686 (2023).

7 Liu, J. *et al.* Organocatalyzed Kinetic Resolution of α-Functionalized Ketones: The Malonate Unit Leads the Way. *ACS Catal.* **10**, 2882-2893 (2020).

8 Maskeri, M. A., Fernandes, A. J., Di Mauro, G., Maulide, N. & Houk, K. N. Taming Keteniminium Reactivity by Steering Reaction Pathways: Computational Predictions and Experimental Validations. *J. Am. Chem. Soc.* **144**, 23358-23367 (2022).

9 Ahmed, A. *et al.* Colchicine Glycorandomization Influences Cytotoxicity and Mechanism of Action. *J. Am. Chem. Soc.* **128**, 14224-14225 (2006).

10 Barluenga, J., Fañanás, F. J., Sanz, R., Marcos, C. & Ignacio, J. M. 2-Arylallyl as a new protecting group for amines, amides and alcohols. *Chem. Commun.*, 933-935 (2005).

11 He, Y.-P., Zhang, C., Fan, M., Wu, Z. & Ma, D. Assembly of Indoline-2-carboxylate-Embodied Dipeptides via Pd-Catalyzed C(sp2)–H Bond Direct Functionalization. *Org. Lett.* **17**, 496-499 (2015).

12 Meng, Y.-N. *et al.* Pd-Catalyzed MIA-Directed Acetoxylation of Benzylamines and Computational Study. *Eur. J. Org. Chem.* **2022**, e202200728 (2022).

13 Liu, Z. *et al.* Hypervalent Iodine Reagents Enable C–H Alkynylation with Iminophenylacetic Acids via Alkoxyl Radicals. *Org. Lett.* **24**, 5951-5956 (2022).

14 Davies, T. Q., Tilby, M. J., Skolc, D., Hall, A. & Willis, M. C. Primary Sulfonamide Synthesis Using the Sulfinylamine Reagent N-Sulfinyl-O-(tert-butyl)hydroxylamine, t-BuONSO. *Org. Lett.* **22**, 9495-9499 (2020).

15 Tercel, M. *et al.* Nitrobenzindoles and their use in cancer therapy. New Zealand. WO 2010/027280 A1. 2010

16 Roth, H. G., Romero, N. A. & Nicewicz, D. A. Experimental and Calculated Electrochemical Potentials of Common Organic Molecules for Applications to Single-Electron Redox Chemistry. *Synlett* **27**, 714-723 (2016).

17 Prier, C. K., Rankic, D. A. & MacMillan, D. W. C. Visible Light Photoredox Catalysis with Transition Metal Complexes: Applications in Organic Synthesis. *Chem. Rev.* **113**, 5322-5363 (2013).

18 Davies, B. & Morris, T. Physiological Parameters in Laboratory Animals and Humans. *Pharm. Res.* **10**, 1093-1095 (1993).

19 Obach, R. S. *et al.* The prediction of human pharmacokinetic parameters from preclinical and in vitro metabolism data. *J. Pharmacol. Exp. Ther.* **283**, 46-58 (1997).

20 Neese, F. The ORCA program system. *WIREs Computational Molecular Science* **2**, 73-78 (2012).

21 Neese, F. Software update: The ORCA program system—Version 5.0. *WIREs Computational Molecular Science* **12**, e1606 (2022).

22 Eckert, F., Pulay, P. & Werner, H.-J. Ab initio geometry optimization for large molecules. *J. Comput. Chem.* **18**, 1473-1483 (1997).

23 Fischer, T. H. & Almlof, J. General methods for geometry and wave function optimization. *J. Phys. Chem.* **96**, 9768-9774 (1992).

24 Neese, F., Wennmohs, F., Hansen, A. & Becker, U. Efficient, approximate and parallel Hartree–Fock and hybrid DFT calculations. A 'chain-of-spheres' algorithm for the Hartree–Fock exchange. *Chem. Phys.* **356**, 98-109 (2009).

25 Chai, J.-D. & Head-Gordon, M. Systematic optimization of long-range corrected hybrid density functionals. *J. Chem. Phys.* **128**, 084106 (2008).

26 Grimme, S., Ehrlich, S. & Goerigk, L. Effect of the damping function in dispersion corrected density functional theory. *J. Comput. Chem.* **32**, 1456-1465 (2011).

27 Grimme, S., Antony, J., Ehrlich, S. & Krieg, H. A consistent and accurate ab initio parametrization of density functional dispersion correction (DFT-D) for the 94 elements H-Pu. *J. Chem. Phys.* **132**, 154104 (2010).

28 Calais, J.-L. Density-functional theory of atoms and molecules. R.G. Parr and W. Yang, Oxford University Press, New York, Oxford, 1989. IX + 333 pp. Price £45.00. *Int. J. Quantum Chem* **47**, 101-101 (1993).

29 Helmich-Paris, B., de Souza, B., Neese, F. & Izsák, R. An improved chain of spheres for exchange algorithm. *J. Chem. Phys.* **155**, 104109 (2021).

30 Neese, F. An improvement of the resolution of the identity approximation for the formation of the Coulomb matrix. *J. Comput. Chem.* **24**, 1740-1747 (2003).

31 Weigend, F. Accurate Coulomb-fitting basis sets for H to Rn. *Phys. Chem. Chem. Phys.* **8**, 1057-1065 (2006).

32 Neese, F. The SHARK integral generation and digestion system. *J. Comput. Chem.* **44**, 381-396 (2023).

33 Hehre, W. J., Ditchfield, R. & Pople, J. A. Self—Consistent Molecular Orbital Methods. XII. Further Extensions of Gaussian—Type Basis Sets for Use in Molecular Orbital Studies of Organic Molecules. *J. Chem. Phys.* **56**, 2257-2261 (1972).

34 Krishnan, R., Binkley, J. S., Seeger, R. & Pople, J. A. Self-consistent molecular orbital methods. XX. A basis set for correlated wave functions. *J. Chem. Phys.* **72**, 650-654 (1980).

35 Matador, E. *et al.* A Photochemical Strategy for the Conversion of Nitroarenes into Rigidified Pyrrolidine Analogues. *J. Am. Chem. Soc.* **145**, 27810-27820 (2023).

36 Pracht, P., Bohle, F. & Grimme, S. Automated exploration of the low-energy chemical space with fast quantum chemical methods. *Phys. Chem. Chem. Phys.* **22**, 7169-7192 (2020).

37 Grimme, S. Exploration of Chemical Compound, Conformer, and Reaction Space with Meta-Dynamics Simulations Based on Tight-Binding Quantum Chemical Calculations. *J. Chem. Theory Comput.* **15**, 2847-2862 (2019).

38 Pracht, P. *et al.* CREST—A program for the exploration of low-energy molecular chemical space. *J. Chem. Phys.* **160**, 114110 (2024).

39 Bannwarth, C. *et al.* Extended tight-binding quantum chemistry methods. *Wiley Interdiscip. Rev. Comput. Mol. Sci.***11**, e1493 (2021).

40 Bannwarth, C., Ehlert, S. & Grimme, S. GFN2-xTB—An Accurate and Broadly Parametrized Self-Consistent Tight-Binding Quantum Chemical Method with Multipole Electrostatics and Density-Dependent Dispersion Contributions. *J. Chem. Theory Comput.* **15**, 1652-1671 (2019).

41 Frisch, M. J. *et al. Gaussian 09, Revision E.01.* (Gaussian, Inc., 2013).

42 Chai, J.-D. & Head-Gordon, M. Long-range corrected hybrid density functionals with damped atom–atom dispersion corrections. *Phys. Chem. Chem. Phys.* **10**, 6615-6620 (2008).

43 Weigend, F. & Ahlrichs, R. Balanced basis sets of split valence, triple zeta valence and quadruple zeta valence quality for H to Rn: Design and assessment of accuracy. *Phys. Chem. Chem. Phys.* **7**, 3297-3305 (2005).

44 Marenich, A. V., Cramer, C. J. & Truhlar, D. G. Universal Solvation Model Based on Solute Electron Density and on a Continuum Model of the Solvent Defined by the Bulk Dielectric Constant and Atomic Surface Tensions. *J. Phys. Chem. B* **113**, 6378-6396 (2009).
45 Ma, J. *et al.* Direct Dearomatization of Pyridines via an Energy-Transfer-Catalyzed Intramolecular [4+2] Cycloaddition. *Chem.* **5**, 2854-2864 (2019).
46 Garrido-García, P. *et al.* Enantioselective photocatalytic synthesis of bicyclo[2.1.1]hexanes as ortho-disubstituted benzene bioisosteres with improved biological activity. *Nat. Chem.* (2025).
47 Pettersen, E. F. *et al.* UCSF Chimera—A visualization system for exploratory research and analysis. *J. Comput. Chem.* **25**, 1605-1612 (2004).
48 RDKit: Open-source cheminformatics. (2025).
49 Kluyver, T. *et al.* in *Positioning and Power in Academic Publishing: Players, Agents and Agendas.* (eds Fernando Loizides & Birgit Schmidt) 87-90 (IOS Press).
50 Harris, C. R. *et al.* Array programming with NumPy. *Nature* **585**, 357-362 (2020).
51 pandas-dev/pandas: Pandas (v1.5.3) (Zenodo, 2023).
52 Firth, N. C., Brown, N. & Blagg, J. Plane of Best Fit: A Novel Method to Characterize the Three-Dimensionality of Molecules. *J. Chem. Inf. Model.* **52**, 2516-2525 (2012).
53 Bertz, S. H. The first general index of molecular complexity. *J. Am. Chem. Soc.* **103**, 3599-3601 (1981).
54 Sauer, W. H. B. & Schwarz, M. K. Molecular Shape Diversity of Combinatorial Libraries: A Prerequisite for Broad Bioactivity. *J. Chem. Inf. Comput. Sci.* **43**, 987-1003 (2003).
55 Prasanna, S. & Doerksen, R. J. Topological Polar Surface Area: A Useful Descriptor in 2D-QSAR. *Curr. Med. Chem.* **16**, 21-41 (2009).
56 Pracht, P. & Grimme, S. Calculation of absolute molecular entropies and heat capacities made simple. *Chem. Sci.* **12**, 6551-6568 (2021).
57 Mandigma, M. J. P., Kaur, J. & Barham, J. P. Organophotocatalytic Mechanisms: Simplicity or Naïvety? Diverting Reactive Pathways by Modifications of Catalyst Structure, Redox States and Substrate Preassemblies. *ChemCatChem* **15**, e202201542 (2023).
58 Strieth-Kalthoff, F., James, M. J., Teders, M., Pitzer, L. & Glorius, F. Energy transfer catalysis mediated by visible light: principles, applications, directions. *Chem. Soc. Rev.* **47**, 7190-7202 (2018).
59 Schlosser, L., Rana, D., Pflüger, P., Katzenburg, F. & Glorius, F. EnTdecker − A Machine Learning-Based Platform for Guiding Substrate Discovery in Energy Transfer Catalysis. *J. Am. Chem. Soc.* **146**, 13266-13275 (2024).
60 Becker, M. R., Richardson, A. D. & Schindler, C. S. Functionalized azetidines via visible light-enabled aza Paternò-Büchi reactions. *Nature Commun.* **10**, 5095 (2019).
61 Baldwin, J. E. Rules for ring closure. *J. Chem. Soc., Chem. Commun.*, 734-736 (1976).
62 Beckwith, A. L. J., Easton, C. J. & Serelis, A. K. Some guidelines for radical reactions. *J. Chem. Soc., Chem. Commun.*, 482-483 (1980).
63 Liu, R. S. H. & Hammond, G. S. Photosensitized internal addition of dienes to olefins. *J. Am. Chem. Soc.* **89**, 4936-4944 (1967).
64 Srinivasan, R. & Carlough, K. H. Mercury(3P1) photosensitized internal cycloaddition reactions in 1,4-, 1,5-, and 1,6-dienes. *J. Am. Chem. Soc.* **89**, 4932-4936 (1967).
65 Gleiter, R. & Sander, W. Light-Induced [2+2] Cycloaddition Reactions of Nonconjugated Dienes—the Effect of Through-Bond Interaction. *Angew. Chem. Int. Ed.* **24**, 566-568 (1985).
66 SAINT integration software version 8.39.0 (Bruker AXS Inc., Madison, WI, 2018).
67 SADABS version 2016/2 (Bruker AXS Inc., Madison, WI, 2016).
68 Dolomanov, O. V., Bourhis, L. J., Gildea, R. J., Howard, J. A. K. & Puschmann, H. Olex2 1.5. *J. Appl. Crystallogr.* **42**, 339 (2009).
69 *International Tables for Crystallography*. Vol. C (Wiley, 2016).
