# Supplementary material for: Breaking the ‘rule-of-five’ to access bridged bicyclic heteroaromatic bioisosteres
Source: Nat Synth. 2026 Feb 11;5(5):790–7. doi: 10.1038/s44160-026-00990-0 (PMC13171625; doi:10.1038/s44160-026-00990-0)
Supplement: Supplementary file 4 — DFT calculations, thermochemical data and XYZ coordinates. [file 44160_2026_990_MOESM4_ESM.pdf]

Supplementary Information

Thermochemical data and XYZ coordinates

Breaking the "Rule-of-Five" to access Bridged Bicyclic Heteroaromatic Bioisosteres

Ze-Xin Zhang, KaiChen Shu, Michael. J. Tilby, Mark Mandigma, Yiheng Guo, Jasper L. Tyler, Adam Noble*, Varinder. K. Aggarwal*

School of Chemistry, University of Bristol, Cantock's Close, Bristol BS8 1TS, United Kingdom

**e-mail: v.aggarwal@bristol.ac.uk and a.noble@bristol.ac.uk*

Summary of thermochemical data

Structure	E	ZPE	H	T.S	T.qh-S	G(T)	qh-G(T)
o SM_excitedtriplet_Phenyl	-1470.532431	0.379388	-1470.13	0.084216	0.078445	-1470.21	-1470.21
o TS6-exo-trigI	-1470.522575	0.379824	-1470.12	0.082541	0.076717	-1470.2	-1470.19
o TS6-exo-trigII	-1470.519239	0.380165	-1470.11	0.080831	0.075458	-1470.2	-1470.19
o TS5-exo-trigI	-1470.519637	0.378627	-1470.12	0.081711	0.076656	-1470.2	-1470.19
o TS5-exo-trigII	-1470.519989	0.378993	-1470.12	0.080677	0.075943	-1470.2	-1470.19
o TS5-exo-trigIII	-1470.517067	0.378891	-1470.11	0.080256	0.075856	-1470.19	-1470.19
o TS5-exo-trigIV	-1470.515201	0.379522	-1470.11	0.080597	0.075876	-1470.19	-1470.19
o TS6-endo-trigI	-1470.496182	0.378643	-1470.09	0.08027	0.07548	-1470.17	-1470.17
o TS6-endo-trigII	-1470.484355	0.379187	-1470.08	0.078323	0.074356	-1470.16	-1470.16
o TS6-endo-trigIII	-1470.481641	0.37953	-1470.08	0.079561	0.074709	-1470.16	-1470.15
o TS6-endo-trigIV	-1470.499875	0.378395	-1470.1	0.080416	0.075551	-1470.18	-1470.17
o TS7-endo-trig	-1470.507058	0.380288	-1470.1	0.079613	0.074841	-1470.18	-1470.18
o SM_excitedtriplet_Methyl	-1278.777104	0.324205	-1278.43	0.07616	0.071452	-1278.51	-1278.5
o TS6exotrigMe	-1278.769738	0.325373	-1278.42	0.074887	0.070517	-1278.5	-1278.49
o TS5exotrigMe	-1278.774609	0.32427	-1278.43	0.073595	0.0701	-1278.5	-1278.5

XYZ coordinates:

```
47
SM_excitedtriplet_Phenyl          Eopt  -1470.532431
C   -2.048097   -0.465497    0.161733
C   -0.816703    0.189619   -0.394513
C    0.216370    2.374441    0.214410
C   -1.034033    3.191220    0.218675
C   -2.931526    0.400406    0.938028
N   -1.449171    3.691798   -0.867205
C   -2.372840   -1.828664   -0.079511
C   -1.552635   -2.703552   -0.833952
C   -1.908811   -4.021971   -1.033724
C   -3.088488   -4.532015   -0.498328
C   -3.912914   -3.693564    0.247410
C   -3.568287   -2.373635    0.452552
O   -2.611418    4.412015   -0.703719
C   -3.013395    4.959686   -1.948775
H   -0.136337   -0.521752   -0.862932
H   -1.114679    0.887166   -1.182524
H    0.909520    2.790554    0.944085
H    0.684997    2.407614   -0.772953
H   -1.570709    3.326825    1.156548
H   -3.685815    1.009355    0.448231
H   -2.801026    0.524303    2.007737
H   -0.627332   -2.349368   -1.267119
H   -1.259294   -4.664442   -1.616301
H   -3.359086   -5.568087   -0.658419
H   -4.832221   -4.078245    0.672884
H   -4.218734   -1.733910    1.037994
H   -3.948120    5.483930   -1.755146
```

H	-2.268142	5.665429	-2.324649
H	-3.181494	4.172043	-2.687412
S	0.960166	0.207461	1.573238
C	2.334938	-0.230035	0.556242
C	3.326465	0.713368	0.306500
C	2.393828	-1.485692	-0.029920
C	4.377269	0.386752	-0.529222
H	3.282314	1.690182	0.769936
C	3.455765	-1.797783	-0.865084
H	1.623000	-2.217800	0.171269
C	4.460248	-0.871726	-1.127035
H	5.152456	1.119861	-0.719608
H	3.503471	-2.780855	-1.317911
N	-0.095474	0.997845	0.605521
O	0.316270	-1.002375	1.987521
O	1.414208	1.144301	2.558372
C	5.618484	-1.213236	-2.017140
H	5.480932	-2.184026	-2.492852
H	5.744563	-0.459138	-2.796674
H	6.547504	-1.243401	-1.442586

47
TS5exotrigI Eopt -1470.519637

C	-0.559608	0.913699	0.141586
C	-0.953439	-0.434928	-0.281537
H	-0.293377	-1.287571	-0.194328
H	-1.942266	-0.586495	-0.702418
C	-0.505671	1.933187	-0.977266
N	0.690698	2.759128	-0.795143
C	1.868059	1.901131	-0.681374
C	1.587160	0.881345	0.381748
N	2.006544	-0.337979	0.140698
O	1.977804	-1.085812	1.287696
C	2.308341	-2.435224	0.999696
H	-1.390333	2.570151	-0.976768
H	-0.459003	1.406898	-1.937057
H	2.081772	1.388709	-1.627325
H	2.728406	2.506932	-0.401395
H	1.528135	1.225641	1.410663
H	2.267853	-2.962259	1.951577
H	1.586909	-2.872244	0.305092
H	3.314438	-2.506965	0.579697
C	-1.006007	1.355491	1.471621
C	-1.256886	0.403822	2.470112
C	-1.162297	2.708867	1.800330
C	-1.651617	0.787058	3.739927
C	-1.563726	3.088485	3.070465
C	-1.808681	2.132604	4.047528
H	-1.133254	-0.648286	2.242751
H	-0.970707	3.474349	1.060633
H	-1.838943	0.032053	4.493847

47
TS5exotrigII Eopt -1470.519989

C	-0.770631	1.808613	1.486193
C	-0.472976	1.152839	0.201828
C	-1.146912	-0.117286	-0.082724
C	-1.805043	-0.798595	0.950399
C	-2.396890	-2.031534	0.737280
C	-2.346713	-2.622652	-0.517901
C	-1.695276	-1.964745	-1.553655
C	-1.099571	-0.733614	-1.341086
C	-0.220721	2.136730	-0.916704
N	1.046629	2.803970	-0.609387
C	2.114162	1.807666	-0.493068
C	1.624924	0.685865	0.377194
N	1.846310	-0.522204	-0.084630
O	1.613417	-1.451808	0.889472
C	1.655248	-2.758475	0.339702
H	-0.079937	1.823510	2.318947
H	-1.720298	2.321639	1.603540
H	-1.845551	-0.349410	1.935975
H	-2.897792	-2.533587	1.556195
H	-2.808069	-3.587708	-0.687218
H	-1.640994	-2.420395	-2.534962
H	-0.580404	-0.262814	-2.164683
H	-1.018741	2.879120	-0.947306
H	-0.162556	1.646272	-1.891759
H	2.378229	1.395103	-1.474129
H	2.993960	2.274372	-0.054142
H	1.581914	0.837656	1.452108
H	1.485327	-3.440719	1.171134

H	-1.686896	4.140545	3.297684
H	-2.119600	2.433472	5.040459
S	0.824087	4.130430	-1.681170
C	1.024232	3.609361	-3.353058
C	-0.097609	3.316395	-4.118381
C	2.299966	3.422264	-3.870209
C	0.066145	2.837030	-5.406563
H	-1.089879	3.468996	-3.714616
C	2.445953	2.945750	-5.161037
H	3.170803	3.657627	-3.272656
C	1.335399	2.641215	-5.946461
H	-0.809517	2.614239	-6.004663
H	3.440785	2.809739	-5.568550
C	1.507522	2.090665	-7.331140
H	0.627884	2.282336	-7.945865
H	2.380496	2.523772	-7.820853
H	1.657016	1.008284	-7.290240
O	2.029259	4.780055	-1.260765
O	-0.428620	4.816122	-1.565047

H	0.870857	-2.887610	-0.409568
H	2.631932	-2.957542	-0.108121
S	1.396035	4.199098	-1.392030
C	1.636516	3.764242	-3.084030
C	0.544041	3.714509	-3.943000
C	2.900129	3.401418	-3.529283
C	0.728655	3.307287	-5.251388
H	-0.439729	3.998044	-3.592604
C	3.067829	2.995530	-4.843191
H	3.747334	3.440944	-2.857391
C	1.989895	2.942559	-5.722565
H	-0.121529	3.273115	-5.922716
H	4.055379	2.715808	-5.190380
C	2.175804	2.524077	-7.150892
H	3.142272	2.042940	-7.300466
H	1.389968	1.833800	-7.461938
H	2.126369	3.393327	-7.811923
O	2.643601	4.665254	-0.863991
O	0.232508	5.029512	-1.300491

47

TS5exotrigIII Eopt -1470.517067

C	-0.620328	0.966434	0.287124
C	-0.734470	-0.481242	0.072646
H	-1.694171	-0.906637	-0.203038
H	0.109657	-1.149427	0.190058
C	-0.667837	1.806664	-0.969248
N	0.333535	2.893806	-0.898852
C	1.325298	2.744795	0.168339
C	1.505246	1.283741	0.455409
H	1.694890	0.636645	-0.396297
H	-1.658753	2.242994	-1.108894
H	-0.465368	1.156142	-1.822553
H	2.273991	3.186180	-0.136872
H	0.992355	3.276910	1.057783
C	-1.162917	1.511454	1.531297
C	-1.232730	0.697098	2.671471
C	-1.583622	2.843819	1.649632
C	-1.696408	1.192529	3.875990
C	-2.052423	3.335465	2.856443
C	-2.107615	2.516265	3.976624
H	-0.902679	-0.333024	2.605078
H	-1.546451	3.504839	0.792579
H	-1.733790	0.544592	4.743475
H	-2.375081	4.367439	2.923634
H	-2.472058	2.905819	4.919163
N	1.938275	0.778150	1.581785
O	1.905162	1.699061	2.595708
C	2.188952	1.067188	3.831935
H	3.187544	0.623560	3.818366
H	2.143445	1.850126	4.587344

47

TS5exotrigIV Eopt -1470.515201

C	-1.189120	1.937382	1.290066
C	-0.652051	1.170716	0.157363
C	-1.246253	-0.142086	-0.120758
C	-2.113171	-0.723775	0.814632
C	-2.669702	-1.973244	0.599612
C	-2.369826	-2.683296	-0.554648
C	-1.498308	-2.131167	-1.485554
C	-0.941035	-0.882227	-1.273530
C	-0.216687	2.049683	-0.994916
N	1.004982	2.729477	-0.564860
C	2.012807	1.734226	-0.194491
C	1.387776	0.709888	0.727485
H	-2.220351	2.274579	1.249926
H	-0.553059	2.333208	2.068778
H	-2.353410	-0.181604	1.721528
H	-3.343257	-2.392949	1.337018
H	-2.807067	-3.659099	-0.726051
H	-1.247988	-2.679752	-2.385616
H	-0.251348	-0.491301	-2.010048
H	-0.987411	2.791845	-1.204553
H	-0.031651	1.474623	-1.906556
H	2.377155	1.210019	-1.085292
H	2.846886	2.227356	0.297880
N	1.469019	0.762123	2.039228
O	1.795958	2.016535	2.476870
C	1.832259	2.042325	3.894990
H	2.585848	1.348309	4.274072
H	2.097616	3.062364	4.168135
H	0.854534	1.790116	4.312251

H	1.445734	0.298201	4.053881	H	1.327347	-0.308310	0.365012
S	0.812619	3.549772	-2.314860	S	1.499861	4.043803	-1.404573
C	1.916584	2.383432	-3.048850	C	1.999546	3.455359	-2.990375
C	1.416811	1.382949	-3.872841	C	1.048037	3.282977	-3.986760
C	3.270752	2.426232	-2.736569	C	3.326297	3.106429	-3.212818
C	2.278789	0.423044	-4.377146	C	1.433682	2.763979	-5.211786
H	0.365091	1.357652	-4.125216	H	0.016804	3.560564	-3.812336
C	4.117521	1.459495	-3.247316	C	3.695506	2.592437	-4.442725
H	3.661375	3.211956	-2.103616	H	4.065022	3.247074	-2.434937
C	3.637372	0.443677	-4.072790	C	2.757716	2.410816	-5.458746
H	1.887566	-0.354858	-5.022138	H	0.691976	2.637658	-5.991415
H	5.173031	1.496081	-3.004514	H	4.732568	2.332474	-4.619977
C	4.572925	-0.581150	-4.642882	C	3.173747	1.833072	-6.779242
H	4.041036	-1.490315	-4.924108	H	2.392946	1.953699	-7.530050
H	5.061622	-0.189859	-5.539233	H	4.085710	2.308356	-7.144464
H	5.355644	-0.839239	-3.928565	H	3.381797	0.764917	-6.676525
O	1.561369	4.731493	-2.005237	O	2.656100	4.554553	-0.730801
O	-0.359252	3.639858	-3.134375	O	0.350367	4.881273	-1.575724

47				47			
TS6endotrigI		Eopt	-1470.496182	TS6endotrigII		Eopt	-1470.484355
C	-0.484939	-1.322094	-1.851955	C	-3.806232	-0.326764	-0.570048
C	-0.257967	-0.927534	-0.454867	C	-2.767858	0.625963	-0.149873
C	1.053451	-0.347164	-0.114909	C	-3.085547	1.499512	0.988243
C	1.967860	0.000416	-1.112961	C	-4.150160	1.176369	1.839734
C	3.160919	0.631704	-0.797719	C	-4.438728	1.951230	2.948925
C	3.464382	0.935654	0.522424	C	-3.665054	3.067767	3.240001
C	2.564188	0.595609	1.525576	C	-2.608246	3.404006	2.403389
C	1.374368	-0.038178	1.212933	C	-2.320322	2.632566	1.289447
N	-1.451461	0.588912	-0.279150	N	-1.395915	-0.652236	0.560449
O	-1.145036	1.479299	-1.300625	O	-0.580013	0.151342	1.348343
C	-0.430831	2.590412	-0.778911	C	-0.852877	-0.094962	2.716150
C	-2.684276	0.147755	-0.406167	C	-0.788392	-0.954515	-0.582378
C	-3.103088	-0.948452	0.511134	C	0.132015	0.026534	-1.228727
C	-0.853399	-1.909929	0.558917	C	-2.035281	1.243782	-1.349683
H	-0.238695	-2.326160	-2.180131	H	-4.555522	-0.018926	-1.291306
H	-0.803729	-0.598076	-2.591844	H	-3.889757	-1.304847	-0.115119
H	1.737659	-0.221381	-2.148469	H	-4.753188	0.301363	1.628352
H	3.856281	0.890267	-1.587277	H	-5.267885	1.681799	3.591967
H	4.394646	1.432750	0.768515	H	-3.886202	3.673370	4.110386
H	2.789152	0.830385	2.559087	H	-2.001965	4.275167	2.621196
H	0.675413	-0.275051	2.005991	H	-1.489140	2.906098	0.654237
H	0.460241	2.275892	-0.234206	H	-0.594958	-1.124267	2.981859
H	-0.139838	3.185375	-1.644190	H	-0.220956	0.596103	3.273277
H	-1.072093	3.186435	-0.123324	H	-1.900686	0.092485	2.956932
H	-3.319672	0.463118	-1.225030	H	-2.279857	0.657815	-2.241367
H	-3.466980	-1.805362	-0.075533	H	-2.422914	2.250275	-1.503303
H	-3.922441	-0.619860	1.152274	H	-1.348612	-1.642771	-1.203850
H	-1.211970	-2.793926	0.018630	H	1.072441	0.140447	-0.693380

H	-0.086455	-2.233947	1.257353
N	-1.963439	-1.342630	1.335721
S	-2.291670	-2.052790	2.775070
C	-2.905733	-3.661438	2.394080
C	-2.017626	-4.717328	2.246919
C	-4.265081	-3.843864	2.165601
C	-2.498569	-5.962825	1.875285
H	-0.960755	-4.571841	2.427225
C	-4.728313	-5.092802	1.795726
H	-4.955249	-3.019081	2.285327
C	-3.854897	-6.170105	1.643297
H	-1.804181	-6.787350	1.766348
H	-5.788536	-5.237773	1.624006
C	-4.379790	-7.515478	1.236844
H	-3.580158	-8.253040	1.173098
H	-5.121358	-7.872598	1.954838
H	-4.873148	-7.456858	0.264085
O	-1.043727	-2.200155	3.463055
O	-3.347471	-1.297520	3.380871

H	0.339644	-0.299567	-2.252160
N	-0.573692	1.330701	-1.213467
S	0.166729	2.554928	-2.001951
C	-0.076047	2.240596	-3.721672
C	-1.233365	2.695494	-4.345297
C	0.844413	1.473586	-4.421905
C	-1.460677	2.377912	-5.671000
H	-1.946180	3.299181	-3.799375
C	0.601423	1.164211	-5.751169
H	1.747203	1.126167	-3.937430
C	-0.550876	1.605797	-6.394119
H	-2.360907	2.736398	-6.156506
H	1.322261	0.567497	-6.297331
C	-0.814033	1.275697	-7.833625
H	-1.801068	0.824491	-7.953919
H	-0.795026	2.181429	-8.444595
H	-0.068190	0.584044	-8.224926
O	-0.529119	3.764267	-1.671773
O	1.565347	2.442638	-1.712768

47

TS6endotrigIII Eopt -1470.481641

C	-3.063337	0.582370	-0.099177
C	-3.092018	1.605597	0.954439
H	-3.896040	2.334692	0.924606
H	-2.219035	1.831903	1.548351
N	-1.550781	-0.660402	0.370043
O	-0.750075	0.110752	1.207543
C	-0.999031	-0.269510	2.549361
C	-0.984481	-0.797250	-0.829258
C	-0.236685	0.339375	-1.447843
C	-2.571950	1.172203	-1.423726
H	-0.711988	-1.311067	2.716693
H	-0.384103	0.386445	3.165173
H	-2.052123	-0.136897	2.810861
H	-2.745715	0.502018	-2.266182
H	-3.137054	2.084967	-1.610102
H	-1.514624	-1.484770	-1.477258
H	0.687414	0.575163	-0.924269
H	-0.009417	0.101426	-2.491212
C	-4.191671	-0.369600	-0.141679
C	-4.502584	-1.134354	-1.271008
C	-4.953050	-0.576951	1.015335
C	-5.536709	-2.058663	-1.242425
C	-5.981805	-1.500954	1.043874
C	-6.281781	-2.248277	-0.088387
H	-3.941136	-1.024209	-2.188510
H	-4.724564	-0.004166	1.906709
H	-5.758165	-2.635291	-2.132440
H	-6.550632	-1.640909	1.955114
H	-7.086585	-2.972699	-0.069747

47

TS6endotrigIV Eopt -1470.499875

C	-0.285014	-1.004492	-0.579691
C	1.018184	-0.645636	0.000374
H	1.867558	-1.305641	-0.141479
H	1.084474	0.129411	0.752383
N	-1.356897	0.544239	-0.178440
O	-1.343247	1.420133	-1.252400
C	-0.186900	2.237654	-1.204014
C	-2.595242	0.224267	0.124762
C	-2.821388	-0.782169	1.199347
C	-0.996035	-2.072261	0.248814
H	0.725377	1.654241	-1.347170
H	-0.294681	2.946933	-2.023963
H	-0.131477	2.776939	-0.254718
H	-3.428760	0.621002	-0.441944
H	-3.607955	-1.479062	0.877849
H	-3.163163	-0.299818	2.117586
H	-1.784239	-2.571010	-0.320287
H	-0.260796	-2.827231	0.524758
C	-0.392734	-1.138337	-2.037476
C	0.669867	-0.771568	-2.875121
C	-1.589008	-1.547583	-2.646432
C	0.552045	-0.834132	-4.252961
C	-1.706458	-1.602380	-4.025090
C	-0.636189	-1.253007	-4.838468
H	1.600772	-0.435374	-2.434978
H	-2.448707	-1.807119	-2.041897
H	1.393759	-0.552970	-4.874782
H	-2.643060	-1.918952	-4.468413
H	-0.729355	-1.303533	-5.916285

N	-1.145535	1.506898	-1.352562	N	-1.574858	-1.498410	1.465339
S	-0.636143	2.907489	-2.007534	S	-1.503009	-2.392764	2.835202
C	-0.724581	2.689883	-3.757625	C	-2.562293	-3.777089	2.563658
C	-1.937969	2.872413	-4.409406	C	-2.046355	-4.942935	2.016280
C	0.397607	2.273812	-4.462048	C	-3.923520	-3.661627	2.825374
C	-2.020642	2.635810	-5.770672	C	-2.900022	-5.997721	1.734631
H	-2.809551	3.207311	-3.862239	H	-0.986421	-5.032471	1.819134
C	0.299124	2.044949	-5.823959	C	-4.761556	-4.722509	2.536519
H	1.342770	2.141802	-3.952681	H	-4.322156	-2.753493	3.257891
C	-0.908063	2.216810	-6.497770	C	-4.265556	-5.904662	1.986255
H	-2.965798	2.785925	-6.278825	H	-2.494540	-6.909057	1.311767
H	1.178501	1.730859	-6.374009	H	-5.821866	-4.632863	2.742280
C	-1.008904	1.934896	-7.967781	C	-5.191949	-7.044895	1.683022
H	-1.850798	2.462422	-8.416419	H	-5.999897	-6.720944	1.023597
H	-0.094858	2.224150	-8.487985	H	-4.664943	-7.869260	1.203136
H	-1.158273	0.865160	-8.137937	H	-5.652547	-7.420713	2.599604
O	-1.577964	3.922937	-1.638207	O	-2.064832	-1.591298	3.881142
O	0.742803	3.053746	-1.647100	O	-0.158395	-2.871503	2.954084

47				47			
TS6exotrigI		Eopt -1470.522575		TS6exotrigII		Eopt -1470.519239	
C	-12.228378	-0.612615	-0.496970	C	-12.073940	-0.217151	0.404754
C	-10.842974	-1.116773	-0.781006	C	-10.679588	-0.755199	0.554724
C	-9.465148	0.902239	-0.420658	C	-9.471606	1.322914	0.008038
C	-10.465842	1.992229	-0.178920	C	-10.635510	2.198002	-0.360156
C	-12.431247	0.801587	-0.160415	C	-12.284944	1.132881	0.965761
N	-10.471194	2.542378	0.990609	H	-10.805114	3.095884	0.228029
C	-13.329333	-1.519216	-0.462794	C	-13.124186	-0.965185	-0.168961
C	-13.177607	-2.913565	-0.658377	C	-12.914478	-2.208565	-0.829780
C	-14.266114	-3.762829	-0.621585	C	-13.956688	-2.891129	-1.417261
C	-15.546665	-3.269809	-0.389647	C	-15.257686	-2.390558	-1.371512
C	-15.721843	-1.904142	-0.186169	C	-15.495395	-1.180095	-0.724710
C	-14.641353	-1.046122	-0.215934	C	-14.461582	-0.478807	-0.142569
O	-11.263870	3.666984	1.003271	H	-10.367254	-0.610212	1.598554
C	-11.318319	4.201955	2.314422	H	-10.638037	-1.817233	0.344861
H	-10.378412	-1.502761	0.138761	H	-8.588871	1.671723	-0.528296
H	-10.893995	-1.943664	-1.484461	H	-9.275247	1.429032	1.079583
H	-8.571629	1.359776	-0.844826	H	-12.047888	1.296301	2.014280
H	-9.188802	0.425941	0.524532	H	-13.004827	1.807860	0.525983
H	-10.866144	2.515553	-1.043799	H	-11.919593	-2.626127	-0.903925
H	-12.668176	1.042444	0.872278	H	-13.758439	-3.828718	-1.922895
H	-12.926291	1.440721	-0.887075	H	-16.070743	-2.932387	-1.837833
H	-12.197985	-3.338848	-0.831382	H	-16.500945	-0.778869	-0.680272
H	-14.115683	-4.824821	-0.774606	H	-14.674658	0.461884	0.347473
H	-16.395334	-3.941872	-0.365220	N	-11.193690	2.038329	-1.508781
H	-16.712914	-1.506393	-0.003107	O	-12.164358	2.988260	-1.722052
H	-14.802537	0.012467	-0.054099	C	-12.845696	2.711907	-2.934864
H	-11.969767	5.072910	2.256608	H	-12.156698	2.739235	-3.782599
H	-11.737717	3.475987	3.015613	H	-13.337755	1.737246	-2.889226
H	-10.324926	4.508243	2.652689	H	-13.593842	3.496070	-3.043109
N	-9.974632	-0.090781	-1.360919	N	-9.712610	-0.079042	-0.327090

S	-8.988774	-0.559503	-2.583582	S	-8.411039	-0.947969	-0.813846
C	-7.766833	-1.602989	-1.854359	C	-7.347987	-1.057903	0.590524
C	-8.012004	-2.963912	-1.724126	C	-7.546850	-2.060454	1.529610
C	-6.601389	-1.044051	-1.344811	C	-6.367548	-0.091979	0.786222
C	-7.080104	-3.763795	-1.085562	C	-6.753758	-2.094423	2.665498
H	-8.918894	-3.397484	-2.124552	H	-8.307911	-2.813907	1.373988
C	-5.679769	-1.858186	-0.710134	C	-5.585045	-0.140056	1.924969
H	-6.411653	0.015879	-1.451179	H	-6.213087	0.685329	0.049714
C	-5.901945	-3.226836	-0.570792	C	-5.765221	-1.138471	2.882143
H	-7.271001	-4.825726	-0.985687	H	-6.906856	-2.879953	3.395842
H	-4.769787	-1.421990	-0.315323	H	-4.818079	0.611080	2.074268
O	-9.778269	-1.353676	-3.477093	C	-4.901943	-1.177058	4.108510
O	-8.340447	0.620383	-3.072991	H	-4.919961	-0.215178	4.624795
C	-4.880696	-4.106247	0.087771	H	-5.232666	-1.948575	4.803507
H	-4.148337	-4.450423	-0.647596	H	-3.862426	-1.381350	3.840994
H	-4.337742	-3.567647	0.865297	O	-8.881032	-2.262957	-1.134064
H	-5.344113	-4.988470	0.530073	O	-7.742011	-0.174425	-1.817004

40			
SM_excitedtriplet_Methyl Eopt -1278.777104			
C	-2.148111	-0.328583	0.123597
C	-0.815310	0.130799	-0.377091
C	0.239143	2.327265	0.197914
C	-1.009095	3.147937	0.215323
C	-3.081035	0.637922	0.673955
N	-1.431527	3.657292	-0.863696
O	-2.587653	4.384463	-0.681646
C	-3.007497	4.933318	-1.919771
H	-0.214732	-0.727907	-0.688828
H	-0.935016	0.757070	-1.268137
H	0.941539	2.747240	0.916929
H	0.695122	2.358097	-0.795411
H	-1.534773	3.285209	1.159002
H	-3.775551	1.184008	0.038192
H	-3.119396	0.854697	1.738799
H	-3.935453	5.463746	-1.710587
H	-2.264561	5.634168	-2.309332
H	-3.193604	4.146253	-2.654743
S	0.980067	0.174067	1.578057
C	2.376325	-0.250641	0.582929
C	3.357983	0.705497	0.341764
C	2.460539	-1.506739	0.001158
C	4.421787	0.392466	-0.482825
H	3.294946	1.682598	0.802406
C	3.535191	-1.805401	-0.822846
H	1.699068	-2.249851	0.196540
C	4.528317	-0.865392	-1.077999
H	5.188342	1.136278	-0.666803

H	3.601919	-2.788755	-1.272730
N	-0.063193	0.952006	0.594850
O	0.341509	-1.042506	1.983309
O	1.415049	1.112953	2.570511
C	5.698279	-1.191447	-1.958767
H	5.576751	-2.163663	-2.435954
H	5.820596	-0.435582	-2.737221
H	6.623256	-1.209923	-1.377216
C	-2.511043	-1.767073	-0.044399
H	-2.421951	-2.084607	-1.091240
H	-1.834639	-2.408798	0.534332
H	-3.531931	-1.967340	0.282214

40				40			
TS5exotrigMe	Eopt -1278.774609			TS6exotrigMe	Eopt -1278.769738		
C	-0.596723	0.914007	0.172238	C	-12.249985	-0.568796	-0.627047
C	-1.054633	-0.444902	-0.092969	C	-10.886809	-1.090118	-0.945276
H	-0.422634	-1.304690	0.095449	C	-9.489092	0.893409	-0.460747
H	-2.084793	-0.621995	-0.390566	C	-10.488807	1.979811	-0.190122
C	-0.514096	1.859274	-0.997320	C	-12.421132	0.707813	0.060873
N	0.663749	2.710108	-0.800778	N	-10.426640	2.571053	0.953626
C	1.869471	1.887552	-0.689872	O	-11.243427	3.681605	0.984277
C	1.643706	0.843496	0.361516	C	-11.221681	4.253611	2.279812
N	2.039384	-0.362795	0.087982	H	-10.434096	-1.548307	-0.048313
O	2.007205	-1.150633	1.218731	H	-10.976163	-1.876163	-1.693187
C	2.336239	-2.486905	0.883365	H	-8.586629	1.365592	-0.848782
H	-1.403891	2.490471	-1.044267	H	-9.229377	0.377623	0.468904
H	-0.428867	1.299717	-1.935793	H	-10.974324	2.449991	-1.042179
H	2.101760	1.393338	-1.640948	H	-12.426404	0.699036	1.152194
H	2.707040	2.523003	-0.405799	H	-13.075677	1.458508	-0.374282
H	1.544106	1.160460	1.396918	H	-11.887967	5.114455	2.239900
H	2.295980	-3.048519	1.815742	H	-11.585455	3.543313	3.026911
H	1.615363	-2.901238	0.173472	H	-10.213166	4.581993	2.545115
H	3.342632	-2.547780	0.460934	N	-9.978297	-0.057508	-1.455449
S	0.771952	4.094971	-1.668777	S	-8.937174	-0.503990	-2.639317
C	1.008369	3.596984	-3.343671	C	-7.747710	-1.560732	-1.874846
C	-0.097176	3.282351	-4.126344	C	-8.003325	-2.921727	-1.765776
C	2.293299	3.445189	-3.845514	C	-6.598203	-1.012565	-1.319596
C	0.094074	2.817919	-5.414465	C	-7.098183	-3.731916	-1.102251
H	-1.097685	3.407554	-3.733510	H	-8.897350	-3.347423	-2.201753
C	2.467485	2.981086	-5.139152	C	-5.702864	-1.836899	-0.660558
H	3.150641	3.695881	-3.234835	H	-6.399990	0.047267	-1.409593
C	1.375920	2.656011	-5.939948	C	-5.935892	-3.205560	-0.542190
H	-0.767797	2.577709	-6.026087	H	-7.297065	-4.793850	-1.019255
H	3.470576	2.870497	-5.533589	H	-4.804874	-1.408850	-0.230870
C	1.567410	2.121111	-7.328037	O	-9.682889	-1.280000	-3.584719
H	0.848239	2.559699	-8.021544	O	-8.264925	0.683242	-3.077293
H	2.574080	2.320260	-7.695376	C	-4.942191	-4.096900	0.142056
H	1.413313	1.038803	-7.340928	H	-4.193214	-4.446195	-0.573940
O	1.954837	4.771601	-1.226823	H	-4.416024	-3.566456	0.936504
O	-0.498142	4.749449	-1.564835	H	-5.426274	-4.976135	0.568065

C	-1.008684	1.522953	1.482286	C	-13.339460	-1.583285	-0.496057
H	-2.096485	1.666558	1.495076	H	-13.261172	-2.352880	-1.268489
H	-0.752042	0.872435	2.320299	H	-13.291729	-2.092153	0.478158
H	-0.539531	2.497049	1.634400	H	-14.324965	-1.117675	-0.565017
